# C-glycoside synthesis through radical cross-coupling of glycohydrazides

**DOI:** 10.1038/s41586-026-10807-x

**Published:** 2026-06-22

**Authors:** Yinliang Guo, Yiheng Li, Benedikt Buchberger, Yixin Liu, Carla Capone, Tapas Adak, Shubham Ojha, Jasper L. Tyler, Philipp Neigenfind, Molhm Nassir, Yu Kawamata, Varinder K. Aggarwal, Phil S. Baran

**Affiliations:** 1 Department of Chemistry, Scripps Research, La Jolla, CA USA; 2 School of Chemistry, University of Bristol, Bristol, UK

**Keywords:** Carbohydrate chemistry, Synthetic chemistry methodology

## Abstract

Carbohydrates are among the most abundant and structurally diverse biomolecules in nature, playing central roles in energy storage, molecular recognition and cell signalling. Within this domain, C-glycosides^1–3^, in which the oxygen atom of the glycosidic bond in O-glycosides is replaced by carbon, have emerged as valuable motifs in medicinal chemistry due to their resistance to enzymatic hydrolysis^2,4^. Of particular importance are C-aryl glycosides, exemplified by the SGLT2 inhibitors dapagliflozin, canagliflozin and empagliflozin, which are frontline therapies for type 2 diabetes^5–7^. However, scalable syntheses of C-aryl glycosides have relied traditionally on protected sugar derivatives, lengthy sequences or conventional cross-couplings that often suffer from poor selectivity, limited scope and extensive protecting-group manipulation^6^. Herein, we report a practical approach to C-aryl glycosides using glycosyl sulfonyl hydrazides as redox-neutral radical precursors for cross-coupling. Prepared directly from unprotected native sugars, these reagents generate glycosyl radicals under mild conditions and enable efficient access to diverse C-aryl glycosides, including all approved SGLT2 inhibitors, natural products such as salmochelins and neopetrosins, and medicinally relevant probes. Beyond anomeric functionalization, this platform enables C–C bond formation at several positions on carbohydrate scaffolds and supports stereoretentive radical coupling that can override inherent stereochemical biases, expanding practical access to carbohydrate-derived therapeutics and chemical tools.

## Abstract (web summary)

A platform for synthesis of C-aryl glycosides using glycosyl sulfonyl hydrazides as redox-neutral radical precursors for cross-coupling produces versatile and readily diversifiable building blocks, expanding practical access to carbohydrate-derived therapeutics and chemical tools.

## Main

**Fig. 1:**
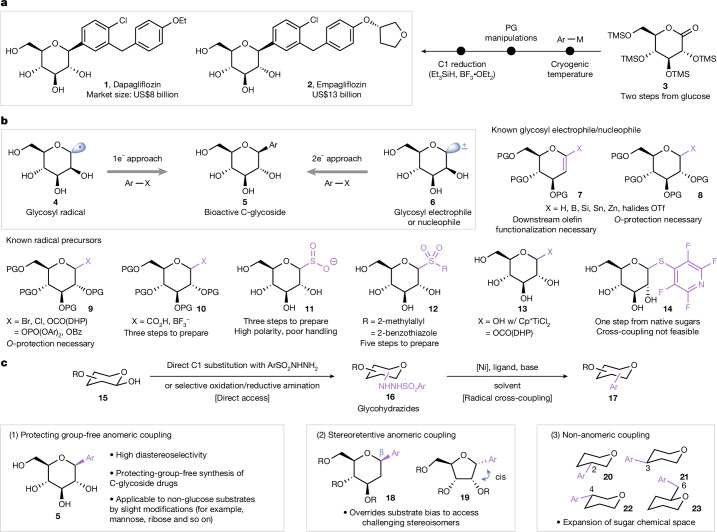
General access to C-glycosides. **a**, C-aryl glycoside drugs: privileged structures for the treatment of type 2 diabetes. **b**, Cross-coupling approach: previous art for 2e^–^ and 1e^–^ mode. **c**, Glycohydrazides: opening up new chemical space through redox-neutral RCC.

Carbohydrates are ubiquitous in biology, where they mediate processes ranging from energy storage to molecular recognition and cell signalling^8,9^. Among carbohydrate-based motifs, C-glycosides, where the anomeric oxygen of an O-glycoside is replaced by carbon, have attracted sustained interest due to enhanced resistance to hydrolytic and enzymatic degradation^2,5^. This feature has made them particularly important pharmacophores, most notably in sodium-glucose cotransporter 2 (SGLT2) inhibitors such as dapagliflozin (**1**) and empagliflozin (**2**), which are used widely for the treatment of type 2 diabetes^5,10^ (Fig. 1a). Five medicines of this type are currently United States (US) Food and Drug Administration (FDA)-approved, with combined annual sales exceeding US$20 billion. Although the clinical success of SGLT2 inhibitors reflects many pharmacological, pharmacokinetic and clinical factors, these compounds also exemplify how a robust C(*sp*³)–C(*sp*²) linkage can contribute to favourable metabolic stability and durable biological activity, underscoring the need for efficient and scalable synthetic strategies to access diverse C-aryl glycoside architectures.

Historically, the synthesis of C-aryl glycosides (**5**) has relied on methods wherein a glycosyl electrophile or nucleophile equivalent is used (**6**)^3^. For example, Friedel–Crafts-type glycosylations with electron-rich arenes or nucleophilic additions of organometallic aryl reagents (for example, aryl lithium or Grignard species) to glycosyl electrophiles. Although these approaches have enabled the construction of key C-glycosidic bonds, they are often plagued by notable limitations including extensive protecting-group manipulations, poor stereoselectivity at the anomeric centre, limited substrate scope and multi-step preparations of activated donors from native sugars. For instance, the general synthesis of **5** relies on polar bond disconnections through a multi-step sequence with only one strategic C–C bond-forming step^6,7,11^.

Recent advances since 2007 in the arena of conventional cross-coupling (cf. **7** and **8**), including Pd- and Ni-catalysed glycosyl cross-couplings^3,6,7,12–14^, have addressed some of these issues but still frequently demand protected sugars, specialized catalysts or organotin intermediates, hindering late-stage diversification and large-scale synthesis (Fig. 1b). In contrast, radical cross-coupling (RCC) can, in principle, offer a more direct and chemoselective approach to such targets by means of a glycosyl radical (**4**). Yet, practical implementation remains limited, as the preparation of these intermediates still typically relies on lengthy sequences involving protecting-group manipulations, particularly in the cases of **9**–**12** (refs. ^15–19^). Against this backdrop, glycosyl donor **14**, introduced by Koh, stands out for its direct preparation from an unprotected native sugar^20^. However, its synthetic utility along with **13** has so far been confined to radical addition reactions (for example, Giese addition), and the application to more versatile RCC has yet to be realized^21–23^.

In this work, a transformative approach to C-aryl glycoside synthesis is presented using easily accessible glycosyl sulfonyl hydrazides as redox-neutral radical precursors for RCC^24–28^ (Fig. 1c). Derived in a single step from unprotected native sugars without the need for protecting groups, stable crystalline glycohydrazides (**16**) enable straightforward generation of glycosyl radicals under mild and scalable conditions, facilitating couplings with a variety of aryl partners. This methodology not only streamlines access to bioactive molecules, including all currently approved SGLT2 inhibitor C-aryl glycosides (**5**), but also unlocks new possibilities for radical-mediated sugar modifications, including cross-couplings at non-anomeric sites (across positions C2–C6, **20**–**23**). Moreover, the unique reactivity of hydrazides can override inherent stereochemical biases that have represented unanswered challenges for radical-based cross-coupling (cf. **18** and **19**) by enabling a stereoretentive RCC approach^26,28^. Collectively, glycohydrazides address longstanding challenges in efficiency, selectivity and scalability in C-glycoside synthesis and provide a practical platform for the rapid exploration of carbohydrate-derived therapeutics, natural products and chemical probes.

## Unprotected glycohydrazide C-arylation

**Fig. 2:**
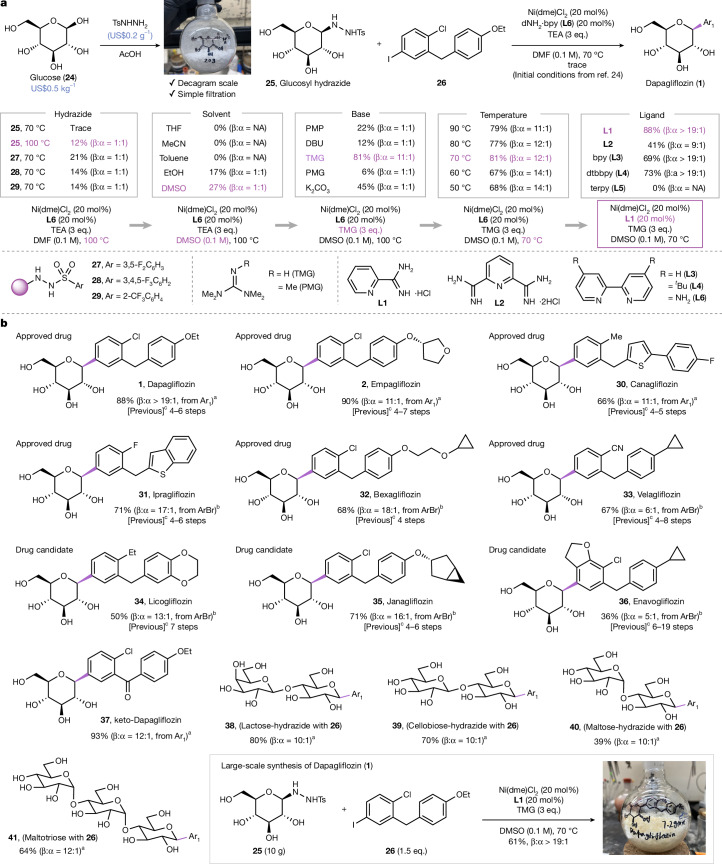
Reaction development and generality. **a**, Reaction development. **b**, Protecting-group-free glucose C1 coupling: Substrate scope. ^a^Reaction condition A: 20 mol% Ni(dme)Cl_2_, 20 mol% **L1**, 3.0 eq. TMG, 0.1 M DMSO, 70 °C. ^b^Reaction condition B: 20 mol% Ni(dme)Cl_2_, 20 mol% **L6**, 3.0 eq. TMG, 0.1 M DMSO, 70 °C. ^c^Step counts were calculated from the sugar source rather than from the coupling partner. Diastereomeric ratio was determined from the crude reaction mixture by ^1^H NMR or liquid chromatography–mass spectrometry (LCMS). Ar_1_ = 1-chloro-2-[(4-ethoxyphenyl)methyl]-benzene; ArBr, 4-bromo-1-chloro-2-[(4-ethoxyphenyl)methyl]benzene; DBU, 1,8-diazabicyclo[5.4.0]undec-7-ene; DMF, dimethylformamide; EtOH, ethanol; MeCN, acetonitrile; eq., equivalents; PMG, pentamethylguanidine; PMP, 1,2,2,6,6-pentamethylpiperidine; THF, tetrahydrofuran.

The development of suitable conditions for RCC-arylation of unprotected glycohydrazides required extensive optimization, the highlights of which are summarized graphically in Fig. 2a (for a more complete summary, see Supplementary Information). Glycohydrazides such as **25** can be prepared easily on a decagram scale by simply stirring a 1:1 mixture of sugar (glucose **24** in the case of **25**) and sulfonylhydrazine in acetic acid followed by simple crystallization^29^. Screening commenced with glucose-derived hydrazide **25** and aryl iodide **26** using the conditions reported previously^24^ for redox-neutral RCC with the identity of the sulfonyl hydrazide, solvent, base, temperature and ligand being evaluated systematically. Sulfonyl hydrazides are uniquely tunable radical precursors; the electronic nature of the sulfonyl group can dictate the rate of radical formation to reach an optimal level of kinetic matching in the coupling process^27^. Glycohydrazide **25**, when subjected to standard conditions at 70 °C, provided only trace quantities of product with a slight increase in temperature (100 °C) leading to 12% yield of coupled product **1** as a 1:1 mixture of diastereoisomers. Although the use of more electron-deficient hydrazides such as **27**, **28** and **29** led to a modest increase in yield at 70 °C, glycohydrazide **25** was chosen for further optimization owing to its lower cost, easy accessibility and suitability for large-scale synthesis of gliflozin-based medicines. Next, a solvent screen was performed (0.1 M), resulting in a higher yield using dimethylsulfoxide (DMSO; 27% yield) but unfortunately still as a 1:1 mixture of isomers. The most striking breakthrough came from an exploration of base. A variety of organic and inorganic bases were evaluated, differing in basicity, leading to an increase in yield (up to 45% with K_2_CO_3_) but still with a 1:1 mixture of diastereomers. Surprisingly, tetramethylguanidine (TMG) was singularly successful in boosting both the yield (81%) and selectivity (β:α = 11:1) as well as reducing the temperature to 70 °C. Although this outcome is difficult to rationalize given the lack of a clear correlation between base strength and either yield or diastereomeric ratio (see Supplementary Information for exhaustive listing of bases screened), we speculate that hydrogen bonding between TMG and the glucose hydroxyl groups may be important, as β-selectivity drops substantially with the corresponding Bn-protected glucose substrate (44%, β:α = 3.6:1). Consistent with this observation, the Niu group also observed high β-selectivity in RCCs with unprotected glucose in the presence of TMG, whereas the corresponding protected substrate showed much lower β-selectivity^17^. Finally, a number of ligands (**L1**–**L5**) were screened to see whether that parameter could be simplified as **L6** (dNH_2_bpy) is more expensive. **L1** was chosen considering the balance of yield (88%) and diastereomeric ratio (β:α > 19:1) observed, but other inexpensive ligands such as bpy (**L3**) or dtbbpy (**L4**) can also be used. Ultimately, a simple protocol using these conditions was developed that involves mixing all reaction components and heating to 70 °C for 1 h followed by a standard aqueous workup.

As depicted in Fig. 1b, glycohydrazide-based RCC could be used to access nearly all currently known FDA-approved gliflozin-based medicines (**1**, **2**, **30**, **32**, **33**) as well as several analogues that are currently in clinical trials (**34**, **35**, **36**). In addition, the known ketone-containing intermediate **37** (ref. ^30^) was prepared, which previously necessitated protecting groups due to the harsh conditions of Grignard addition. Accessing these structures using the less expensive aryl bromide was also possible (**31**, **32**, **33**, **34**, **35**, **36**) using **L6**. Remarkably, di- and tri-saccharide aryl-linked structures such as **38**, **39**, **40** and **41** could also be accessed directly from inexpensive lactose, cellobiose, maltose and maltotriose, respectively.

The general procedure could be applied to a decagram-scale synthesis of **1**. For this scale-up, glycohydrazide **25** was prepared directly from commercially available dextrose powder (purchased at Walmart for US$5 per pound) by treatment with TsNHNH_2_ in 70% household vinegar and stirring for 6 h, followed by simple ether precipitation to afford the crystalline intermediate. The key cross-coupling took place smoothly to provide **1** in 61% isolated yield with high diastereomeric ratio (β:α > 19:1) after extraction and chromatography. On larger scales, the product should be purified easily through direct recrystallization without the need for column chromatography^31^.

**Fig. 3:**
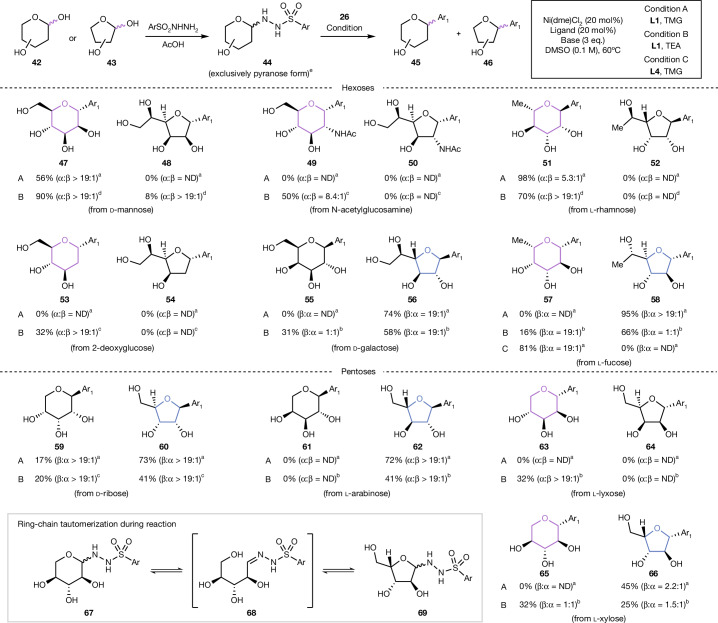
PG-free sugar C1 coupling: substrate scope. The structures of ribose, xylose and galactose hydrazides were further confirmed by X-ray crystallography. Diastereomeric ratio was determined from the crude reaction mixture by ^1^H NMR spectroscopy or LCMS. ^a^Toluenesulfonyl hydrazide. ^b^3,5-difluorobenzenesulfonyl hydrazide. ^c^3,4,5-trifluorobenzenesulfonyl hydrazide. ^d^2-(trifluoromethyl)benzenesulfonyl hydrazide. ^e^Structures were assigned using two-dimensional NMR spectroscopy (see Supplementary Information for details). ND, not determined.

To further investigate the substrate scope, a series of glycohydrazides were prepared from native hexoses and pentoses using a common protocol (TsNHNH_2_/acetic acid), as shown in Fig. 3. Notably, all of the resulting glycohydrazides adopt the pyranose form, as confirmed by X-ray crystallography and two-dimensional nuclear magnetic resonance (NMR) spectroscopy analyses, despite the conventional assumption that pentoses preferentially exist as furanoses whereas hexoses predominantly adopt pyranose structures. More intriguingly, these glycohydrazides exhibit markedly divergent reactivity under different reaction conditions: hexose-derived hydrazides can furnish either pyranose or furanose products, and a similar variability is observed for pentose-derived substrates, presumably arising from the unique ring–chain tautomerization behaviour of glycohydrazides (cf. **67**–**69**). Following systematic optimization, it was found that both the base and ligand play decisive roles in controlling the yield and selectivity of the main product. For hexose-derived substrates, pyranose products are formed preferentially. For example, mannose-, N-acetylglucosamine-, rhamnose- and 2-deoxyglucose-derived hydrazides deliver products **47**, **49**, **51** and **53**, respectively, with high diastereoselectivity. In contrast, the galactose-derived hydrazide yields predominantly the furanose product **56**. Notably, the reaction of fucose-derived hydrazide exhibits pronounced ligand dependence on the reactive tautomer: with **L1** (condition A or B), the furanose product **58** is formed predominantly, whereas using **L4** (condition C) switches the selectivity to the pyranose isomer **57** as the main product. For pentose-derived substrates, ribose-, arabinose- and xylose-derived hydrazides furnish mainly the corresponding furanose products **60**, **62** and **66**, whereas the lyxose-derived hydrazide instead affords the pyranose product **63**. Overall, this transformation exhibits broad substrate generality and well-defined chemo- and stereoselectivity, enabling efficient access to either furanose or pyranose C1-glycosylation products in a substrate-dependent manner, with ligand- and base-dependent modulation of selectivity demonstrated in representative cases. These features provide a versatile platform for the rapid construction of structurally diverse C1-glycosides, offering potential utility in synthesis, chemical biology and medicinal chemistry. The ligand-controlled divergency in the ring size of glycosyl RCC described above has not been described previously.

## Application to natural product synthesis

**Fig. 4:**
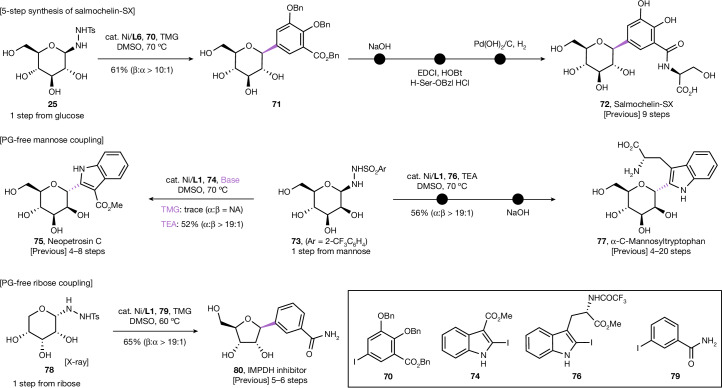
Synthetic applications towards natural products and drug derivatives. Diastereomeric ratio was determined from the crude reaction mixture by ^1^H NMR spectroscopy or LCMS. cat., catalyst.

The strategic application of redox-neutral glycohydrazide-based RCC to a variety of natural products and medicinally relevant structures beyond gliflozins was next explored, as documented in Fig. 4. Salmochelin-SX (**72**)—a C-aryl glycosidic natural product in the salmochelin family known as siderophores excreted by *Salmonella enterica* and uropathogenic *Escherichia coli* strains under low-iron stress^32^—was procured previously through a nine-step polar approach relying on conventional cross-coupling, pyrophoric reagents and protecting-group interchanges^13^. In contrast, a four-step approach commencing with crystalline glycohydrazide **25** led to **72** after coupling with aryl iodide **70** followed by a sequence of hydrolysis, amidation and debenzylation. The critical RCC took place in 61% isolated yield, favouring the desired diastereomer (β:α > 10:1). Beyond glucose-derived hydrazide **25**, mannose-derived hydrazide **73** likewise enabled efficient access to C-glycosides. Notably, neopetrosin C (**75**)—a rare indole C-glycoside alkaloid whose congeners have been reported to exhibit moderate hepatoprotective activity^33^—was prepared directly from **73** in a single step in 52% isolated yield with α:β > 19:1. In this case, the desired cross-coupling product was obtained under modified conditions using a more electron-deficient sulfonyl group (Ar = 2-CF_3_C_6_H_4_) and Et_3_N (TEA) in place of the more strongly basic TMG. The pronounced α-selectivity probably reflects a matched scenario in which both steric and anomeric effects converge to favour formation of the α-anomer, consistent with previous studies of RCC in related systems^17,34^. RCC has been applied to this natural product but it requires the use of expensive peracetyl mannosyl bromide (US$218 per 1 g commercial compound that needs to be stabilized by 2% CaCO_3_) by means of photoinduced electron transfer involving numerous additives and expensive iridium photocatalysts^35^. A polar-bond-based disconnection strategy using a Larock coupling of alkynyl glycoside traversing through ten steps is also known^36^. Similarly, the naturally occurring tryptophan-mannose conjugate **77** can be procured from **73** and **76** in 56% isolated yield as a single diastereomer (α-anomer) after alkaline hydrolysis of the Trp-containing TFA motif. Conventional polar strategies required more than 20 steps^37^ and redox-reliant RCC could achieve the same transformation, albeit requiring a cocktail of additives and an expensive Ir-catalyst^38^. Finally, the ribose-derived IMPDH inhibitor **80** could be accessed concisely from ribohydrazide **78** and *m*-iodobenzamide **79** as a single diastereomer (β-anomer) in 65% isolated yield, representing a substantial simplification relative to the previous five-step route involving extensive protecting-group manipulations^39^.

## Pre-radical stereocontrol

**Fig. 5:**
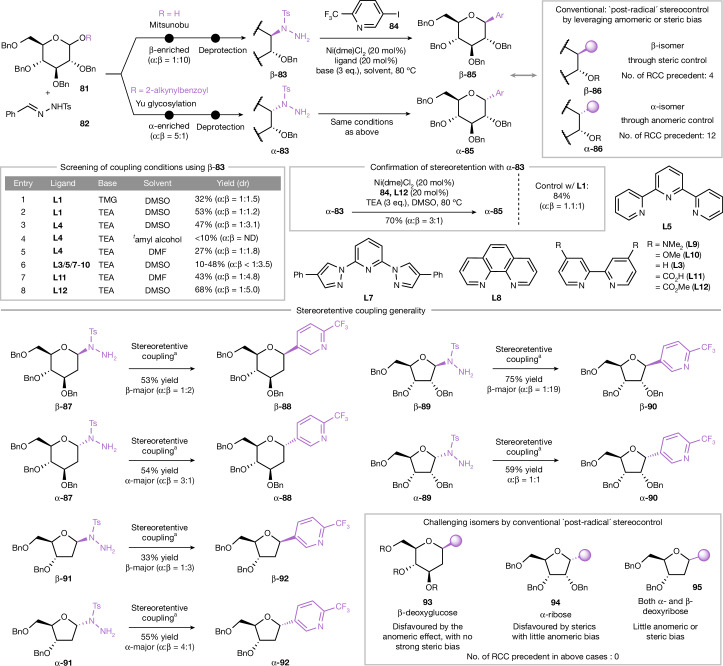
Stereoretentive RCC: programmable C1 stereochemistry overriding substrate biases. Diastereomeric ratio was determined from the crude reaction mixture by ^1^H NMR spectroscopy or LCMS. ^a^Stereoretentive condition: 20 mol% Ni(dme)Cl_2_, 20 mol% **L12**, 3.0 eq. TEA, 0.1 M DMSO, 80 °C. dr, diastereomeric ratio.

Up to this stage, stereochemical control in our redox-neutral platform arises exclusively from post-radical control, wherein product configuration is set during bond formation through the interplay of substrate stereoelectronic bias and catalyst steric effects. To our knowledge, all previously reported RCC approaches (Giese, as in refs. ^20,40^; Minisci, as in ref. ^21^ and analogous innate radical additions are not included as they are not considered cross-coupling, which involves catalytic organometallic intermediates) to C-glycosides operate by this same principle^17^. Although high selectivity and, in some cases, stereodivergence can be achieved, these outcomes are typically confined to particular substrates rather than representing a general solution. This limitation is evident across carbohydrate scaffolds: whereas glucose-derived systems have furnished numerous stereoselective examples by leveraging either steric effects (favouring the β-anomer^18,19,38,41^) or the anomeric effect (favouring the α-anomer^15,34,42–47^), several other scaffold classes still lack any stereoselective RCC manifold (Fig. 5). Collectively, these observations underscore the inherent substrate dependence of post-radical stereocontrol and the need for a more general, programmable approach.

Departing from this paradigm, programmable pre-radical stereocontrol was envisioned building on the recent discovery of stereoretentive RCC^26^ (Fig. 5). For these studies, Bn-protected sugars were used to simplify the preparation and purification of either anomeric series (α or β). The divergent preparation of either the β- or α-glycohydrazides could be accomplished commencing with **81** using either a Mitsunobu reaction or through Yu-glycosylation^48^ with benzaldehyde *p*-toluenesulfonylhydrazone **82**, respectively. With configurationally enriched anomers in hand, stereoretentive RCC was investigated using a variety of ligands, bases and solvents (see Supplementary Information for a more complete listing). In line with previous reports on substrate controlled RCC of protected glucose derivatives, the result is a near 1:1 mixture of products (entry 1) in 32% yield (Fig. 2). Reducing the strength of the base to TEA improved the yield albeit with a poor stereoselectivity (entry 2). After an extensive ligand screen (entries 3–8) and in accordance with results from our studies of stereoretentive RCC arylation^26^, electron-deficient bipyridine ligand **L12** enabled product formation in good yield (68%) with synthetically useful levels of stereocontrol (5:1) favouring the β-product. Turning attention to α-**83**, the same set of conditions could be applied to deliver 70% of adduct **85** in a 3:1 ratio favouring the α-product, unequivocally demonstrating that the stereochemistry was retained through the process. Unsurprisingly, the use of **L1** (non-stereoretentive ligand) delivered a 1:1 mixture of isomers once again, highlighting the critical importance of the ligand.

Stereoretentive couplings of this type were explored on three additional substrates, such as 2-deoxyglycohydrazides β-**87** and α-**87**, ribohydrazides β-**89** and α-**89**, and 2-deoxyribohydrazides β-**91** and α-**91**. In most cases, a modest to good level of stereoretention could be observed, thereby enabling the practitioner to enrich diastereochemical outcomes independent from inherent substrate biases. The results presented herein represent an important advance in stereoretentive radical C-glycosylation given past precedent. For instance, ribose systems favour β-products exclusively due to steric considerations; in 2-deoxyglucose and 2-deoxyribose, the stereoelectronic effect dominates to give the α-product^44^. Notably, several anomeric configurations including β-2-deoxyglucose (**93**), α-ribose (**94**) and β-2-deoxyribose (**95**) have not been accessed previously through RCC.

## Peripheral C–C bond formation

**Fig. 6:**
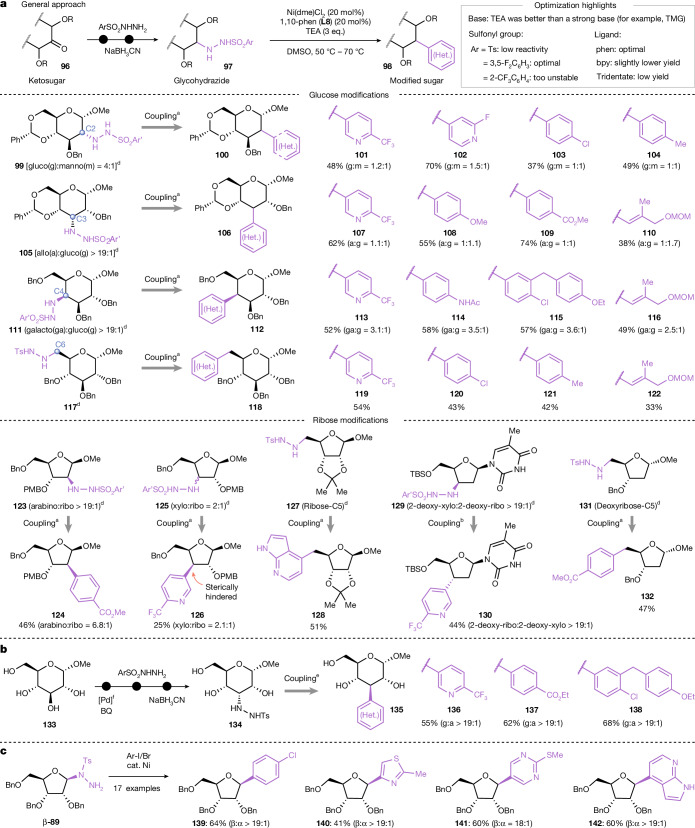
Peripheral C–C bond formation. **a**, Coupling at C2–C6 positions. **b**, Selective oxidation followed by C3 coupling. **c**, A redox-neutral platform to access nucleoside analogues. ^a^Condition: 20 mol% Ni(dme)Cl_2_, 20 mol% **L8**, 3.0 eq. TEA, 0.1 M DMSO, 50–70 °C. ^b^Condition: 20 mol% Ni(dme)Cl_2_, 20 mol% **L6**, 3.0 eq. PMP, 0.1 M DMF, 75 °C. ^c^Condition: 20 mol% Ni(dme)Cl_2_, 20 mol% **L4**, 3.0 eq. TEA, 0.1 M DMF, 100 °C. ^d^Step counts for synthesizing the hydrazides: glucose C2 hydrazide **99** (three steps from commercially available **SF5**); glucose C3 hydrazide **105** (three steps from **SF5**); glucose C4 hydrazide **111** (four steps from **SF5**); glucose C6 hydrazide **117** (four steps from commercially available **SF9**); ribose C2 hydrazide **123** (five steps from commercially available **SF12**); ribose C3 hydrazide **125** (five steps from **SF12**); ribose C5 hydrazide **127** (two steps from commercially available **SF15**); 2-deoxyribose C3 hydrazide **129** (five steps from commercially available **SF16**); 2-deoxyribose C5 hydrazide **131** (six steps from **SF16**). ^e^Condition: 20 mol% Ni(dme)Cl_2_, 20 mol% **L1**, 3.0 eq. TEA, 0.1 M DMSO, 75 °C. ^f^[(neocuproine)Pd(μ-OAc)]_2_[OTf]_2_. See Supplementary Information for detailed procedures and full listing of 17 examples in **c**. Diastereomeric ratio was determined from the crude reaction mixture by ^1^H NMR spectroscopy or LCMS.

The scope of glycohydrazide-based RCC was explored to enable peripheral C–C bond formation at positions beyond the anomeric centre, providing a systematic exploration of RCC across all alcohol-containing positions of riboses and glucoses. Accessing such chemical space could potentially open many new opportunities for diversification of carbohydrate scaffolds. The results of these investigations are depicted in Fig. 6a and commence with the preparation of 2-, 3-, 4- and 6-substituted glucohydrazides **99**, **105**, **111** and **117** by means of reductive amination from the corresponding carbonyl compounds. For these studies, additional optimization was conducted leading to the use of difluorophenyl sulfonyl hydrazide, 1,10-phenanthroline as ligand and TEA as base (see Supplementary Information for summary). Using these conditions, a library of 20 RCC products (16 of which are depicted herein, see Supplementary Information for full listing) was obtained in synthetically useful yields with post-radical stereocontrol (stereoretentive RCC was not attempted, nor was optimization of any individual reaction). Notably, the first examples of RCC-based sugar olefinations were also achieved in synthetically useful yields, providing adducts **110**, **116**, **122**. Similarly, ribose- and deoxyribose-derived hydrazides **123**, **125**, **127**, **129** and **131** were prepared and subjected to RCC to produce a library of 19 RCC-derived adducts, 5 of which are depicted in Fig. 6a. Of particular significance, glycohydrazide-based RCC proved amenable to unprotected glucoside **134**. Methyl 3-keto-α-d-glucopyranoside was accessed through selective oxidation of α-d-glucopyranoside **133** (refs. ^49,50^), followed by reductive amination to furnish the corresponding α-hydrazide **134**. Subsequent coupling delivered adducts **136**, **137** and **138** in good yield with high diastereoselectivity (β:α > 19:1; Fig. 6b). These results further indicate that, in addition to protecting-group strategies that facilitates handling and purification, unprotected glycohydrazides can also exhibit suitable reactivity and selectivity in RCC.

Finally, given the broad importance of unnatural nucleosides across biology and medicine, we sought to demonstrate concise entry into this chemical space (Fig. 6c). Using conditions related closely to those developed for the non-anomeric couplings in Fig. 6a, C1-arylated ribose derivatives **139**–**142** were obtained in synthetically useful yields, with 13 additional examples provided in the Supplementary Information. The efficiency of this transformation is comparable to that of the recent photochemical approach reported by Britton and colleagues^51^, yet the ribohydrazide platform offers a practical advantage by avoiding the engineering constraints associated with photochemical reaction setups. Notably, high β-selectivity is achieved without invoking a stereoretentive manifold, as the intrinsic steric bias of the ribose scaffold is sufficient to enforce conventional post-radical stereocontrol.

## Conclusion

The past decade has witnessed a vibrant and growing effort to enable synthetic elaboration of carbohydrates without the protecting-group sequences^17,20,23,52–58^ and redox manipulations that long dominated the field. The present work joins and extends this trajectory by addressing a distinct and hitherto unmet challenge: the absence of a practical, versatile radical glycosyl donor suitable for modular cross-coupling. By converting unprotected sugars in a single step into stable sulfonyl hydrazides that serve as such donors, a direct, efficient, highly chemoselective and scalable route to C1-glycosides has been unlocked. This chemistry provides rapid access to current FDA-approved SGLT2 inhibitors, challenging natural products and a host of new glyco-architectures—including modifications at non-anomeric positions and stereoisomers previously considered out of reach. In doing so, it shifts carbohydrates from being among the most cumbersome classes of molecules to synthesize into versatile and readily diversifiable building blocks. We anticipate that this platform, amenable to derivatization at several sites on sugars and to stereoretentive coupling, will catalyse a substantial expansion in the exploration of carbohydrate chemical space and help unleash the full therapeutic potential of glycomimetics in the coming years.

## Online content

Any methods, additional references, Nature Portfolio reporting summaries, source data, extended data, supplementary information, acknowledgements, peer review information; details of author contributions and competing interests; and statements of data and code availability are available at 10.1038/s41586-026-10807-x.

## Supplementary information

Supplementary Information Experimental procedures, characterization data and NMR spectra.

Supplementary Data 1 Crystallographic data for compound Ribose-hydrazide 78.

Supplementary Data 2 Crystallographic data for compound Galactose-hydrazide β-SH12.

Supplementary Data 3 Crystallographic data for compound Xylose-hydrazide β-SH21.

Supplementary Data 4 Crystallographic data for compound Mannose-arylated product α-47.

Peer Review File

## Data Availability

The data that support the findings in this work are available within the paper and Supplementary Information.
