# Supplementary material for: C-glycoside synthesis through radical cross-coupling of glycohydrazides
Source: Nature. 2026 Jun 22;655(8125):1195–203. doi: 10.1038/s41586-026-10807-x (PMC13421352; doi:10.1038/s41586-026-10807-x)
Supplement: Supplementary file 1 — Experimental procedures, characterization data and NMR spectra. [file 41586_2026_10807_MOESM1_ESM.pdf]

nature portfolio

https://doi.org/10.1038/s41586-026-10807-x

Supplementary information

C-glycoside synthesis through radical cross-coupling of glycohydrazides

In the format provided by the authors and unedited

C-glycoside synthesis via radical cross-coupling of glycohydrazides

Authors: Yinliang Guo[1†], Yiheng Li[1†], Benedikt Buchberger[1], Yixin Liu[1], Carla Capone[1], Tapas Adak[2], Shubham Ojha[2], Jasper L. Tyler[2], Philipp Neigenfind[1], Molhm Nassir[1], Yu Kawamata*[1], Varinder K. Aggarwal*[2], Phil S. Baran*[1]

Affiliations:

[1]Department of Chemistry, Scripps Research, 10550 North Torrey Pines Road, La Jolla, CA, 92037, United States.

[2]School of Chemistry, University of Bristol, Cantock's Close, Bristol, BS8 1TS, U.K.

[†]These authors contributed equally to this work.

[*]Correspondence to: yukawama@scripps.edu, V.Aggarwal@bristol.ac.uk, pbaran@scripps.edu

Table of Contents

C-Glycoside Synthesis via Radical Cross-Coupling of Glycohydrazides 1

1. General Experimental Information 5

2. Optimization 6

2.1 RCC-arylation of Unprotected Glycohydrazides 6

2.2 Stereoretentive RCC-arylation of Bn-protected Glycohydrazides 12

2.3 RCC-arylation of C2-C6 Sugar Sulfonylhydrazides 13

2.4 RCC-arylation at Unprotected Glucoside C3 Position.. 16

3. Extended Reaction Scope.. 17

3.1 PG-free Glucose C1 coupling 17

3.2 RCC-arylation at Protected Glucose C2/C3/C4/C6 positions 18

3.3 RCC-arylation at Protected Ribose and 2-Deoxyribose C2/C3/C5 position 19

3.4 RCC-arylation at Bn-protected Ribose C1 position.. 20

3.5 RCC-arylation at Unprotected Glucoside C3 Position 21

4. Synthetic Procedures... 22

4.1 General Synthetic Procedures for native sugar C1-hydrazide................................ 22

4.2 General Synthetic Procedure for cross-coupling of glucose C1 hydrazides and aryl halides 23

4.2.1 Graphical Experimental Guide for General Procedure B................................ 24

4.3 General Synthetic Procedure for Bn-protected sugar C1-hydrazide 26

4.3.1 Synthesis of glucose C1 hydrazide 27

4.3.2 Synthesis of 2-deoxy-glucose C1 hydrazide.. 28

4.3.3 Synthesis of ribose C1 hydrazide 28

4.3.4 Synthesis of 2-deoxy-ribose C1 hydrazide... 29

4.4 General Synthetic Procedures for stereoretentive cross-coupling of protected sugar C1-hydrazides and aryl halides 31

4.4.1 Graphical Experimental Guide for General Procedure E................................ 32

4.5 Synthetic Procedures for C2 to C6 sugar hydrazide.. 34

4.5.1 Synthesis of glucose C2 hydrazide 36

4.5.2 Synthesis of glucose C3 hydrazide 36

4.5.3 Synthesis of glucose C4 hydrazide 36

4.5.4 Synthesis of glucose C6 hydrazide 37

4.5.5 Synthesis of ribose C2 and C3 hydrazide 38
4.5.6 Synthesis of ribose C5 hydrazide 38
4.5.7 Synthesis of 2-deoxy-ribose C3 hydrazide 39
4.5.8 Synthesis of 2-deoxy-ribose C5 hydrazide 40
4.5.9 Synthesis of C3 Thymidine hydrazide 109 42
4.6 General Synthetic Procedure for cross-coupling of sugar hydrazides and aryl halides 43
4.6.1 General Synthetic Procedure for cross-coupling of sugar C2-C6 hydrazides and aryl halides 43
4.6.2 Graphical Experimental Guide for General Procedure H 44
4.6.3 Synthesis of C3-Glycohydrazide (w/o PG): 47
4.6.4 General procedure for C3 arylation of unprotected sugar 48
4.6.5 General procedure for C1 arylation of Bn-protected ribose 49
4.7 Synthetic Procedures of Aryl Halides 50
5. Frequently Asked Questions 52
6. Characterization Data 55
6.1 Characterization Data of Native Sugar Sulfonylhydrazides 55
6.2 Characterization Data of Gliflozins and Related Compounds 62
6.2.1 Coupling partners 62
6.2.2 Coupling products 65
6.3 Characterization Data of Bn-protected Sugar Sulfonylhydrazides 82
6.3.1 Characterization of C1-Hydrazides and RCC-products (for stereorententive coupling) 82
6.3.2 Characterization of C2-C6-Hydrazide 107
6.4 Characterization Data of C2-C6 Arylated Products 145
6.5 Characterization data of C1-arylated Ribose 198
7. Applications 214
7.1 Summary of Previous Synthetic Routes 214
7.2 Synthesis of Salmochelin-SX 215
7.3 Synthesis of Neopetrosin C 220
7.4 Synthesis of a-C-Mannosyltryptophan 222
7.5 Synthesis of IMPDH Inhibitor 225

8. Gram-Scale Synthesis **226**

9. RCC-Arylation of Different Sugars **230**

10. Selective Oxidation Followed by C3 Coupling **275**

11. X-ray Crystallography **285**

12. References **323**

13. NMR Spectra **326**

1. General Experimental Information

All air and/or moisture sensitive reactions were performed under an atmosphere of dry argon using anhydrous solvents and standard Schlenk techniques. All reagents and substrates that were purchased, were used without further purification. Yields refer to chromatographically and spectroscopically (^{1}H NMR) homogeneous material, unless otherwise stated. Reactions were monitored by thin layer chromatography (TLC) carried out on 0.25 mm E. Merck silica plates (60F-254), using UV light as the visualizing agent and/or KMnO4, and heat as a developing agent. Flash silica gel chromatography was performed using E. Merck silica gel (60, particle size 0.043 – 0.063 mm). NMR spectra were recorded on Bruker DRX600, DRX-500 and AMX-400 instruments; chemical shifts are reported in parts per million (δ in ppm) and are referenced to residual solvent signal ($CDCl_3$ – 7.26 ppm 1H NMR, 77.16 ppm ^{13}C NMR; CD_3OD – 3.31 ppm ^{1}H NMR, 49.00 ppm ^{13}C NMR; DMSO-d6 – 2.50 ppm ^{1}H NMR, 39.52 ppm ^{13}C NMR). All coupling constants (J) are reported in Hertz (Hz). Splitting patterns are assigned as s = singlet, br = broad singlet, d = doublet, t = triplet, q = quartet, p = pentet, m = multiplet. High-resolution mass spectra (HRMS) were recorded on an Agilent LC/MSD TOF mass spectrometer by electrospray ionization time-of-flight (ESI-TOF) reflection experiments. Diastereomeric ratios were determined by crude NMR. Optical rotations were measured on an Anton Paar MCP 100.

2. Optimization

2.1 RCC-arylation of Unprotected Glycohydrazides

We evaluated the feasibility of the radical cross-coupling reaction of unprotected glycohydrazides using glucose derived sulfonyl hydrazide and aryl iodide. The yield and diastereomeric ratio were determined by crude NMR.

Table S1

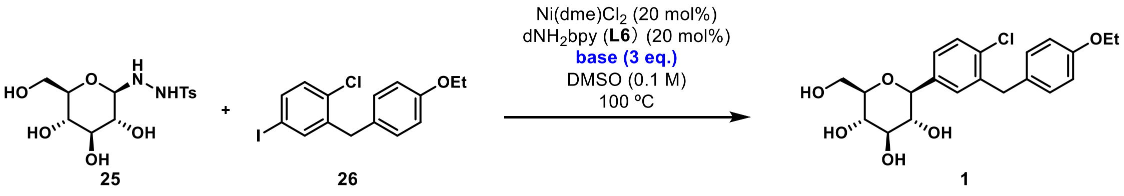

Entry	Variations	Yield	β:α
1	Et_2NH	22%	1:1
2	DBN	30%	10.6:1
3	TBD	—	—
4	pyridine	—	—
5	DABCO	21%	1:1
6	quinuclidine	25%	1:1
7	1,2,3-triphenylguanidine	—	—
8	4-methylmorpholine	—	—
9	Cs_2CO_3	—	—
10	DIPEA	9%	1:1
11	TMEDA	2%	1:1
12	N-methylpyrrolidine	19%	1:1
13	piperidine	22%	1:1
14	diisopropylamine	14%	1:1
15	$tBuNH_2$	27%	1:1
16	2,6-lutidine	—	—
17	imidazole	—	—
18	K_2CO_3	45%	1:1
19	BTMG	6%	1:1
20	DBU	12%	1:1
21	PMP	22%	1:1
22	TEA	27%	1:1
23	TMG	81%	11:1

Through base screening, we found two bases that gave good diastereomeric ratio—DBN (entry 2) and TMG (entry 3). TMG is crucial for the success of the reaction giving a 81% yield and 11:1 (β:α) diastereomeric ratio. At the same time, the temperature of the reaction could be lowered to 70 °C with the use of stronger base.

Table S2

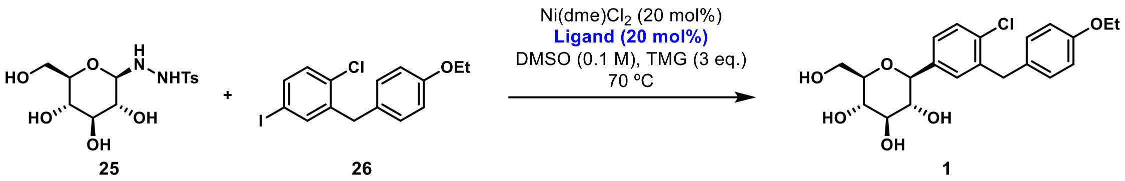

Entry	Ligand	Yield	β:α
1	**L4**	73%	>19:1
2	**L3**	69%	>19:1
3	**L9**	75%	>19:1
4	**L10**	73%	13:1
5	**L11**	71%	11:1
6	**L6**	81%	12:1
7	**L14**	35%	6:1
8	**L20**	54%	18:1
9	**L22**	88%	8:1
10	**L26**	67%	10:1
11	**L27**	67%	>19:1
12	**L28**	30%	4:1
13	**L30**	50%	2:1
14	**L31**	87%	13:1
15	**L1**	88%	>19:1
16	**L2**	41%	9:1
17	**L42**	46%	2:1
18	**L43**	50%	3:1

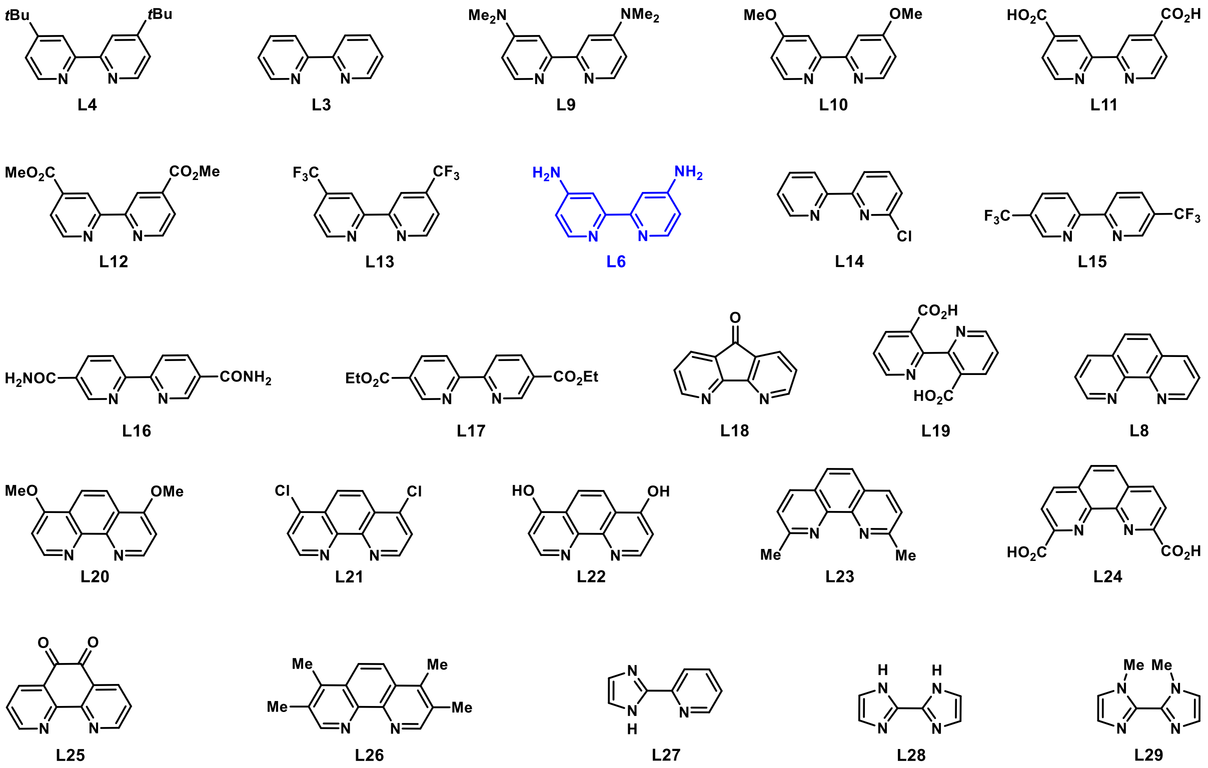

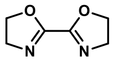
L30

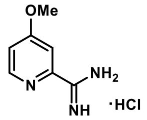
L31

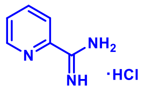
L1

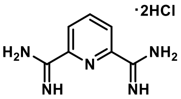
L2

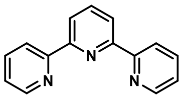
L5

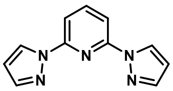
L32

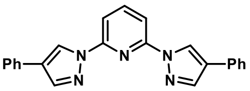
L7

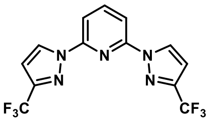
L33

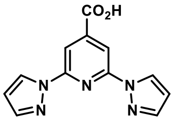
L34

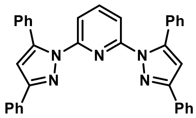
L35

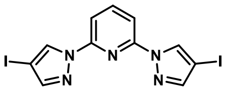
L36

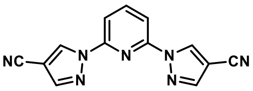
L37

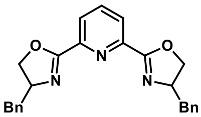
L38

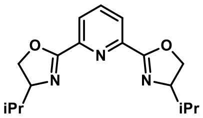
L39

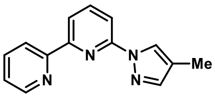
L40

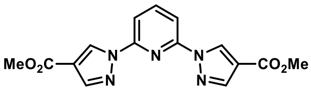
L41

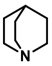
L42

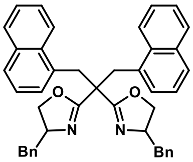
L43

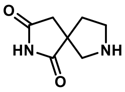
L44

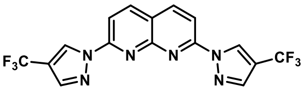
L45

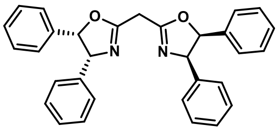
L46

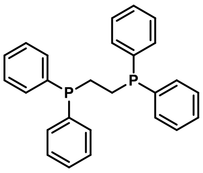
L47

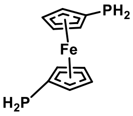
L48

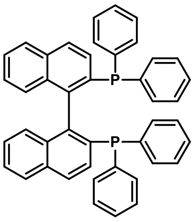
L49

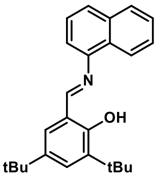
L50

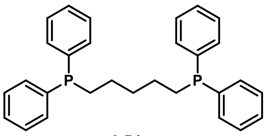
L51

As shown in Tables S2, the bidentate ligands including bipyridine ligands, phenanthroline ligands, and pyridine carboxamidine ligands provided good yield and diastereomeric ratios (Tables S2, entry 6, entry 9, and entry 15). In particular, the pyridine-2-carboxamidine (**L1**) exhibited a promising diastereomeric ratio of >19:1 (β:α) (Tables S2, entry 15). The tridentate ligands do not give products.

Note: Other ligands listed here but not shown on the table either gave trace or no product according to crude NMR.

Table S3

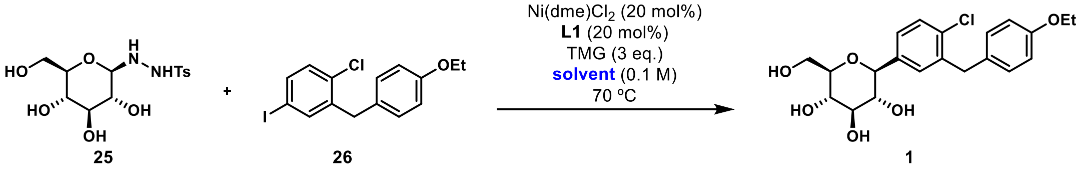

Entry	Variations	Yield	β:α
1	DMSO	88%	>19:1
2	DMF	65%	10:1
3	DMA	62%	12:1
4	NMP	43%	>19:1
5	N-methylformamide	36%	>19:1
6	MeCN:DMSO (4:1)	32%	>19:1
7	N-methylacetamide	—	—
8	sulfolane	—	—
9	acetone	—	—
10	butanone	—	—
11	acetonitrile	—	—
12	glyme	—	—
13	nBuOH	—	—
14	EtOH + DMSO (100eq)	—	—
15	EtOH + H_2O (100eq)	—	—
16	EtOH	—	—
17	MeOH	—	—
18	iPrOH	—	—
19	*t*-amyl-OH	—	—
20	TFE	—	—
21	ethylene glycol	—	—

In Table S3, solvent screening is summarized. DMSO was found to be the solvent giving the best yield and diastereomeric ratio (Table S3, entry 1). DMF could be an alternative solvent providing good yield and diastereomeric ratio (entry 2).

Table S4

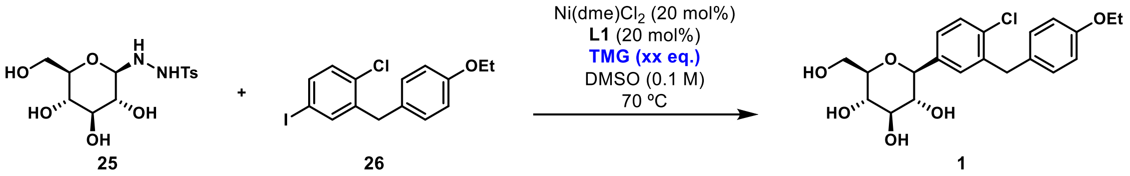

Entry	Variations	Yield	β:α
1	1 eq.	45%	3.5:1
2	2 eq.	76%	9.7:1
3	3 eq.	88%	>19:1
4	4 eq.	62%	>19:1
5	5 eq.	60%	>19:1

Table S4 represents the effect of TMG equivalents. Increasing the equivalents of TMG improves the diastereomeric ratio, but excessive TMG eventually reduces the yield (Table S4, entries 3-5). 3 equivalents of TMG were found to be the optimal stoichiometry (Table S4, entry 3).

Table S5

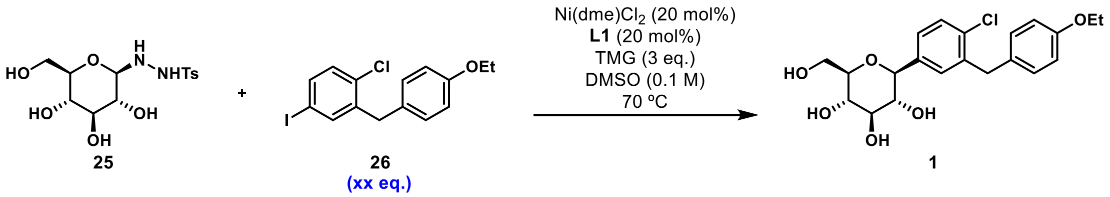

Entry	Variations	Yield	β:α
1	1.0 eq.	64%	>19:1
2	1.2 eq.	71%	>19:1
3	1.5 eq.	88%	>19:1
4	2.0 eq.	83%	>19:1

Table S5 represents the effect of coupling partner equivalents.

Table S6

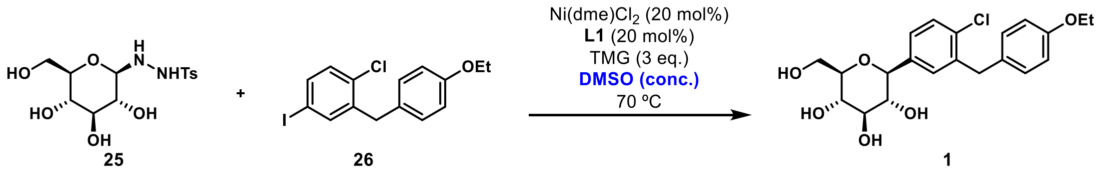

Entry	Variations	Yield	β:α
1	0.1 M	88%	>19:1
2	0.2 M	74%	>19:1
3	0.3 M	74%	>19:1
4	0.4 M	70%	>19:1
5	0.5 M	64%	17:1
6	0.8 M	55%	16:1
7	1.0 M	51%	15:1

Table S6 represents the effect of reaction concentration.

Table S7

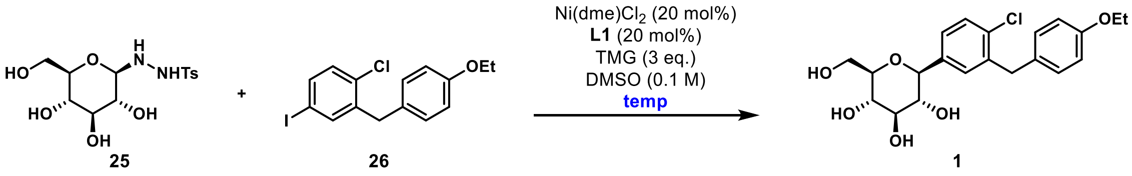

Entry	Variations	Yield	β:α
1	90 ºC	82%	14:1
2	80 ºC	80%	16:1
3	70 ºC	88%	>19:1
4	60 ºC	72%	>19:1
5	50 ºC	64%	>19:1
6	40 ºC	53%	>19:1
7	30 ºC	34%	>19:1

Table S7 represents the effect of reaction temperature. Lower temperatures led to lower yields.

Table S8

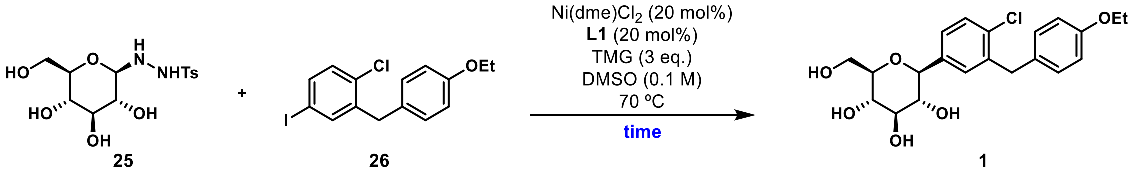

Entry	Variations	Yield	β:α
1	15 min	90%	>19:1
2	30 min	91%	>19:1
3	45 min	92%	>19:1
4	1 hr	88%	>19:1
5	2 hr	90%	>19:1
6	3 hr	85%	>19:1

Table S8 represents the reaction time. The reaction can be done within 15 minutes.

2.2 Stereoretentive RCC-arylation of Bn-protected Glycohydrazides

Table S9

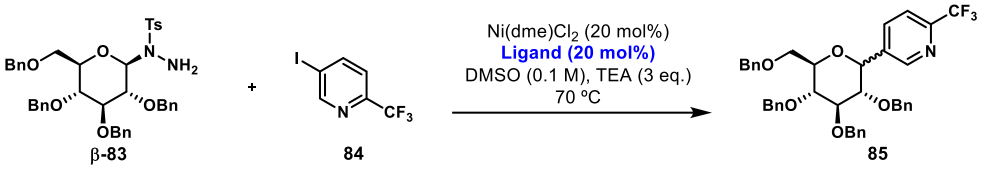

Entry	Ligand	Yield	β:α
1	**L4**	47%	3.1:1
2	**L3**	48%	3.5:1
3	**L5**	12%	1.2:1
4	**L7**	10%	1.4:1
5	**L8**	43%	3.4:1
6	**L9**	39%	2.3:1
7	**L10**	40%	3.1:1
8	**L11**	43%	4.8:1
9	**L12**	68%	5.0:1

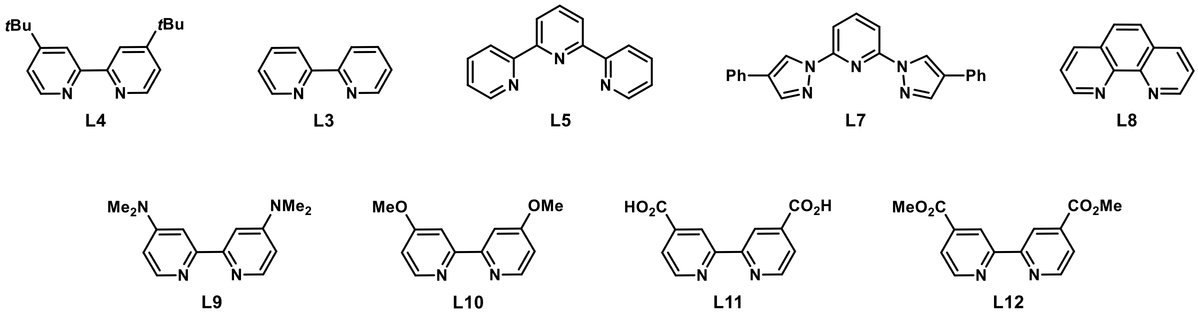

Table S9 shows that tridentate ligands (entry 3 and entry 4) provided both low yield and diastereoselectivity. On the other hand, bidentate ligands gave better yields and diastereomeric ratios. Among the bipyridine ligands, electron-withdrawing ligands (entry 8 and entry 9) are shown to be the optimal ligands for stereoretentive RCC-arylation of Bn-protected glycohydrazides.

2.3 RCC-arylation of C2-C6 Sugar Sulfonylhydrazides

We evaluated the feasibility of the radical cross-coupling reaction of C2-C6 sugar sulfonylhydrazides using glucose derived C4 sulfonyl hydrazide and aryl iodide. The yield and diastereomeric ratio were determined by crude ^{1}H NMR.

Table S10

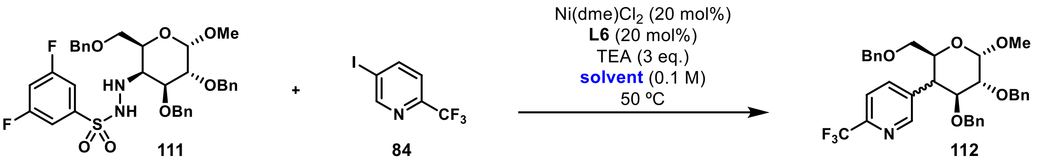

Entry	Variations	Yield	galacto(ga):gluco(g)
1	TFE	—	—
2	DMSO	34%	1.7:1
3	EtOAc	—	—
4	2-methyl THF	—	—
5	1,4-dioxane	—	—
6	tBuOH	—	—
7	toluene	—	—
8	EtOH	44%	2.1:1
9	DMA	24%	1.8:1
10	NMP	20%	1.7:1
11	N-methylacetamide	45%	2.1:1
12	N-methylformamide	36%	2.1:1
13	N-methylacetamide:H_2O (5:1)	—	—
14	amylOH	—	—
15	*i*PrOH	—	—
16	allyl alcohol	—	—
17	glycerol	—	—
18	ethylene glycol	—	—
19	MeCN	—	—
20	HFIP	—	—
21	MeOH	38%	2.1:1
22	THF	—	—
23	DMF	20%	1.5:1

Table S10 shows the preliminary solvent screening for RCC-arylation of glucose-derived C4 sulfonyl hydrazide. Aprotic polar solvents generally gave better results than protic solvents except ethanol gave a 44% yield and methanol gave a 38% yield in this specific case (entry 8 and entry 21). After trying the solvents which gave higher yields in this table on different aryl halides, we eventually decided to proceed with DMSO (entry 2), which gave the highest yields across different sulfonyl hydrazides and aryl halides.

Table S11

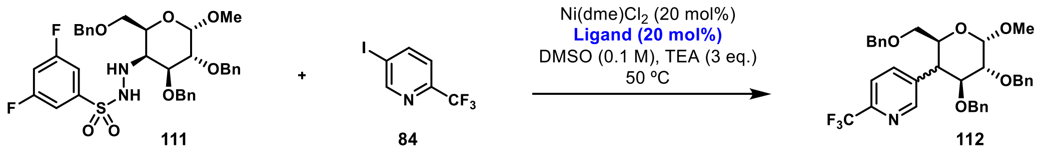

111 + 84 → 112

Entry	Ligand	Yield	ga:g
1	L3	50%	2.9:1
2	L12	18%	1.6:1
3	L10	38%	2.2:1
4	L11	24%	1.3:1
5	L15	20%	1.4:1
6	L16	17%	1.4:1
7	L17	<10%	—
8	L14	14%	1.3:1
9	L52	<10%	—
10	L53	<10%	—
11	L27	<10%	—
12	L29	27%	1.3:1
13	L30	<10%	—
14	L8	52%	3.1:1
15	L21	19%	2:1
16	L55	49%	2.6:1
17	L22	25%	1.1:1
18	L20	30%	1.8:1
19	L26	43%	2.3:1
20	L25	22%	1.8:1
21	L1	31%	1:1
22	L56	<10%	—

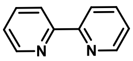
L3
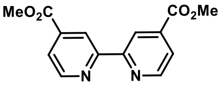
L12
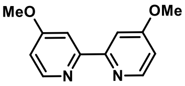
L10
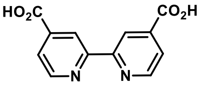
L11
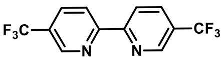
L15
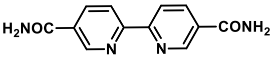
L16
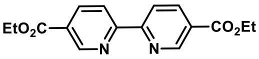
L17
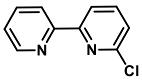
L14
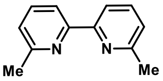
L52
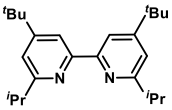
L53
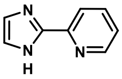
L27
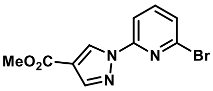
L54
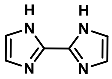
L28
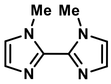
L29
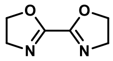
L30
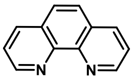
L8
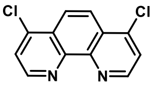
L21
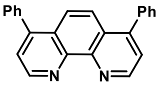
L55
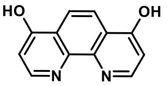
L22
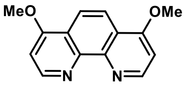
L20
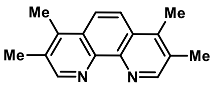
L26
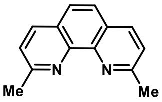
L23
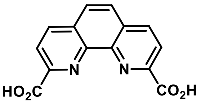
L24
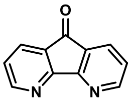
L18
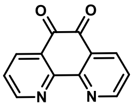
L25

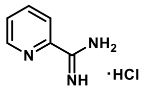

L1

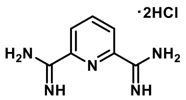

L2

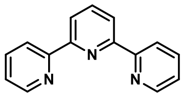

L5

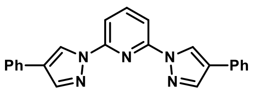

L7

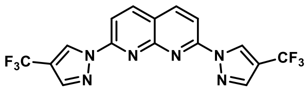

L45

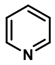

L56

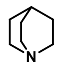

L42

Table S11 shows the ligand effects for RCC-arylation of glucose-derived C4 sulfonyl hydrazide. Similar to what has been observed before, tridentate ligands perform poorly (**L2**/**L5**/**L7**), while bidentate ligands give better yields (entry 1-21). Among bipyridine ligands, the unsubstituted bipyridine ligand (**L3**, entry 1) gave the highest yield 50% (ga:g = 2.9:1). At the same time, the unsubstituted phenanthroline ligand (**L8**, entry 14) gave the highest yield 52% (ga:g = 3.1:1). After testing bpy (**L3**) and 1,10-phen (**L8**) in the coupling reaction with different sugar hydrazide precursors and aryl halides, **L8** gave higher yields in general and was therefore selected for RCC-arylation of glucose/ribose-derived C2-C6 sulfonyl hydrazides.

Note: Other ligands listed here but not shown on the table either gave trace or no product, according to crude NMR.

2.4 RCC-arylation at Unprotected Glucoside C3 Position.

Table S12

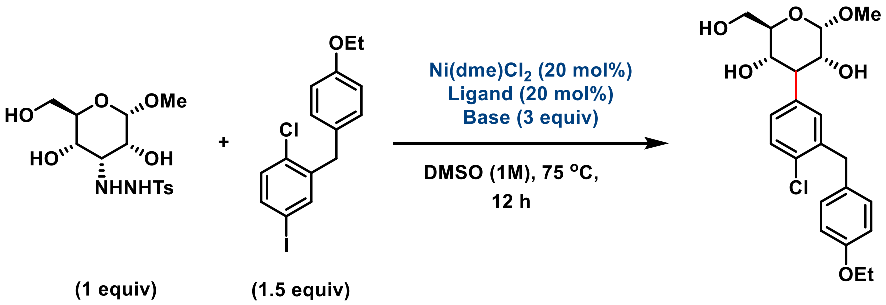

Ligand (20 mol%)	Base (3 equiv)	% Yield (gluco/allo)	Ligand (20 mol%)	Base (3 equiv)	% Yield (gluco/allo)
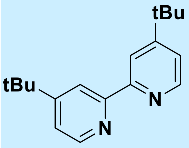	TMG	40% (5:1)	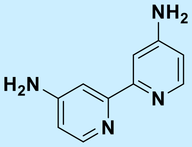	TMG	27% (6:1)
	TEA	39% (>20:1)		TEA	76% (>20:1)
	PMP	33% (>20:1)		PMP	43% (>20:1)
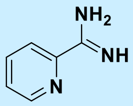	TMG	59 % (5:1)	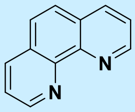	TMG	41% (4:1)
	TEA	88% (>20:1)		TEA	67% (>20:1)
	PMP	83% (>20:1)		PMP	34% (>20:1)

3. Extended Reaction Scope

3.1 PG-free Glucose C1 coupling

Table S13

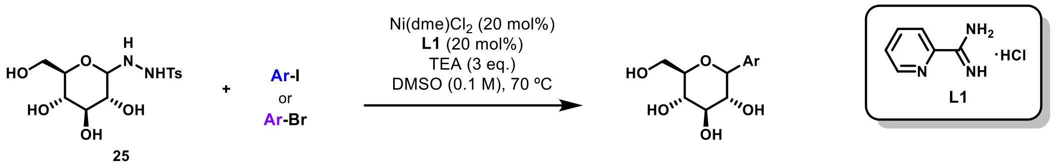

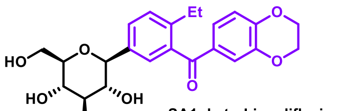

SA1: keto-Licogliflozin
71% (β:α = 10:1)

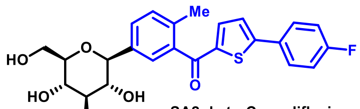

SA2: keto-Canagliflozin
61% (β:α = 10:1)

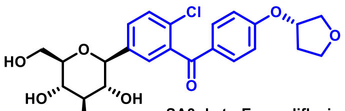

SA3: keto-Empagliflozin
62% (β:α = 7:1)

3.2 RCC-arylation at Protected Glucose C2/C3/C4/C6 positions

Table S14

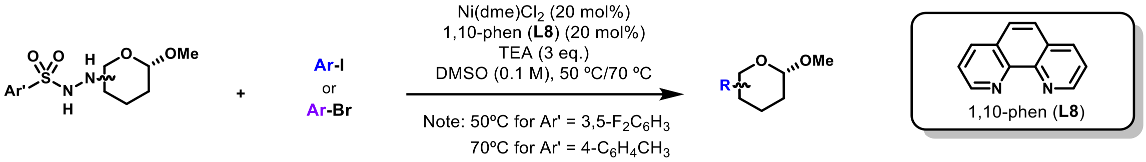

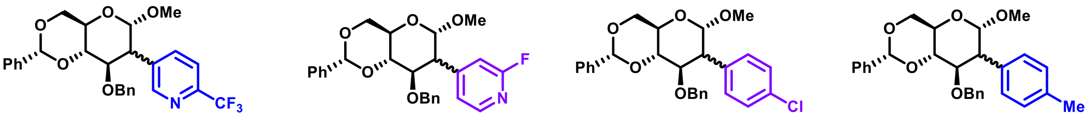

111: 48% (gluco(g):manno(m) = 1.2:1) **102**: 70% (g:m = 1.5:1) **103**: 37% (g:m = 1:1) **104**: 49% (g:m = 1:1)

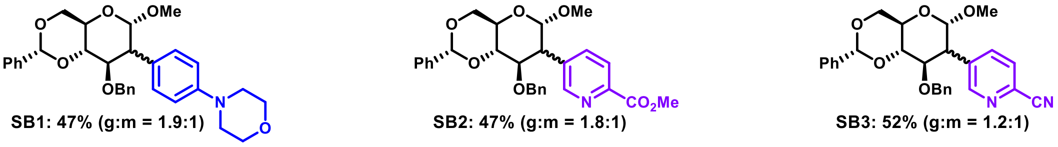

SB1: 47% (g:m = 1.9:1) **SB2**: 47% (g:m = 1.8:1) **SB3**: 52% (g:m = 1.2:1)

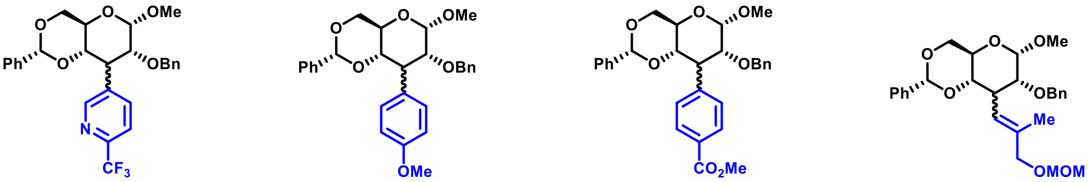

107: 62% (allo(a):gluco(g) = 1.1:1) **108**: 55% (a:g = 1:1.1) **109**: 74% (a:g = 1:1) **110**: 38% (a:g = 1:1.7)

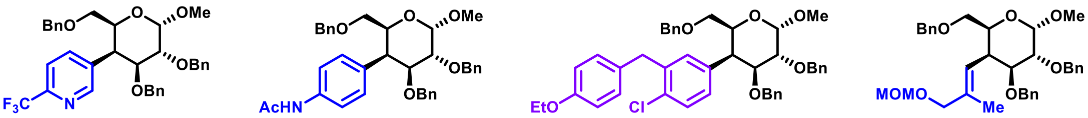

113: 52% (galacto(ga):gluco(g) = 3.1:1) **114**: 58% (ga:g = 3.5:1) **115**: 57% (ga:g = 3.6:1) **116**: 49% (ga:g = 2.5:1)

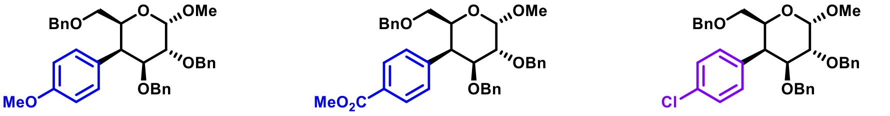

SB4: 50% (ga:g = 2.8:1) **SB5**: 75% (ga:g = 3.1:1) **SB6**: 59% (ga:g = 2.2:1)

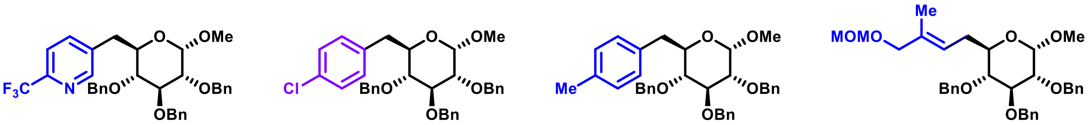

119: 54% **120**: 43% **121**: 42% **122**: 33%

3.3 RCC-arylation at Protected Ribose and 2-Deoxyribose C2/C3/C5 position

Table S15

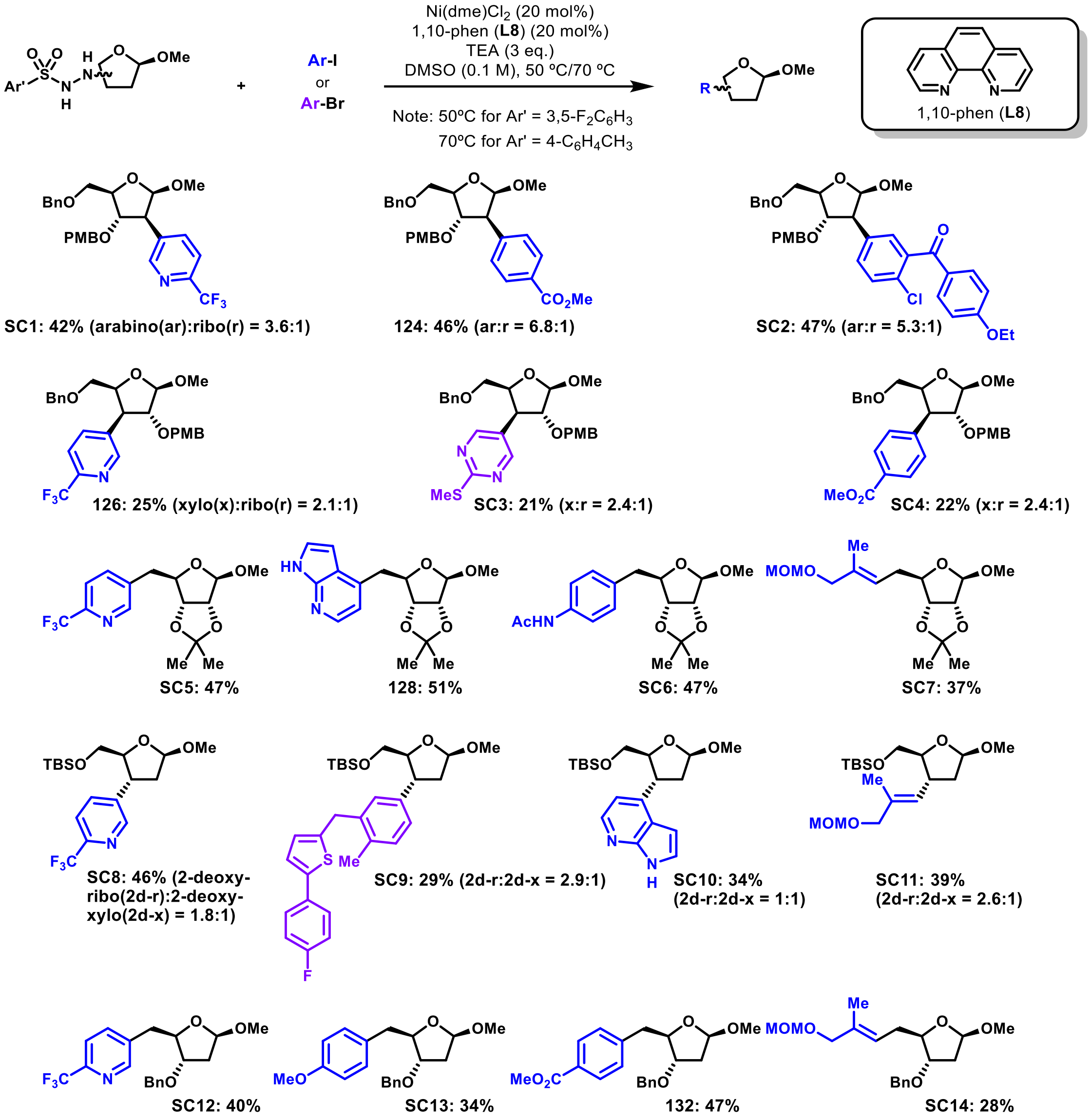

3.4 RCC-arylation at Bn-protected Ribose C1 position

Table S16

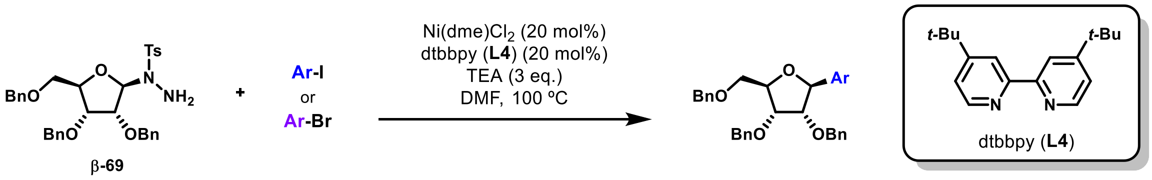

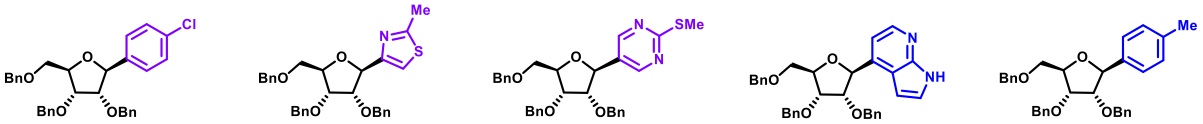

139: 64% (β:α > 19:1) **140**: 41% (β:α > 19:1) **141**: 68% (β:α = 10.7:1) **142**: 60% (β:α = 17.7:1) **SD1**: 50% (β:α > 19:1)

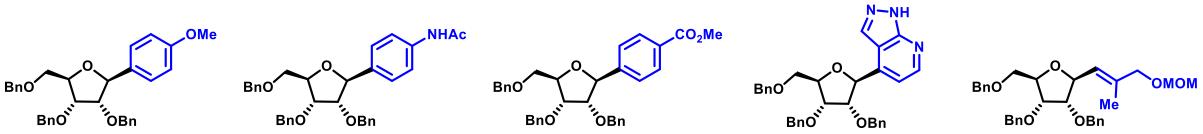

SD2: 48% (β:α > 19:1) **SD3**: 42% (β:α> 19:1) **SD4**: 54% (β:α > 19:1) **SD5**: 52% (β:α > 19:1) **SD6**: 36% (β:α > 19:1)

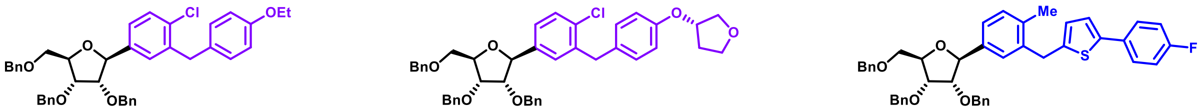

SD7: 56% (β:α > 19:1) **SD8**: 64% (β:α > 19:1) **SD9**:44% (β:α > 19:1)

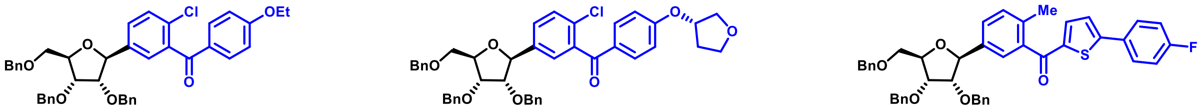

SD10: 52% (β:α = 14.4:1) **SD11**: 62% (β:α = 15.9:1) **SD12**: 55% (β:α = 11.2:1)

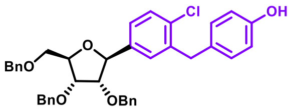

SD13: 59% (β:α > 19:1)

3.5 RCC-arylation at Unprotected Glucoside C3 Position

Table S17

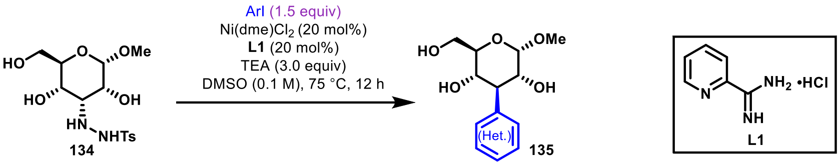

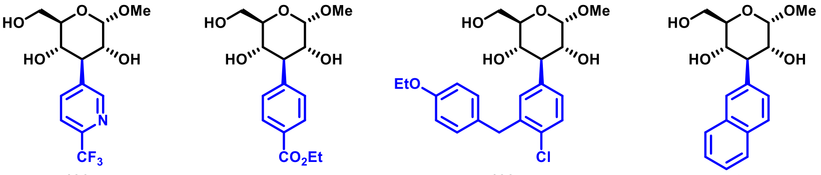

136	137	138	SL4
55% (g:a > 19:1)	62% (g:a > 19:1)	68% (g:a > 19:1)	74% (g:a > 19:1)

4. Synthetic Procedures

4.1 General Synthetic Procedures for native sugar C1-hydrazide

General Procedure A1:

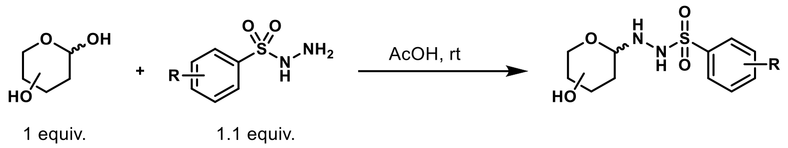

In a dry vial, sugar (5.0 mmol, 1 equiv.) and sulfonylhydrazide (5.5 mmol, 1.1 equiv.) were added, followed by AcOH (25 mL). The reaction mixture was stirred for 6 h at room temperature. Upon completion of the reaction, as indicated by TLC, the solvent was removed under reduced pressure. The resulting oily residue was stirred while diethyl ether (50 mL) was added, leading to the precipitation of a white solid. The mixture was stirred for an additional 20 minutes, then filtered and washed with diethyl ether (3×20 mL), affording the sugar hydrazide as a white solid.

General Procedure A2:

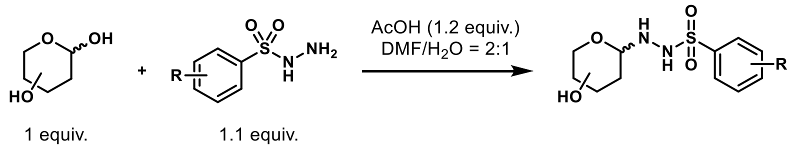

To a stirred solution of sugar (5.0 mmol, 1 equiv.) and sulfonylhydrazide (5.5 mmol, 1.1 equiv.) in DMF/H_2O = 2:1 was added AcOH (6.0 mmol, 0.34 mL, 1.2 equiv). The reaction mixture was heated to 37 °C and stirred for 1.5 days. Upon completion of the reaction, as indicated by TLC, the reaction mixture was poured into Et_2O (200 mL) and stirred for 18 h, leading to the precipitation of a white solid. The precipitate was filtered and washed with diethyl ether (3×20 mL), affording the sugar hydrazide as a white solid.

4.2 General Synthetic Procedure for cross-coupling of glucose C1 hydrazides and aryl halides

General Procedure B:

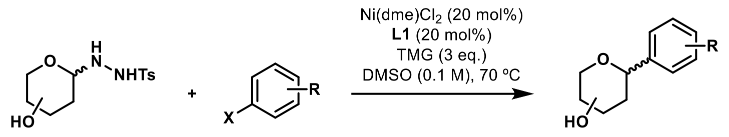

To a reaction tube with a stir bar were added sugar hydrazide (0.1 mmol, 1.0 equiv.), aryl halide (0.15 mmol, 1.5 equiv.), Ni(dme)Cl_2 (20 mol%, 0.2 equiv.), and Ligand (20 mol%, 0.2 equiv., **L1** for aryl iodide and di-NH_2-bpy (**L6**) for aryl bromide). The tube was capped with a septa cap, then evacuated and backfilled with argon three times. Pre-degassed DMSO (1 mL) was added and the mixture was stirred for 1 minute, followed by the addition of TMG (or TEA) (0.3 mmol, 3.0 equiv.). The reaction mixture was heated to 70 °C and stirred overnight. After the reaction was completed, the reaction mixture was directly purified by a C18 column (RediSep Rf reversed phase C18 column 30g, eluting with a gradient flow over 12 minutes of 100:0 A/B to 0:100 A/B, 214 nm UV detection). Solvent A is H_2O with 0.1% formic acid; solvent B is MeCN. At a flow rate of 35 mL/min, the desired product has a retention time of 6-8 minutes.

Note: The purified products can be further purified using preparative HPLC for separating the diastereomers.

4.2.1 Graphical Experimental Guide for General Procedure B

*Photos were taken of the coupling between glucose hydrazide **25** and 1-chloro-2-[(4-ethoxyphenyl)methyl]-4-iodbenzene (aryl iodide) **26**.*

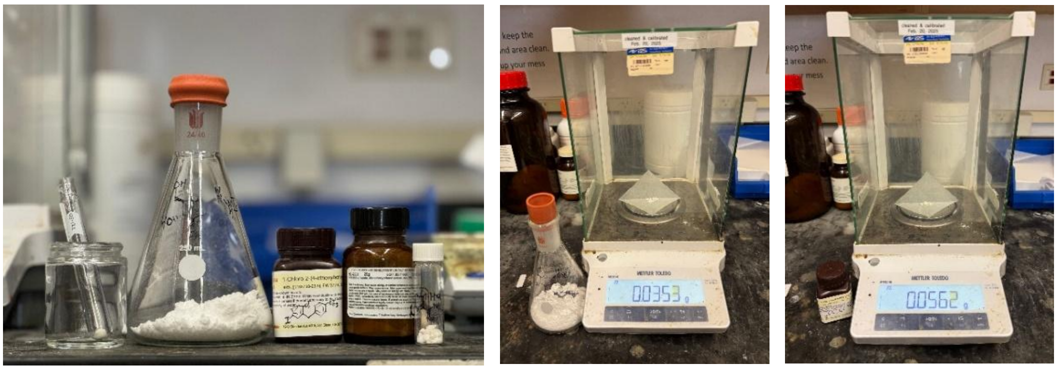

(Left) All reagents (glucose hydrazide **25**, aryl iodide **26**, Ni(dme)Cl_2, and **L1**) for the reaction and a clean tube with a stir bar. **(Center)** Glucose hydrazide **25** was weighed out. (Right) Aryl iodide **26** was weighed out.

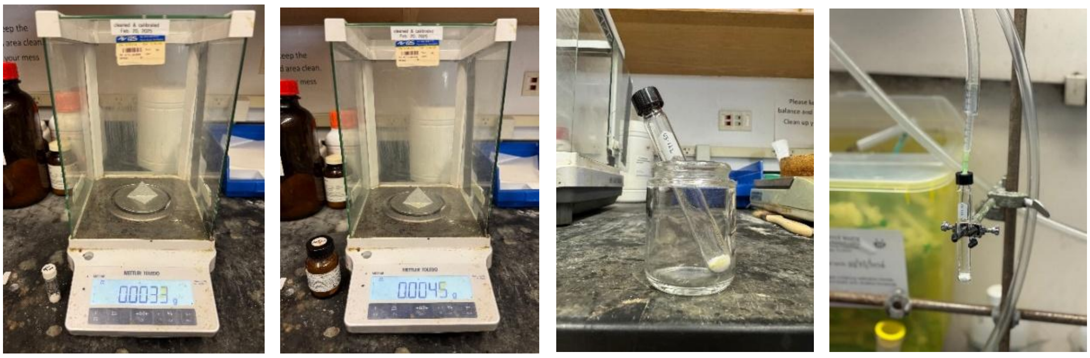

(Left) L1 was weighed out. **(Center Left)** Ni(dme)Cl2 was weight out. **(Center Right)** The reaction tube was capped. **(Right)** The reaction tube was evacuated on a Schlenk line.

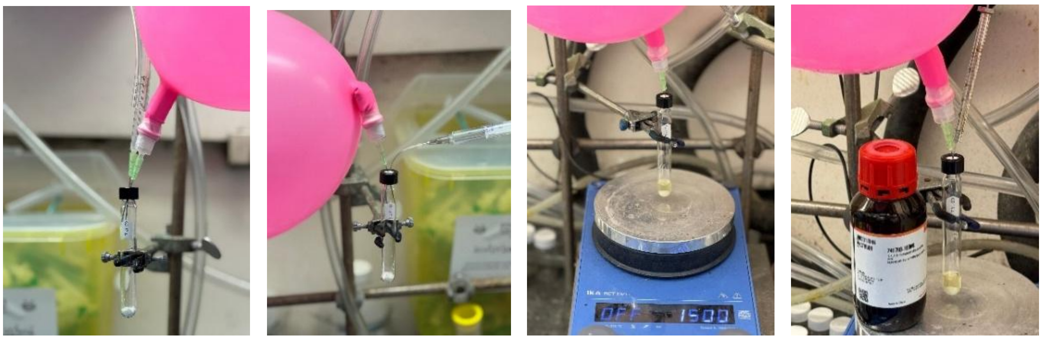

(Left) The reaction tube was refilled with argon. Evacuation and back-filling were repeated three times. **(Center Left)** Pre-degassed DMSO (1 mL) was added via syringe under an argon atmosphere. **(Center Right)** Reaction mixture after DMSO addition.

(Right) TMG (38 μL) was added via micro syringe.

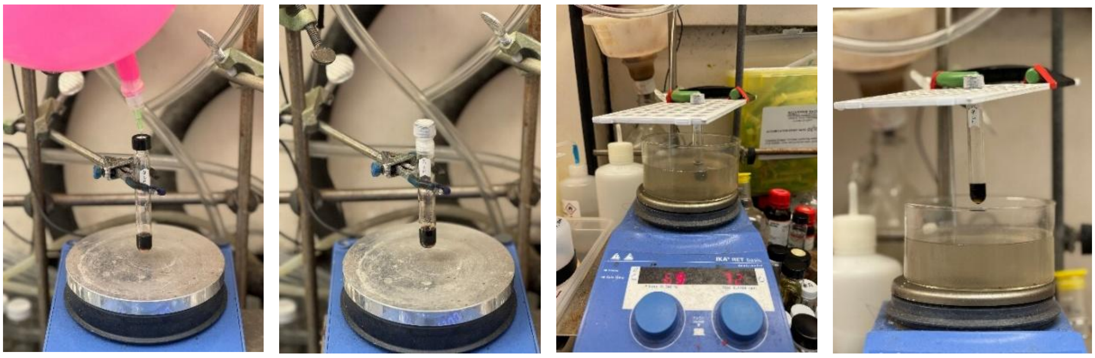

(Left) The reaction mixture after the addition of TMG. **(Center Left)** The reaction tube was sealed with parafilm. **(Center Right)** The reaction mixture was submerged in a preheated oil bath at 70 °C. **(Right)** The reaction mixture after the reaction was done.

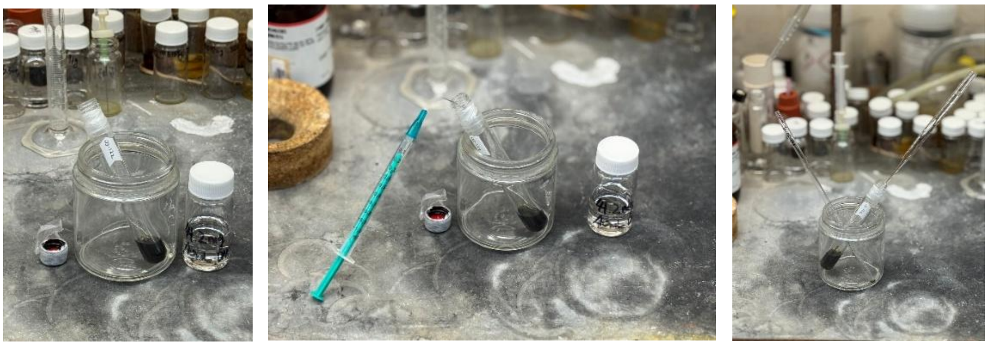

(Left) 1,3,5-trimethoxybenzene was used as internal standard for crude NMR yield determination. **(Center)** 0.3 mL of internal standard stock solution (41.2 mg/10 mL DMSO) was added to the reaction mixture. **(Right)** A small amount of crude mixture was put into the NMR tube and ready for crude NMR yield determination.

4.3 General Synthetic Procedure for Bn-protected sugar C1-hydrazide

General Procedure C1: Mitsunobu reaction procedure

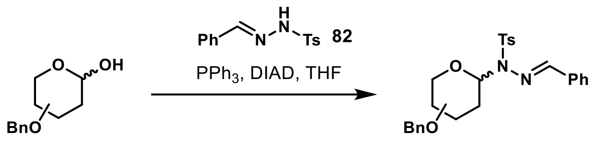

To a stirred solution of the triphenylphosphine (2.0 equiv.) in THF (0.2 M) was added diisopropyl azodicarboxylate (DIAD, 1.8 equiv.) at 0 °C. The reaction mixture was stirred at 0 °C for 10 minutes, after which a solution of the benzyl-protected sugar (1.0 equiv.) and hydrazide reagent **82** (1.8 equiv.) in THF (0.2 M) was added dropwise over 10 min at the same temperature. Upon completion of the reaction (monitored by TLC), the reaction mixture was concentrated and the residue was purified by flash column chromatography (silica gel, Hexane/EtOAc 20:1 to 2:1) to afford the desired product.

General Procedure C2: Yu glycosylation procedure

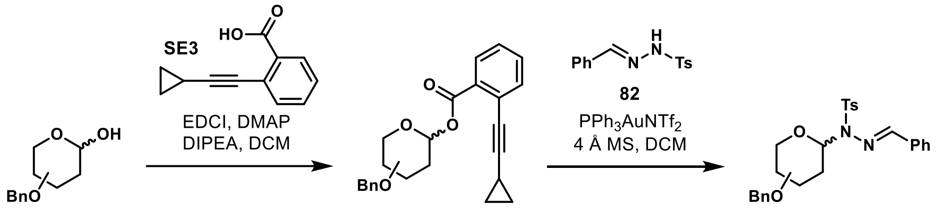

To a stirred solution of benzyl-protected sugar (1.0 equiv), acid **SE3** (1.8 equiv), and DMAP (1.0 equiv) in DCM (0.1 mL) at 0 °C were added DIPEA (2.0 equiv) and EDCI (1.8 equiv). The reaction mixture was then allowed to warm to room temperature and stirred for 10 h. Upon completion of the reaction (monitored by TLC), the reaction mixture was purified directly by flash column chromatography (silica gel, Hexane/EtOAc 20:1 to 2:1) to afford the Yu glycosylation precursor.

To a stirred solution of synthesized ester (1.0 equiv.), hydrazide reagent **82** (1.5 equiv.) and 4 Å MS (1g/mmol synthesized ester starting material) in DCM (100 mL) was added PPh_3AuNTf_2 (0.15 equiv.) at room temperature. The reaction mixture was stirred for 1 h and then directly purified by flash column chromatography (silica gel, Hexane/EtOAc 20:1 to 2:1) to afford the desired product.

*Note: The acid **SE3** and gold catalyst (PPh_3AuNTf_2) were prepared according to the literature procedure.* [1]

General Procedure D: deprotection

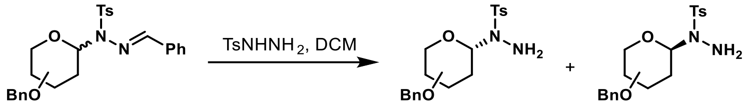

To a stirred solution of hydrazone (1.0 equiv.) in DCM (0.05 mL) was added TsNHNH2 (5.0 equiv.) at room temperature. Then the reaction mixture was heated to 30 °C for 12 h. Upon completion of the reaction (monitored by TLC), the reaction mixture was purified directly by flash column chromatography (silica gel, DCM/EtOAc 200:1 to 20:1) to afford the hydrazide.

4.3.1 Synthesis of glucose C1 hydrazide

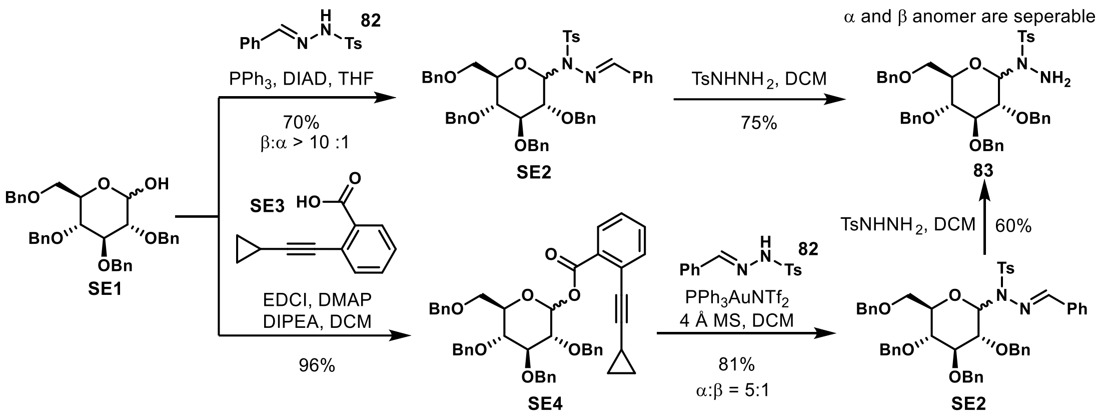

Following **General Procedures C1 and D**, the reaction was conducted on a 4.0 mmol scale. The crude product was purified by flash column chromatography (silica gel, DCM/EtOAc 200:1 to 20:1) to afford the desired hydrazide **82** (53% yield, 2 steps, α:β < 1:10) as a colorless oil.

Following **General Procedures C2 and D**, the reaction was conducted on a 4.0 mmol scale. The crude product was purified by flash column chromatography (silica gel, DCM/EtOAc 200:1 to 20:1) to afford the desired hydrazide **82** (47% yield, 3 steps, α:β = 5:1) as a colorless oil.

Note: The α and β anomers were separable by flash column chromatography (silica gel, DCM/EtOAc 200:1 to 20:1). ***SE1*** *is commercially available.*

4.3.2 Synthesis of 2-deoxy-glucose C1 hydrazide

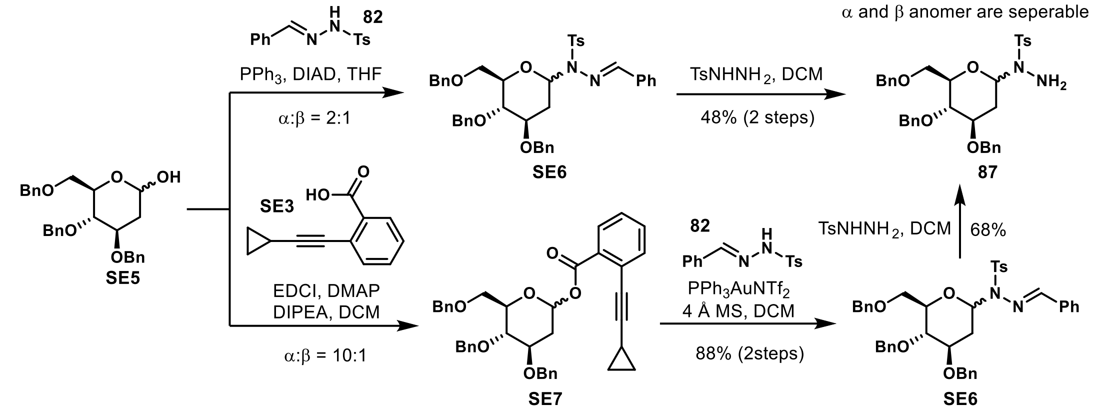

Following **General Procedures C1 and D**, the reaction was conducted on a 2.0 mmol scale. The crude product was purified by flash column chromatography (silica gel, DCM/EtOAc 200:1 to 20:1) to afford the desired hydrazide **87** (48% yield, 2 steps, α:β = 2:1) as a colorless oil.

Following **General Procedures C2 and D**, the reaction was conducted on a 6.0 mmol scale. The crude product was purified by flash column chromatography (silica gel, DCM/EtOAc 200:1 to 20:1) to afford the desired hydrazide **87** (60% yield, 3 steps, α:β = 10:1) as a colorless oil.

Note: The α and β anomers were separable by flash column chromatography (silica gel, DCM/EtOAc 200:1 to 20:1). ***SE5*** *was prepared according to literature procedure.*[2]

4.3.3 Synthesis of ribose C1 hydrazide

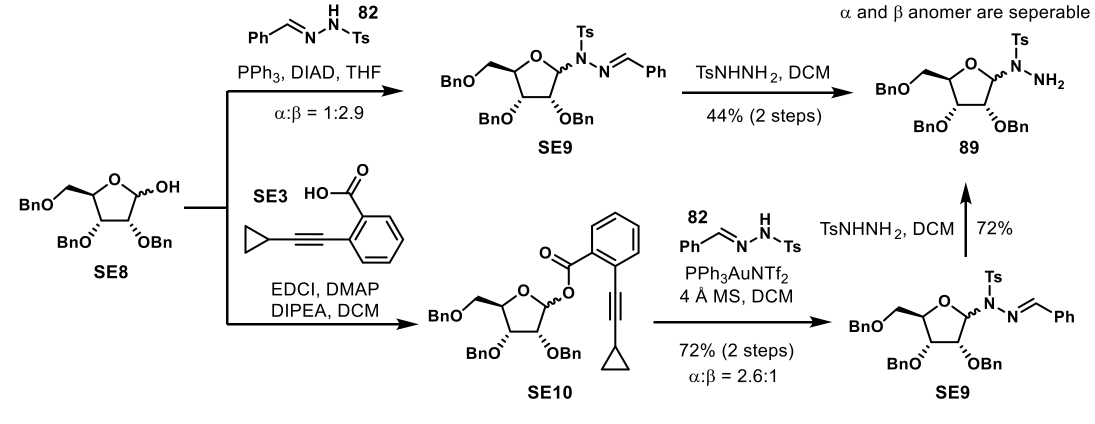

Following **General Procedures C1 and D**, the reaction was conducted on a 11.9 mmol scale. The crude product was purified by flash column chromatography (silica gel, DCM/EtOAc 200:1 to 20:1) to afford the desired hydrazide **69** (44% yield, 2 steps,

α:β =1:2.9) as a colorless oil.

Following **General Procedures C2 and D**, the reaction was conducted on a 5.0 mmol scale. The crude product was purified by flash column chromatography (silica gel, DCM/EtOAc 200:1 to 20:1) to afford the desired hydrazide **89** (47% yield, 3 steps, α:β = 2.6:1) as a colorless oil.

Note: The α and β anomers were separable by flash column chromatography (silica gel, Hexane/ether 50:1 to 1:1). ***SE8*** *is commercially available.*

4.3.4 Synthesis of 2-deoxy-ribose C1 hydrazide

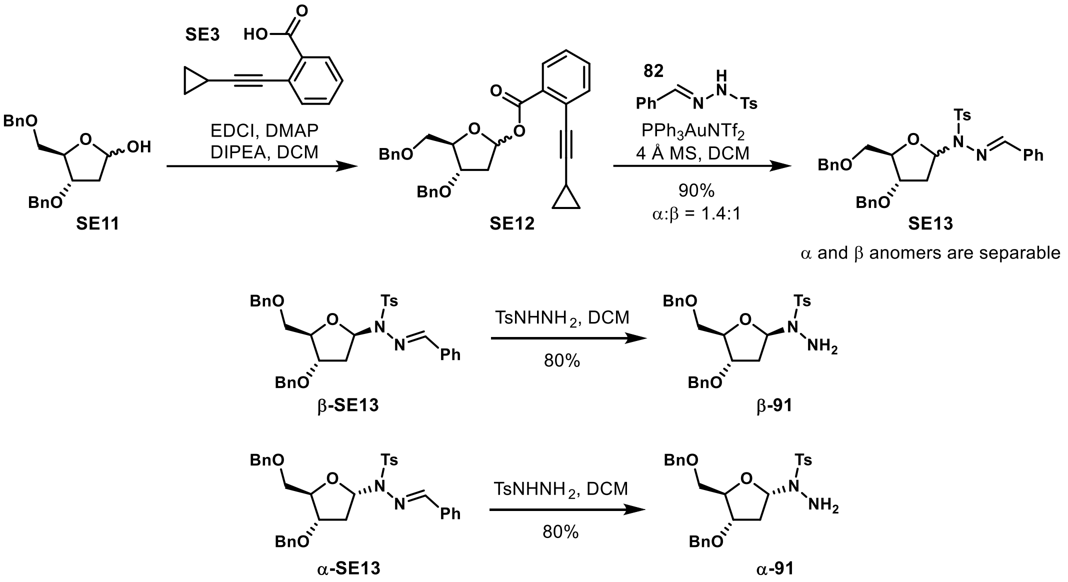

Following **General Procedures C2**, the reaction was conducted on a 5.0 mmol scale. The crude product was purified by flash column chromatography (silica gel, DCM/EtOAc 200:1 to 20:1) to afford α and β anomer **SE13** (90% yield, 2 steps, α:β = 1.4:1), which were separable.

Starting from the **α-SE13** and **β-SE13** respectively, the reactions were carried out on a 1.0 mmol scale following **General Procedures D**. The crude products were purified by flash column chromatography (silica gel, DCM/EtOAc 200:1 to 20:1) to afford the desired hydrazide **α-91** and **β-91** (80% yield) respectively.

Note: ***SE11*** *was prepared according to the literature procedure.*[3] *The hydrazide* ***71*** *was unstable on silica gel and underwent epimerization upon purification. The addition of* Et_3N *to the silica gel effectively suppressed epimerization and stabilized the product*

during chromatography.

4.4 General Synthetic Procedures for stereoretentive cross-coupling of protected sugar C1-hydrazides and aryl halides

General Procedure E:

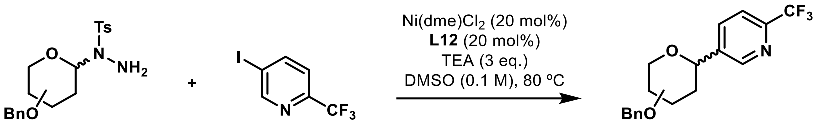

To a reaction tube equipped with a stir bar were added sugar hydrazide (0.1 mmol, 1.0 equiv.), aryl halide (0.2 mmol, 2.0 equiv.), Ni(dme)Cl_2 (0.2 mmol, 0.2 equiv.), and **L12** (0.2 mmol, 0.2 equiv.). The tube was capped with a septum cap, then evacuated and backfilled with argon three times. Pre-degassed DMSO (1 mL) was added and the mixture was stirred for 1 minute, followed by the addition of TEA (0.3 mmol, 3.0 equiv.). The reaction mixture was heated to 80 °C and stirred overnight. After the reaction was completed, water (5 mL) was added. The aqueous phase was extracted with EtOAc (3 x 5 mL). The combined organic phase was washed with brine (2 mL), dried over Na_2SO_4, and concentrated under reduced pressure. The crude residue was purified by flash column chromatography (silica gel, hexane/EtOAc 50:1 to 1:1) to afford the desired product as a colorless oil.

4.4.1 Graphical Experimental Guide for General Procedure E

*Photos were taken of the coupling between glucose branched hydrazide **β-83** and 5-iodo-2-(trifluoromethyl)pyridine (aryl iodide) **84**.*

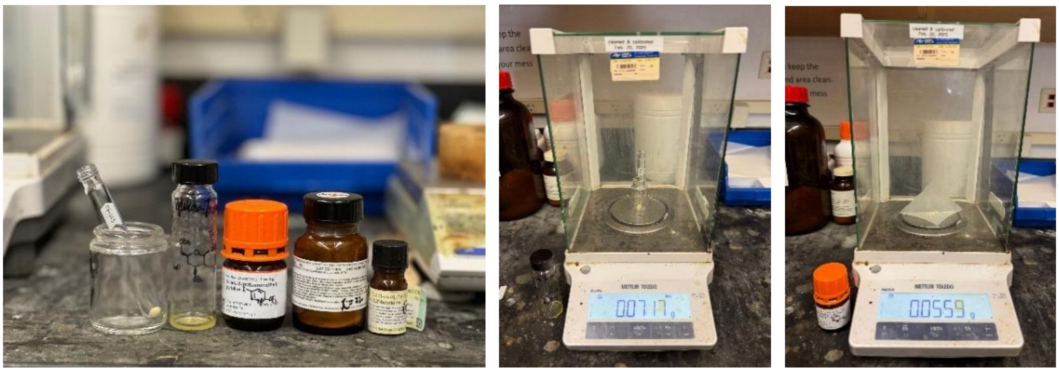

(Left) All reagents (glucose branched hydrazide **β-83**, aryl iodide **84**, $Ni(dme)Cl_2$, and **L12**) for the reaction and a clean tube with a stir bar. **(Center)** Glucose branched hydrazide **β-83** was weighed out. (Right) Aryl iodide **84** was weighed out.

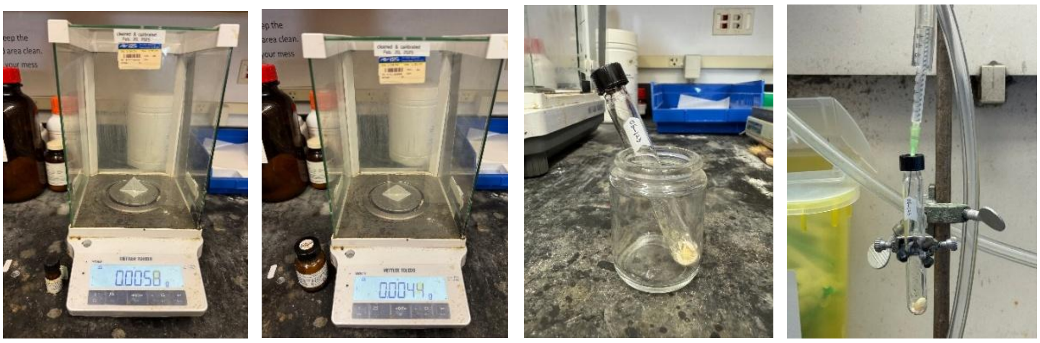

(Left) L12 was weighed out. **(Center Left)** $Ni(dme)Cl_2$ was weight out. **(Center Right)** The reaction tube was capped. **(Right)** The reaction tube was evacuated on a Schlenk line.

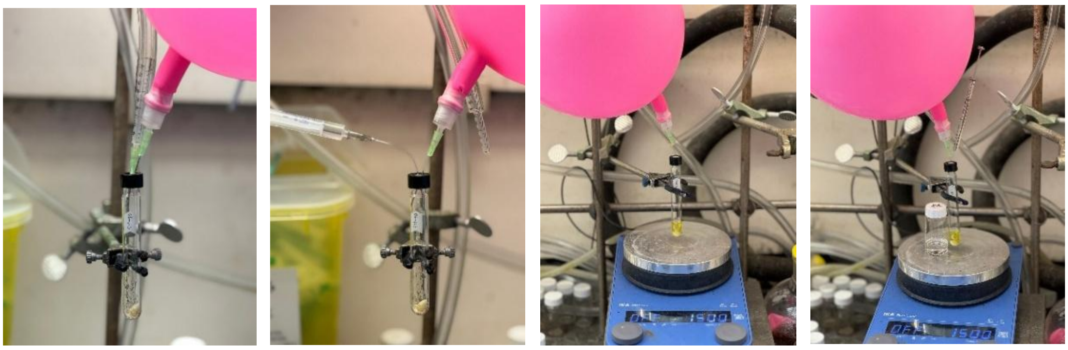

(Left) The reaction tube was refilled with argon. Evacuation and back-filling were repeated three times. **(Center Left)** Pre-degassed DMSO (1 mL) was added via syringe under an argon atmosphere. **(Center Right)** Reaction mixture after DMSO addition.

(Right) TEA (42 μL) was added via micro syringe.

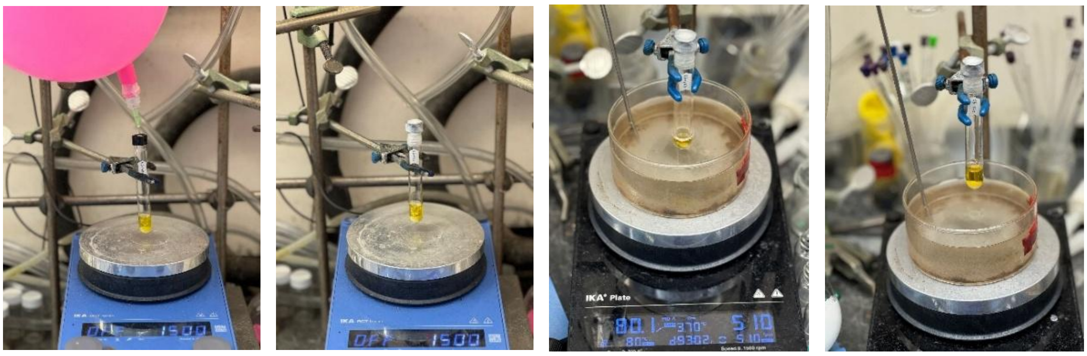

(Left) The reaction mixture after the addition of TEA. **(Center Left)** The reaction tube was sealed with parafilm. **(Center Right)** The reaction mixture was submerged in a preheated oil bath at 80 °C. **(Right)** The reaction mixture after the reaction was done.

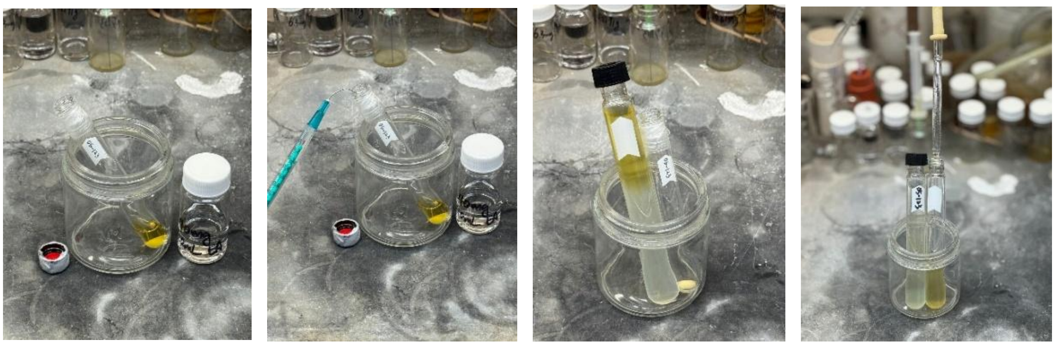

(Left) 1,3,5-trimethoxybenzene was used as internal standard for crude NMR yield determination. **(Center Left)** 0.3 mL of internal standard stock solution (40 mg/10 mL EtOAc) was added to the reaction mixture. **(Center Right)** The reaction mixture was diluted with EtOAc and H_2O. **(Right)** The aqueous phase was extracted with EtOAc three times.

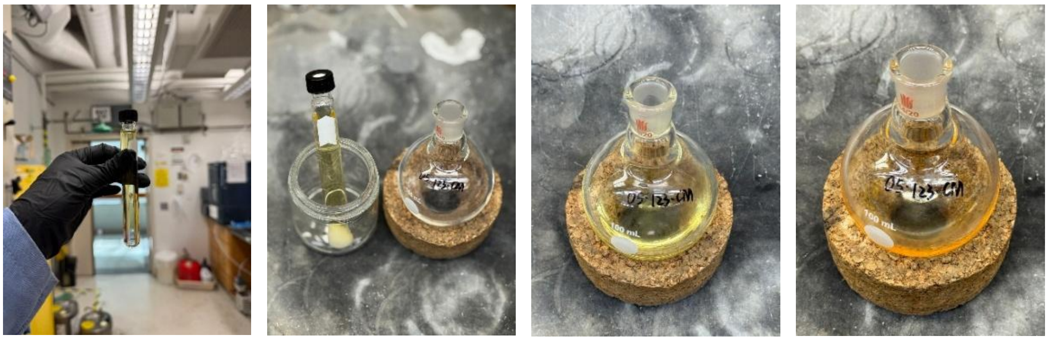

(Left) The combined organic phase was washed with saturated brine solution. **(Center Left)** The organic phase was dried over Na_2SO_4. **(Center Right)** The organic phase was transferred to a 100 mL round-bottom flask. **(Right)** The concentrated crude mixture.

4.5 Synthetic Procedures for C2 to C6 sugar hydrazide

General Procedure F1:

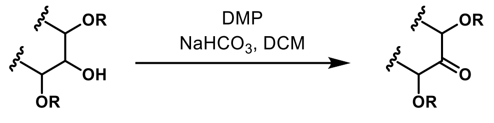

To a stirred solution of alcohol **SF1** (1.0 equiv.) in DCM (0.05M) was added $NaHCO_3$ (10.0 equiv.) and DMP (2.0 equiv.) sequentially at room temperature, and the reaction mixture was stirred for 2 h. Upon completion (monitored by TLC), the reaction mixture was directly purified by flash column chromatography (silica gel, Hexane/EtOAc 20:1 to 1:1) to afford the ketone **SF2**.

General Procedure F2:

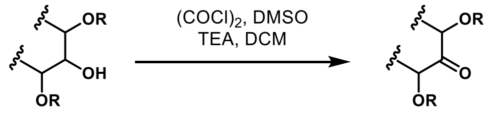

To a stirred solution of oxalyl chloride (2.0 equiv.) in DCM (0.5 M) at –78 °C was added DMSO (4.0 equiv.). After 10 min, a solution of the alcohol **SF1** (1.0 equiv.) in DCM (1 M) was added dropwise, and the reaction mixture was stirred at –78 °C for 1 h. Then, Et_3N (5.0 equiv.) was then added, and the reaction mixture was allowed to warm to 0 °C and stirred for an additional 30 min. The reaction was quenched with saturated aqueous $NaHCO_3$ (50 mL) and extracted with DCM (3×50 mL). The combined organic layers were washed with brine, dried over Na_2SO_4, filtered, and concentrated under reduced pressure. The crude SF2 was used directly in the subsequent step without further purification.

General Procedure G1:

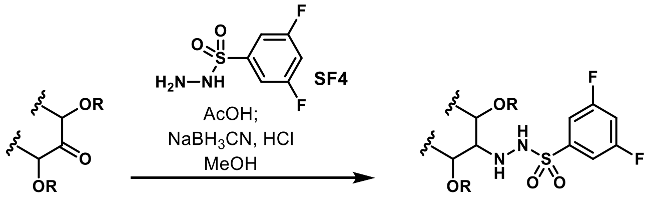

To a stirred solution of the above ketone **SF2** (1.0 equiv.) in AcOH (0.1 M) was added 3,5-difluorobenzenesulfonohydrazide **SF4** (1.2 equiv.) at room temperature. After stirring for 1 h at room temperature, the reaction mixture was concentrated to remove AcOH. The resulting residue was dissolved in MeOH (0.1), and $NaCNBH_3$ (1.0 equiv.) and HCl (4 M in dioxane, 1.5 equiv.) were added sequentially at 0 °C. The reaction mixture was stirred at 0 °C for 2 h, then quenched with saturated aqueous $NaHCO_3$ solution (50 mL), followed by the addition of H_2O (50 mL), and extracted with EtOAc (3 × 100 mL). The combined organic layers were washed with brine (50 mL), dried over Na_2SO_4, filtered, and concentrated under reduced pressure. The residue was purified by flash column chromatography (silica gel, hexane/EtOAc 20:1 to 1:1) to afford the desired hydrazide **SF3**.

General Procedure G2:

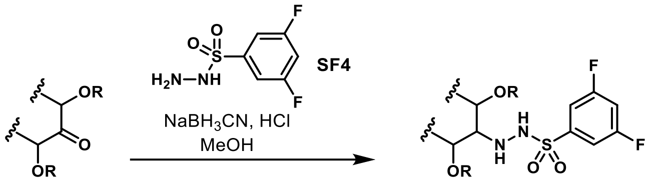

To a stirred solution of the ketone **SF2** (1.0 equiv.) in MeOH (0.1 M) was added 3,5-difluorobenzenesulfonohydrazide **SF4** (1.2 equiv.) at room temperature. After stirring for 1 h at room temperature, the reaction mixture was cooled to 0 °C, and $NaCNBH_3$ (2.0 equiv) and HCl (4 M in dioxane, 1.5 equiv) were added sequentially. The reaction mixture was stirred at 0 °C for 2 h, then quenched with saturated aqueous $NaHCO_3$ solution (50 mL), followed by the addition of H_2O (50 mL). The mixture was extracted with EtOAc (3×100 mL). The combined organic layers were washed with brine (50 mL), dried over Na_2SO_4, filtered, and concentrated under reduced pressure.

The residue was purified by flash column chromatography (silica gel, hexane/EtOAc 20:1 to 1:1) to afford the desired hydrazide **SF3**.

4.5.1 Synthesis of glucose C2 hydrazide

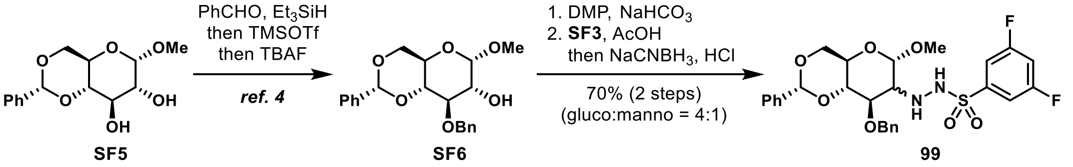

Following **General Procedures F1 and G1**, the reaction was conducted on a 10.7 mmol scale. The crude product was purified by flash column chromatography (silica gel, hexane/EtOAc 20:1 to 1:1) to afford the desired hydrazide **99** (4.2 g, 70% yield, 2 steps, gluco:manno = 4:1) as a white solid.

*Note: **SF6** was prepared according to the literature procedure.* [4]

4.5.2 Synthesis of glucose C3 hydrazide

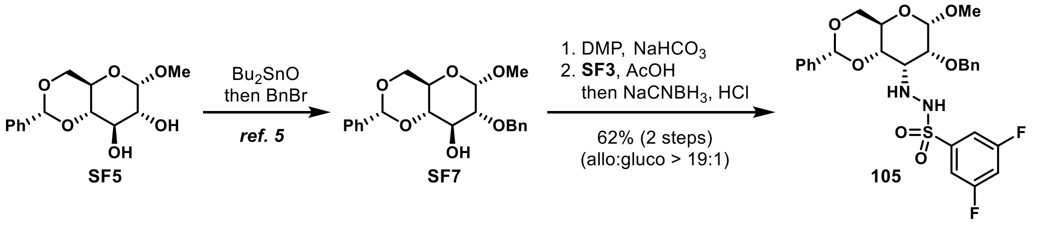

Following **General Procedures F1 and G1**, the reaction was conducted on a 12.7 mmol scale. The crude product was purified by flash column chromatography (silica gel, hexane/EtOAc 20:1 to 1:1) to afford the desired hydrazide **105** (4.4 g, 62% yield, 2 steps, allo:gluco > 19:1) as a white solid.

*Note: **SF7** was prepared according to the literature procedure.* [5]

4.5.3 Synthesis of glucose C4 hydrazide

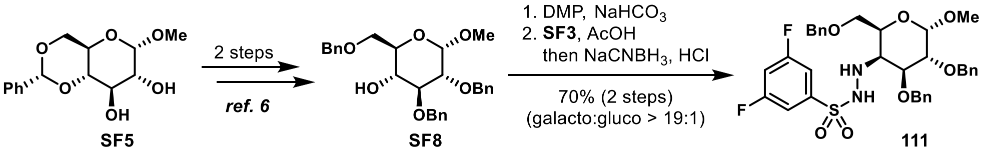

Following **General Procedures F1 and G1**, the reaction was conducted on a 12.3

mmol scale. The crude product was purified by flash column chromatography (silica gel, hexane/EtOAc 20:1 to 1:1) to afford the desired hydrazide **111** (5.6 g, 81% yield, 2 steps galacto:gluco > 19:1) as a white solid.

*Note: **SF8** was prepared according to the literature procedure.* [6]

4.5.4 Synthesis of glucose C6 hydrazide

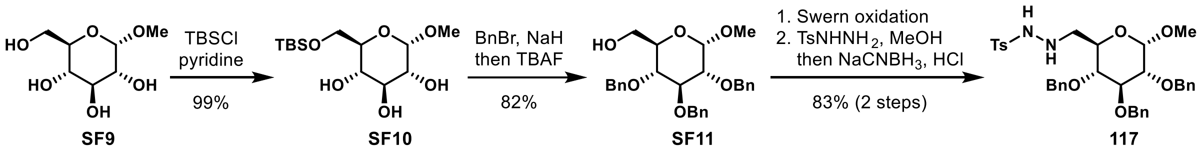

Preparation of the Primary Alcohol **SF11** (Two-Step Sequence):

Step 1. TBS Protection:

To a stirred solution of the alcohol **SF9** (5.82 g, 30 mmol, 1.0 equiv.) in pyridine (12 mL) at 0 °C was added TBSCl (4.5 g, 30 mmol, 1.0 equiv.). The reaction mixture was allowed to warm to room temperature and stirred for 2 h. The reaction was quenched with saturated aqueous $CuSO_4$ solution to make a clear blue solution and extracted with EtOAc (3×50 mL). The combined organic layers were dried over Na_2SO_4, filtered, and concentrated under reduced pressure. The residue was purified by flash column chromatography (silica gel, DCM/MeOH 100:1 to 10:1) to afford product **SF10** (9.14 g, 29.6 mmol, 99% yield) as a colorless oil.

Step 2. Benzylation and Deprotection:

To a stirred solution of **SF10** (9.14 g, 29.6 mmol, 1.0 equiv.) in DMF (59 mL) at 0 °C was added NaH (60% dispersion in mineral oil, 5.33 g, 133 mmol, 4.5 equiv.) and BnBr (15.8 mL, 133 mmol, 4.5 equiv.). The reaction mixture was allowed to warm to room temperature and stirred for 12 h. The reaction was quenched with H_2O (2 mL) and concentrated under reduced pressure. The resulting residue was treated with TBAF (1 m in THF, 59.2 mL, 2.0 equiv.) in THF at room temperature and stirred for 3 h. The reaction mixture was concentrated under reduced pressure, and the residue was purified by flash column chromatography (silica gel, hexane/EtOAc 20:1 to 1:1) to afford product **SF11** (11.3 g, 24.3 mmol, 82% yield) as a colorless oil.

Conversion to Hydrazide:

Starting from the primary alcohol, the reaction was carried out on a 10.0 mmol scale following **General Procedures F2 and G2**. The crude product was purified by flash column chromatography (silica gel, hexane/EtOAc 20:1 to 1:1) to afford the desired hydrazide **117** (5.3 g, 83% yield, 2 steps) as a white solid.

*Note: **SF10** was prepared according to the literature procedure.*[7] ***SF11** was prepared according to the literature procedure.*[8]

4.5.5 Synthesis of ribose C2 and C3 hydrazide

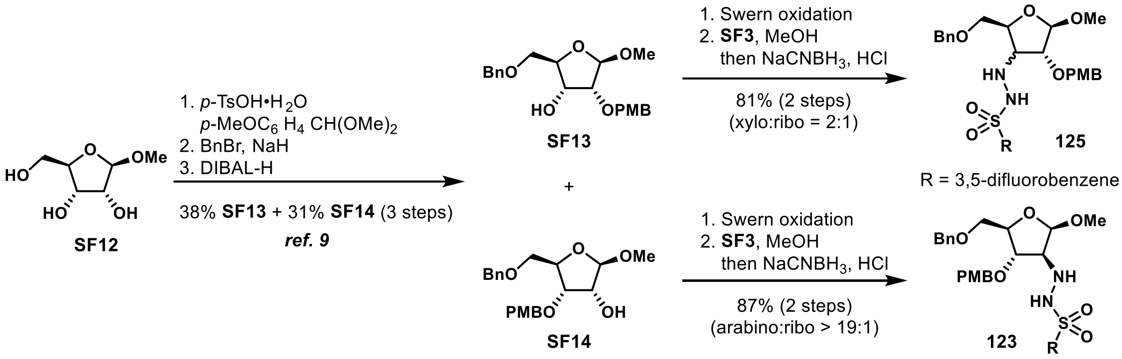

For C2 hydrazide, Starting from **SF14**, the reaction was carried out on a 6.0 mmol scale following **General Procedures F2 and G2**. The crude product was purified by flash column chromatography (silica gel, hexane/EtOAc 20:1 to 1:1) to afford the desired hydrazide **123** (2.94 g, 87% yield, 2 steps, arabino:ribo > 19:1) as a white solid.

For C3 hydrazide, Starting from **SF13**, the reaction was carried out on a 12.5 mmol scale following **General Procedures F2 and G2**. The crude product was purified by flash column chromatography (silica gel, hexane/EtOAc 20:1 to 1:1) to afford the desired hydrazide **125** (5.7 g, 81% yield, 2 steps, xylo:ribo = 2:1) as a white solid.

*Note: **SF13** and **SF14** were prepared according to the literature procedure.*[9]

4.5.6 Synthesis of ribose C5 hydrazide

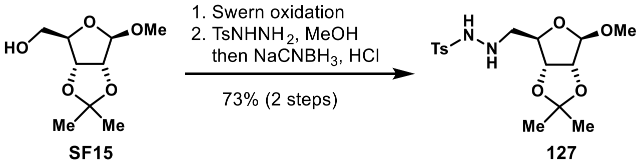

Following **General Procedures F2 and G2**, the reaction was conducted on a 5.0 mmol scale. The crude product was purified by flash column chromatography (silica

gel, hexane/EtOAc 20:1 to 1:1) to afford the desired hydrazide **127** (1.36 g, 73% yield, 2 steps) as a white solid.

Note: ***SF15*** *is commercially available.*

4.5.7 Synthesis of 2-deoxy-ribose C3 hydrazide

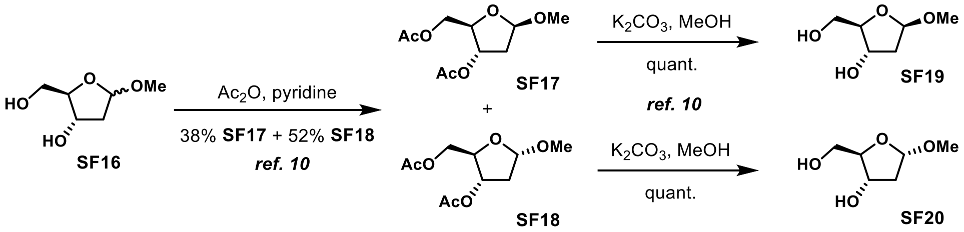

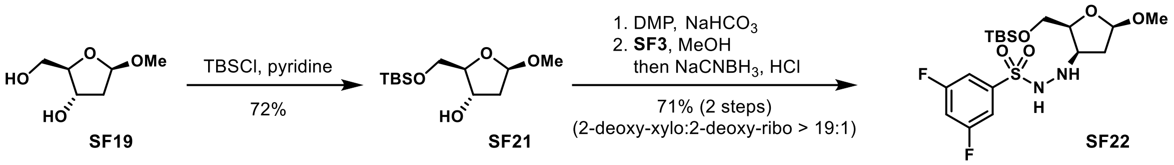

TBS Protection:

To a stirred solution of the alcohol **SF19** (2.05 g, 13.8 mmol, 1.0 equiv.) in pyridine (5.5 mL) at 0 °C was added TBSCl (2.29 g, 15.2 mmol, 1.0 equiv.). The reaction mixture was allowed to warm to room temperature and stirred for 2 h. The reaction was quenched with saturated aqueous $CuSO_4$ solution to make a clear blue solution and extracted with EtOAc (3×50 mL). The combined organic layers were dried over Na_2SO_4, filtered, and concentrated under reduced pressure. The residue was purified by flash column chromatography (silica gel, DCM/MeOH 100:1 to 10:1) to afford product **SF21** (2.6 g, 9.9 mmol, 72% yield) as a colorless oil.

Following **General Procedures F2 and G2**, the reaction was conducted on a 8.9 mmol scale. The crude product was purified by flash column chromatography (silica gel, hexane/EtOAc 20:1 to 1:1) to afford the desired hydrazide **SF22** (3.0 g, 71% yield, 2 steps, 2-deoxy-xylo:2-deoxy-ribo > 19:1) as a white solid.

Note: ***SF19*** *and* ***SF20*** *were prepared according to the literature procedure.* [10]

4.5.8 Synthesis of 2-deoxy-ribose C5 hydrazide

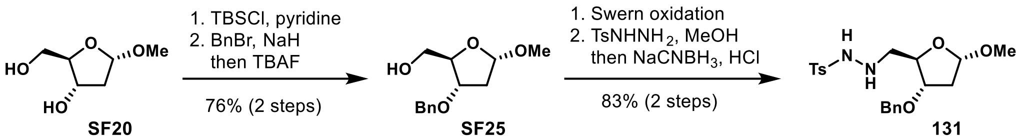

Preparation of the Primary Alcohol (Two-Step Sequence):

Step 1. TBS Protection:

To a stirred solution of the alcohol **SF20** (2.29 g, 18.8 mmol, 1.0 equiv.) in pyridine (7.5 mL) at 0 °C was added TBSCl (2.84 g, 18.8 mmol, 1.0 equiv.). The reaction mixture was allowed to warm to room temperature and stirred for 2 h. The reaction was quenched with saturated aqueous $CuSO_4$ solution to make a clear blue solution and extracted with EtOAc (3×50 mL). The combined organic layers were dried over Na_2SO_4, filtered, and concentrated under reduced pressure. The residue was purified by flash column chromatography (silica gel, DCM/MeOH 100:1 to 10:1) to afford C5-OH silylated product (3.94 g, 15 mmol, 80% yield) as a colorless oil.

Step 2. Benzylation and Deprotection:

To a stirred solution of C5-OH silylated compound (3.94 g, 15 mmol, 1.0 equiv.) in DMF (30 mL) at 0 °C was added NaH (60% dispersion in mineral oil, 900 mg, 22.5 mmol, 1.5 equiv.) and BnBr (1.83 mL, 22.5 mmol, 1.5 equiv.). The reaction mixture was allowed to warm to room temperature and stirred for 12 h. The reaction was quenched with H_2O (2 mL) and concentrated under reduced pressure. The resulting residue was treated with TBAF (1 m in THF, 30 mL, 2.0 equiv.) in THF at room temperature and stirred for 3 h. The reaction mixture was concentrated under reduced pressure, and the residue was purified by flash column chromatography (silica gel, hexane/EtOAc 20:1 to 1:1) to afford product **SF25** (3.4 g, 14.4 mmol, 96% yield) as a colorless oil.

Conversion to Hydrazide:

Starting from the primary alcohol, the reaction was carried out on a 10.0 mmol scale following **General Procedures F2 and G2**. The crude product was purified by flash column chromatography (silica gel, hexane/EtOAc 20:1 to 1:1) to afford the desired hydrazide **131** (3.37 g, 83% yield, 2 steps) as a white solid.

Note: ***SF20*** *was prepared according to the literature procedure.* [10]

4.5.9 Synthesis of C3 Thymidine hydrazide 109

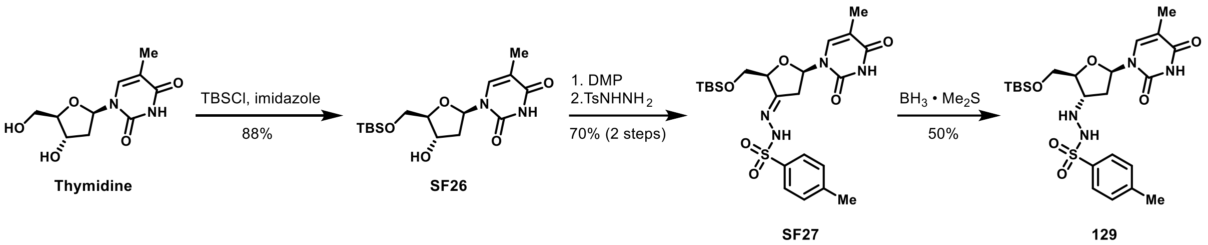

To a stirred solution of thymidine (18 g, 74 mmol, 1.0 equiv.) and imidazole (10.1 g, 149 mmol, 2.0 equiv.) in DMF (100 mL) at 0°C was added dropwise TBSCl (11.2 g, 74 mmol, 1.0 equiv.) dissolved in DMF (50 mL). The mixture was allowed to warm to room temperature and stirred overnight. Upon completion (monitored by TLC), water (300 mL) was added to form a white precipitate which was filtered and washed with water to give alcohol **SF26** (23.2 g, 88% yield) as a white solid which was used in the next step without further purification.

To a stirred solution of alcohol **SF26** (4 g, 11.2 mmol, 1.0 equiv.) in DCM (250 mL) at 0°C was added DMP (9.5 g, 22.4 mmol, 2.0 equiv.) sequentially, and the reaction mixture was allowed to warm to room temperature and stirred for 4 h. Upon completion (monitored by TLC), half of the solvent was evaporated and replaced with MeOH before $TsNHNH_2$ (2.5 g, 13.4 mmol, 1.2 equiv.) was added sequentially at room temperature and the mixture was stirred overnight. Upon completion (monitored by TLC), the solution was diluted with water (200 mL) and extracted with DCM (3 x 100). The combined organic layers were washed with brine (1 x 50), dried over Na_2SO_4 and purified by flash column chromatography (silica gel, Hexane/EtOAc 4:1 to 1:1) to afford the hydrazone **SF27** (4.1 g, 7.8 mmol, 70% yield) as a white solid.

To a stirred solution of hydrazone **SF27** (400 mg, 0.77 mmol, 1.0 equiv.) in THF (1.5 mL) at 0°C was added dropwise a $BH_3 \cdot Me_2S$ solution (0.38 mL, 2 M in THF, 5.0 equiv.). The reaction was allowed to warm to room temperature and stirred overnight. Upon completion (monitored by TLC), the solution was quenched with aq. sat. NH_4Cl solution (4 mL), extracted with EtOAc (3 x 10 mL) and the combined organic layers washed with water (1 x 15 mL) and brine (1 x 10 mL) and dried over Na_2SO_4 before the crude was purified by flash column chromatography (silica gel, Hexane/EtOAc 1:1 to 1:2) to afford the hydrazide **129** (199 mg, 0.38 mmol, 50% yield) as a white solid.

4.6 General Synthetic Procedure for cross-coupling of sugar hydrazides and aryl halides

4.6.1 General Synthetic Procedure for cross-coupling of sugar C2-C6 hydrazides and aryl halides

General Procedure H:

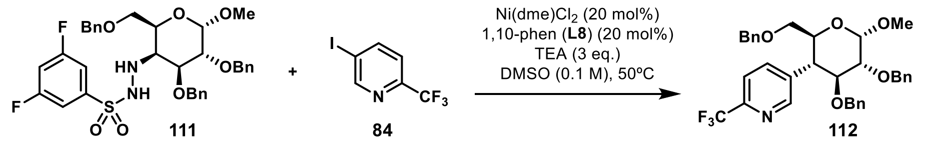

To a reaction tube with a stir bar were added sugar hydrazide (0.1 mmol, 1.0 equiv.), aryl halide (0.20 mmol, 2.0 equiv.), Ni(dme)Cl_2 (20 mol%, 0.2 equiv.), and 1,10-phenanthroline (**L8**) (20 mol%, 0.2 equiv.). The tube was capped with a septum cap, then evacuated and backfilled with argon three times. Pre-degassed DMSO (1 mL) was added and the mixture was stirred for 1 minute, followed by the addition of TEA (0.3 mmol, 3.0 equiv.). The reaction mixture was heated to 50 °C and stirred overnight. After the reaction was completed, water (5 mL) was added. The aqueous phase was extracted with EtOAc (3 x 5 mL). The combined organic phase was washed with brine (2 mL), dried over Na_2SO_4, and concentrated under reduced pressure. The crude residue was purified by flash column chromatography (silica gel, hexane/EtOAc 50:1 to 1:1) to afford desired product.

4.6.2 Graphical Experimental Guide for General Procedure H

*Photos were taken of the coupling between glucose C4 hydrazide **111** and 5-iodo-2-(trifluoromethyl)pyridine (aryl iodide) **84**.*

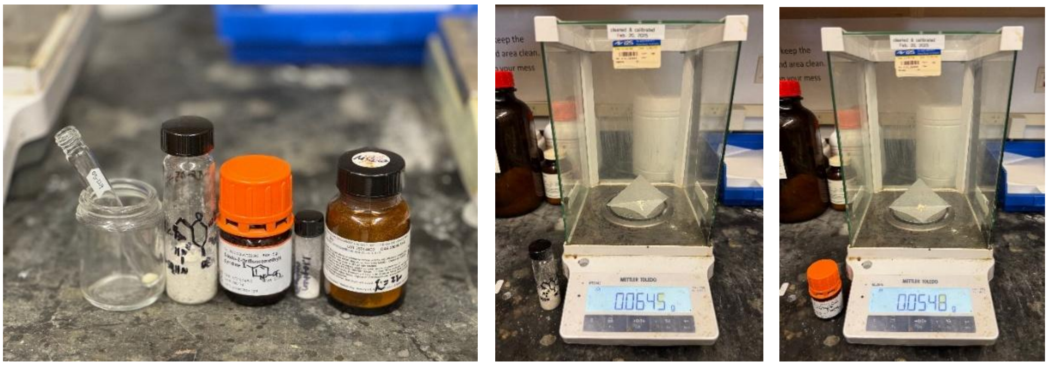

(Left) All reagents (glucose C4 hydrazide **111**, aryl iodide **84**, Ni(dme)Cl_2, and **L8**) for the reaction and a clean tube with a stir bar. **(Center)** glucose C4 hydrazide **111** was weighed out. (Right) Aryl iodide **84** was weighed out.

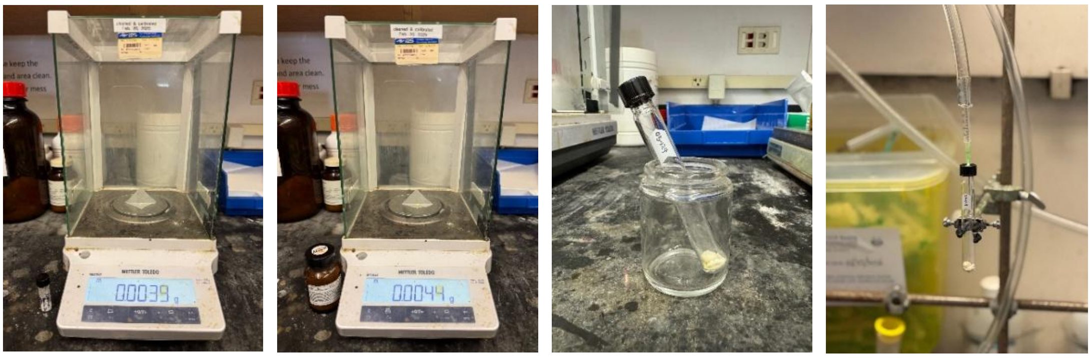

(Left) L8 was weighed out. **(Center Left)** Ni(dme)Cl_2 was weight out. **(Center Right)** The reaction tube was capped. **(Right)** The reaction tube was evacuated on a Schlenk line.

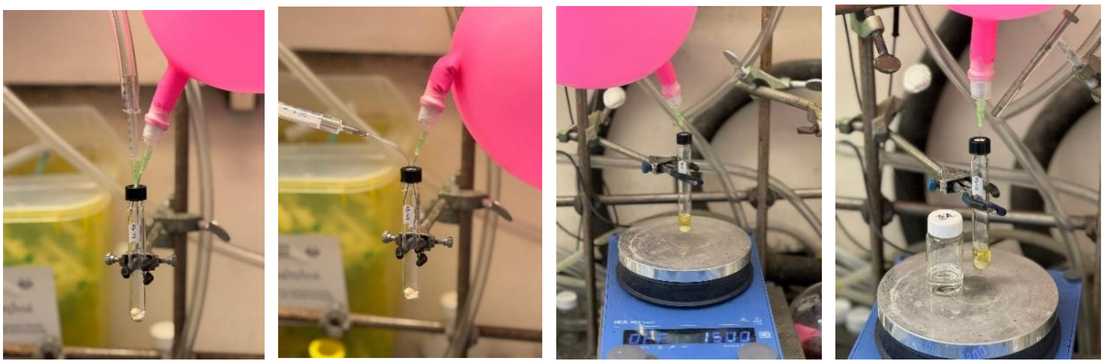

(Left) The reaction tube was refilled with argon. Evacuation and back-filling were repeated three times. **(Center Left)** Pre-degassed DMSO (1 mL) was added via syringe under an argon atmosphere. **(Center Right)** Reaction mixture after DMSO addition.

(Right) TEA (42 μL) was added via micro syringe.

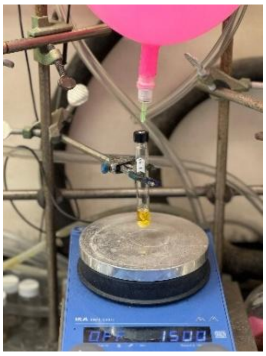
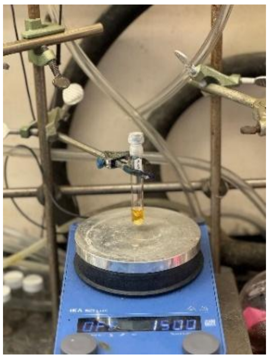
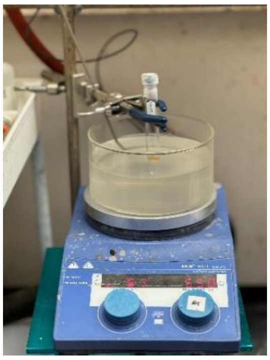
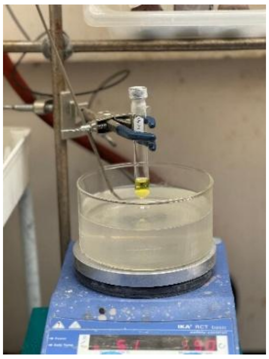

(Left) The reaction mixture after the addition of TEA. **(Center Left)** The reaction tube was sealed with parafilm. **(Center Right)** The reaction mixture was submerged in a preheated oil bath at 50 °C. **(Right)** The reaction mixture after the reaction was done.

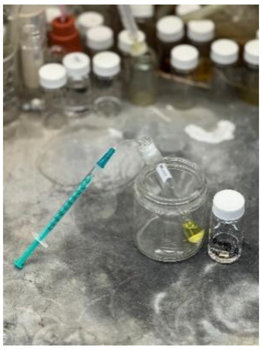
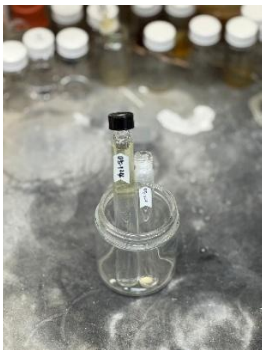
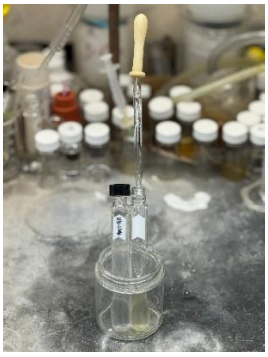
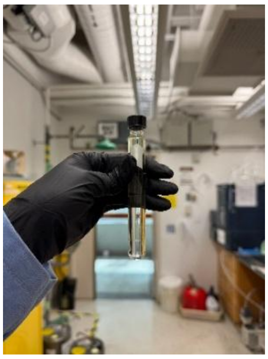

(Left) 1,3,5-trimethoxybenzene was used as internal standard for crude NMR yield determination. 0.3 mL of internal standard stock solution (40 mg/10 mL EtOAc) was added to the reaction mixture. **(Center Left)** The reaction mixture was diluted with EtOAc and H_2O. **(Center Right)** The aqueous phase was extracted with EtOAc three times. **(Right)** The combined organic phase was washed with saturated brine solution.

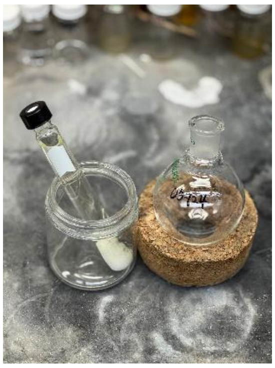
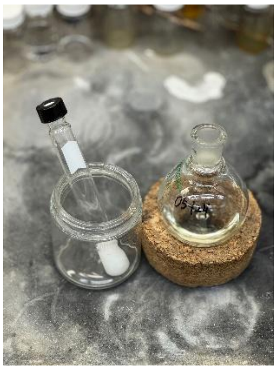
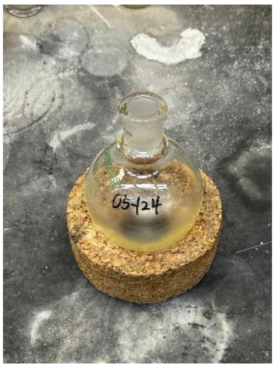

(Left) The organic phase was dried over Na_2SO_4. **(Center)** The organic phase was transferred to a 100 mL round-bottom flask. **(Right)** The concentrated crude mixture.

4.6.3 Synthesis of C3-Glycohydrazide (w/o PG):

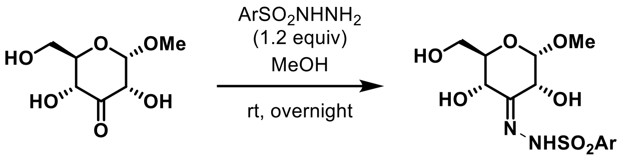

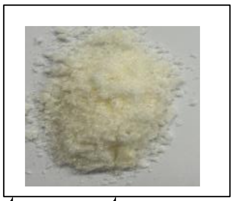

Keto sugar (1.0 equiv) was dissolved in methanol (50 mL) at room temperature, and sulfonyl hydrazine (1.2 equiv) was added. The reaction progress was monitored by TLC using p-anisaldehyde stain. Upon completion, the solvent was removed under reduced pressure. The crude residue was purified by silica gel column chromatography using a gradient of ethyl acetate/hexane (1:1 to 20:1) to afford the corresponding sulfonyl hydrazone (E/Z mixture) as a colorless solid.

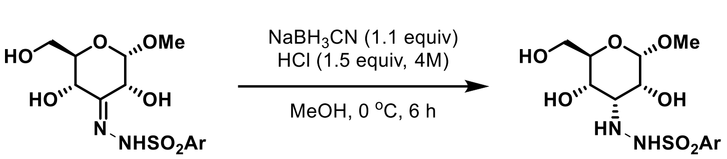

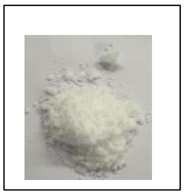

Sulfonyl hydrazone (10 mmol, 1.0 equiv) was dissolved in methanol (50 mL), and $NaBH_3CN$ (11 mmol, 1.1 equiv) was added, followed by dropwise addition of HCl (4.0 M in dioxane, 1.5 equiv) at 0 °C. The reaction was stirred at this temperature until complete, as determined by TLC (*p*-anisaldehyde stain). The mixture was diluted with ethyl acetate and concentrated under reduced pressure. Purification by silica gel column chromatography (hexane/ethyl acetate, 1:1 to 1:20) afforded the glyconylhydrazide as a colorless solid.

4.6.4 General procedure for C3 arylation of unprotected sugar

General Procedure I:

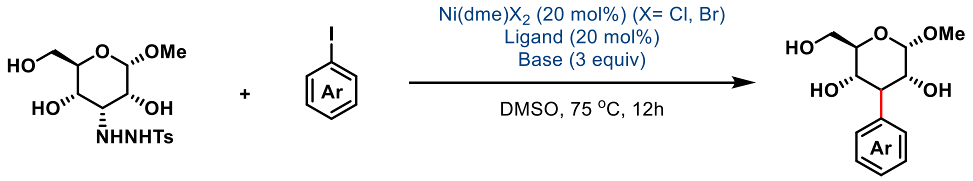

A C-3 glycohydrazide (0.10 mmol, 1.0 equiv), iodoarene (0.15 mmol, 1.5 equiv), Ni catalyst (0.02 mmol, 20 mol%), and ligand (0.02 mmol, 20 mol%) were combined in DMSO (1.0 mL, 0.1 M). The reaction mixture was stirred, and base (3.0 equiv) was added. The resulting mixture was stirred at 75 °C for 12 h. Upon completion, the reaction was quenched with formic acid to neutralize the base. The crude reaction mixture was directly subjected to purification by reverse-phase flash chromatography on a C18 column (Teledyne), using a gradient of H_2O to MeCN (100:0 to 0:100 over 20 min), to afford the desired product as a colourless solid.

4.6.5 General procedure for C1 arylation of Bn-protected ribose

General Procedure I:

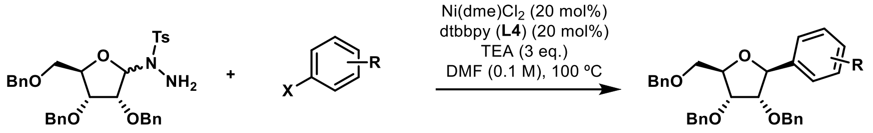

To a reaction tube equipped with a stir bar were added sugar hydrazide (0.1 mmol, 1.0 equiv.), aryl halide (0.2 mmol, 2.0 equiv.), Ni(dme)Cl_2 (0.2 mmol, 0.2 equiv.), and **L4** (0.2 mmol, 0.2 equiv.). The tube was capped with a septum cap, then evacuated and backfilled with argon three times. Pre-degassed DMF (1 mL) was added and the mixture was stirred for 1 minute, followed by the addition of TEA (0.3 mmol, 3.0 equiv.). The reaction mixture was heated to 80 °C and stirred overnight. After the reaction was completed, water (5 mL) was added. The aqueous phase was extracted with EtOAc (3 x 5 mL). The combined organic phase was washed with brine (2 mL), dried over Na_2SO_4, and concentrated under reduced pressure. The crude residue was purified by flash column chromatography (silica gel, hexane/EtOAc 50:1 to 1:1) to afford the desired product as a colorless oil.

4.7 Synthetic Procedures of Aryl Halides

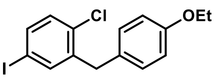

SG1

CAS: 1103738-29-9
(67$/100g)

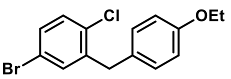

SG2

CAS: 461432-23-5
(50$/100g)

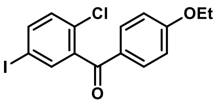

SG3

CAS: 1103738-26-6
(70$/5g)

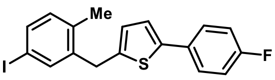

SG4

CAS: 898566-17-1
(92$/10g)

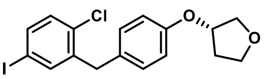

SG5

CAS: 915095-94-2
(69$/100g)

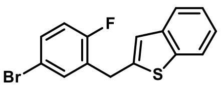

SG6

CAS: 1034305-17-3
(100$/5g)

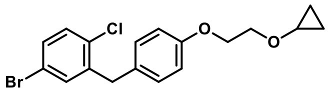

SG7

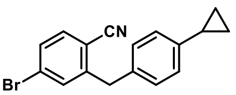

SG8

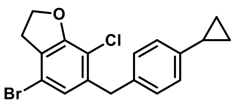

SG9

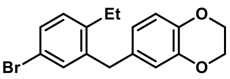

SG10

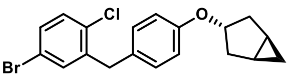

SG11

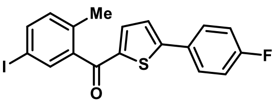

SG23

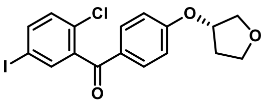

SG24

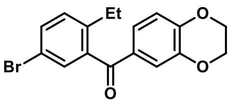

SG25

Synthesis of SG7:

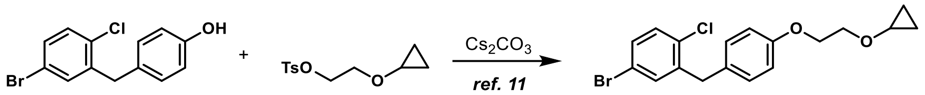

SG12 CAS: 864070-18-8 (50$/25g) — **SG13** CAS: 862728-59-4 (86$/5g) — **SG7**

SG7 was prepared according to the literature procedure. [11]

Synthesis of SG8:

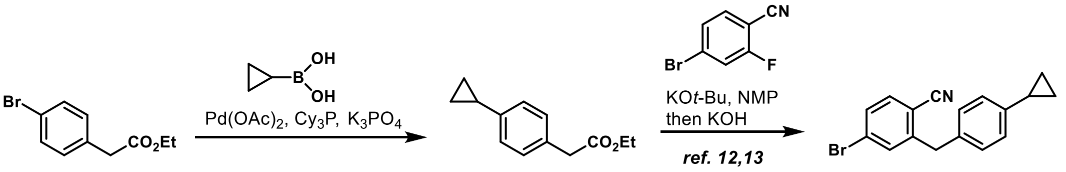

SG14 — **SG15** — **SG8**

SG8 was prepared according to the literature procedure. [12,13]

Synthesis of SG9:

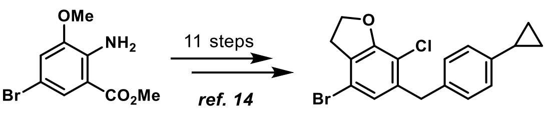

SG16 — **SG9**

SG9 was prepared according to the literature procedure. [14]

Synthesis of SG10:

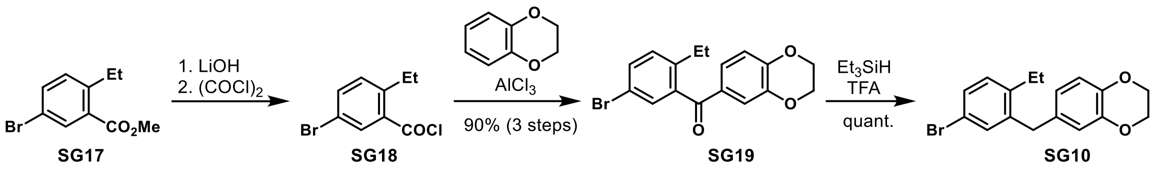

SG10 was prepared according to the literature procedure. [15]

Synthesis of SG11:

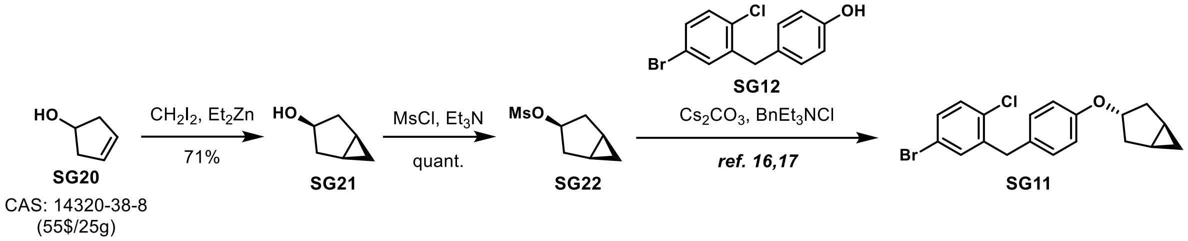

SG11 was prepared according to the literature procedure. [16,17]

5. Frequently Asked Questions

Question 1: How should I monitor the reaction, particularly for reactions conducted in DMSO as the solvent?

Answer: For unprotected sugars, we typically use ^1H NMR to confirm both the identity of the compound and the progress of the reaction. A small aliquot of the reaction mixture (ca. 0.05 mL) is taken and dissolved in 0.5 mL of CD_3OD or DMSO-d_6. The crude ^1H NMR spectrum is then recorded using a solvent-suppression pulse sequence (to attenuate the residual DMSO signal), which allows clearer observation of the sugar proton signals. For protected sugars, the reaction can be monitored either by crude ^1H NMR or by TLC analysis, using UV visualization (254 nm) and PMB staining.

Question 2: How should the reaction be worked up when DMSO is used as the solvent in a Ni-catalyzed cross-coupling reaction?

Answer: For a 0.1 mmol-scale RCC-arylation of unprotected glycohydrazides, the crude mixture was purified directly by reverse-phase column chromatography. For larger-scale reactions, the reaction was quenched by the addition of water and extracted with EtOAc (3×). The combined organic layers were washed with brine (100 mL), dried over Na_2SO_4, and concentrated under reduced pressure. The crude residue was then purified by flash column chromatography (silica gel, DCM/EtOAc 10:1 to 1:1, followed by DCM/MeOH 100:1 to 9:1).

For some cases where DMF is used as the solvent, the workup can be simpler. The reaction mixture can be directly concentrated, and the crude product can be analyzed by ^1H NMR or purified directly by column chromatography.

Question 3: What are the typical side products of the reaction?

Answer: The major side products in the radical cross-coupling of glycohydrazides are typically the elimination product and the reduced product. The elimination product contains a double bond between the C1 and C2 positions, whereas in the reduced product the C1 position is replaced by hydrogen.

Question 4: Which reaction conditions should I use, especially for choice of the ligand and base?

Answer: Reaction conditions were varied depending on the type of sugar hydrazide. For unprotected sugar hydrazides, only glucose hydrazide performs well when TMG is used as the base, giving excellent yield and diastereoselectivity. In contrast, other sugar hydrazides react better with Et_3N as the base; using the stronger base TMG in these cases leads to significantly lower yields. In addition, **L1** was used as the ligand for aryl iodides, while di-NH_2-bpy (**L6**) was employed for aryl bromides.

For hydrazides derived from different positions of the sugars, 1,10-phenanthroline (**L8**) was used as the ligand together with Et_3N as the base.

For C1 stereoretentive reactions, **L12** was used as the ligand with Et_3N as the base.

Question 5: Are sugar hydrazides stable on the bench?

Answer: The majority of unprotected sugar hydrazides are bench-stable solids, particularly those derived from tosyl hydrazide, 3,5-difluorobenzenesulfonyl hydrazide, and 3,4,5-trifluorobenzenesulfonyl hydrazide. In contrast, 2-(trifluoromethyl)benzenesulfonyl hydrazides can also be readily obtained but tend to decompose at room temperature. For protected sugar hydrazides, the corresponding tosyl hydrazides and 3,5-difluorobenzenesulfonyl hydrazides can be synthesized following the general procedure, although they are not sufficiently stable for long-term storage at room temperature. These compounds can be stored neat in a −20 °C freezer for several months. In contrast, 3,4,5-trifluorobenzenesulfonyl hydrazides and 2-(trifluoromethyl)benzenesulfonyl hydrazides could not be obtained, likely due to their high reactivity, which leads to decomposition during the synthesis.

Question 6: Are the reactions with sugar hydrazides air and/or moisture sensitive?

Answer: Broadly speaking, air has a detrimental effect on the reaction. When the reaction was conducted under air on a 0.1 mmol scale, the yield decreased significantly from 70% to 15%. Therefore, conducting the reaction under an argon atmosphere is

recommended. In contrast, the coupling reaction is relatively insensitive to moisture. For example, in the arylation of sulfonyl hydrazides, the addition of 100 equiv of water still afforded a 50% yield.

Question 7: Can the reaction be scaled up or be run more concentrated?

Answer: Due to the homogeneous nature of the reaction, it can be conveniently scaled up, as demonstrated on a decagram scale in the laboratory. In general, the reaction was conducted at a concentration of 0.1 M, which provided the highest yield. Increasing the concentration resulted in some loss of efficiency; for example, when the concentration was increased to 1.0 M, the yield decreased to 51%.

Question 8: Why were catalytic amounts of AcOH used instead of neat AcOH for disaccharide and trisaccharide hydrazide synthesis?

Answer: Disaccharides and trisaccharides are more sensitive to acidic conditions due to the presence of glycosidic linkages. Neat AcOH can promote glycosidic bond cleavage or anomerization. Therefore, catalytic amounts of AcOH were used to facilitate hydrazide formation while minimizing acid-promoted decomposition.

6. Characterization Data

6.1 Characterization Data of Native Sugar Sulfonylhydrazides

Glucosyl hydrazide 25

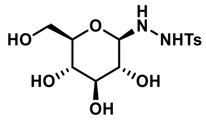

Following **General Procedure A1** with glucose (**24**) (5 mmol) and *p*-toluenesulfonyl hydrazide, purification through precipitation and filtration afforded glucosyl hydrazide **25** (1.7 g, 95% yield) as white solid.

$[\alpha]_D^{25}$= –37.6° (c = 0.5, DMSO)

^{1}H NMR (600 MHz, $(CD_3)_2SO$) δ 8.84 (s, 1H), 7.73 (d, *J* = 8.2 Hz, 2H), 7.37 (d, *J* = 8.0 Hz, 2H), 5.41 (d, *J* = 7.8 Hz, 1H), 4.93 (d, *J* = 3.8 Hz, 1H), 4.88 (d, *J* = 4.8 Hz, 1H), 4.44 (dd, *J* = 8.0, 4.1 Hz, 1H), 4.29 (d, *J* = 3.1 Hz, 1H), 3.63 (ddd, *J* = 11.9, 8.1, 1.9 Hz, 1H), 3.45 (t, *J* = 8.0 Hz, 1H), 3.37 – 3.32 (m, 1H), 3.05 – 2.99 (m, 2H), 2.94 – 2.87 (m, 2H), 2.38 (s, 3H).

^{13}C NMR (151 MHz, $(CD_3)_2SO$) δ 142.8, 136.7, 129.4, 127.5, 90.0, 78.1, 77.0, 70.7, 70.5, 61.9, 21.1.

HRMS (m/z): (ESI) calc. $C_{13}H_{20}N_2NaO_7S$ $[M+Na]^+$: 371.0889 found 371.0877.

Lactose-hydrazide SH1

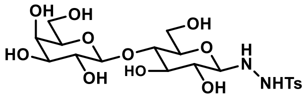

Following **General Procedure A2** with lactose (5 mmol) and *p*-toluenesulfonyl hydrazide, purification through precipitation and filtration afforded lactose-hydrazide **SH1** (2.3 g, 92% yield) as white solid.

$[\alpha]_D^{25}$= –8.2° (c = 0.5, DMSO)

^{1}H NMR (600 MHz, $(CD_3)_2SO$) δ 8.85 (s, 1H), 7.72 (d, *J* = 8.1 Hz, 2H), 7.37 (d, *J* = 8.0 Hz, 2H), 5.41 (d, *J* = 7.8 Hz, 1H), 5.11 (d, *J* = 4.1 Hz, 1H), 4.79 (d, *J* = 4.8 Hz, 1H), 4.68 (s, 1H), 4.65 (t, *J* = 5.1 Hz, 1H), 4.51 (d, *J* = 4.6 Hz, 1H), 4.47 (dd, *J* = 7.7, 4.7 Hz, 1H), 4.42 (d, *J* = 4.3 Hz, 1H), 4.15 (d, *J* = 6.8 Hz, 1H), 3.76 (ddd, *J* = 11.8, 7.7, 2.3 Hz, 1H), 3.60 (t, *J* = 3.7 Hz, 1H), 3.57 (t, *J* = 8.4 Hz, 1H), 3.53 – 3.42 (m, 4H), 3.33 – 3.26 (m, 2H), 3.22 (td, *J* = 8.5, 1.8 Hz, 1H), 3.16 – 3.06 (m, 3H), 2.39 (s, 3H).

^{13}C NMR (151 MHz, $(CD_3)_2SO$) δ 142.9, 136.7, 129.4, 127.5, 103.9, 89.9, 81.1, 76.0, 75.5, 75.3, 73.2, 70.5, 68.1, 61.2, 60.4, 21.1.

HRMS (m/z): (ESI) calc. $C_{19}H_{31}N_2O_{12}S$ $[M+H]^+$: 511.1602 found 511.1598.

Cellobiose-hydrazide SH2

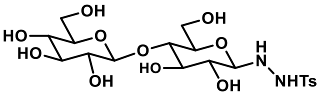

Following **General Procedure A2** with cellobiose (5 mmol) and *p*-toluenesulfonyl hydrazide, purification through precipitation and filtration afforded cellobiose-hydrazide **SH2** (2.4 g, 94% yield) as white solid.

$[\alpha]_D^{25}$= –45.6° (c = 0.25, DMSO)

^{1}H NMR (600 MHz, (CD3)2SO) δ 8.85 (s, 1H), 7.72 (d, J = 8.2 Hz, 2H), 7.37 (d, J = 8.1 Hz, 2H), 5.40 (d, J = 7.8 Hz, 1H), 5.25 (d, J = 4.9 Hz, 1H), 5.02 (d, J = 5.0 Hz, 1H), 4.99 (d, J = 5.5 Hz, 1H), 4.66 (d, J = 2.2 Hz, 1H), 4.59 (t, J = 5.3 Hz, 1H), 4.48 (dd, J = 7.6, 4.7 Hz, 1H), 4.41 (d, J = 4.4 Hz, 1H), 4.21 (d, J = 7.9 Hz, 1H), 3.76 (ddd, J = 11.8, 7.6, 2.4 Hz, 1H), 3.70 – 3.66 (m, 1H), 3.57 (t, J = 8.3 Hz, 1H), 3.49 (dt, J = 11.2, 5.2 Hz, 1H), 3.39 (dt, J = 11.9, 6.3 Hz, 1H), 3.24 – 3.06 (m, 5H), 3.03 (td, J = 9.2, 5.4 Hz, 1H), 2.96 (td, J = 8.5, 4.9 Hz, 1H), 2.39 (s, 3H).

^{13}C NMR (151 MHz, (CD3)2SO) δ 142.8, 136.6, 129.4, 127.5, 103.2, 90.0, 81.0, 76.8, 76.5, 76.0, 75.3, 73.3, 70.5, 70.0, 61.2, 61.0, 21.0.

HRMS (m/z): (ESI) calc. $C_{19}H_{31}N_2O_{12}S$ $[M+H]^+$: 511.1602 found 511.1590.

Maltose-hydrazide SH3

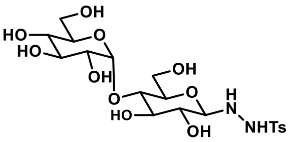

Following **General Procedure A2** with maltose (5 mmol) and *p*-toluenesulfonyl hydrazide, purification through precipitation and filtration afforded maltose-hydrazide **SH3** (2.2 g, 85% yield) as white solid.

$[a]_{\mathrm{D}}^{25}$= +67.2° (c = 0.5, DMSO)

^{1}H NMR (600 MHz, D_2O) δ 7.78 (d, *J* = 8.8 Hz, 2H), 7.45 (d, *J* = 7.9 Hz, 2H), 4.02 – 3.24 (m, 15H), 2.42 (s, 3H).

^{13}C NMR (151 MHz, D_2O) δ 145.6, 133.3, 129.9, 127.7, 97.8, 90.1, 76.4, 75.3, 73.0, 71.7, 70.2, 69.5, 69.3, 65.5, 60.5, 20.7.

HRMS (m/z): (ESI) calc. $C_{19}H_{31}N_2NaO_{12}S$ $[M+H]^+$: 511.1602 found 511.1594.

Maltotriose-hydrazide SH4

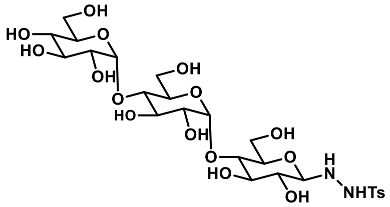

Following **General Procedure A2** with maltotriose (5 mmol) and *p*-toluenesulfonyl hydrazide, purification through precipitation and filtration afforded maltotriose-hydrazide **SH4** (2.9 g, 88% yield) as white solid.

$[\alpha]_D^{25}$= +29.0° (c = 0.5, DMSO)

^{1}H NMR (600 MHz, $(CD_3)_2SO$) δ 8.88 (s, 1H), 7.72 (d, *J* = 7.9 Hz, 2H), 7.37 (d, *J* = 7.9 Hz, 2H), 5.55 (d, *J* = 6.3 Hz, 1H), 5.51 (d, *J* = 3.1 Hz, 1H), 5.50 – 5.42 (m, 3H), 4.99 (dd, *J* = 8.9, 3.7 Hz, 2H), 4.91 (dd, *J* = 10.0, 5.3 Hz, 2H), 4.53 (dt, *J* = 20.0, 5.6 Hz, 2H), 4.45 (dd, *J* = 7.5, 4.4 Hz, 1H), 4.40 (d, *J* = 4.3 Hz, 1H), 3.71 – 3.37 (m, 11H), 3.32 – 3.26 (m, 3H), 3.22 (t, *J* = 5.1 Hz, 1H), 3.17 (t, *J* = 9.2 Hz, 1H), 3.13 – 3.03 (m, 3H), 2.39 (s, 3H).

^{13}C NMR (151 MHz, $(CD_3)_2SO$) δ 143.3, 137.1, 129.9, 127.9, 101.3, 101.0, 90.5, 80.7, 80.0, 77.1, 76.6, 74.0, 73.8, 73.6, 73.0, 72.4, 72.2, 70.7, 70.3, 61.8, 61.2, 60.7, 60.2, 21.5.

HRMS (m/z): (ESI) calc. $C_{25}H_{41}N_2O_{17}S$ $[M+H]^+$: 673.2126 found 673.2111.

Mannose-hydrazide 73

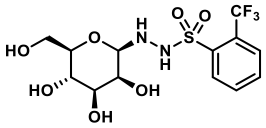

Following **General Procedure A1** with D-mannose (5 mmol) and 2-(trifluoromethyl)benzenesulfonyl hydrazide, purification through precipitation and filtration afforded mannose-hydrazide **73** (1.9 g, 94% yield) as white solid.

$[\alpha]_{D}^{25}$= +53.4° (c = 0.5, DMSO)

^{1}H NMR (600 MHz, $(CD_3)_2SO$) δ 9.15 (s, 1H), 8.18 (d, *J* = 7.6 Hz, 1H), 7.98 (dd, *J* = 7.4, 1.8 Hz, 1H), 7.89 – 7.83 (m, 2H), 4.96 (d, *J* = 11.4 Hz, 1H), 4.78 (d, *J* = 4.8 Hz, 1H), 4.73 (s, 2H), 4.48 (dd, *J* = 9.7, 2.9 Hz, 1H), 3.76 (d, *J* = 11.3 Hz, 1H), 3.67 (t, *J* = 9.1 Hz, 1H), 3.46 (s, 1H), 3.39 – 3.36 (m, 1H), 3.18 – 3.10 (m, 2H), 2.95 (td, *J* = 8.5, 2.3 Hz, 1H).

^{13}C NMR (151 MHz, $(CD_3)_2SO$) δ 138.1, 133.14, 133.11, 131.6, 128.3 (q, $^{3}J_{C,F}$ = 6.3 Hz), 126.6 (q, $^{2}J_{C,F}$ = 32.7 Hz), 122.9 (q, $^{1}J_{C,F}$ = 274.0 Hz), 87.8, 78.3, 74.1, 69.6, 67.6, 62.3.

HRMS (m/z): (ESI) calc. $C_{13}H_{18}F_3N_2O_7S$ $[M+H]^+$: 403.0787 found 403.0785.

Ribose-hydrazide 78

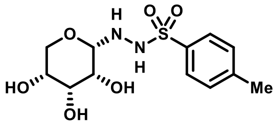

Following **General Procedure A1** with D-Ribose (5 mmol) and *p*-toluenesulfonyl hydrazide purification through precipitation and filtration afforded ribose-hydrazide **78** (1.6 g, 91% yield) as white solid.

$[\alpha]_D^{25}$= +82.4° (c = 0.25, DMSO)

^{1}H NMR (600 MHz, $(CD_3)_2SO$) δ 8.81 (d, *J* = 3.4 Hz, 1H), 7.70 (d, *J* = 8.1 Hz, 2H), 7.38 (d, *J* = 7.9 Hz, 2H), 4.93 (dd, *J* = 23.1, 5.6 Hz, 2H), 4.57 (d, *J* = 11.0 Hz, 1H), 4.52 (d, *J* = 7.7 Hz, 1H), 3.92 (d, *J* = 10.8 Hz, 1H), 3.66 (dd, *J* = 12.0, 4.3 Hz, 1H), 3.53 (d, *J* = 5.8 Hz, 1H), 3.46 (t, *J* = 7.4 Hz, 2H), 3.31 (dd, *J* = 12.1, 2.2 Hz, 1H), 2.38 (s, 3H).

^{13}C NMR (151 MHz, $(CD_3)_2SO$) δ 142.9, 136.1, 129.4, 127.6, 88.6, 70.2, 69.0, 68.3, 65.3, 21.1.

HRMS (m/z): (ESI) calc. for $C_{12}H_{18}N_2O_6S$ $[M+H]^+$: 341.0783 found 341.0773

6.2 Characterization Data of Gliflozins and Related Compounds

6.2.1 Coupling partners

Compound SG7

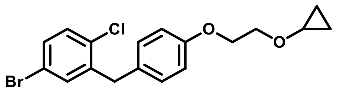

Following the procedures laid out in section **4.7 Synthetic Procedures of Aryl Halides**.

^{1}H NMR (600 MHz, $CDCl_3$) δ 7.27 (dd, *J* = 8.4, 2.5 Hz, 1H), 7.24 – 7.20 (m, 2H), 7.08 (d, *J* = 8.6 Hz, 2H), 6.86 (d, *J* = 8.6 Hz, 2H), 4.13 – 4.05 (m, 2H), 3.98 (s, 2H), 3.86 – 3.84 (m, 2H), 3.40 (tt, *J* = 6.0, 3.0 Hz, 1H), 0.65 – 0.61 (m, 2H), 0.52 – 0.47 (m, 2H).

^{13}C NMR (151 MHz, $CDCl_3$) δ 157.6, 141.4, 133.7, 133.2, 131.0, 130.8, 130.7, 130.1, 120.6, 114.9, 69.2, 67.2, 53.8, 38.3, 5.8.

HRMS (m/z): (ESI) calc. for $C_{18}H_{19}BrClO_2$ $[M+H]^+$ 381.0257; found 381.0258.

Compound SG8

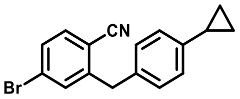

Following the procedures laid out in section **4.7 Synthetic Procedures of Aryl Halides**.

^{1}H NMR (600 MHz, $CDCl_3$) δ 7.48 (d, *J* = 8.2 Hz, 1H), 7.44 (dd, *J* = 8.2, 1.9 Hz, 1H), 7.42 (d, *J* = 1.8 Hz, 1H), 7.12 (d, *J* = 8.2 Hz, 2H), 7.03 (d, *J* = 8.2 Hz, 2H), 4.12 (s, 2H), 1.91 – 1.84 (m, 1H), 0.99 – 0.91 (m, 2H), 0.71 – 0.64 (m, 2H).

^{13}C NMR (151 MHz, $CDCl_3$) δ 147.3, 143.0, 134.9, 134.0, 133.3, 130.3, 129.0, 128.2, 126.2, 117.7, 111.4, 39.7, 15.2, 9.4.

HRMS (m/z): (ESI) calc. for $C_{17}H_{15}BrN$ $[M+H]^+$ 312.0388; found 312.0396.

Compound SG9

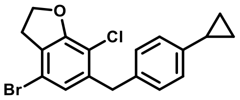

Following the procedures laid out in section **4.7 Synthetic Procedures of Aryl Halides**.

^{1}H NMR (600 MHz, $CDCl_3$) δ 7.08 (d, *J* = 8.2 Hz, 2H), 7.00 (d, *J* = 8.2 Hz, 2H), 6.81 (s, 1H), 4.70 (t, *J* = 8.8 Hz, 2H), 3.98 (s, 2H), 3.26 (t, *J* = 8.8 Hz, 2H), 1.87 (tt, *J* = 8.4,

5.0 Hz, 1H), 0.96 – 0.91 (m, 2H), 0.67 (dt, *J* = 6.6, 4.6 Hz, 2H).

^{13}C NMR (151 MHz, $CDCl_3$) δ 156.8, 142.2, 140.8, 136.0, 128.9, 127.4, 125.9, 125.3, 116.8, 114.6, 71.8, 38.1, 32.1, 15.2, 9.3.

HRMS (m/z): (ESI) calc. for $C_{18}H_{15}BrClO$ $[M–H]^-$ 360.9995; found 360.9992.

Compound SG10

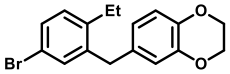

Following the procedures laid out in section **4.7 Synthetic Procedures of Aryl Halides**.

^{1}H NMR (600 MHz, $CDCl_3$) δ 7.31 (dd, *J* = 8.2, 2.2 Hz, 1H), 7.22 (d, *J* = 2.2 Hz, 1H), 7.06 (d, *J* = 8.2 Hz, 1H), 6.79 (d, *J* = 8.8 Hz, 1H), 6.61 – 6.57 (m, 2H), 4.24 (s, 4H), 3.86 (s, 2H), 2.57 (q, *J* = 7.5 Hz, 2H), 1.14 (t, *J* = 7.5 Hz, 3H).

^{13}C NMR (151 MHz, $CDCl_3$) δ 143.5, 142.0, 141.4, 140.8, 133.4, 132.9, 130.2, 129.7, 121.7, 119.6, 117.4, 117.3, 64.5, 64.4, 37.8, 25.4, 14.8.

HRMS (m/z): (ESI) calc. for $C_{17}H_{18}BrO_2$ $[M+H]^+$ 332.0412 found 332.0420.

Compound SG11

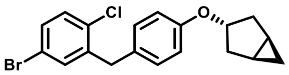

Following the procedures laid out in section **4.7 Synthetic Procedures of Aryl Halides**.

^{1}H NMR (600 MHz, $CDCl_3$) δ 7.27 (dd, *J* = 8.4, 2.4 Hz, 1H), 7.25 (d, *J* = 2.4 Hz, 1H), 7.22 (d, *J* = 8.4 Hz, 1H), 7.06 (d, *J* = 8.6 Hz, 2H), 6.80 (d, *J* = 8.6 Hz, 2H), 4.39 (p, *J* = 7.3 Hz, 1H), 3.98 (s, 2H), 2.34 (dd, *J* = 12.9, 7.0 Hz, 2H), 1.93 (ddd, *J* = 12.1, 7.5, 3.3 Hz, 2H), 1.38 – 1.35 (m, 2H), 0.45 (td, *J* = 8.1, 5.1 Hz, 1H), 0.05 (q, *J* = 4.2 Hz, 1H).

^{13}C NMR (151 MHz, $CDCl_3$) δ 157.3, 141.4, 133.7, 133.2, 131.0, 130.7, 130.5, 130.0, 120.6, 115.7, 76.9, 38.3, 34.6, 15.0, 9.9.

HRMS (m/z): (ESI) calc. for $C_{19}H_{19}BrClO$ $[M+H]^+$ 377.0308; found 377.0307.

Compound SG19

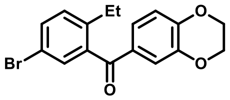

Following the procedures laid out in section **4.7 Synthetic Procedures of Aryl Halides**.

^{1}H NMR (600 MHz, $CDCl_3$) δ 7.50 (dd, *J* = 8.3, 2.1 Hz, 1H), 7.35 (d, *J* = 2.1 Hz, 2H), 7.31 (dd, *J* = 8.4, 2.1 Hz, 1H), 7.19 (d, *J* = 8.2 Hz, 1H), 6.90 (d, *J* = 8.5 Hz, 1H), 4.34 – 4.31 (m, 2H), 4.30 – 4.26 (m, 2H), 2.56 (q, *J* = 7.5 Hz, 2H), 1.12 (t, *J* = 7.6 Hz, 3H).

^{13}C NMR (151 MHz, $CDCl_3$) δ 195.7, 148.8, 143.5, 141.5, 140.8, 132.9, 131.1, 130.9, 130.3, 124.7, 119.6, 119.0, 117.5, 64.9, 64.2, 26.0, 15.8.

HRMS (m/z): (ESI) calc. for $C_{17}H_{16}BrO_3$ $[M+H]^+$ 347.0283; found 347.0278.

6.2.2 Coupling products

Dapagliflozin (1)

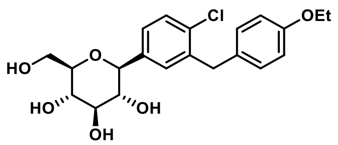

Following **General Procedure B** with glucosyl hydrazide **25** (0.1 mmol), aryl iodide **26**, and base TMG, purification by reverse phase column chromatography afforded Dapagliflozin (**1**) (36 mg, 88% yield, β:α > 19:1).

$[\alpha]_D^{20}$ = +12.1° (c = 1.0, MeOH)

^{1}H NMR (600 MHz, CD3OD) δ 7.36 – 7.30 (m, 2H), 7.27 (dd, *J* = 8.2, 2.1 Hz, 1H), 7.09 (d, *J* = 8.6 Hz, 2H), 6.79 (d, *J* = 8.7 Hz, 2H), 4.08 (d, *J* = 9.5 Hz, 1H), 4.05 (d, *J* = 15.1 Hz, 1H), 4.01 – 3.96 (m, 3H), 3.87 (dd, *J* = 12.0, 1.9 Hz, 1H), 3.69 (dd, *J* = 12.0, 5.2 Hz, 1H), 3.45 (t, *J* = 8.4 Hz, 1H), 3.42 – 3.35 (m, 2H), 3.29 (t, *J* = 9.1 Hz, 1H), 1.35 (t, *J* = 7.0 Hz, 3H).

^{13}C NMR (151 MHz, CD3OD) δ 158.8, 140.0, 139.9, 134.4, 132.9, 131.9, 130.8, 130.1, 128.2, 115.4, 82.9, 82.2, 79.7, 76.5, 71.8, 64.4, 63.1, 39.2, 15.2.

HRMS (m/z): (ESI) calc. for $C_{21}H_{25}ClNaO_6$ $[M+Na]^+$ 431.1237; found 431.1224.

Canagliflozin (30)

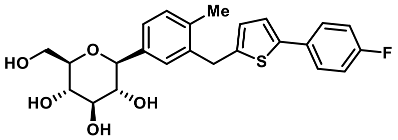

Following **General Procedure B** with glucosyl hydrazide **25** (0.1 mmol), aryl iodide **SG4**, and base TMG, purification by reverse phase column chromatography afforded Canagliflozin (**30**) (29 mg, 66% yield, β:α = 11:1).

$[\alpha]_D^{20}$ = +18.6° (c = 1.0, MeOH)

^{1}H NMR (600 MHz, CD3OD) δ 7.54 – 7.51 (m, 2H), 7.31 (s, 1H), 7.24 (dd, *J* = 7.7, 1.8 Hz, 1H), 7.16 (d, *J* = 7.8 Hz, 1H), 7.11 (d, *J* = 3.6 Hz, 1H), 7.06 (t, *J* = 8.7 Hz, 2H), 6.70 (d, *J* = 3.6 Hz, 1H), 4.19 – 4.08 (m, 3H), 3.88 (dd, *J* = 12.0, 1.9 Hz, 1H), 3.70 (dd, *J* = 11.9, 5.3 Hz, 1H), 3.50 – 3.45 (m, 1H), 3.44 – 3.36 (m, 3H), 2.30 (s, 3H).

^{13}C NMR (151 MHz, CD3OD) δ 163.4 (d, $^1J_{C,F}$ = 245.2 Hz), 145.1, 142.5, 139.2, 138.7, 137.3, 132.5 (d, $^4J_{C,F}$ = 3.5 Hz), 131.2, 130.4, 128.1 (d, $^3J_{C,F}$ = 7.9 Hz), 127.5, 127.1, 123.9, 116.6 (d, $^2J_{C,F}$ = 22.0 Hz), 83.5, 82.2, 79.8, 76.4, 72.0, 63.2, 34.9, 19.3.

HRMS (m/z): (ESI) calc. for $C_{24}H_{25}FNaO_5S$ $[M+Na]^+$ 467.1304; found 467.1312.

Empagliflozin (2)

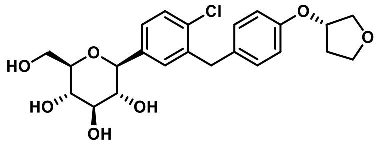

Following **General Procedure B** with glucosyl hydrazide **25** (0.1 mmol), aryl iodide **SG5**, and base TMG, purification by reverse phase column chromatography afforded Empagliflozin (**2**) (40 mg, 90% yield, β:α = 11:1).

$[\alpha]_D^{20}$ = +15.9° (c = 1.0, MeOH)

¹H NMR (600 MHz, CD3OD) δ 7.36 – 7.32 (m, 2H), 7.28 (dd, *J* = 8.2, 2.1 Hz, 1H), 7.11 (d, *J* = 8.6 Hz, 2H), 6.79 (d, *J* = 8.6 Hz, 2H), 4.96 (td, *J* = 4.4, 2.2 Hz, 1H), 4.09 (d, *J* = 9.5 Hz, 1H), 4.03 (q, *J* = 15.1 Hz, 2H), 3.95 – 3.83 (m, 5H), 3.69 (dd, *J* = 12.0, 5.2 Hz, 1H), 3.45 (t, *J* = 8.4 Hz, 1H), 3.42 – 3.35 (m, 2H), 3.29 (t, *J* = 9.1 Hz, 1H), 2.20 (dtd, *J* = 14.3, 8.5, 6.0 Hz, 1H), 2.11 – 2.04 (m, 1H).

¹³C NMR (151 MHz, CD3OD) δ 157.2, 140.1, 139.8, 134.4, 133.4, 131.9, 131.0, 130.1, 128.3, 116.4, 82.9, 82.2, 79.7, 78.5, 76.5, 74.0, 71.8, 68.1, 63.1, 39.2, 33.9.

HRMS (m/z): (ESI) calc. for $C_{23}H_{28}ClO_7$ $[M+H]^+$ 451.1524; found 451.1540.

Ipragliflozin (31)

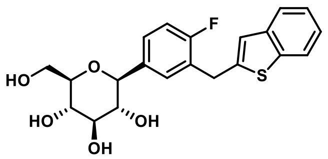

Following **General Procedure B** with glucosyl hydrazide **25** (0.1 mmol), aryl bromide **SG6**, and base TMG, purification by reverse phase column chromatography afforded Ipragliflozin (**31**) (29 mg, 71% yield, β:α = 17:1).

$[\alpha]_D^{20}$= +21.8° (c = 0.5, MeOH)

^{1}H NMR (600 MHz, CD3OD) δ 7.73 (d, *J* = 7.9 Hz, 1H), 7.65 (d, *J* = 7.8 Hz, 1H), 7.43 (dd, *J* = 7.4, 2.2 Hz, 1H), 7.35 (ddd, *J* = 8.4, 5.0, 2.3 Hz, 1H), 7.28 (ddd, *J* = 8.0, 7.1, 1.1 Hz, 1H), 7.23 (ddd, *J* = 8.2, 7.1, 1.3 Hz, 1H), 7.08 (dd, *J* = 9.8, 8.5 Hz, 1H), 7.05 (s, 1H), 4.32 – 4.20 (m, 2H), 4.11 (d, *J* = 9.5 Hz, 1H), 3.87 (dd, *J* = 12.0, 1.9 Hz, 1H), 3.69 (dd, *J* = 11.9, 5.2 Hz, 1H), 3.45 (t, *J* = 8.4 Hz, 1H), 3.42 – 3.36 (m, 2H), 3.34 – 3.29 (m, 1H).

^{13}C NMR (151 MHz, CD3OD) δ 189.9 (d, $^1J_{C,F}$ = 244.9 Hz), 173.2, 169.6, 169.3, 165.4 (d, $^4J_{C,F}$ = 3.5 Hz), 160.0 (d, $^3J_{C,F}$ = 4.3 Hz), 157.5 (d, $^3J_{C,F}$ = 8.2 Hz), 155.6 (d, $^2J_{C,F}$ = 15.9 Hz), 153.4, 152.9, 152.2, 151.1, 151.0, 144.1 (d, $^2J_{C,F}$ = 22.5 Hz), 111.0, 110.4, 107.9, 104.7, 100.0, 91.2, 58.8.

HRMS (m/z): (ESI) calc. for $C_{21}H_{21}FNaO_5S$ $[M+Na]^+$ 427.0991; found 427.0989.

Bexagliflozin (32)

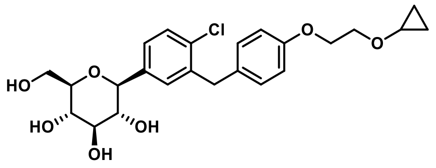

Following **General Procedure B** with glucosyl hydrazide **25** (0.1 mmol), aryl bromide **SG7**, and base TMG, purification by reverse phase column chromatography afforded Bexagliflozin (**32**) (32 mg, 68% yield, β:α = 18:1).

$[\alpha]_D^{20}$= +11.5° (c = 1.0, MeOH)

^{1}H NMR (600 MHz, CD3OD) δ 7.36 – 7.31 (m, 2H), 7.28 (dd, *J* = 8.2, 2.1 Hz, 1H), 7.10 (d, *J* = 8.7 Hz, 2H), 6.83 (d, *J* = 8.6 Hz, 2H), 4.09 (d, *J* = 9.5 Hz, 1H), 4.07 – 4.04 (m, 3H), 4.00 (d, *J* = 15.1 Hz, 1H), 3.87 (dd, *J* = 12.0, 1.9 Hz, 1H), 3.84 – 3.81 (m, 2H), 3.69 (dd, *J* = 12.0, 5.2 Hz, 1H), 3.45 (t, *J* = 8.6 Hz, 1H), 3.42 – 3.35 (m, 3H), 3.29 (dd, *J* = 9.5, 8.8 Hz, 1H), 0.59 – 0.54 (m, 2H), 0.50 – 0.46 (m, 2H).

^{13}C NMR (151 MHz, CD3OD) δ 158.7, 140.0, 139.8, 134.4, 133.3, 131.9, 130.9, 130.1, 128.2, 115.5, 82.9, 82.2, 79.7, 76.5, 71.8, 70.2, 68.3, 63.1, 54.4, 39.2, 5.9.

HRMS (m/z): (ESI) calc. for $C_{24}H_{29}ClNaO_7$ $[M+Na]^+$ 487.1500; found 487.1499.

Velagliflozin (33)

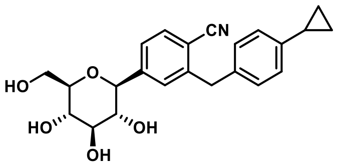

Following **General Procedure B** with glucosyl hydrazide **25** (0.1 mmol), aryl bromide **SG8**, and base TMG, purification by reverse phase column chromatography afforded Velagliflozin (**33**) (27 mg, 67% yield, β:α = 6:1).

$[\boldsymbol{\alpha}]_{\mathbf{D}}^{\mathbf{20}}$= +5.6° (c = 0.5, MeOH)

^{1}H NMR (600 MHz, CD3OD) δ 7.65 (d, *J* = 8.0 Hz, 1H), 7.49 (s, 1H), 7.46 (d, *J* = 1.6 Hz, 1H), 7.11 (d, *J* = 8.2 Hz, 2H), 6.98 (d, *J* = 8.1 Hz, 2H), 4.15 (dd, *J* = 24.9, 9.2 Hz, 3H), 3.88 (dd, *J* = 12.0, 1.9 Hz, 1H), 3.71 (dd, *J* = 12.0, 5.2 Hz, 1H), 3.46 (t, *J* = 8.4 Hz, 1H), 3.43 – 3.36 (m, 2H), 3.24 (t, *J* = 9.1 Hz, 1H), 1.85 (tt, *J* = 8.4, 5.1 Hz, 1H), 0.94 – 0.89 (m, 2H), 0.65 – 0.59 (m, 2H).

^{13}C NMR (151 MHz, CD3OD) δ 175.0, 174.3, 171.8, 165.7, 161.8, 159.0, 157.9, 155.5, 155.0, 147.4, 140.7, 110.9, 110.4, 107.8, 104.6, 99.8, 91.1, 68.8, 43.9, 37.7.

HRMS (m/z): (ESI) calc. for $C_{23}H_{26}NO_5$ $[M+H]^+$ 396.1811; found 396.1804.

Licogliflozin (34)

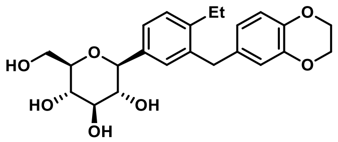

Following **General Procedure B** with glucosyl hydrazide **25** (0.1 mmol), aryl bromide **SG10**, and base TMG, purification by reverse phase column chromatography afforded Licogliflozin (**34**) (21 mg, 50% yield, β:α = 13:1).

$[\boldsymbol{\alpha}]_{\mathbf{D}}^{\mathbf{20}}$= +13.2° (c = 0.5, MeOH)

^{1}H NMR (600 MHz, CD3OD) δ 7.25 (dd, *J* = 7.8, 1.9 Hz, 1H), 7.20 – 7.15 (m, 2H), 6.69 (d, *J* = 8.2 Hz, 1H), 6.58 (dd, *J* = 8.2, 2.1 Hz, 1H), 6.54 (d, *J* = 2.0 Hz, 1H), 4.17 (s, 4H), 4.08 (d, *J* = 9.4 Hz, 1H), 3.95 – 3.85 (m, 3H), 3.69 (dd, *J* = 11.9, 5.2 Hz, 1H), 3.47 (t, *J* = 8.3 Hz, 1H), 3.43 – 3.37 (m, 3H), 2.58 (q, *J* = 7.5 Hz, 2H), 1.07 (t, *J* = 7.6 Hz, 3H).

^{13}C NMR (151 MHz, CD3OD) δ 144.8, 143.5, 143.2, 139.3, 138.2, 135.5, 131.3, 129.5, 127.3, 122.5, 118.2, 117.9, 83.6, 82.2, 79.8, 76.3, 72.0, 65.6, 65.5, 63.2, 39.1, 26.5, 15.5.

HRMS (m/z): (ESI) calc. for $C_{23}H_{28}NaO_7$ $[M+Na]^+$ 439.1733; found 439.1732.

Janagliflozin (35)

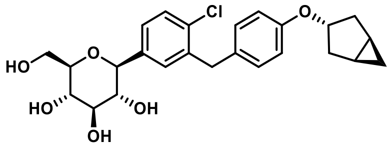

Following **General Procedure B** with glucosyl hydrazide **25** (0.1 mmol), aryl bromide **SG11**, and base TMG, purification by reverse phase column chromatography afforded Janagliflozin (**35**) (33 mg, 71% yield, β:α = 16:1).

$[\boldsymbol{\alpha}]_{\mathbf{D}}^{\mathbf{20}}$= +12.4° (c = 1.0, MeOH)

^{1}H NMR (600 MHz, CD3OD) δ 7.4 – 7.3 (m, 2H), 7.3 (dd, *J* = 8.2, 2.1 Hz, 1H), 7.1 (d, *J* = 8.5 Hz, 2H), 6.8 (d, *J* = 8.5 Hz, 2H), 4.4 (p, *J* = 7.3 Hz, 1H), 4.1 (d, *J* = 9.5 Hz, 1H), 4.0 (q, *J* = 15.1 Hz, 2H), 3.9 (dd, *J* = 12.0, 2.1 Hz, 1H), 3.7 (dd, *J* = 11.9, 5.1 Hz, 1H), 3.4 (t, *J* = 8.4 Hz, 1H), 3.4 – 3.4 (m, 2H), 3.3 (t, *J* = 9.2 Hz, 1H), 2.3 (dd, *J* = 12.8, 7.0 Hz, 2H), 1.8 (ddd, *J* = 12.1, 7.5, 3.3 Hz, 2H), 1.3 (dt, *J* = 8.1, 3.7 Hz, 2H), 0.4 (td, *J* = 8.1, 5.1 Hz, 1H), 0.1 (q, *J* = 4.3 Hz, 1H).

^{13}C NMR (151 MHz, CD3OD) δ 158.3, 140.0, 139.9, 134.4, 131.9, 130.8, 130.1, 128.2, 116.4, 82.9, 82.2, 79.7, 77.7, 76.5, 71.8, 63.1, 39.2, 35.4, 15.8, 10.1.

HRMS (m/z): (ESI) calc. for $C_{25}H_{29}ClNaO_6$ $[M+Na]^+$ 483.1550; found 483.1562.

Enavogliflozin (36)

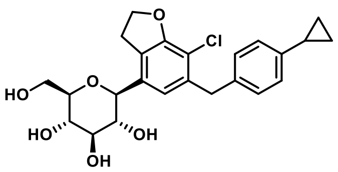

Following **General Procedure B** with glucosyl hydrazide **25** (0.1 mmol), aryl bromide **SG9**, and base TMG, purification by reverse phase column chromatography afforded Enavogliflozin (**36**) (16 mg, 36% yield, β:α = 5:1).

$[\boldsymbol{\alpha}]_{\mathbf{D}}^{\mathbf{20}}$= +9.2° (c = 0.25, MeOH)

^{1}H NMR (600 MHz, CD3OD) δ 7.05 (d, *J* = 7.9 Hz, 2H), 6.94 (d, *J* = 7.9 Hz, 2H), 6.83 (s, 1H), 4.61 (t, *J* = 8.7 Hz, 2H), 4.13 (d, *J* = 8.9 Hz, 1H), 3.98 (q, *J* = 15.0 Hz, 2H), 3.87 (d, *J* = 12.0 Hz, 1H), 3.67 (dd, *J* = 12.0, 4.9 Hz, 1H), 3.48 – 3.39 (m, 3H), 3.38 – 3.27 (m, 3H), 1.84 (td, *J* = 8.5, 4.4 Hz, 1H), 0.93 – 0.87 (m, 2H), 0.64 – 0.57 (m, 2H).

^{13}C NMR (151 MHz, CD3OD) δ 157.7, 143.0, 139.5, 138.2, 135.6, 129.6, 127.3, 126.5, 123.4, 115.5, 82.3, 81.7, 79.7, 75.7, 73.2, 71.9, 63.2, 39.1, 30.7, 15.8, 9.4.

HRMS (m/z): (ESI) calc. for $C_{24}H_{27}ClNaO_6$ $[M+Na]^+$ 469.1394; found 469.1397.

Keto-Dapagliflozin (37)

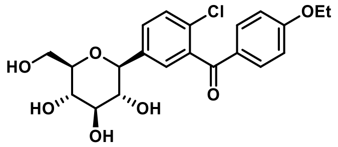

Following **General Procedure B** with glucosyl hydrazide **25** (0.1 mmol), aryl iodide **SG3**, and base TMG, purification by reverse phase column chromatography afforded keto-dapagliflozin (**37**) (39 mg, 93% yield, β:α = 12:1).

$[\alpha]_D^{20}$= +10.5° (c = 1.0, MeOH)

^{1}H NMR (600 MHz, CD3OD) δ 7.76 (d, *J* = 8.8 Hz, 2H), 7.57 (dd, *J* = 8.4, 2.1 Hz, 1H), 7.48 (d, *J* = 8.3 Hz, 1H), 7.45 (d, *J* = 2.1 Hz, 1H), 6.99 (d, *J* = 8.9 Hz, 2H), 4.20 (d, *J* = 9.4 Hz, 1H), 4.13 (q, *J* = 7.0 Hz, 2H), 3.88 (dd, *J* = 12.0, 1.7 Hz, 1H), 3.71 (dd, *J* = 11.9, 5.0 Hz, 1H), 3.48 (t, *J* = 8.6 Hz, 1H), 3.44 – 3.38 (m, 2H), 3.28 (t, *J* = 9.1 Hz, 1H), 1.42 (t, *J* = 7.0 Hz, 3H).

^{13}C NMR (151 MHz, CD3OD) δ 195.8, 165.4, 140.4, 139.8, 133.7, 131.5, 131.1, 130.5, 130.3, 129.1, 115.5, 82.3, 82.2, 79.7, 76.6, 71.7, 65.1, 63.0, 14.9.

HRMS (m/z): (ESI) calc. for $C_{21}H_{24}ClO_7$ $[M+H]^+$ 423.1211; found 423.1212.

Keto-Licogliflozin (SA1)

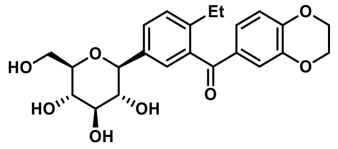

Following **General Procedure B** with glucosyl hydrazide **25** (0.1 mmol), aryl bromide **SG25**, and base TMG, purification by reverse phase column chromatography afforded keto-licogliflozin (**SA1**) (31 mg, 71% yield, β:α = 10:1).

$[\alpha]_D^{20}$= +15.0° (c = 0.5, MeOH)

^{1}H NMR (600 MHz, CD3OD) δ 7.52 (dd, *J* = 7.9, 1.8 Hz, 1H), 7.34 (d, *J* = 8.0 Hz, 1H), 7.33 – 7.27 (m, 3H), 6.91 (d, *J* = 8.4 Hz, 1H), 4.35 – 4.30 (m, 2H), 4.29 – 4.23 (m, 2H), 4.15 (d, *J* = 9.4 Hz, 1H), 3.87 (dd, *J* = 12.0, 1.5 Hz, 1H), 3.71 – 3.66 (m, 1H), 3.48 – 3.45 (m, 1H), 3.43 – 3.37 (m, 2H), 3.34 (d, *J* = 9.3 Hz, 1H), 2.59 (qd, *J* = 7.3, 3.2 Hz, 2H), 1.10 (t, *J* = 7.5 Hz, 3H).

^{13}C NMR (151 MHz, CD3OD) δ 199.4, 150.3, 145.0, 143.2, 139.6, 138.3, 132.4, 130.6, 130.2, 128.3, 125.7, 120.3, 118.3, 83.0, 82.2, 79.7, 76.6, 71.9, 66.1, 65.4, 63.1, 27.1, 16.4.

HRMS (m/z): (ESI) calc. for $C_{23}H_{27}O_8$ $[M+H]^+$ 431.1706; found 431.1712.

Keto-Canagliflozin (SA2)

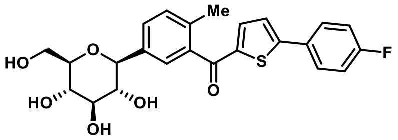

Following **General Procedure B** with glucosyl hydrazide **25** (0.1 mmol), aryl iodide **SG23**, and base TMG, purification by reverse phase column chromatography afforded keto-canagliflozin (**SA2**) (28 mg, 61% yield, β:α = 10:1).

$[\alpha]_D^{20}$= +6.2° (c = 0.5, MeOH)

^{1}H NMR (600 MHz, CD3OD) δ 7.81 – 7.75 (m, 2H), 7.55 (s, 1H), 7.53 (dd, J = 7.9, 1.9 Hz, 1H), 7.47 (d, J = 4.0 Hz, 1H), 7.43 (d, J = 4.0 Hz, 1H), 7.33 (d, J = 7.9 Hz, 1H), 7.20 (t, J = 8.7 Hz, 2H), 4.19 (d, J = 9.4 Hz, 1H), 3.89 (dd, J = 11.9, 1.6 Hz, 1H), 3.73 – 3.69 (m, 1H), 3.49 (t, J = 8.7 Hz, 1H), 3.45 – 3.39 (m, 2H), 3.35 (t, J = 9.2 Hz, 1H), 2.35 (s, 3H).

^{13}C NMR (151 MHz, CD_3OD) δ 192.0, 164.8 (d, $^1J_{C,F}$ = 248.6 Hz), 154.3, 144.4, 138.9, 138.9, 138.4, 137.1, 131.9, 131.0 (d, $^4J_{C,F}$ = 3.8 Hz), 131.0, 129.5 (d, $^3J_{C,F}$ = 8.3 Hz), 128.7, 125.8, 117.2 (d, $^2J_{C,F}$ = 22.3 Hz), 82.9, 82.2, 79.7, 76.6, 71.8, 63.1, 19.5.

HRMS (m/z): (ESI) calc. for $C_{24}H_{24}FO_6S$ $[M+H]^+$ 459.1278; found 459.1295.

Keto-Empagliflozin (SA3)

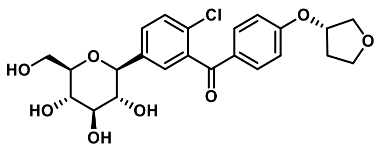

Following **General Procedure B** with glucosyl hydrazide **25** (0.1 mmol), aryl iodide **SG24**, and base TMG, purification by reverse phase column chromatography afforded keto-empagliflozin (**SA3**) (29 mg, 62% yield, β:α = 7:1).

$[\boldsymbol{a}]_{\mathbf{D}}^{\mathbf{20}}$= +15.2° (c = 0.5, MeOH)

^{1}H NMR (600 MHz, CD3OD) δ 7.77 (d, *J* = 8.9 Hz, 2H), 7.58 (dd, *J* = 8.4, 2.1 Hz, 1H), 7.48 (d, *J* = 8.3 Hz, 1H), 7.46 (d, *J* = 2.0 Hz, 1H), 7.00 (d, *J* = 8.9 Hz, 2H), 5.16 – 5.10 (m, 1H), 4.20 (d, *J* = 9.5 Hz, 1H), 3.99 (dd, *J* = 10.3, 4.5 Hz, 1H), 3.98 – 3.92 (m, 2H), 3.91 – 3.86 (m, 2H), 3.71 (dd, *J* = 11.9, 4.9 Hz, 1H), 3.48 (t, *J* = 8.7 Hz, 1H), 3.44 – 3.38 (m, 2H), 3.28 (t, *J* = 9.1 Hz, 1H), 2.30 (dtd, *J* = 14.3, 8.4, 6.0 Hz, 1H), 2.14 – 2.09 (m, 1H).

^{13}C NMR (151 MHz, CD3OD) δ 195.7, 163.8, 140.4, 139.7, 133.8, 131.6, 131.1, 130.7, 130.5, 129.2, 116.4, 82.3, 82.2, 79.7, 79.2, 76.6, 73.9, 71.7, 68.1, 63.0, 33.9.

HRMS (m/z): (ESI) calc. for $C_{23}H_{26}ClO_8$ $[M+H]^+$ 465.1316; found 465.1319.

Compound 38 (from Lactose-hydrazide SH1)

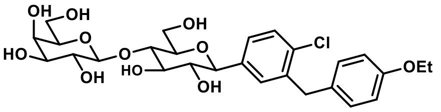

Following **General Procedure B** with lactose-hydrazide **SH1** (0.1 mmol), aryl iodide **26**, and base TMG, purification by reverse phase column chromatography afforded compound **38** (46 mg, 80% yield, β:α = 10:1).

$[\boldsymbol{\alpha}]_{\mathbf{D}}^{\mathbf{20}}$ = +12.8° (c = 1.0, MeOH)

^{1}H NMR (600 MHz, CD$_3$OD) δ 7.36 – 7.32 (m, 2H), 7.28 (dd, *J* = 8.2, 2.1 Hz, 1H), 7.09 (d, *J* = 8.6 Hz, 2H), 6.79 (d, *J* = 8.7 Hz, 2H), 4.40 (d, *J* = 7.7 Hz, 1H), 4.11 (d, *J* = 9.6 Hz, 1H), 4.05 (d, *J* = 15.1 Hz, 1H), 4.01 – 3.95 (m, 3H), 3.91 (dd, *J* = 12.2, 2.5 Hz, 1H), 3.86 (dd, *J* = 12.2, 4.2 Hz, 1H), 3.83 – 3.78 (m, 2H), 3.73 – 3.66 (m, 2H), 3.64 – 3.55 (m, 3H), 3.51 (ddd, *J* = 9.8, 5.5, 3.0 Hz, 2H), 3.37 (t, *J* = 9.2 Hz, 1H), 1.35 (t, *J* = 7.0 Hz, 3H).

^{13}C NMR (151 MHz, CD$_3$OD) δ 158.8, 139.9, 139.8, 134.5, 132.8, 131.9, 130.8, 130.1, 128.2, 115.4, 105.1, 82.7, 80.7, 80.7, 78.0, 77.1, 76.1, 74.8, 72.6, 70.3, 64.4, 62.5, 62.2, 39.2, 15.2.

HRMS (m/z): (ESI) calc. for $C_{27}H_{36}ClO_{11}$ $[M+H]^+$ 571.1946; found 571.1944.

Compound 39 (from Cellobiose-hydrazide SH2)

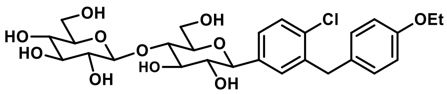

Following **General Procedure B** with Cellobiose-hydrazide **SH2** (0.1 mmol), aryl iodide **26**, and base TMG, purification by reverse phase column chromatography afforded compound **39** (40 mg, 70% yield, β:α = 10:1).

$[\boldsymbol{a}]_{\mathbf{D}}^{\mathbf{20}}$ = +9.4° (c = 1.0, MeOH)

^{1}H NMR (600 MHz, CD_3OD) δ 7.35 – 7.32 (m, 2H), 7.28 (dd, *J* = 8.2, 2.1 Hz, 1H), 7.09 (d, *J* = 8.6 Hz, 2H), 6.79 (d, *J* = 8.6 Hz, 2H), 4.45 (d, *J* = 7.9 Hz, 1H), 4.11 (d, *J* = 9.5 Hz, 1H), 4.05 (d, *J* = 15.1 Hz, 1H), 4.01 – 3.95 (m, 3H), 3.89 (dq, *J* = 8.4, 2.8 Hz, 3H), 3.71 – 3.65 (m, 2H), 3.60 (t, *J* = 8.8 Hz, 1H), 3.50 (dt, *J* = 9.7, 3.3 Hz, 1H), 3.41 – 3.33 (m, 4H), 3.26 (dd, *J* = 9.2, 7.8 Hz, 1H), 1.35 (t, *J* = 7.0 Hz, 3H).

^{13}C NMR (151 MHz, CD_3OD) δ 158.8, 139.9, 139.8, 134.5, 132.9, 131.9, 130.8, 130.1, 128.2, 115.4, 104.6, 82.7, 80.8, 80.7, 78.1, 78.0, 77.9, 76.2, 74.9, 71.4, 64.4, 62.5, 62.1, 39.2, 15.2.

HRMS (m/z): (ESI) calc. for $C_{27}H_{36}ClO_{11}$ $[M+H]^{+}$ 571.1946; found 571.1942.

Compound 40 (from Maltose-hydrazide SH3)

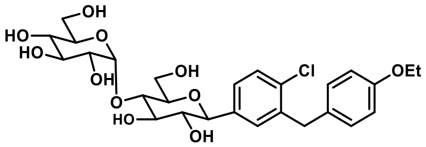

Following **General Procedure B** with Maltose-hydrazide **SH3** (0.1 mmol), aryl iodide **26**, and base TMG, purification by reverse phase column chromatography afforded compound **40** (22 mg, 39% yield, β:α = 10:1).

$[\boldsymbol{\alpha}]_{\mathbf{D}}^{\mathbf{20}}$ = +61.0° (c = 0.2, MeOH)

^{1}H NMR (600 MHz, CD_3OD) δ 7.34 (s, 1H), 7.29 (s, 1H), 7.25 (dd, *J* = 8.2, 2.2 Hz, 1H), 7.10 (d, *J* = 8.3 Hz, 2H), 6.81 (d, *J* = 8.5 Hz, 2H), 4.87 (d, *J* = 3.7 Hz, 1H), 4.08 (d, *J* = 9.5 Hz, 1H), 4.06 – 3.96 (m, 4H), 3.90 (dd, *J* = 11.3, 5.4 Hz, 1H), 3.83 (dd, *J* = 11.3, 2.1 Hz, 1H), 3.75 – 3.71 (m, 1H), 3.62 (dt, *J* = 9.5, 4.7 Hz, 3H), 3.56 (t, *J* = 6.7 Hz, 1H), 3.46 (p, *J* = 8.9 Hz, 2H), 3.39 – 3.33 (m, 2H), 3.30 – 3.26 (m, 1H), 1.36 (t, *J* = 7.0 Hz, 3H).

^{13}C NMR (151 MHz, CD_3OD) δ 158.9, 140.0, 139.9, 134.5, 132.8, 131.8, 130.9, 130.2, 128.1, 115.5, 100.2, 82.9, 80.8, 79.7, 76.3, 75.1, 73.7, 73.5, 71.9, 71.6, 68.2, 64.4, 62.5, 39.2, 15.2.

HRMS (m/z): (ESI) calc. for $C_{27}H_{29}ClO_8$ $[M–3OH]^+$ 517.1629; found 517.1631.

Compound 41 (from Maltotriose-hydrazide SH4)

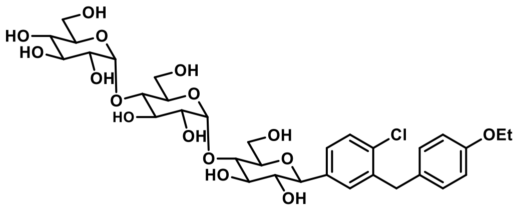

Following **General Procedure B** with Maltotriose-hydrazide **SH4** (0.1 mmol), aryl iodide **26**, and base TMG, purification by reverse phase column chromatography afforded compound **41** (47 mg, 64% yield, β:α = 12:1).

$[\boldsymbol{a}]_{\mathbf{D}}^{\mathbf{20}}$ = +82.6° (c = 0.5, MeOH)

^{1}H NMR (600 MHz, CD_3OD) δ 7.37 – 7.31 (m, 2H), 7.28 (dd, *J* = 8.3, 2.1 Hz, 1H), 7.09 (d, *J* = 8.2 Hz, 2H), 6.79 (d, *J* = 8.3 Hz, 2H), 5.21 (d, *J* = 3.7 Hz, 1H), 5.16 (d, *J* = 3.8 Hz, 1H), 4.10 (d, *J* = 9.6 Hz, 1H), 4.05 (d, *J* = 15.1 Hz, 1H), 4.01 – 3.95 (m, 3H), 3.93 – 3.76 (m, 7H), 3.66 (ddt, *J* = 32.7, 11.9, 9.2 Hz, 5H), 3.52 (ddd, *J* = 9.4, 6.5, 2.6 Hz, 2H), 3.46 (ddd, *J* = 13.7, 9.1, 3.5 Hz, 2H), 3.39 – 3.32 (m, 2H), 3.27 (t, *J* = 9.2 Hz, 1H), 1.36 (t, *J* = 7.0 Hz, 3H).

^{13}C NMR (151 MHz, CD_3OD) δ 157.4, 138.5, 138.4, 133.1, 131.5, 130.5, 129.4, 128.7, 126.8, 114.0, 101.5, 101.4, 81.4, 80.2, 80.0, 79.4, 78.1, 74.6, 73.7, 73.6, 73.4, 72.9, 72.5, 72.0, 70.1, 63.0, 61.3, 61.1, 60.7, 37.8, 13.8.

HRMS (m/z): (ESI) calc. for $C_{33}H_{45}ClNaO_{16}$ $[M+Na]^+$ 755.2294; found 755.2277.

6.3 Characterization Data of Bn-protected Sugar Sulfonylhydrazides

6.3.1 Characterization of C1-Hydrazides and RCC-products (for stereorententive coupling)

6.3.1.1 Synthetic precursors

Compound SE4

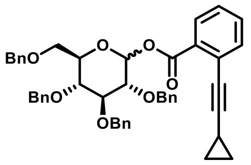

Following general procedures laid out in section **4.3.1 Synthesis of glucose C1 hydrazide** with Bn-protected glucose **SE1** and acid **SE3**, purification through flash column chromatography afforded ester **SE4**.

The two anomers were characterized as a mixture (α:β = 1:6.2):

^{1}H NMR (600 MHz, $CDCl_3$) δ 7.97 (dd, *J* = 7.9, 1.4 Hz, 1H), 7.94 (dd, *J* = 8.0, 1.4 Hz, 6H), 7.53 – 7.41 (m, 17H), 7.36 – 7.24 (m, 112H), 7.21 (s, 31H), 7.18 (dd, *J* = 7.6, 1.9 Hz, 13H), 7.12 – 7.10 (m, 2H), 6.68 (d, *J* = 3.5 Hz, 1H, **$H_{\alpha\text{-anomer}}$**), 5.90 (d, *J* = 7.8 Hz, 6H, **$H_{\beta\text{-anomer}}$**), 4.98 (s, 1H), 4.93 (d, *J* = 11.0 Hz, 6H), 4.88 – 4.75 (m, 29H), 4.68 – 4.62 (m, 9H), 4.59 (d, *J* = 10.7 Hz, 6H), 4.54 – 4.48 (m, 9H), 4.11 – 4.08 (m, 1H), 3.86 – 3.74 (m, 36H), 3.71 – 3.65 (m, 8H), 0.93 – 0.81 (m, 29H), 0.77 – 0.72 (m, 2H).

^{13}C NMR (151 MHz, $CDCl_3$) δ 164.6, 164.1, 138.8, 138.6, 138.3, 138.2, 138.1, 138.0, 138.0, 137.9, 134.8, 134.5, 132.3, 132.1, 131.1, 131.0, 130.9, 130.5, 128.6, 128.5, 128.5, 128.3, 128.2, 128.1, 128.1, 128.0, 128.0, 127.9, 127.9, 127.8, 127.8, 127.2, 127.1, 125.6, 125.2, 94.7, 91.0, 85.0, 81.9, 81.2, 79.2, 75.9, 75.8, 75.8, 75.4, 75.2, 75.2, 75.1, 74.7, 73.7, 73.6, 73.4, 73.1, 68.3, 68.2, 9.1, 9.1,

HRMS (m/z): (ESI) calc. for $C_{46}H_{44}NaO_7$ $[M+Na]^+$: 731.2985 found 731.2974

Compound SE2

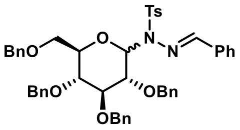

Following general procedures laid out in section **4.3.1 Synthesis of glucose C1 hydrazide**, purification through flash column chromatography afforded hydrazone **SE2**.

Alpha anomer: The compound was isolated as a mixture (α:β = 3:1) and characterized from there.

^{1}H NMR (600 MHz, $CDCl_3$) δ 8.62 (s, 1H), 7.79 – 7.77 (m, 2H), 7.68 – 7.66 (m, 2H), 7.46 – 7.14 (m, 23H), 7.02 (d, *J* = 7.8 Hz, 2H), 6.29 (d, *J* = 6.7 Hz, 1H), 4.86 (dd, *J* = 12.9, 11.3 Hz, 2H), 4.79 (d, *J* = 11.1 Hz, 1H), 4.66 (d, *J* = 11.5 Hz, 1H), 4.60 (d, *J* = 11.1 Hz, 1H), 4.49 – 4.42 (m, 3H), 4.37 (d, *J* = 8.7 Hz, 1H), 3.99 – 3.94 (m, 2H), 3.70 (dd, *J* = 10.1, 8.6 Hz, 1H), 3.34 (dd, *J* = 10.6, 3.2 Hz, 1H), 2.94 (dd, *J* = 10.6, 2.0 Hz, 1H), 2.28 (s, 3H).

^{13}C NMR (151 MHz, $CDCl_3$) δ 160.3, 144.1, 139.0, 138.7, 138.1, 137.8, 136.0, 133.8, 131.4, 129.4, 129.0, 128.8, 128.6, 128.54, 128.50, 128.35, 128.32, 128.26, 128.2, 127.93, 127.90, 127.6, 127.5, 82.5, 81.9, 78.4, 77.4, 75.2, 74.6, 74.5, 73.7, 72.2, 68.0, 21.6.

HRMS (m/z): (ESI) calc. for $C_{48}H_{49}N_2O_7S$ $[M+H]^+$:797.3260 found 797.3262

Beta anomer: The compound was isolated as a mixture (α:β = 1:6) and characterized from there.

^{1}H NMR (600 MHz, $CDCl_3$) δ 8.52 (s, 1H), 7.89 – 7.85 (m, 2H), 7.57 – 7.55 (m, 2H), 7.38 – 7.23 (m, 23H), 7.06 (d, *J* = 7.9 Hz, 2H), 5.58 (d, *J* = 9.0 Hz, 1H), 4.94 (d, *J* = 10.7 Hz, 1H), 4.89 (d, *J* = 2.7 Hz, 1H), 4.88 (d, *J* = 2.9 Hz, 1H), 4.70 (d, *J* = 6.3 Hz, 2H), 4.65 (d, *J* = 10.8 Hz, 1H), 4.54 (d, *J* = 12.0 Hz, 1H), 4.38 (d, *J* = 11.9 Hz, 1H), 4.15 (t, *J* = 9.0 Hz, 1H), 3.87 – 3.82 (m, 1H), 3.78 – 3.74 (m, 1H), 3.67 – 3.66 (m, 2H), 3.63 – 3.60 (m, 1H), 2.29 (s, 3H).

^{13}C NMR (151 MHz, $CDCl_3$) δ 152.3, 144.4, 138.51, 138.46, 138.3, 138.2, 135.5, 134.3, 130.7, 129.5, 129.0, 128.7, 128.6, 128.5, 128.4, 128.2, 128.1, 128.0, 127.9, 127.80,

127.76, 127.6, 88.2, 86.3, 78.6, 77.9, 76.1, 75.2, 74.8, 73.5, 69.0, 21.7.

HRMS (m/z): (ESI) calc. for $C_{48}H_{49}N_2O_7S$ $[M+H]^+$: 797.3260 found 797.3260

Compound SE6

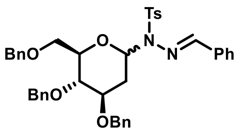

Following general procedures laid out in section **4.3.2 Synthesis of 2-deoxy-glucose C1 hydrazide**, purification through flash column chromatography afforded hydrazone **SE6**.

β-SE6:

$[\alpha]_D^{20}$ = +24.0° (c = 0.5, $CHCl_3$)

^{1}H NMR (600 MHz, $CDCl_3$) δ 8.64 (s, 1H), 7.80 (d, *J* = 8.4 Hz, 2H), 7.63 (d, *J* = 7.0 Hz, 2H), 7.44 – 7.39 (m, 1H), 7.38 – 7.22 (m, 17H), 7.12 (d, *J* = 7.8 Hz, 2H), 5.56 (dd, *J* = 11.0, 2.3 Hz, 1H), 4.90 (d, *J* = 10.9 Hz, 1H), 4.67 (d, *J* = 11.5 Hz, 1H), 4.61 (dd, *J* = 11.2, 2.5 Hz, 2H), 4.49 (d, *J* = 12.0 Hz, 1H), 4.29 (d, *J* = 12.0 Hz, 1H), 3.81 – 3.73 (m, 2H), 3.59 – 3.52 (m, 2H), 3.52 – 3.47 (m, 1H), 2.32 (s, 3H), 2.17 (ddd, *J* = 11.8, 5.0, 2.5 Hz, 1H), 2.14 – 2.04 (m, 1H).

^{13}C NMR (151 MHz, $CDCl_3$) δ 158.9, 144.3, 138.7, 138.5, 138.2, 135.4, 133.9, 131.2, 129.4, 129.2, 128.8, 128.6, 128.51, 128.46, 128.3, 128.2, 127.9, 127.8, 127.60, 127.56, 85.1, 80.2, 77.9, 77.5, 75.2, 73.5, 71.9, 69.2, 34.4, 21.7.

HRMS (m/z): (ESI) calc. for $C_{41}H_{43}N_2O_6S$ $[M+H]^+$: 691.2842 found 691.2842

α-SE6:

$[\alpha]_D^{20}$ = +56.8° (c = 0.5, $CHCl_3$)

^{1}H NMR (600 MHz, $CDCl_3$) δ 8.66 (s, 1H), 7.80 (d, *J* = 8.3 Hz, 2H), 7.70 – 7.65 (m, 2H), 7.54 – 7.50 (m, 1H), 7.47 – 7.32 (m, 11H), 7.29 – 7.24 (m, 5H), 7.16 – 7.12 (m, 2H), 5.99 (dd, *J* = 6.1, 3.2 Hz, 1H), 4.87 (d, *J* = 11.2 Hz, 1H), 4.63 – 4.50 (m, 5H), 4.21 (ddd, *J* = 9.4, 7.2, 4.8 Hz, 1H), 3.98 (ddd, *J* = 9.1, 3.8, 2.4 Hz, 1H), 3.71 (dd, *J* = 9.1, 7.3 Hz, 1H), 3.61 (dd, *J* = 10.6, 3.8 Hz, 1H), 3.29 (dd, *J* = 10.6, 2.4 Hz, 1H), 2.42 (ddd, *J* = 14.0, 5.0, 3.2 Hz, 1H), 2.38 (s, 3H), 2.08 (ddd, *J* = 14.0, 9.5, 6.2 Hz, 1H).

^{13}C NMR (151 MHz, $CDCl_3$) δ 160.5, 144.2, 138.7, 138.3, 135.2, 133.7, 131.5, 129.4, 129.0, 128.9, 128.5, 128.4, 128.1, 127.89, 127.87, 127.8, 127.78, 127.73, 83.5, 76.5, 74.5, 74.1, 73.6, 71.5, 68.5, 33.0, 21.7.

HRMS (m/z): (ESI) calc. for $C_{41}H_{43}N_2O_6S$ $[M+H]^+$: 691.2842 found 691.2835

Compound SE7

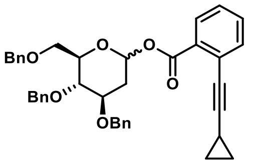

Following general procedures laid out in section **4.3.2 Synthesis of 2-deoxy-glucose C1 hydrazide**, purification through flash column chromatography afforded ester **SE7**. The two anomers were not separated and characterized as a mixture (α:β = 1:2.8):

^{1}H NMR (600 MHz, $CDCl_3$) δ 7.98 (dd, *J* = 8.0, 1.4 Hz, 3H), 7.87 (dd, *J* = 7.9, 1.4 Hz, 1H), 7.51 – 7.46 (m, 4H), 7.45 – 7.41 (m, 4H), 7.38 – 7.25 (m, 55H), 7.24 – 7.21 (m, 6H), 7.17 (dd, *J* = 7.5, 2.0 Hz, 2H), 6.56 (dd, *J* = 3.5, 1.6 Hz, 1H, $\mathbf{H_{\alpha\text{-anomer}}}$), 5.96 (dd, *J* = 9.9, 2.2 Hz, 3H, $\mathbf{H_{\beta\text{-anomer}}}$), 4.93 (d, *J* = 10.8 Hz, 4H), 4.72 (d, *J* = 11.6 Hz, 4H), 4.71 – 4.68 (m, 2H), 4.67 – 4.57 (m, 11H), 4.53 (d, *J* = 12.1 Hz, 4H), 4.18 (ddd, *J* = 11.7, 9.1, 5.0 Hz, 1H), 4.10 (ddd, *J* = 10.0, 3.4, 2.0 Hz, 1H), 3.87 – 3.77 (m, 11H), 3.75 – 3.70 (m, 4H), 3.62 (ddd, *J* = 9.5, 3.9, 2.2 Hz, 3H), 2.53 (ddd, *J* = 12.3, 4.9, 2.2 Hz, 3H), 2.47 (ddd, *J* = 13.5, 5.0, 1.7 Hz, 1H), 2.00 – 1.89 (m, 4H), 1.57 (tt, *J* = 8.3, 5.0 Hz, 1H), 1.51 (tt, *J* = 8.0, 5.2 Hz, 3H), 0.92 – 0.85 (m, 11H), 0.83 – 0.81 (m, 2H), 0.77 – 0.74 (m, 2H).

^{13}C NMR (151 MHz, $CDCl_3$) δ 164.7, 164.3, 138.5, 138.5, 138.4, 138.3, 138.2, 138.1, 134.7, 134.4, 132.1, 132.0, 130.9, 130.8, 130.8, 128.6, 128.5, 128.5, 128.4, 128.4, 128.1, 128.0, 127.9, 127.8, 127.8, 127.8, 127.8, 127.7, 127.1, 127.0, 125.2, 124.9, 99.9, 99.9, 93.4, 92.9, 79.1, 77.7, 77.5, 76.0, 75.2, 75.1, 74.6, 74.0, 73.6, 73.6, 72.1, 71.8, 68.7, 68.5, 35.5, 34.7, 9.1, 9.0, 0.8, 0.8.

HRMS (m/z): (ESI) calc. for $C_{39}H_{39}O_6$ $[M+H]^+$: 603.2747 found 603.2739

Compound SE9

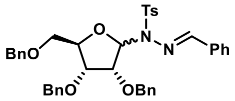

Following general procedures laid out in section **4.3.3 Synthesis of ribose C1 hydrazide**, purification through flash column chromatography afforded hydrazone **SE9**. **The two anomers were not separated and characterized as a mixture (α:β = 2.4:1):**

^{1}H NMR (600 MHz, $CDCl_3$) δ .57 (s, 1H), 8.55 (s, 2H), 7.81 – 7.78 (m, 7H), 7.54 – 7.52 (m, 5H), 7.43 – 7.26 (m, 33H), 7.25 – 7.15 (m, 33H), 7.10 – 7.07 (m, 7H), 6.31 (d, *J* = 5.7 Hz, 2H, **$H_{α\text{-anomer}}$**), 6.21 – 6.20 (m, 1H, **$H_{β\text{-anomer}}$**), 4.86 (d, *J* = 11.6 Hz, 2H), 4.66 – 4.52 (m, 8H), 4.43 (d, *J* = 12.0 Hz, 4H), 4.38 (d, *J* = 12.1 Hz, 2H), 4.33 (ddd, *J* = 6.4, 3.8, 2.8 Hz, 3H), 4.30 (d, *J* = 11.6 Hz, 3H), 4.26 – 4.20 (m, 6H), 4.07 – 4.03 (m, 2H), 3.97 (t, *J* = 6.4 Hz, 2H), 3.54 (dd, *J* = 11.1, 3.4 Hz, 1H), 3.50 (dd, *J* = 11.0, 2.8 Hz, 2H), 3.42 (dd, *J* = 11.1, 5.3 Hz, 1H), 3.38 (dd, *J* = 11.0, 3.9 Hz, 2H), 2.34 (s, 7H), 2.27 (s, 3H).

^{13}C NMR (151 MHz, $CDCl_3$) δ 160.5, 158.0, 144.4, 143.9, 138.4, 138.3, 137.9, 137.8, 137.6, 135.6, 135.2, 134.4, 133.8, 131.1, 130.7, 129.5, 129.4, 129.0, 128.9, 128.7, 128.5, 128.5, 128.4, 128.3, 128.3, 128.1, 128.0, 128.0, 128.0, 127.9, 127.8, 127.8, 127.7, 127.7, 127.5, 93.0, 88.8, 82.9, 81.2, 78.8, 77.0, 73.4, 73.3, 72.9, 72.7, 72.3, 72.1, 70.2, 69.8, 21.7, 21.6.

HRMS (m/z): (ESI) calc. for $C_{40}H_{41}N_2O_6S$ $[M+H]^+$: 677.2685 found 677.2704

Compound SE10

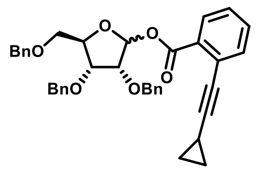

Following general procedures laid out in section **4.3.3 Synthesis of ribose C1 hydrazide**, purification through flash column chromatography afforded ester **SE10**. **The two anomers were not separated and characterized as a mixture (dr = 1:8):**

^{1}H NMR (600 MHz, $CDCl_3$) δ 8.0 (dd, *J* = 8.0, 1.4 Hz, 1H), 7.7 (dd, *J* = 7.9, 1.3 Hz, 8H), 7.5 (ddd, *J* = 9.4, 7.9, 1.3 Hz, 26H), 7.4 – 7.3 (m, 94H), 7.2 (d, *J* = 2.5 Hz, 43H), 7.1 (td, *J* = 7.6, 1.3 Hz, 8H), 7.1 (td, *J* = 7.7, 1.3 Hz, 1H), 6.6 (d, *J* = 4.2 Hz, 1H), 6.5 (s, 8H), 4.9 (d, *J* = 12.2 Hz, 8H), 4.8 (d, *J* = 11.6 Hz, 1H), 4.7 – 4.7 (m, 9H), 4.6 – 4.4 (m, 49H), 4.3 (dd, *J* = 7.9, 4.6 Hz, 8H), 4.1 (d, *J* = 4.6 Hz, 9H), 4.0 (dd, *J* = 6.1, 2.5 Hz, 1H), 3.8 (dd, *J* = 11.1, 3.2 Hz, 8H), 3.7 (dd, *J* = 11.1, 4.5 Hz, 8H), 3.5 (d, *J* = 3.7 Hz, 2H), 1.5 (tt, *J* = 7.4, 5.4 Hz, 1H), 1.4 – 1.4 (m, 8H), 0.9 – 0.8 (m, 39H).

^{13}C NMR (151 MHz, $CDCl_3$) δ 165.0, 164.7, 138.4, 138.3, 138.0, 137.8, 137.7, 137.6, 134.4, 134.1, 131.9, 131.7, 131.6, 131.4, 131.1, 130.7, 128.6, 128.6, 128.5, 128.4, 128.3, 128.1, 128.1, 128.0, 127.9, 127.9, 127.7, 127.6, 127.5, 127.1, 127.0, 125.2, 125.0, 99.8, 99.7, 99.6, 95.3, 84.4, 81.8, 78.8, 78.7, 77.2, 76.3, 74.6, 73.7, 73.4, 73.1, 72.8, 72.6, 72.2, 70.0, 69.9, 9.1, 9.1, 9.0, 9.0, 0.9, 0.8.

HRMS (m/z): (ESI) calc. for $C_{38}H_{37}O_6$ $[M+H]^+$: 589.2590 found 589.2584

Compound SE12

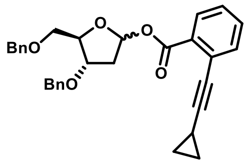

Following general procedures laid out in section **4.3.4 Synthesis of 2-deoxy-ribose C1 hydrazide**, purification through flash column chromatography afforded ester **SE12**.

The two anomers were characterized as a mixture (dr = 1.5:1):

^{1}H NMR (600 MHz, $CDCl_3$) δ 8.08 (d, *J* = 8.0 Hz, 3H), 7.91 (d, *J* = 8.0 Hz, 1H), 7.78 (d, *J* = 7.9 Hz, 2H), 7.52 (d, *J* = 7.8 Hz, 3H), 7.50 – 7.44 (m, 6H), 7.42 – 7.23 (m, 70H), 7.19 (dt, *J* = 18.2, 7.7 Hz, 4H), 6.61 (dd, *J* = 9.2, 5.3 Hz, 3H), 5.55 (dd, *J* = 5.0, 2.6 Hz, 1H), 5.48 (d, *J* = 4.1 Hz, 1H), 4.62 – 4.43 (m, 29H), 4.40 – 4.34 (m, 4H), 4.27 (s, 2H), 4.22 – 4.20 (m, 1H), 4.11 (d, *J* = 4.8 Hz, 1H), 3.69 – 3.49 (m, 12H), 3.36 (dd, *J* = 10.2, 5.9 Hz, 1H), 2.55 (dd, *J* = 13.9, 6.8 Hz, 2H), 2.48 – 2.36 (m, 5H), 2.26 – 2.18 (m, 2H), 2.15 – 2.08 (m, 4H), 1.53 (dtd, *J* = 15.2, 8.1, 3.7 Hz, 4H), 1.45 – 1.37 (m, 2H), 0.98 – 0.82 (m, 26H).

^{13}C NMR (151 MHz, $CDCl_3$) δ 169.0, 165.4, 165.2, 138.2, 138.1, 138.1, 138.0, 137.5, 137.2, 134.4, 134.3, 134.2, 132.5, 131.8, 131.7, 131.7, 131.5, 130.9, 130.7, 130.5, 128.7, 128.7, 128.6, 128.5, 128.5, 128.5, 128.2, 128.0, 128.0, 127.9, 127.9, 127.8, 127.8, 127.8, 127.7, 127.6, 127.1, 127.0, 125.0, 124.8, 124.3, 101.4, 99.6, 99.6, 99.5, 85.0, 84.4, 83.4, 82.6, 80.2, 79.8, 79.2, 78.9, 74.7, 74.7, 74.2, 73.9, 73.7, 73.6, 73.5, 71.9, 71.6, 71.5, 71.5, 71.4, 71.0, 70.5, 70.1, 42.0, 39.2, 39.2, 38.3, 9.1, 9.0, 0.8, 0.8, 0.7.

HRMS (m/z): (ESI) calc. for $C_{31}H_{31}O_5$ $[M+H]^+$: 483.2171 found 483.2174

Compound SE13

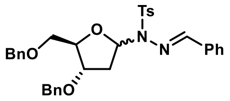

Following general procedures laid out in section **4.3.4 Synthesis of 2-deoxy-ribose C1 hydrazide**, purification through flash column chromatography afforded hydrazone **SE13**.

β-SE13:

$[\boldsymbol{\alpha}]_{\mathbf{D}}^{\mathbf{20}}$ = +66.2° (c = 0.5, $CHCl_3$)

^{1}H NMR (600 MHz, $CDCl_3$) δ 8.61 (s, 1H), 7.80 (d, *J* = 8.4 Hz, 2H), 7.65 (dd, *J* = 8.3, 1.4 Hz, 2H), 7.38 – 7.25 (m, 11H), 7.22 – 7.20 (m, 2H), 7.18 (d, *J* = 7.9 Hz, 2H), 6.26 (dd, *J* = 7.7, 5.5 Hz, 1H), 4.52 (d, *J* = 12.0 Hz, 1H), 4.47 (d, *J* = 13.6 Hz, 2H), 4.40 (d, *J* = 11.7 Hz, 1H), 4.24 (ddd, *J* = 6.2, 4.6, 3.0 Hz, 1H), 4.07 (dt, *J* = 8.2, 6.1 Hz, 1H), 3.59 (dd, *J* = 10.8, 3.0 Hz, 1H), 3.49 (dd, *J* = 10.8, 4.7 Hz, 1H), 2.47 (dt, *J* = 13.7, 7.9 Hz, 1H), 2.33 (s, 3H), 2.32 – 2.28 (m, 1H).

^{13}C NMR (151 MHz, $CDCl_3$) δ 157.8, 144.3, 138.3, 138.0, 135.2, 134.3, 130.9, 129.5, 128.8, 128.7, 128.52, 128.48, 128.3, 127.9, 127.82, 127.79, 89.6, 82.9, 79.0, 73.5, 72.3, 70.4, 35.6, 21.7.

HRMS (m/z): (ESI) calc. for $C_{33}H_{35}N_2O_5S$ $[M+H]^+$: 571.2267 found 571.2278

α-SE13:

$[\boldsymbol{\alpha}]_{\mathbf{D}}^{\mathbf{20}}$ = –27.4° (c = 0.5, $CHCl_3$)

^{1}H NMR (600 MHz, $CDCl_3$) δ 8.63 (s, 1H), 7.77 (d, *J* = 8.4 Hz, 2H), 7.51 (dd, *J* = 8.2, 1.3 Hz, 2H), 7.43 (ddt, *J* = 8.7, 6.9, 1.3 Hz, 1H), 7.34 (dd, *J* = 8.3, 7.1 Hz, 2H), 7.31 – 7.22 (m, 8H), 7.20 (d, *J* = 7.8 Hz, 2H), 7.16 – 7.13 (m, 2H), 6.33 (dd, *J* = 7.4, 4.5 Hz, 1H), 4.50 – 4.44 (m, 2H), 4.30 (d, *J* = 12.0 Hz, 1H), 4.25 (d, *J* = 12.1 Hz, 1H), 4.10 (tt, *J* = 6.5, 4.8 Hz, 2H), 3.43 (dd, *J* = 10.3, 4.9 Hz, 1H), 3.32 (dd, *J* = 10.3, 5.6 Hz, 1H), 2.33 – 2.27 (m, 4H), 2.15 (ddd, *J* = 13.3, 7.5, 5.3 Hz, 1H).

^{13}C NMR (151 MHz, $CDCl_3$) δ 159.8, 144.3, 138.3, 137.9, 135.3, 134.0, 131.3, 129.4, 128.95, 128.92, 128.5, 128.4, 128.1, 127.9, 127.62, 127.58, 90.2, 83.4, 79.0, 73.3, 71.7, 71.0, 35.6, 21.7.

HRMS (m/z): (ESI) calc. for $C_{33}H_{35}N_2O_5S$ $[M+H]^+$: 571.2267 found 571.2261

6.3.1.2. Hydrazides

Glucose Hydrazide α-83

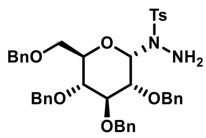

Following general procedures laid out in section **4.3.1 Synthesis of glucose C1 hydrazide**, purification through flash column chromatography afforded hydrazide **α-83**.

$[\alpha]_D^{20}$ = +65.1° (c = 1.0, $CHCl_3$)

^{1}H NMR (600 MHz, $CDCl_3$) δ 7.82 (d, *J* = 8.3 Hz, 2H), 7.36 – 7.26 (m, 16H), 7.26 – 7.24 (m, 2H), 7.15 – 7.12 (m, 2H), 7.09 (d, *J* = 8.0 Hz, 2H), 6.01 (d, *J* = 6.5 Hz, 1H), 4.93 (d, *J* = 11.0 Hz, 1H), 4.79 (d, *J* = 10.9 Hz, 1H), 4.77 (d, *J* = 11.0 Hz, 1H), 4.73 (d, *J* = 11.2 Hz, 1H), 4.68 (t, *J* = 8.6 Hz, 1H), 4.59 (d, *J* = 11.1 Hz, 1H), 4.54 – 4.47 (m, 3H), 3.92 (dd, *J* = 8.9, 6.5 Hz, 1H), 3.87 – 3.81 (m, 3H), 3.63 (dd, *J* = 10.0, 8.4 Hz, 1H), 3.54 (dd, *J* = 10.5, 4.1 Hz, 1H), 3.48 (dd, *J* = 10.5, 2.1 Hz, 1H), 2.31 (s, 3H).

^{13}C NMR (151 MHz, $CDCl_3$) δ 144.3, 138.9, 138.3, 138.1, 137.7, 134.1, 129.8, 128.57, 128.56, 128.5, 128.5, 128.4, 128.2, 128.1, 128.0, 127.9, 127.8, 127.7, 82.1, 81.5, 78.9, 77.8, 75.2, 75.0, 73.7, 73.5, 73.0, 69.1, 21.7.

HRMS (m/z): (ESI) calc. for $C_{41}H_{45}N_2O_7S$ $[M+H]^+$: 709.2947 found 709.2912.

Glucose Hydrazide β-83

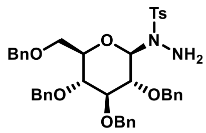

Following general procedures laid out in section **4.3.1 Synthesis of glucose C1 hydrazide**, purification through flash column chromatography afforded hydrazide **β-83**.

$[\alpha]_D^{25}$ = –4.8° (c = 0.25, $CHCl_3$)

^{1}H NMR (600 MHz, $CDCl_3$) δ 7.78 (d, *J* = 8.3 Hz, 2H), 7.37 (dd, *J* = 8.1, 1.6 Hz, 2H), 7.34 – 7.25 (m, 14H), 7.22 – 7.14 (m, 6H), 5.17 (d, *J* = 8.7 Hz, 1H), 4.94 (d, *J* = 10.9 Hz, 1H), 4.85 (d, *J* = 5.7 Hz, 1H), 4.83 (d, *J* = 5.8 Hz, 1H), 4.81 (d, *J* = 10.9 Hz, 1H), 4.75 (d, *J* = 11.0 Hz, 1H), 4.55 (d, *J* = 10.9 Hz, 1H), 4.35 (d, *J* = 12.2 Hz, 1H), 4.29 (d, *J* = 12.2 Hz, 1H), 3.89 (t, *J* = 8.9 Hz, 1H), 3.76 (t, *J* = 8.8 Hz, 1H), 3.58 (dd, *J* = 11.0, 4.2 Hz, 1H), 3.53 – 3.44 (m, 2H), 3.38 (dd, *J* = 11.0, 1.7 Hz, 1H), 2.34 (s, 3H).

^{13}C NMR (151 MHz, $CDCl_3$) δ 144.3, 138.7, 138.3, 138.2, 133.5, 129.5, 129.2, 128.57, 128.55, 128.53, 128.51, 128.1, 128.0, 127.9, 127.8, 127.7, 127.5, 86.9, 85.9, 77.5, 77.1, 76.5, 75.9, 75.1, 74.4, 73.5, 68.9, 21.7.

HRMS (m/z): (ESI) calc. for $C_{41}H_{45}N_2O_7S$ $[M+H]^+$: 709.2947 found 709.2902.

2-deoxyglucose Hydrazide α-87

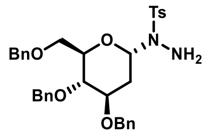

Following general procedures laid out in section **4.3.2 Synthesis of 2-deoxy-glucose C1 hydrazide**, purification through flash column chromatography afforded hydrazide **α-87**.

$[\alpha]_D^{20}$ = –10.6° (c = 0.5, $CHCl_3$)

^{1}H NMR (600 MHz, $CDCl_3$) δ 7.84 (d, *J* = 8.3 Hz, 2H), 7.37 – 7.25 (m, 13H), 7.21 – 7.17 (m, 2H), 7.10 (d, *J* = 8.1 Hz, 2H), 5.83 (dd, *J* = 8.5, 4.2 Hz, 1H), 4.60 (d, *J* = 12.0 Hz, 1H), 4.54 (d, *J* = 11.7 Hz, 1H), 4.50 (d, *J* = 12.0 Hz, 1H), 4.48 – 4.42 (m, 3H), 4.02 (q, *J* = 4.2 Hz, 1H), 3.93 (td, *J* = 6.0, 4.0 Hz, 1H), 3.69 (dd, *J* = 10.3, 6.4 Hz, 1H), 3.49 (dd, *J* = 10.4, 3.9 Hz, 1H), 3.43 (dd, *J* = 5.4, 4.0 Hz, 1H), 2.43 (ddd, *J* = 13.9, 8.5, 3.6 Hz, 1H), 2.32 (s, 3H), 1.96 (dt, *J* = 13.9, 4.7 Hz, 1H).

^{13}C NMR (151 MHz, $CDCl_3$) δ 144.1, 138.3, 138.2, 138.0, 133.2, 129.45, 129.39, 128.6, 128.54, 128.51, 128.0, 127.95, 127.93, 127.84, 127.79, 79.3, 75.2, 75.1, 74.1, 73.5, 72.4, 71.3, 69.1, 29.7, 21.7.

HRMS (m/z): (ESI) calc. for $C_{34}H_{39}N_2O_6S$ $[M+H]^+$: 603.2529 found 603.2479.

2-deoxyglucose Hydrazide β-87

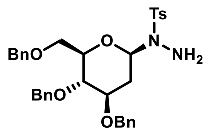

Following general procedures laid out in section **4.3.2 Synthesis of 2-deoxy-glucose C1 hydrazide**, purification through flash column chromatography afforded hydrazide **β-87**.

$[\alpha]_D^{25}$ = +24.0° (c = 0.25, $CHCl_3$)

^{1}H NMR (600 MHz, $CDCl_3$) δ 7.80 (d, *J* = 8.3 Hz, 2H), 7.37 – 7.26 (m, 11H), 7.24 – 7.20 (m, 4H), 7.15 (d, *J* = 7.9 Hz, 2H), 5.32 (dd, *J* = 11.0, 2.2 Hz, 1H), 4.90 (d, *J* = 11.0 Hz, 1H), 4.70 (d, *J* = 11.6 Hz, 1H), 4.64 (d, *J* = 11.6 Hz, 1H), 4.57 (d, *J* = 11.0 Hz, 1H), 4.36 (d, *J* = 12.2 Hz, 1H), 4.31 (d, *J* = 12.2 Hz, 1H), 3.77 (ddd, *J* = 11.5, 8.6, 5.0 Hz, 1H), 3.61 (dd, *J* = 10.9, 4.5 Hz, 1H), 3.46 (ddd, *J* = 9.7, 4.5, 1.8 Hz, 1H), 3.44 – 3.37 (m, 2H), 3.08 (s, 2H), 2.34 (s, 3H), 2.23 (ddd, *J* = 12.6, 5.1, 2.2 Hz, 1H), 2.06 (dt, *J* = 12.6, 11.3 Hz, 1H).

^{13}C NMR (151 MHz, $CDCl_3$) δ 144.2, 138.40, 138.37, 138.3, 133.3, 129.5, 129.3, 128.6, 128.5, 128.1, 127.90, 127.86, 127.7, 127.5, 83.7, 79.9, 77.6, 76.7, 75.1, 73.5, 71.8, 69.3, 34.0, 21.7.

HRMS (m/z): (ESI) calc. for $C_{34}H_{39}N_2O_6S$ $[M+H]^+$: 603.2529 found 603.2474.

Ribose hydrazide α-89

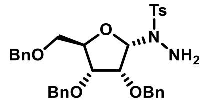

Following general procedures laid out in section **4.3.3 Synthesis of ribose C1 hydrazide**, purification through flash column chromatography afforded hydrazide **α-89**.

$[\alpha]_D^{20}$ = +56.0° (c = 0.1, $CHCl_3$)

^{1}H NMR (600 MHz, $CDCl_3$) δ 7.81 (d, *J* = 8.2 Hz, 2H), 7.40 – 7.29 (m, 9H), 7.26 – 7.22 (m, 6H), 7.17 (dd, *J* = 6.6, 2.9 Hz, 2H), 6.24 (d, *J* = 6.8 Hz, 1H), 4.79 (d, *J* = 11.2 Hz, 1H), 4.63 (d, *J* = 11.4 Hz, 1H), 4.59 (d, *J* = 11.2 Hz, 1H), 4.51 (d, *J* = 12.0 Hz, 1H), 4.48 (d, *J* = 11.4 Hz, 1H), 4.45 – 4.41 (m, 2H), 4.23 (q, *J* = 3.1 Hz, 1H), 3.98 (dd, *J* = 5.7, 2.2 Hz, 1H), 3.44 (dd, *J* = 10.4, 3.8 Hz, 1H), 3.39 (dd, *J* = 10.3, 3.1 Hz, 1H), 2.39 (s, 3H).

^{13}C NMR (151 MHz, $CDCl_3$) δ 143.5, 137.8, 137.6, 137.5, 135.3, 129.3, 128.69, 128.66, 128.6, 128.3, 128.1, 128.03, 127.99, 127.96, 127.7, 87.3, 82.4, 78.2, 78.0, 73.7, 73.5, 73.4, 70.8, 21.7.

HRMS (m/z): (ESI) calc. for $C_{33}H_{37}N_2O_6S$ $[M+H]^+$: 589.2372 found 589.2324.

Ribose hydrazide β-89

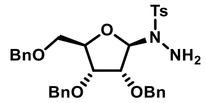

Following general procedures laid out in section **4.3.3 Synthesis of ribose C1 hydrazide**, purification through flash column chromatography afforded hydrazide **β-89**.

$[\alpha]_D^{20}$ = –9.8° (c = 0.5, $CHCl_3$)

^{1}H NMR (600 MHz, $CDCl_3$) δ 7.78 (d, *J* = 8.1 Hz, 2H), 7.41 – 7.24 (m, 15H), 7.04 – 7.00 (m, 2H), 5.95 (d, *J* = 2.2 Hz, 1H), 4.75 (d, *J* = 12.1 Hz, 1H), 4.64 (d, *J* = 6.7 Hz, 1H), 4.62 (d, *J* = 6.4 Hz, 1H), 4.45 (d, *J* = 11.8 Hz, 1H), 4.16 (q, *J* = 11.8 Hz, 2H), 4.11 – 4.05 (m, 3H), 3.43 (dd, *J* = 10.8, 3.1 Hz, 1H), 3.36 (dd, *J* = 10.8, 3.4 Hz, 1H), 2.70 (s, 2H), 2.40 (s, 3H).

^{13}C NMR (151 MHz, $CDCl_3$) δ 144.2, 138.0, 137.9, 137.6, 133.2, 129.4, 128.6, 128.5, 128.5, 128.3, 128.1, 128.0, 128.0, 127.8, 127.4, 91.5, 81.0, 78.4, 76.2, 73.3, 72.4, 72.0, 69.6, 21.7.

HRMS (m/z): (ESI) calc. for $C_{33}H_{37}N_2O_6S$ $[M+H]^+$: 589.2372 found 589.2307.

2-deoxyribose Hydrazide α-91

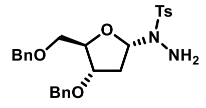

Following general procedures laid out in section **4.3.4 Synthesis of 2-deoxy-ribose C1 hydrazide**, purification through flash column chromatography afforded hydrazide **α-91**.

$[\boldsymbol{a}]_{\mathbf{D}}^{\mathbf{20}}$ = +56.8° (c = 0.25, $CHCl_3$)

^{1}H NMR (600 MHz, $CDCl_3$) δ 7.81 (d, *J* = 8.3 Hz, 2H), 7.37 (tt, *J* = 6.8, 1.0 Hz, 2H), 7.33 – 7.27 (m, 7H), 7.25 – 7.22 (m, 3H), 6.13 (dd, *J* = 7.8, 5.0 Hz, 1H), 4.52 – 4.42 (m, 4H), 4.19 (q, *J* = 4.1 Hz, 1H), 4.02 (dt, *J* = 7.2, 3.7 Hz, 1H), 3.45 – 3.37 (m, 2H), 2.56 (dt, *J* = 14.4, 7.4 Hz, 1H), 2.39 (s, 3H), 2.18 (dt, *J* = 14.1, 4.5 Hz, 1H).

^{13}C NMR (151 MHz, $CDCl_3$) δ 144.0, 138.1, 137.6, 133.9, 129.4, 129.2, 128.7, 128.0, 127.9, 127.8, 127.7, 88.8, 83.8, 79.0, 73.6, 71.9, 70.9, 36.3, 21.7.

HRMS (m/z): (ESI) calc. for $C_{26}H_{31}N_2O_5S$ $[M+H]^+$: 483.1954 found 483.1944.

2-deoxyribose Hydrazide β-91

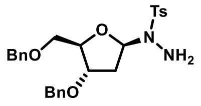

Following general procedures laid out in section **4.3.4 Synthesis of 2-deoxy-ribose C1 hydrazide**, purification through flash column chromatography afforded hydrazide **β-91**.

$[\alpha]_D^{20}$ = –40° (c = 0.1, $CHCl_3$)

^{1}H NMR (600 MHz, $CDCl_3$) δ 7.81 (d, *J* = 8.3 Hz, 2H), 7.36 – 7.24 (m, 10H), 7.10 (dd, *J* = 7.4, 2.1 Hz, 2H), 6.13 (t, *J* = 6.6 Hz, 1H), 4.53 (d, *J* = 11.8 Hz, 1H), 4.48 (d, *J* = 11.7 Hz, 1H), 4.26 (s, 2H), 4.14 (dt, *J* = 6.5, 3.8 Hz, 1H), 4.00 (q, *J* = 4.0 Hz, 1H), 3.40 (dd, *J* = 10.4, 4.3 Hz, 1H), 3.36 (dd, *J* = 10.4, 4.1 Hz, 1H), 2.40 (s, 3H), 2.35 (dt, *J* = 13.1, 6.4 Hz, 1H), 2.21 (ddd, *J* = 13.6, 6.8, 4.0 Hz, 1H).

^{13}C NMR (151 MHz, $CDCl_3$) δ 144.0, 137.9, 133.5, 129.37, 129.36, 128.6, 128.5, 128.0, 127.89, 127.87, 127.6, 88.8, 83.0, 78.7, 73.3, 71.6, 70.5, 35.2, 21.8.

HRMS (m/z): (ESI) calc. for $C_{26}H_{31}N_2O_5S$ $[M+H]^+$: 483.1954 found 483.1930.

6.3.1.3. Stereorentive coupling products

Compound 85

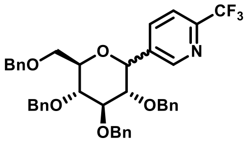

Following **General Procedure E** with Bn-protected glucose hydrazide **β-83** (0.1 mmol) and 2-trifluoromethyl-5-iodo-pyridine **84**, purification through flash column chromatography afforded product **85** (46 mg, 68% yield, β:α = 5:1).

Following **General Procedure E** with Bn-protected glucose hydrazide **α-83** (0.1 mmol) and 2-trifluoromethyl-5-iodo-pyridine **84**, purification through flash column chromatography afforded product **85** (47 mg, 70% yield, α:β = 3:1).

Compound α-85:

$[\alpha]_D^{25}$ = +0.5° (c = 0.2, $CHCl_3$)

^{1}H NMR (600 MHz, $CDCl_3$) δ 9.03 (d, J = 2.0 Hz, 1H), 8.20 (dd, J = 8.3, 2.1 Hz, 1H), 7.63 (d, J = 8.2 Hz, 1H), 7.38 – 7.25 (m, 16H), 7.19 – 7.16 (m, 2H), 7.13 (dd, J = 7.3, 2.3 Hz, 2H), 5.20 (d, J = 4.9 Hz, 1H), 4.88 (d, J = 11.2 Hz, 1H), 4.79 (d, J = 4.5 Hz, 1H), 4.77 – 4.73 (m, 2H), 4.62 (d, J = 12.2 Hz, 1H), 4.58 (d, J = 11.7 Hz, 1H), 4.50 (dd, J = 11.6, 2.8 Hz, 2H), 3.99 (dd, J = 8.3, 5.0 Hz, 1H), 3.92 (t, J = 7.9 Hz, 1H), 3.77 (dd, J = 9.3, 7.4 Hz, 1H), 3.69 (dd, J = 10.6, 4.2 Hz, 1H), 3.65 (dd, J = 10.5, 2.6 Hz, 1H), 3.54 (ddd, J = 9.3, 4.2, 2.6 Hz, 1H).

^{13}C NMR (151 MHz, $CDCl_3$) δ 150.0, 147.2 (q, $^2J_{C,F}$ = 34.6 Hz), 138.2, 138.0, 137.9, 137.36, 137.35, 137.4, 128.28, 128.27, 128.5, 128.2, 128.1, 128.00, 127.99, 127.98, 127.9, 121.7 (q, $^1J_{C,F}$ = 273.9 Hz), 120.1, 81.1, 80.0, 77.5, 75.0, 74.7, 73.8, 73.6, 73.3, 72.0, 68.7.

HRMS (m/z): (ESI) calc. for $C_{40}H_{39}F_3NO_5$ $[M+H]^+$: 670.2780 found 670.2783

Compound β-85:

$[\alpha]_D^{25}$ = +25.9° (c = 1.0, $CHCl_3$)

^{1}H NMR (600 MHz, $CDCl_3$) δ 8.73 (d, *J* = 2.0 Hz, 1H), 7.81 (dd, *J* = 8.0, 2.1 Hz, 1H), 7.58 – 7.53 (m, 1H), 7.38 – 7.26 (m, 13H), 7.23 – 7.15 (m, 5H), 6.91 – 6.87 (m, 2H), 5.01 – 4.93 (m, 2H), 4.88 (d, *J* = 10.7 Hz, 1H), 4.67 – 4.58 (m, 3H), 4.56 (d, *J* = 12.2 Hz, 1H), 4.33 (d, *J* = 9.5 Hz, 1H), 4.03 (d, *J* = 10.9 Hz, 1H), 3.86 (t, *J* = 9.0 Hz, 1H), 3.82 – 3.73 (m, 3H), 3.63 (ddd, *J* = 9.7, 4.3, 2.0 Hz, 1H), 3.44 (dd, *J* = 9.5, 8.9 Hz, 1H).

^{13}C NMR (151 MHz, $CDCl_3$) δ 149.4, 148.1, 147.9, 147.6, 147.4, 138.4, 138.1, 138.0, 138.0, 136.9, 136.1, 128.7, 128.6, 128.6, 128.5, 128.5, 128.1, 128.1, 128.1, 127.9, 127.8, 127.8, 124.4, 122.6, 120.8, 120.1, 118.9, 87.1, 82.7, 79.6, 78.7, 78.3, 75.9, 75.4, 75.2, 73.6, 69.0.

HRMS (m/z): (ESI) calc. for $C_{40}H_{39}F_3NO_5$ $[M+H]^+$: 670.2780 found 670.2783

Compound 88

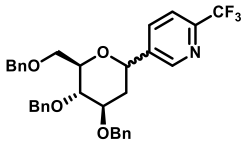

Following **General Procedure E** with Bn-protected 2-deoxyglucose hydrazide **β-87** (0.1 mmol) and 2-trifluoromethyl-5-iodo-pyridine **84**, purification through flash column chromatography afforded product **88** (30 mg, 53% yield, β:α = 2:1).

Following **General Procedure E** with Bn-protected 2-deoxyglucose hydrazide **α-87** (0.1 mmol) and 2-trifluoromethyl-5-iodo-pyridine **84**, purification through flash column chromatography afforded product **88** (30.4 mg, 54% yield, α:β = 3:1).

Characterized as a 1:1.4 mixture

^{1}H NMR (600 MHz, $CDCl_3$) δ 8.7 (d, J = 2.0 Hz, 1H), 7.9 (dd, J = 8.1, 2.1 Hz, 1H), 7.8 (dd, J = 8.2, 2.1 Hz, 1H), 7.7 (d, J = 0.8 Hz, 1H), 7.6 (d, J = 0.8 Hz, 1H), 7.4 – 7.3 (m, 31H), 7.3 – 7.2 (m, 3H), 7.2 – 7.2 (m, 2H), 5.1 (t, J = 5.2 Hz, 1H), 5.0 (d, J = 10.8 Hz, 1H), 4.7 (d, J = 1.6 Hz, 1H), 4.7 – 4.7 (m, 3H), 4.7 – 4.6 (m, 5H), 4.6 – 4.5 (m, 4H), 3.9 – 3.8 (m, 5H), 3.8 (td, J = 6.0, 3.4 Hz, 1H), 3.7 (dd, J = 10.1, 3.4 Hz, 1H), 3.7 – 3.6 (m, 5H), 2.4 – 2.4 (m, 2H), 2.1 (ddd, J = 14.0, 8.1, 4.5 Hz, 1H), 1.7 (dt, J = 13.0, 11.6 Hz, 3H).

^{13}C NMR (151 MHz, $CDCl_3$) δ 148.4, 148.0, 147.9 – 146.9 (m), 140.3, 139.7, 138.4, 138.3, 138.3, 138.2, 138.1, 135.6, 135.0, 128.7, 128.6, 128.6, 128.5, 128.2, 128.1, 128.0, 128.0, 127.9, 127.9, 127.8, 121.6 (q, J = 274.0 Hz), 120.4 (d, J = 3.2 Hz), 80.8, 79.6, 78.1, 75.7, 75.5, 75.4, 74.8, 74.2, 73.7, 73.6, 73.5, 72.1, 71.9, 69.5, 69.0, 68.4, 39.1, 32.7.

HRMS (m/z): (ESI) calc. $C_{33}H_{32}F_3NO_4$ $[M+H]^+$: 564.2362 found 564.2373

Compound 90

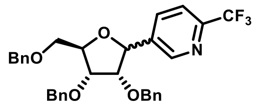

Following **General Procedure E** with Bn-protected ribose hydrazide **β-89** (0.1 mmol) and 2-trifluoromethyl-5-iodo-pyridine **84**, purification through flash column chromatography afforded product **90** (41 mg, 75% yield, β:α > 19:1).

Following **General Procedure E** with Bn-protected ribose hydrazide **α-89** (0.1 mmol) and 2-trifluoromethyl-5-iodo-pyridine **84**, purification through flash column chromatography afforded product **90** (32 mg, 59% yield, α:β = 1:1).

Compound α-90:

$[\alpha]_D^{25}$ = –39.0° (c = 0.5, $CHCl_3$)

^{1}H NMR (600 MHz, $CDCl_3$) δ 8.59 (d, *J* = 2.0 Hz, 1H), 7.92 (dd, *J* = 8.1, 2.1 Hz, 1H), 7.61 (d, *J* = 0.8 Hz, 1H), 7.36 – 7.25 (m, 10H), 7.23 – 7.16 (m, 3H), 6.89 – 6.87 (m, 2H), 5.16 (d, *J* = 3.8 Hz, 1H), 4.59 (d, *J* = 12.3 Hz, 2H), 4.51 (d, *J* = 12.1 Hz, 1H), 4.48 (d, *J* = 11.8 Hz, 1H), 4.44 (dt, *J* = 7.7, 3.2 Hz, 1H), 4.41 (d, *J* = 11.9 Hz, 1H), 4.31 (dd, *J* = 7.6, 4.1 Hz, 1H), 4.19 – 4.14 (m, 2H), 3.77 (dd, *J* = 10.8, 2.8 Hz, 1H), 3.61 (dd, *J* = 10.8, 3.5 Hz, 1H).

^{13}C NMR (151 MHz, $CDCl_3$) δ 148.9, 147.4 (q, $^2J_{C,F}$ = 34.8 Hz), 138.2, 137.7, 137.4, 137.1, 128.6, 128.5, 128.4, 128.2, 128.0, 127.98, 127.94, 127.92, 121.8 (q, $^1J_{C,F}$ = 273.7 Hz), 119.8, 80.7, 80.06, 80.05, 79.0, 73.7, 73.6, 73.0, 69.8.

HRMS (m/z): (ESI) calc. for $C_{32}H_{31}F_3NO_4$ $[M+H]^+$: 550.2205 found 550.2219

Compound β-90:

$[\alpha]_D^{25}$ = +11.8° (c = 0.5, $CHCl_3$)

^{1}H NMR (600 MHz, $CDCl_3$) δ 8.72 (d, *J* = 2.0 Hz, 1H), 7.85 (dd, *J* = 8.1, 2.1 Hz, 1H), 7.50 (dd, *J* = 8.1, 0.8 Hz, 1H), 7.37 – 7.30 (m, 8H), 7.27 – 7.21 (m, 4H), 7.15 – 7.12 (m, 2H), 5.06 (d, *J* = 7.7 Hz, 1H), 4.62 (s, 2H), 4.56 (dd, *J* = 11.9, 6.9 Hz, 2H), 4.52 (d,

J = 11.9 Hz, 1H), 4.39 – 4.35 (m, 2H), 4.03 (dd, J = 5.0, 2.7 Hz, 1H), 3.78 (dd, J = 7.7, 5.0 Hz, 1H), 3.65 (dd, J = 10.3, 3.9 Hz, 1H), 3.58 (dd, J = 10.3, 3.3 Hz, 1H).

^{13}C NMR (151 MHz, $CDCl_3$) δ 148.3, 147.5 (q, $^{2}J_{C,F}$ = 34.6 Hz), 139.5, 137.9, 137.7, 137.2, 135.1, 128.64, 128.62, 128.5, 128.3, 128.2, 128.17, 128.14, 128.0, 127.8, 121.7 (q, $^{1}J_{C,F}$ = 273.7 Hz), 120.1, 83.8, 82.8, 79.5, 77.3, 73.7, 72.7, 72.1, 70.5.

HRMS (m/z): (ESI) calc. for $C_{32}H_{31}F_3NNaO_4$ $[M+Na]^+$: 572.2025 found 572.2013

Compound 92

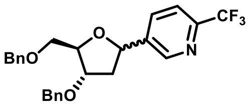

Following **General Procedure E** with Bn-protected 2-deoxyribose hydrazide **β-91** (0.1 mmol) and 2-trifluoromethyl-5-iodo-pyridine **84**, purification through flash column chromatography afforded product **92** (15 mg, 33% yield, β:α = 3:1).

Following **General Procedure E** with Bn-protected 2-deoxyribose hydrazide **α-91** (0.1 mmol) and 2-trifluoromethyl-5-iodo-pyridine **84**, purification through flash column chromatography afforded product **92** (24 mg, 55% yield, α:β = 4:1).

Compound α-92:

$[\alpha]_D^{25}$ = +29.4° (c = 0.5, $CHCl_3$)

^{1}H NMR (600 MHz, $CDCl_3$) δ 8.68 (d, *J* = 2.1 Hz, 1H), 7.95 (ddd, *J* = 8.1, 1.5, 0.7 Hz, 1H), 7.63 (dd, *J* = 8.2, 0.8 Hz, 1H), 7.38 – 7.25 (m, 8H), 7.16 – 7.14 (m, 2H), 5.26 (t, *J* = 7.0 Hz, 1H), 4.62 – 4.55 (m, 2H), 4.48 – 4.39 (m, 3H), 4.26 (ddd, *J* = 6.4, 4.4, 3.3 Hz, 1H), 3.64 – 3.58 (m, 2H), 2.69 (ddd, *J* = 13.1, 7.6, 6.4 Hz, 1H), 2.08 (ddd, *J* = 13.1, 6.5, 4.4 Hz, 1H).

^{13}C NMR (151 MHz, $CDCl_3$) δ 148.0, 147.2 (q, $^2J_{C,F}$ = 34.5 Hz), 142.3, 138.1, 137.7, 135.0, 128.60, 128.57, 127.95, 127.92, 127.8, 127.7, 121.8 (q, $^1J_{C,F}$ = 273.7 Hz), 120.3, 83.8, 80.9, 77.9, 73.7, 71.7, 70.9, 40.6.

HRMS (m/z): (ESI) calc. for $C_{25}H_{25}F_3NO_3$ $[M+H]^+$: 444.1787 found 444.1791

Compound β-92:

$[\alpha]_D^{25}$ = +17.0° (c = 0.2, $CHCl_3$)

^{1}H NMR (600 MHz, $CDCl_3$) δ 8.70 (d, *J* = 2.1 Hz, 1H), 7.89 (dd, *J* = 8.1, 2.1 Hz, 1H), 7.61 (d, *J* = 8.1 Hz, 1H), 7.38 – 7.28 (m, 10H), 5.24 (dd, *J* = 10.7, 5.3 Hz, 1H), 4.61 – 4.55 (m, 4H), 4.35 (td, *J* = 4.5, 2.0 Hz, 1H), 4.21 (d, *J* = 5.8 Hz, 1H), 3.65 (dd, *J* = 10.1, 4.2 Hz, 1H), 3.60 (dd, *J* = 10.2, 4.8 Hz, 1H), 2.45 (ddd, *J* = 13.2, 5.3, 1.2 Hz, 1H), 1.91

(ddd, J = 13.1, 10.7, 5.7 Hz, 1H).

^{13}C NMR (151 MHz, $CDCl_3$) δ 148.2, 147.5 (q, $^{2}J_{C,F}$ = 34.6 Hz), 141.0, 138.0, 137.9, 135.1, 128.7, 128.6, 128.0, 127.94, 127.87, 127.8, 121.7 (q, $^{1}J_{C,F}$ = 273.7 Hz), 120.3, 84.4, 81.5, 77.9, 73.7, 71.3, 71.0, 41.5.

HRMS (m/z): (ESI) calc. for $C_{25}H_{25}F_3NO_3$ $[M+H]^+$: 444.1787 found 444.1794

6.3.2 Characterization of C2-C6-Hydrazide

6.3.2.1 Synthetic precursors

Compound SF6

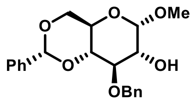

Following general procedures laid out in section **4.5.1 Synthesis of glucose C2 hydrazide**, purification through flash column chromatography afforded compound **SF6**.

$[\alpha]_D^{20}$ = +85.8° (c = 0.5, $CHCl_3$)

^{1}H NMR (600 MHz, $CDCl_3$) δ 7.54 – 7.50 (m, 2H), 7.43 – 7.37 (m, 5H), 7.36 – 7.32 (m, 2H), 7.31 – 7.28 (m, 1H), 5.59 (s, 1H), 4.98 (d, *J* = 11.6 Hz, 1H), 4.83 – 4.79 (m, 2H), 4.32 (dd, *J* = 10.1, 4.7 Hz, 1H), 3.88 – 3.83 (m, 2H), 3.80 – 3.71 (m, 2H), 3.66 (t, *J* = 9.3 Hz, 1H), 3.45 (s, 3H), 2.49 (d, *J* = 7.4 Hz, 1H).

^{13}C NMR (151 MHz, $CDCl_3$) δ 138.5, 137.4, 129.0, 128.4, 128.3, 128.1, 127.8, 126.1, 101.3, 99.9, 82.0, 78.9, 74.8, 72.4, 69.0, 62.6, 55.4.

HRMS (m/z): (ESI) calc. for $C_{21}H_{25}O_6$ $[M+H]^+$: 373.1651 found 373.1651

*Note: The compound **SF6** NMR spectra is in agreement with literature reported data*[18].

Compound SF7

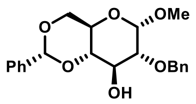

Following general procedures laid out in section **4.5.2 Synthesis of glucose C3 hydrazide**, purification through flash column chromatography afforded compound **SF7**.

$[\alpha]_D^{20}$ = +36.4° (c = 0.5, $CHCl_3$)

^{1}H NMR (600 MHz, $CDCl_3$) δ 7.49 (dd, *J* = 7.5, 2.1 Hz, 2H), 7.40 – 7.30 (m, 8H), 5.52 (s, 1H), 4.79 (d, *J* = 12.2 Hz, 1H), 4.69 (s, 1H), 4.61 (d, *J* = 3.6 Hz, 1H), 4.26 (dd, *J* = 10.2, 4.8 Hz, 1H), 4.15 (t, *J* = 9.3 Hz, 1H), 3.82 (td, *J* = 10.0, 4.9 Hz, 1H), 3.70 (t, *J* = 10.3 Hz, 1H), 3.54 – 3.44 (m, 2H), 3.38 (s, 3H), 2.65 (s, 1H).

^{13}C NMR (151 MHz, $CDCl_3$) δ 138.0, 137.2, 129.3, 128.7, 128.4, 128.3, 126.4, 102.1, 98.7, 81.3, 79.7, 73.5, 70.4, 69.1, 62.1, 55.5.

HRMS (m/z): (ESI) calc. for $C_{21}H_{25}O_6$ $[M+H]^+$: 373.1651 found 373.1653

*Note: The compound **SF7** NMR spectra is in agreement with literature reported data*[18].

Compound SF22

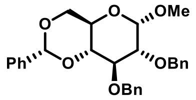

Following general procedures laid out in section **4.5.3 Synthesis of glucose C4 hydrazide**, purification through flash column chromatography afforded compound **SF22**.

$[\alpha]_D^{20}$ = –31.0° (c = 0.5, $CHCl_3$)

^{1}H NMR (600 MHz, $CDCl_3$) δ 7.50 (dd, *J* = 7.8, 1.8 Hz, 2H), 7.42 – 7.27 (m, 13H), 5.57 (s, 1H), 4.93 (d, *J* = 11.3 Hz, 1H), 4.89 – 4.83 (m, 2H), 4.71 (d, *J* = 12.2 Hz, 1H), 4.61 (d, *J* = 3.7 Hz, 1H), 4.28 (dd, *J* = 10.2, 4.8 Hz, 1H), 4.06 (t, *J* = 9.3 Hz, 1H), 3.84 (td, *J* = 10.0, 4.8 Hz, 1H), 3.72 (t, *J* = 10.3 Hz, 1H), 3.62 (t, *J* = 9.4 Hz, 1H), 3.57 (dd, *J* = 9.3, 3.7 Hz, 1H), 3.42 (s, 3H).

^{13}C NMR (151 MHz, $CDCl_3$) δ 138.8, 138.2, 137.5, 129.0, 128.6, 128.4, 128.3, 128.25, 128.16, 128.0, 127.7, 126.1, 101.4, 99.3, 82.2, 79.2, 78.7, 75.5, 73.9, 69.2, 62.4, 55.5.

HRMS (m/z): (ESI) calc. for $C_{28}H_{31}O_6$ $[M+H]^+$: 463.2121 found 463.2116.

*Note: The compound **SF22** NMR spectra is in agreement with literature reported data.* [19]

Compound SF8

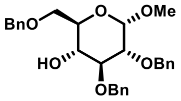

Following general procedures laid out in section **4.5.3 Synthesis of glucose C4 hydrazide**, purification through flash column chromatography afforded compound **SF8**.

$[\alpha]_D^{20}$ = +13.2° (c = 0.5, $CHCl_3$)

^{1}H NMR (600 MHz, $CDCl_3$) δ 7.39 – 7.27 (m, 15H), 5.01 (d, *J* = 11.4 Hz, 1H), 4.77 (d, *J* = 12.1 Hz, 1H), 4.74 (d, *J* = 11.4 Hz, 1H), 4.66 (d, *J* = 12.0 Hz, 1H), 4.64 (d, *J* = 3.6 Hz, 1H), 4.59 (d, *J* = 12.1 Hz, 1H), 4.54 (d, *J* = 12.2 Hz, 1H), 3.79 (t, *J* = 9.2 Hz, 1H), 3.73 – 3.66 (m, 3H), 3.61 (t, *J* = 9.2 Hz, 1H), 3.54 (dd, *J* = 9.6, 3.5 Hz, 1H), 3.39 (s, 3H).

^{13}C NMR (151 MHz, $CDCl_3$) δ 138.9, 138.2, 138.1, 128.7, 128.6, 128.5, 128.3, 128.14, 128.09, 128.0, 127.99, 127.77, 98.3, 81.6, 79.7, 75.6, 73.7, 73.3, 70.8, 70.0, 69.6, 55.4.

HRMS (m/z): (ESI) calc. for $C_{28}H_{32}NaO_6$ $[M+Na]^+$: 487.2097 found 487.2097.

*Note: The compound **SF8** NMR spectra is in agreement with literature reported data.* [20]

Compound SF10

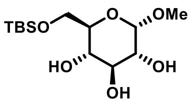

Following general procedures laid out in section **4.5.4 Synthesis of glucose C6 hydrazide**, purification through flash column chromatography afforded compound **SF10**.

$[\alpha]_D^{20}$ = +99.0° (c = 0.5, $CHCl_3$)

^{1}H NMR (600 MHz, $CDCl_3$) δ 5.00 (d, *J* = 3.9 Hz, 1H), 4.71 (d, *J* = 3.7 Hz, 1H), 4.26 (d, *J* = 3.9 Hz, 1H), 4.08 (d, *J* = 8.2 Hz, 1H), 3.87 (dd, *J* = 11.1, 3.2 Hz, 1H), 3.82 – 3.72 (m, 2H), 3.55 (ddd, *J* = 9.3, 5.5, 3.2 Hz, 1H), 3.51 – 3.47 (m, 1H), 3.43 – 3.35 (m, 4H), 0.89 (s, 9H), 0.07 (s, 6H).

^{13}C NMR (151 MHz, $CDCl_3$) δ 99.4, 74.4, 72.1, 71.6, 71.3, 63.6, 55.1, 26.0, 18.5, -5.2.

HRMS (m/z): (ESI) calc. for $C_{13}H_{28}NaO_6Si$ $[M+Na]^+$: 331.1553 found 331.1558.

*Note: The compound **SF10** NMR spectra is in agreement with literature reported data.* [21]

Compound SF11

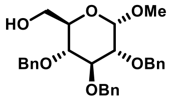

Following general procedures laid out in section **4.5.4 Synthesis of glucose C6 hydrazide**, purification through flash column chromatography afforded compound **SF11**.

$[\alpha]_D^{20}$ = +23.0° (c = 0.5, $CHCl_3$)

^{1}H NMR (600 MHz, $CDCl_3$) δ 7.39 – 7.28 (m, 15H), 5.00 (d, J = 10.9 Hz, 1H), 4.90 (d, J = 11.0 Hz, 1H), 4.85 (d, J = 10.9 Hz, 1H), 4.81 (d, J = 12.1 Hz, 1H), 4.66 (dd, J = 13.4, 11.5 Hz, 2H), 4.58 (d, J = 3.5 Hz, 1H), 4.02 (t, J = 9.3 Hz, 1H), 3.78 (dt, J = 12.1, 3.1 Hz, 1H), 3.72 – 3.68 (m, 1H), 3.66 (ddd, J = 10.0, 4.0, 2.7 Hz, 1H), 3.56 – 3.49 (m, 2H), 3.37 (s, 3H), 1.69 (t, J = 6.3 Hz, 1H).

^{13}C NMR (151 MHz, $CDCl_3$) δ 138.8, 138.2, 128.61, 128.60, 128.5, 128.3, 128.2, 128.17, 128.10, 128.0, 127.8, 98.3, 82.1, 80.0, 77.5, 75.9, 75.2, 73.6, 70.8, 62.0, 55.3.

HRMS (m/z): (ESI) calc. for $C_{28}H_{32}NaO_6$ $[M+Na]^+$: 487.2097 found 487.2101.

*Note: The compound **SF11** NMR spectra is in agreement with literature reported data.* [22]

Compound SF23

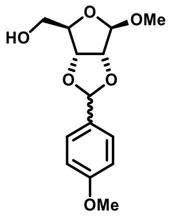

Following general procedures laid out in section **4.5.5 Synthesis of ribose C2 and C3 hydrazide**, purification through flash column chromatography afforded compound **SF23** (d.r. = 1:1).

$[\alpha]_D^{20}$ = –45.8° (c = 0.5, $CHCl_3$)

^{1}H NMR (600 MHz, $CDCl_3$) δ 7.44 – 7.40 (m, 2H), 7.40 – 7.36 (m, 2H), 6.92 – 6.88 (m, 4H), 5.92 (s, 1H), 5.72 (s, 1H), 5.11 (d, J = 18.5 Hz, 2H), 4.99 – 4.97 (m, 1H), 4.89 (d, J = 6.1 Hz, 1H), 4.71 (d, J = 5.6 Hz, 1H), 4.67 (d, J = 6.2 Hz, 1H), 4.60 (dd, J = 3.4, 2.3 Hz, 1H), 4.52 (td, J = 2.9, 0.9 Hz, 1H), 3.81 (s, 6H), 3.75 – 3.63 (m, 4H), 3.46 (s, 6H), 3.34 (dd, J = 10.7, 2.6 Hz, 1H), 3.17 (dd, J = 10.1, 3.1 Hz, 1H).

^{13}C NMR (151 MHz, $CDCl_3$) δ 160.9, 160.8, 128.4, 128.3, 128.2, 128.0, 113.9, 109.7, 109.3, 105.8, 104.2, 88.2, 86.2, 85.1, 82.4, 81.0, 64.2, 64.1, 55.71, 55.69, 55.4.

HRMS (m/z): (ESI) calc. for $C_{14}H_{19}O_6$ $[M+H]^+$: 283.1182 found 283.1178.

*Note: The compound **SF23** NMR spectra is in agreement with literature reported data.* [9]

Compound SF24

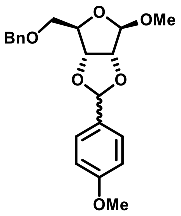

Following general procedures laid out in section **4.5.5 Synthesis of ribose C2 and C3 hydrazide**, purification through flash column chromatography afforded compound **SF24** (d.r. = 1:1).

$[\alpha]_D^{20}$ = –27.4° (c = 0.5, $CHCl_3$)

^{1}H NMR (600 MHz, $CDCl_3$) δ 7.45 – 7.42 (m, 2H), 7.41 – 7.38 (m, 2H), 7.36 (dd, *J* = 4.4, 1.7 Hz, 8H), 7.30 (dddd, *J* = 8.8, 5.3, 4.1, 1.4 Hz, 2H), 6.93 – 6.89 (m, 4H), 5.94 (s, 1H), 5.75 (s, 1H), 5.12 (s, 1H), 5.08 (s, 1H), 4.86 (dd, *J* = 5.6, 1.3 Hz, 1H), 4.76 (dd, *J* = 6.2, 0.9 Hz, 1H), 4.66 (dd, *J* = 7.2, 5.9 Hz, 2H), 4.61 – 4.54 (m, 5H), 4.50 (ddd, *J* = 7.9, 6.5, 1.3 Hz, 1H), 3.81 (s, 6H), 3.58 (ddd, *J* = 9.6, 6.6, 0.9 Hz, 2H), 3.52 (ddd, *J* = 9.7, 8.0, 2.0 Hz, 2H), 3.33 (s, 3H), 3.33 (s, 3H).

^{13}C NMR (151 MHz, $CDCl_3$) δ 160.9, 160.7, 138.1, 138.0, 128.53, 128.49, 128.4, 128.2, 127.9, 127.87, 127.84, 127.82, 113.92, 113.89, 109.1, 108.9, 106.1, 104.2, 85.8, 85.1, 84.6, 84.3, 82.8, 82.0, 73.4, 73.3, 71.2, 71.1, 55.4, 54.95, 54.93.

HRMS (m/z): (ESI) calc. for $C_{21}H_{25}O_6$ $[M+H]^+$: 373.1651 found 373.1646.

*Note: The compound **SF24** NMR spectra is in agreement with literature reported data.* [9]

Compound SF13

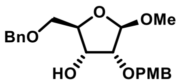

Following general procedures laid out in section **4.5.5 Synthesis of ribose C2 and C3 hydrazide**, purification through flash column chromatography afforded compound **SF13**.

$[\alpha]_D^{20}$ = +13.4° (c = 0.5, $CHCl_3$)

^{1}H NMR (600 MHz, $CDCl_3$) δ 7.37 – 7.31 (m, 4H), 7.29 – 7.25 (m, 3H), 6.91 – 6.87 (m, 2H), 4.89 (d, *J* = 1.2 Hz, 1H), 4.67 (d, *J* = 11.3 Hz, 1H), 4.60 (s, 2H), 4.55 (d, *J* = 11.3 Hz, 1H), 4.14 (ddd, *J* = 8.7, 6.3, 5.1 Hz, 1H), 4.09 (td, *J* = 6.3, 3.8 Hz, 1H), 3.84 (dd, *J* = 5.1, 1.2 Hz, 1H), 3.81 (s, 3H), 3.65 (dd, *J* = 10.4, 3.8 Hz, 1H), 3.54 (dd, *J* = 10.4, 6.4 Hz, 1H), 3.34 (s, 3H).

^{13}C NMR (151 MHz, $CDCl_3$) δ 159.7, 138.3, 129.8, 129.3, 128.5, 127.8, 127.7, 114.1, 105.9, 83.3, 81.7, 73.4, 72.7, 71.8, 71.7, 55.4, 55.3.

HRMS (m/z): (ESI) calc. for $C_{21}H_{26}NaO_6$ $[M+Na]^+$: 397.1627 found 397.1624.

*Note: The compound **SF13** NMR spectra is in agreement with literature reported data.* [9]

Compound SF14

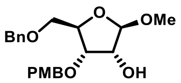

Following general procedures laid out in section **4.5.5 Synthesis of ribose C2 and C3 hydrazide**, purification through flash column chromatography afforded compound **SF14**.

$[\alpha]_D^{20}$ = –27.4° (c = 0.5, $CHCl_3$)

^{1}H NMR (600 MHz, $CDCl_3$) δ 7.37 – 7.33 (m, 4H), 7.32 – 7.27 (m, 1H), 7.24 – 7.20 (m, 2H), 6.88 – 6.84 (m, 2H), 4.86 (s, 1H), 4.57 (d, *J* = 2.6 Hz, 2H), 4.50 (s, 2H), 4.21 (td, *J* = 6.0, 4.9 Hz, 1H), 4.05 (dd, *J* = 6.3, 4.8 Hz, 1H), 3.99 (dd, *J* = 4.9, 2.5 Hz, 1H), 3.80 (s, 3H), 3.57 – 3.51 (m, 2H), 2.72 (d, *J* = 3.1 Hz, 1H).

^{13}C NMR (151 MHz, $CDCl_3$) δ 159.7, 138.3, 129.8, 129.3, 128.5, 127.8, 127.7, 114.1, 108.6, 80.7, 79.3, 73.4, 72.6, 71.7, 55.4, 55.2.

HRMS (m/z): (ESI) calc. for $C_{21}H_{26}NaO_6$ $[M+Na]^+$: 397.1627 found 397.1631.

*Note: The compound **SF14** NMR spectra is in agreement with literature reported data.* [9]

Compound SF19

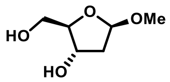

Following general procedures laid out in section **4.5.7 Synthesis of 2-deoxy-ribose C3 hydrazide**, purification through flash column chromatography afforded compound **SF19**.

^{1}H NMR (600 MHz, $CDCl_3$) δ 5.14 (d, *J* = 5.7 Hz, 1H), 4.57 – 4.52 (m, 1H), 4.09 (q, *J* = 3.6 Hz, 1H), 3.74 (d, *J* = 11.9 Hz, 1H), 3.69 – 3.61 (m, 1H), 3.40 (s, 3H), 2.58 (d, *J* = 6.6 Hz, 1H), 2.29 (dd, *J* = 14.0, 6.9 Hz, 1H), 2.14 (dt, *J* = 13.9, 5.3 Hz, 1H), 1.84 (d, *J* = 4.1 Hz, 1H).

^{13}C NMR (151 MHz, $CDCl_3$) δ 105.7, 87.8, 72.5, 63.7, 55.6, 42.9.

Note: The compound ***SF19*** *NMR spectra is in agreement with literature reported data.* [23]

Compound SF20

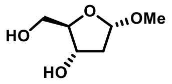

Following general procedures laid out in section **4.5.8 Synthesis of 2-deoxy-ribose C5 hydrazide**, purification through flash column chromatography afforded compound **SF20**.

^{1}H NMR (600 MHz, $CDCl_3$) δ 5.11 (d, *J* = 4.6 Hz, 1H), 4.18 – 4.12 (m, 2H), 3.73 (dt, *J* = 12.2, 4.4 Hz, 1H), 3.61 (dt, *J* = 11.7, 5.7 Hz, 1H), 3.39 (s, 3H), 2.76 (d, *J* = 10.7 Hz, 1H), 2.16 – 2.07 (m, 1H), 2.02 (d, *J* = 13.7 Hz, 1H), 1.79 (t, *J* = 6.2 Hz, 1H).

^{13}C NMR (151 MHz, $CDCl_3$) δ 105.7, 87.6, 73.0, 63.3, 55.0, 41.8.

*Note: The compound **SF20** NMR spectra is in agreement with literature reported data.* [23]

Compound SF21

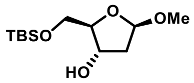

Following general procedures laid out in section **4.5.7 Synthesis of 2-deoxy-ribose C3 hydrazide**, purification through flash column chromatography afforded compound **SF21**.

^{1}H NMR (600 MHz, $CDCl_3$) δ 5.05 (dd, J = 5.4, 2.0 Hz, 1H), 4.41 (q, J = 5.9 Hz, 1H), 3.86 (dt, J = 8.1, 4.8 Hz, 1H), 3.77 (dd, J = 9.9, 5.3 Hz, 1H), 3.53 (dd, J = 9.9, 8.1 Hz, 1H), 3.31 (s, 3H), 2.21 (ddd, J = 13.3, 6.8, 2.0 Hz, 1H), 2.11 (s, 1H), 2.06 (ddd, J = 13.4, 6.6, 5.4 Hz, 1H), 0.89 (s, 9H), 0.07 (s, 6H).

^{13}C NMR (151 MHz, $CDCl_3$) δ 105.1, 85.8, 73.7, 65.1, 55.2, 41.0, 26.0, 18.4, -5.26, -5.31..

*Note: The compound **SF21** NMR spectra is in agreement with literature reported data.* [24]

Compound SF25

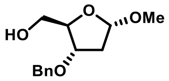

Following general procedures laid out in section **4.5.8 Synthesis of 2-deoxy-ribose C5 hydrazide**, purification through flash column chromatography afforded compound **SF25**.

$[\alpha]_D^{20}$ = +173.8° (c = 0.5, $CHCl_3$)

^{1}H NMR (600 MHz, $CDCl_3$) δ 7.35 – 7.31 (m, 4H), 7.30 – 7.26 (m, 1H), 5.05 (dd, *J* = 5.5, 1.5 Hz, 1H), 4.58 (d, *J* = 12.2 Hz, 1H), 4.49 (d, *J* = 12.2 Hz, 1H), 4.14 (td, *J* = 4.4, 3.2 Hz, 1H), 3.99 (ddd, *J* = 8.0, 4.8, 3.0 Hz, 1H), 3.77 (d, *J* = 11.9 Hz, 1H), 3.59 – 3.54 (m, 1H), 3.39 (s, 3H), 2.21 (ddd, *J* = 13.8, 8.2, 5.5 Hz, 1H), 2.06 – 2.00 (m, 2H).

^{13}C NMR (151 MHz, $CDCl_3$) δ 138.1, 128.5, 127.90, 127.85, 105.3, 83.2, 78.1, 71.9, 62.8, 55.2, 39.3.

HRMS (m/z): (ESI) calc. for $C_{13}H_{18}NaO_4$ $[M+Na]^+$: 261.1103 found 261.1101.

*Note: The compound **SF25** NMR spectra is in agreement with literature reported data.* [25]

Compound SF27

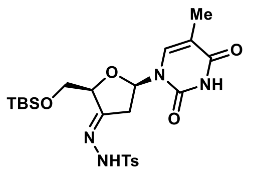

Following general procedures laid out in section **4.5.9 Synthesis of C3 Thymidine hydrazide**, purification through flash column chromatography afforded compound **SF27**.

$[\alpha]_D^{25}$ = +73.6° (c = 1.0, $CHCl_3$)

^{1}H NMR (600 MHz, CD_3CN) δ 9.05 (s, 1H), 8.52 (s, 1H), 7.80 – 7.74 (m, 2H), 7.43 (d, *J* = 1.3 Hz, 1H), 7.38 (d, *J* = 7.9 Hz, 2H), 6.18 (dd, *J* = 7.4, 6.7 Hz, 1H), 4.41 (p, *J* = 1.8 Hz, 1H), 3.89 (dd, *J* = 11.4, 2.1 Hz, 1H), 3.70 (dd, *J* = 11.4, 3.6 Hz, 1H), 3.07 (ddd, *J* = 18.1, 6.7, 0.6 Hz, 1H), 2.41 (s, 3H), 2.44 – 2.37 (m, 1H), 1.80 (d, *J* = 1.3 Hz, 3H), 0.79 (s, 9H), -0.06 (s, 3H), -0.10 (s, 3H).

^{13}C NMR (151 MHz, CD_3CN) δ 164.5, 159.1, 151.2, 145.3, 136.7, 136.3, 130.6, 128.8, 111.5, 84.2, 81.9, 65.7, 35.3, 26.2, 21.6, 18.9, 12.7, -5.3, -5.5.

HRMS (m/z): (ESI) calc. for $C_{23}H_{34}N_4NaO_6SSi$ $[M+Na]^+$: 545.1866 found 545.1871

6.3.2.2. Hydrazides

Compound gluco-99

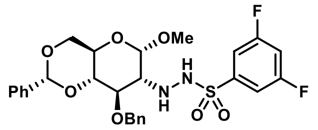

COSY, NOESY and HSQC spectra are attached below.

Following general procedures laid out in section **4.5.1 Synthesis of glucose C2 hydrazide**, purification through flash column chromatography afforded compound **gluco-99** (gluco:manno = 4:1).

$[\alpha]_D^{20}$ = +7.2° (c = 0.5, $CHCl_3$)

^{1}H NMR (600 MHz, $CDCl_3$) δ 7.49 – 7.45 (m, 2H), 7.44 – 7.30 (m, 11H), 7.08 – 7.03 (m, 2H), 5.55 (s, 1H), 4.99 (d, *J* = 11.4 Hz, 1H), 4.70 (d, *J* = 11.4 Hz, 1H), 4.59 (d, *J* = 3.5 Hz, 1H), 4.25 (d, *J* = 5.5 Hz, 1H), 3.94 (t, *J* = 9.4 Hz, 1H), 3.81 (d, *J* = 9.6 Hz, 1H), 3.73 (d, *J* = 7.2 Hz, 2H), 3.65 – 3.58 (m, 1H), 3.32 (s, 3H), 2.77 (td, *J* = 9.4, 3.5 Hz, 1H).

^{13}C NMR (151 MHz, $CDCl_3$) δ 163.6, 163.5, 161.9, 161.8, 141.9, 141.89, 141.83, 137.5, 137.3, 129.2, 129.0, 128.53, 128.47, 128.4, 126.1, 111.93, 111.88, 111.8, 111.7, 109.0, 108.8, 108.6, 101.5, 99.5, 83.0, 79.8, 74.9, 69.1, 62.54, 62.47, 55.4.

HRMS (m/z): (ESI) calc. for $C_{27}H_{29}F_2N_2O_7S$ $[M+H]^+$: 563.1664 found 563.1655.

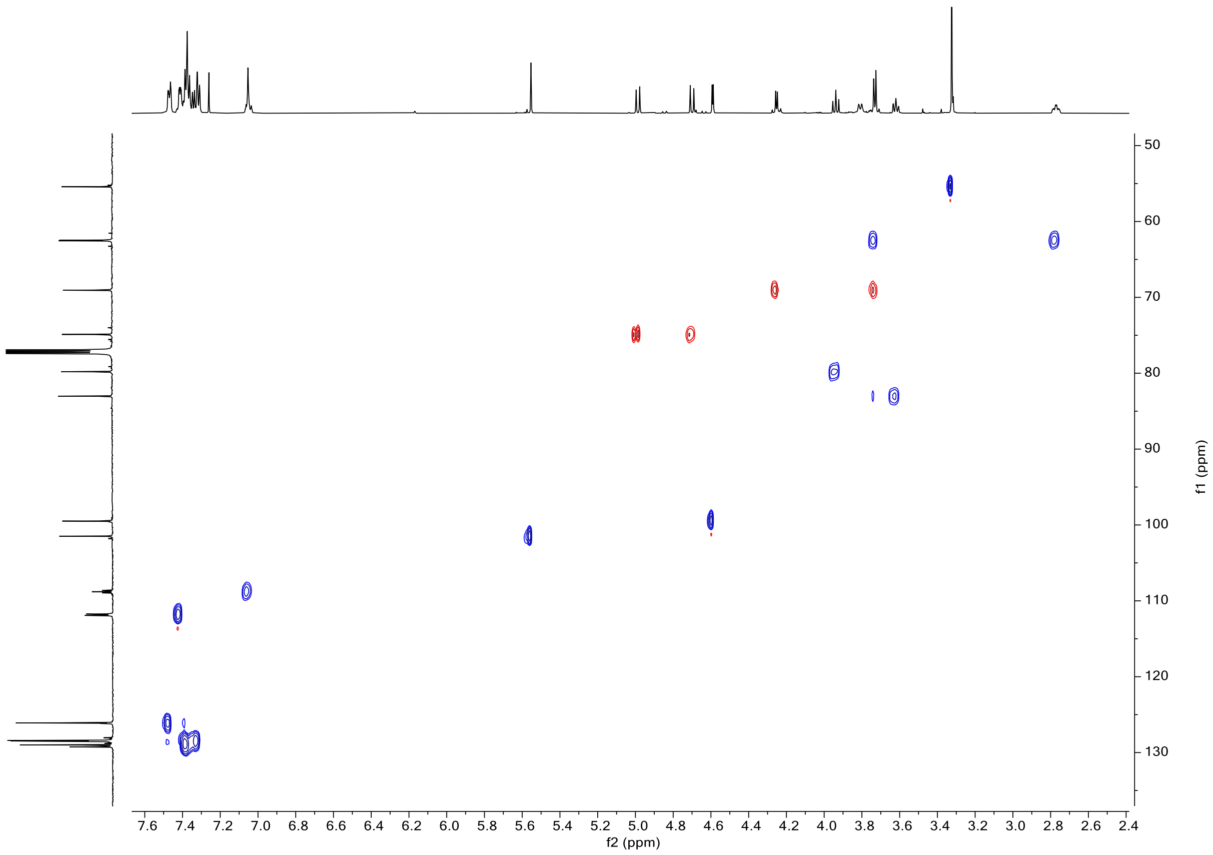

H-C HSQC NMR Spectrum of glucose-C2 hydrazide **gluco-99**

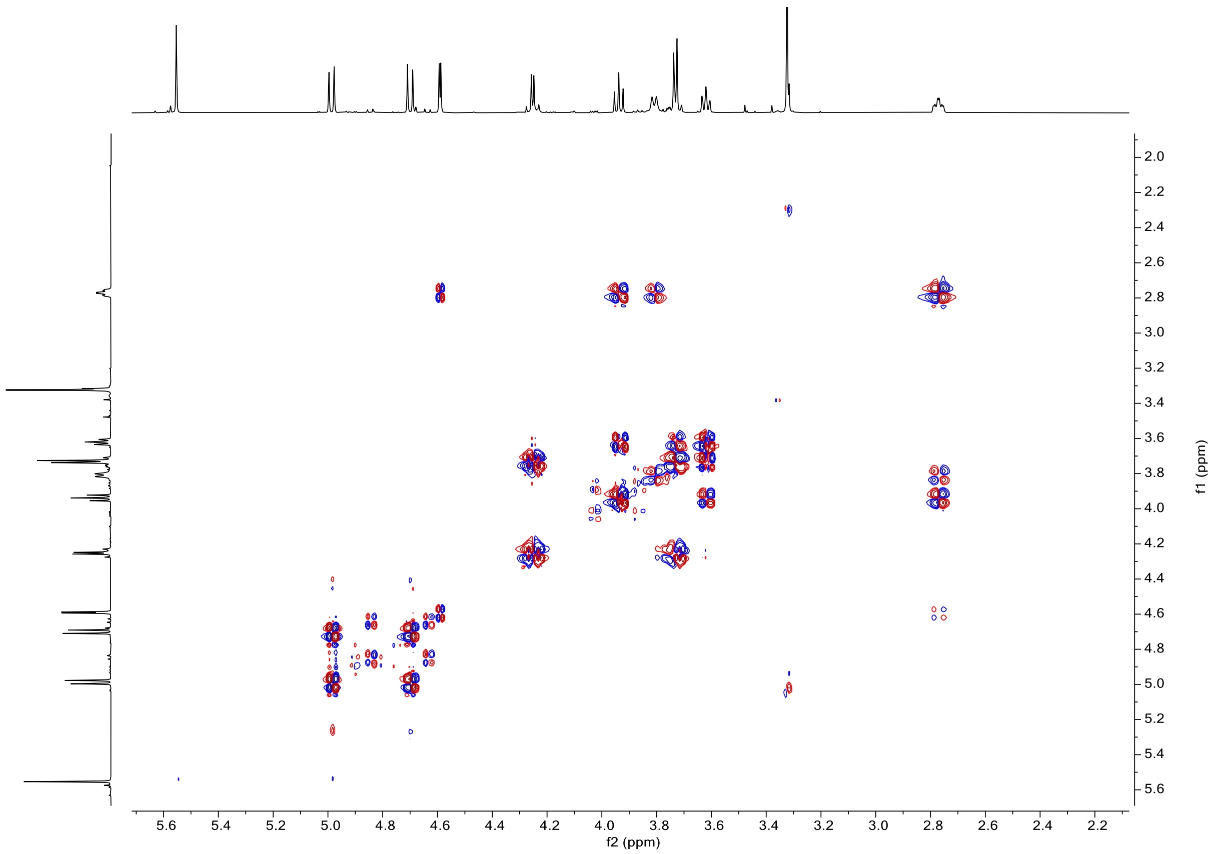

H-H COSY NMR Spectrum of glucose-C2 hydrazide **gluco-99**

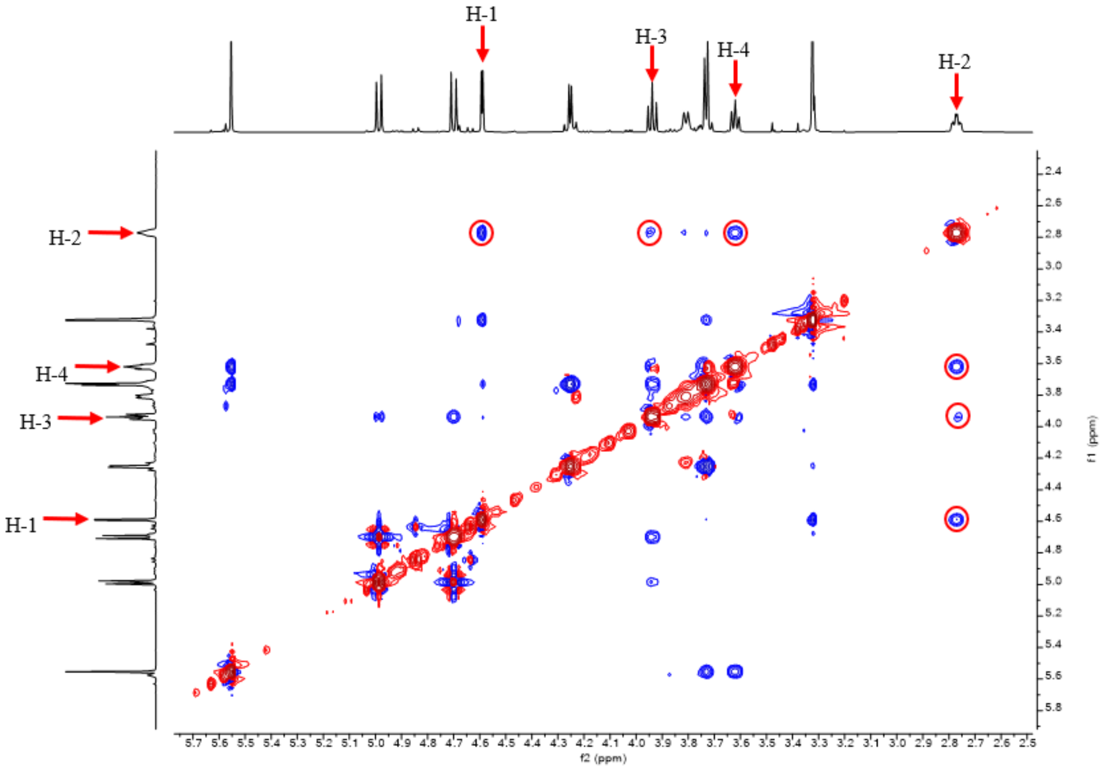

NOESY NMR Spectrum of glucose-C2 hydrazide **gluco-99**

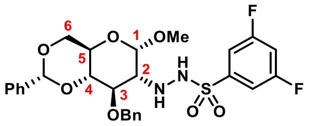

Compound allo-105

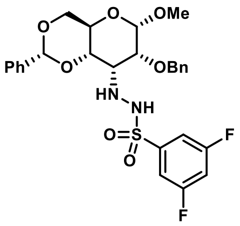

COSY, NOESY and HSQC spectra are attached below.

Following general procedures laid out in section **4.5.2 Synthesis of glucose C3 hydrazide**, purification through flash column chromatography afforded compound **allo-105** (allo:gluco > 19:1).

$[\alpha]_D^{20}$ = –34.8° (c = 1.5, $CHCl_3$)

^{1}H NMR (600 MHz, $CDCl_3$) δ 7.51 – 7.46 (m, 4H), 7.44 – 7.31 (m, 7H), 7.16 (s, 1H), 6.98 (tt, *J* = 8.5, 2.4 Hz, 1H), 5.46 (s, 1H), 4.87 (s, 1H), 4.83 (d, *J* = 12.3 Hz, 1H), 4.65 (d, *J* = 3.4 Hz, 1H), 4.61 (d, *J* = 12.3 Hz, 1H), 4.31 (dd, *J* = 10.4, 5.1 Hz, 1H), 3.99 (s, 1H), 3.92 (td, *J* = 10.1, 5.1 Hz, 1H), 3.67 (t, *J* = 10.3 Hz, 1H), 3.60 (dd, *J* = 9.9, 3.6 Hz, 1H), 3.52 (dd, *J* = 4.4, 3.5 Hz, 1H), 3.39 (s, 3H).

^{13}C NMR (151 MHz, $CDCl_3$) δ 162.6 (dd, *J* = 253.7, 11.5 Hz), 142.1 (t, *J* = 8.6 Hz), 137.1, 136.8, 129.5, 128.8, 128.6, 128.3, 128.2, 126.0, 112.3 – 111.8 (m), 108.5 (t, *J* = 25.0 Hz), 102.3, 99.1, 80.2, 73.2, 70.3, 69.2, 58.3, 57.3, 55.9.

HRMS (m/z): (ESI) calc. for $C_{27}H_{29}F_2N_2O_7S$ $[M+H]^+$: 563.1664 found 563.1652.

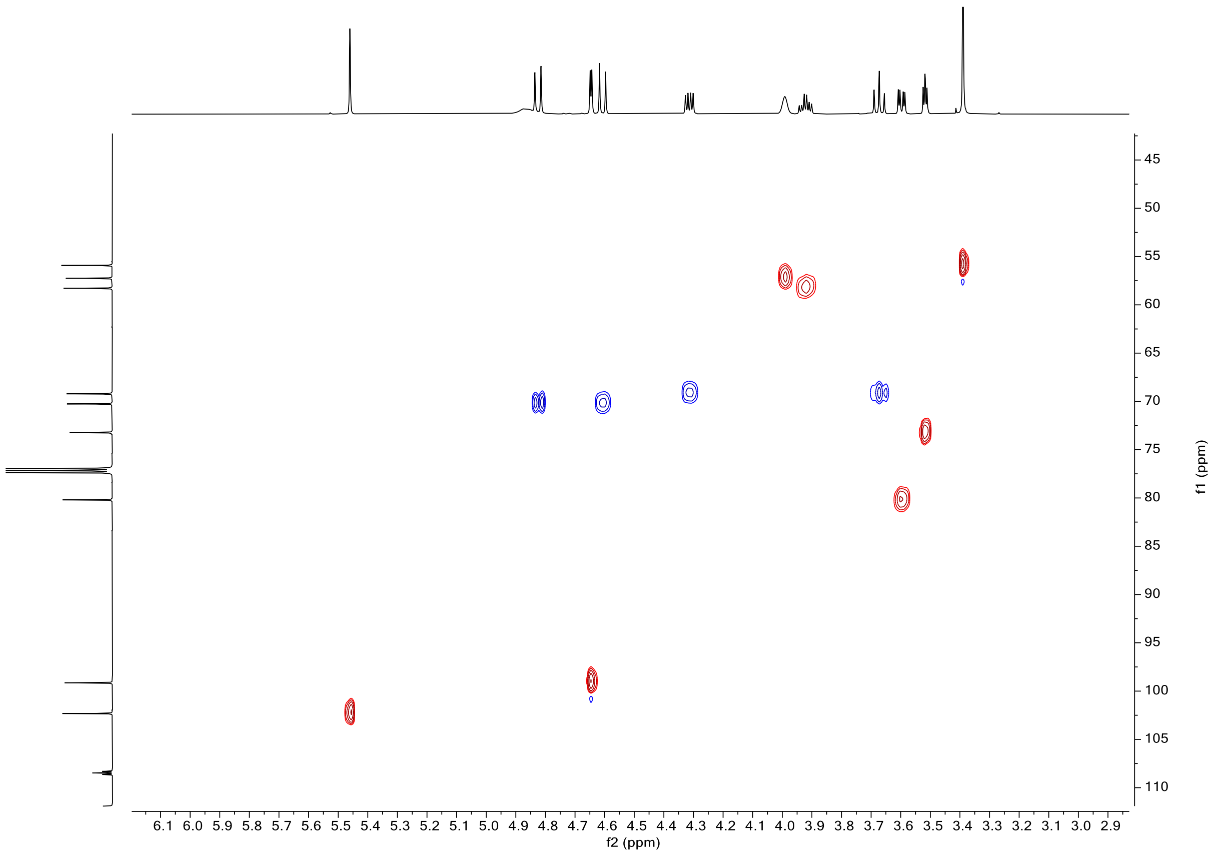

H-C HSQC NMR Spectrum of glucose-C3 hydrazide **allo-105**

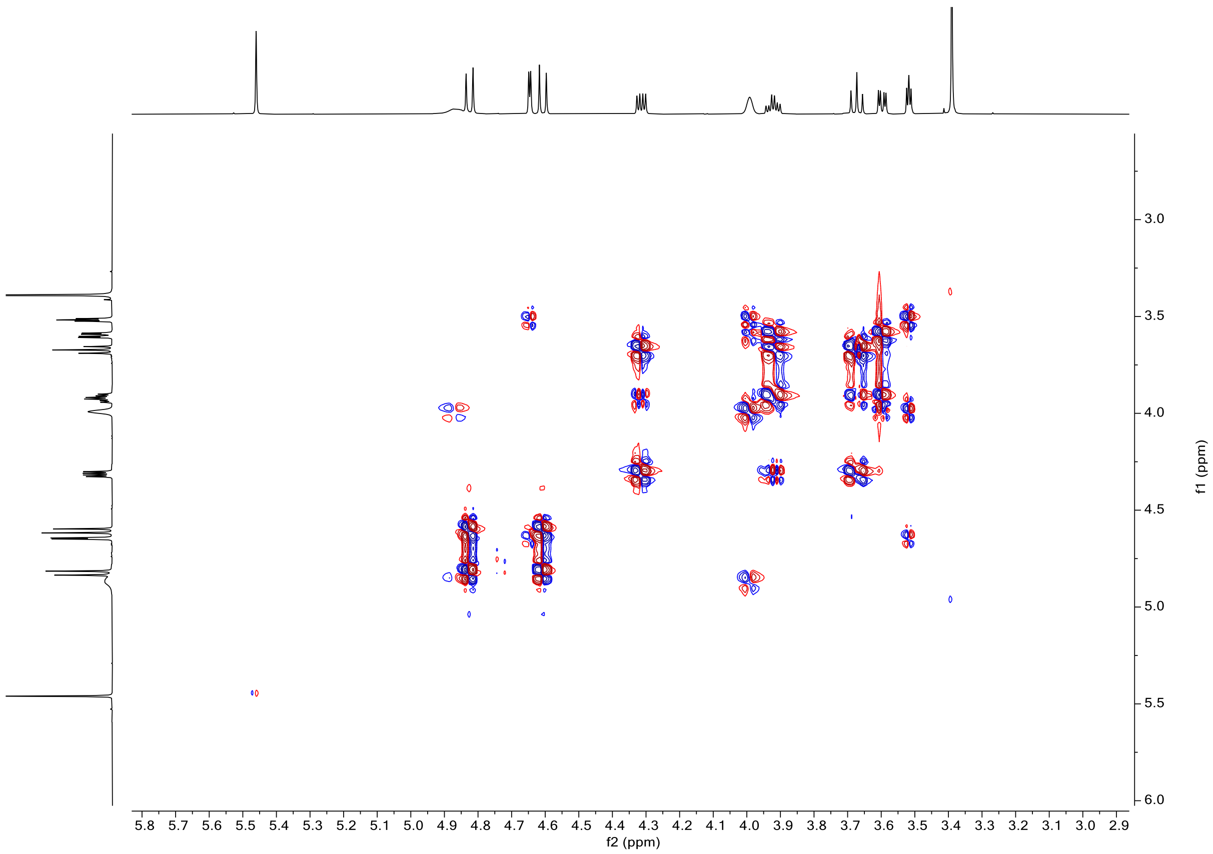

H-H COSY NMR Spectrum of glucose-C3 hydrazide **allo-105**

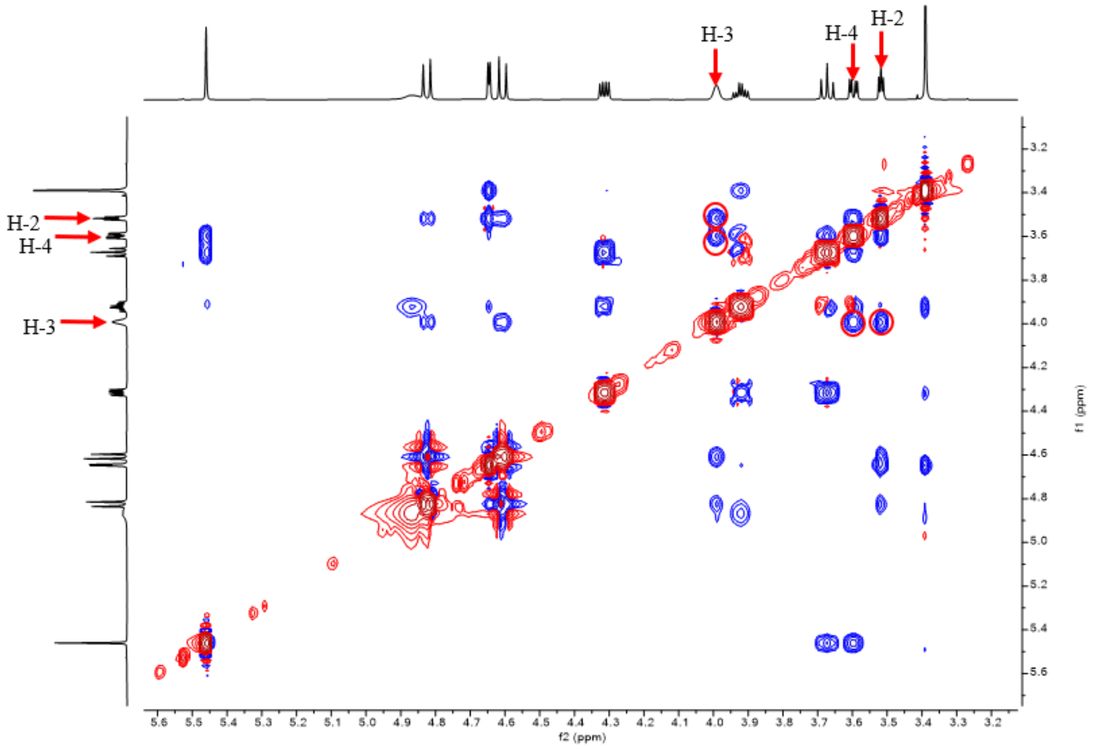

NOESY NMR Spectrum of glucose-C3 hydrazide **allo-105**

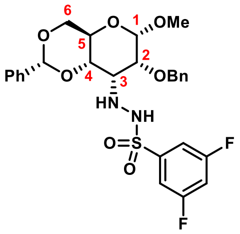

Compound galacto-111

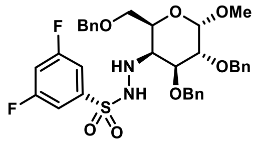

COSY, NOESY and HSQC spectra are attached below.

Following general procedures laid out in section **4.5.3 Synthesis of glucose C4 hydrazide**, purification through flash column chromatography afforded compound **galacto-111** (galacto:gluco > 19:1).

$[\alpha]_D^{20}$ = +6.7° (c = 1.5, $CHCl_3$)

^{1}H NMR (600 MHz, $CDCl_3$) δ 7.43 – 7.28 (m, 17H), 6.97 (tt, *J* = 8.4, 2.4 Hz, 1H), 4.83 (d, *J* = 11.3 Hz, 1H), 4.79 (d, *J* = 12.1 Hz, 1H), 4.73 (d, *J* = 11.3 Hz, 1H), 4.66 (d, *J* = 12.1 Hz, 1H), 4.60 (d, *J* = 3.8 Hz, 1H), 4.53 (s, 2H), 3.98 (dd, *J* = 10.1, 4.0 Hz, 1H), 3.91 (ddd, *J* = 6.4, 4.1, 1.9 Hz, 1H), 3.64 (dd, *J* = 10.1, 3.9 Hz, 1H), 3.62 – 3.54 (m, 2H), 3.35 (s, 3H), 3.31 (dd, *J* = 4.1, 1.9 Hz, 1H).

^{13}C NMR (151 MHz, $CDCl_3$) δ 163.5, 163.4, 161.8, 161.7, 142.0, 141.92, 141.86, 137.94, 137.90, 137.7, 128.9, 128.64, 128.55, 128.4, 128.2, 128.1, 127.9, 127.7, 111.9, 111.8, 111.72, 111.68, 108.8, 108.6, 108.4, 98.2, 80.0, 76.3, 74.4, 73.7, 73.4, 70.4, 68.5, 59.5, 55.4.

HRMS (m/z): (ESI) calc. for $C_{34}H_{37}F_2N_2O_7S$ $[M+H]^+$: 655.2290 found 655.2279.

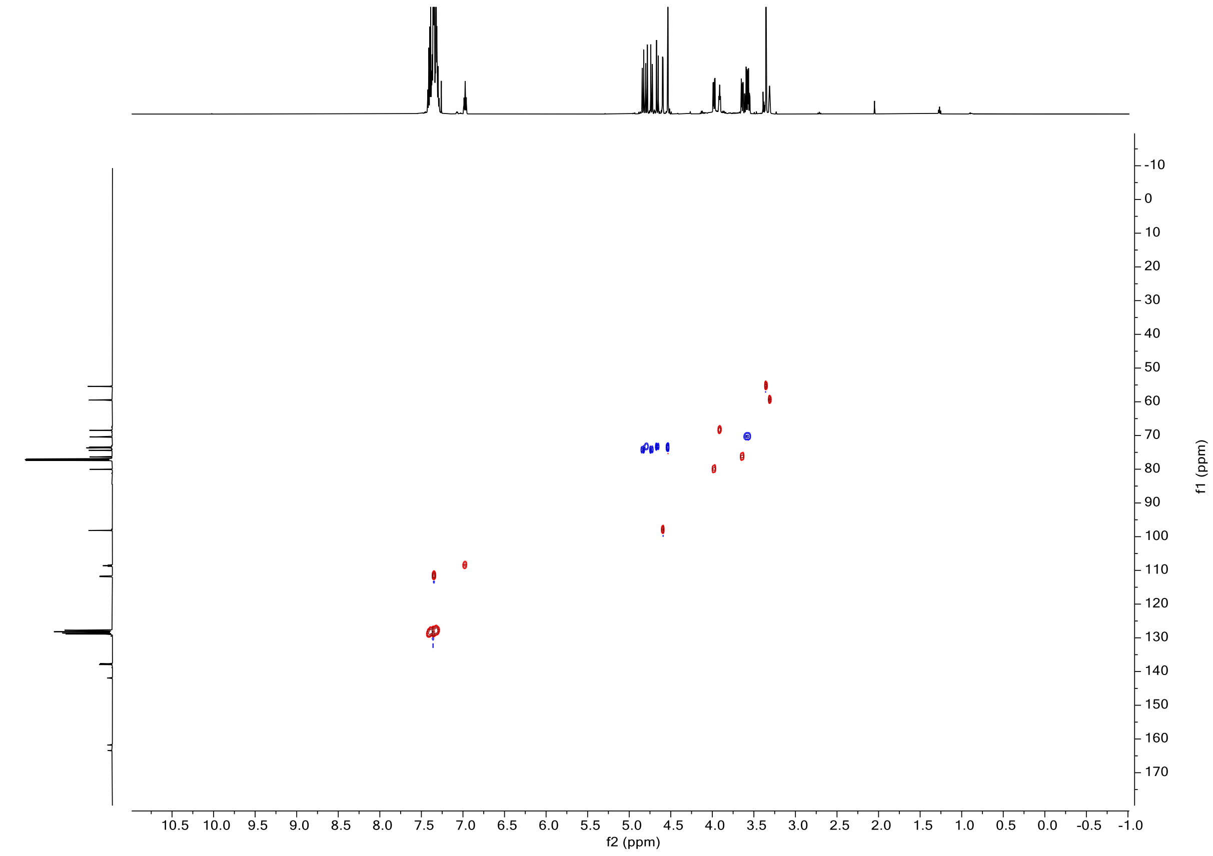

H-C HSQC NMR Spectrum of glucose-C4 hydrazide **galacto-111**

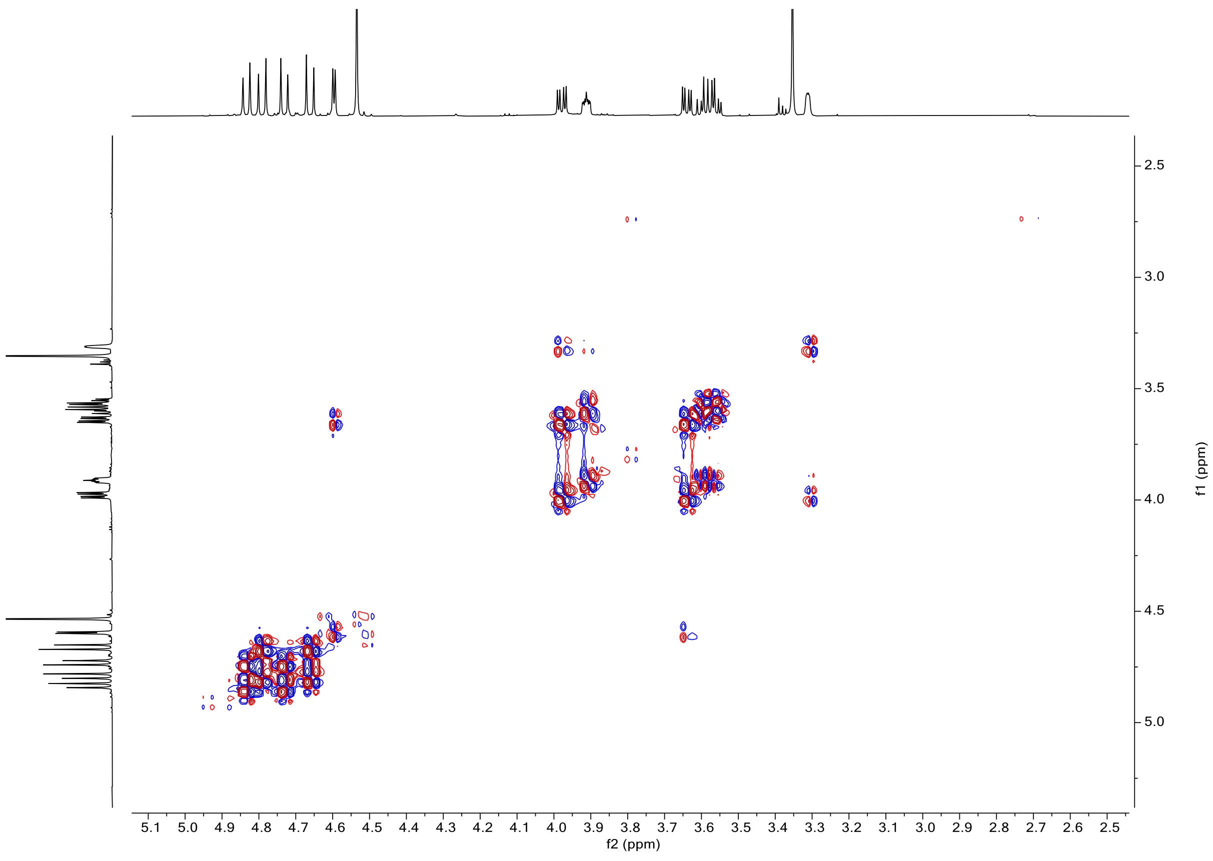

H-H COSY NMR Spectrum of glucose-C3 hydrazide **galacto-111**

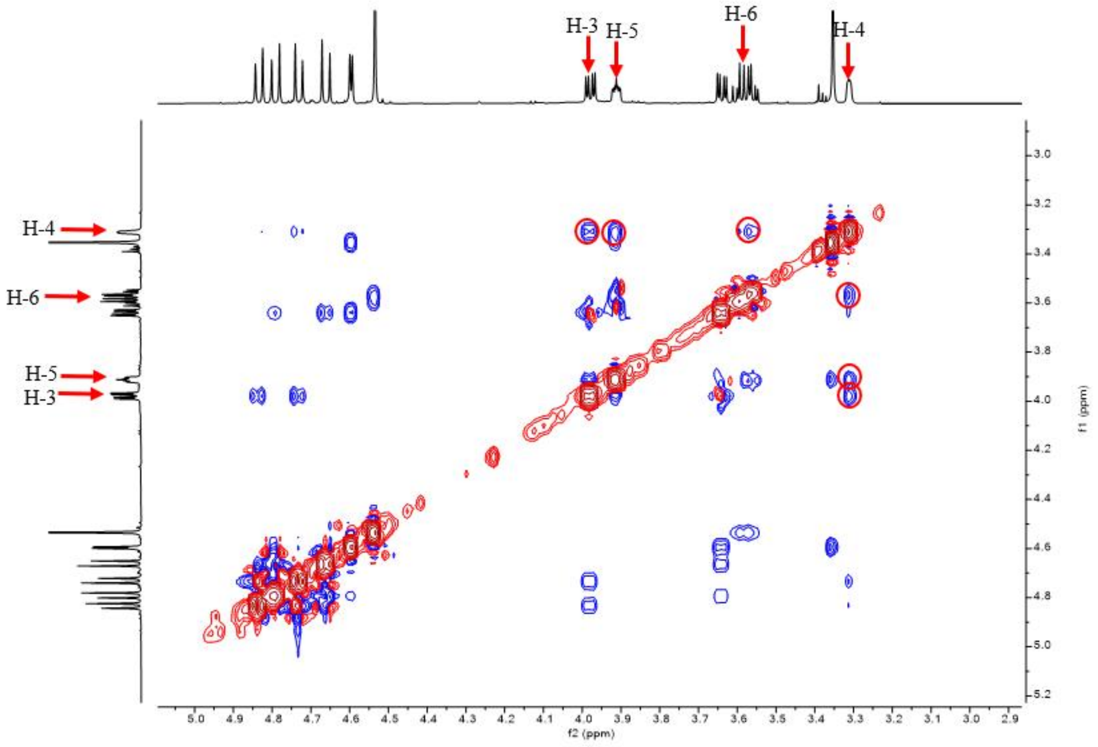

NOESY NMR Spectrum of glucose-C4 hydrazide **galacto-111**

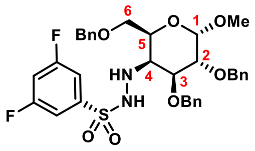

Compound gluco-117

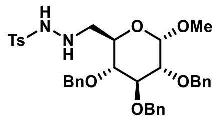

Following general procedures laid out in section **4.5.4 Synthesis of glucose C6 hydrazide**, purification through flash column chromatography afforded compound **gluco-117**.

$[\alpha]_D^{20}$ = +6.0° (c = 0.5, $CHCl_3$)

^{1}H NMR (600 MHz, $CDCl_3$) δ 7.77 (d, *J* = 8.3 Hz, 2H), 7.36 – 7.24 (m, 15H), 7.21 – 7.17 (m, 2H), 6.02 (s, 1H), 4.95 (d, *J* = 10.8 Hz, 1H), 4.79 – 4.76 (m, 3H), 4.63 (d, *J* = 12.1 Hz, 1H), 4.50 (d, *J* = 3.5 Hz, 1H), 4.47 (d, *J* = 10.8 Hz, 1H), 3.91 (dd, *J* = 9.6, 8.9 Hz, 1H), 3.75 (s, 1H), 3.65 (ddd, *J* = 9.6, 6.2, 3.1 Hz, 1H), 3.42 (dd, *J* = 9.7, 3.6 Hz, 1H), 3.33 (s, 3H), 3.26 (dd, *J* = 10.0, 8.8 Hz, 1H), 2.98 (d, *J* = 13.1 Hz, 1H), 2.86 – 2.80 (m, 1H), 2.37 (s, 3H).

^{13}C NMR (151 MHz, $CDCl_3$) δ 144.1, 138.7, 138.1, 138.0, 135.5, 129.7, 128.7, 128.62, 128.59, 128.3, 128.24, 128.20, 128.14, 128.06, 127.9, 98.1, 81.9, 79.9, 78.6, 75.9, 75.1, 73.6, 70.3, 55.6, 51.6, 21.7.

HRMS (m/z): (ESI) calc. for $C_{35}H_{40}N_2O_7SNa$ $[M+Na]^+$: 655.2454 found 655.2440.

Compound arabino-123

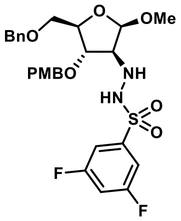

COSY, NOESY and HSQC spectra are attached below.

Following general procedures laid out in section **4.5.5 Synthesis of ribose C2 and C3 hydrazide**, purification through flash column chromatography afforded compound **arabino-123** (arabino:ribo > 19:1).

$[\alpha]_D^{20}$ = –30.4° (c = 0.5, $CHCl_3$)

^{1}H NMR (600 MHz, $CDCl_3$) δ 7.49 – 7.45 (m, 2H), 7.37 – 7.27 (m, 6H), 7.19 (d, *J* = 8.4 Hz, 2H), 7.07 – 7.01 (m, 1H), 6.88 – 6.84 (m, 2H), 6.35 (s, 1H), 4.87 (d, *J* = 4.5 Hz, 1H), 4.57 – 4.48 (m, 3H), 4.48 (s, 1H), 4.16 – 4.11 (m, 1H), 4.05 (q, *J* = 5.2 Hz, 1H), 3.80 (s, 3H), 3.74 (t, *J* = 5.5 Hz, 1H), 3.49 (dt, *J* = 10.5, 5.4 Hz, 1H), 3.46 (d, *J* = 5.3 Hz, 2H), 3.34 (s, 3H).

^{13}C NMR (151 MHz, $CDCl_3$) δ 162.8 (dd, *J* = 254.5, 11.6 Hz), 159.5, 141.6, 137.9, 129.64, 129.61, 128.5, 127.9, 114.0, 112.1 – 111.7 (m), 108.9 (t, *J* = 25.0 Hz), 102.8, 81.5, 81.3, 73.5, 71.6, 71.5, 68.7, 55.4 (d, *J* = 9.3 Hz).

HRMS (m/z): (ESI) calc. for $C_{27}H_{31}F_2N_2O_7S$ $[M+H]^+$: 565.1820 found 565.1817.

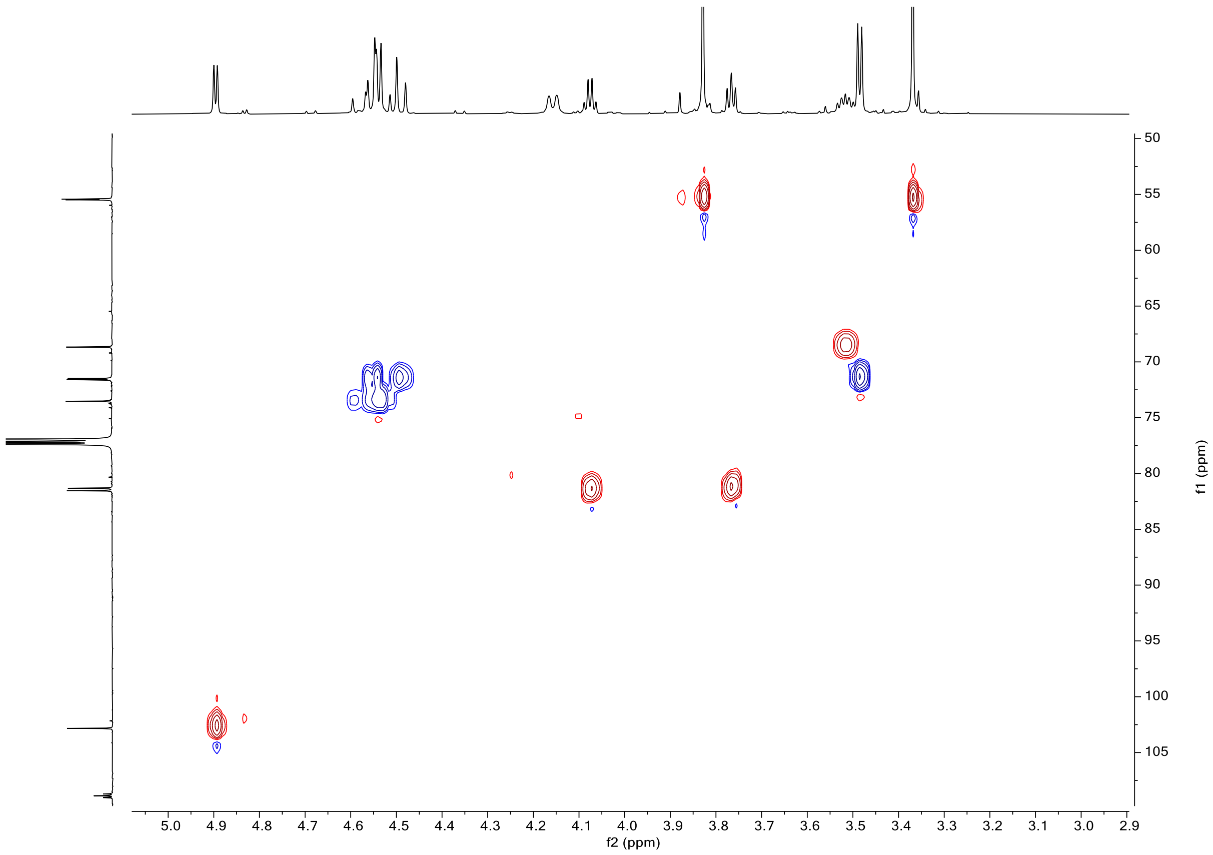

H-C HSQC NMR Spectrum of ribose-C2 hydrazide **arabino-123**

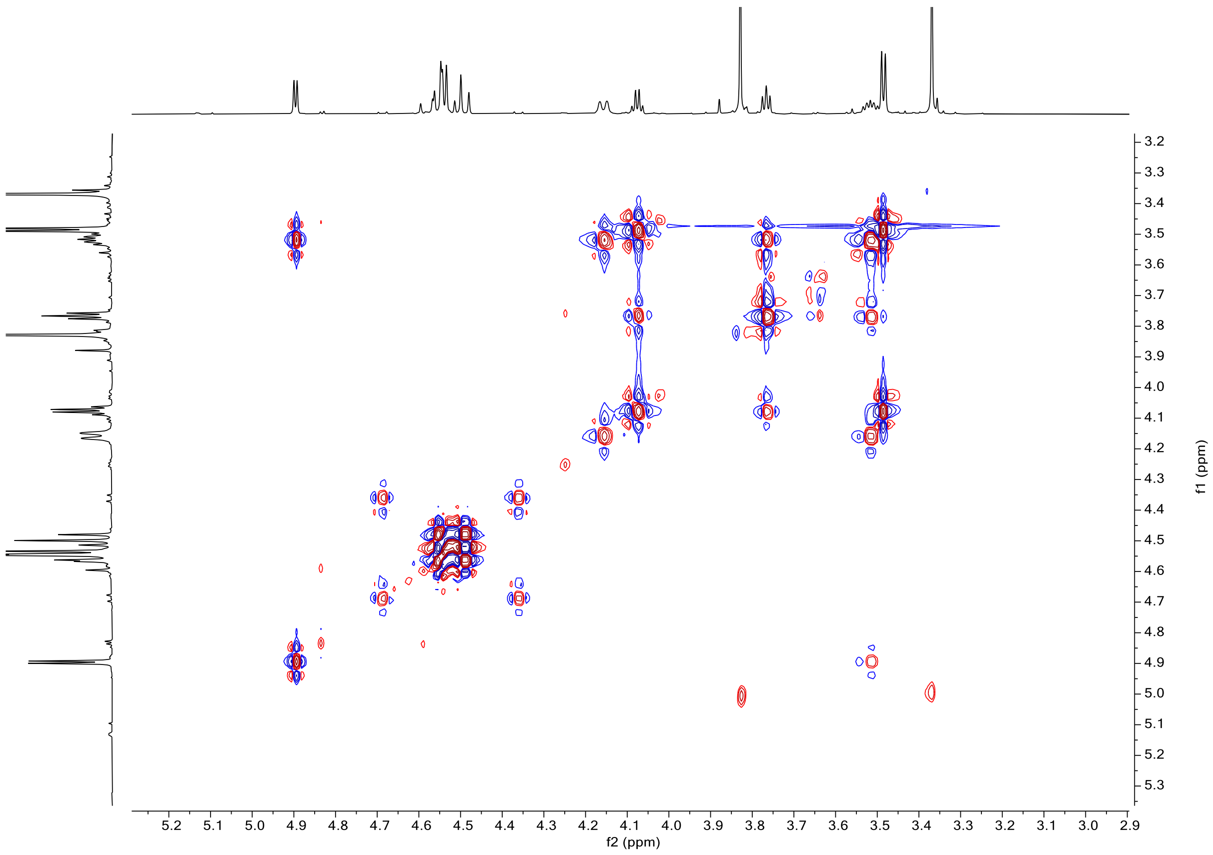

H-H COSY NMR Spectrum of ribose-C2 hydrazide **arabino-123**

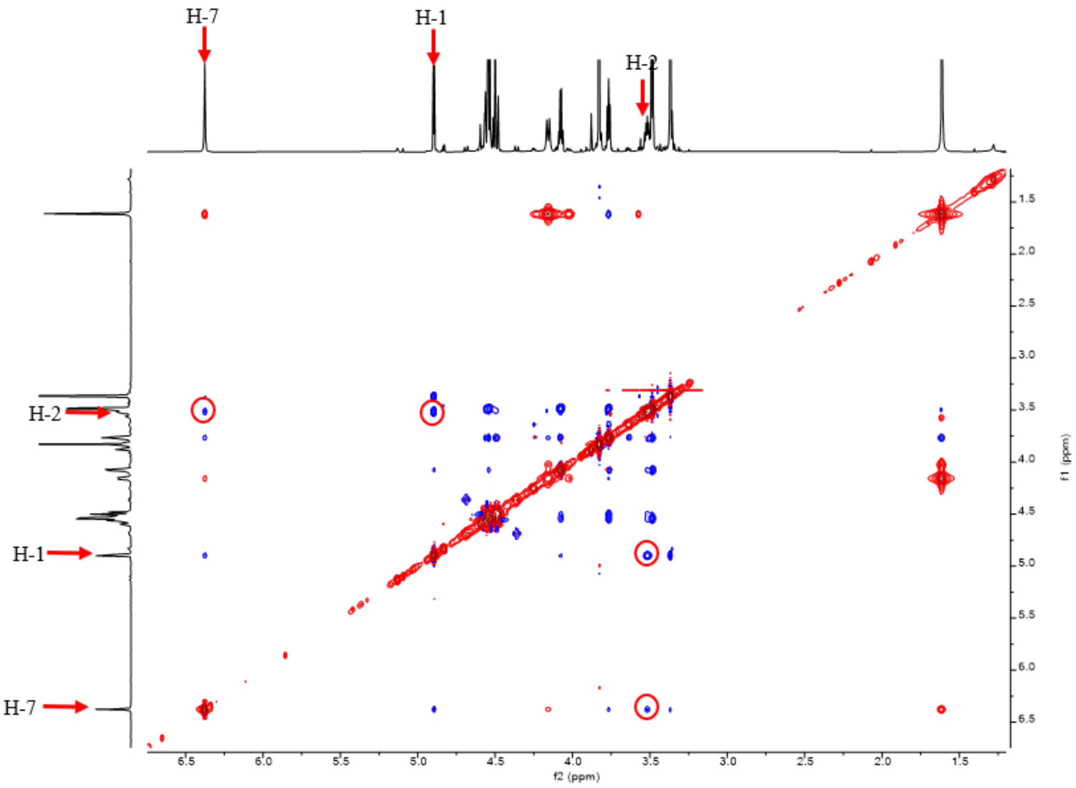

NOESY NMR Spectrum of ribose-C2 hydrazide **arabino-123**

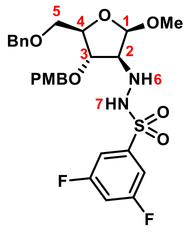

Compound 125

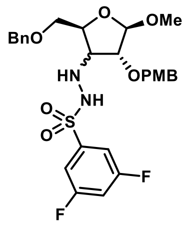

COSY, NOESY and HSQC spectra are attached below.

Following general procedures laid out in section **4.5.5 Synthesis of ribose C2 and C3 hydrazide**, purification through flash column chromatography afforded compound **125** (xylo:ribo = 2:1).

Characterized as a 1:2 mixture.

^{1}H NMR (600 MHz, $CDCl_3$) δ 7.4 – 7.3 (m, 20H), 7.3 – 7.2 (m, 6H), 7.0 – 7.0 (m, 5H), 6.9 – 6.9 (m, 6H), 4.8 (s, 1H), 4.7 (s, 2H), 4.6 (d, *J* = 11.4 Hz, 1H), 4.6 – 4.6 (m, 4H), 4.6 (d, *J* = 11.4 Hz, 2H), 4.5 – 4.4 (m, 4H), 4.4 – 4.3 (m, 2H), 4.2 (s, 1H), 3.9 (dt, *J* = 8.0, 5.9 Hz, 1H), 3.8 (s, 3H), 3.8 (s, 6H), 3.8 (d, *J* = 1.8 Hz, 2H), 3.8 (d, *J* = 5.0 Hz, 1H), 3.7 (dd, *J* = 10.5, 4.6 Hz, 2H), 3.7 (dd, *J* = 10.4, 7.5 Hz, 2H), 3.5 (dd, *J* = 9.7, 5.6 Hz, 1H), 3.4 (s, 2H), 3.4 – 3.3 (m, 1H), 3.2 (s, 6H), 3.2 (s, 3H).

^{13}C NMR (151 MHz, $CDCl_3$) δ 163.5 (dd, *J* = 11.7, 7.4 Hz), 161.8 (dd, *J* = 11.6, 7.4 Hz), 159.9, 159.6, 142.0 – 141.5 (m), 137.4, 136.9, 129.9, 129.7, 129.2, 128.9, 128.7, 128.5, 128.2, 128.1, 114.4, 114.1, 111.8 (dt, *J* = 22.0, 7.8 Hz), 108.6 (t, *J* = 25.0 Hz), 106.9, 105.7, 85.1, 80.8, 80.6, 79.3, 74.3, 73.8, 72.5, 71.9, 71.4, 68.9, 65.7, 64.5, 55.5, 55.4, 55.2, 54.8.

HRMS (m/z): (ESI) calc. for $C_{27}H_{31}F_2N_2O_7S$ $[M+H]^+$: 565.1820 found 565.1815.

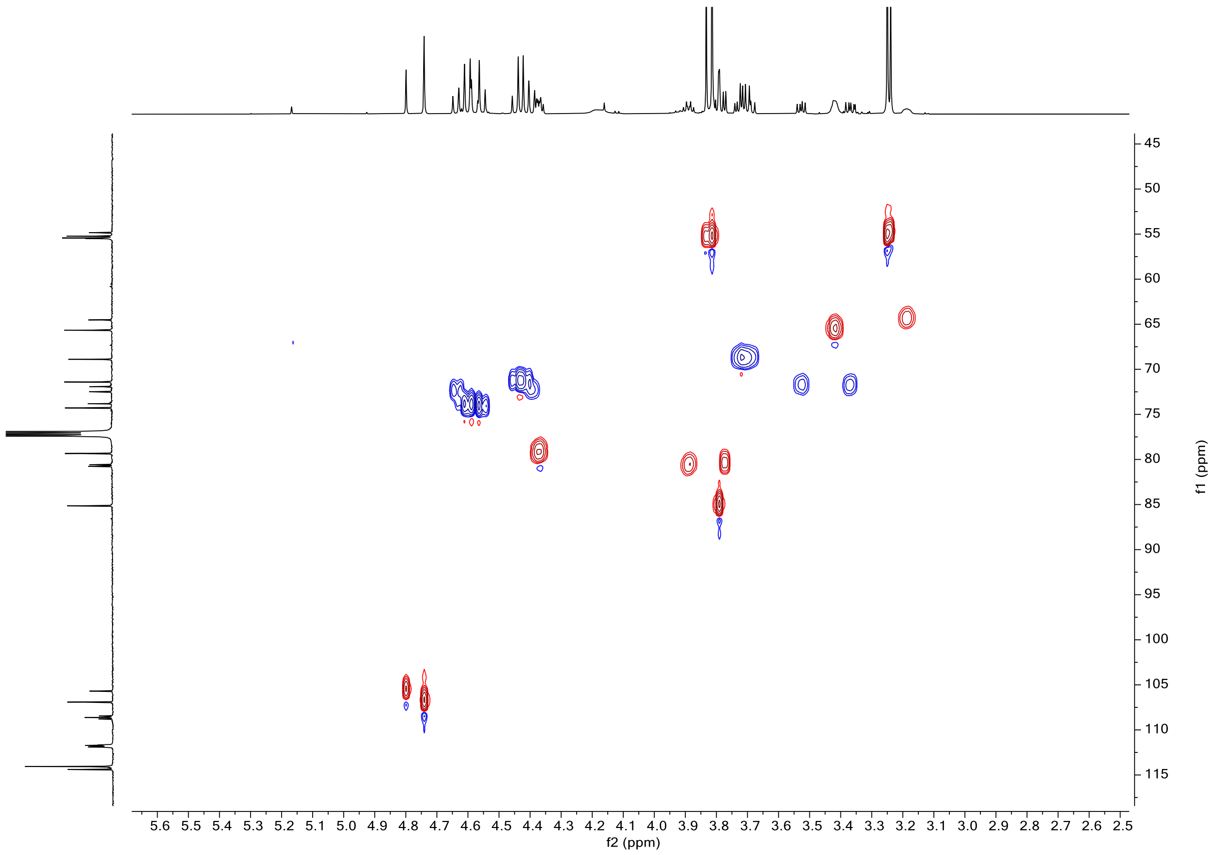

H-C HSQC NMR Spectrum of ribose-C3 hydrazide **125** (xylo:ribo = 2:1)

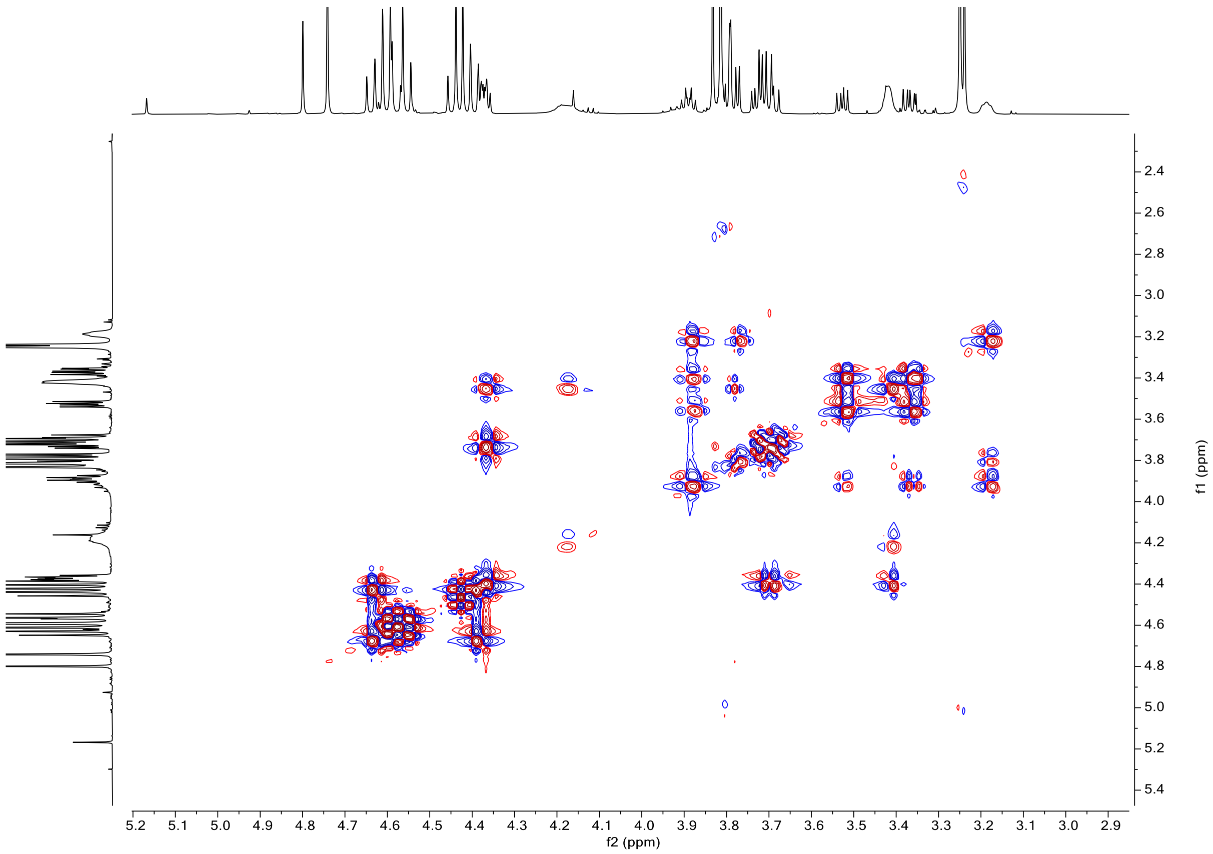

H-H COSY NMR Spectrum of ribose-C3 hydrazide **125** (xylo:ribo = 2:1)

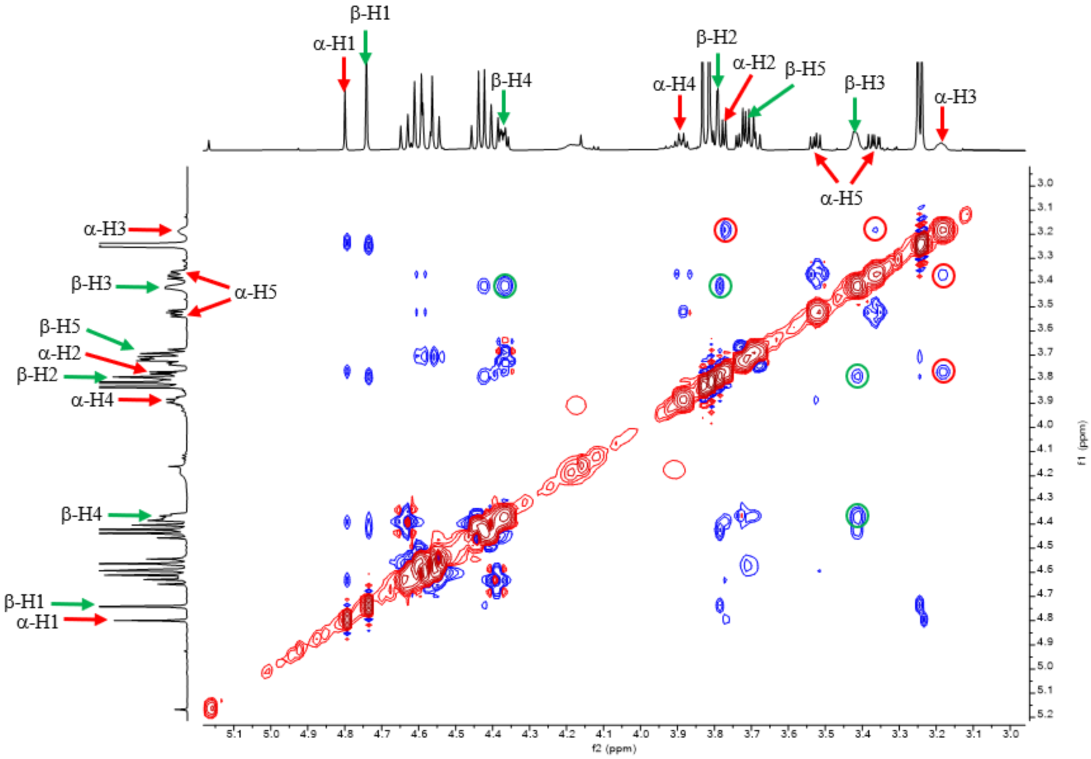

NOESY NMR Spectrum of ribose-C3 hydrazide **125** (xylo:ribo = 2:1)

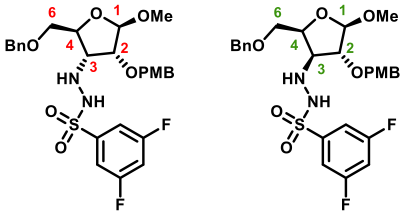

ribo-133 **xylo-133**

Compound 127

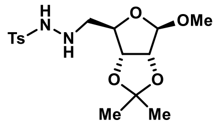

Following general procedures laid out in section **4.5.6 Synthesis of ribose C5 hydrazide**, purification through flash column chromatography afforded compound **127**.

$[\alpha]_D^{25}$ = –18° (c = 0.5, $CHCl_3$)

^{1}H NMR (600 MHz, $CDCl_3$) δ 7.81 (d, *J* = 8.3 Hz, 2H), 7.32 (d, *J* = 8.5 Hz, 2H), 6.11 (s, 1H), 4.90 (s, 1H), 4.52 (dd, *J* = 5.9, 1.1 Hz, 1H), 4.49 (d, *J* = 6.0 Hz, 1H), 4.23 (ddd, *J* = 7.8, 5.3, 1.1 Hz, 1H), 3.27 (s, 3H), 2.90 – 2.80 (m, 2H), 2.43 (s, 3H), 1.44 (s, 3H), 1.28 (s, 3H).

^{13}C NMR (151 MHz, $CDCl_3$) δ 144.3, 135.4, 129.8, 128.2, 112.6, 110.1, 85.3, 84.9, 82.4, 55.6, 54.7, 26.6, 25.0, 21.7.

HRMS (m/z): (ESI) calc. for $C_{16}H_{25}N_2O_6S$ $[M+H]^+$: 373.1433 found 373.1429.

Compound 2-deoxy-xylo-129

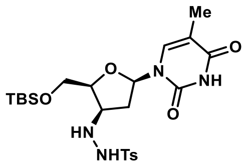

Following general procedures laid out in section **4.5.9 Synthesis of C3 Thymidine hydrazide**, purification through flash column chromatography afforded compound **2-deoxy-xylo-129**.

$[\boldsymbol{\alpha}]_{\mathbf{D}}^{\mathbf{25}}$ = –6.4° (c = 1.0, $CHCl_3$)

^{1}H NMR (600 MHz, CD_3CN) δ 9.27 (s, 1H), 7.69 (d, *J* = 8.3 Hz, 2H), 7.65 (d, *J* = 1.3 Hz, 1H), 7.42 – 7.34 (m, 2H), 6.99 (d, *J* = 3.6 Hz, 1H), 5.98 (dd, *J* = 7.5, 4.8 Hz, 1H), 4.16 (t, *J* = 3.8 Hz, 1H), 3.95 (q, *J* = 5.3 Hz, 1H), 3.89 – 3.82 (m, 2H), 3.60 (ddt, *J* = 7.0, 5.2, 3.6 Hz, 1H), 2.46 (dt, *J* = 13.5, 6.8 Hz, 1H), 2.41 (s, 3H), 2.11 (ddd, *J* = 14.2, 4.8, 3.3 Hz, 1H), 1.87 (d, *J* = 1.2 Hz, 3H), 0.79 (s, 9H), -0.01 (s, 3H), -0.02 (s, 3H).

^{13}C NMR (151 MHz, CD_3CN) δ 165.0, 151.6, 145.1, 137.9, 136.4, 130.7, 128.6, 110.3, 85.1, 81.7, 62.5, 61.4, 37.5, 26.2, 21.5, 18.8, 12.8, -5.4.

HRMS (m/z): (ESI) calc. for $C_{23}H_{36}N_4O_6SSi$ $[M+H]^+$: 525.220 found 525.2204.

Compound 2-deoxy-ribo-131

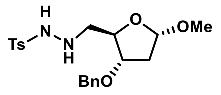

Following general procedures laid out in section **4.5.8 Synthesis of 2-deoxy-ribose C5 hydrazide**, purification through flash column chromatography afforded compound **2-deoxy-ribo-131**.

$[\alpha]_D^{20}$ = +58.2° (c = 0.5, $CHCl_3$)

^{1}H NMR (600 MHz, $CDCl_3$) δ 7.79 (d, J = 8.3 Hz, 2H), 7.37 – 7.26 (m, 7H), 6.22 (s, 1H), 4.96 (dd, J = 5.6, 1.7 Hz, 1H), 4.51 (d, J = 12.1 Hz, 1H), 4.40 (d, J = 12.0 Hz, 1H), 4.05 (ddd, J = 7.0, 5.5, 3.8 Hz, 1H), 3.73 (ddd, J = 8.6, 5.4, 3.6 Hz, 1H), 3.35 (s, 3H), 2.94 (dd, J = 13.2, 3.8 Hz, 1H), 2.71 (dd, J = 13.2, 7.0 Hz, 1H), 2.41 (s, 3H), 2.18 (ddd, J = 13.9, 8.3, 5.6 Hz, 1H), 1.95 (ddd, J = 14.0, 3.6, 1.6 Hz, 1H), 1.63 (s, 1H).

^{13}C NMR (151 MHz, $CDCl_3$) δ 144.1, 137.7, 135.5, 129.7, 128.6, 128.3, 128.04, 127.95, 105.0, 81.4, 79.5, 72.0, 55.4, 53.2, 38.8, 21.7.

HRMS (m/z): (ESI) calc. for $C_{20}H_{27}N_2O_5S$ $[M+H]^+$: 407.1641 found 407.1638.

Compound 2-deoxy-xylo-SF22

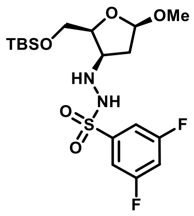

Following general procedures laid out in section **4.5.7 Synthesis of 2-deoxy-ribose C3 hydrazide**, purification through flash column chromatography afforded compound **2-deoxy-xylo-SF22**.

$[\alpha]_D^{20}$ = –34.4° (c = 0.5, $CHCl_3$)

^{1}H NMR (600 MHz, $CDCl_3$) δ 7.69 (s, 1H), 7.47 (dt, *J* = 7.6, 3.7 Hz, 2H), 7.03 (tt, *J* = 8.5, 2.3 Hz, 1H), 4.83 (d, *J* = 4.9 Hz, 1H), 3.98 – 3.91 (m, 2H), 3.78 (dd, *J* = 11.0, 8.8 Hz, 1H), 3.35 (ddd, *J* = 7.6, 4.7, 1.5 Hz, 1H), 3.26 (s, 3H), 2.09 (ddd, *J* = 13.8, 7.6, 5.0 Hz, 1H), 1.92 (dd, *J* = 14.3, 1.1 Hz, 1H), 0.93 (s, 9H), 0.14 (s, 6H).

^{13}C NMR (151 MHz, $CDCl_3$) δ 162.7 (dd, *J* = 254.0, 11.5 Hz), 142.4 (t, *J* = 8.5 Hz), 112.0 – 111.6 (m), 108.4 (t, *J* = 25.1 Hz), 104.2, 82.0, 62.1, 61.5, 55.1, 38.7, 25.9, 18.3, -5.3, -5.4.

HRMS (m/z): (ESI) calc. $C_{18}H_{31}F_2N_2O_5SSi$ $[M+H]^+$: 452.1613 found 452.1621

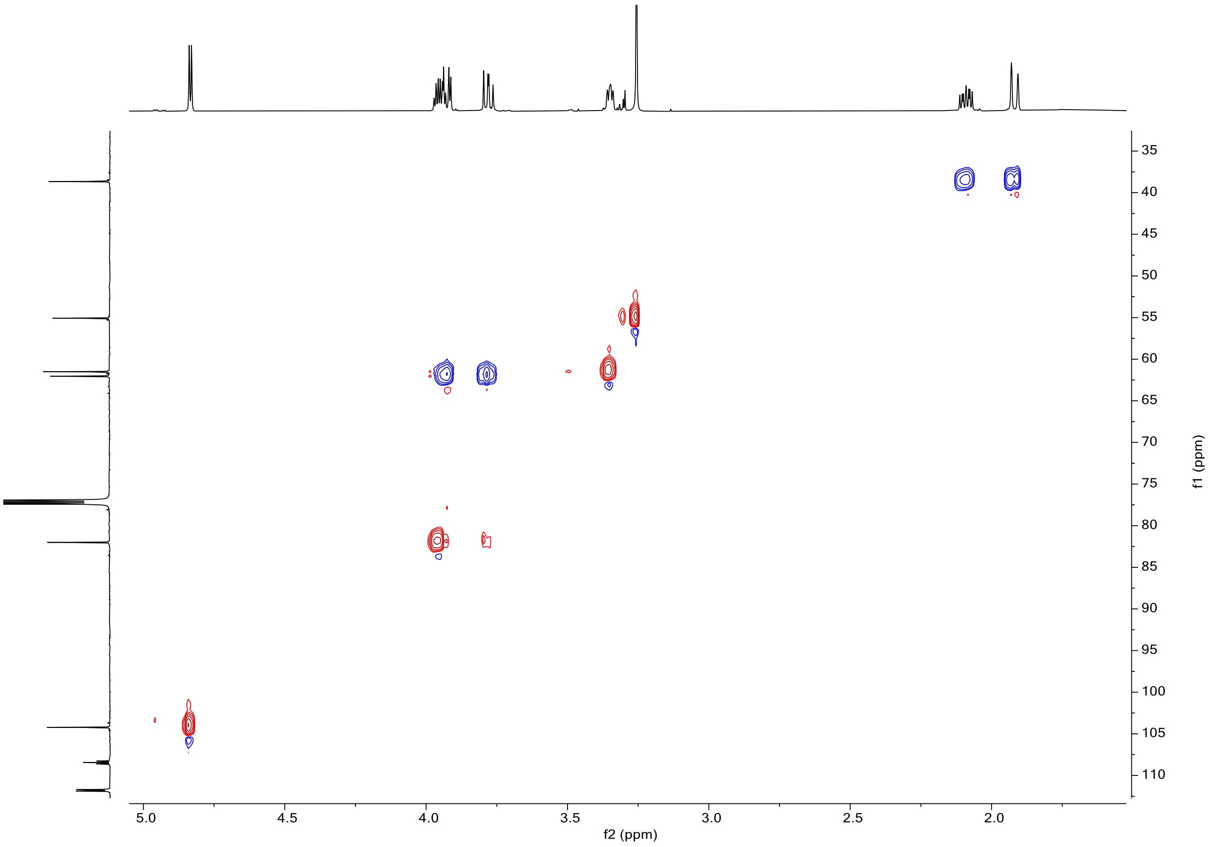

H-C HSQC NMR Spectrum of ribose-C3 hydrazide **SF22**

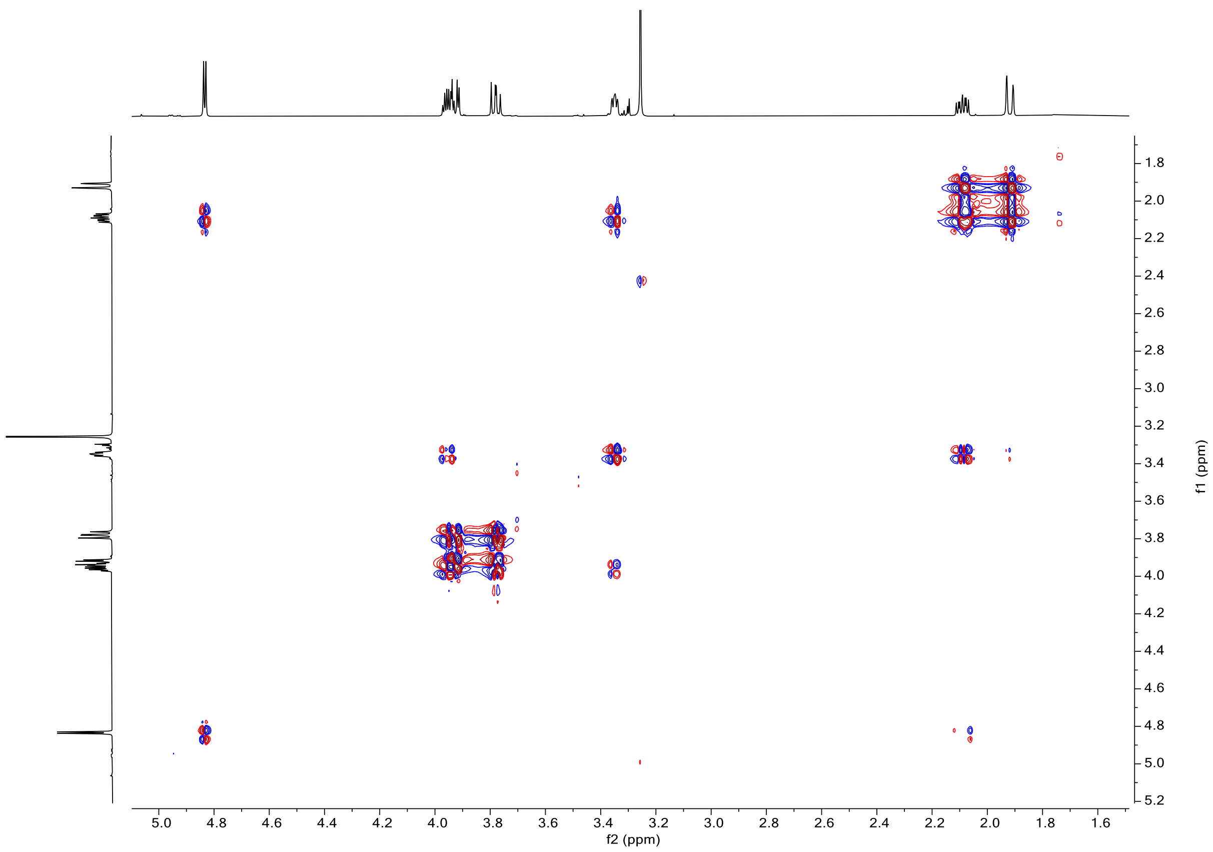

H-H COSY NMR Spectrum of ribose-C3 hydrazide **SF22**

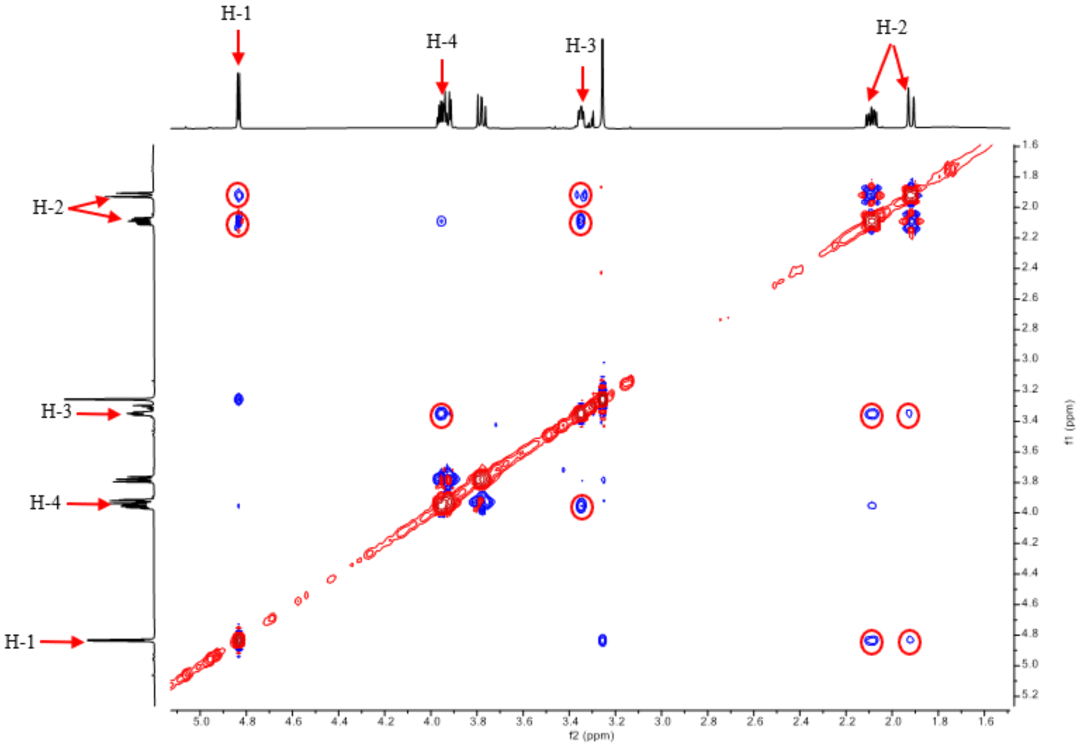

NOESY NMR Spectrum of ribose-C3 hydrazide **SF22**

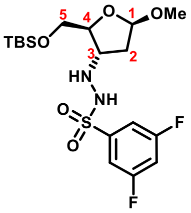

6.4 Characterization Data of C2-C6 Arylated Products

Compound 101

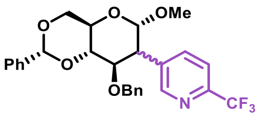

COSY, NOESY and HSQC spectra of both isomers respectively are attached below.

Following **General Procedure H** with glucose C2 hydrazide **99** (0.1 mmol) and 2-trifluoromethyl-5-iodo-pyridine **84**, purification through flash column chromatography afforded product **101** (24 mg, 48% yield, manno:gluco = 1:1.2).

Compound g-101:

$[\alpha]_D^{25}$ = +110° (c = 0.5, $CHCl_3$)

^{1}H NMR (600 MHz, $CDCl_3$) δ 8.60 (d, *J* = 2.11 Hz, 1H), 7.66 (dd, *J* = 8.07, 2.12 Hz, 1H), 7.56 – 7.52 (m, 2H), 7.48 (dd, *J* = 8.09, 0.80 Hz, 1H), 7.43 – 7.37 (m, 3H), 7.21 – 7.18 (m, 1H), 7.16 – 7.11 (m, 2H), 6.84 – 6.81 (m, 2H), 5.68 (s, 1H), 4.74 (d, *J* = 11.63 Hz, 1H), 4.69 (d, *J* = 3.41 Hz, 1H), 4.43 (d, *J* = 11.63 Hz, 1H), 4.35 (dd, *J* = 10.30, 4.85 Hz, 1H), 4.23 (dd, *J* = 10.96, 8.70 Hz, 1H), 4.03 (td, *J* = 9.97, 4.82 Hz, 1H), 3.90 – 3.84 (m, 2H), 3.29 (s, 3H), 3.18 (dd, *J* = 10.99, 3.41 Hz, 1H).

^{13}C NMR (151 MHz, $CDCl_3$) δ 151.5, 146.91 (q, $^2J_{C,F}$ = 34.6 Hz), 138.5, 137.5, 136.1, 129.2, 128.5, 128.4, 128.3, 127.9, 126.1, 121.79 (q, $^1J_{C,F}$ = 274.0 Hz), 119.9, 119.1, 101.7, 100.9, 84.7, 75.1, 74.7, 69.3, 63.0, 55.4, 51.1.

HRMS (m/z): (ESI) calc. for $C_{27}H_{27}F_3NO_5$ $[M+H]^+$: 502.1841 found 502.1847

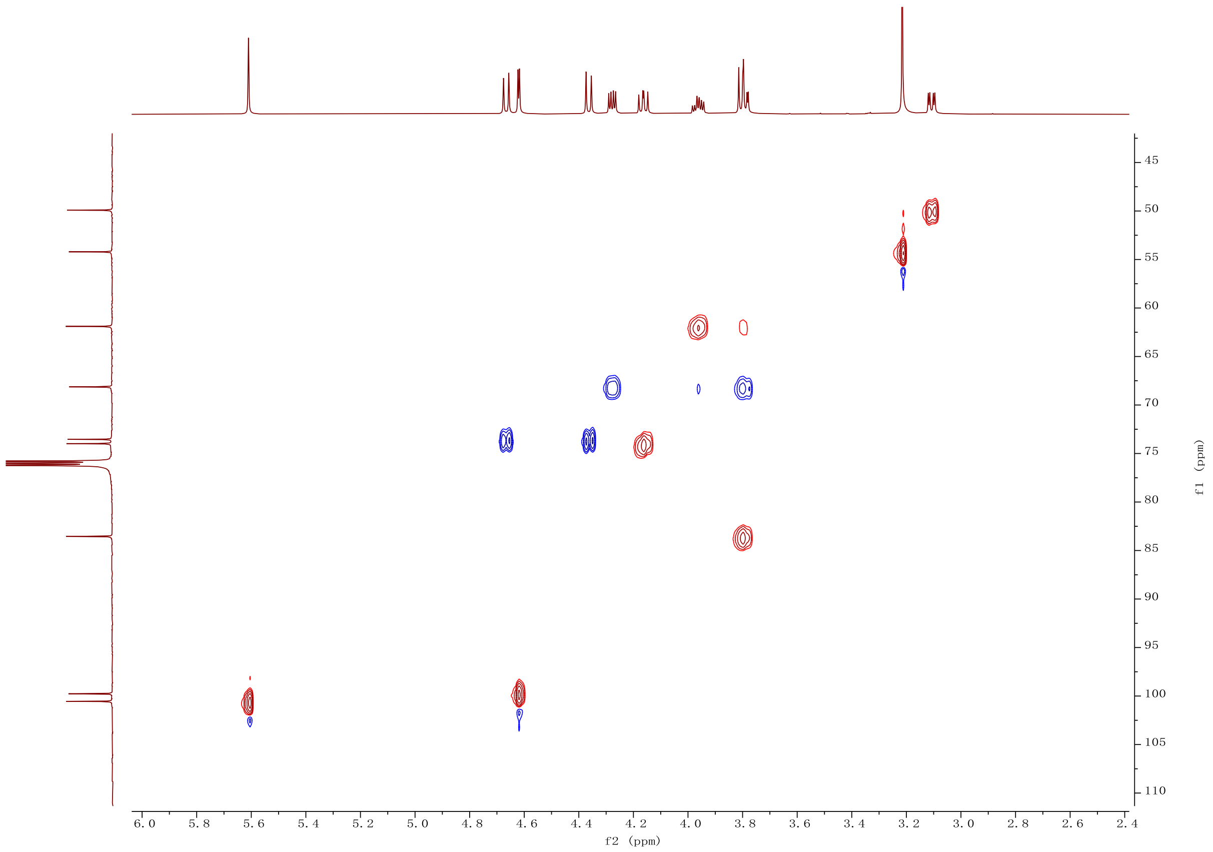

H-C HSQC NMR Spectrum of compound **g-101**

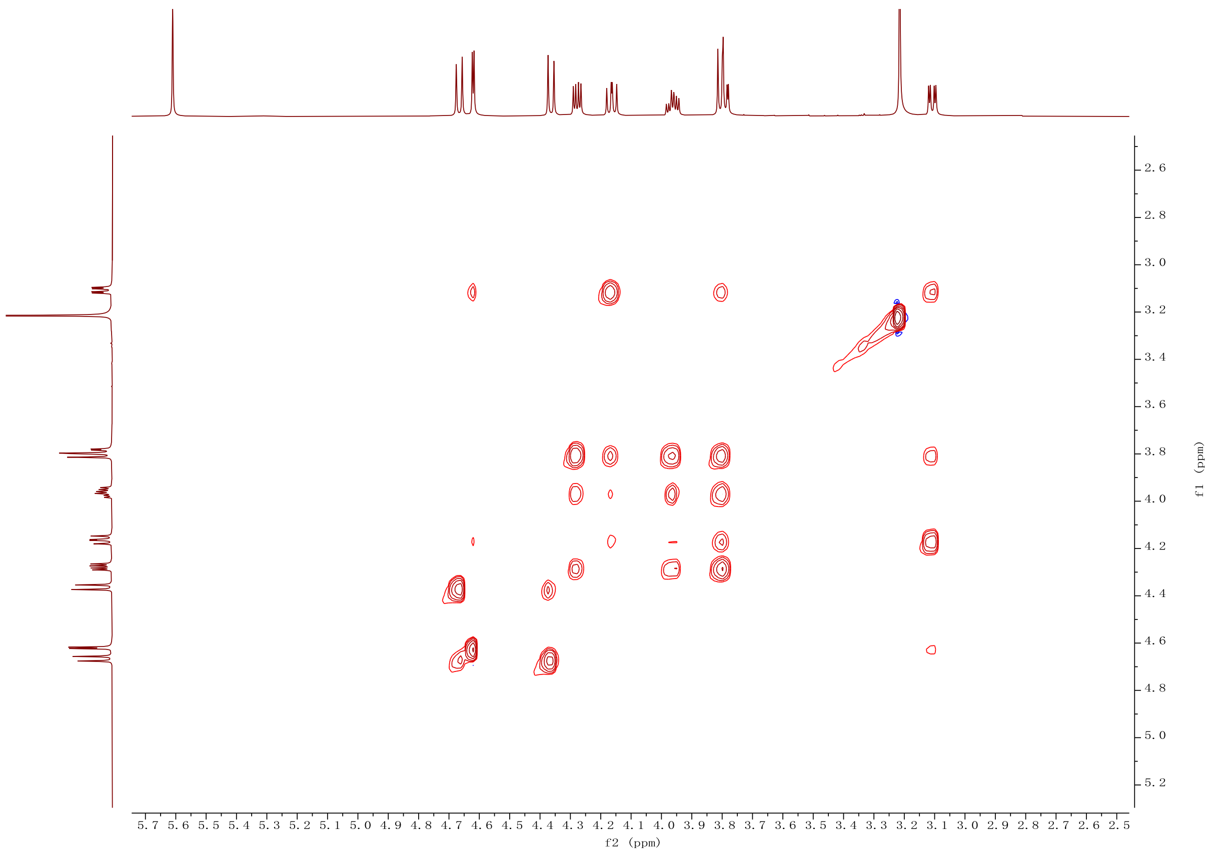

H-H COSY NMR Spectrum of compound **g-101**

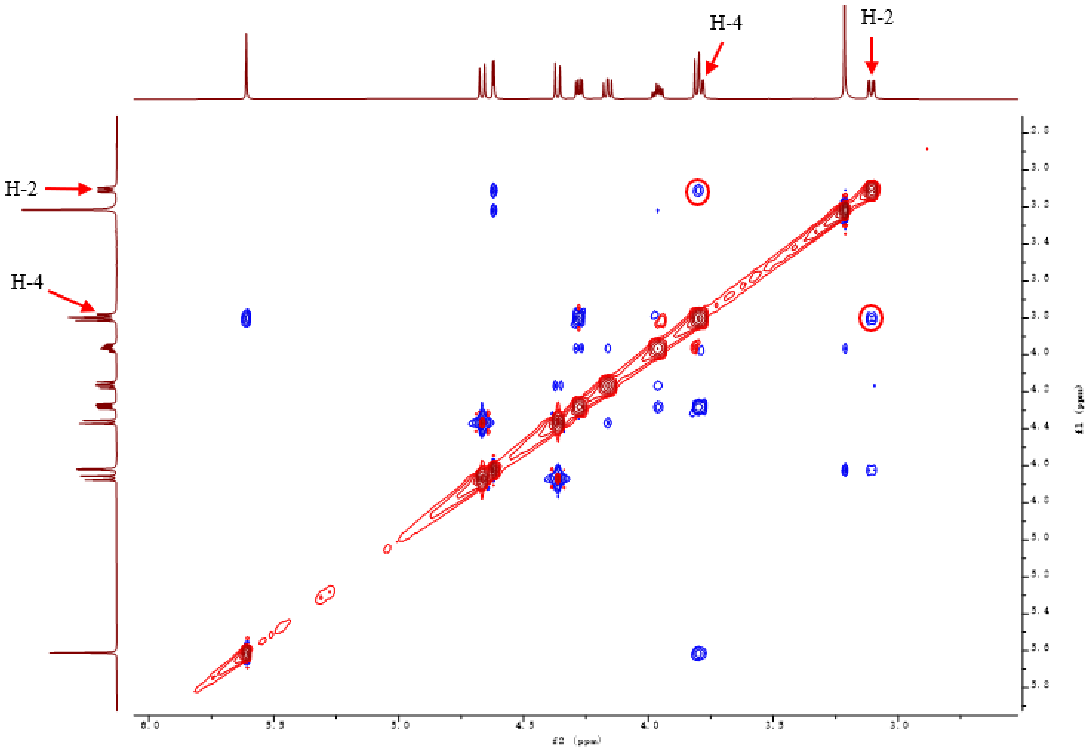

NOESY NMR Spectrum of **g-101**

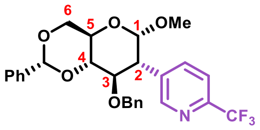

Compound m-101:

$[\alpha]_D^{25}$ = –19.6° (c = 0.5, $CHCl_3$)

^{1}H NMR (600 MHz, $CDCl_3$) δ 8.83 (d, J = 2.1 Hz, 1H), 8.01 (dd, J = 8.1, 2.2 Hz, 1H), 7.65 (dd, J = 8.1, 0.8 Hz, 1H), 7.46 – 7.42 (m, 2H), 7.39 – 7.26 (m, 8H), 5.53 (s, 1H), 4.97 (s, 1H), 4.84 (d, J = 12.2 Hz, 1H), 4.74 (d, J = 12.2 Hz, 1H), 4.36 (dd, J = 10.4, 4.7 Hz, 1H), 4.30 (dd, J = 10.1, 6.2 Hz, 1H), 3.99 (td, J = 10.0, 4.7 Hz, 1H), 3.87 (t, J = 10.4 Hz, 1H), 3.74 (t, J = 9.8 Hz, 1H), 3.60 (d, J = 6.2 Hz, 1H), 3.41 (s, 3H).

^{13}C NMR (151 MHz, $CDCl_3$) δ 151.7, 146.8 (q, $^2J_{C,F}$= 34.7 Hz), 138.7, 138.3, 137.4, 136.4, 129.1, 128.6, 128.4, 127.8, 127.7, 126.1, 121.8 (q, $^1J_{C,F}$ = 273.9 Hz), 119.8, 101.7, 101.4, 79.4, 74.2, 73.9, 69.0, 64.4, 55.1, 47.9.

HRMS (m/z): (ESI) calc. for $C_{27}H_{27}F_3NO_5$ $[M+H]^+$: 502.1841 found 502.1839

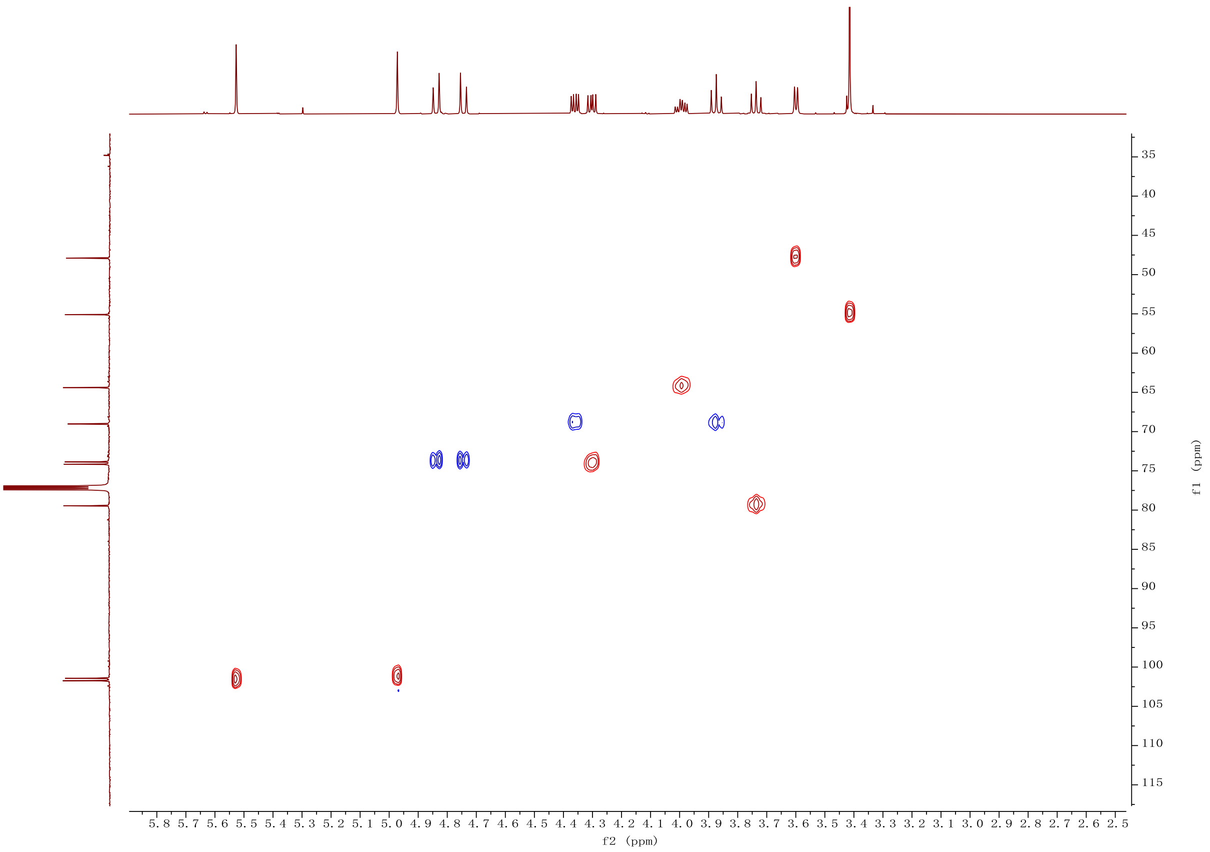

H-C HSQC NMR Spectrum of compound **m-101**

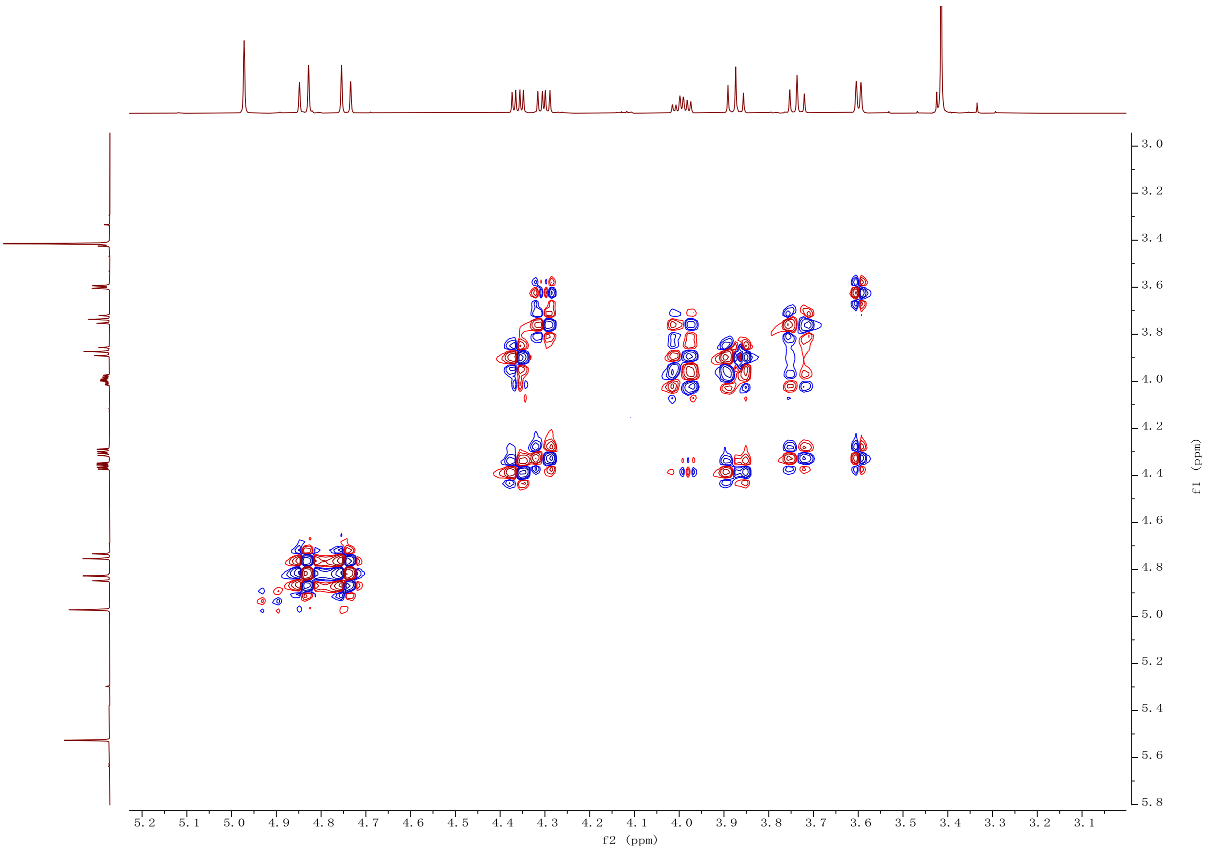

H-H COSY NMR Spectrum of compound **m-101**

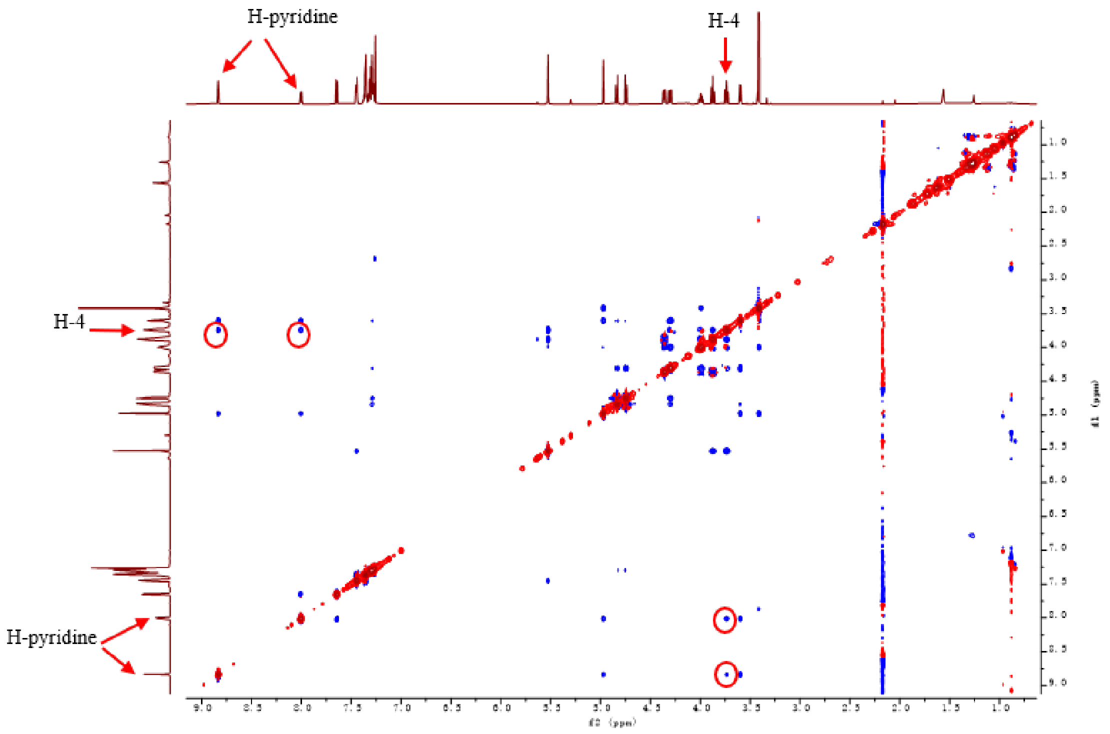

NOESY NMR Spectrum of **m-101**

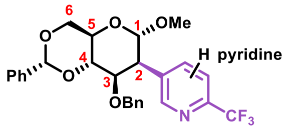

Compound 102

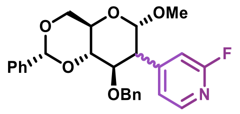

Following **General Procedure H** with glucose C2 hydrazide **99** (0.1 mmol) and 4-bromo-2-fluoropyridine, purification through flash column chromatography afforded product **102** (31.6 mg, 70% yield, manno:gluco = 1:1.5).

Compound g-102:

$[\alpha]_D^{20}$= –22.3° (c = 0.6, $CHCl_3$)

^{1}H NMR (600 MHz, $CDCl_3$) δ 8.09 (d, *J* = 5.1 Hz, 1H), 7.54 (dd, *J* = 8.0, 1.7 Hz, 2H), 7.44 – 7.37 (m, 3H), 7.24 – 7.17 (m, 3H), 7.04 (dt, *J* = 5.3, 1.6 Hz, 1H), 6.93 – 6.91 (m, 2H), 6.79 (s, 1H), 5.67 (s, 1H), 4.76 (d, *J* = 11.4 Hz, 1H), 4.69 (d, *J* = 3.3 Hz, 1H), 4.49 (d, *J* = 11.4 Hz, 1H), 4.34 (dd, *J* = 10.3, 4.8 Hz, 1H), 4.24 (dd, *J* = 10.9, 8.7 Hz, 1H), 4.01 (td, *J* = 10.0, 4.8 Hz, 1H), 3.85 (td, *J* = 9.8, 6.0 Hz, 2H), 3.28 (s, 3H), 3.13 (dd, *J* = 10.9, 3.4 Hz, 1H).

^{13}C NMR (151 MHz, $CDCl_3$) δ 163.9 (d, $^1J_{C,F}$ = 238.0 Hz), 152.1 (d, $^3J_{C,F}$ = 8.0 Hz), 147.1 (d, $^3J_{C,F}$ = 15.0 Hz), 137.5, 129.2, 128.5, 128.39, 128.35, 128.0, 126.1, 123.0 (d, $^4J_{C,F}$ = 4.4 Hz), 110.7 (d, $^2J_{C,F}$ = 37.4 Hz), 101.7, 100.8, 84.7, 74.8, 69.3, 63.0, 55.4.

HRMS (m/z): (ESI) calc. $C_{26}H_{27}FNO_5$ $[M+H]^+$: 452.1873 found 452.1878

Compound m-102:

$[\alpha]_D^{20}$= +144.3° (c = 0.6, $CHCl_3$)

^{1}H NMR (600 MHz, $CDCl_3$) δ 8.15 (d, *J* = 5.2 Hz, 1H), 7.45 (dd, *J* = 7.5, 2.3 Hz, 2H), 7.39 – 7.27 (m, 9H), 7.09 (s, 1H), 5.56 (s, 1H), 4.93 (s, 1H), 4.84 (d, *J* = 12.3 Hz, 1H), 4.74 (d, *J* = 12.3 Hz, 1H), 4.35 (dd, *J* = 10.1, 4.5 Hz, 1H), 4.28 (dd, *J* = 10.1, 6.3 Hz, 1H), 3.99 – 3.94 (m, 1H), 3.90 (t, *J* = 10.3 Hz, 1H), 3.78 (t, *J* = 9.8 Hz, 1H), 3.54 (d, *J* = 6.3 Hz, 1H), 3.40 (s, 3H).

^{13}C NMR (151 MHz, $CDCl_3$) δ 163.9 (d, $^1J_{C,F}$ = 237.8 Hz), 152.2 (d, $^3J_{C,F}$ = 7.8 Hz), 146.9 (d, $^3J_{C,F}$ = 15.0 Hz), 138.3, 137.5, 129.1, 128.5, 128.3, 127.8, 127.5, 126.1, 123.5 (d, $^4J_{C,F}$ = 3.8 Hz), 111.1 (d, $^2J_{C,F}$ = 37.8 Hz), 101.7, 101.2, 79.3, 74.2, 73.7, 69.0, 55.1, 49.4 (d, $^4J_{C,F}$ = 2.7 Hz).

HRMS (m/z): (ESI) calc. $C_{26}H_{27}FNO_5$ $[M+H]^+$: 452.1873 found 452.1883

Compound 103

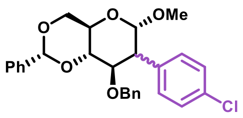

Following **General Procedure H** with glucose C2 hydrazide **99** (0.1 mmol) and 4-bromochlorobenzene, purification through flash column chromatography afforded product **103** (17 mg, 37% yield, manno:gluco = 1:1).

Compound g-103:

$[\boldsymbol{a}]_{\mathbf{D}}^{\mathbf{25}}$= +68.8° (c = 0.5, $CHCl_3$)

^{1}H NMR (600 MHz, $CDCl_3$) δ 7.55 – 7.52 (m, 2H), 7.44 – 7.36 (m, 3H), 7.26 – 7.21 (m, 4H), 7.21 – 7.14 (m, 3H), 6.90 – 6.86 (m, 2H), 5.66 (s, 1H), 4.70 (d, *J* = 11.4 Hz, 1H), 4.67 (d, *J* = 3.4 Hz, 1H), 4.40 (d, *J* = 11.3 Hz, 1H), 4.33 (dd, *J* = 10.2, 4.8 Hz, 1H), 4.22 (dd, *J* = 11.0, 8.7 Hz, 1H), 4.01 (td, *J* = 10.0, 4.8 Hz, 1H), 3.88 – 3.80 (m, 2H), 3.28 (s, 3H), 3.08 (dd, *J* = 11.0, 3.4 Hz, 1H).

^{13}C NMR (151 MHz, $CDCl_3$) δ 138.1, 137.7, 135.5, 133.0, 131.4, 129.1, 128.4, 128.3, 128.3, 128.2, 127.7, 126.2, 101.8, 101.6, 84.8, 76.1, 74.9, 69.4, 63.1, 55.4, 53.2.

HRMS (m/z): (ESI) calc. for $C_{27}H_{28}ClO_5$ $[M+H]^+$: 467.1625 found 467.1637

Compound m-103:

$[\boldsymbol{a}]_{\mathbf{D}}^{\mathbf{25}}$= –47.8° (c = 0.5, $CHCl_3$)

^{1}H NMR (600 MHz, $CDCl_3$) δ 7.47 – 7.41 (m, 4H), 7.38 – 7.33 (m, 3H), 7.31 – 7.29 (m, 6H), 7.28 – 7.23 (m, 1H), 5.54 (s, 1H), 4.93 (s, 1H), 4.82 (d, *J* = 12.4 Hz, 1H), 4.75 (d, *J* = 12.4 Hz, 1H), 4.34 (dd, *J* = 10.0, 4.4 Hz, 1H), 4.22 (dd, *J* = 10.0, 6.2 Hz, 1H), 3.95 (td, *J* = 9.8, 4.4 Hz, 1H), 3.89 (t, *J* = 10.2 Hz, 1H), 3.84 (t, *J* = 9.7 Hz, 1H), 3.52 (d, *J* = 6.2 Hz, 1H), 3.38 (s, 3H).

^{13}C NMR (151 MHz, $CDCl_3$) δ 138.8, 137.7, 135.7, 133.2, 131.6, 129.0, 128.4, 128.3, 128.2, 127.5, 127.5, 126.1, 102.3, 101.6, 79.3, 74.6, 73.2, 69.1, 64.5, 54.9, 49.3.

HRMS (m/z): (ESI) calc. for $C_{27}H_{28}ClO_5$ $[M+H]^+$: 467.1625 found 467.1620.

Compound 104

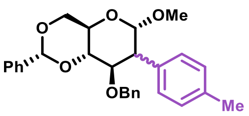

Following **General Procedure H** with glucose C2 hydrazide **99** (0.1 mmol) and 4-bromotoluene, purification through flash column chromatography afforded product **104** (22 mg, 49% yield, manno:gluco = 1:1).

Compound g-104:

$[\boldsymbol{a}]_{\mathbf{D}}^{\mathbf{25}}$= +22.1° (c = 0.5, $CHCl_3$)

^{1}H NMR (600 MHz, $CDCl_3$) δ 7.56 – 7.51 (m, 1H), 7.43 – 7.35 (m, 2H), 7.23 (d, *J* = 8.0 Hz, 1H), 7.19 – 7.11 (m, 3H), 6.88 – 6.85 (m, 1H), 5.66 (s, 1H), 4.70 (d, *J* = 3.4 Hz, 1H), 4.67 (d, *J* = 11.3 Hz, 1H), 4.38 (d, *J* = 11.3 Hz, 1H), 4.33 (dd, *J* = 10.2, 4.8 Hz, 1H), 4.26 (dd, *J* = 11.0, 8.7 Hz, 1H), 4.02 (td, *J* = 10.0, 4.8 Hz, 1H), 3.83 (dt, *J* = 14.2, 9.7 Hz, 1H), 3.28 (s, 2H), 3.09 (dd, *J* = 11.0, 3.4 Hz, 1H), 2.37 (s, 2H).

^{13}C NMR (151 MHz, $CDCl_3$) δ 138.5, 137.8, 136.7, 133.9, 130.0, 129.0, 128.9, 128.4, 128.1, 128.1, 127.5, 126.2, 102.3, 101.6, 84.7, 76.6, 74.9, 69.5, 63.1, 55.3, 53.5, 21.3.

HRMS (m/z): (ESI) calc. for $C_{28}H_{30}NaO_5$ $[M+Na]^+$: 469.1991 found 469.1984

Compound m-104:

$[\boldsymbol{a}]_{\mathbf{D}}^{\mathbf{25}}$= –13.0° (c = 0.5, $CHCl_3$)

^{1}H NMR (600 MHz, $CDCl_3$) δ 7.48 – 7.43 (m, 2H), 7.39 (d, *J* = 8.1 Hz, 2H), 7.37 – 7.32 (m, 5H), 7.32 – 7.27 (m, 2H), 7.26 – 7.23 (m, 1H), 7.17 – 7.14 (m, 2H), 5.54 (s, 1H), 4.95 (s, 1H), 4.79 (q, *J* = 12.6 Hz, 2H), 4.37 – 4.33 (m, 1H), 4.20 (dd, *J* = 9.7, 6.2 Hz, 1H), 3.98 – 3.88 (m, 3H), 3.53 (d, *J* = 6.1 Hz, 1H), 3.38 (s, 3H), 2.34 (s, 3H).

^{13}C NMR (151 MHz, $CDCl_3$) δ 139.0, 137.8, 136.8, 134.2, 130.1, 128.9, 128.9, 128.3, 128.3, 127.4, 127.4, 126.1, 102.8, 101.6, 79.3, 74.8, 72.9, 69.2, 64.5, 54.9, 49.5, 21.2.

HRMS (m/z): (ESI) calc. for $C_{28}H_{30}NaO_5$ $[M+Na]^+$: 469.1991 found 469.1990

Compound 107

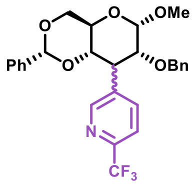

COSY, NOESY and HSQC spectra of both isomers respectively are attached below.

Following **General Procedure H** with glucose C3 hydrazide **105** (0.1 mmol) and 2-trifluoromethyl-5-iodo-pyridine **84**, purification through flash column chromatography afforded product **115** (31 mg, 62% yield, gluco:allo = 1:1.1).

Compound a-107:

$[\boldsymbol{a}]_{\mathbf{D}}^{\mathbf{25}}$= +7.2° (c = 0.5, $CHCl_3$)

^{1}H NMR (600 MHz, $CDCl_3$) δ 8.92 (d, J = 2.2 Hz, 1H), 8.45 (dd, J = 8.2, 2.2 Hz, 1H), 7.61 (d, J = 8.2 Hz, 1H), 7.36 – 7.29 (m, 6H), 7.28 – 7.25 (m, 4H), 5.52 (s, 1H), 4.80 (dd, J = 2.9, 1.4 Hz, 1H), 4.57 (d, J = 4.1 Hz, 2H), 4.29 (dd, J = 10.6, 5.1 Hz, 1H), 4.04 – 4.01 (m, 2H), 3.93 (td, J = 9.9, 5.1 Hz, 1H), 3.89 – 3.85 (m, 1H), 3.67 (t, J = 10.2 Hz, 1H), 3.47 (s, 3H).

^{13}C NMR (151 MHz, $CDCl_3$) δ 153.3, 146.0 (q, $^2J_{C,F}$ = 34.4 Hz), 140.1, 137.1, 137.0, 134.4, 129.3, 128.8, 128.5, 128.4, 128.1, 126.1, 122.0 (q, $^1J_{C,F}$ = 273.8 Hz), 119.3, 101.9, 98.0, 74.3, 71.6, 69.6, 59.0, 55.7, 43.9.

HRMS (m/z): (ESI) calc. for $C_{27}H_{27}F_3NO_5$ $[M+H]^+$: 502.1841 found 502.1833

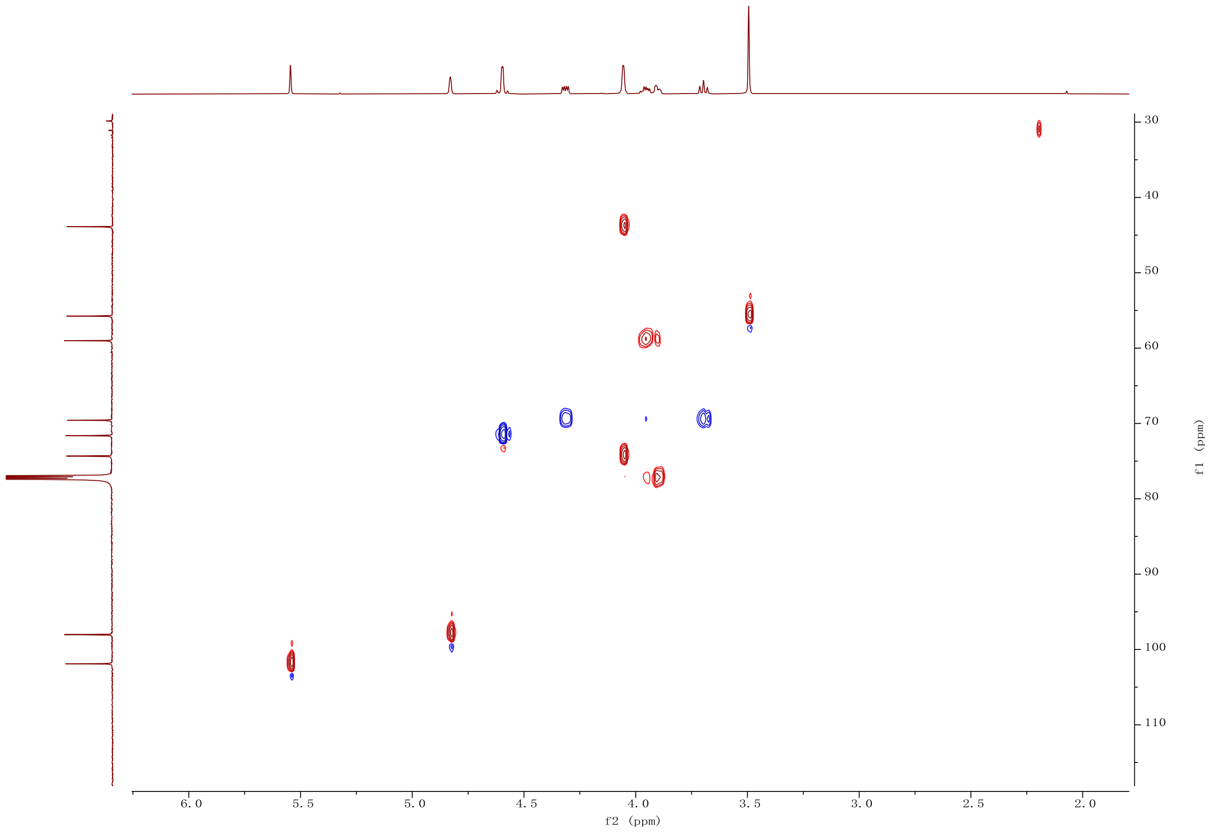

H-C HSQC NMR Spectrum of compound **a-107**

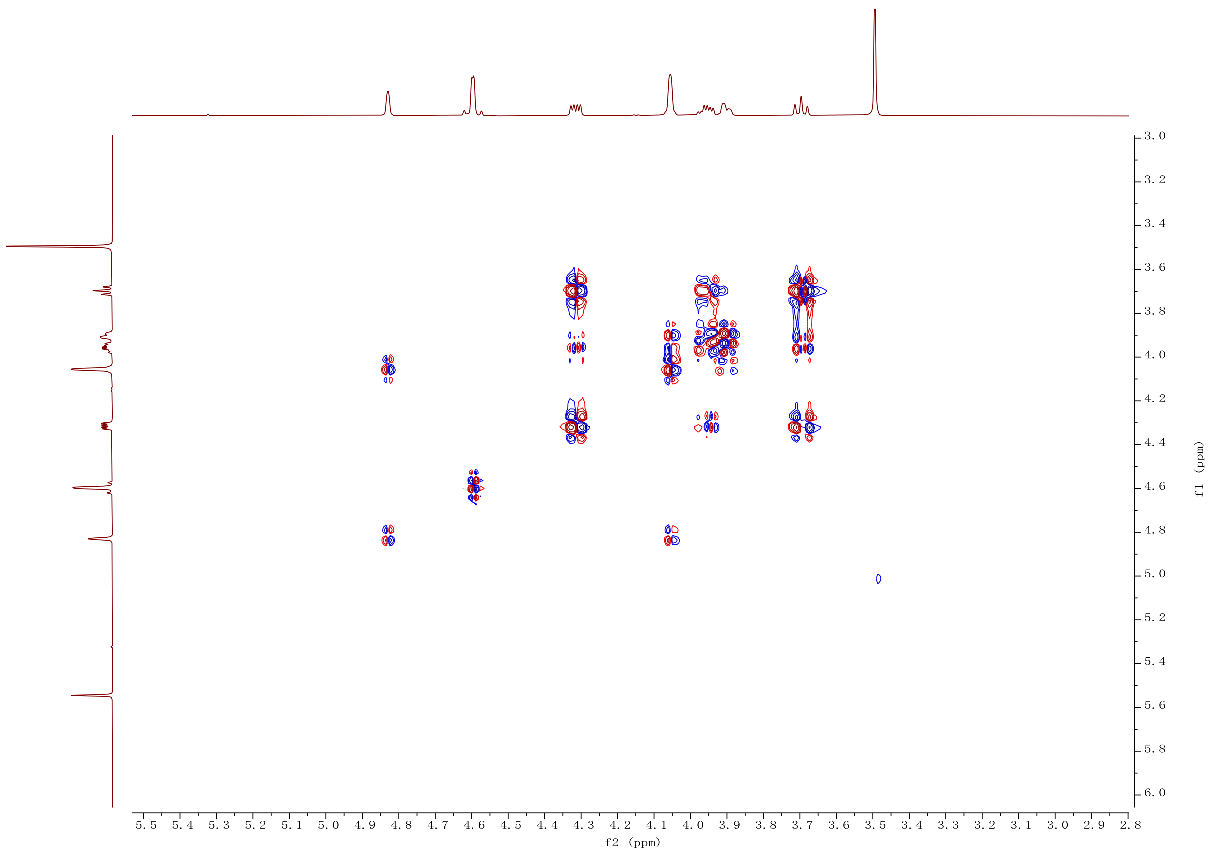

H-H COSY NMR Spectrum of compound **a-107**

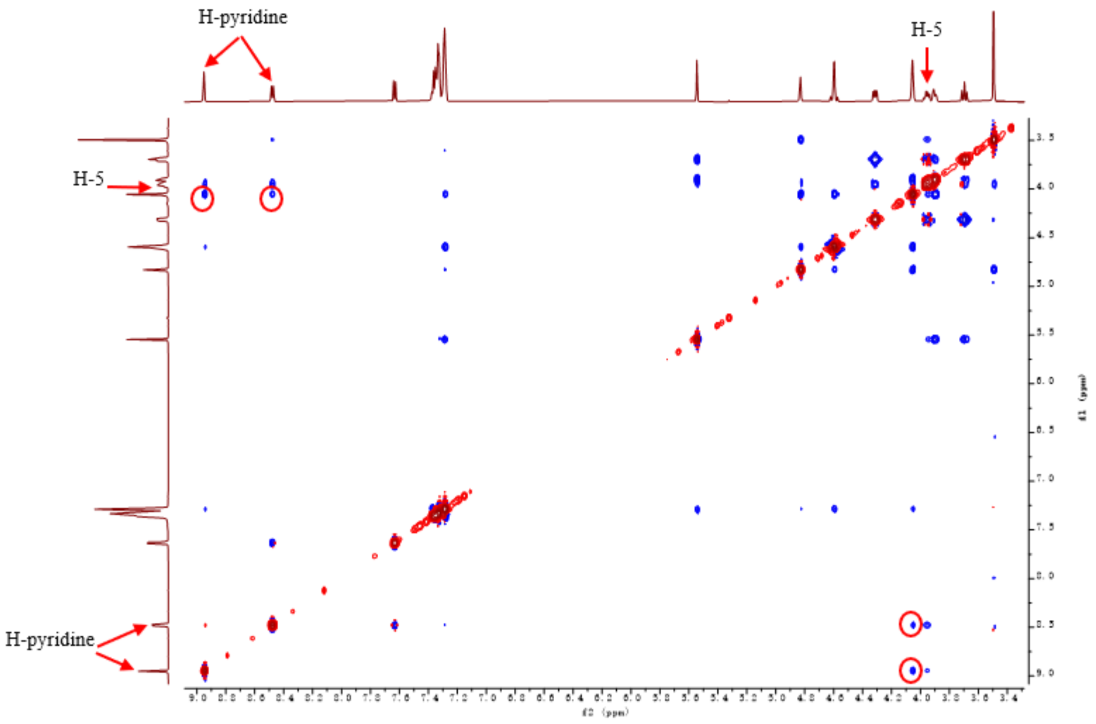

NOESY NMR Spectrum of **a-107**

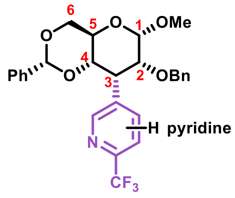

Compound g-107:

$[\alpha]_D^{25}$= –2.0° (c = 0.5, $CHCl_3$)

^{1}H NMR (600 MHz, $CDCl_3$) δ 8.62 (s, 1H), 7.63 (dd, *J* = 8.1, 2.2 Hz, 1H), 7.55 (d, *J* = 8.1 Hz, 1H), 7.28 (s, 5H), 7.26 – 7.19 (m, 3H), 6.94 (d, *J* = 6.7 Hz, 2H), 5.41 (s, 1H), 4.82 (d, *J* = 3.3 Hz, 1H), 4.40 (d, *J* = 12.3 Hz, 1H), 4.34 – 4.30 (m, 2H), 3.98 (ddd, *J* = 10.4, 9.1, 4.9 Hz, 1H), 3.78 – 3.70 (m, 2H), 3.65 (dd, *J* = 10.9, 3.4 Hz, 1H), 3.53 – 3.48 (m, 4H).

^{13}C NMR (151 MHz, $CDCl_3$) δ 150.4, 146.8 (q, $^2J_{C,F}$ = 34.6 Hz), 137.3, 136.9, 136.89, 136.84, 129.2, 128.6, 128.3, 128.2, 126.1, 121.8 (q, $^1J_{C,F}$ = 274.3 Hz), 120.1 (q, $^3J_{C,F}$ = 2.8 Hz), 101.7, 97.4, 79.3, 72.5, 69.3, 63.7, 55.6, 45.2.

HRMS (m/z): (ESI) calc. for $C_{27}H_{27}F_3NO_5$ $[M+H]^+$: 502.1841 found 502.1833

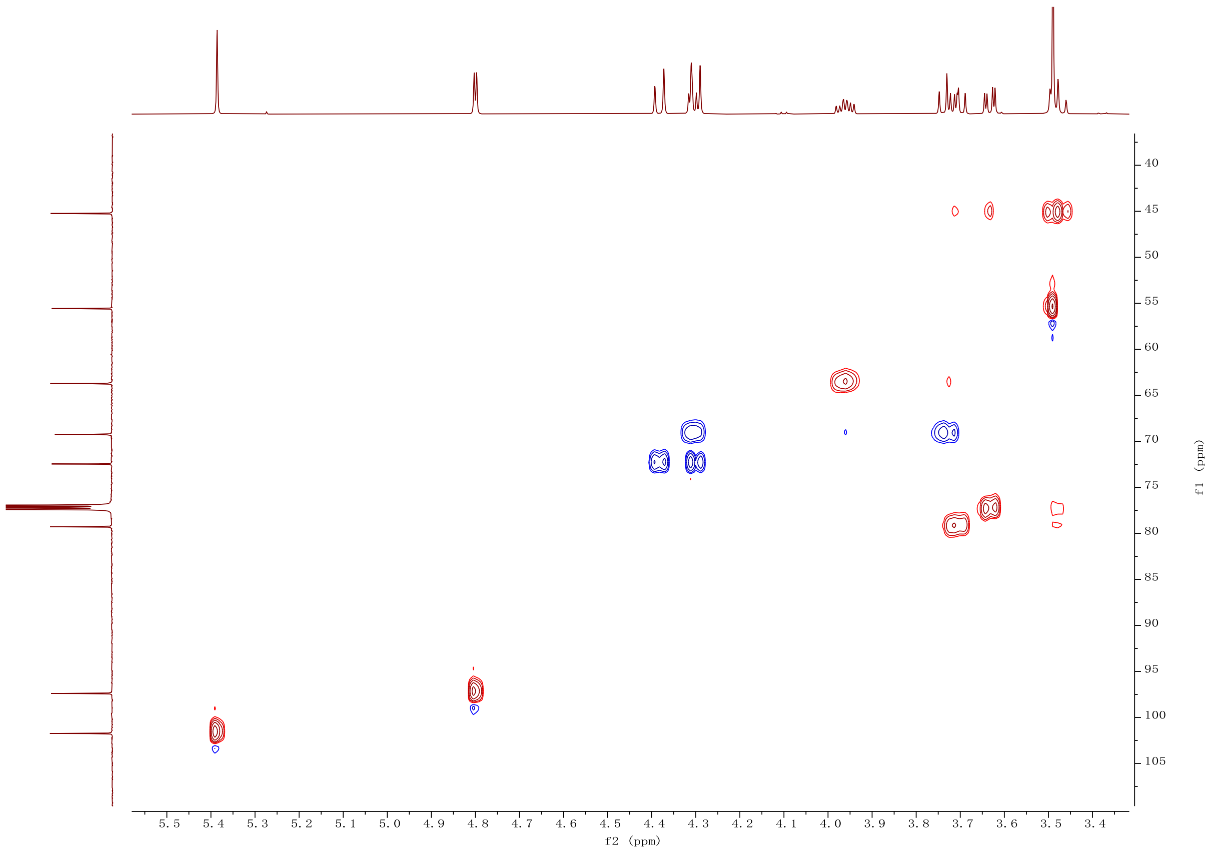

H-C HSQC NMR Spectrum of compound **g-107**

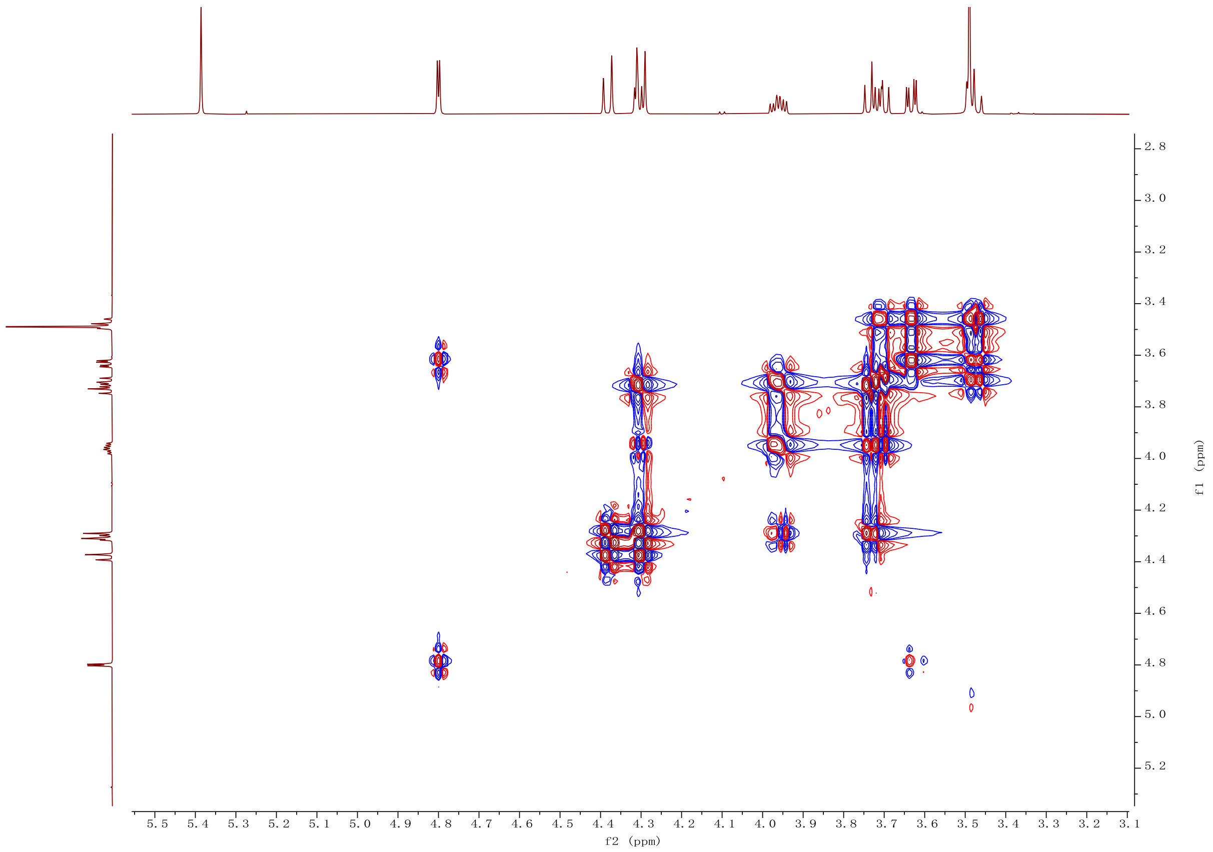

H-H COSY NMR Spectrum of compound **g-107**

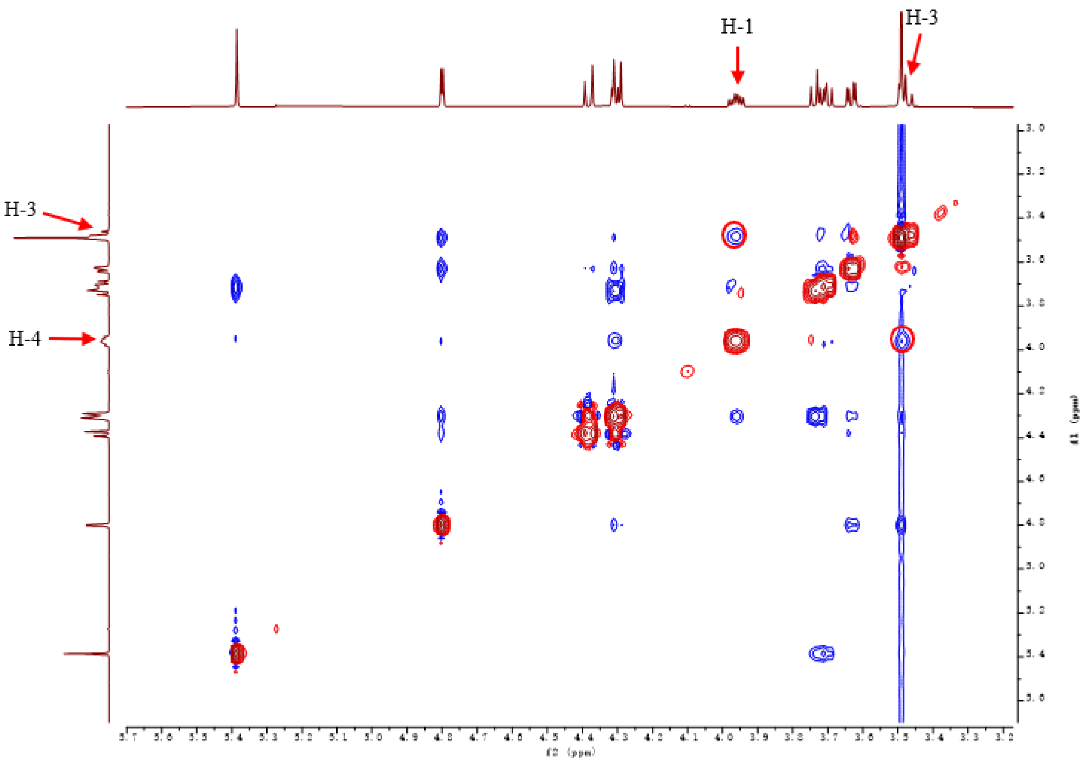

NOESY NMR Spectrum of **g-107**

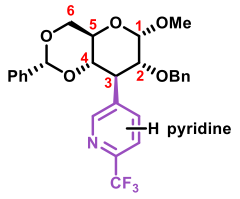

Compound 108

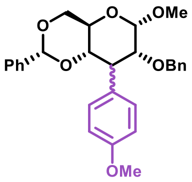

Following **General Procedure H** with glucose C3 hydrazide **105** (0.1 mmol) and *p*-iodoanisole, purification through flash column chromatography afforded product **108** (25 mg, 55% yield, gluco:allo = 1.1:1).

Compound a-108:

$[\alpha]_D^{25}$ = +7.6° (c = 0.5, $CHCl_3$)

^{1}H NMR (600 MHz, $CDCl_3$) δ 7.68 (d, *J* = 8.3 Hz, 2H), 7.36 – 7.27 (m, 10H), 6.86 (d, *J* = 8.7 Hz, 2H), 5.50 (s, 1H), 4.76 (d, *J* = 4.0 Hz, 1H), 4.61 (d, *J* = 12.5 Hz, 1H), 4.50 (d, *J* = 12.6 Hz, 1H), 4.27 (dd, *J* = 10.6, 5.3 Hz, 1H), 4.06 (td, *J* = 9.9, 5.2 Hz, 1H), 3.99 – 3.92 (m, 2H), 3.82 (s, 3H), 3.76 (dd, *J* = 10.0, 5.8 Hz, 1H), 3.63 (t, *J* = 10.2 Hz, 1H), 3.48 (s, 3H).

^{13}C NMR (151 MHz, $CDCl_3$) δ 158.3, 137.7, 137.6, 132.8, 129.1, 128.6, 128.3, 128.13, 128.08, 126.9, 126.4, 112.9, 101.7, 98.5, 78.0, 74.3, 70.7, 69.7, 58.9, 55.6, 55.3, 45.3.

HRMS (m/z): (ESI) calc. for $C_{28}H_{30}NaO_6$ $[M+Na]^+$: 485.1940 found 485.1984

Compound g-108:

$[\alpha]_D^{25}$ = –10.1° (c = 0.5, $CHCl_3$)

^{1}H NMR (600 MHz, $CDCl_3$) δ 7.32 – 7.30 (m, 2H), 7.28 – 7.26 (m, 3H), 7.24 – 7.20 (m, 5H), 7.03 – 6.99 (m, 2H), 6.89 – 6.85 (m, 2H), 5.41 (s, 1H), 4.68 (d, *J* = 3.4 Hz, 1H), 4.32 (d, *J* = 12.3 Hz, 1H), 4.29 (dd, *J* = 10.3, 4.9 Hz, 1H), 4.15 (d, *J* = 12.3 Hz, 1H), 3.95 (ddd, *J* = 10.3, 9.1, 4.8 Hz, 1H), 3.82 (s, 3H), 3.72 (t, *J* = 10.4 Hz, 1H), 3.67 (dd, *J* = 10.9, 9.2 Hz, 1H), 3.60 (dd, *J* = 10.9, 3.5 Hz, 1H), 3.48 (s, 3H), 3.35 (t, *J* = 10.9 Hz, 1H).

^{13}C NMR (151 MHz, $CDCl_3$) δ 158.7, 137.9, 137.5, 129.8, 129.5, 128.9, 128.4, 128.2, 128.1, 127.9, 126.1, 113.8, 101.5, 98.4, 80.2, 78.7, 72.8, 69.4, 63.9, 55.44, 55.37, 46.9.

HRMS (m/z): (ESI) calc. for $C_{28}H_{30}NaO_6$ $[M+Na]^+$: 485.1940 found 485.1941

Compound 109

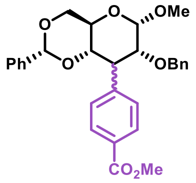

Following **General Procedure H** with glucose C3 hydrazide **105** (0.1 mmol) and 4-iodobenzoate, purification through flash column chromatography afforded product **109** (36 mg, 74% yield, gluco:allo = 1:1.1).

Compound a-109:

$[\alpha]_D^{25}$ = +11.2° (c = 0.25, $CHCl_3$)

^{1}H NMR (600 MHz, $CDCl_3$) δ 7.97 (d, *J* = 8.5 Hz, 2H), 7.85 (d, *J* = 8.3 Hz, 2H), 7.35 – 7.23 (m, 10H), 5.50 (s, 1H), 4.77 (d, *J* = 4.1 Hz, 1H), 4.60 (d, *J* = 12.5 Hz, 1H), 4.52 (d, *J* = 12.5 Hz, 1H), 4.28 (dd, *J* = 10.5, 5.3 Hz, 1H), 4.10 – 4.02 (m, 2H), 3.98 (dd, *J* = 6.6, 4.2 Hz, 1H), 3.92 (s, 3H), 3.83 (dd, *J* = 9.9, 6.1 Hz, 1H), 3.65 (t, *J* = 10.3 Hz, 1H), 3.47 (s, 3H).

^{13}C NMR (151 MHz, $CDCl_3$) δ 167.6, 140.7, 137.4, 137.4, 131.8, 129.2, 128.7, 128.4, 128.3, 128.2, 128.1, 126.3, 101.8, 98.3, 77.8, 74.4, 71.1, 69.7, 59.0, 55.6, 52.1, 46.1.

HRMS (m/z): (ESI) calc. for $C_{29}H_{31}O_7$ $[M+H]^+$: 491.2070 found 491.2069

Compound g-109:

$[\alpha]_D^{25}$ = –10.8° (c = 0.5, $CHCl_3$)

^{1}H NMR (600 MHz, $CDCl_3$) δ 8.00 (d, *J* = 8.3 Hz, 2H), 7.36 (d, *J* = 8.4 Hz, 2H), 7.28 – 7.19 (m, 8H), 6.96 (dd, *J* = 7.8, 1.7 Hz, 2H), 5.40 (s, 1H), 4.73 (d, *J* = 3.4 Hz, 1H), 4.33 – 4.31 (m, 1H), 4.31 – 4.29 (m, 1H), 4.19 (d, *J* = 12.3 Hz, 1H), 3.96 (ddd, *J* = 10.3, 9.1, 4.9 Hz, 1H), 3.93 (s, 3H), 3.76 – 3.71 (m, 2H), 3.67 (dd, *J* = 10.9, 3.4 Hz, 1H), 3.49 (s, 3H), 3.47 (d, *J* = 10.9 Hz, 1H).

^{13}C NMR (151 MHz, $CDCl_3$) δ 167.3, 143.5, 137.4, 137.2, 129.7, 129.0, 129.0, 128.7, 128.5, 128.2, 128.1, 128.1, 126.0, 101.5, 98.0, 80.0, 78.1, 72.7, 69.4, 63.8, 55.5, 52.2, 47.8.

HRMS (m/z): (ESI) calc. for $C_{29}H_{30}NaO_7$ $[M+Na]^+$: 513.1889 found 513.1876

Compound 110

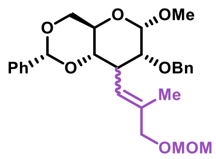

Following **General Procedure H** with glucose C3 hydrazide **105** (0.1 mmol) and vinyl iodide, purification through flash column chromatography afforded product **110** (18 mg, 38% yield, gluco:allo = 1.7:1).

Compound a-110:

$[\alpha]_D^{20}$= –10.5° (c = 0.2, $CHCl_3$)

^{1}H NMR (600 MHz, $CDCl_3$) δ 7.42 – 7.39 (m, 2H), 7.37 – 7.29 (m, 8H), 6.03 (dd, *J* = 10.0, 1.9 Hz, 1H), 5.47 (s, 1H), 4.70 (d, *J* = 3.7 Hz, 1H), 4.67 – 4.63 (m, 2H), 4.55 – 4.49 (m, 2H), 4.26 (dd, *J* = 10.3, 5.1 Hz, 1H), 4.09 – 4.01 (m, 2H), 3.96 (td, *J* = 9.9, 5.1 Hz, 1H), 3.66 – 3.58 (m, 3H), 3.55 (dt, *J* = 10.2, 5.1 Hz, 1H), 3.45 (s, 3H), 3.35 (s, 3H), 1.71 (d, *J* = 1.5 Hz, 3H).

^{13}C NMR (151 MHz, $CDCl_3$) δ 137.69, 137.66, 136.8, 128.9, 128.5, 128.2, 128.0, 126.2, 123.5, 101.3, 99.2, 94.5, 78.6, 74.6, 73.4, 70.7, 69.5, 59.1, 55.7, 55.3, 38.8, 14.6.

HRMS (m/z): (ESI) calc. for $C_{27}H_{34}NaO_7$ $[M+Na]^+$: 493.2202 found 493.2206

Compound g-110:

$[\alpha]_D^{20}$= –3.7° (c = 0.6, $CHCl_3$)

^{1}H NMR (600 MHz, $CDCl_3$) δ 7.44 – 7.41 (m, 2H), 7.36 – 7.27 (m, 8H), 5.43 (s, 1H), 5.22 (dq, *J* = 9.1, 1.3 Hz, 1H), 4.65 (d, *J* = 3.4 Hz, 1H), 4.63 – 4.56 (m, 4H), 4.24 (dd, *J* = 10.3, 4.9 Hz, 1H), 4.00 (s, 2H), 3.84 (ddd, *J* = 10.4, 9.0, 4.8 Hz, 1H), 3.65 (t, *J* = 10.3 Hz, 1H), 3.44 (s, 3H), 3.37 (dd, *J* = 10.3, 3.4 Hz, 1H), 3.35 (s, 3H), 3.26 (dd, *J* = 10.5, 9.0 Hz, 1H), 3.20 (td, *J* = 10.4, 9.1 Hz, 1H), 1.73 (d, *J* = 1.4 Hz, 3H).

^{13}C NMR (151 MHz, $CDCl_3$) δ 138.2, 137.7, 137.3, 128.9, 128.5, 128.3, 128.2, 128.0, 126.1, 125.1, 101.6, 97.9, 94.8, 80.4, 78.3, 72.7, 72.6, 69.4, 63.4, 55.4, 55.3, 40.8, 15.3.

HRMS (m/z): (ESI) calc. $C_{27}H_{35}O_7$ $[M+H]^+$: 471.2383 found 471.2362

Compound 113

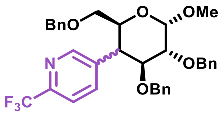

COSY, NOESY and HSQC spectra of both isomers respectively are attached below.

Following **General Procedure H** with glucose C4 hydrazide **111** (0.1 mmol) and 2-trifluoromethyl-5-iodo-pyridine **84**, purification through flash column chromatography afforded product **113** (31 mg, 52% yield, galacto:gluco = 3.1:1).

Compound g-113:

$[\alpha]_D^{25}$= –7.0° (c = 0.2, $CHCl_3$)

^{1}H NMR (600 MHz, $CDCl_3$) δ 8.48 (d, *J* = 2.2 Hz, 1H), 7.45 – 7.28 (m, 8H), 7.28 – 7.22 (m, 2H), 7.17 – 7.13 (m, 3H), 7.10 (t, *J* = 7.4 Hz, 2H), 6.77 (d, *J* = 6.9 Hz, 2H), 4.83 (d, *J* = 12.0 Hz, 1H), 4.78 (d, *J* = 3.4 Hz, 1H), 4.71 (d, *J* = 3.2 Hz, 2H), 4.69 (d, *J* = 2.7 Hz, 1H), 4.46 (d, *J* = 12.0 Hz, 1H), 4.28 (d, *J* = 12.0 Hz, 1H), 4.19 (d, *J* = 11.4 Hz, 1H), 4.04 (dd, *J* = 10.8, 9.1 Hz, 1H), 3.99 (ddd, *J* = 11.1, 3.9, 2.2 Hz, 1H), 3.69 (dd, *J* = 9.1, 3.5 Hz, 1H), 3.46 (s, 3H), 3.35 (dd, *J* = 10.8, 2.2 Hz, 1H), 3.13 (t, *J* = 10.9 Hz, 1H), 3.06 (dd, *J* = 10.8, 3.9 Hz, 1H).

^{13}C NMR (151 MHz, $CDCl_3$) δ 150.6, 146.9, 146.7, 138.1, 137.8, 137.6, 137.5, 136.5, 128.7, 128.5, 128.4, 128.3, 128.2, 128.2, 127.9, 127.9, 127.8, 122.6, 120.8, 120.2, 98.9, 81.1, 78.8, 75.3, 73.6, 73.4, 70.9, 69.3, 55.8, 47.7.

HRMS (m/z): (ESI) calc. for $C_{34}H_{35}F_3NO_5$ $[M+H]^+$: 594.2467 found 594.2463

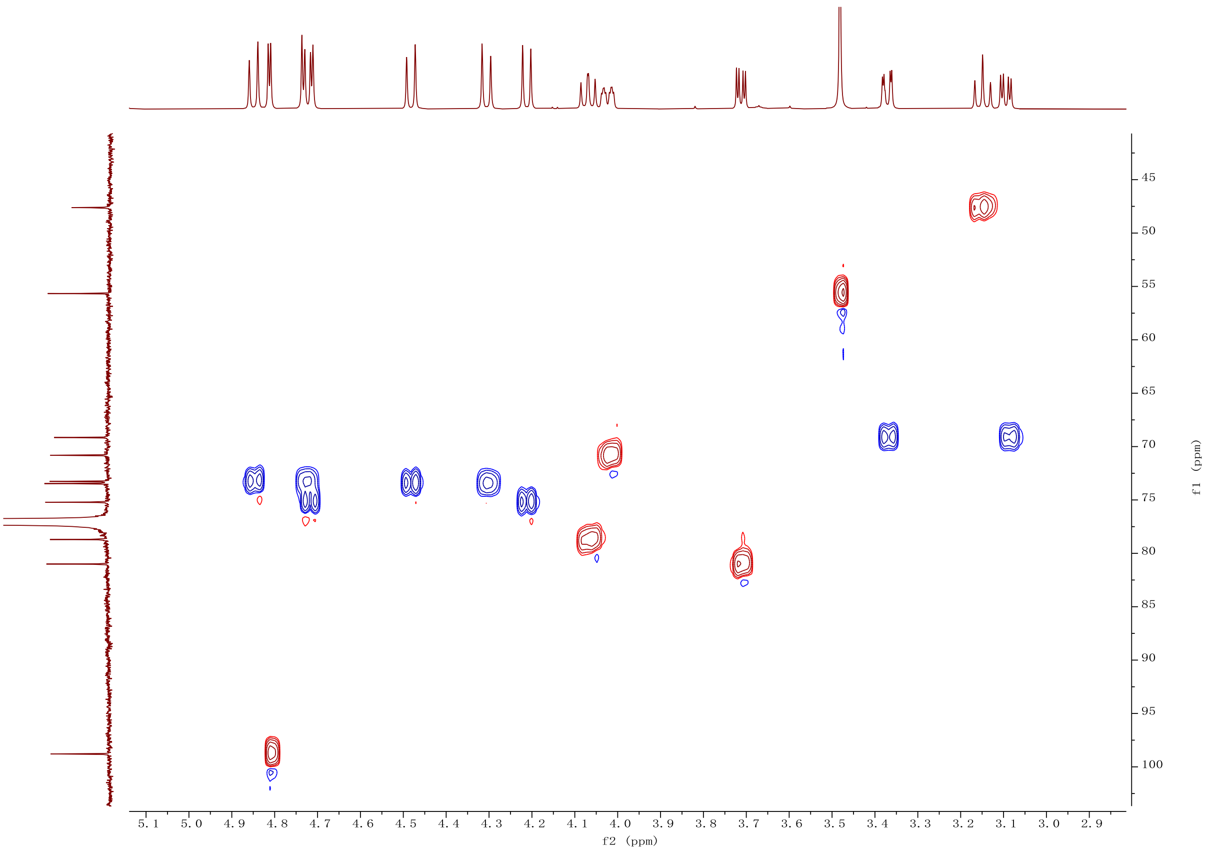

H-C HSQC NMR Spectrum of compound **g-113**

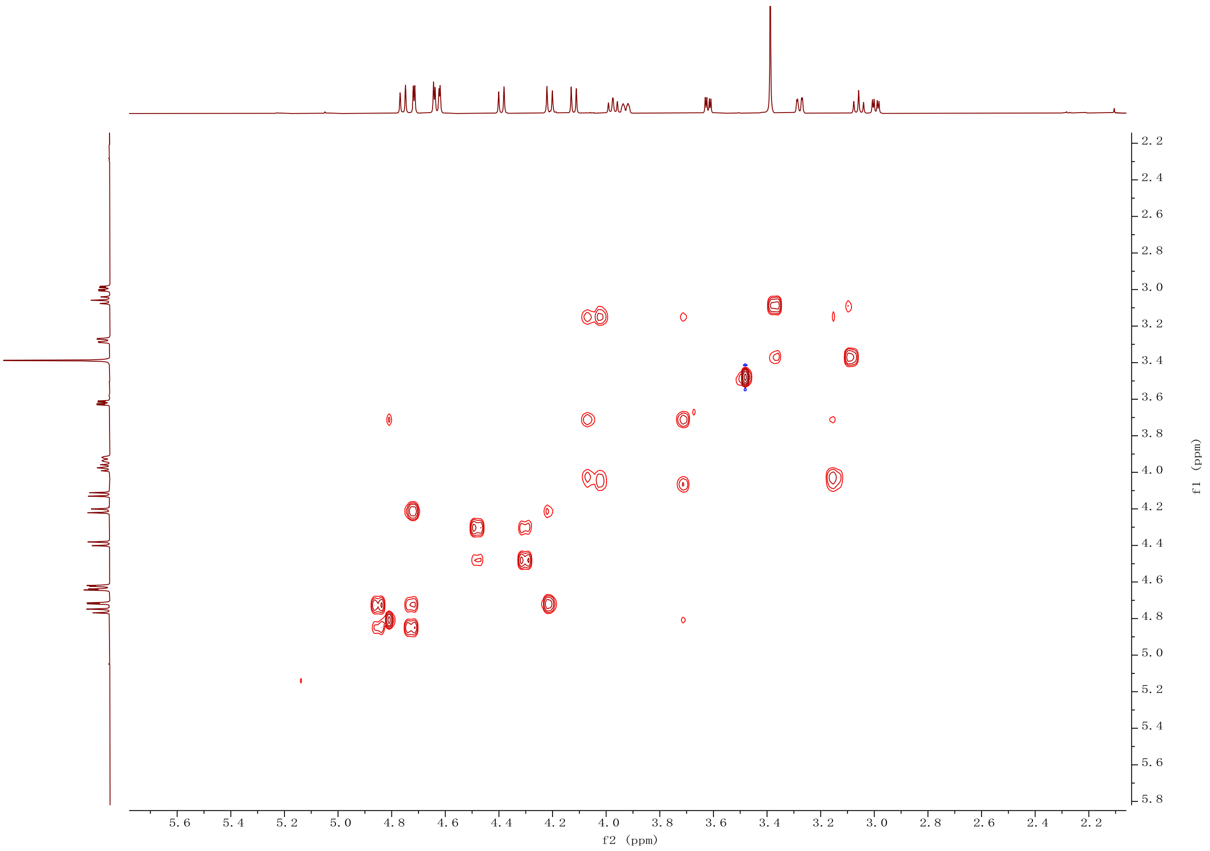

H-H COSY NMR Spectrum of compound **g-113**

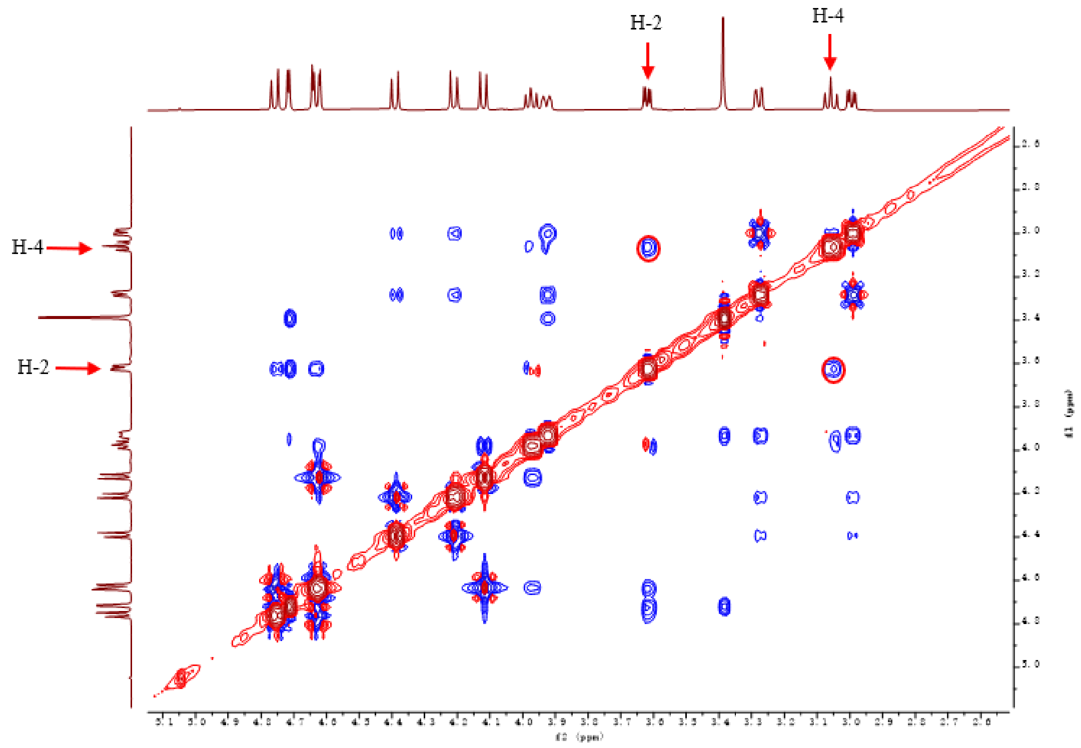

NOESY NMR Spectrum of **g-113**

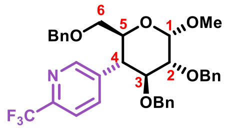

Compound ga-113:

$[\alpha]_D^{25}$= +9.6° (c = 0.5, $CHCl_3$)

^{1}H NMR (600 MHz, $CDCl_3$) δ 8.69 (d, *J* = 2.0 Hz, 1H), 7.91 (dd, *J* = 8.1, 2.1 Hz, 1H), 7.55 (d, *J* = 8.1 Hz, 1H), 7.35 – 7.23 (m, 13H), 7.14 – 7.09 (m, 2H), 4.80 (d, *J* = 3.8 Hz, 1H), 4.77 – 4.71 (m, 3H), 4.54 (d, *J* = 11.9 Hz, 1H), 4.44 (d, *J* = 12.0 Hz, 1H), 4.40 (td, *J* = 6.7, 2.7 Hz, 1H), 4.27 – 4.20 (m, 2H), 3.67 (dd, *J* = 10.2, 3.8 Hz, 1H), 3.46 – 3.40 (m, 5H), 3.16 (dd, *J* = 9.6, 6.8 Hz, 1H).

^{13}C NMR (151 MHz, $CDCl_3$) δ 152.3, 146.7 (q, $^2J_{C,F}$ = 34.8 Hz), 139.1, 138.2, 137.5, 136.1, 128.6, 128.5, 128.5, 128.2, 128.0, 127.9, 127.9, 127.7, 121.8 (q, $^1J_{C,F}$ = 273.7 Hz), 119.7, 99.0, 76.7, 73.8, 73.5, 73.5, 69.5, 68.2, 55.6, 45.6.

HRMS (m/z): (ESI) calc. for $C_{34}H_{35}F_3NO_5$ $[M+H]^+$: 594.2467 found 594.2467

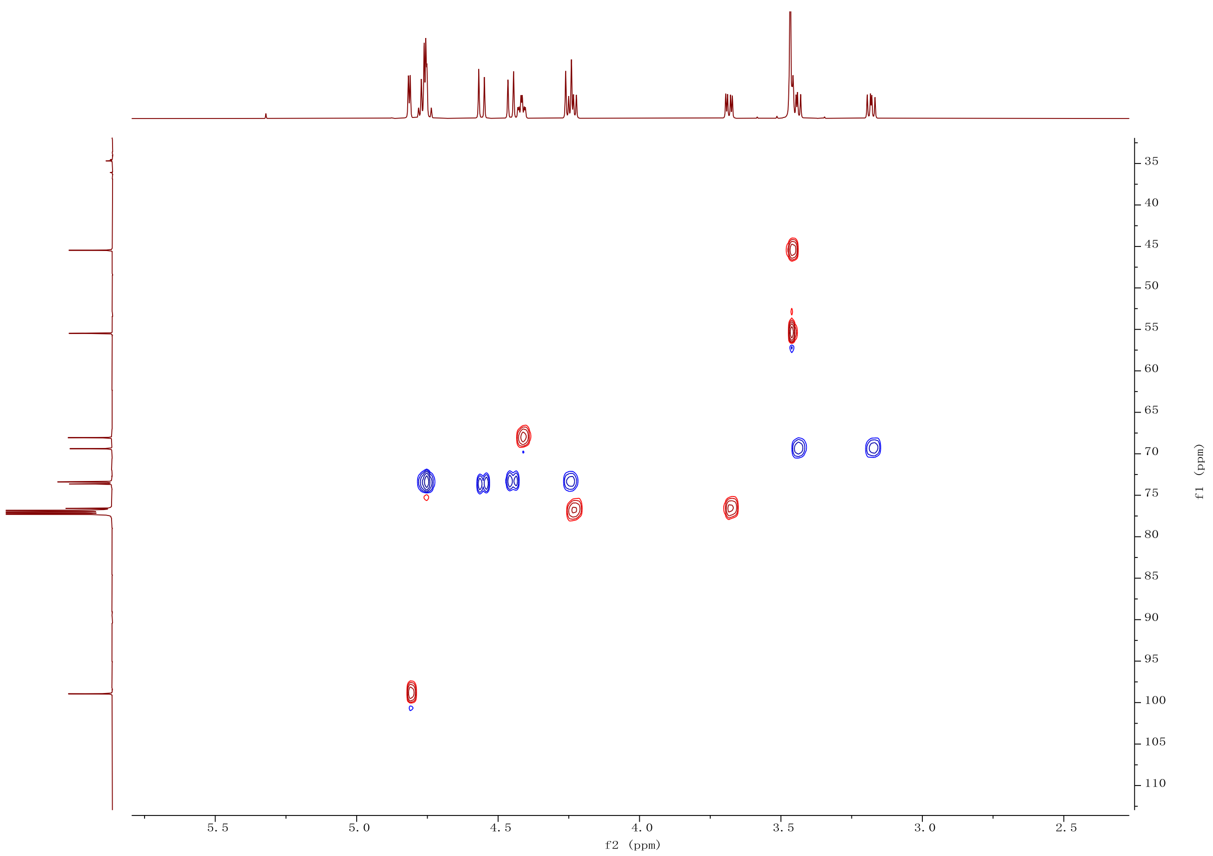

H-C HSQC NMR Spectrum of compound **ga-113**

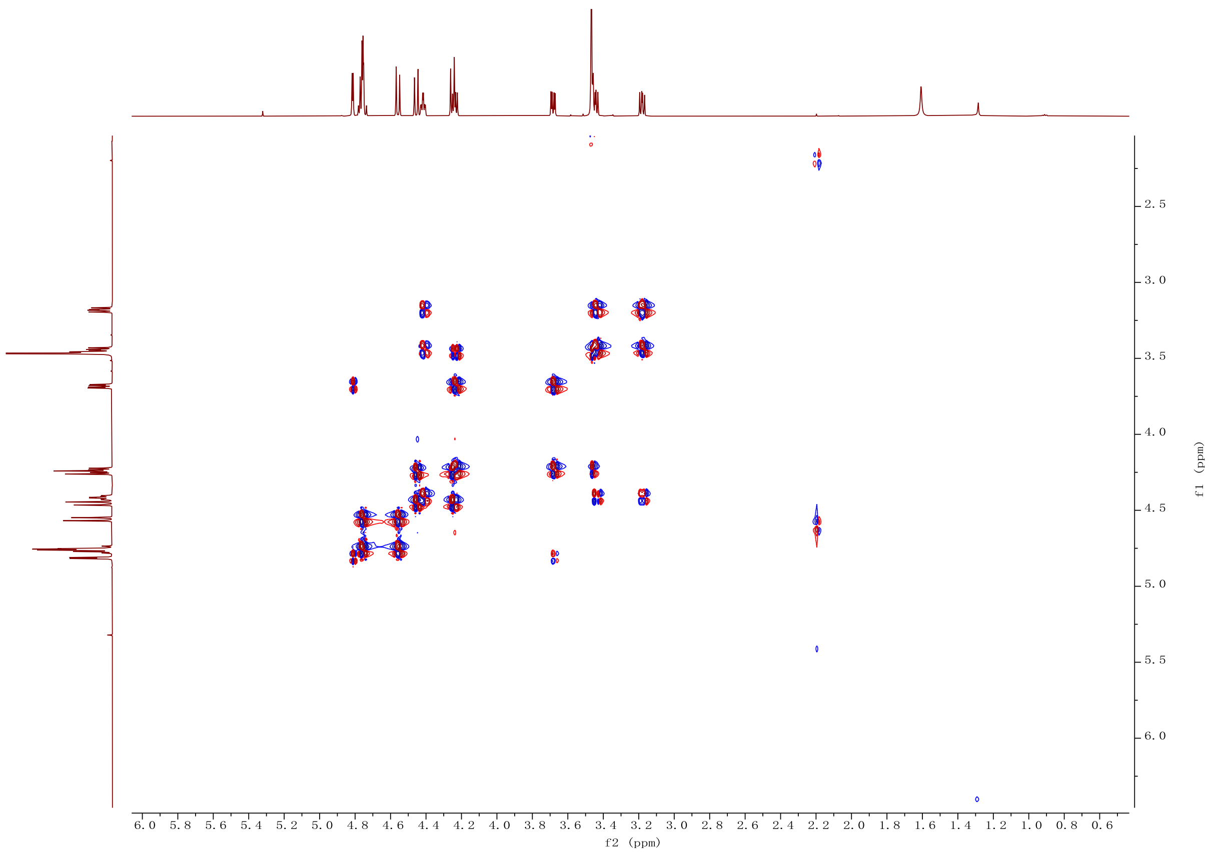

H-H COSY NMR Spectrum of compound **ga-113**

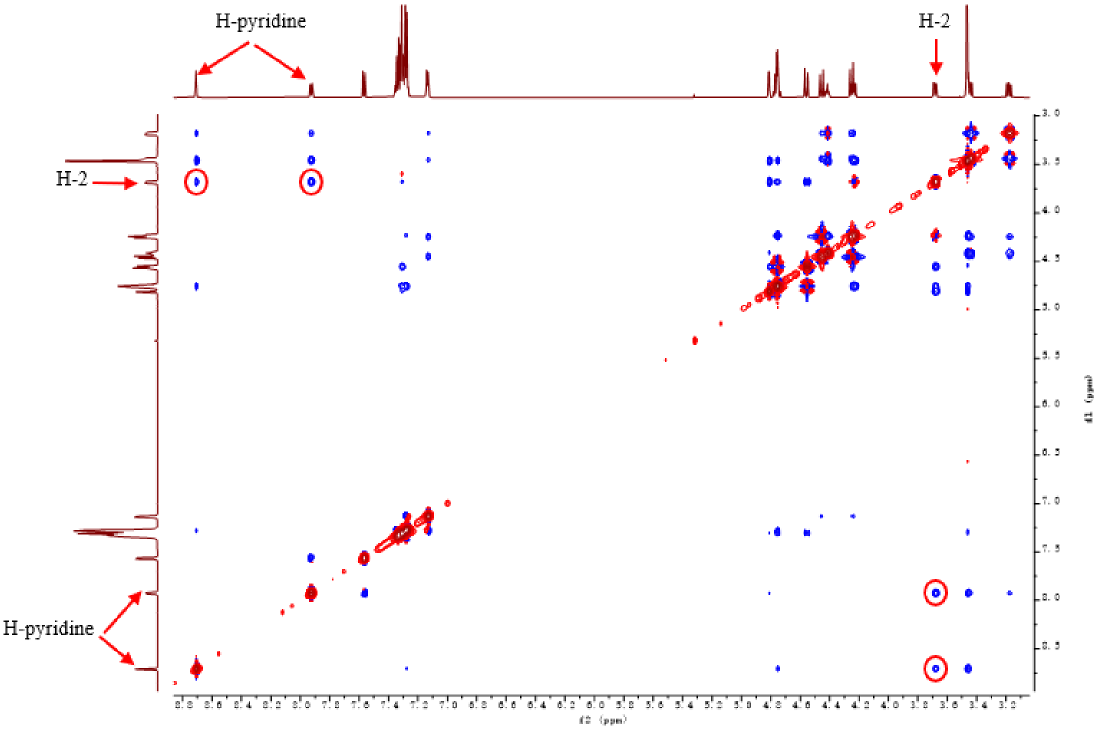

NOESY NMR Spectrum of **ga-113**

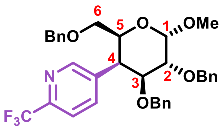

Compound 114

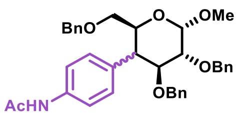

Following **General Procedure H** with glucose C4 hydrazide **111** (0.1 mmol) and 4'-iodoacetanilide, purification through flash column chromatography afforded product **114** (34 mg, 58% yield, galacto:gluco = 3.5:1).

Compound g-114:

$[\alpha]_D^{25}$ = –10.4° (c = 0.25, $CHCl_3$)

^{1}H NMR (600 MHz, $CDCl_3$) δ 7.43 (d, *J* = 8.1 Hz, 2H), 7.39 – 7.35 (m, 2H), 7.34 – 7.26 (m, 6H), 7.25 – 7.11 (m, 10H), 6.88 (dd, *J* = 6.5, 2.9 Hz, 2H), 4.82 (d, *J* = 12.1 Hz, 1H), 4.77 (d, *J* = 3.5 Hz, 1H), 4.68 (d, *J* = 12.1 Hz, 1H), 4.59 (d, *J* = 10.5 Hz, 1H), 4.44 (d, *J* = 12.0 Hz, 1H), 4.34 (d, *J* = 12.0 Hz, 1H), 4.09 (dd, *J* = 10.7, 9.3 Hz, 1H), 4.04 – 4.00 (m, 2H), 3.64 (dd, *J* = 9.3, 3.5 Hz, 1H), 3.46 (s, 3H), 3.38 (dd, *J* = 10.7, 2.1 Hz, 1H), 3.19 (dd, *J* = 10.7, 4.6 Hz, 1H), 3.00 (t, *J* = 10.9 Hz, 1H), 2.20 (s, 3H).

^{13}C NMR (151 MHz, $CDCl_3$) δ 168.2, 138.5, 138.4, 138.2, 136.9, 134.9, 128.6, 128.4, 128.3, 128.2, 128.2, 128.0, 127.7, 127.6, 127.5, 119.9, 99.0, 80.8, 80.7, 75.7, 73.5, 73.4, 71.7, 69.9, 55.6, 50.0, 24.9.

HRMS (m/z): (ESI) calc. for $C_{36}H_{40}NO_6$ $[M+H]^+$: 582.2856 found 582.2860

Compound ga-114:

$[\alpha]_D^{25}$ = +52.0° (c = 0.25, $CHCl_3$)

^{1}H NMR (600 MHz, $CDCl_3$) δ 7.42 (d, *J* = 8.7 Hz, 2H), 7.35 (d, *J* = 8.6 Hz, 2H), 7.31 – 7.26 (m, 10H), 7.26 – 7.23 (m, 2H), 7.21 – 7.19 (m, 2H), 7.17 (s, 1H), 4.81 (d, *J* = 3.8 Hz, 1H), 4.77 – 4.72 (m, 2H), 4.68 (d, *J* = 11.8 Hz, 1H), 4.54 (d, *J* = 11.9 Hz, 1H), 4.43 (d, *J* = 11.9 Hz, 1H), 4.33 (ddd, *J* = 7.6, 5.2, 2.9 Hz, 1H), 4.27 (d, *J* = 11.9 Hz, 1H), 4.16 (dd, *J* = 10.2, 6.1 Hz, 1H), 3.84 (dd, *J* = 10.2, 3.9 Hz, 1H), 3.45 – 3.40 (m, 4H), 3.32 (dd, *J* = 6.1, 2.9 Hz, 1H), 3.23 (dd, *J* = 10.0, 5.3 Hz, 1H), 2.18 (s, 3H).

^{13}C NMR (151 MHz, $CDCl_3$) δ 168.3, 138.8, 138.7, 138.2, 136.8, 132.7, 131.5, 128.44, 128.39, 128.2, 127.82, 127.79, 127.7, 127.6, 119.4, 99.2, 77.7, 76.6, 73.8, 73.4, 72.6, 71.0, 69.2, 55.3, 47.4, 24.8.

HRMS (m/z): (ESI) calc. for $C_{36}H_{40}NO_6$ $[M+H]^+$: 582.2856 found 582.2864

Compound 115

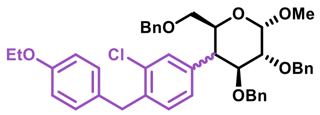

Following **General Procedure H** with glucose C4 hydrazide **111** (0.1 mmol) and aryl bromide, purification through flash column chromatography afforded product **115** (40 mg, 57% yield, galacto:gluco = 3.6:1).

Compound g-115:

$[\alpha]_D^{25}$ = –16.0° (c = 0.2, $CHCl_3$)

^{1}H NMR (600 MHz, $CDCl_3$) δ 7.37 – 7.35 (m, 2H), 7.34 – 7.30 (m, 2H), 7.30 – 7.27 (m, 3H), 7.25 (d, *J* = 8.1 Hz, 2H), 7.18 (d, *J* = 7.1 Hz, 3H), 7.14 (tt, *J* = 6.9, 1.7 Hz, 2H), 7.04 (d, *J* = 2.2 Hz, 1H), 7.01 – 6.97 (m, 3H), 6.82 (d, *J* = 6.8 Hz, 2H), 6.69 (d, *J* = 8.7 Hz, 2H), 4.80 (d, *J* = 12.0 Hz, 1H), 4.75 (d, *J* = 3.5 Hz, 1H), 4.66 (d, *J* = 12.0 Hz, 1H), 4.59 (d, *J* = 10.8 Hz, 1H), 4.37 (d, *J* = 12.0 Hz, 1H), 4.29 (d, *J* = 12.0 Hz, 1H), 4.06 – 3.99 (m, 2H), 3.98 – 3.90 (m, 5H), 3.62 (dd, *J* = 9.3, 3.5 Hz, 1H), 3.44 (s, 3H), 3.35 (dd, *J* = 10.7, 2.1 Hz, 1H), 3.13 (dd, *J* = 10.7, 4.4 Hz, 1H), 2.97 (t, *J* = 10.9 Hz, 1H), 1.36 (t, *J* = 7.0 Hz, 3H).

^{13}C NMR (151 MHz, $CDCl_3$) δ 157.5, 139.4, 138.40, 138.36, 138.1, 137.8, 132.8, 131.5, 130.1, 129.8, 129.7, 128.6, 128.5, 128.4, 128.2, 128.0, 127.8, 127.6, 127.5, 114.6, 99.0, 80.9, 80.4, 75.7, 73.5, 73.4, 71.6, 69.9, 63.4, 55.6, 50.0, 38.4, 15.0.

HRMS (m/z): (ESI) calc. for $C_{43}H_{45}ClNaO_6$ $[M+Na]^+$: 715.2802 found 715.2809

Compound ga-115:

$[\alpha]_D^{25}$ = +44.4° (c = 0.25, $CHCl_3$)

^{1}H NMR (600 MHz, $CDCl_3$) δ 7.32 – 7.27 (m, 10H), 7.27 – 7.25 (m, 2H), 7.25 – 7.21 (m, 3H), 7.18 – 7.14 (m, 2H), 7.11 (d, *J* = 2.2 Hz, 1H), 7.03 (d, *J* = 8.6 Hz, 2H), 6.75 (d, *J* = 8.6 Hz, 2H), 4.74 (dd, *J* = 7.9, 4.0 Hz, 2H), 4.69 – 4.62 (m, 2H), 4.52 (d, *J* = 11.9 Hz, 1H), 4.42 (d, *J* = 12.0 Hz, 1H), 4.30 (ddd, *J* = 6.9, 5.7, 2.8 Hz, 1H), 4.25 (d, *J* = 11.9 Hz, 1H), 4.13 (dd, *J* = 10.2, 6.1 Hz, 1H), 3.99 – 3.93 (m, 4H), 3.74 (dd, *J* = 10.2, 3.9 Hz, 1H), 3.42 (s, 3H), 3.39 (dd, *J* = 9.9, 7.0 Hz, 1H), 3.27 (dd, *J* = 6.1, 2.9 Hz, 1H), 3.19 (dd, *J* = 9.9, 5.8 Hz, 1H), 1.38 (t, *J* = 7.0 Hz, 3H).

^{13}C NMR (151 MHz, $CDCl_3$) δ 157.5, 138.8, 138.6, 138.5, 138.1, 135.5, 133.8, 132.8,

131.5, 130.1, 129.7, 129.1, 128.5, 128.4, 128.2, 127.8, 127.8, 127.7, 127.6, 127.5, 114.5, 99.1, 76.5, 73.8, 73.4, 72.6, 70.6, 68.9, 63.5, 55.4, 47.3, 38.5, 15.0.

HRMS (m/z): (ESI) calc. for $C_{43}H_{45}ClNaO_6$ $[M+Na]^+$: 715.2802 found 715.2801

Compound 116

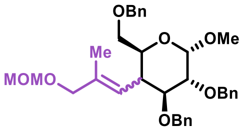

Following **General Procedure H** with glucose C4 hydrazide **111** (0.1 mmol) and vinyl iodide, purification through flash column chromatography afforded product **116** (28 mg, 49% yield, galacto:gluco = 2.5:1).

Compound g-116:

$[\alpha]_D^{25}$ = +10° (c = 0.25, $CHCl_3$)

^{1}H NMR (600 MHz, $CDCl_3$) δ 7.37 – 7.35 (m, 2H), 7.33 – 7.26 (m, 11H), 7.25 (d, *J* = 6.8 Hz, 2H), 5.18 (dd, *J* = 10.1, 1.4 Hz, 1H), 4.80 (dd, *J* = 11.4, 7.3 Hz, 2H), 4.70 (d, *J* = 3.5 Hz, 1H), 4.65 (d, *J* = 12.1 Hz, 1H), 4.57 – 4.48 (m, 5H), 3.93 – 3.85 (m, 2H), 3.74 – 3.71 (m, 1H), 3.69 (t, *J* = 9.7 Hz, 1H), 3.58 (dd, *J* = 9.4, 3.5 Hz, 1H), 3.51 (dd, *J* = 10.6, 2.2 Hz, 1H), 3.43 (dd, *J* = 10.6, 5.0 Hz, 1H), 3.40 (s, 3H), 3.34 (s, 3H), 2.79 (q, *J* = 10.3 Hz, 1H), 1.66 (d, *J* = 1.4 Hz, 3H).

^{13}C NMR (151 MHz, $CDCl_3$) δ 139.0, 138.6, 138.3, 136.7, 128.5, 128.39, 128.35, 128.22, 128.20, 127.9, 127.7, 127.64, 127.62, 124.3, 98.9, 95.4, 80.5, 80.0, 75.7, 73.6, 73.4, 72.6, 70.7, 70.6, 55.38, 55.36, 43.6, 15.2.

HRMS (m/z): (ESI) calc. for $C_{34}H_{42}NaO_7$ $[M+Na]^+$: 585.2828 found 585.2827

Compound ga-116:

$[\alpha]_D^{25}$ = +26.8° (c = 0.25, $CHCl_3$)

^{1}H NMR (600 MHz, $CDCl_3$) δ 7.37 – 7.24 (m, 15H), 5.53 (dd, *J* = 11.0, 1.4 Hz, 1H), 4.81 (d, *J* = 12.1 Hz, 1H), 4.69 (d, *J* = 3.9 Hz, 1H), 4.65 (d, *J* = 2.8 Hz, 2H), 4.63 (d, *J* = 12.1 Hz, 1H), 4.59 (d, *J* = 6.4 Hz, 1H), 4.54 (d, *J* = 6.4 Hz, 1H), 4.52 (d, *J* = 11.9 Hz, 1H), 4.44 (d, *J* = 11.9 Hz, 1H), 4.12 (td, *J* = 6.2, 2.2 Hz, 1H), 4.02 (dd, *J* = 10.1, 5.3 Hz, 1H), 3.98 (s, 2H), 3.71 (dd, *J* = 10.1, 3.9 Hz, 1H), 3.44 (dd, *J* = 9.8, 6.8 Hz, 1H), 3.40 (s, 3H), 3.36 – 3.31 (m, 4H), 3.14 (ddd, *J* = 11.0, 5.3, 2.3 Hz, 1H), 1.67 (d, *J* = 1.5 Hz, 3H).

^{13}C NMR (151 MHz, $CDCl_3$) δ 138.9, 138.8, 138.2, 136.8, 128.51, 128.46, 128.4, 128.1, 127.8, 127.79, 127.77, 127.6, 121.5, 99.1, 95.0, 78.2, 76.8, 73.61, 73.59, 73.1, 72.5,

71.0, 68.7, 55.4, 55.3, 40.6, 14.8.

HRMS (m/z): (ESI) calc. for $C_{34}H_{42}NaO_7$ $[M+Na]^+$: 585.2828 found 585.2809.

Compound 119

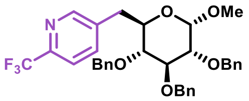

Following **General Procedure H** with glucose C6 hydrazide **117** (0.1 mmol) and 2-trifluoromethyl-5-iodo-pyridine **84**, purification through flash column chromatography afforded product **119** (32 mg, 54% yield).

$[\alpha]_D^{25}$ = +7.5° (c = 0.5, $CHCl_3$)

^{1}H NMR (600 MHz, $CDCl_3$) δ 8.56 (d, J = 2.1 Hz, 1H), 7.67 (dd, J = 8.0, 2.1 Hz, 1H), 7.56 (dd, J = 8.0, 0.8 Hz, 1H), 7.38 – 7.28 (m, 14H), 5.00 (t, J = 10.9 Hz, 2H), 4.82 (d, J = 10.8 Hz, 1H), 4.78 (d, J = 12.1 Hz, 1H), 4.64 (d, J = 12.2 Hz, 2H), 4.45 (d, J = 3.6 Hz, 1H), 3.99 (dd, J = 9.6, 8.8 Hz, 1H), 3.76 (td, J = 9.7, 2.6 Hz, 1H), 3.51 (dd, J = 9.6, 3.6 Hz, 1H), 3.25 (dd, J = 9.7, 8.8 Hz, 1H), 3.14 (dd, J = 14.1, 2.5 Hz, 1H), 3.00 (s, 3H), 2.62 (dd, J = 14.1, 9.6 Hz, 1H).

^{13}C NMR (151 MHz, $CDCl_3$) δ 151.1, 146.4 (q, $^2J_{C,F}$ = 34.6 Hz), 138.6, 138.2, 138.1, 137.7, 128.7, 128.64, 128.62, 128.23, 128.19, 128.15, 128.13, 128.08, 127.9, 121.8 (q, $^1J_{C,F}$ = 273.7 Hz), 119.9 (q, $^3J_{C,F}$ = 3.0 Hz), 97.9, 82.1, 81.3, 80.2, 76.0, 75.3, 73.5, 70.6, 55.2, 35.0.

HRMS (m/z): (ESI) calc. for $C_{34}H_{35}F_3NO_5$ $[M+H]^+$: 594.2467 found 594.2480

Compound 120

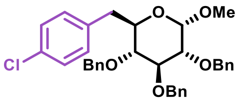

Following **General Procedure H** with glucose C6 hydrazide **117** (0.1 mmol) and 4-bromochlorobenzene, purification through flash column chromatography afforded product **120** (24 mg, 43% yield).

$[\alpha]_D^{20}$ = +23.8 ° (c = 0.6, $CHCl_3$)

^{1}H NMR (600 MHz, $CDCl_3$) δ 7.38 – 7.27 (m, 15H), 7.24 – 7.20 (m, 2H), 7.14 (dd, *J* = 8.7, 2.0 Hz, 2H), 4.98 (dd, *J* = 22.1, 10.8 Hz, 2H), 4.80 (dd, *J* = 26.3, 11.3 Hz, 2H), 4.64 (dd, *J* = 11.6, 5.4 Hz, 2H), 4.47 (d, *J* = 3.7 Hz, 1H), 3.98 (t, *J* = 9.0 Hz, 1H), 3.76 (td, *J* = 9.8, 2.5 Hz, 1H), 3.51 (dd, *J* = 9.5, 3.6 Hz, 1H), 3.25 (t, *J* = 9.0 Hz, 1H), 3.11 (dd, *J* = 14.0, 2.5 Hz, 1H), 3.00 (s, 3H), 2.51 (dd, *J* = 13.9, 9.7 Hz, 1H).

^{13}C NMR (151 MHz, $CDCl_3$) δ 138.8, 138.33, 138.25, 137.4, 132.1, 130.8, 128.63, 128.59, 128.3, 128.23, 128.18, 128.04, 127.99, 127.8, 97.8, 82.4, 81.7, 80.3, 75.9, 75.3, 73.4, 71.2, 54.9, 37.3.

HRMS (m/z): (ESI) calc. $C_{34}H_{35}ClNaO_5$ $[M+Na]^+$: 581.2071 found 581.2085

Compound 121

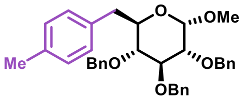

Following **General Procedure H** with glucose C6 hydrazide **117** (0.1 mmol) and 4-bromotoluene, purification through flash column chromatography afforded product **121** (23 mg, 42% yield).

$[\boldsymbol{a}]_{\mathbf{D}}^{\mathbf{25}}$ = +2.3° (c = 0.5, $CHCl_3$)

^{1}H NMR (600 MHz, $CDCl_3$) δ 7.39 – 7.26 (m, 14H), 7.10 (d, *J* = 8.0 Hz, 2H), 7.06 (d, *J* = 7.8 Hz, 2H), 5.00 (d, *J* = 10.8 Hz, 1H), 4.95 (d, *J* = 10.9 Hz, 1H), 4.83 (d, *J* = 10.8 Hz, 1H), 4.78 (d, *J* = 12.1 Hz, 1H), 4.66 (d, *J* = 3.2 Hz, 1H), 4.64 (d, *J* = 4.4 Hz, 1H), 4.49 (d, *J* = 3.6 Hz, 1H), 4.00 (t, *J* = 9.2 Hz, 1H), 3.82 (td, *J* = 9.8, 2.4 Hz, 1H), 3.52 (dd, *J* = 9.6, 3.6 Hz, 1H), 3.27 (t, *J* = 9.3 Hz, 1H), 3.14 (dd, *J* = 14.0, 2.4 Hz, 1H), 3.02 (s, 3H), 2.52 (dd, *J* = 14.1, 9.8 Hz, 1H), 2.30 (s, 3H).

^{13}C NMR (151 MHz, $CDCl_3$) δ 138.8, 138.4, 138.3, 135.67, 135.66, 129.3, 128.9, 128.60, 128.57, 128.22, 128.19, 128.1, 128.0, 127.9, 127.8, 97.7, 82.4, 81.9, 80.3, 75.9, 75.3, 73.4, 71.4, 54.9, 37.4, 21.2.

HRMS (m/z): (ESI) calc. for $C_{35}H_{38}NaO_5$ $[M+Na]^+$: 561.2617 found 561.2613

Compound 122

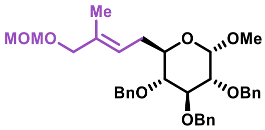

Following **General Procedure H** with glucose C6 hydrazide **117** (0.1 mmol) and vinyl iodide, purification through flash column chromatography afforded product **12** (19 mg, 33% yield).

$[\alpha]_D^{25}$ = +5.4° (c = 0.5, $CHCl_3$)

^{1}H NMR (600 MHz, $CDCl_3$) δ 7.38 – 7.26 (m, 15H), 5.51 (ddd, *J* = 7.6, 6.1, 1.4 Hz, 1H), 4.98 (d, *J* = 10.8 Hz, 1H), 4.91 (d, *J* = 11.0 Hz, 1H), 4.83 – 4.76 (m, 2H), 4.67 (d, *J* = 12.2 Hz, 1H), 4.61 – 4.58 (m, 3H), 4.54 (d, *J* = 3.6 Hz, 1H), 3.97 (t, *J* = 9.2 Hz, 1H), 3.91 (s, 2H), 3.65 (td, *J* = 9.0, 3.0 Hz, 1H), 3.51 (dd, *J* = 9.7, 3.6 Hz, 1H), 3.35 (s, 3H), 3.34 (s, 3H), 3.24 (dd, *J* = 9.7, 8.8 Hz, 1H), 2.52 (dd, *J* = 15.3, 4.5 Hz, 1H), 2.17 (dt, *J* = 15.6, 7.9 Hz, 1H), 1.63 (d, *J* = 1.4 Hz, 3H).

^{13}C NMR (151 MHz, $CDCl_3$) δ 138.9, 138.4, 138.3, 133.9, 128.59, 128.57, 128.3, 128.2, 128.0, 127.9, 127.8, 124.0, 97.9, 95.4, 82.3, 81.6, 80.3, 75.9, 75.3, 73.5, 73.2, 70.1, 55.3, 55.1, 29.8, 14.4.

HRMS (m/z): (ESI) calc. for $C_{34}H_{42}NaO_7$ $[M+Na]^+$: 585.2828 found 585.2821

Compound arabino-124

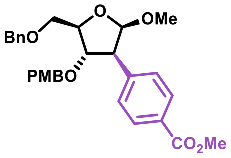

Following **General Procedure H** with ribose C2 hydrazide **123** (0.1 mmol) and 4-iodobenzoate, purification through flash column chromatography afforded product **124** (23 mg, 46% yield, arabino:ribo = 6.8:1).

Compound arabino-124:

$[\alpha]_D^{20}$ = –59.0° (c = 0.1, $CHCl_3$)

^{1}H NMR (600 MHz, $CDCl_3$) δ 7.96 – 7.93 (m, 2H), 7.40 – 7.34 (m, 6H), 7.33 – 7.29 (m, 1H), 6.98 – 6.94 (m, 2H), 6.75 – 6.71 (m, 2H), 4.96 (d, *J* = 4.8 Hz, 1H), 4.62 (s, 2H), 4.35 (dd, *J* = 8.9, 5.5 Hz, 1H), 4.31 – 4.25 (m, 3H), 3.92 (s, 3H), 3.76 (s, 3H), 3.66 – 3.59 (m, 2H), 3.54 (dd, *J* = 8.9, 4.8 Hz, 1H), 3.22 (s, 3H).

^{13}C NMR (151 MHz, $CDCl_3$) δ 167.2, 159.3, 142.0, 138.3, 129.9, 129.6, 129.5, 128.9, 128.6, 127.9, 127.8, 113.8, 105.5, 84.0, 82.6, 73.5, 72.7, 72.1, 57.2, 55.4, 55.0, 52.2.

HRMS (m/z): (ESI) calc. $C_{29}H_{33}O_7$ $[M+H]^+$: 493.2226 found 493.2216

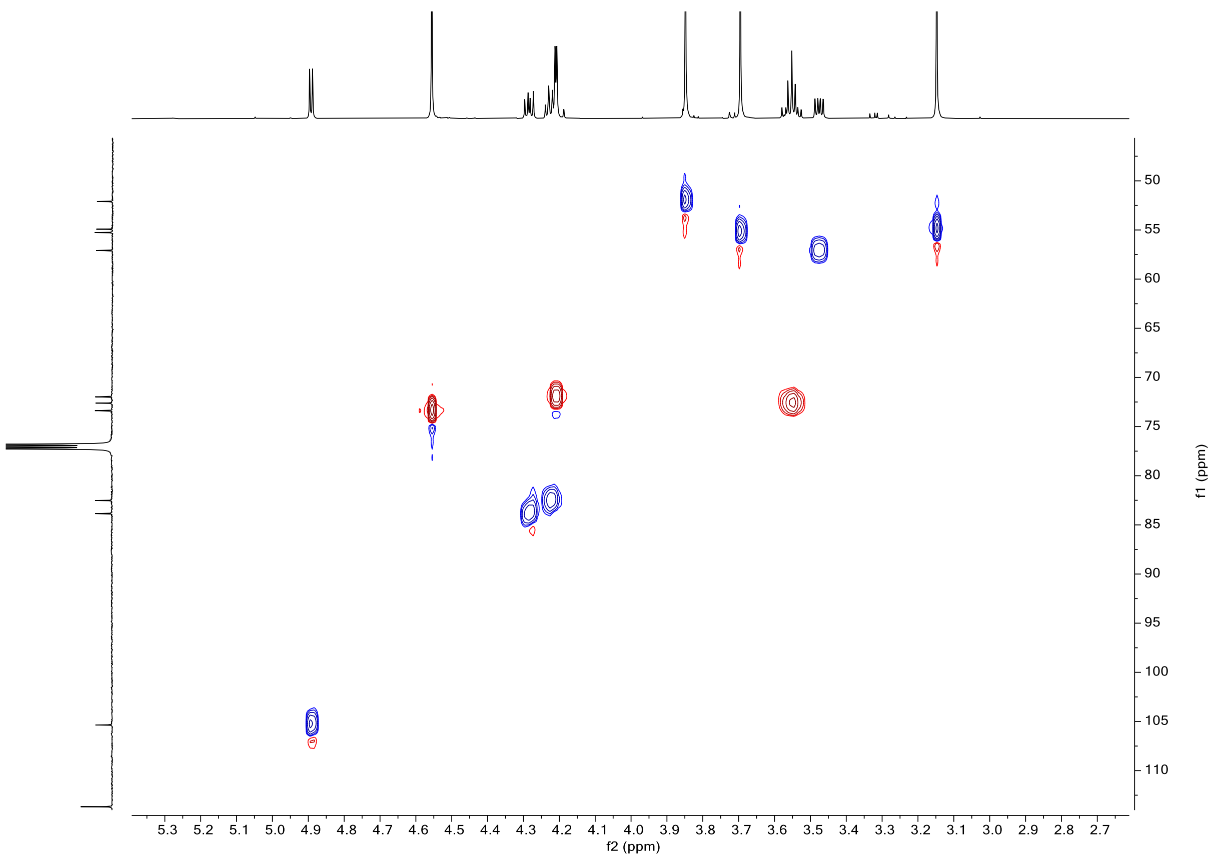

H-C HSQC NMR Spectrum of compound **arabino-124**

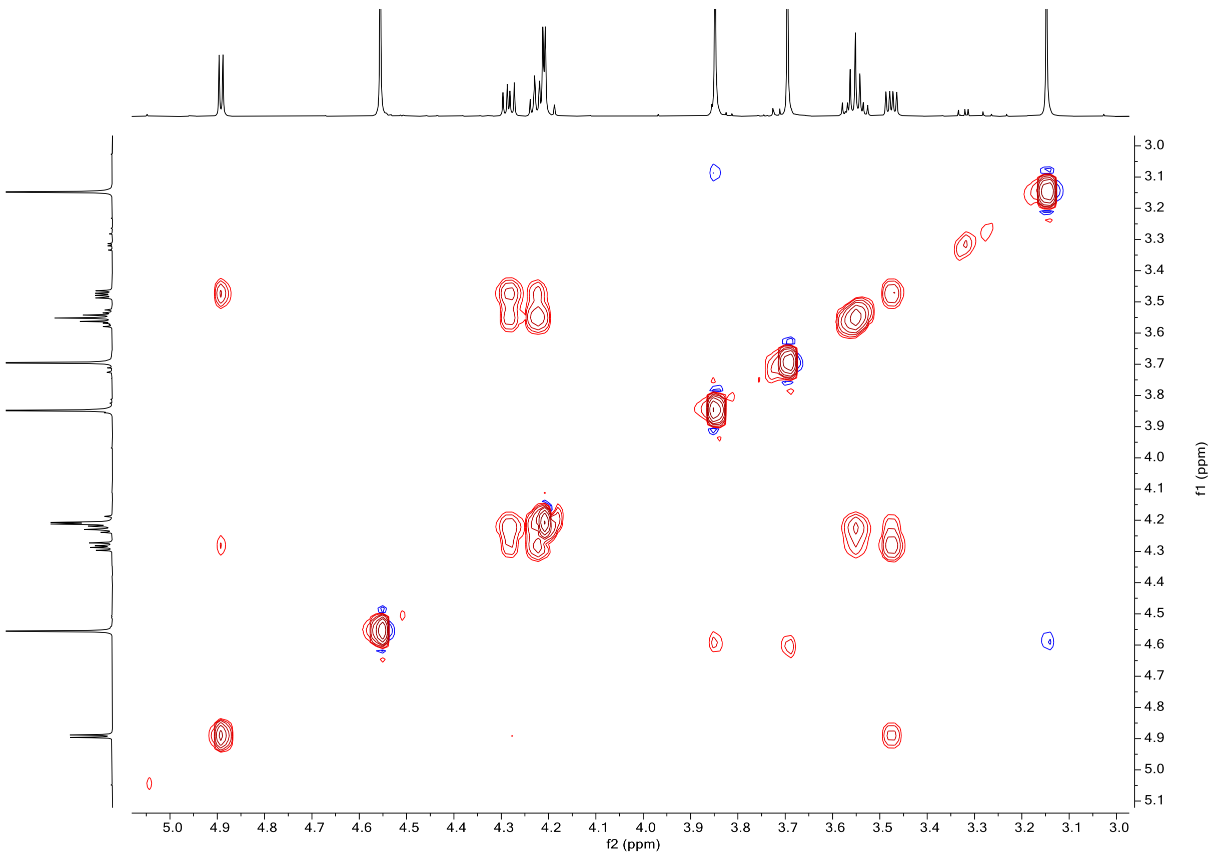

H-H COSY NMR Spectrum of compound **arabino-124**

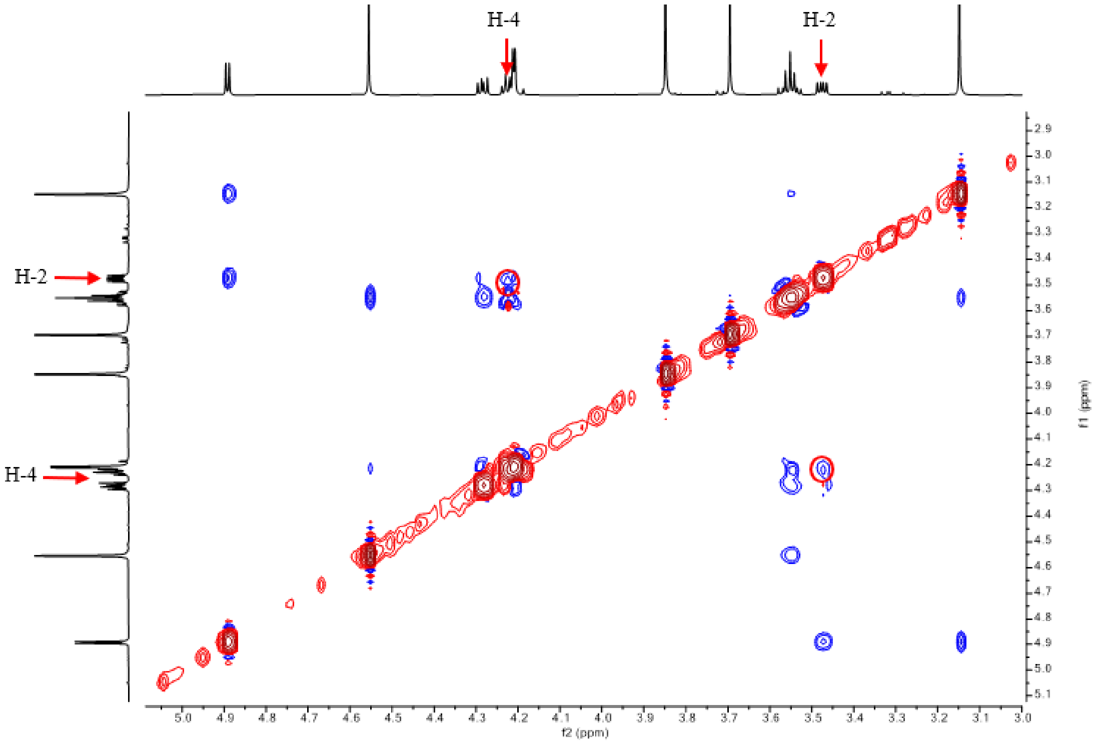

NOESY NMR Spectrum of **arabino-124**

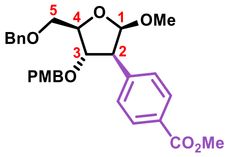

Compound 126

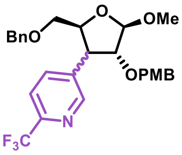

COSY, NOESY and HSQC spectra of both isomers respectively are attached below.

Following **General Procedure H** with ribose C3 hydrazide **125** (0.1 mmol) and 2-trifluoromethyl-5-iodo-pyridine **84**, purification through flash column chromatography afforded product **126** (13 mg, 25% yield, xylo:ribo = 2.1:1).

Compound ribo-126:

$[\alpha]_D^{25}$ = –16.8° (c = 0.25, $CHCl_3$)

^{1}H NMR (600 MHz, $CDCl_3$) δ 8.59 (d, J = 2.2 Hz, 1H), 7.61 (dd, J = 8.1, 2.2 Hz, 1H), 7.55 (dd, J = 8.1, 0.8 Hz, 1H), 7.31 – 7.26 (m, 2H), 7.26 – 7.24 (m, 1H), 7.22 – 7.19 (m, 2H), 7.01 (d, J = 8.6 Hz, 2H), 6.74 (d, J = 8.7 Hz, 2H), 4.82 (d, J = 3.9 Hz, 1H), 4.53 – 4.43 (m, 3H), 4.37 (d, J = 12.0 Hz, 1H), 4.22 (ddd, J = 8.9, 6.4, 5.0 Hz, 1H), 4.00 (dd, J = 10.4, 4.0 Hz, 1H), 3.78 (s, 3H), 3.59 (dd, J = 9.9, 6.4 Hz, 1H), 3.51 (dd, J = 10.0, 5.0 Hz, 1H), 3.46 – 3.42 (m, 1H), 3.41 (s, 3H).

^{13}C NMR (151 MHz, $CDCl_3$) δ 159.6, 150.2, 147.0 (q, $^2J_{C,F}$ = 34.7 Hz), 138.6, 137.8, 136.4, 129.8, 129.1, 128.5, 127.9, 127.7, 121.7 (q, $^1J_{C,F}$ = 273.3 Hz), 120.4, 113.9, 101.3, 83.9, 81.7, 73.6, 73.3, 72.6, 55.4, 55.0, 48.2.

HRMS (m/z): (ESI) calc. for $C_{27}H_{29}F_3NO_5$ $[M+H]^+$: 504.1998 found 504.1998

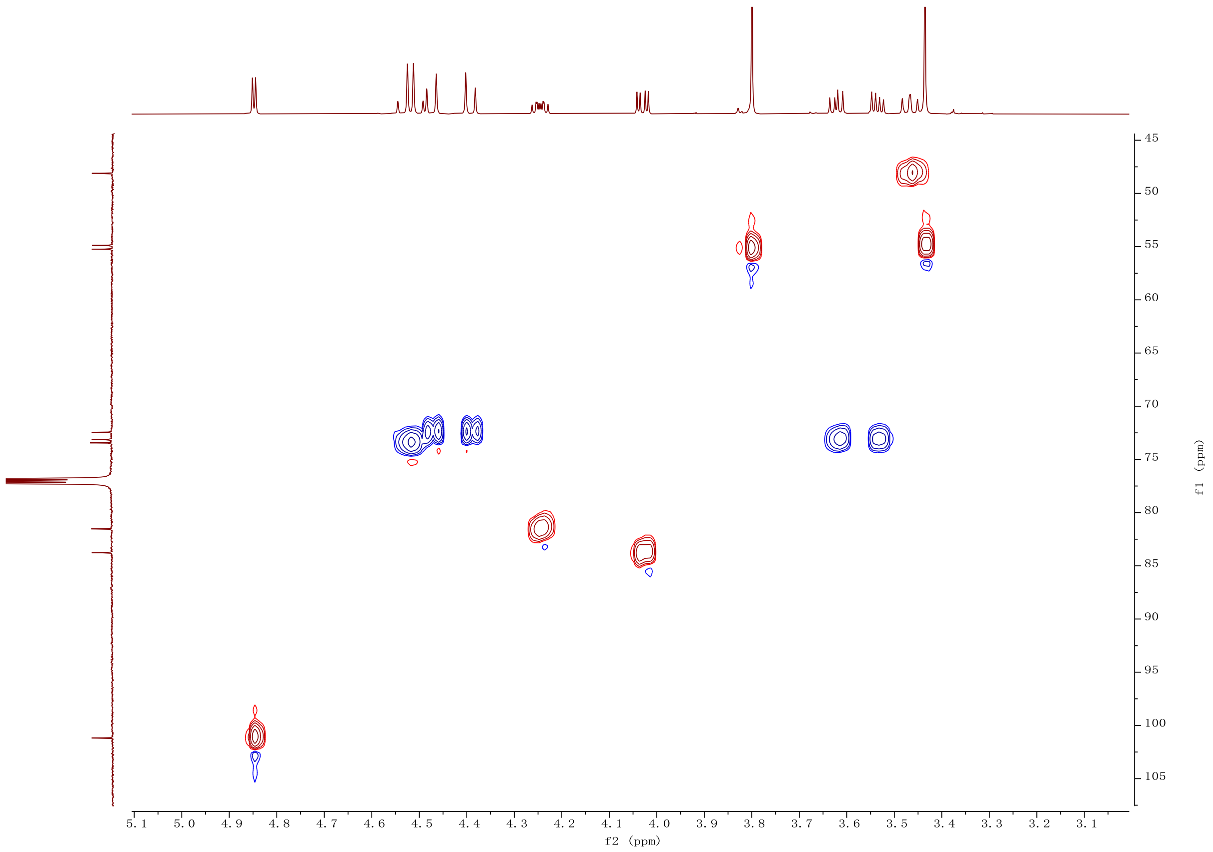

H-C HSQC NMR Spectrum of compound **ribo-126**

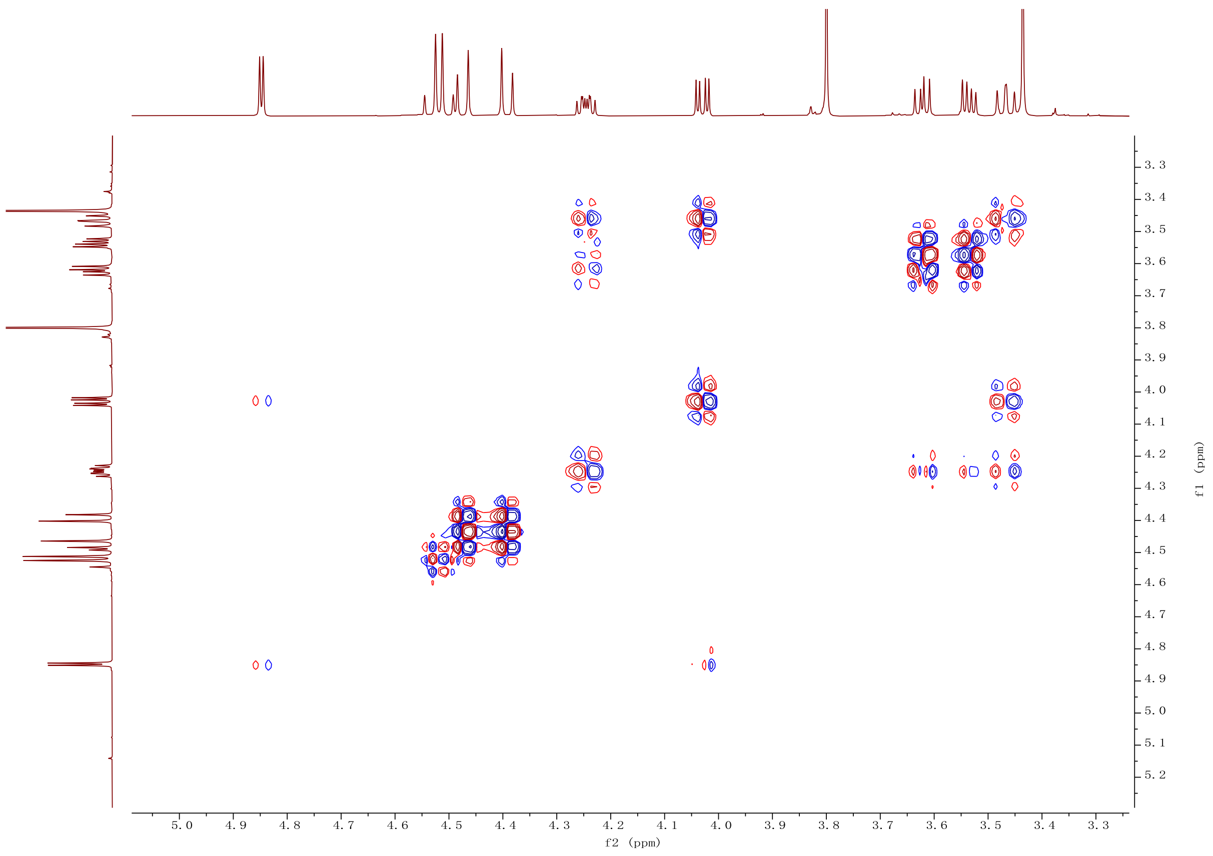

H-H COSY NMR Spectrum of compound **ribo-126**

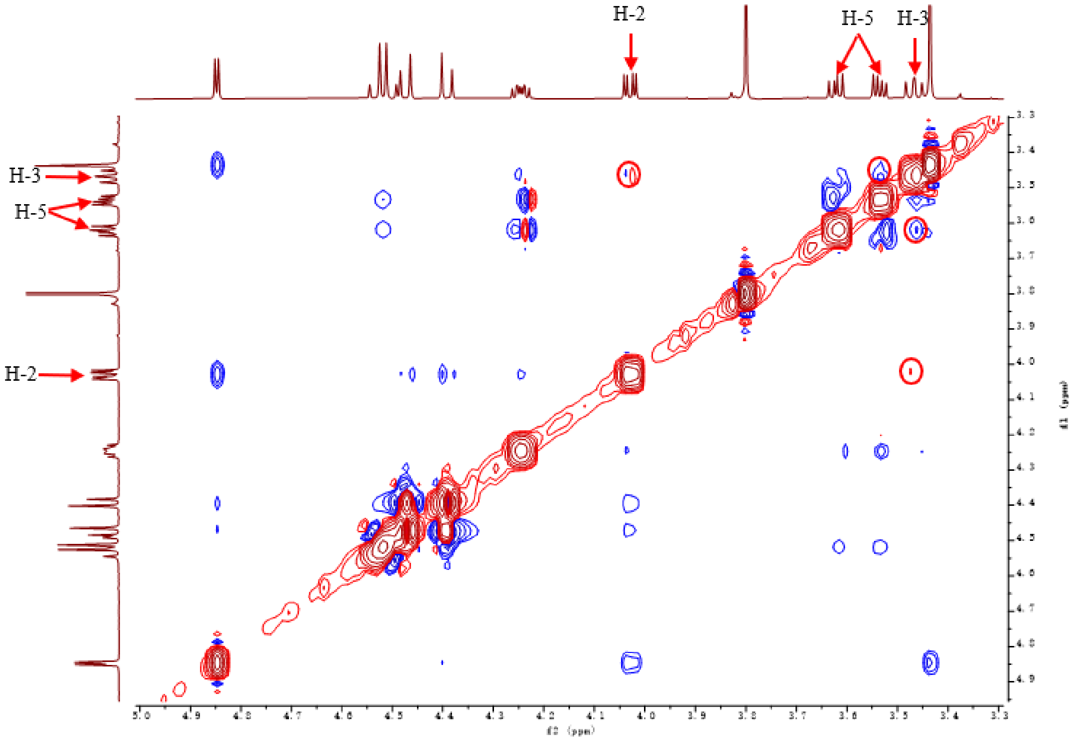

NOESY NMR Spectrum of **ribo-126**

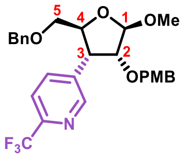

Compound xylo-126:

$[\alpha]_D^{25}$ = +38.8° (c = 0.25, $CHCl_3$)

^{1}H NMR (600 MHz, $CDCl_3$) δ 8.60 (d, *J* = 2.1 Hz, 1H), 7.76 (dd, *J* = 8.1, 2.2 Hz, 1H), 7.53 (d, *J* = 8.1 Hz, 1H), 7.29 – 7.26 (m, 2H), 7.26 – 7.23 (m, 1H), 7.18 (d, *J* = 8.6 Hz, 2H), 7.10 (d, *J* = 6.3 Hz, 2H), 6.83 (d, *J* = 8.6 Hz, 2H), 5.07 (d, *J* = 1.9 Hz, 1H), 4.63 (dt, *J* = 7.2, 6.0 Hz, 1H), 4.52 (d, *J* = 11.4 Hz, 1H), 4.46 (d, *J* = 11.4 Hz, 1H), 4.28 (d, *J* = 11.8 Hz, 1H), 4.18 (d, *J* = 11.8 Hz, 1H), 4.14 (dd, *J* = 3.6, 1.9 Hz, 1H), 3.79 (s, 3H), 3.55 (dd, *J* = 7.2, 3.5 Hz, 1H), 3.49 (s, 3H), 3.32 (dd, *J* = 9.9, 5.8 Hz, 1H), 3.08 (dd, *J* = 9.9, 6.4 Hz, 1H).

^{13}C NMR (151 MHz, $CDCl_3$) δ 159.6, 150.8, 146.7 (q, $^2J_{C,F}$ = 34.6 Hz), 137.8, 137.5, 137.1, 129.7, 129.3, 128.5, 127.9, 127.8, 121.8 (q, $^1J_{C,F}$ = 274.0 Hz), 120.0, 114.0, 109.9, 88.7, 79.9, 73.5, 72.2, 69.9, 56.4, 55.4, 50.2.

HRMS (m/z): (ESI) calc. for $C_{27}H_{29}F_3NO_5$ $[M+H]^+$: 504.1998 found 504.1995

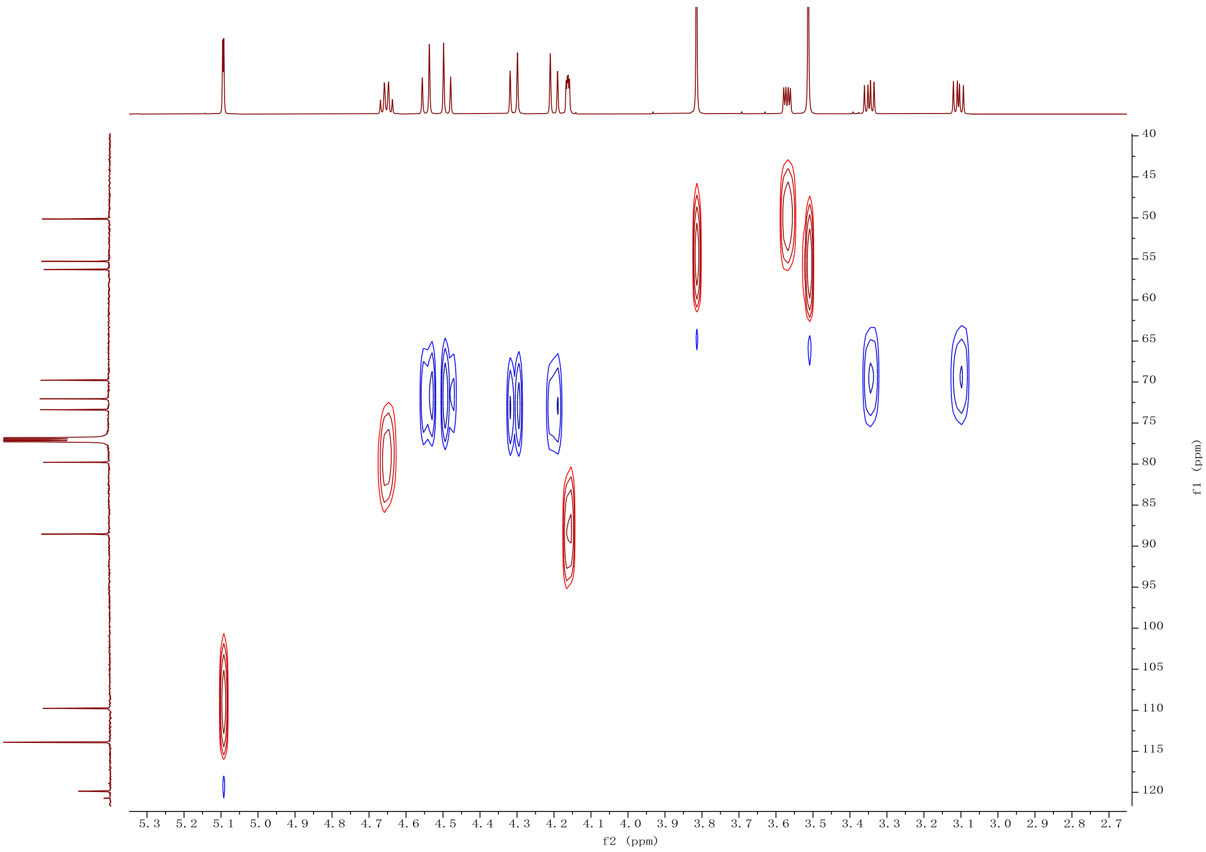

H-C HSQC NMR Spectrum of compound **xylo-126**

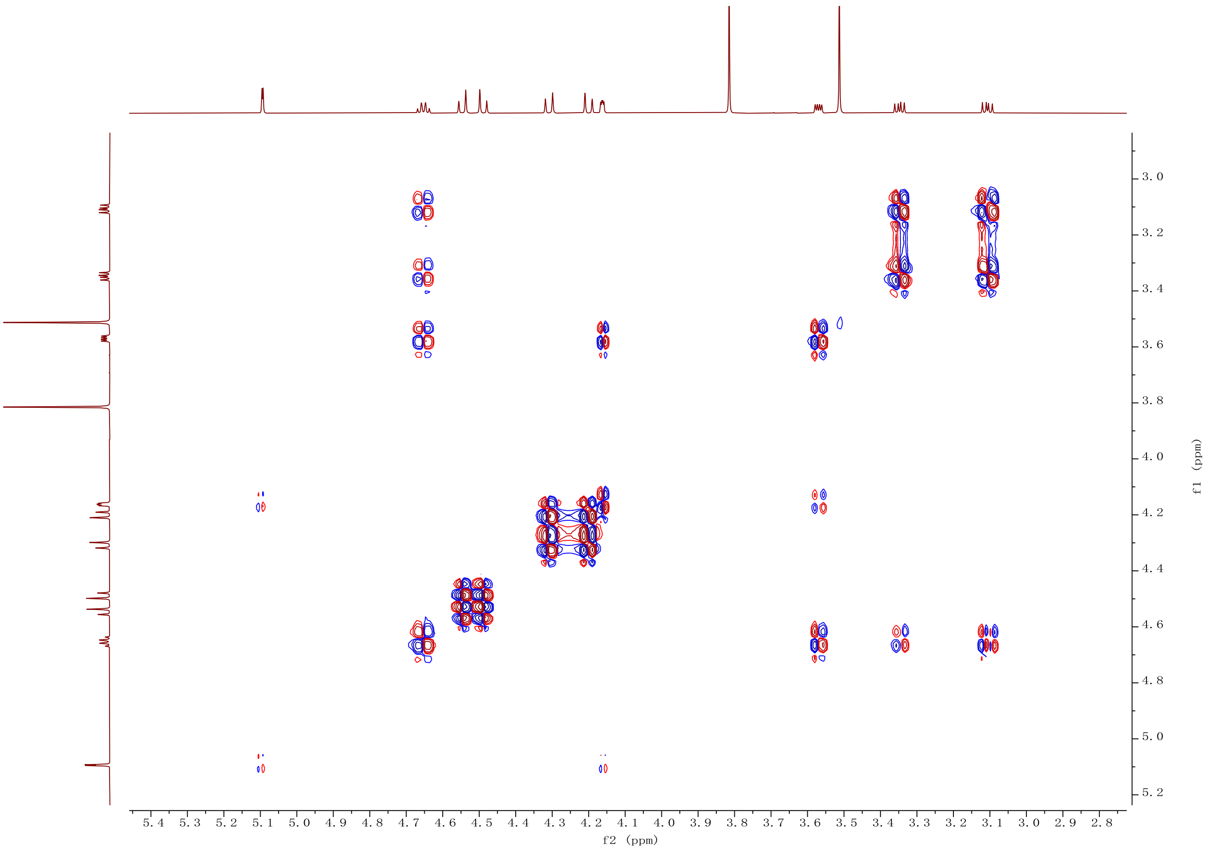

H-H COSY NMR Spectrum of compound **xylo-126**

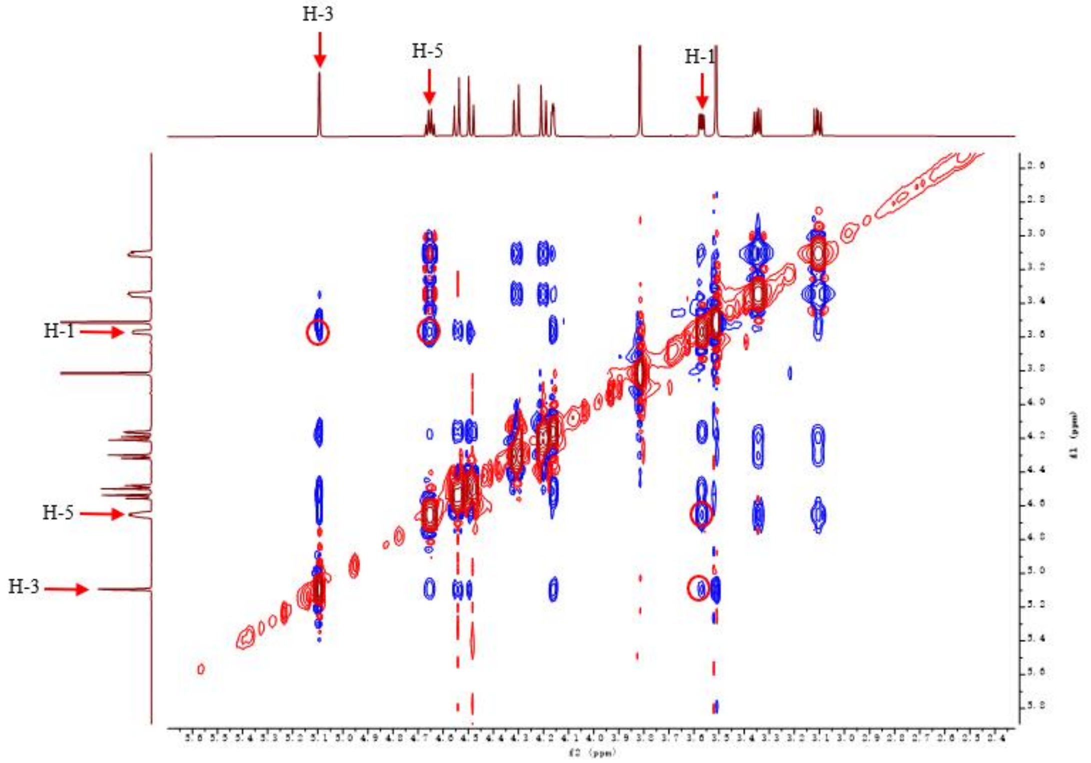

NOESY NMR Spectrum of **xylo-126**

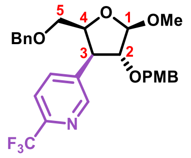

Compound 128

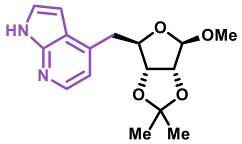

COSY, NOESY and HSQC spectra of both isomers respectively are attached below.

Following **General Procedure H** with ribose C5 hydrazide **127** (0.1 mmol) and 4-iodo-7-azaindole, purification through flash column chromatography afforded product **128** (16 mg, 51% yield).

$[\alpha]_D^{25}$ = –6.9° (c = 0.5, $CHCl_3$)

^{1}H NMR (600 MHz, $CDCl_3$) δ 10.38 (s, 1H), 8.27 (d, *J* = 4.9 Hz, 1H), 7.35 (dd, *J* = 3.5, 1.8 Hz, 1H), 7.00 (d, *J* = 4.9 Hz, 1H), 6.56 (dd, *J* = 3.6, 1.3 Hz, 1H), 5.01 (s, 1H), 4.73 – 4.68 (m, 2H), 4.66 (dd, *J* = 5.9, 0.9 Hz, 1H), 3.37 (s, 3H), 3.28 (dd, *J* = 14.2, 7.3 Hz, 1H), 3.16 (dd, *J* = 14.2, 8.7 Hz, 1H), 1.45 (s, 3H), 1.27 (s, 3H).

^{13}C NMR (151 MHz, $CDCl_3$) δ 148.8, 143.1, 139.6, 124.8, 120.4, 116.3, 112.5, 110.1, 99.2, 86.5, 85.7, 83.7, 55.3, 38.4, 26.6, 25.1.

HRMS (m/z): (ESI) calc. for $C_{16}H_{21}N_2O_4$ $[M+H]^+$: 305.1501 found 305.1503

Compound 2-deoxy-ribo-130

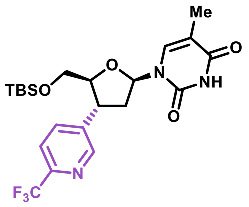

Following **General Procedure H** with 2-deoxyribose C5 hydrazide **129** (0.1 mmol) and 5-bromo-2-(trifluoromethyl)pyridine, purification through flash column chromatography afforded product **2-deoxy-ribo-130** (20.4 mg, 44% yield).

$[\alpha]_D^{25}$ = +10.1 (c = 1.0, $CHCl_3$)

^{1}H NMR (600 MHz, CD_3CN) δ 8.86 (s, 1H), 8.63 (d, *J* = 2.1 Hz, 1H), 7.78 (dd, *J* = 8.1, 2.2 Hz, 1H), 7.70 (d, *J* = 8.1 Hz, 1H), 7.61 (d, *J* = 1.3 Hz, 1H), 6.27 (dd, *J* = 7.2, 3.3 Hz, 1H), 4.10 (dt, *J* = 8.9, 2.6 Hz, 1H), 4.03 (dd, *J* = 11.8, 2.5 Hz, 1H), 3.66 (dt, *J* = 10.5, 8.7 Hz, 1H), 3.63 (dd, *J* = 11.8, 2.6 Hz, 1H), 2.67 (ddd, *J* = 14.0, 10.3, 7.3 Hz, 1H), 2.54 (ddd, *J* = 14.0, 8.5, 3.3 Hz, 1H), 1.97 (d, *J* = 1.2 Hz, 3H), 0.91 (s, 9H), 0.09 (s, 3H), 0.08 (s, 3H).

^{13}C NMR (151 MHz, CD_3CN) δ 163.9, 150.4, 149.9, 147.6 (q, $^2J_{C,F}$ = 34.9 Hz), 138.3, 136.2, 135.4, 121.5 (q, $^1J_{C,F}$ = 273.9 Hz), 120.8 (q, $^3J_{C,F}$ = 2.8 Hz), 110.8, 87.2, 85.0, 61.6, 41.1, 40.6, 26.1, 18.6, 12.9, -5.18, -5.19.

HRMS (m/z): (ESI) calc. for $C_{22}H_{31}F_3N_3O_4Si$ $[M+H]^+$: 486.2036 found 486.2025

Compound 132

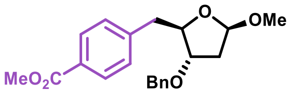

Following **General Procedure H** with 2-deoxyribose C5 hydrazide **131** (0.1 mmol) and 4-iodobenzoate, purification through flash column chromatography afforded product **132** (17 mg, 47% yield).

$[\alpha]_D^{25}$ = +77.6° (c = 0.25, $CHCl_3$)

^{1}H NMR (600 MHz, $CDCl_3$) δ 7.93 (d, *J* = 8.2 Hz, 2H), 7.32 – 7.27 (m, 3H), 7.24 (d, *J* = 8.2 Hz, 2H), 7.20 – 7.17 (m, 2H), 4.99 (dd, *J* = 5.6, 1.5 Hz, 1H), 4.45 (d, *J* = 12.2 Hz, 1H), 4.35 (d, *J* = 12.2 Hz, 1H), 4.32 (dt, *J* = 6.7, 5.3 Hz, 1H), 3.91 (s, 3H), 3.72 (ddd, *J* = 8.2, 5.0, 3.3 Hz, 1H), 3.35 (s, 3H), 2.94 – 2.84 (m, 2H), 2.11 (ddd, *J* = 13.8, 8.1, 5.6 Hz, 1H), 1.95 (ddd, *J* = 14.0, 3.4, 1.5 Hz, 1H).

^{13}C NMR (151 MHz, $CDCl_3$) δ 167.3, 143.5, 138.0, 129.7, 129.6, 128.5, 128.4, 128.0, 127.9, 104.7, 82.3, 80.5, 71.7, 55.2, 52.2, 39.5, 38.7.

HRMS (m/z): (ESI) calc. for $C_{21}H_{24}NaO_5$ $[M+Na]^+$: 379.1521found 379.1521

Compound SC8

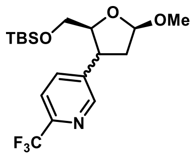

Following **General Procedure H** with 2-deoxyribose C3 hydrazide **SP22** (0.1 mmol) and 5-iodo-2-(trifluoromethyl)pyridine, purification through flash column chromatography afforded product **SC8** (18.8 mg, 48% yield 2-deoxy-xylo: 2-deoxy-ribo = 1:1.8).

Compound 2-deoxy-ribo-SC8:

$[\alpha]_D^{20}$ = –61.6 ° (c = 0.25, $CHCl_3$)

^{1}H NMR (600 MHz, $CDCl_3$) δ 8.63 (d, J = 2.2 Hz, 1H), 7.76 (dd, J = 8.2, 2.2 Hz, 1H), 7.63 (dd, J = 8.1, 0.8 Hz, 1H), 5.14 (d, J = 4.8 Hz, 1H), 4.11 (ddd, J = 8.1, 6.3, 4.9 Hz, 1H), 3.77 (dd, J = 10.3, 5.0 Hz, 1H), 3.67 (dd, J = 10.3, 6.3 Hz, 1H), 3.58 (dt, J = 11.4, 7.7 Hz, 1H), 3.38 (s, 3H), 2.39 (dd, J = 12.8, 7.3 Hz, 1H), 2.18 (ddd, J = 12.8, 11.4, 4.9 Hz, 1H), 0.79 (s, 9H), 0.02 (s, 3H), -0.02 (s, 3H).

^{13}C NMR (151 MHz, $CDCl_3$) δ 150.1, 146.8 (q, $^2J_{C,F}$ = 34.9 Hz), 141.0, 136.3, 121.7 (q, $^1J_{C,F}$ = 274.0 Hz), 120.4, 105.0, 86.8, 65.8, 54.8, 43.6, 42.1, 29.9, 25.8, 18.3, -5.4 (d, $^3J_{C,F}$ = 10.6 Hz).

HRMS (m/z): (ESI) calc. $C_{18}H_{29}F_3NO_3Si$ $[M+H]^+$: 392.1869 found 392.1866

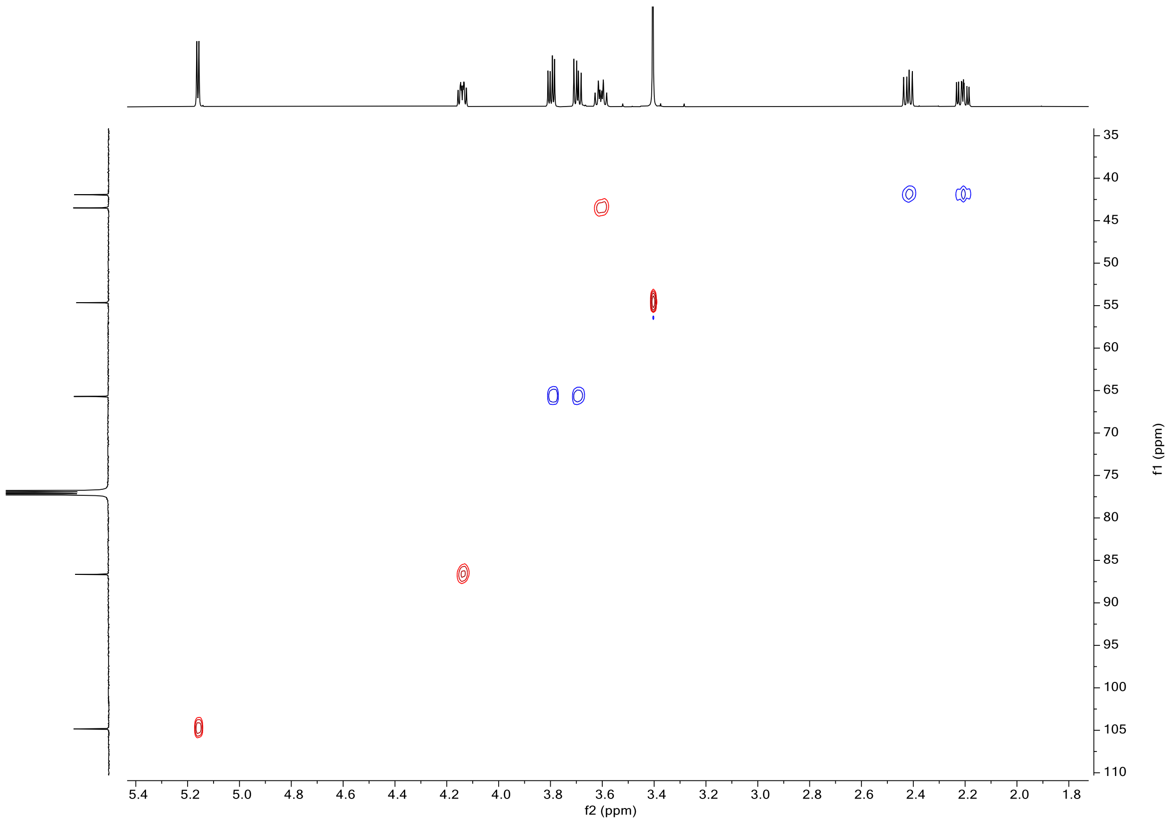

H-C HSQC NMR Spectrum of compound **2-deoxy-ribo-SC8**

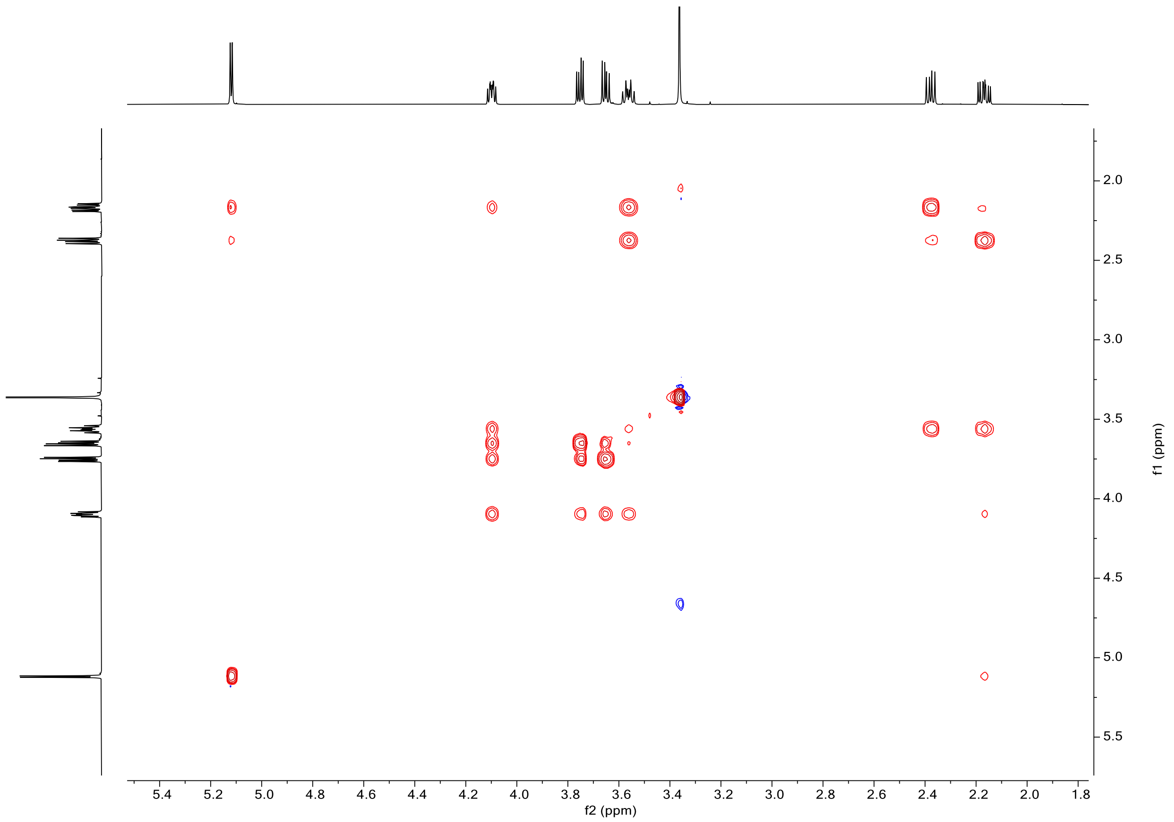

H-H COSY NMR Spectrum of compound **2-deoxy-ribo-SC8**

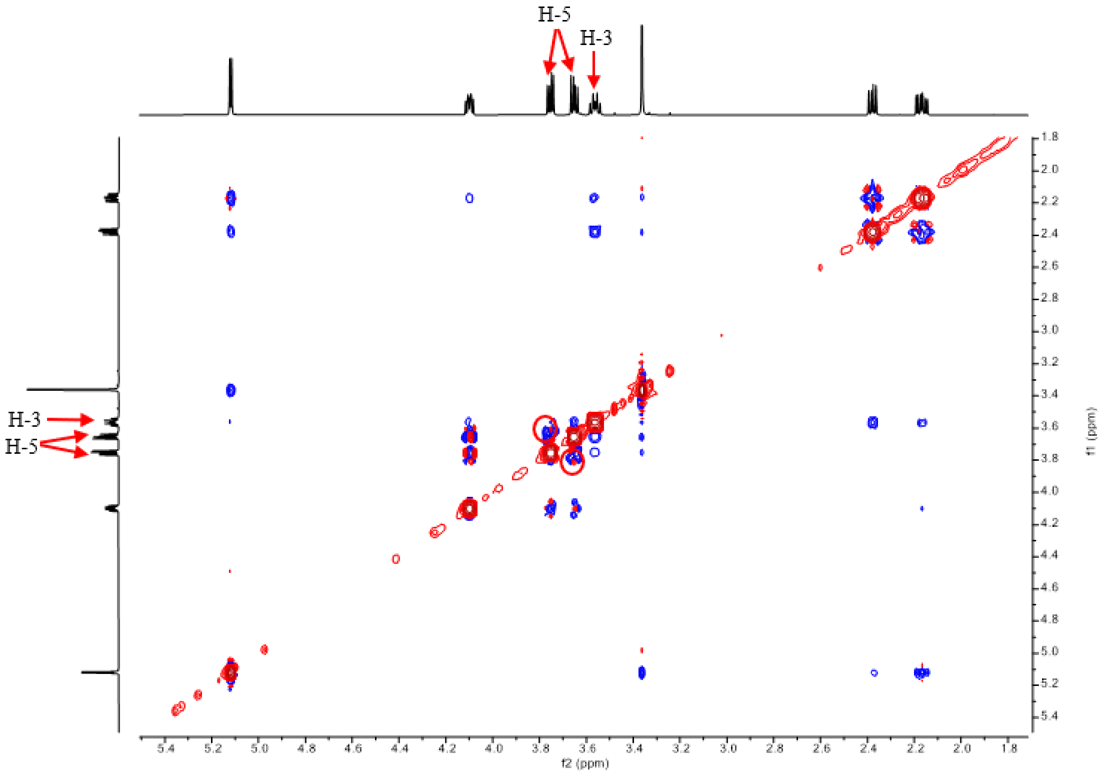

NOESY NMR Spectrum of **2-deoxy-ribo-SC8**

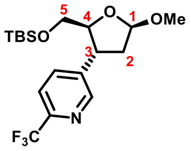

Compound 2-deoxy-xylo-SC8:

$[\boldsymbol{a}]_{\mathbf{D}}^{\mathbf{20}}$ = –20.0 ° (c = 0.1, $CHCl_3$)

^{1}H NMR (600 MHz, $CDCl_3$) δ 8.69 (s, 1H), 8.00 (d, *J* = 8.1 Hz, 1H), 7.61 (d, *J* = 8.1 Hz, 1H), 5.17 (dd, *J* = 6.3, 2.8 Hz, 1H), 4.30 (td, *J* = 7.5, 5.1 Hz, 1H), 3.64 – 3.59 (m, 1H), 3.52 (dd, *J* = 10.4, 4.9 Hz, 1H), 3.46 (s, 3H), 3.27 (dd, *J* = 10.4, 7.9 Hz, 1H), 2.69 – 2.63 (m, 1H), 2.17 (ddd, *J* = 13.4, 5.1, 2.9 Hz, 1H), 0.75 (s, 9H), -0.12 (s, 3H), -0.19 (s, 3H).

^{13}C NMR (151 MHz, $CDCl_3$) δ 151.2, 146.4 (q, $^2J_{C,F}$ = 33.6 Hz), 139.2, 137.9, 119.9, 105.7, 82.3, 62.8, 56.1, 42.9, 39.0, 29.9, 25.8, -5.7.

HRMS (m/z): (ESI) calc. $C_{18}H_{29}F_3NO_3Si$ $[M+H]^+$: 392.1869 found 392.1863

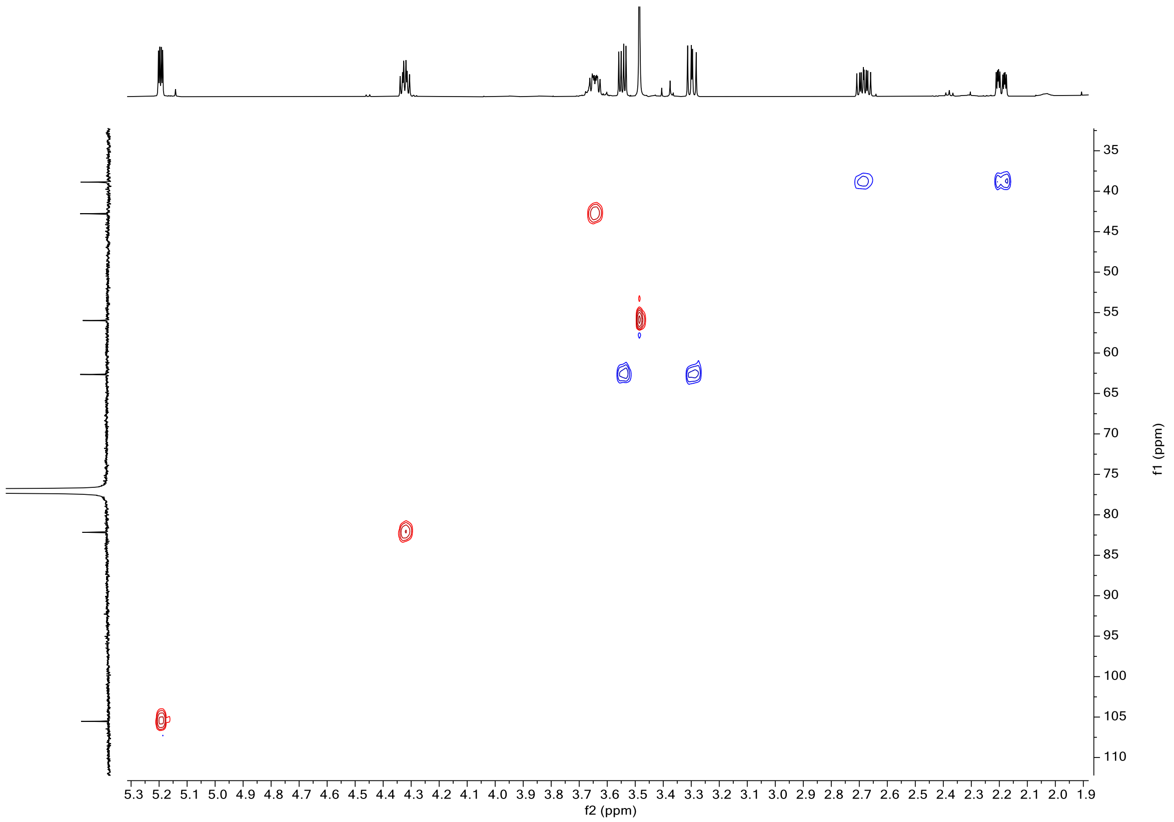

H-C HSQC NMR Spectrum of compound **2-deoxy-xylo-SC8**

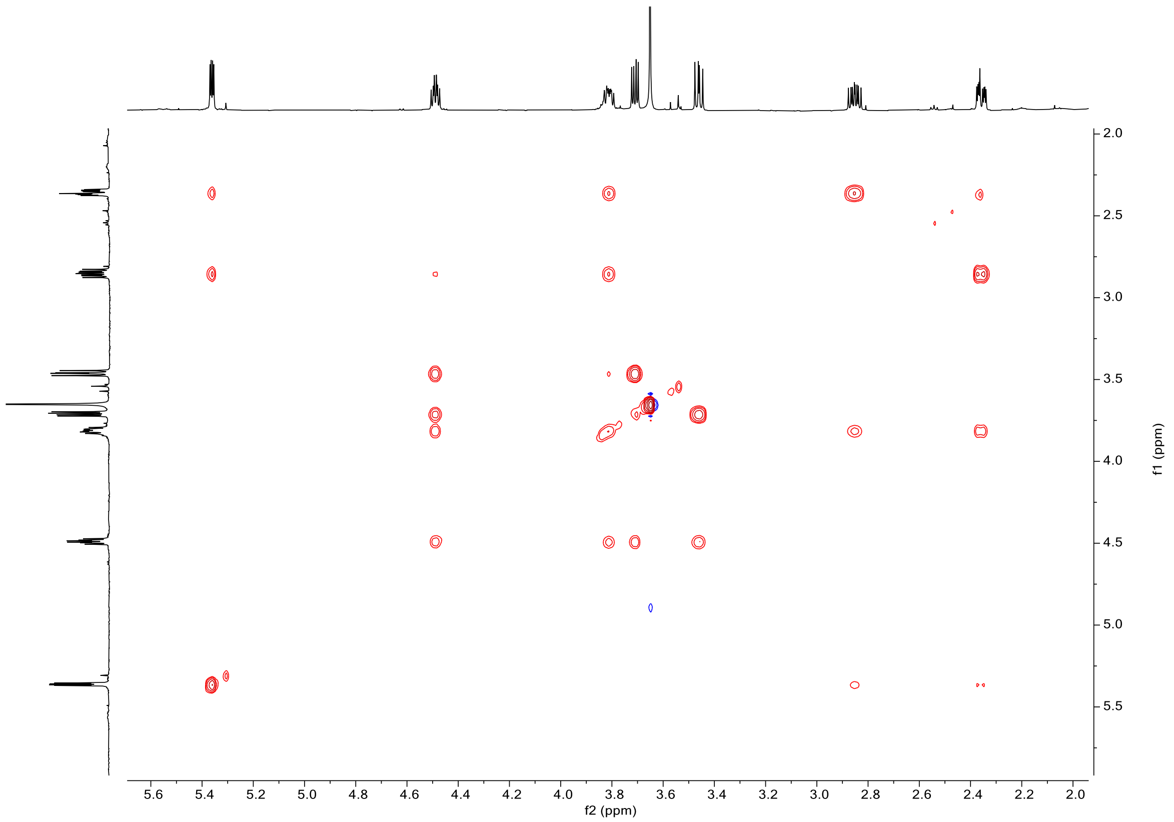

H-H COSY NMR Spectrum of compound **2-deoxy-xylo-SC8**

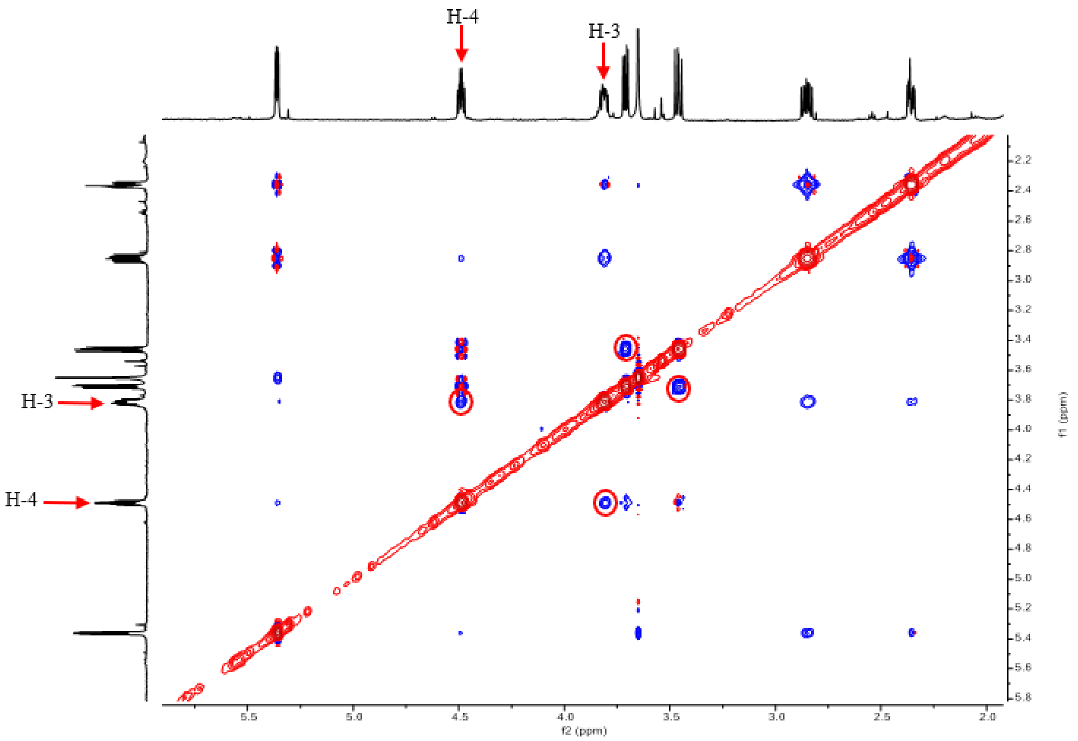

NOESY NMR Spectrum of **2-deoxy-xylo-SC8**

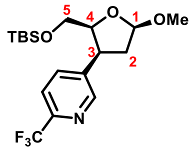

6.5 Characterization data of C1-arylated Ribose

Compound 139

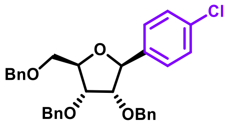

Following **General Procedure I** with Bn-protected ribose hydrazide **89** (0.1 mmol) and 1-bromo-4-chlorobenzene, purification through flash column chromatography afforded product **139** (33.0 mg, 64% yield, β:α > 19:1).

$[\boldsymbol{\alpha}]_{\mathbf{D}}^{\mathbf{20}}$ = –39.8° (c = 0.5, $CHCl_3$)

^{1}H NMR (600 MHz, $CDCl_3$) δ 7.36 – 7.23 (m, 17H), 7.18 – 7.14 (m, 2H), 4.97 (d, *J* = 7.0 Hz, 1H), 4.63 – 4.56 (m, 3H), 4.53 (d, *J* = 12.0 Hz, 1H), 4.49 (d, *J* = 12.0 Hz, 1H), 4.41 (d, *J* = 12.0 Hz, 1H), 4.33 (q, *J* = 3.8 Hz, 1H), 4.00 (dd, *J* = 5.2, 3.6 Hz, 1H), 3.74 (dd, *J* = 7.1, 5.1 Hz, 1H), 3.65 (dd, *J* = 10.4, 4.1 Hz, 1H), 3.60 (dd, *J* = 10.4, 3.7 Hz, 1H).

^{13}C NMR (151 MHz, $CDCl_3$) δ 139.1, 138.2, 138.0, 137.7, 133.5, 128.54, 128.46, 128.3, 128.0, 127.91, 127.89, 127.91, 127.89, 127.7, 83.9, 82.1, 81.9, 77.4, 73.6, 72.5, 72.1, 70.5.

HRMS (m/z): (ESI) calc. $C_{32}H_{31}ClNaO_4$ $[M+Na]^+$: 537.1809 found 537.1823

Compound 140

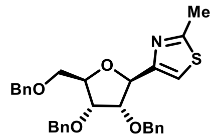

Following **General Procedure I** with Bn-protected ribose hydrazide **89** (0.1 mmol) and 4-bromo-2-methylthiazole, purification through flash column chromatography afforded product **140** (20.6 mg, 41% yield, β:α > 19:1).

$[\boldsymbol{\alpha}]_{\mathbf{D}}^{\mathbf{20}}$ = +29.8° (c = 0.4, $CHCl_3$)

^{1}H NMR (600 MHz, $CDCl_3$) δ 7.35 – 7.25 (m, 15H), 7.15 (d, *J* = 0.9 Hz, 1H), 5.20 (dd, *J* = 4.1, 0.9 Hz, 1H), 4.69 (d, *J* = 12.0 Hz, 1H), 4.65 (d, *J* = 12.0 Hz, 1H), 4.61 (d, *J* = 12.0 Hz, 1H), 4.59 (d, *J* = 11.9 Hz, 1H), 4.53 (d, *J* = 11.9 Hz, 1H), 4.46 (d, *J* = 11.8 Hz, 1H), 4.35 (dt, *J* = 6.6, 3.9 Hz, 1H), 4.18 (dd, *J* = 5.1, 4.0 Hz, 1H), 4.06 (dd, *J* = 6.6, 5.1 Hz, 1H), 3.76 (dd, *J* = 10.7, 3.6 Hz, 1H), 3.65 (dd, *J* = 10.7, 4.2 Hz, 1H), 2.68 (s, 3H).

^{13}C NMR (151 MHz, $CDCl_3$) δ 166.3, 155.2, 138.4, 138.1, 138.1, 128.5, 128.4, 128.2, 128.0, 127.8, 127.8, 127.8, 127.7, 116.0, 80.9, 80.7, 80.6, 77.3, 73.5, 72.1, 71.9, 69.9, 19.4.

HRMS (m/z): (ESI) calc. $C_{30}H_{32}NO_4S$ $[M+H]^+$: 502.2052 found 502.2064

Compound 141

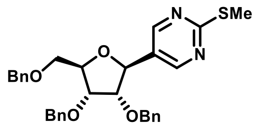

Following **General Procedure I** with Bn-protected ribose hydrazide **89** (0.1 mmol) and 5-bromo-2-(methylthio)pyrimidine, purification through flash column chromatography afforded product **141** (32 mg, 60% yield, β:α = 18:1).

$[\alpha]_D^{20}$ = –56.8° (c = 0.4, $CHCl_3$)

^{1}H NMR (600 MHz, $CDCl_3$) δ 8.50 (s, 2H), 7.37 – 7.29 (m, 8H), 7.27 – 7.23 (m, 5H), 7.17 – 7.13 (m, 2H), 4.90 (d, *J* = 8.0 Hz, 1H), 4.62 – 4.55 (m, 3H), 4.52 (d, *J* = 11.8 Hz, 1H), 4.49 (d, *J* = 12.0 Hz, 1H), 4.36 (d, *J* = 11.8 Hz, 1H), 4.33 (td, *J* = 3.7, 2.5 Hz, 1H), 4.00 (dd, *J* = 5.1, 2.5 Hz, 1H), 3.77 (dd, *J* = 8.0, 5.1 Hz, 1H), 3.58 (dd, *J* = 10.3, 4.0 Hz, 1H), 3.53 (dd, *J* = 10.3, 3.5 Hz, 1H), 2.57 (s, 3H).

^{13}C NMR (151 MHz, $CDCl_3$) δ 172.2, 155.6, 137.9, 137.7, 137.2, 128.62, 128.57, 128.5, 128.3, 128.2, 128.1, 128.0, 127.8, 83.8, 82.8, 78.1, 77.3, 73.7, 72.8, 72.1, 70.5, 14.3.

HRMS (m/z): (ESI) calc. $C_{31}H_{33}N_2O_4S$ $[M+H]^+$: 529.2161 found 529.2169

Compound 142

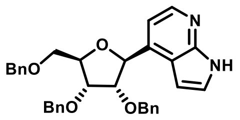

Following **General Procedure I** with Bn-protected ribose hydrazide **89** (0.1 mmol) and 3-iodo-1H-pyrrolo[2,3-b]pyridine, purification through flash column chromatography afforded product **142** (31.2 mg, 60% yield, β:α > 19:1).

$[\alpha]_D^{20}$ = +21.6° (c = 0.5, $CHCl_3$)

^{1}H NMR (600 MHz, $CDCl_3$) δ 10.46 (s, 1H), 8.24 (d, *J* = 4.9 Hz, 1H), 7.39 – 7.26 (m, 12H), 7.25 – 7.21 (m, 3H), 7.19 – 7.15 (m, 2H), 6.66 (dd, *J* = 3.6, 1.7 Hz, 1H), 5.42 (d, *J* = 6.0 Hz, 1H), 4.66 (d, *J* = 12.0 Hz, 2H), 4.57 (dd, *J* = 11.9, 4.2 Hz, 2H), 4.51 (d, *J* = 2.2 Hz, 2H), 4.43 (dt, *J* = 4.9, 3.7 Hz, 1H), 4.11 (t, *J* = 5.0 Hz, 1H), 4.04 (dd, *J* = 6.0, 5.2 Hz, 1H), 3.81 (dd, *J* = 10.5, 3.6 Hz, 1H), 3.71 (dd, *J* = 10.5, 3.8 Hz, 1H).

^{13}C NMR (151 MHz, $CDCl_3$) δ 148.9, 143.0, 141.9, 138.2, 138.0, 137.8, 128.5, 128.4, 128.2, 127.9, 127.8, 124.8, 118.0, 113.2, 100.2, 83.1, 82.0, 81.5, 77.7, 73.6, 72.7, 72.4, 70.2.

HRMS (m/z): (ESI) calc. $C_{33}H_{32}N_2NaO_4$ $[M+Na]^+$: 543.2260 found 543.2256

Compound SD1

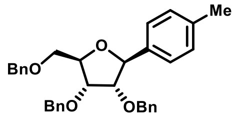

Following **General Procedure I** with Bn-protected ribose hydrazide **89** (0.1 mmol) and 1-iodo-4-methylbenzene, purification through flash column chromatography afforded product **SD1** (24.7 mg, 50% yield, β:α > 19:1).

$[\boldsymbol{\alpha}]_{\mathbf{D}}^{\mathbf{20}}$ = –17.7° (c = 0.3, $CHCl_3$)

^{1}H NMR (600 MHz, $CDCl_3$) δ 7.36 – 7.25 (m, 15H), 7.20 (dd, *J* = 7.1, 2.5 Hz, 2H), 7.11 (d, *J* = 7.8 Hz, 2H), 5.01 (d, *J* = 6.5 Hz, 1H), 4.63 – 4.54 (m, 4H), 4.48 (s, 2H), 4.34 (q, *J* = 4.1 Hz, 1H), 4.01 (dd, *J* = 5.3, 4.2 Hz, 1H), 3.81 (dd, *J* = 6.6, 5.2 Hz, 1H), 3.67 (dd, *J* = 10.4, 4.2 Hz, 1H), 3.64 (dd, *J* = 10.4, 4.1 Hz, 1H), 2.34 (s, 3H).

^{13}C NMR (151 MHz, $CDCl_3$) δ 138.3, 138.1, 138.0, 137.49, 137.47, 129.1, 128.5, 128.4, 128.2, 127.9, 127.7, 126.4, 83.7, 82.7, 81.7, 77.6, 73.6, 72.3, 72.0, 70.6, 21.3.

HRMS (m/z): (ESI) calc. $C_{33}H_{34}NaO_4$ $[M+Na]^+$: 517.2355 found 517.2369

Compound SD2

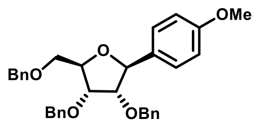

Following **General Procedure I** with Bn-protected ribose hydrazide **89** (0.1 mmol) and 1-iodo-4-methoxybenzene, purification through flash column chromatography afforded product **SD2** (24.5 mg, 48% yield, β:α > 19:1).

$[\boldsymbol{\alpha}]_{\mathbf{D}}^{\mathbf{20}}$ = –31.0° (c = 0.2, $CHCl_3$)

^{1}H NMR (600 MHz, $CDCl_3$) δ 7.37 – 7.27 (m, 12H), 7.27 – 7.24 (m, 3H), 7.18 (dd, *J* = 6.8, 2.8 Hz, 2H), 6.85 – 6.81 (m, 2H), 4.97 (d, *J* = 6.8 Hz, 1H), 4.64 – 4.57 (m, 3H), 4.55 (d, *J* = 12.0 Hz, 1H), 4.47 (d, *J* = 2.3 Hz, 2H), 4.32 (q, *J* = 4.0 Hz, 1H), 4.01 (dd, *J* = 5.3, 3.9 Hz, 1H), 3.83 – 3.78 (m, 4H), 3.66 (dd, *J* = 10.3, 4.2 Hz, 1H), 3.62 (dd, *J* = 10.4, 3.9 Hz, 1H).

^{13}C NMR (151 MHz, $CDCl_3$) δ 159.4, 138.3, 138.1, 138.0, 132.5, 128.5, 128.4, 128.2, 127.89, 127.87, 127.8, 127.7, 113.8, 83.7, 82.4, 81.8, 77.6, 73.6, 72.3, 72.0, 70.6, 55.4.

HRMS (m/z): (ESI) calc. $C_{33}H_{34}NaO_5$ $[M+Na]^+$: 533.2304 found 533.2299

Compound SD3

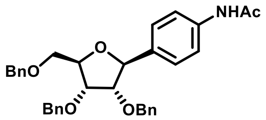

Following **General Procedure I** with Bn-protected ribose hydrazide **89** (0.1 mmol) and N-(4-iodophenyl)acetamide, purification through flash column chromatography afforded product **SD3** (22.6 mg, 42% yield, β:α > 19:1).

$[\alpha]_D^{20}$ = –24.0° (c = 0.3, $CHCl_3$)

^{1}H NMR (600 MHz, $CDCl_3$) δ 7.42 (d, *J* = 8.6 Hz, 2H), 7.36 – 7.27 (m, 13H), 7.25 (d, *J* = 2.0 Hz, 2H), 7.20 – 7.17 (m, 2H), 7.13 (s, 1H), 4.98 (d, *J* = 6.7 Hz, 1H), 4.61 – 4.57 (m, 3H), 4.55 (d, *J* = 5.2 Hz, 1H), 4.49 – 4.43 (m, 2H), 4.33 (q, *J* = 4.0 Hz, 1H), 3.99 (dd, *J* = 5.2, 4.0 Hz, 1H), 3.78 (dd, *J* = 6.7, 5.2 Hz, 1H), 3.66 (dd, *J* = 10.4, 4.1 Hz, 1H), 3.61 (dd, *J* = 10.4, 4.0 Hz, 1H), 2.18 (s, 3H).

^{13}C NMR (151 MHz, $CDCl_3$) δ 168.3, 138.3, 138.0, 137.9, 137.4, 136.5, 128.53, 128.52, 128.46, 128.2, 127.9, 127.84, 127.79, 127.76, 127.2, 119.8, 83.8, 82.4, 81.8, 77.5, 73.6, 72.4, 72.1, 70.5, 24.8.

HRMS (m/z): (ESI) calc. $C_{34}H_{35}NNaO_5$ $[M+Na]^+$: 560.2413 found 560.2425

Compound SD4

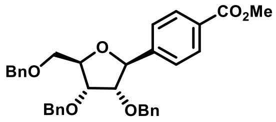

Following **General Procedure I** with Bn-protected ribose hydrazide **89** (0.1 mmol) and methyl 4-iodobenzoate, purification through flash column chromatography afforded product **SD4** (29.1 mg, 54% yield, β:α > 19:1).

$[\alpha]_D^{20}$ = –31.4° (c = 0.5, $CHCl_3$)

^{1}H NMR (600 MHz, $CDCl_3$) δ 7.94 (d, *J* = 8.3 Hz, 2H), 7.44 (d, *J* = 8.0 Hz, 2H), 7.34 – 7.25 (m, 10H), 7.24 – 7.22 (m, 3H), 7.16 – 7.13 (m, 2H), 5.03 (d, *J* = 6.9 Hz, 1H), 4.61 – 4.55 (m, 3H), 4.52 (d, *J* = 11.9 Hz, 1H), 4.47 (d, *J* = 12.0 Hz, 1H), 4.40 (d, *J* = 12.0 Hz, 1H), 4.34 (q, *J* = 4.1 Hz, 1H), 3.98 (t, *J* = 4.4 Hz, 1H), 3.89 (s, 3H), 3.75 (dd, *J* = 7.0, 5.2 Hz, 1H), 3.65 (dd, *J* = 10.4, 4.0 Hz, 1H), 3.60 (dd, *J* = 10.3, 3.7 Hz, 1H).

^{13}C NMR (151 MHz, $CDCl_3$) δ 167.2, 145.9, 138.1, 137.9, 137.7, 129.7, 129.6, 128.6, 128.5, 128.3, 128.0, 127.9, 127.9, 127.9, 126.3, 83.9, 82.2, 82.1, 77.5, 73.6, 72.5, 72.1, 70.5, 52.2.

HRMS (m/z): (ESI) calc. $C_{34}H_{35}O_6$ $[M+H]^+$: 539.2434 found 539.2440

Compound SD6

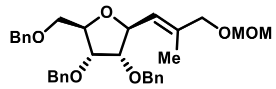

Following **General Procedure I** with Bn-protected ribose hydrazide **89** (0.1 mmol) and (E)-1-iodo-3-(methoxymethoxy)-2-methylprop-1-ene, purification through flash column chromatography afforded product **SD6** (18.7 mg, 36% yield, β:α > 19:1).

$[\boldsymbol{\alpha}]_{\mathbf{D}}^{\mathbf{20}}$ = —1.0 ° (c = 0.1, $CHCl_3$)

^{1}H NMR (600 MHz, $CDCl_3$) δ 7.34 – 7.26 (m, 15H), 5.42 (dq, *J* = 8.9, 1.3 Hz, 1H), 4.77 (dd, *J* = 8.9, 6.6 Hz, 1H), 4.62 – 4.55 (m, 5H), 4.55 (d, *J* = 6.8 Hz, 1H), 4.53 (d, *J* = 6.7 Hz, 1H), 4.49 (d, *J* = 12.0 Hz, 1H), 4.19 (q, *J* = 4.3 Hz, 1H), 3.94 (d, *J* = 1.3 Hz, 2H), 3.92 (dd, *J* = 5.4, 4.2 Hz, 1H), 3.67 (dd, *J* = 6.6, 5.4 Hz, 1H), 3.50 (d, *J* = 4.3 Hz, 2H), 3.36 (s, 3H), 1.76 (d, *J* = 1.4 Hz, 3H).

^{13}C NMR (151 MHz, $CDCl_3$) δ 138.3, 138.2, 138.1, 137.5, 128.50, 128.48, 128.3, 127.91, 127.85, 127.82, 127.77, 127.7, 125.7, 95.7, 82.2, 81.7, 77.8, 77.4, 73.6, 72.4, 72.3, 72.1, 70.5, 55.5, 14.8.

HRMS (m/z): (ESI) calc. $C_{32}H_{38}NaO_6$ $[M+Na]^+$: 541.2566 found 541.2578

Compound SD7

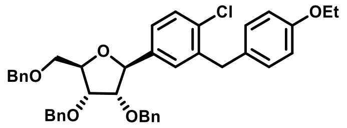

Following **General Procedure I** with Bn-protected ribose hydrazide **89** (0.1 mmol) and **SG2**, purification through flash column chromatography afforded product **SD7** (36.3 mg, 56% yield, β:α > 19:1).

$[\boldsymbol{\alpha}]_{\mathbf{D}}^{\mathbf{20}}$ = –13.6° (c = 0.8, $CHCl_3$)

^{1}H NMR (600 MHz, $CDCl_3$) δ 7.42 – 7.30 (m, 15H), 7.27 – 7.25 (m, 1H), 7.21 (dd, *J* = 7.3, 2.2 Hz, 2H), 7.13 – 7.09 (m, 2H), 6.86 – 6.82 (m, 2H), 5.00 (d, *J* = 6.9 Hz, 1H), 4.69 – 4.63 (m, 2H), 4.62 (d, *J* = 12.0 Hz, 1H), 4.58 (d, *J* = 12.0 Hz, 1H), 4.53 (d, *J* = 12.0 Hz, 1H), 4.46 (d, *J* = 12.0 Hz, 1H), 4.39 (q, *J* = 3.9 Hz, 1H), 4.07 – 4.01 (m, 5H), 3.80 (dd, *J* = 7.0, 5.2 Hz, 1H), 3.69 (dd, *J* = 10.4, 4.1 Hz, 1H), 3.64 (dd, *J* = 10.4, 3.8 Hz, 1H), 1.46 (t, *J* = 7.0 Hz, 3H).

^{13}C NMR (151 MHz, $CDCl_3$) δ 157.5, 139.3, 139.0, 138.2, 138.0, 137.7, 133.4, 131.5, 129.9, 129.5, 129.1, 128.5, 128.4, 128.2, 128.0, 127.9, 127.79, 127.68, 127.7, 125.5, 114.5, 83.8, 82.0, 81.9, 77.4, 73.6, 72.4, 72.0, 70.5, 63.5, 38.5, 15.0.

HRMS (m/z): (ESI) calc. $C_{41}H_{41}ClNaO_5$ $[M+Na]^+$: 671.2540 found 671.2564

Compound SD8

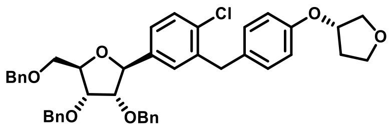

Following **General Procedure I** with Bn-protected ribose hydrazide **89** (0.1 mmol) and (S)-3-(4-(5-bromo-2-chlorobenzyl)phenoxy)tetrahydrofuran, purification through flash column chromatography afforded product **SD8** (44.2 mg, 64% yield, β:α > 19:1).

$[\boldsymbol{\alpha}]_{\mathbf{D}}^{\mathbf{20}}$ = –19.3° (c = 0.4, $CHCl_3$)

^{1}H NMR (600 MHz, $CDCl_3$) δ 7.34 – 7.22 (m, 14H), 7.20 (dq, *J* = 4.5, 2.2 Hz, 2H), 7.15 – 7.11 (m, 2H), 7.06 – 7.02 (m, 2H), 6.75 – 6.71 (m, 2H), 4.92 (d, *J* = 7.0 Hz, 1H), 4.84 (ddt, *J* = 6.5, 4.5, 2.1 Hz, 1H), 4.61 – 4.56 (m, 2H), 4.54 (d, *J* = 12.0 Hz, 1H), 4.50 (d, *J* = 12.0 Hz, 1H), 4.45 (d, *J* = 12.1 Hz, 1H), 4.38 (d, *J* = 12.0 Hz, 1H), 4.31 (q, *J* = 3.8 Hz, 1H), 4.00 – 3.92 (m, 6H), 3.88 (td, *J* = 8.3, 4.4 Hz, 1H), 3.73 (dd, *J* = 7.1, 5.2 Hz, 1H), 3.62 (dd, *J* = 10.4, 4.1 Hz, 1H), 3.57 (dd, *J* = 10.4, 3.8 Hz, 1H), 2.19 – 2.08 (m, 2H).

^{13}C NMR (151 MHz, $CDCl_3$) δ 155.9, 139.4, 138.8, 138.2, 137.9, 137.7, 133.4, 132.0, 130.1, 129.6, 129.2, 128.5, 128.4, 128.3, 128.0, 127.88, 127.86, 127.8, 127.7, 125.6, 115.4, 83.8, 82.1, 81.9, 77.4, 73.6, 73.3, 72.4, 72.0, 70.5, 67.3, 38.5, 33.1.

HRMS (m/z): (ESI) calc. $C_{43}H_{43}ClNaO_6$ $[M+Na]^+$: 713.2646 found 713.2639

Compound SD9

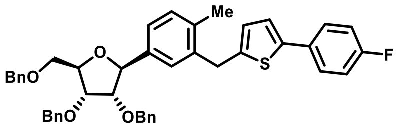

Following **General Procedure I** with Bn-protected ribose hydrazide **89** (0.1 mmol) and **SG4**, purification through flash column chromatography afforded product **SD9** (30.1 mg, 44% yield, β:α > 19:1).

$[\alpha]_D^{20}$ = –8.1° (c = 0.8, $CHCl_3$)

^{1}H NMR (600 MHz, $CDCl_3$) δ 7.46 – 7.42 (m, 2H), 7.34 – 7.26 (m, 11H), 7.25 – 7.22 (m, 4H), 7.21 – 7.18 (m, 2H), 7.12 (d, *J* = 7.8 Hz, 1H), 7.02 – 6.97 (m, 3H), 6.62 (d, *J* = 3.5 Hz, 1H), 5.01 (d, *J* = 6.6 Hz, 1H), 4.62 (d, *J* = 12.0 Hz, 1H), 4.58 (d, *J* = 11.4 Hz, 2H), 4.53 (d, *J* = 12.0 Hz, 1H), 4.48 (s, 2H), 4.34 (q, *J* = 4.1 Hz, 1H), 4.06 (s, 2H), 4.02 (dd, *J* = 5.2, 4.1 Hz, 1H), 3.83 (dd, *J* = 6.6, 5.2 Hz, 1H), 3.67 (dd, *J* = 10.4, 4.2 Hz, 1H), 3.63 (dd, *J* = 10.4, 4.0 Hz, 1H), 2.31 (s, 3H).

^{13}C NMR (151 MHz, $CDCl_3$) δ 162.2 (d, $^1J_{C,F}$ = 246.6 Hz), 143.7, 141.5, 138.4, 138.3, 138.2, 138.1, 138.0, 136.0, 131.0 (d, $^3J_{C,F}$ = 3.3 Hz), 130.6, 128.5, 128.4, 128.2, 127.9, 127.74, 127.71, 127.68, 127.2 (d, $^2J_{C,F}$ = 8.1 Hz), 126.0, 125.1, 122.8, 115.8 (d, $^2J_{C,F}$ = 21.7 Hz), 83.9, 82.6, 81.8, 77.6, 73.6, 72.4, 72.0, 70.6, 34.3, 19.4.

HRMS (m/z): (ESI) calc. $C_{44}H_{42}FO_4S$ $[M+H]^+$: 685.2788 found 685.2767

Compound SD10

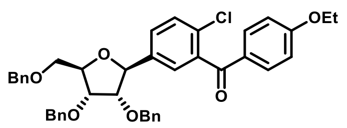

Following **General Procedure I** with Bn-protected ribose hydrazide **89** (0.1 mmol) and **SG1**, purification through flash column chromatography afforded product **SD10** (34.5 mg, 52% yield, β:α = 14.4:1).

$[\alpha]_D^{20}$ = –45.2° (c = 0.5, $CHCl_3$)

^{1}H NMR (600 MHz, $CDCl_3$) δ 7.77 – 7.72 (m, 2H), 7.44 (ddd, *J* = 8.4, 2.2, 0.6 Hz, 1H), 7.38 (d, *J* = 2.1 Hz, 1H), 7.36 – 7.27 (m, 9H), 7.26 – 7.21 (m, 5H), 7.18 – 7.16 (m, 2H), 6.90 – 6.86 (m, 2H), 5.00 (d, *J* = 7.0 Hz, 1H), 4.59 (d, *J* = 1.7 Hz, 2H), 4.52 (d, *J* = 11.9 Hz, 1H), 4.51 – 4.45 (m, 2H), 4.41 (d, *J* = 11.9 Hz, 1H), 4.33 (q, *J* = 3.8 Hz, 1H), 4.09 (q, *J* = 7.0 Hz, 2H), 3.99 (dd, *J* = 5.1, 3.6 Hz, 1H), 3.76 (dd, *J* = 7.0, 5.1 Hz, 1H), 3.61 (dd, *J* = 10.4, 4.1 Hz, 1H), 3.56 (dd, *J* = 10.4, 3.8 Hz, 1H), 1.44 (t, *J* = 7.0 Hz, 3H).

^{13}C NMR (151 MHz, $CDCl_3$) δ 193.9, 163.6, 139.6, 138.9, 138.0, 137.9, 137.6, 132.7, 130.4, 129.9, 129.3, 128.8, 128.6, 128.56, 128.54, 128.50, 128.02, 127.97, 127.8, 127.7, 126.7, 114.4, 83.9, 82.2, 81.5, 77.4, 73.5, 72.6, 72.1, 70.3, 64.0, 14.8.

HRMS (m/z): (ESI) calc. $C_{41}H_{40}ClO_6$ $[M+H]^+$: 663.2513 found 663.2520

Compound SD11

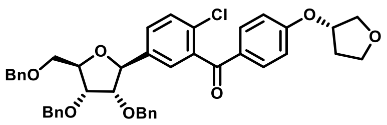

Following **General Procedure I** with Bn-protected ribose hydrazide **89** (0.1 mmol) and **SG24**, purification through flash column chromatography afforded product **SD11** (43.7 mg, 62% yield, β:α = 15.9:1).

$[\alpha]_D^{20}$ = –34.3 ° (c = 0.6, $CHCl_3$)

^{1}H NMR (600 MHz, $CDCl_3$) δ 7.75 (d, *J* = 8.8 Hz, 2H), 7.45 (dd, *J* = 8.3, 2.2 Hz, 1H), 7.40 – 7.19 (m, 15H), 7.17 (dd, *J* = 6.6, 3.0 Hz, 2H), 6.86 (d, *J* = 8.9 Hz, 2H), 5.01 – 4.94 (m, 2H), 4.59 (s, 2H), 4.52 (d, *J* = 11.9 Hz, 1H), 4.50 – 4.44 (m, 2H), 4.40 (d, *J* = 11.9 Hz, 1H), 4.33 (q, *J* = 3.8 Hz, 1H), 4.04 – 3.96 (m, 4H), 3.91 (td, *J* = 8.4, 4.2 Hz, 1H), 3.76 (dd, *J* = 7.1, 5.1 Hz, 1H), 3.61 (dd, *J* = 10.4, 4.1 Hz, 1H), 3.55 (dd, *J* = 10.3, 3.8 Hz, 1H), 2.28 – 2.19 (m, 1H), 2.16 – 2.10 (m, 1H).

^{13}C NMR (151 MHz, $CDCl_3$) δ 193.8, 162.0, 139.6, 138.7, 138.0, 137.8, 137.6, 132.7, 130.4, 130.0, 129.7, 128.8, 128.6, 128.54, 128.50, 128.3, 128.03, 128.01, 127.98, 127.8, 127.7, 126.7, 115.1, 84.0, 82.3, 81.5, 77.8, 77.4, 73.5, 73.1, 72.6, 72.1, 70.4, 67.3, 33.1.

HRMS (m/z): (ESI) calc. $C_{43}H_{42}ClO_7$ $[M+H]^+$: 705.2619 found 705.2618

Compound SD12

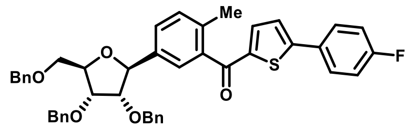

Following **General Procedure I** with Bn-protected ribose hydrazide **89** (0.1 mmol) and **SG23**, purification through flash column chromatography afforded product **SD12** (38.4 mg, 55% yield, β:α = 11.2:1).

$[\alpha]_D^{20}$ = 54.5° (c = 0.2, $CHCl_3$)

^{1}H NMR (600 MHz, $CDCl_3$) δ 7.62 – 7.58 (m, 2H), 7.52 (d, *J* = 1.8 Hz, 1H), 7.44 (dd, *J* = 7.9, 1.9 Hz, 1H), 7.35 – 7.26 (m, 9H), 7.25 – 7.20 (m, 6H), 7.18 (dd, *J* = 7.0, 2.6 Hz, 2H), 7.13 – 7.08 (m, 3H), 5.03 (d, *J* = 6.9 Hz, 1H), 4.62 – 4.57 (m, 2H), 4.54 – 4.46 (m, 3H), 4.43 (d, *J* = 12.0 Hz, 1H), 4.34 (q, *J* = 3.9 Hz, 1H), 4.01 (dd, *J* = 5.2, 3.7 Hz, 1H), 3.80 (dd, *J* = 7.0, 5.1 Hz, 1H), 3.63 (dd, *J* = 10.4, 4.1 Hz, 1H), 3.58 (dd, *J* = 10.4, 3.9 Hz, 1H), 2.40 (s, 3H).

^{13}C NMR (151 MHz, $CDCl_3$) δ 190.2, 163.4 (d, $^1J_{C,F}$ = 249.9 Hz), 152.6, 143.6, 138.1, 138.0, 137.9, 137.8, 137.7, 136.8, 136.3, 131.3, 129.8 (d, $^3J_{C,F}$ = 3.4 Hz), 128.54, 128.53, 128.48, 128.4, 128.42, 128.30, 128.0, 127.98, 127.95, 127.8, 127.6, 126.2, 124.1, 116.4 (d, $^2J_{C,F}$ = 22.0 Hz), 84.0, 82.1, 82.0, 73.5, 72.5, 72.1, 70.5, 19.6.

HRMS (m/z): (ESI) calc. $C_{44}H_{40}FO_5S$ $[M+H]^+$: 699.2580 found 699.2582

Compound SD13

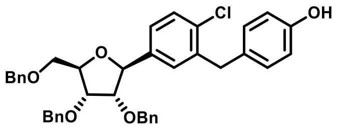

Following **General Procedure I** with Bn-protected ribose hydrazide **89** (0.1 mmol) and 4-(5-bromo-2-chlorobenzyl)phenol, purification through flash column chromatography afforded product **SD13** (36.6 mg, 59% yield, β:α > 19:1).

$[\boldsymbol{\alpha}]_{\mathbf{D}}^{\mathbf{20}}$ = –16.0° (c = 1.0, $CHCl_3$)

^{1}H NMR (600 MHz, $CDCl_3$) δ 7.33 – 7.21 (m, 14H), 7.19 – 7.15 (m, 2H), 7.13 – 7.09 (m, 2H), 6.97 – 6.94 (m, 2H), 6.67 – 6.63 (m, 2H), 4.92 (d, *J* = 7.1 Hz, 2H), 4.91 (s, 2H), 4.60 – 4.55 (m, 2H), 4.53 (d, *J* = 12.0 Hz, 1H), 4.49 (d, *J* = 12.0 Hz, 1H), 4.44 (d, *J* = 12.1 Hz, 1H), 4.37 (d, *J* = 12.1 Hz, 1H), 4.31 (q, *J* = 3.8 Hz, 1H), 3.97 (dd, *J* = 5.2, 3.6 Hz, 1H), 3.93 (d, *J* = 3.0 Hz, 2H), 3.72 (dd, *J* = 7.0, 5.2 Hz, 1H), 3.60 (dd, *J* = 10.4, 4.1 Hz, 1H), 3.55 (dd, *J* = 10.4, 3.8 Hz, 1H).

^{13}C NMR (151 MHz, $CDCl_3$) δ 154.1, 139.2, 138.9, 138.1, 137.9, 137.7, 133.5, 131.6, 130.1, 129.6, 129.1, 128.5, 128.4, 128.3, 128.0, 127.91, 127.87, 127.8, 127.7, 125.6, 115.4, 83.8, 82.0, 81.9, 77.4, 73.6, 72.4, 72.0, 70.5, 38.5.

HRMS (m/z): (ESI) calc. $C_{39}H_{37}ClNaO_5$ $[M+Na]^+$: 643.2227 found 643.2228

7. Applications

7.1 Summary of Previous Synthetic Routes

Salmochelin-SX (72) Synthesis[26]

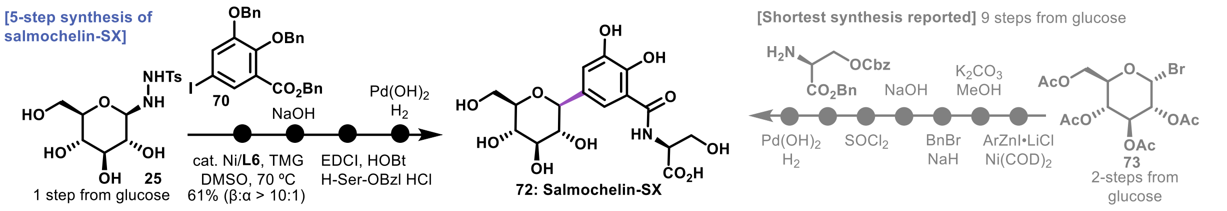

Neopetrosin C (75) Synthesis[27,28]

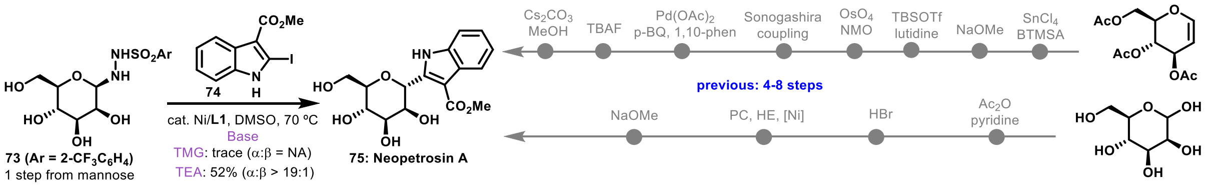

a-C-Mannosyltryptophan (77) Synthesis[29-33]

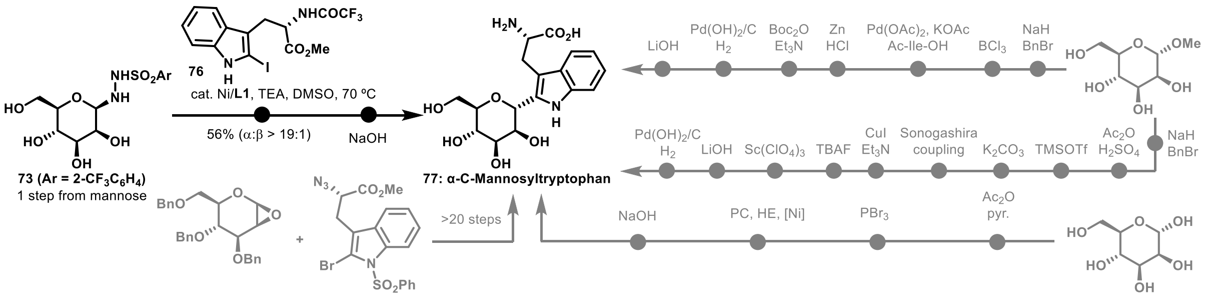

IMPDH inhibitor (80) Synthesis[34,35]

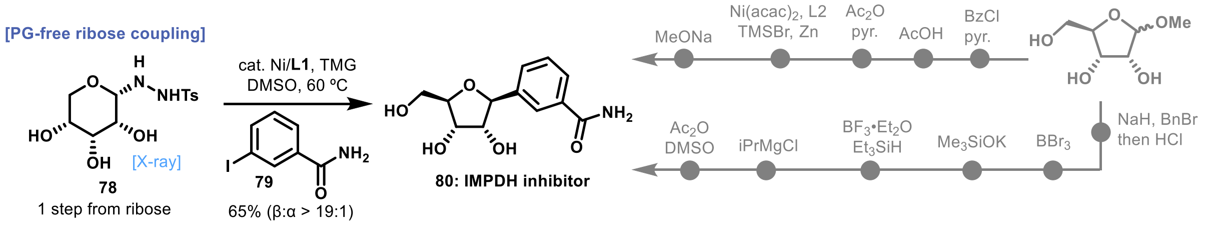

7.2 Synthesis of Salmochelin-SX

Aryl iodide 70

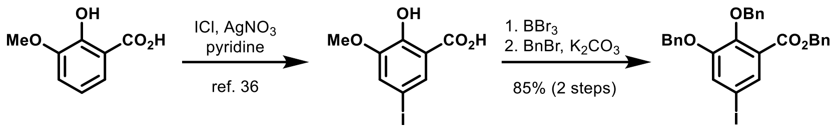

The first step was carried out according to a reported literature procedure. [36]

To a stirred solution of aryl iodide **SI1** (1.0 g, 3.4 mmol, 1.0 equiv.) in DCM (6.8 mL) was added BBr_3 in DCM (7.48 mL, 7.48 mmol, 2.2 equiv.) at –78 °C. After stirring for 12 h, the reaction was quenched by addition of methanol and directly concentrated under reduced pressure. The crude product was used without further purification.

To a stirred solution of phenol **SI2** in Acetone (7 mL) was added BnBr (3.7 mL, 20.4 mmol, 6.0 equiv.) and K_2CO_3 (2.8 mg, 20.4 mmol, 6.0 equiv.). The reaction mixture was heated to reflux. After stirring for 12 h, the reaction was cooled to room temperature and filtered. The filtrate was concentrated under reduced pressure. The residue was purified by flash column chromatography (silica gel, Hexane/EtOAc 100:1 to 10:1) to afford aryl iodide **70** (1.59 g, 2.89 mmol, 85% yield, 2 steps) as a colorless oil.

^{1}H NMR (600 MHz, $CDCl_3$) δ 7.7 (d, J = 2.1 Hz, 1H), 7.5 – 7.4 (m, 11H), 7.3 – 7.2 (m, 5H), 5.3 (s, 2H), 5.1 (s, 2H), 5.0 (s, 2H).

^{13}C NMR (151 MHz, $CDCl_3$) δ 164.8, 153.6, 148.4, 137.0, 135.9, 135.7, 131.6, 128.81, 128.75, 128.6, 128.51, 128.49, 128.4, 128.2, 127.9, 126.6, 86.5, 75.8, 71.6, 67.4.

HRMS (m/z): (ESI) calc. $C_{28}H_{24}IO_4$ $[M+H]^+$: 550.0641 found 550.0622

Compound 71

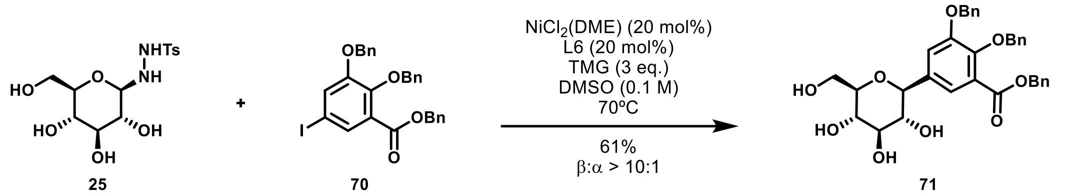

Following **General Procedure B** with glucosyl hydrazide **25** (0.5 mmol), aryl iodide **70**, **L6**, and base TMG, purification by reverse phase column chromatography afforded the title compound **71** (179 mg, 61% yield, β:α > 10:1) as colorless oil.

$[\alpha]_D^{20}$ = + 4.7° (c = 1.0, MeOH)

^{1}H NMR (600 MHz, CD_3OD) δ 7.50 – 7.44 (m, 3H), 7.43 – 7.38 (m, 3H), 7.38 – 7.30 (m, 6H), 7.25 – 7.17 (m, 5H), 5.28 (s, 2H), 5.22 – 5.11 (m, 2H), 4.96 (s, 2H), 4.15 (d, *J* = 9.4 Hz, 1H), 3.90 (dd, *J* = 12.1, 2.2 Hz, 1H), 3.74 (dd, *J* = 12.0, 5.4 Hz, 1H), 3.51 – 3.43 (m, 2H), 3.43 – 3.39 (m, 1H), 3.36 – 3.32 (m, 1H).

^{13}C NMR (151 MHz, CD_3OD) δ 167.9, 153.8, 148.5, 138.6, 138.1, 137.43, 137.40, 129.7, 129.59, 129.57, 129.5, 129.3, 129.2, 129.1, 128.9, 127.3, 122.8, 118.3, 82.8, 82.2, 79.7, 76.6, 76.4, 72.3, 71.7, 68.1, 63.0.

HRMS (m/z): (ESI) calcd. for $C_{34}H_{35}O_9$ $[M+H]^+$: 586.2203 found 586.2208

Compound SI4

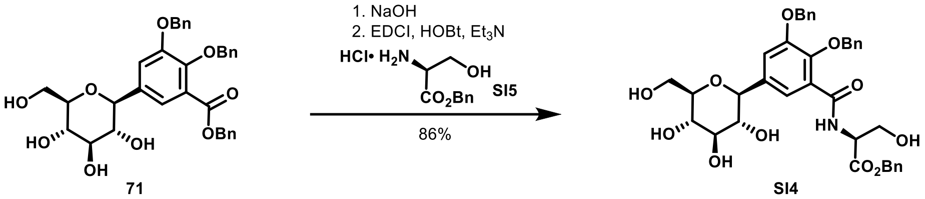

To a stirred solution of compound **71** (146 mg, 0.25 mmol, 1.0 equiv.) in THF/MeOH/H_2O (1:1:1) was added NaOH (20 mg, 0.5 mmol, 2.0 equiv.). After stirring for 5 h, the reaction mixture was concentrated under reduced pressure to remove MeOH and THF. Then, 1 M HCl (0.55 mL) was added to neutralize the reaction mixture which was purified by C18 column (RediSep Rf reversed phase C18 column 30g, eluting with a gradient flow over 12 minutes of 100:0 A/B to 0:100 A/B, 214 nm UV detection). Solvent A is H_2O with 0.1% formic acid; solvent B is MeCN. At a flow rate of 35 mL/min, desired product has a retention time of 8 minutes. The fractions with the desired product were collected and concentrated under reduced pressure to afford carboxylic acid product.

To a stirred solution of the carboxylic acid (60 mg, 0.12 mmol, 1.0 equiv.) in DMF (1mL) was added H-Ser-OBzl·HCl (37.3 mg, 0.24 mmol, 2.0 equiv.), HOBt·H_2O (16.2 mg, 0.12 mmol, 1.0 equiv.) and Et_3N (66.7 μL, 0.48 mmol, 4.0 equiv.). Then the reaction mixture was cooled to 0 °C and EDCI (46 mg, 0.24 mmol, 2.0 equiv.) was added to the reaction mixture. After stirring for 10 h, the reaction mixture was purified by C18 column (RediSep Rf reversed phase C18 column 30g, eluting with a gradient flow over 12 minutes of 100:0 A/B to 0:100 A/B, 214 nm UV detection). Solvent A is H_2O with 0.1% formic acid; solvent B is MeCN. At a flow rate of 35 mL/min, desired product has a retention time of 8 minutes. The fractions with the desired product were collected and concentrated under reduced pressure to afford product **SI4** (69.5 mg, 0.1 mmol, 86% yield) as colorless oil.

$[\alpha]_D^{20}$ = + 16.8° (c = 0.5, MeOH)

^{1}H NMR (600 MHz, CD_3OD) δ 7.61 (d, *J* = 2.0 Hz, 1H), 7.50 (dt, *J* = 5.9, 1.5 Hz, 2H), 7.45 (d, *J* = 2.0 Hz, 1H), 7.42 – 7.35 (m, 5H), 7.35 – 7.29 (m, 5H), 7.24 (t, *J* = 7.3 Hz, 1H), 7.20 – 7.16 (m, 2H), 5.23 – 5.17 (m, 4H), 5.08 (d, *J* = 10.4 Hz, 1H), 5.03 (d, *J* =

10.3 Hz, 1H), 4.71 (t, *J* = 4.0 Hz, 1H), 4.16 (d, *J* = 9.4 Hz, 1H), 3.95 (dd, *J* = 11.2, 4.1 Hz, 1H), 3.90 (dd, *J* = 12.0, 2.2 Hz, 1H), 3.82 (dd, *J* = 11.2, 3.9 Hz, 1H), 3.74 (dd, *J* = 12.1, 5.4 Hz, 1H), 3.50 (t, *J* = 8.6 Hz, 1H), 3.46 (t, *J* = 8.9 Hz, 1H), 3.44 – 3.39 (m, 1H), 3.36 – 3.32 (m, 1H).

^{13}C NMR (151 MHz, CD_3OD) δ 171.7, 167.8, 153.2, 147.6, 138.0, 137.7, 137.5, 137.1, 130.4, 129.7, 129.6, 129.4, 129.30, 129.26, 129.2, 127.5, 122.5, 118.1, 82.9, 82.1, 79.7, 77.0, 76.5, 72.4, 71.7, 68.2, 63.0, 62.7, 56.8.

HRMS (m/z): (ESI) calcd. for $C_{37}H_{40}NO_{11}$ [M+H]$^+$: 674.2596, found 674.2592.

Salmochelin-SX (72)

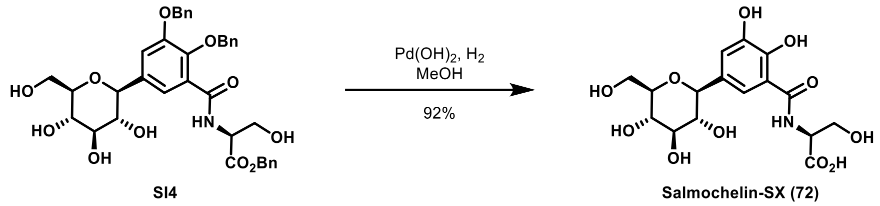

To a stirred solution of compound **SI4** (3 mg, 0.0045 mmol, 1 equiv.) in MeOH (0.5 mL) was added 20 wt.% $Pd(OH)_2$ (3.2 mg, 0.0045 mmol, 1.0 equiv.). The reaction mixture was bubbled with hydrogen balloon for 3 minutes. Then the reaction was stirred under hydrogen atmosphere for 10 hours. The reaction suspension was filtered through Celite and washed with MeOH. The organic solution was collected, and the solvent was removed under reduced pressure to afford salmochelin-SX (**72**) (1.5 mg, 0.00369 mmol, 82%) as light-yellow oil.

$[\boldsymbol{\alpha}]_{\boldsymbol{D}}^{\mathbf{20}}$ = + 20.0° (c = 0.1, MeOH)

^{1}H NMR (600 MHz, CD_3OD) δ 7.44 (d, *J* = 1.9 Hz, 1H), 7.04 (d, *J* = 1.9 Hz, 1H), 4.70 (t, *J* = 4.3 Hz, 1H), 4.05 (d, *J* = 9.6 Hz, 1H), 4.03 – 4.00 (m, 1H), 3.96 (dd, *J* = 11.3, 3.8 Hz, 1H), 3.87 (dd, *J* = 12.0, 2.2 Hz, 1H), 3.72 (dd, *J* = 12.0, 5.4 Hz, 1H), 3.45 (p, *J* = 8.8 Hz, 2H), 3.41 – 3.34 (m, 2H).

^{13}C NMR (151 MHz, CD_3OD) δ 173.9, 170.8, 149.6, 146.9, 131.4, 119.5, 118.9, 116.5, 82.2, 82.1, 79.7, 76.3, 71.8, 63.0, 56.7, 49.6.

HRMS (m/z): (ESI) calcd. for $C_{16}H_{22}NO_{11}$ $[M+H]^+$: 404.1193, found 404.1193.

Note: The NMR spectra is consistent with the literature reported data. [26]

7.3 Synthesis of Neopetrosin C

2-Iodoindole 74

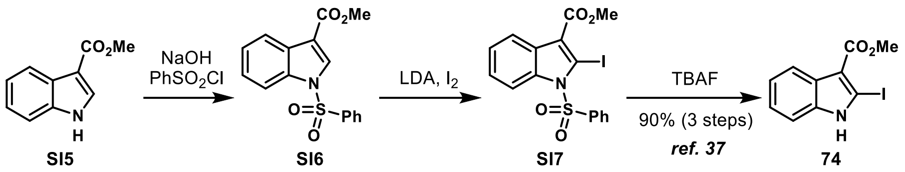

The coupling partner was prepared according to the literature procedure.[37]

^{1}H NMR (600 MHz, CD_3OD) δ 8.02 – 7.97 (m, 1H), 7.37 – 7.33 (m, 1H), 7.16 – 7.09 (m, 2H), 3.91 (s, 3H).

^{13}C NMR (151 MHz, CD_3OD) δ 166.7, 140.5, 128.2, 123.8, 122.5, 121.7, 112.0, 111.8, 88.4, 51.3.

HRMS (m/z): (ESI) calcd. for $C_{10}H_9INO_2$ $[M+H]^+$: 301.9678, found 301.9682.

Neopetrosin C (75)

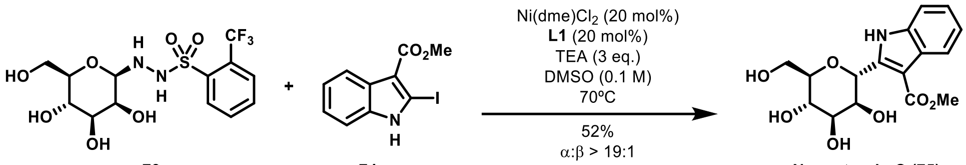

Following **General Procedure B** with mannosyl sulfonylhydrazide **73** (0.1 mmol), aryl 2-iodoindole **74**, **L1**, and base TEA, purification by reverse phase column chromatography afforded neopetrosin C (**75**) (18 mg, 52% yield, α:β > 19:1) as a white solid.

$[\alpha]_D^{20}$ = 16.4° (c = 0.5, MeOH)

NMR in CD_3OD:

^{1}H NMR (600 MHz, CD_3OD) δ 8.03 – 7.98 (m, 1H), 7.43 (dt, *J* = 8.1, 1.0 Hz, 1H), 7.17 (dddd, *J* = 20.1, 8.1, 7.1, 1.3 Hz, 2H), 5.87 (d, *J* = 9.1 Hz, 1H), 4.28 (dd, *J* = 12.2, 8.3 Hz, 1H), 4.13 (dd, *J* = 9.1, 3.0 Hz, 1H), 3.98 (ddd, *J* = 8.2, 4.1, 2.2 Hz, 1H), 3.96 – 3.94 (m, 1H), 3.93 (s, 4H), 3.74 (dd, *J* = 12.2, 4.1 Hz, 1H).

^{13}C NMR (151 MHz, CD_3OD) δ 167.4, 145.2, 135.5, 126.4, 122.3, 121.1, 120.9, 111.3, 104.6, 80.3, 71.0, 69.5, 69.2, 65.9, 59.7, 50.1.

NMR in DMSO-d_6:

^{1}H NMR (600 MHz, DMSO-d_6) δ 11.88 (s, 1H), 7.94 (dd, *J* = 7.6, 1.4 Hz, 1H), 7.48 (d, *J* = 7.8 Hz, 1H), 7.20 – 7.11 (m, 2H), 5.69 (d, *J* = 9.6 Hz, 1H), 5.11 (s, 1H), 5.04 (d, *J* = 4.1 Hz, 1H), 4.49 (t, *J* = 5.5 Hz, 1H), 4.33 (d, *J* = 8.3 Hz, 1H), 4.03 (ddd, *J* = 10.5, 8.3, 2.9 Hz, 1H), 3.92 (ddd, *J* = 11.1, 7.5, 2.8 Hz, 1H), 3.83 – 3.81 (m, 4H), 3.81 – 3.74 (m, 2H), 3.68 (dt, *J* = 10.7, 4.8 Hz, 1H).

^{13}C NMR (151 MHz, DMSO-d_6) δ 165.8, 145.8, 135.3, 126.1, 122.2, 121.0, 120.8, 112.1, 104.4, 80.3, 71.3, 69.3, 68.1, 64.5, 59.5, 50.7.

HRMS (m/z): (ESI) calcd. for $C_{16}H_{20}NO_7$ $[M+H]^+$: 338.1240, found 338.1237.

Note: The NMR spectra is consistent with the literature reported data (DMSO-d_6). [27]

7.4 Synthesis of a-C-Mannosyltryptophan

2-idodoindole 76

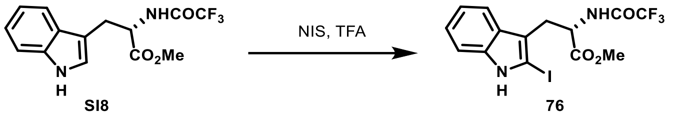

To a round-bottom flask with equipped with a stir bar was added indole **SI8** (315 mg, 1 mmol, 1 equiv.) dissolved in DCM (33 mL) and TFA (3.3 mL). N-Iodosuccinimide (NIS, 225 mg, 1 mmol, 1 equiv.) was then added. The mixture was stirred at room temperature for 4 hours and concentrated under reduced pressure. The crude mixture was purified by flash column chromatography (silica gel, Hexane/EtOAc 10:1 to 1:1) to afford iodoindole **76** (180 mg, 0.41 mmol, 41%) as a yellow solid.

$[\alpha]_D^{25}$ = +37.0° (c = 0.5, $CHCl_3$)

^{1}H NMR (600 MHz, $CDCl_3$) δ 8.16 (s, 1H), 7.48 (dd, J = 7.9, 1.2 Hz, 1H), 7.33 – 7.30 (m, 1H), 7.16 (ddd, J = 8.2, 7.1, 1.2 Hz, 1H), 7.11 (ddd, J = 8.0, 7.0, 1.1 Hz, 1H), 6.90 (d, J = 7.9 Hz, 1H), 4.93 (dt, J = 8.1, 5.9 Hz, 1H), 3.74 (s, 3H), 3.32 (d, J = 5.9 Hz, 2H).

^{13}C NMR (151 MHz, $CDCl_3$) δ 170.8, 156.9 (q, $^2J_{C,F}$ = 37.4 Hz), 138.9, 127.5, 123.0, 120.6, 117.7, 115.7 (q, $^1J_{C,F}$ = 287.8 Hz), 110.7, 79.7, 53.1, 29.4.

HRMS (m/z): (ESI) calc. $C_{14}H_{13}F_3IN_2O_3$ $[M+H]^+$: 440.9923 found 440.9912

Compound SI9

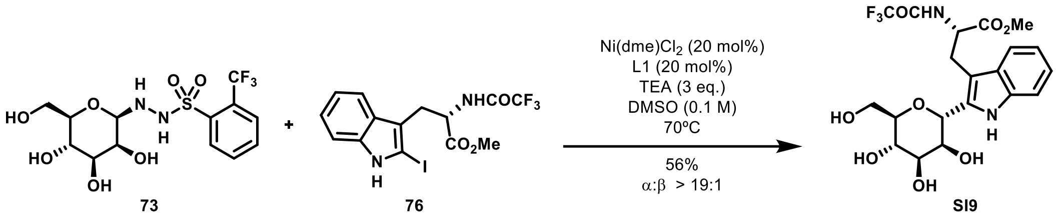

Following **General Procedure B** with mannosyl sulfonylhydrazide **73** (0.2 mmol), 2-idodoindole **76**, **L1**, and base TEA, purification by reverse phase column chromatography afforded the title compound **SI9** (53 mg, 56% yield, α:β > 19:1) as a colorless oil.

$[\alpha]_D^{20}$ = + 41.0° (c = 1.0, MeOH)

^{1}H NMR (600 MHz, CD_3OD) δ 7.52 (d, J = 7.9 Hz, 1H), 7.33 (d, J = 8.1 Hz, 1H), 7.13 – 7.07 (m, 1H), 7.05 – 6.99 (m, 1H), 5.11 (d, J = 8.0 Hz, 1H), 4.81 (dd, J = 9.4, 6.5 Hz, 1H), 4.23 (dd, J = 8.0, 3.3 Hz, 1H), 4.14 (dd, J = 11.9, 7.4 Hz, 1H), 3.98 (dd, J = 5.1, 3.3 Hz, 1H), 3.90 (dd, J = 5.2, 3.3 Hz, 1H), 3.83 (dt, J = 7.4, 3.6 Hz, 1H), 3.79 (dd, J = 11.9, 4.0 Hz, 1H), 3.67 (s, 3H), 3.47 (dd, J = 14.6, 6.5 Hz, 1H), 3.36 (dd, J = 14.5, 9.5 Hz, 1H).

^{13}C NMR (151 MHz, CD_3OD) δ 173.1, 159.0 (q, $^2J_{C,F}$ = 37.6 Hz), 137.4, 135.3, 129.0, 122.9, 120.1, 119.0, 117.2 (q, $^1J_{C,F}$ = 286.7 Hz), 112.2, 108.7, 80.6, 72.3, 70.6, 69.6, 69.1, 61.5, 55.2, 53.1, 27.0.

HRMS (m/z): (ESI) calcd. for $C_{20}H_{24}F_3N_2O_8$ $[M+H]^+$: 477.1485, found 477.1477.

Compound 77

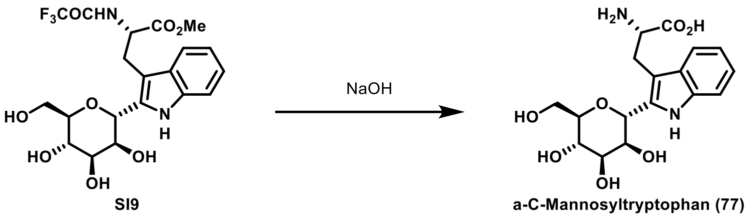

A reported procedure was followed[30]. A 2 M NaOH solution in H_2O (36 μL, 0.0714 mmol, 2 equiv.) was added to a stirred solution of compound **SI9** (17 mg, 0.0357 mmol, 1 equiv.) in THF (0.3 mL) and H_2O (0.3 mL). The reaction mixture was stirred for 2 hours and concentrated under reduced pressure. Purification by preparative reversed-phase HPLC to yield α-C-Mannosyltryptophan (**77**) as a colorless amorphous solid (7.5 mg, 57%).

$[\alpha]_D^{20}$ = + 13.0° (c = 0.1, MeOH)

^{1}H NMR (600 MHz, D_2O) δ 7.71 (d, *J* = 8.0 Hz, 1H), 7.50 (d, *J* = 8.2 Hz, 1H), 7.28 (ddd, *J* = 8.1, 6.9, 1.1 Hz, 1H), 7.21 – 7.16 (m, 1H), 5.15 (d, *J* = 8.3 Hz, 1H), 4.41 (dd, *J* = 8.3, 3.3 Hz, 1H), 4.24 (dd, *J* = 12.6, 9.0 Hz, 1H), 4.10 (dd, *J* = 5.1, 3.3 Hz, 1H), 4.01 (dd, *J* = 9.1, 5.1 Hz, 1H), 3.92 (dd, *J* = 5.1, 3.2 Hz, 1H), 3.87 (dt, *J* = 9.2, 3.2 Hz, 1H), 3.70 (dd, *J* = 12.6, 3.3 Hz, 1H), 3.53 (dd, *J* = 15.3, 5.2 Hz, 1H), 3.34 (dd, *J* = 15.3, 9.1 Hz, 1H).

^{13}C NMR (151 MHz, D_2O) δ 174.3, 135.9, 133.3, 126.9, 122.8, 119.7, 118.6, 111.8, 108.1, 78.9, 70.3, 68.8, 67.5, 65.8, 58.8, 55.0, 25.7.

HRMS (m/z): (ESI) calcd. for $C_{17}H_{23}N_2O_7$ $[M+H]^+$: 367.1505, found 367.1496.

Note: The NMR spectra is consistent with the literature reported data[30].

7.5 Synthesis of IMPDH Inhibitor

IMPDH inhibitor (80)

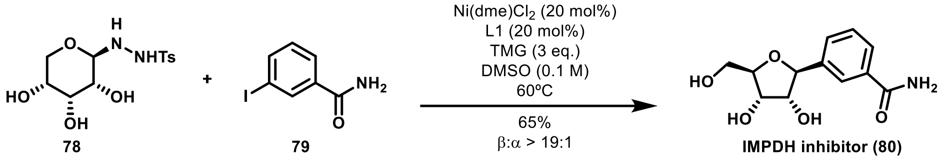

Following **General Procedure B** with ribosyl sulfonylhydrazide **78** (0.1 mmol), aryl iodide **79**, **L1**, and base TEA, purification by reverse phase column chromatography afforded IMPDH inhibitor (**80**) (11 mg, 43% yield, β:α > 19:1) as colorless oil.

$[\alpha]_D^{20}$ = – 20.0° (c = 0.1, MeOH)

^{1}H NMR (600 MHz, DMSO) δ 7.95 (s, 1H), 7.85 (d, *J* = 1.8 Hz, 1H), 7.76 (dd, *J* = 7.7, 1.7 Hz, 1H), 7.57 – 7.53 (m, 1H), 7.40 (t, *J* = 7.6 Hz, 1H), 7.36 (s, 1H), 5.02 (d, *J* = 7.0 Hz, 1H), 4.93 (d, *J* = 4.8 Hz, 1H), 4.82 (t, *J* = 5.6 Hz, 1H), 4.59 (d, *J* = 7.1 Hz, 1H), 3.89 (q, *J* = 4.4 Hz, 1H), 3.82 (q, *J* = 4.3 Hz, 1H), 3.70 (td, *J* = 7.0, 5.2 Hz, 1H), 3.61 – 3.52 (m, 2H).

^{13}C NMR (151 MHz, DMSO) δ 168.0, 141.5, 134.1, 129.2, 127.93, 126.4, 125.5, 85.3, 82.8, 77.6, 71.4, 62.0.

HRMS (m/z): (ESI) calcd. for $C_{12}H_{16}NO_5$ $[M+H]^+$: 254.1028, found 254.1023.

Note: The NMR spectra is consistent with the literature reported data. [38]

8. Gram-Scale Synthesis

Glucose toluenesulfonylhydrazide 27

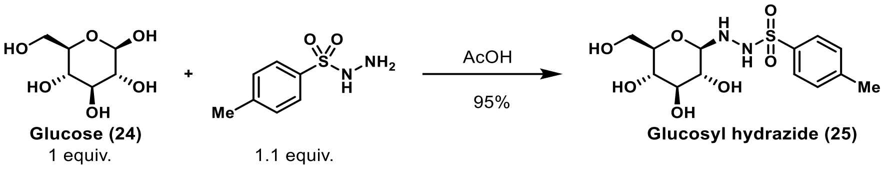

In a dry 1L round-bottom flask, dextrose powder **24** (18 g, 100 mmol, 1.0 equiv.) and *p*-toluenesulfonyl hydrazide (20.5 g, 110 mmol, 1.1 equiv.) were added, followed by 75% vinegar (500 mL). The reaction mixture was stirred for 6 h at room temperature. Upon completion of the reaction, as indicated by TLC. To the suspension diethyl ether (400 mL) was added, and the resulting reaction mixture was stirred for 20 minutes, filtered, and washed with diethyl ether, affording glucosyl hydrazide (**25**) (33.1 g, 95 mmol, 95% yield).

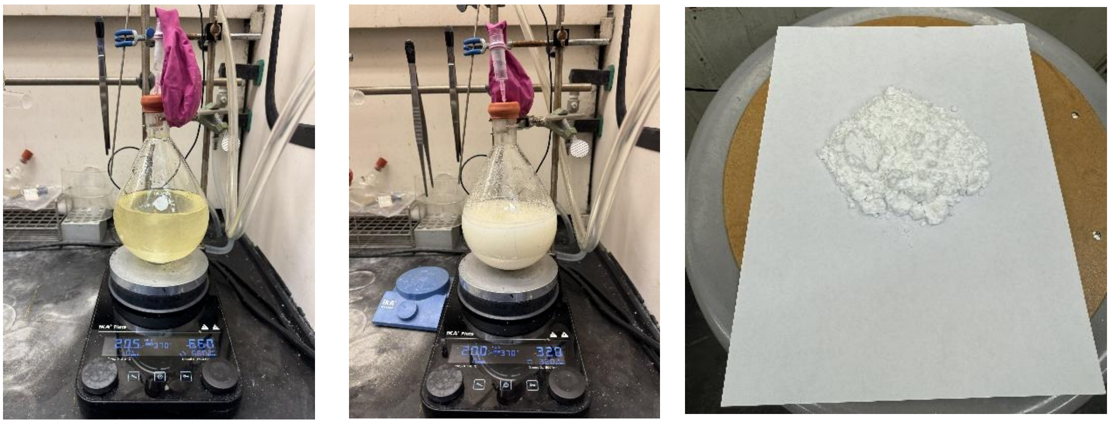

(Left) Reaction mixture at the beginning. **(Center)** Reaction mixture after 6 hours. **(Right)** Product glucosyl hydrazide (**27**).

Dapagliflozin (1)

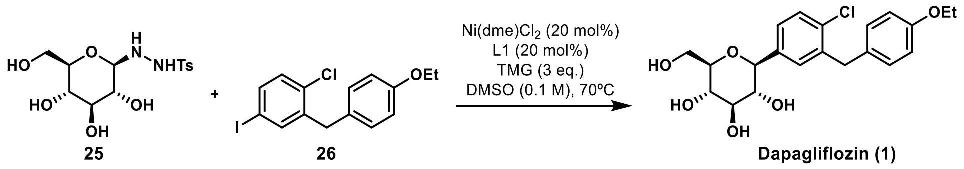

To a reaction flask (1 L) with a stir bar were added sugar hydrazide **25** (10 g, 28.7 mmol, 1.0 equiv.), 1-chloro-2-[(4-ethoxyphenyl)methyl]-4-iodobenzene **26** (16 g, 43 mmol, 1.5 equiv.), Ni(dme)Cl_2 (847 mg, 5.74 mmol, 0.2 equiv.), and pyridine-2-carboxamidine hydrochloride (905 g, 5.74 mmol, 0.2 equiv.). The flask was capped with a septum, then evacuated and backfilled with argon three times. Pre-degassed DMSO (287 mL) was added through canula transfer and the mixture was stirred for 10 minutes, followed by the addition of TMG (10.8 mL, 86.1 mmol, 3.0 equiv.). The reaction mixture was heated to 70 °C and stirred overnight. After the reaction was complete, water (500 mL) was added. The aqueous phase was extracted with EtOAc (3 x 500 mL). The combined organic phase was washed with brine (100 mL), dried over Na_2SO_4, and concentrated under reduced pressure. The crude residue was purified by flash column chromatography (silica gel, DCM/EtOAc 10:1 to 1:1 then DCM/MeOH 100:1 to 9:1) to afford Dapagliflozin (**1**) (7.2 g, 17.6 mmol, 62% yield) as light-yellow foam.

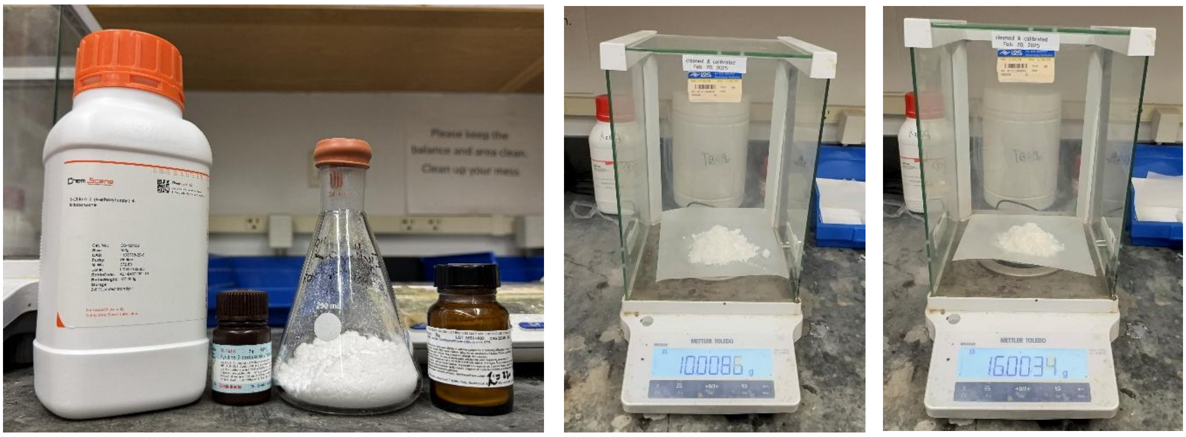

(Left) Reagents for the reaction. **(Center)** Glucosyl hydrazide **25**. **(Right)** 1-chloro-2-[(4-ethoxyphenyl)methyl]-4-iodobenzene **26**.

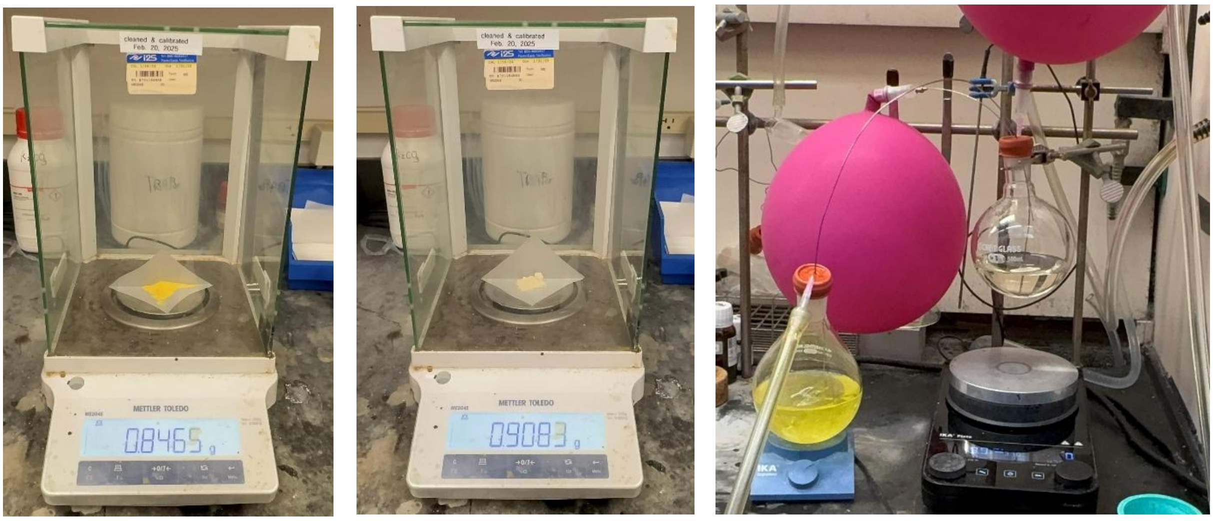

(Left) $NiCl_2(DME)$. **(Center)** pyridine-2-carboxamidine hydrochloride **L1**. **(Right)** cannula transfer of pre-degassed DMSO to the reaction flask.

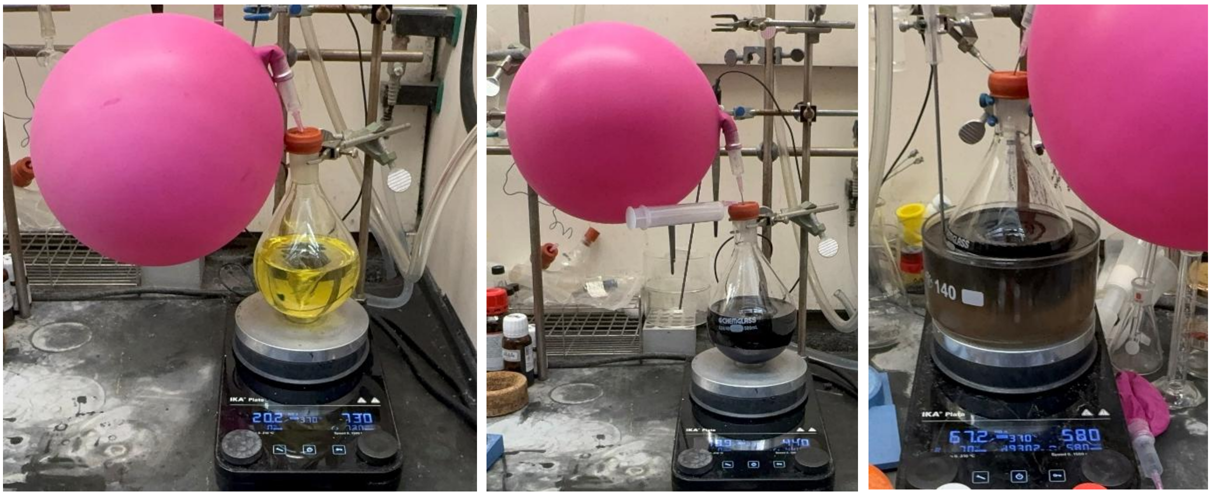

(Left) reaction mixture after DMSO addition. **(Center)** reaction mixture after TMG addition. **(Right)** reaction mixture was stirred at 70 °C overnight.

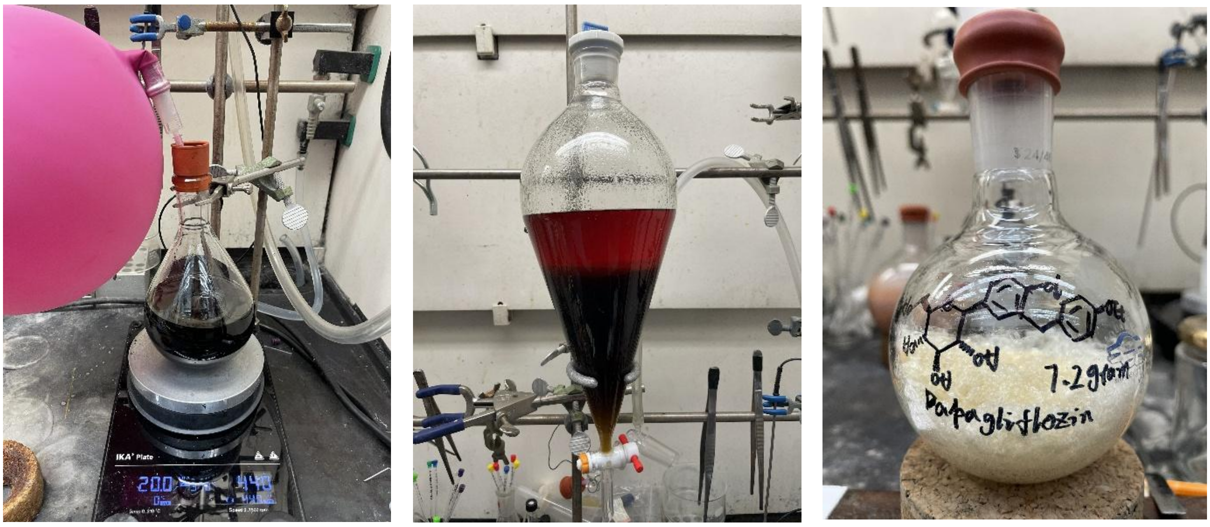

(Left) reaction mixture after being stirred overnight. **(Center)** reaction workup. **(Right)** Dapagliflozin **1**.

9. RCC-Arylation of Different Sugars

Table S18 Summary of Different Sugar Hydrazides

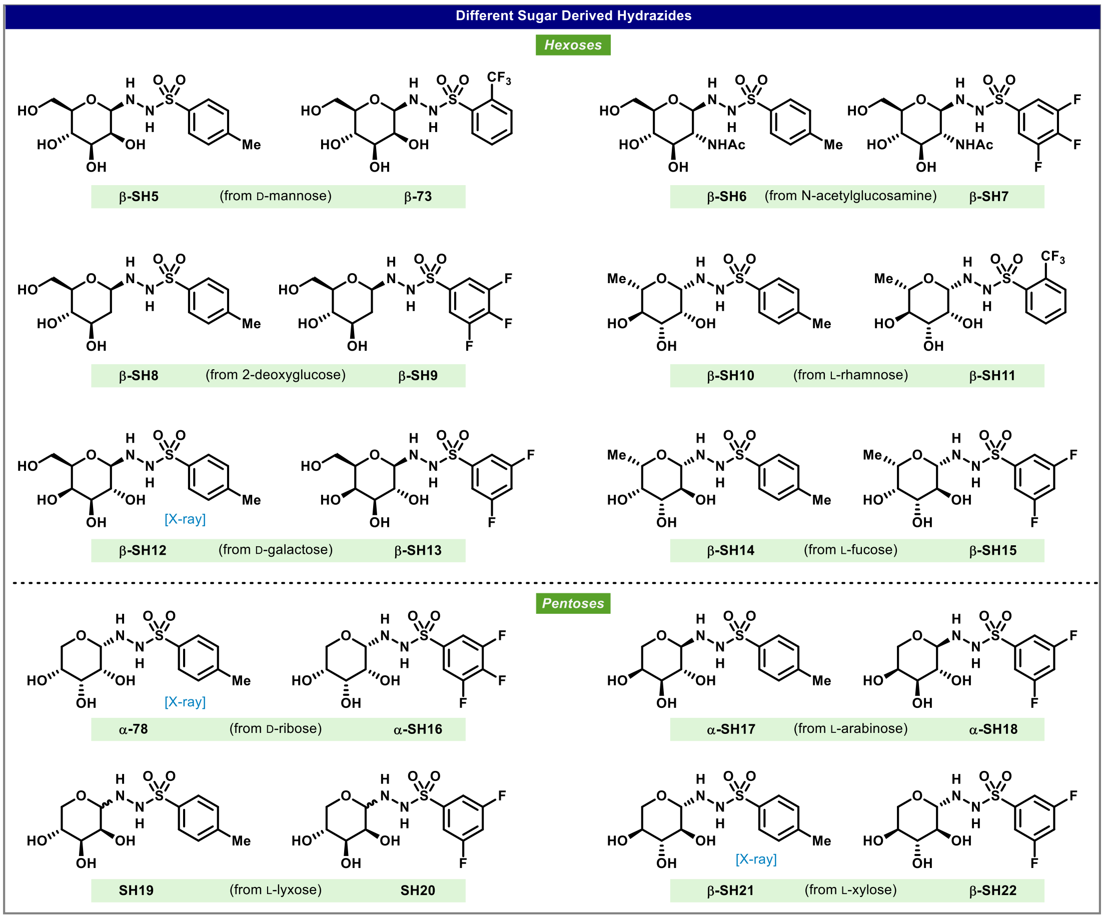

Manuscript Figure 3.

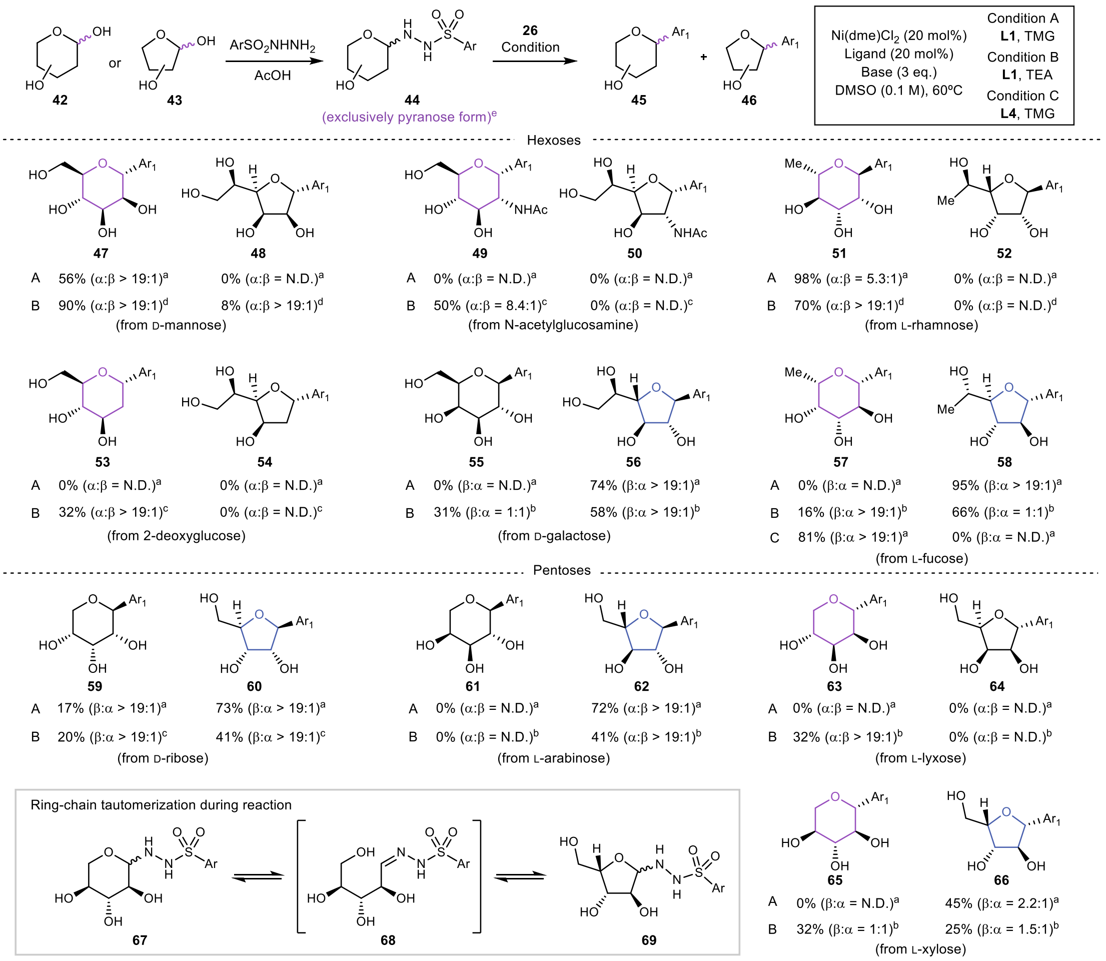

Figure 3. [a]Toluenesulfonyl hydrazide. [b]3,5-difluorobenzenesulfonyl hydrazide. [c]3,4,5-trifluorobenzenesulfonyl hydrazide. [d]2-(trifluoromethyl)benzenesulfonyl hydrazide. [e]Structures are determined using 2D NMR (see SI for details). Ribose, xylose, and galactose hydrazides' structures are further confirmed by X-ray crystallography. Diastereomeric ratio was determined from the crude reaction mixture prior to purification. Ar_1 = 1-chloro-2-[(4-ethoxyphenyl)methyl]-benzene.

In the case of xylose derived hydrazide, it led to a mixture of four diastereomers (Condition B). If tautomerization needs to be suppressed, other than tuning condition, an alternative is to employ protected branched sugar hydrazides as shown below.

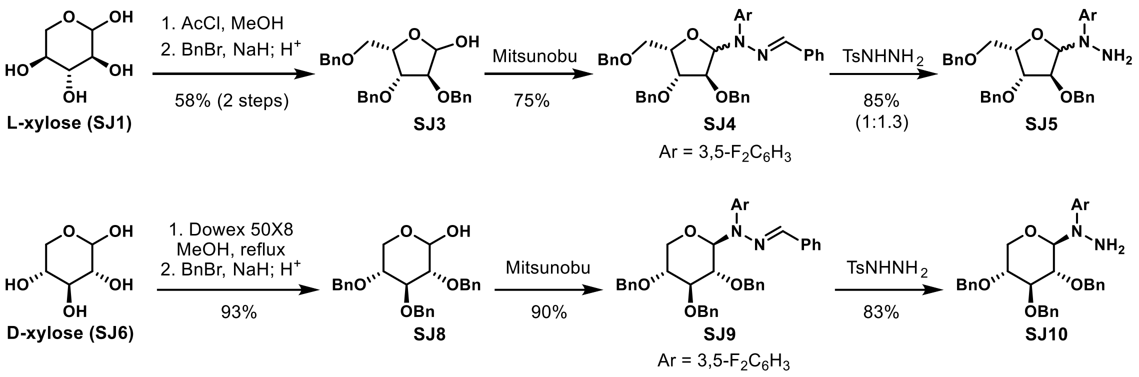

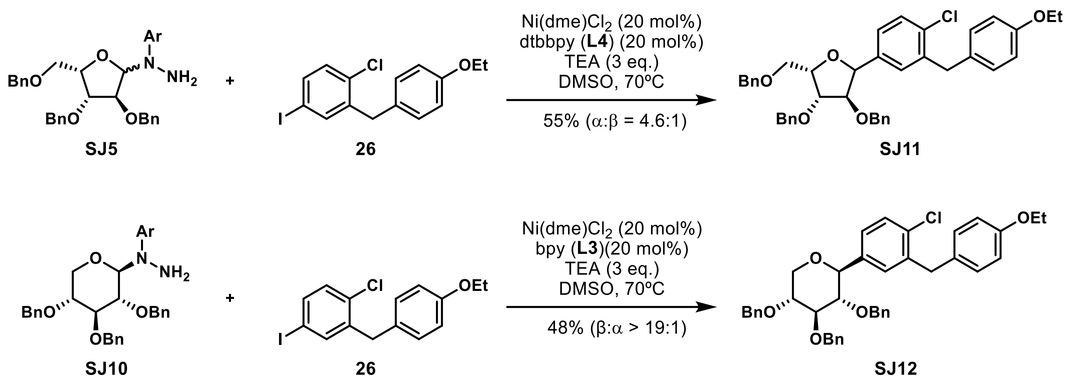

L-xylose (**SJ1**) and D-xylose (**SJ6**) were used to synthesize branched hydrazide **SJ5** and **SJ10** respectively. General procedure **E** could be followed with different ligands to get **SJ11** (55%, α:β = 4.6:1) and **SJ12** (48%, β:α >19:1) respectively.

Compound β-SH5

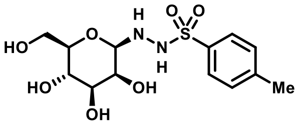

Following **General Procedure A1** with D-mannose (5 mmol) and *p*-toluenesulfonyl hydrazide purification through precipitation and filtration afforded mannose-hydrazide **β-SH5** (1.6 g, 91% yield) as white solid.

$[\alpha]_D^{25}$= –92.6° (c = 0.5, DMSO)

^{1}H NMR (600 MHz, $(CD_3)_2SO$) δ 8.91 (d, *J* = 3.0 Hz, 1H), 7.73 – 7.69 (m, 2H), 7.38 (d, *J* = 8.1 Hz, 2H), 5.30 (dd, *J* = 5.6, 2.8 Hz, 1H), 4.98 (d, *J* = 4.7 Hz, 1H), 4.93 (d, *J* = 5.1 Hz, 1H), 4.09 (d, *J* = 3.7 Hz, 1H), 3.64 – 3.55 (m, 2H), 3.22 (ddt, *J* = 10.4, 8.6, 5.2 Hz, 1H), 3.14 (td, *J* = 8.7, 3.7 Hz, 1H), 3.08 (td, *J* = 8.7, 4.7 Hz, 1H), 2.92 (t, *J* = 10.8 Hz, 1H), 2.38 (s, 3H).

^{13}C NMR (151 MHz, $(CD_3)_2SO$) δ 142.9, 136.3, 129.4, 127.5, 91.2, 76.6, 70.1, 69.7, 66.8, 21.1.

HRMS (m/z): (ESI) calc. for $C_{13}H_{20}N_2O_7S$ $[M+H]^+$: 349.1069 found 349.1057

Compound β-SH6

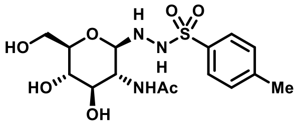

Following **General Procedure A1** with D-N-acetylglucosamine (5 mmol) and *p*-toluenesulfonyl hydrazide purification through precipitation and filtration afforded N-acetylglucosamine-hydrazide **β-SH6** (1.6 g, 81% yield) as white solid.

$[\alpha]_D^{25}$= –49.6° (c = 0.25, DMSO)

^{1}H NMR (600 MHz, $(CD_3)_2SO$)) δ 8.70 (d, *J* = 1.8 Hz, 1H), 7.66 (d, *J* = 8.3 Hz, 1H), 7.64 (d, *J* = 8.2 Hz, 2H), 7.36 (d, *J* = 8.1 Hz, 2H), 4.96 (d, *J* = 5.3 Hz, 1H), 4.88 (d, *J* = 4.9 Hz, 1H), 4.76 (dd, *J* = 9.7, 1.8 Hz, 1H), 4.44 (dd, *J* = 8.2, 3.9 Hz, 1H), 3.73 (t, *J* = 9.5 Hz, 1H), 3.65 (ddd, *J* = 11.7, 8.2, 2.2 Hz, 1H), 3.38 – 3.32 (m, 2H), 3.27 – 3.20 (m, 2H), 2.96 (ddd, *J* = 9.4, 7.0, 2.2 Hz, 1H), 2.91 (ddd, *J* = 9.8, 8.1, 5.3 Hz, 1H), 2.39 (s, 3H), 1.83 (s, 3H).

^{13}C NMR (151 MHz, $(CD_3)_2SO$)) δ 169.5, 142.9, 136.2, 129.3, 127.6, 90.3, 77.8, 74.3, 71.2, 61.9, 53.4, 23.0, 21.1.

HRMS (m/z): (ESI) calc. for $C_{15}H_{24}N_3O_7S$ $[M+H]^+$: 390.1335 found 390.1339

Compound β-SH7

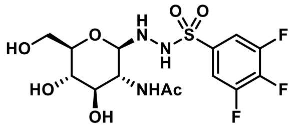

Following **General Procedure A1** with N-acetylglucosamine (5 mmol) and 3,4,5-trifluorobenzenesulfonyl hydrazide, purification through precipitation and filtration afforded N-acetylglucosamine-hydrazide **β-SH7** (1.9 g, 90% yield) as white solid.

$[\alpha]_D^{25}$= –57.0° (c = 0.1, DMSO)

^{1}H NMR (600 MHz, $(CD_3)_2SO$) δ 9.08 (s, 1H), 7.65 – 7.58 (m, 3H), 5.25 (d, *J* = 10.1 Hz, 1H), 4.95 (d, *J* = 5.4 Hz, 1H), 4.91 (d, *J* = 5.0 Hz, 1H), 4.40 (dd, *J* = 8.1, 3.8 Hz, 1H), 3.68 – 3.61 (m, 2H), 3.35 (s, 1H), 3.27 – 3.16 (m, 2H), 2.96 (ddd, *J* = 9.6, 7.2, 2.2 Hz, 1H), 2.90 (ddd, *J* = 9.7, 8.1, 5.4 Hz, 1H), 1.80 (s, 3H).

^{13}C NMR (151 MHz, $(CD_3)_2SO$) δ 169.3, 150.9 (d, *J* = 10.1 Hz), 149.2 (d, *J* = 10.0 Hz), 142.7 (t, *J* = 15.4 Hz), 141.0 (t, *J* = 15.3 Hz), 135.4 (d, *J* = 5.3 Hz), 113.1 (dd, *J* = 18.4, 5.2 Hz), 90.0, 77.9, 74.2, 71.1, 61.9, 53.5, 22.9.

HRMS (m/z): (ESI) calc. for $C_{14}H_{19}F_3N_3O_7S$ $[M+H]^+$: 430.0896 found 430.0892

Compound β-SH8

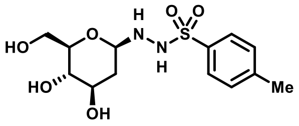

Following **General Procedure A1** with D-2-deoxyglucose (5 mmol) and *p*-toluenesulfonyl hydrazide purification through precipitation and filtration afforded 2-deoxyglucose-hydrazide **β-SH8** (1.5 g, 91% yield) as white solid.

$[\alpha]_D^{25}$= –54.2° (c = 0.5, DMSO)

^{1}H NMR (600 MHz, $(CD_3)_2SO$)) δ 8.76 (s, 1H), 7.69 (d, *J* = 8.1 Hz, 2H), 7.39 (d, *J* = 8.0 Hz, 2H), 5.47 (d, *J* = 10.2 Hz, 1H), 4.81 (dd, *J* = 5.0, 3.6 Hz, 2H), 4.45 (dd, *J* = 9.2, 3.3 Hz, 1H), 3.64 (ddd, *J* = 11.5, 9.1, 2.3 Hz, 1H), 3.53 (td, *J* = 10.6, 1.9 Hz, 1H), 3.35 – 3.31 (m, 1H), 3.14 (ddt, *J* = 11.6, 10.0, 5.0 Hz, 1H), 2.83 (ddd, *J* = 9.8, 7.6, 2.3 Hz, 1H), 2.74 (td, *J* = 9.0, 5.2 Hz, 1H), 2.39 (s, 3H), 1.60 (ddd, *J* = 12.7, 5.2, 1.8 Hz, 1H), 1.18 (q, *J* = 11.7 Hz, 1H).

^{13}C NMR (151 MHz, $(CD_3)_2SO$)) δ 142.9, 136.9, 129.4, 127.4, 86.1, 78.2, 72.1, 71.3, 62.2, 36.8, 21.1.

HRMS (m/z): (ESI) calc. for $C_{13}H_{21}N_2O_6S$ $[M+H]^+$: 333.1120 found 333.1110

Compound β-SH9

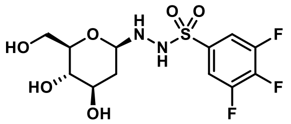

Following **General Procedure A1** with 2-deoxyglucose (5 mmol) and 3,4,5-trifluorobenzenesulfonyl hydrazide, purification through precipitation and filtration afforded 2-deoxyglucose-hydrazide **β-SH9** (1.7 g, 93% yield) as white solid.

$[\alpha]_D^{25}$= –10.4° (c = 0.25, DMSO)

^{1}H NMR (600 MHz, $(CD_3)_2SO$) δ 9.18 (d, *J* = 1.4 Hz, 1H), 7.70 (t, *J* = 6.5 Hz, 2H), 5.64 (dd, *J* = 9.4, 1.3 Hz, 1H), 4.82 (d, *J* = 5.2 Hz, 2H), 4.36 (dd, *J* = 8.0, 4.0 Hz, 1H), 3.79 (td, *J* = 10.2, 1.9 Hz, 1H), 3.66 (ddd, *J* = 11.4, 8.0, 2.4 Hz, 1H), 3.37 (ddd, *J* = 11.5, 7.2, 4.2 Hz, 1H), 3.30 (ddt, *J* = 11.5, 8.5, 5.0 Hz, 1H), 2.96 (ddd, *J* = 9.5, 7.1, 2.3 Hz, 1H), 2.80 (td, *J* = 9.0, 5.1 Hz, 1H), 1.69 (ddd, *J* = 12.6, 5.1, 2.0 Hz, 1H), 1.21 (q, *J* = 11.4 Hz, 1H).

^{13}C NMR (151 MHz, $(CD_3)_2SO$) δ 150.9 (d, *J* = 9.9 Hz), 149.3 (d, *J* = 10.1 Hz), 142.5 (t, *J* = 15.2 Hz), 140.9 (t, *J* = 15.2 Hz), 136.1 (d, *J* = 5.2 Hz), 112.9 (d, *J* = 23.2 Hz), 86.2, 78.0, 72.0, 71.1, 62.0, 36.8.

HRMS (m/z): (ESI) calc. for $C_{12}H_{16}F_2N_2O_7S$ $[M+H]^+$: 372.0603 found 372.0612

Compound β-SH10

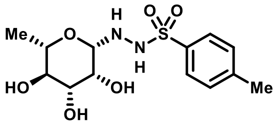

Following **General Procedure A1** with L-rhamnose (5 mmol) and *p*-toluenesulfonyl hydrazide purification through precipitation and filtration afforded rhamnose-hydrazide **β-SH10** (1.4 g, 83% yield) as white solid.

$[\alpha]_D^{25}$= +122.4° (c = 0.5, DMSO)

^{1}H NMR (600 MHz, $(CD_3)_2SO$)) δ 8.76 (s, 1H), 7.69 (d, *J* = 8.2 Hz, 2H), 7.38 (d, *J* = 8.0 Hz, 2H), 4.71 (dd, *J* = 5.0, 2.6 Hz, 2H), 4.68 (d, *J* = 5.4 Hz, 1H), 4.28 (dd, *J* = 11.3, 3.4 Hz, 1H), 3.85 (d, *J* = 11.2 Hz, 1H), 3.49 (dd, *J* = 4.9, 3.6 Hz, 1H), 3.13 (ddd, *J* = 8.7, 5.4, 3.2 Hz, 1H), 3.08 – 2.98 (m, 2H), 2.38 (s, 3H), 1.15 (d, *J* = 5.7 Hz, 3H).

^{13}C NMR (151 MHz, $(CD_3)_2SO$)) δ 142.9, 136.1, 129.4, 127.6, 88.3, 73.9, 73.0, 72.0, 70.0, 21.0, 18.0.

HRMS (m/z): (ESI) calc. for $C_{13}H_{21}N_2O_6S$ $[M+H]^+$: 333.1120 found 333.1110

Compound β-SH11

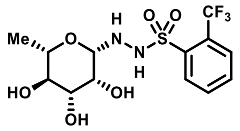

Following **General Procedure A1** with L-rhamnose (5 mmol) and 2-(trifluoromethyl)benzenesulfonyl hydrazide, purification through precipitation and filtration afforded rhamnose-hydrazide **β-SH11** (1.8 g, 92% yield) as white solid.

$[\alpha]_D^{25}$= +9.5° (c = 1.0, DMSO)

^{1}H NMR (600 MHz, $(CD_3)_2SO$) δ 9.24 (d, *J* = 2.9 Hz, 1H), 8.12 (d, *J* = 7.8 Hz, 1H), 7.97 (d, *J* = 7.7 Hz, 1H), 7.89 – 7.81 (m, 2H), 4.82 (dd, *J* = 11.0, 3.1 Hz, 1H), 4.76 – 4.67 (m, 3H), 3.91 (d, *J* = 10.9 Hz, 1H), 3.52 (s, 1H), 3.15 (dd, *J* = 9.2, 3.2 Hz, 1H), 3.06 (t, *J* = 9.4 Hz, 1H), 3.02 – 2.97 (m, 1H), 1.10 (d, *J* = 6.0 Hz, 3H).

^{13}C NMR (151 MHz, $(CD_3)_2SO$) δ 138.8, 133.5, 133.3, 131.2, 128.7 (q, $^3J_{C,F}$ = 6.2 Hz), 127.2 (q, $^2J_{C,F}$ = 32.5 Hz), 123.3 (q, $^1J_{C,F}$ = 274.0 Hz), 89.0, 74.3, 73.5, 72.5, 70.4, 18.4.

HRMS (m/z): (ESI) calc. for $C_{13}H_{18}F_3N_2O_6S$ $[M+H]^+$: 387.0838 found 387.0833

Compound β-SH12

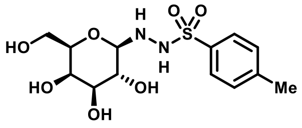

Following **General Procedure A1** with D-Galactose (5 mmol) and *p*-toluenesulfonyl hydrazide purification through precipitation and filtration afforded galactose-hydrazide **β-SH12** (1.6 g, 92% yield) as white solid.

$[\alpha]_D^{25}$= –1.8° (c = 0.5, DMSO)

^{1}H NMR (600 MHz, $(CD_3)_2SO$) δ 8.87 (s, 1H), 7.72 (d, *J* = 8.3 Hz, 2H), 7.36 (d, *J* = 8.1 Hz, 2H), 5.27 (d, *J* = 6.8 Hz, 1H), 4.73 (d, *J* = 5.6 Hz, 1H), 4.46 (dd, *J* = 6.5, 5.1 Hz, 1H), 4.29 (d, *J* = 4.3 Hz, 1H), 4.07 (d, *J* = 3.7 Hz, 1H), 3.59 (t, *J* = 4.0 Hz, 1H), 3.52 – 3.43 (m, 3H), 3.39 (td, *J* = 9.0, 3.7 Hz, 1H), 3.23 – 3.19 (m, 2H), 2.38 (s, 3H).

^{13}C NMR (151 MHz, $(CD_3)_2SO$) δ 142.8, 136.6, 129.4, 127.5, 90.7, 76.5, 73.6, 68.3, 67.8, 60.7, 21.1.

HRMS (m/z): (ESI) calc. for $C_{13}H_{21}N_2O_7S$ $[M+H]^+$: 349.1069 found 349.1065

Compound β-SH13

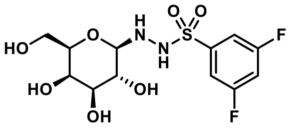

Following **General Procedure A1** with D-galactose (5 mmol) and 3,5-difluorobenzenesulfonyl hydrazide, purification through precipitation and filtration afforded galactose-hydrazide **β-SH13** (1.6 g, 90% yield) as white solid.

$[\alpha]_D^{25}$= –14.0° (c = 0.25, DMSO)

^{1}H NMR (600 MHz, $(CD_3)_2SO$) δ 9.19 (d, *J* = 2.0 Hz, 1H), 7.61 (tt, *J* = 9.2, 2.3 Hz, 1H), 7.52 (dd, *J* = 6.7, 2.3 Hz, 2H), 5.58 (dd, *J* = 7.6, 2.1 Hz, 1H), 4.75 (d, *J* = 5.5 Hz, 1H), 4.43 (t, *J* = 5.8 Hz, 1H), 4.30 (d, *J* = 4.2 Hz, 1H), 4.17 (d, *J* = 3.9 Hz, 1H), 3.59 (t, *J* = 3.9 Hz, 1H), 3.49 (t, *J* = 8.2 Hz, 1H), 3.45 (t, *J* = 6.0 Hz, 2H), 3.37 – 3.32 (m, 1H), 3.22 (td, *J* = 6.0, 2.1 Hz, 2H).

^{13}C NMR (151 MHz, $(CD_3)_2SO$) δ 162.9 (d, *J* = 12.2 Hz), 161.2 (d, *J* = 12.4 Hz), 142.7 (t, *J* = 8.5 Hz), 111.4 – 111.2 (m), 108.4 (t, *J* = 25.8 Hz), 91.0, 76.4, 73.7, 68.2, 68.0, 60.7.

HRMS (m/z): (ESI) calc. for $C_{12}H_{17}F_2N_2O_7S$ $[M+H]^+$: 371.0725 found 371.0730

Compound β-SH14

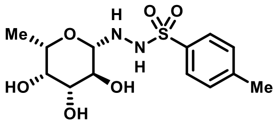

Following **General Procedure A1** with L-fucose (5 mmol) and *p*-toluenesulfonyl hydrazide purification through precipitation and filtration afforded fucose-hydrazide **β-SH14** (1.5 g, 89% yield) as white solid.

$[\alpha]_D^{25}$= –12.4° (c = 0.5, DMSO)

^{1}H NMR (600 MHz, $(CD_3)_2SO$)) δ 8.91 (d, *J* = 2.7 Hz, 1H), 7.71 (d, *J* = 8.2 Hz, 2H), 7.37 (d, *J* = 8.0 Hz, 2H), 5.18 (dd, *J* = 5.3, 2.7 Hz, 1H), 4.70 (d, *J* = 5.6 Hz, 1H), 4.35 (d, *J* = 4.3 Hz, 1H), 3.90 (d, *J* = 3.4 Hz, 1H), 3.60 (dd, *J* = 8.7, 5.1 Hz, 1H), 3.48 – 3.40 (m, 2H), 3.39 (t, *J* = 3.9 Hz, 1H), 3.30 (dt, *J* = 8.8, 3.9 Hz, 1H), 2.38 (s, 3H), 1.08 (d, *J* = 6.4 Hz, 3H).

^{13}C NMR (151 MHz, $(CD_3)_2SO$)) δ 142.8, 136.3, 129.4, 127.5, 90.7, 73.6, 71.2, 67.2, 21.0, 16.8.

HRMS (m/z): (ESI) calc. for $C_{13}H_{20}N_2NaO_6S$ $[M+Na]^+$:355.0940 found 355.0930

Compound β-SH15

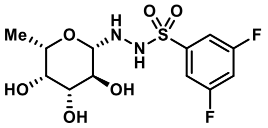

Following **General Procedure A1** with L-fucose (5 mmol) and 3,5-difluorobenzenesulfonyl hydrazide, purification through precipitation and filtration afforded fucose-hydrazide **β-SH15** (1.6 g, 91% yield) as white solid.

$[\alpha]_D^{25}$= –4.0° (c = 0.25, DMSO)

^{1}H NMR (600 MHz, $(CD_3)_2SO$) δ 9.22 (d, *J* = 2.8 Hz, 1H), 7.61 (tt, *J* = 9.2, 2.4 Hz, 1H), 7.54 – 7.51 (m, 2H), 5.48 (dd, *J* = 6.2, 2.8 Hz, 1H), 4.71 (d, *J* = 5.7 Hz, 1H), 4.35 (d, *J* = 4.3 Hz, 1H), 3.98 (d, *J* = 3.6 Hz, 1H), 3.59 (dd, *J* = 8.7, 6.1 Hz, 1H), 3.42 (q, *J* = 6.4 Hz, 1H), 3.40 – 3.35 (m, 2H), 3.28 (ddd, *J* = 9.3, 5.8, 3.4 Hz, 1H), 1.07 (d, *J* = 6.4 Hz, 3H).

^{13}C NMR (151 MHz, $(CD_3)_2SO$) δ 162.8 (d, *J* = 12.1 Hz), 161.2 (d, *J* = 12.3 Hz), 142.6 (t, *J* = 8.6 Hz), 111.8 – 111.0 (m), 108.3 (t, *J* = 25.8 Hz), 91.0, 73.7, 71.2, 71.1, 67.4, 16.8.

HRMS (m/z): (ESI) calc. for $C_{12}H_{15}F_2N_2O_6S$ $[M–H]^-$: 353.0619 found 353.0623

Compound α-SH16

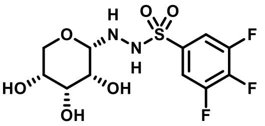

Following **General Procedure A1** with ribose (5 mmol) and 3,4,5-trifluorobenzenesulfonyl hydrazide, purification through precipitation and filtration afforded ribose-hydrazide **α-SH16** (1.6 g, 91% yield) as white solid.

$[\alpha]_D^{25}$= –34.4° (c = 0.5, DMSO)

^{1}H NMR (600 MHz, $(CD_3)_2SO$) δ 9.18 (d, *J* = 2.5 Hz, 1H), 7.76 (t, *J* = 6.6 Hz, 2H), 5.48 (dd, *J* = 6.8, 2.1 Hz, 1H), 4.71 (d, *J* = 3.7 Hz, 1H), 4.60 (d, *J* = 6.8 Hz, 1H), 4.09 (d, *J* = 5.1 Hz, 1H), 3.93 (dd, *J* = 8.4, 6.7 Hz, 1H), 3.83 (d, *J* = 3.2 Hz, 1H), 3.45 – 3.37 (m, 2H), 3.36 – 3.31 (m, 2H), 3.23 (ddd, *J* = 8.2, 5.1, 2.8 Hz, 1H).

^{13}C NMR (151 MHz, $(CD_3)_2SO$) δ 150.8 (d, *J* = 9.9 Hz), 149.1 (d, *J* = 9.9 Hz), 142.5 (t, *J* = 15.4 Hz), 140.8 (t, *J* = 15.2 Hz), 135.6 (d, *J* = 6.0 Hz), 113.3 (dd, *J* = 18.5, 5.1 Hz), 87.3, 70.1, 67.9, 67.2, 63.9.

HRMS (m/z): (ESI) calc. $C_{11}H_{14}F_3N_2O_6S$ $[M+H]^+$: 359.0525 found 359.0515.

Compound α-SH17

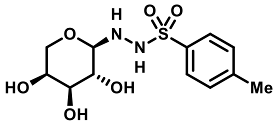

Following **General Procedure A1** with L-Arabinose (5 mmol) and *p*-toluenesulfonyl hydrazide purification through precipitation and filtration afforded arabinose-hydrazide **α-SH17** (1.4 g, 86% yield) as white solid.

$[\alpha]_D^{25}$= –10.4° (c = 0.5, DMSO)

^{1}H NMR (600 MHz, $(CD_3)_2SO$) δ 8.87 (d, *J* = 3.1 Hz, 1H), 7.70 (d, *J* = 8.3 Hz, 2H), 7.38 (d, *J* = 8.0 Hz, 2H), 5.21 (dd, *J* = 7.2, 3.0 Hz, 1H), 4.87 (d, *J* = 5.0 Hz, 1H), 4.50 (d, *J* = 4.3 Hz, 1H), 4.30 (d, *J* = 3.8 Hz, 1H), 3.71 (t, *J* = 6.7 Hz, 1H), 3.62 (tt, *J* = 4.6, 2.7 Hz, 1H), 3.58 (dd, *J* = 11.8, 4.7 Hz, 1H), 3.49 (ddd, *J* = 7.7, 6.4, 3.8 Hz, 1H), 3.39 (ddd, *J* = 8.0, 4.9, 3.2 Hz, 1H), 3.28 (dd, *J* = 11.8, 2.4 Hz, 1H), 2.38 (s, 3H).

^{13}C NMR (151 MHz, $(CD_3)_2SO$) δ 143.3, 136.8, 129.9, 127.9, 90.9, 72.5, 68.4, 67.2, 64.9, 21.5.

HRMS (m/z): (ESI) calc. for $C_{12}H_{18}N_2NaO_6S$ $[M+H]^+$: 341.0783 found 341.0777

Compound α-SH18

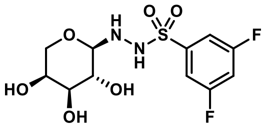

Following **General Procedure A1** with L-arabinose (5 mmol) and 3,5-difluorobenzenesulfonyl hydrazide, purification through precipitation and filtration afforded arabinose-hydrazide **α-SH18** (1.4 g, 81% yield) as white solid.

$[\alpha]_D^{25}$= –11.0° (c = 0.5, DMSO)

^{1}H NMR (600 MHz, $(CD_3)_2SO$) δ 9.22 (d, *J* = 2.6 Hz, 1H), 7.62 (tt, *J* = 9.2, 2.4 Hz, 1H), 7.51 (dd, *J* = 4.9, 1.8 Hz, 2H), 5.49 (dd, *J* = 7.5, 2.8 Hz, 1H), 4.85 (d, *J* = 4.9 Hz, 1H), 4.50 (d, *J* = 3.9 Hz, 1H), 4.29 (d, *J* = 3.8 Hz, 1H), 3.66 (t, *J* = 7.2 Hz, 1H), 3.61 (s, 1H), 3.59 (dd, *J* = 11.7, 4.0 Hz, 1H), 3.43 (td, *J* = 7.6, 3.1 Hz, 1H), 3.37 – 3.33 (m, 1H), 3.30 (dd, *J* = 11.7, 1.9 Hz, 1H).

^{13}C NMR (151 MHz, $(CD_3)_2SO$) δ 162.9 (d, *J* = 12.1 Hz), 161.2 (d, *J* = 12.3 Hz), 142.6 (t, *J* = 8.5 Hz), 111.4 – 111.0 (m), 108.4 (t, *J* = 25.6 Hz), 90.9, 72.4, 68.1, 67.2, 65.3.

HRMS (m/z): (ESI) calc. for $C_{11}H_{14}F_2N_2NaO_6S$ $[M+Na]^+$: 363.0438 found 363.0431

Compound SH19

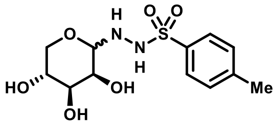

Following **General Procedure A1** with D-Lyxose (5 mmol) and *p*-toluenesulfonyl hydrazide purification through precipitation and filtration afforded lyxose-hydrazide **SH19** (1.4 g, 89% yield, α:β = 1:1) as white solid.

$[\alpha]_D^{25}$= +53.8° (c = 0.5, DMSO)

^{1}H NMR (600 MHz, $(CD_3)_2SO$) δ 8.87 (d, *J* = 2.9 Hz, 1H), 8.78 (d, *J* = 3.4 Hz, 1H), 7.69 (dd, *J* = 8.2, 6.2 Hz, 4H), 7.38 (dd, *J* = 8.3, 3.0 Hz, 4H), 5.21 (dd, *J* = 5.5, 2.8 Hz, 1H), 4.82 – 4.80 (m, 2H), 4.75 (t, *J* = 4.9 Hz, 2H), 4.70 (d, *J* = 5.1 Hz, 1H), 4.39 (dd, *J* = 11.2, 3.4 Hz, 1H), 4.04 – 3.99 (m, 2H), 3.85 (dd, *J* = 11.1, 1.4 Hz, 1H), 3.69 (d, *J* = 3.9 Hz, 1H), 3.64 (dd, *J* = 11.5, 4.8 Hz, 2H), 3.56 – 3.40 (m, 5H), 3.15 (ddd, *J* = 8.8, 5.3, 3.2 Hz, 1H), 2.88 (dd, *J* = 11.3, 9.6 Hz, 1H), 2.38 (s, 6H).

^{13}C NMR (151 MHz, $(CD_3)_2SO$) δ 142.9, 142.8, 136.4, 136.1, 129.5, 129.4, 127.5, 127.5, 89.1, 87.6, 74.0, 70.6, 69.3, 69.1, 66.6, 66.3, 65.8, 65.2, 21.0.

HRMS (m/z): (ESI) calc. for $C_{12}H_{19}N_2O_6S$ $[M+H]^+$: 317.0807 found 317.0799

Compound SH20

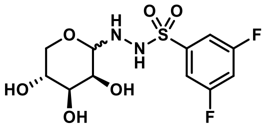

Following **General Procedure A1** with D-lyxose (5 mmol) and 3,5-difluorobenzenesulfonyl hydrazide, purification through precipitation and filtration afforded lyxose-hydrazide **SH20** (1.4 g, 82% yield, α:β = 1:1) as white solid.

$[\alpha]_D^{25}$= +24° (c = 0.2, DMSO)

^{1}H NMR (600 MHz, $(CD_3)_2SO$) δ 9.22 (d, *J* = 2.8 Hz, 1H), 9.17 (d, *J* = 3.2 Hz, 1H), 7.63 (dt, *J* = 9.5, 4.7 Hz, 2H), 7.51 – 7.48 (m, 4H), 5.51 (dd, *J* = 6.4, 2.6 Hz, 1H), 4.82 (d, *J* = 4.4 Hz, 2H), 4.80 – 4.72 (m, 4H), 4.06 (d, *J* = 4.7 Hz, 1H), 4.01 (t, *J* = 6.9 Hz, 1H), 3.90 (d, *J* = 10.9 Hz, 1H), 3.67 – 3.64 (m, 2H), 3.59 – 3.54 (m, 2H), 3.54 – 3.48 (m, 2H), 3.48 – 3.39 (m, 2H), 3.19 (p, *J* = 5.1 Hz, 1H), 2.90 (t, *J* = 10.4 Hz, 1H).

^{13}C NMR (151 MHz, $(CD_3)_2SO$) δ 162.9 (dd, *J* = 12.2, 4.8 Hz), 161.2 (dd, *J* = 12.2, 4.4 Hz), 142.7 (t, *J* = 8.5 Hz), 142.6 (t, *J* = 8.4 Hz), 111.6 – 111.0 (m), 108.4 (q, *J* = 26.2 Hz), 89.3, 87.9, 73.9, 70.6, 69.2, 69.0, 66.6, 66.2, 66.0, 65.0.

HRMS (m/z): (ESI) calc. for $C_{11}H_{13}F_2N_2O_6S$ $[M–H]^-$: 339.0462 found 339.0461

Compound β-SH21

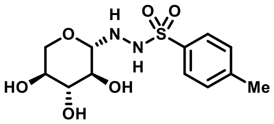

Following **General Procedure A1** with L-xylose (5 mmol) and *p*-toluenesulfonyl hydrazide purification through precipitation and filtration afforded xylose-hydrazide **β-SH21** (1.3 g, 81% yield) as white solid.

$[\alpha]_D^{25}$= +24.8° (c = 0.5, DMSO)

^{1}H NMR (600 MHz, MeOD) δ 7.78 (d, *J* = 8.0 Hz, 2H), 7.38 (d, *J* = 8.0 Hz, 2H), 3.78 (dd, *J* = 11.3, 5.4 Hz, 1H), 3.69 (d, *J* = 8.7 Hz, 1H), 3.44 (qd, *J* = 8.8, 4.8 Hz, 2H), 3.30 (t, *J* = 10.3 Hz, 1H), 2.43 (s, 3H).

^{13}C NMR (151 MHz, MeOD) δ 145.1, 137.1, 130.6, 129.1, 92.3, 78.1, 71.3, 71.2, 68.3, 21.5.

HRMS (m/z): (ESI) calc. for $C_{12}H_{17}N_2O_6S$ [M–H]$^-$: 317.0807 found 317.0810

Compound β-SH22

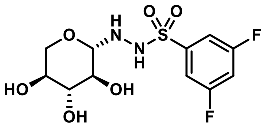

Following **General Procedure A1** with L-xylose (5 mmol) and 3,5-difluorobenzenesulfonyl hydrazide, purification through precipitation and filtration afforded xylose-hydrazide **β-SH22** (1.4 g, 85% yield) as white solid.

$[\alpha]_D^{25}$= +38.6° (c = 0.5, DMSO)

^{1}H NMR (600 MHz, $(CD_3)_2SO$) δ 9.23 (d, *J* = 2.4 Hz, 1H), 7.62 (tt, *J* = 9.1, 2.4 Hz, 1H), 7.53 – 7.51 (m, 2H), 5.58 (dd, *J* = 6.7, 2.5 Hz, 1H), 4.98 (d, *J* = 3.9 Hz, 1H), 4.92 (d, *J* = 5.0 Hz, 1H), 4.23 (d, *J* = 3.5 Hz, 1H), 3.59 (ddd, *J* = 11.5, 9.6, 5.8 Hz, 2H), 3.20 (ddd, *J* = 13.7, 7.3, 4.4 Hz, 1H), 3.09 – 3.02 (m, 2H), 2.92 (t, *J* = 10.8 Hz, 1H).

^{13}C NMR (151 MHz, $(CD_3)_2SO$) δ 163.3 (d, *J* = 12.2 Hz), 161.6 (d, *J* = 12.2 Hz), 143.0 (t, *J* = 8.5 Hz), 112.1 – 111.5 (m), 108.8 (t, *J* = 25.8 Hz), 91.9, 77.3, 70.9, 70.1, 67.2.

HRMS (m/z): (ESI) calc. for $C_{11}H_{14}F_2N_2NaO_6S$ $[M+Na]^+$: 363.0438 found 363.0431

General Synthetic Procedure for cross-coupling of sugar C1 hydrazides and aryl halides

General Procedure J1 (Condition A):

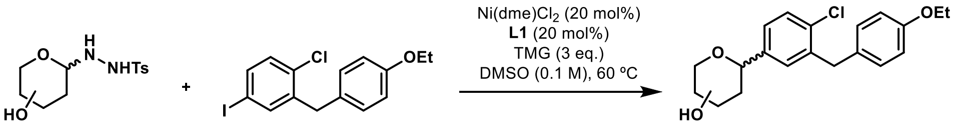

To a reaction tube with a stir bar were added sugar hydrazide (0.20 mmol, 2.0 equiv.), aryl halide **26** (0.10 mmol, 1.0 equiv.), Ni(dme)Cl_2 (20 mol%, 0.2 equiv.), and **L1** (20 mol%, 0.2 equiv.). The tube was capped with a septa cap, then evacuated and backfilled with argon three times. Pre-degassed DMSO (1 mL) was added and the mixture was stirred for 1 minute, followed by the addition of TMG (0.3 mmol, 3.0 equiv.). The reaction mixture was heated to 60 °C and stirred overnight. After the reaction was completed, the reaction mixture was directly purified by a C18 column (RediSep Rf reversed phase C18 column 30g, eluting with a gradient flow over 12 minutes of 100:0 A/B to 0:100 A/B, 214 nm UV detection). Solvent A is H_2O with 0.1% formic acid; solvent B is MeCN. At a flow rate of 35 mL/min, the desired product has a retention time of 6-8 minutes.

Note: The purified products can be further purified using preparative HPLC for separating the diastereomers.

General Procedure J2 (Condition B):

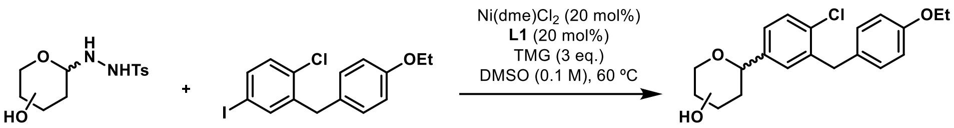

To a reaction tube with a stir bar were added sugar hydrazide (0.20 mmol, 2.0 equiv.), aryl halide **26** (0.10 mmol, 1.0 equiv.), Ni(dme)Cl_2 (20 mol%, 0.2 equiv.), and **L1** (20 mol%, 0.2 equiv.). The tube was capped with a septa cap, then evacuated and backfilled with argon three times. Pre-degassed DMSO (1 mL) was added and the mixture was stirred for 1 minute, followed by the addition of TEA (0.3 mmol, 3.0 equiv.). The reaction mixture was heated to 60 °C and stirred overnight. After the reaction was completed, the reaction mixture was directly purified by a C18 column (RediSep Rf reversed phase C18 column 30g, eluting with a gradient flow over 12 minutes of 100:0 A/B to 0:100 A/B, 214 nm UV detection). Solvent A is H_2O with 0.1% formic acid; solvent B is MeCN. At a flow rate of 35 mL/min, the desired product has a retention time of 6-8 minutes.

Note: The purified products can be further purified using preparative HPLC for separating the diastereomers.

General Procedure J3 (Condition C):

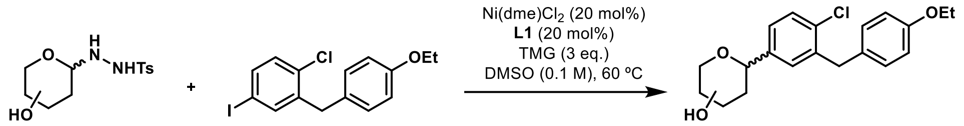

To a reaction tube with a stir bar were added sugar hydrazide (0.20 mmol, 2.0 equiv.), aryl halide **26** (0.10 mmol, 1.0 equiv.), Ni(dme)Cl_2 (20 mol%, 0.2 equiv.), and **L4** (20 mol%, 0.2 equiv.). The tube was capped with a septa cap, then evacuated and backfilled with argon three times. Pre-degassed DMSO (1 mL) was added and the mixture was stirred for 1 minute, followed by the addition of TEA (0.3 mmol, 3.0 equiv.). The reaction mixture was heated to 60 °C and stirred overnight. After the reaction was completed, the reaction mixture was directly purified by a C18 column (RediSep Rf reversed phase C18 column 30g, eluting with a gradient flow over 12 minutes of 100:0 A/B to 0:100 A/B, 214 nm UV detection). Solvent A is H_2O with 0.1% formic acid; solvent B is MeCN. At a flow rate of 35 mL/min, the desired product has a retention time of 6-8 minutes.

Note: The purified products can be further purified using preparative HPLC for separating the diastereomers.

Compound 47 and 48

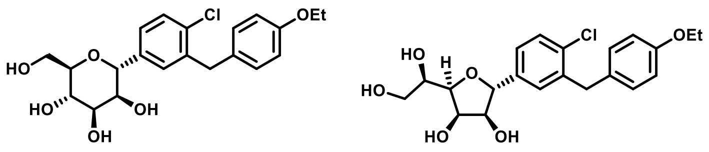

Following **General Procedure J1** with mannose-hydrazide **β-SH5** (0.2 mmol), purification by reverse phase column chromatography afforded compound **47** (23 mg, 56% yield, α:β > 19:1) as colorless oil.

Following **General Procedure J2** with mannose-hydrazide **β-73** (0.2 mmol), purification by reverse phase column chromatography afforded compound **47** (37 mg, 90% yield, α:β > 19:1) and **48** (3 mg, 8% yield, α:β > 19:1) as colorless oil.

α–47

$[a]_D^{25}$= +40.3° (c = 1.0, MeOH)

^{1}H NMR (600 MHz, CD_3OD) δ 7.38 – 7.35 (m, 2H), 7.31 – 7.28 (m, 1H), 7.11 – 7.07 (m, 2H), 6.82 – 6.78 (m, 2H), 4.89 (d, *J* = 4.1 Hz, 1H), 4.32 (t, *J* = 3.6 Hz, 1H), 4.06 – 3.99 (m, 2H), 4.00 – 3.95 (m, 2H), 3.83 (dd, *J* = 11.9, 6.4 Hz, 1H), 3.77 – 3.72 (m, 2H), 3.55 (dd, *J* = 7.8, 3.1 Hz, 1H), 3.43 (td, *J* = 7.0, 2.9 Hz, 1H), 1.35 (t, *J* = 7.0 Hz, 3H).

^{13}C NMR (151 MHz, CD_3OD) δ 158.9, 140.7, 138.7, 134.06, 132.8, 130.9, 130.7, 130.6, 127.1, 115.5, 77.9, 77.6, 72.7, 71.6, 69.6, 64.4, 62.6, 39.2, 15.2.

HRMS (m/z): (ESI) calc. for $C_{21}H_{26}ClO_6$ $[M+H]^+$: 409.1418 found 409.1411.

α–48

$[a]_D^{25}$= +26.0° (c = 0.1, MeOH)

^{1}H NMR (600 MHz, CD_3OD) δ 7.34 (d, *J* = 8.2 Hz, 1H), 7.28 – 7.22 (m, 2H), 7.08 (d, *J* = 8.6 Hz, 2H), 6.80 (d, *J* = 8.7 Hz, 2H), 4.69 (d, *J* = 8.6 Hz, 1H), 4.26 (t, *J* = 3.7 Hz, 1H), 4.08 (dd, *J* = 8.3, 3.2 Hz, 1H), 4.04 – 3.96 (m, 5H), 3.94 (dd, *J* = 8.6, 4.1 Hz, 1H), 3.79 (dd, *J* = 11.5, 3.2 Hz, 1H), 3.62 (dd, *J* = 11.5, 6.0 Hz, 1H), 1.36 (t, *J* = 7.0 Hz, 3H).

^{13}C NMR (151 MHz, CD_3OD) δ 158.9, 142.2, 140.3, 134.0, 132.8, 130.8, 130.4, 129.8, 126.3, 115.4, 83.1, 81.7, 81.1, 73.7, 71.7, 64.9, 64.4, 39.2, 15.2.

HRMS (m/z): (ESI) calc. for $C_{21}H_{25}ClNaO_6$ $[M+Na]^+$: 431.1237 found 431.1227.

Compound 49

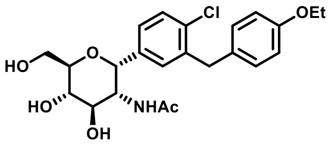

α–49

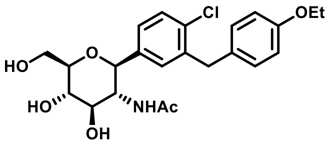

β–49

Following **General Procedure J2** with N-acetylglucosamine-hydrazide **β-SH7** (0.2 mmol), purification by reverse phase column chromatography afforded compound **49** (22 mg, 50% yield, α:β = 8.4:1) as colorless oil.

α–49

$[\alpha]_D^{25}$= +67.4° (c = 0.5, MeOH)

^{1}H NMR (600 MHz, CD_3OD) δ 8.53 (s, 1H), 7.32 (d, *J* = 2.1 Hz, 1H), 7.31 (d, *J* = 8.3 Hz, 1H), 7.26 (dd, *J* = 8.4, 2.1 Hz, 1H), 7.07 (d, *J* = 8.3 Hz, 2H), 6.82 – 6.77 (m, 2H), 5.20 (d, *J* = 3.3 Hz, 1H), 4.14 (dd, *J* = 5.4, 3.4 Hz, 1H), 4.07 – 3.94 (m, 5H), 3.89 – 3.82 (m, 2H), 3.69 (dd, *J* = 12.0, 3.9 Hz, 1H), 3.61 (t, *J* = 4.4 Hz, 1H), 1.72 (s, 3H), 1.35 (t, *J* = 7.0 Hz, 3H).

^{13}C NMR (151 MHz, CD_3OD) δ 172.5, 158.8, 140.0, 139.5, 133.9, 133.0, 131.1, 130.8, 130.0, 127.5, 115.5, 80.3, 71.4, 70.8, 70.1, 64.4, 61.4, 54.4, 39.2, 22.6, 15.2.

HRMS (m/z): (ESI) calc. for $C_{23}H_{29}ClNO_6$ $[M+H]^+$: 450.1683 found 450.1687.

β–49

$[\alpha]_D^{25}$= +9.5° (c = 0.2, MeOH)

^{1}H NMR (600 MHz, CD_3OD) δ 7.31 (d, *J* = 8.2 Hz, 1H), 7.27 (dd, *J* = 8.3, 2.1 Hz, 1H), 7.21 (d, *J* = 2.1 Hz, 1H), 7.08 – 7.03 (m, 2H), 6.82 – 6.78 (m, 2H), 4.21 (d, *J* = 10.1 Hz, 1H), 4.05 (d, *J* = 15.2 Hz, 1H), 4.00 (d, *J* = 7.0 Hz, 1H), 3.99 – 3.94 (m, 2H), 3.89 (dd, *J* = 12.0, 2.2 Hz, 1H), 3.80 (t, *J* = 10.0 Hz, 1H), 3.71 (dd, *J* = 12.0, 5.8 Hz, 1H), 3.53 (dd, *J* = 10.0, 8.6 Hz, 1H), 3.43 (dd, *J* = 9.7, 8.6 Hz, 1H), 3.38 (ddd, *J* = 9.7, 5.8, 2.2 Hz, 1H), 1.67 (s, 3H), 1.36 (t, *J* = 7.0 Hz, 3H).

^{13}C NMR (151 MHz, CD_3OD) δ 139.6, 139.3, 134.6, 132.9, 131.7, 130.8, 130.2, 128.0, 115.5, 82.4, 81.6, 77.3, 72.3, 64.4, 63.1, 58.3, 39.1, 22.7, 15.2.

HRMS (m/z): (ESI) calc. for $C_{23}H_{29}ClNO_6$ $[M+H]^+$: 450.1683 found 450.1690.

Compound 51

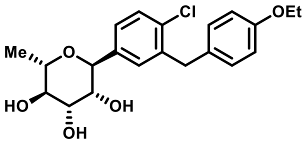

α–51

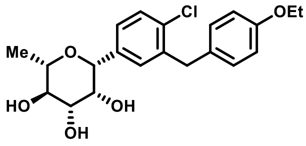

β–51

Following **General Procedure J1** with rhamnose-hydrazide **β-SH10** (0.2 mmol), purification by reverse phase column chromatography afforded compound **51** (38 mg, 98% yield, α:β = 5.3:1) as colorless oil.

Following **General Procedure J2** with rhamnose-hydrazide **β-SH11** (0.2 mmol), purification by reverse phase column chromatography afforded compound **51** (27 mg, 70% yield, α:β > 19:1) as colorless oil.

α–51

$[\alpha]_D^{25}$= –41.9° (c = 1.0, MeOH)

^{1}H NMR (600 MHz, CD_3OD) δ 7.37 (d, *J* = 8.1 Hz, 1H), 7.26 – 7.20 (m, 2H), 7.11 – 7.06 (m, 2H), 6.85 – 6.79 (m, 2H), 4.84 (d, *J* = 3.6 Hz, 1H), 4.35 (t, *J* = 3.3 Hz, 1H), 4.06 (d, *J* = 15.4 Hz, 1H), 4.01 – 3.96 (m, 3H), 3.53 (t, *J* = 7.9 Hz, 1H), 3.47 (dd, *J* = 8.2, 3.1 Hz, 1H), 3.36 (d, *J* = 12.4 Hz, 1H), 1.36 (t, *J* = 7.0 Hz, 3H), 1.26 (d, *J* = 6.4 Hz, 3H).

^{13}C NMR (151 MHz, CD_3OD) δ 158.9, 140.8, 138.7, 134.0, 132.6, 131.1, 130.9, 130.6, 130.3, 126.8, 115.6, 77.9, 74.2, 72.7, 71.7, 64.4, 39.2, 17.8, 15.2.

HRMS (m/z): (ESI) calc. for $C_{21}H_{25}ClNaO_5$ $[M+Na]^+$: 415.1288 found 415.1282.

β–52

$[\alpha]_D^{25}$= –23.0° (c = 0.2, MeOH)

^{1}H NMR (600 MHz, CD_3OD) δ 7.34 – 7.29 (m, 2H), 7.25 (dd, *J* = 8.2, 2.1 Hz, 1H), 7.10 – 7.06 (m, 2H), 6.81 – 6.77 (m, 2H), 4.50 (d, *J* = 1.1 Hz, 1H), 4.01 (s, 2H), 3.98 (q, *J* = 7.0 Hz, 2H), 3.91 (dd, *J* = 3.4, 1.2 Hz, 1H), 3.58 (dd, *J* = 9.2, 3.4 Hz, 1H), 3.40 (t, *J* = 9.2 Hz, 1H), 3.38 – 3.33 (m, 1H), 1.37 – 1.34 (m, 6H).

^{13}C NMR (151 MHz, CD_3OD) δ 158.8, 139.9, 139.7, 133.7, 133.0, 130.8, 130.5, 129.9, 127.2, 115.4, 80.4, 77.8, 76.4, 74.1, 74.0, 64.4, 39.3, 18.3, 15.2.

HRMS (m/z): (ESI) calc. for $C_{21}H_{24}ClO_4$ $[M–OH]^+$: 375.1363 found 375.1349.

Compound 53

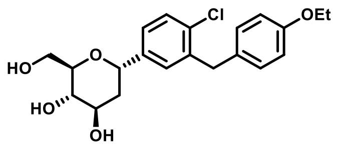

α–53

Following **General Procedure J2** with 2-deoxyglucose-hydrazide **β-SH9** (0.2 mmol), purification by reverse phase column chromatography afforded compound **53** (13 mg, 32% yield, α:β > 19:1) as colorless oil.

α–53

$[\alpha]_D^{25}$= +49.4° (c = 0.5, MeOH)

^{1}H NMR (600 MHz, CD_3OD) δ 7.38 – 7.34 (m, 2H), 7.30 (dd, *J* = 8.3, 2.2 Hz, 1H), 7.11 – 7.07 (m, 2H), 6.83 – 6.79 (m, 2H), 5.06 (dd, *J* = 5.8, 2.7 Hz, 1H), 4.06 – 4.00 (m, 2H), 4.01 – 3.96 (m, 2H), 3.75 (dd, *J* = 11.8, 2.9 Hz, 1H), 3.71 (dd, *J* = 11.8, 5.6 Hz, 1H), 3.53 (ddd, *J* = 10.8, 8.0, 4.4 Hz, 1H), 3.31 – 3.29 (m, 1H), 3.25 (ddd, *J* = 8.7, 5.6, 2.9 Hz, 1H), 2.54 (ddd, *J* = 13.7, 4.5, 2.7 Hz, 1H), 1.92 (ddd, *J* = 13.8, 10.9, 5.5 Hz, 1H), 1.36 (t, *J* = 7.0 Hz, 3H).

^{13}C NMR (151 MHz, CD_3OD) δ 158.9, 140.8, 140.5, 133.8, 132.8, 130.9, 130.6, 130.5, 127.0, 115.5, 76.6, 73.3, 73.1, 70.2, 64.4, 62.9, 39.3, 35.6, 15.2.

HRMS (m/z): (ESI) calc. for $C_{21}H_{26}ClO_5$ $[M+H]^+$: 393.1469 found 393.1469.

Compound 55 and 56

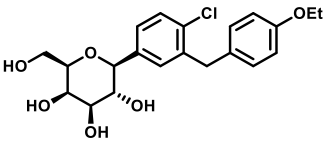
β−55

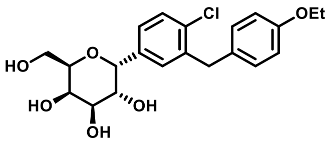
α−55

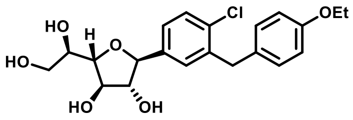
β−56

Following **General Procedure J1** with galactose-hydrazide **β-SH12** (0.2 mmol), purification by reverse phase column chromatography afforded compound **56** (30 mg, 74% yield, β:α > 19:1) as colorless oil.

Following **General Procedure J2** with galactose-hydrazide **β-SH3** (0.2 mmol), purification by reverse phase column chromatography afforded compound **55** (12 mg, 31% yield, β:α = 1:1) and **56** (24 mg, 58% yield, β:α > 19:1) as colorless oil.

β−55

$[\alpha]_D^{25}$= +21.0° (c = 0.1, MeOH)

^{1}H NMR (600 MHz, CD_3OD) δ 7.39 (d, *J* = 1.8 Hz, 1H), 7.35 – 7.30 (m, 2H), 7.10 (d, *J* = 8.3 Hz, 2H), 6.79 (d, *J* = 8.4 Hz, 2H), 4.08 – 3.98 (m, 4H), 3.97 (d, *J* = 7.0 Hz, 1H), 3.95 (d, *J* = 3.3 Hz, 1H), 3.77 (dd, *J* = 11.4, 6.9 Hz, 1H), 3.71 (dd, *J* = 11.4, 5.1 Hz, 1H), 3.65 (t, *J* = 9.4 Hz, 1H), 3.62 (dd, *J* = 6.8, 5.2 Hz, 1H), 3.55 (dd, *J* = 9.4, 3.3 Hz, 1H), 1.35 (t, *J* = 7.0 Hz, 3H).

^{13}C NMR (151 MHz, CD_3OD) δ 158.8, 140.2, 139.9, 134.4, 133.0, 132.2, 130.8, 130.1, 128.5, 115.4, 83.7, 80.9, 76.4, 73.4, 71.0, 64.4, 62.9, 39.3, 15.2.

HRMS (m/z): (ESI) calc. for $C_{21}H_{25}ClNaO_6$ $[M+Na]^+$: 431.1237 found 431.1233.

α−55

$[\alpha]_D^{25}$= +11.5° (c = 0.2, MeOH)

^{1}H NMR (600 MHz, CD_3OD) δ 7.46 (s, 1H), 7.40 (d, *J* = 8.3 Hz, 1H), 7.31 (dd, *J* = 8.3, 1.7 Hz, 1H), 7.12 – 7.07 (m, 2H), 6.81 – 6.77 (m, 2H), 5.06 (d, *J* = 2.6 Hz, 1H), 4.12 (dd, *J* = 5.3, 3.0 Hz, 1H), 4.07 (dd, *J* = 12.4, 8.1 Hz, 1H), 4.01 (s, 2H), 4.01 – 3.96 (m, 2H), 3.93 – 3.85 (m, 3H), 3.76 – 3.71 (m, 1H), 1.35 (td, *J* = 7.1, 1.7 Hz, 3H).

^{13}C NMR (151 MHz, CD_3OD) δ 158.8, 139.8, 139.6, 133.6, 133.0, 131.5, 130.8, 129.9,

128.1, 115.4, 78.1, 73.4, 72.3, 72.0, 67.8, 64.4, 60.2, 39.3, 15.2.

HRMS (m/z): (ESI) calc. for $C_{21}H_{25}ClNaO_6$ $[M+Na]^+$: 431.1237 found 431.1229.

β–56

$[\alpha]_D^{25}$= –20.0° (c = 0.2, MeOH)

H NMR (600 MHz, CD_3OD) δ 7.35 (d, *J* = 8.2 Hz, 1H), 7.30 (d, *J* = 2.1 Hz, 1H), 7.26 (dd, *J* = 8.2, 2.2 Hz, 1H), 7.10 – 7.05 (m, 2H), 6.83 – 6.77 (m, 2H), 4.65 (d, *J* = 7.8 Hz, 1H), 4.23 (t, *J* = 7.0 Hz, 1H), 4.07 – 3.93 (m, 5H), 3.86 (dd, *J* = 7.9, 6.8 Hz, 1H), 3.73 (ddd, *J* = 6.9, 5.8, 3.0 Hz, 1H), 3.67 – 3.60 (m, 2H), 1.36 (t, *J* = 7.0 Hz, 3H).

^{13}C NMR (151 MHz, CD_3OD) δ 158.9, 141.7, 140.3, 134.1, 132.8, 130.8, 130.4, 130.1, 126.6, 115.4, 84.7, 84.6, 83.4, 78.4, 72.9, 64.4, 64.3, 39.2, 15.2.

HRMS (m/z): (ESI) calc. for $C_{21}H_{25}ClNaO_6$ $[M+Na]^+$: 431.1237 found 431.1241.

Compound 57 and 58

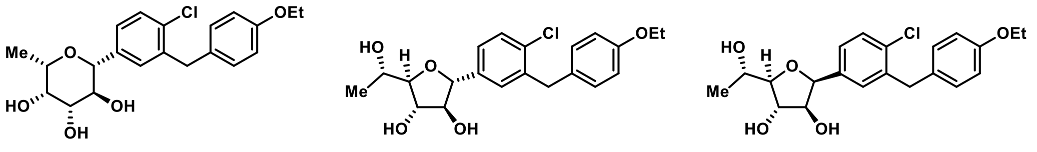

β–57 β–58 α–58

Following **General Procedure J1** with fucose-hydrazide **β-SH14** (0.2 mmol), purification by reverse phase column chromatography afforded compound **58** (37 mg, 95% yield, α:β > 19:1) as colorless oil.

Following **General Procedure J2** with fucose-hydrazide **β-SH15** (0.2 mmol), purification by reverse phase column chromatography afforded compound **57** (6 mg, 16% yield, β:α > 19:1) and **58** (26 mg, 66% yield, β:α = 1:1) as colorless oil.

Following **General Procedure J3** with fucose-hydrazide **β-SH14** (0.2 mmol), purification by reverse phase column chromatography afforded compound **57** (32 mg, 81% yield, β:α > 19:1) as colorless oil.

α–58

$[\alpha]_D^{25}$= +21.6° (c = 0.5, MeOH)

^{1}H NMR (600 MHz, CD_3OD) δ 7.35 (d, *J* = 8.2 Hz, 1H), 7.30 (d, *J* = 2.0 Hz, 1H), 7.26 (dd, *J* = 8.2, 2.1 Hz, 1H), 7.08 (d, *J* = 8.3 Hz, 2H), 6.80 (d, *J* = 8.3 Hz, 2H), 4.62 (d, *J* = 7.8 Hz, 1H), 4.07 (t, *J* = 6.8 Hz, 1H), 4.02 (d, *J* = 9.4 Hz, 2H), 3.99 (q, *J* = 7.0 Hz, 2H), 3.87 – 3.83 (m, 2H), 3.69 (dd, *J* = 6.7, 4.8 Hz, 1H), 1.36 (t, *J* = 7.0 Hz, 3H), 1.24 (d, *J* = 6.4 Hz, 3H).

^{13}C NMR (151 MHz, CD_3OD) δ 158.9, 141.5, 140.3, 134.1, 132.8, 130.8, 130.3, 130.1, 126.6, 115.4, 87.7, 85.1, 84.2, 79.2, 68.7, 64.4, 39.2, 19.5, 15.2.

HRMS (m/z): (ESI) calc. for $C_{21}H_{25}ClNaO_5$ $[M+Na]^+$: 415.1288 found 415.1278.

β–58

$[\alpha]_D^{25}$= –9.4° (c = 0.5, MeOH)

^{1}H NMR (600 MHz, CD_3OD) δ 7.44 – 7.40 (m, 2H), 7.31 (d, *J* = 8.9 Hz, 1H), 7.10 – 7.07 (m, 2H), 6.82 – 6.76 (m, 2H), 5.00 (d, *J* = 3.4 Hz, 1H), 4.02 – 3.99 (m, 3H), 3.97

(dd, J = 13.8, 6.8 Hz, 2H), 3.90 (dd, J = 6.5, 3.3 Hz, 1H), 3.88 – 3.84 (m, 2H), 1.35 (t, J = 7.0 Hz, 3H), 1.30 (d, J = 6.4 Hz, 3H).

^{13}C NMR (151 MHz, CD_3OD) δ 158.8, 139.9, 139.6, 133.6, 133.0, 131.5, 130.8, 129.9, 128.1, 115.4, 73.0, 72.8, 72.3, 72.2, 69.4, 64.4, 39.3, 15.2, 15.0.

HRMS (m/z): (ESI) calc. for $C_{21}H_{25}ClNaO_5$ $[M+Na]^+$: 415.1288 found 415.1277.

β–57

$[\alpha]_D^{25}$= –11.0° (c = 0.2, MeOH)

^{1}H NMR (600 MHz, CD_3OD) δ 7.38 (d, J = 1.9 Hz, 1H), 7.36 – 7.28 (m, 2H), 7.10 (d, J = 8.6 Hz, 2H), 6.79 (d, J = 8.6 Hz, 2H), 4.05 (d, J = 15.1 Hz, 1H), 4.02 – 3.91 (m, 4H), 3.78 – 3.70 (m, 2H), 3.61 (t, J = 9.3 Hz, 1H), 3.55 (dd, J = 9.4, 3.3 Hz, 1H), 1.35 (t, J = 7.0 Hz, 3H), 1.27 (d, J = 6.4 Hz, 3H).

^{13}C NMR (151 MHz, CD_3OD) δ 158.8, 140.3, 139.9, 134.4, 133.0, 132.2, 130.8, 130.1, 128.5, 115.4, 83.6, 76.6, 76.1, 73.7, 73.1, 64.4, 39.3, 17.2, 15.2.

HRMS (m/z): (ESI) calc. for $C_{21}H_{25}ClNaO_5$ $[M+Na]^+$: 415.1288 found 415.1278.

Compound 59 and 60

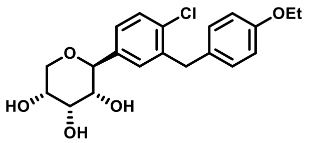
β–59

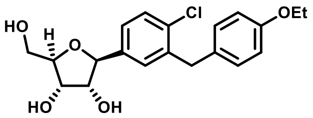
β–60

Following **General Procedure J1** with ribose-hydrazide **β-78** (0.2 mmol), purification by reverse phase column chromatography afforded compound **59** (6 mg, 17% yield, β:α > 19:1) and **60** (28 mg, 73% yield, β:α > 19:1) as colorless oil.

Following **General Procedure J2** with ribose-hydrazide **β-SH16** (0.2 mmol), purification by reverse phase column chromatography afforded compound **59** (7 mg, 20% yield, β:α > 19:1) and **60** (16 mg, 41% yield, β:α > 19:1) as colorless oil.

β–60

$[\alpha]_D^{25}$= –25.0° (c = 0.5, MeOH)

^{1}H NMR (600 MHz, CD_3OD) δ 7.34 (d, *J* = 8.3 Hz, 2H), 7.30 (dd, *J* = 8.2, 2.1 Hz, 1H), 7.10 – 7.06 (m, 2H), 6.82 – 6.78 (m, 2H), 4.64 (d, *J* = 6.8 Hz, 1H), 4.06 – 3.96 (m, 5H), 3.95 (q, *J* = 4.2 Hz, 1H), 3.79 (dd, *J* = 6.8, 5.5 Hz, 1H), 3.75 (dd, *J* = 11.9, 3.8 Hz, 1H), 3.68 (dd, *J* = 12.0, 4.9 Hz, 1H), 1.35 (t, *J* = 7.0 Hz, 3H).

^{13}C NMR (151 MHz, CD_3OD) δ 158.9, 141.1, 140.3, 134.2, 132.9, 130.8, 130.3, 130.2, 126.7, 115.4, 86.5, 84.8, 79.2, 72.9, 64.4, 63.6, 39.2, 15.2.

HRMS (m/z): (ESI) calc. for $C_{20}H_{23}ClNaO_5$ $[M+Na]^+$: 401.1132 found 401.1119.

β–59

$[\alpha]_D^{25}$= –16.0° (c = 0.2, MeOH)

^{1}H NMR (600 MHz, CD_3OD) δ 7.33 (d, *J* = 8.2 Hz, 1H), 7.26 (d, *J* = 2.1 Hz, 1H), 7.22 (dd, *J* = 8.2, 2.3 Hz, 1H), 7.09 (d, *J* = 8.6 Hz, 2H), 6.80 (d, *J* = 8.6 Hz, 2H), 4.31 (d, *J* = 9.6 Hz, 1H), 4.08 (t, *J* = 2.8 Hz, 1H), 4.03 – 3.94 (m, 4H), 3.77 (ddd, *J* = 10.8, 5.4, 2.8 Hz, 1H), 3.71 – 3.60 (m, 2H), 3.42 (dd, *J* = 9.7, 2.8 Hz, 1H), 1.36 (t, *J* = 7.0 Hz, 3H).

^{13}C NMR (151 MHz, CD_3OD) δ 158.9, 140.5, 139.9, 134.3, 132.9, 131.7, 130.9, 130.8, 130.1, 128.1, 115.4, 115.4, 78.0, 73.6, 72.7, 68.8, 66.8, 64.4, 39.2, 15.2.

HRMS (m/z): (ESI) calc. for $C_{20}H_{23}ClNaO_5$ $[M+Na]^+$: 401.1132 found 401.1142.

Compound 62

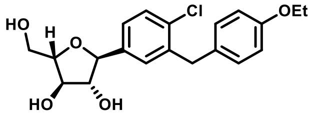

β–62

Following **General Procedure J1** with arabinose-hydrazide **α-SH17** (0.2 mmol), purification by reverse phase column chromatography afforded compound **62** (27 mg, 72% yield, β:α > 19:1) as colorless oil.

Following **General Procedure J2** with arabinose-hydrazide **α-SH18** (0.2 mmol), purification by reverse phase column chromatography afforded compound **62** (16 mg, 41% yield, β:α > 19:1) as colorless oil.

β–62

$[\alpha]_D^{25}$ = –22.5° (c = 0.2, MeOH)

^{1}H NMR (600 MHz, CD_3OD) δ 7.35 (d, *J* = 8.2 Hz, 1H), 7.31 (d, *J* = 2.1 Hz, 1H), 7.26 (dd, *J* = 8.3, 2.2 Hz, 1H), 7.10 – 7.05 (m, 2H), 6.82 – 6.77 (m, 2H), 4.64 (d, *J* = 7.5 Hz, 1H), 4.07 (t, *J* = 6.6 Hz, 1H), 4.02 (d, *J* = 5.3 Hz, 2H), 4.01 – 3.94 (m, 3H), 3.87 (dd, *J* = 7.6, 6.4 Hz, 1H), 3.76 (dd, *J* = 12.0, 3.2 Hz, 1H), 3.68 (dd, *J* = 12.0, 5.2 Hz, 1H), 1.36 (t, *J* = 7.0 Hz, 3H).

^{13}C NMR (151 MHz, CD_3OD) δ 158.9, 141.5, 140.3, 134.1, 132.8, 130.8, 130.4, 130.0, 126.6, 115.4, 85.0, 85.0, 84.5, 78.3, 64.4, 63.2, 39.2, 15.2.

HRMS (m/z): (ESI) calc. for $C_{20}H_{23}ClNaO_5$ $[M+Na]^+$: 401.1132 found 401.1128.

Compound 63

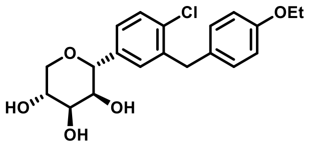

α–63

Following **General Procedure J2** with lyxose-hydrazide **SH20** (0.2 mmol), purification by reverse phase column chromatography afforded compound **63** (12 mg, 32% yield, α:β > 19:1) as colorless oil.

α–63

$[\alpha]_D^{25}$= +6.5° (c = 0.2, MeOH)

^{1}H NMR (600 MHz, CD_3OD) δ 7.37 (d, *J* = 2.1 Hz, 1H), 7.33 (d, *J* = 8.2 Hz, 1H), 7.29 (dd, *J* = 8.3, 2.1 Hz, 1H), 7.12 – 7.07 (m, 2H), 6.82 – 6.76 (m, 2H), 4.37 (d, *J* = 9.7 Hz, 1H), 4.06 – 3.91 (m, 6H), 3.81 – 3.71 (m, 3H), 1.35 (t, *J* = 7.0 Hz, 3H).

^{13}C NMR (151 MHz, CD_3OD) δ 158.8, 140.8, 139.9, 134.2, 133.0, 131.9, 130.8, 130.0, 128.3, 115.4, 79.0, 72.0, 71.8, 70.7, 68.3, 64.4, 39.3, 15.2.

HRMS (m/z): (ESI) calc. for $C_{20}H_{22}ClO_4$ $[M\text{-}OH]^+$: 361.1207 found 361.1196.

Compound 65 and Compound 66

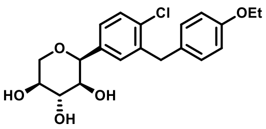

α–65

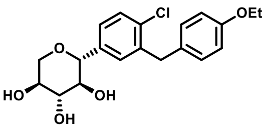

β–65

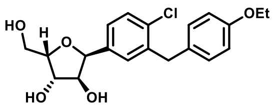

α–66

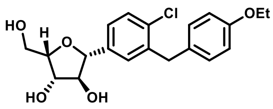

β–66

Following **General Procedure J1** with xylose-hydrazide **β-SH21** (0.2 mmol), purification by reverse phase column chromatography afforded compound **66** (17 mg, 45% yield, β:α = 2.2:1) as colorless oil.

Following **General Procedure J2** with xylose-hydrazide **β-SH22** (0.2 mmol), purification by reverse phase column chromatography afforded compound **65** (12 mg, 32% yield, β:α = 1:1) and **66** (9 mg, 25% yield, β:α = 1.5:1) as colorless oil.

α–65:

$[\alpha]_D^{25}$= +47.5° (c = 0.2, MeOH)

^{1}H NMR (600 MHz, CD_3OD) δ 7.32 (dt, *J* = 8.2, 1.5 Hz, 1H), 7.28 – 7.21 (m, 2H), 7.08 (dd, *J* = 8.5, 2.5 Hz, 2H), 6.80 (dt, *J* = 8.7, 1.5 Hz, 2H), 4.73 (s, 1H), 4.06 – 3.90 (m, 7H), 3.61 (dt, *J* = 3.1, 1.5 Hz, 1H), 3.58 (dq, *J* = 3.2, 1.5 Hz, 1H), 1.36 (tt, *J* = 7.0, 1.5 Hz, 3H).

^{13}C NMR (151 MHz, CD_3OD) δ 158.8, 140.4, 139.8, 133.6, 132.9, 130.8, 130.4, 129.9, 127.1, 115.4, 77.3, 73.6, 70.1, 69.7, 69.7, 64.4, 39.3, 15.2.

HRMS (m/z): (ESI) calc. for $C_{20}H_{23}ClNaO_5$ $[M+Na]^+$: 401.1132 found 401.1122.

β–65:

$[\alpha]_D^{25}$= +12.5° (c = 0.2, MeOH)

^{1}H NMR (600 MHz, CD_3OD) δ 7.34 (d, *J* = 8.2 Hz, 1H), 7.25 (d, *J* = 2.1 Hz, 1H), 7.21 (dd, *J* = 8.2, 2.2 Hz, 1H), 7.11 – 7.06 (m, 2H), 6.83 – 6.77 (m, 2H), 4.06 – 3.97 (m, 5H), 3.95 (dd, *J* = 11.1, 5.4 Hz, 1H), 3.59 (ddd, *J* = 10.6, 9.0, 5.5 Hz, 1H), 3.38 (t, *J* = 8.9 Hz, 1H), 3.30 – 3.22 (m, 2H), 1.36 (t, *J* = 7.0 Hz, 3H).

^{13}C NMR (151 MHz, CD_3OD) δ 158.9, 140.1, 140.0, 134.5, 132.8, 131.6, 130.9, 130.1, 128.0, 115.5, 83.8, 79.8, 76.4, 71.5, 71.4, 64.4, 39.2, 15.2.

HRMS (m/z): (ESI) calc. for $C_{20}H_{22}ClO_4$ $[M–OH]^+$: 361.1207 found 361.1219.

α–66:

$[\alpha]_D^{25}$= +47.0° (c = 0.1, MeOH)

^{1}H NMR (600 MHz, CD_3OD) δ 7.32 (d, *J* = 8.2 Hz, 1H), 7.28 (d, *J* = 2.1 Hz, 1H), 7.21 (dd, *J* = 8.2, 2.1 Hz, 1H), 7.12 – 7.06 (m, 2H), 6.82 – 6.77 (m, 2H), 5.14 (d, *J* = 3.2 Hz, 1H), 4.34 (ddd, *J* = 6.4, 5.1, 3.6 Hz, 1H), 4.25 (dd, *J* = 3.6, 1.4 Hz, 1H), 4.06 (dd, *J* = 3.2, 1.4 Hz, 1H), 4.00 (d, *J* = 7.0 Hz, 1H), 3.97 (d, *J* = 7.0 Hz, 1H), 3.84 (dd, *J* = 11.5, 5.1 Hz, 1H), 3.80 (dd, *J* = 11.5, 6.4 Hz, 1H), 1.35 (t, *J* = 7.0 Hz, 3H).

^{13}C NMR (151 MHz, CD_3OD) δ 158.8, 139.7, 138.5, 133.7, 133.0, 131.2, 130.8, 129.9, 127.8, 115.4, 83.5, 82.8, 80.2, 78.8, 64.4, 62.0, 39.3, 15.2.

HRMS (m/z): (ESI) calc. for $C_{20}H_{22}ClO_4$ $[M–OH]^+$: 361.1207 found 361.1202.

β–66:

$[\alpha]_D^{25}$= +18.0° (c = 0.1, MeOH)

^{1}H NMR (600 MHz, CD_3OD) δ 7.37 (d, *J* = 1.7 Hz, 1H), 7.32 (d, *J* = 2.0 Hz, 2H), 7.11 – 7.05 (m, 2H), 6.82 – 6.77 (m, 2H), 4.54 (d, *J* = 4.7 Hz, 1H), 4.15 – 4.09 (m, 2H), 4.01 (s, 2H), 4.00 (d, *J* = 7.0 Hz, 1H), 3.98 (d, *J* = 7.0 Hz, 1H), 3.91 – 3.87 (m, 2H), 3.84 (dd, *J* = 11.7, 5.6 Hz, 1H), 1.36 (t, *J* = 7.0 Hz, 3H).

^{13}C NMR (151 MHz, CD_3OD) δ 158.9, 141.3, 140.1, 134.0, 132.9, 130.8, 130.4, 130.2, 126.9, 115.5, 87.2, 86.1, 83.0, 79.7, 64.4, 62.0, 39.2, 15.2.

HRMS (m/z): (ESI) calc. for $C_{20}H_{22}ClO_4$ $[M–OH]^+$: 361.1207 found 361.1193.

Compound SJ4

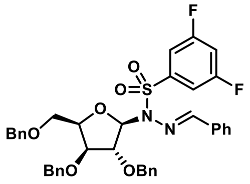

Reaction gave a 1:1.5 mixture of beta/alpha anomers.
Characterized as a 1:1 mixture of beta/alpha anomers.

^{1}H NMR (600 MHz, $CDCl_3$) δ 8.60 (s, 1H), 8.43 (s, 1H), 7.58 – 7.48 (m, 5H), 7.48 – 7.24 (m, 32H), 7.22 – 7.19 (m, 3H), 7.17 – 7.13 (m, 4H), 6.92 (dtt, *J* = 14.9, 8.4, 2.3 Hz, 2H), 6.19 (d, *J* = 6.5 Hz, 1H), 5.99 (d, *J* = 4.5 Hz, 1H), 4.76 (d, *J* = 11.5 Hz, 1H), 4.56 – 4.52 (m, 3H), 4.51 – 4.38 (m, 9H), 4.34 (t, *J* = 6.5 Hz, 1H), 4.31 – 4.27 (m, 2H), 4.25 (dd, *J* = 4.5, 2.5 Hz, 1H), 4.09 (dd, *J* = 5.2, 2.5 Hz, 1H), 3.80 (dd, *J* = 10.4, 5.0 Hz, 1H), 3.67 (dd, *J* = 10.4, 6.5 Hz, 1H), 3.62 (dd, *J* = 10.9, 3.6 Hz, 1H), 3.46 (dd, *J* = 10.9, 6.1 Hz, 1H).

^{13}C NMR (151 MHz, $CDCl_3$) δ 166.9, 162.4 (ddd, *J* = 253.6, 11.5, 7.4 Hz), 158.6, 142.0 (t, *J* = 8.7 Hz), 141.6 (t, *J* = 8.7 Hz), 138.5, 138.2, 137.9, 137.5, 137.4, 137.2, 133.5, 133.2, 132.1, 131.3, 129.1, 128.8, 128.7, 128.62, 128.57, 128.5, 128.42, 128.40, 128.2, 128.14, 128.07, 127.99, 127.96, 127.93, 127.87, 127.79, 127.76, 127.7, 127.6, 112.6 – 112.1 (m), 108.9 (td, *J* = 25.0, 16.0 Hz), 93.4, 86.4, 83.8, 82.6, 82.0, 81.5, 79.2, 73.5, 73.4, 72.6, 72.4, 72.19, 72.15, 70.0, 68.4.

HRMS (m/z): (ESI) calc. $C_{39}H_{37}F_2N_2O_6S$ $[M+H]^+$: 699.2340 found 699.2336

Compound SJ5

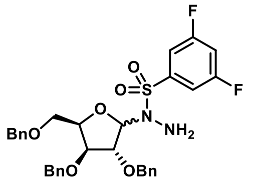

Compound β-SJ5:

$[\boldsymbol{a}]_{\mathbf{D}}^{\mathbf{20}}$ = +52.0° (c = 1, $CHCl_3$)

^{1}H NMR (600 MHz, $CDCl_3$) δ 7.53 – 7.49 (m, 2H), 7.40 – 7.27 (m, 11H), 7.20 (dt, *J* = 7.0, 2.9 Hz, 3H), 7.00 (tt, *J* = 8.4, 2.3 Hz, 1H), 5.82 (d, *J* = 3.5 Hz, 1H), 4.70 (d, *J* = 11.9 Hz, 1H), 4.58 (d, *J* = 11.9 Hz, 1H), 4.54 (d, *J* = 11.7 Hz, 1H), 4.47 (d, *J* = 11.8 Hz, 1H), 4.44 (d, *J* = 11.7 Hz, 1H), 4.38 (d, *J* = 11.7 Hz, 1H), 4.23 (q, *J* = 5.1 Hz, 1H), 4.13 (dd, *J* = 3.6, 2.8 Hz, 1H), 4.04 (dd, *J* = 5.0, 2.8 Hz, 1H), 3.65 (dd, *J* = 10.4, 4.9 Hz, 1H), 3.61 (s, 2H), 3.54 (dd, *J* = 10.4, 5.5 Hz, 1H).

^{13}C NMR (151 MHz, $CDCl_3$) δ 162.4 (dd, *J* = 253.6, 11.4 Hz), 140.3 (t, *J* = 8.6 Hz), 137.9, 137.3, 137.2, 128.7, 128.6, 128.3, 128.0, 127.9, 127.81, 127.76, 112.9 – 112.4 (m), 108.7 (t, *J* = 25.0 Hz), 92.4, 83.2, 81.0, 79.6, 73.5, 72.5, 72.0, 68.0.

HRMS (m/z): (ESI) calc. $C_{32}H_{33}F_2N_2O_6S$ $[M+H]^+$: 611.2027 found 611.2030

Compound α-SJ5:

$[\boldsymbol{a}]_{\mathbf{D}}^{\mathbf{20}}$= –20.2° (c = 1.0, $CHCl_3$)

^{1}H NMR (600 MHz, $CDCl_3$) δ 7.49 (qd, *J* = 6.1, 2.4 Hz, 2H), 7.38 – 7.28 (m, 14H), 7.26 – 7.24 (m, 1H), 7.00 (tt, *J* = 8.5, 2.3 Hz, 1H), 6.10 (d, *J* = 5.6 Hz, 1H), 4.63 (d, *J* = 11.2 Hz, 1H), 4.60 (d, *J* = 12.0 Hz, 1H), 4.55 – 4.47 (m, 4H), 4.35 – 4.30 (m, 2H), 4.12 (dd, *J* = 5.5, 4.1 Hz, 1H), 3.87 (s, 2H), 3.64 (dd, *J* = 10.3, 4.4 Hz, 1H), 3.59 (dd, *J* = 10.3, 4.9 Hz, 1H).

^{13}C NMR (151 MHz, $CDCl_3$) δ 162.5 (dd, *J* = 253.8, 11.5 Hz), 140.7 (t, *J* = 8.5 Hz), 138.0, 137.5, 136.9, 128.7, 128.6, 128.4, 128.3, 128.2, 128.0, 127.74, 127.67, 112.4 – 111.8 (m), 108.6 (t, *J* = 25.1 Hz), 86.9, 83.1, 81.1, 78.9, 73.6, 73.4, 72.6, 68.8.

HRMS (m/z): (ESI) calc. $C_{32}H_{33}F_2N_2O_6S$ $[M+H]^+$: 611.2027 found 611.2033

Compound SJ11

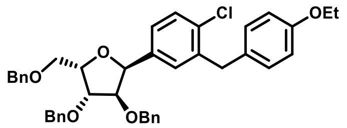

Following **General Procedure E** with Bn-protected xylose hydrazide **SJ5** (0.1 mmol), aryl iodide **26**, and dtbbpy (**L4**), purification through flash column chromatography afforded product **SJ11** (36 mg, 55% yield, α:β = 4.6:1).

$[\boldsymbol{\alpha}]_{\mathbf{D}}^{\mathbf{20}}$ = +65.0° (c = 0.2, $CHCl_3$)

^{1}H NMR (600 MHz, $CDCl_3$) δ 7.36 – 7.26 (m, 12H), 7.22 – 7.17 (m, 4H), 7.16 – 7.13 (m, 2H), 7.04 – 7.01 (m, 2H), 6.79 – 6.75 (m, 2H), 4.78 (d, *J* = 3.7 Hz, 1H), 4.63 (d, *J* = 12.0 Hz, 1H), 4.54 (d, *J* = 12.0 Hz, 1H), 4.46 – 4.40 (m, 3H), 4.03 (dd, *J* = 4.1, 1.4 Hz, 1H), 3.98 – 3.93 (m, 4H), 3.90 (dd, *J* = 3.7, 1.4 Hz, 1H), 3.85 (dd, *J* = 10.1, 5.2 Hz, 1H), 3.81 (dd, *J* = 10.0, 6.3 Hz, 1H), 1.38 (t, *J* = 7.0 Hz, 3H).

^{13}C NMR (151 MHz, $CDCl_3$) δ 157.5, 139.6, 139.0, 138.3, 137.9, 137.6, 133.3, 131.5, 130.0, 129.5, 129.4, 128.6, 128.5, 128.5, 128.1, 127.9, 127.9, 127.7, 127.7, 125.8, 114.5, 89.3, 85.4, 83.1, 80.8, 73.6, 72.1, 71.6, 68.4, 63.5, 38.5, 15.0.

HRMS (m/z): (ESI) calc. $C_{41}H_{41}ClNaO_5$ $[M+Na]^+$: 671.2540 found 671.2558

Compound SJ9

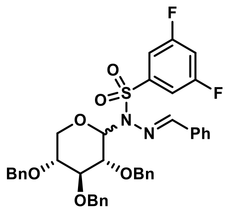

Reaction gave a 2.2:1 mixture of beta/alpha anomers.
Characterized as a 2:1 mixture of beta/alpha anomers.

^{1}H NMR (600 MHz, $CDCl_3$) δ 8.57 (s, 1H), 8.46 (s, 2H), 7.66 – 7.60 (m, 3H), 7.57 – 7.54 (m, 5H), 7.50 – 7.45 (m, 4H), 7.42 – 7.18 (m, 75H), 7.15 – 7.09 (m, 5H), 7.03 (tt, J = 8.5, 2.2 Hz, 1H), 6.98 (ddd, J = 8.4, 6.0, 2.3 Hz, 1H), 6.92 (tt, J = 8.5, 2.4 Hz, 2H), 5.98 (d, J = 6.6 Hz, 1H), 5.34 (d, J = 8.9 Hz, 2H), 4.91 – 4.84 (m, 4H), 4.79 (d, J = 10.7 Hz, 2H), 4.74 (d, J = 11.4 Hz, 1H), 4.68 (dd, J = 11.3, 7.5 Hz, 5H), 4.64 – 4.58 (m, 6H), 4.55 – 4.49 (m, 5H), 4.02 (t, J = 8.7 Hz, 1H), 3.96 (dd, J = 11.5, 5.3 Hz, 2H), 3.87 (t, J = 8.9 Hz, 2H), 3.77 (dd, J = 9.1, 6.6 Hz, 1H), 3.70 (t, J = 9.0 Hz, 2H), 3.61 (ddd, J = 10.4, 9.0, 5.3 Hz, 2H), 3.57 – 3.46 (m, 2H), 3.37 (dd, J = 10.4, 5.3 Hz, 1H), 3.31 (dd, J = 11.5, 10.4 Hz, 2H).

^{13}C NMR (151 MHz, $CDCl_3$) δ 166.9, 163.6, 163.6, 163.4, 163.3, 163.2, 163.1, 162.0, 161.9, 161.7, 161.6, 161.5, 161.5, 159.8, 148.2, 142.4, 142.3, 142.3, 141.9, 141.8, 141.8, 140.8, 140.8, 140.7, 138.5, 138.5, 138.4, 138.2, 138.0, 137.7, 134.8, 133.5, 133.1, 133.0, 132.3, 131.8, 130.7, 129.2, 129.2, 128.9, 128.9, 128.9, 128.7, 128.6, 128.5, 128.4, 128.3, 128.2, 128.2, 128.1, 128.0, 127.9, 127.8, 127.8, 127.6, 127.0, 112.4, 112.4, 112.4, 112.3, 112.2, 112.0, 112.0, 111.9, 111.8, 109.4, 109.2, 109.1, 109.0, 108.9, 108.7, 89.0, 85.6, 83.0, 80.7, 78.3, 78.1, 77.7, 76.1, 75.2, 74.9, 73.5, 73.2, 72.7, 66.1, 64.7, 52.1.

HRMS (m/z): (ESI) calc. $C_{39}H_{37}F_2N_2O_6S$ $[M+H]^+$: 699.2340 found 699.2365

Compound SJ10

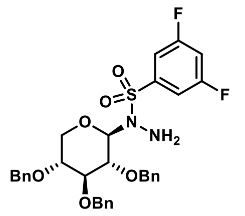

Only one anomer characterized:

$[\alpha]_D^{20}$ = +28.5 ° (c = 0.2, $CHCl_3$)

^{1}H NMR (600 MHz, $CDCl_3$) δ 7.44 – 7.39 (m, 2H), 7.36 – 7.27 (m, 15H), 7.04 (tt, *J* = 8.4, 2.3 Hz, 1H), 5.05 (d, *J* = 8.3 Hz, 1H), 4.94 (d, *J* = 10.9 Hz, 1H), 4.88 (d, *J* = 10.8 Hz, 1H), 4.78 (d, *J* = 11.3 Hz, 1H), 4.75 (d, *J* = 11.3 Hz, 1H), 4.68 (d, *J* = 11.7 Hz, 1H), 4.60 (d, *J* = 11.7 Hz, 1H), 3.79 (dd, *J* = 11.4, 5.4 Hz, 1H), 3.75 – 3.68 (m, 2H), 3.50 – 3.45 (m, 1H), 3.31 (s, 2H), 3.24 (dd, *J* = 11.4, 10.6 Hz, 1H).

^{13}C NMR (151 MHz, $CDCl_3$) δ 162.7 (dd, *J* = 254.5, 11.4 Hz), 140.3 (d, *J* = 8.6 Hz), 138.6, 138.04, 138.01, 128.68, 128.66, 128.59, 128.59, 128.12, 128.10, 127.94, 127.89, 112.6 – 112.1 (m), 109.1 (t, *J* = 25.1 Hz), 87.4, 85.3, 77.4, 75.9, 75.8, 74.5, 73.5, 65.5.

HRMS (m/z): (ESI) calc. $C_{32}H_{33}F_2N_2O_6S$ $[M+H]^+$: 611.2027 found 611.2029

Compound SJ12

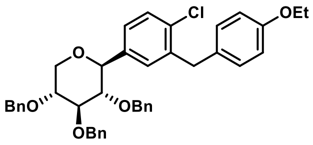

Following **General Procedure E** with Bn-protected xylose hydrazide **SJ10** (0.1 mmol), aryl iodide **26**, and bpy (**L3**), purification through flash column chromatography afforded product **SJ12** (30 mg, 48% yield, β:α > 19:1).

$[\alpha]_D^{20}$ = –5.1° (c = 1.0, $CHCl_3$)

^{1}H NMR (600 MHz, $CDCl_3$) δ 7.39 – 7.29 (m, 11H), 7.26 – 7.18 (m, 5H), 7.06 – 7.02 (m, 2H), 6.92 – 6.87 (m, 2H), 6.78 – 6.74 (m, 2H), 4.96 (d, *J* = 10.9 Hz, 1H), 4.87 (d, *J* = 10.9 Hz, 1H), 4.77 (d, *J* = 11.6 Hz, 1H), 4.70 (s, 1H), 4.43 (d, *J* = 10.5 Hz, 1H), 4.12 – 4.04 (m, 3H), 4.00 – 3.94 (m, 3H), 3.84 (d, *J* = 10.5 Hz, 1H), 3.77 – 3.69 (m, 2H), 3.40 (dd, *J* = 9.5, 8.5 Hz, 1H), 3.33 (dd, *J* = 11.4, 10.1 Hz, 1H), 1.40 (t, *J* = 7.0 Hz, 3H).

^{13}C NMR (151 MHz, $CDCl_3$) δ 157.5, 139.2, 138.7, 138.3, 138.1, 137.8, 134.1, 131.3, 130.4, 129.9, 129.6, 128.6, 128.5, 128.4, 128.13, 128.07, 128.03, 127.98, 127.80, 127.77, 126.7, 114.6, 86.1, 83.7, 81.9, 78.6, 75.9, 75.2, 73.5, 68.5, 63.5, 38.4, 15.0.

HRMS (m/z): (ESI) calc. $C_{41}H_{41}ClNaO_5$ $[M+Na]^+$: 671.2540 found 671.2552

10. Selective Oxidation Followed by C3 Coupling

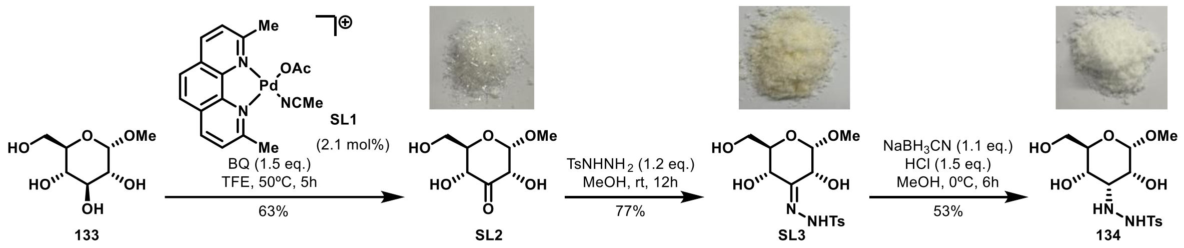

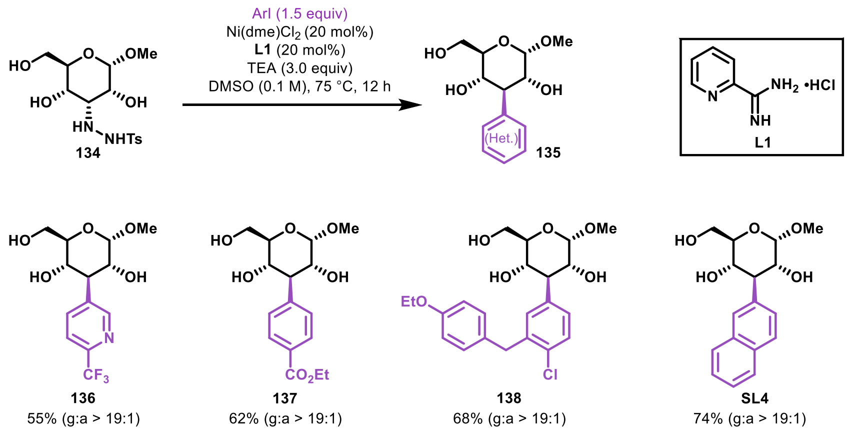

Synthesis of [H-neocuproine][OTf] SL6: [39,40]

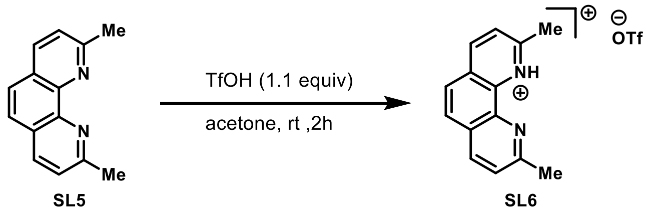

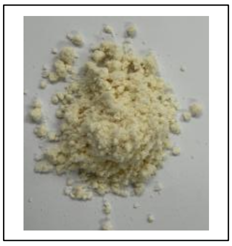

Neocuproine (8.24 g, 39.6 mmol) was dissolved in distilled acetone (120 mL), and triflic acid (3.5 mL, 39.6 mmol) was added with stirring. The solution initially turned dark red and gradually became light orange over several minutes, during which a white precipitate began to form. After 2 h, diethyl ether (400 mL) was added to induce further precipitation. The resulting white solid was collected by filtration, washed with diethyl ether, and dried under reduced pressure to afford compound **SL6** as a white fluffy powder (13.33 g, 37.32 mmol, 94% yield).

^{1}H NMR (400 MHz, CD_3CN) δ 8.71 (d, J = 8.5 Hz, 2H), 8.09 (s, 2H), 7.93 (d, J = 8.4 Hz, 2H), 3.01 (s, 6H).

^{13}C NMR (101 MHz, CD_3CN) δ 160.1, 142.5, 137.5, 128.7, 127.6, 127.4, 123.7, 120.5, 23.1.

HRMS (m/z): (ESI) calc. for $C_{14}H_{13}N_2^+$ $[M+H]^+$: 209.1079 found 209.1080.

Synthesis of [(neocuproine)Pd(μ-OAc)]$_2$[OTf]$_2$ SL1: [39]

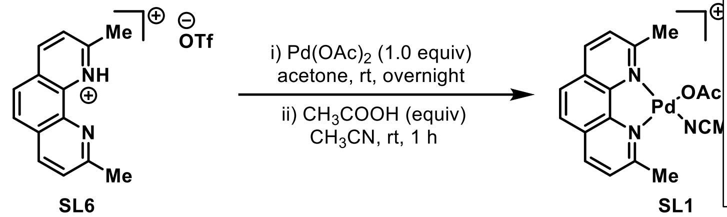

SL6 SL1

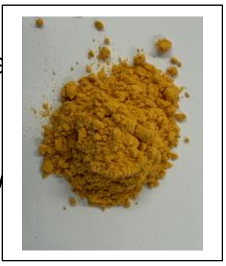

[H-neocuproine][OTf] (2.87 g, 8.02 mmol) was added in two equal portions, 30 min apart, to a stirred solution of $Pd(OAc)_2$ (1.80 g, 8.02 mmol) in acetone (160 mL, dried over $MgSO_4$ and distilled). After 16 h, an orange precipitate had formed and the solution was red-orange. The precipitate was collected by filtration, washed with diethyl ether, and dried under reduced pressure to afford a dull orange powder (2.99 g, 71% yield).

For further purification, the first crop was dissolved in acetonitrile (60 mL) and acetic acid (1.5 mL). After stirring for 1 h, the cloudy solution was filtered through a glass frit and then through syringe filters. Diethyl ether (325 mL) was added to the filtrate to induce precipitation of an orange solid, which was collected by filtration, washed with diethyl ether, and dried under reduced pressure to afford complex **SL1** as a fine bright orange powder (2.71 g, 65% yield). ^{1}H NMR (CD_3CN) and mass spectrometry data were consistent with literature values, indicating a high-purity product.

^{1}H NMR (400 MHz, CD_3CN) δ 8.26 (d, J = 8.4 Hz, 2H), 7.69 (s, 2H), 7.38 (d, J = 8.4 Hz, 2H), 2.62 (s, 6H), 2.24 (s, 3H).

^{13}C NMR (101 MHz, CD_3CN) δ 188.7, 166.8, 147.1, 141.4, 129.3, 129.1, 128.2, 123.7, 120.5, 24.8, 23.9.

Site-selective C3-oxidation of Methyl α-D-glucopyranoside SL2: [39,40]

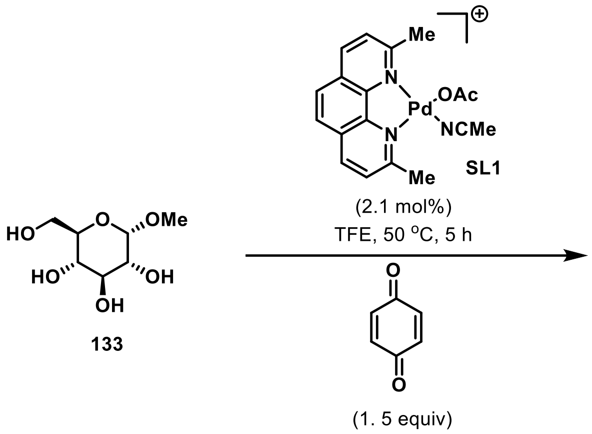

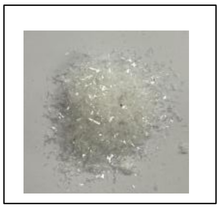

Methyl α-D-glucopyranoside (9.71 g, 50 mmol, 1.0 equiv) was dissolved in 2,2,2-trifluoroethanol (TFE, 200 mL), and the Pd catalyst (2.1 mol%, 1.05 mmol) was added. The reaction mixture was stirred at 50 °C for 5 h. Reaction progress was monitored by TLC using *p*-anisaldehyde stain. Upon completion, the solvent was removed under reduced pressure, and the crude residue was purified by silica gel column chromatography using ethyl acetate/hexane (1:1 to 3:1) as eluent to afford the ketosugar **SL2** as a colorless solid (6.11 g, 63% yield).

^{1}H NMR (400 MHz, CD_3CN) δ 5.04 (dd, *J* = 4.3, 0.6 Hz, 1H), 4.36 (ddd, *J* = 8.4, 4.3, 1.6 Hz, 1H), 4.22 (ddd, *J* = 9.7, 5.3, 1.6 Hz, 1H), 3.80 (ddd, *J* = 12.2, 5.5, 2.3 Hz, 1H), 3.75 – 3.69 (m, 1H), 3.68 (d, *J* = 5.3 Hz, 1H), 3.54 (d, *J* = 8.4 Hz, 2H), 3.35 (s, 3H), 2.96 (dd, *J* = 6.9, 5.6 Hz, 1H).

^{13}C NMR (101 MHz, CD_3CN) δ 207.2, 103.3, 76.5, 75.7, 73.0, 62.1, 55.7.

HRMS (m/z): (ESI) calc. for $C_7H_{12}O_6Na^+$ $[M+Na]^+$: 215.0526 found 215.0524.

Synthesis of C3-Glycohydrazones SL3:

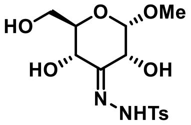

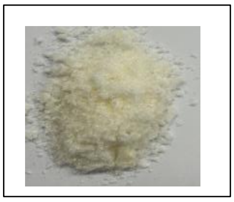

Following general procedure laid out in section **4.6.3 Synthesis of C3-Glycohydrazide (w/o PG)**, the title compound was prepared from ketosugar (4 g, 20.82 mmol) and tosyl hydrazine (5.81 g, 31.22 mmol), the product **SL3** was obtained as a colourless solid (5.78 g, 77% yield).

^{1}H NMR (400 MHz, CD_3OD) δ 7.76 (dd, *J* = 8.4, 2.8 Hz, 2H), 7.43 – 7.28 (m, 2H), 4.74 (d, *J* = 3.6 Hz, 1H, minor), 4.68 (d, *J* = 3.5 Hz, 1H, major), 4.53 (d, *J* = 3.6 Hz, 1H, major), 4.44 (d, *J* = 9.7 Hz, 1H, minor), 4.06 (d, *J* = 3.6 Hz, 1H, minor), 3.93 (d, *J* = 9.6 Hz, 1H, major), 3.81 – 3.63 (m, 2H, mixture), 3.59 – 3.52 (m, 1H, minor), 3.27 (s, 1H), 2.43 (s, 3H, major).

^{13}C NMR (101 MHz, CD_3OD) δ 151.9, 151.7, 145.3, 145.3, 137.2, 137.1, 130.7, 130.6, 128.8, 128.7, 102.2, 101.9, 77.1, 76.0, 75.3, 72.4, 71.9, 69.1, 62.3, 61.6, 55.6, 55.4, 21.5.

HRMS (m/z): (ESI) calc. for $C_{14}H_{21}N_2O_7S^+$ $[M+H]^+$: 361.1064 found 361.1049.

Synthesis of C3-Glycohydrazide 134

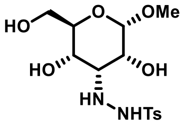

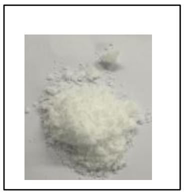

Following general procedure laid out in section **4.6.3 Synthesis of C3-Glycohydrazide (w/o PG)**, the title compound was prepared from C-3 sugar hydrazone **SL3** (3 g, 8.32 mmol). The product **134** was obtained as a colorless solid (1.61 g, 53% yield).

^{1}H NMR (600 MHz, CD_3OD) δ 7.79 (d, J = 8.3 Hz, 2H), 7.41 (d, J = 8.0 Hz, 2H), 4.55 (dd, J = 3.7, 0.8 Hz, 1H), 3.83 (dd, J = 11.7, 1.7 Hz, 1H), 3.71 – 3.61 (m, 2H), 3.49 – 3.40 (m, 2H), 3.36 (appear as t, J = 3.8 Hz, 1H), 3.28 (s, 3H), 2.45 (s, 3H).

^{13}C NMR (151 MHz, CD_3OD) δ 145.4, 136.9, 130.7, 129.1, 100.9, 70.3, 68.7, 67.8, 66.7, 62.6, 55.6, 21.5.

HRMS (m/z): (ESI) calc. for $C_{14}H_{22}N_2O_7SNa^+$ $[M+Na]^+$: 385.1045 found 385.1058.

Compound 136

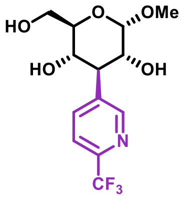

Following **General Procedure I** with C3-glycohydrazide **134** (0.10 mmol) and 5-Iodo-2-(trifluoromethyl)pyridine (0.15 mmol), purification through reverse-phase flash column chromatography afforded product **136** as a colorless solid (18.2 mg, 55% yield, g:a > 19:1).

^{1}H NMR (600 MHz, CD_3OD) δ 8.62 (d, *J* = 2.1 Hz, 1H), 8.02 (dd, *J* = 8.1, 2.1 Hz, 1H), 7.80 (d, *J* = 8.1 Hz, 1H), 4.74 (d, *J* = 3.5 Hz, 1H), 3.93 – 3.81 (m, 2H), 3.74 (dd, *J* = 11.7, 5.1 Hz, 1H), 3.70 – 3.62 (m, 2H), 3.51 (s, 3H), 3.17 (t, *J* = 10.55, 1H).

^{13}C NMR (151 MHz, CD_3OD) δ 152.4, 147.3, 147.1, 141.4, 139.1, 124.1, 122.3, 121.6, 121.5, 100.5, 74.5, 71.9, 69.9, 62.6, 55.6, 50.8.

HRMS (m/z): (ESI) calc. for $C_{13}H_{16}NO_5F_3Na^+$ $[M+Na]^+$: 346.0878 found 346.0871.

Compound 137

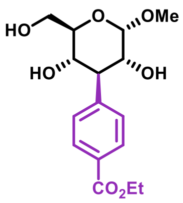

Following **General Procedure I** with C3-glycohydrazide **134** (0.10 mmol) and ethyl-4-iodo-benzoate (0.15 mmol), purification through reverse-phase flash column chromatography afforded product **137** as a colorless solid (20.4 mg, 62% yield, g:a > 19:1).

^{1}H NMR (600 MHz, CD_3OD) δ 7.98 (d, *J* = 8.3 Hz, 2H), 7.44 (d, *J* = 8.3 Hz, 2H), 4.72 (d, *J* = 3.5 Hz, 1H), 4.35 (q, *J* = 7.1 Hz, 2H), 3.82 (ddd, *J* = 19.2, 11.5, 2.8 Hz, 2H), 3.72 (dd, *J* = 11.7, 4.9 Hz, 1H), 3.69 – 3.63 (m, 2H), 3.50 (s, 3H), 3.13 – 3.06 (t, *J* =10.52, 1H), 1.38 (t, *J* = 7.1 Hz, 3H).

^{13}C NMR (151 MHz, CD_3OD) δ 166.8, 145.9, 129.0, 128.9, 128.6, 99.3, 73.0, 71.0, 69.0, 61.4, 60.6, 54.1, 51.9, 13.2.

HRMS (m/z): (ESI) calc. for $C_{16}H_{22}O_7Na^+$ $[M+Na]^+$: 349.1263 found 349.1271.

Compound 138

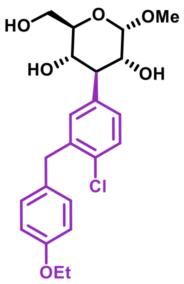

Following **General Procedure I** with C3-glycohydrazide **134** (0.10 mmol) and aryl iodide **28** (0.15 mmol), purification through reverse-phase flash column chromatography afforded product **138** as a colorless solid (28.9 mg, 68% yield, g:a > 19:1).

^{1}H NMR (400 MHz, CD_3OD) δ 7.33 (d, *J* = 8.1 Hz, 1H), 7.26 – 7.14 (m, 2H), 7.11 (d, *J* = 8.7 Hz, 2H), 6.79 (d, *J* = 8.6 Hz, 2H), 4.68 (d, *J* = 3.5 Hz, 1H), 4.02 (s, 2H), 3.98 (d, *J* = 7.0 Hz, 2H), 3.81 (dd, *J* = 11.7, 2.2 Hz, 1H), 3.70 (dt, *J* = 11.7, 4.3 Hz, 2H), 3.65 – 3.51 (m, 2H), 3.47 (s, 3H), 2.92 (t, *J* = 10.5, 1H), 1.36 (t, *J* = 7.0 Hz, 3H).

^{13}C NMR (151 MHz, CD_3OD) δ 158.8, 140.4, 140.0, 133.3, 133.2, 133.0, 130.9, 130.3, 129.1, 115.4, 100.7, 74.4, 72.4, 70.4, 64.4, 62.8, 55.5, 52.7, 39.3, 15.2.

HRMS (m/z): (ESI) calc. for $C_{22}H_{27}O_6ClNa^+$ $[M+Na]^+$: 445.1394 found 445.1396.

Compound SL4

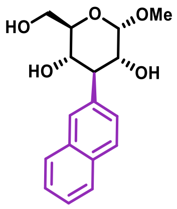

Following **General Procedure I** with C3-glycohydrazide **134** (0.10 mmol) and 2-Iodonaphthalene (0.15 mmol), purification through reverse-phase flash column chromatography afforded product **SL4** as a colorless solid (22.8 mg, 74% yield, g:a > 19:1).

^{1}H NMR (600 MHz, CD_3OD) δ 7.86 – 7.80 (m, 3H), 7.79 (s, 1H), 7.50 (dd, *J* = 8.5, 1.7 Hz, 1H), 7.43 (ddd, *J* = 9.3, 7.6, 1.4 Hz, 2H), 4.75 (d, *J* = 3.5 Hz, 1H), 3.91 (dd, *J* = 11.2, 3.5 Hz, 1H), 3.87 (dd, *J* = 11.6, 2.4 Hz, 1H), 3.81 – 3.73 (m, 2H), 3.70 (ddd, *J* = 9.7, 5.5, 2.3 Hz, 1H), 3.52 (s, 3H), 3.17 (t, *J* = 10.7 Hz, 1H).

^{13}C NMR (151 MHz, CD_3OD) δ 138.6, 135.1, 134.2, 129.4, 128.8, 128.7, 128.5, 127.7, 126.8, 126.4, 100.8, 74.5, 72.6, 70.5, 62.9, 55.5, 53.4.

HRMS (m/z): (ESI) calc. for $C_{17}H_{20}O_5Na^+$ $[M+Na]^+$: 327.1208 found 327.1217.

11. X-ray Crystallography

Crystal Structure of Ribose-hydrazide 78

The single crystal X-ray diffraction studies were carried out on a Bruker D8-Venture 3-circle diffractometer equipped with a Photon3 detector and Mo K_α radiation (λ = 0.7107 Å).

Crystals of the subject compound were used as received. A 0.12 x 0.02 x 0.02 mm piece of a crystal was mounted on a Cryoloop with Paratone oil. Data were collected in a nitrogen gas stream at 100(2) K using ω scans. Crystal-to-detector distance was 50 mm and exposure time was 10 or 60 seconds per frame using a scan width of 0.70°. Data collection was 100 % complete to 25.242° in θ. A total of 17061 reflections were collected covering the indices, −6<=h<=6, −10<=k<=10, −36<=l<=36. 2770 reflections were found to be symmetry independent, with a R_{int} of 0.0792. Indexing and unit cell refinement indicated a **P**rimitive, **Orthorhombic** lattice. The space group was found to be $\boldsymbol{P2_12_12_1}$. The data were integrated using the Bruker SAINT Software program and scaled using the SADABS software program. Solution by direct methods (SHELXT) produced a complete phasing model consistent with the proposed structure.

All nonhydrogen atoms were refined anisotropically by full-matrix least-squares (SHELXL-2014). All carbon bonded hydrogen atoms were placed using a riding model. Their positions were constrained relative to their parent atom using the

appropriate HFIX command in SHELXL-2014. Crystallographic data are summarized in Table 1.

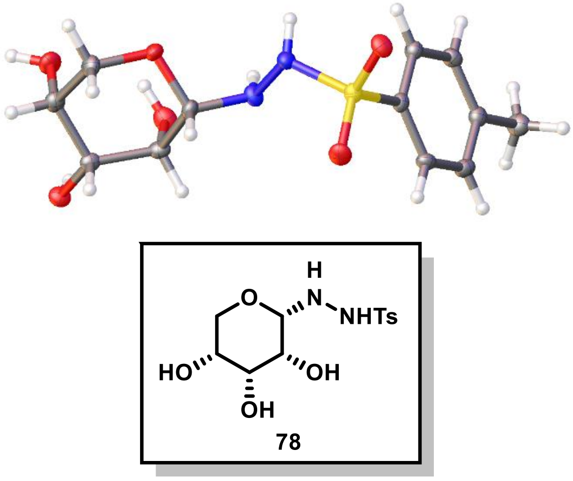

CCDC-2550313

Table 1. Crystal data and structure refinement for ribose-hydrazide 78.

Identification code	baran992_0m_a	
Empirical formula	C12 H18 N2 O6 S	
Formula weight	318.34	
Temperature	100 K	
Wavelength	0.71073 Å	
Crystal system	Orthorhombic	
Space group	$P2_12_12_1$	
Unit cell dimensions	a = 5.5104(6) Å	α= 90°.
	b = 8.0986(9) Å	β= 90°.
	c = 30.376(4) Å	γ = 90°.
Volume	1355.6(3) $Å^3$	
Z	4	
Density (calculated)	1.560 Mg/m^3	
Absorption coefficient	0.270 mm^{-1}	
F(000)	672	
Crystal size	0.12 x 0.02 x 0.02 mm^3	
Theta range for data collection	1.341 to 26.417°.	
Index ranges	-6<=h<=6, -10<=k<=10, -36<=l<=38	
Reflections collected	17061	
Independent reflections	2770 [R(int) = 0.0792]	
Completeness to theta = 25.242°	100.0 %	
Absorption correction	Semi-empirical from equivalents	
Max. and min. transmission	0.7454 and 0.6248	
Refinement method	Full-matrix least-squares on F^2	
Data / restraints / parameters	2770 / 2 / 200	
Goodness-of-fit on F^2	1.081	
Final R indices [I>2sigma(I)]	R1 = 0.0411, wR2 = 0.0804	
R indices (all data)	R1 = 0.0563, wR2 = 0.0871	
Absolute structure parameter	0.09(7)	
Largest diff. peak and hole	0.262 and -0.394 e.$Å^{-3}$	

Table 2. Atomic coordinates (x 10^4) and equivalent isotropic displacement parameters (Å^{2}x 10^3) for ribose-hydrazide 78. U(eq) is defined as one third of the trace of the orthogonalized U^{ij} tensor.

	x	y	z	U(eq)
S(1)	6707(2)	360(1)	3805(1)	17(1)
O(1)	2740(5)	3395(3)	2849(1)	19(1)
O(2)	-650(5)	5992(4)	2692(1)	24(1)
O(3)	1877(5)	8304(3)	3223(1)	20(1)
O(4)	453(5)	5237(4)	3576(1)	26(1)
O(5)	8636(4)	1528(3)	3855(1)	22(1)
O(6)	7272(5)	-1267(3)	3637(1)	21(1)
N(1)	3694(6)	2624(4)	3582(1)	18(1)
N(2)	4744(6)	1089(4)	3449(1)	18(1)
C(1)	3091(7)	4613(5)	2512(1)	21(1)
C(2)	1930(7)	6235(5)	2641(1)	20(1)
C(3)	2991(7)	6808(5)	3075(1)	17(1)
C(4)	2883(7)	5482(5)	3427(1)	19(1)
C(5)	3951(7)	3868(5)	3253(1)	17(1)
C(6)	5230(6)	156(5)	4316(1)	16(1)
C(7)	3111(7)	-764(4)	4342(1)	18(1)
C(8)	1969(7)	-941(5)	4748(1)	18(1)
C(9)	2927(7)	-226(5)	5127(1)	18(1)
C(10)	1662(8)	-398(5)	5563(1)	24(1)
C(11)	5084(7)	669(5)	5091(1)	20(1)
C(12)	6240(7)	871(5)	4689(1)	18(1)

Table 3. Bond lengths [Å] and angles [°] for ribose-hydrazide 78.

S(1)-O(5)	1.432(3)	C(10)-H(10C)	0.9800
S(1)-O(6)	1.446(3)	C(11)-H(11)	0.9500
S(1)-N(2)	1.639(3)	C(11)-C(12)	1.389(5)
S(1)-C(6)	1.762(4)	C(12)-H(12)	0.9500
O(1)-C(1)	1.434(4)		
O(1)-C(5)	1.446(4)	O(5)-S(1)-O(6)	118.71(17)
O(2)-H(2)	0.8400	O(5)-S(1)-N(2)	108.81(17)
O(2)-C(2)	1.444(5)	O(5)-S(1)-C(6)	108.04(17)
O(3)-H(3)	0.8400	O(6)-S(1)-N(2)	103.75(16)
O(3)-C(3)	1.430(4)	O(6)-S(1)-C(6)	108.96(17)
O(4)-H(4)	0.8400	N(2)-S(1)-C(6)	108.12(17)
O(4)-C(4)	1.427(4)	C(1)-O(1)-C(5)	111.1(3)
N(1)-H(1)	0.882(13)	C(2)-O(2)-H(2)	109.5
N(1)-N(2)	1.430(4)	C(3)-O(3)-H(3)	109.5
N(1)-C(5)	1.427(5)	C(4)-O(4)-H(4)	109.5
N(2)-H(2A)	0.880(13)	N(2)-N(1)-H(1)	113(3)
C(1)-H(1A)	0.9900	C(5)-N(1)-H(1)	109(3)
C(1)-H(1B)	0.9900	C(5)-N(1)-N(2)	112.0(3)
C(1)-C(2)	1.512(5)	S(1)-N(2)-H(2A)	113(3)
C(2)-H(2B)	1.0000	N(1)-N(2)-S(1)	113.2(2)
C(2)-C(3)	1.515(5)	N(1)-N(2)-H(2A)	115(3)
C(3)-H(3A)	1.0000	O(1)-C(1)-H(1A)	109.5
C(3)-C(4)	1.518(5)	O(1)-C(1)-H(1B)	109.5
C(4)-H(4A)	1.0000	O(1)-C(1)-C(2)	110.9(3)
C(4)-C(5)	1.528(5)	H(1A)-C(1)-H(1B)	108.1
C(5)-H(5)	1.0000	C(2)-C(1)-H(1A)	109.5
C(6)-C(7)	1.387(5)	C(2)-C(1)-H(1B)	109.5
C(6)-C(12)	1.387(5)	O(2)-C(2)-C(1)	109.1(3)
C(7)-H(7)	0.9500	O(2)-C(2)-H(2B)	109.8
C(7)-C(8)	1.391(5)	O(2)-C(2)-C(3)	109.2(3)
C(8)-H(8)	0.9500	C(1)-C(2)-H(2B)	109.8
C(8)-C(9)	1.393(5)	C(1)-C(2)-C(3)	109.1(3)
C(9)-C(10)	1.502(5)	C(3)-C(2)-H(2B)	109.8
C(9)-C(11)	1.397(5)	O(3)-C(3)-C(2)	111.5(3)
C(10)-H(10A)	0.9800	O(3)-C(3)-H(3A)	107.1
C(10)-H(10B)	0.9800	O(3)-C(3)-C(4)	111.2(3)

C(2)-C(3)-H(3A)	107.1
C(2)-C(3)-C(4)	112.4(3)
C(4)-C(3)-H(3A)	107.1
O(4)-C(4)-C(3)	111.0(3)
O(4)-C(4)-H(4A)	108.3
O(4)-C(4)-C(5)	110.7(3)
C(3)-C(4)-H(4A)	108.3
C(3)-C(4)-C(5)	110.2(3)
C(5)-C(4)-H(4A)	108.3
O(1)-C(5)-C(4)	110.0(3)
O(1)-C(5)-H(5)	108.9
N(1)-C(5)-O(1)	111.2(3)
N(1)-C(5)-C(4)	108.8(3)
N(1)-C(5)-H(5)	108.9
C(4)-C(5)-H(5)	108.9
C(7)-C(6)-S(1)	119.3(3)
C(12)-C(6)-S(1)	119.7(3)
C(12)-C(6)-C(7)	121.0(3)
C(6)-C(7)-H(7)	120.5
C(6)-C(7)-C(8)	119.1(3)
C(8)-C(7)-H(7)	120.5
C(7)-C(8)-H(8)	119.4
C(7)-C(8)-C(9)	121.2(3)
C(9)-C(8)-H(8)	119.4
C(8)-C(9)-C(10)	120.9(3)
C(8)-C(9)-C(11)	118.3(3)
C(11)-C(9)-C(10)	120.8(3)
C(9)-C(10)-H(10A)	109.5
C(9)-C(10)-H(10B)	109.5
C(9)-C(10)-H(10C)	109.5
H(10A)-C(10)-H(10B)	109.5
H(10A)-C(10)-H(10C)	109.5
H(10B)-C(10)-H(10C)	109.5
C(9)-C(11)-H(11)	119.3
C(12)-C(11)-C(9)	121.3(3)
C(12)-C(11)-H(11)	119.3
C(6)-C(12)-C(11)	119.0(3)
C(6)-C(12)-H(12)	120.5
C(11)-C(12)-H(12)	120.5

Symmetry transformations used to generate equivalent atoms:

Table 4. Anisotropic displacement parameters ($Å^2 x 10^3$) for ribose-hydrazide 78. The anisotropic displacement factor exponent takes the form: $-2\pi^2[h^2 a^{*2}U^{11} + ... + 2 h k a^* b^* U^{12}]$

	U^{11}	U^{22}	U^{33}	U^{23}	U^{13}	U^{12}
S(1)	14(1)	19(1)	19(1)	2(1)	2(1)	1(1)
O(1)	21(1)	19(1)	17(1)	-1(1)	-6(1)	-1(1)
O(2)	15(1)	27(2)	31(2)	2(1)	-6(1)	2(1)
O(3)	18(1)	18(1)	23(1)	-4(1)	3(1)	3(1)
O(4)	22(1)	25(2)	31(2)	2(1)	9(1)	2(1)
O(5)	12(1)	26(1)	28(2)	4(1)	3(1)	-5(1)
O(6)	21(1)	17(1)	25(1)	-1(1)	6(1)	5(1)
N(1)	17(2)	16(2)	21(2)	1(1)	5(1)	3(1)
N(2)	20(2)	15(2)	18(2)	-1(1)	-2(1)	1(1)
C(1)	24(2)	22(2)	16(2)	3(2)	1(2)	0(2)
C(2)	15(2)	22(2)	23(2)	2(2)	0(2)	0(2)
C(3)	12(2)	19(2)	19(2)	-3(2)	1(2)	2(2)
C(4)	17(2)	21(2)	18(2)	-5(2)	2(2)	-2(2)
C(5)	15(2)	20(2)	16(2)	3(2)	-3(1)	0(2)
C(6)	16(2)	16(2)	16(2)	1(2)	1(1)	4(2)
C(7)	18(2)	18(2)	16(2)	0(1)	0(2)	1(2)
C(8)	15(2)	17(2)	24(2)	0(2)	2(2)	-1(2)
C(9)	18(2)	18(2)	19(2)	0(2)	0(2)	3(2)
C(10)	25(2)	27(2)	20(2)	-2(2)	4(2)	-1(2)
C(11)	18(2)	20(2)	21(2)	-4(2)	-3(2)	2(2)
C(12)	15(2)	16(2)	23(2)	-2(2)	-2(2)	-2(2)

Table 5. Hydrogen coordinates (x 10^4) and isotropic displacement parameters (Å^{2}x 10^3) for ribose-hydrazide 78.

	x	y	z	U(eq)
H(2)	-1396	6586	2510	37
H(3)	543	8085	3343	29
H(4)	-439	5001	3361	39
H(1)	2150(30)	2540(50)	3652(12)	22
H(2A)	3700(60)	340(40)	3358(12)	21
H(1A)	2371	4216	2233	25
H(1B)	4851	4782	2464	25
H(2B)	2243	7081	2408	24
H(3A)	4744	7057	3022	20
H(4A)	3886	5853	3683	23
H(5)	5716	4035	3190	20
H(7)	2449	-1266	4086	21
H(8)	510	-1562	4767	22
H(10A)	-5	-780	5515	37
H(10B)	1633	674	5712	37
H(10C)	2533	-1201	5745	37
H(11)	5775	1149	5348	23
H(12)	7700	1490	4669	22

Crystal Structure of Galactose-hydrazide β-SH12

The single crystal X-ray diffraction studies were carried out on a Bruker D8-Venture 3-circle diffractometer equipped with a Photon3 detector and Mo K_α radiation (λ = 0.7107 Å).

Crystals of the subject compound were used as received. A 0.34 x 0.07 x 0.05 mm piece of a crystal was mounted on a Cryoloop with Paratone oil. Data were collected in a nitrogen gas stream at 100(2) K using ω scans. Crystal-to-detector distance was 50 mm and exposure time was 1 or 5 seconds per frame using a scan width of 0.70°. Data collection was 99.8 % complete to 25.242° in θ. A total of 23863 reflections were collected covering the indices, −8<=h<=8, −11<=k<=11, −33<=l<=33. 3146 reflections were found to be symmetry independent, with a R_{int} of 0.0700. Indexing and unit cell refinement indicated a **P**rimitive, **Orthorhombic** lattice. The space group was found to be $\boldsymbol{P2_12_12_1}$. The data were integrated using the Bruker SAINT Software program and scaled using the SADABS software program. Solution by direct methods (SHELXT) produced a complete phasing model consistent with the proposed structure.

All nonhydrogen atoms were refined anisotropically by full-matrix least-squares (SHELXL-2014). All carbon bonded hydrogen atoms were placed using a riding model. Their positions were constrained relative to their parent atom using the appropriate HFIX command in SHELXL-2014. Crystallographic data are summarized in Table 1.

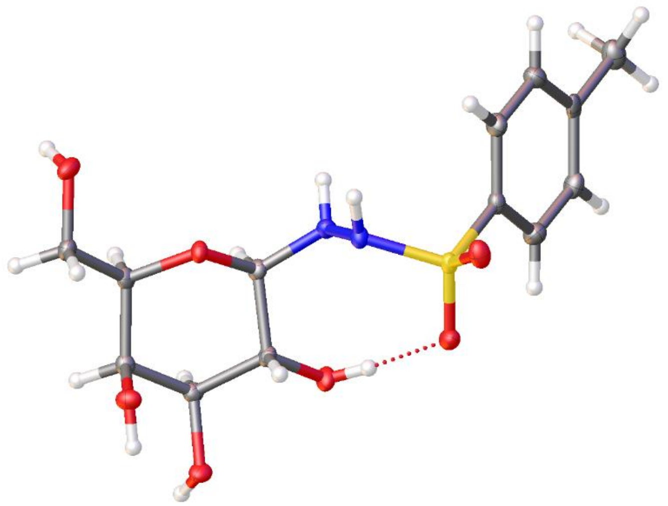

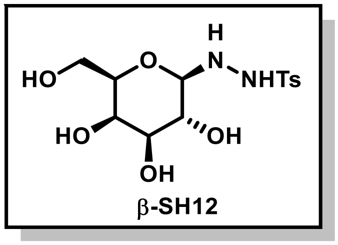

CCDC-2547217

Table 1. Crystal data and structure refinement for galactose-hydrazide β-SH12.

Identification code	baran990_0m_a	
Empirical formula	C13 H20 N2 O7 S	
Formula weight	348.37	
Temperature	100 K	
Wavelength	0.71073 Å	
Crystal system	Orthorhombic	
Space group	$P2_12_12_1$	
Unit cell dimensions	a = 6.4436(5) Å	α= 90°.
	b = 9.0354(7) Å	β= 90°.
	c = 26.548(2) Å	γ = 90°.
Volume	1545.6(2) Å^3	
Z	4	
Density (calculated)	1.497 Mg/m^3	
Absorption coefficient	0.249 mm^{-1}	
F(000)	736	
Crystal size	0.34 x 0.07 x 0.05 mm^3	
Theta range for data collection	2.381 to 26.363°.	
Index ranges	-8<=h<=8, -11<=k<=11, -33<=l<=33	
Reflections collected	23863	
Independent reflections	3146 [R(int) = 0.0700]	
Completeness to theta = 25.242°	99.8 %	
Absorption correction	Semi-empirical from equivalents	
Max. and min. transmission	0.6466 and 0.5698	
Refinement method	Full-matrix least-squares on F^2	
Data / restraints / parameters	3146 / 2 / 219	
Goodness-of-fit on F^2	1.088	
Final R indices [I>2sigma(I)]	R1 = 0.0307, wR2 = 0.0651	
R indices (all data)	R1 = 0.0363, wR2 = 0.0675	
Absolute structure parameter	0.03(5)	
Largest diff. peak and hole	0.187 and -0.292 e.Å^{-3}	

Table 2. Atomic coordinates (x 10^4) and equivalent isotropic displacement parameters (Å^{2}x 10^3) for galactose-hydrazide β-SH12. U(eq) is defined as one third of the trace of the orthogonalized U^{ij} tensor.

	x	y	z	U(eq)
S(1)	4583(1)	8936(1)	6080(1)	15(1)
O(5)	9435(3)	7857(2)	6782(1)	18(1)
O(6)	6789(3)	9233(2)	6047(1)	19(1)
O(1)	5202(3)	7987(2)	7702(1)	14(1)
O(7)	3176(3)	10095(2)	5937(1)	21(1)
O(4)	11327(3)	9161(2)	7620(1)	17(1)
O(3)	8093(3)	10092(2)	8184(1)	17(1)
O(2)	3004(3)	7481(2)	8596(1)	19(1)
N(1)	5064(3)	7269(2)	6839(1)	14(1)
N(2)	4074(3)	8593(2)	6676(1)	15(1)
C(1)	4942(4)	8238(3)	8593(1)	16(1)
C(3)	8422(4)	8530(3)	8153(1)	14(1)
C(5)	8251(4)	8344(3)	7207(1)	14(1)
C(8)	2142(4)	6685(3)	5741(1)	18(1)
C(4)	9624(4)	8175(3)	7670(1)	14(1)
C(6)	6321(4)	7396(3)	7279(1)	14(1)
C(12)	5224(4)	5487(3)	5137(1)	19(1)
C(2)	6333(4)	7748(3)	8162(1)	14(1)
C(9)	1748(4)	5430(3)	5455(1)	19(1)
C(10)	3288(4)	4811(3)	5151(1)	18(1)
C(11)	2857(5)	3432(3)	4851(1)	24(1)
C(7)	4095(4)	7326(3)	5723(1)	15(1)
C(13)	5659(4)	6730(3)	5425(1)	20(1)

Table 3. Bond lengths [Å] and angles [°] for galactose-hydrazide β-SH12.

S(1)-O(6)	1.4492(18)	C(12)-C(13)	1.387(4)
S(1)-O(7)	1.4365(19)	C(2)-H(2B)	1.0000
S(1)-N(2)	1.644(2)	C(9)-H(9)	0.9500
S(1)-C(7)	1.765(3)	C(9)-C(10)	1.396(4)
O(5)-H(5)	0.8400	C(10)-C(11)	1.504(4)
O(5)-C(5)	1.431(3)	C(11)-H(11A)	0.9800
O(1)-C(6)	1.438(3)	C(11)-H(11B)	0.9800
O(1)-C(2)	1.438(3)	C(11)-H(11C)	0.9800
O(4)-H(4)	0.8400	C(7)-C(13)	1.390(4)
O(4)-C(4)	1.420(3)	C(13)-H(13)	0.9500
O(3)-H(3)	0.8400		
O(3)-C(3)	1.430(3)	O(6)-S(1)-N(2)	106.78(11)
O(2)-H(2)	0.8400	O(6)-S(1)-C(7)	107.14(11)
O(2)-C(1)	1.424(3)	O(7)-S(1)-O(6)	117.92(12)
N(1)-H(1)	0.872(13)	O(7)-S(1)-N(2)	105.46(11)
N(1)-N(2)	1.423(3)	O(7)-S(1)-C(7)	110.26(11)
N(1)-C(6)	1.427(3)	N(2)-S(1)-C(7)	108.99(11)
N(2)-H(2A)	0.885(13)	C(5)-O(5)-H(5)	109.5
C(1)-H(1A)	0.9900	C(6)-O(1)-C(2)	110.69(17)
C(1)-H(1B)	0.9900	C(4)-O(4)-H(4)	109.5
C(1)-C(2)	1.519(3)	C(3)-O(3)-H(3)	109.5
C(3)-H(3A)	1.0000	C(1)-O(2)-H(2)	109.5
C(3)-C(4)	1.532(3)	N(2)-N(1)-H(1)	109.3(18)
C(3)-C(2)	1.521(3)	N(2)-N(1)-C(6)	115.82(19)
C(5)-H(5A)	1.0000	C(6)-N(1)-H(1)	113.7(19)
C(5)-C(4)	1.523(3)	S(1)-N(2)-H(2A)	111.1(19)
C(5)-C(6)	1.522(3)	N(1)-N(2)-S(1)	111.18(15)
C(8)-H(8)	0.9500	N(1)-N(2)-H(2A)	115.7(18)
C(8)-C(9)	1.388(4)	O(2)-C(1)-H(1A)	109.1
C(8)-C(7)	1.386(4)	O(2)-C(1)-H(1B)	109.1
C(4)-H(4A)	1.0000	O(2)-C(1)-C(2)	112.4(2)
C(6)-H(6)	1.0000	H(1A)-C(1)-H(1B)	107.8
C(12)-H(12)	0.9500	C(2)-C(1)-H(1A)	109.1
C(12)-C(10)	1.389(4)	C(2)-C(1)-H(1B)	109.1

O(3)-C(3)-H(3A)	109.0
O(3)-C(3)-C(4)	109.2(2)
O(3)-C(3)-C(2)	109.1(2)
C(4)-C(3)-H(3A)	109.0
C(2)-C(3)-H(3A)	109.0
C(2)-C(3)-C(4)	111.4(2)
O(5)-C(5)-H(5A)	110.0
O(5)-C(5)-C(4)	107.22(19)
O(5)-C(5)-C(6)	111.2(2)
C(4)-C(5)-H(5A)	110.0
C(6)-C(5)-H(5A)	110.0
C(6)-C(5)-C(4)	108.42(19)
C(9)-C(8)-H(8)	120.4
C(7)-C(8)-H(8)	120.4
C(7)-C(8)-C(9)	119.3(2)
O(4)-C(4)-C(3)	109.76(19)
O(4)-C(4)-C(5)	108.09(18)
O(4)-C(4)-H(4A)	109.3
C(3)-C(4)-H(4A)	109.3
C(5)-C(4)-C(3)	111.18(19)
C(5)-C(4)-H(4A)	109.3
O(1)-C(6)-C(5)	107.44(19)
O(1)-C(6)-H(6)	107.5
N(1)-C(6)-O(1)	112.66(19)
N(1)-C(6)-C(5)	113.9(2)
N(1)-C(6)-H(6)	107.5
C(5)-C(6)-H(6)	107.5
C(10)-C(12)-H(12)	119.2
C(13)-C(12)-H(12)	119.2
C(13)-C(12)-C(10)	121.5(3)
O(1)-C(2)-C(1)	107.24(19)
O(1)-C(2)-C(3)	111.40(19)
O(1)-C(2)-H(2B)	108.2
C(1)-C(2)-C(3)	113.5(2)
C(1)-C(2)-H(2B)	108.2
C(3)-C(2)-H(2B)	108.2
C(8)-C(9)-H(9)	119.6
C(8)-C(9)-C(10)	120.9(3)
C(10)-C(9)-H(9)	119.6
C(12)-C(10)-C(9)	118.5(2)
C(12)-C(10)-C(11)	121.1(2)
C(9)-C(10)-C(11)	120.4(2)
C(10)-C(11)-H(11A)	109.5
C(10)-C(11)-H(11B)	109.5
C(10)-C(11)-H(11C)	109.5
H(11A)-C(11)-H(11B)	109.5
H(11A)-C(11)-H(11C)	109.5
H(11B)-C(11)-H(11C)	109.5
C(8)-C(7)-S(1)	119.20(19)
C(8)-C(7)-C(13)	121.0(2)
C(13)-C(7)-S(1)	119.75(19)
C(12)-C(13)-C(7)	118.7(3)
C(12)-C(13)-H(13)	120.6
C(7)-C(13)-H(13)	120.6

Symmetry transformations used to generate equivalent atoms:

Table 4. Anisotropic displacement parameters (Å^{2}x 10^3) for galactose-hydrazide β-SH12. The anisotropic displacement factor exponent takes the form: $-2\pi^2$[h^2 $a^{*2}U^{11}$ + ... + 2 h k a* b* U^{12}]

	U^{11}	U^{22}	U^{33}	U^{23}	U^{13}	U^{12}
S(1)	19(1)	15(1)	10(1)	-1(1)	-1(1)	-2(1)
O(5)	16(1)	26(1)	12(1)	-1(1)	3(1)	2(1)
O(6)	20(1)	23(1)	15(1)	-1(1)	1(1)	-6(1)
O(1)	14(1)	18(1)	10(1)	1(1)	1(1)	2(1)
O(7)	28(1)	17(1)	17(1)	0(1)	-4(1)	3(1)
O(4)	10(1)	21(1)	19(1)	3(1)	-1(1)	0(1)
O(3)	16(1)	15(1)	22(1)	-3(1)	2(1)	-1(1)
O(2)	15(1)	23(1)	18(1)	7(1)	2(1)	0(1)
N(1)	16(1)	13(1)	14(1)	0(1)	0(1)	-2(1)
N(2)	16(1)	18(1)	11(1)	-1(1)	0(1)	2(1)
C(1)	18(1)	18(1)	12(1)	2(1)	1(1)	-1(1)
C(3)	16(1)	12(1)	13(1)	-1(1)	-1(1)	2(1)
C(5)	13(1)	17(1)	11(1)	0(1)	3(1)	2(1)
C(8)	18(1)	22(1)	15(1)	-3(1)	2(1)	1(1)
C(4)	12(1)	15(1)	16(1)	1(1)	-1(1)	1(1)
C(6)	14(1)	16(1)	12(1)	-2(1)	2(1)	2(1)
C(12)	24(1)	18(1)	14(1)	0(1)	4(1)	4(1)
C(2)	16(1)	14(1)	12(1)	2(1)	-2(1)	3(1)
C(9)	20(1)	20(1)	19(1)	-1(1)	-2(1)	-4(1)
C(10)	28(2)	16(1)	9(1)	3(1)	-3(1)	0(1)
C(11)	32(2)	20(1)	21(1)	-1(1)	-2(1)	2(1)
C(7)	19(1)	16(1)	11(1)	0(1)	-1(1)	1(1)
C(13)	20(1)	21(1)	18(1)	1(1)	2(1)	-3(1)

Table 5. Hydrogen coordinates (x 10^4) and isotropic displacement parameters (Å^{2}x 10^3) for galactose-hydrazide β-SH12.

	x	y	z	U(eq)
H(5)	8824	8104	6515	27
H(4)	12419	8737	7715	25
H(3)	9230	10534	8146	26
H(2)	3170	6624	8711	28
H(1)	4150(30)	6560(20)	6855(10)	17
H(2A)	2730(20)	8650(30)	6737(10)	18
H(1A)	5664	8056	8917	20
H(1B)	4688	9315	8565	20
H(3A)	9258	8202	8450	17
H(5A)	7847	9405	7163	17
H(8)	1085	7100	5947	22
H(4A)	10154	7136	7688	17
H(6)	6793	6378	7372	17
H(12)	6274	5088	4926	23
H(2B)	6588	6661	8198	17
H(9)	412	4987	5466	23
H(11A)	3907	3325	4587	36
H(11B)	1479	3505	4697	36
H(11C)	2903	2569	5075	36
H(13)	7000	7166	5418	24

Crystal Structure of Xylose-hydrazide β-SH21

The single crystal X-ray diffraction studies were carried out on a Bruker D8-Venture 3-circle diffractometer equipped with a Photon3 detector and Mo K_α radiation (λ = 0.7107 Å).

Crystals of the subject compound were used as received. A 0.34 x 0.1 x 0.04 mm piece of a crystal was mounted on a Cryoloop with Paratone oil. Data were collected in a nitrogen gas stream at 100(2) K using ω scans. Crystal-to-detector distance was 50 mm and exposure time was 1 or 5 seconds per frame using a scan width of 0.70°. Data collection was 99.8 % complete to 25.242° in θ. A total of 20599 reflections were collected covering the indices, −13<=h<=13, −6<=k<=6, −31<=l<=31. 2948 reflections were found to be symmetry independent, with a R_{int} of 0.0942. Indexing and unit cell refinement indicated a **C-**centered, **Monoclinic** lattice. The space group was found to be ***C*2**. The data were integrated using the Bruker SAINT Software program and scaled using the SADABS software program. Solution by direct methods (SHELXT) produced a complete phasing model consistent with the proposed structure.

All nonhydrogen atoms were refined anisotropically by full-matrix least-squares (SHELXL-2014). All carbon bonded hydrogen atoms were placed using a riding model. Their positions were constrained relative to their parent atom using the appropriate HFIX command in SHELXL-2014. Crystallographic data are summarized in Table 1.

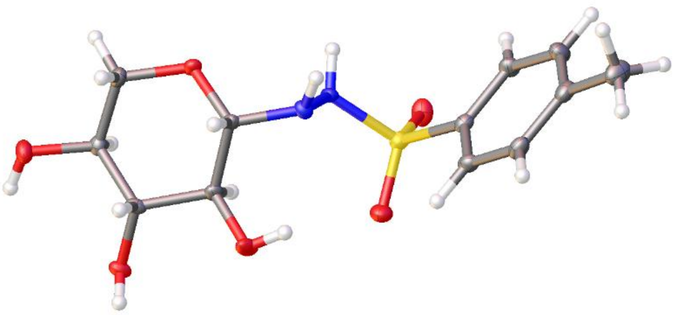

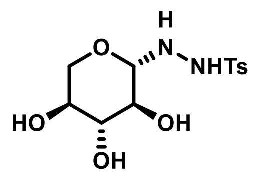

β-SH21

CCDC-2547216

Table 1. Crystal data and structure refinement for xylose-hydrazide β-SH21.

Identification code	baran989b_0m_a	
Empirical formula	C12 H18 N2 O6 S	
Formula weight	318.34	
Temperature	100 K	
Wavelength	0.71073 Å	
Crystal system	Monoclinic	
Space group	C2	
Unit cell dimensions	a = 11.016(3) Å	α= 90°.
	b = 5.1835(16) Å	β= 99.485(11)°.
	c = 25.404(8) Å	γ = 90°.
Volume	1430.8(7) $Å^3$	
Z	4	
Density (calculated)	1.478 Mg/m^3	
Absorption coefficient	0.256 mm^{-1}	
F(000)	672	
Crystal size	0.34 x 0.1 x 0.04 mm^3	
Theta range for data collection	2.439 to 26.407°.	
Index ranges	-13<=h<=13, -6<=k<=6, -31<=l<=31	
Reflections collected	20599	
Independent reflections	2948 [R(int) = 0.0942]	
Completeness to theta = 25.242°	99.8 %	
Absorption correction	Semi-empirical from equivalents	
Max. and min. transmission	0.7454 and 0.4482	
Refinement method	Full-matrix least-squares on F^2	
Data / restraints / parameters	2948 / 3 / 200	
Goodness-of-fit on F^2	1.035	
Final R indices [I>2sigma(I)]	R1 = 0.0535, wR2 = 0.1318	
R indices (all data)	R1 = 0.0583, wR2 = 0.1350	
Absolute structure parameter	-0.12(15)	
Largest diff. peak and hole	0.301 and -0.357 e.$Å^{-3}$	

Table 2. Atomic coordinates (x 10^4) and equivalent isotropic displacement parameters (Å^{2}x 10^3) for xylose-hydrazide β-SH21. U(eq) is defined as one third of the trace of the orthogonalized U^{ij} tensor.

	x	y	z	U(eq)
S(1)	4424(1)	7648(2)	2172(1)	15(1)
O(1)	4244(2)	4460(6)	3732(1)	15(1)
O(3)	1749(3)	10254(6)	3927(1)	16(1)
O(2)	2971(3)	7296(6)	4833(1)	19(1)
O(6)	5594(3)	8831(6)	2156(1)	21(1)
O(4)	1567(3)	7600(8)	2942(1)	23(1)
O(5)	3350(3)	9213(6)	2158(1)	21(1)
N(1)	3595(3)	4445(7)	2795(1)	14(1)
N(2)	4617(3)	6023(7)	2732(1)	13(1)
C(2)	3331(3)	7326(8)	4320(2)	14(1)
C(1)	3901(4)	4707(9)	4249(2)	16(1)
C(7)	2930(4)	4654(9)	1446(2)	17(1)
C(3)	2233(3)	7738(9)	3879(2)	14(1)
C(9)	3695(4)	1795(9)	821(2)	19(1)
C(4)	2632(3)	7400(9)	3341(2)	14(1)
C(5)	3219(3)	4756(8)	3302(2)	14(1)
C(8)	2721(4)	2870(10)	1036(2)	19(1)
C(12)	5119(4)	4395(9)	1435(2)	17(1)
C(11)	4893(4)	2608(11)	1024(2)	21(1)
C(10)	3469(5)	-257(10)	391(2)	27(1)
C(6)	4128(4)	5422(8)	1642(2)	14(1)

Table 3. Bond lengths [Å] and angles [°] for xylose-hydrazide β-SH21.

S(1)-O(6)	1.434(3)	C(12)-C(11)	1.386(6)
S(1)-O(5)	1.431(3)	C(12)-C(6)	1.393(6)
S(1)-N(2)	1.637(4)	C(11)-H(11)	0.9500
S(1)-C(6)	1.763(4)	C(10)-H(10A)	0.9800
O(1)-C(1)	1.430(5)	C(10)-H(10B)	0.9800
O(1)-C(5)	1.444(5)	C(10)-H(10C)	0.9800
O(3)-H(3)	0.8400		
O(3)-C(3)	1.422(5)	O(6)-S(1)-N(2)	104.89(18)
O(2)-H(2)	0.8400	O(6)-S(1)-C(6)	108.17(19)
O(2)-C(2)	1.422(4)	O(5)-S(1)-O(6)	120.0(2)
O(4)-H(4)	0.8400	O(5)-S(1)-N(2)	107.54(18)
O(4)-C(4)	1.423(4)	O(5)-S(1)-C(6)	107.70(19)
N(1)-H(1)	0.878(14)	N(2)-S(1)-C(6)	107.98(19)
N(1)-N(2)	1.422(5)	C(1)-O(1)-C(5)	113.0(3)
N(1)-C(5)	1.426(5)	C(3)-O(3)-H(3)	109.5
N(2)-H(2A)	0.875(14)	C(2)-O(2)-H(2)	109.5
C(2)-H(2B)	1.0000	C(4)-O(4)-H(4)	109.5
C(2)-C(1)	1.519(6)	N(2)-N(1)-H(1)	113(3)
C(2)-C(3)	1.523(5)	N(2)-N(1)-C(5)	113.0(3)
C(1)-H(1A)	0.9900	C(5)-N(1)-H(1)	112(3)
C(1)-H(1B)	0.9900	S(1)-N(2)-H(2A)	116(3)
C(7)-H(7)	0.9500	N(1)-N(2)-S(1)	113.7(3)
C(7)-C(8)	1.383(6)	N(1)-N(2)-H(2A)	110(3)
C(7)-C(6)	1.390(6)	O(2)-C(2)-H(2B)	109.8
C(3)-H(3A)	1.0000	O(2)-C(2)-C(1)	106.4(3)
C(3)-C(4)	1.513(5)	O(2)-C(2)-C(3)	111.5(3)
C(9)-C(8)	1.398(6)	C(1)-C(2)-H(2B)	109.8
C(9)-C(11)	1.401(6)	C(1)-C(2)-C(3)	109.4(3)
C(9)-C(10)	1.515(6)	C(3)-C(2)-H(2B)	109.8
C(4)-H(4A)	1.0000	O(1)-C(1)-C(2)	111.5(3)
C(4)-C(5)	1.526(6)	O(1)-C(1)-H(1A)	109.3
C(5)-H(5)	1.0000	O(1)-C(1)-H(1B)	109.3
C(8)-H(8)	0.9500	C(2)-C(1)-H(1A)	109.3
C(12)-H(12)	0.9500	C(2)-C(1)-H(1B)	109.3

H(1A)-C(1)-H(1B)	108.0	C(9)-C(10)-H(10C)	109.5
C(8)-C(7)-H(7)	120.3	H(10A)-C(10)-H(10B)	109.5
C(8)-C(7)-C(6)	119.4(4)	H(10A)-C(10)-H(10C)	109.5
C(6)-C(7)-H(7)	120.3	H(10B)-C(10)-H(10C)	109.5
O(3)-C(3)-C(2)	109.2(3)	C(7)-C(6)-S(1)	120.5(3)
O(3)-C(3)-H(3A)	109.2	C(7)-C(6)-C(12)	120.8(4)
O(3)-C(3)-C(4)	110.6(4)	C(12)-C(6)-S(1)	118.7(3)
C(2)-C(3)-H(3A)	109.2		
C(4)-C(3)-C(2)	109.5(3)		
C(4)-C(3)-H(3A)	109.2		
C(8)-C(9)-C(11)	118.2(4)		
C(8)-C(9)-C(10)	121.1(4)		
C(11)-C(9)-C(10)	120.7(4)		
O(4)-C(4)-C(3)	107.9(3)		
O(4)-C(4)-H(4A)	109.7		
O(4)-C(4)-C(5)	109.2(3)		
C(3)-C(4)-H(4A)	109.7		
C(3)-C(4)-C(5)	110.6(4)		
C(5)-C(4)-H(4A)	109.7		
O(1)-C(5)-C(4)	109.5(3)		
O(1)-C(5)-H(5)	108.5		
N(1)-C(5)-O(1)	111.3(3)		
N(1)-C(5)-C(4)	110.4(3)		
N(1)-C(5)-H(5)	108.5		
C(4)-C(5)-H(5)	108.5		
C(7)-C(8)-C(9)	121.2(4)		
C(7)-C(8)-H(8)	119.4		
C(9)-C(8)-H(8)	119.4		
C(11)-C(12)-H(12)	120.5		
C(11)-C(12)-C(6)	119.0(4)		
C(6)-C(12)-H(12)	120.5		
C(9)-C(11)-H(11)	119.3		
C(12)-C(11)-C(9)	121.3(4)		
C(12)-C(11)-H(11)	119.3		
C(9)-C(10)-H(10A)	109.5		
C(9)-C(10)-H(10B)	109.5		

Symmetry transformations used to generate equivalent atoms:

Table 4. Anisotropic displacement parameters ($Å^2 x 10^3$) for xylose-hydrazide β-SH21. The anisotropic displacement factor exponent takes the form: $-2\pi^2[h^2 a^{*2}U^{11} + ... + 2 h k a^* b^* U^{12}]$

	U^{11}	U^{22}	U^{33}	U^{23}	U^{13}	U^{12}
S(1)	15(1)	14(1)	14(1)	0(1)	1(1)	0(1)
O(1)	9(1)	20(2)	14(1)	2(1)	0(1)	2(1)
O(3)	12(1)	17(2)	20(2)	-4(1)	2(1)	3(1)
O(2)	25(2)	20(2)	14(1)	-1(1)	7(1)	1(1)
O(6)	24(2)	21(2)	19(2)	-2(1)	5(1)	-9(1)
O(4)	17(1)	33(2)	16(1)	-6(2)	-5(1)	12(2)
O(5)	22(2)	19(2)	21(2)	0(1)	3(1)	7(1)
N(1)	13(2)	13(2)	15(2)	-3(2)	3(1)	-1(1)
N(2)	10(2)	16(2)	14(2)	1(1)	3(1)	4(1)
C(2)	13(2)	16(2)	13(2)	0(2)	3(1)	-2(2)
C(1)	17(2)	20(2)	12(2)	2(2)	3(2)	1(2)
C(7)	13(2)	19(2)	18(2)	3(2)	2(2)	1(2)
C(3)	10(2)	13(2)	19(2)	-2(2)	2(1)	1(2)
C(9)	25(2)	18(2)	14(2)	3(2)	0(2)	1(2)
C(4)	9(2)	19(2)	14(2)	0(2)	-1(1)	2(2)
C(5)	8(2)	17(2)	15(2)	3(2)	1(1)	1(2)
C(8)	16(2)	24(2)	16(2)	4(2)	-1(1)	-3(2)
C(12)	11(2)	22(2)	18(2)	-1(2)	1(2)	0(2)
C(11)	19(2)	25(2)	18(2)	-4(2)	3(2)	6(2)
C(10)	34(3)	22(2)	22(2)	-4(2)	-4(2)	1(2)
C(6)	15(2)	14(2)	14(2)	2(2)	0(2)	-1(2)

Table 5. Hydrogen coordinates (x 10^4) and isotropic displacement parameters ($Å^2$x 10^3) for xylose-hydrazide β-SH21.

	x	y	z	U(eq)
H(3)	983	10230	3829	25
H(2)	2688	8748	4896	29
H(4)	1679	6774	2670	35
H(1)	3700(40)	2820(40)	2717(17)	16
H(2A)	5300(30)	5130(90)	2807(18)	16
H(2B)	3955	8715	4305	17
H(1A)	4638	4473	4526	20
H(1B)	3303	3335	4297	20
H(7)	2260	5349	1592	20
H(3A)	1584	6433	3917	17
H(4A)	3233	8782	3288	17
H(5)	2593	3402	3341	16
H(8)	1901	2366	898	23
H(12)	5937	4910	1573	20
H(11)	5564	1922	878	25
H(10A)	3724	-1942	546	40
H(10B)	3945	149	108	40
H(10C)	2591	-306	241	40

Crystal Structure of Mannose-arylated product α-47

The single crystal X-ray diffraction studies were carried out on a Bruker D8-Venture 3-circle diffractometer equipped with a Photon3 detector and Mo K_{α} radiation (λ = 0.7107 Å).

Crystals of the subject compound were used as received. A 0.2 x 0.05 x 0.05 mm piece of a crystal was mounted on a Cryoloop with Paratone oil. Data were collected in a nitrogen gas stream at 100(2) K using ω scans. Crystal-to-detector distance was 50 mm and exposure time was 1 or 5 seconds per frame using a scan width of 0.70°. Data collection was 99.9 % complete to 25.242° in θ. A total of 17750 reflections were collected covering the indices, −12<=h<=12, −7<=k<=7, −21<=l<=21. 4053 reflections were found to be symmetry independent, with a R_{int} of 0.0770. Indexing and unit cell refinement indicated a **P**rimitive, **Monoclinic** lattice. The space group was found to be $\boldsymbol{P2_1}$. The data were integrated using the Bruker SAINT Software program and scaled using the SADABS software program. Solution by direct methods (SHELXT) produced a complete phasing model consistent with the proposed structure.

All nonhydrogen atoms were refined anisotropically by full-matrix least-squares (SHELXL-2014). All carbon bonded hydrogen atoms were placed using a riding model. Their positions were constrained relative to their parent atom using the appropriate HFIX command in SHELXL-2014. Crystallographic data are summarized in Table 1.

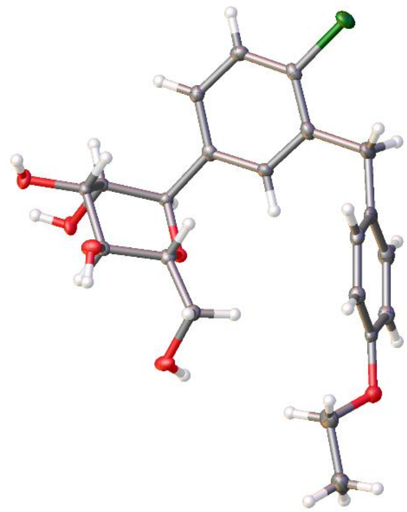

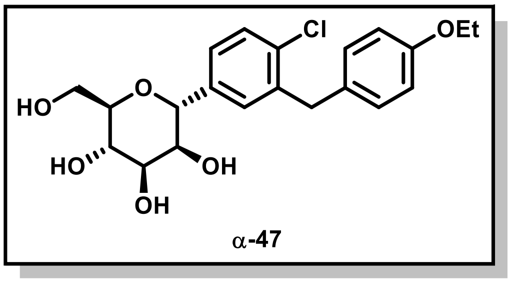

CCDC-2548674

Table 1. Crystal data and structure refinement for α-47.

Identification code	baran991_0m_a	
Empirical formula	C21 H25 Cl O6	
Formula weight	408.86	
Temperature	100 K	
Wavelength	0.71073 Å	
Crystal system	Monoclinic	
Space group	P 1 21 1	
Unit cell dimensions	a = 9.6547(17) Å	α= 90°.
	b = 6.0828(8) Å	β= 101.667(7)°.
	c = 17.164(2) Å	γ = 90°.
Volume	987.2(3) $Å^3$	
Z	2	
Density (calculated)	1.375 Mg/m^3	
Absorption coefficient	0.229 mm^{-1}	
F(000)	432	
Crystal size	0.2 x 0.05 x 0.05 mm^3	
Theta range for data collection	2.154 to 26.437°.	
Index ranges	-12<=h<=12, -7<=k<=7, -21<=l<=21	
Reflections collected	17750	
Independent reflections	4053 [R(int) = 0.0770]	
Completeness to theta = 25.242°	99.9 %	
Absorption correction	Semi-empirical from equivalents	
Max. and min. transmission	0.7454 and 0.5857	
Refinement method	Full-matrix least-squares on F^2	
Data / restraints / parameters	4053 / 1 / 258	
Goodness-of-fit on F^2	1.059	
Final R indices [I>2sigma(I)]	R1 = 0.0383, wR2 = 0.0775	
R indices (all data)	R1 = 0.0462, wR2 = 0.0812	
Absolute structure parameter	0.05(4)	
Largest diff. peak and hole	0.350 and -0.315 e.$Å^{-3}$	

Table 2. Atomic coordinates (x 10^4) and equivalent isotropic displacement parameters ($Å^2$x 10^3) for α-47. U(eq) is defined as one third of the trace of the orthogonalized U^{ij} tensor.

	x	y	z	U(eq)
Cl(1)	1954(1)	2590(1)	4390(1)	21(1)
O(3)	5219(2)	317(3)	9052(1)	17(1)
O(4)	7684(2)	-968(3)	8445(1)	17(1)
O(1)	5921(2)	5231(3)	7942(1)	12(1)
O(2)	5290(2)	6063(3)	9480(1)	19(1)
O(5)	8647(2)	3273(3)	8188(1)	16(1)
O(6)	1074(2)	10560(3)	8474(1)	19(1)
C(1)	4336(3)	5018(5)	8838(2)	16(1)
C(19)	1057(3)	10966(6)	9846(2)	30(1)
C(18)	1281(4)	9388(6)	9215(2)	28(1)
C(7)	5357(3)	3705(5)	6589(2)	14(1)
C(13)	1648(3)	5938(6)	5676(2)	24(1)
C(16)	1804(3)	10397(5)	7249(2)	18(1)
C(3)	6048(3)	1866(5)	8720(2)	13(1)
C(5)	7466(3)	2307(5)	7660(2)	13(1)
C(12)	4105(3)	4944(5)	6459(2)	15(1)
C(10)	3251(3)	3054(5)	5247(2)	16(1)
C(9)	4468(3)	1807(5)	5356(2)	17(1)
C(15)	1932(3)	9300(6)	6554(2)	21(1)
C(11)	3020(3)	4629(5)	5793(2)	17(1)
C(21)	782(3)	6172(5)	6967(2)	18(1)
C(2)	5098(3)	3708(5)	8307(2)	13(1)
C(20)	643(3)	7232(5)	7664(2)	18(1)
C(17)	1176(3)	9351(5)	7805(2)	15(1)
C(14)	1440(3)	7168(5)	6412(2)	19(1)
C(8)	5521(3)	2129(5)	6035(2)	16(1)
C(4)	6693(3)	668(5)	8099(2)	13(1)
C(6)	6511(3)	4232(5)	7321(2)	13(1)

Table 3. Bond lengths [Å] and angles [°] for α-47.

Cl(1)-C(10)	1.752(3)	C(3)-C(4)	1.523(4)
O(3)-H(3)	0.8400	C(5)-H(5A)	1.0000
O(3)-C(3)	1.429(3)	C(5)-C(4)	1.532(4)
O(4)-H(4)	0.8400	C(5)-C(6)	1.530(4)
O(4)-C(4)	1.424(3)	C(12)-H(12)	0.9500
O(1)-C(2)	1.443(3)	C(12)-C(11)	1.399(4)
O(1)-C(6)	1.441(3)	C(10)-C(9)	1.380(4)
O(2)-H(2)	0.8400	C(10)-C(11)	1.388(4)
O(2)-C(1)	1.434(3)	C(9)-H(9)	0.9500
O(5)-H(5)	0.8400	C(9)-C(8)	1.397(4)
O(5)-C(5)	1.431(3)	C(15)-H(15)	0.9500
O(6)-C(18)	1.438(4)	C(15)-C(14)	1.385(5)
O(6)-C(17)	1.383(3)	C(21)-H(21)	0.9500
C(1)-H(1A)	0.9900	C(21)-C(20)	1.389(4)
C(1)-H(1B)	0.9900	C(21)-C(14)	1.387(4)
C(1)-C(2)	1.510(4)	C(2)-H(2A)	1.0000
C(19)-H(19A)	0.9800	C(20)-H(20)	0.9500
C(19)-H(19B)	0.9800	C(20)-C(17)	1.391(4)
C(19)-H(19C)	0.9800	C(8)-H(8)	0.9500
C(19)-C(18)	1.494(4)	C(4)-H(4A)	1.0000
C(18)-H(18A)	0.9900	C(6)-H(6)	1.0000
C(18)-H(18B)	0.9900		
C(7)-C(12)	1.403(4)	C(3)-O(3)-H(3)	109.5
C(7)-C(8)	1.382(4)	C(4)-O(4)-H(4)	109.5
C(7)-C(6)	1.535(4)	C(6)-O(1)-C(2)	112.7(2)
C(13)-H(13A)	0.9900	C(1)-O(2)-H(2)	109.5
C(13)-H(13B)	0.9900	C(5)-O(5)-H(5)	109.5
C(13)-C(11)	1.523(4)	C(17)-O(6)-C(18)	116.8(2)
C(13)-C(14)	1.517(4)	O(2)-C(1)-H(1A)	109.1
C(16)-H(16)	0.9500	O(2)-C(1)-H(1B)	109.1
C(16)-C(15)	1.394(4)	O(2)-C(1)-C(2)	112.5(2)
C(16)-C(17)	1.385(4)	H(1A)-C(1)-H(1B)	107.8
C(3)-H(3A)	1.0000	C(2)-C(1)-H(1A)	109.1
C(3)-C(2)	1.528(4)	C(2)-C(1)-H(1B)	109.1

H(19A)-C(19)-H(19B)	109.5	C(7)-C(12)-H(12)	118.8
H(19A)-C(19)-H(19C)	109.5	C(11)-C(12)-C(7)	122.4(3)
H(19B)-C(19)-H(19C)	109.5	C(11)-C(12)-H(12)	118.8
C(18)-C(19)-H(19A)	109.5	C(9)-C(10)-Cl(1)	118.0(2)
C(18)-C(19)-H(19B)	109.5	C(9)-C(10)-C(11)	122.5(3)
C(18)-C(19)-H(19C)	109.5	C(11)-C(10)-Cl(1)	119.5(2)
O(6)-C(18)-C(19)	107.9(3)	C(10)-C(9)-H(9)	120.3
O(6)-C(18)-H(18A)	110.1	C(10)-C(9)-C(8)	119.4(3)
O(6)-C(18)-H(18B)	110.1	C(8)-C(9)-H(9)	120.3
C(19)-C(18)-H(18A)	110.1	C(16)-C(15)-H(15)	119.6
C(19)-C(18)-H(18B)	110.1	C(14)-C(15)-C(16)	120.8(3)
H(18A)-C(18)-H(18B)	108.4	C(14)-C(15)-H(15)	119.6
C(12)-C(7)-C(6)	118.6(2)	C(12)-C(11)-C(13)	121.6(3)
C(8)-C(7)-C(12)	118.5(3)	C(10)-C(11)-C(13)	121.7(3)
C(8)-C(7)-C(6)	122.9(2)	C(10)-C(11)-C(12)	116.7(3)
H(13A)-C(13)-H(13B)	107.7	C(20)-C(21)-H(21)	119.1
C(11)-C(13)-H(13A)	108.8	C(14)-C(21)-H(21)	119.1
C(11)-C(13)-H(13B)	108.8	C(14)-C(21)-C(20)	121.8(3)
C(14)-C(13)-H(13A)	108.8	O(1)-C(2)-C(1)	107.5(2)
C(14)-C(13)-H(13B)	108.8	O(1)-C(2)-C(3)	109.9(2)
C(14)-C(13)-C(11)	113.9(2)	O(1)-C(2)-H(2A)	108.0
C(15)-C(16)-H(16)	120.1	C(1)-C(2)-C(3)	115.3(2)
C(17)-C(16)-H(16)	120.1	C(1)-C(2)-H(2A)	108.0
C(17)-C(16)-C(15)	119.8(3)	C(3)-C(2)-H(2A)	108.0
O(3)-C(3)-H(3A)	110.3	C(21)-C(20)-H(20)	120.6
O(3)-C(3)-C(2)	109.7(2)	C(21)-C(20)-C(17)	118.9(3)
O(3)-C(3)-C(4)	107.8(2)	C(17)-C(20)-H(20)	120.6
C(2)-C(3)-H(3A)	110.3	O(6)-C(17)-C(16)	116.3(3)
C(4)-C(3)-H(3A)	110.3	O(6)-C(17)-C(20)	123.4(3)
C(4)-C(3)-C(2)	108.5(2)	C(16)-C(17)-C(20)	120.3(3)
O(5)-C(5)-H(5A)	109.5	C(15)-C(14)-C(13)	120.7(3)
O(5)-C(5)-C(4)	111.1(2)	C(15)-C(14)-C(21)	118.4(3)
O(5)-C(5)-C(6)	105.6(2)	C(21)-C(14)-C(13)	120.9(3)
C(4)-C(5)-H(5A)	109.5	C(7)-C(8)-C(9)	120.4(3)
C(6)-C(5)-H(5A)	109.5	C(7)-C(8)-H(8)	119.8
C(6)-C(5)-C(4)	111.6(2)	C(9)-C(8)-H(8)	119.8

O(4)-C(4)-C(3)	112.2(2)
O(4)-C(4)-C(5)	107.8(2)
O(4)-C(4)-H(4A)	108.9
C(3)-C(4)-C(5)	110.0(2)
C(3)-C(4)-H(4A)	108.9
C(5)-C(4)-H(4A)	108.9
O(1)-C(6)-C(7)	111.3(2)
O(1)-C(6)-C(5)	109.8(2)
O(1)-C(6)-H(6)	106.5
C(7)-C(6)-H(6)	106.5
C(5)-C(6)-C(7)	115.7(2)
C(5)-C(6)-H(6)	106.5

Symmetry transformations used to generate equivalent atoms:

Table 4. Anisotropic displacement parameters (Å^{2}x 10^3) for α-47. The anisotropic displacement factor exponent takes the form: -2π^2[h^2 a*^{2}U^{11} + ... + 2 h k a* b* U^{12}]

	U^{11}	U^{22}	U^{33}	U^{23}	U^{13}	U^{12}
Cl(1)	15(1)	29(1)	16(1)	-4(1)	-2(1)	-3(1)
O(3)	23(1)	14(1)	16(1)	0(1)	9(1)	-4(1)
O(4)	16(1)	12(1)	23(1)	3(1)	1(1)	2(1)
O(1)	13(1)	13(1)	12(1)	0(1)	3(1)	-1(1)
O(2)	29(1)	12(1)	16(1)	-1(1)	7(1)	-2(1)
O(5)	9(1)	16(1)	21(1)	0(1)	-3(1)	0(1)
O(6)	18(1)	20(1)	18(1)	-1(1)	4(1)	3(1)
C(1)	17(1)	16(2)	16(1)	0(1)	5(1)	1(1)
C(19)	30(2)	38(2)	20(2)	-1(2)	1(1)	9(2)
C(18)	35(2)	29(2)	18(1)	3(2)	4(1)	7(2)
C(7)	13(1)	16(2)	12(1)	3(1)	4(1)	-2(1)
C(13)	18(1)	32(2)	18(1)	-5(2)	-4(1)	7(1)
C(16)	13(1)	17(2)	23(1)	2(1)	1(1)	-2(1)
C(3)	15(1)	12(2)	12(1)	1(1)	1(1)	-2(1)
C(5)	11(1)	13(2)	13(1)	-2(1)	1(1)	0(1)
C(12)	16(1)	17(2)	11(1)	-1(1)	3(1)	1(1)
C(10)	14(1)	20(2)	14(1)	2(1)	0(1)	-3(1)
C(9)	20(1)	17(2)	15(1)	-1(1)	6(1)	-1(1)
C(15)	16(1)	27(2)	21(1)	6(1)	5(1)	3(1)
C(11)	15(1)	22(2)	14(1)	3(1)	1(1)	1(1)
C(21)	13(1)	14(2)	23(1)	0(1)	-1(1)	0(1)
C(2)	13(1)	12(2)	15(1)	0(1)	2(1)	-3(1)
C(20)	16(1)	20(2)	18(1)	2(1)	4(1)	0(1)
C(17)	10(1)	16(2)	18(1)	-1(1)	-2(1)	2(1)
C(14)	12(1)	25(2)	17(1)	-2(1)	-1(1)	7(1)
C(8)	15(1)	15(2)	17(1)	1(1)	2(1)	1(1)
C(4)	13(1)	12(2)	12(1)	0(1)	0(1)	1(1)
C(6)	12(1)	12(2)	13(1)	-1(1)	3(1)	1(1)

Table 5. Hydrogen coordinates (x 10^4) and isotropic displacement parameters (Å^{2}x 10^3) for α-47.

	x	y	z	U(eq)
H(3)	5133	747	9505	26
H(4)	7279	-2196	8420	26
H(2)	5443	7359	9351	28
H(5)	9291	2332	8310	24
H(1A)	3746	6155	8516	19
H(1B)	3698	4029	9059	19
H(19A)	67	11439	9740	45
H(19B)	1671	12248	9844	45
H(19C)	1287	10244	10366	45
H(18A)	2251	8778	9347	33
H(18B)	601	8156	9176	33
H(13A)	1635	7008	5240	28
H(13B)	843	4919	5508	28
H(16)	2146	11857	7341	22
H(3A)	6811	2486	9145	16
H(5A)	7802	1537	7218	15
H(12)	3991	6036	6836	18
H(9)	4590	738	4972	20
H(15)	2362	10022	6173	25
H(21)	416	4727	6868	21
H(2A)	4371	3033	7875	16
H(20)	192	6521	8039	21
H(8)	6355	1259	6117	19
H(4A)	5918	-48	7706	16
H(6)	7139	5362	7148	15

12. References

1. Li, Y. *et al.* Synthesis of kaempferol 3-O-[2″,3″- and 2″,4″-di-O-(E)-p-coumaroyl]-α-l-rhamnopyranosides. *Synlett* 915–918 (2011).

2. Costantino, V., Imperatore, C., Fattorusso, E. & Mangoni, A. A mild and easy one-pot procedure for the synthesis of 2-deoxysugars from glycals. *Tetrahedron Lett.* **41**, 9177–9180 (2000).

3. Quintiliani, M., Balzarini, J. & McGuigan, C. Design, synthesis, and biological evaluation of C1–phosphonamidate analogues of 2-deoxy-d-ribose-1-phosphate. *Tetrahedron* **69**, 9111–9119 (2013).

4. Balmond, E. I., Coe, D. M., Galan, M. C. & McGarrigle, E. M. α-Selective organocatalytic synthesis of 2-deoxygalactosides. *Angew. Chem. Int. Ed.* **51**, 9152–9155 (2012).

5. Konrad, D. B. *et al.* A concise synthesis of tetrodotoxin. *Science* **377**, 411–415 (2022).

6. Kalikanda, J. & Li, Z. Study of the stereoselectivity of 2-azido-2-deoxygalactosyl donors: relationship to the steric factors of glycosyl acceptors. *Carbohydr. Res.* **346**, 2380–2383 (2011).

7. Saehlim, N., Athipornchai, A., Sirion, U. & Saeeng, R. New class of alkynyl glycoside analogues as tyrosinase inhibitors. *Bioorg. Med. Chem. Lett.* **30**, 127276 (2020).

8. Kotammagari, T. K., Gonnade, R. G. & Bhattacharya, A. K. Biomimetic total synthesis of angiopterlactone B and other potential natural products. *Org. Lett.* **19**, 3564–3567 (2017).

9. Riley, A. M., Jenkins, D. J., Marwood, R. D. & Potter, B. V. L. Synthesis of glucopyranoside-based ligands for d-myo-inositol 1,4,5-trisphosphate receptors. *Carbohydr. Res.* **337**, 1067–1082 (2002).

10. Calabrese, C. *et al.* Observation of the unbiased conformers of putative DNA-scaffold ribosugars. *ACS Cent. Sci.* **6**, 293–303 (2020).

11. Li, Y. *et al.* Discovery of a potent, selective renal sodium-dependent glucose cotransporter 2 (SGLT2) inhibitor (HSK0935) for the treatment of type 2 diabetes. *J. Med. Chem.* **60**, 4173–4184 (2017).

12. Eckhardt, M., Hofmann, B. F., Eickelmann, P., Sauer, A. & Thomas, L. Glucopyranosyl-substituted benzonitrile derivatives, pharmaceutical compositions containing such compounds, their use and process for their manufacture. Patent (2010).

13. Thomas, M. E. F. H. P. E. A. S. L. Glucopyranosyl-substituted benzonitrile derivatives, pharmaceutical compositions containing such compounds, their use and process for their manufacture. Patent (2007).

14. Jung, K. L. *et al.* Diphenylmethane derivatives as SGLT2 inhibitors. Patent (2015).

15. Gu, Z. et. al. Pyran glucoside derivatives and their pharmaceutical applications. *CN Patent Appl.* 108203432 A (2018).

16. Frank, W. Optically pure benzyl-4-chlorophenyl-C-glucoside derivative. Patent (2016).

17. Frank, W. Optically pure benzyl-4-chlorophenyl-C-glucoside derivatives as SGLT inhibitors (diabetes mellitus). Patent (2014).

18. Meng, S., Zhong, W., Yao, W. & Li, Z. Stereoselective phenylselenoglycosylation of glycals bearing a fused carbonate moiety toward the synthesis of 2-deoxy-β-galactosides and β-mannosides. *Org. Lett.* **22**, 2981–2986 (2020).

19. Wang, D.-d. *et al.* Synthesis of MeON-neoglycosides of digoxigenin with 6-deoxy- and 2,6-dideoxy-d-glucose derivatives and their anticancer activity. *Bioorg. Med. Chem. Lett.* **27**, 3359–3364 (2017).

20. Trinderup, H. H., Andersen, S. M., Heuckendorff, M. & Jensen, H. H. How do various reaction parameters influence anomeric selectivity in chemical glycosylation with thioglycosides and NIS/TfOH activation? *Eur. J. Org. Chem.* **2021**, 3251–3259 (2021).

21. Alsarraf, J., Petitpoisson, L. & Pichette, A. Catalytic site-selective carbamoylation of pyranosides. *Org. Lett.* **23**, 6052–6056 (2021).

22. Qian, P.-z., Yao, W., Huang, L.-b., Meng, X.-b. & Li, Z.-j. A mild and efficient method for the selective cleavage of primary p-methoxybenzyl protecting group of saccharides by $Co_2(CO)_8$–Me_2PhSiH–CO system. *Tetrahedron Lett.* **56**, 5238–5241 (2015).

23. Adamo, M. F. A. & Pergoli, R. Studies on the generation of unnatural C-nucleosides with 1-alkynyl-2-deoxy-d-riboses. *Org. Lett.* **9**, 4443–4446 (2007).

24. Ichikawa, Y.-i., Kusama, H., Fujita, K., Okauchi, T. & Narasaka, K. Stereoselective β-C- and β-S-glycosylation of 2-deoxyribofuranose derivatives controlled by the 3-hydroxy protective group. *Bull. Chem. Soc. Jpn.* **62**, 7 (1989).

25. Siriwardena, A. *et al.* Sugar-modified foldamers as conformationally defined and biologically distinct glycopeptide mimics. *Angew. Chem. Int. Ed.* **52**, 10221–10226 (2013).

26. Gong, H. & Gagné, M. R. Diastereoselective Ni-catalyzed Negishi cross-coupling approach to saturated, fully oxygenated C-alkyl and C-aryl glycosides. *J. Am. Chem. Soc.* **130**, 12177–12183 (2008).

27. Xia, Y. *et al.* Synthesis of 2-indolyl C-glycoside neopetrosins A and C and congeners via Ni-catalyzed photoreductive cross-coupling. *Org. Lett.* **25**, 6741–6745 (2023).

28. Syafii, M. I., Kimura, R., Ito, S., Miyasaka, T. & Nishikawa, T. Concise total synthesis of neopterosin A and C by a Pd-catalyzed tandem reaction. *Synthesis* (2025).

29. Nishikawa, T. *et al.* Stereocontrolled syntheses of α-C-mannosyltryptophan and its analogues. *Org. Biomol. Chem.* **3**, 687–700 (2005).

30. Mao, R. *et al.* Synthesis of C-mannosylated glycopeptides enabled by Ni-catalyzed photoreductive cross-coupling reactions. *J. Am. Chem. Soc.* **143**, 12699–12707 (2021).

31. Manabe, S. & Ito, Y. Total synthesis of novel subclass of glyco-amino acid structure motif: C2-α-l-C-mannosylpyranosyl-l-tryptophan. *J. Am. Chem. Soc.* **121**, 9754–9755 (1999).

32. Wang, Q. *et al.* Palladium-catalysed C−H glycosylation for synthesis of C-aryl glycosides. *Nat. Catal.* **2**, 793–800 (2019).

33. Wang, Q. *et al.* Total synthesis of C-α-mannosyl tryptophan via palladium-catalyzed C–H glycosylation. *CCS Chem.* **3**, 1729–1736 (2021).

34. Li, Y. *et al.* Chemoselective and diastereoselective synthesis of C-aryl nucleoside analogues by nickel-catalyzed cross-coupling of furanosyl acetates with aryl iodides. *Angew. Chem. Int. Ed.* **61**, e202110391 (2022).

35. Bonnac, L. F. *et al.* Efficient synthesis of benzamide riboside, a potential anticancer agent. *Nucleosides Nucleotides Nucleic Acids* **26**, 1249–1253 (2007).

36. Joshua, V. A., Sharma, K. & Abrams, D. N. New short synthesis of (5)-2,3-dimethoxy-N-[(1-ethyl-2-pyrrolidinyl)methyl]-5-iodobenzamide: dopamine D2 receptor. *Synth. Commun.* **38**, 434–440 (2008).

37. Beccalli, E. M., Broggini, G., Marchesini, A. & Rossi, E. Intramolecular Heck reaction of 2- and 3-iodoindole derivatives for the synthesis of β- and γ-carbolinones. *Tetrahedron* **58**, 6673–6678 (2002).

38. Štefko, M., Pohl, R. & Hocek, M. Synthesis of benzamide-C-ribonucleosides by Pd-catalyzed aminocarbonylations. *Tetrahedron* **65**, 4471–4483 (2009).

39. Ho, W. C., Chung, K., Ingram, A. J. & Waymouth, R. M. Pd-catalyzed aerobic oxidation reactions: strategies to increase catalyst lifetimes. *J. Am. Chem. Soc.* **140**, 748–757 (2018).

40. Jäger, M., Hartmann, M., de Vries, J. G. & Minnaard, A. J. Catalytic regioselective oxidation of glycosides. *Angew. Chem. Int. Ed.* **52**, 7809–7812 (2013).

13. NMR Spectra

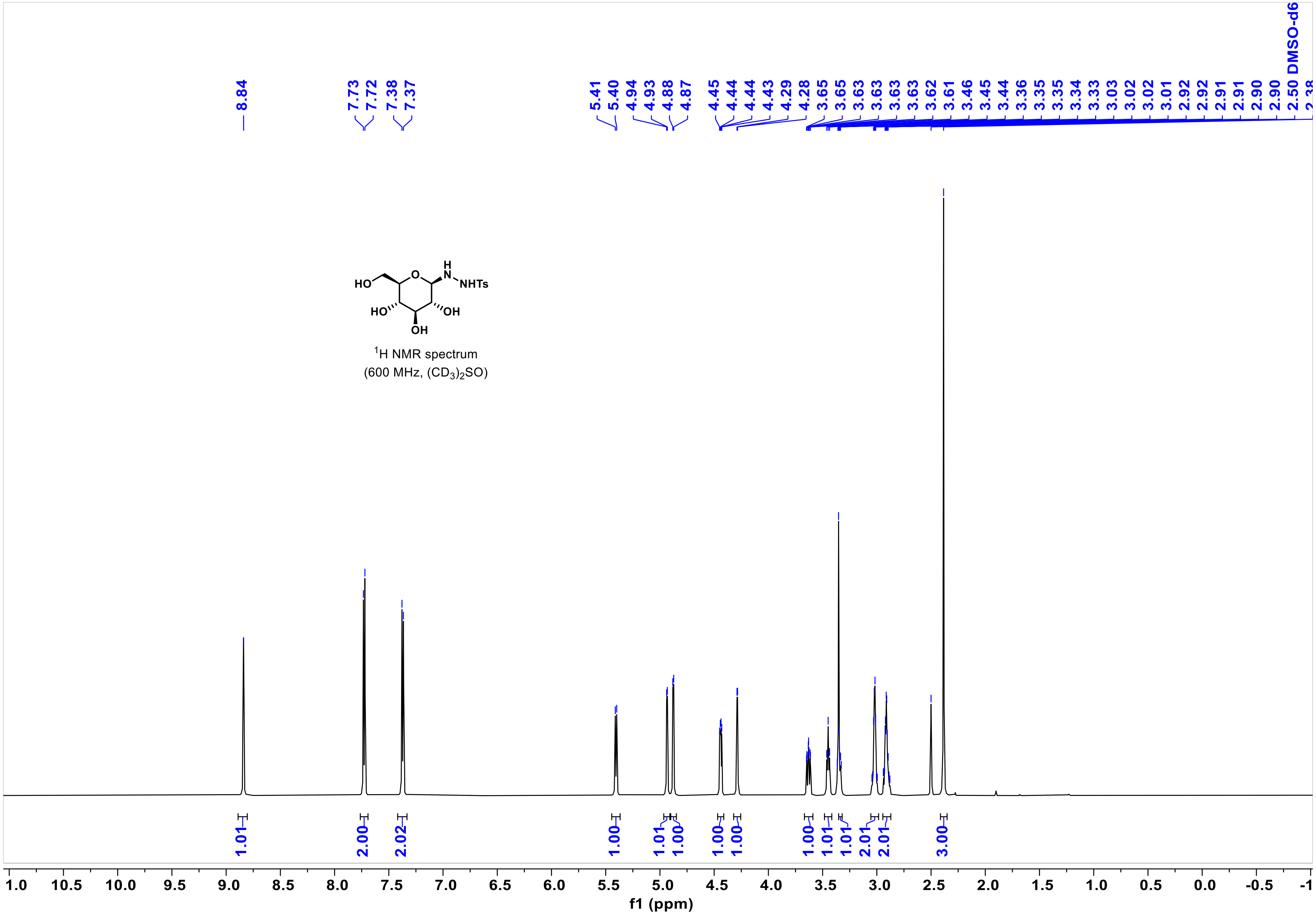

^{1}H NMR of **Glucosyl-hydrazide 25**

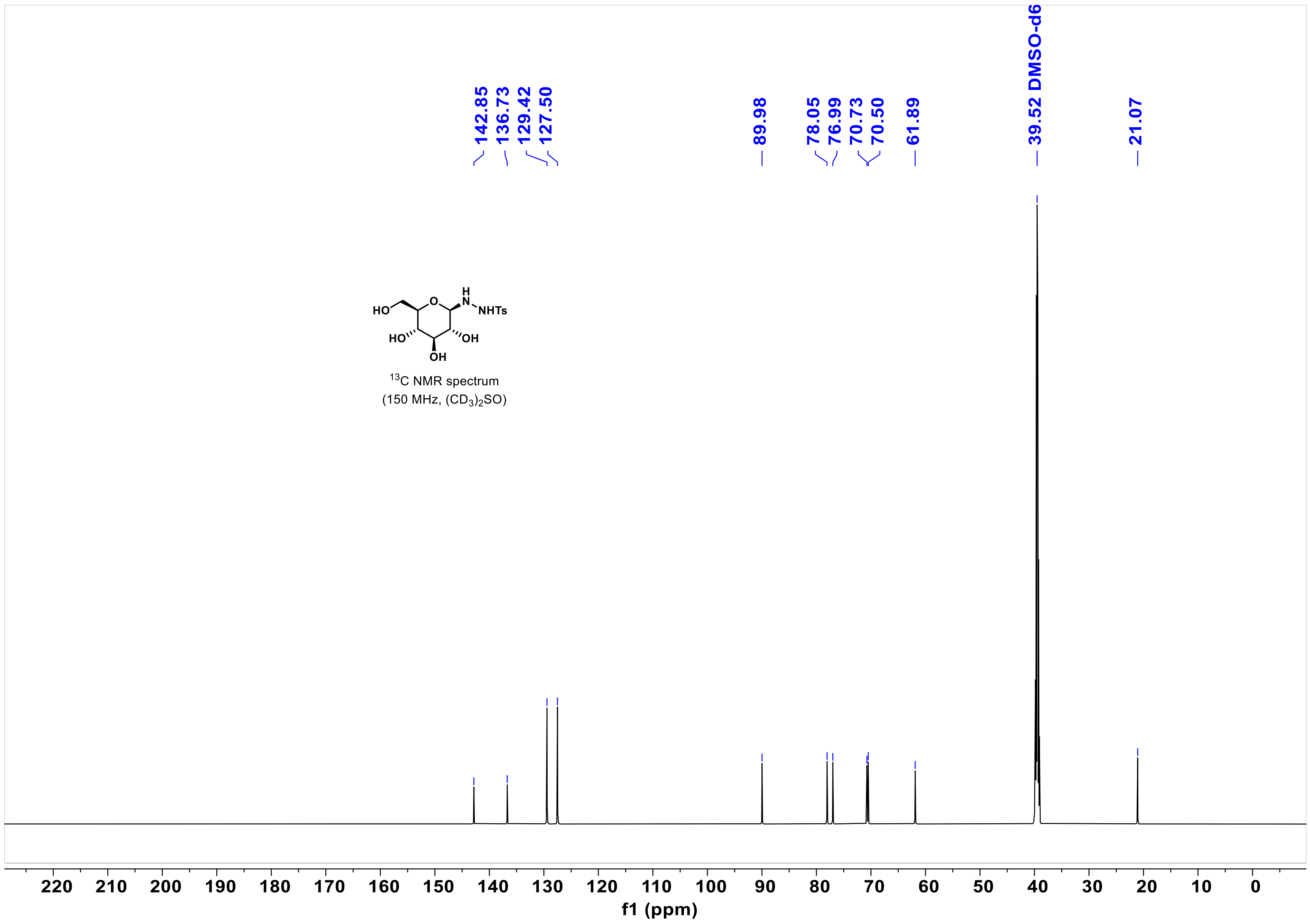

^{13}C NMR of **Glucosyl-hydrazide 25**

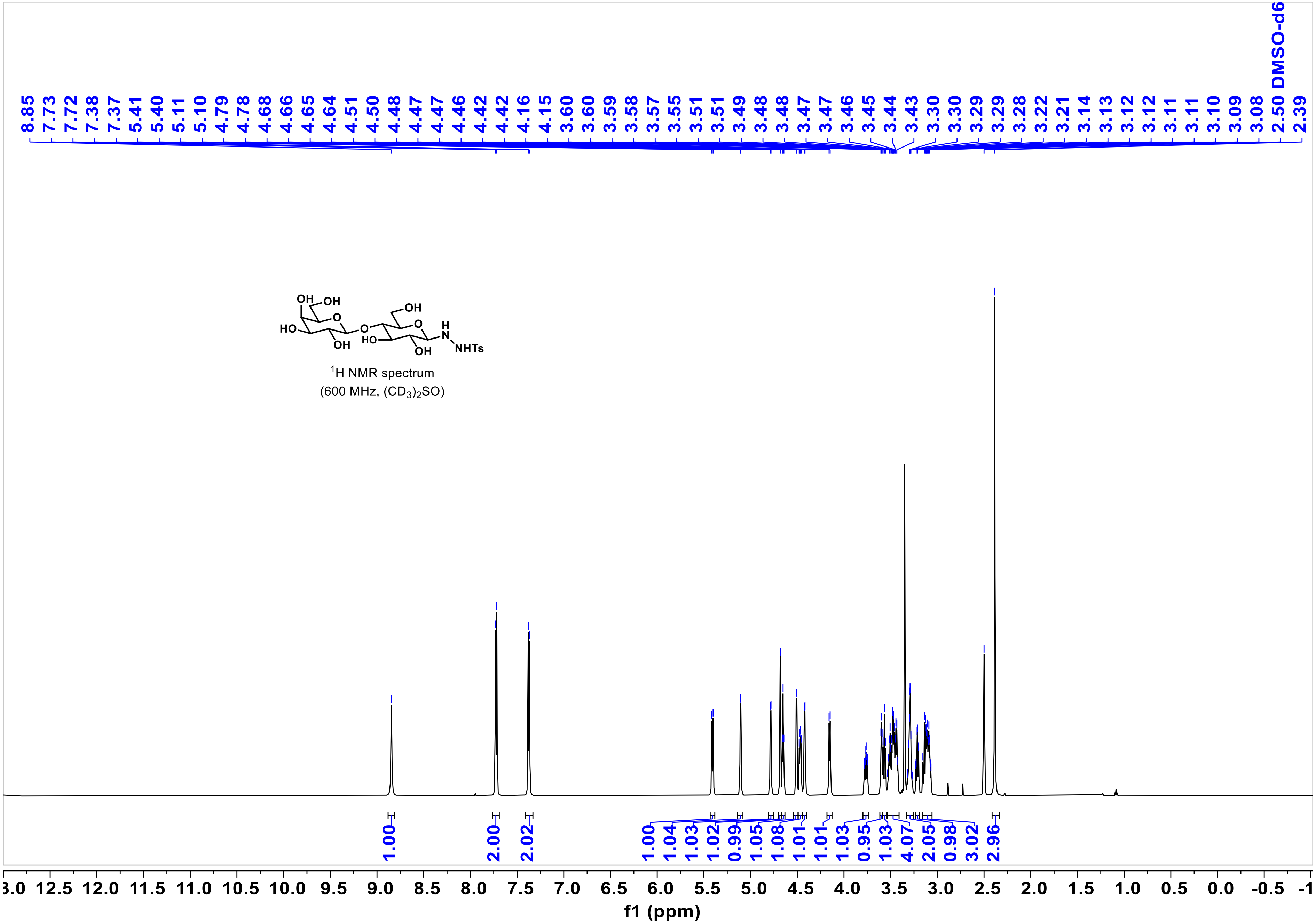

^{1}H NMR of **Lactose-hydrazide SH1**

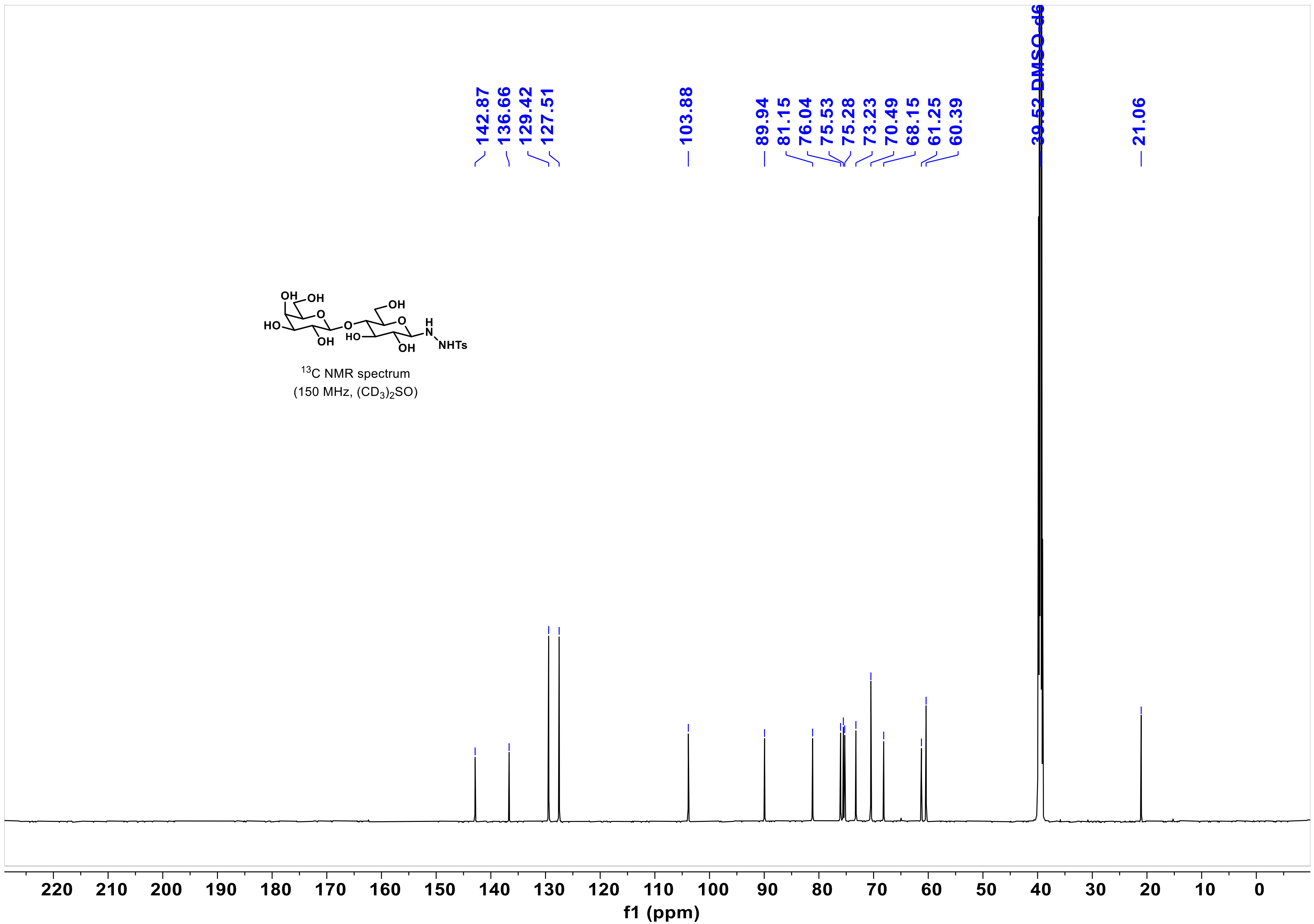

^{13}C NMR of **Lactose-hydrazide SH1**

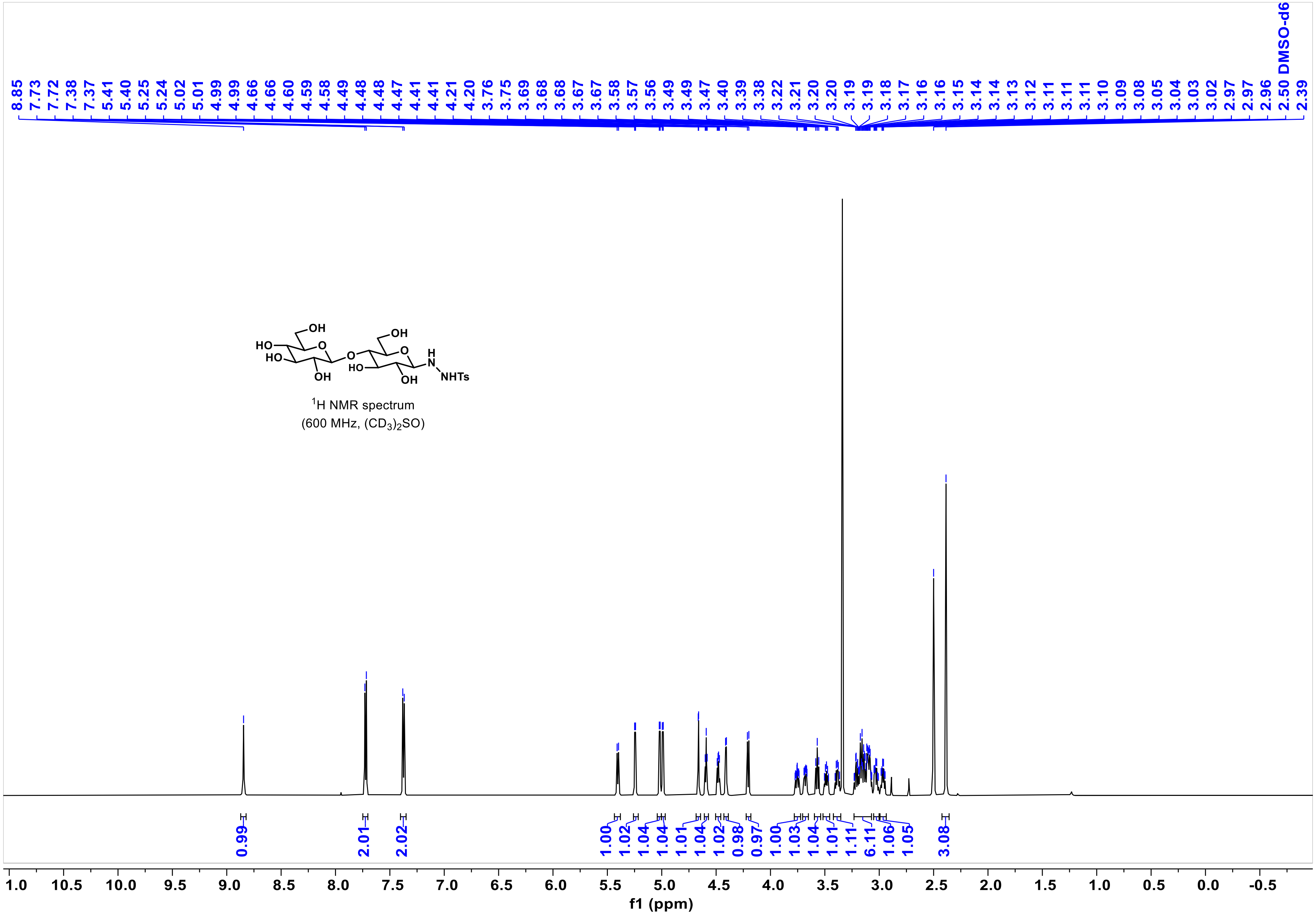

^{1}H NMR of **Cellobiose-hydrazide SH2**

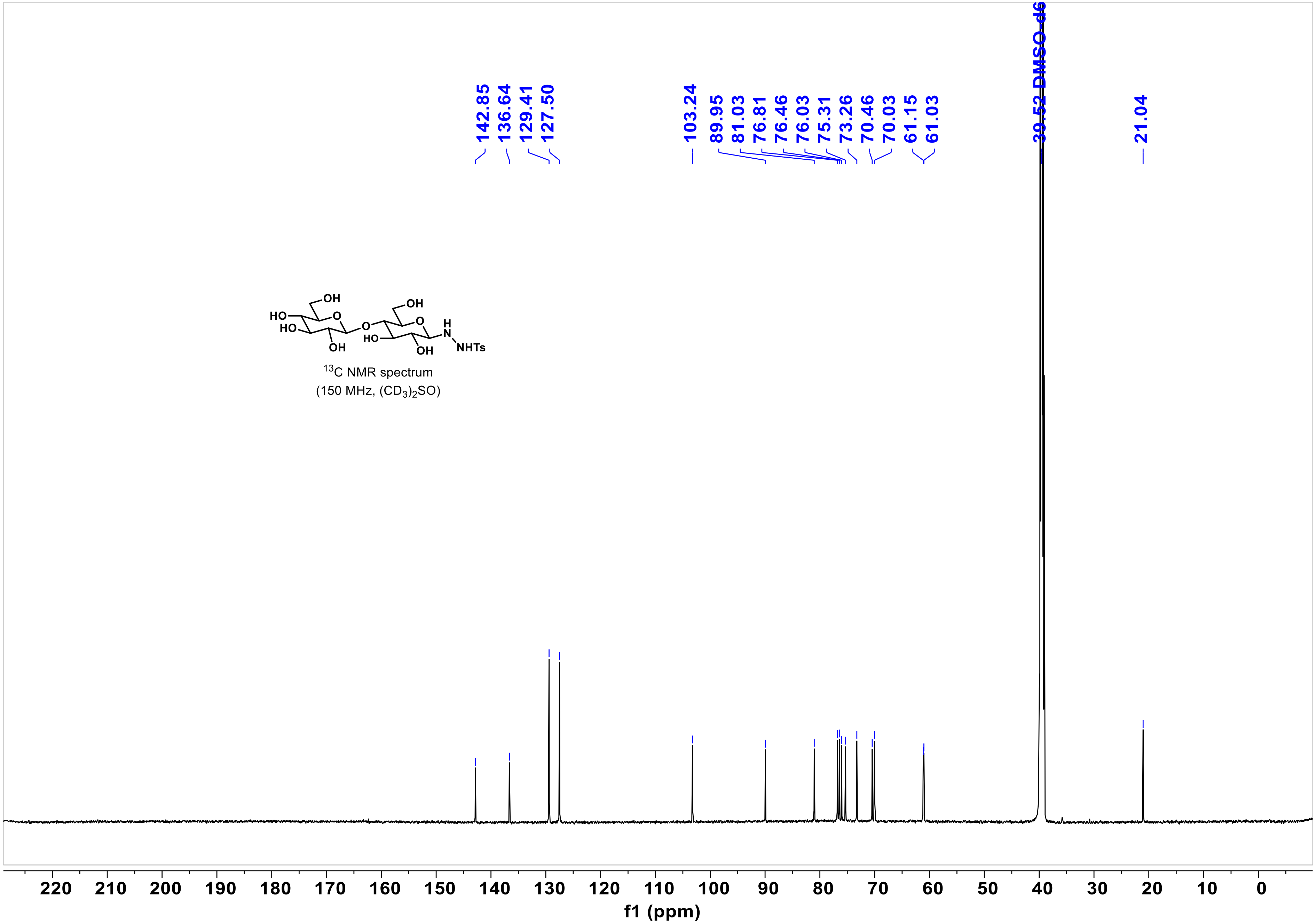

^{13}C NMR of **Cellobiose-hydrazide SH2**

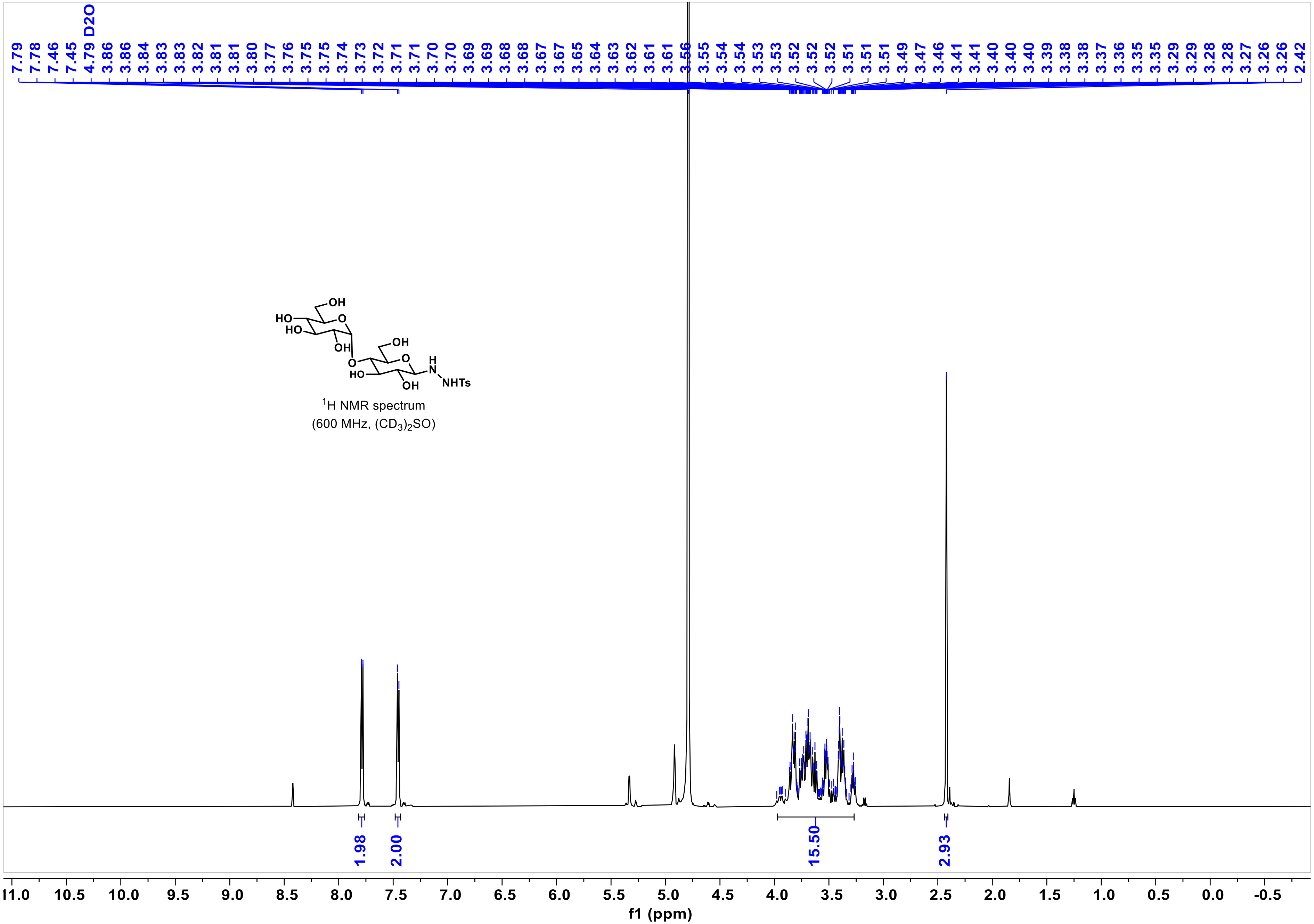

^{1}H NMR of **Maltose-hydrazide SH3**

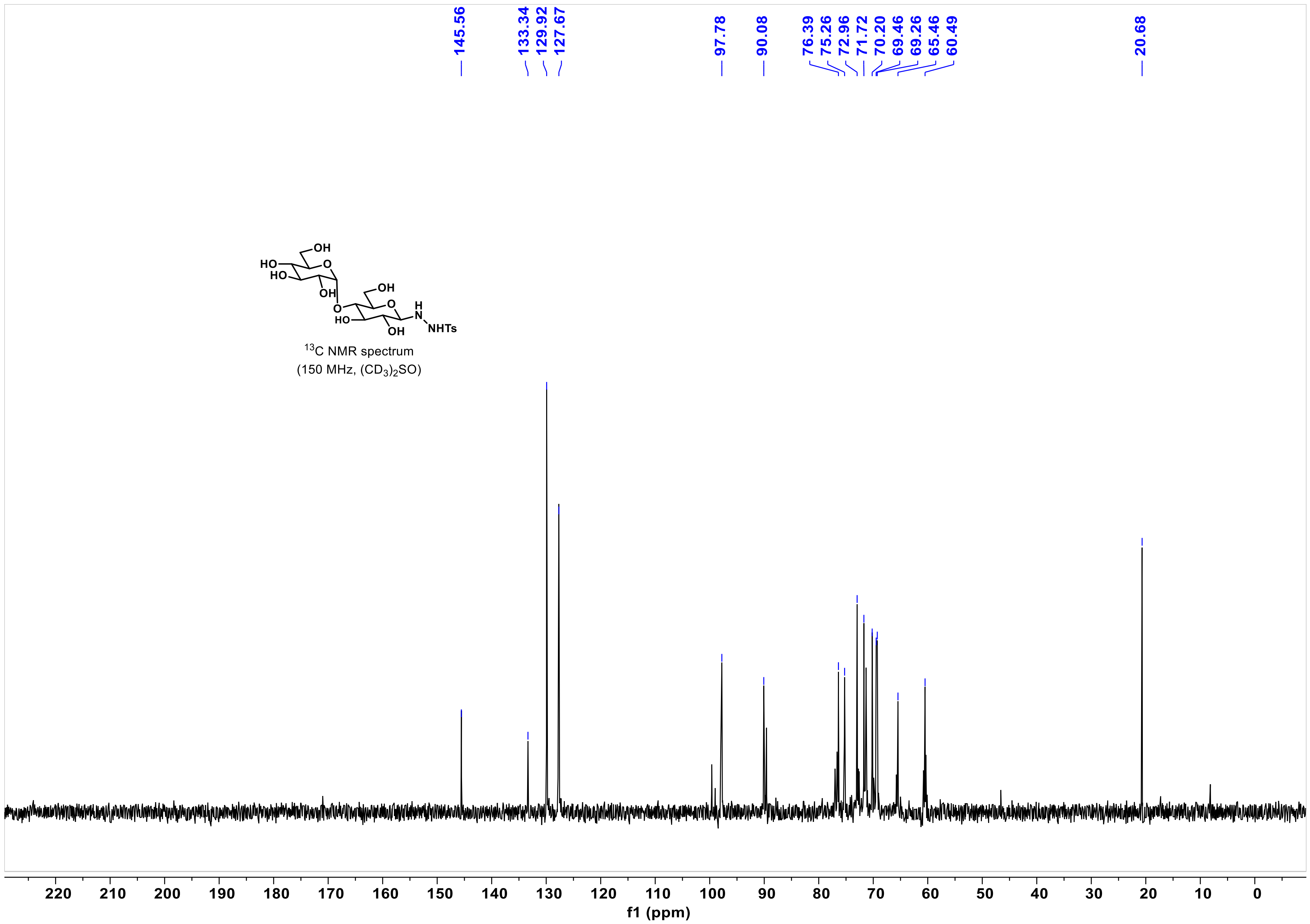

^{13}C NMR of **Maltose-hydrazide SH3**

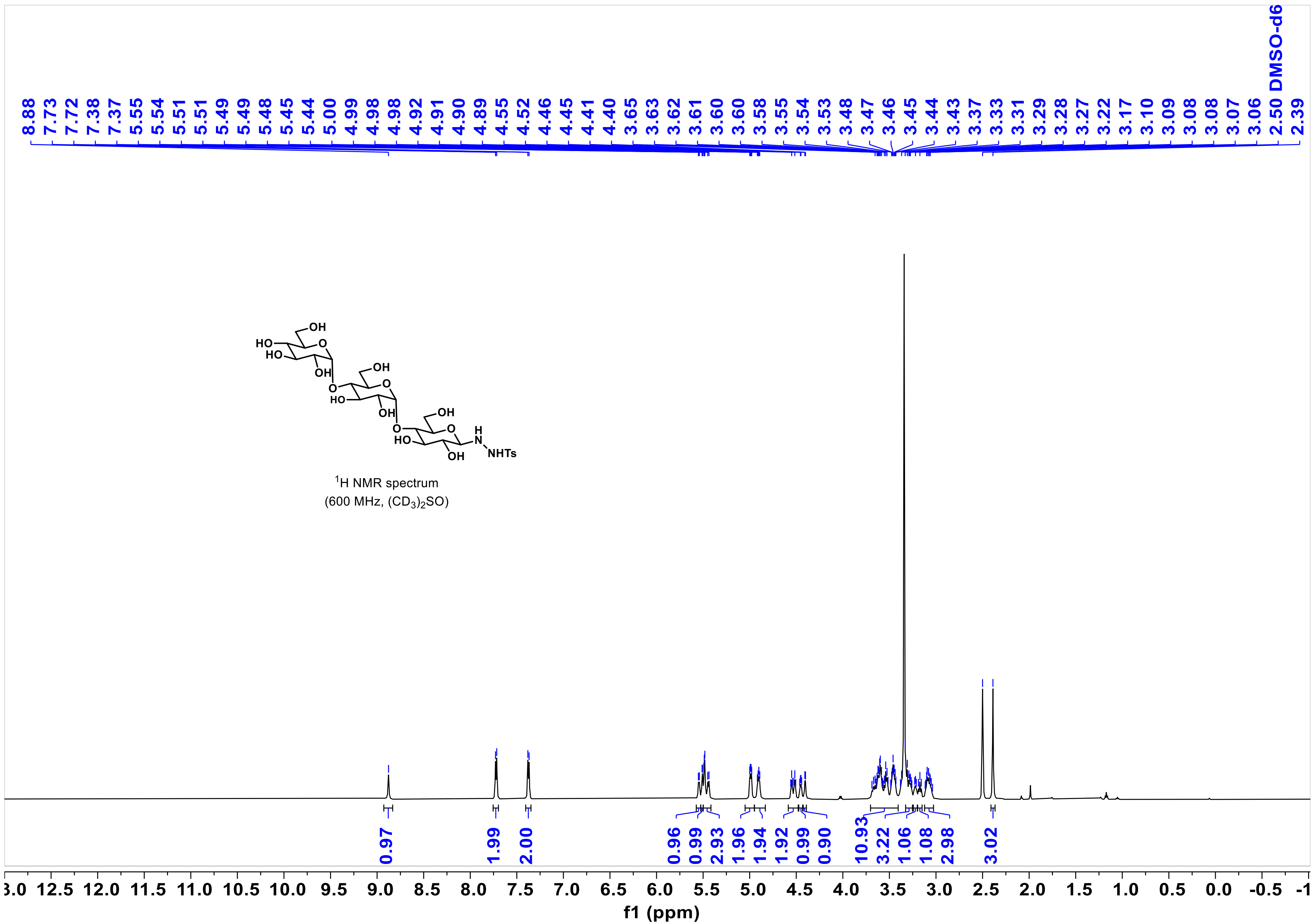

^{1}H NMR of **Maltotriose-hydrazide SH4**

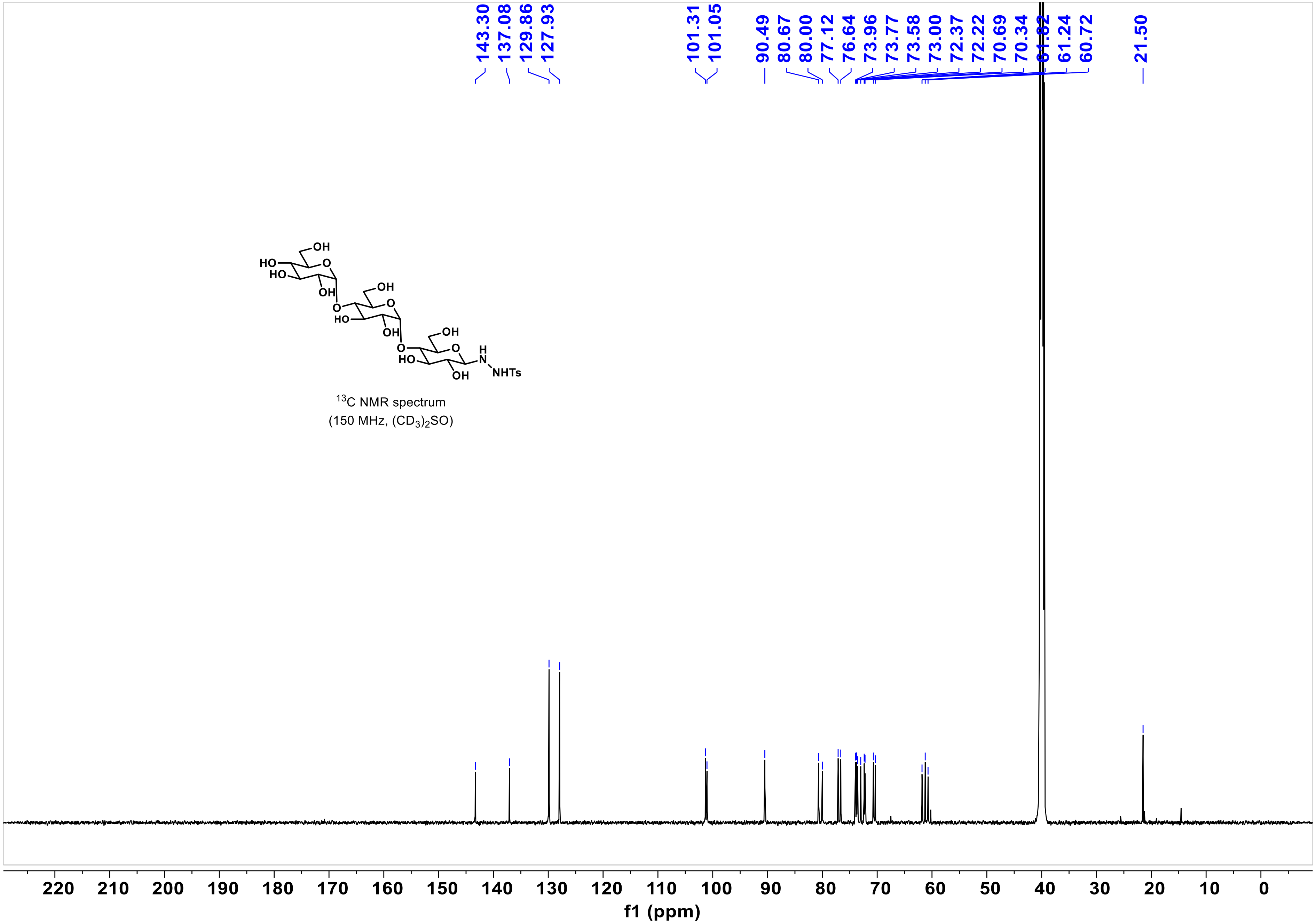

^{13}C NMR of **Maltotriose-hydrazide SH4**

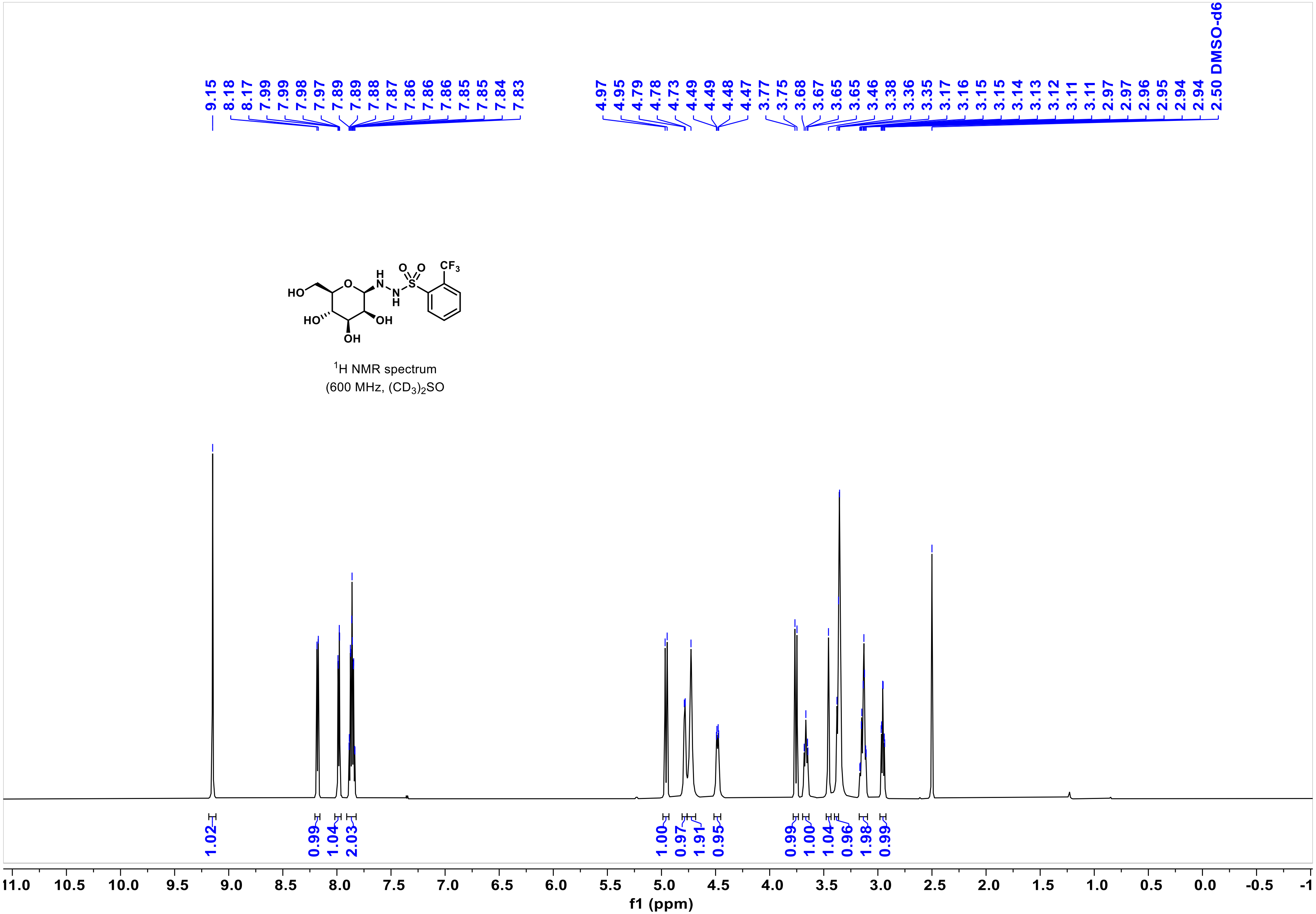

^{1}H NMR of **Mannose-hydrazide 73**

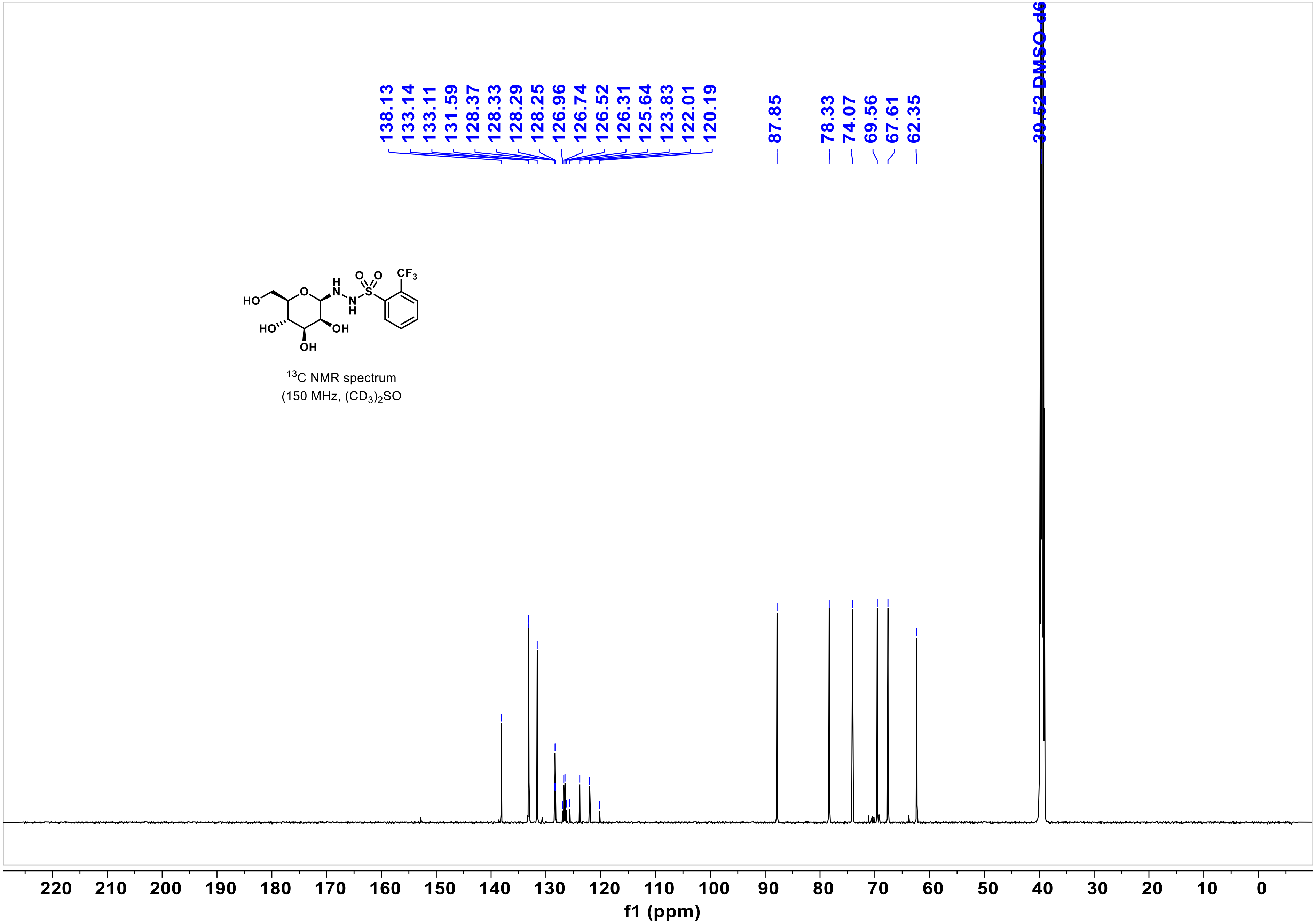

^{13}C NMR of **Mannose-hydrazide 73**

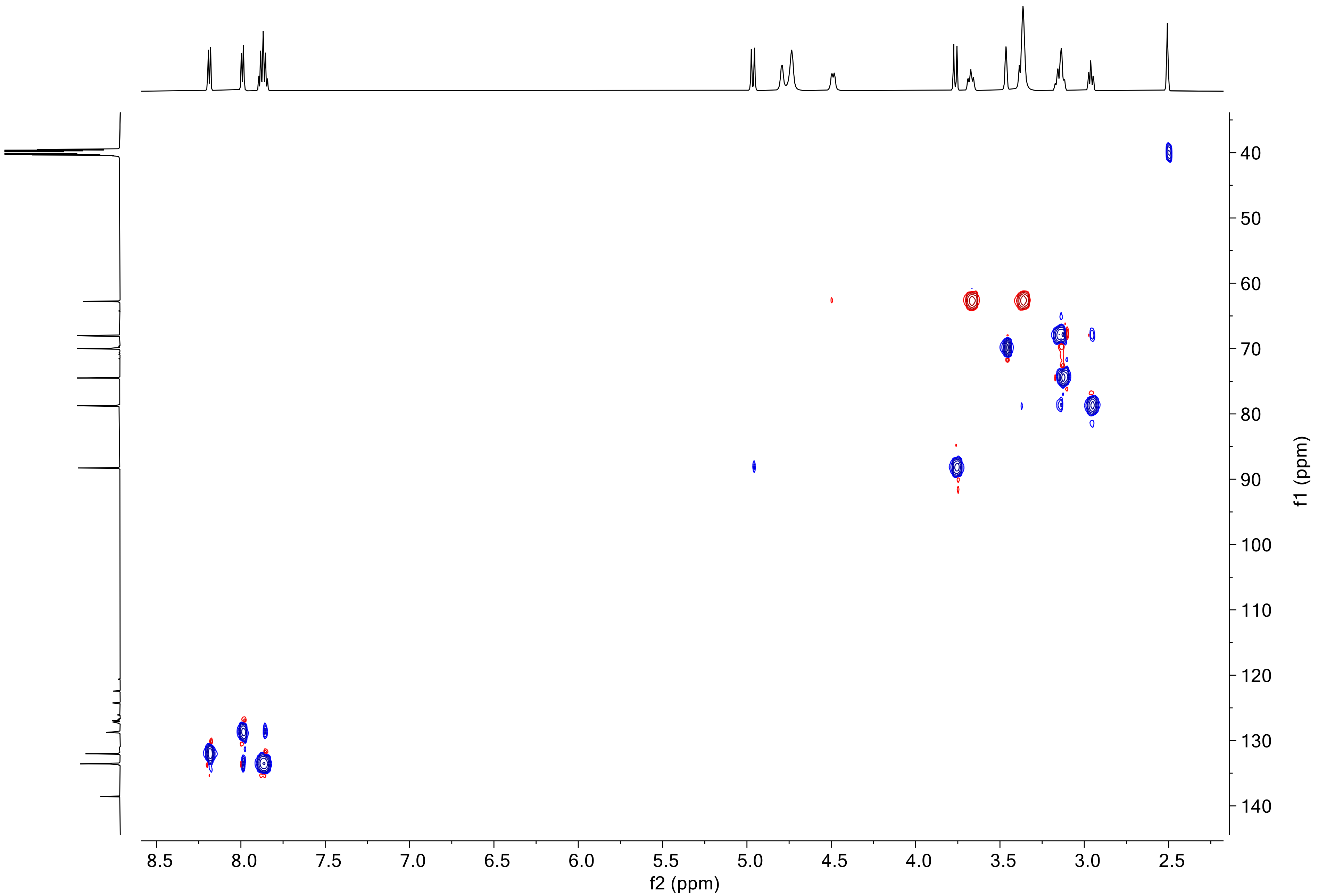

H-H HSQC NMR Spectrum of **Mannose-hydrazide 73**

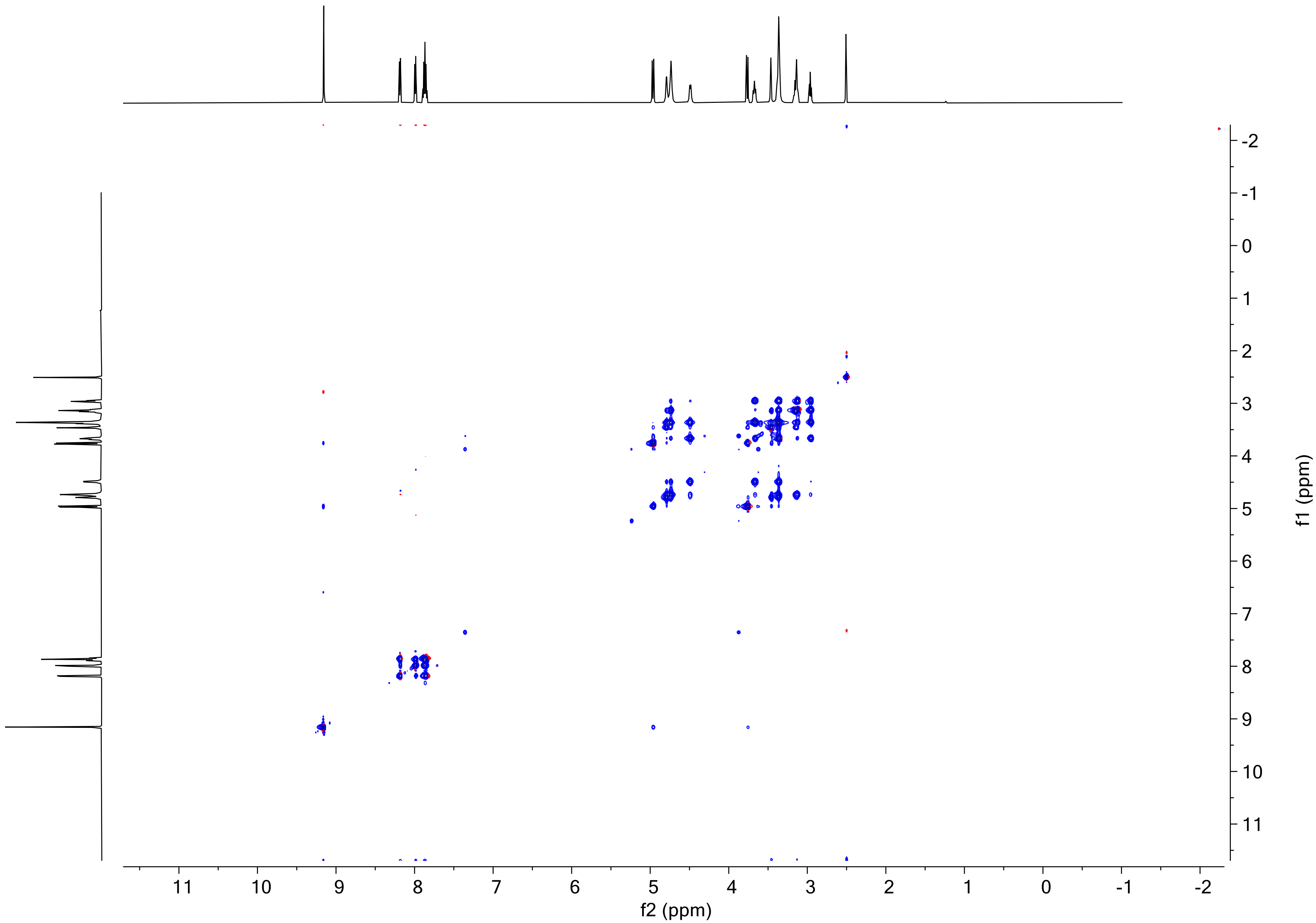

H-H COSY NMR Spectrum of **Mannose-hydrazide 73**

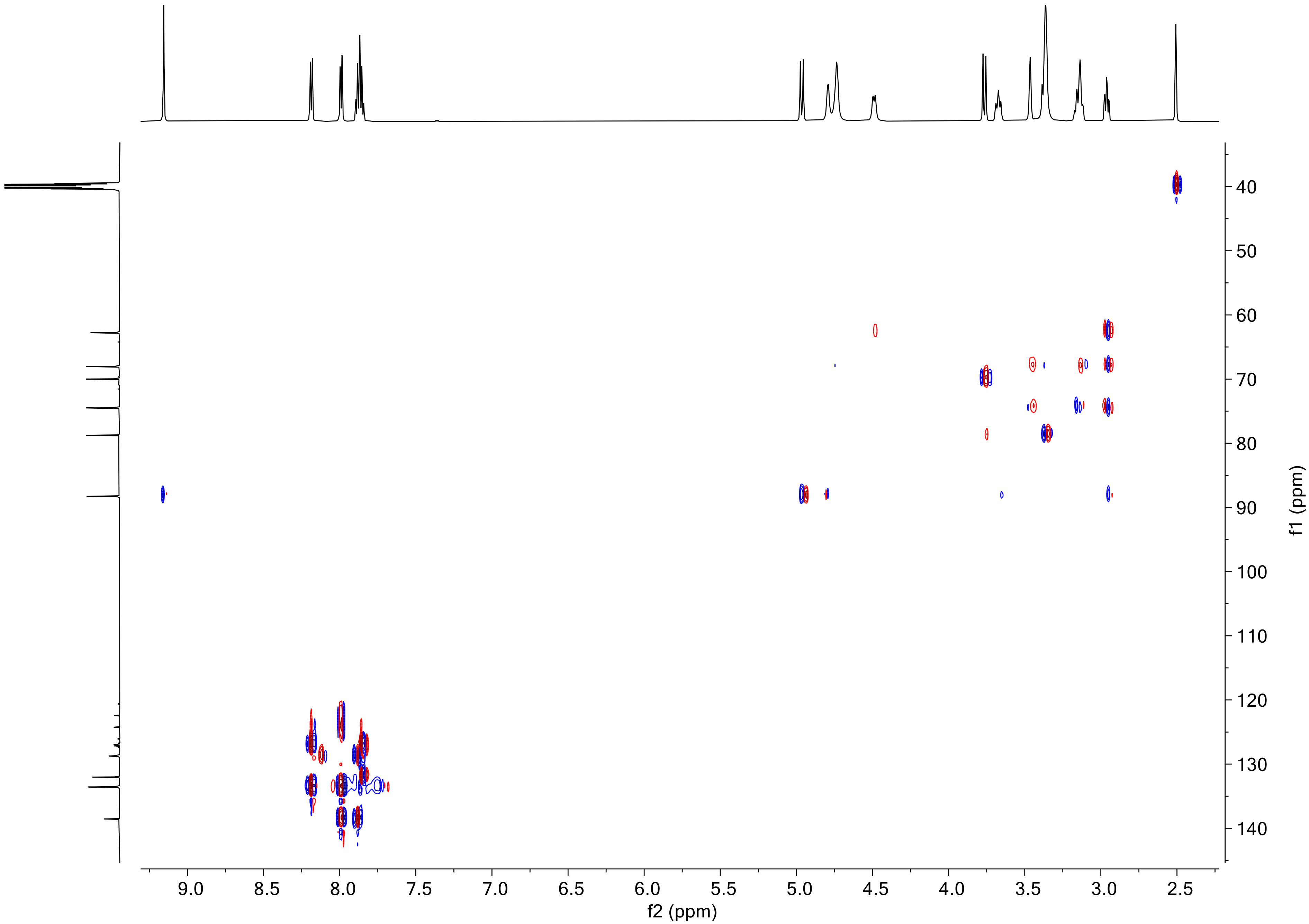

H-C HMBC NMR Spectrum of **Mannose-hydrazide 73**

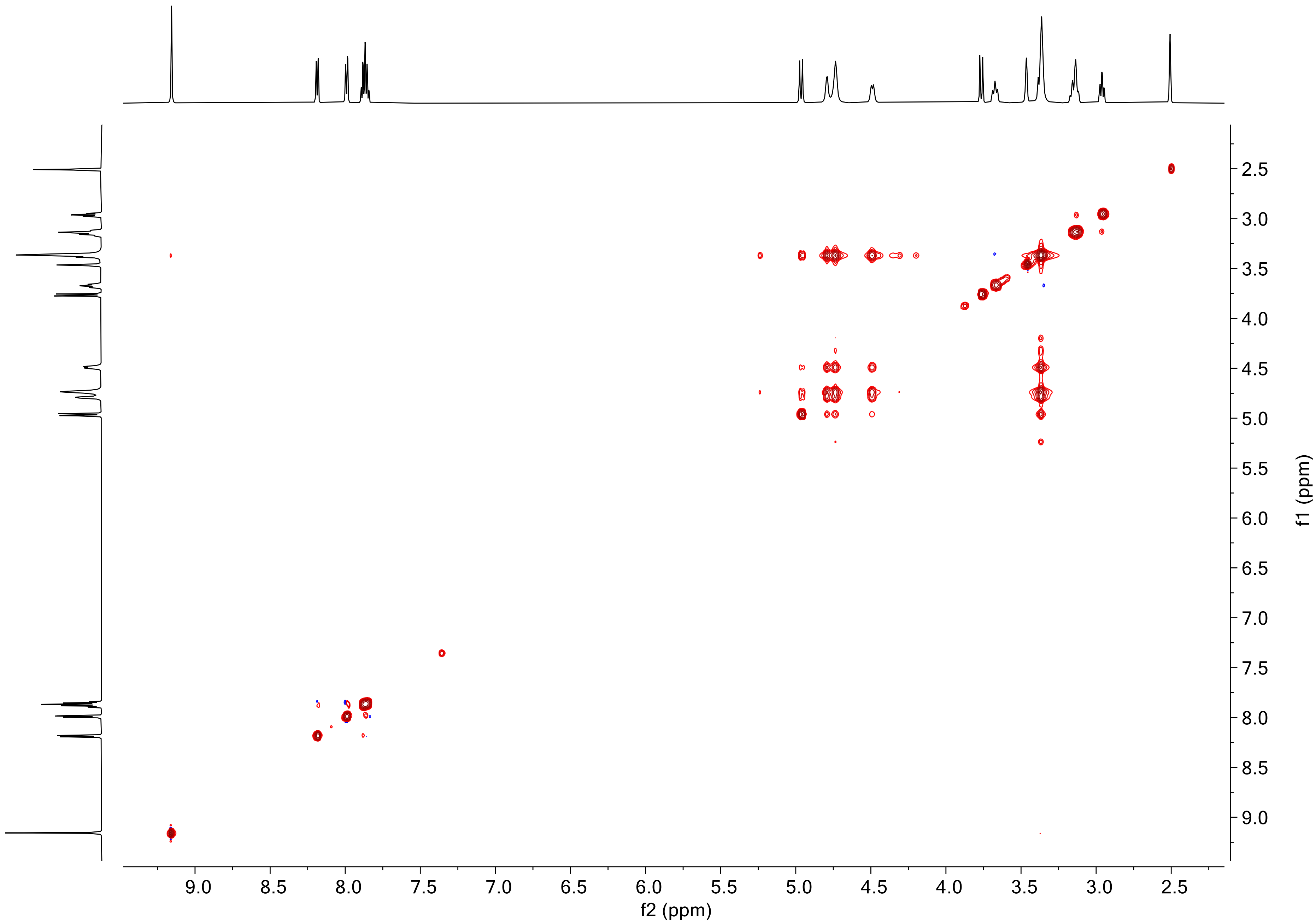

H-H NOESY NMR Spectrum of **Mannose-hydrazide 73**

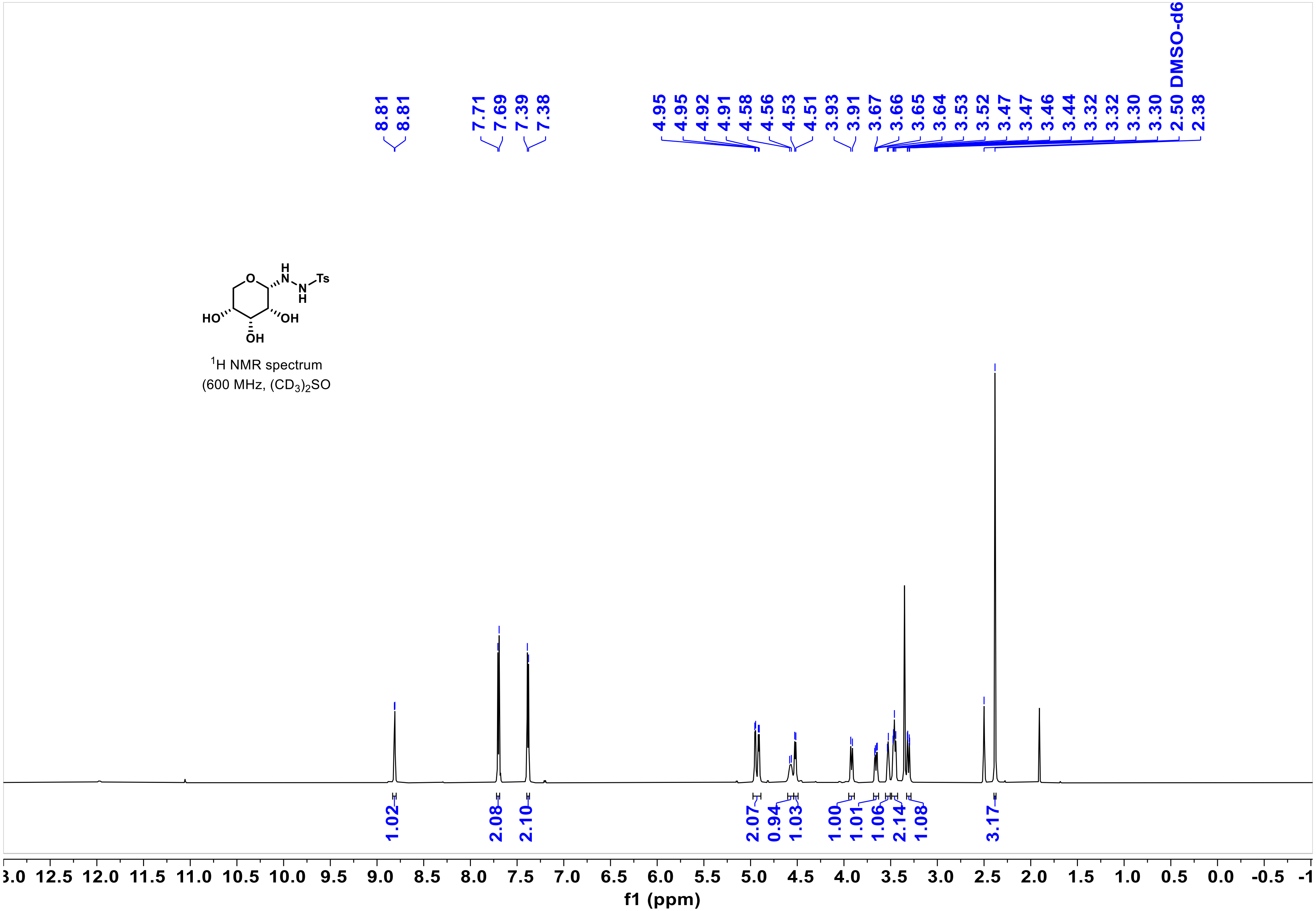

^{1}H NMR of compound **Ribose-hydrazide 78**

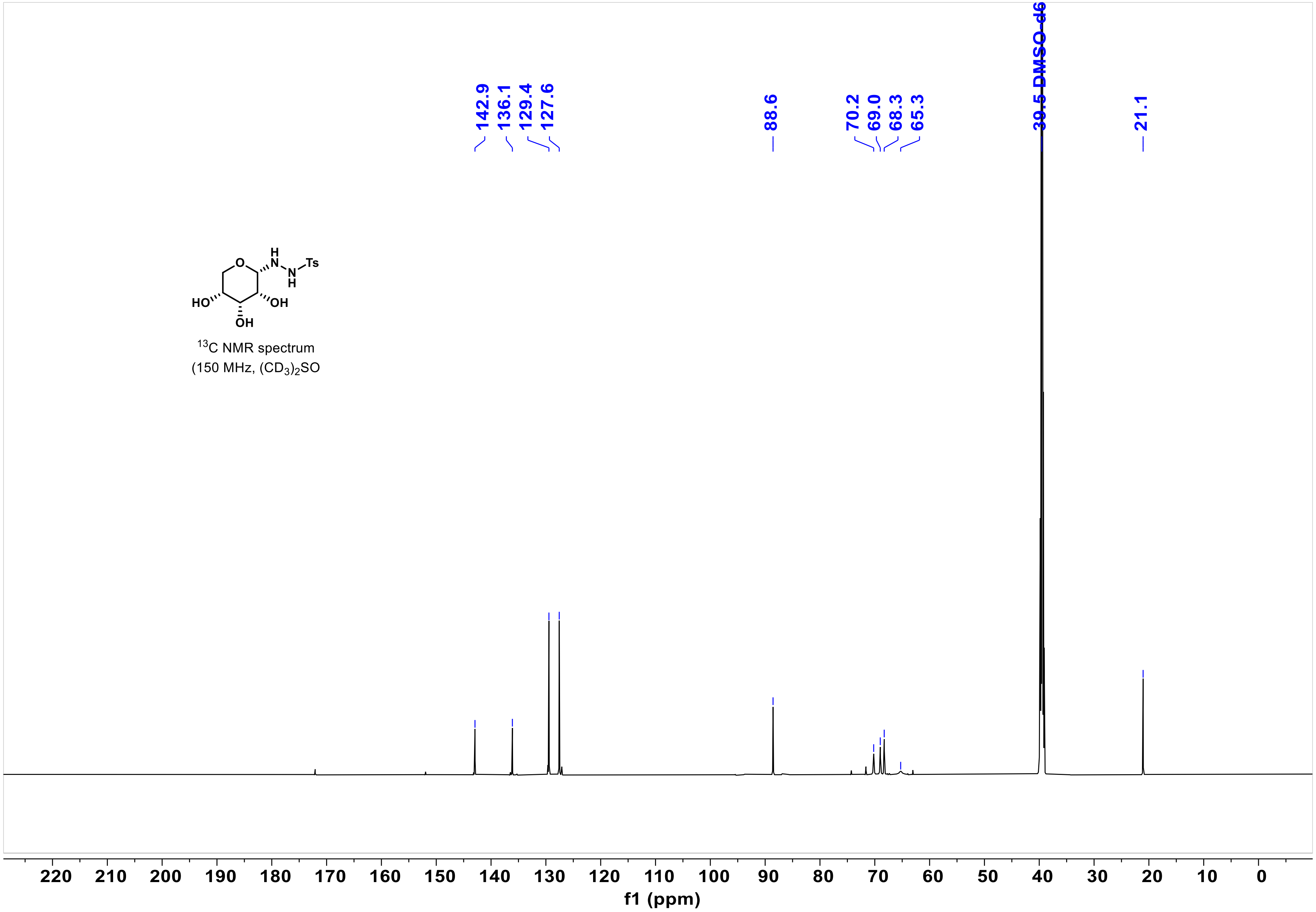

^{13}C NMR of compound **Ribose-hydrazide 78**

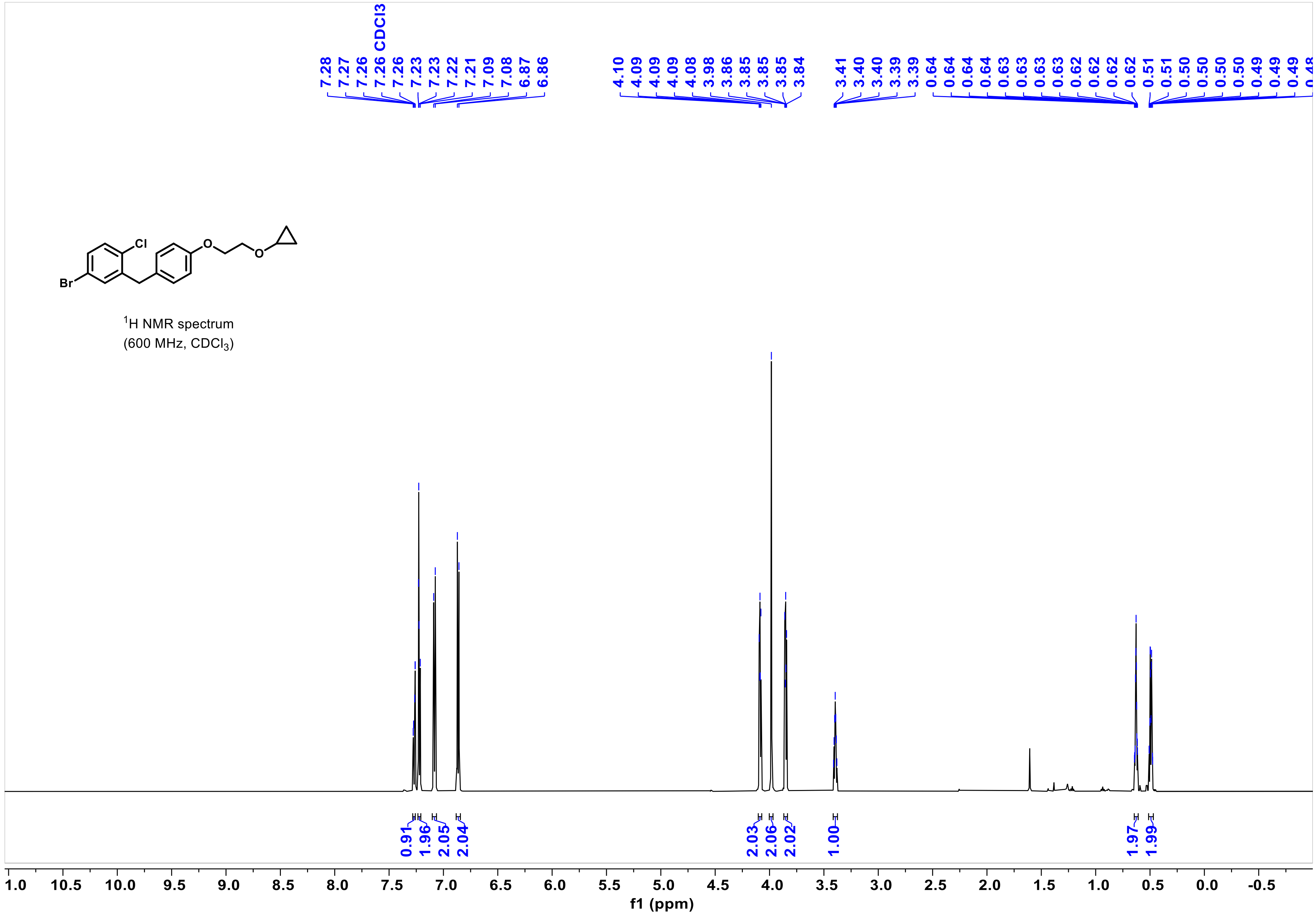

^{1}H NMR of compound **SG7**

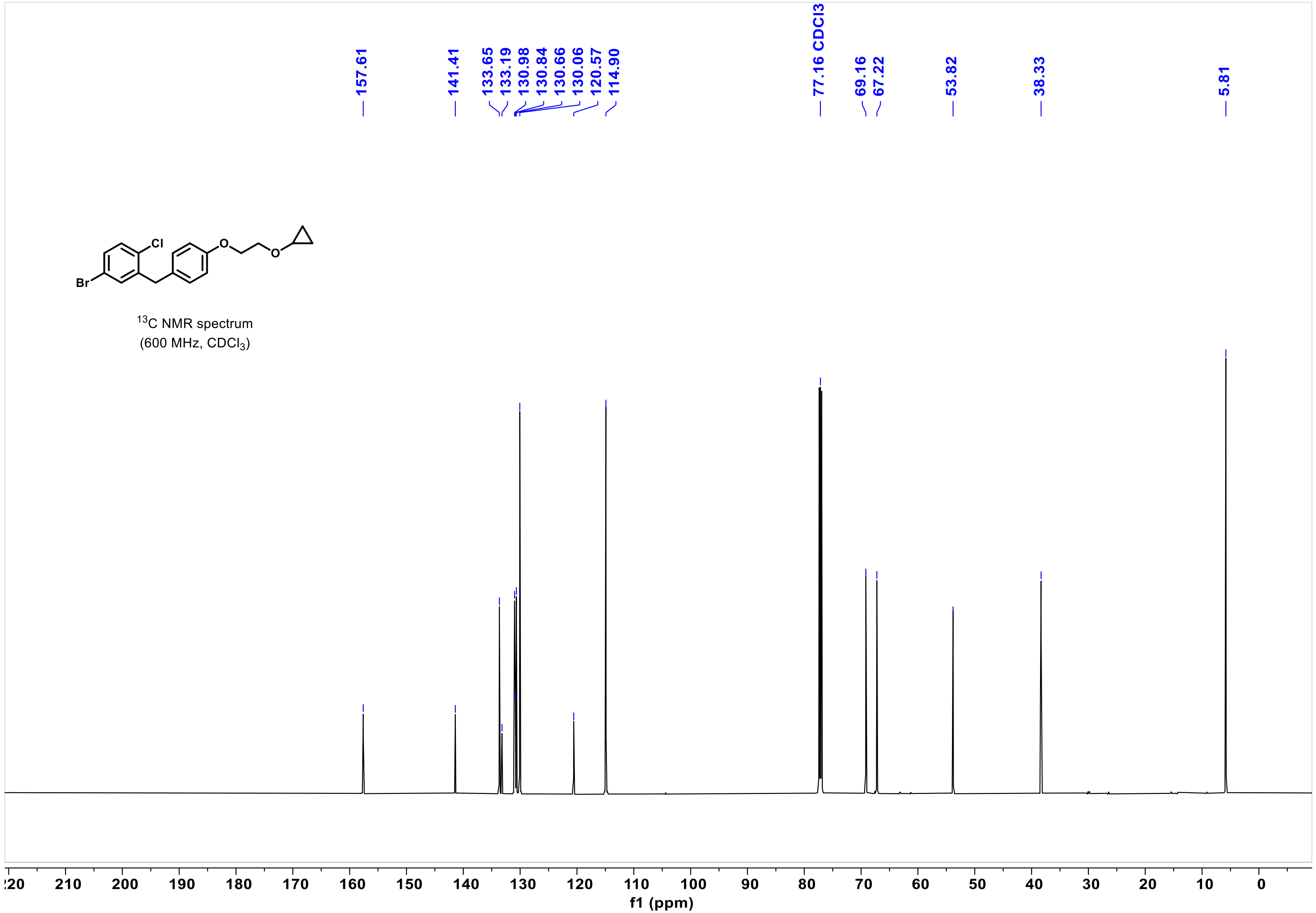

^{13}C NMR of compound **SG7**

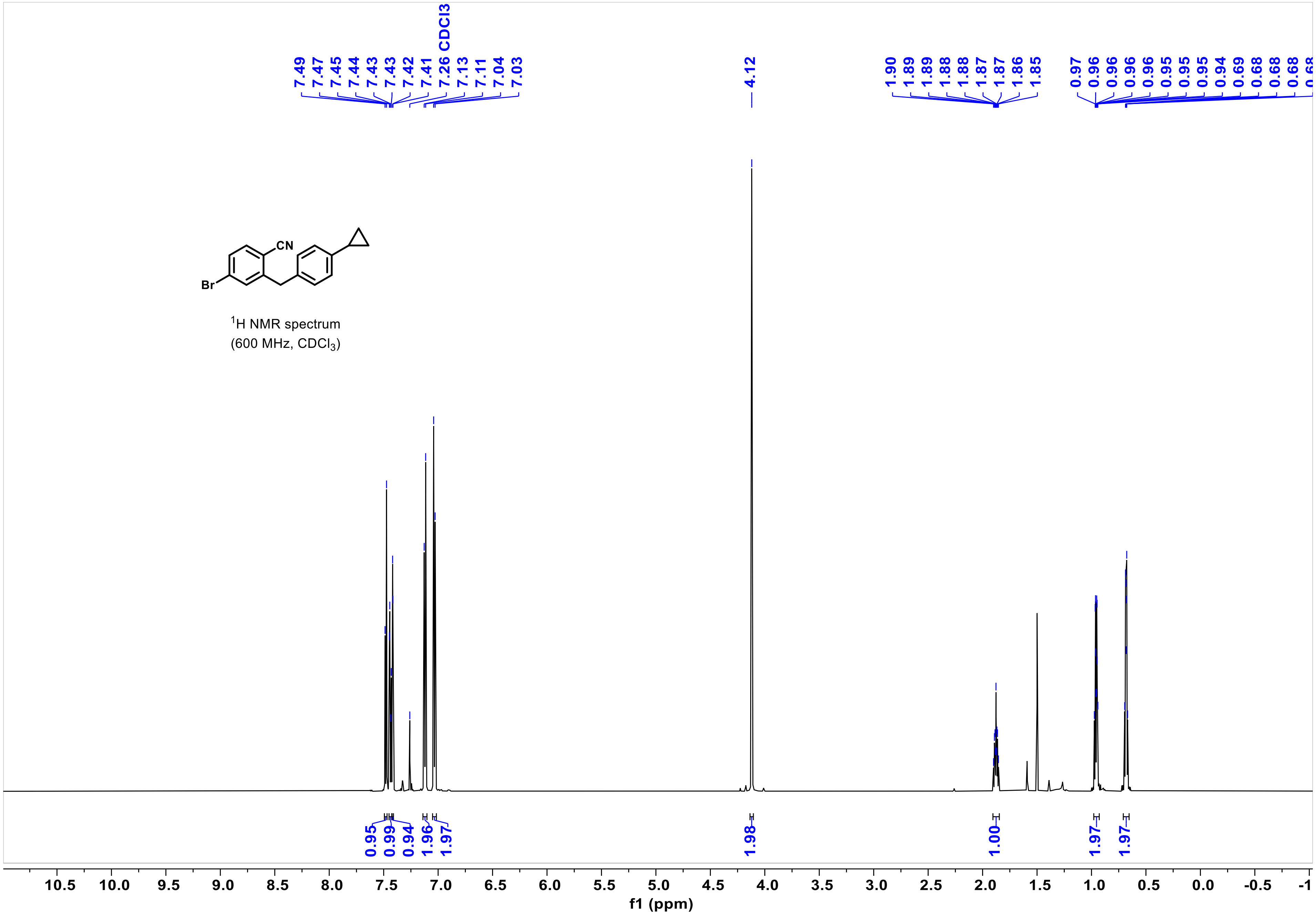

^{1}H NMR of compound **SG8**

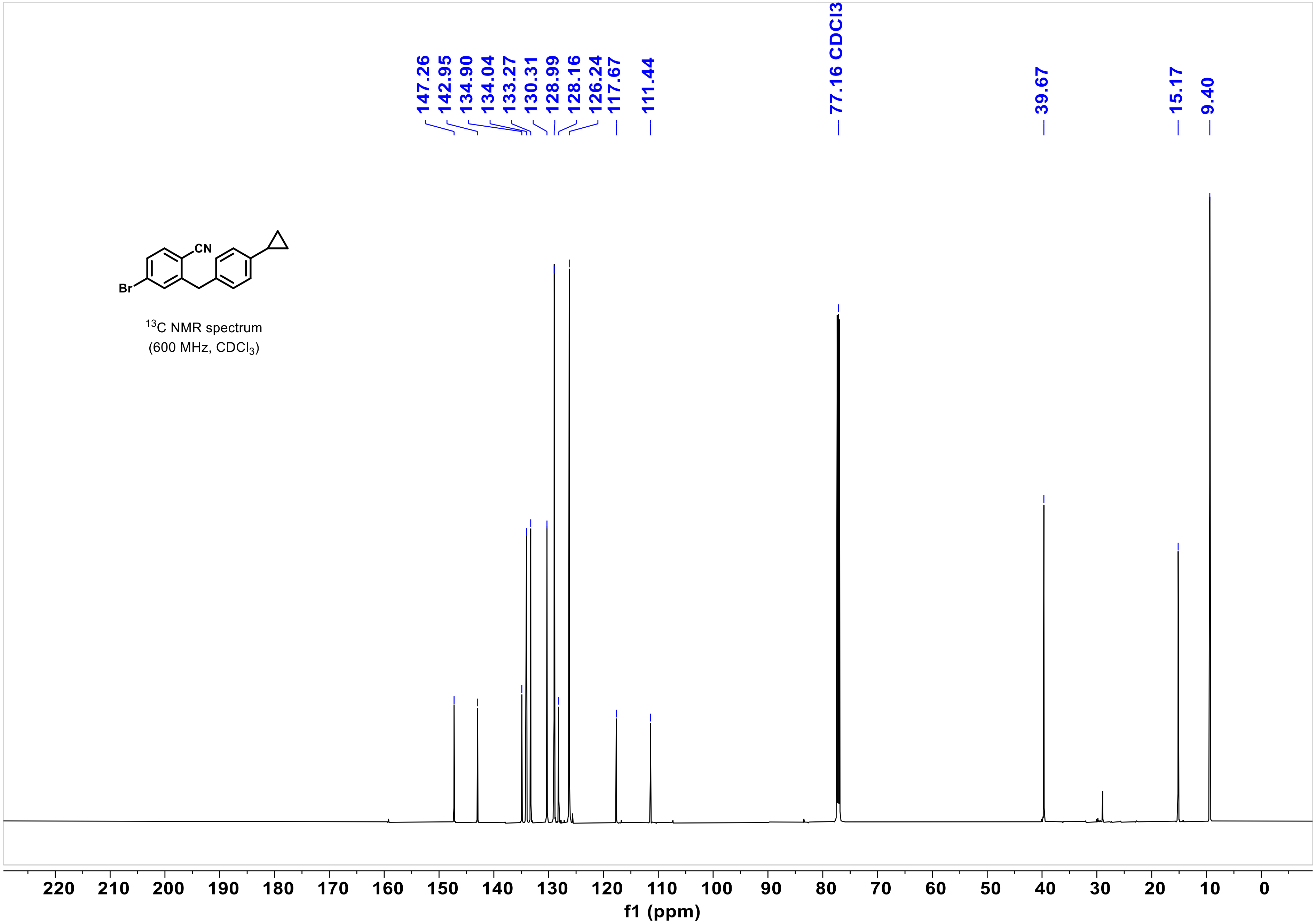

^{13}C NMR of compound **SG8**

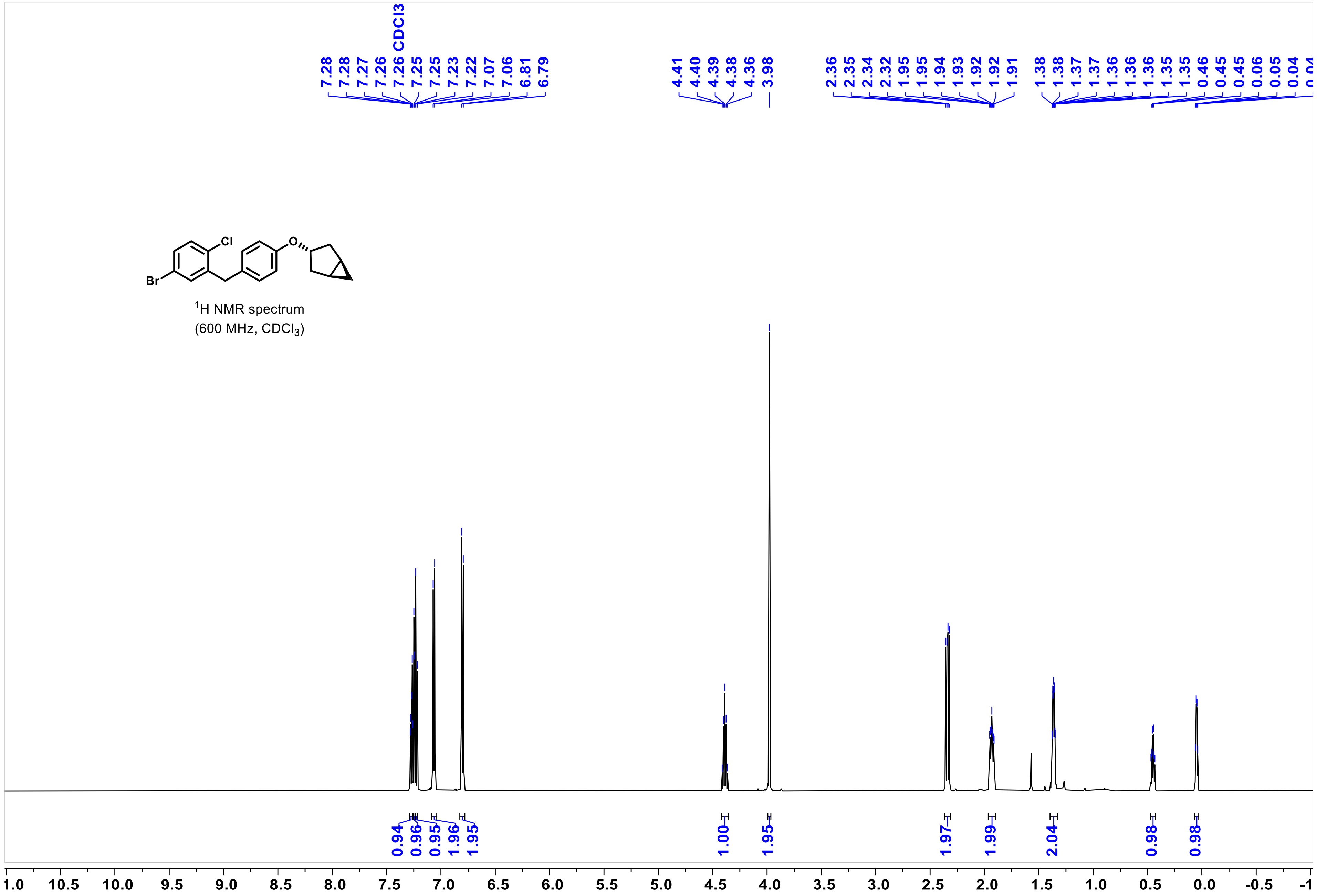

^{1}H NMR of compound **SG11**

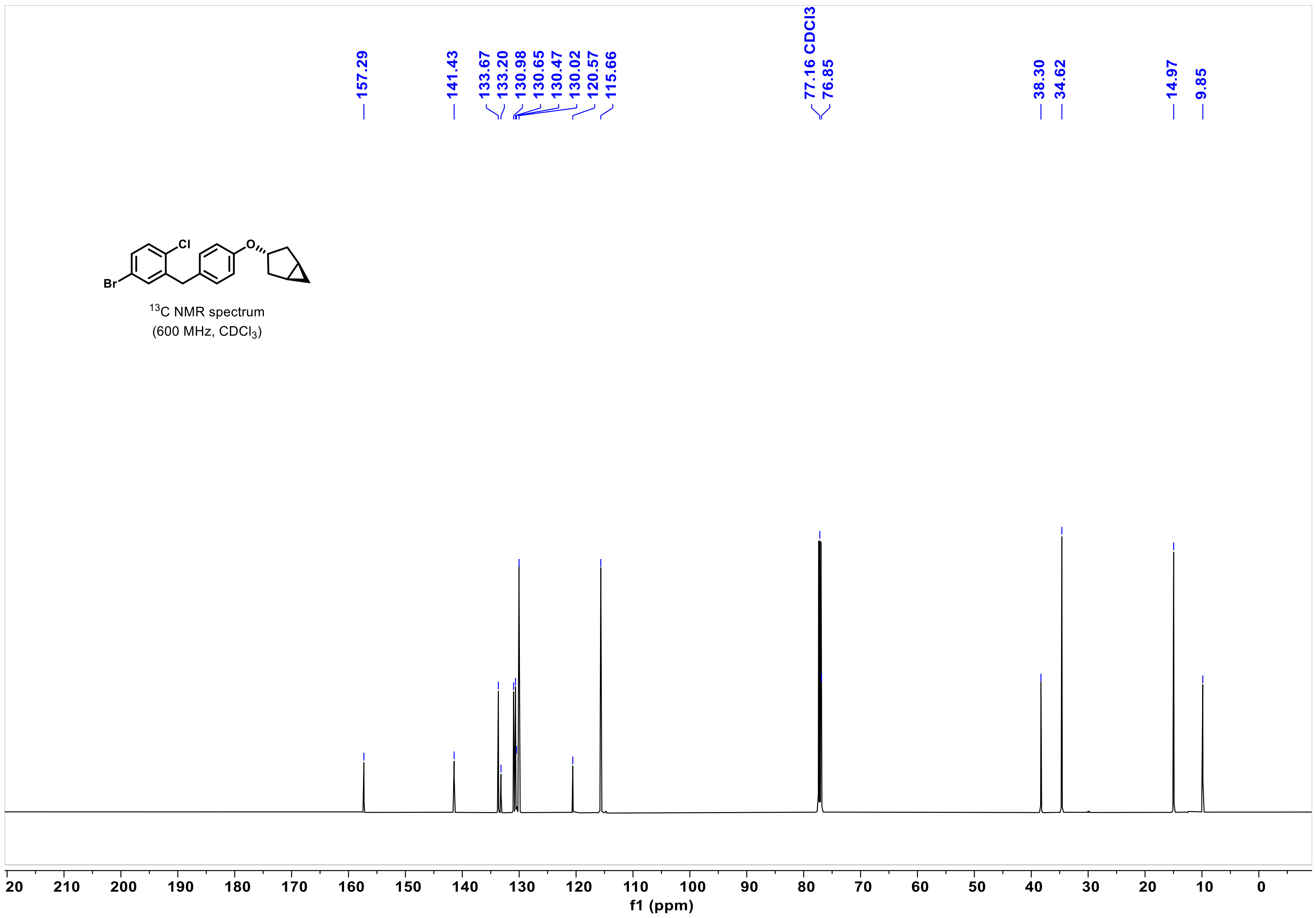

^{13}C NMR of compound **SG11**

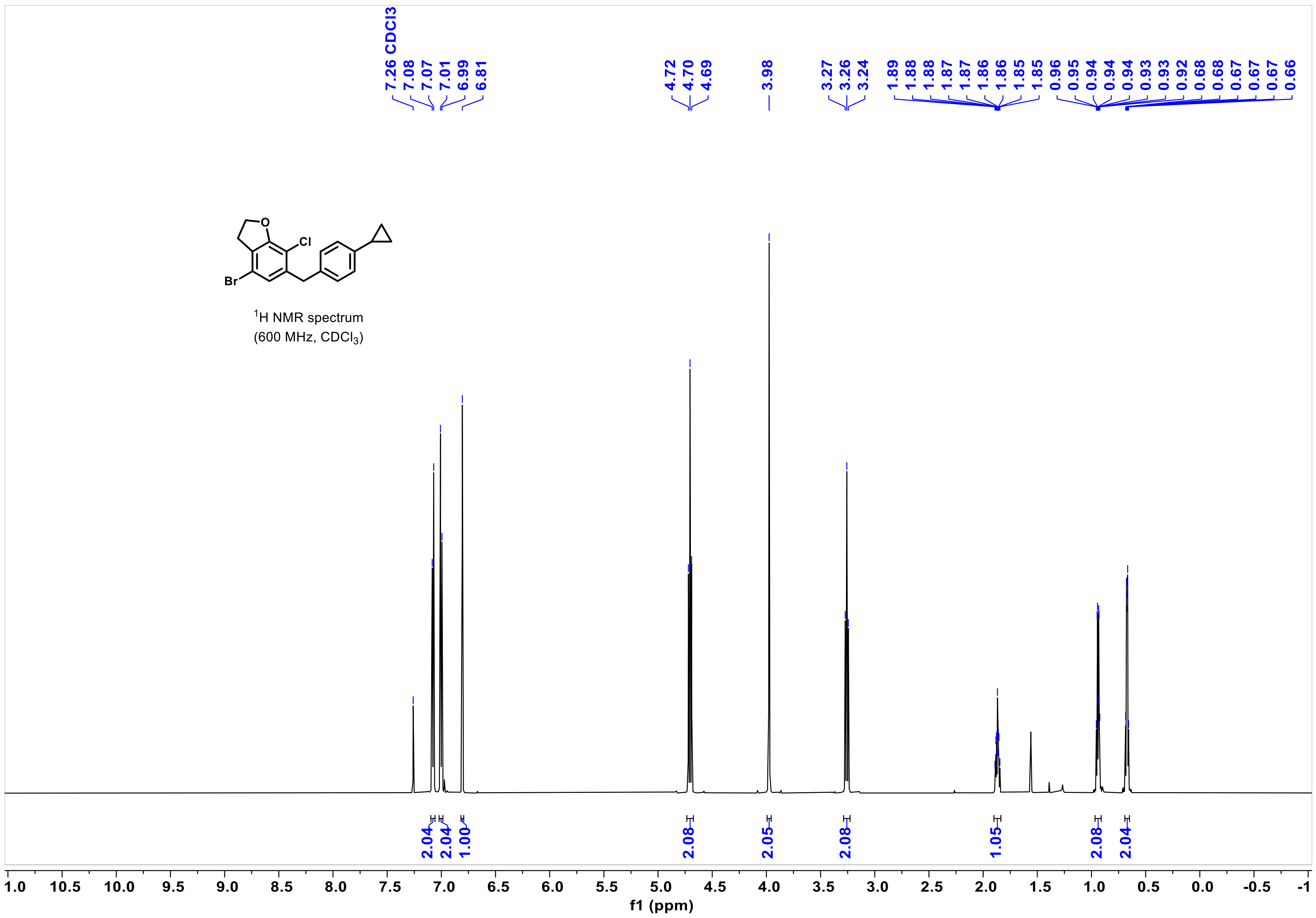

^{1}H NMR of compound **SG9**

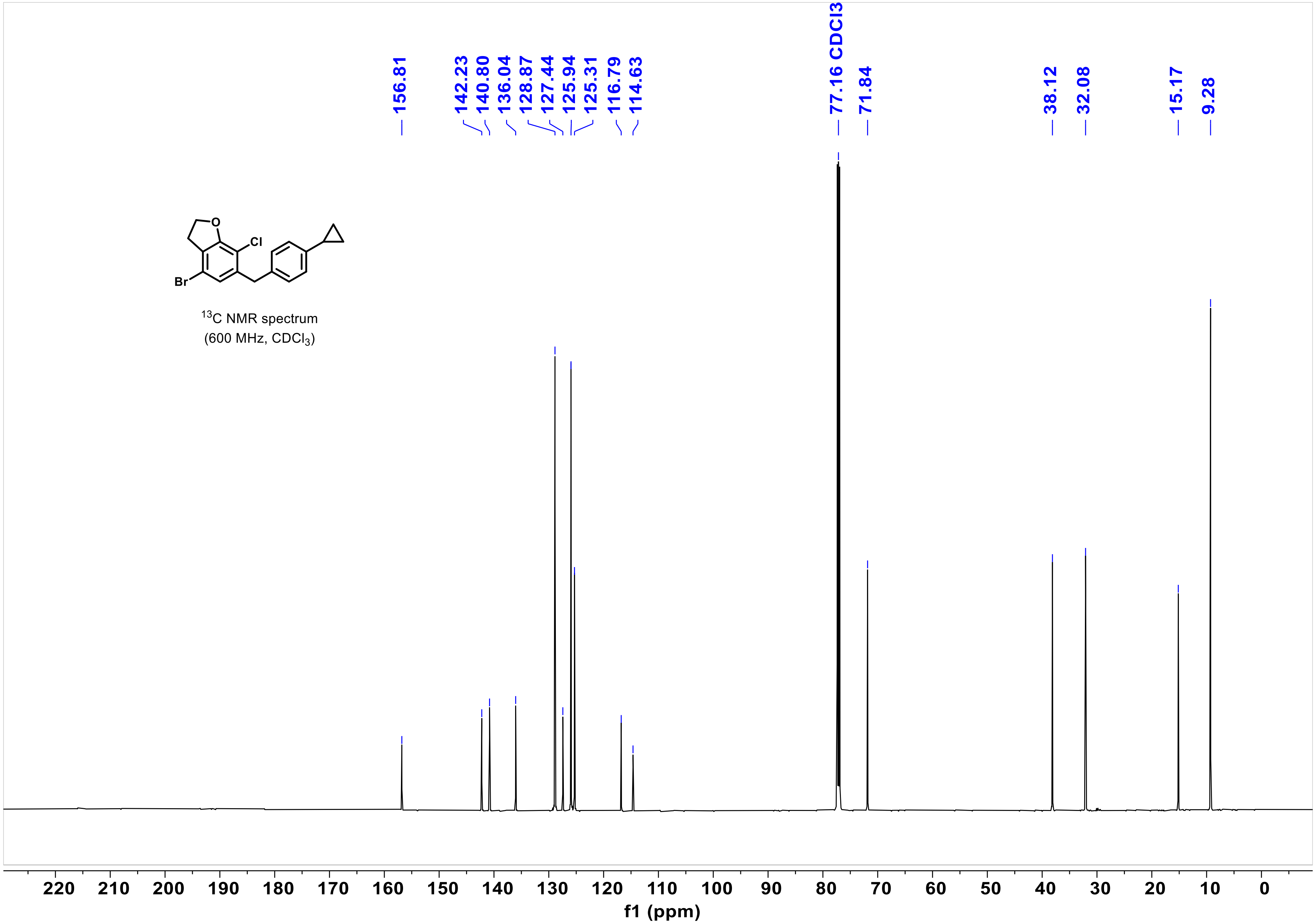

^{13}C NMR of compound **SG9**

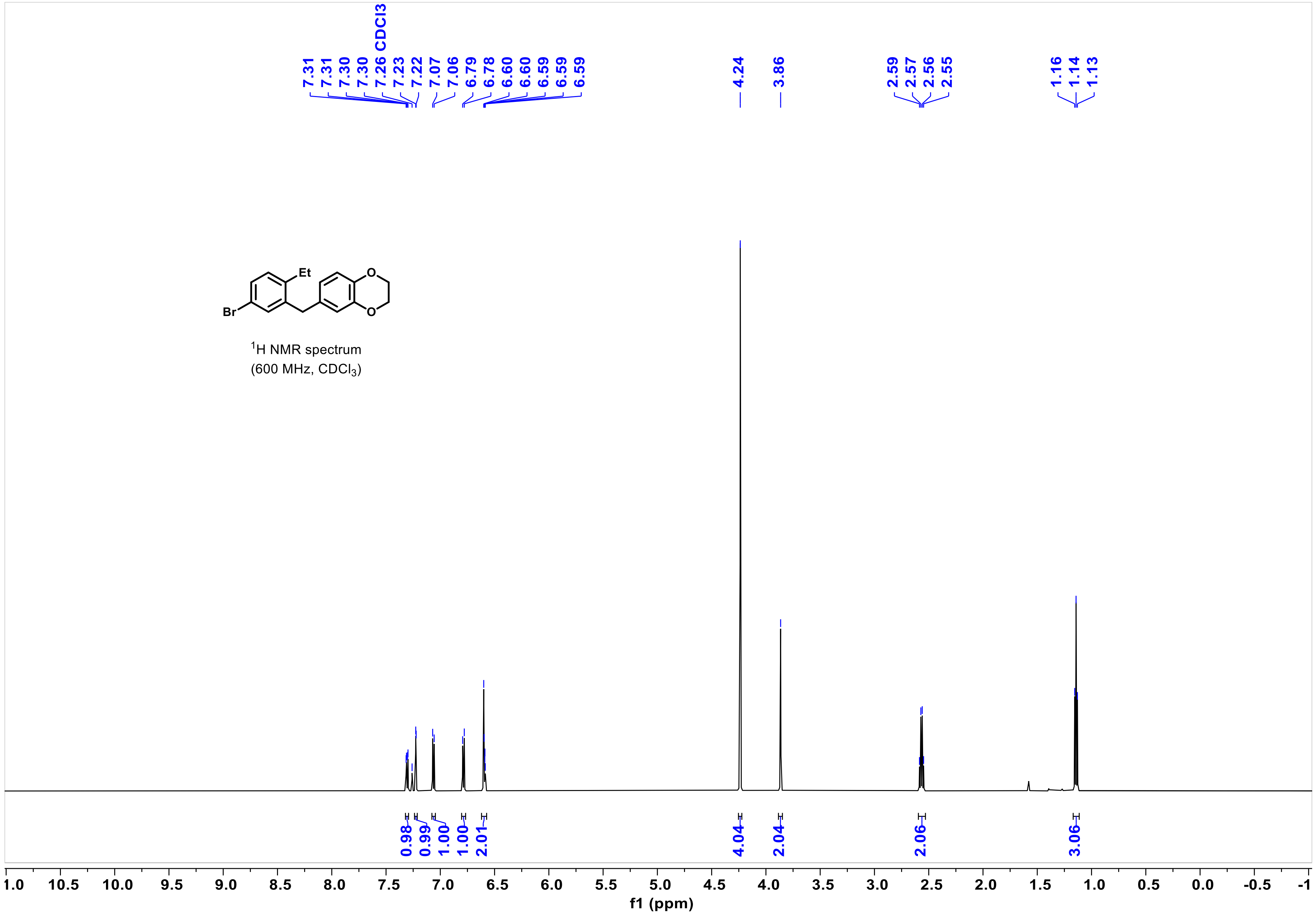

^{1}H NMR of compound **SG10**

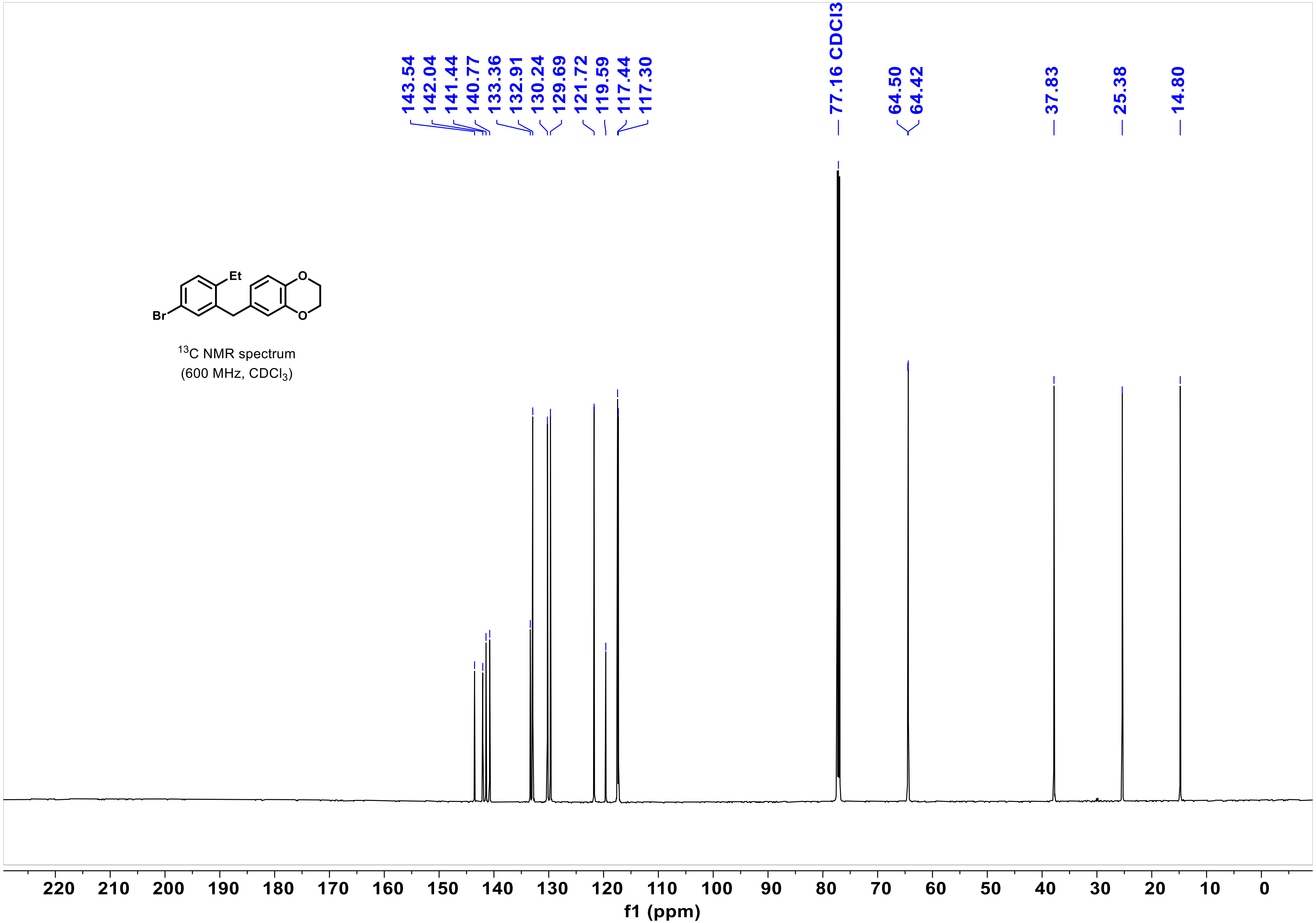

^{13}C NMR of compound **SG10**

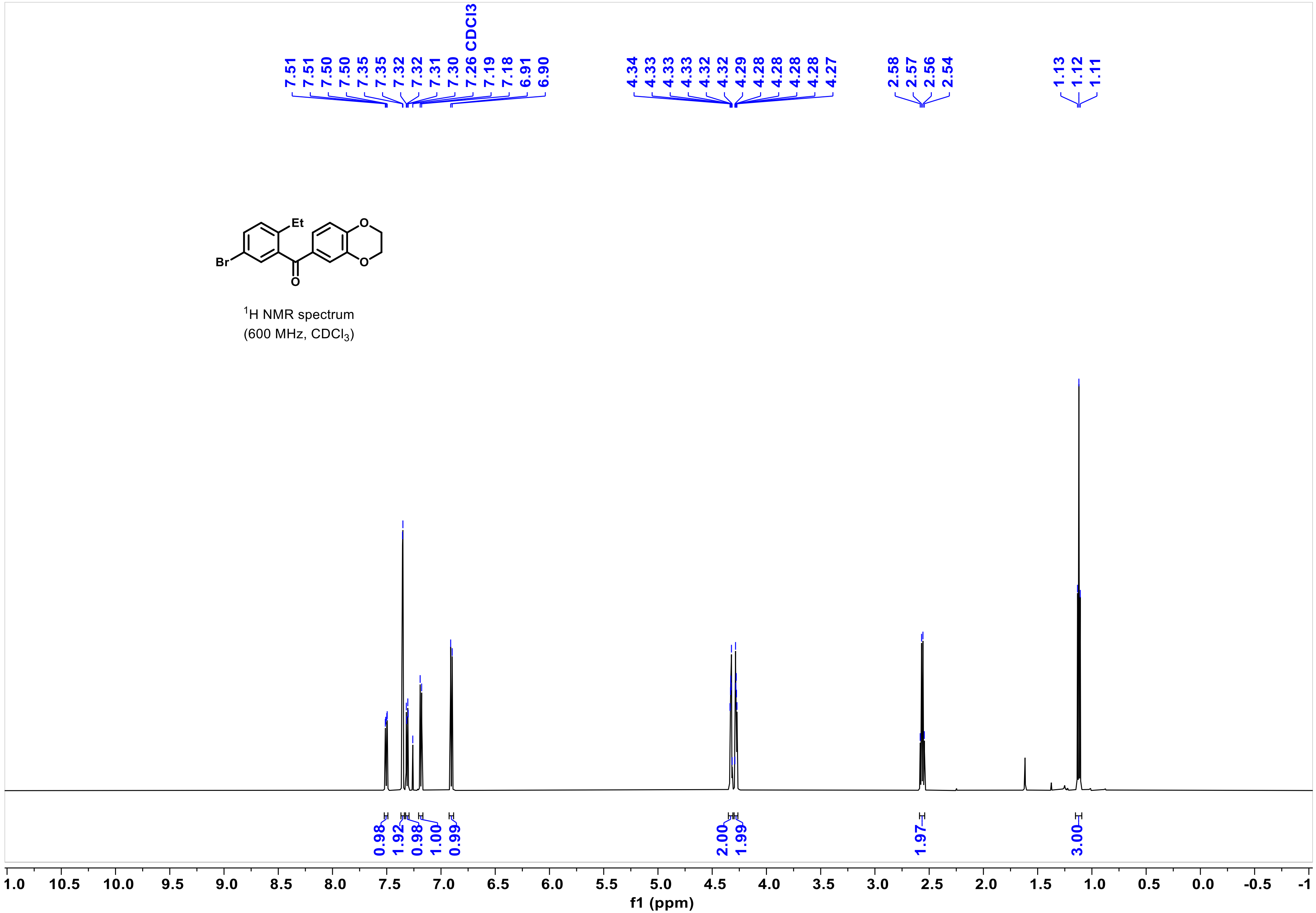

^{1}H NMR of compound **SG25**

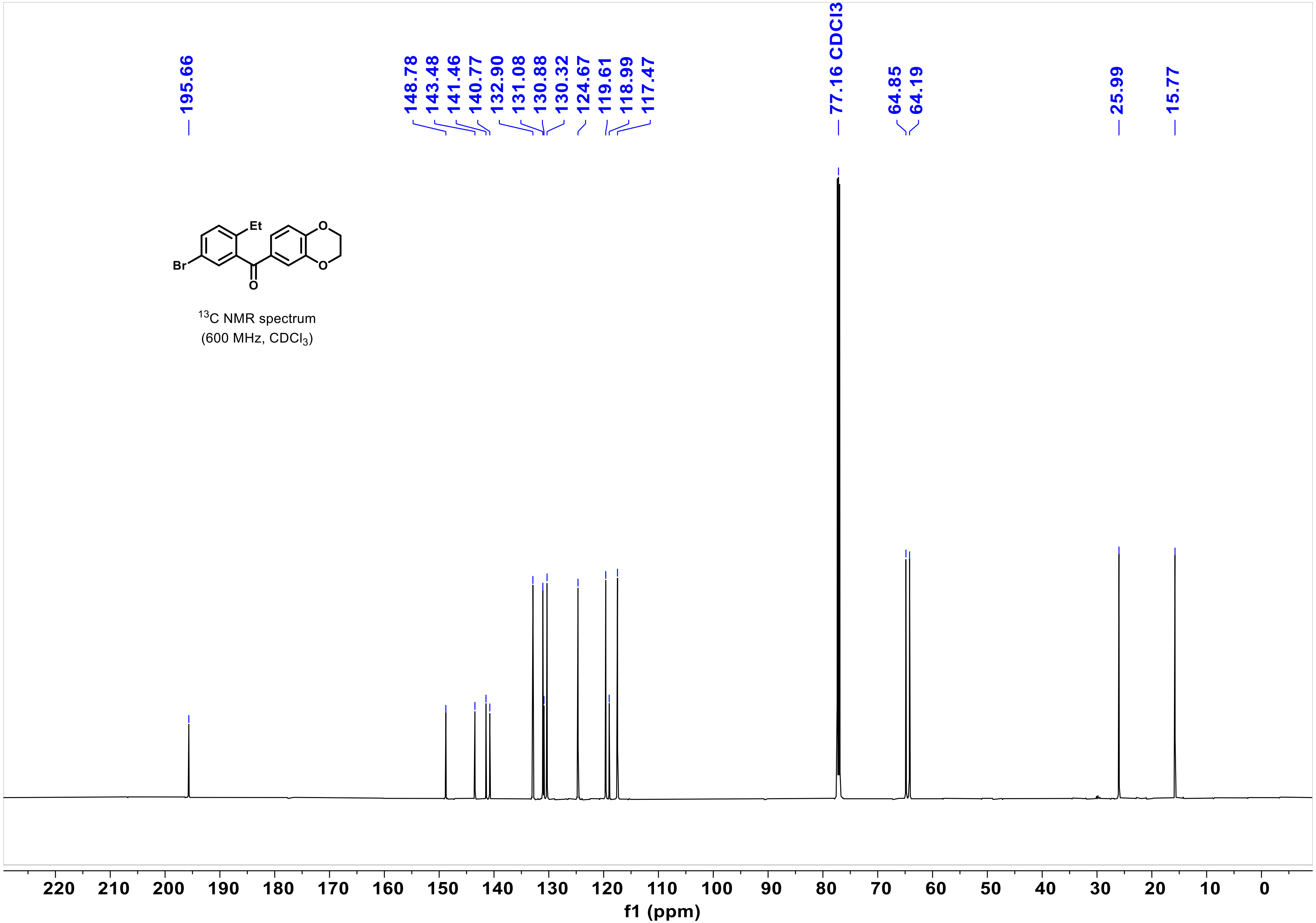

^{13}C NMR of compound **SG25**

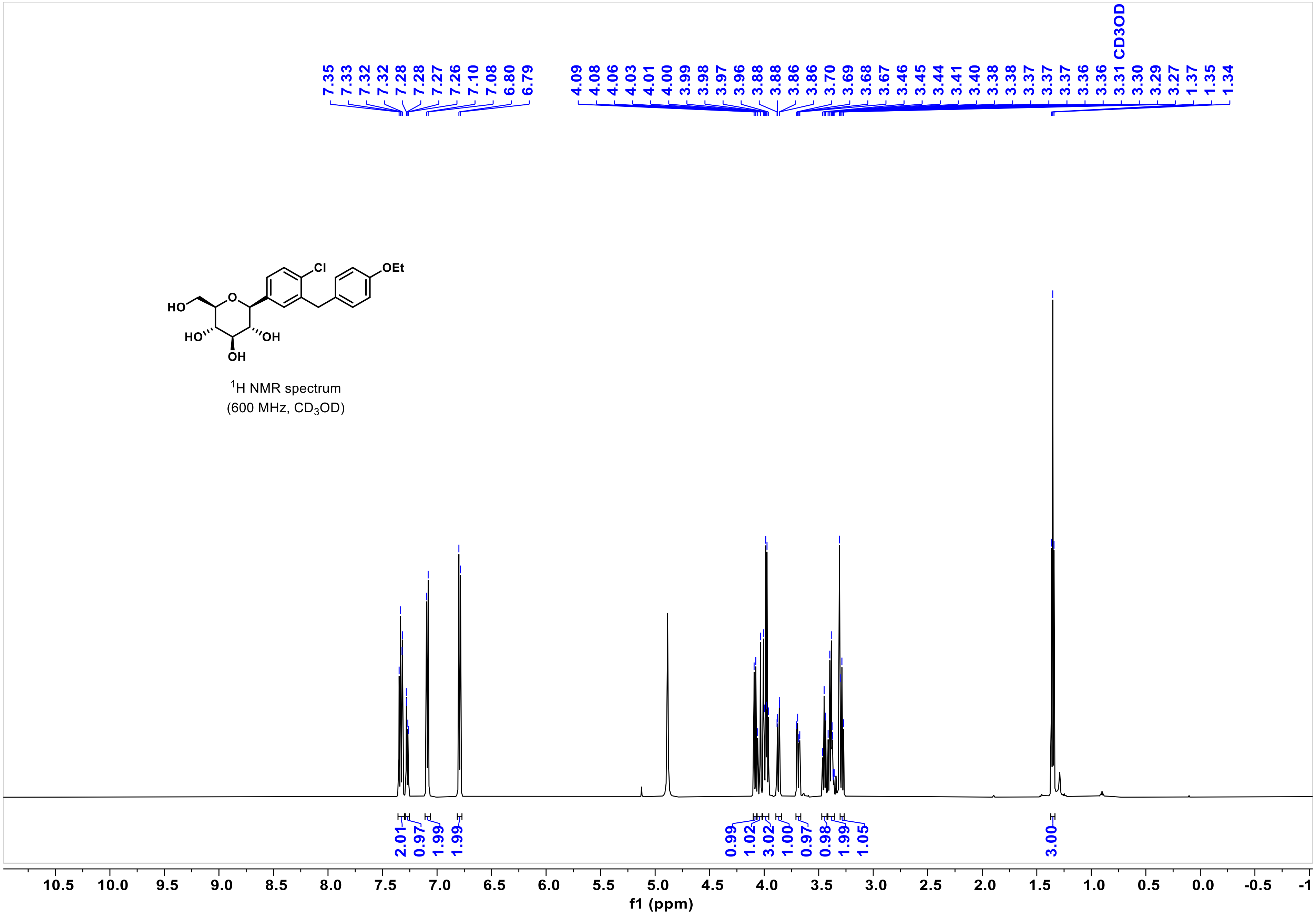

^{1}H NMR of **Dapagliflozin (1)**

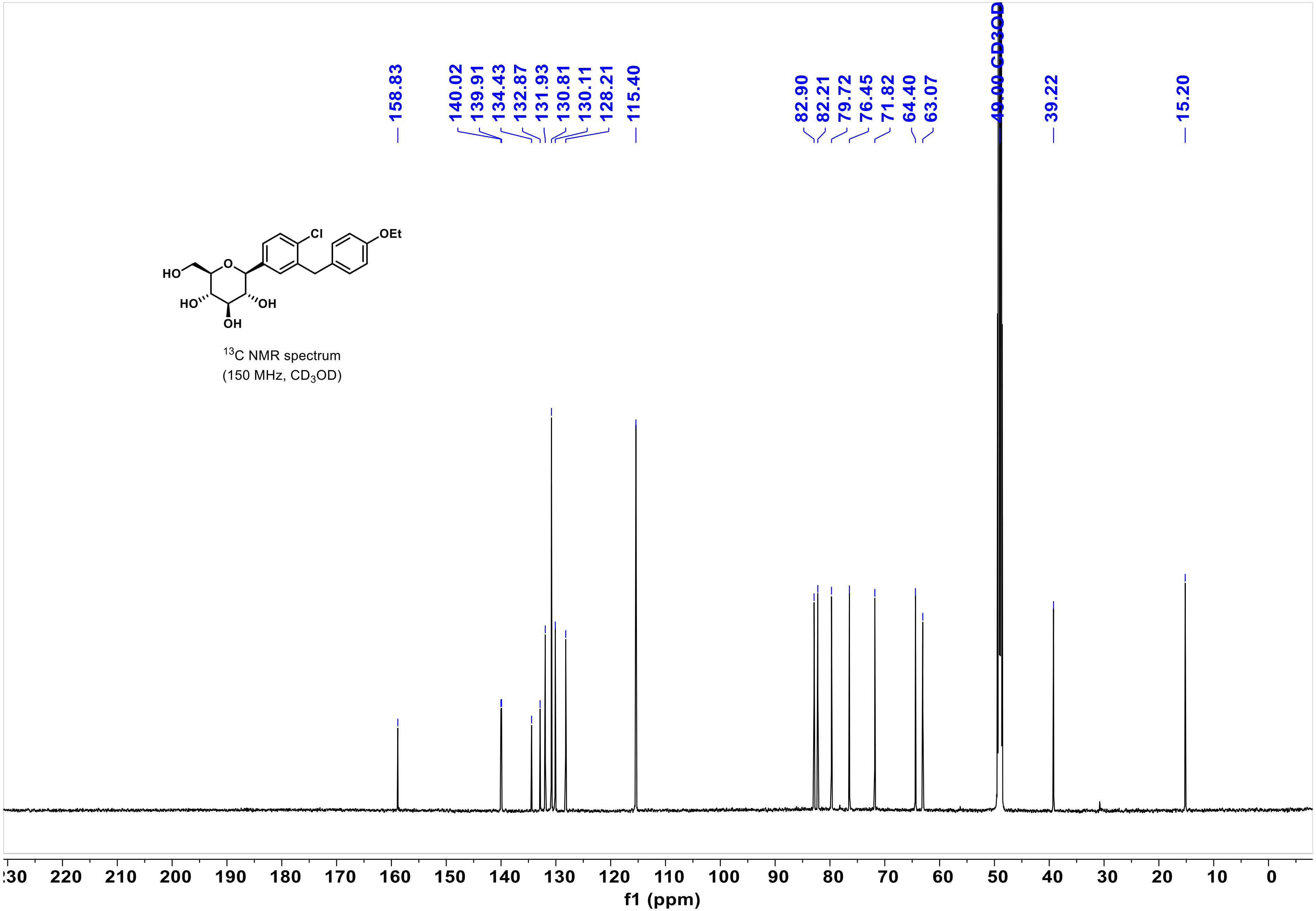

^{13}C NMR of **Dapagliflozin (1)**

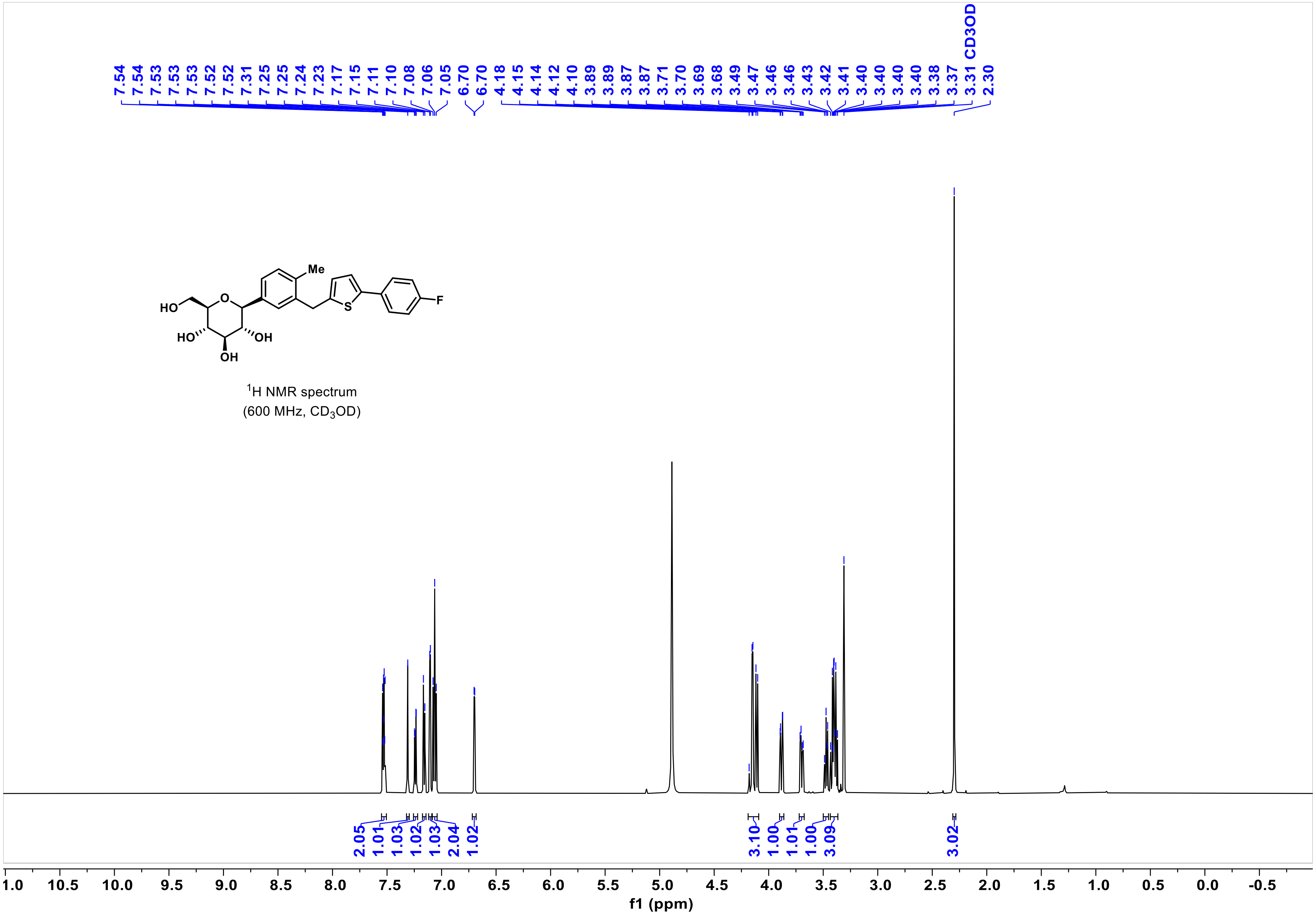

^{1}H NMR of **Canagliflozin (30)**

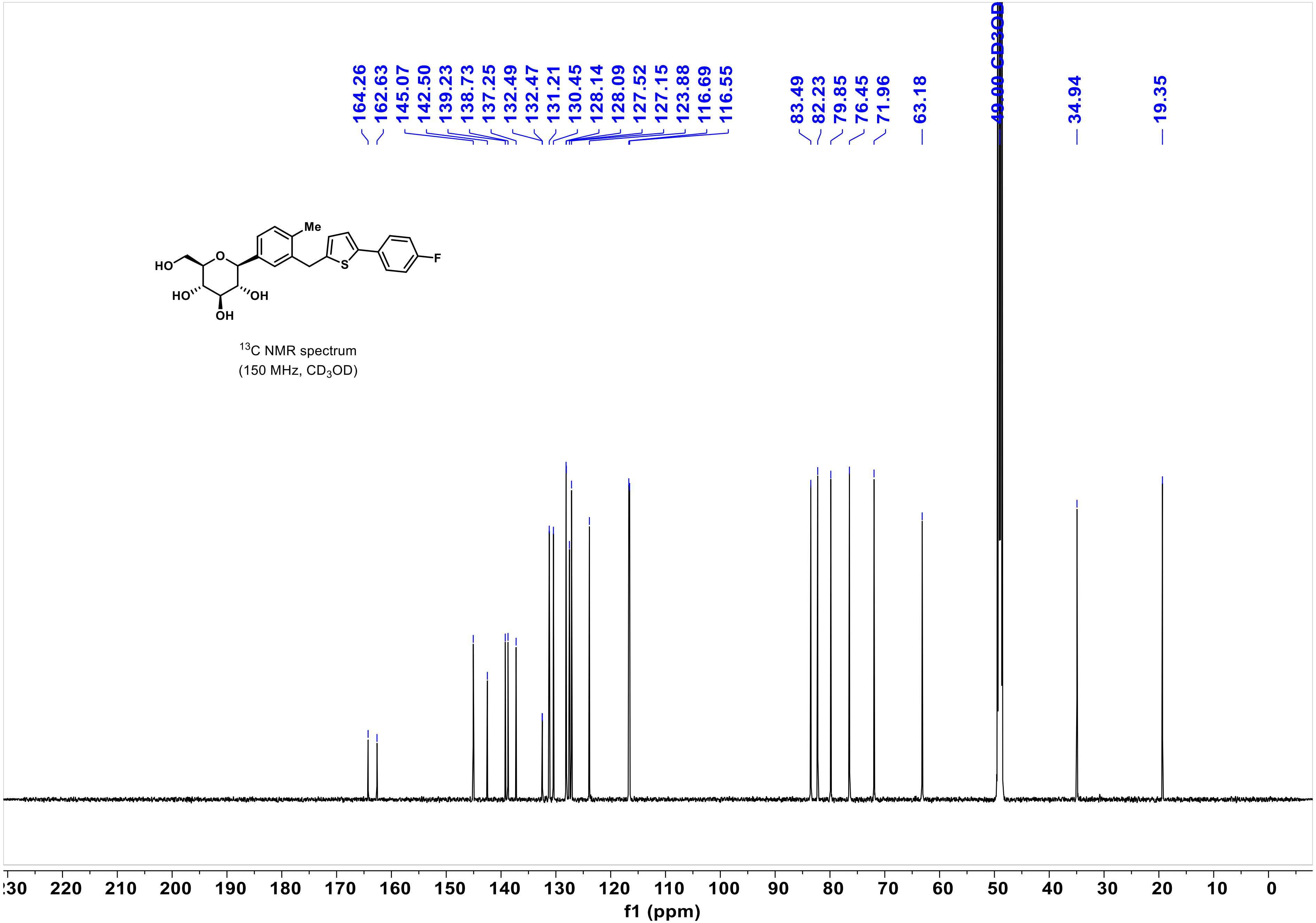

^{13}C NMR of **Canagliflozin (30)**

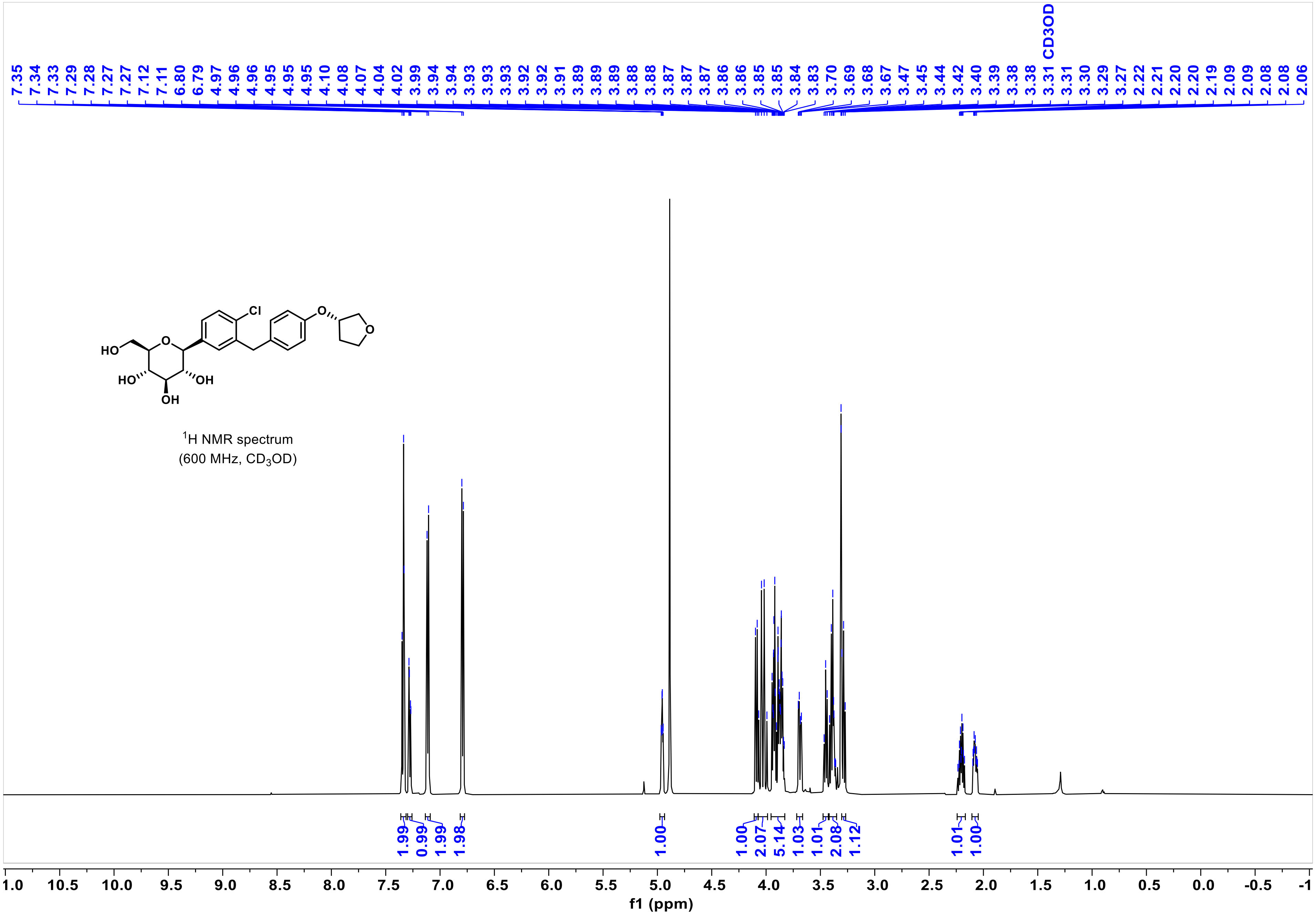

^{1}H NMR of **Empagliflozin (2)**

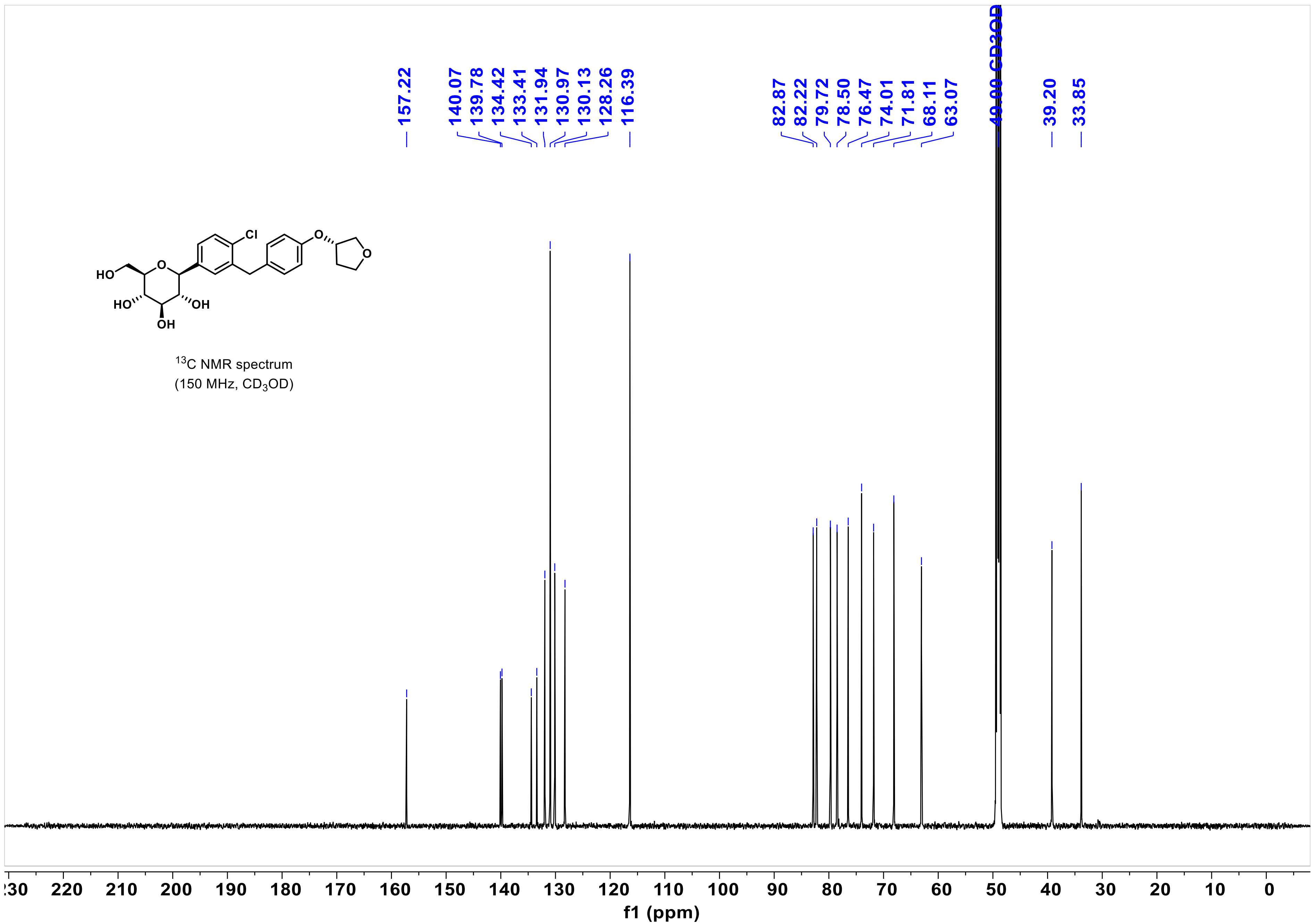

^{13}C NMR of **Empagliflozin (2)**

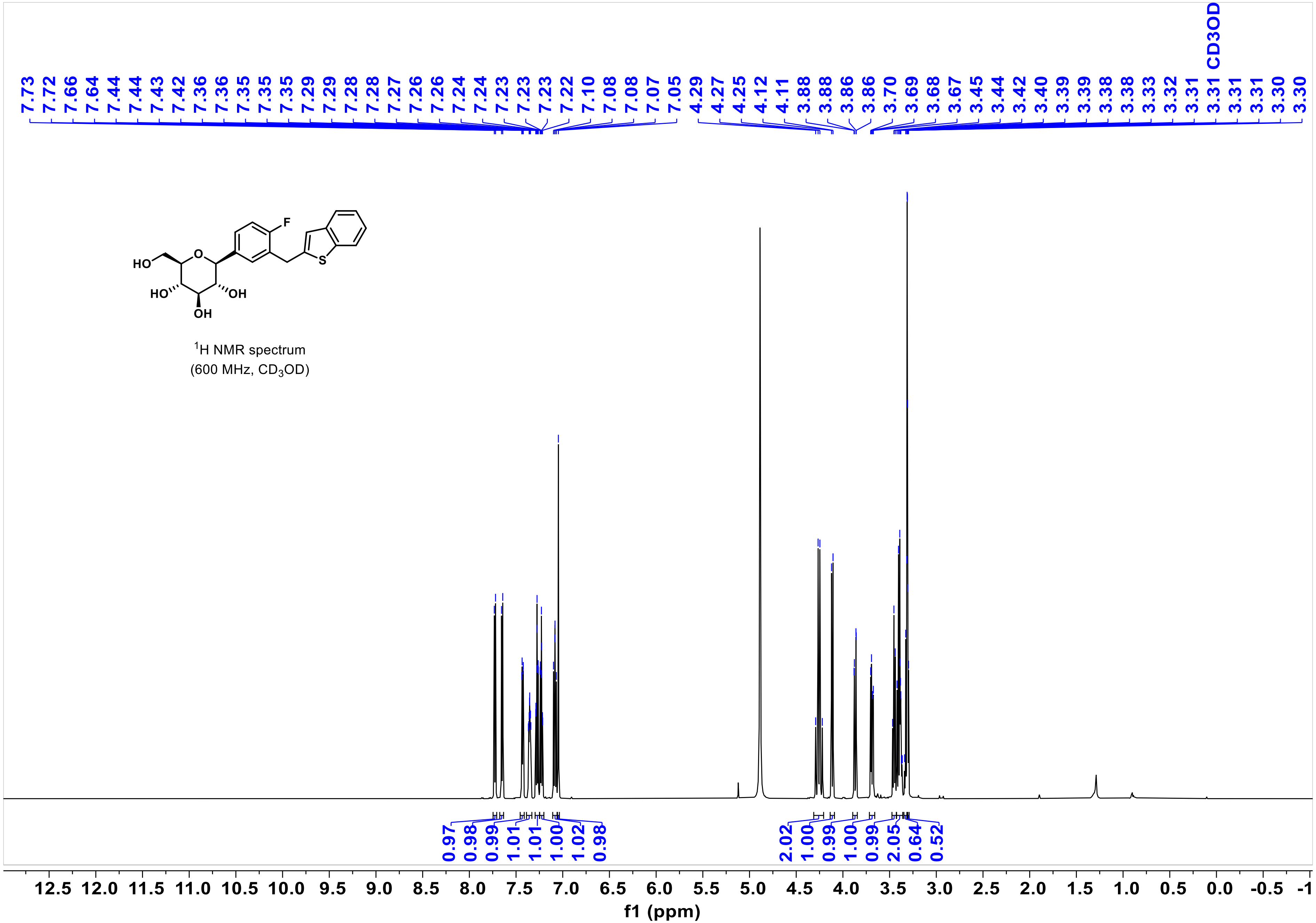

^{1}H NMR of **Ipragliflozin (31)**

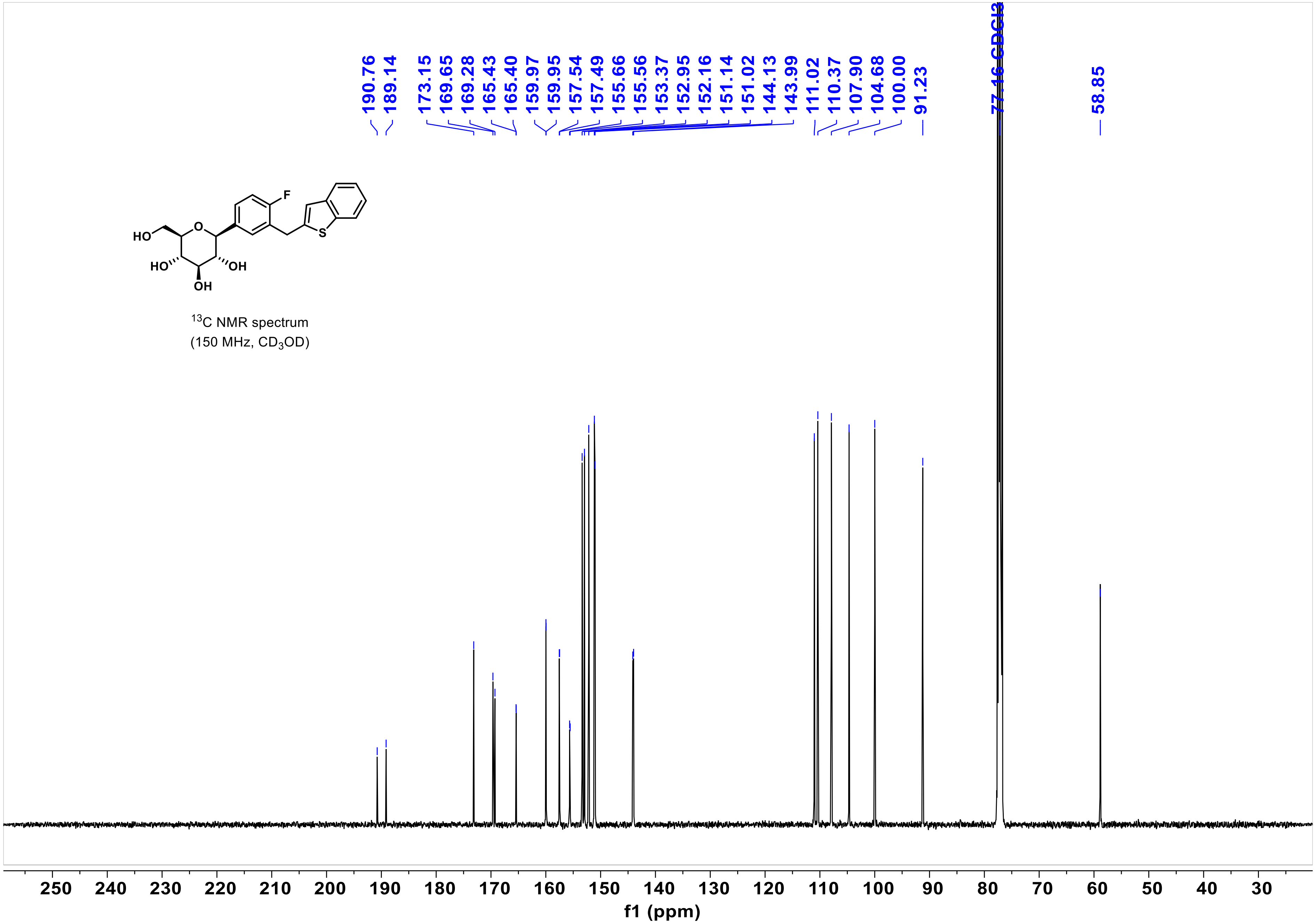

^{13}C NMR of **Ipragliflozin (31)**

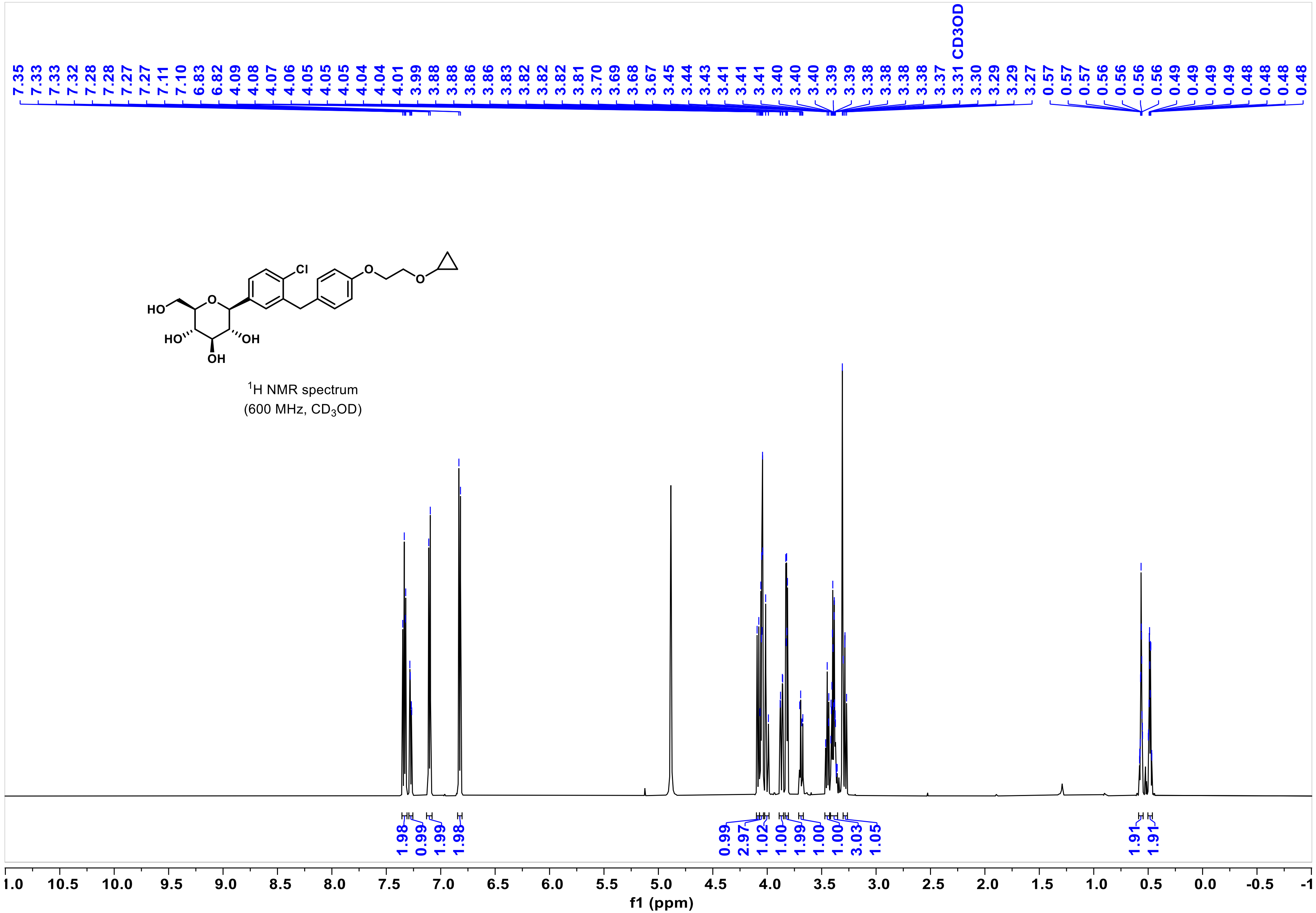

^{1}H NMR of **Bexagliflozin (32)**

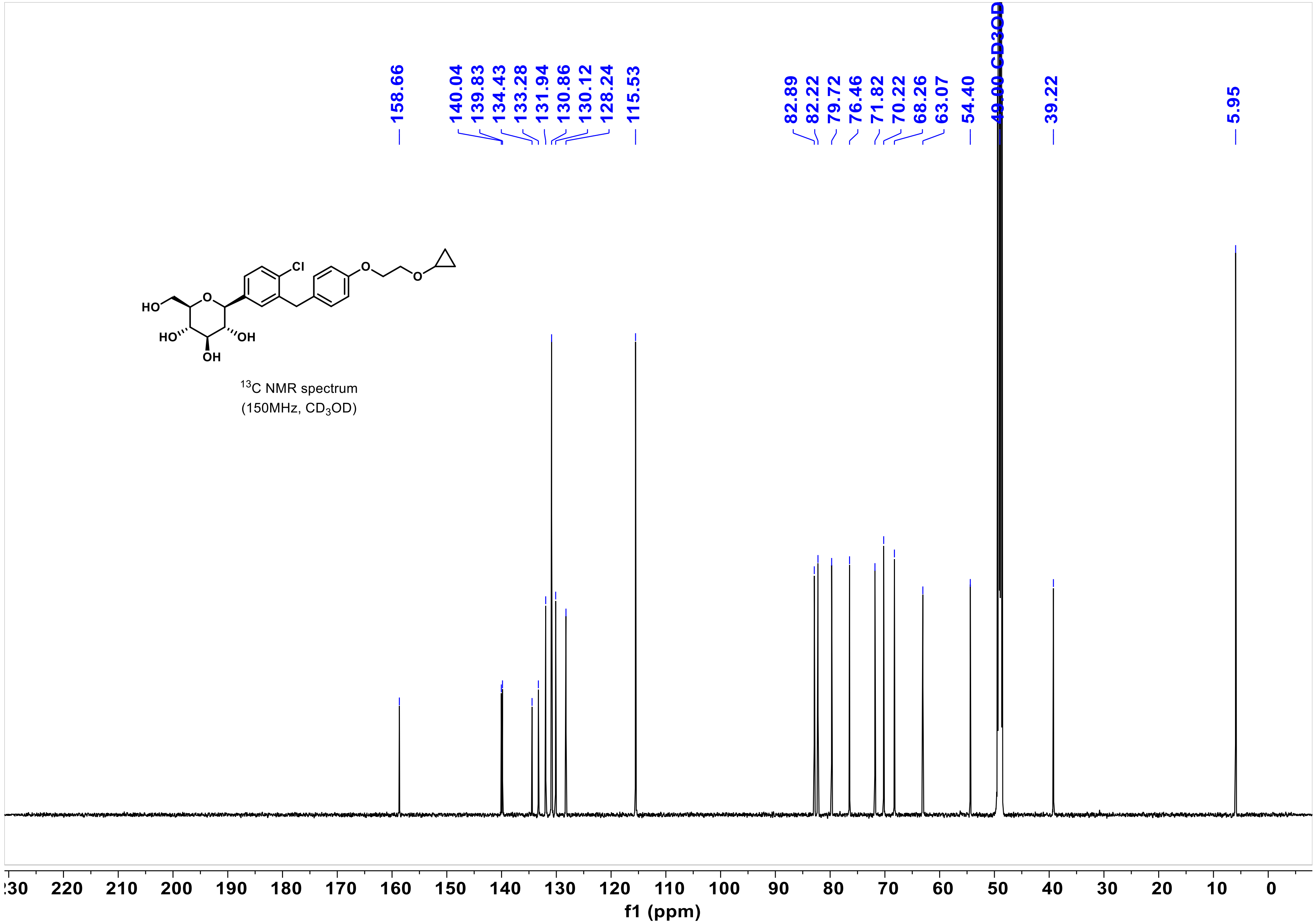

^{13}C NMR of **Bexagliflozin (32)**

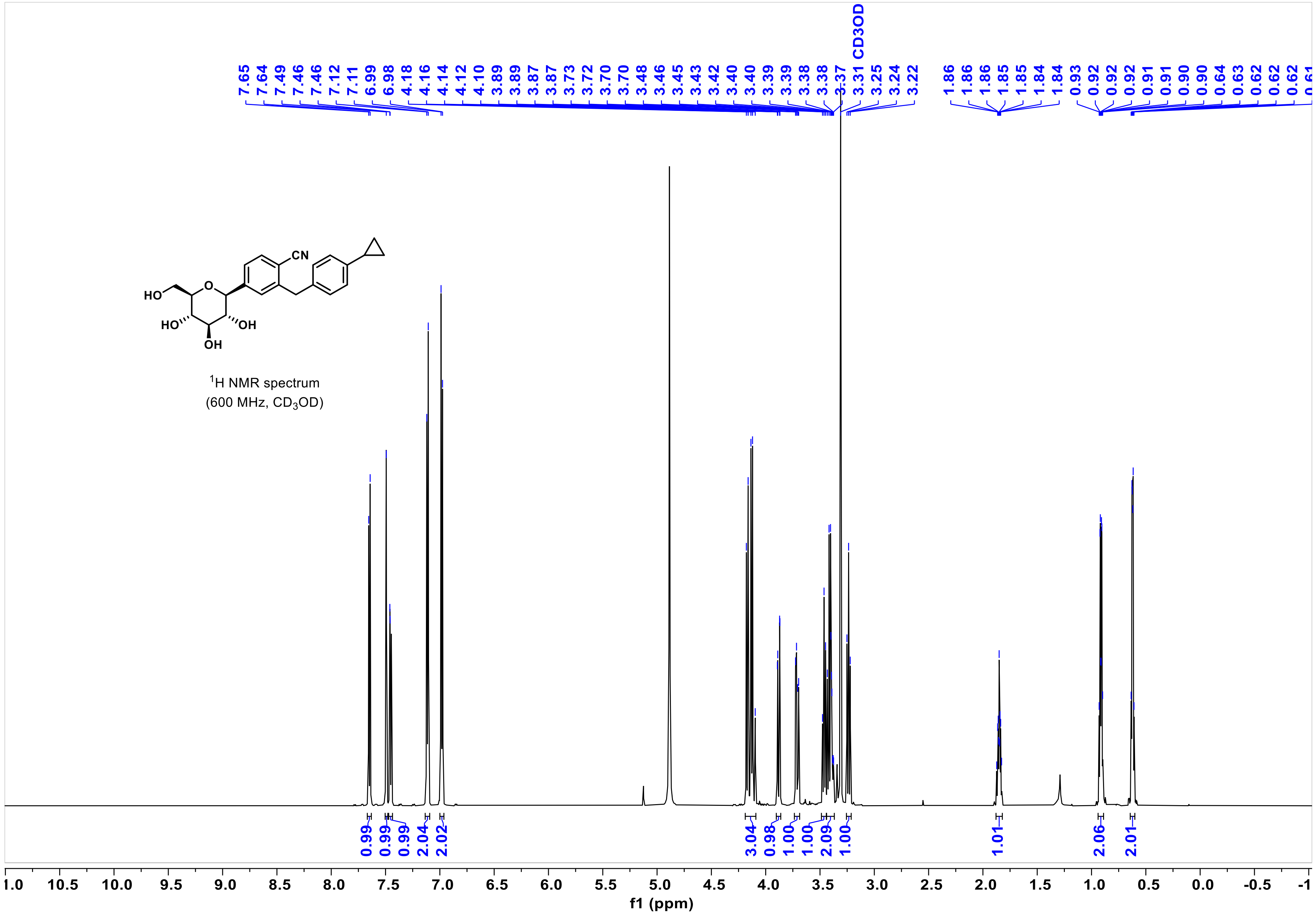

^{1}H NMR of **Velagliflozin (33)**

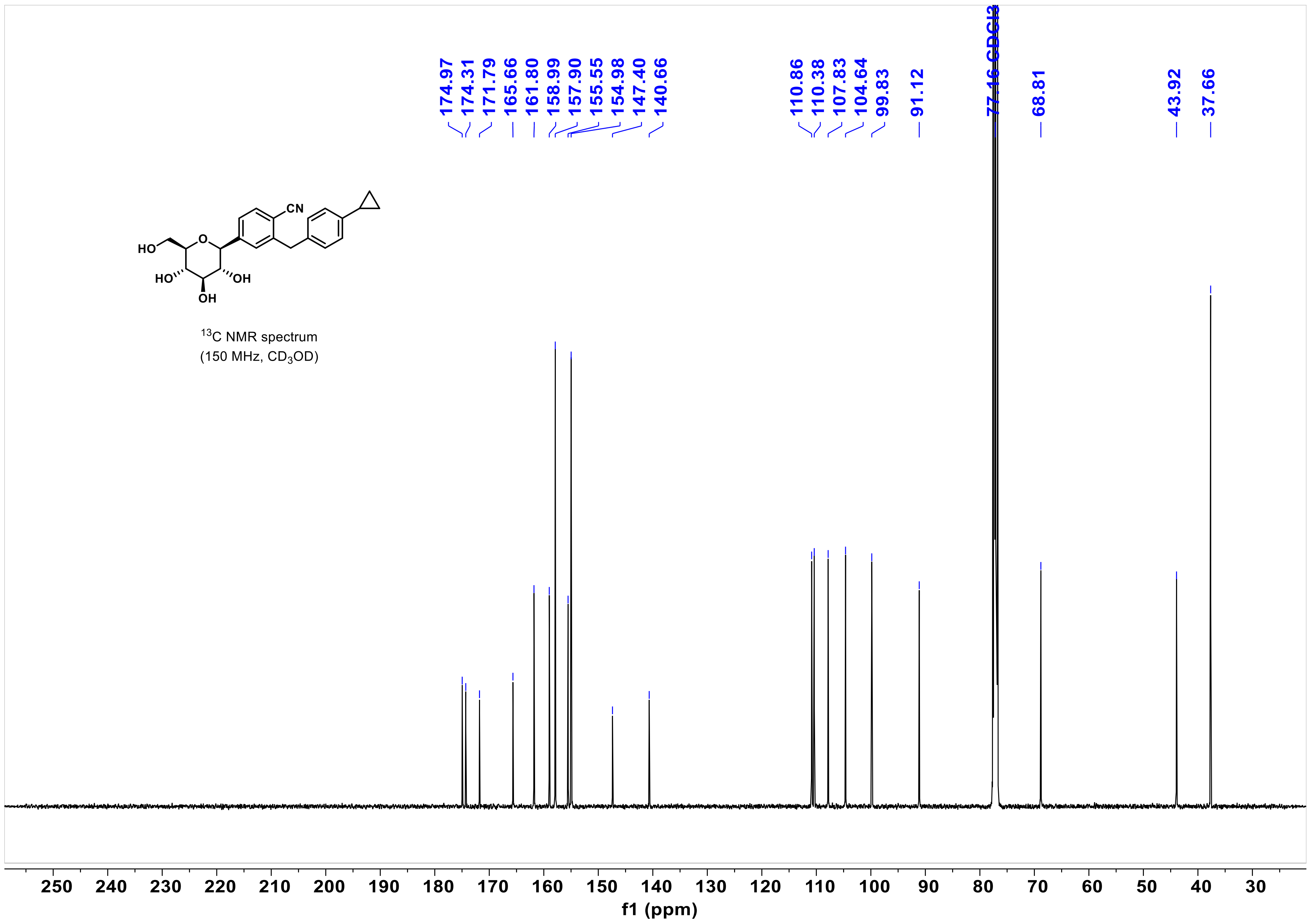

^{13}C NMR of **Velagliflozin (33)**

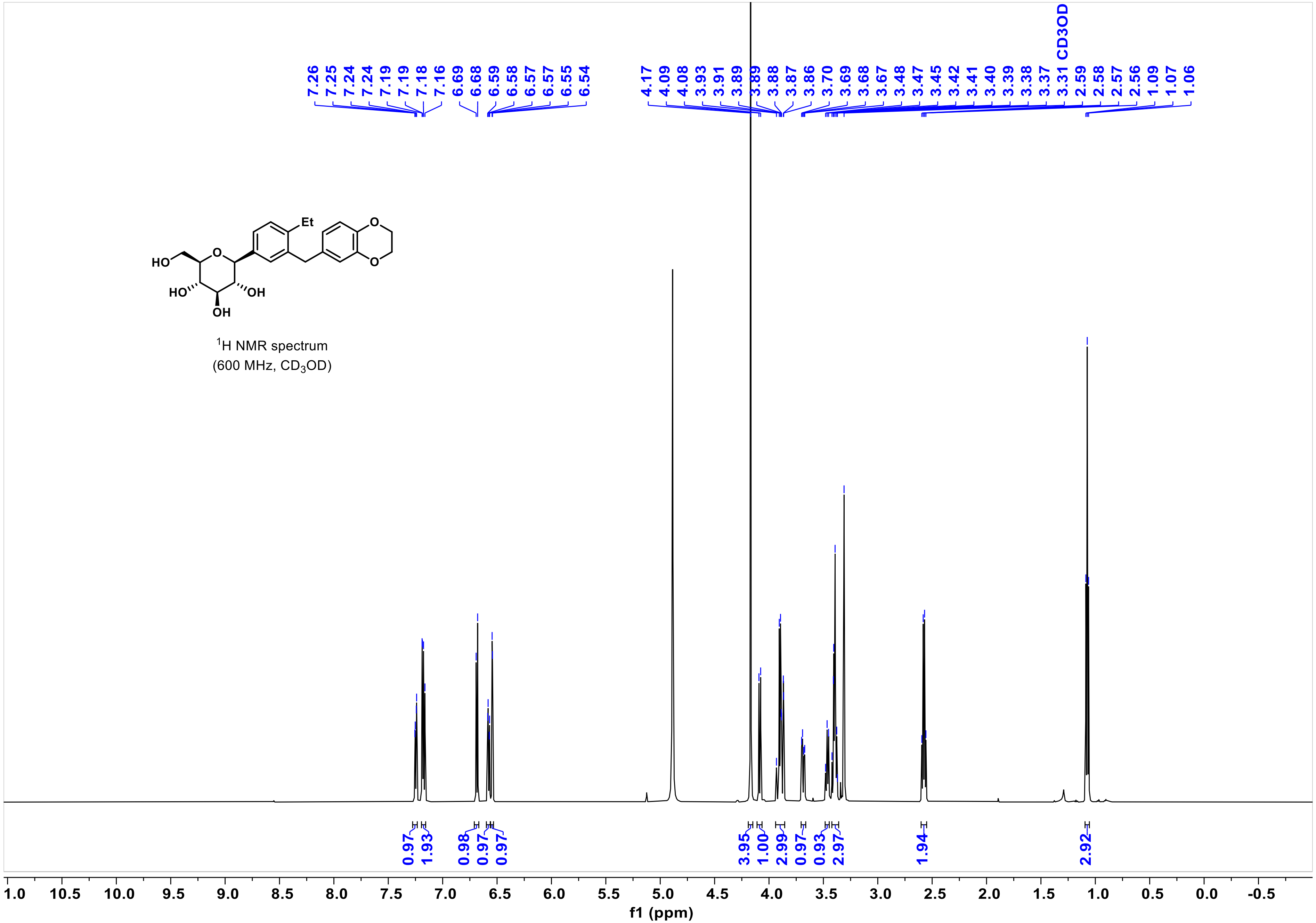

^{1}H NMR of **Licogliflozin (34)**

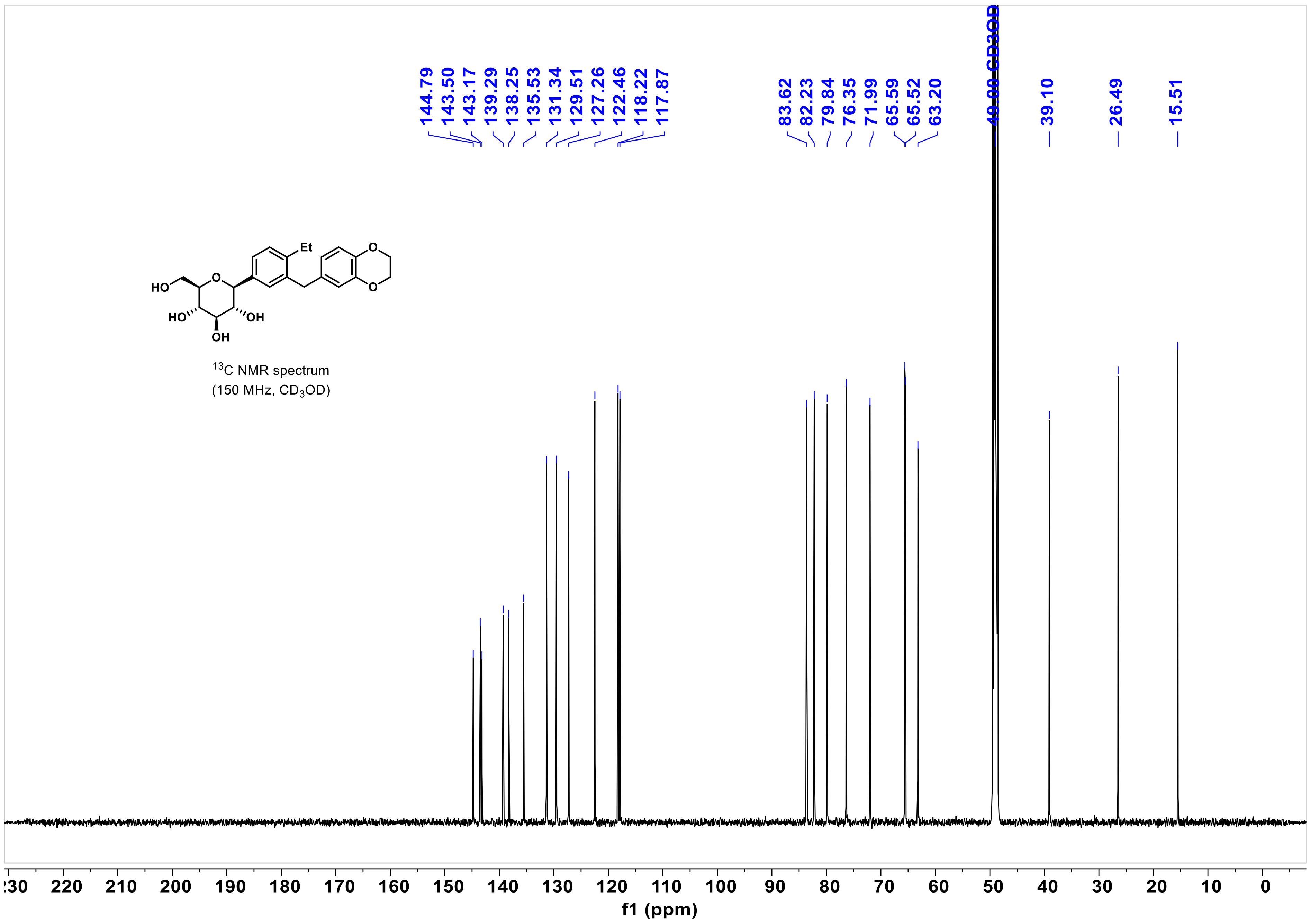

^{13}C NMR of **Licogliflozin (34)**

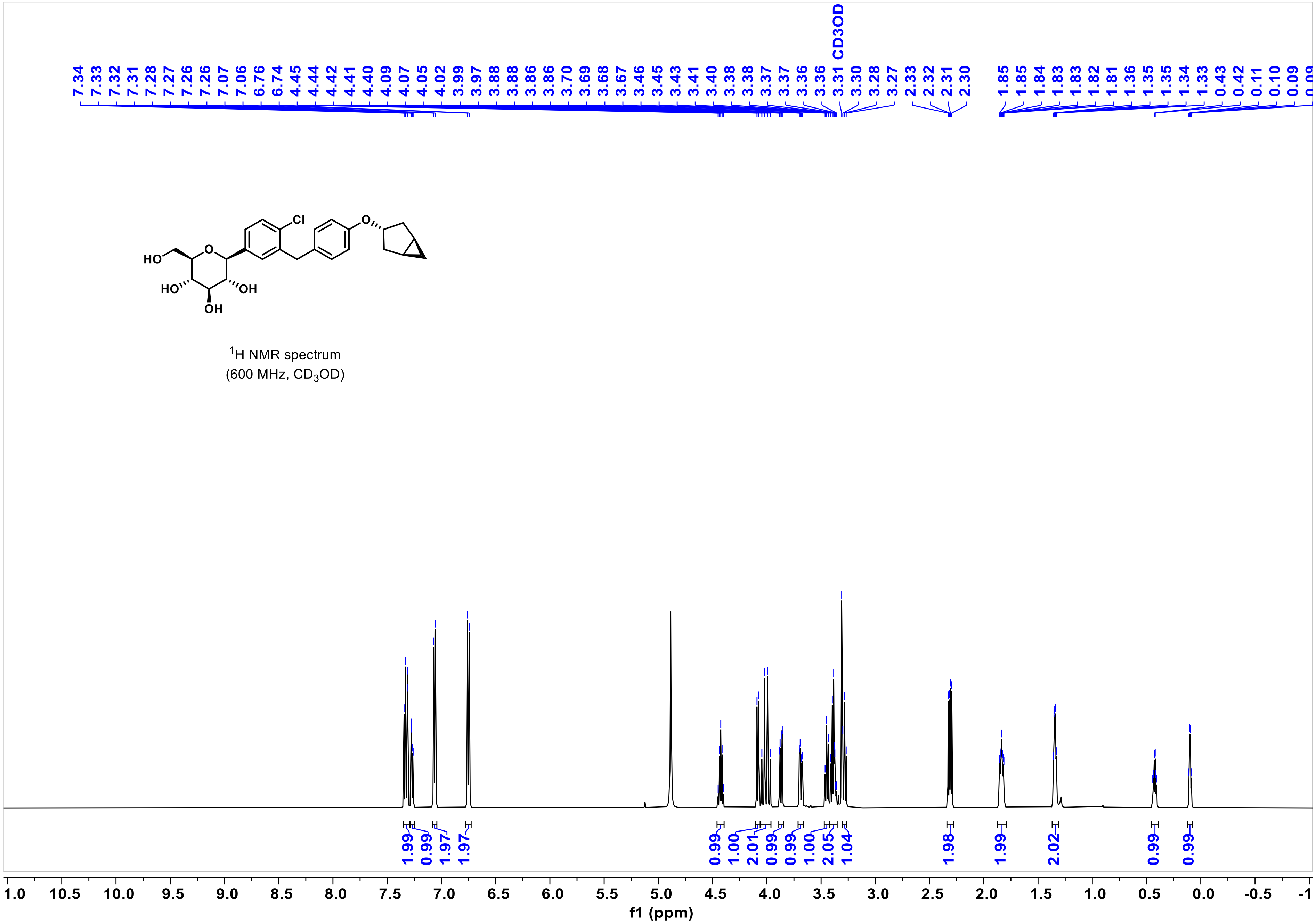

^{1}H NMR of **Janagliflozin (35)**

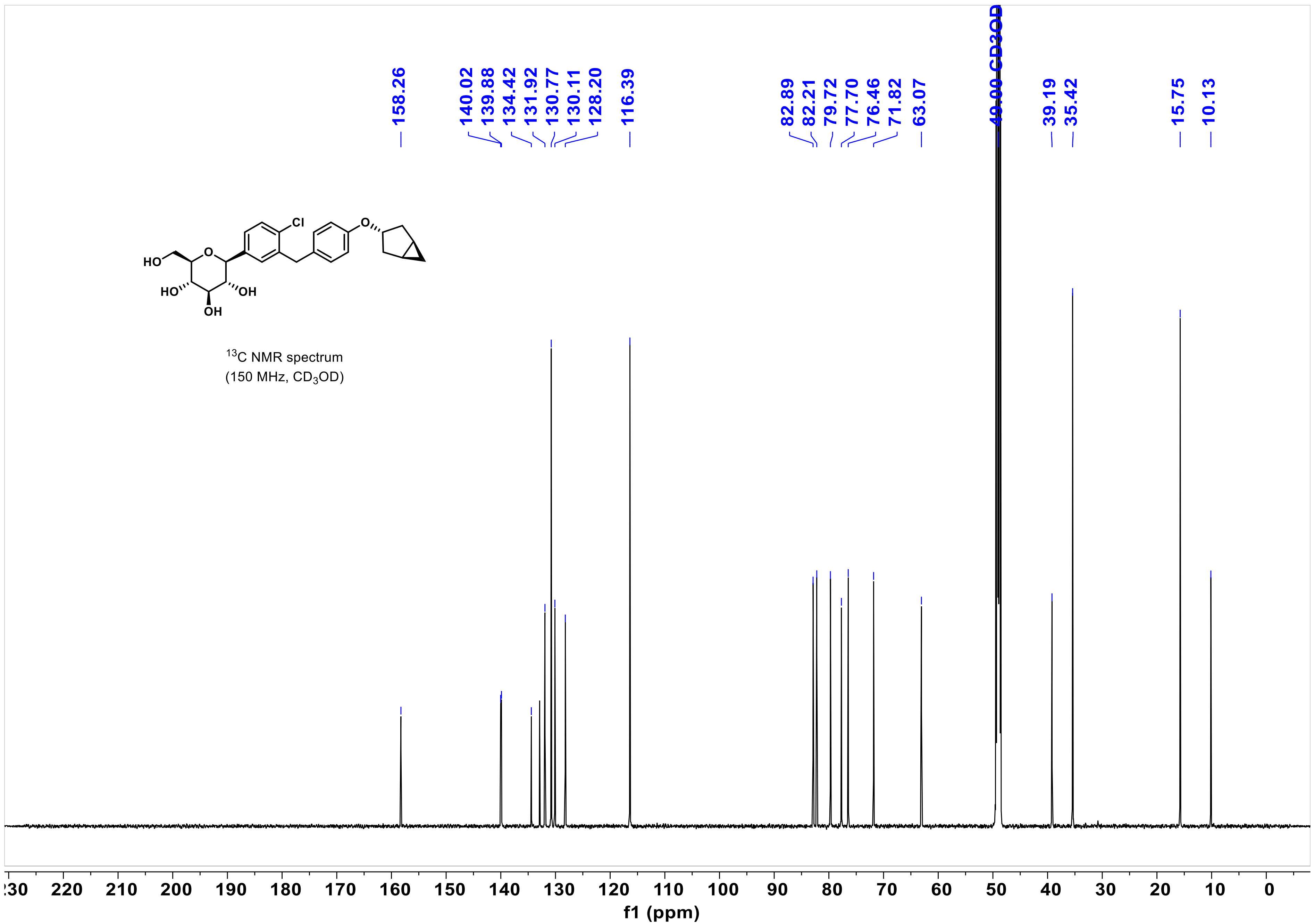

^{13}C NMR of **Janagliflozin (35)**

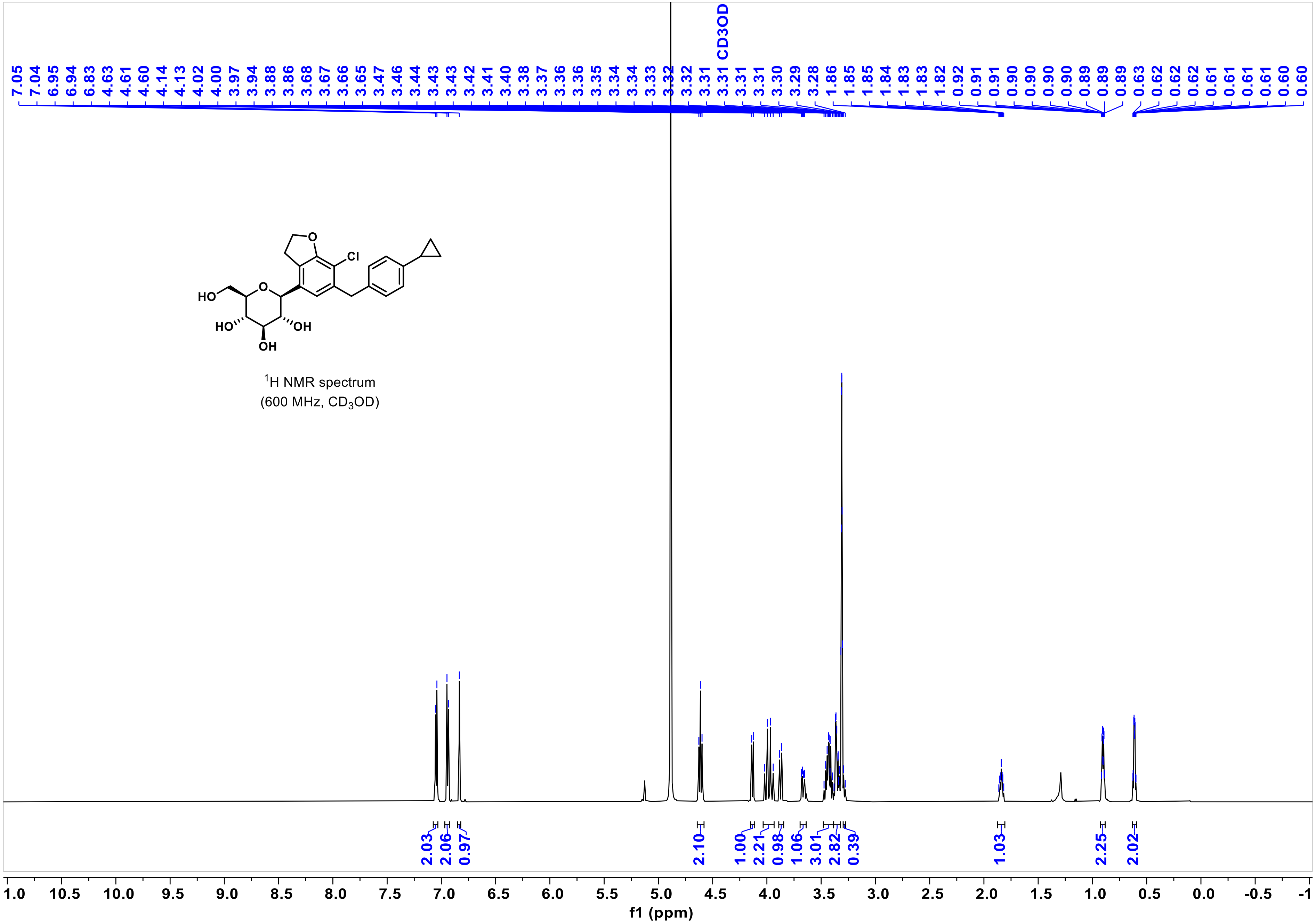

^{1}H NMR of **Enavogliflozin (36)**

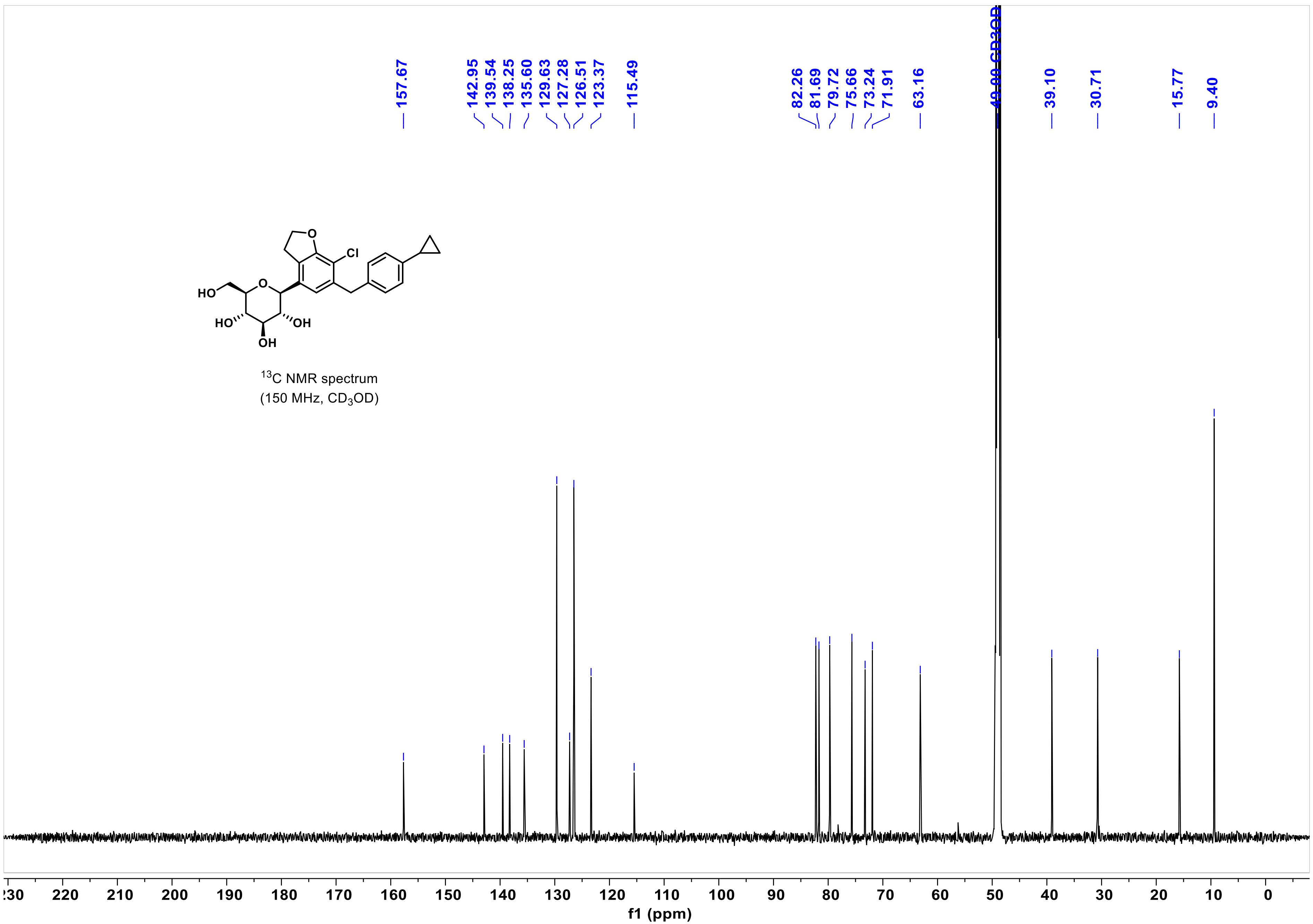

^{13}C NMR of **Enavogliflozin (36)**

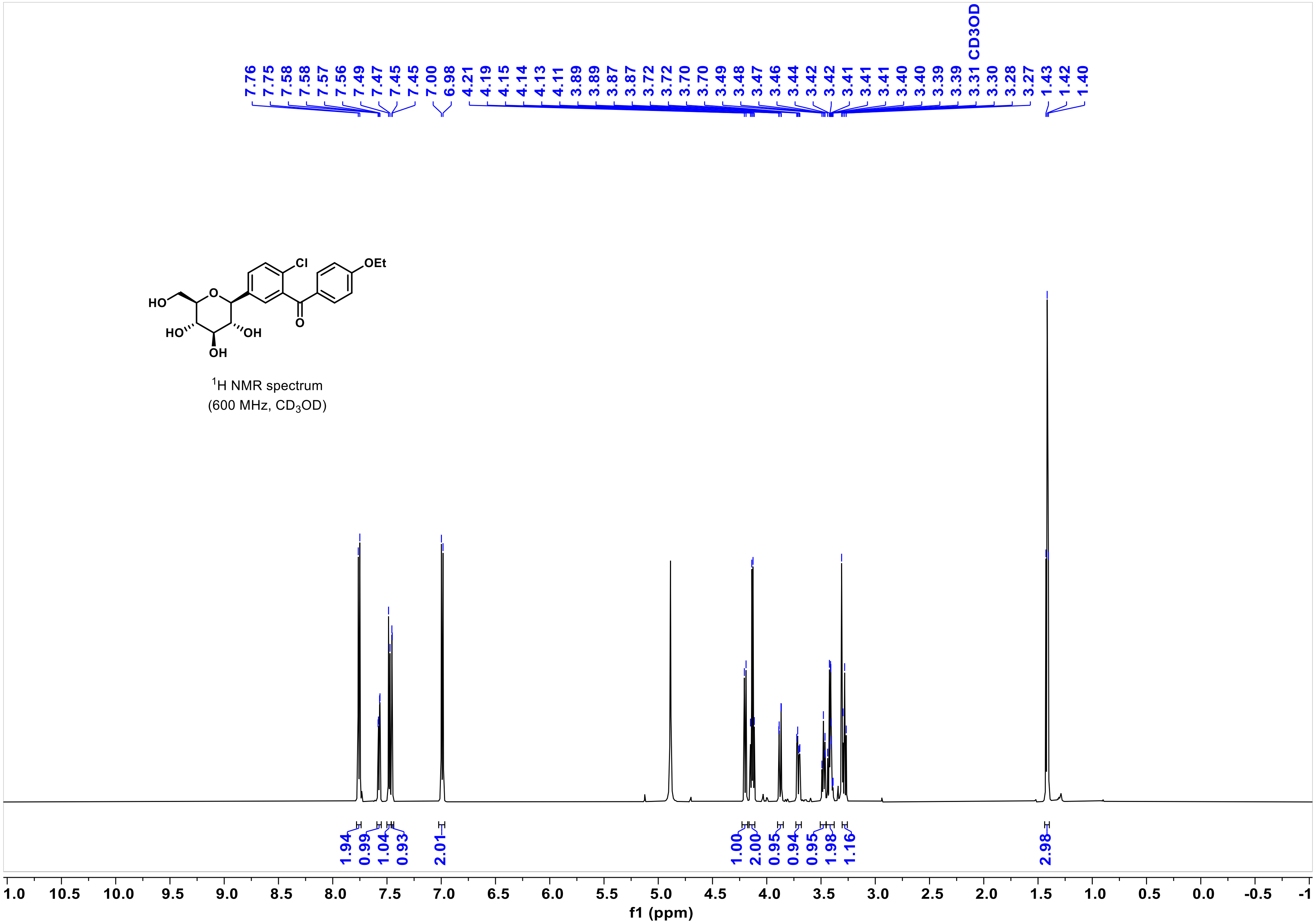

^{1}H NMR of **Keto-Dapagliflozin (37)**

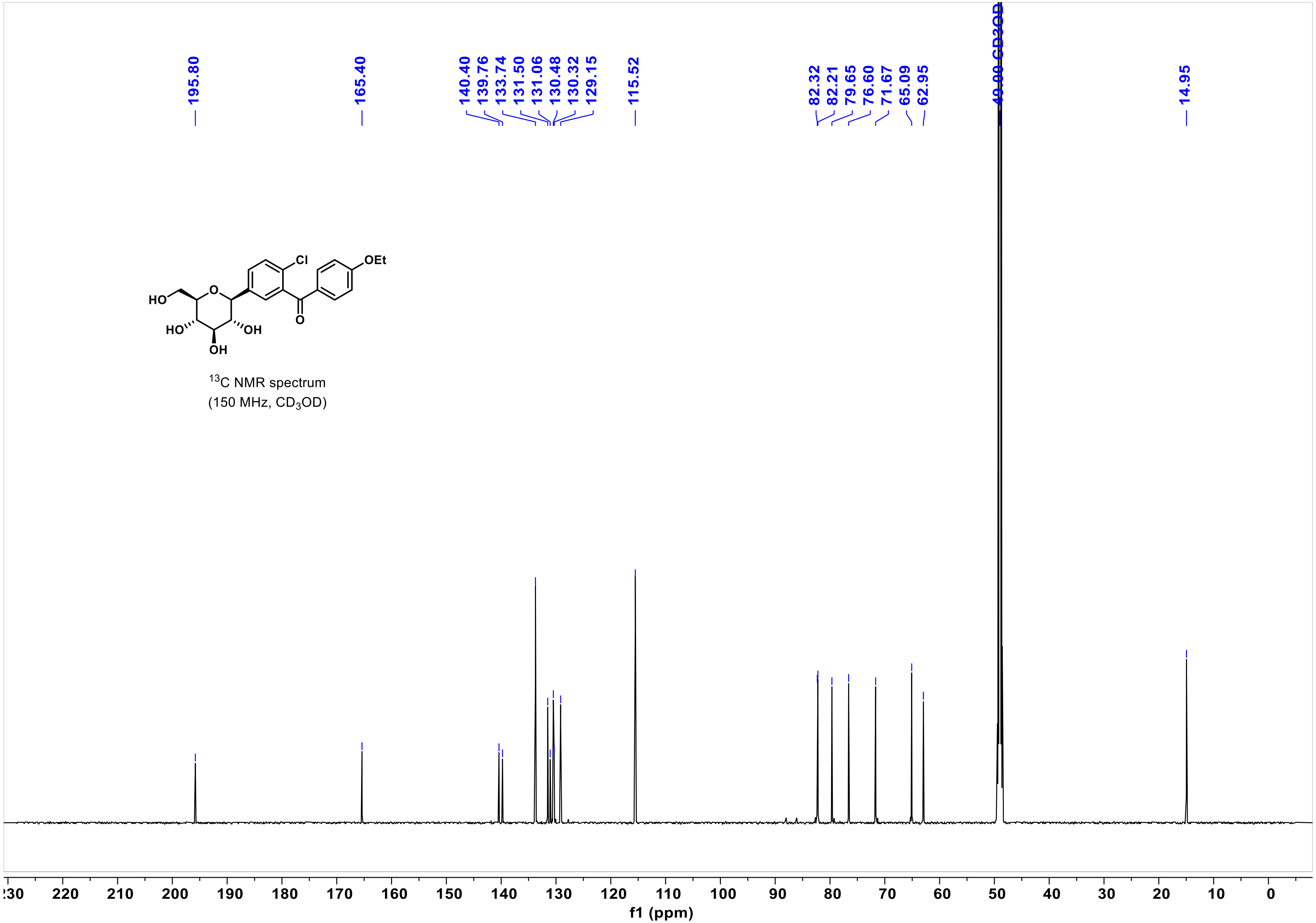

^{13}C NMR of **Keto-Dapagliflozin (37)**

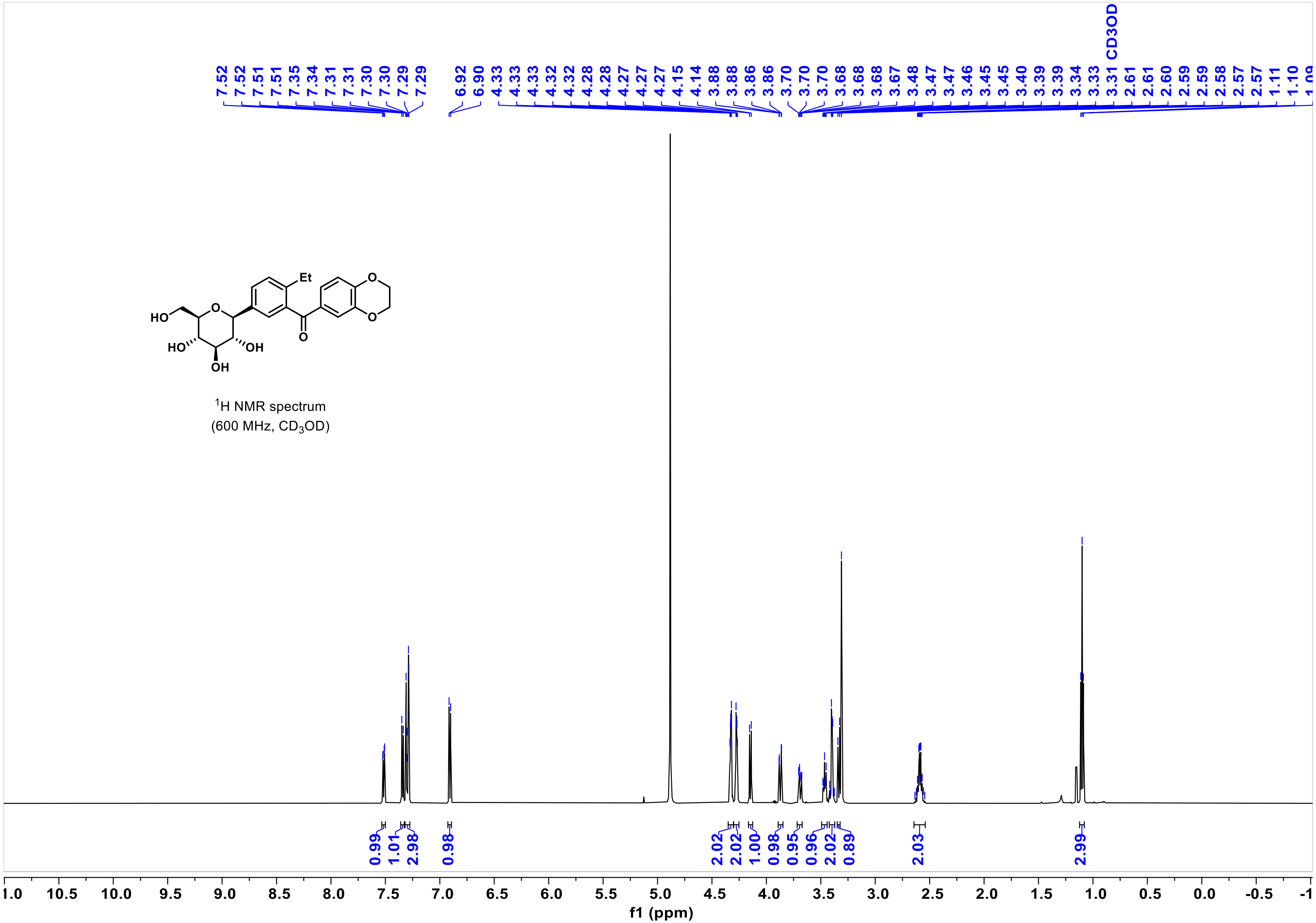

^{1}H NMR of **Keto-Licogliflozin (SA1)**

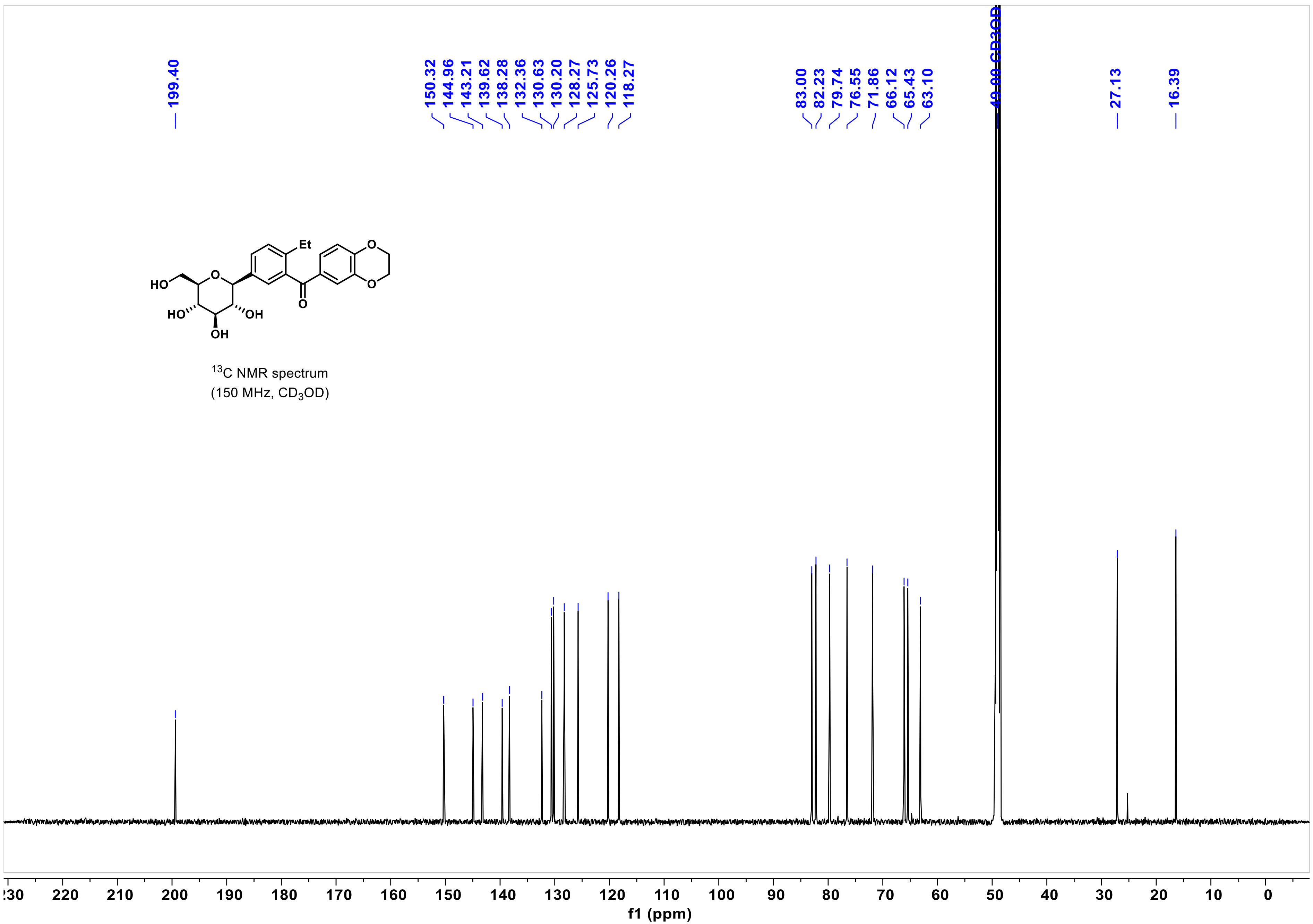

^{13}C NMR of **Keto-Licogliflozin (SA1)**

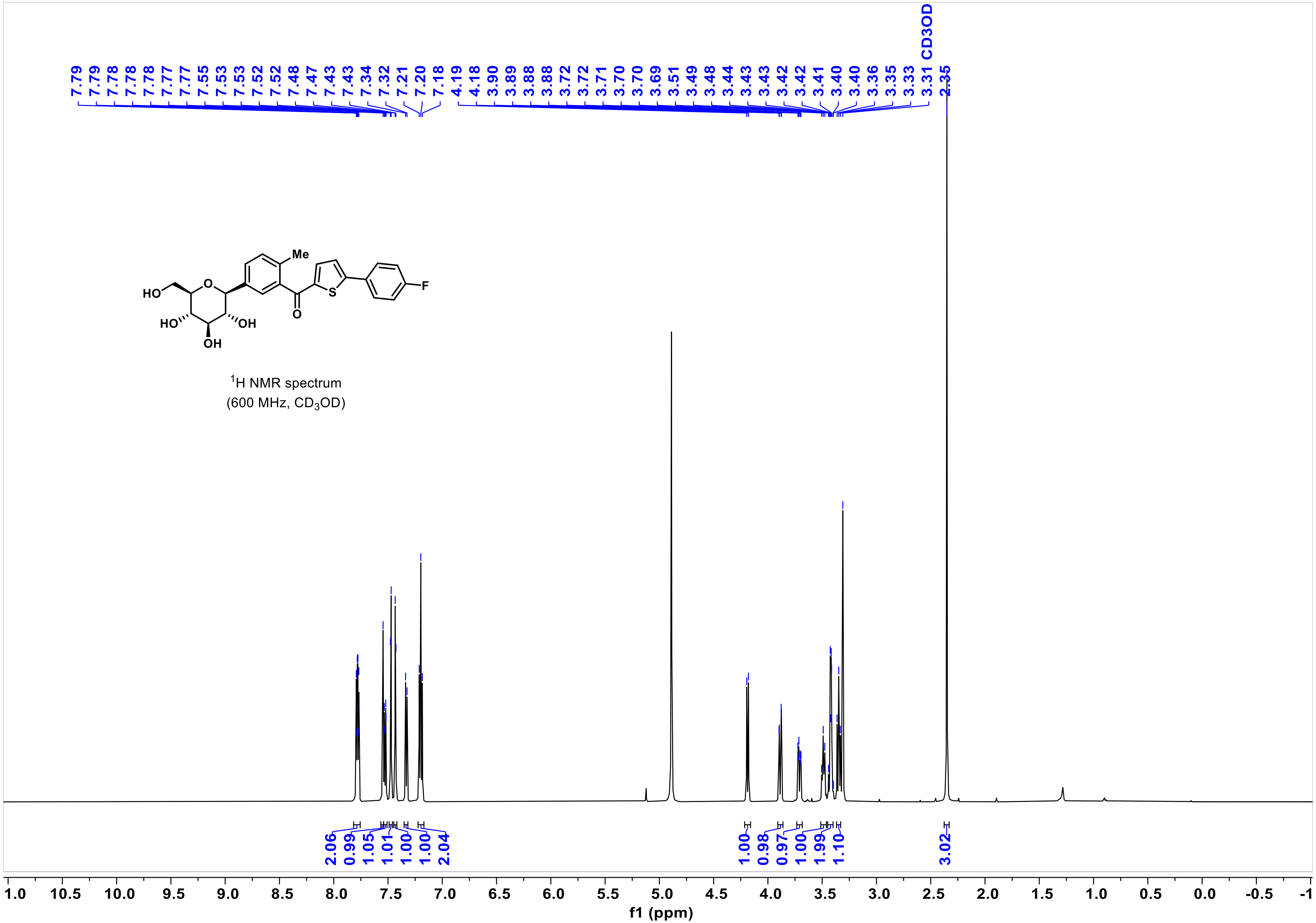

^{1}H NMR of **Keto-Canagliflozin (SA2)**

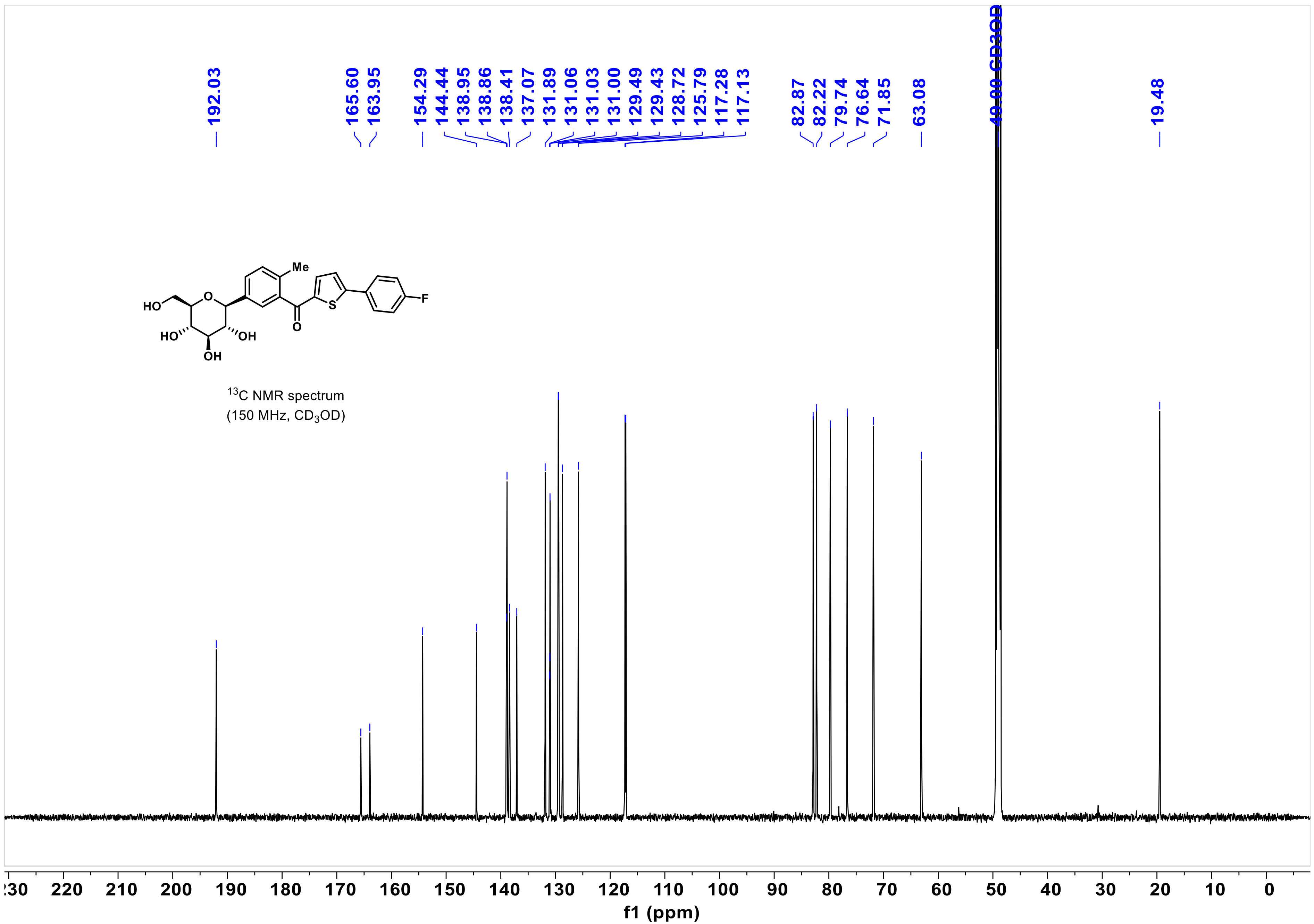

^{13}C NMR of **Keto-Canagliflozin (SA2)**

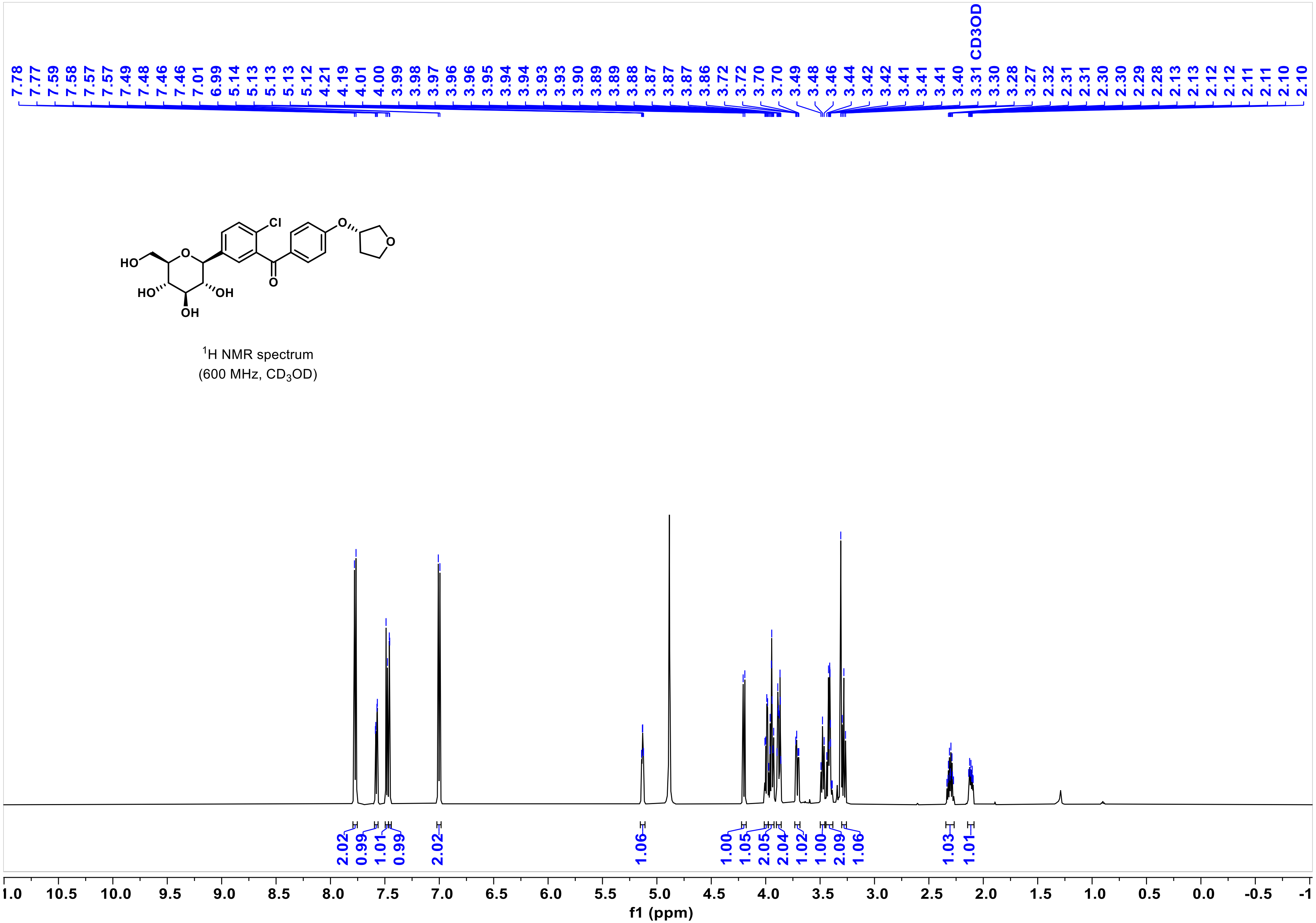

^{1}H NMR of **Keto-Empagliflozin (SA3)**

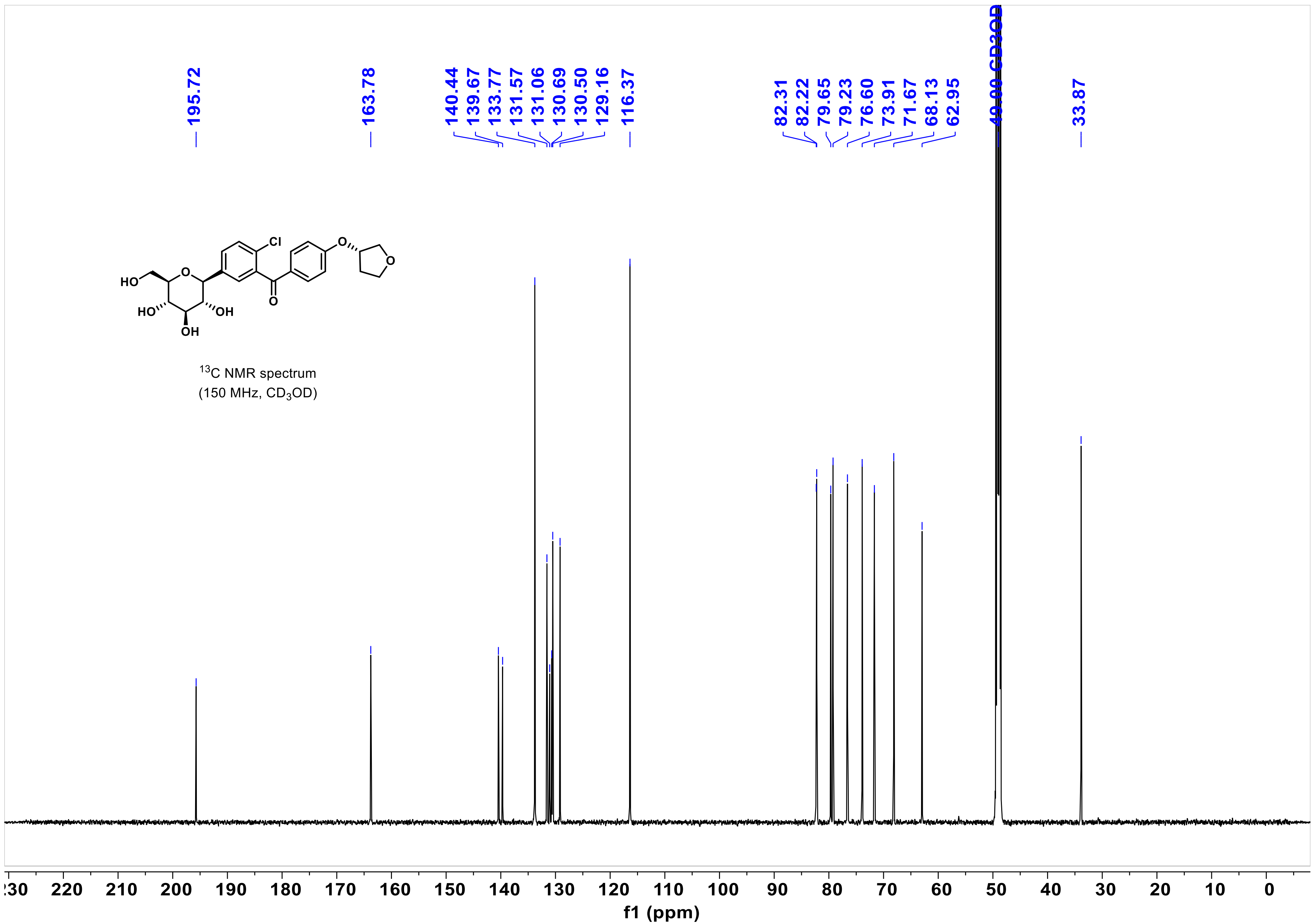

^{13}C NMR of **Keto-Empagliflozin (SA3)**

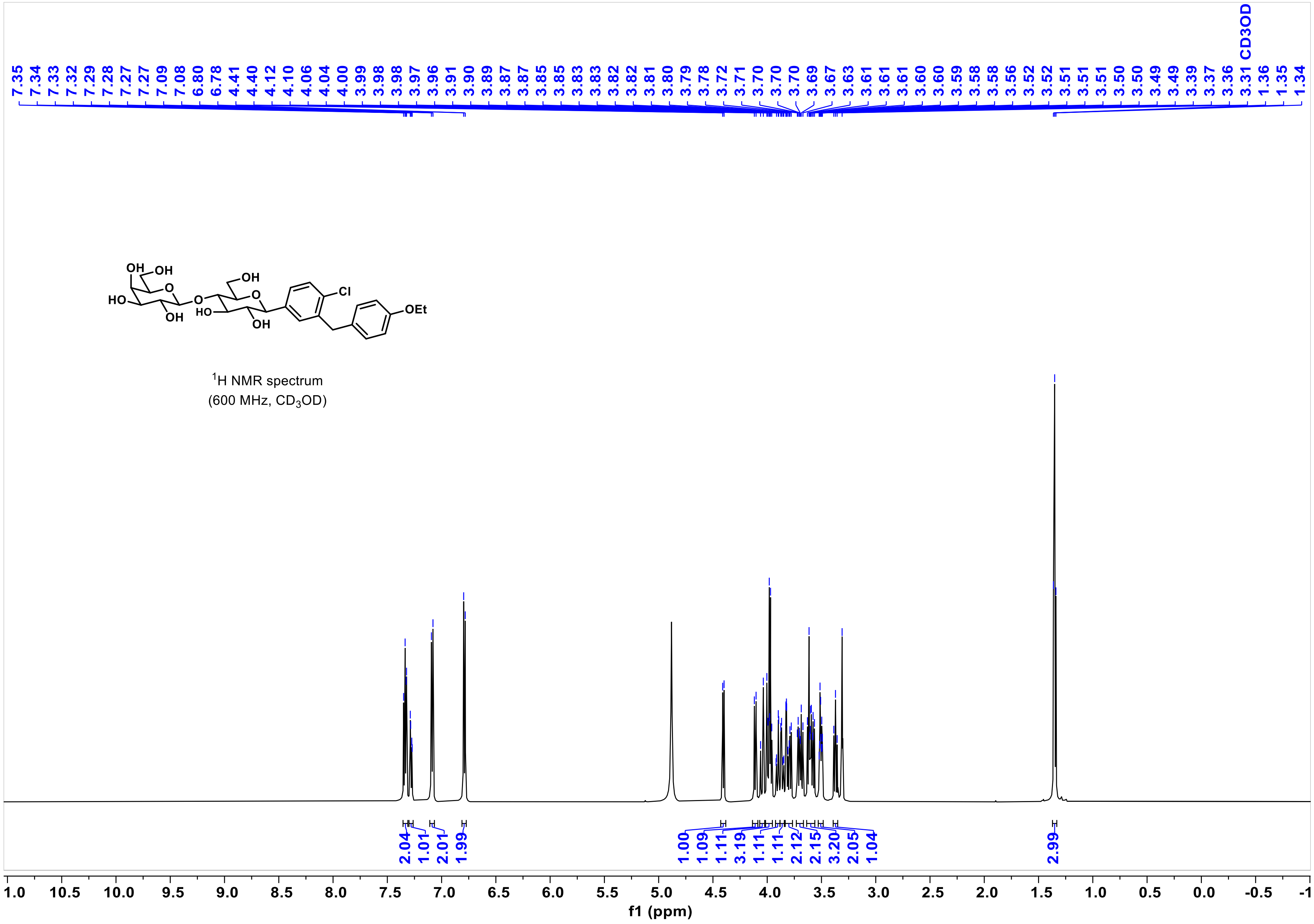

^{1}H NMR of compound **38**

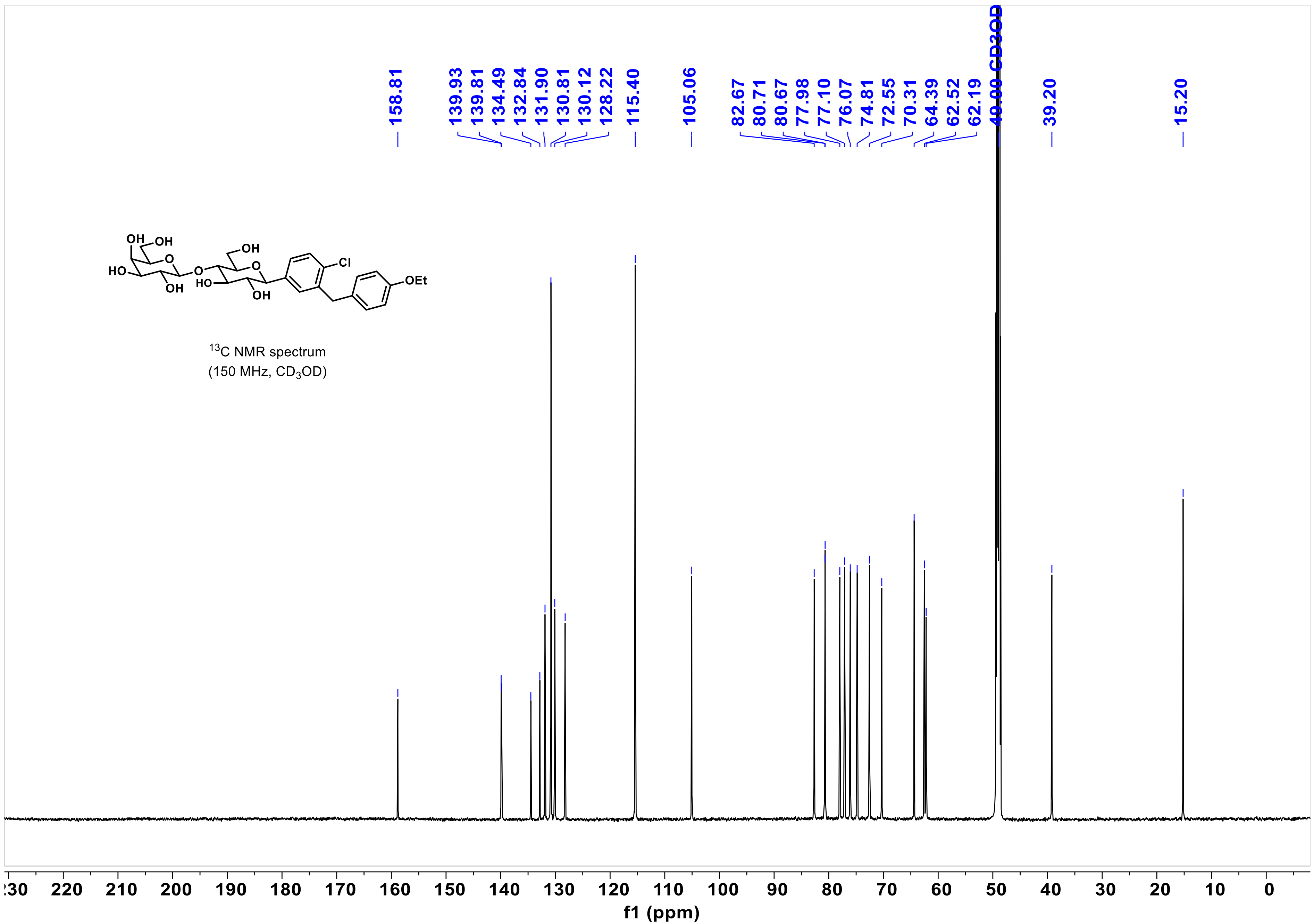

^{13}C NMR of compound **38**

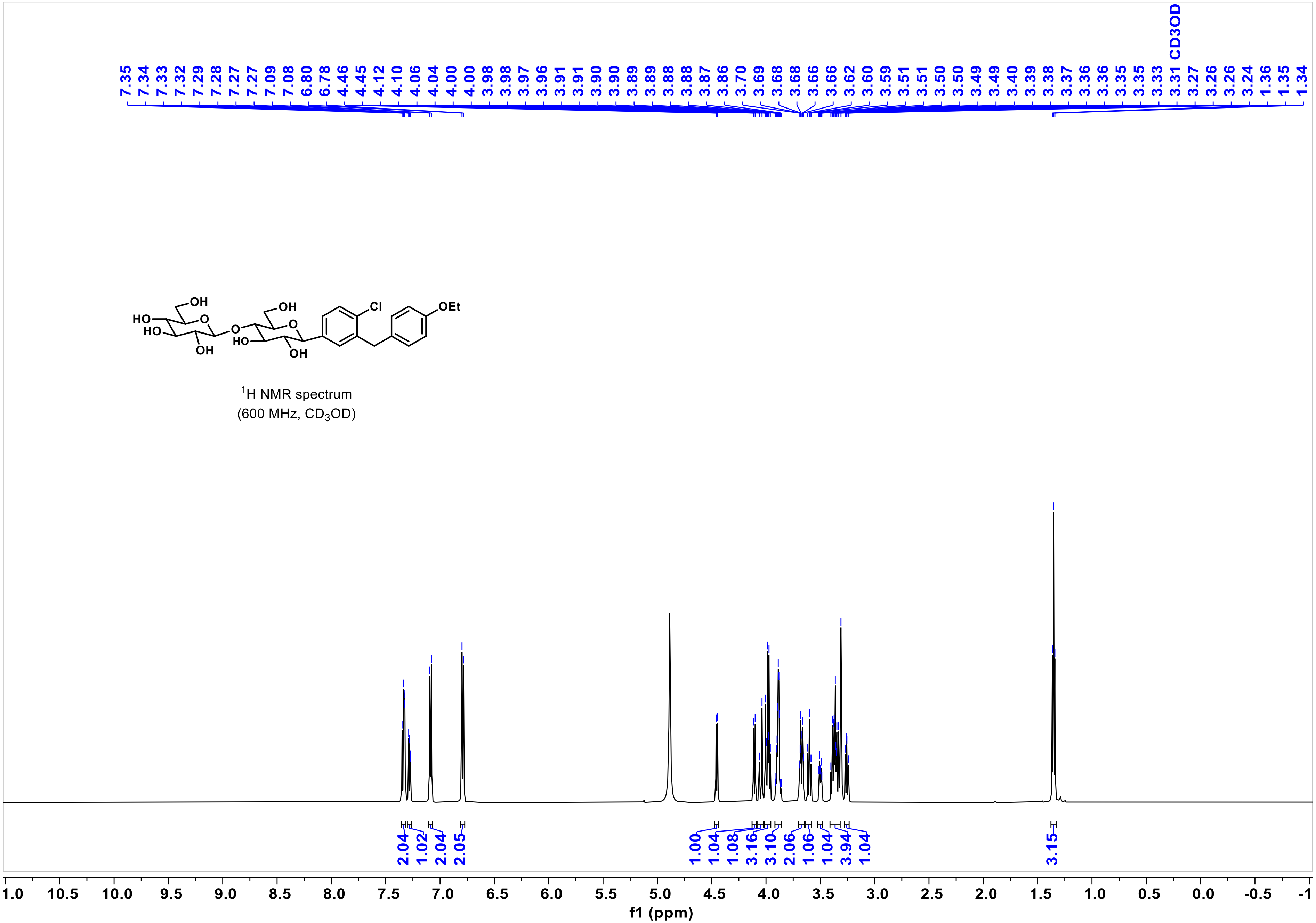

^{1}H NMR of compound **39**

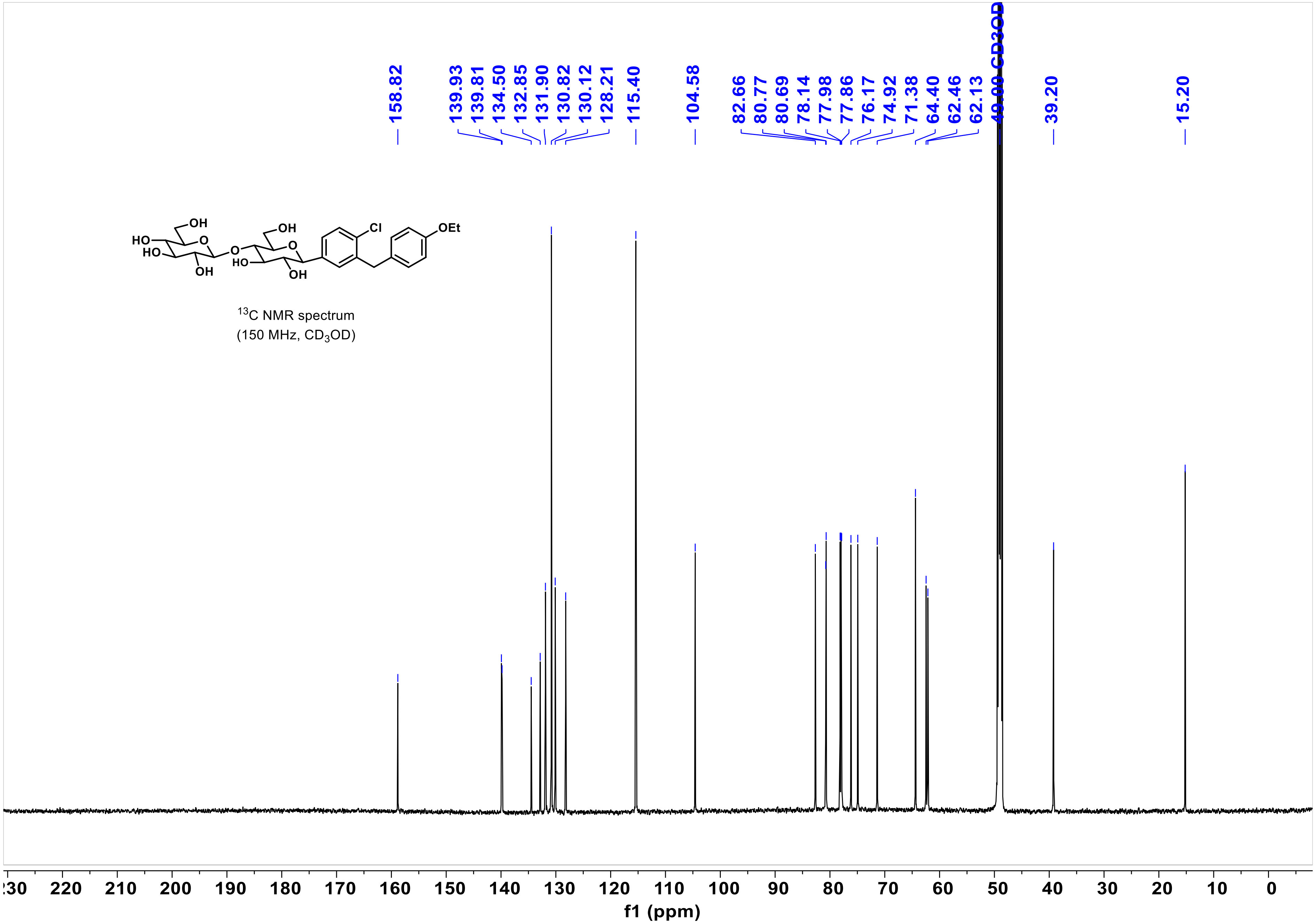

^{13}C NMR of compound **39**

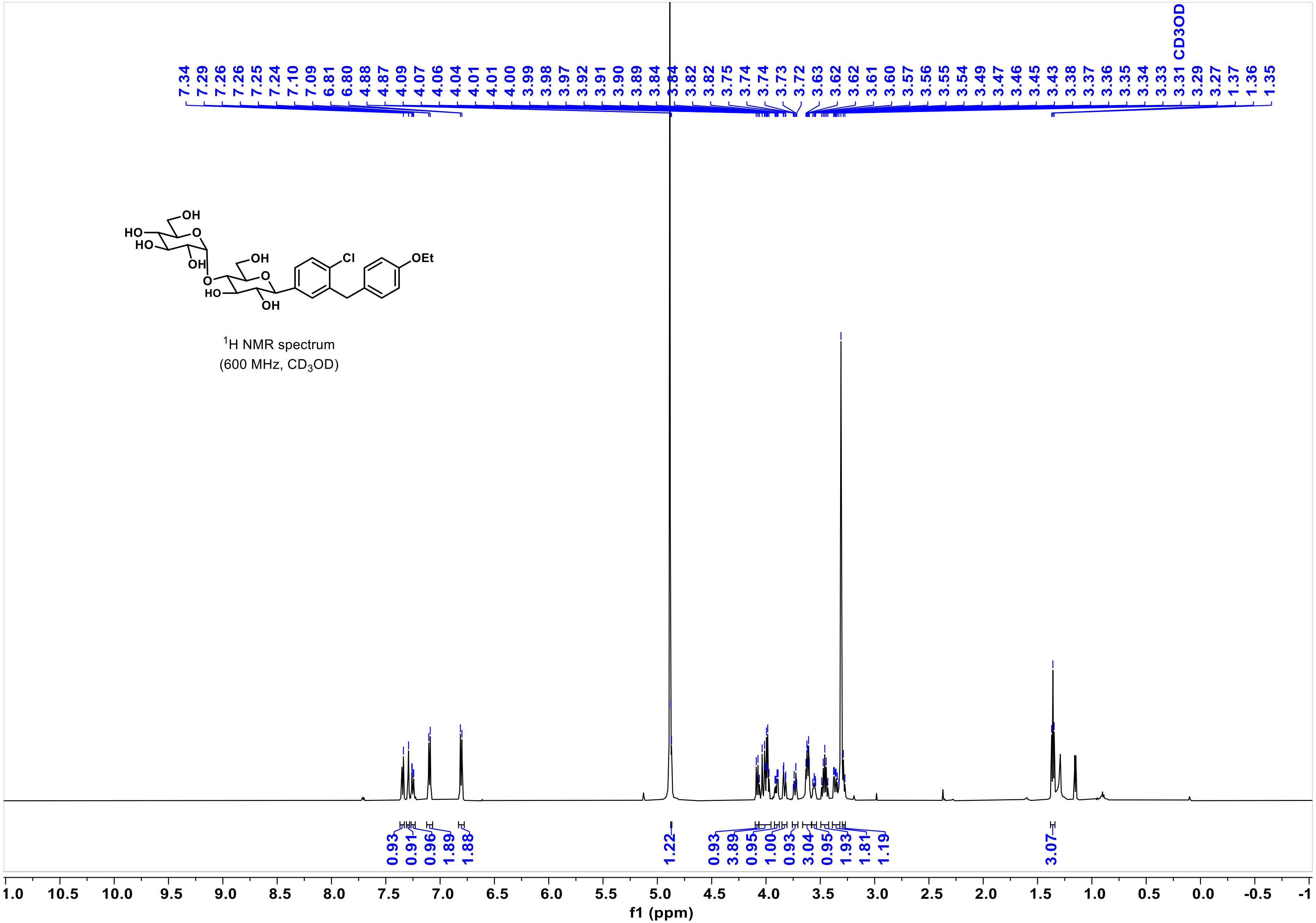

^{1}H NMR of compound **40**

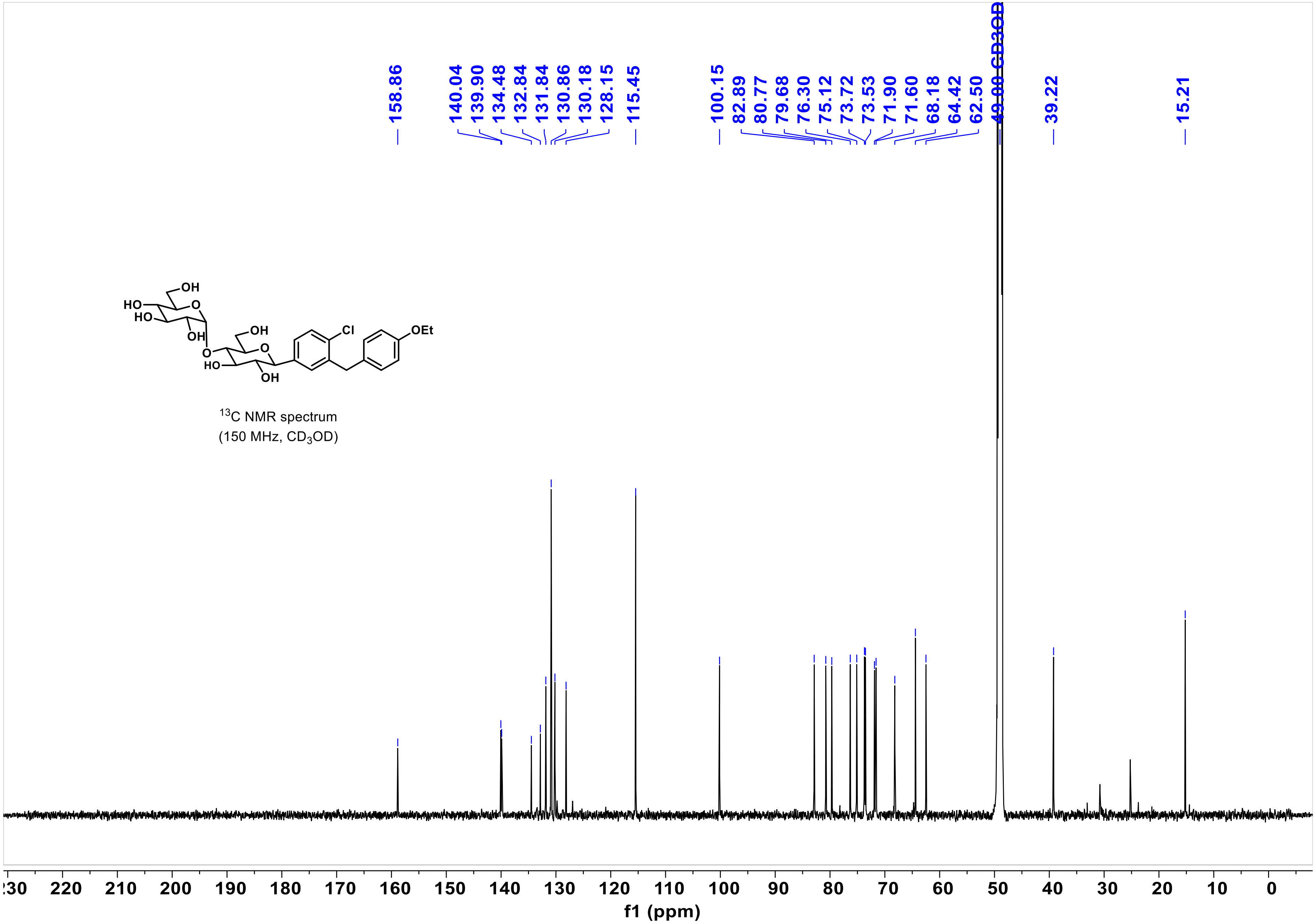

^{13}C NMR of compound **40**

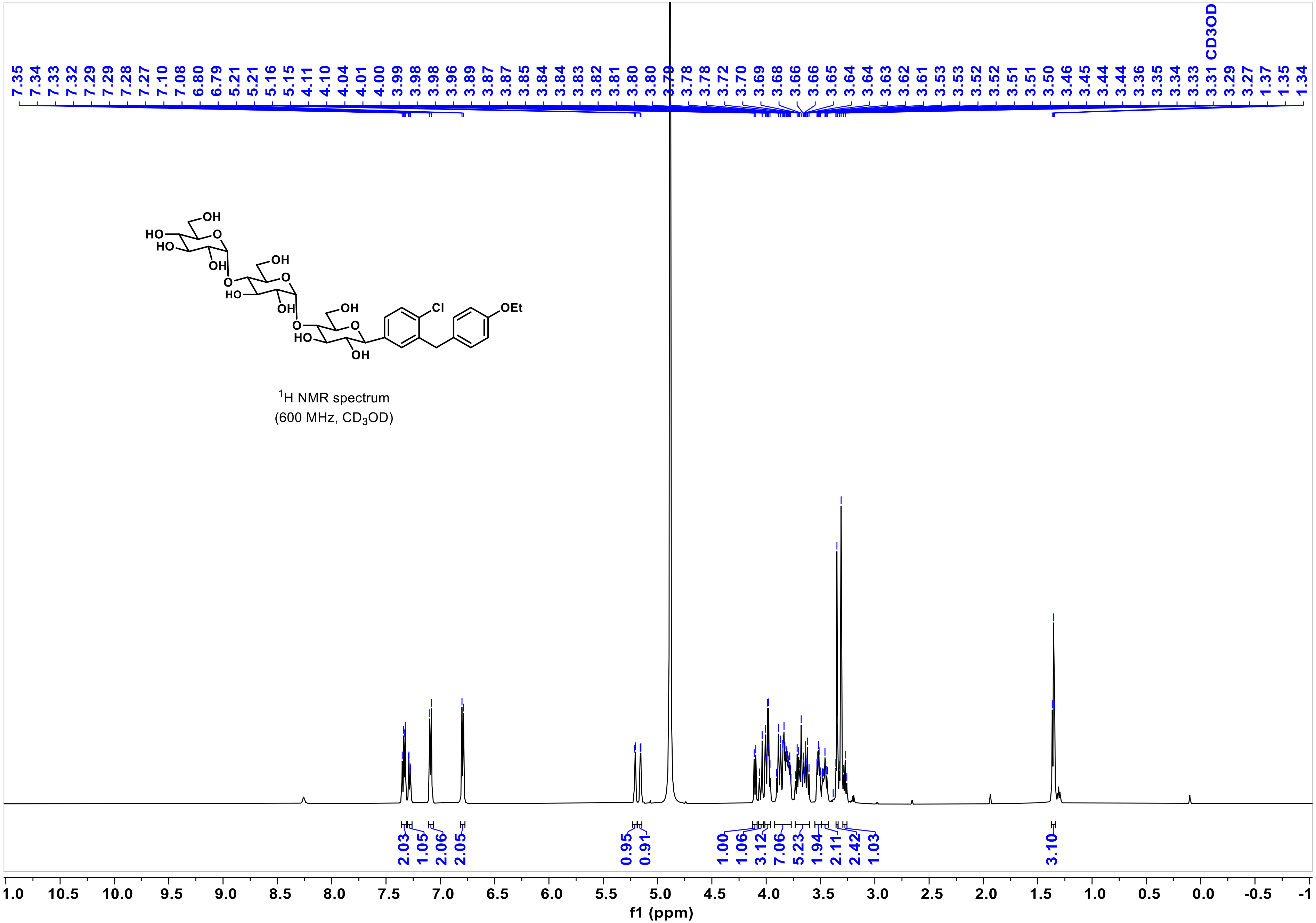

^{1}H NMR of compound **41**

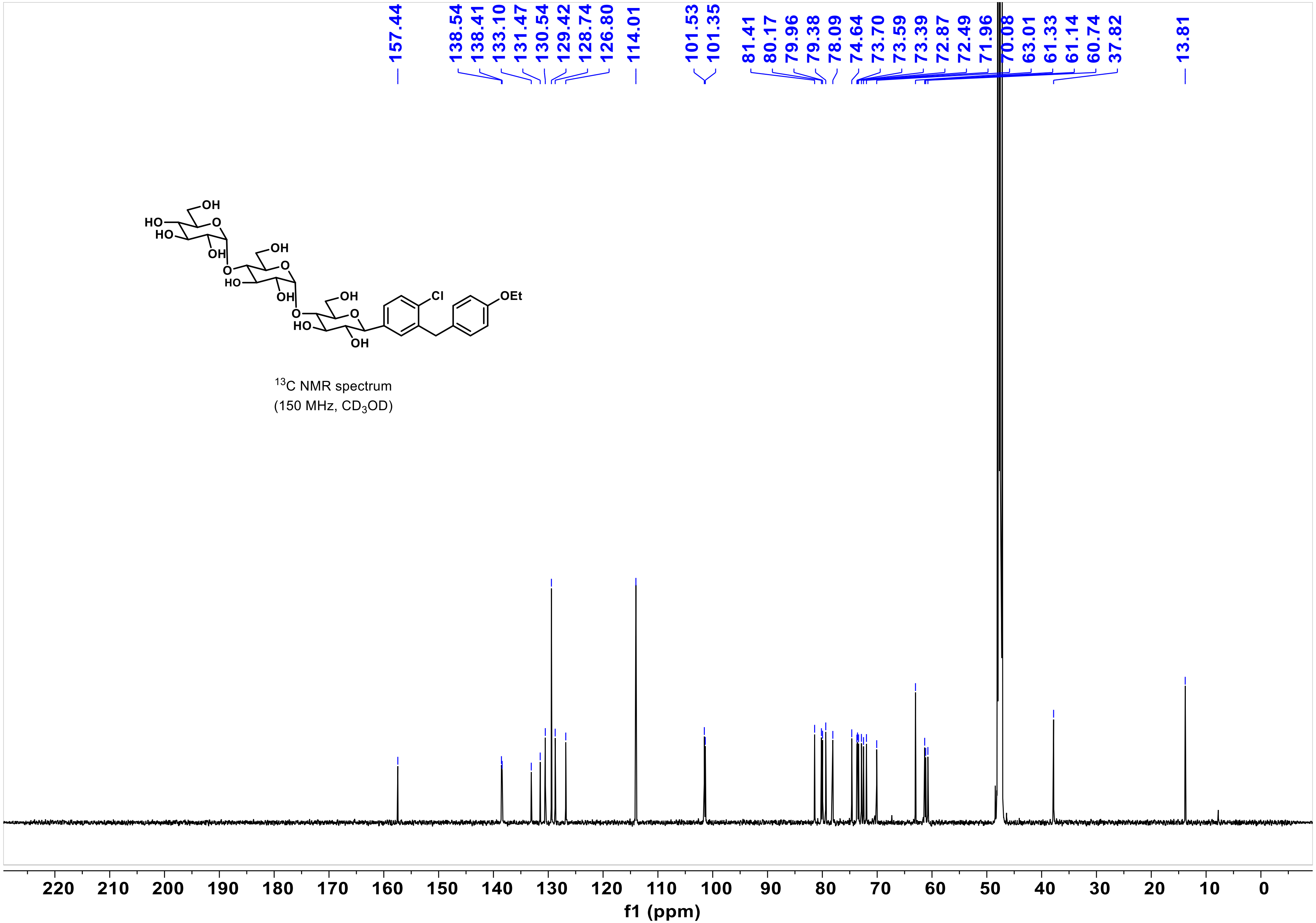

^{13}C NMR of compound **41**

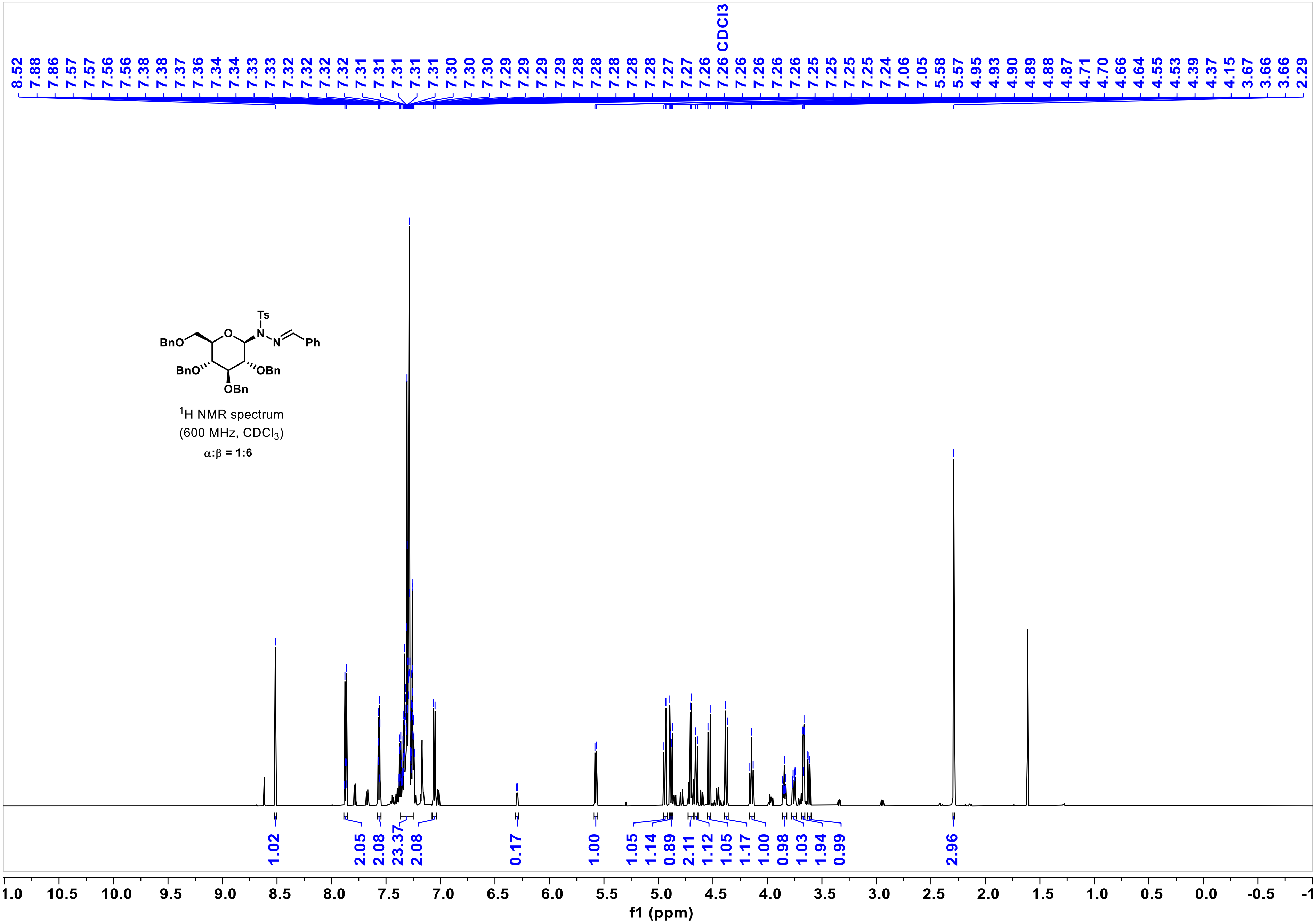

^{1}H NMR of compound **SE2** (α:β = 1:6)

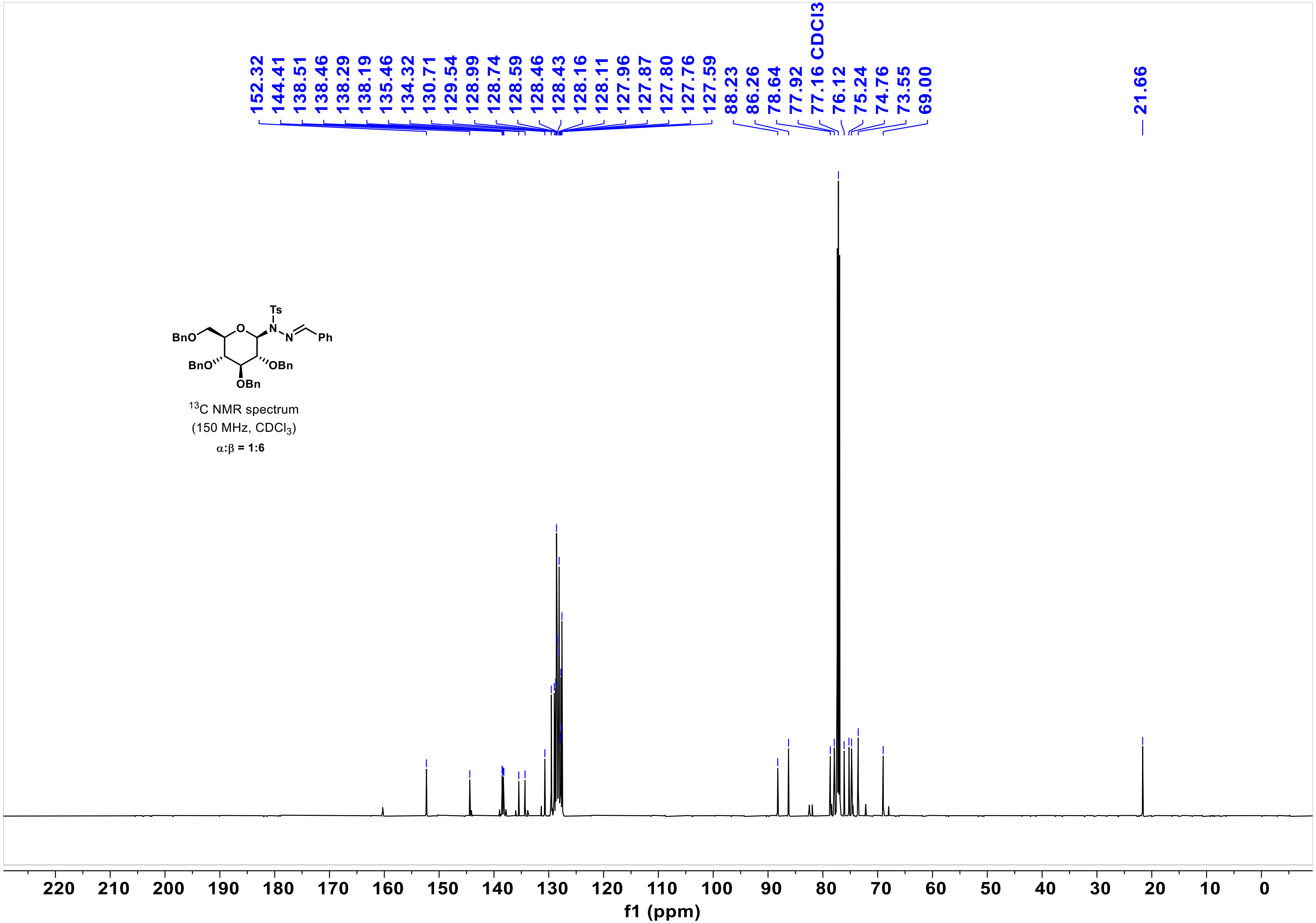

^{1}H NMR of ompound **SE2** (α:β = 1:6)

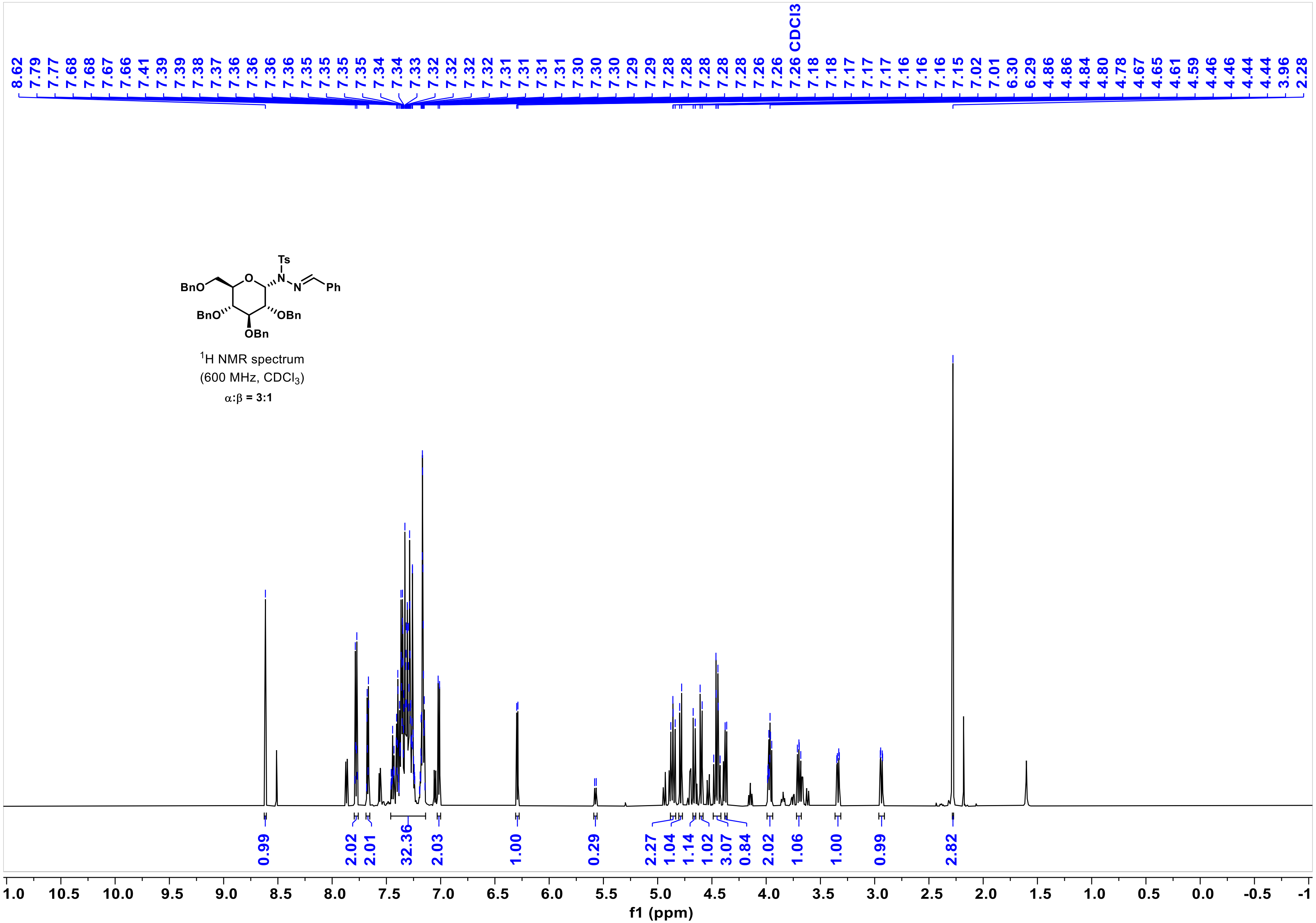

^{1}H NMR of compound **SE2** (α:β = 3:1)

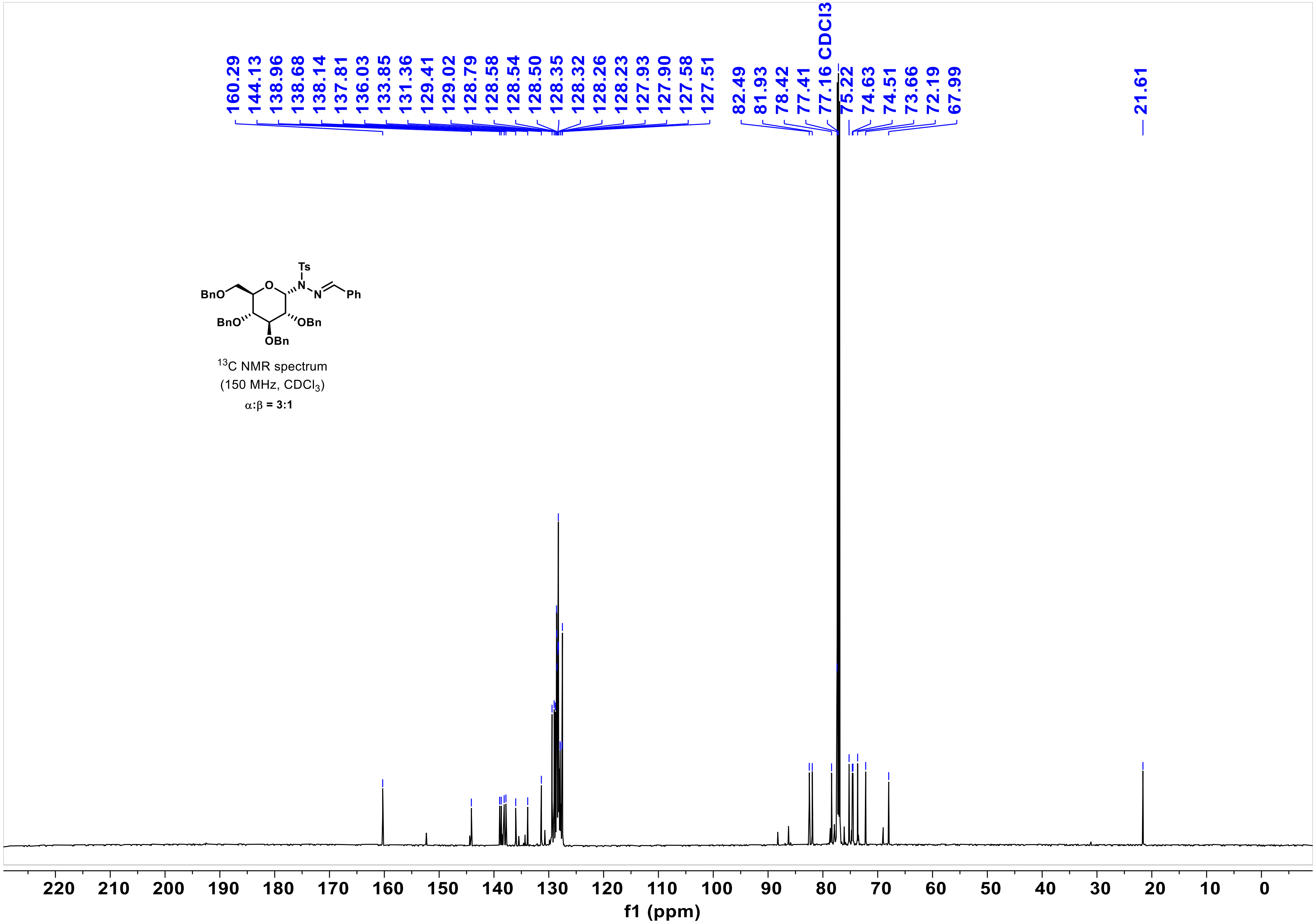

^{13}C NMR of compound **SE2** (α:β = 3:1)

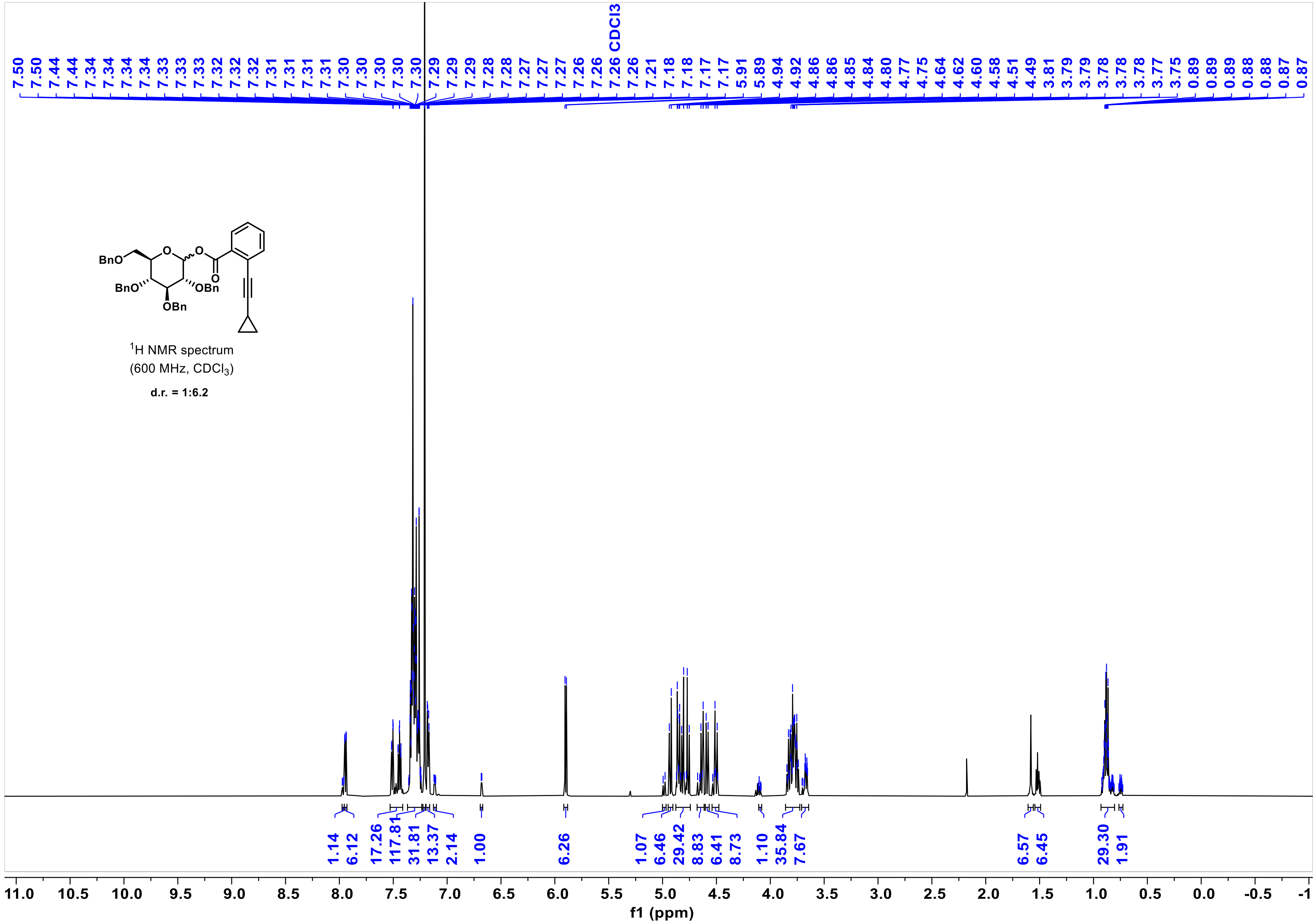

^{1}H NMR of compound **SE4**

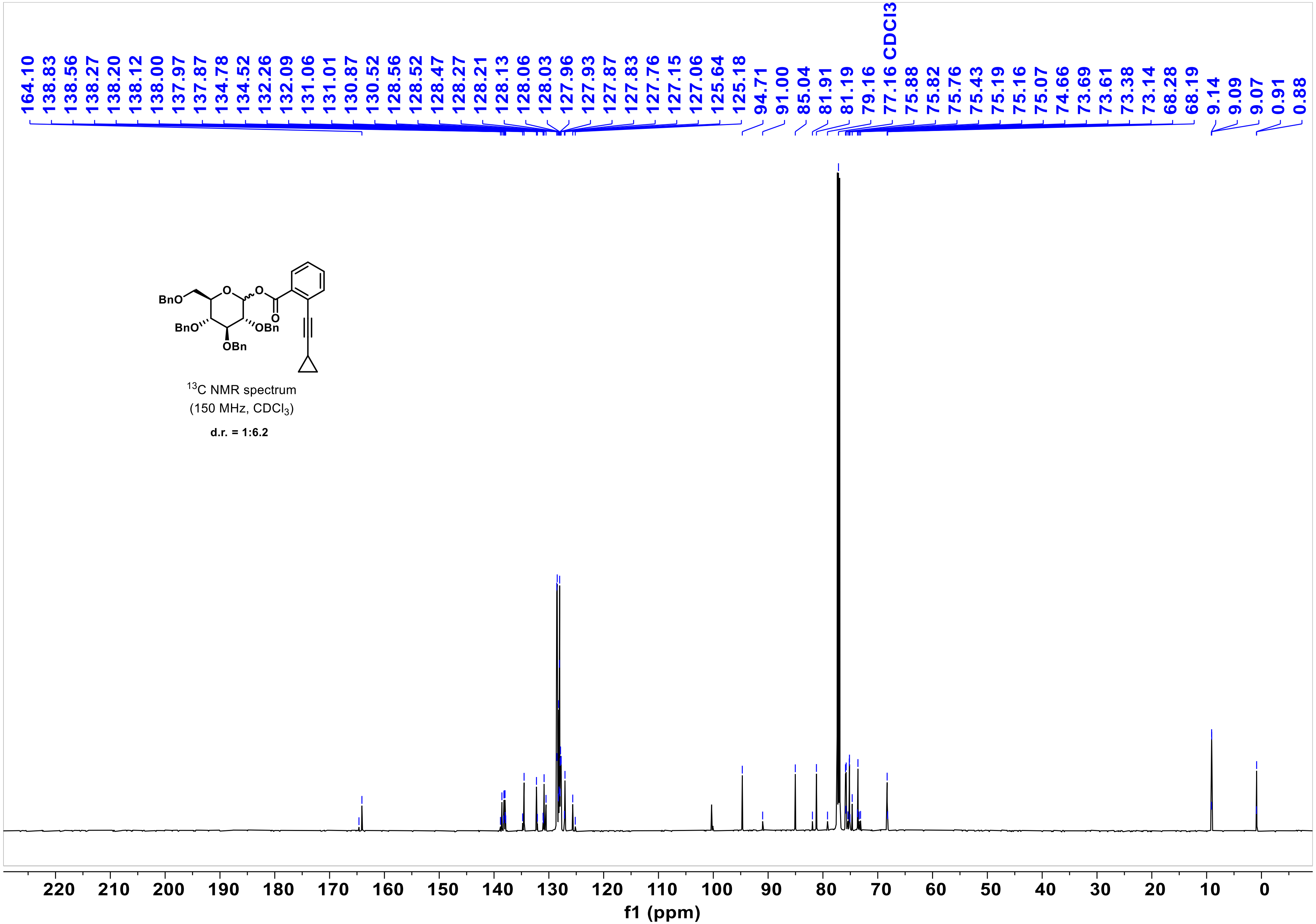

^{13}C NMR of compound **SE4**

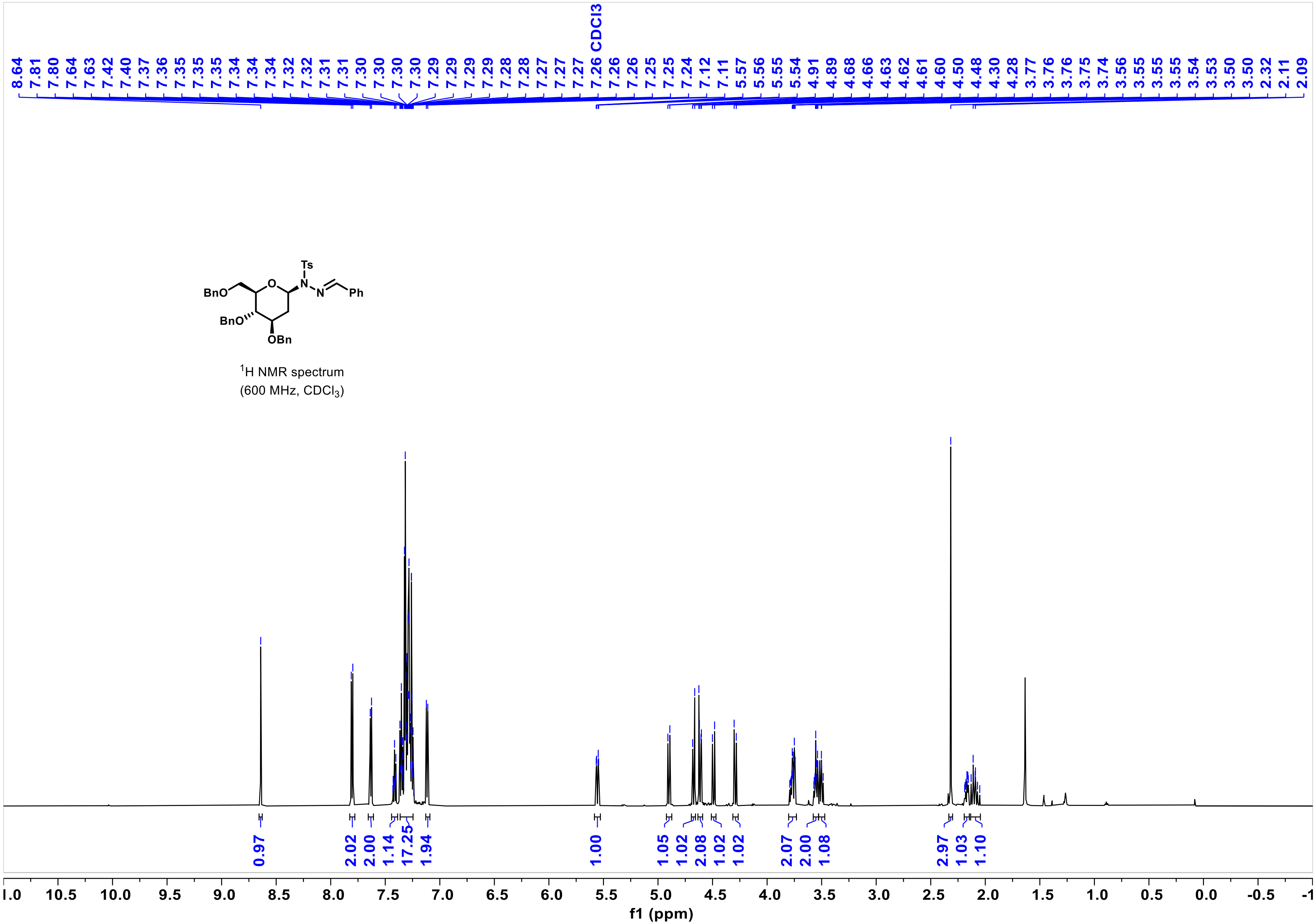

^{1}H NMR of compound **β-SE6**

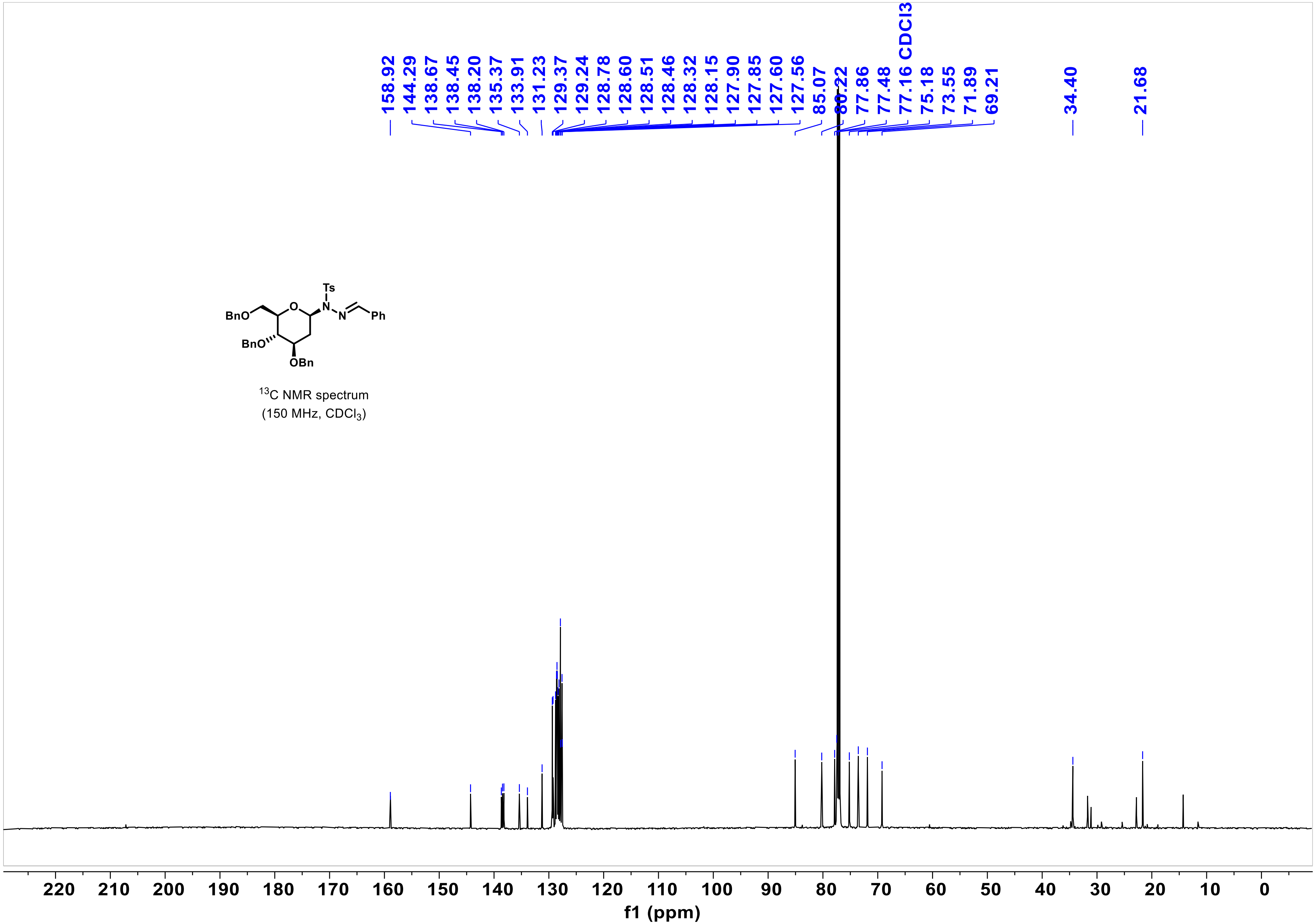

^{13}C NMR of compound **β-SE6**

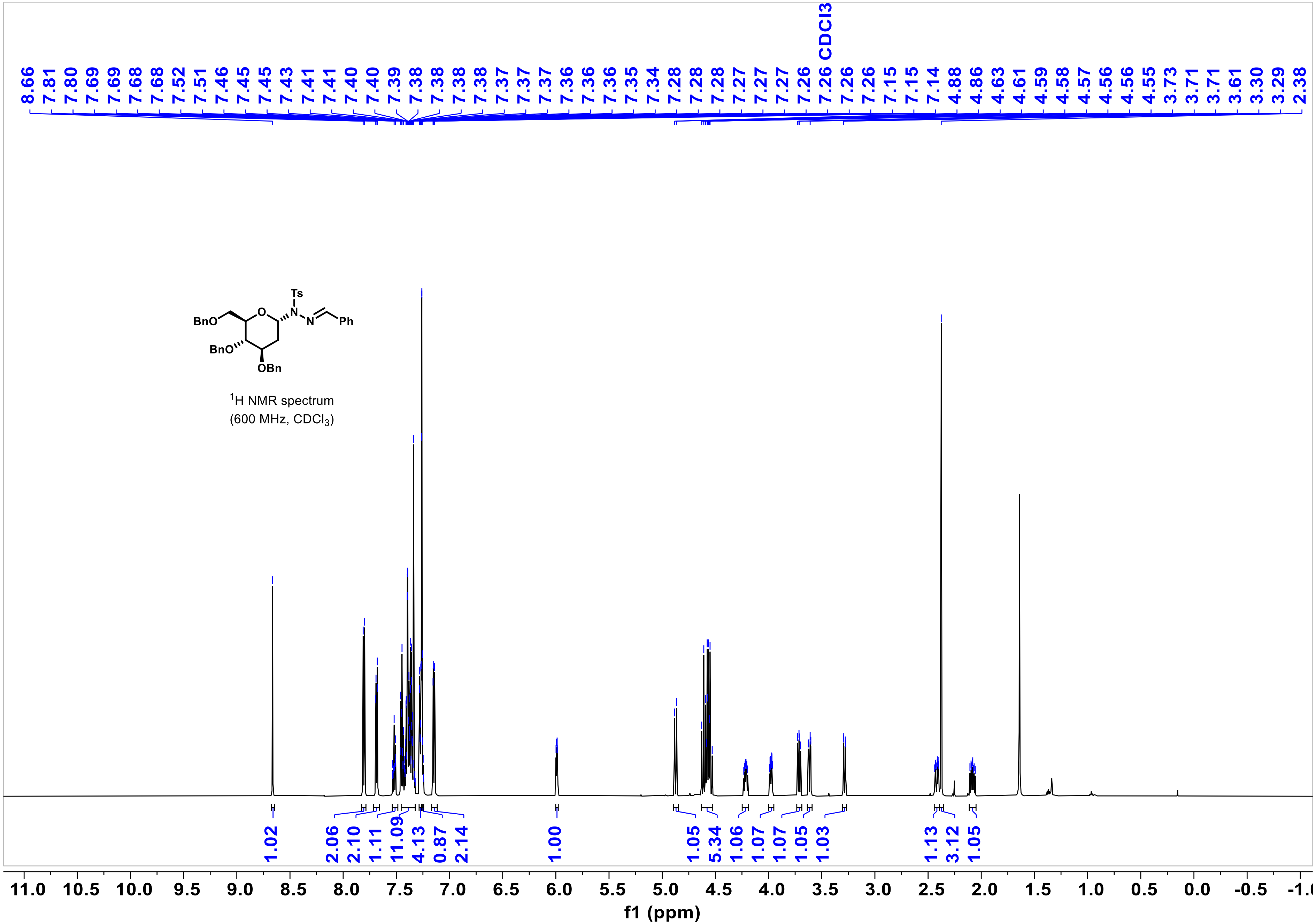

^{1}H NMR of compound **α-SE6**

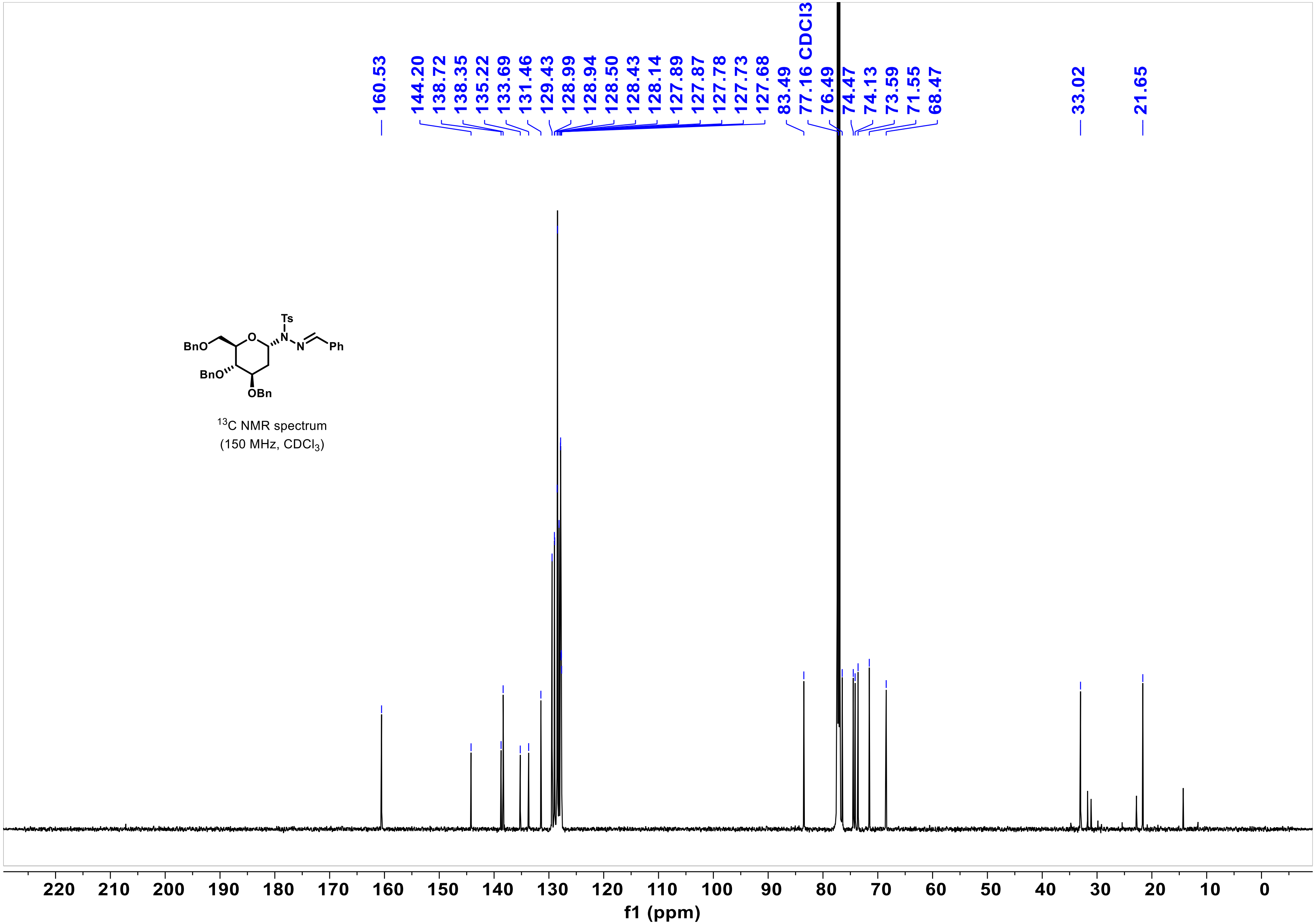

^{13}C NMR of compound **α-SE6**

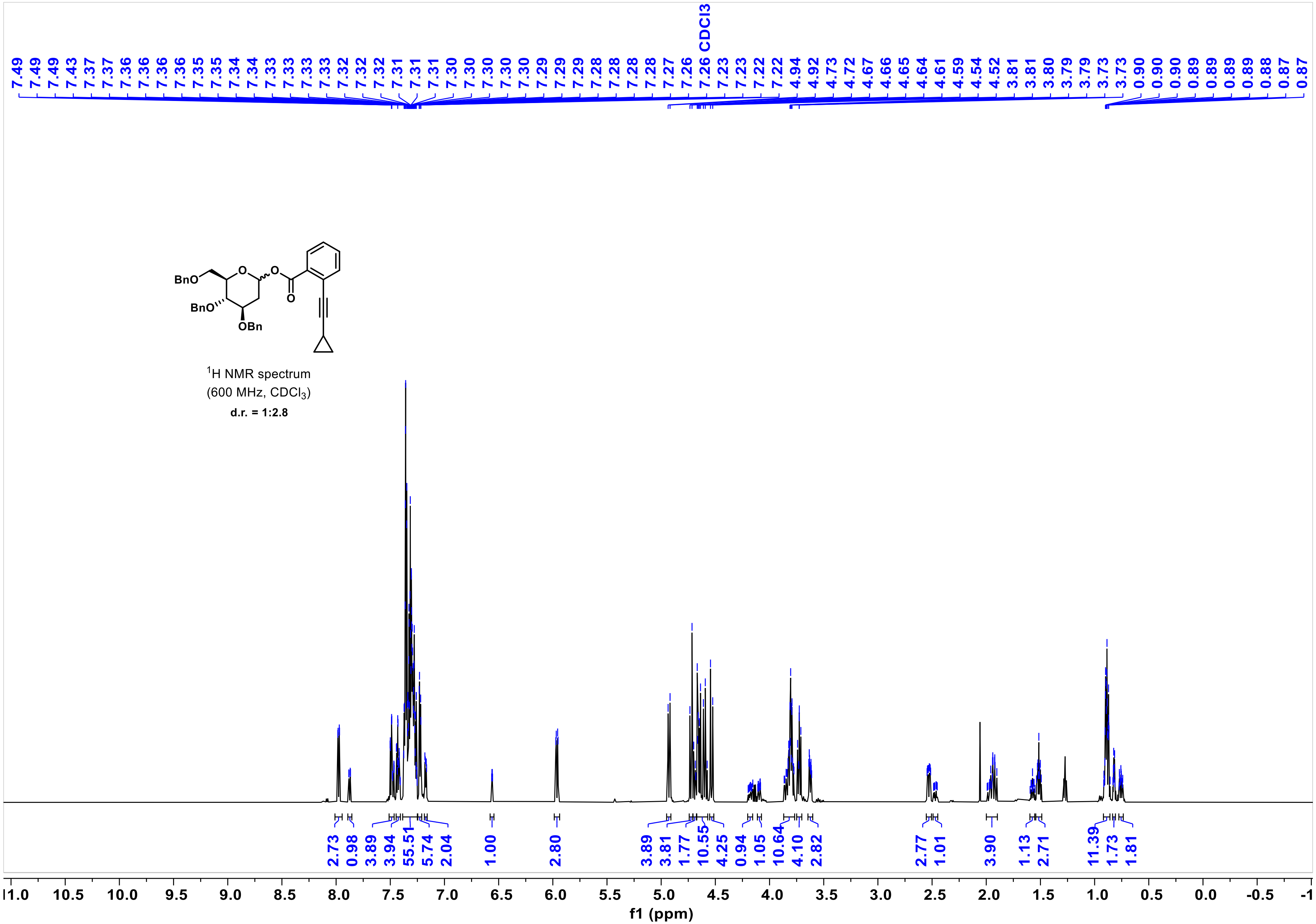

^{1}H NMR of compound **SE7**

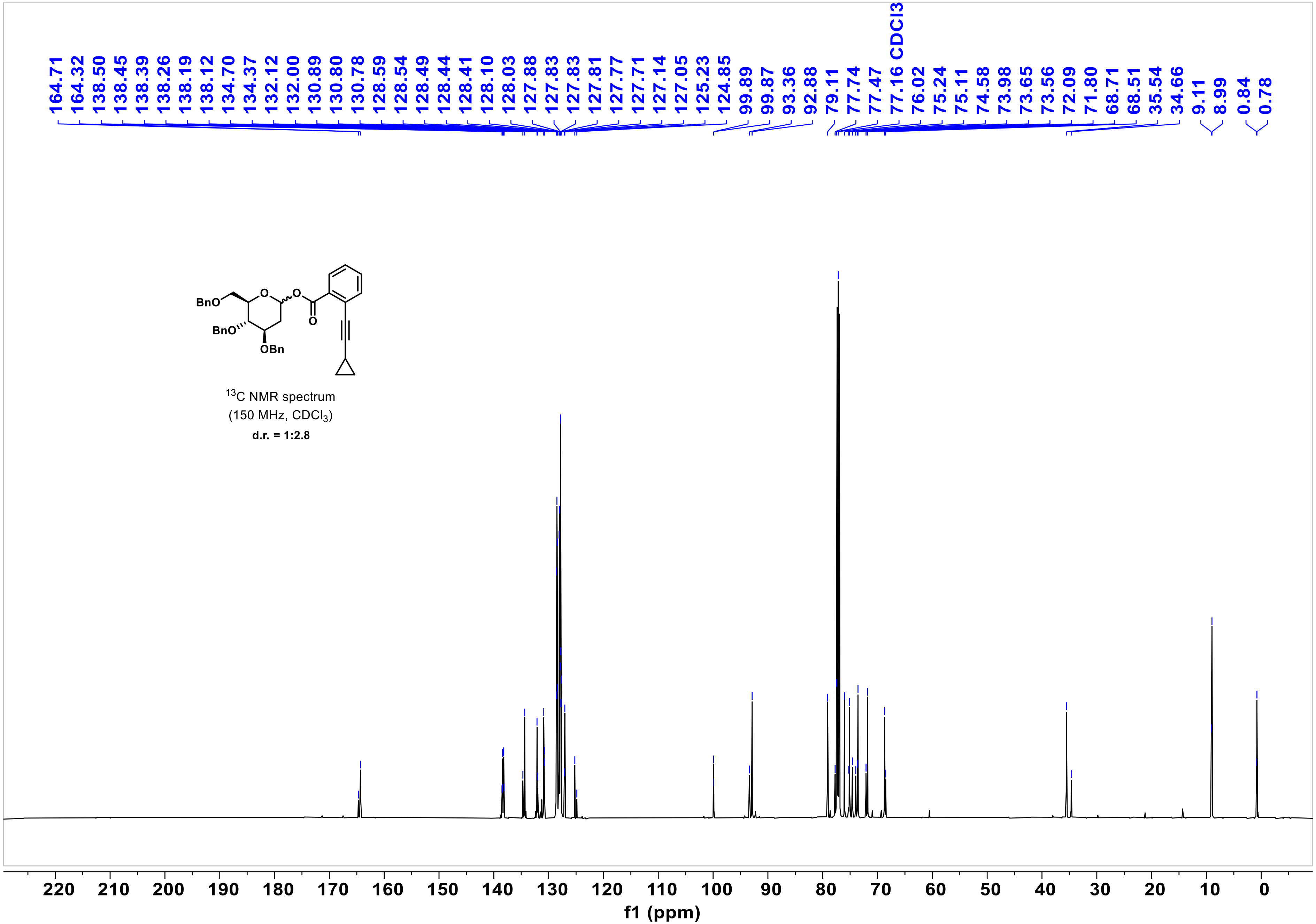

^{13}C NMR of compound **SE7**

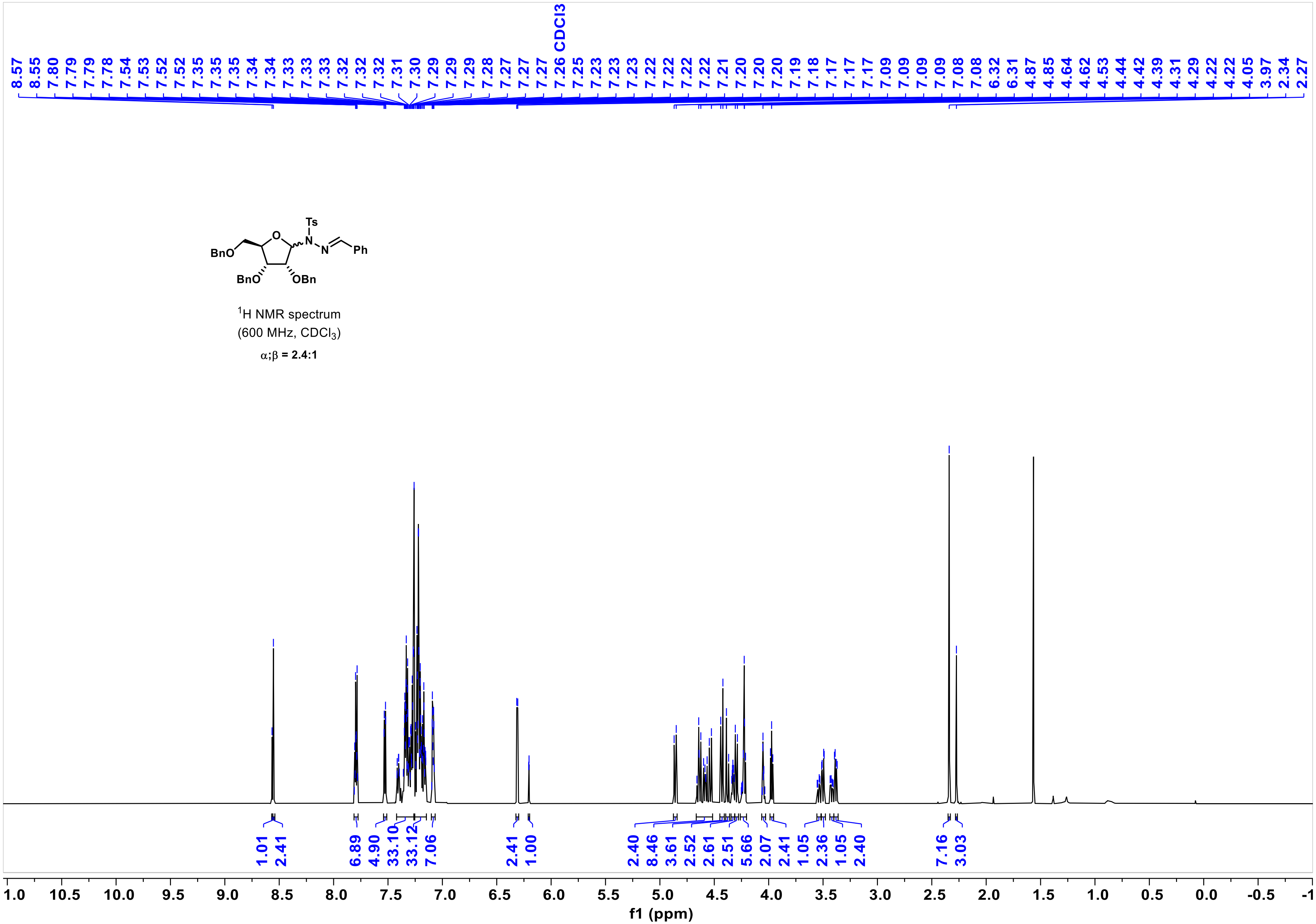

^{1}H NMR of compound **SE9** (α:β = 2.4:1)

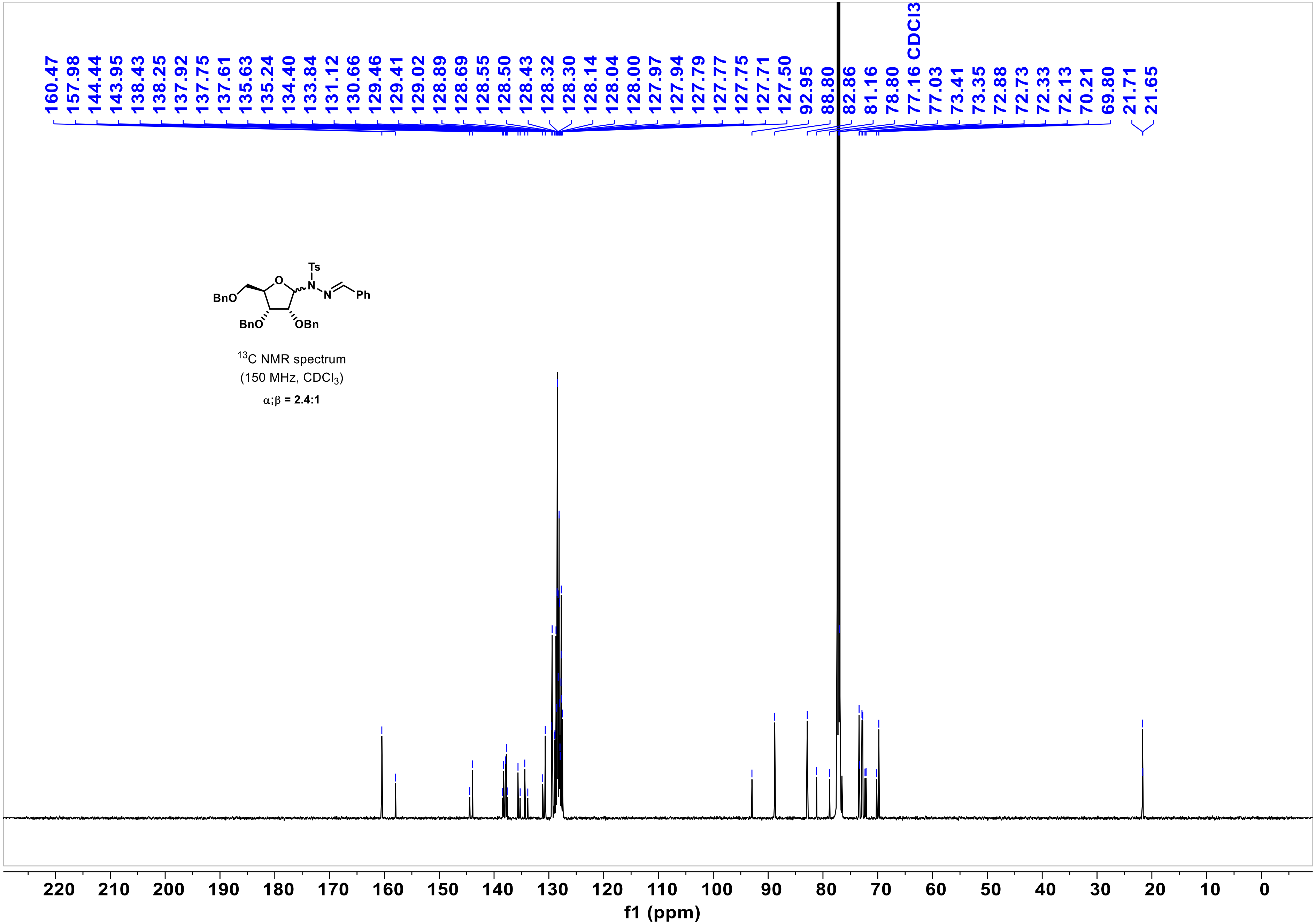

^{13}C NMR of compound **SE9** (α:β = 2.4:1)

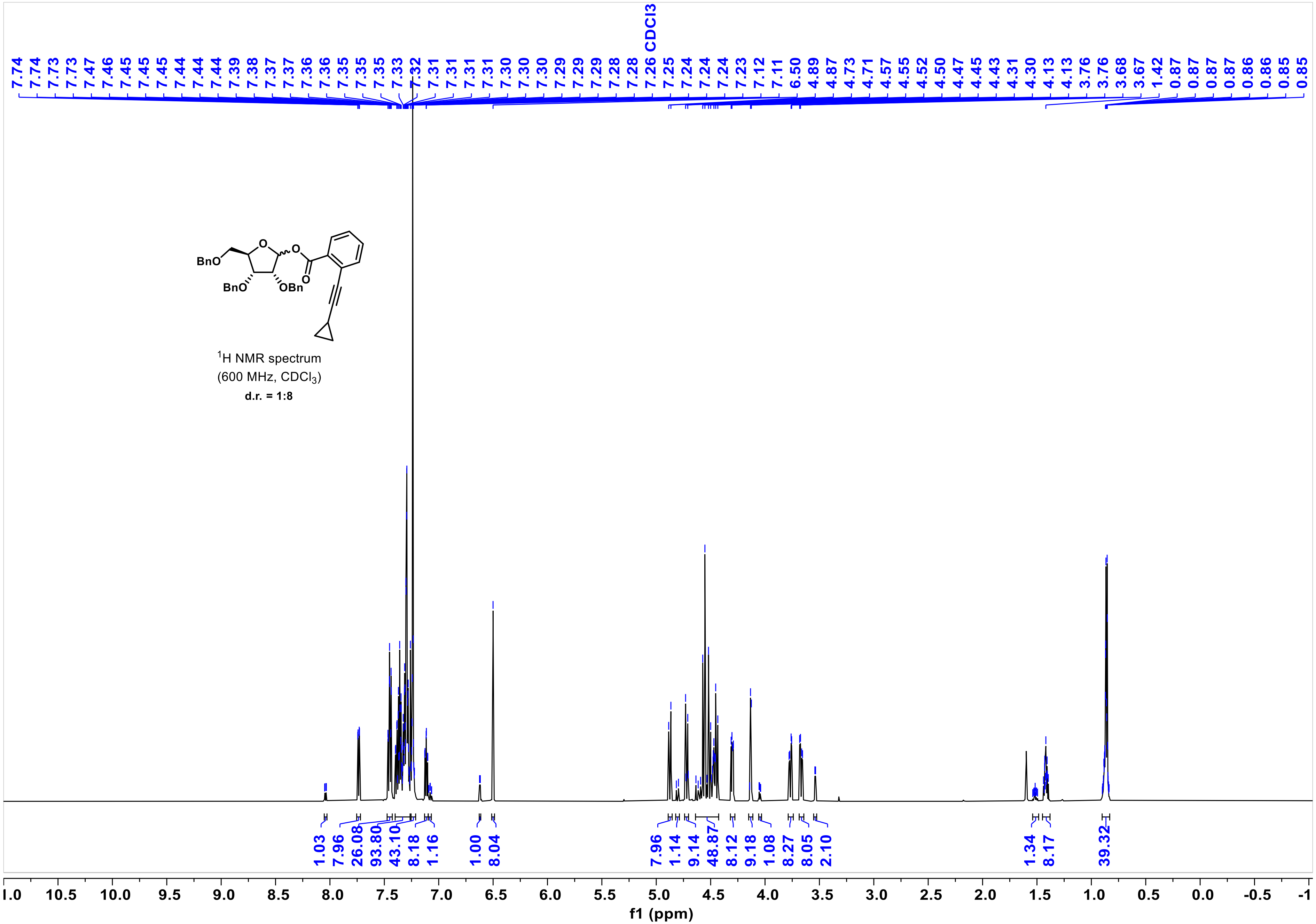

^{1}H NMR of compound **SE10**

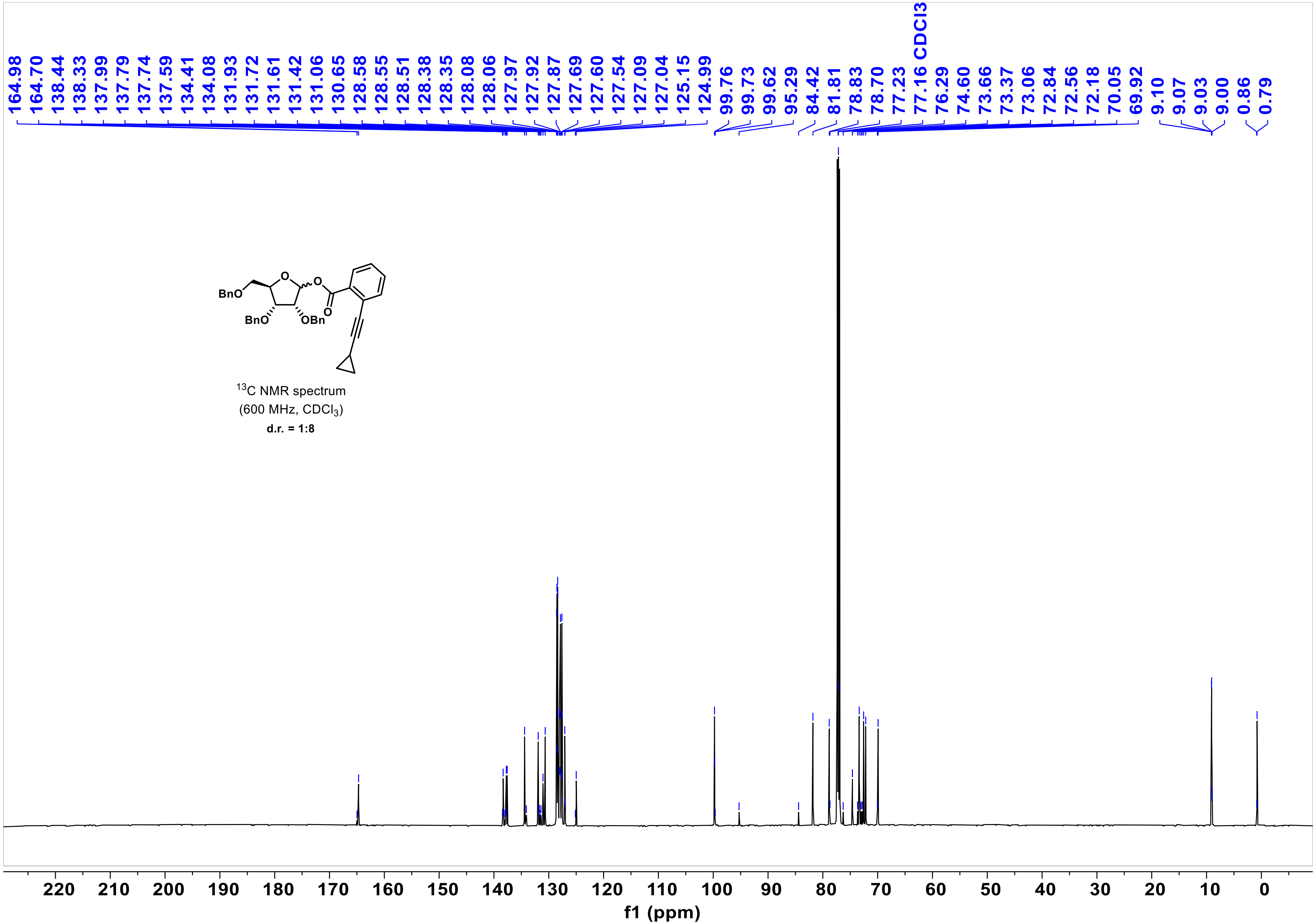

^{13}C NMR of compound **SE10**

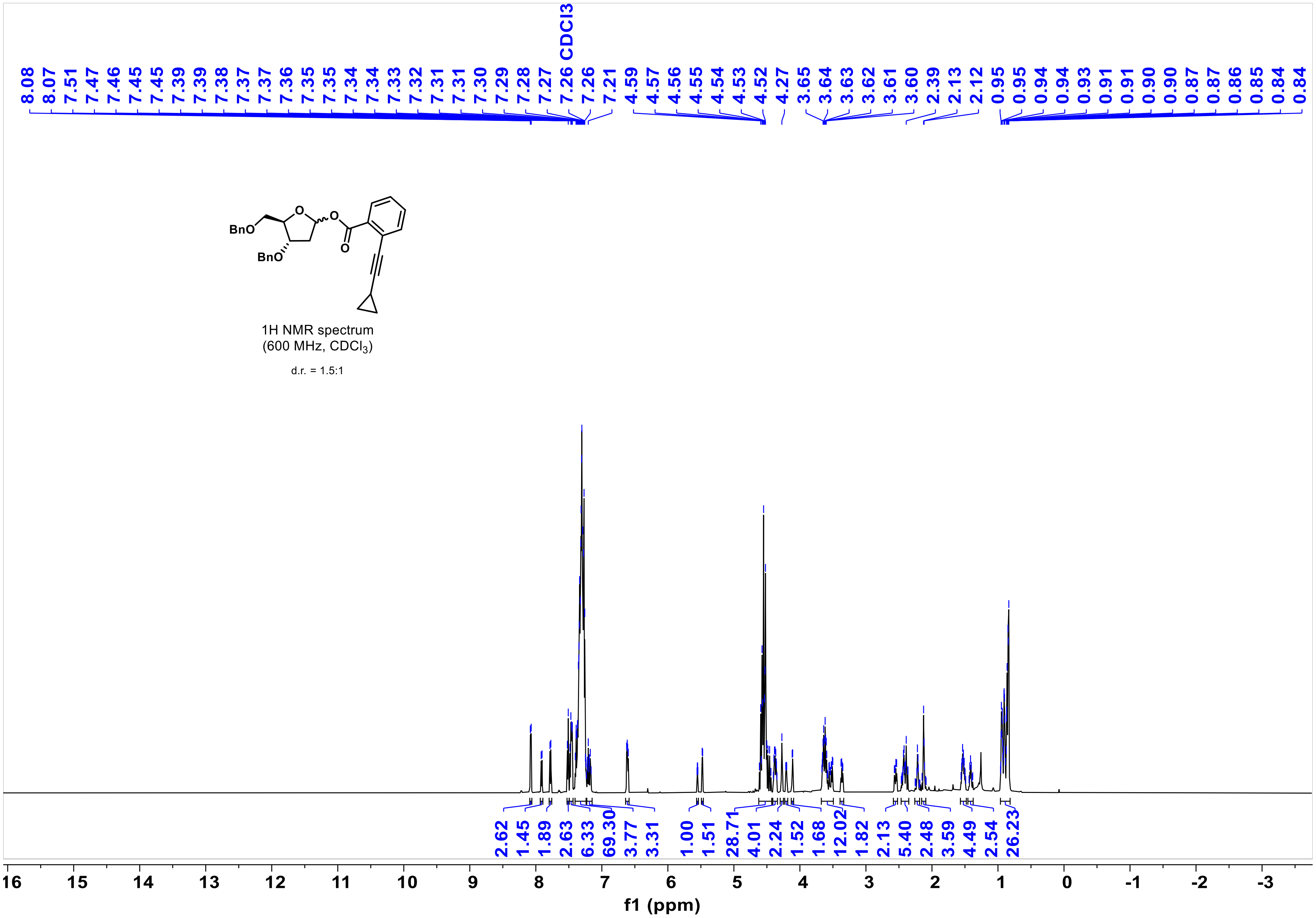

^{1}H NMR of compound **SE12**

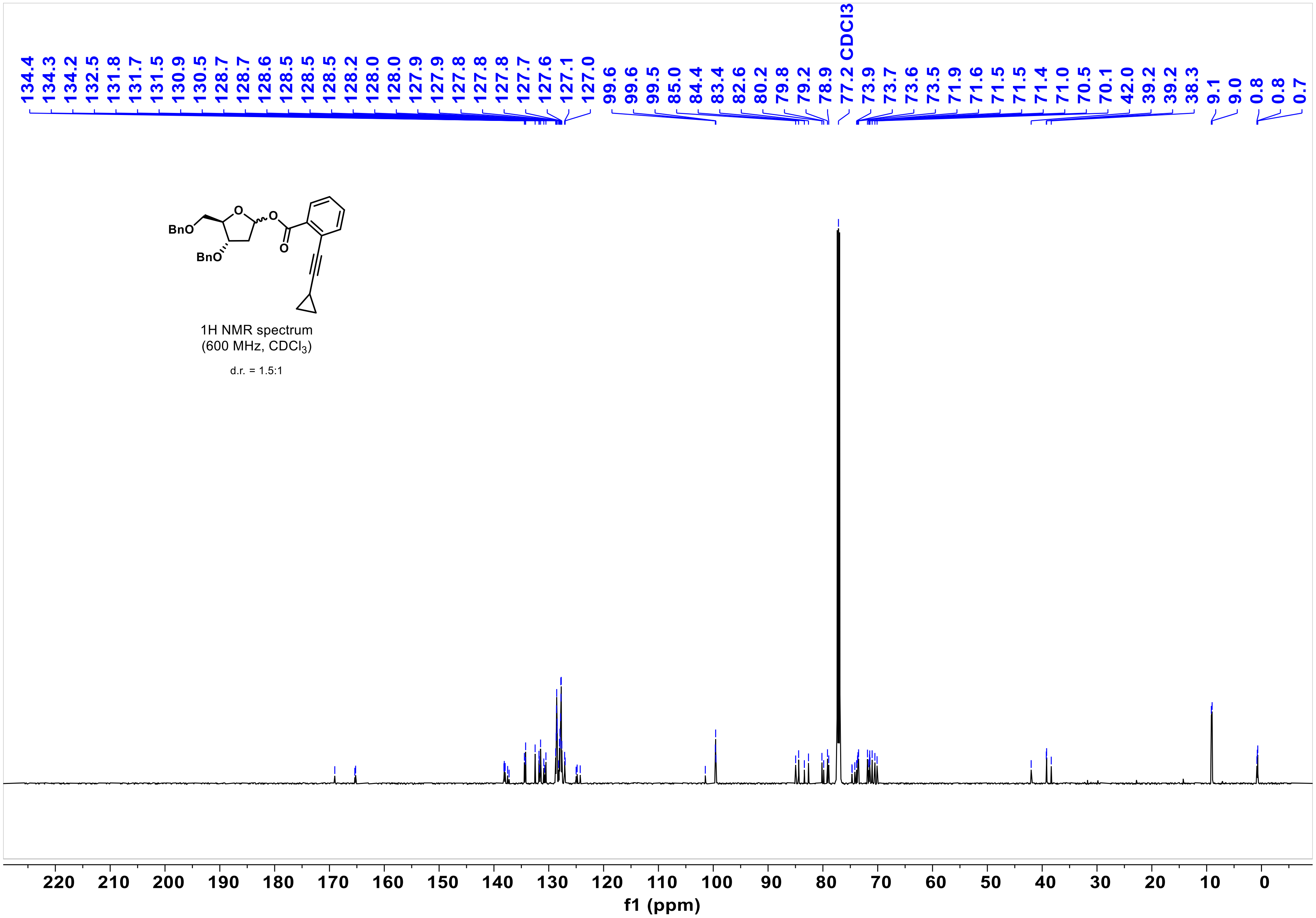

^{13}C NMR of compound **SE12**

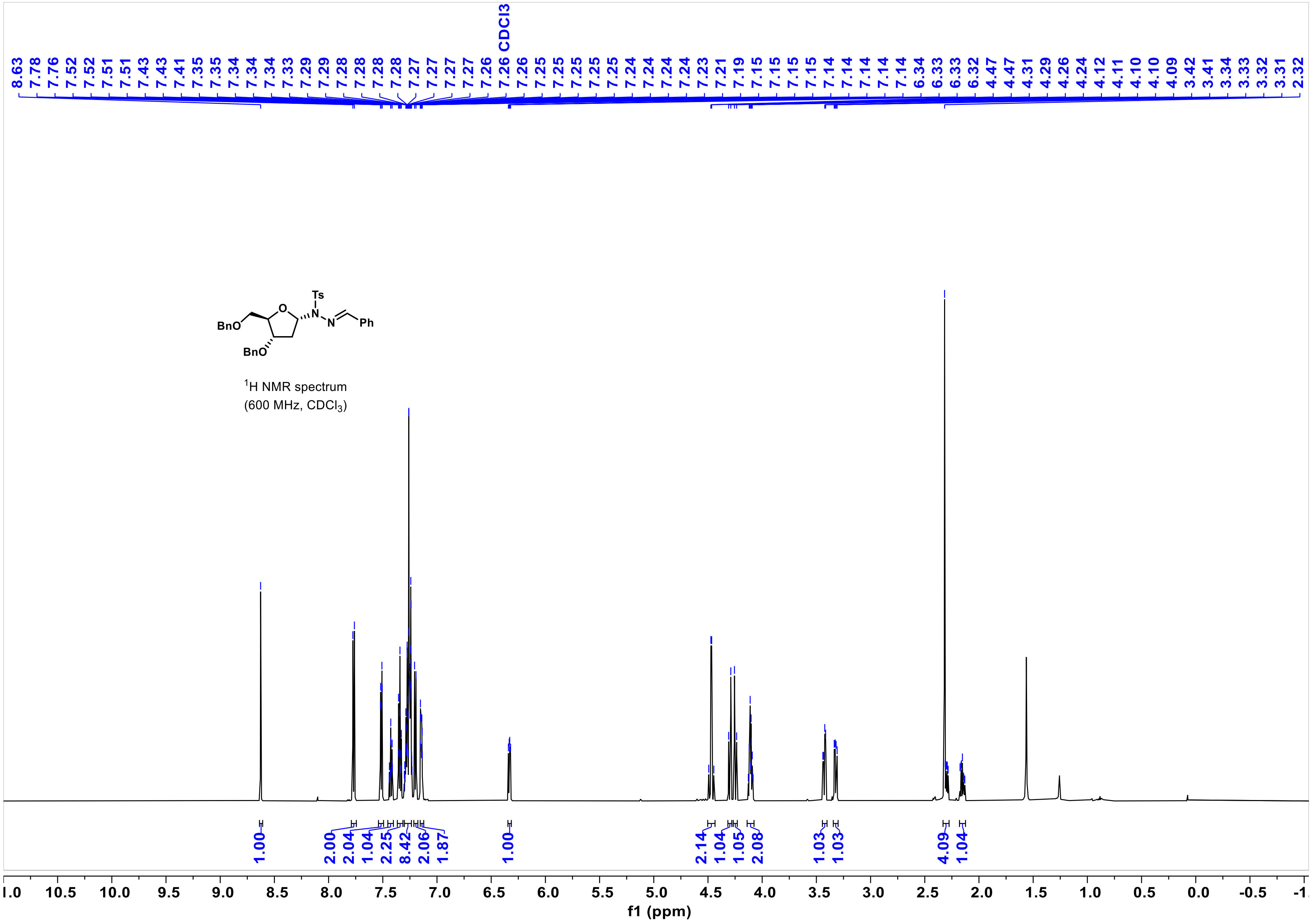

^{1}H NMR of compound **α-SE13**

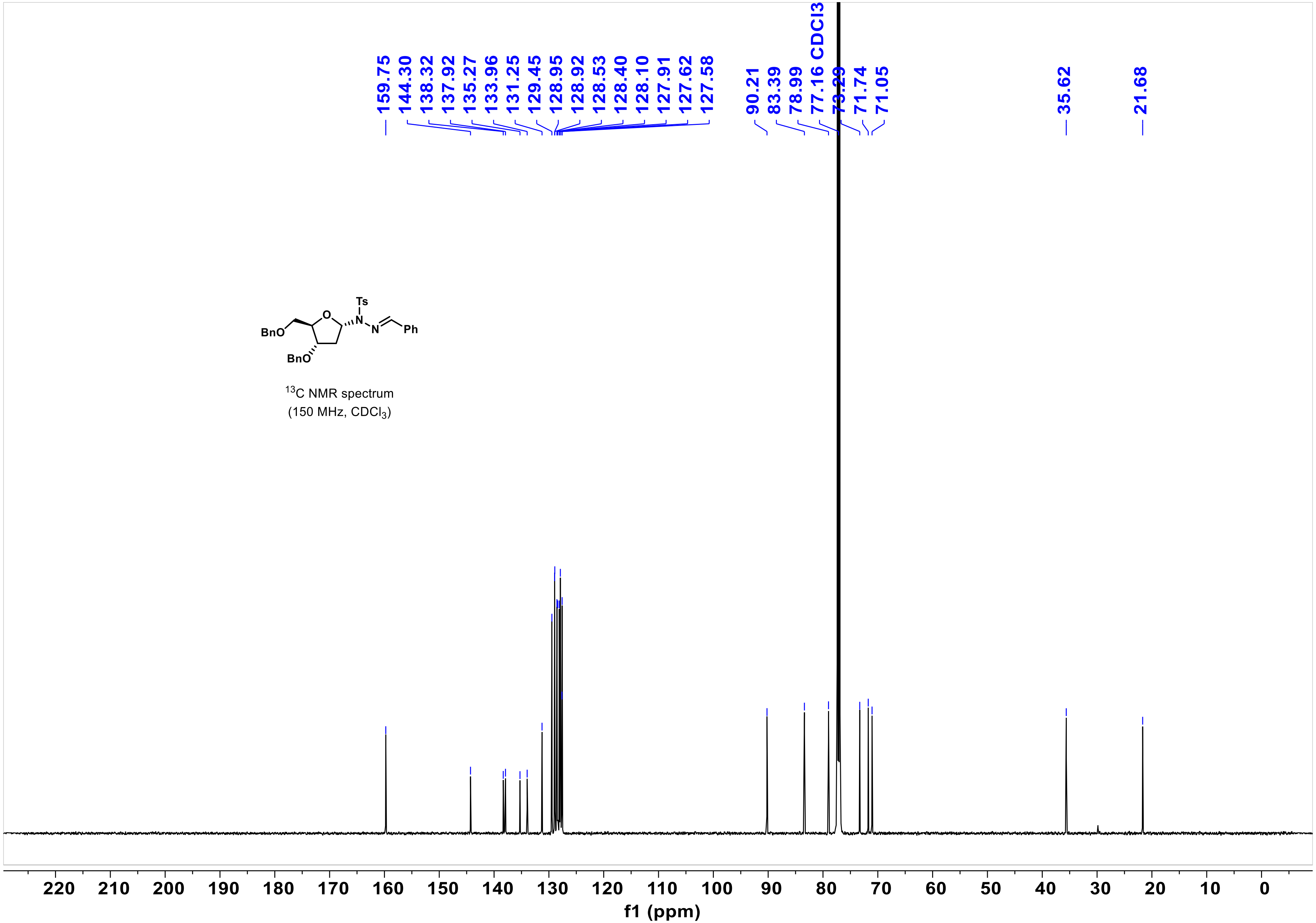

^{13}C NMR of compound **α-SE13**

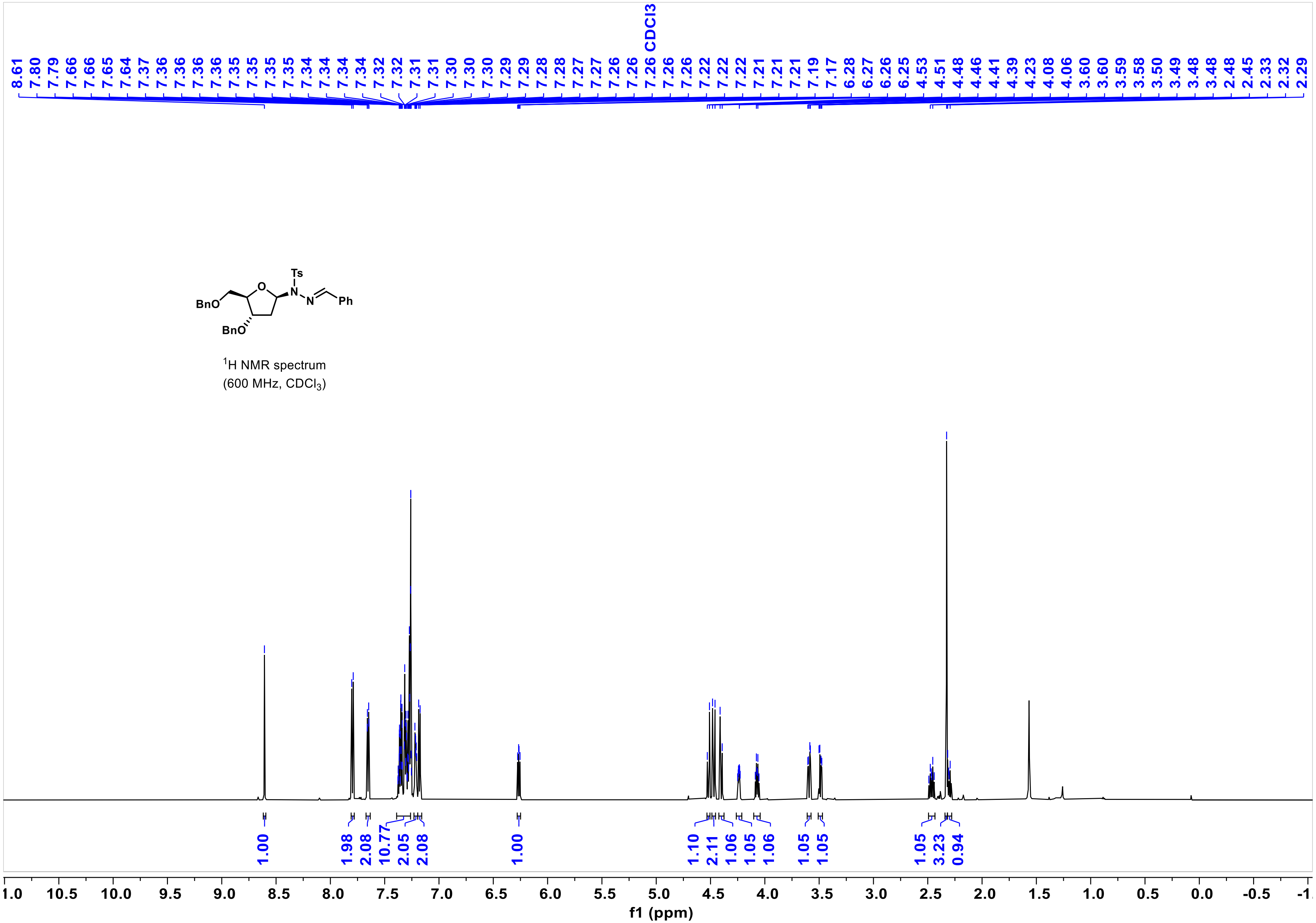

^{1}H NMR of compound **β-SE13**

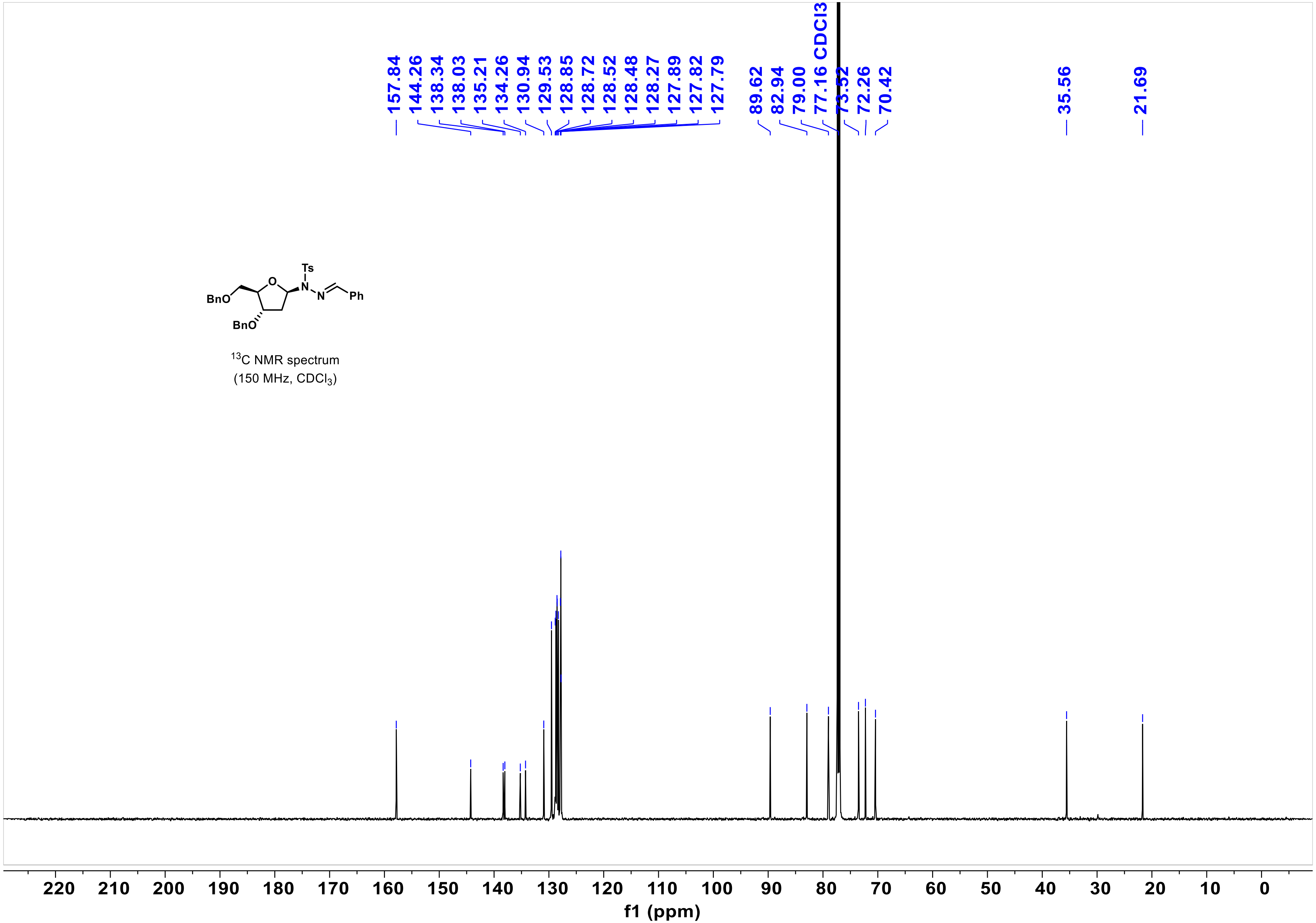

^{13}C NMR of compound **β-SE13**

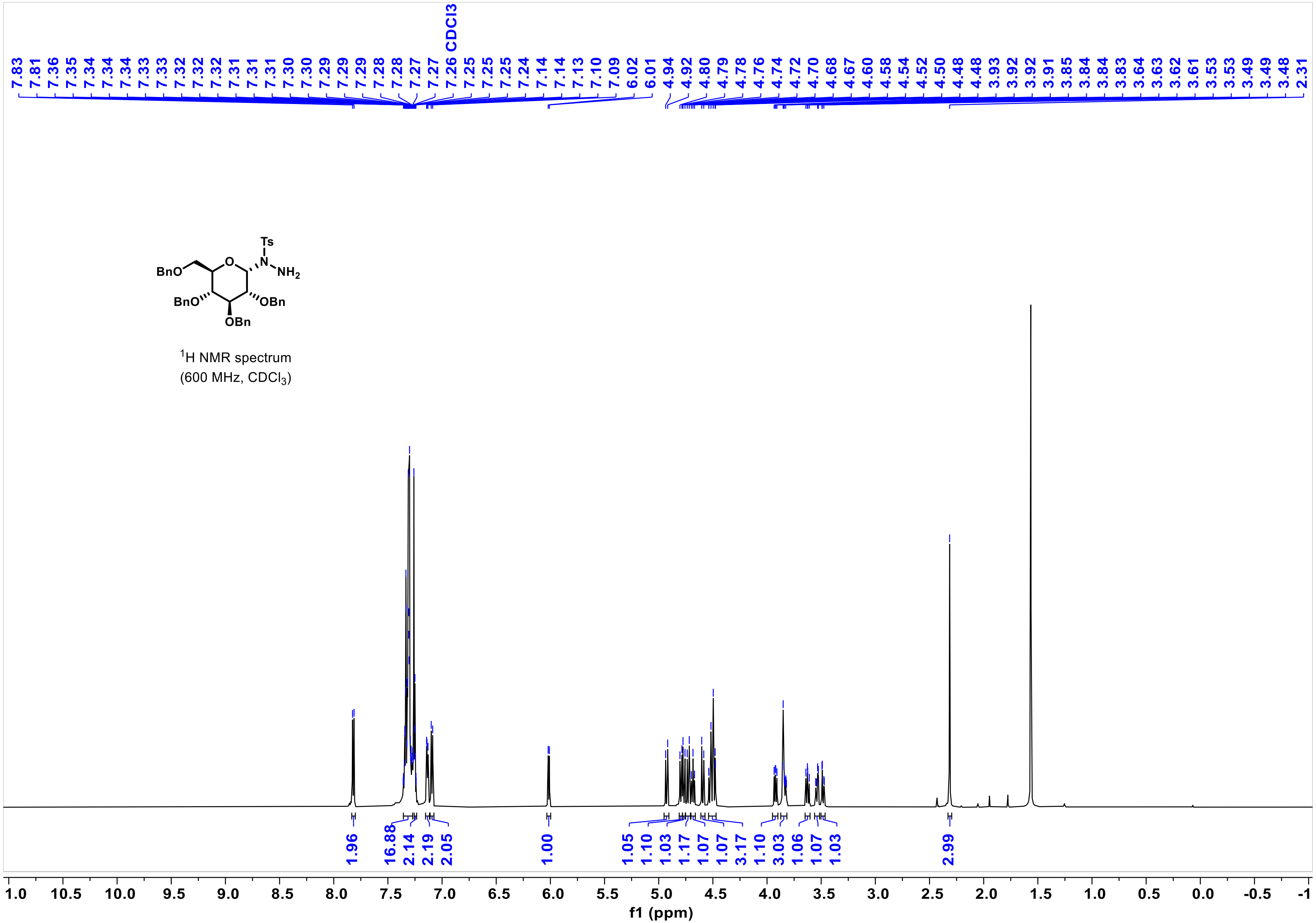

^{1}H NMR of compound **α-83**

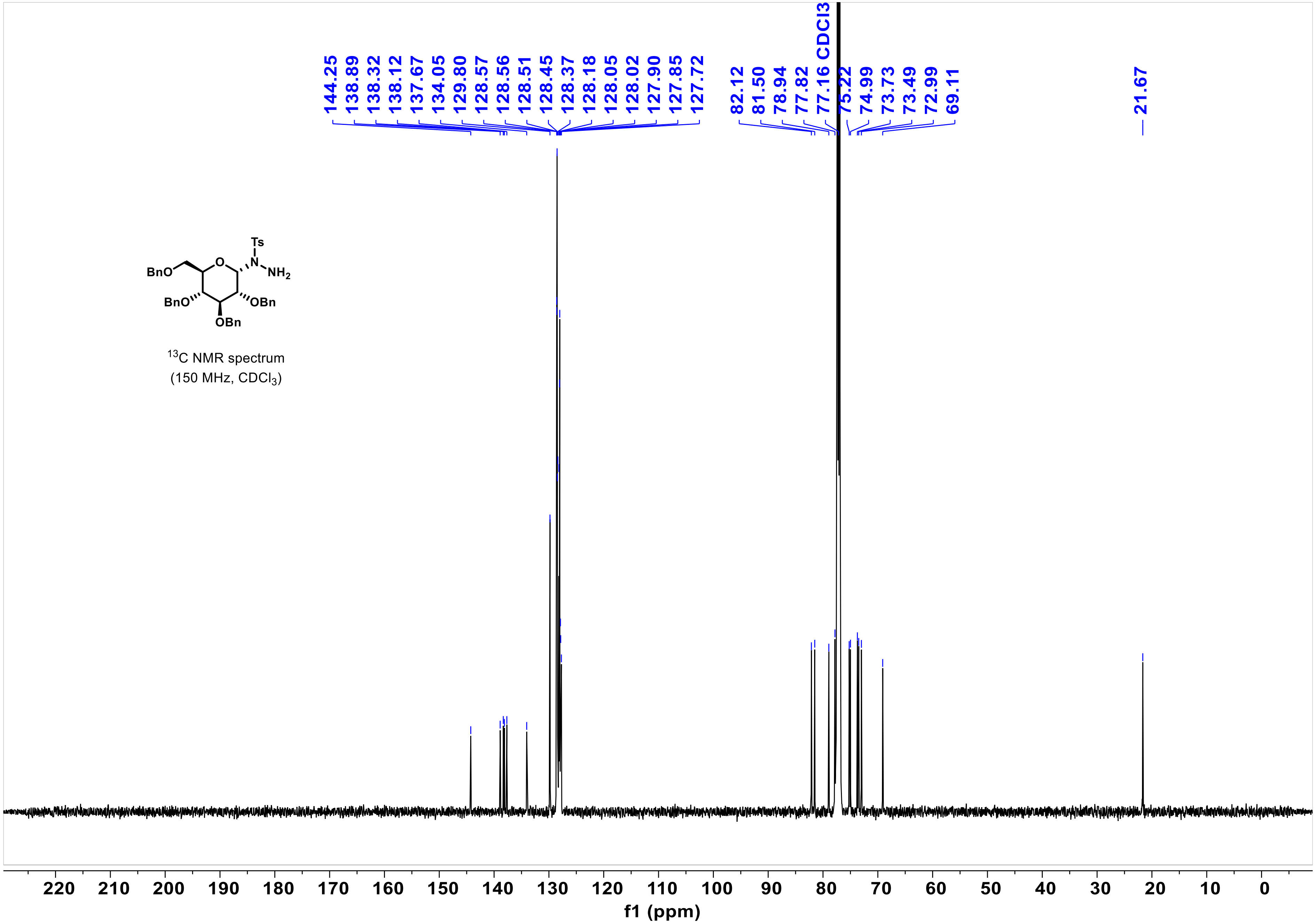

^{13}C NMR of compound **α-83**

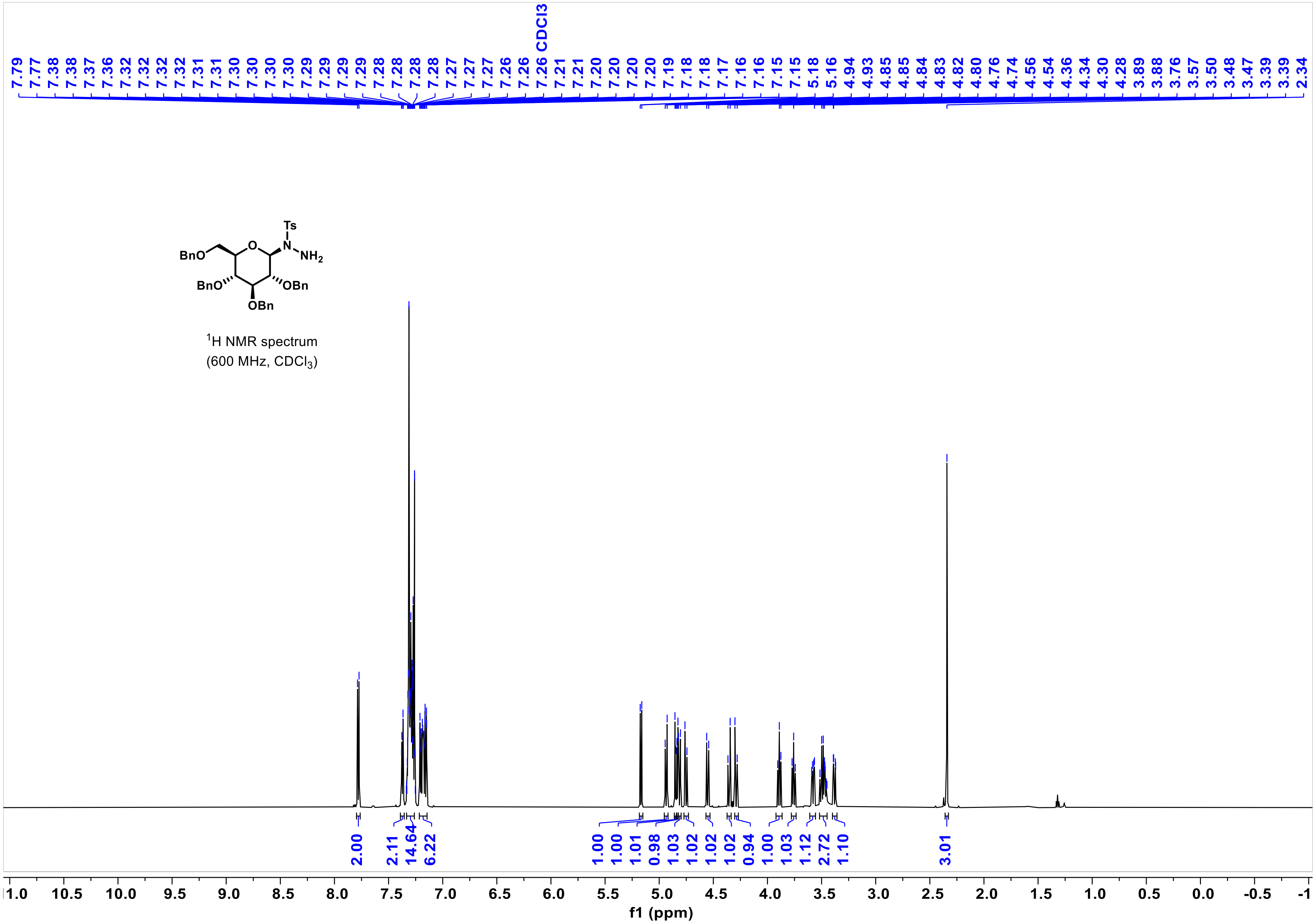

^{1}H NMR of compound **β-83**

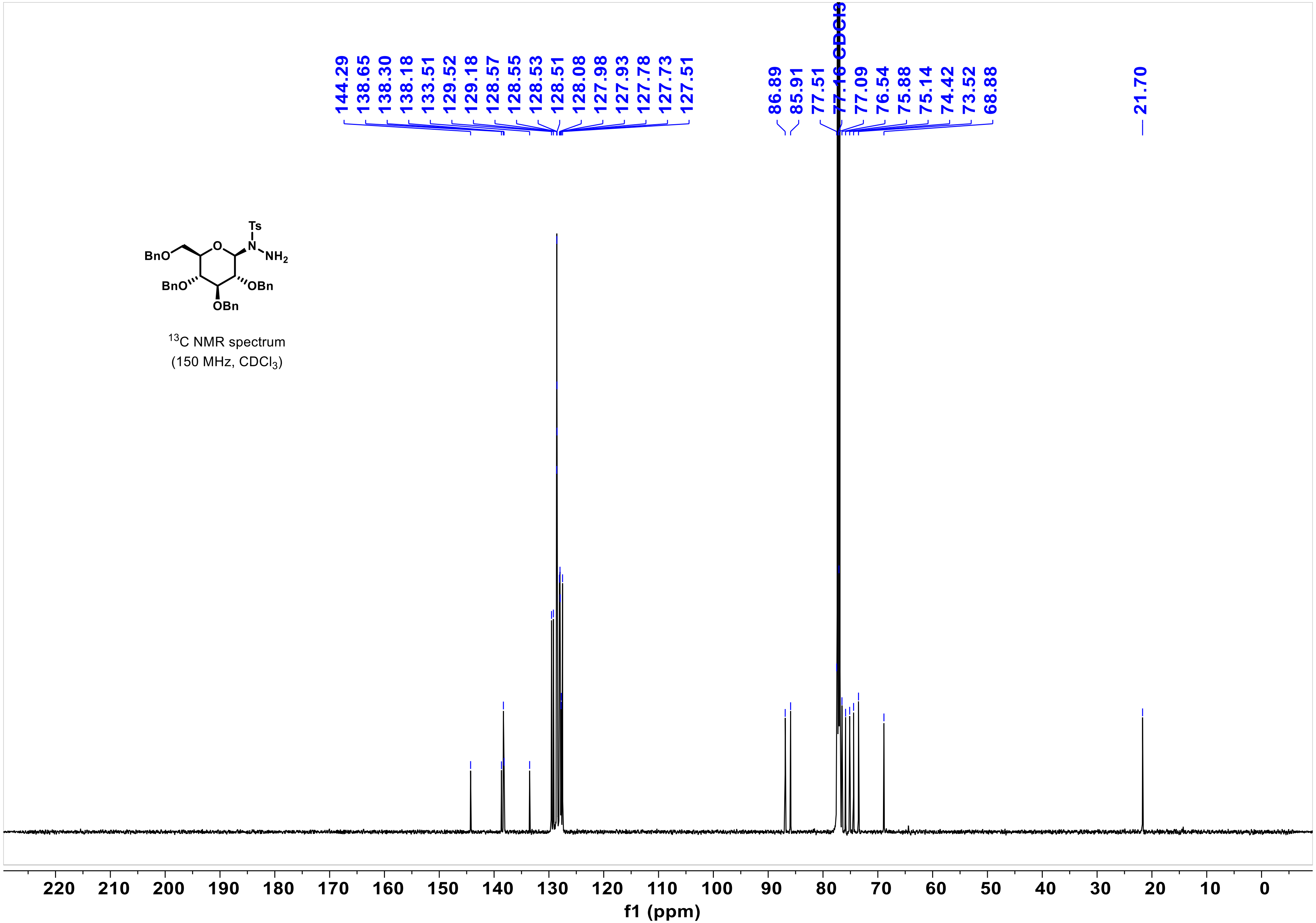

^{13}C NMR of compound **β-83**

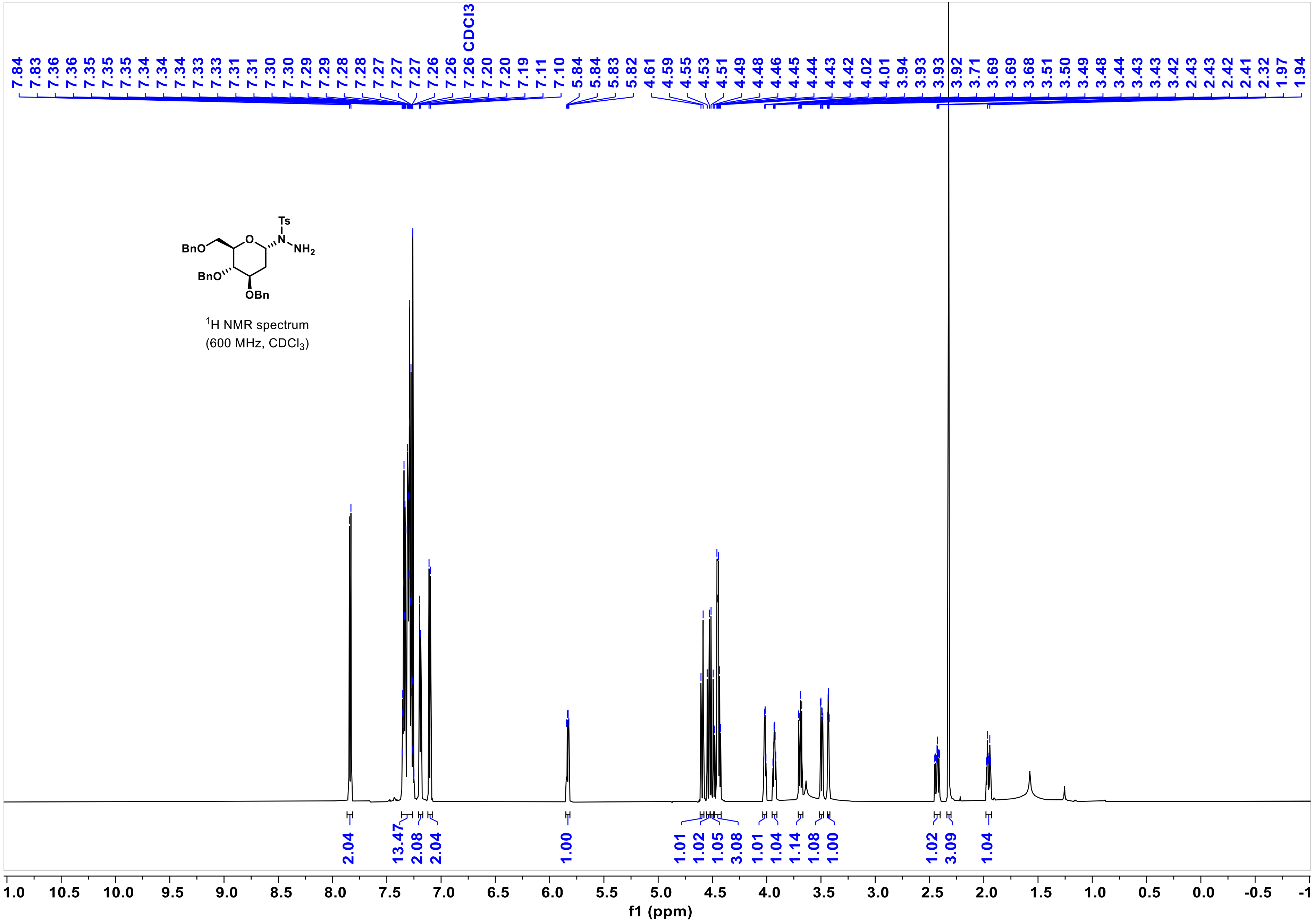

^{1}H NMR of compound **α-87**

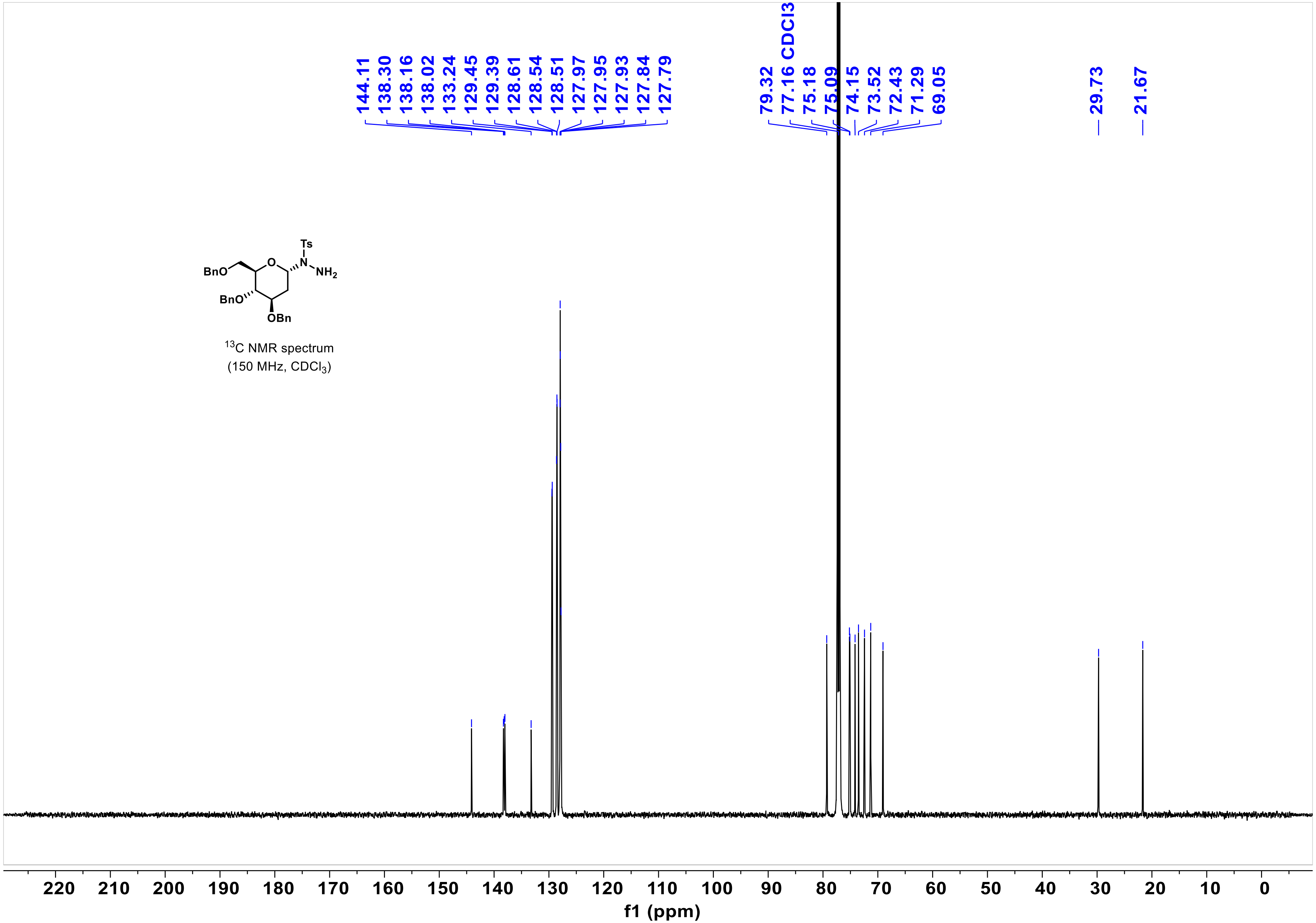

^{13}C NMR of compound **α-87**

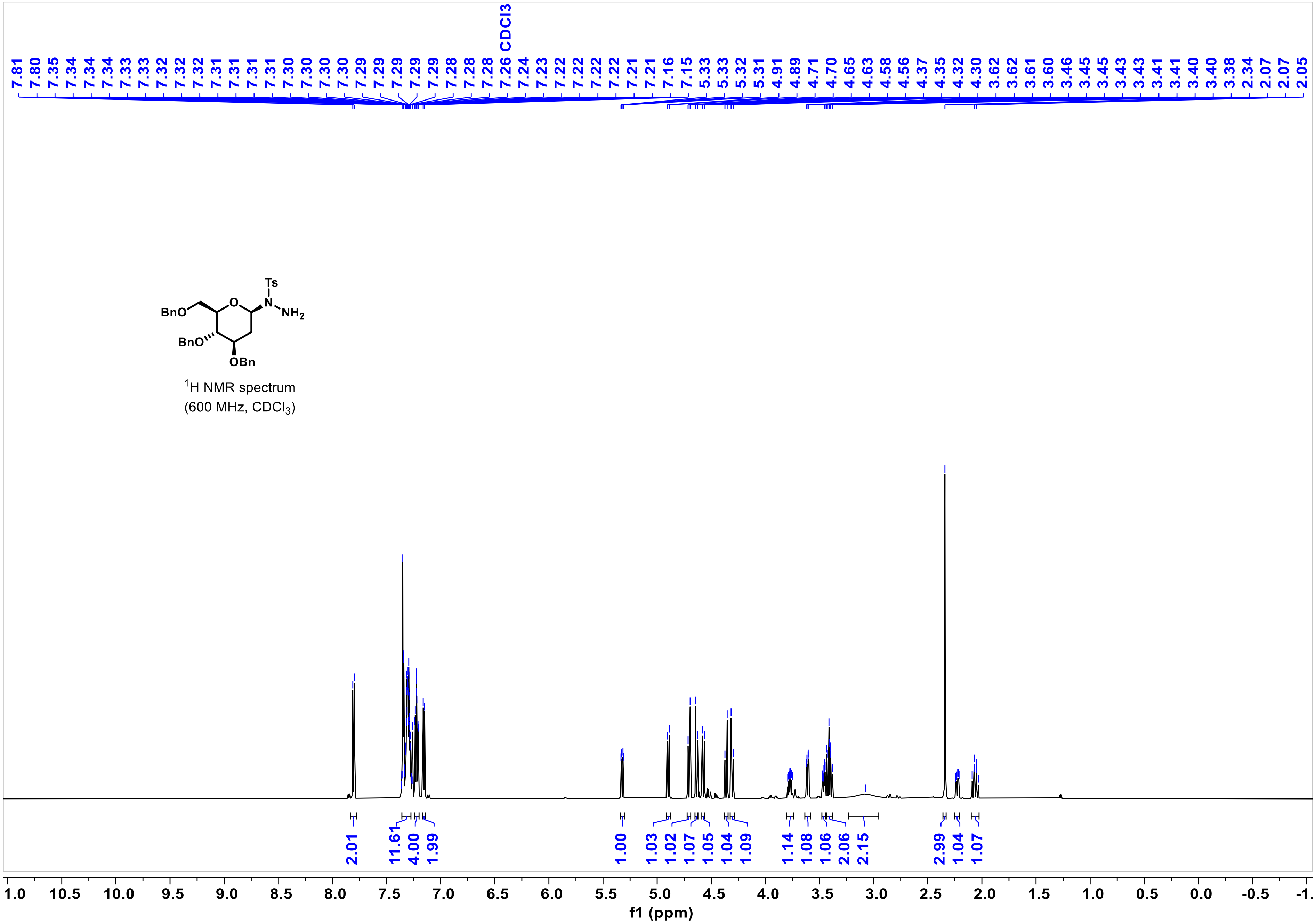

^{1}H NMR of compound **β-87**

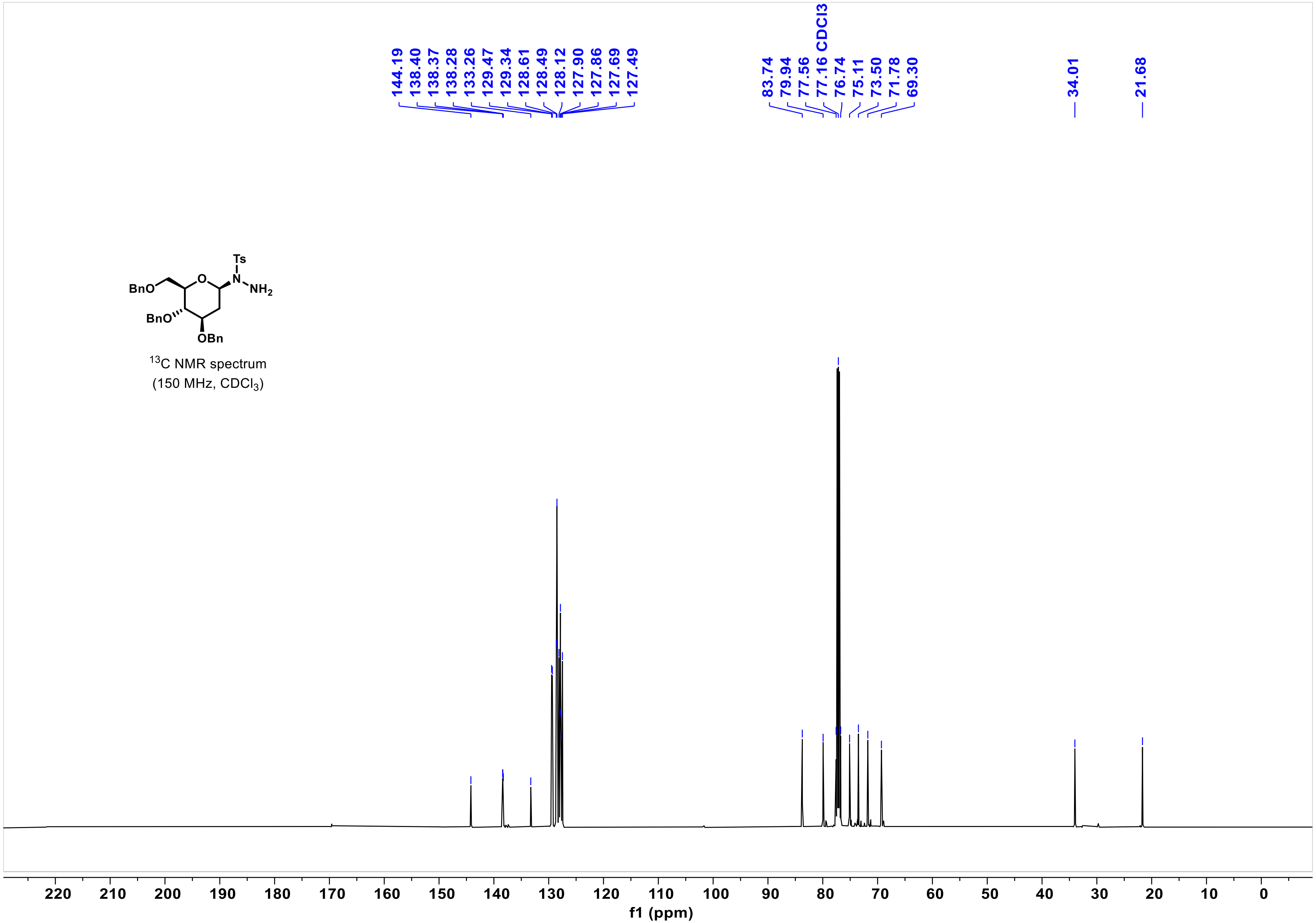

^{13}C NMR of compound **β-87**

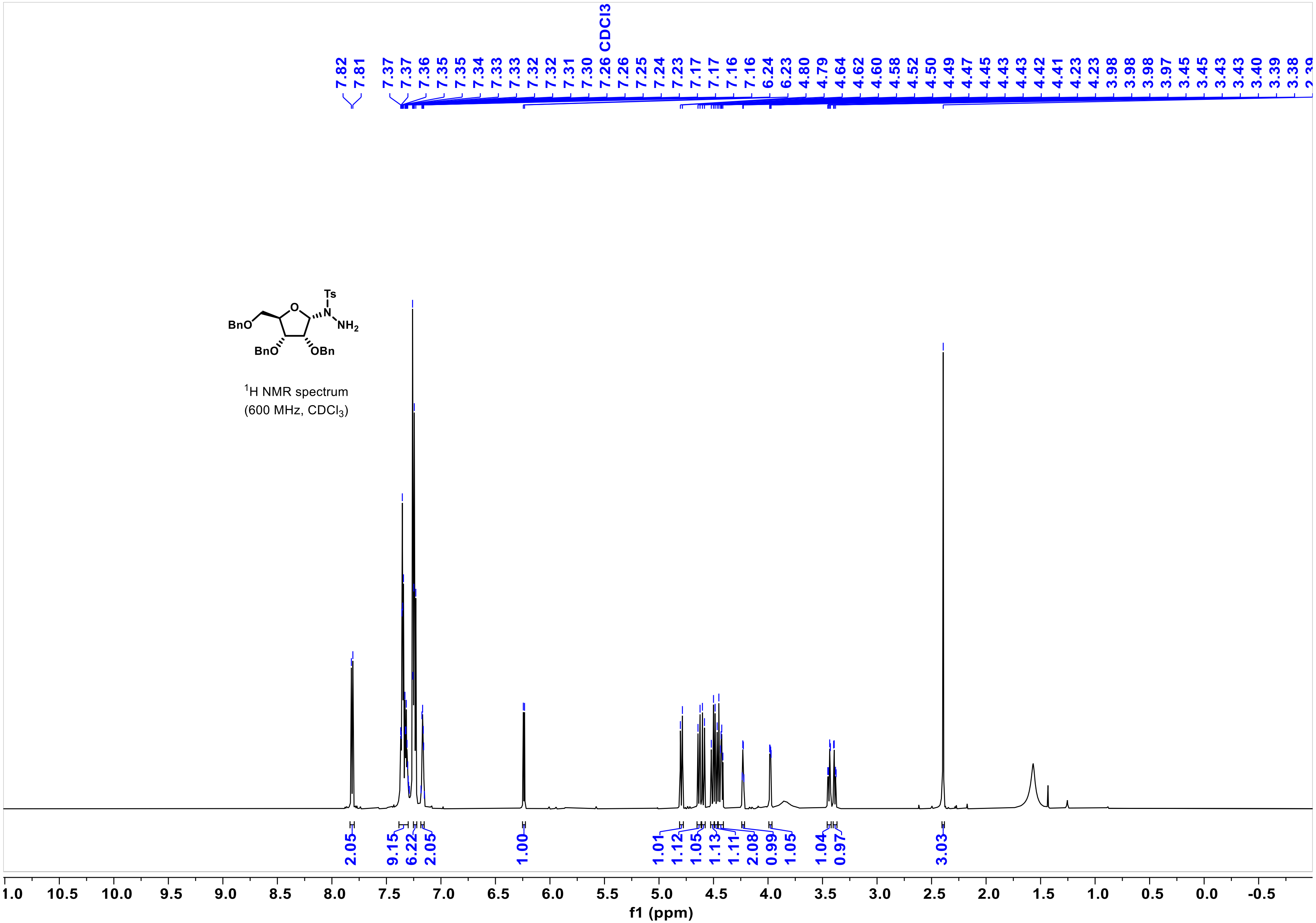

^{1}H NMR of compound **α-89**

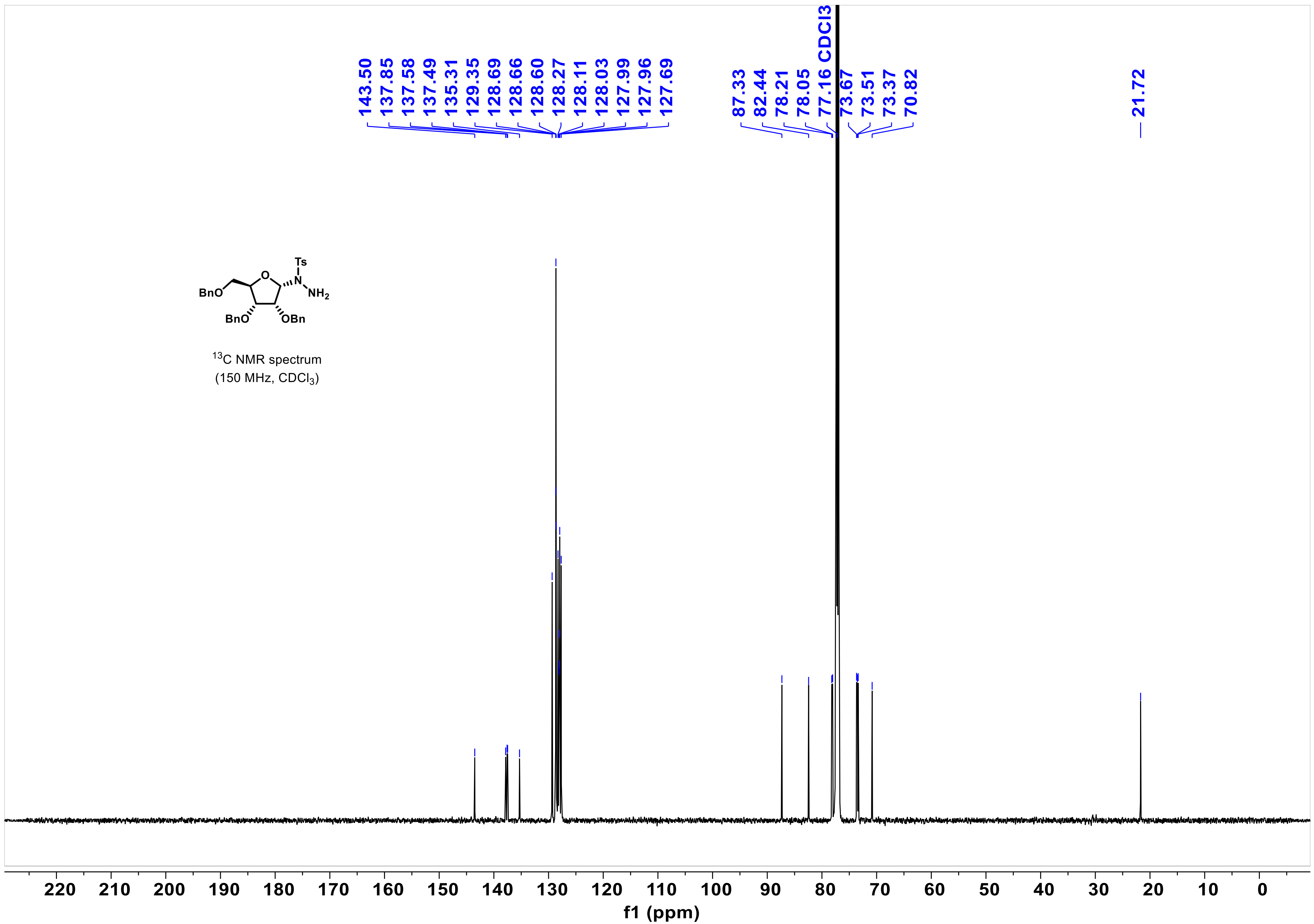

^{13}C NMR of compound **α-89**

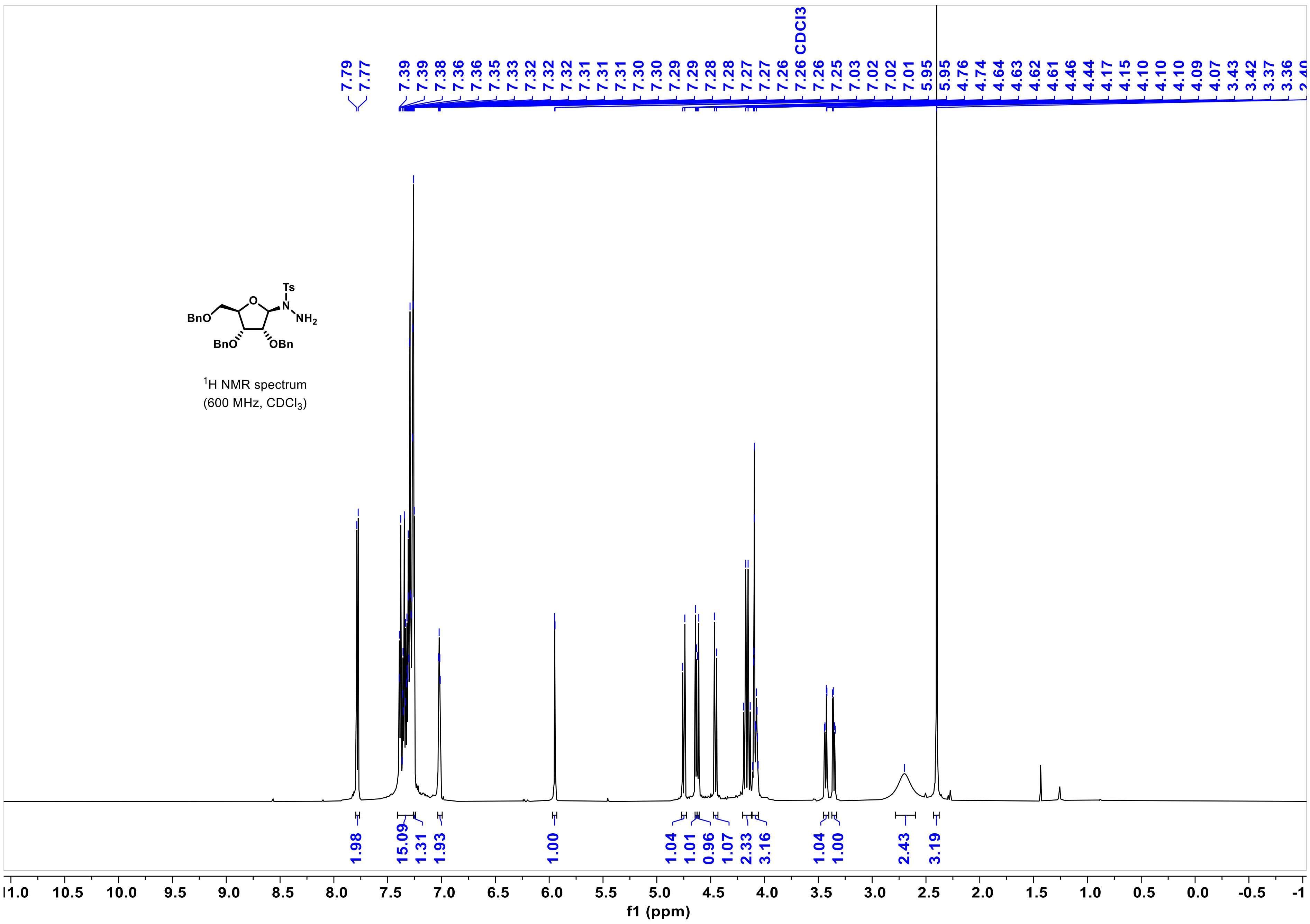

^{1}H NMR of compound **β-89**

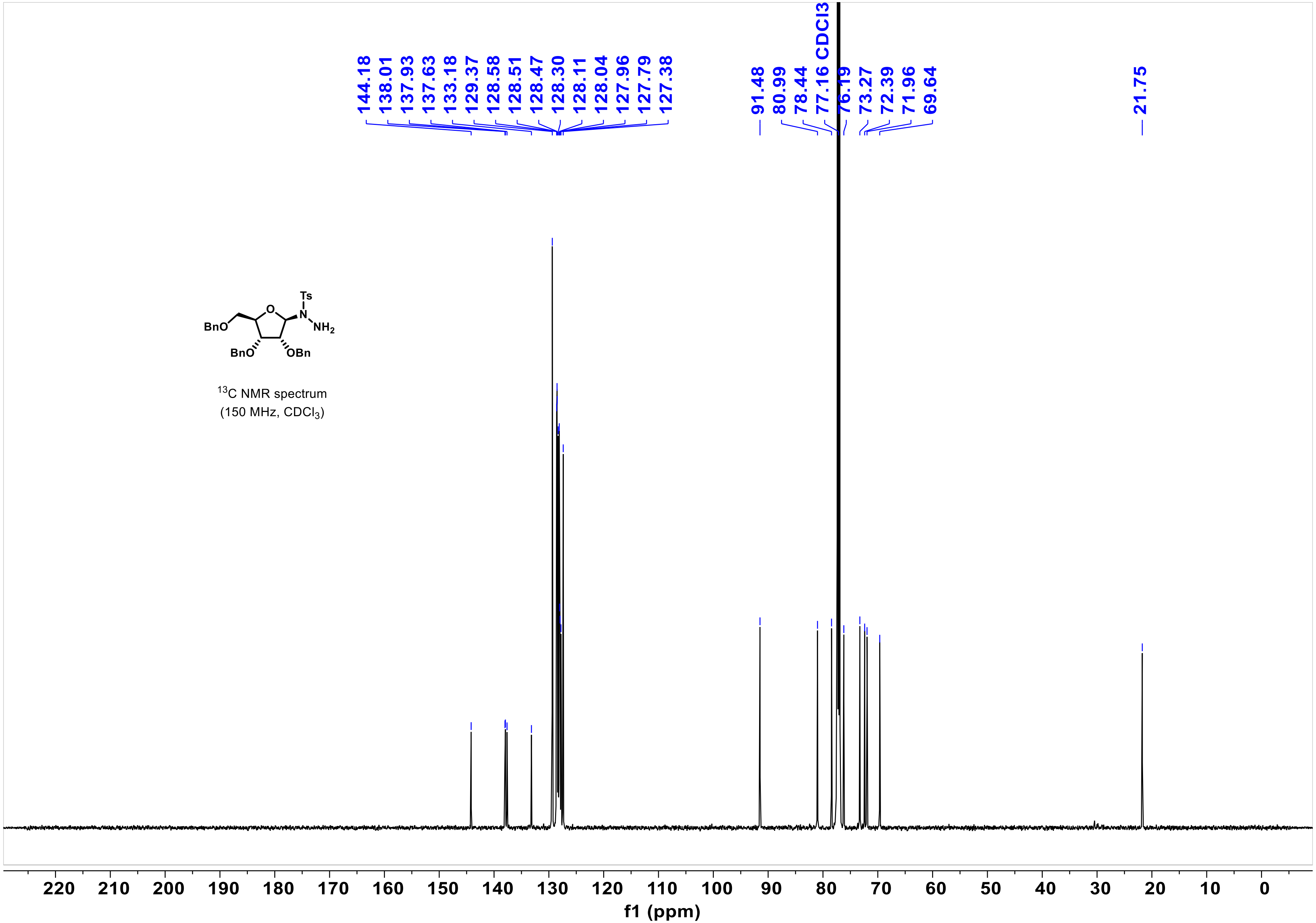

^{13}C NMR of compound **β-89**

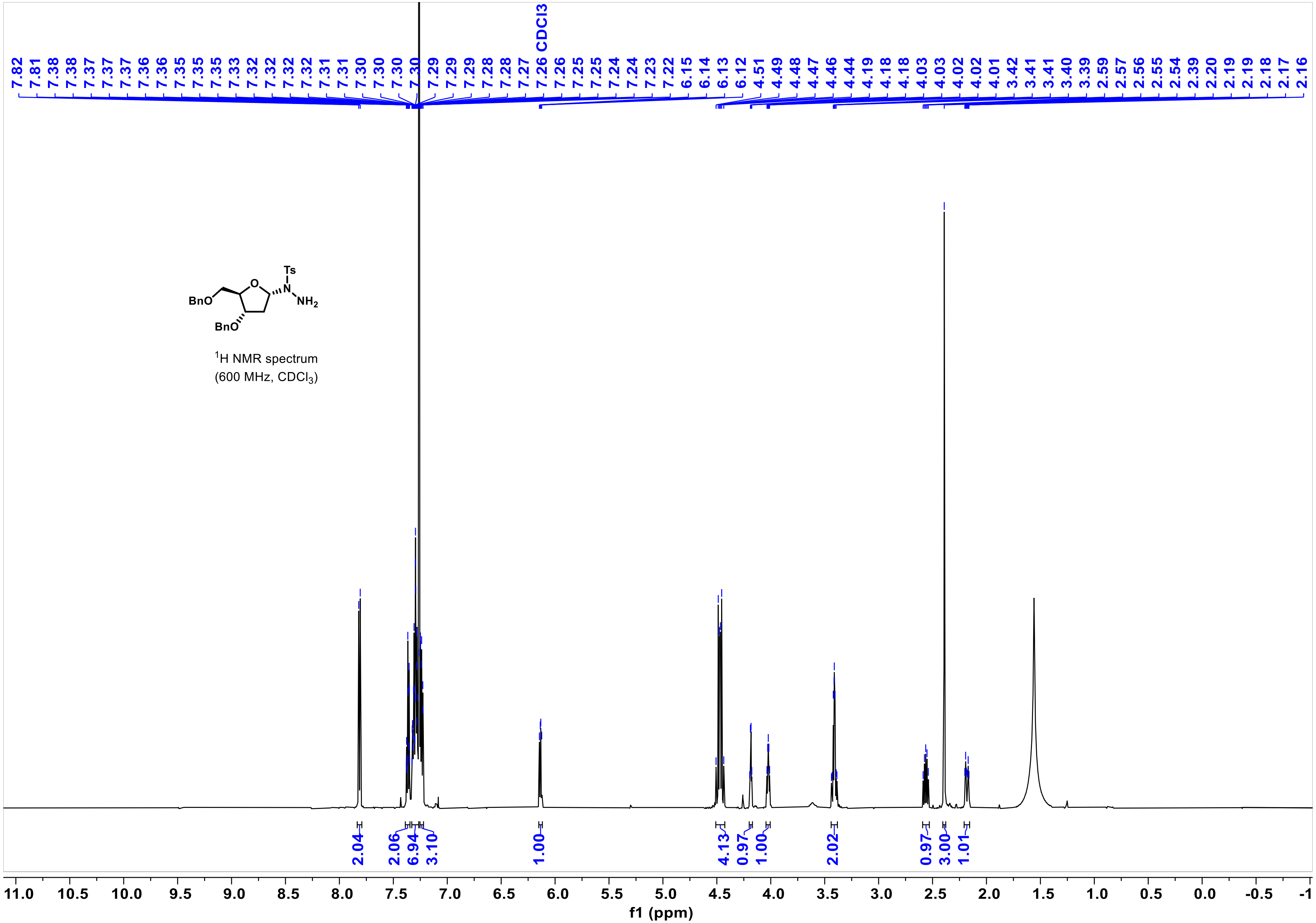

^{1}H NMR of compound **α-91**

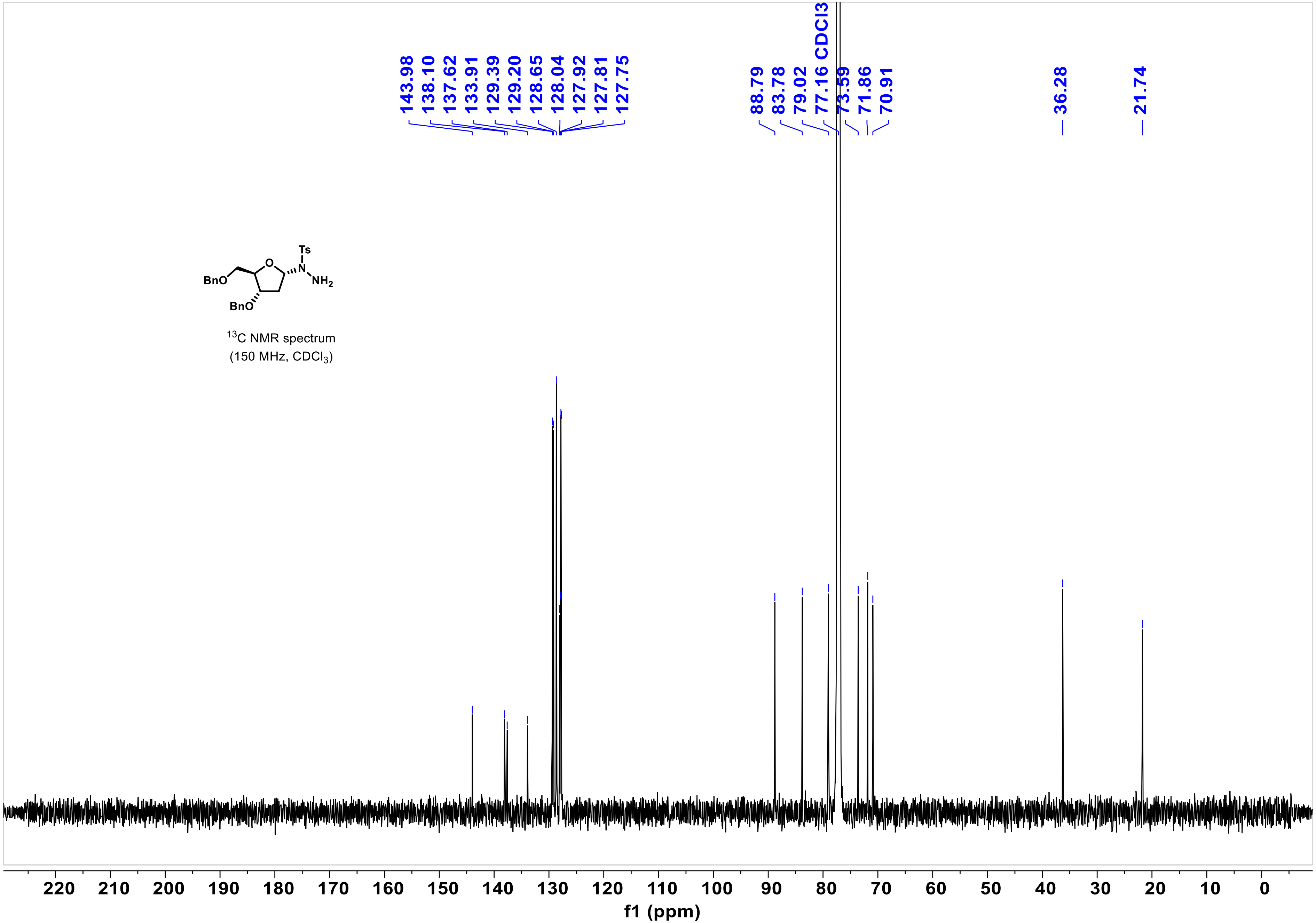

^{13}C NMR of compound **α-91**

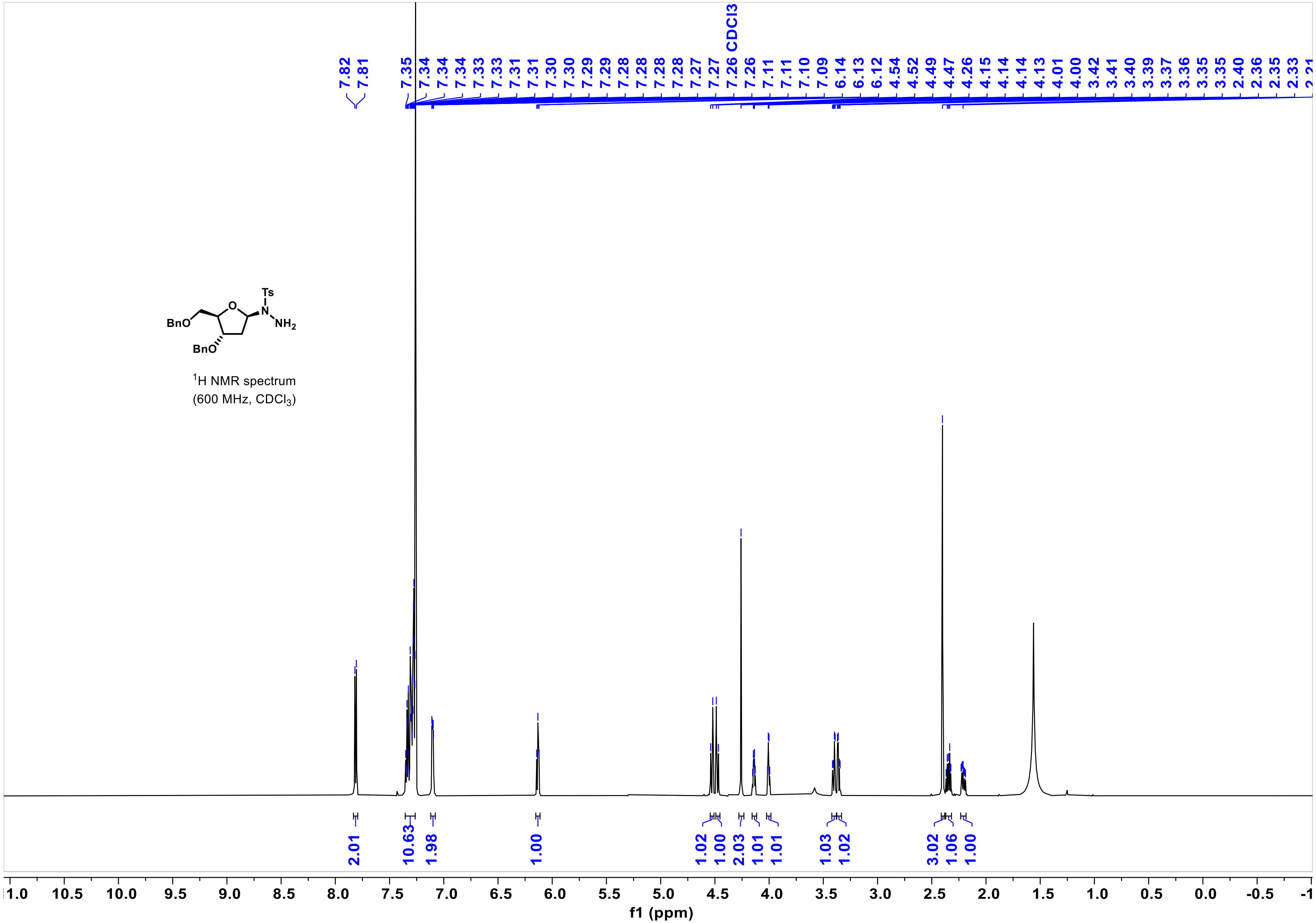

^{1}H NMR of compound **β-91**

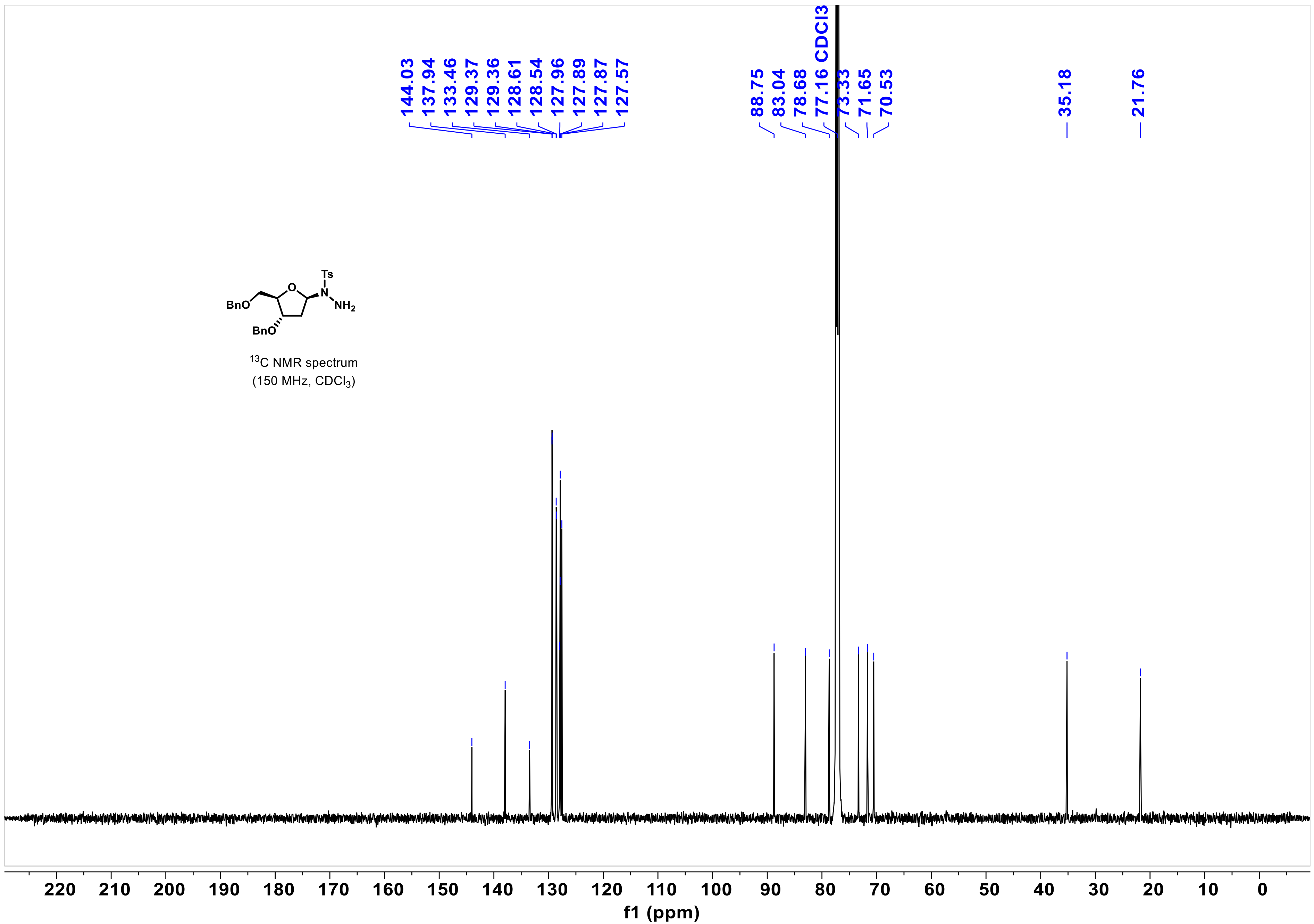

^{13}C NMR of compound **β-91**

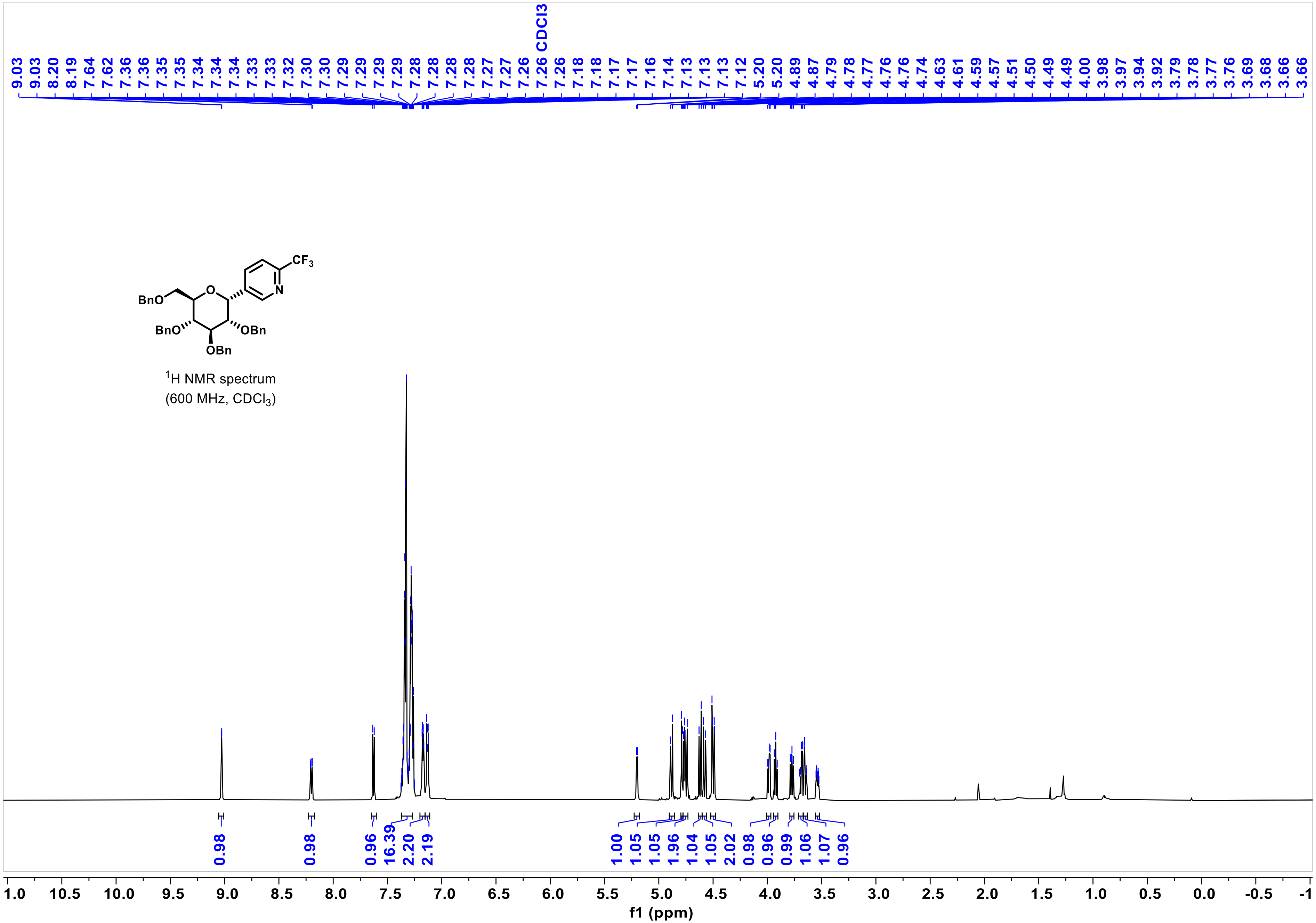

^{1}H NMR of compound **α-85**

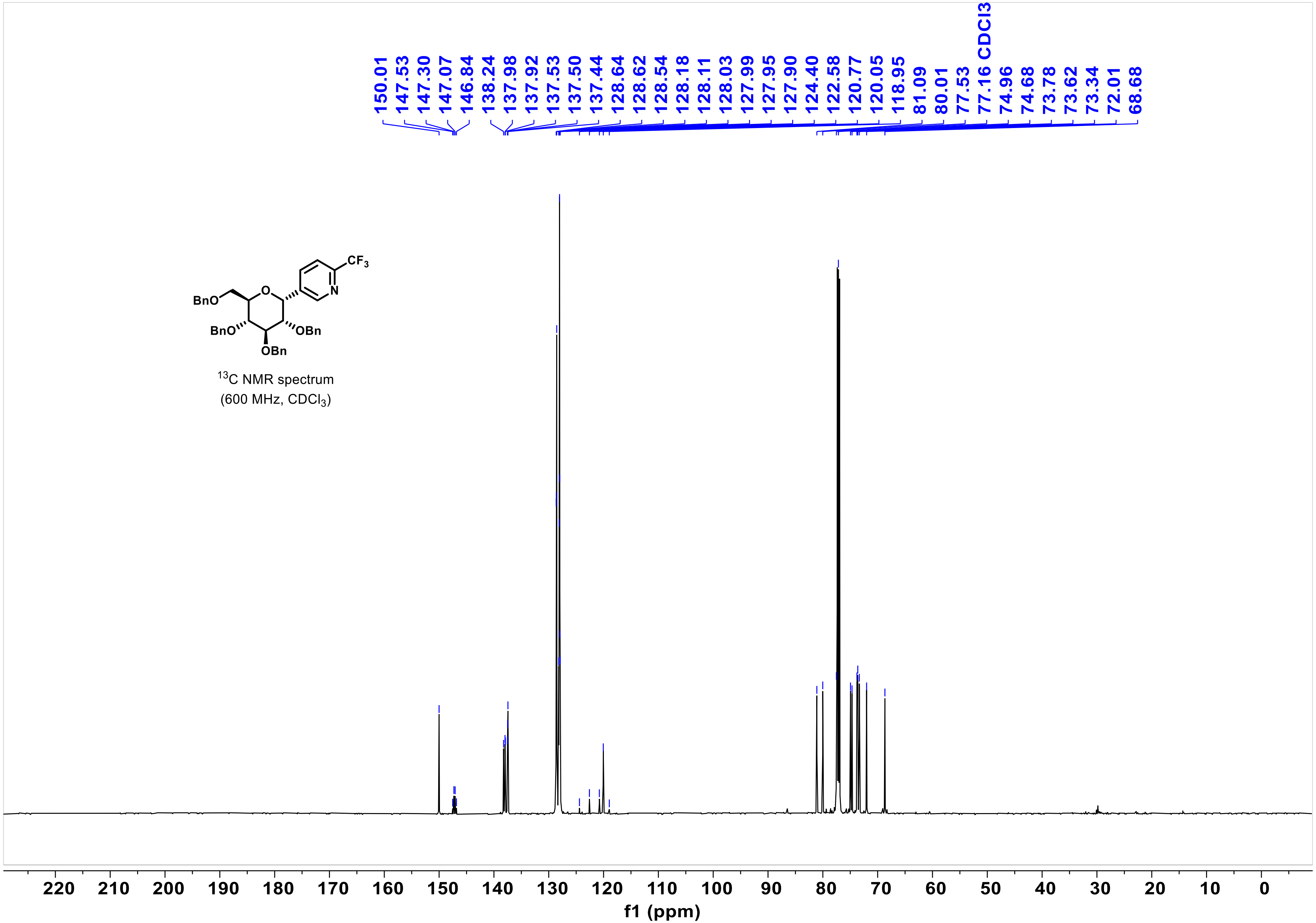

^{13}C NMR of compound **α-85**

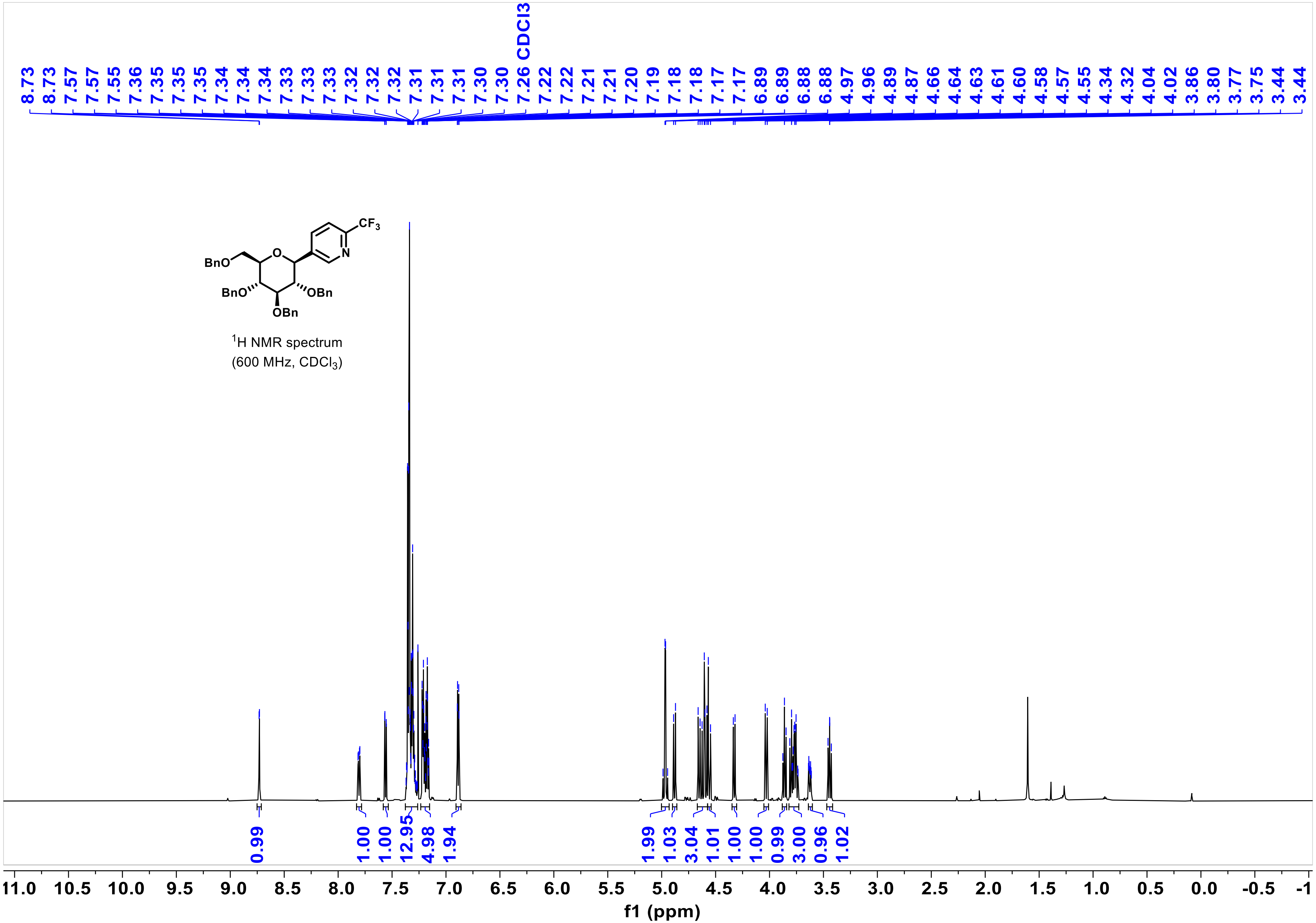

^{1}H NMR of compound **β-85**

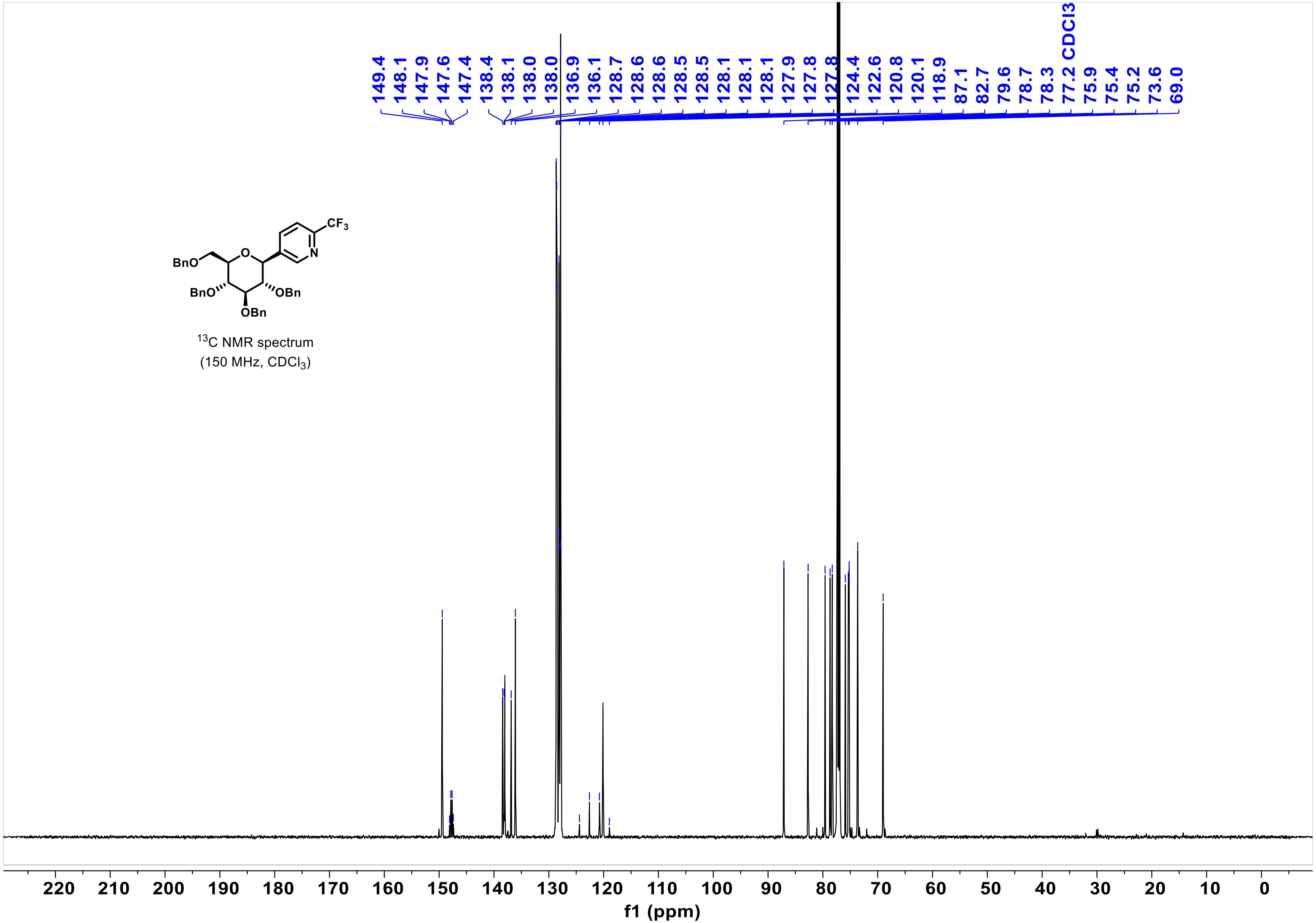

^{13}C NMR of compound **β-85**

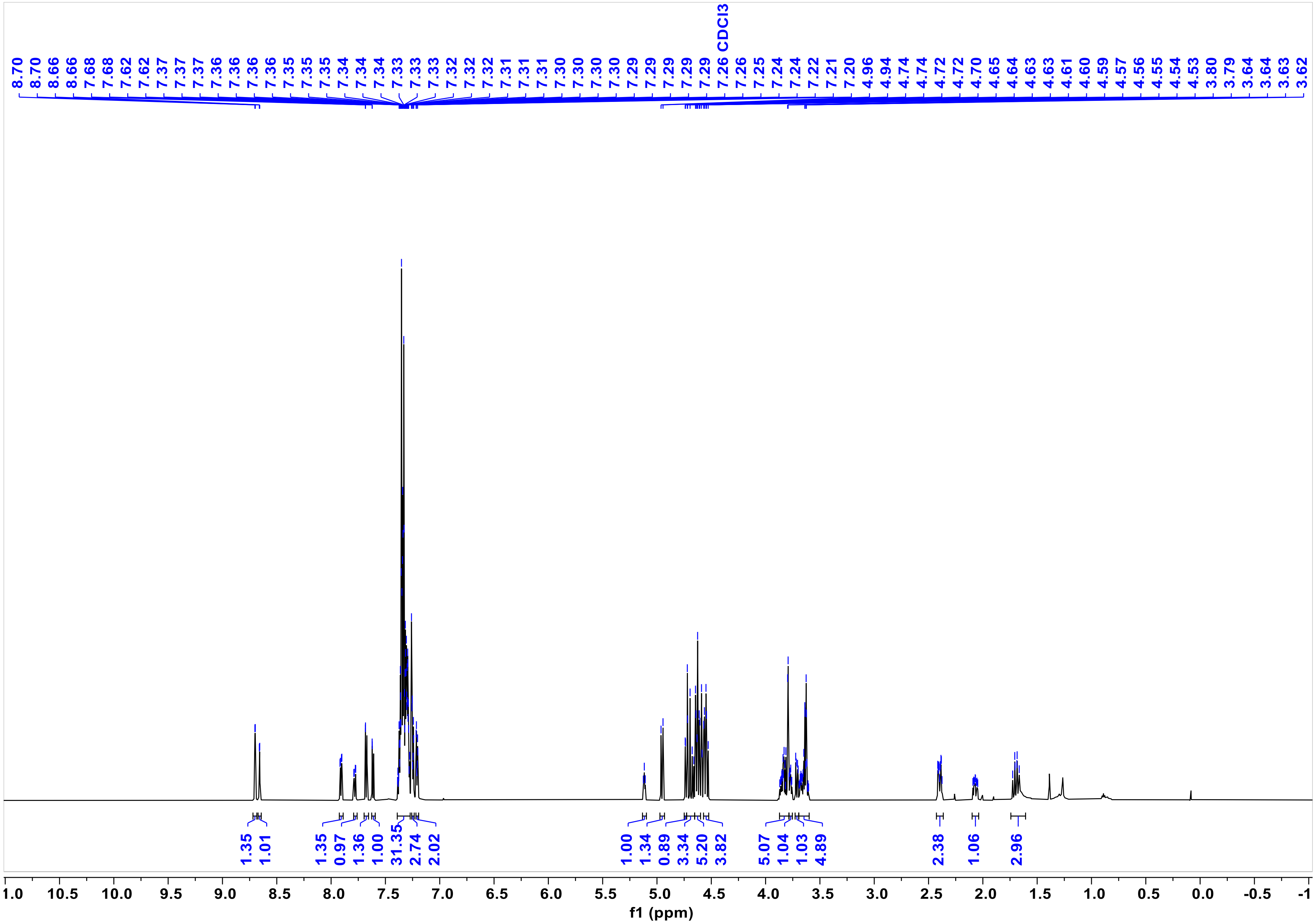

^{1}H NMR of compound **88** (α:β = 2:3)

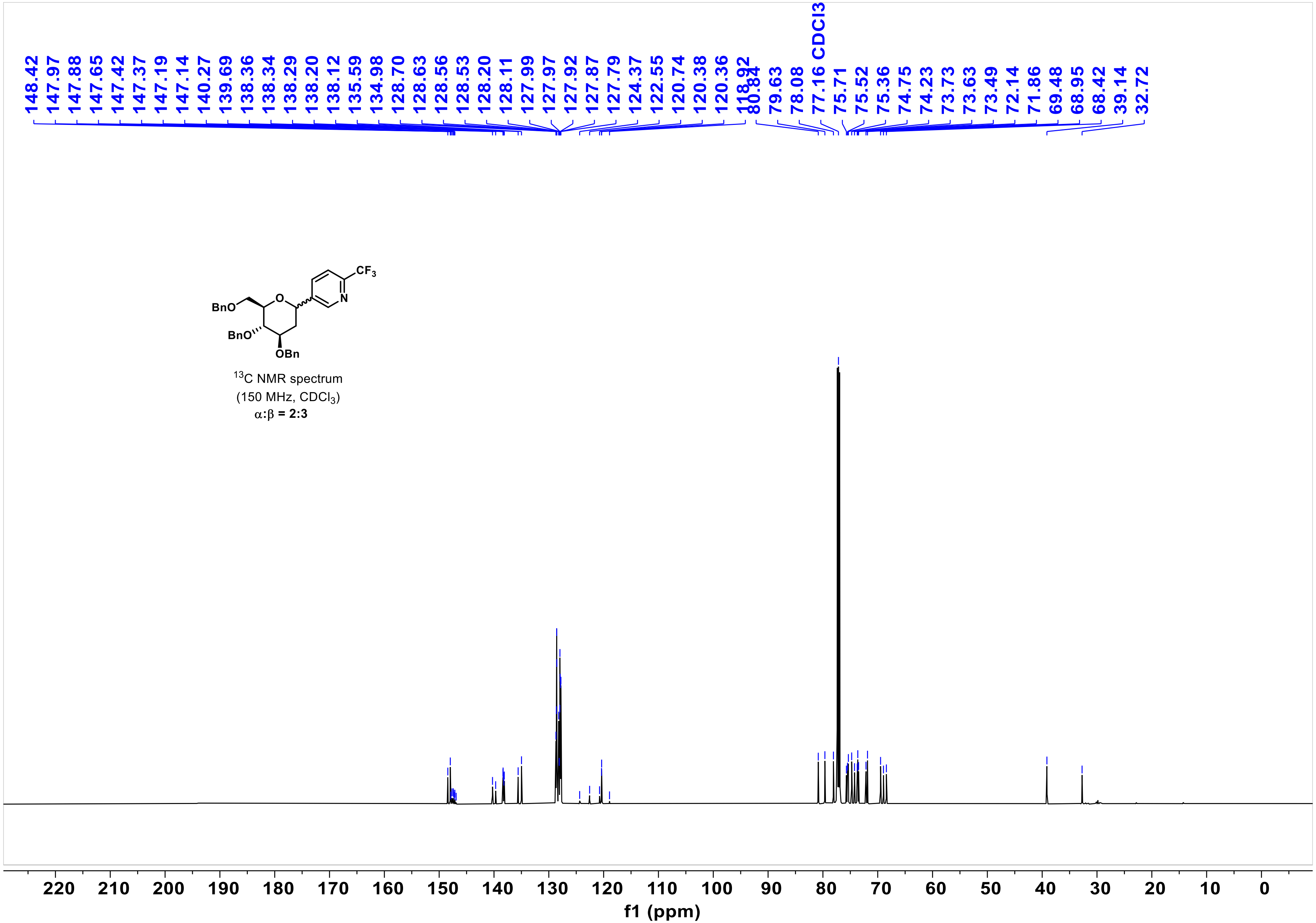

^{13}C NMR of compound **88** (α:β = 2:3)

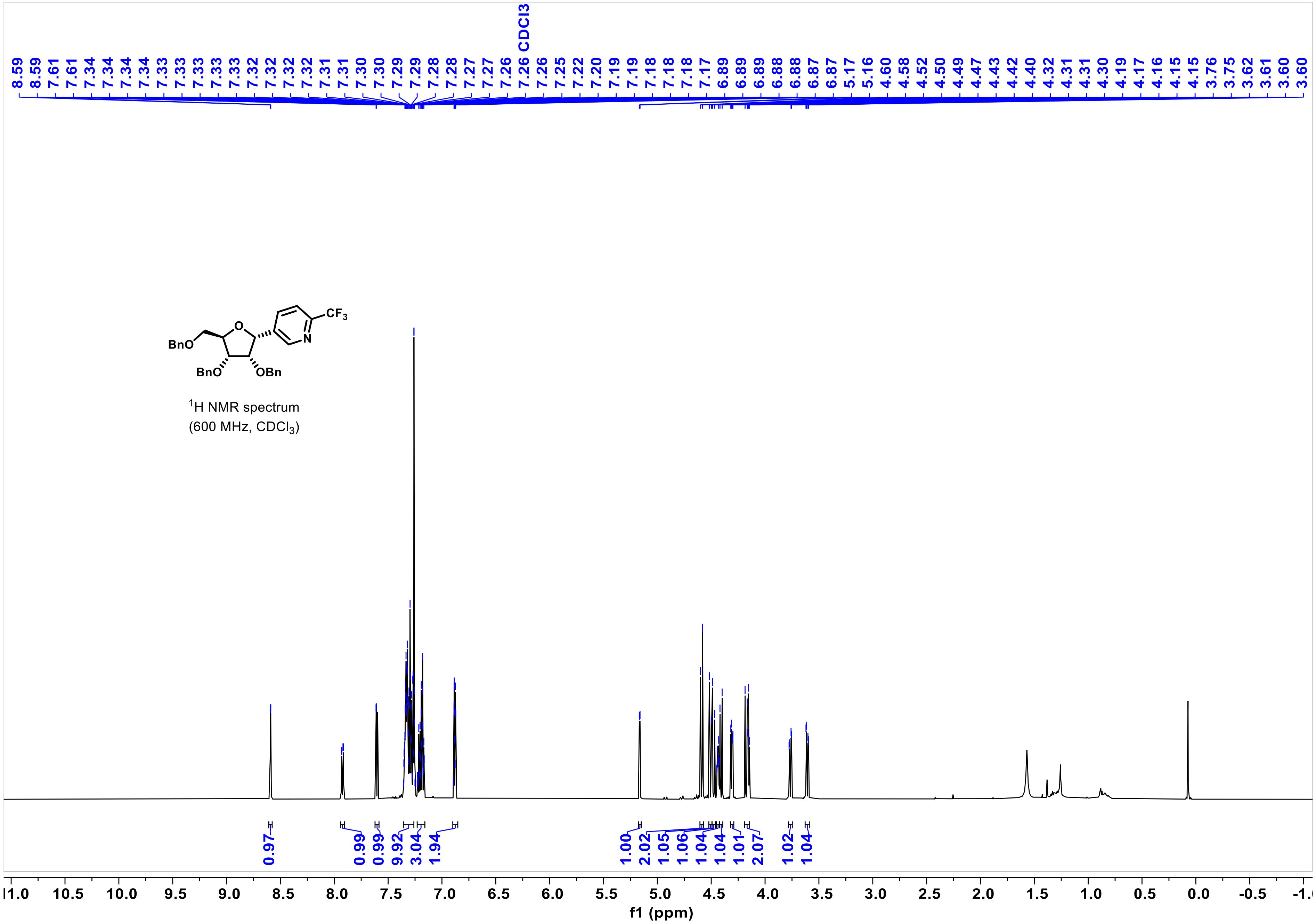

^{1}H NMR of compound **α-90**

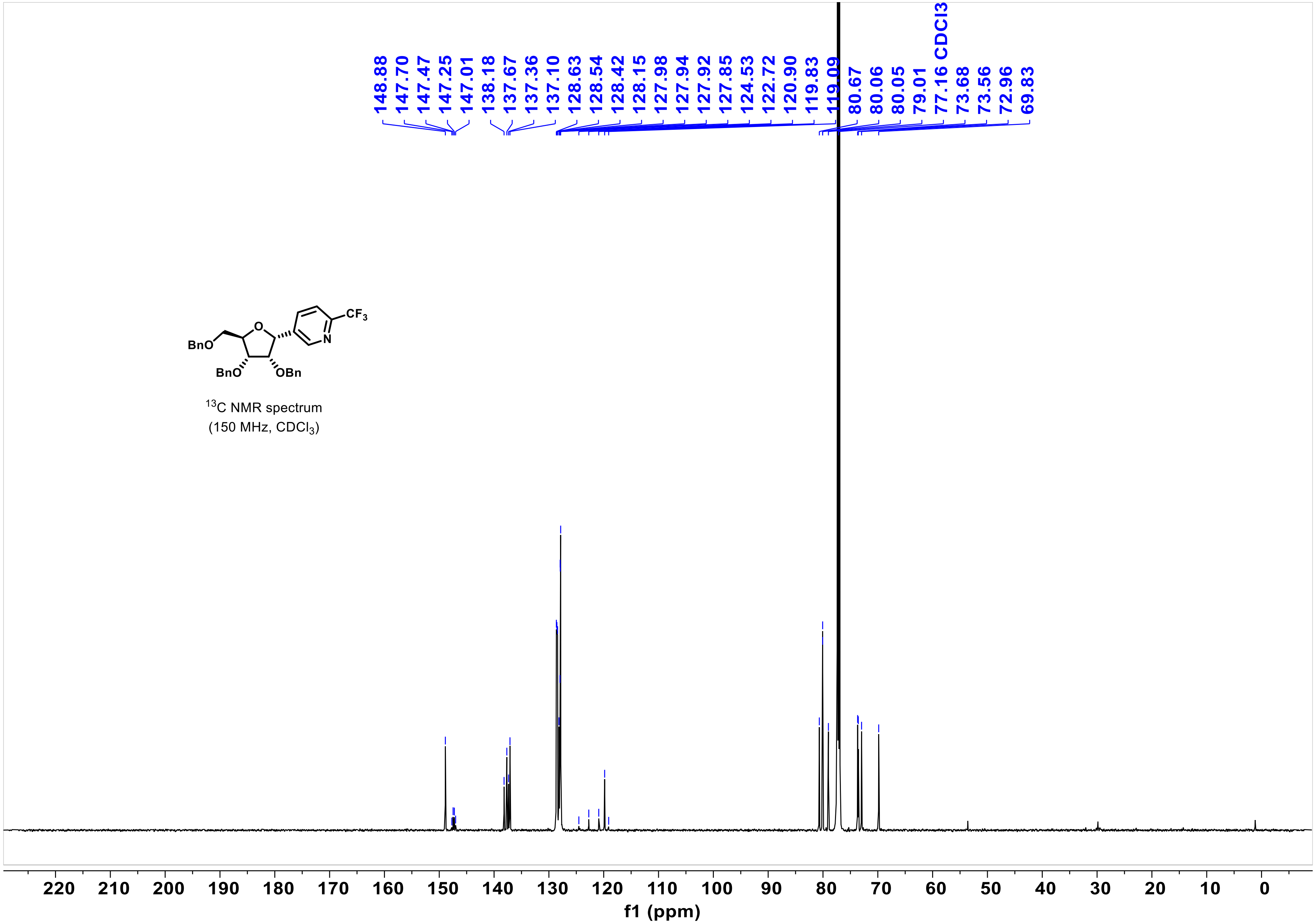

^{13}C NMR of compound **α-90**

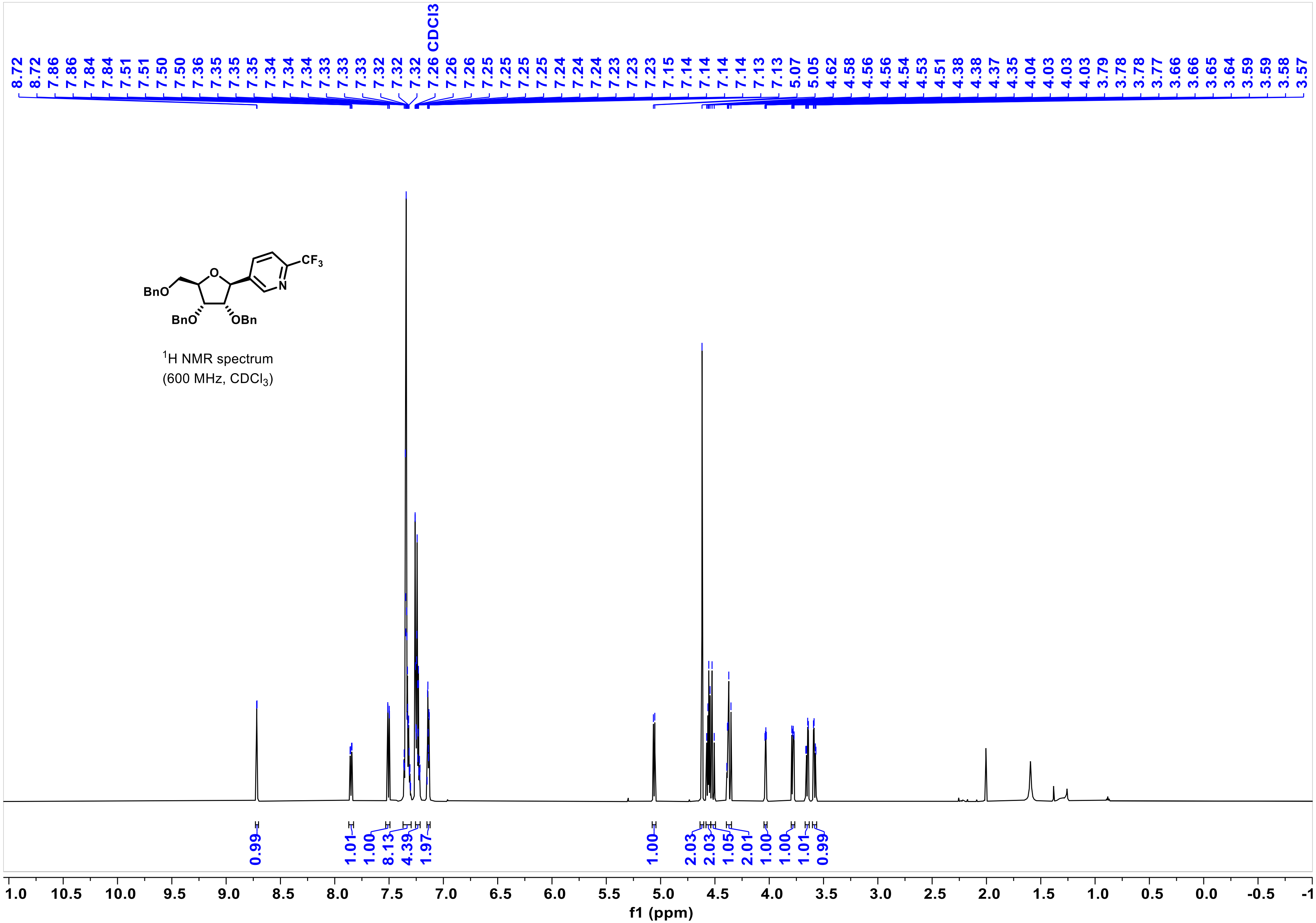

^{1}H NMR of compound **β-90**

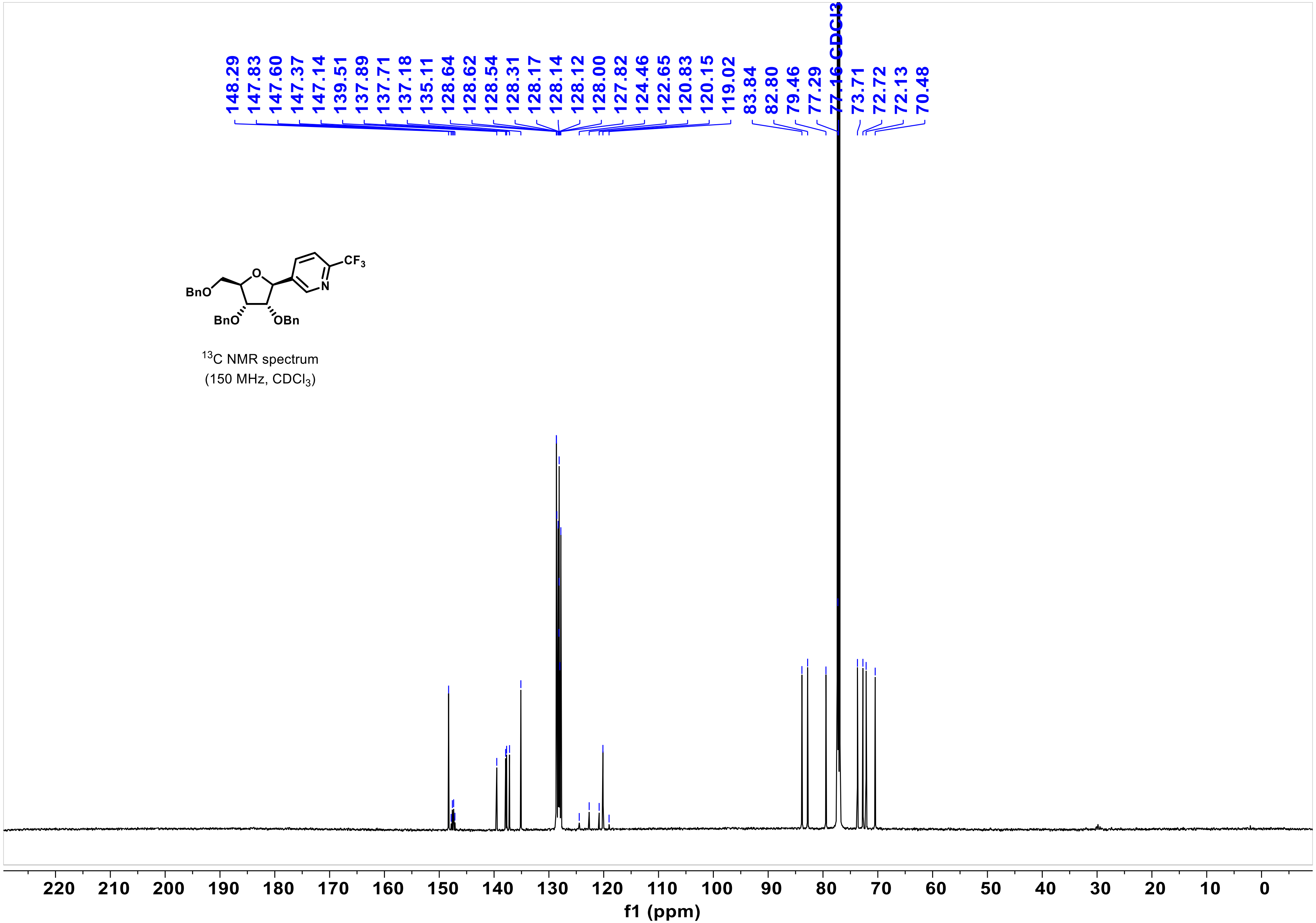

^{13}C NMR of compound **β-90**

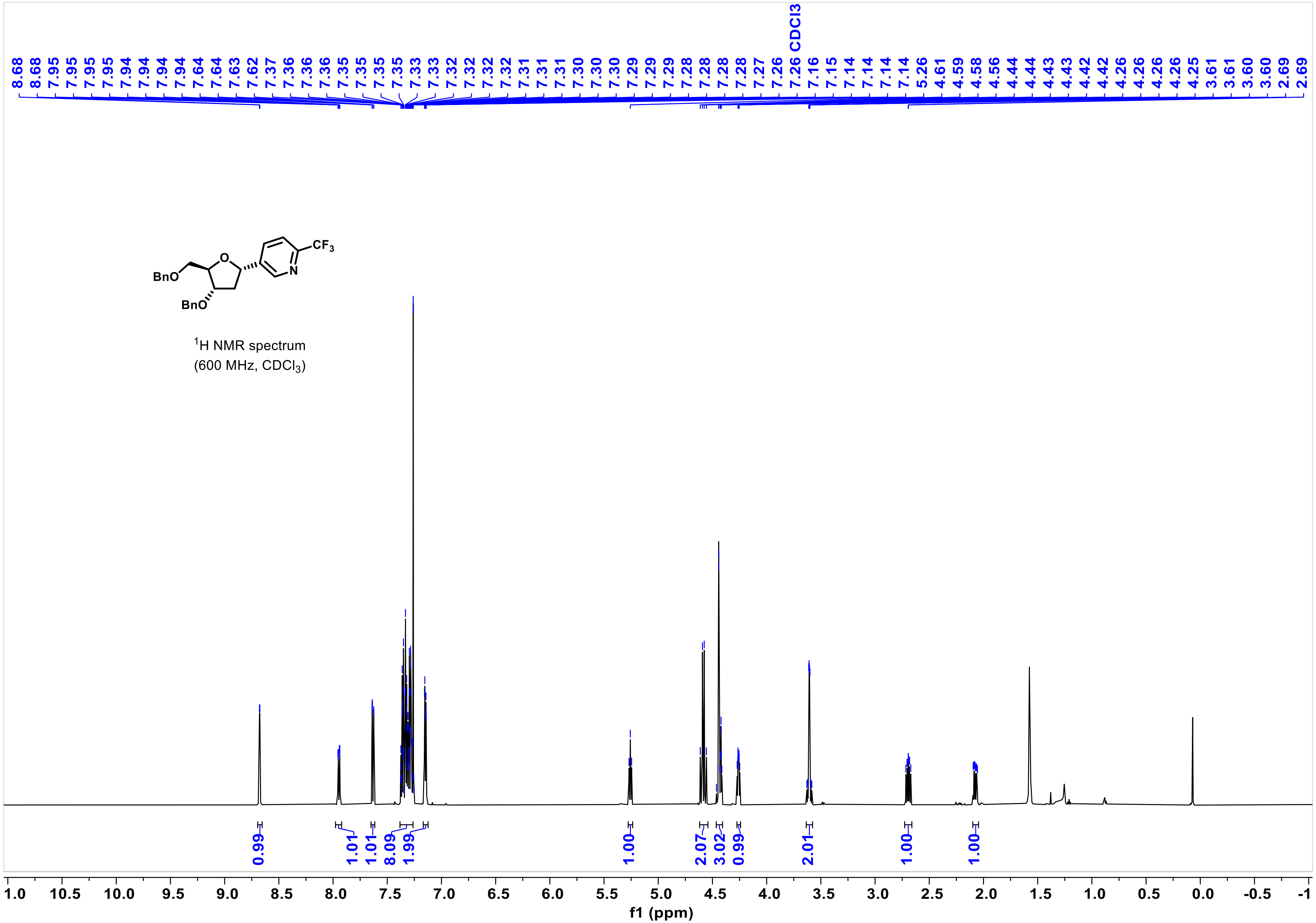

^{1}H NMR of compound **α-92**

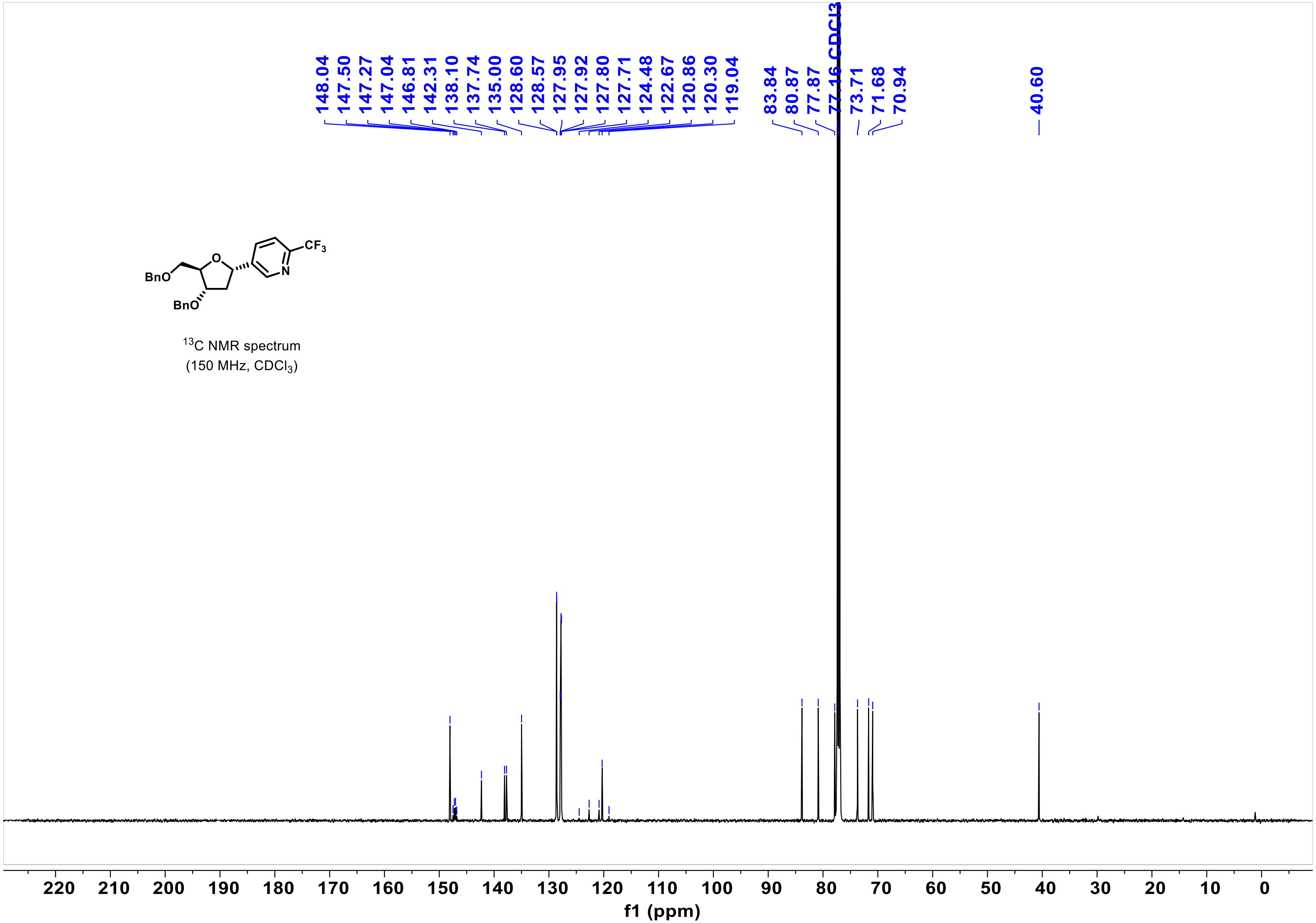

^{13}C NMR of compound **α-92**

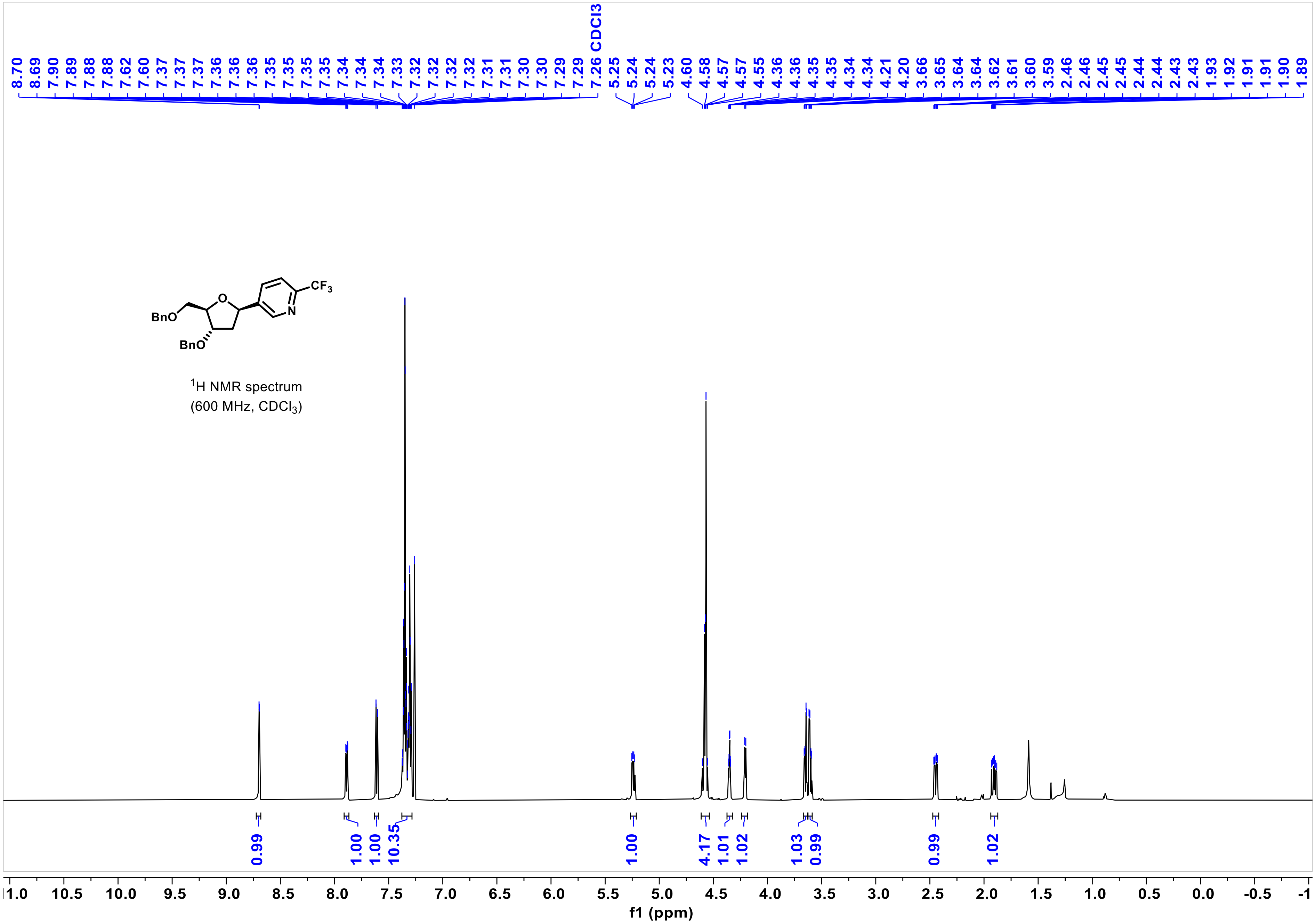

^{1}H NMR of compound **β-92**

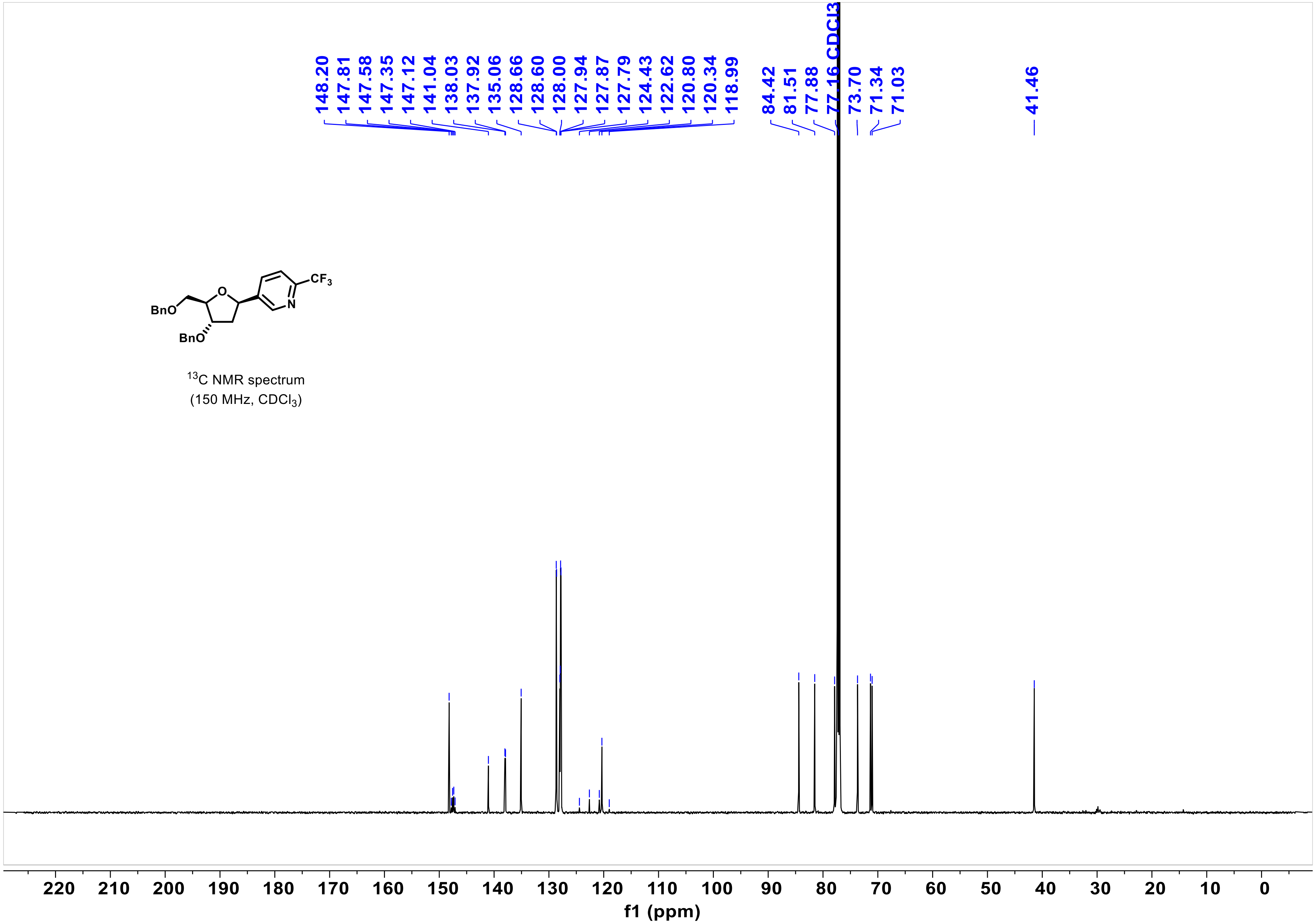

^{13}C NMR of compound **β-92**

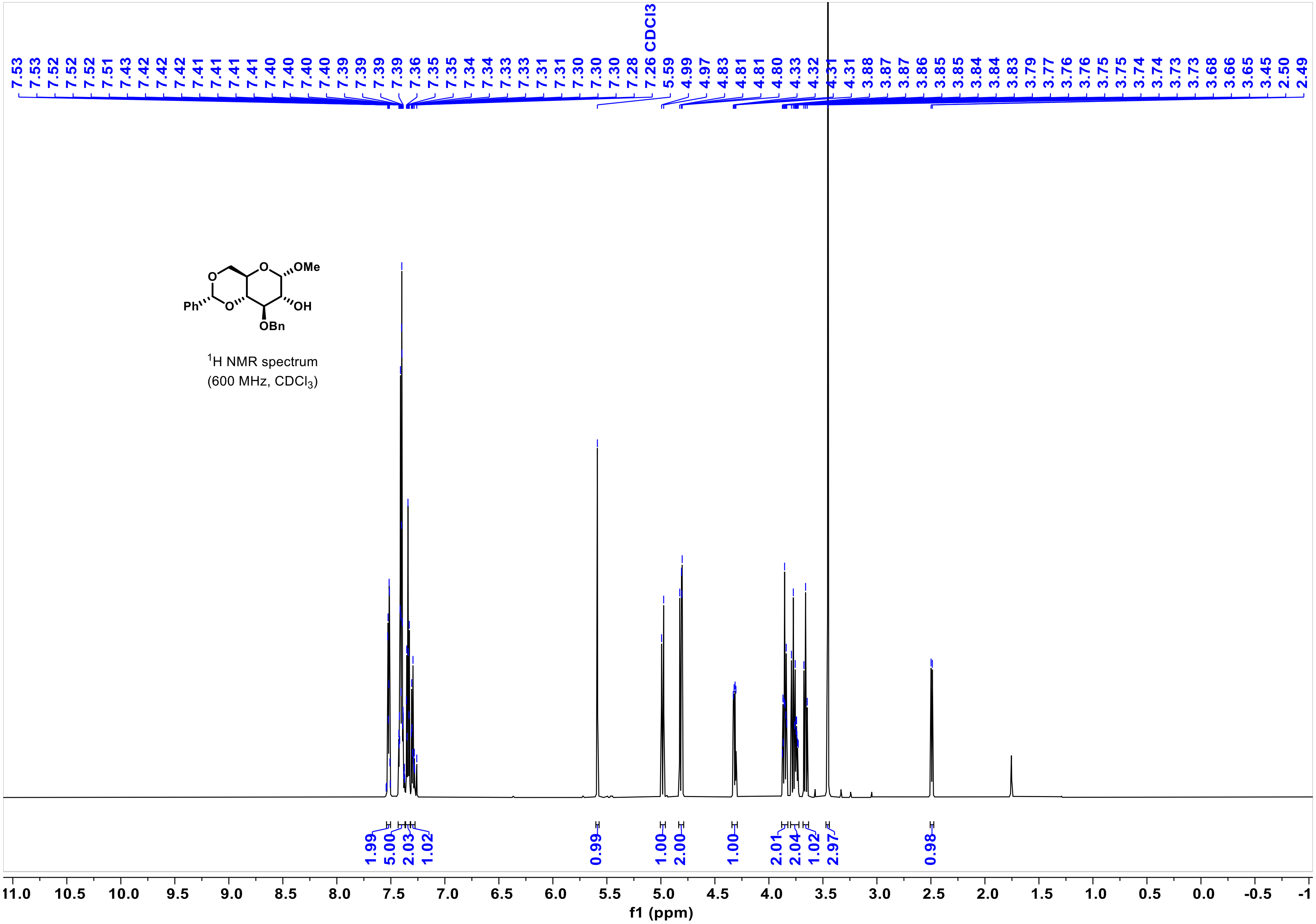

^{1}H NMR of compound **SF6**

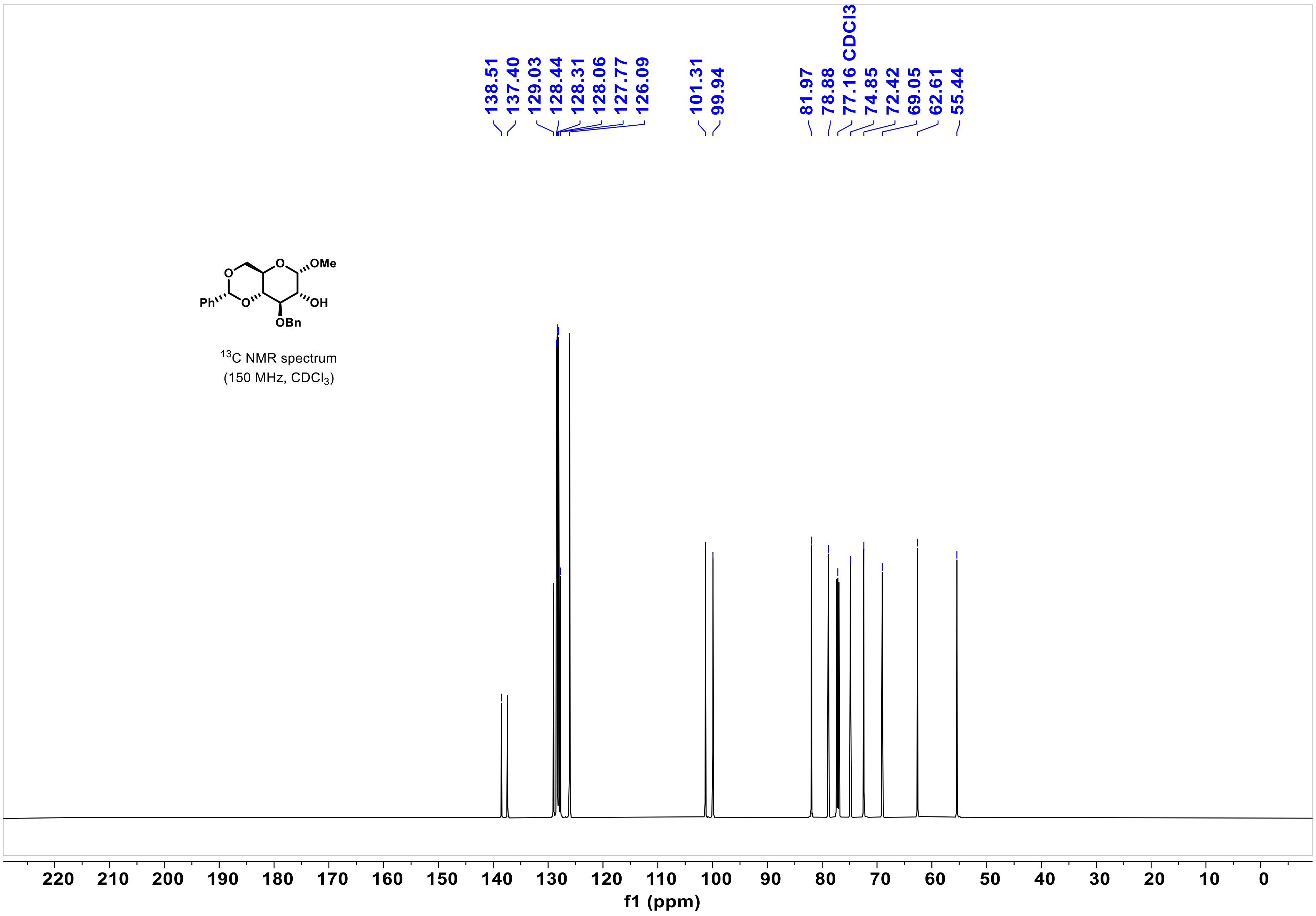

^{13}C NMR of compound **SF6**

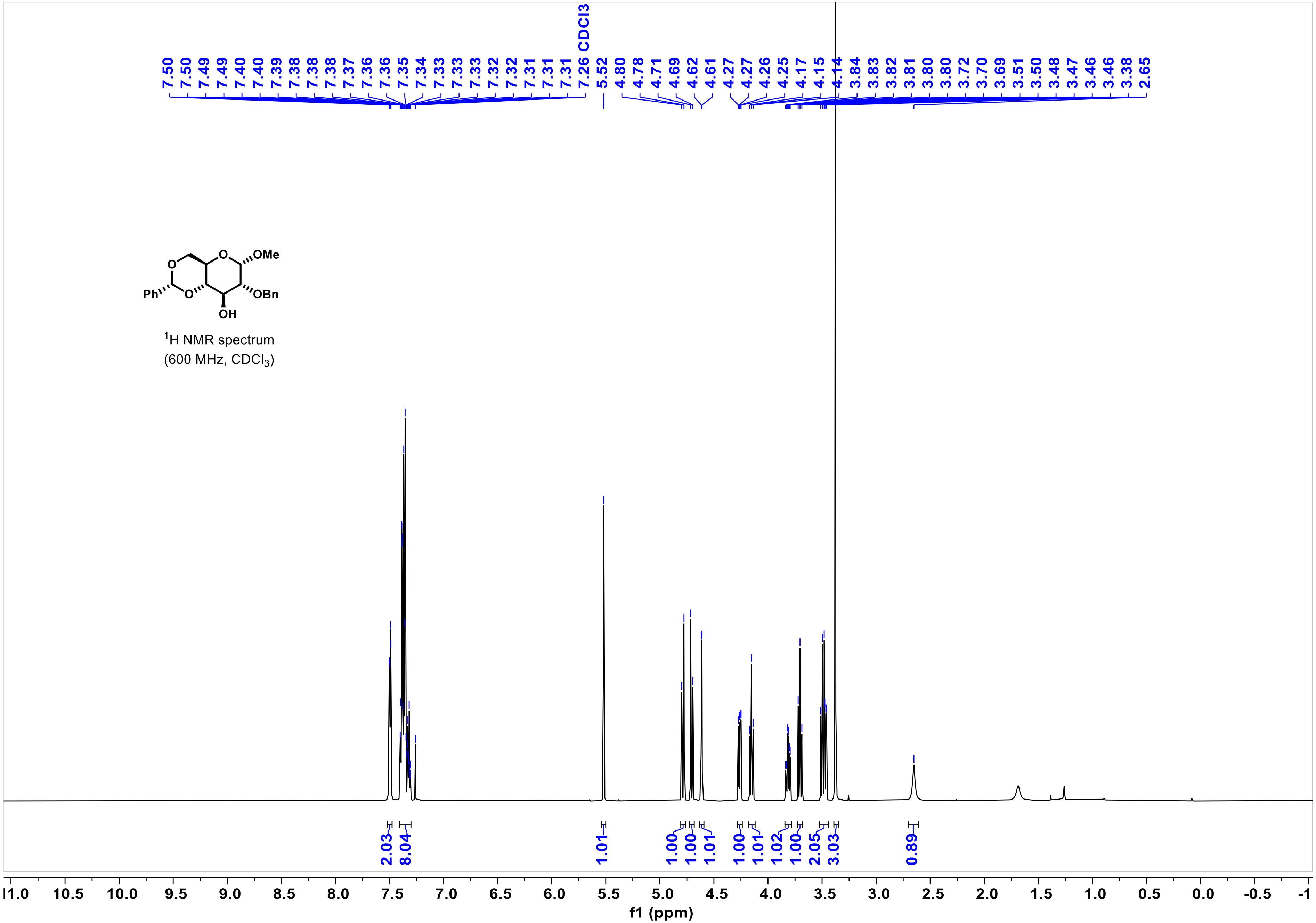

^{1}H NMR of compound **SF7**

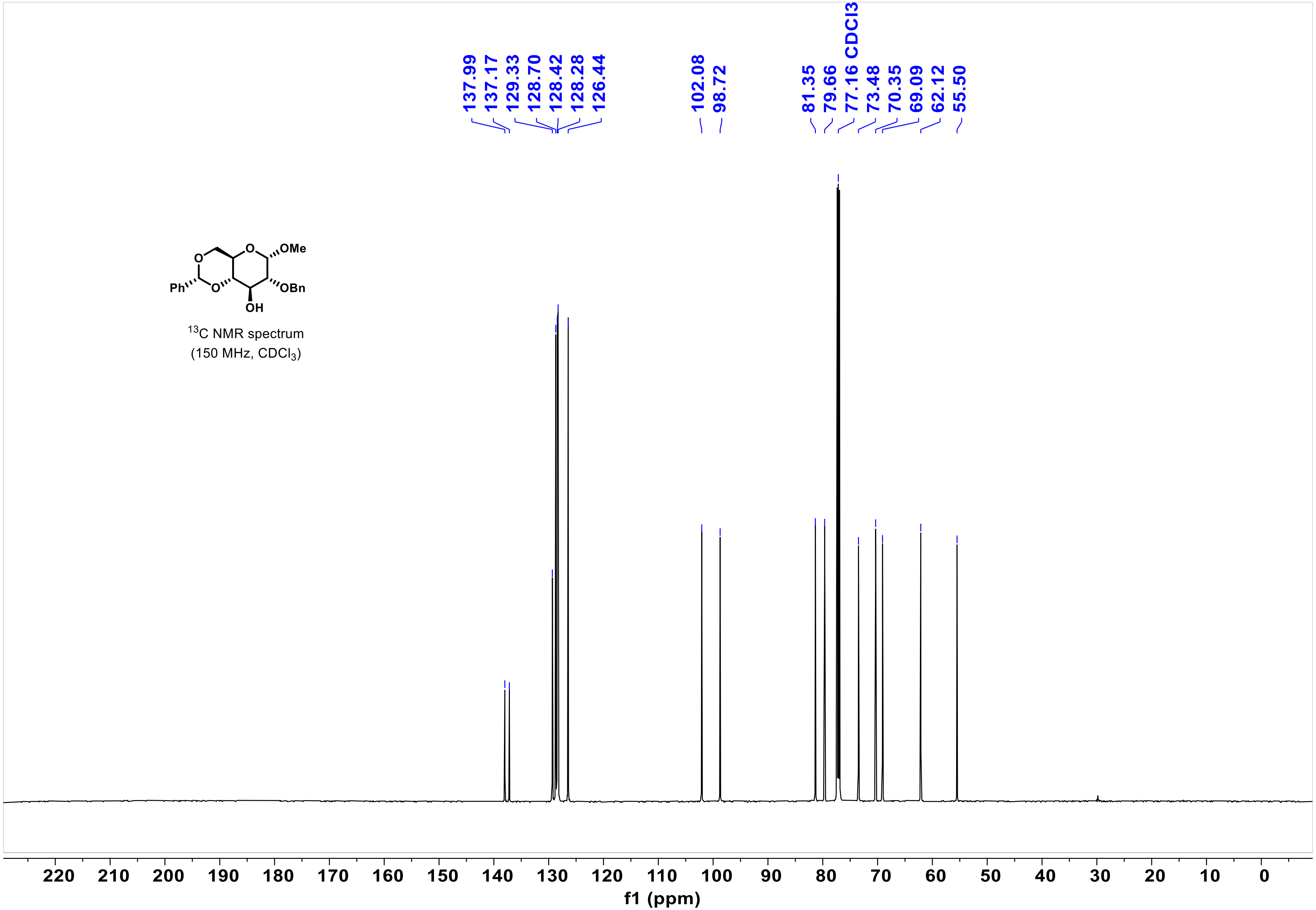

^{13}C NMR of compound **SF7**

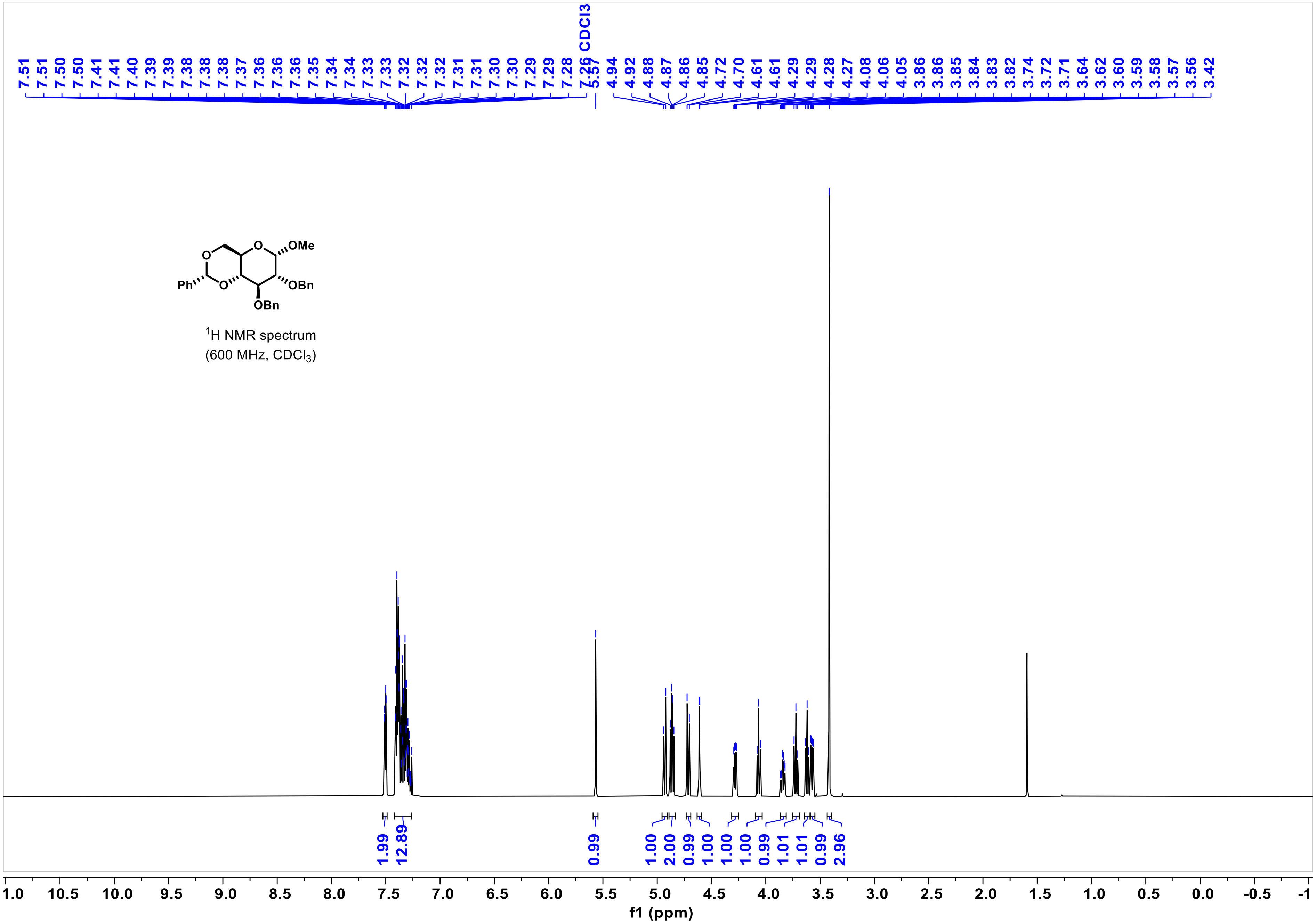

^{1}H NMR of compound **SF22**

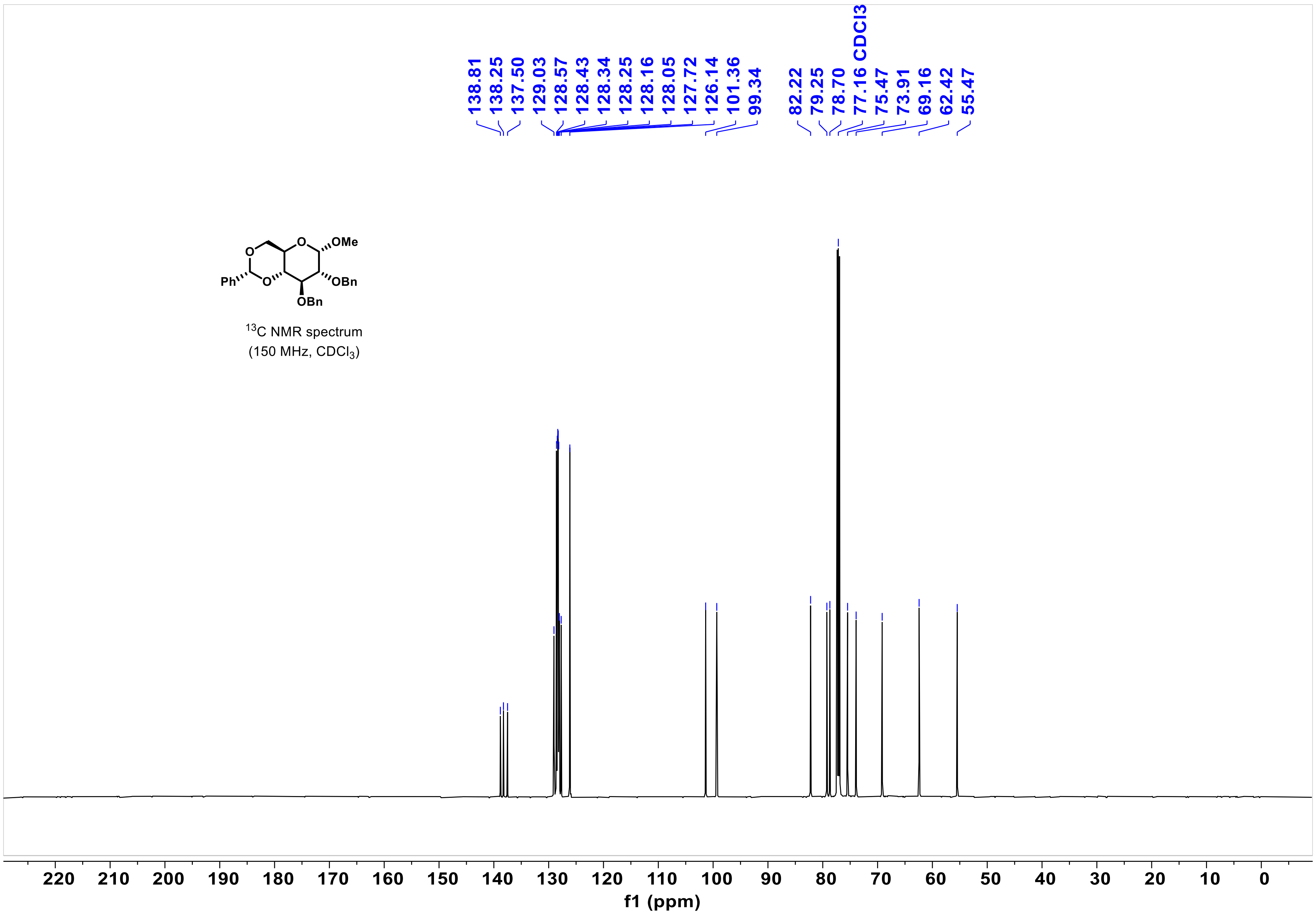

^{13}C NMR of compound **SF22**

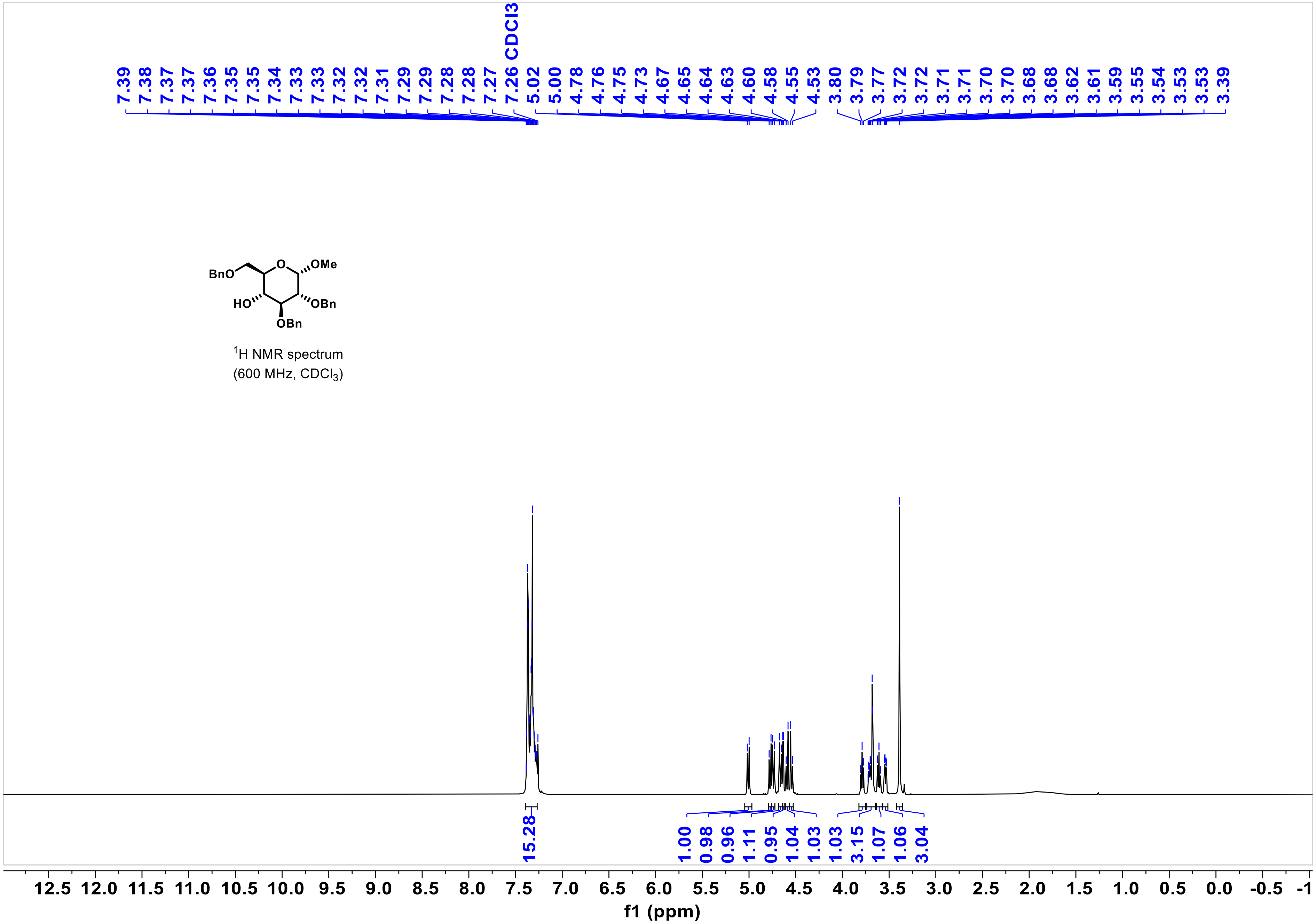

^{1}H NMR of compound **SF8**

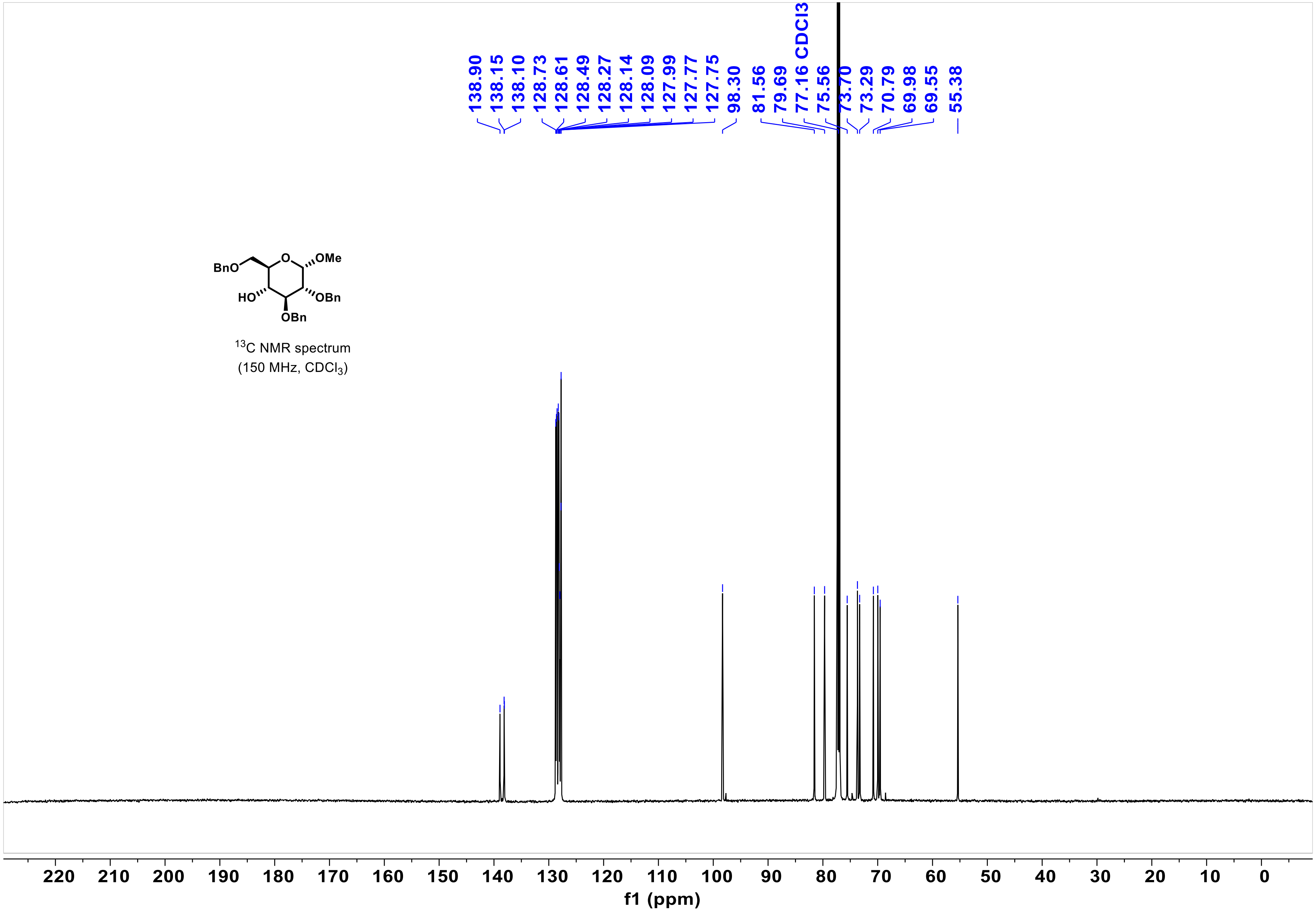

^{13}C NMR of compound **SF8**

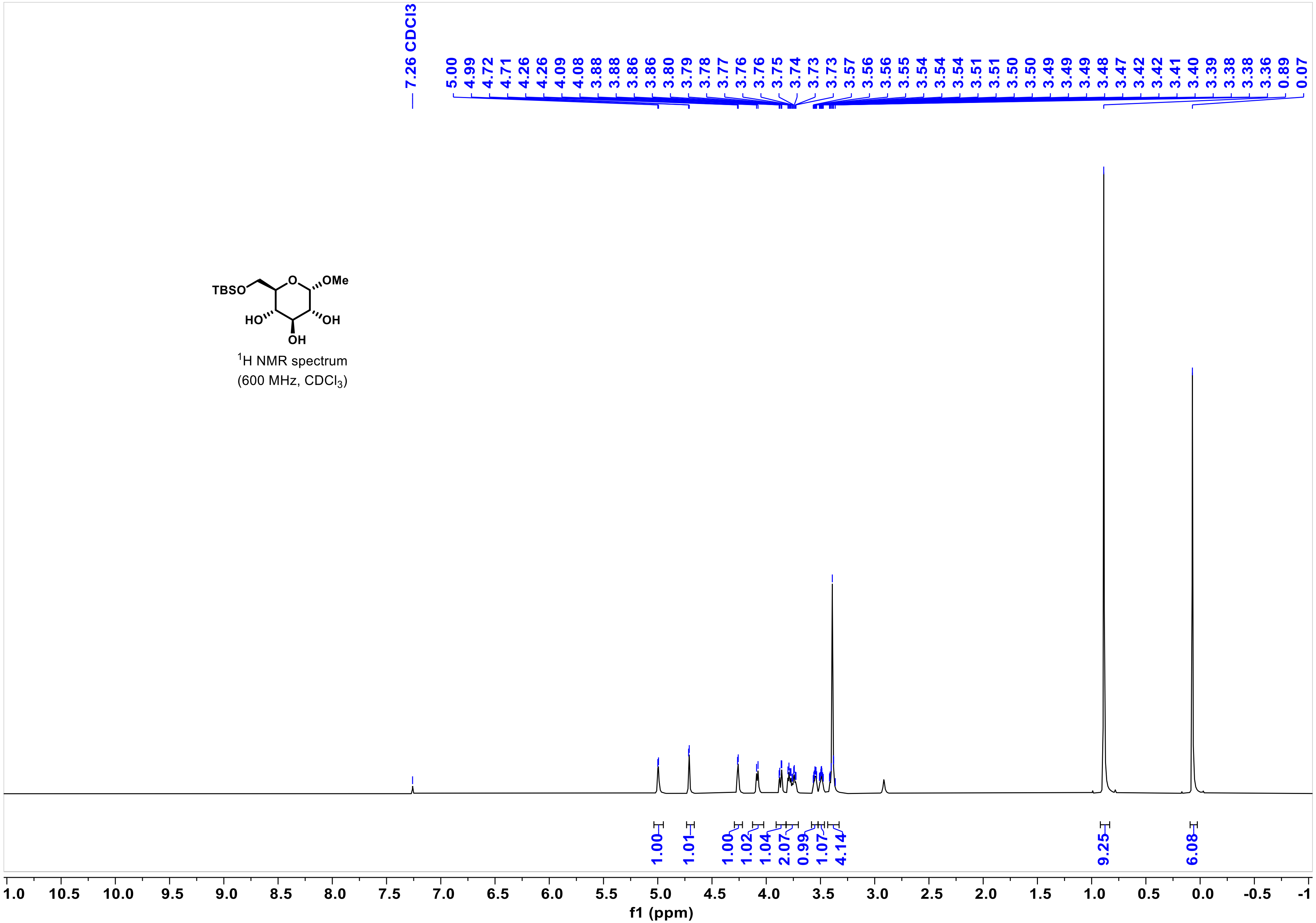

^{1}H NMR of compound **SF10**

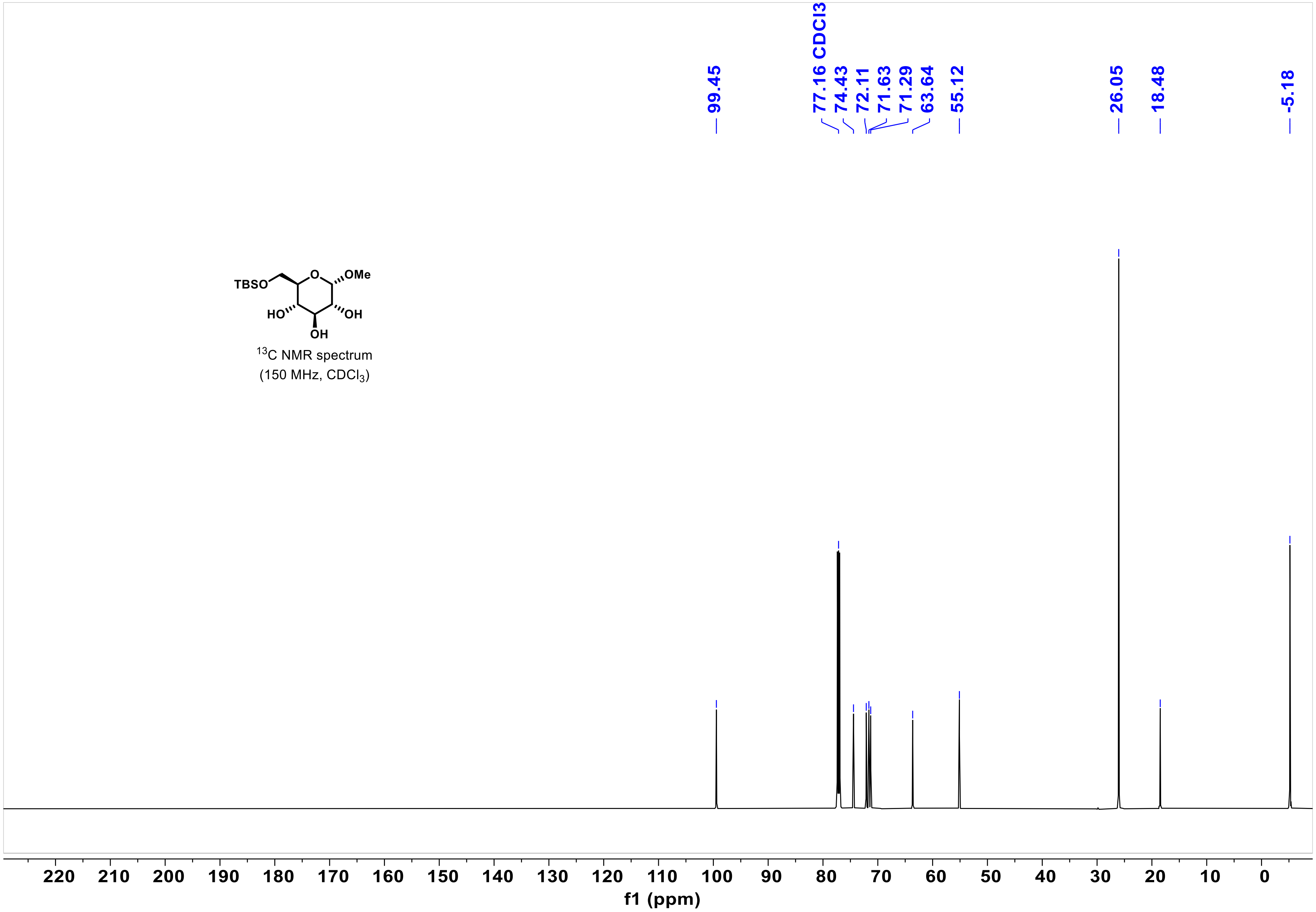

^{13}C NMR of compound **SF10**

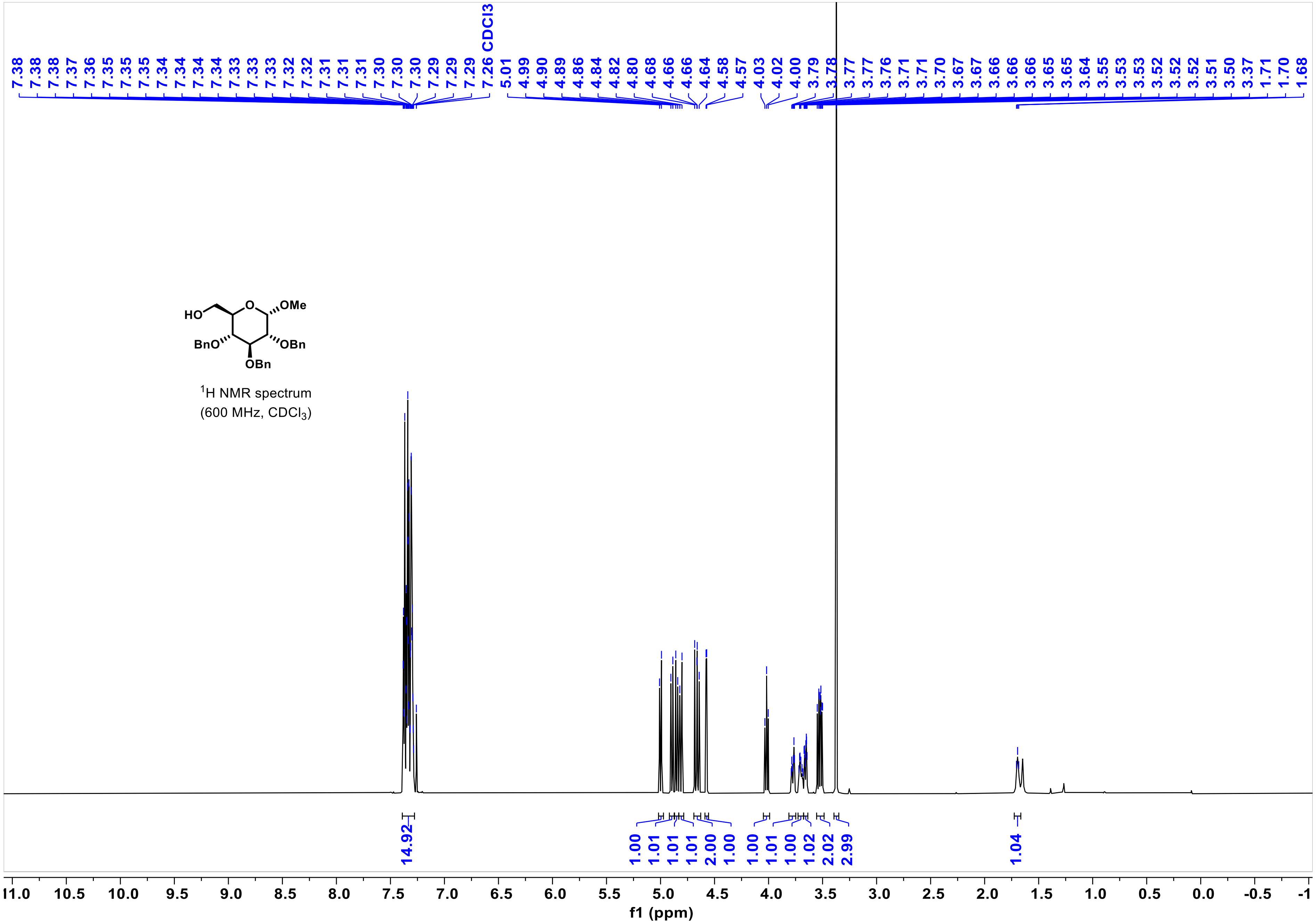

^{1}H NMR of compound **SF11**

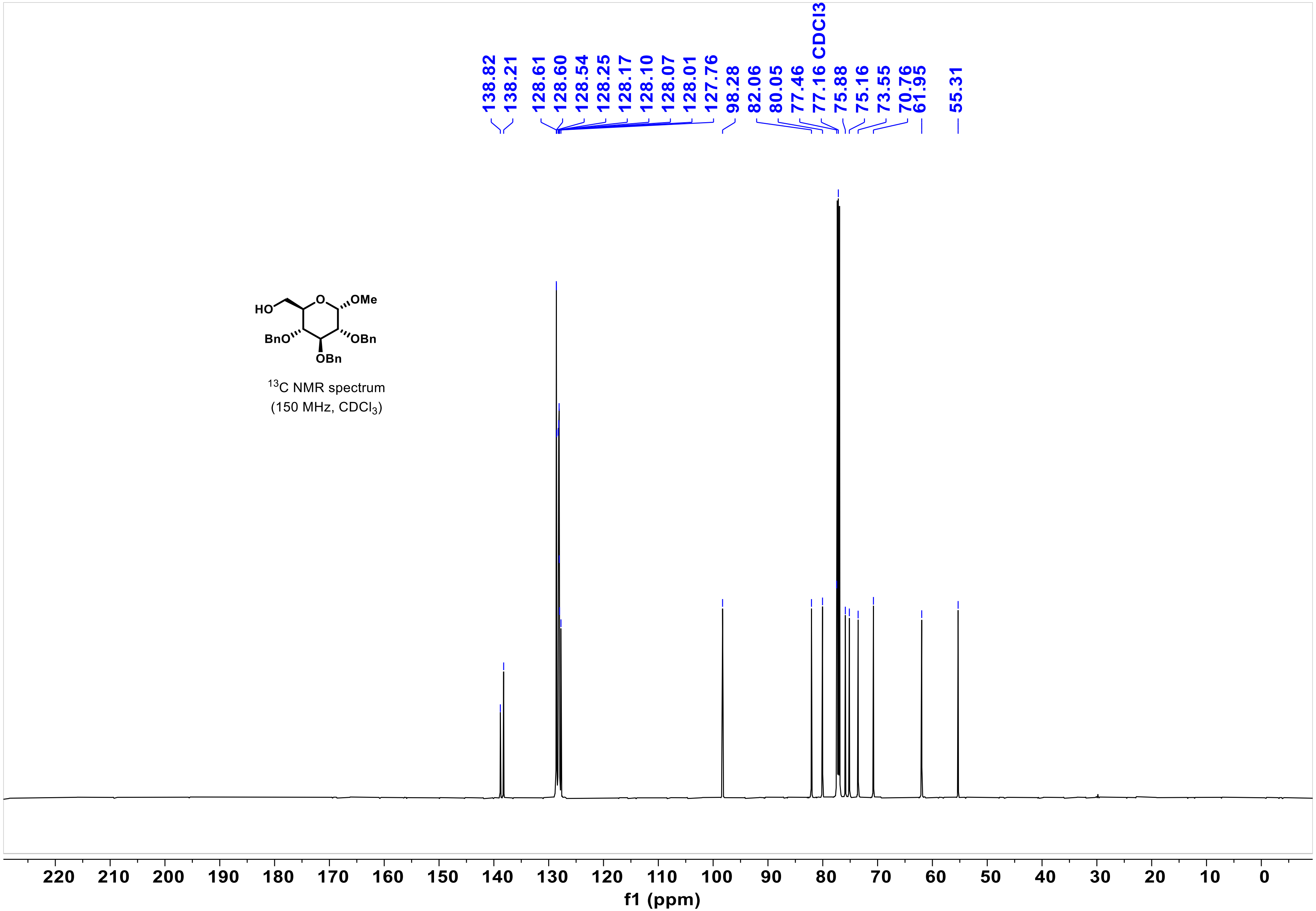

^{13}C NMR of compound **SF11**

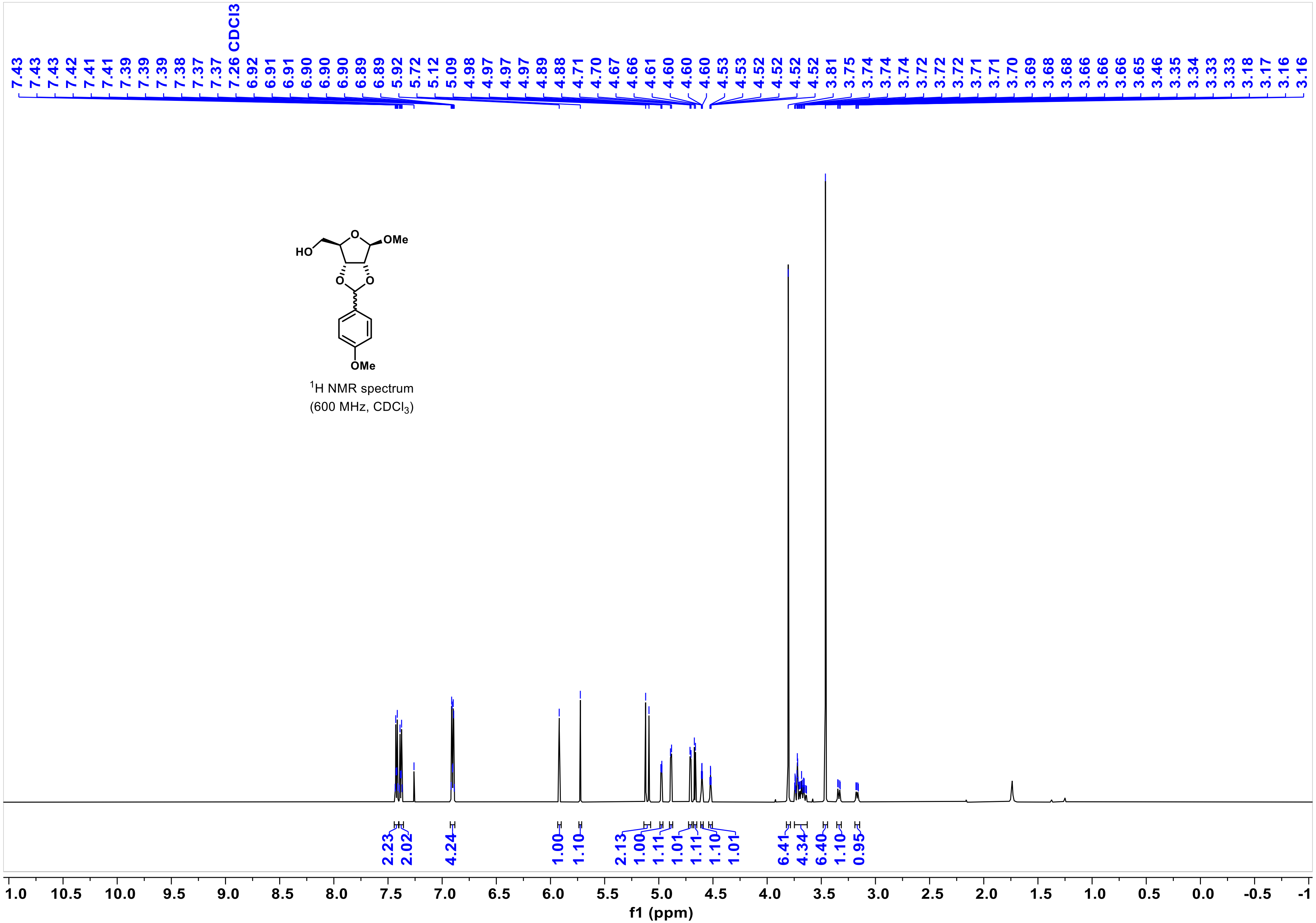

^{1}H NMR of compound **SF23**

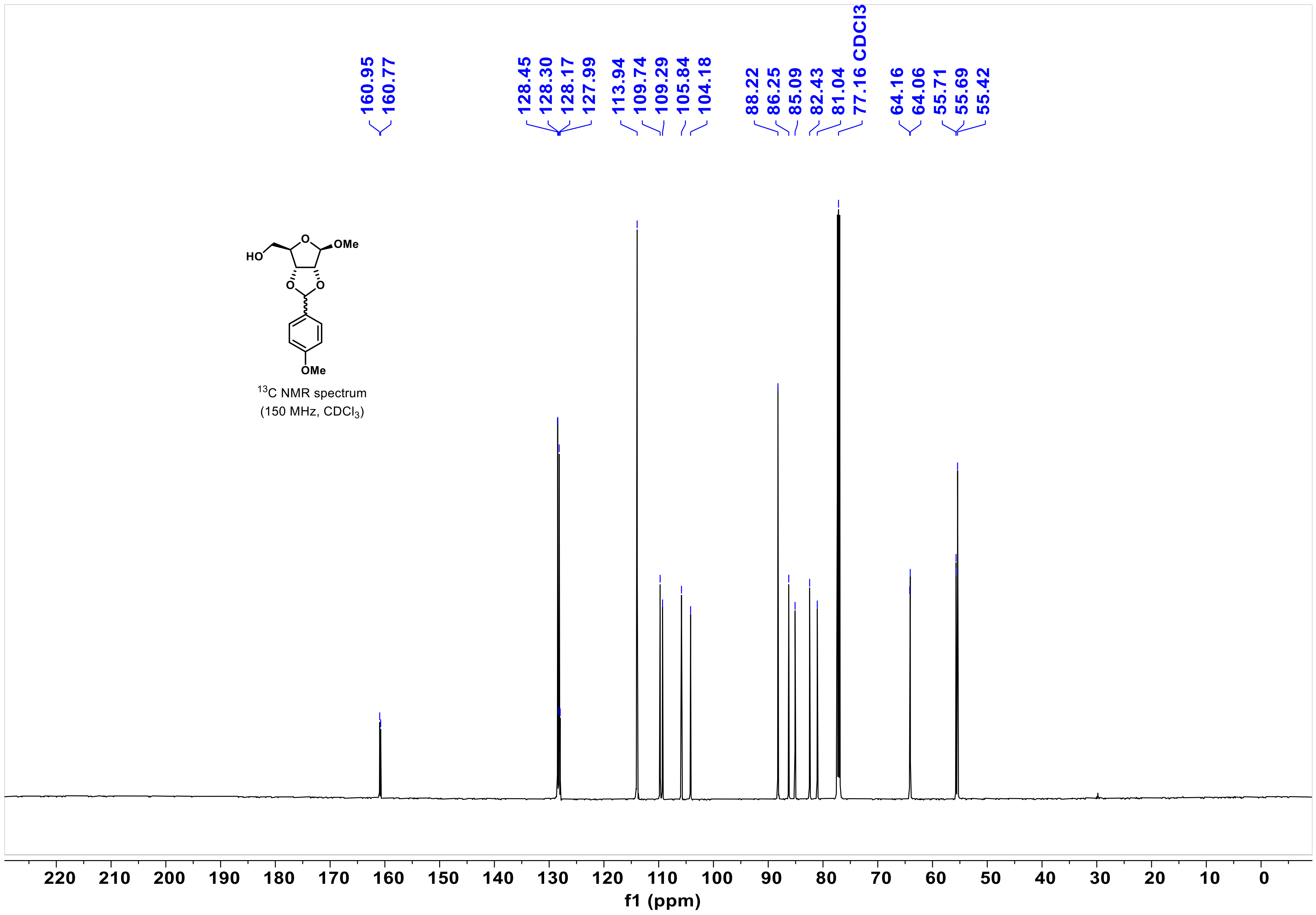

^{13}C NMR of compound **SF23**

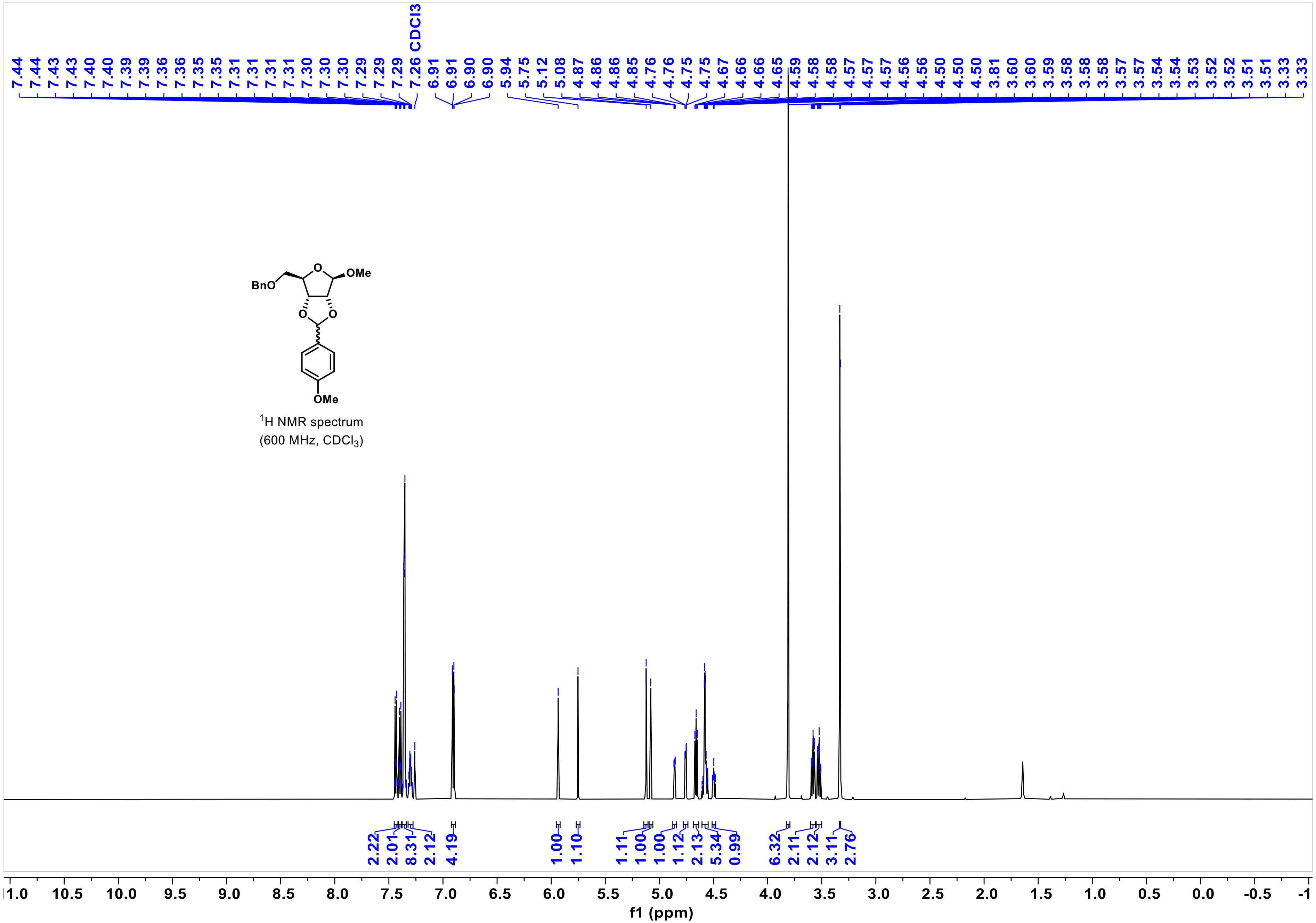

¹H NMR of compound **SF24**

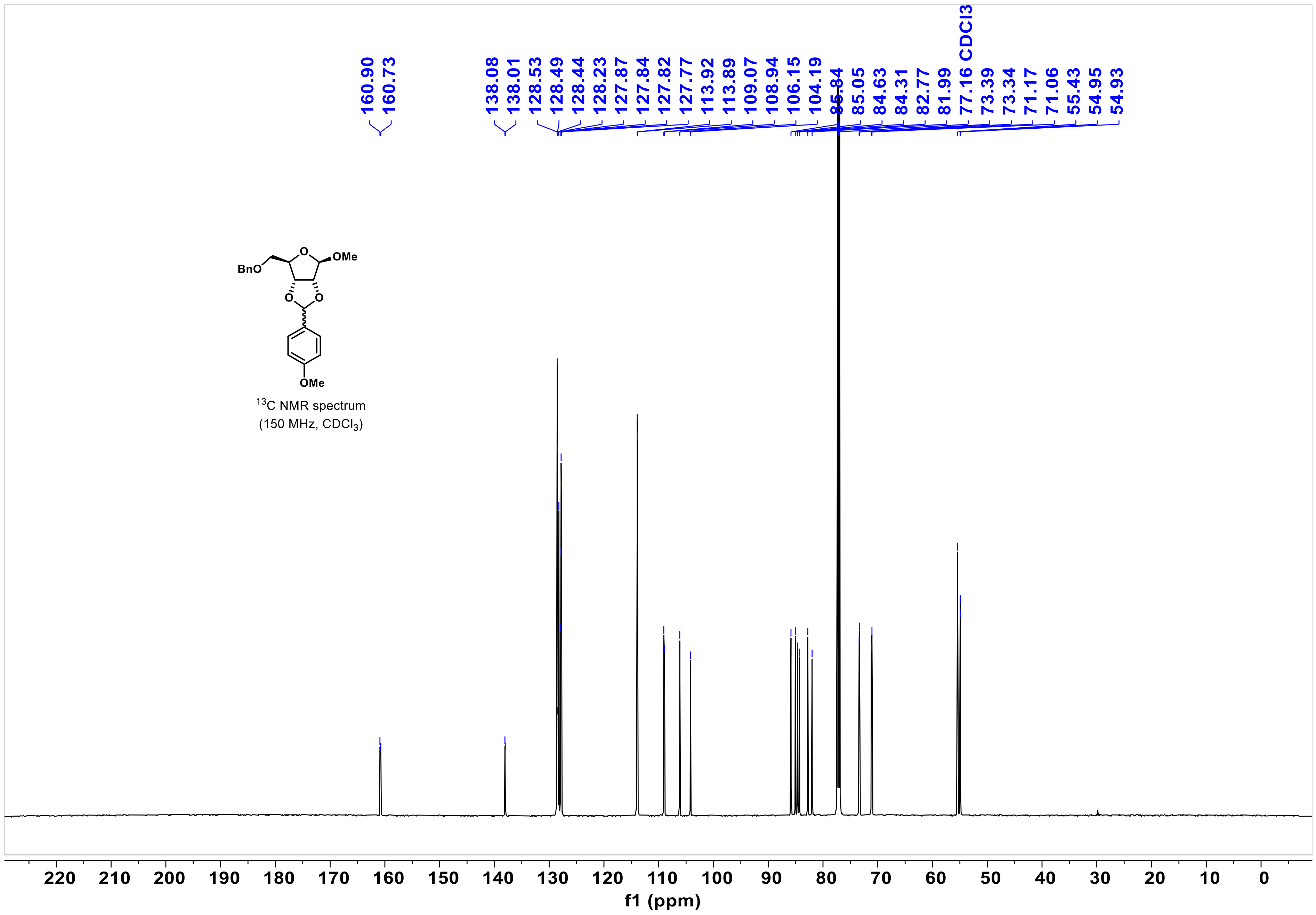

^{13}C NMR of compound **SF24**

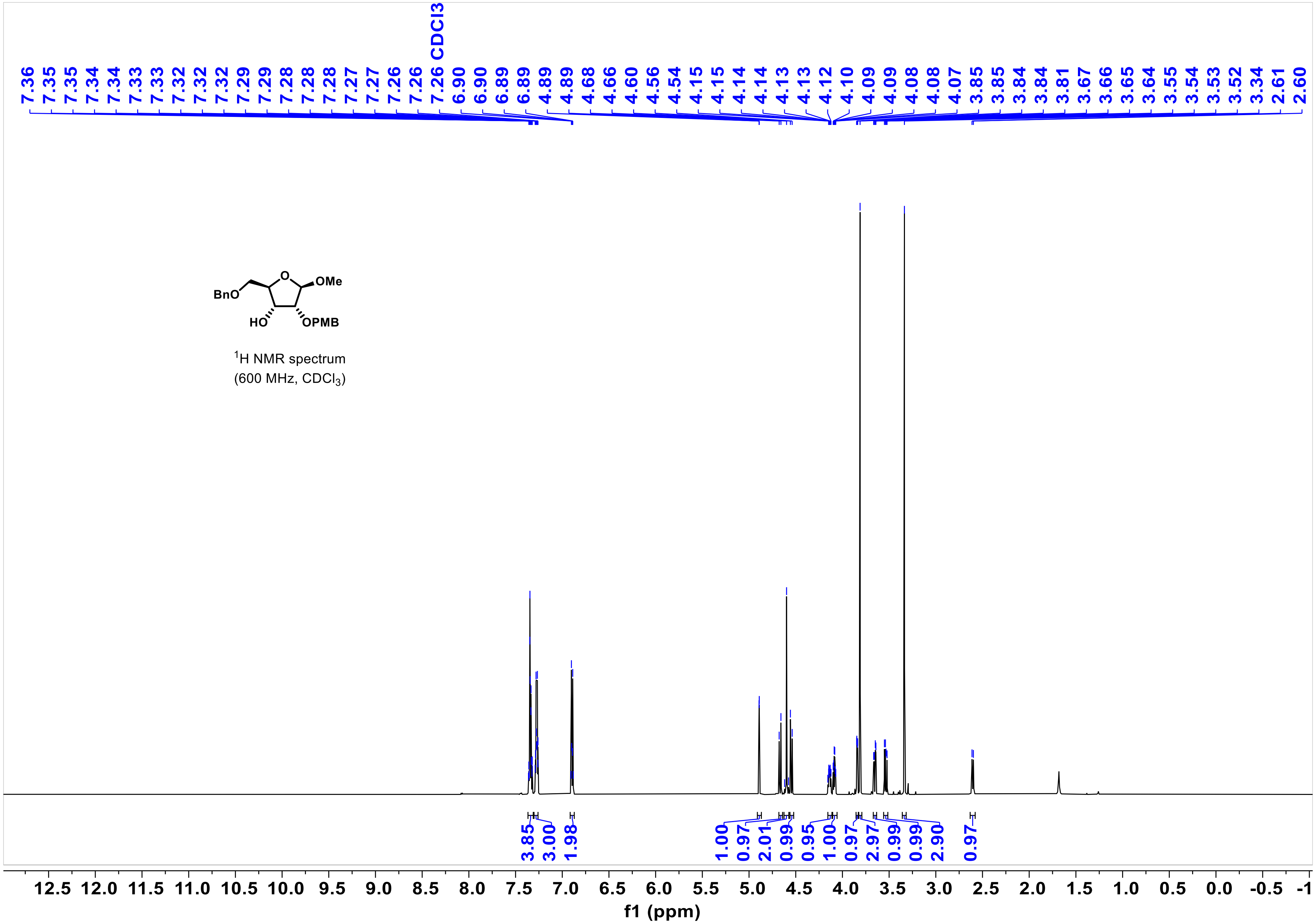

^{1}H NMR of compound **SF13**

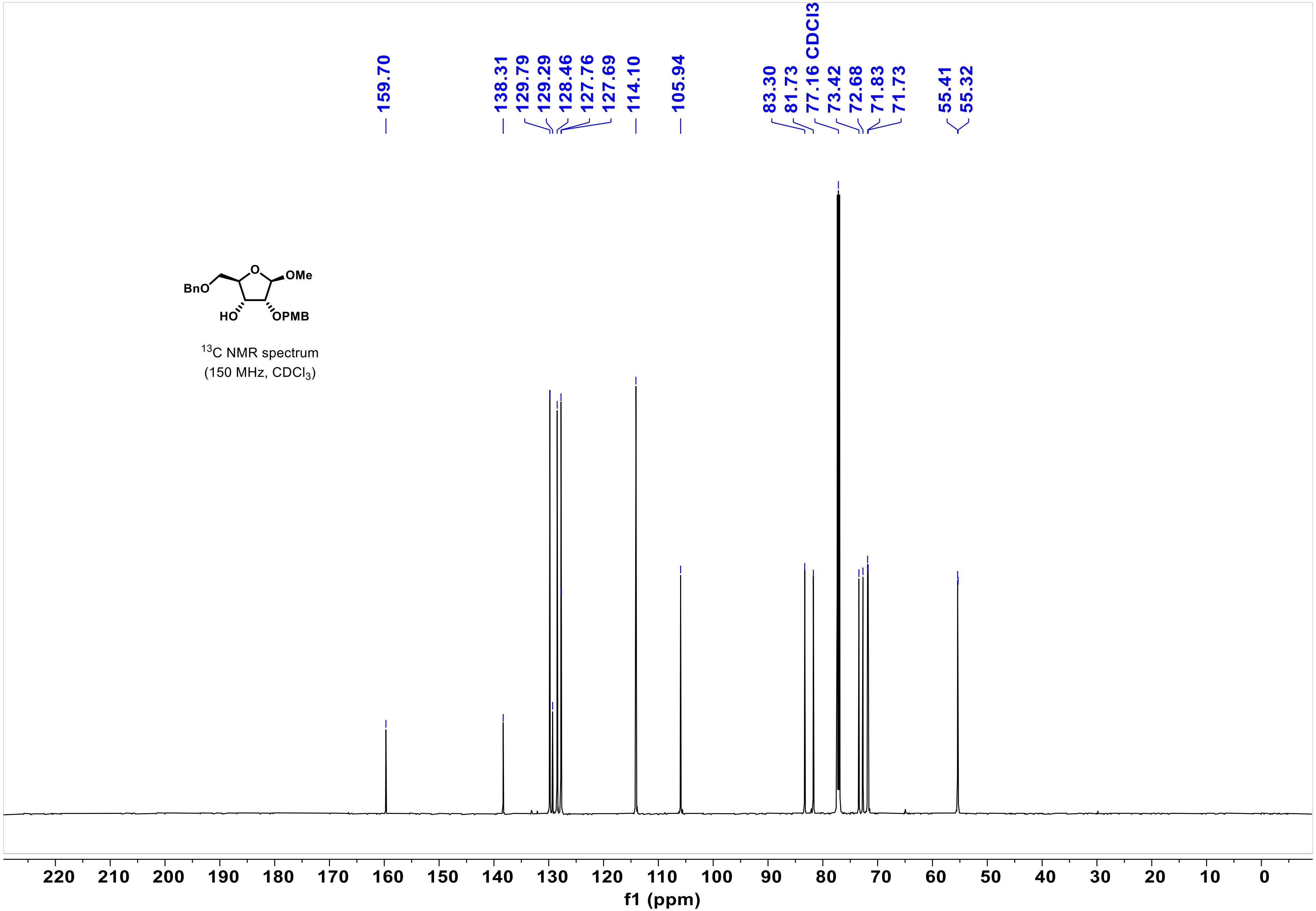

^{13}C NMR of compound **SF13**

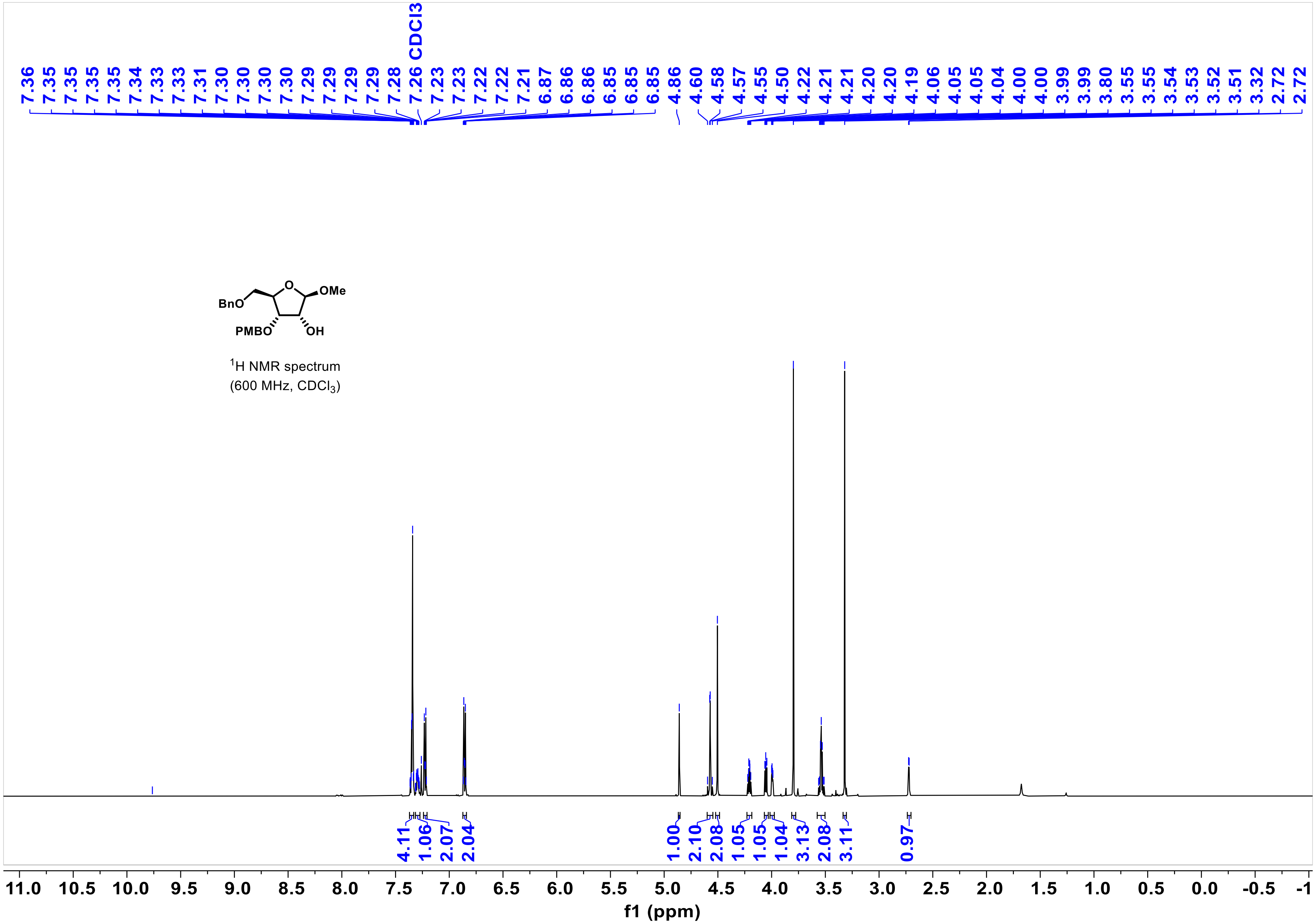

^{1}H NMR of compound **SF14**

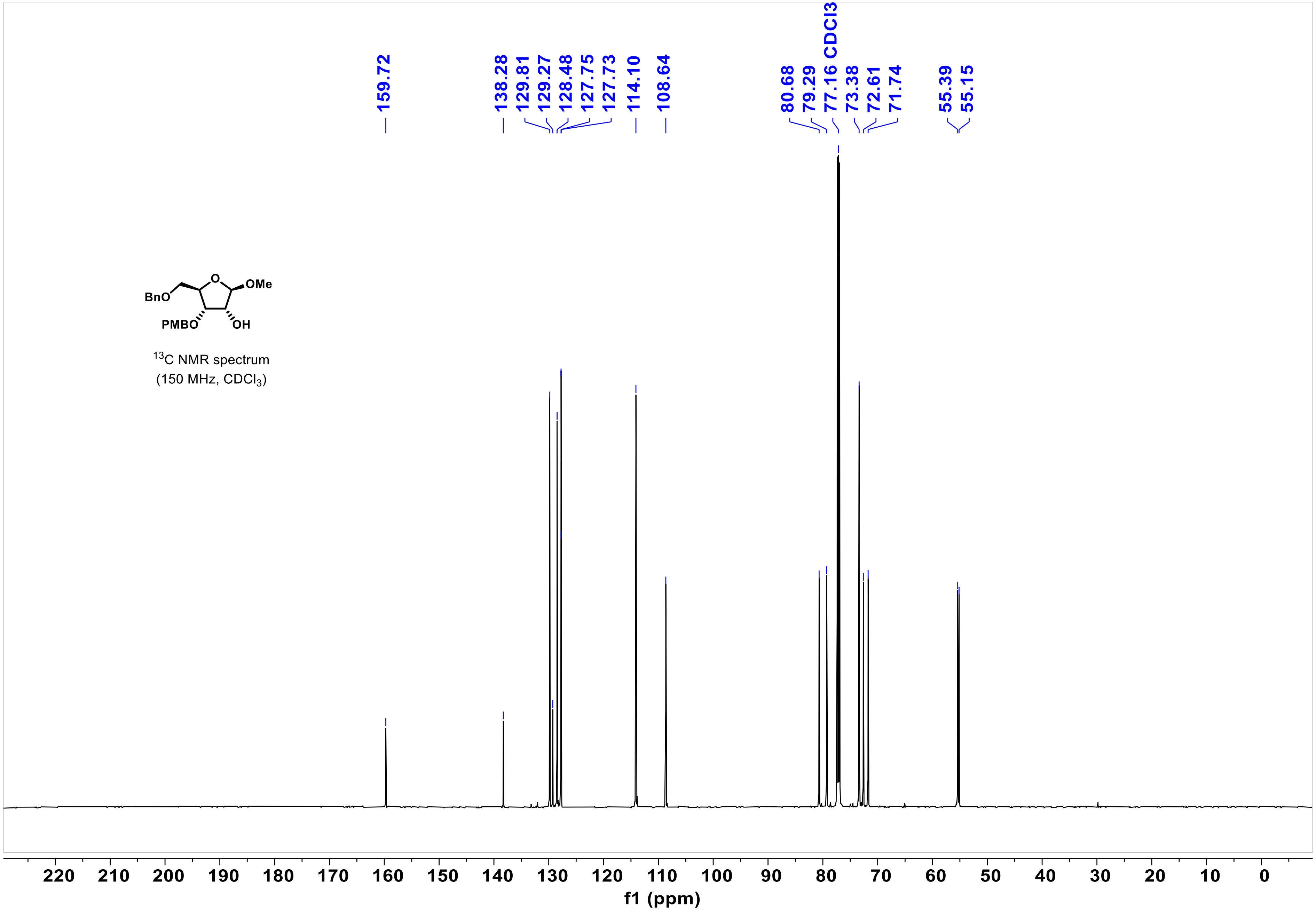

^{13}C NMR of compound **SF14**

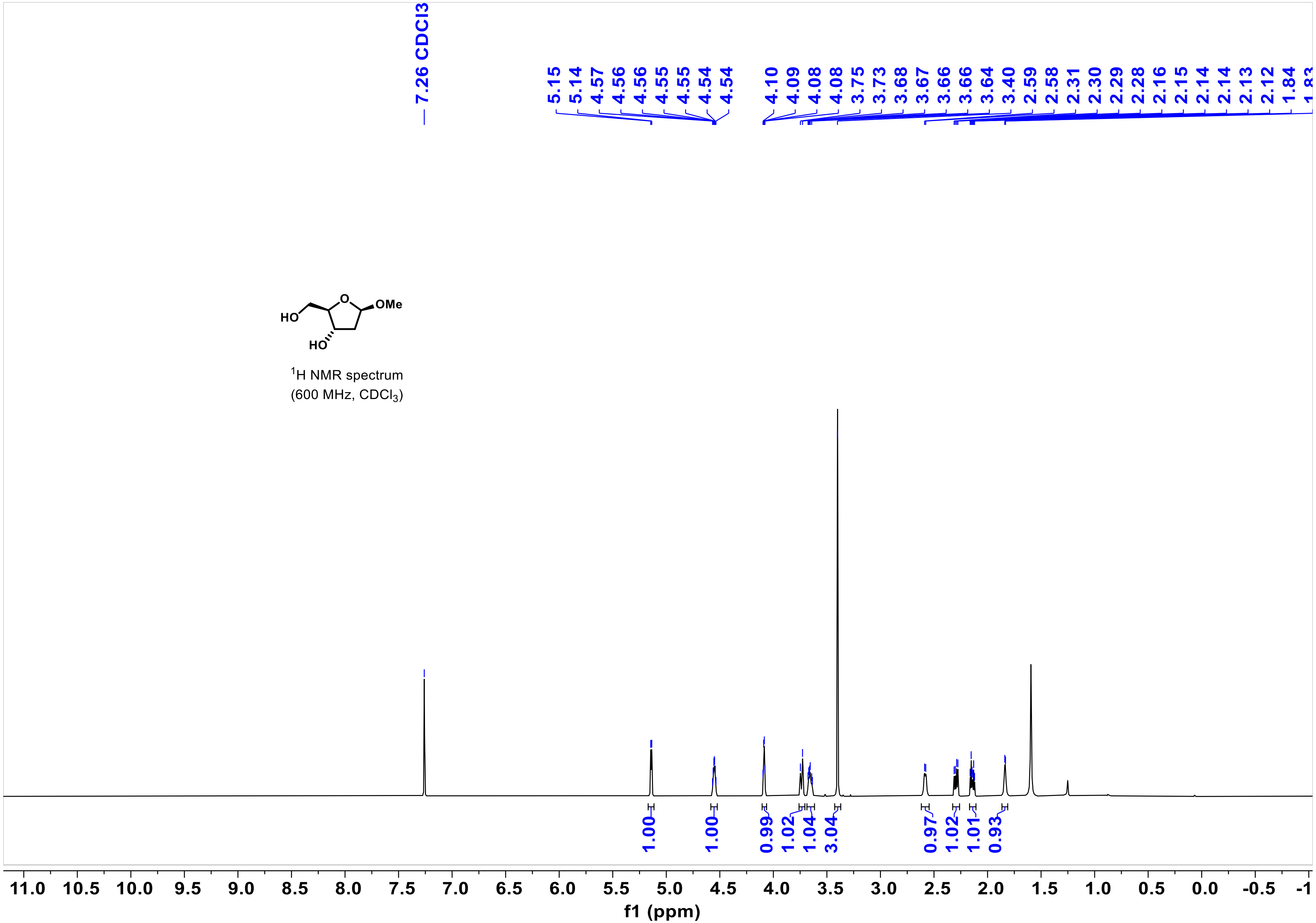

^{1}H NMR of compound **SF19**

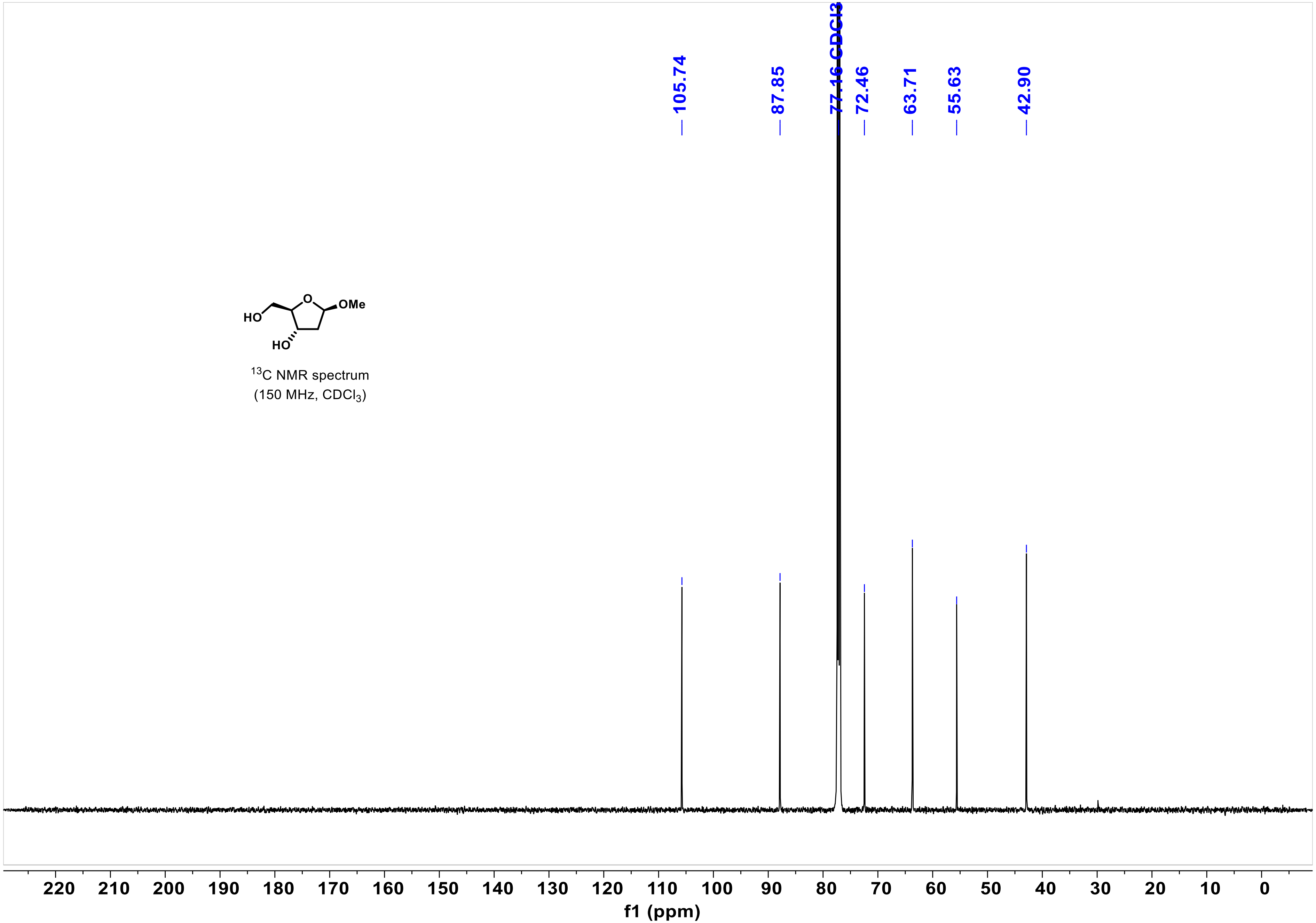

^{13}C NMR of compound **SF19**

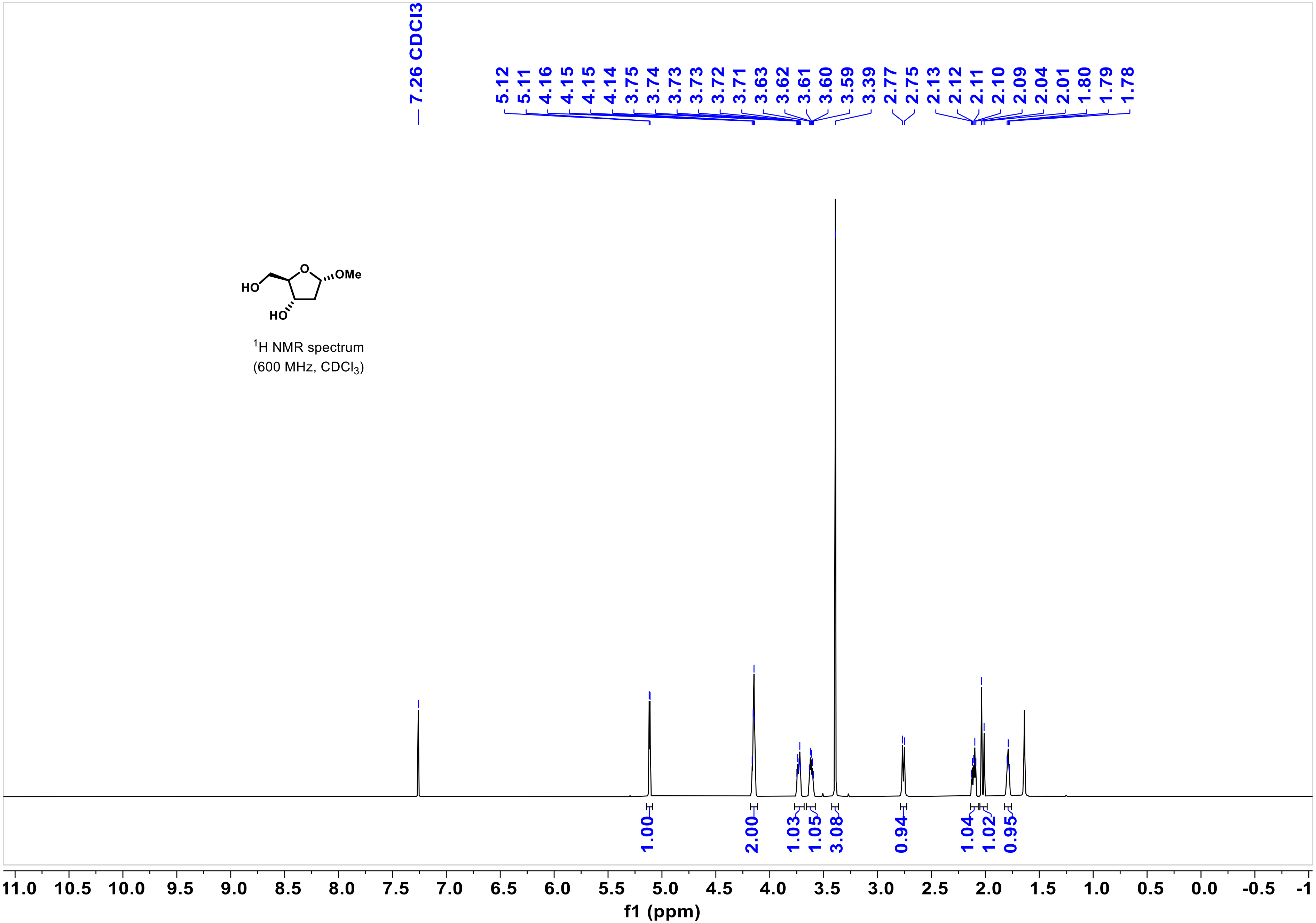

^{1}H NMR of compound **SF20**

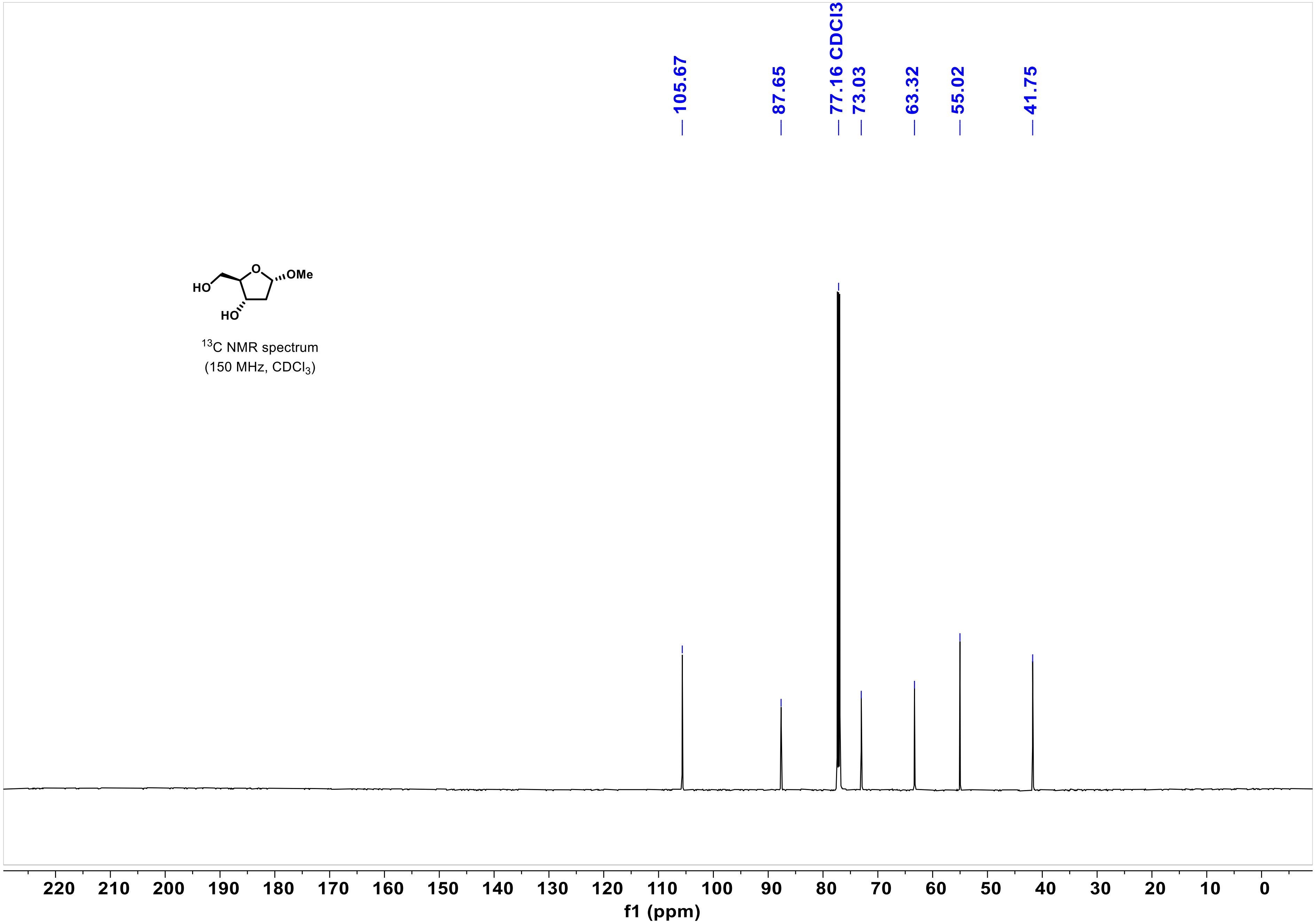

^{13}C NMR of compound **SF20**

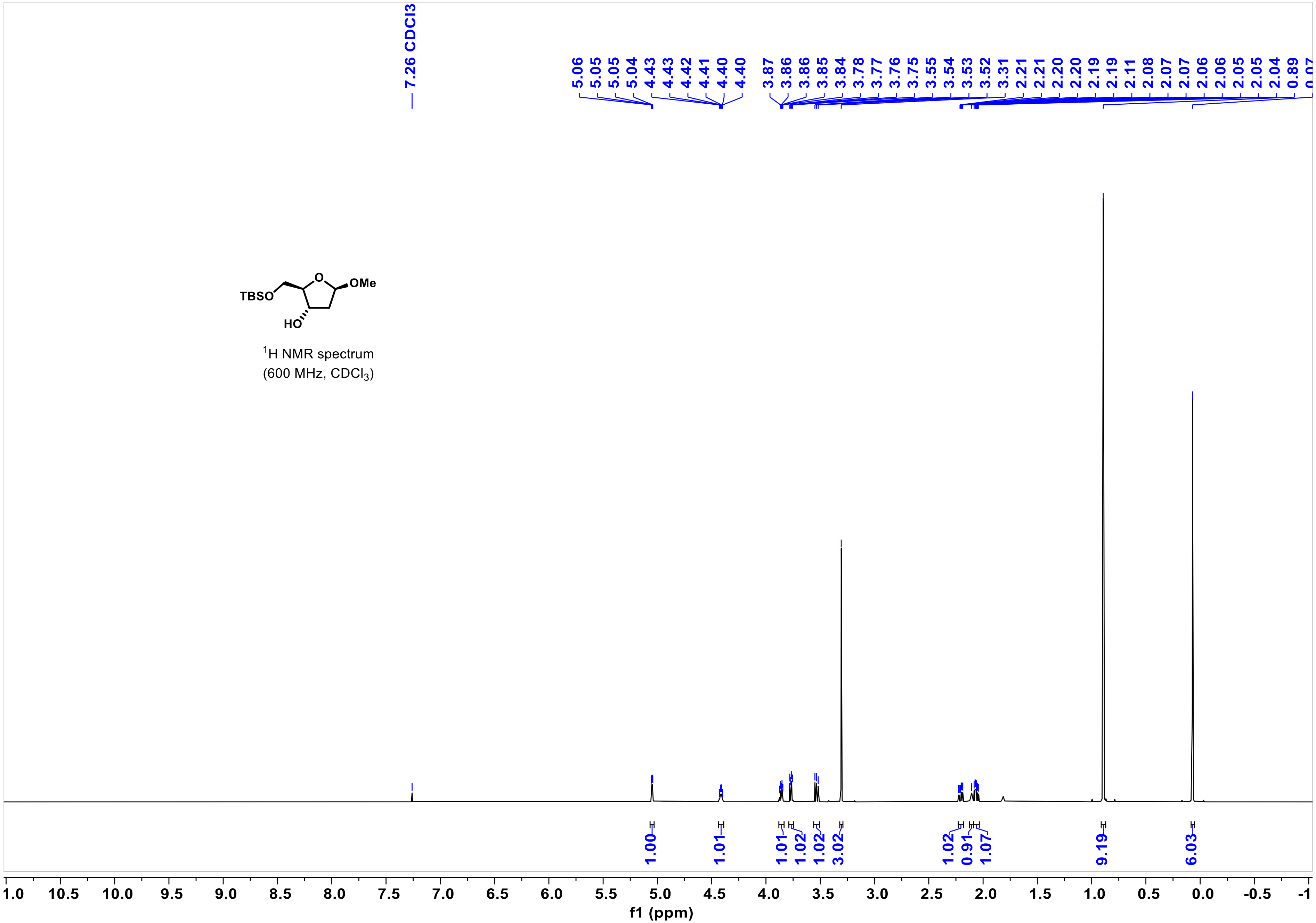

^{1}H NMR of compound **SF21**

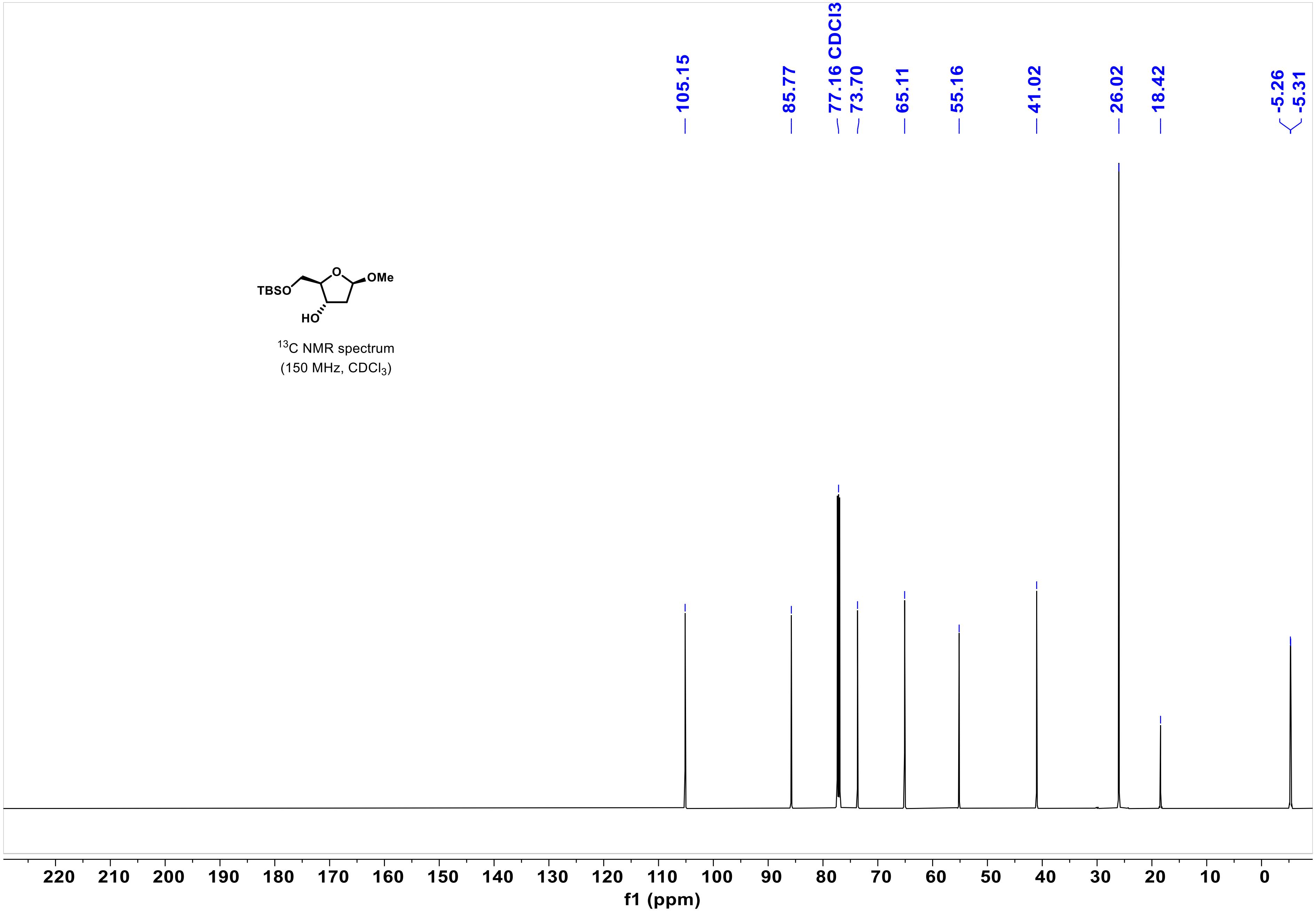

^{13}C NMR of compound **SF21**

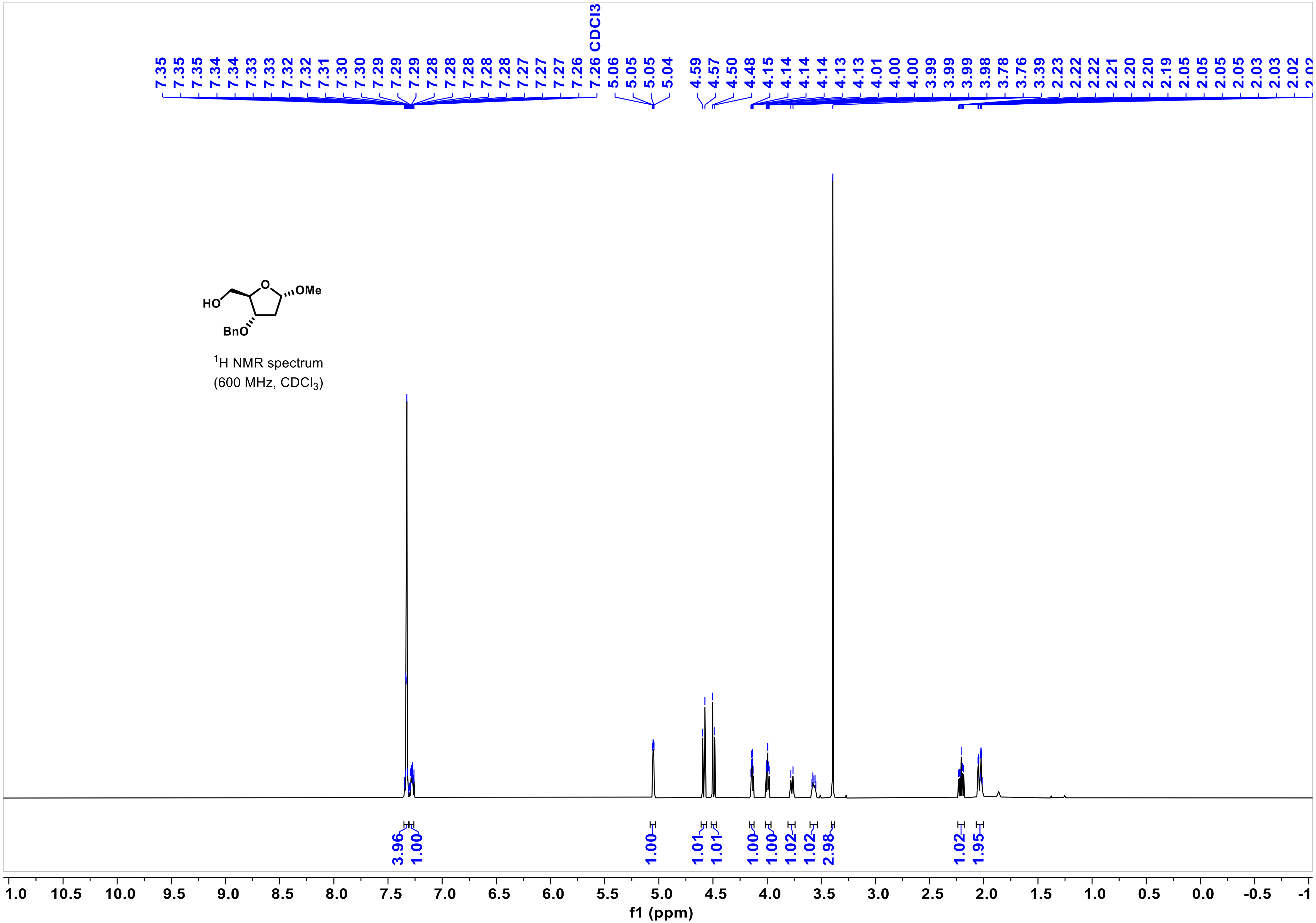

^{1}H NMR of compound **SF25**

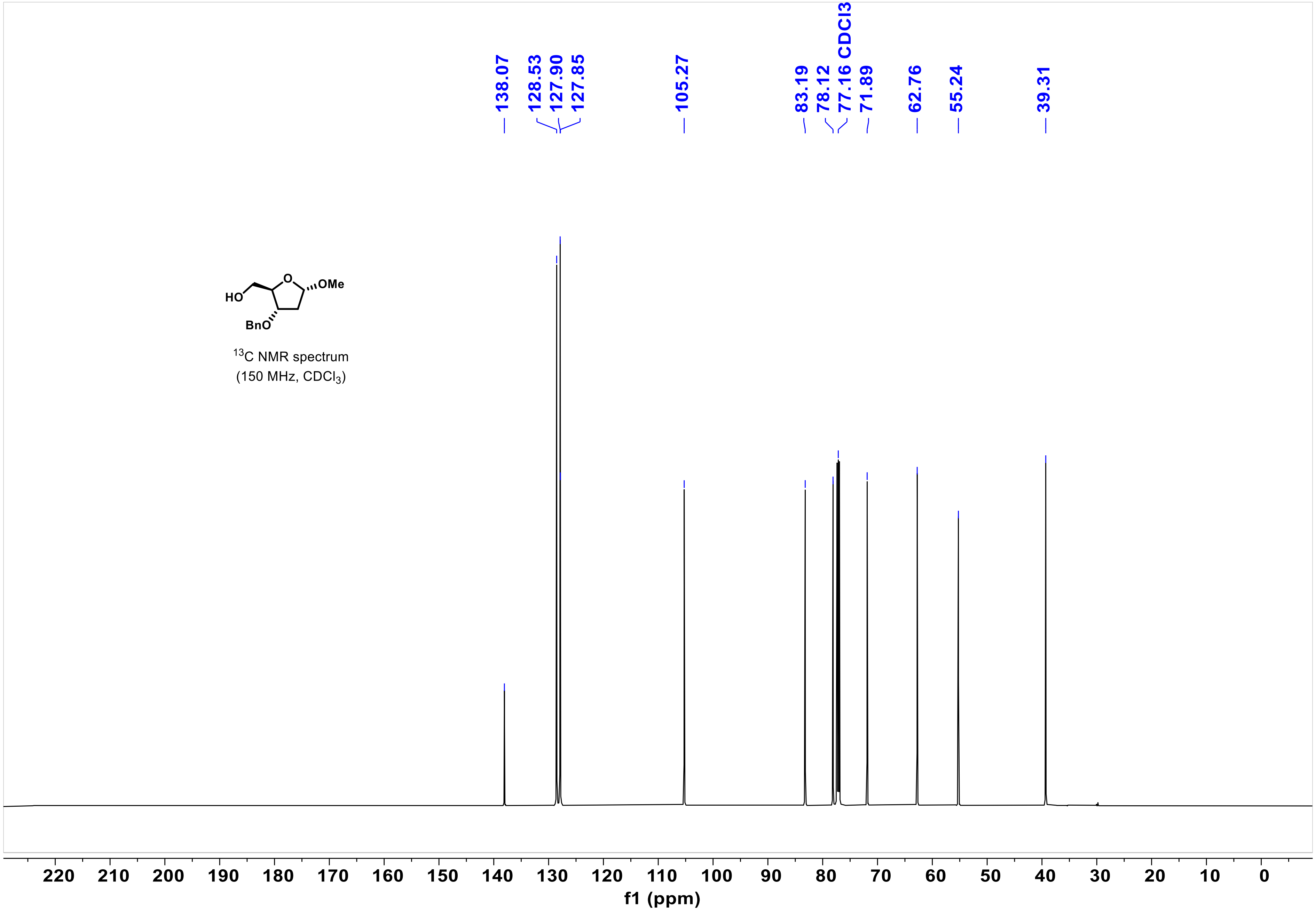

^{13}C NMR of compound **SF25**

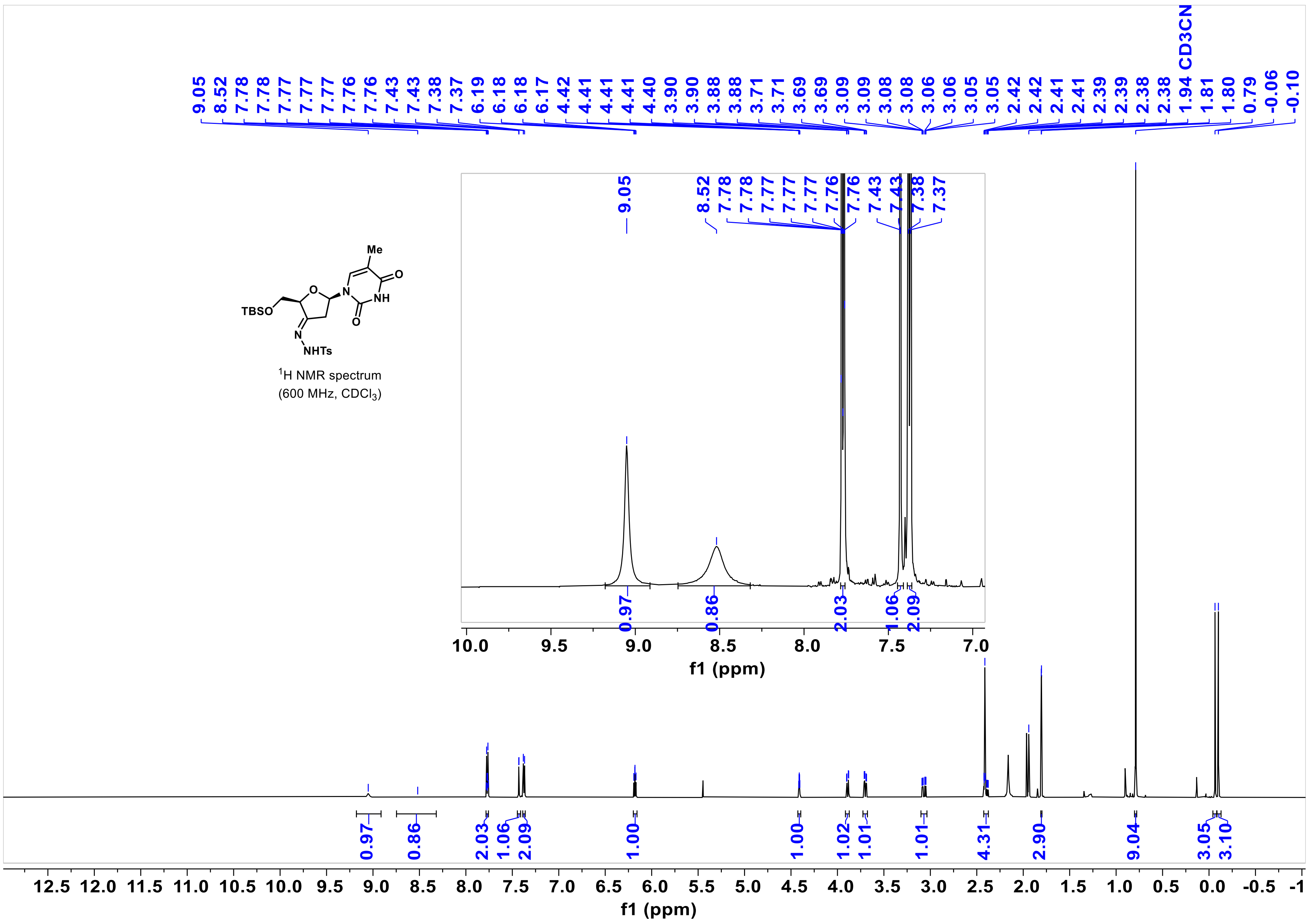

^{1}H NMR of compound **SF27**

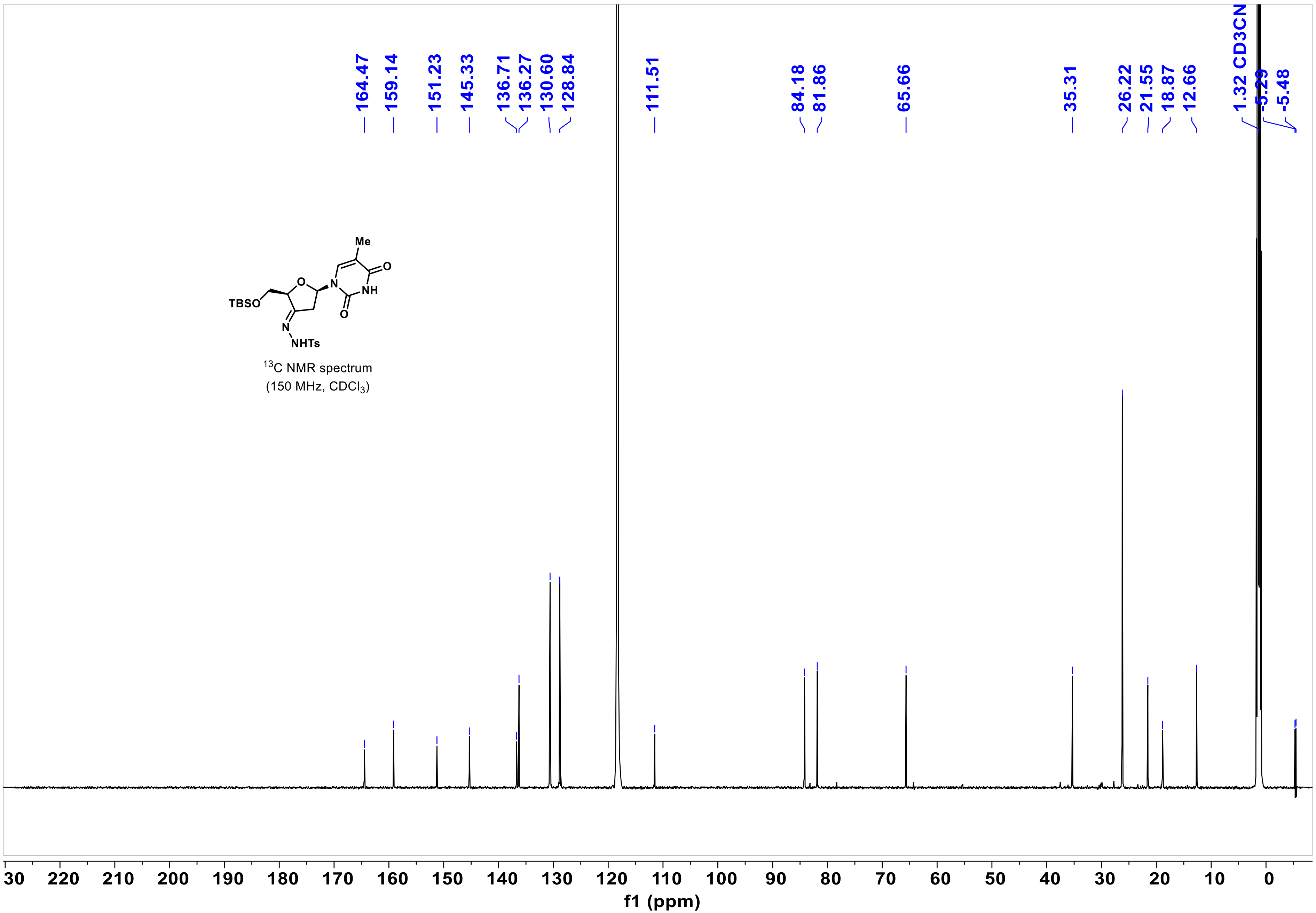

^{13}C NMR of compound **SF27**

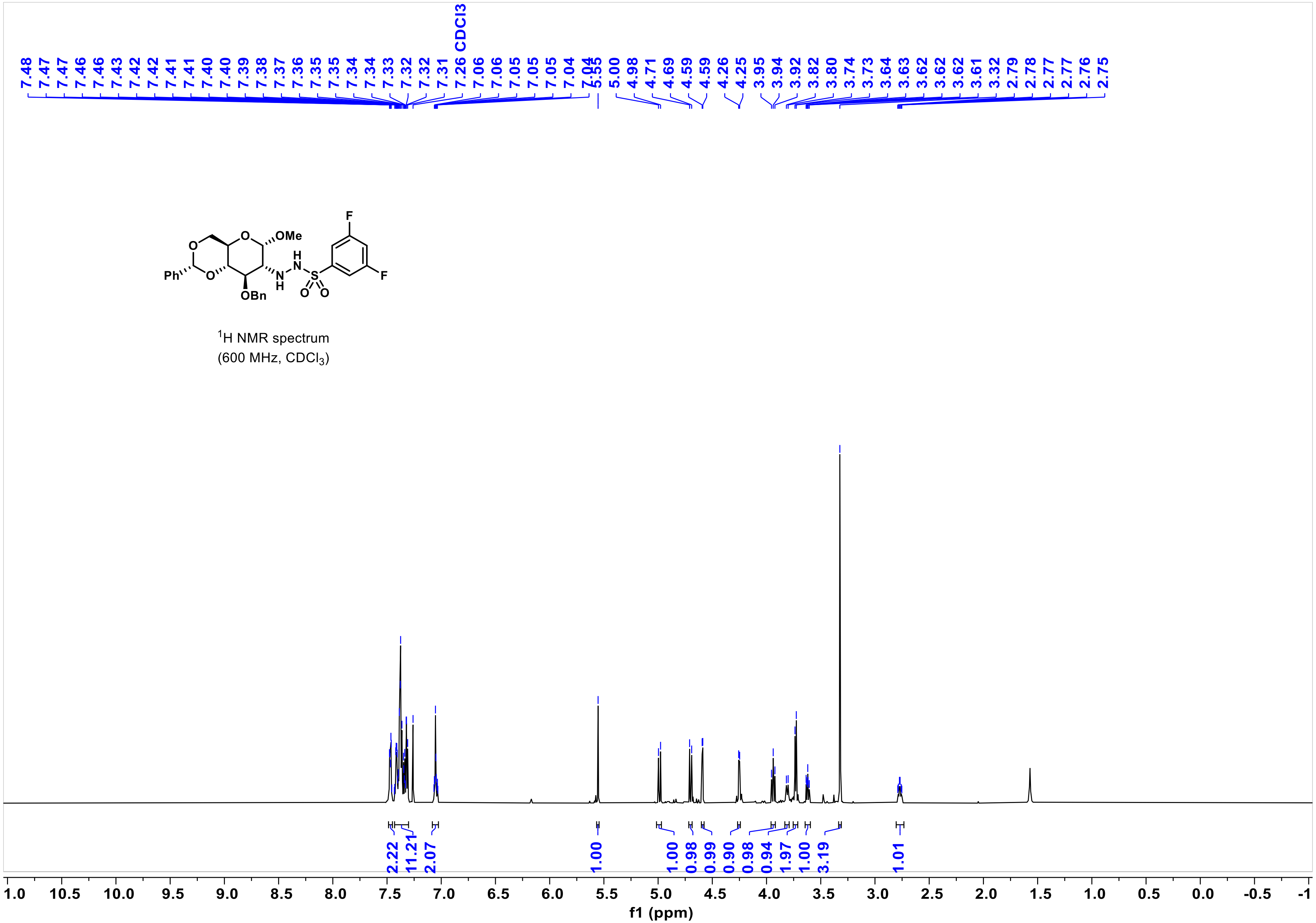

^{1}H NMR of compound **gluco-99**

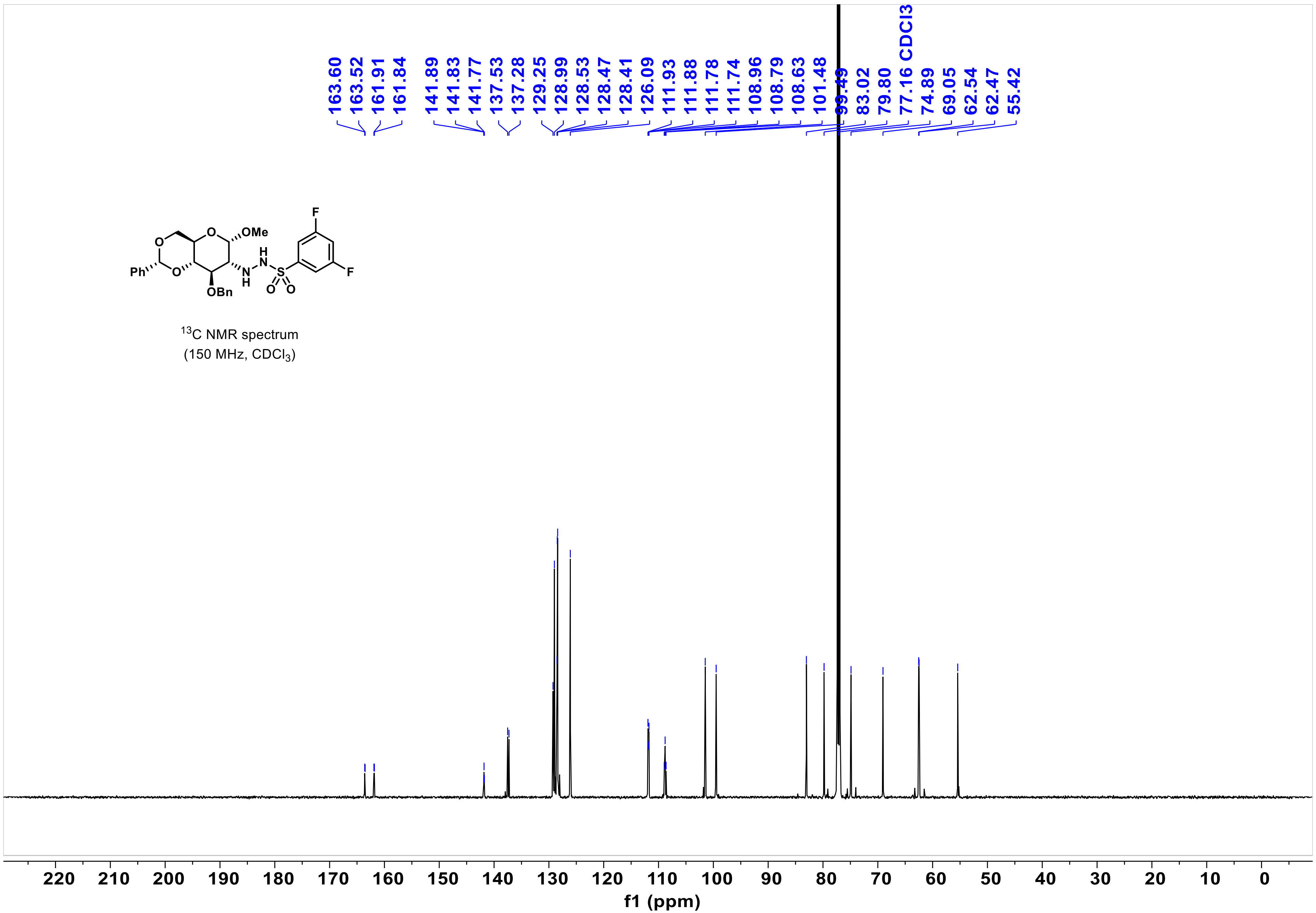

^{13}C NMR of compound **gluco-99**

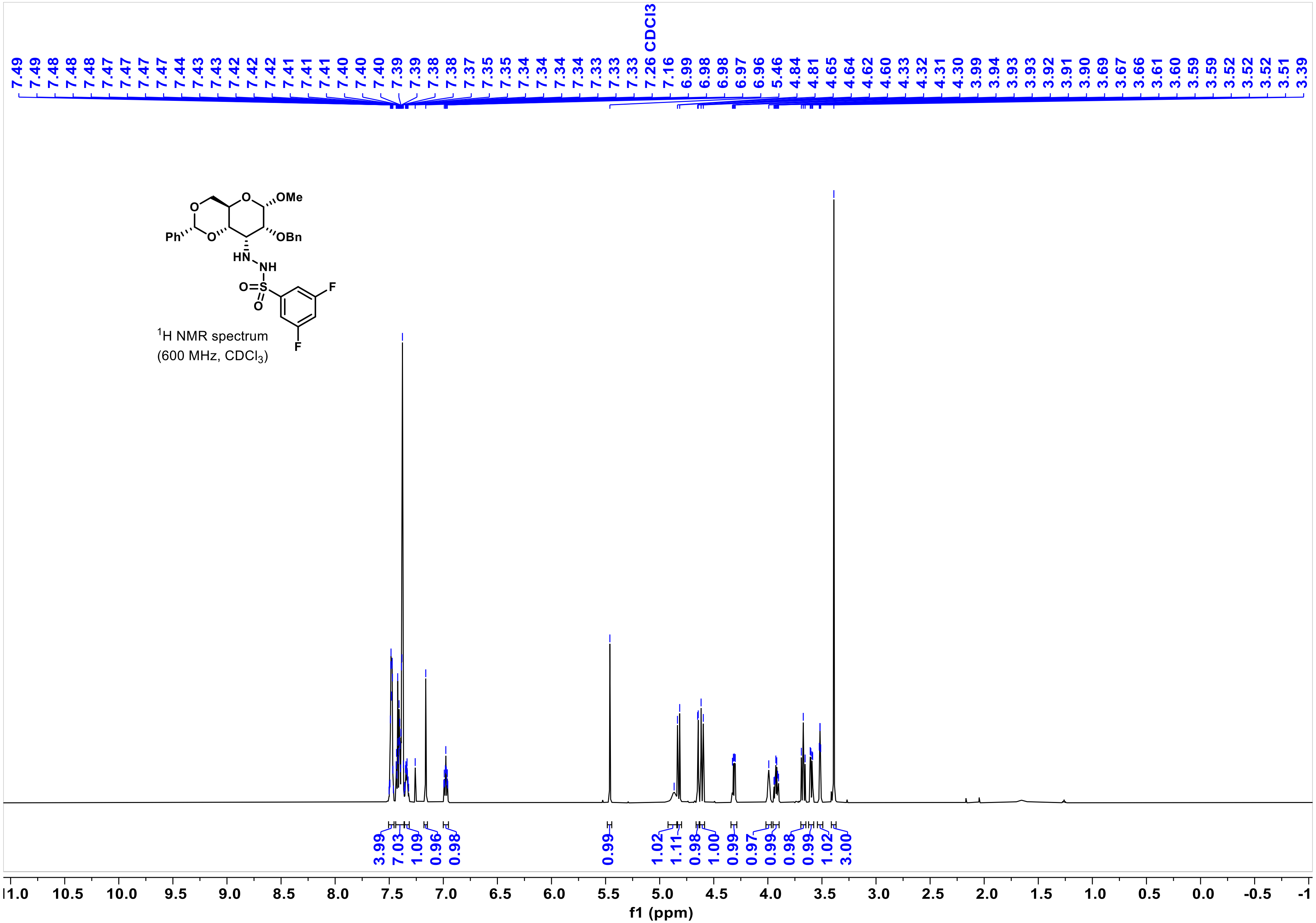

^{1}H NMR of compound **allo-105**

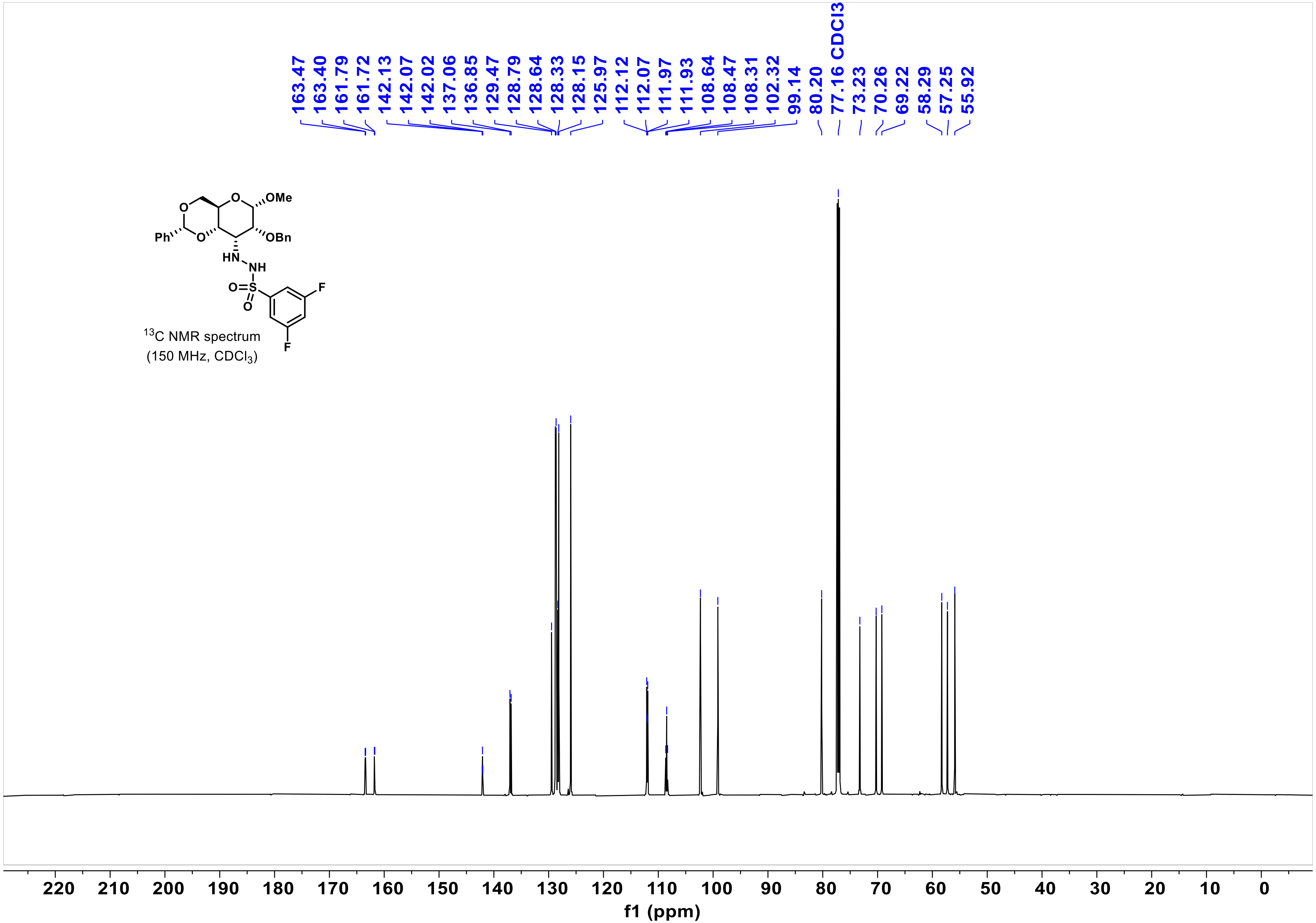

^{13}C NMR of compound **allo-105**

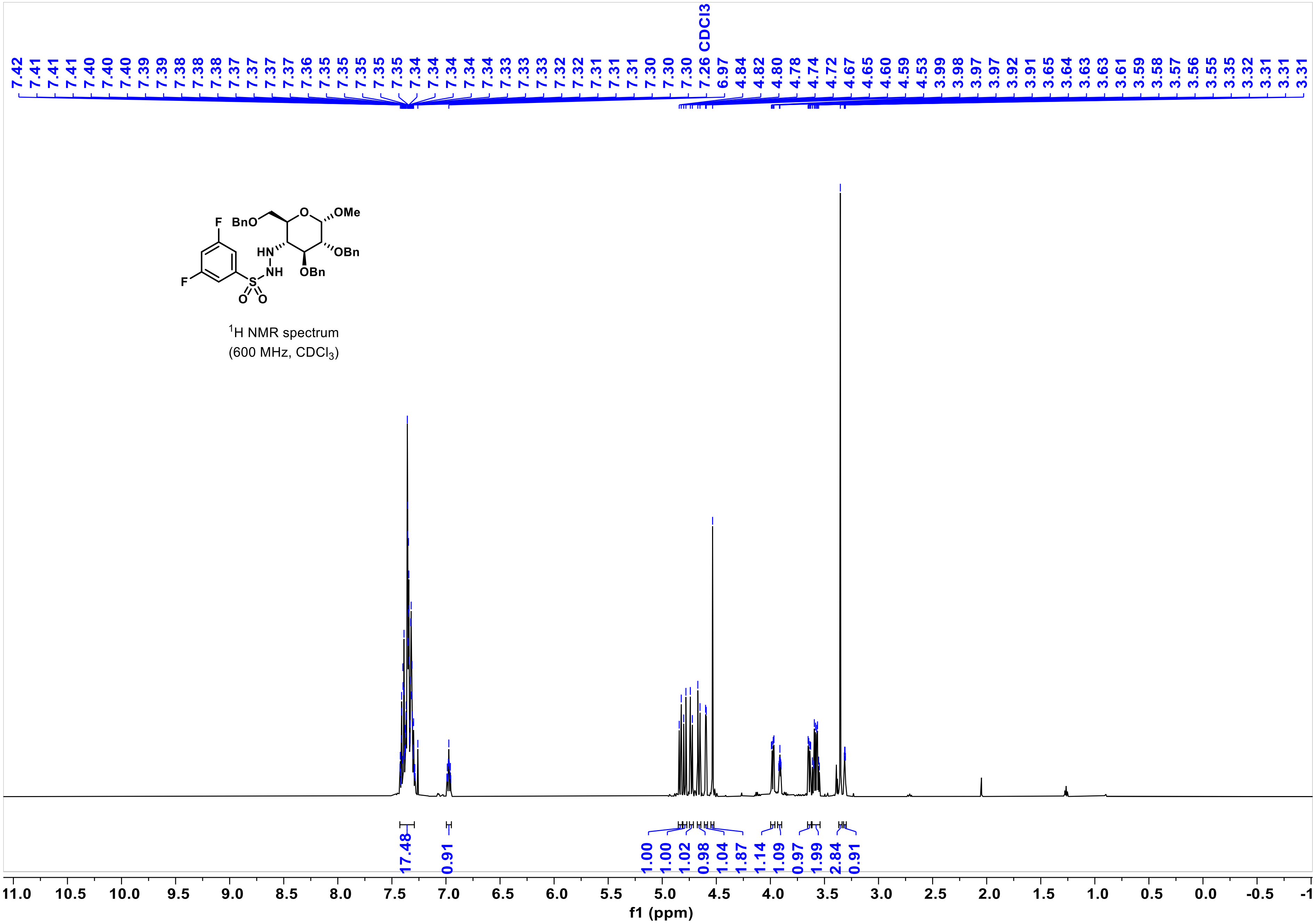

^{1}H NMR of compound **galacto-111**

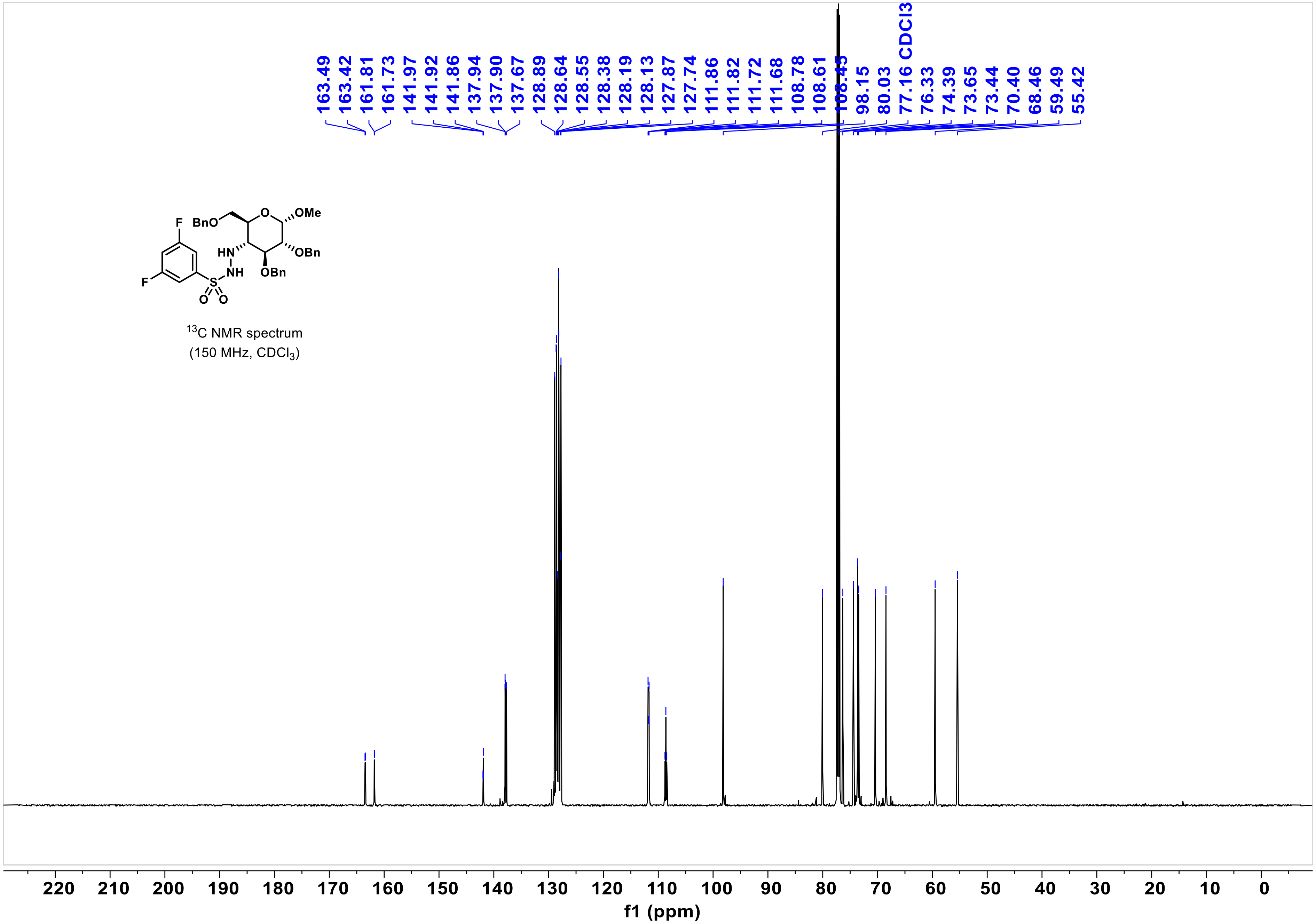

^{13}C NMR of compound **galacto-111**

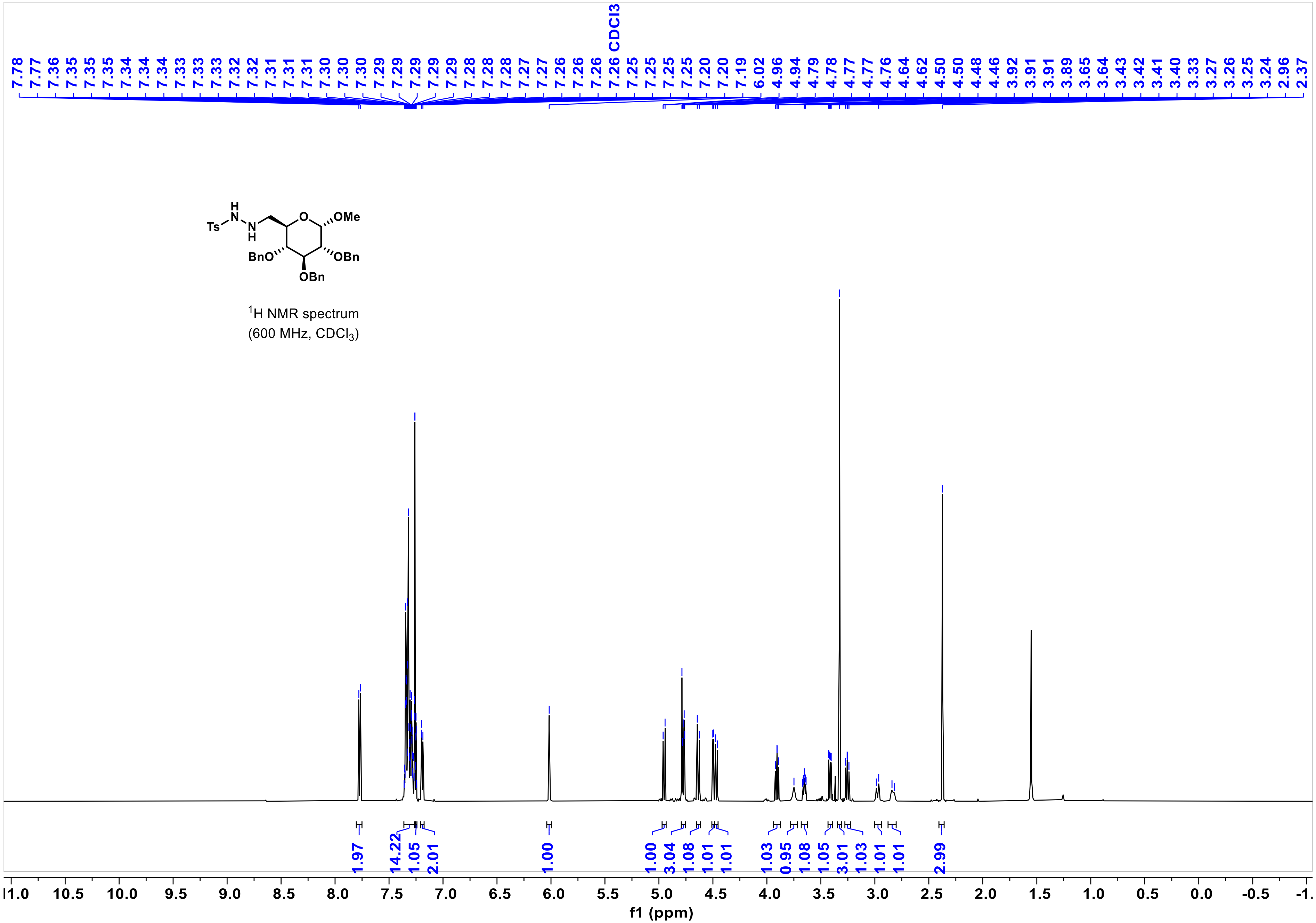

^{1}H NMR of compound **gluco-117**

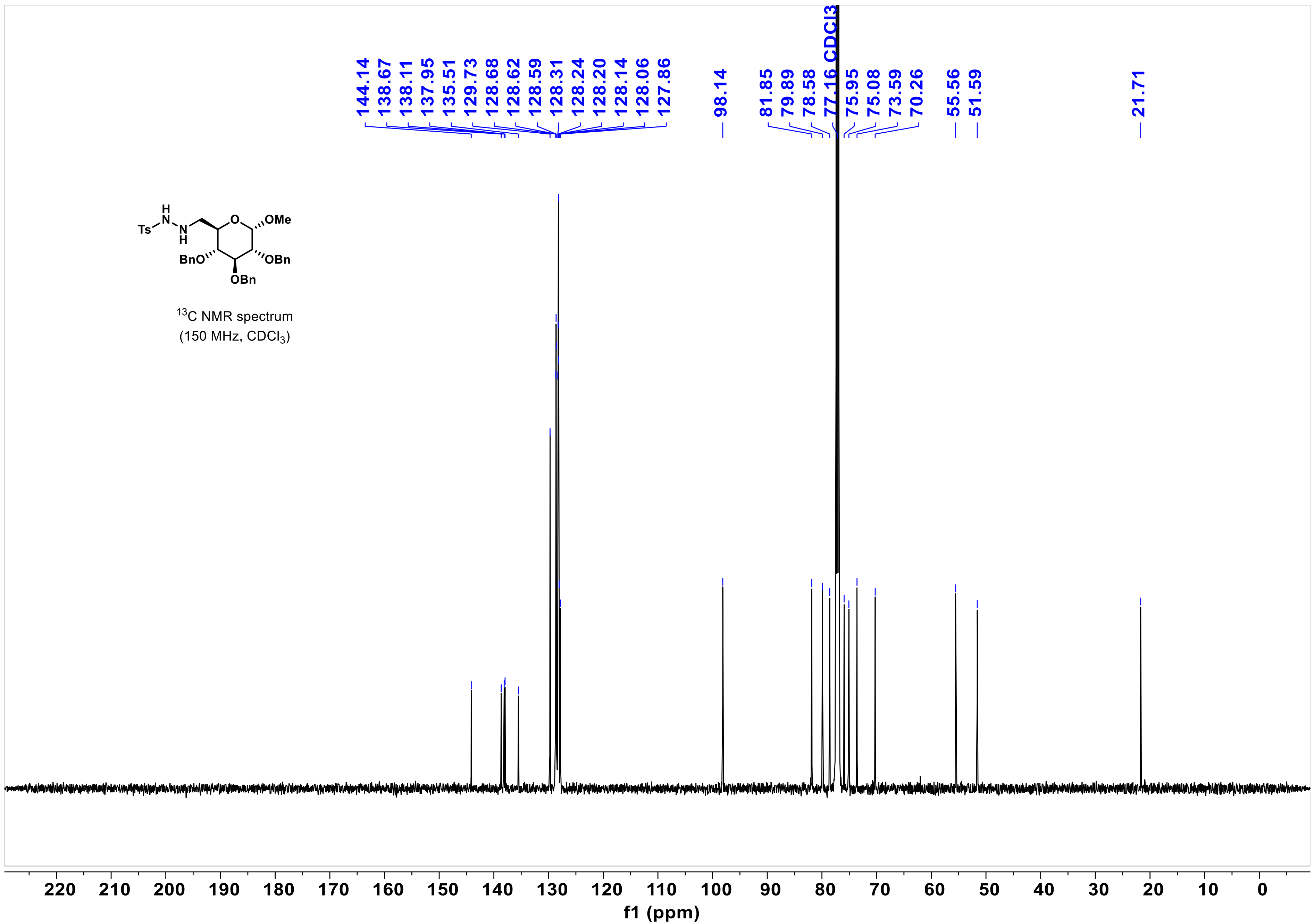

^{13}C NMR of compound **gluco-117**

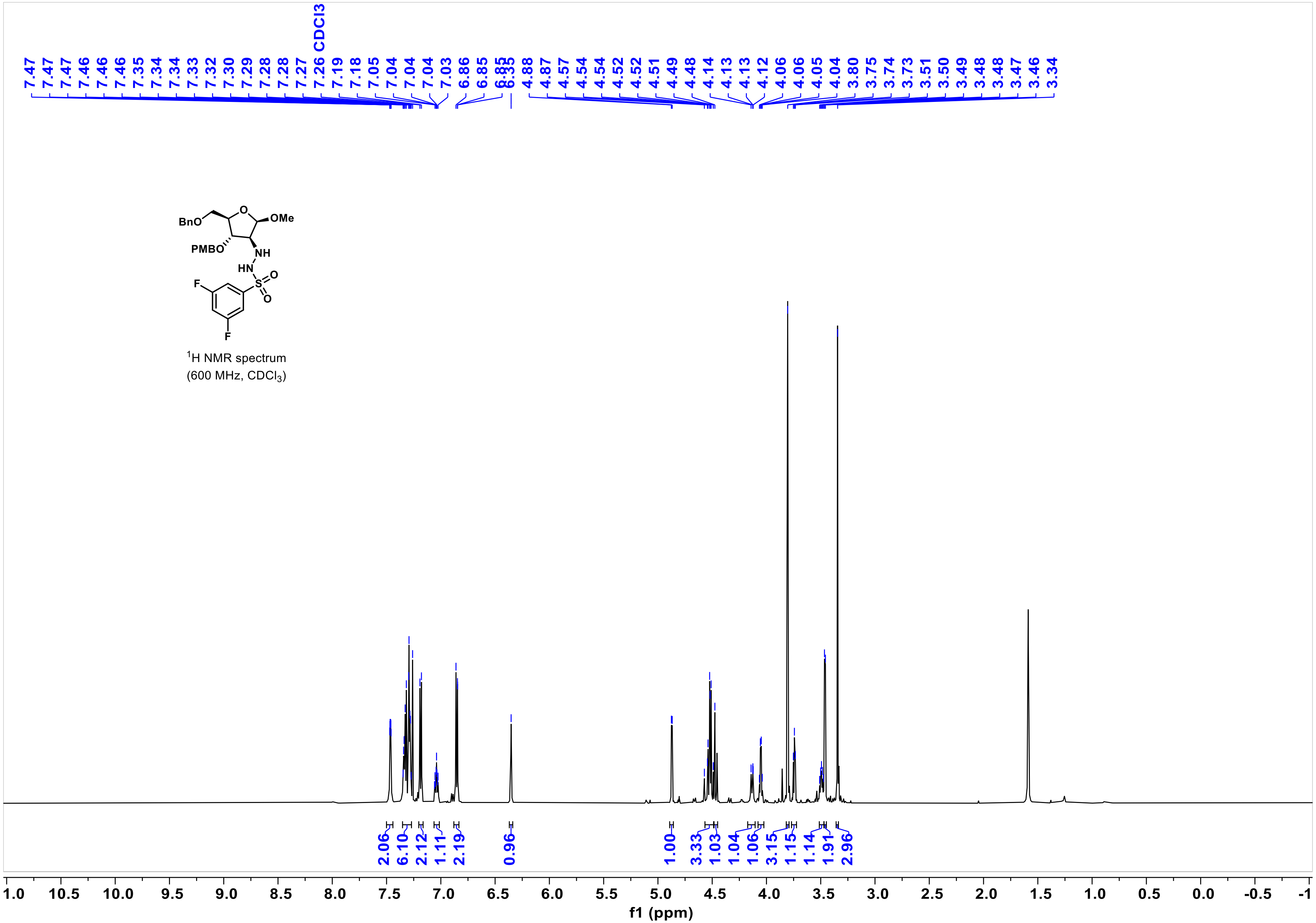

^{1}H NMR of compound **arabino123**

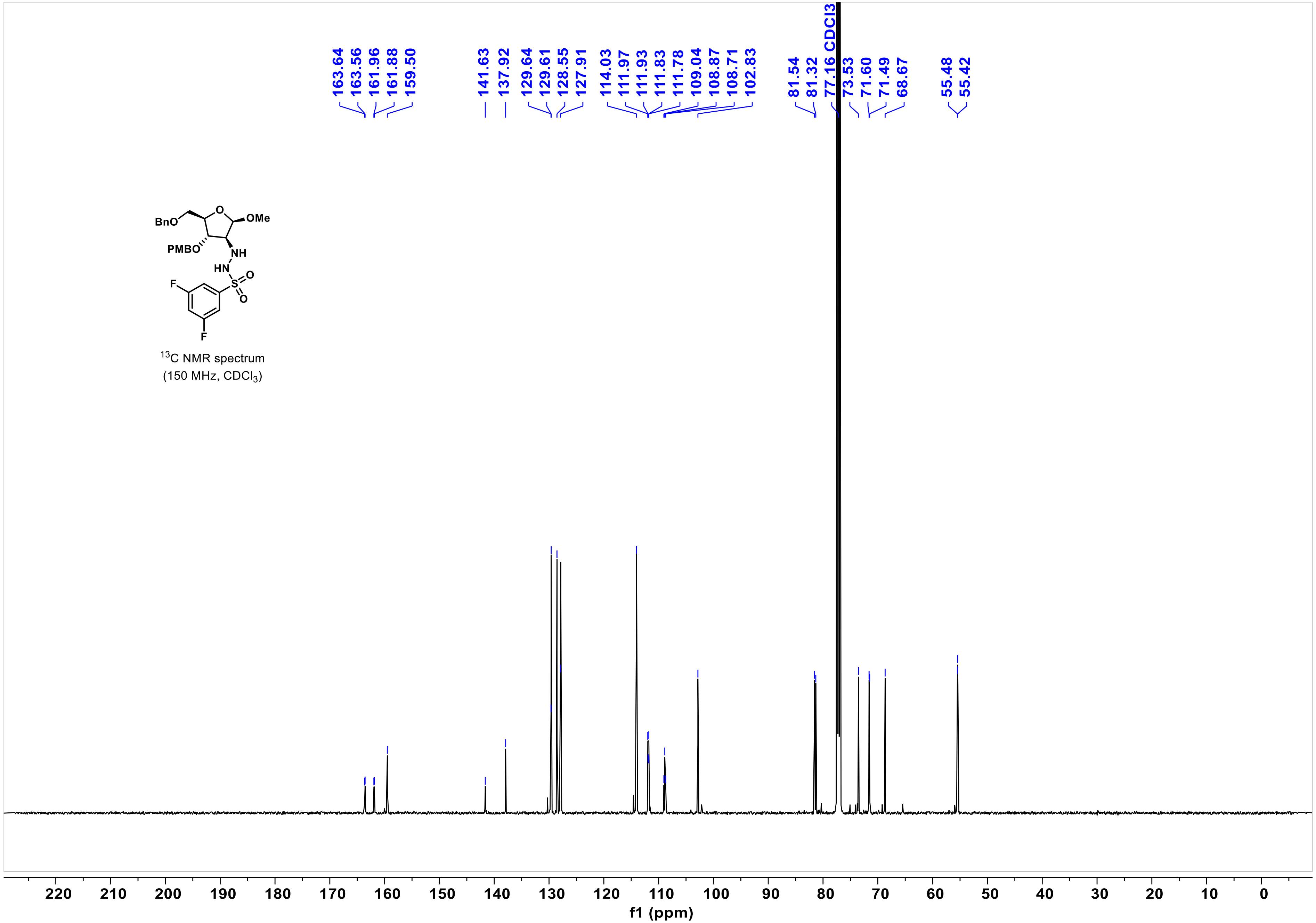

^{13}C NMR of compound **arabino123**

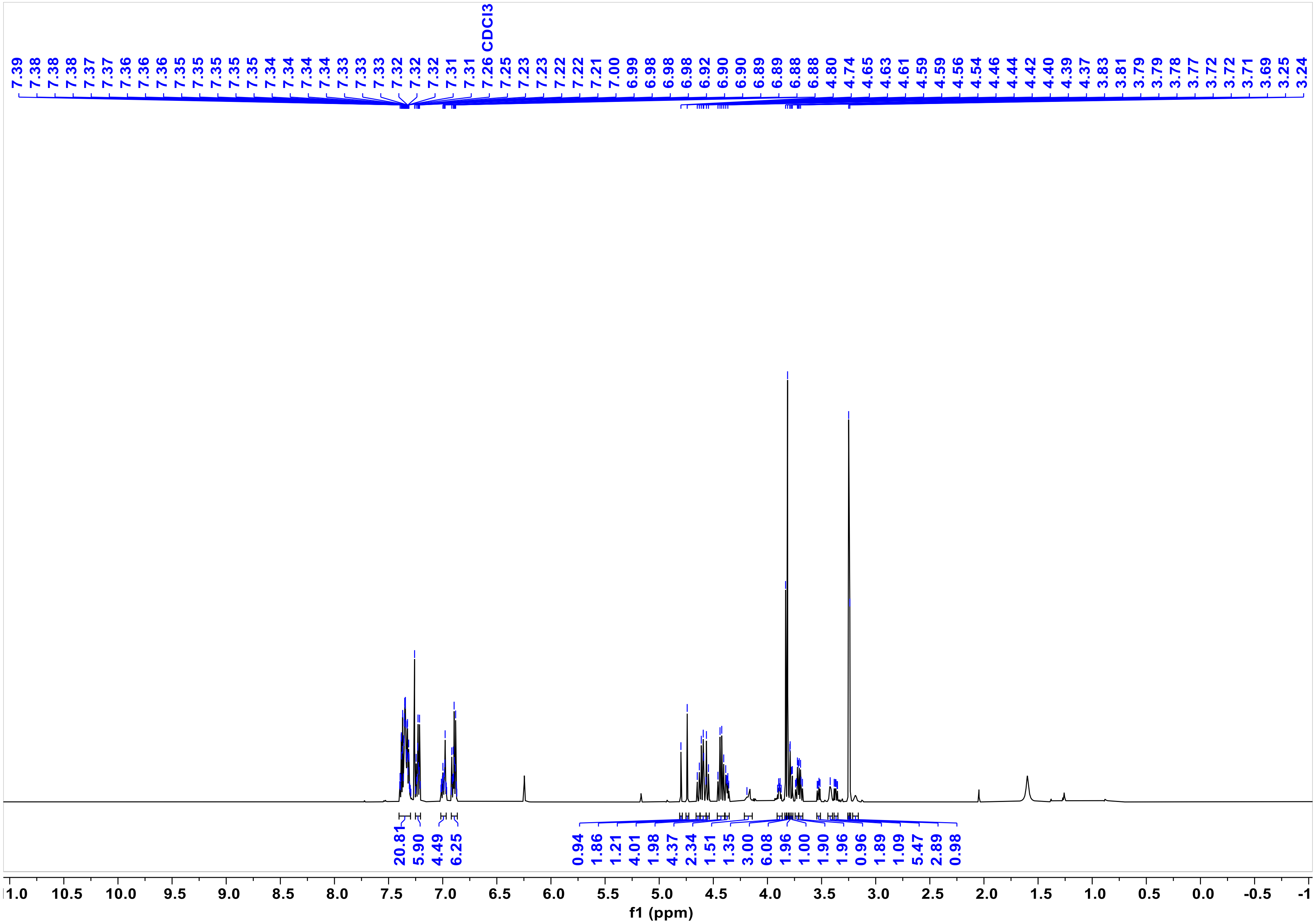

^{1}H NMR of compound **125** (ribo:xylo = 1:2)

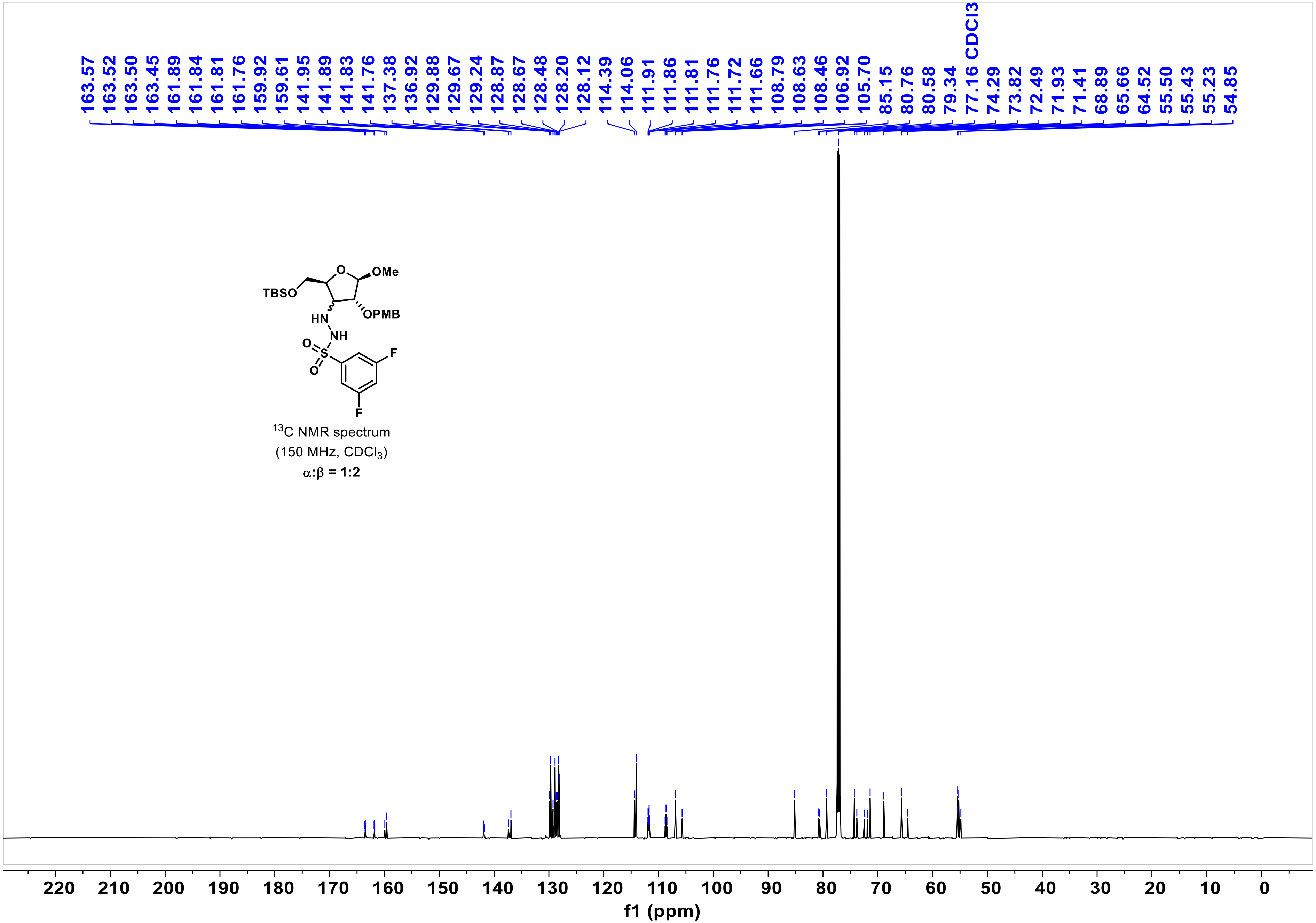

^{13}C NMR of compound **125** (ribo:xylo = 1:2)

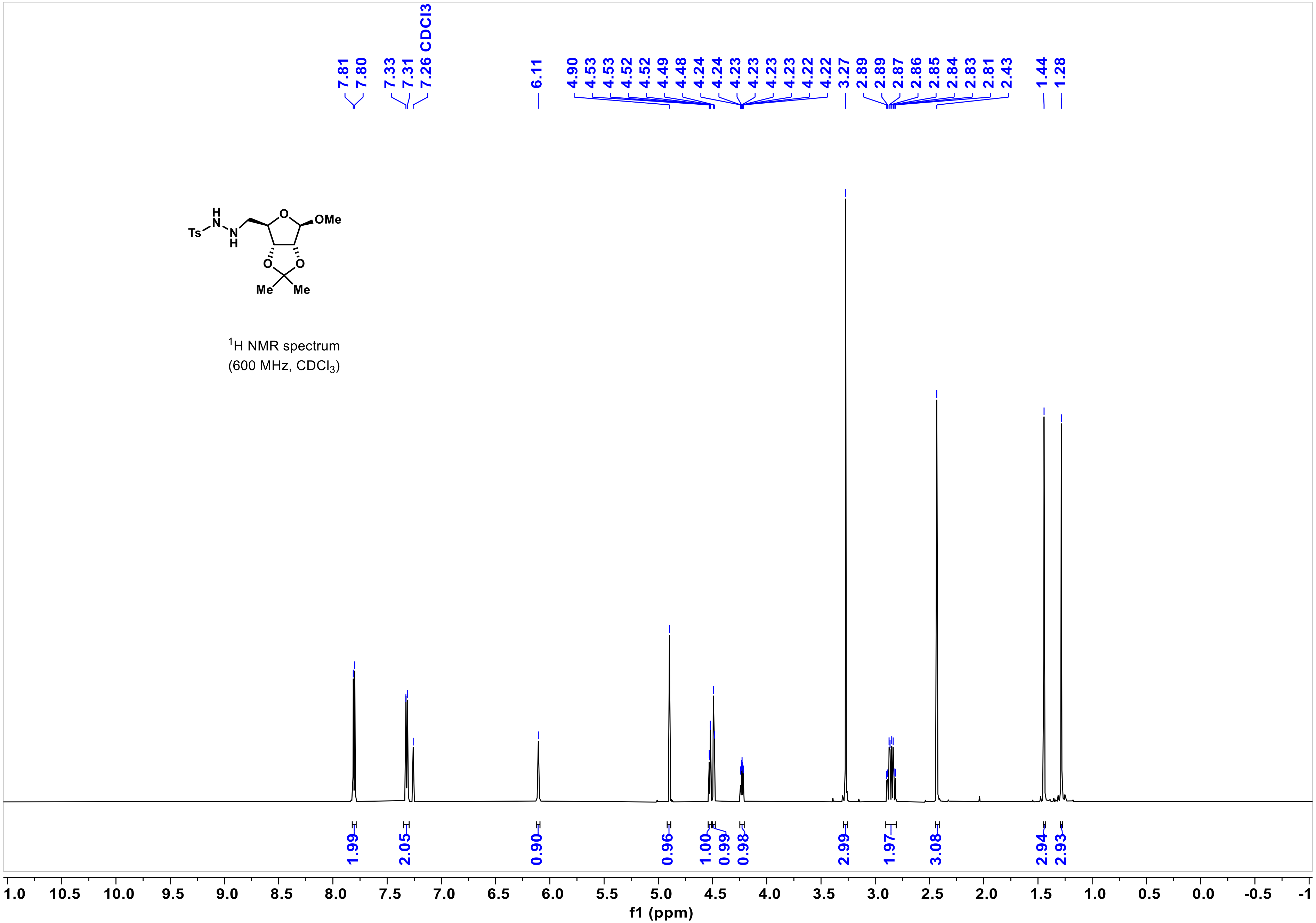

^{1}H NMR of compound **127**

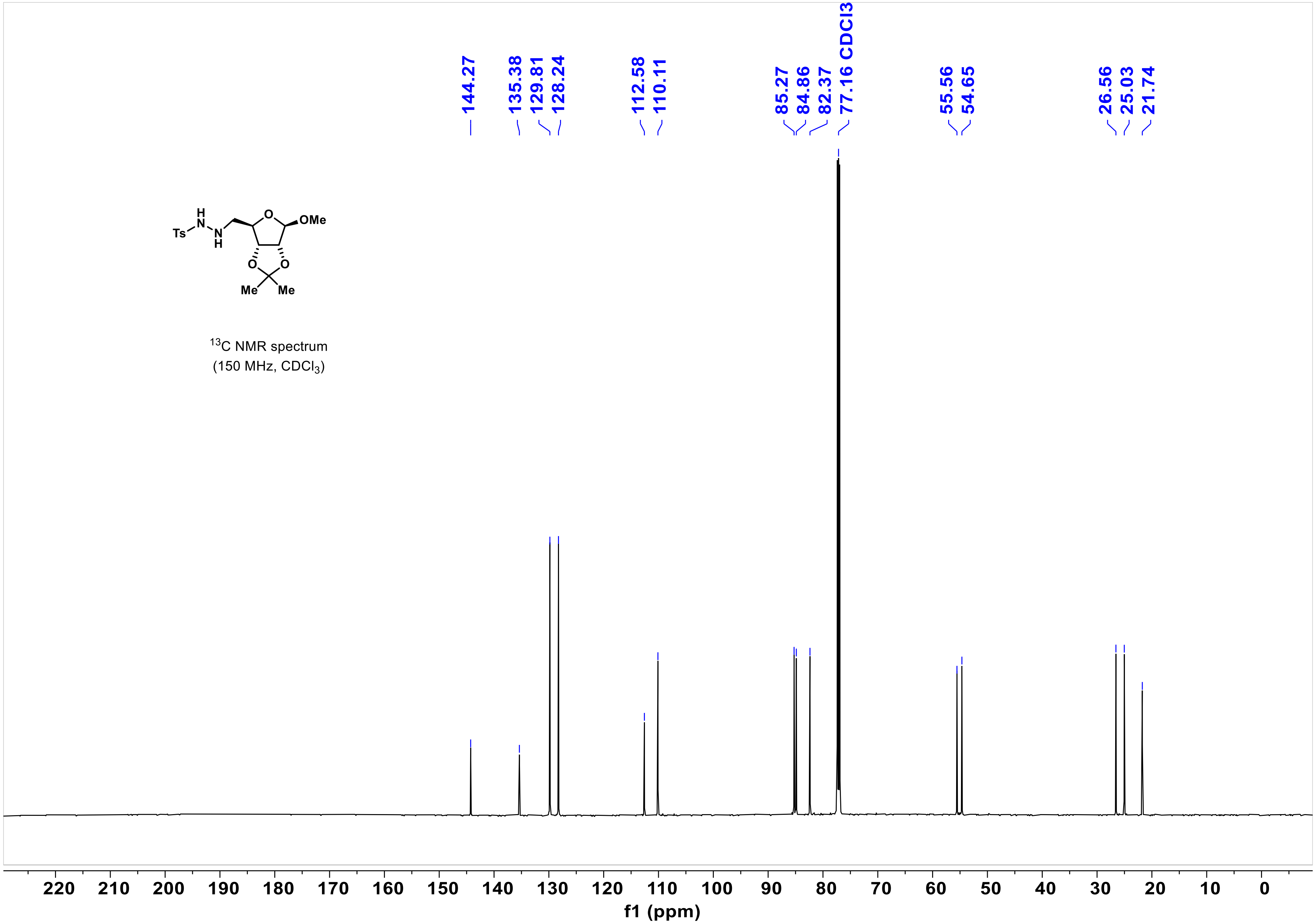

^{13}C NMR of compound **127**

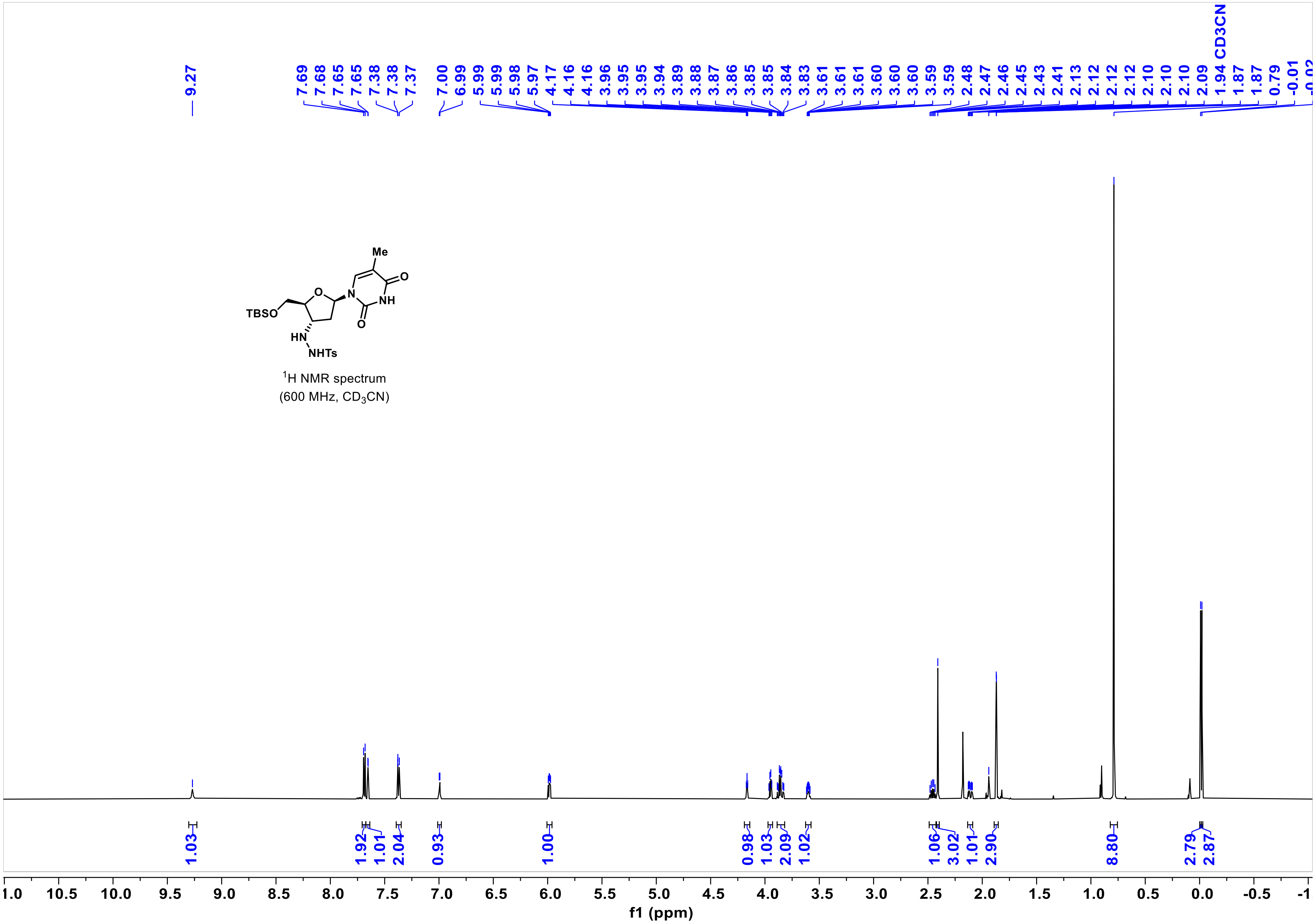

^{1}H NMR of compound **2-deoxy-xylo-129**

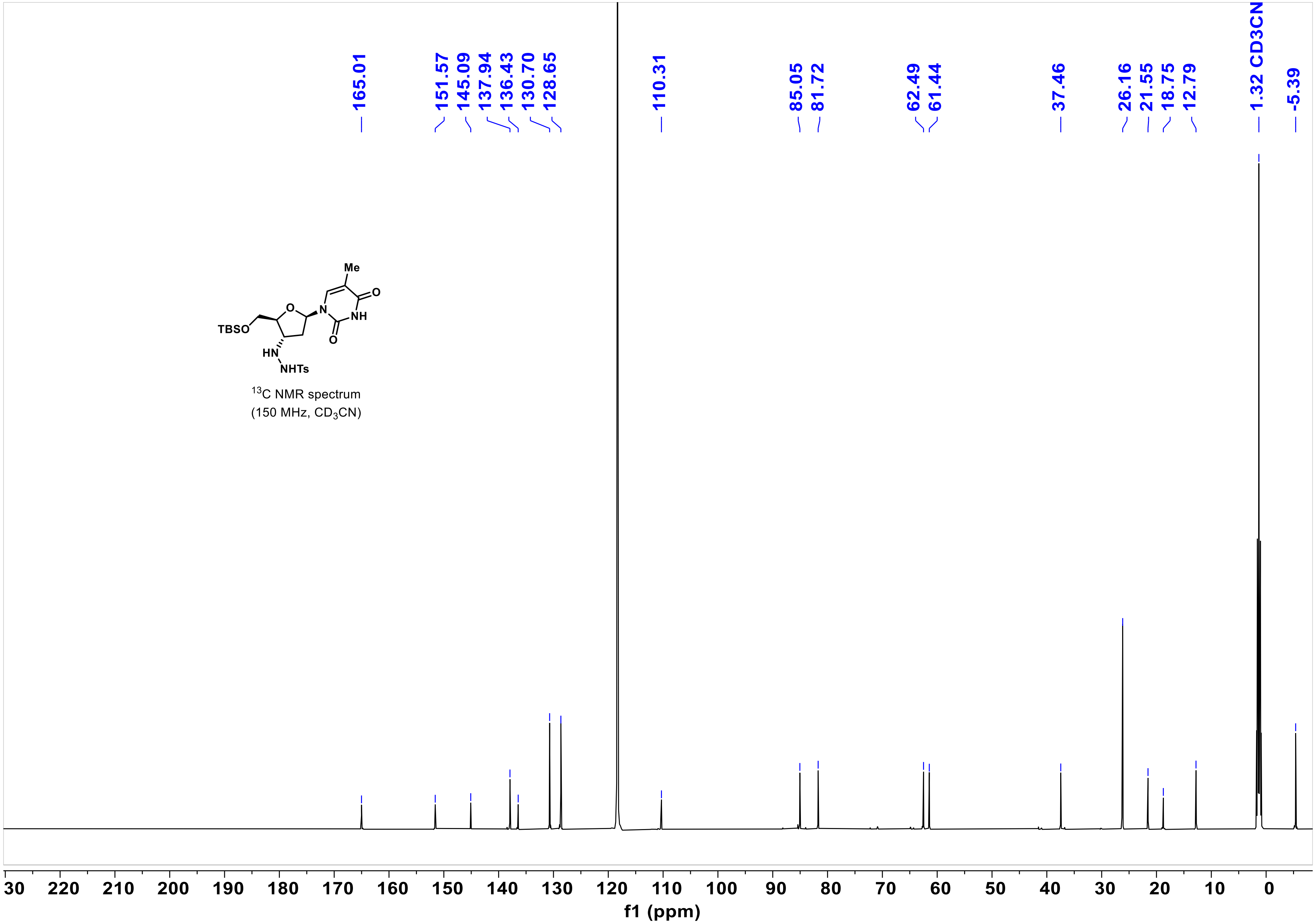

^{13}C NMR of compound **2-deoxy-xylo-129**

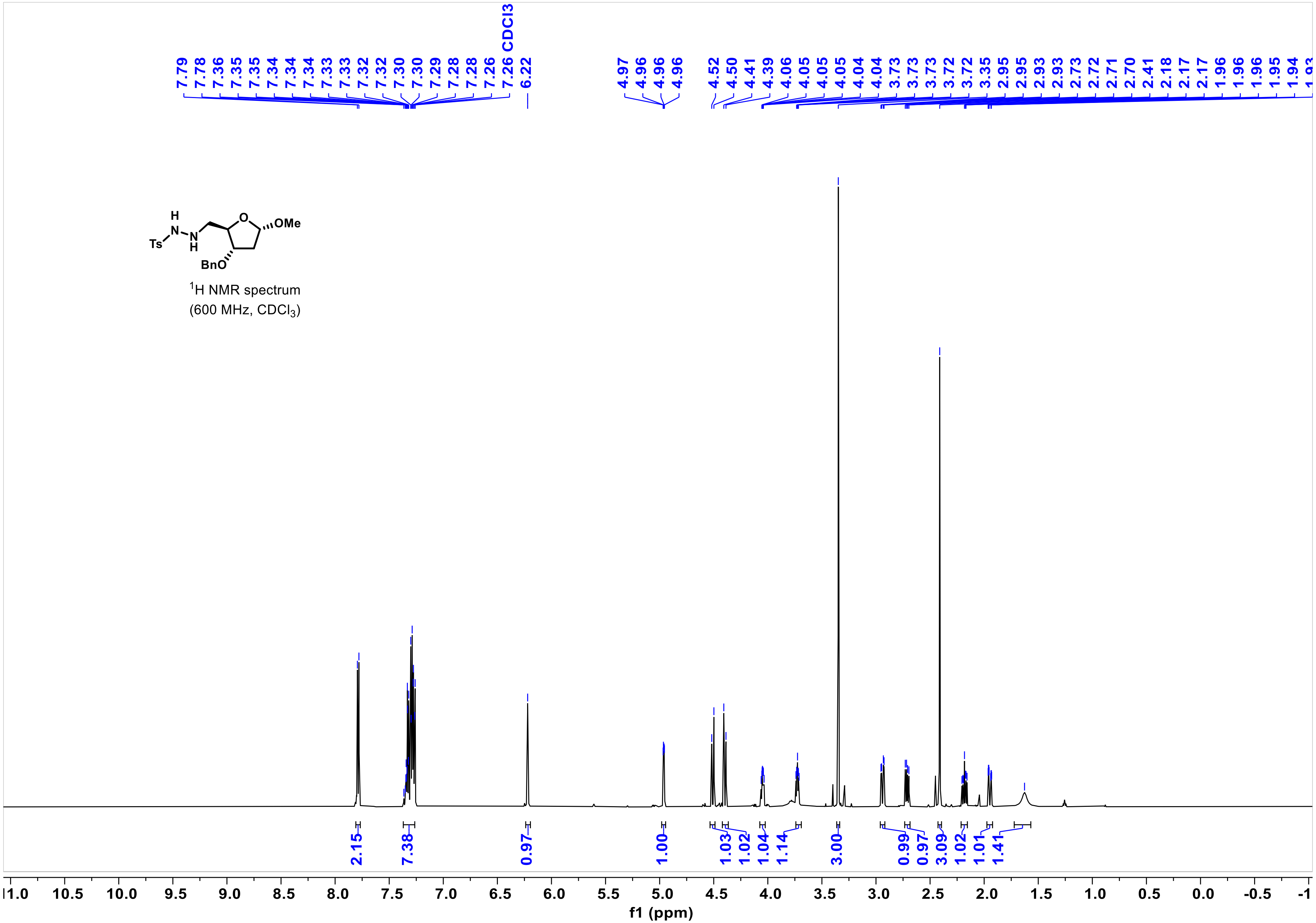

^{1}H NMR of compound **2-deoxy-ribo-131**

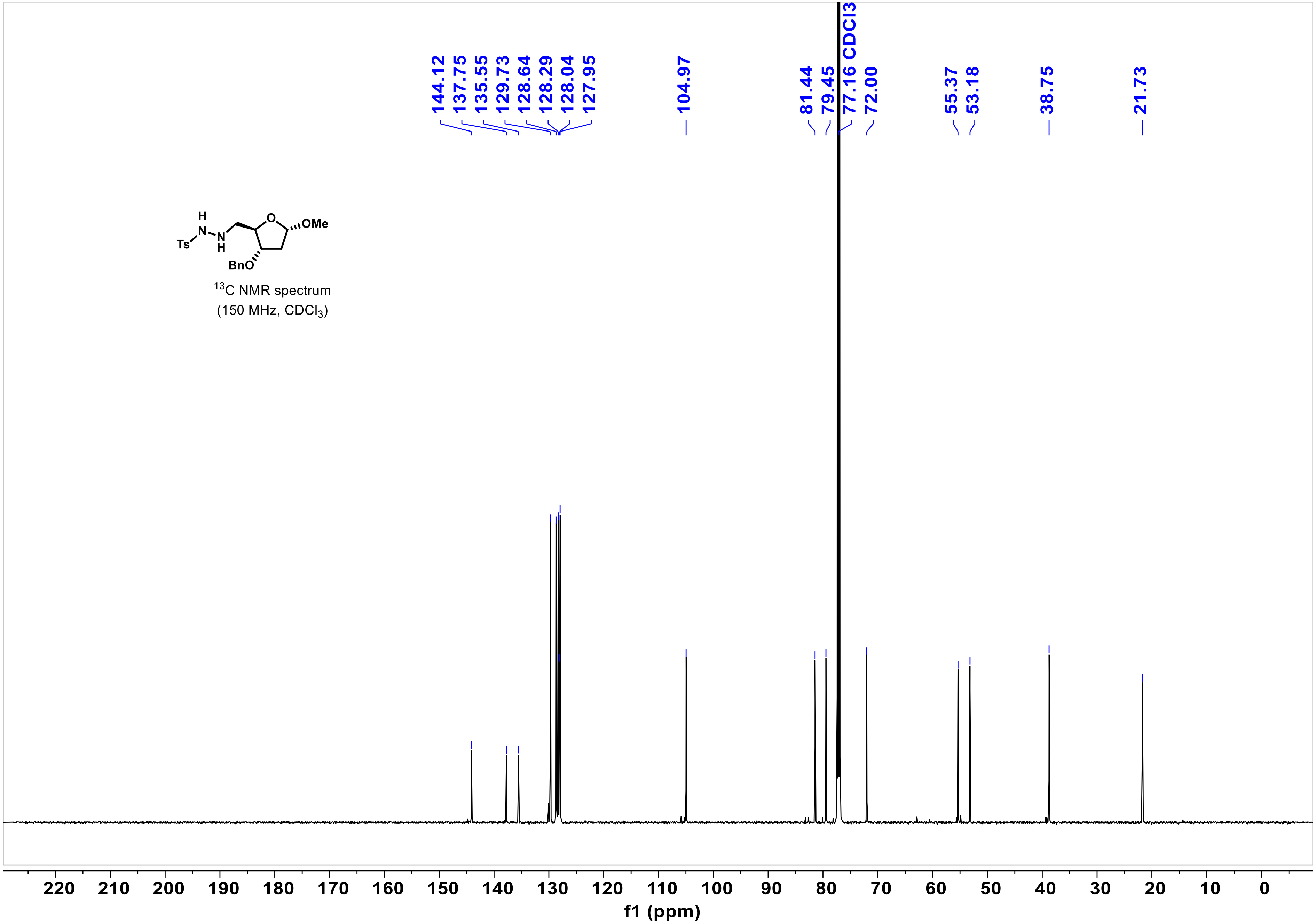

^{13}C NMR of compound **2-deoxy-ribo-131**

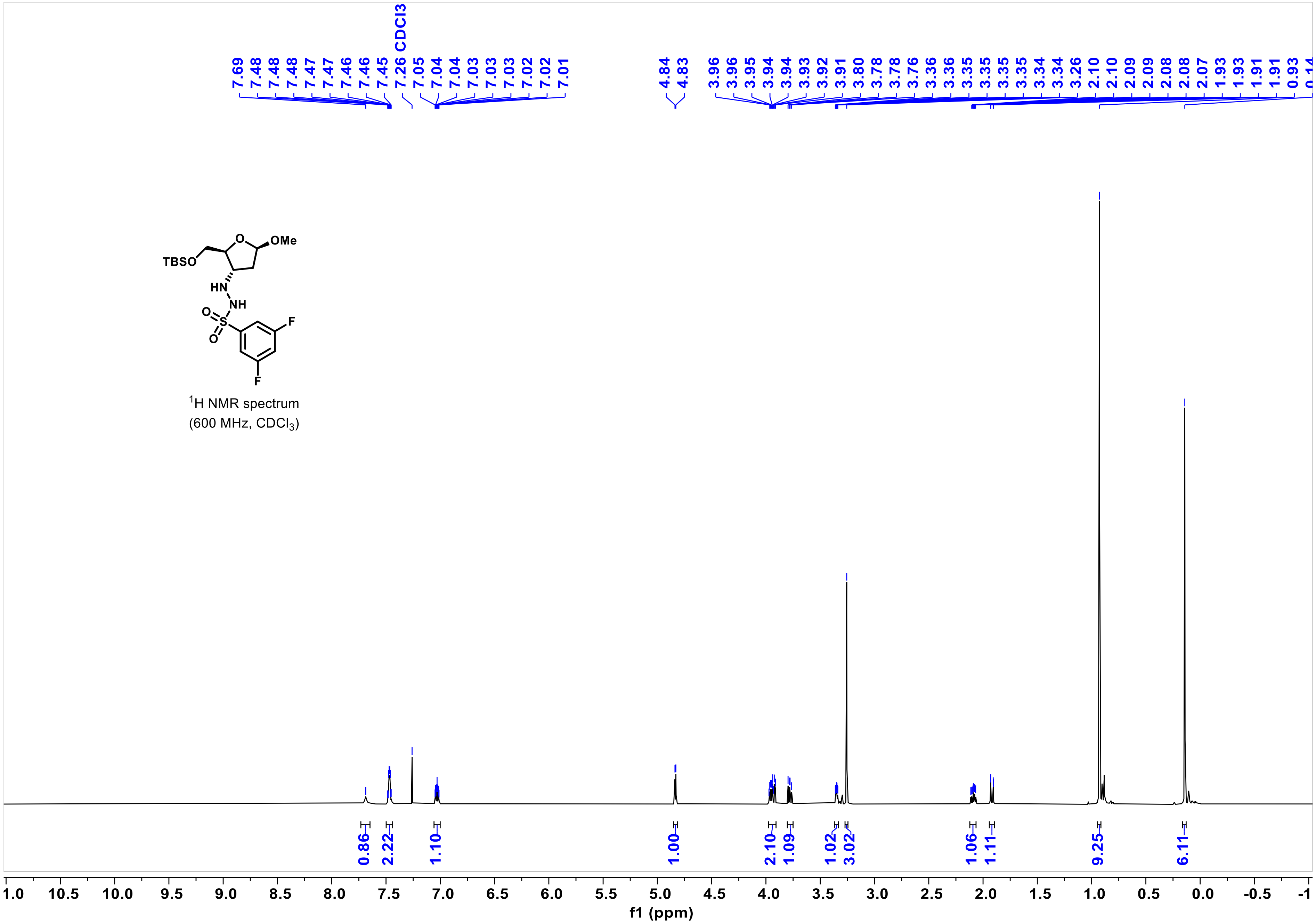

^{1}H NMR of compound **2-deoxy-xylo-SF22**

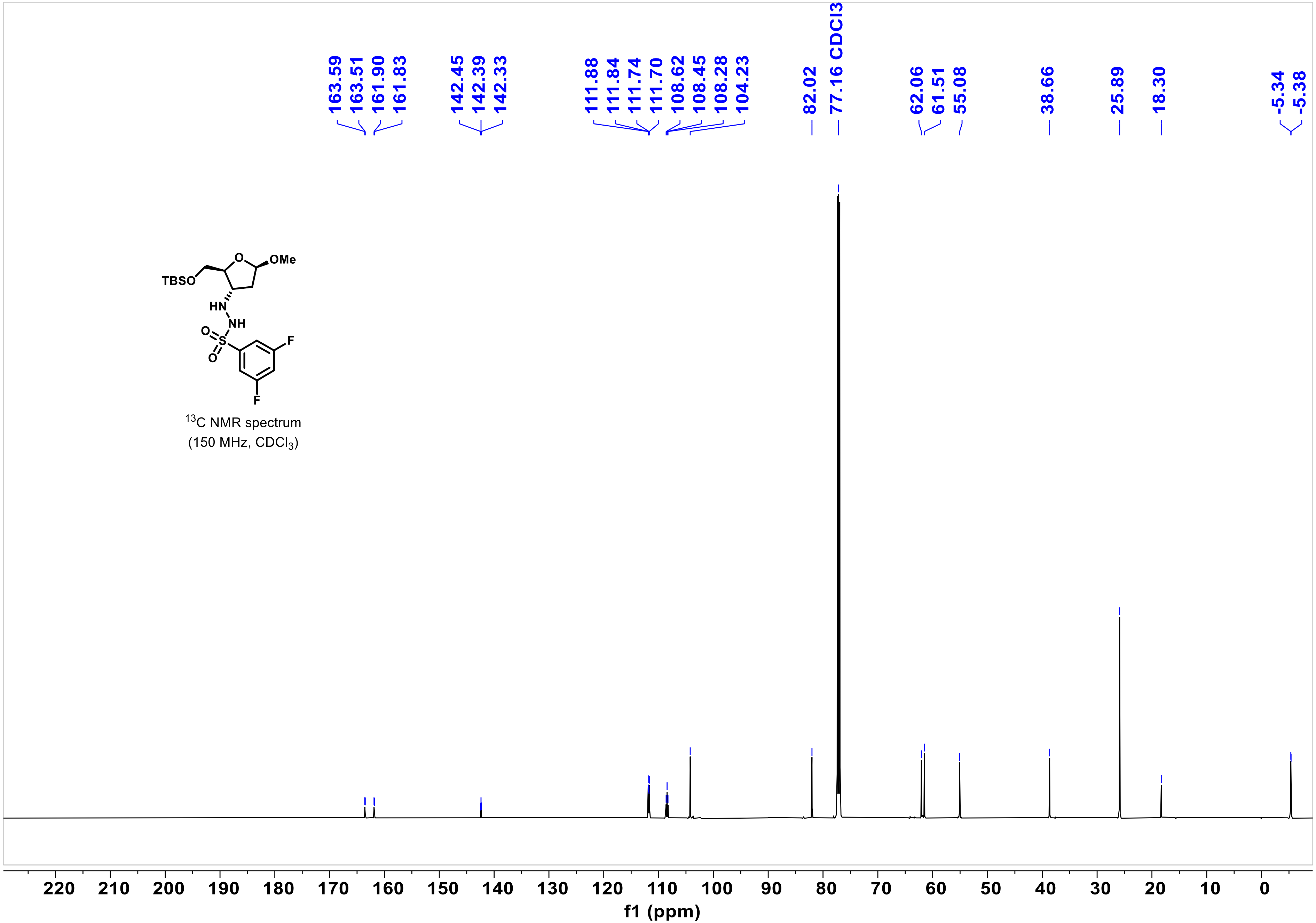

^{13}C NMR of compound **2-deoxy-xylo-SF22**

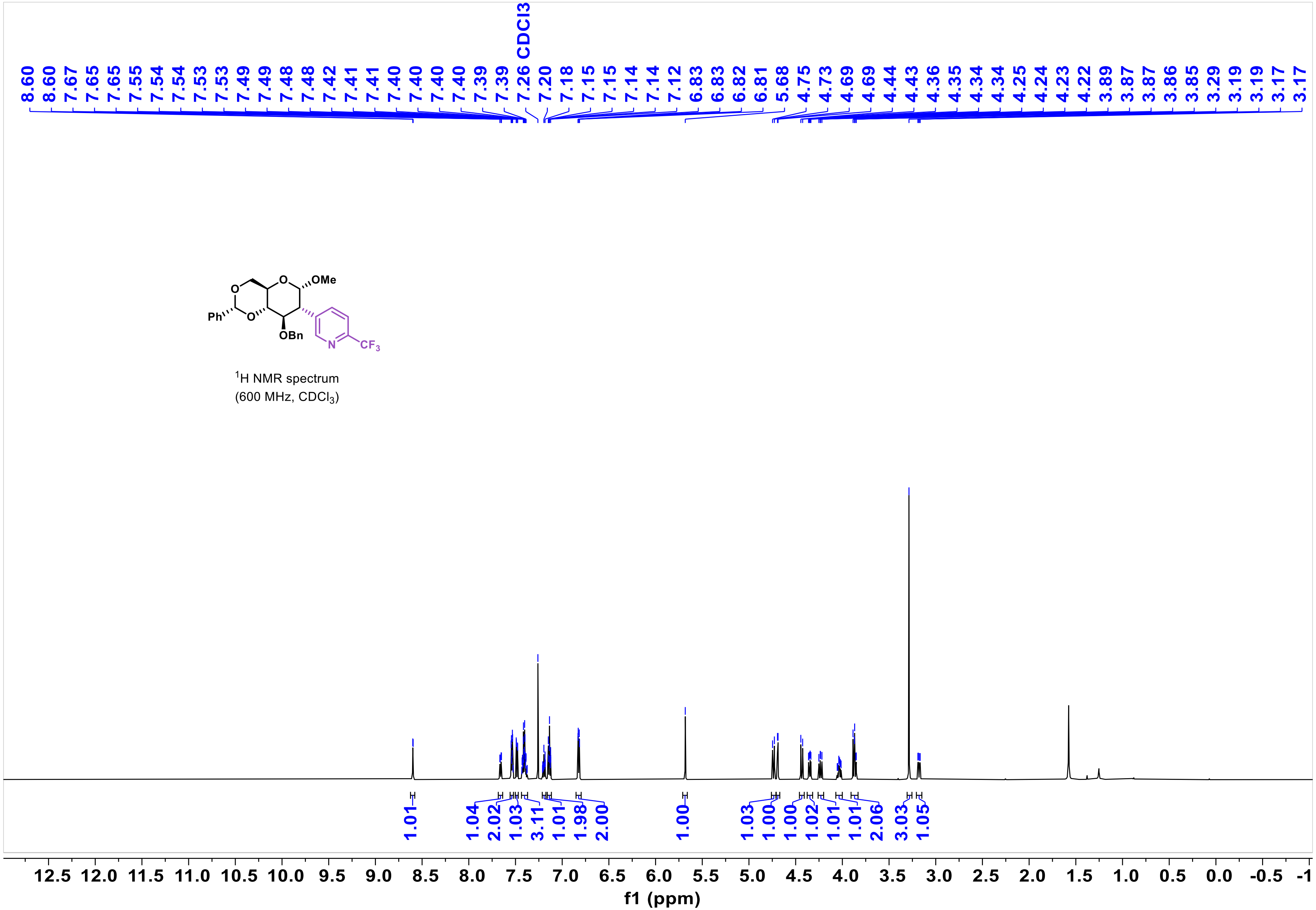

^{1}H NMR of compound **g-101**

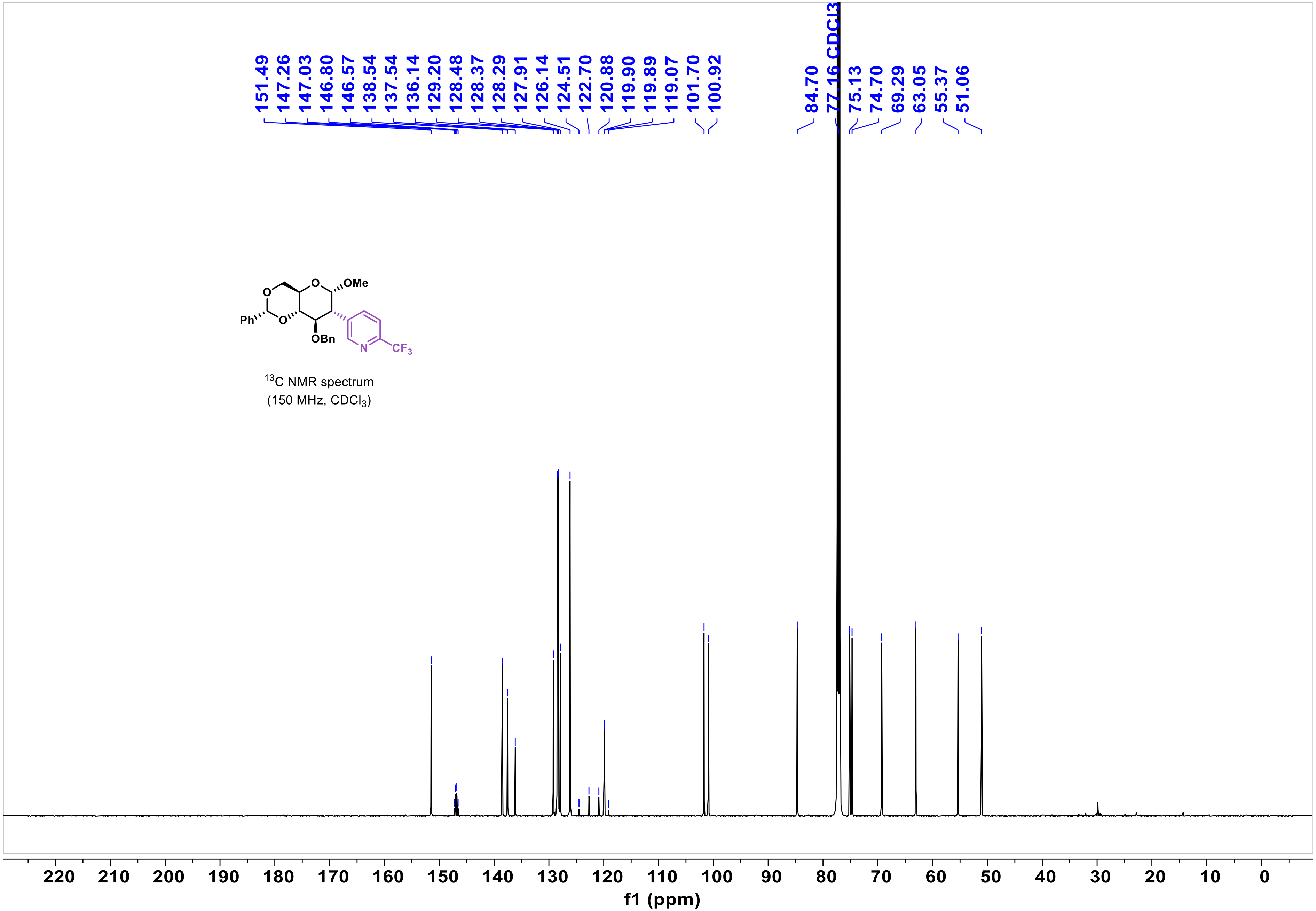

^{13}C NMR of compound **g-101**

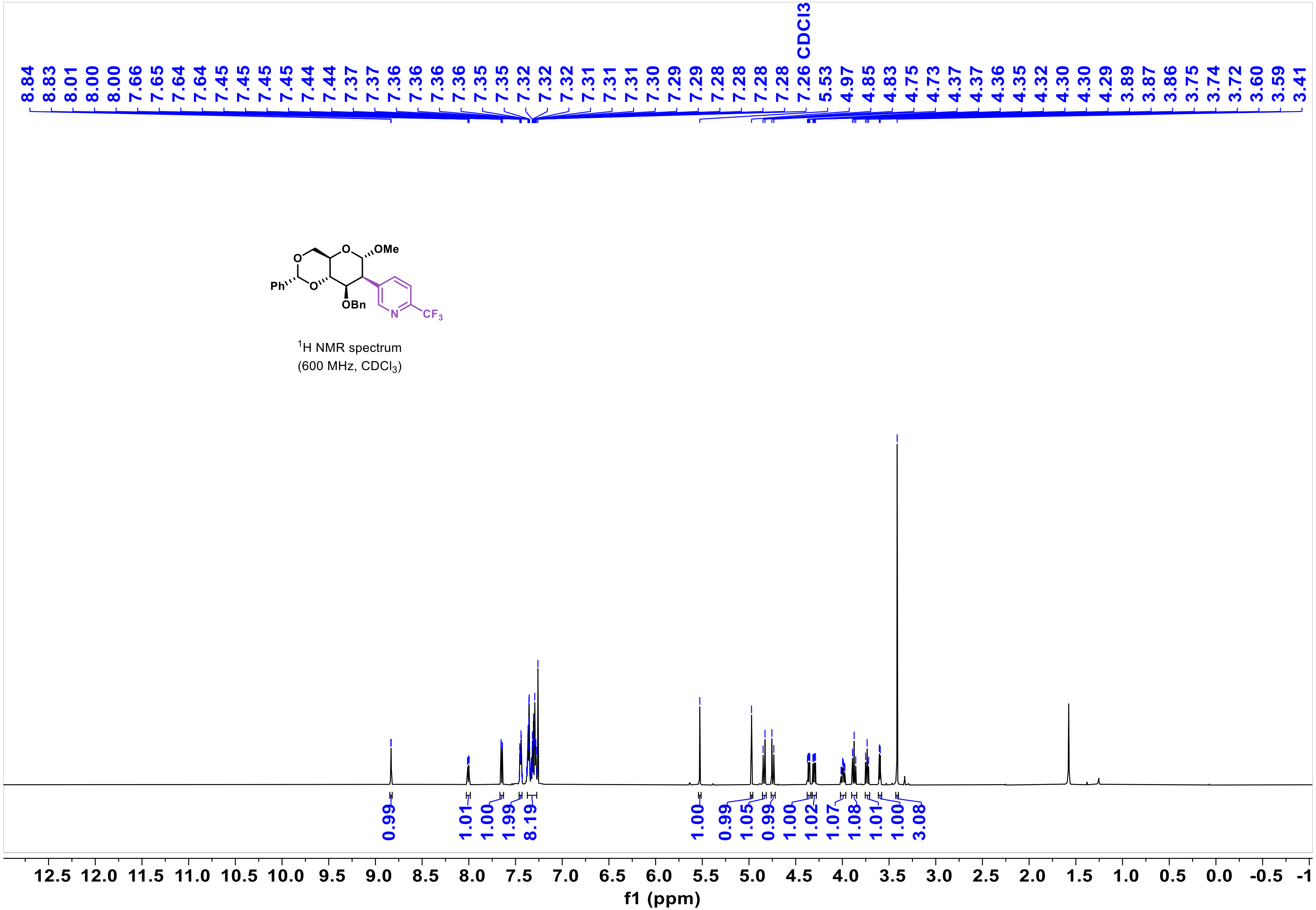

^{1}H NMR of compound **m-101**

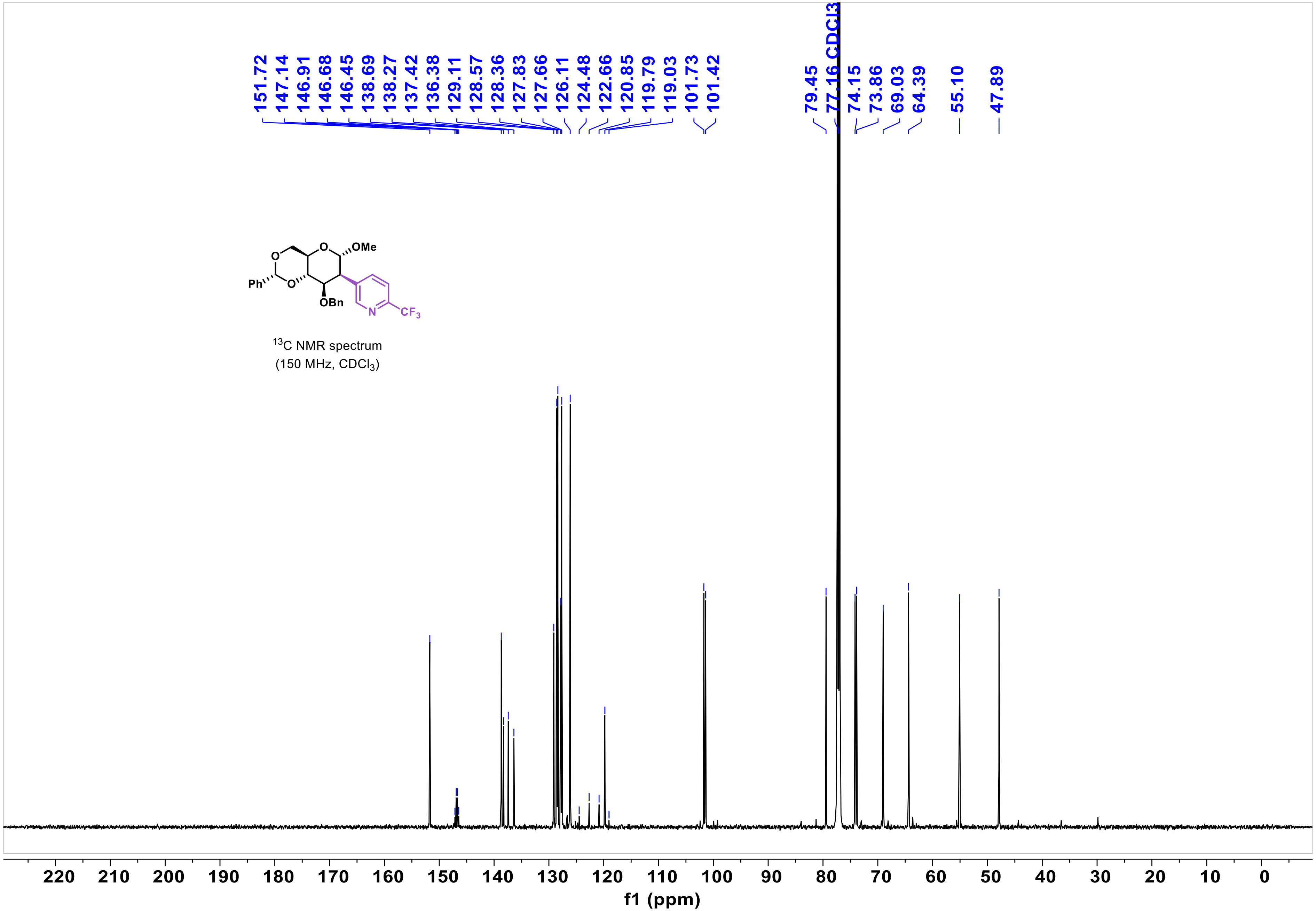

^{13}C NMR of compound **m-101**

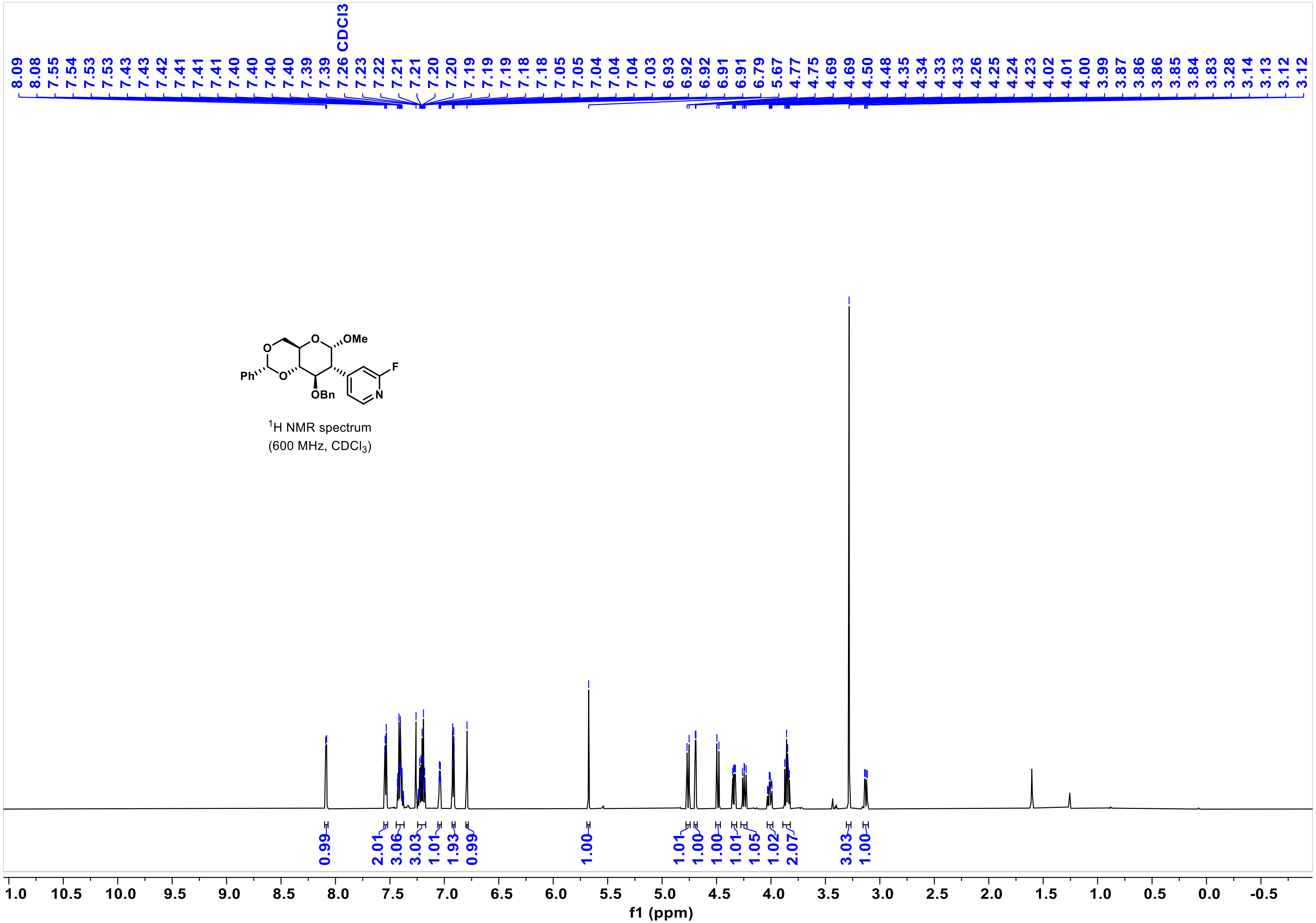

^{1}H NMR of compound **g-102**

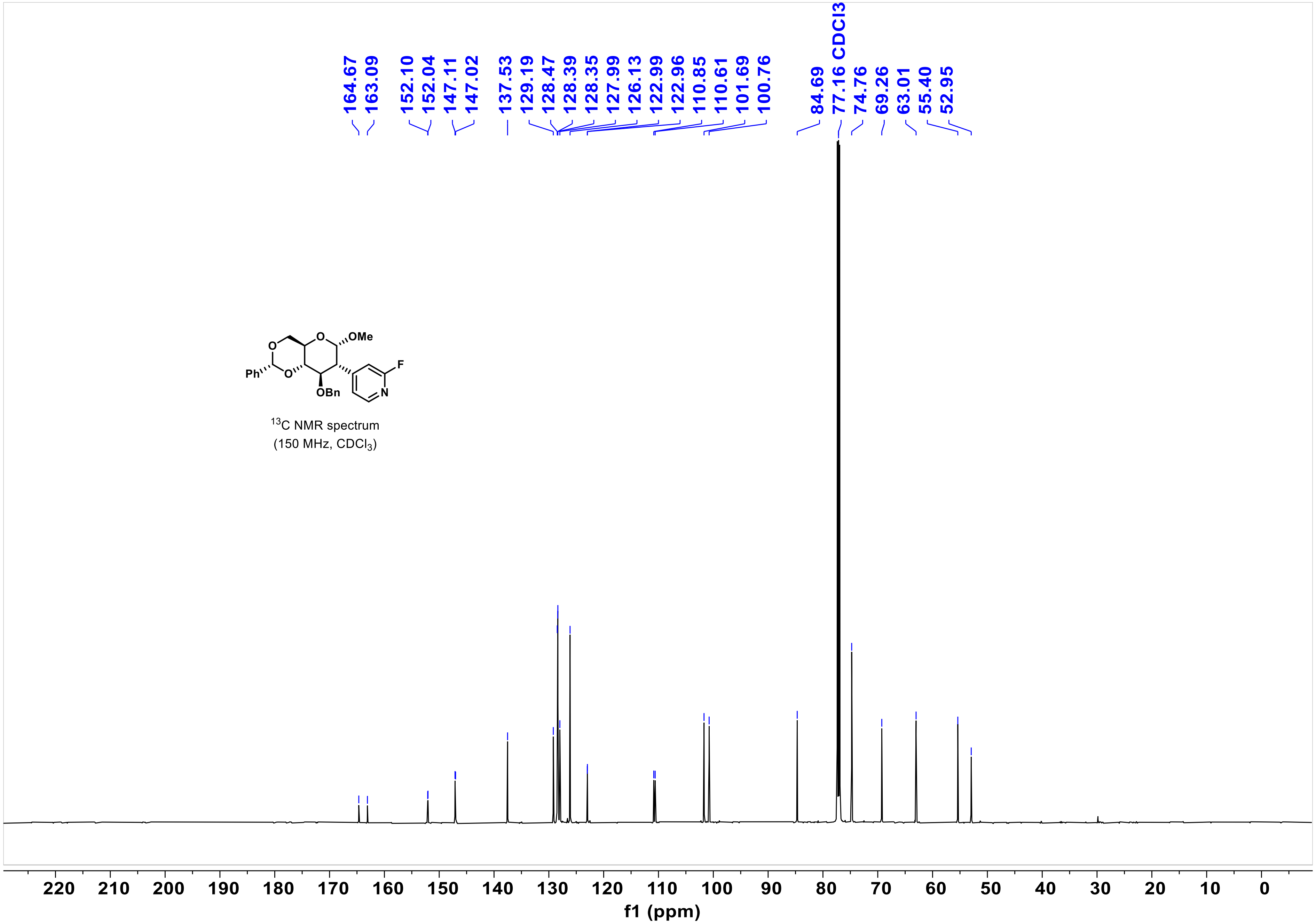

^{13}C NMR of compound **g-102**

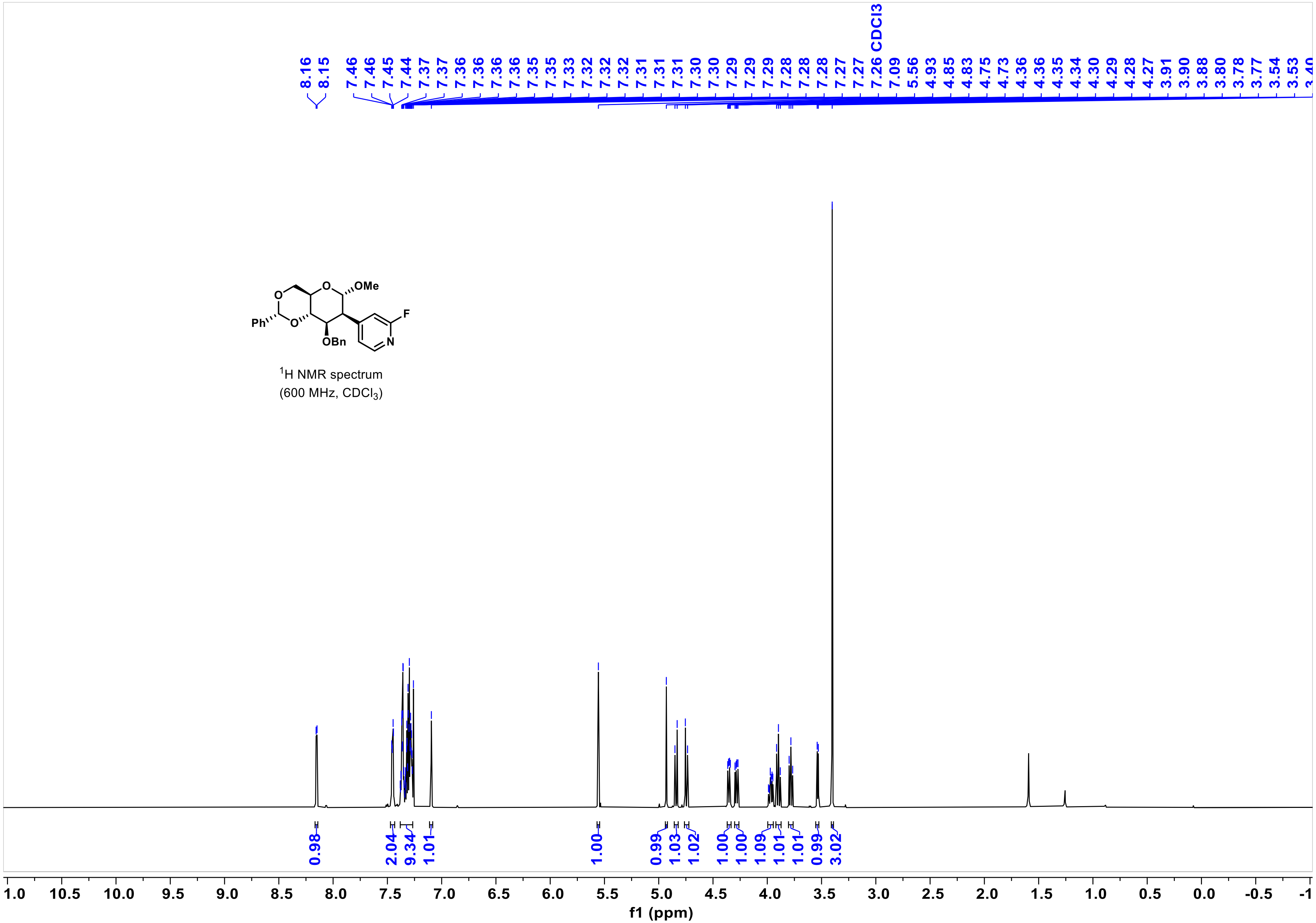

^{1}H NMR of compound **m-102**

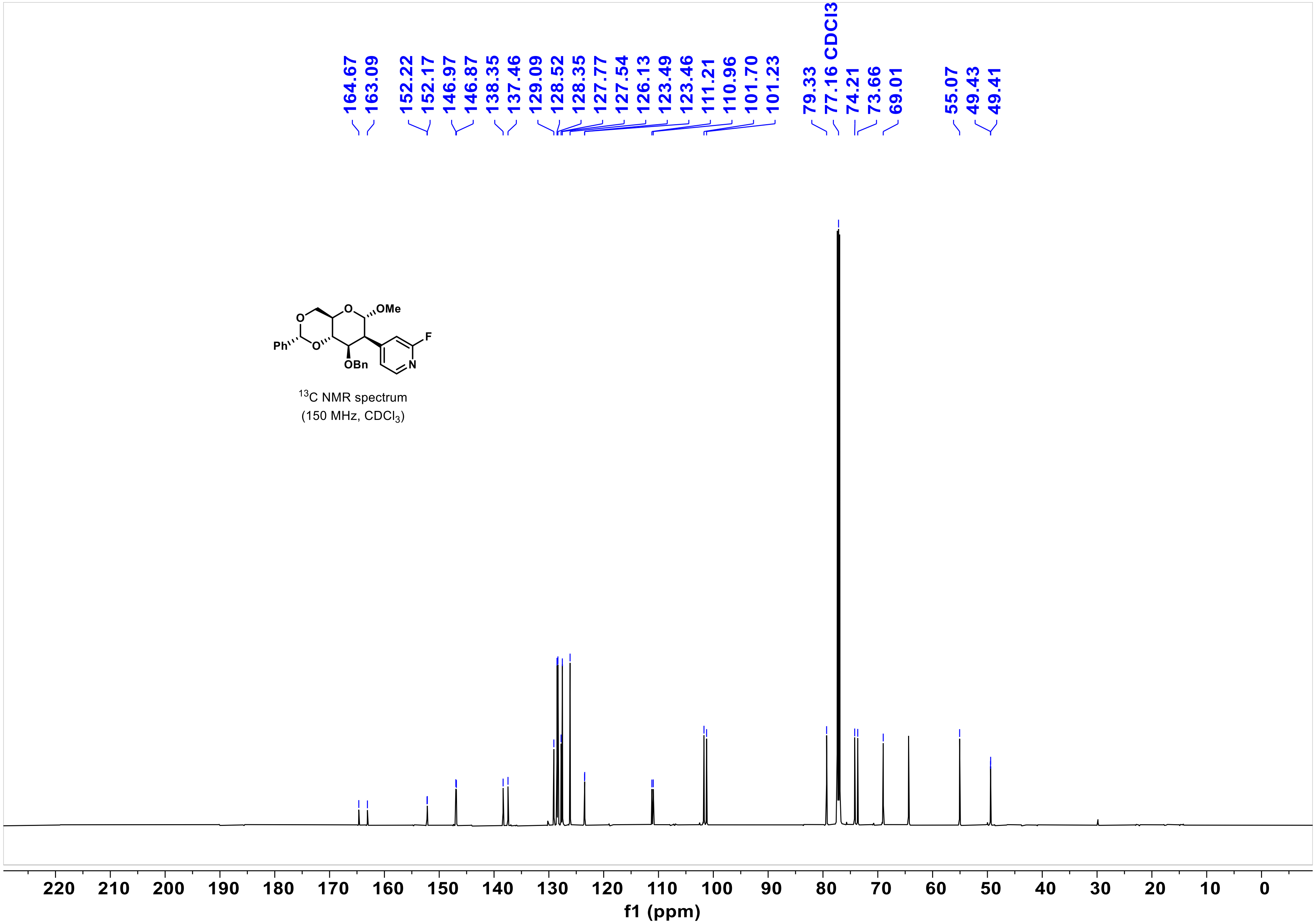

^{13}C NMR of compound **m-102**

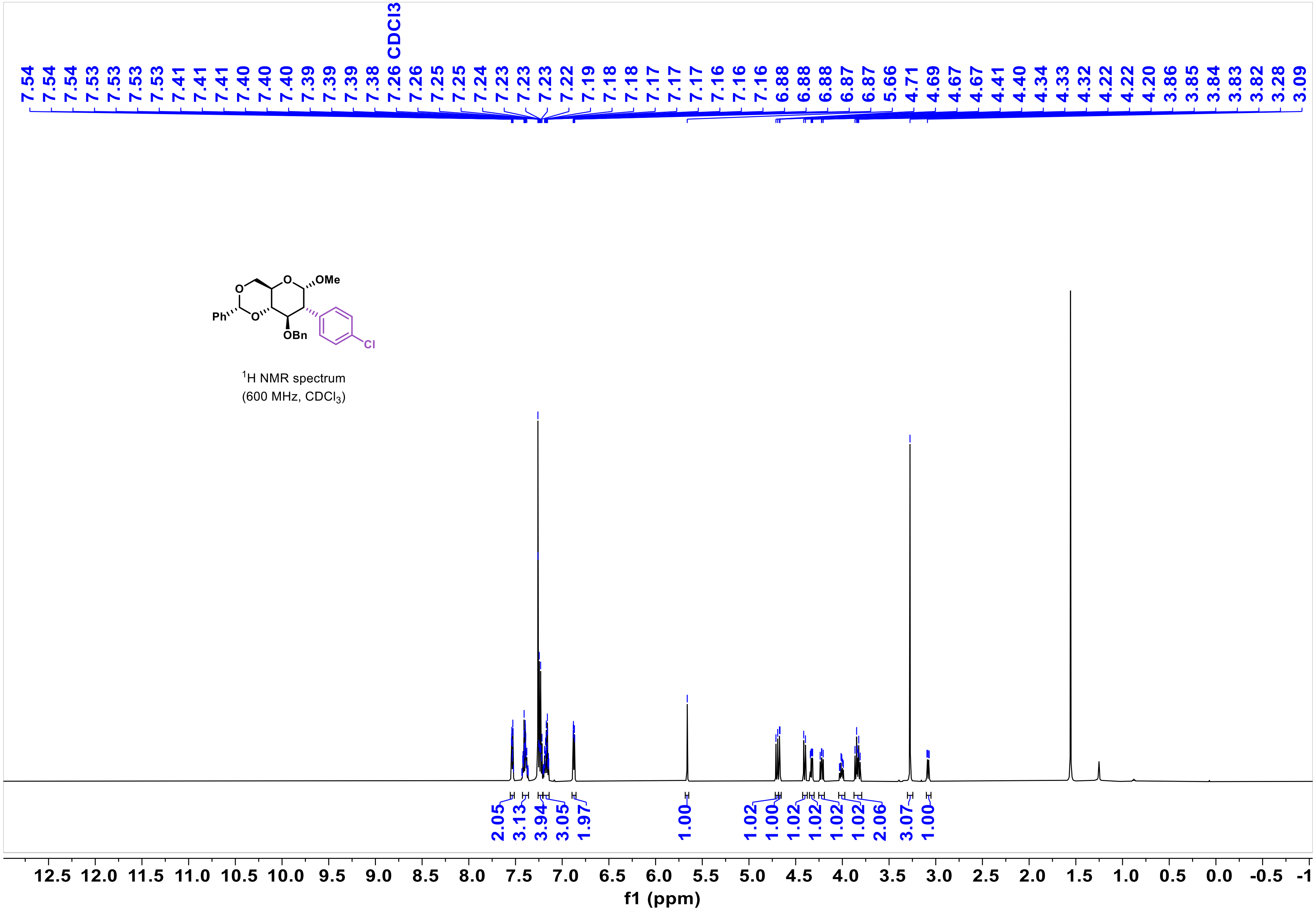

^{1}H NMR of compound **g-103**

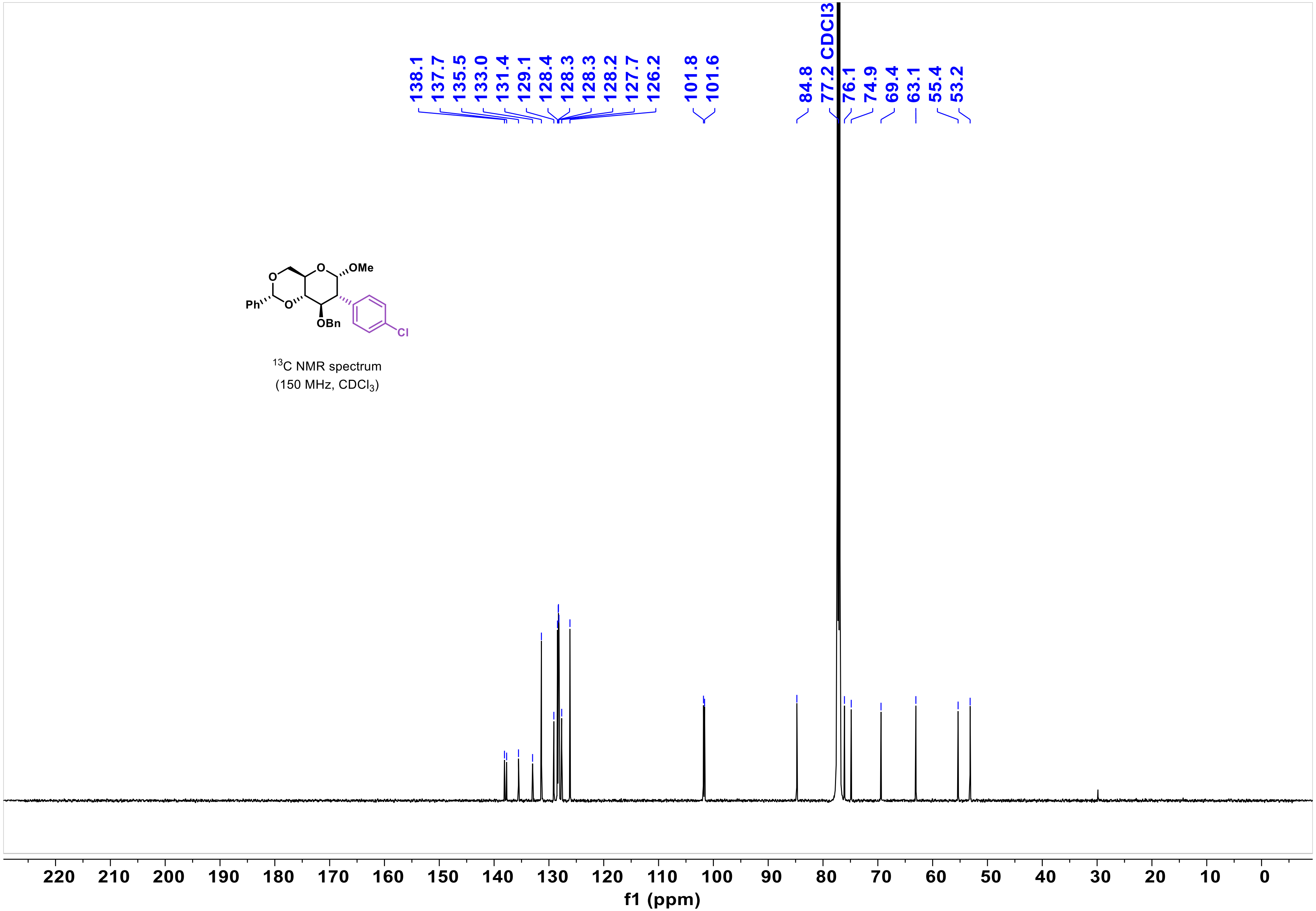

^{13}C NMR of compound **g-103**

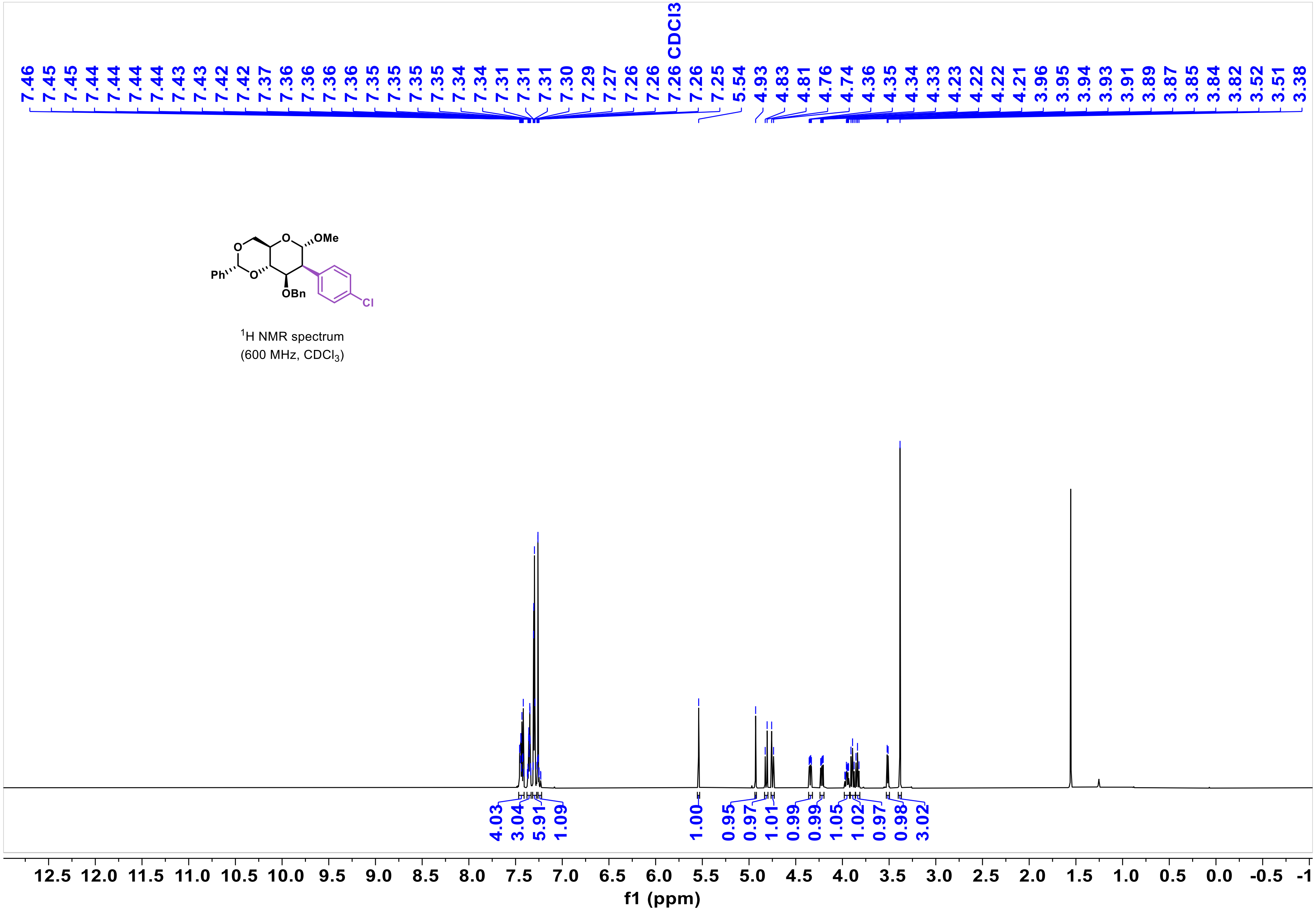

^{1}H NMR of compound **m-103**

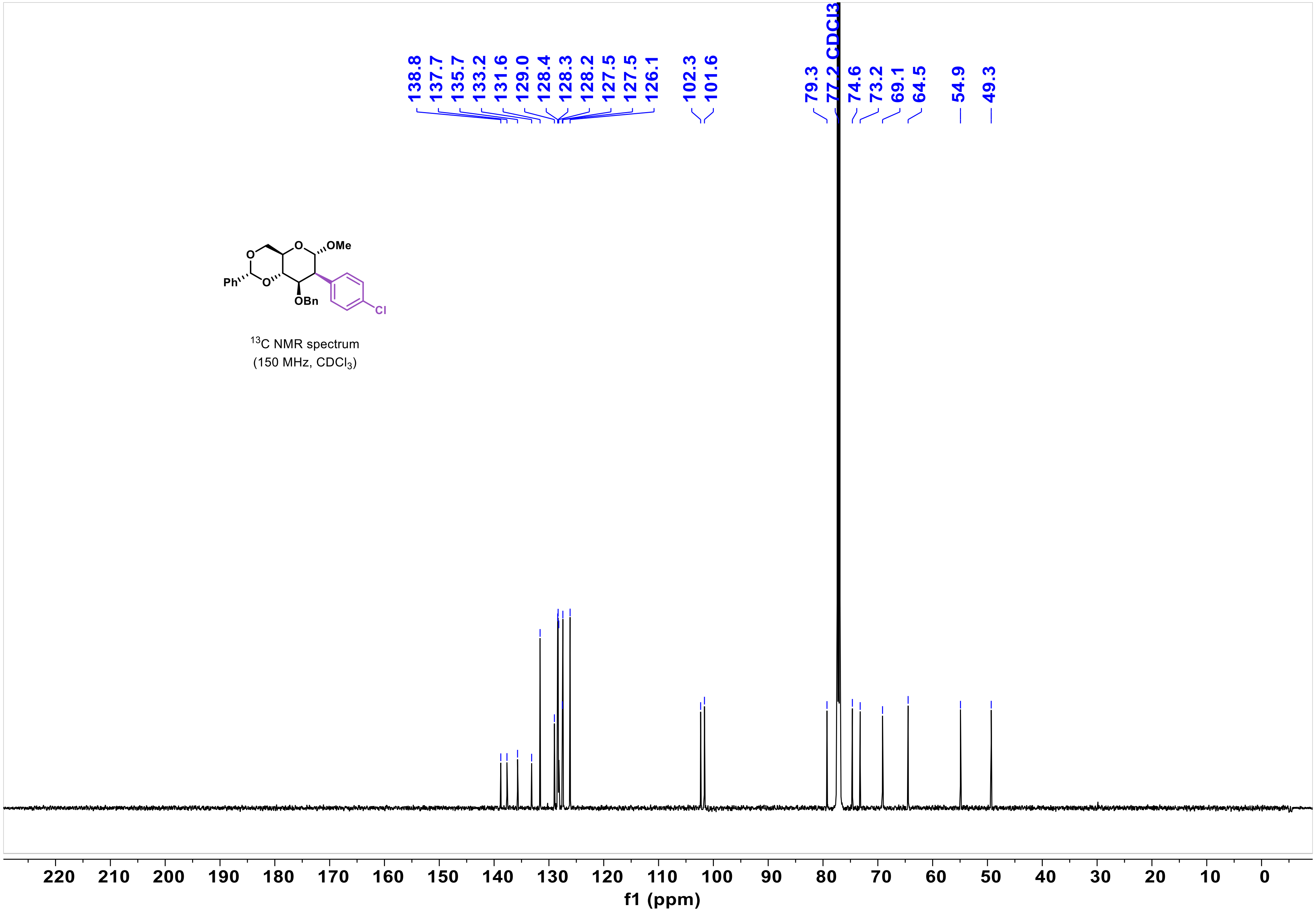

^{13}C NMR of compound **m-103**

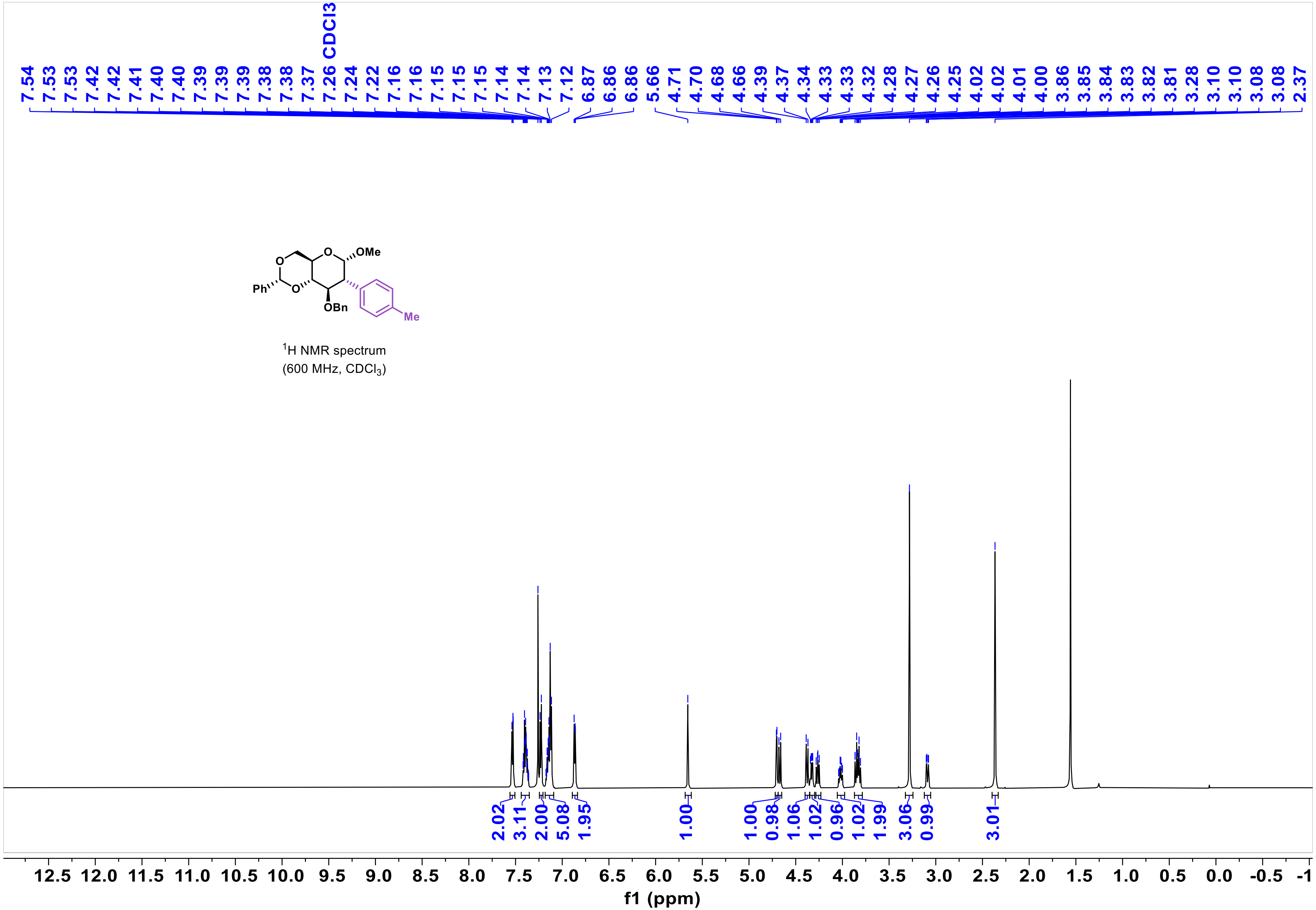

^{1}H NMR of compound **g-104**

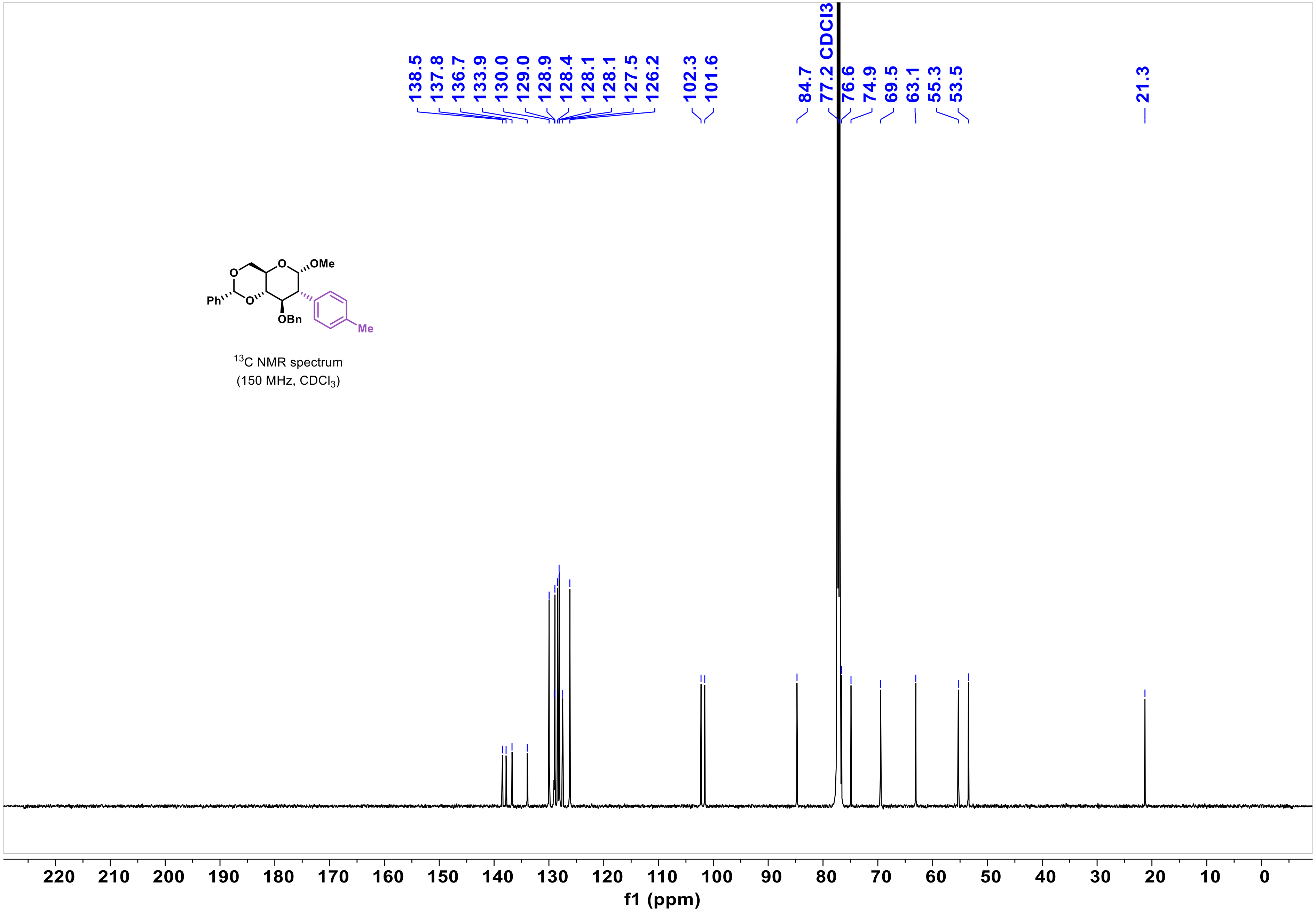

^{13}C NMR of compound **g-104**

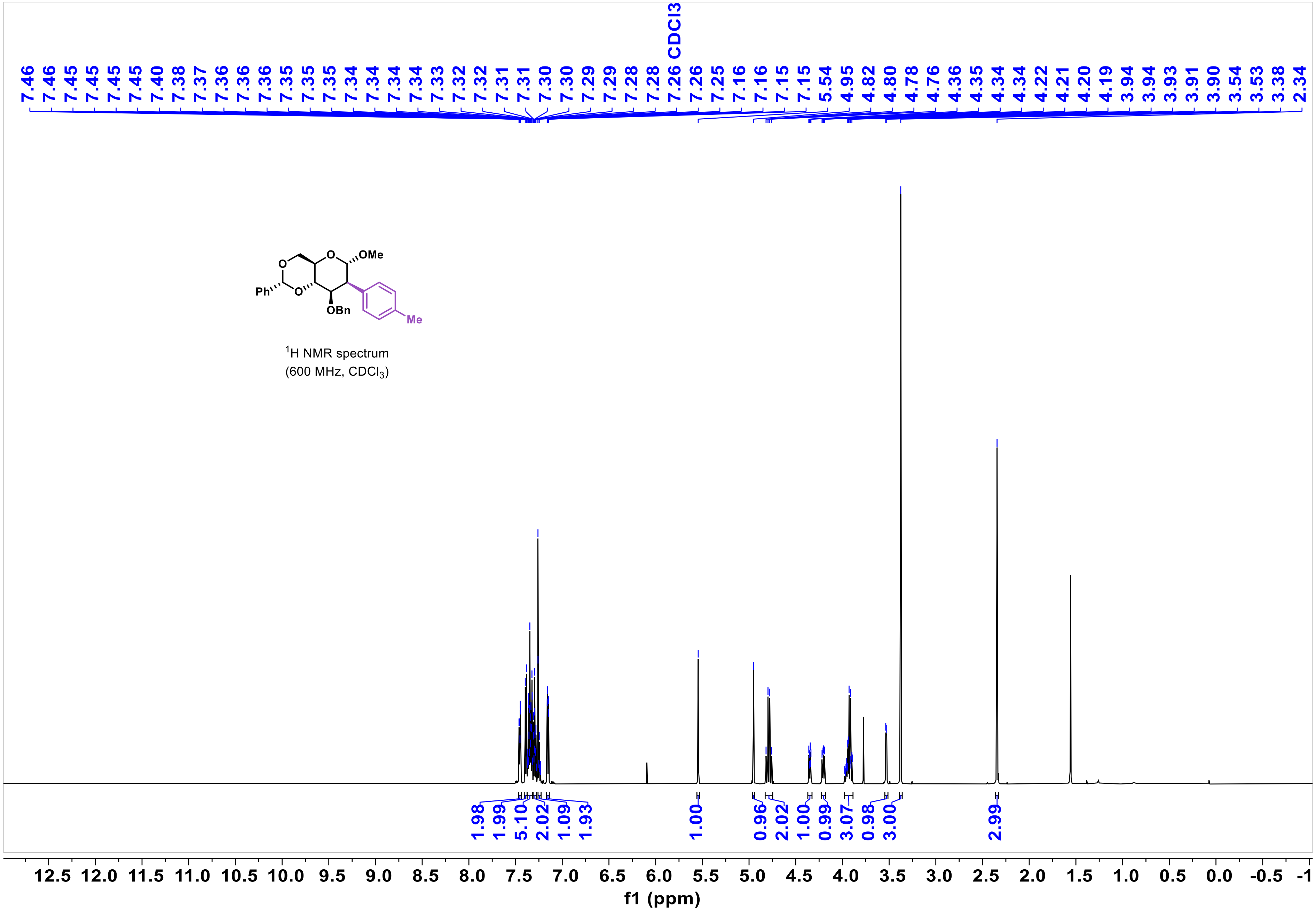

^{1}H NMR of compound **m-104**

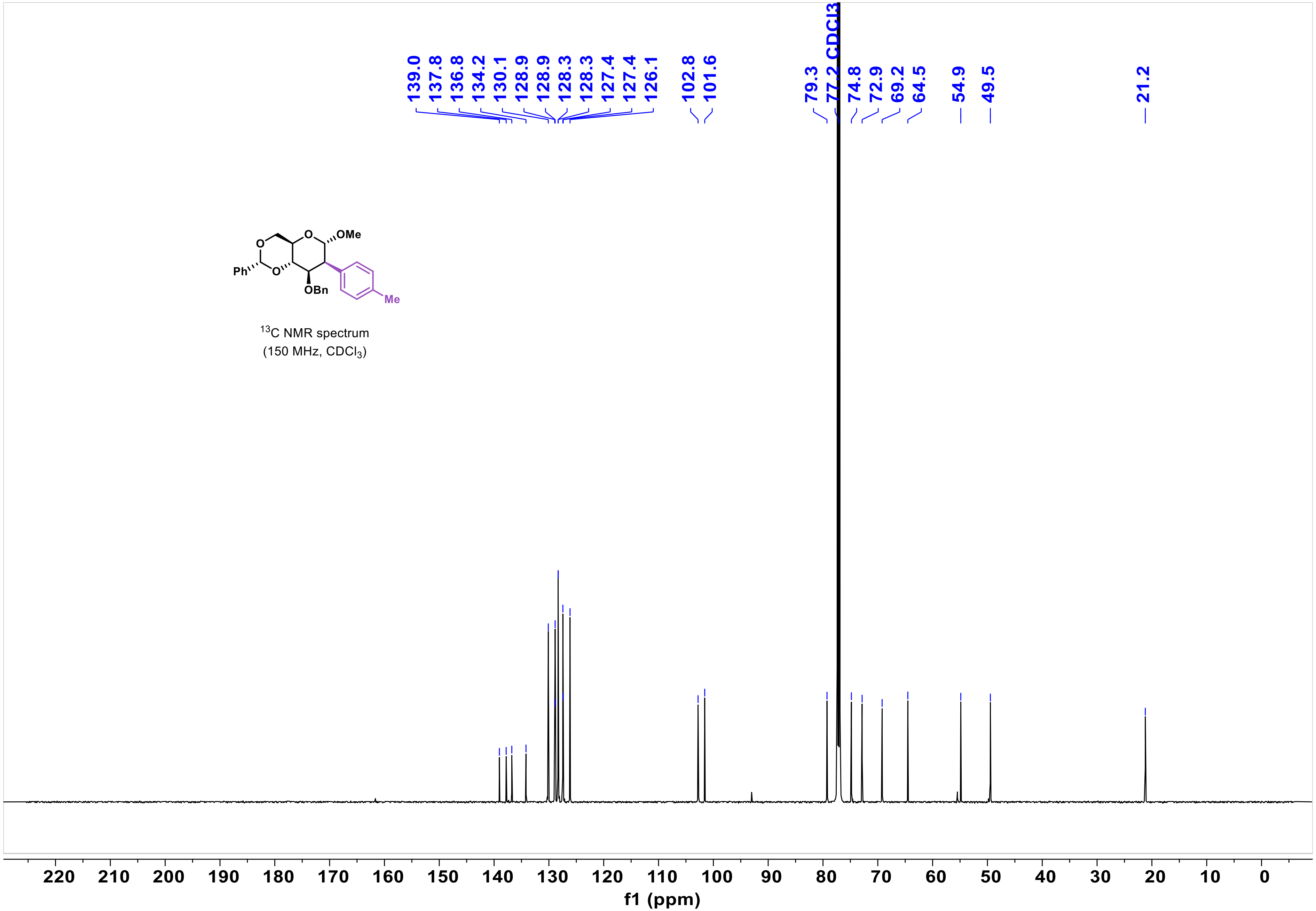

^{13}C NMR of compound **m-104**

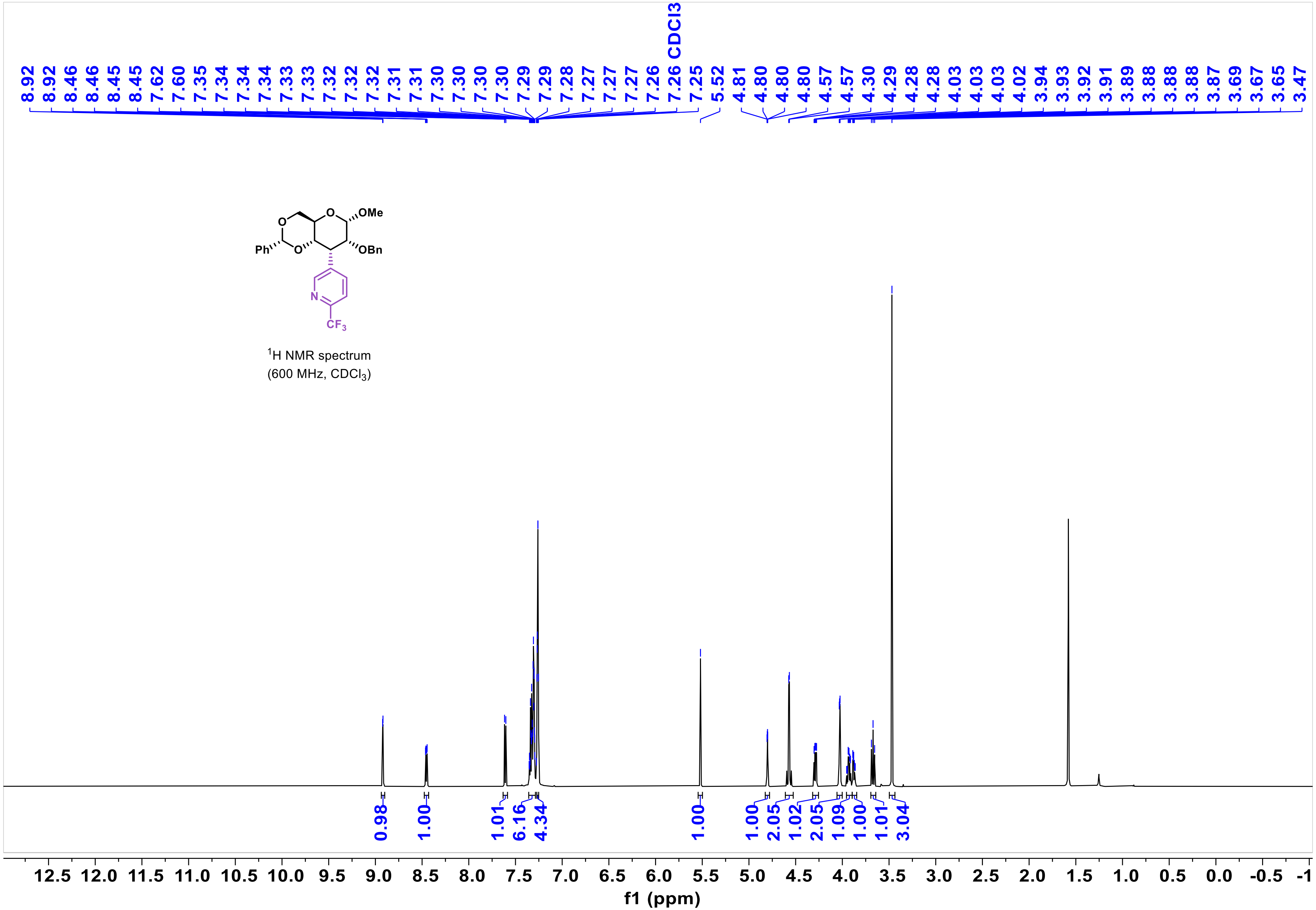

^{1}H NMR of compound **a-107**

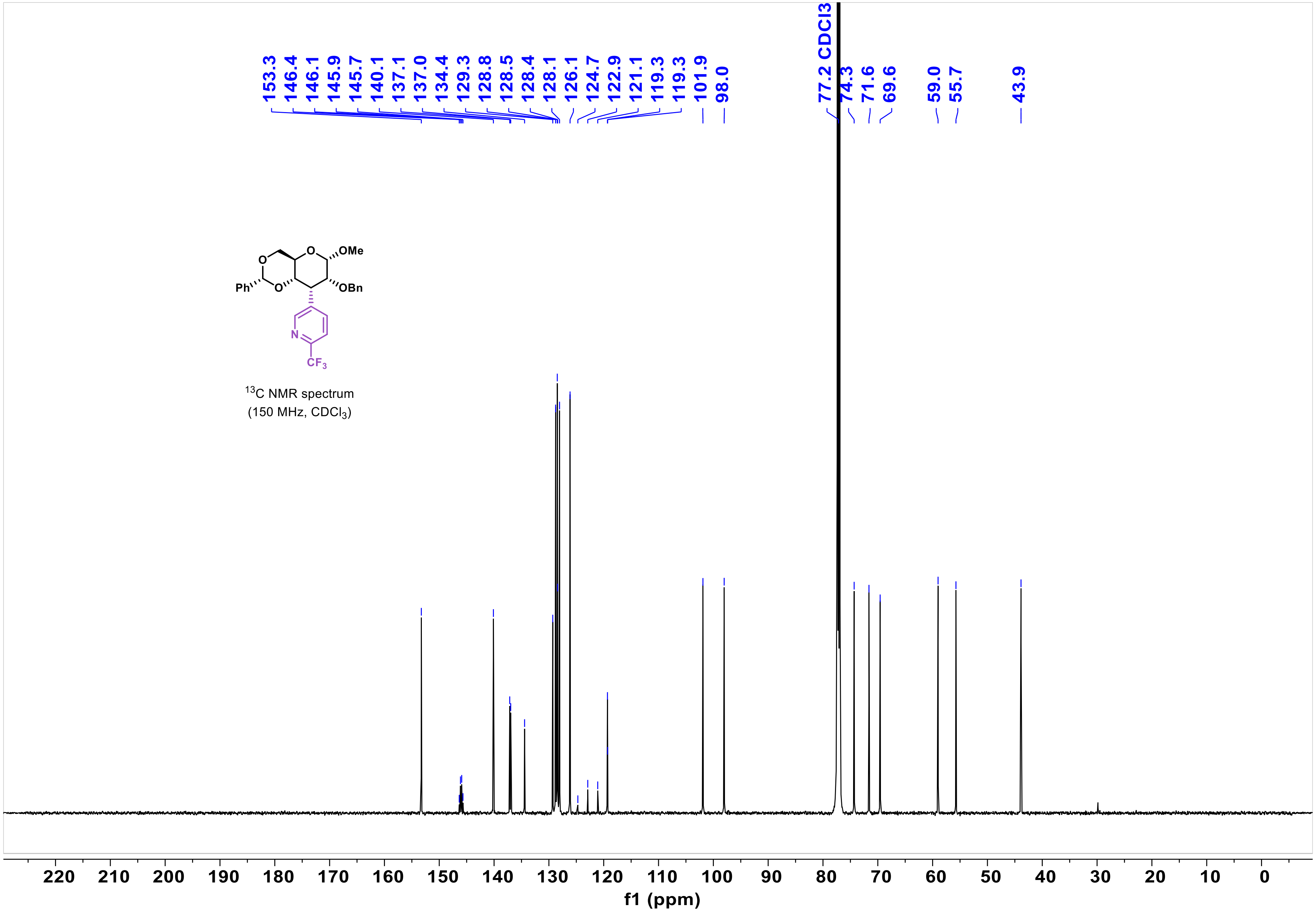

^{13}C NMR of compound **a-107**

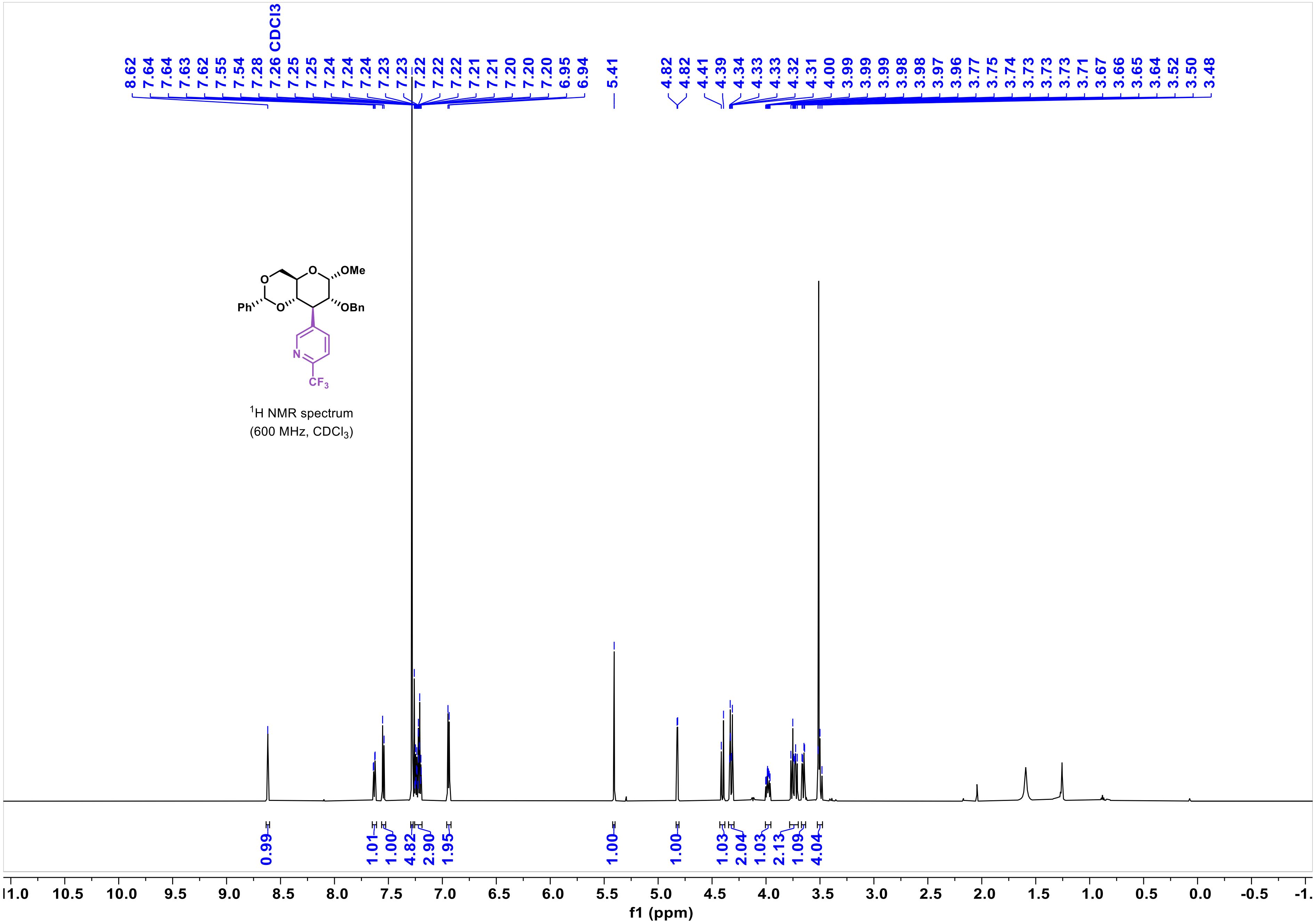

^{1}H NMR of compound **g-107**

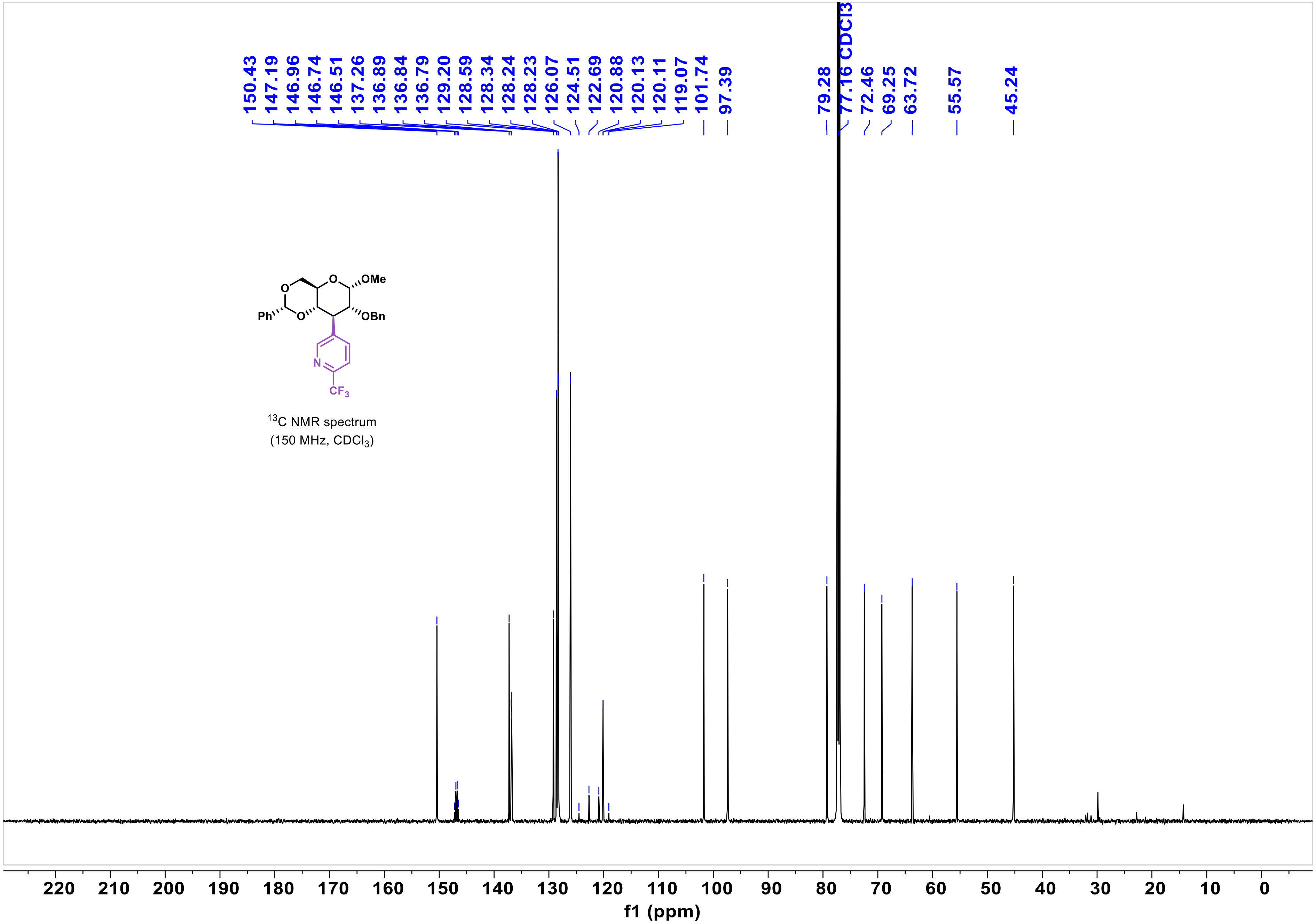

^{13}C NMR of compound **g-107**

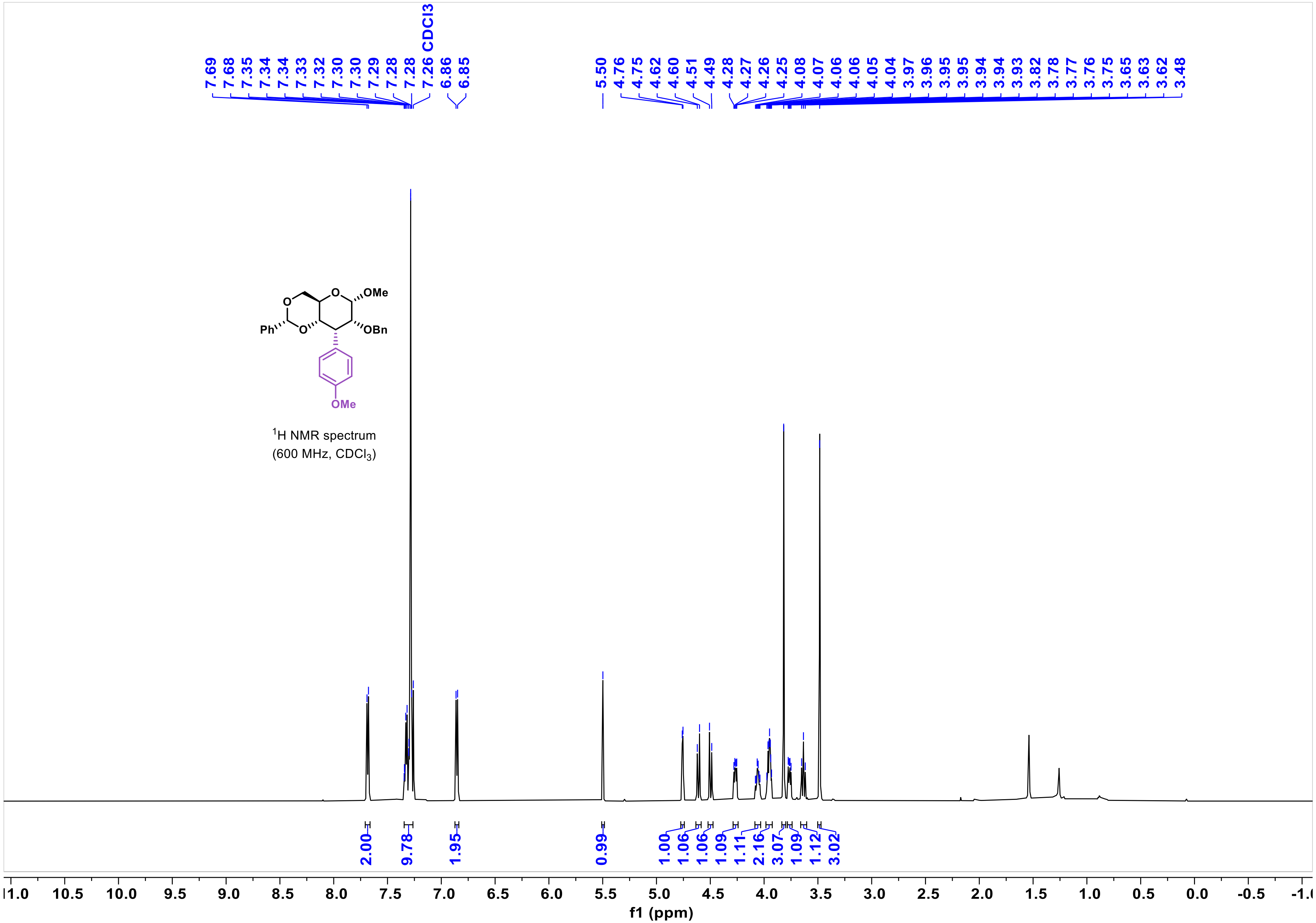

^{1}H NMR of compound **a-108**

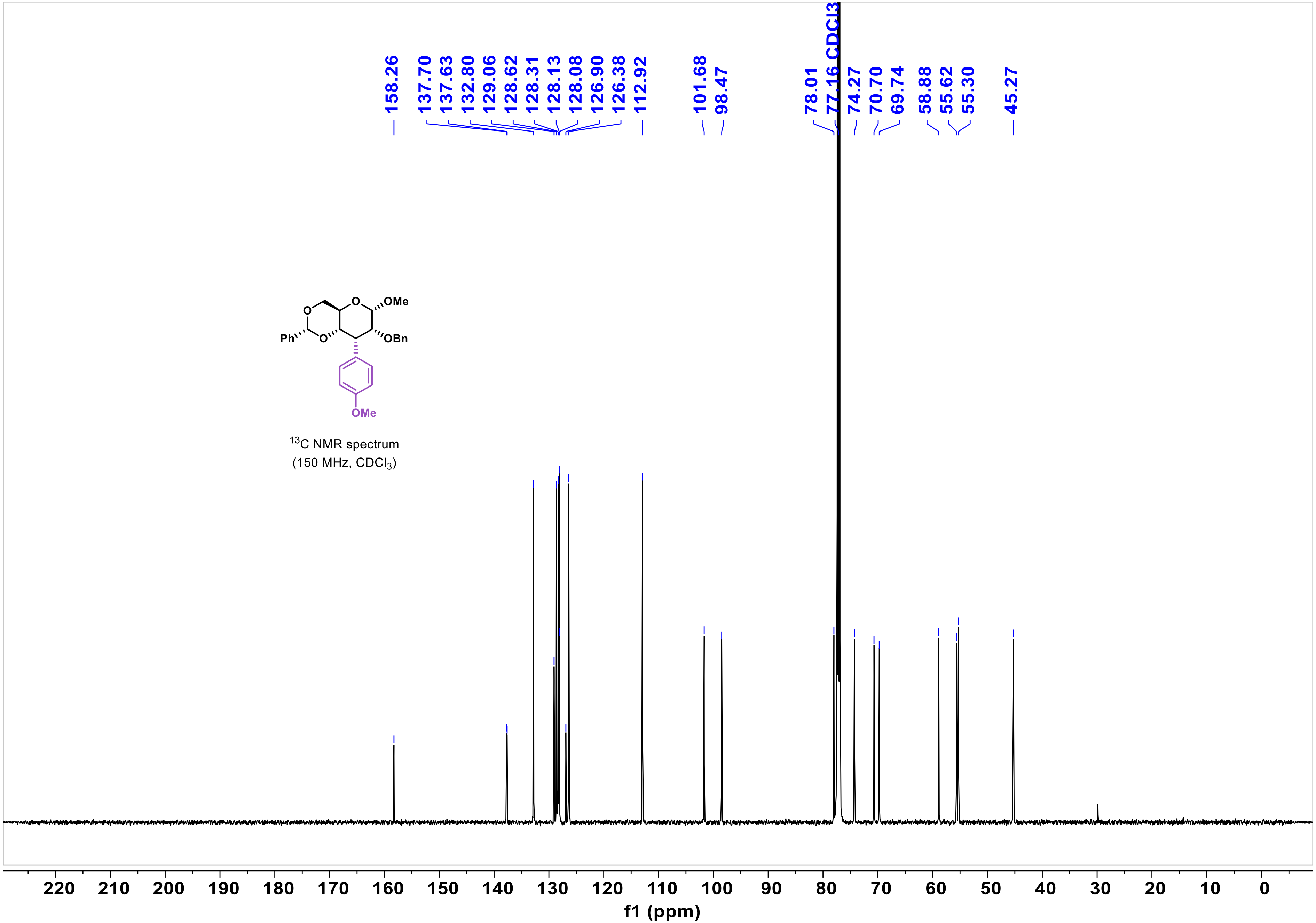

^{13}C NMR of compound **a-108**

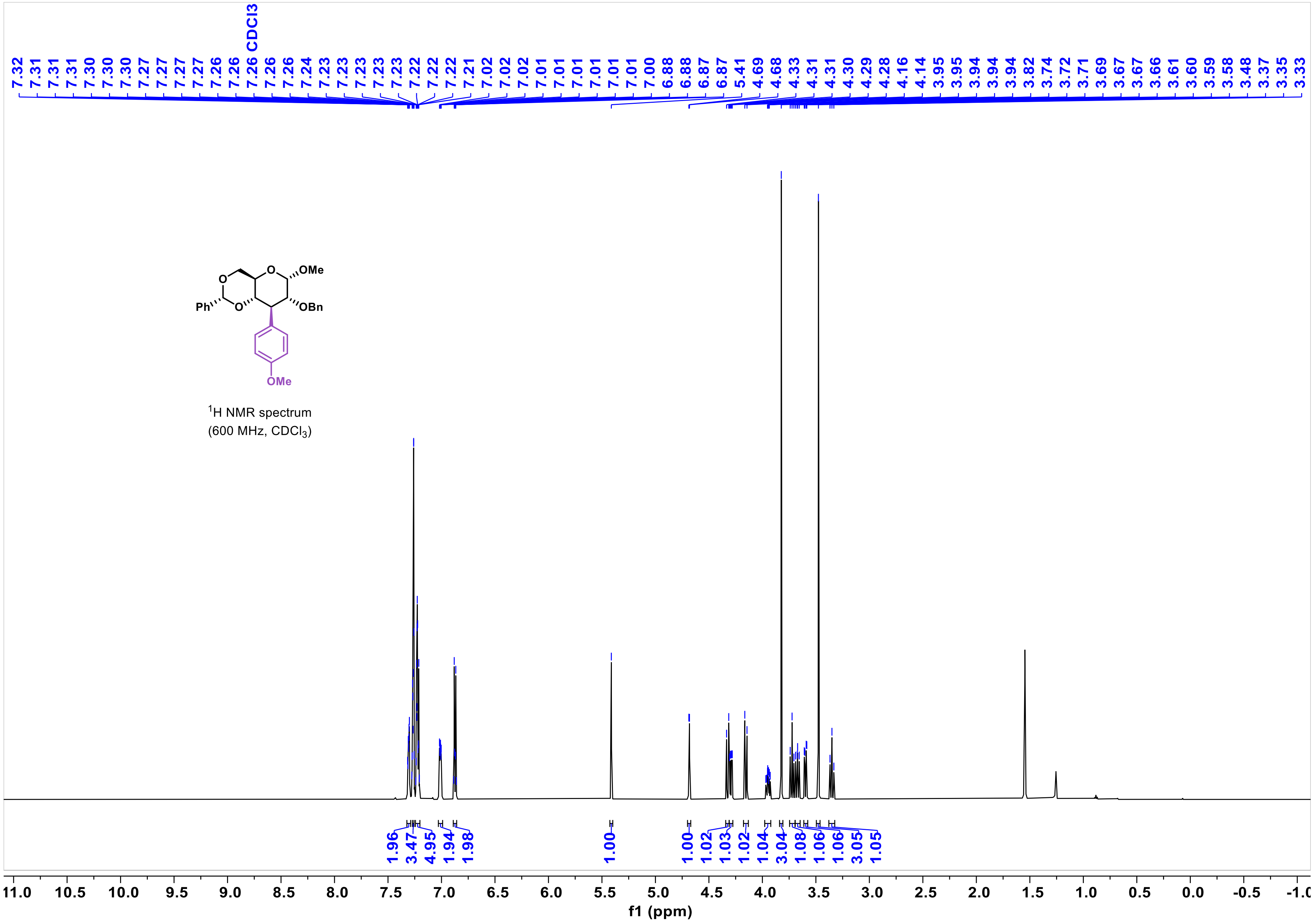

^{1}H NMR of compound **g-108**

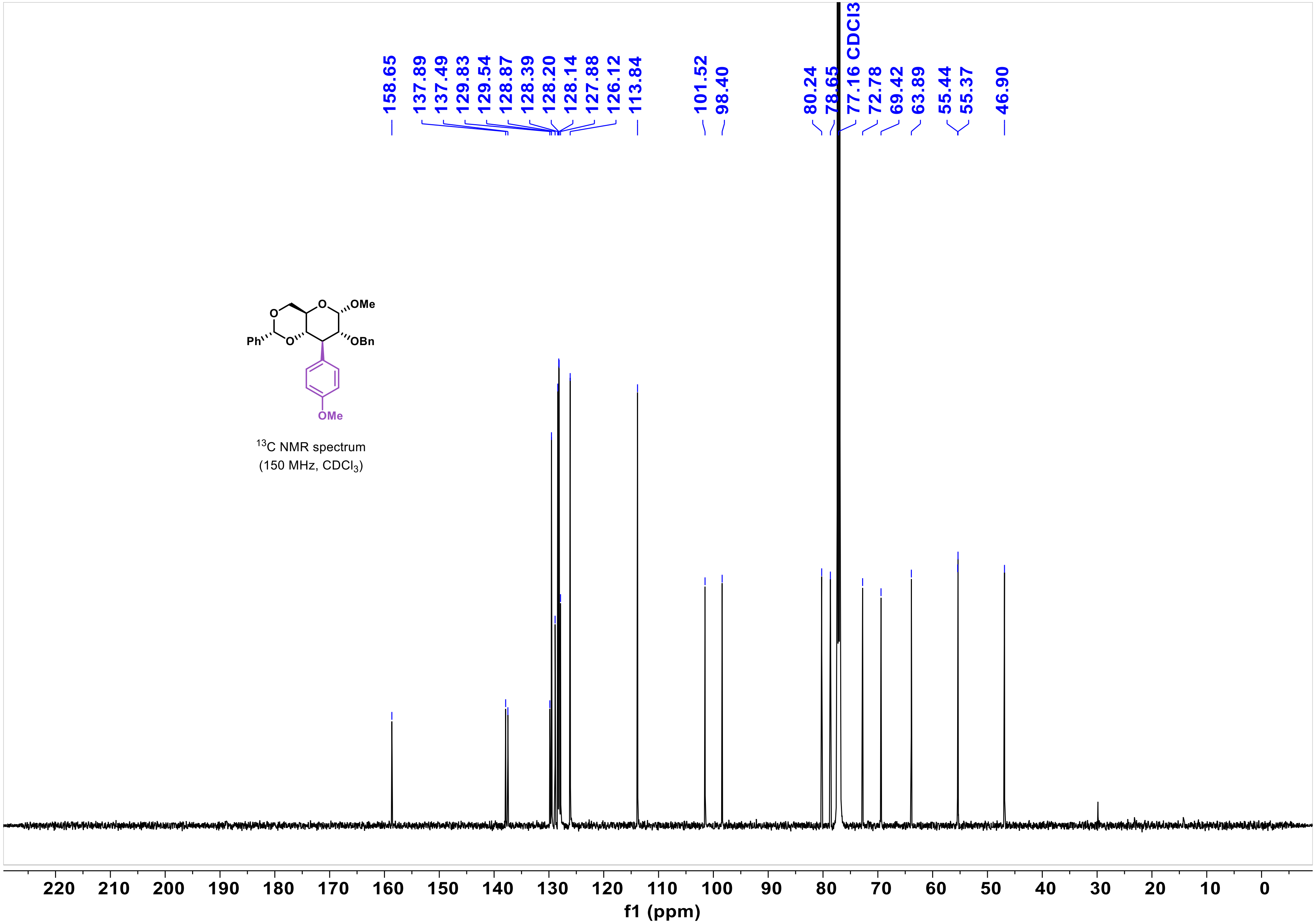

^{13}C NMR of compound **g-108**

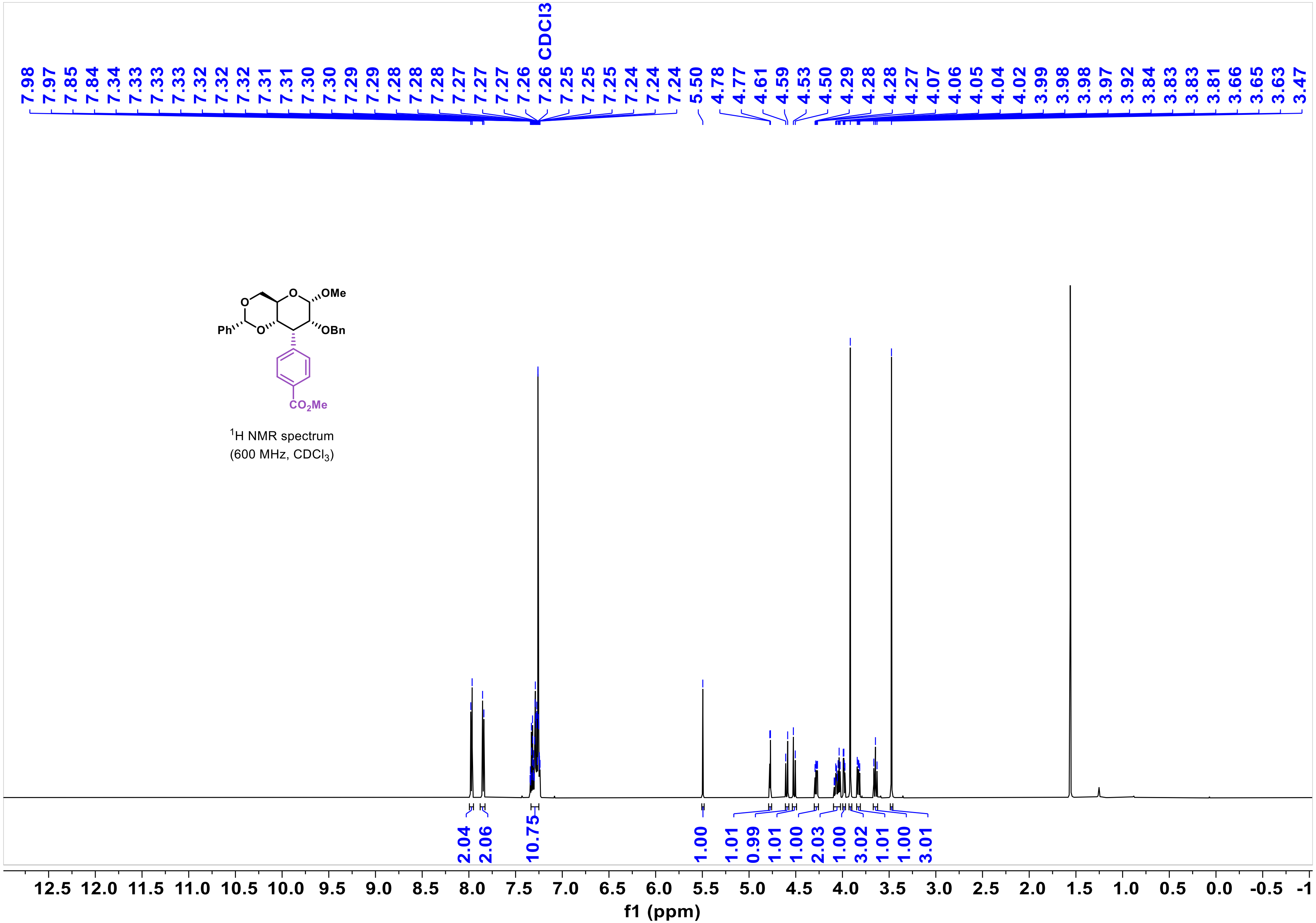

^{1}H NMR of compound **a-109**

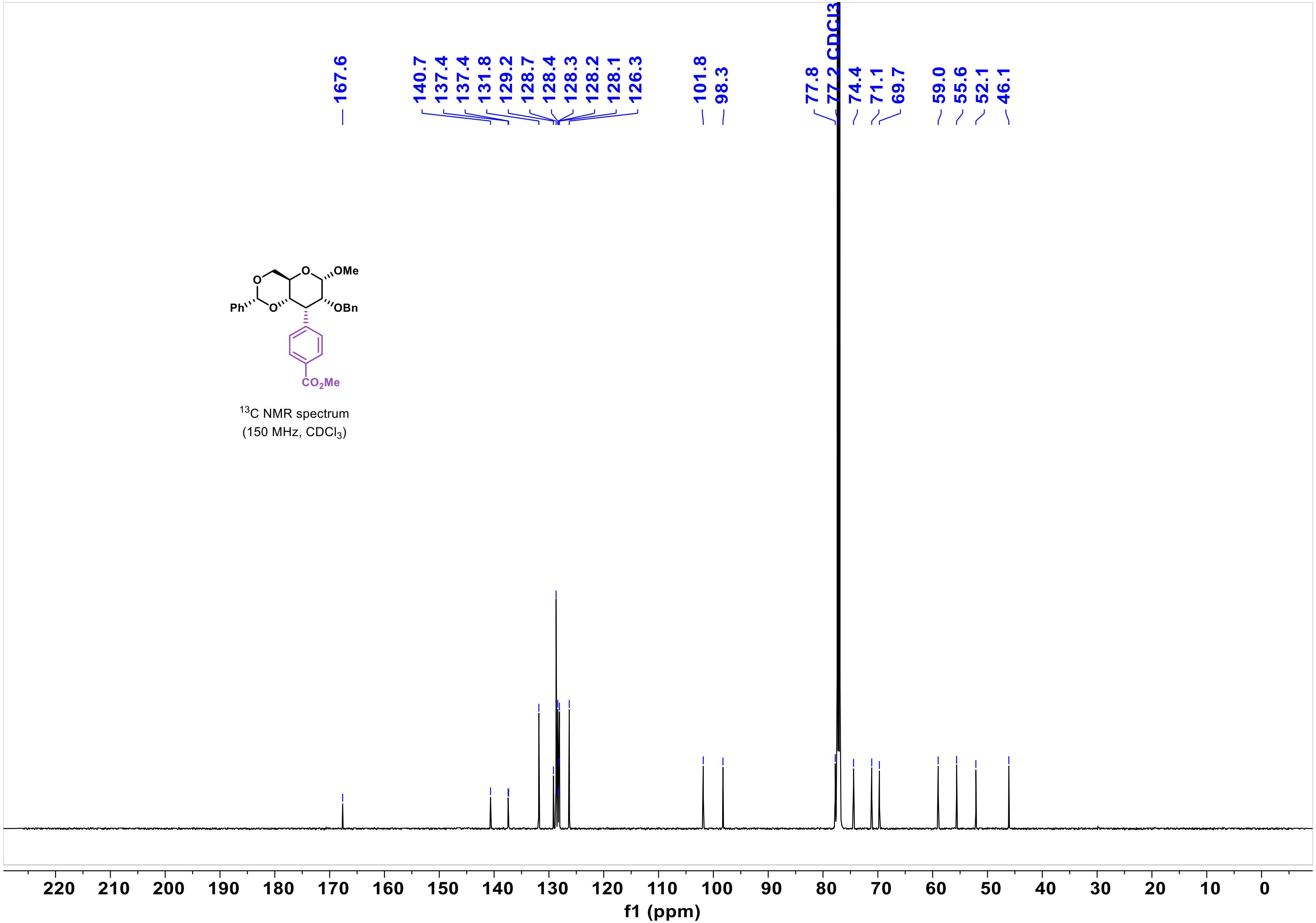

^{13}C NMR of compound **a-109**

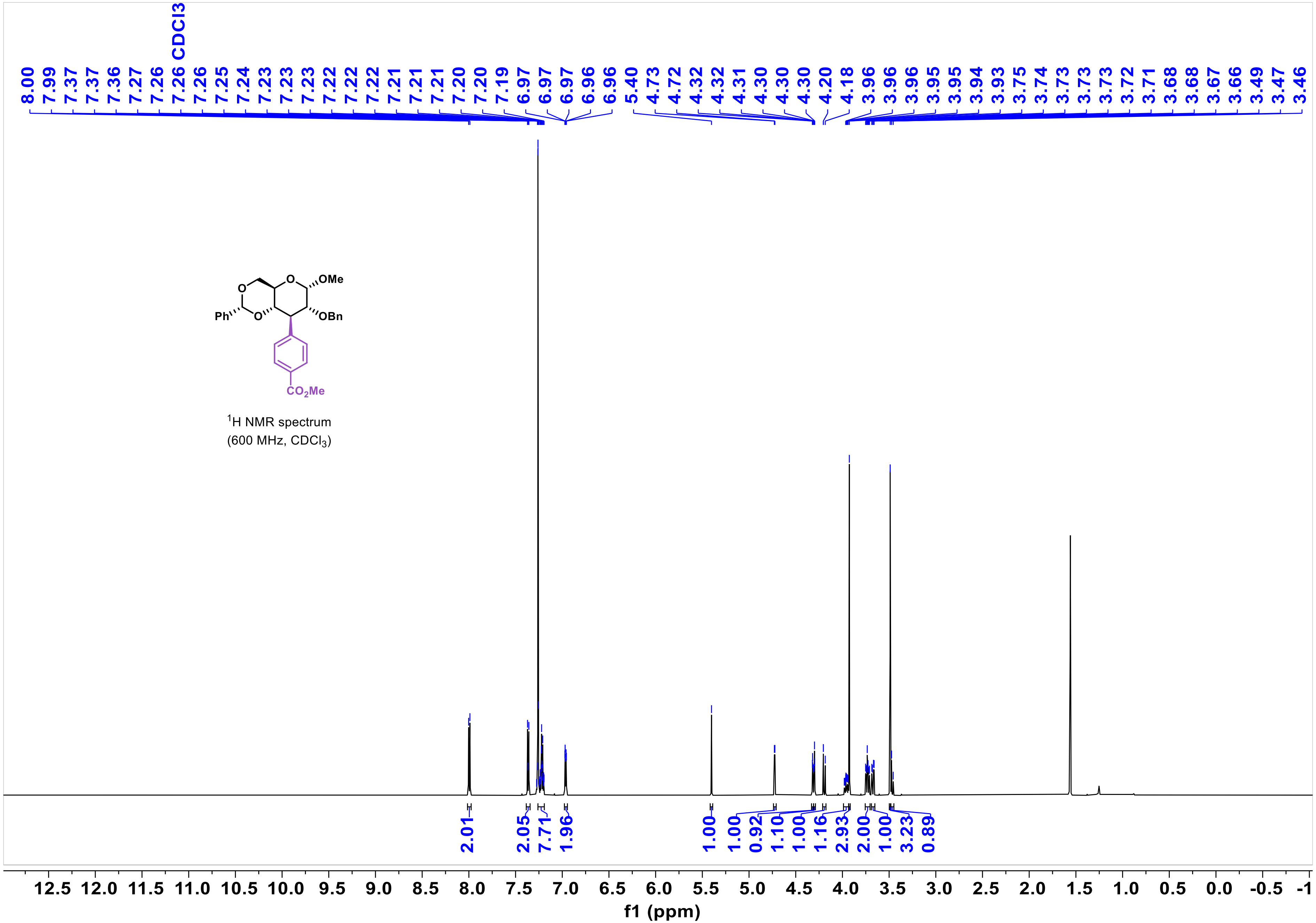

^{1}H NMR of compound **g-109**

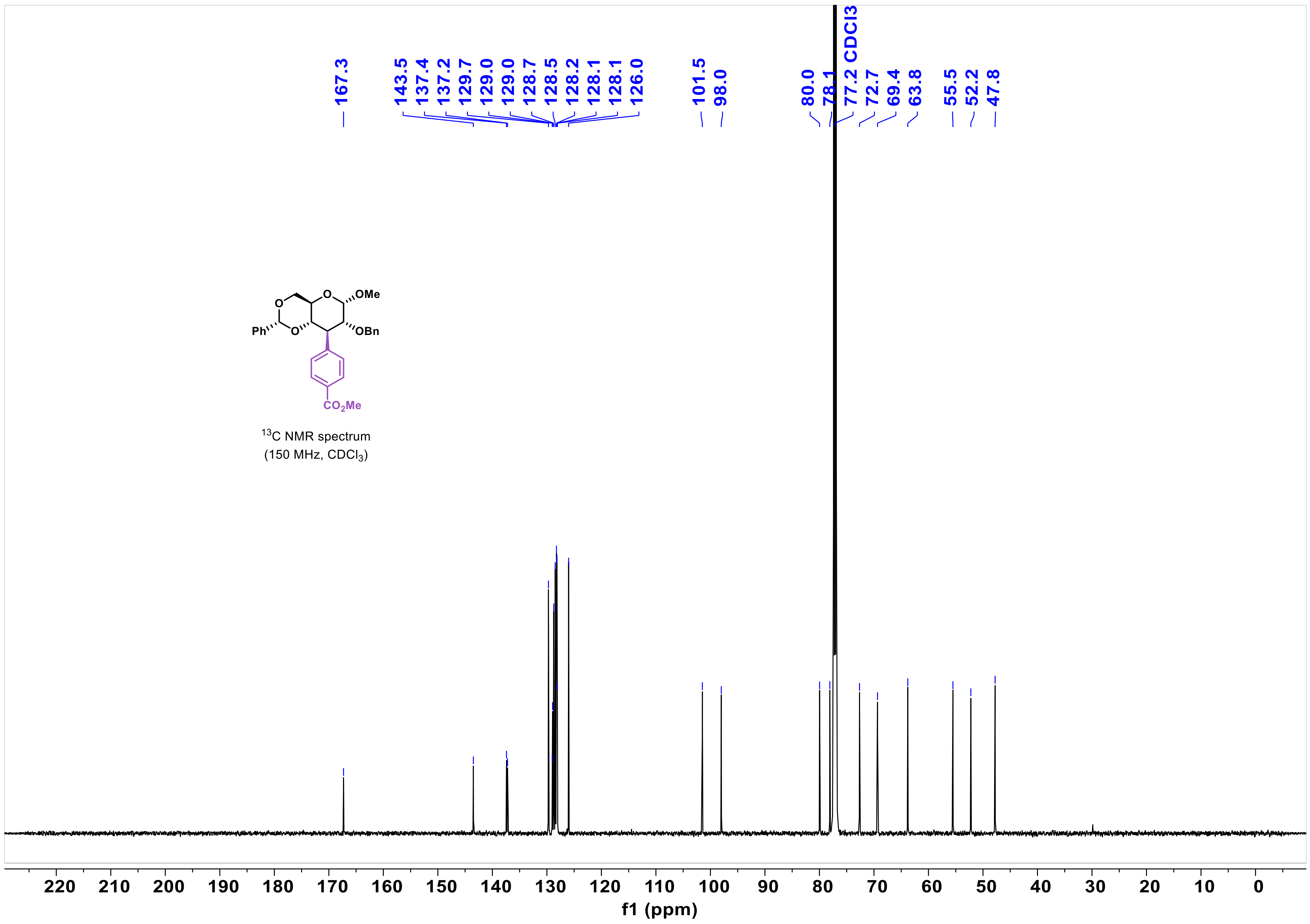

^{13}C NMR of compound **g-109**

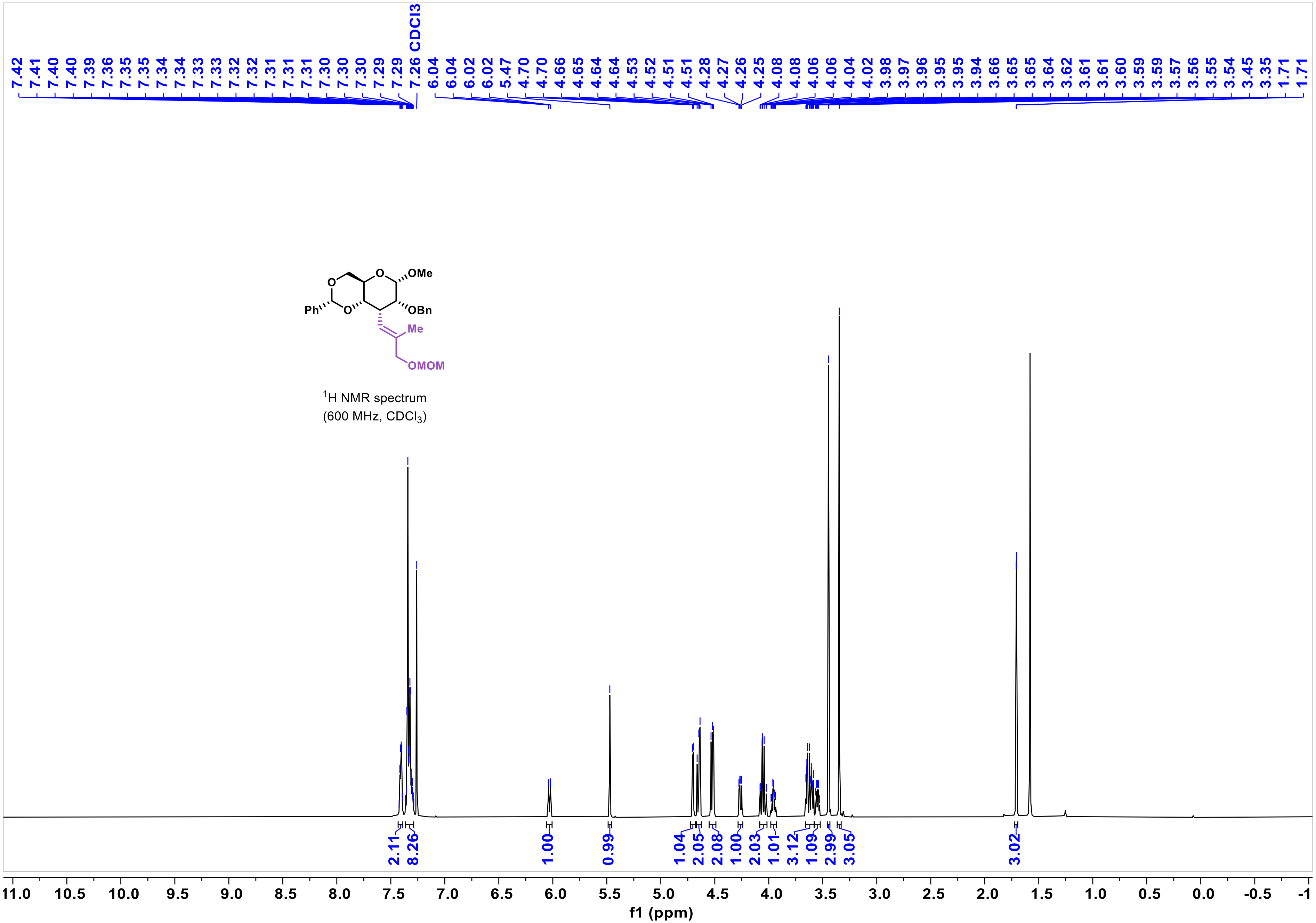

^{1}H NMR of compound **a-110**

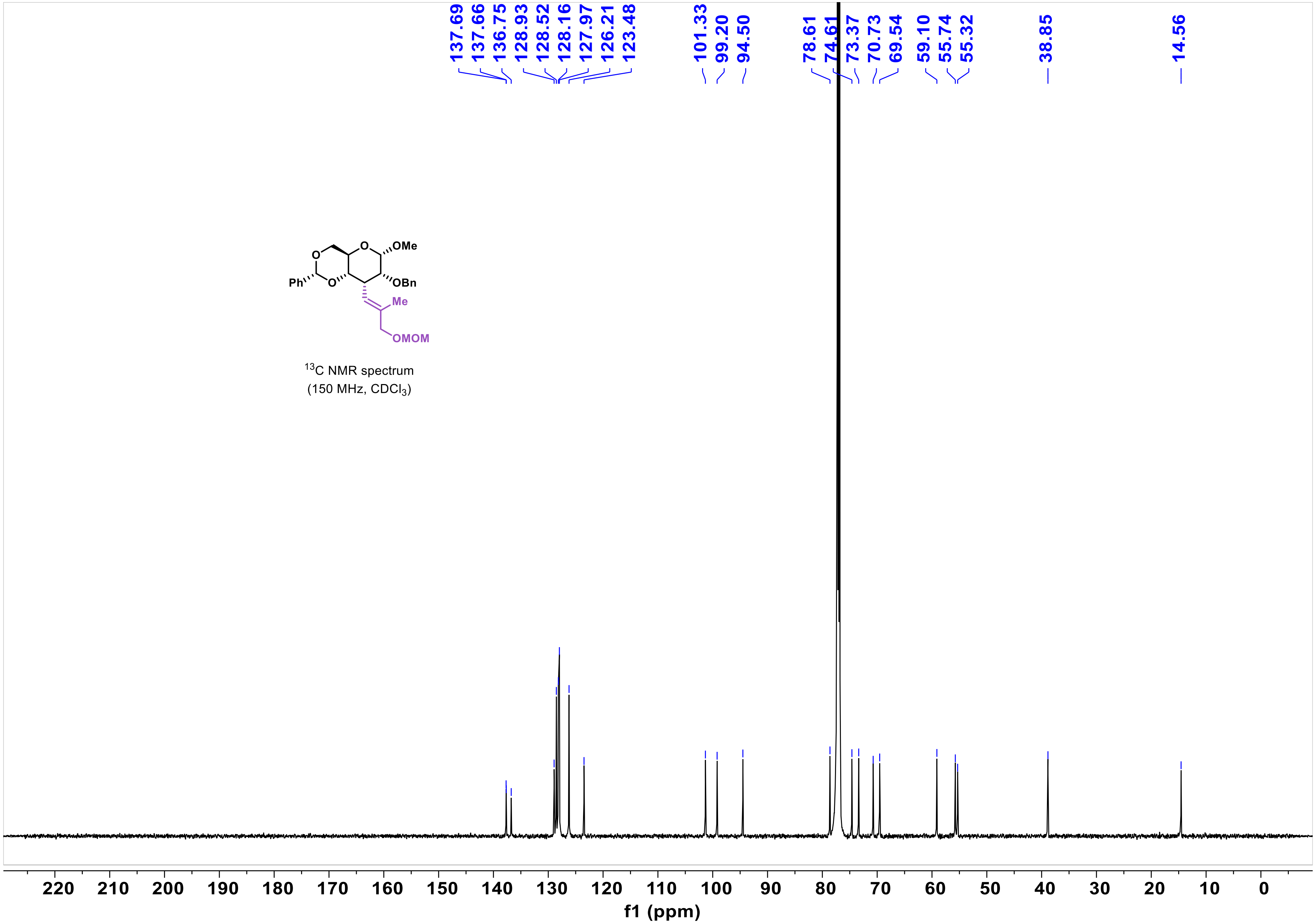

^{13}C NMR of compound **a-110**

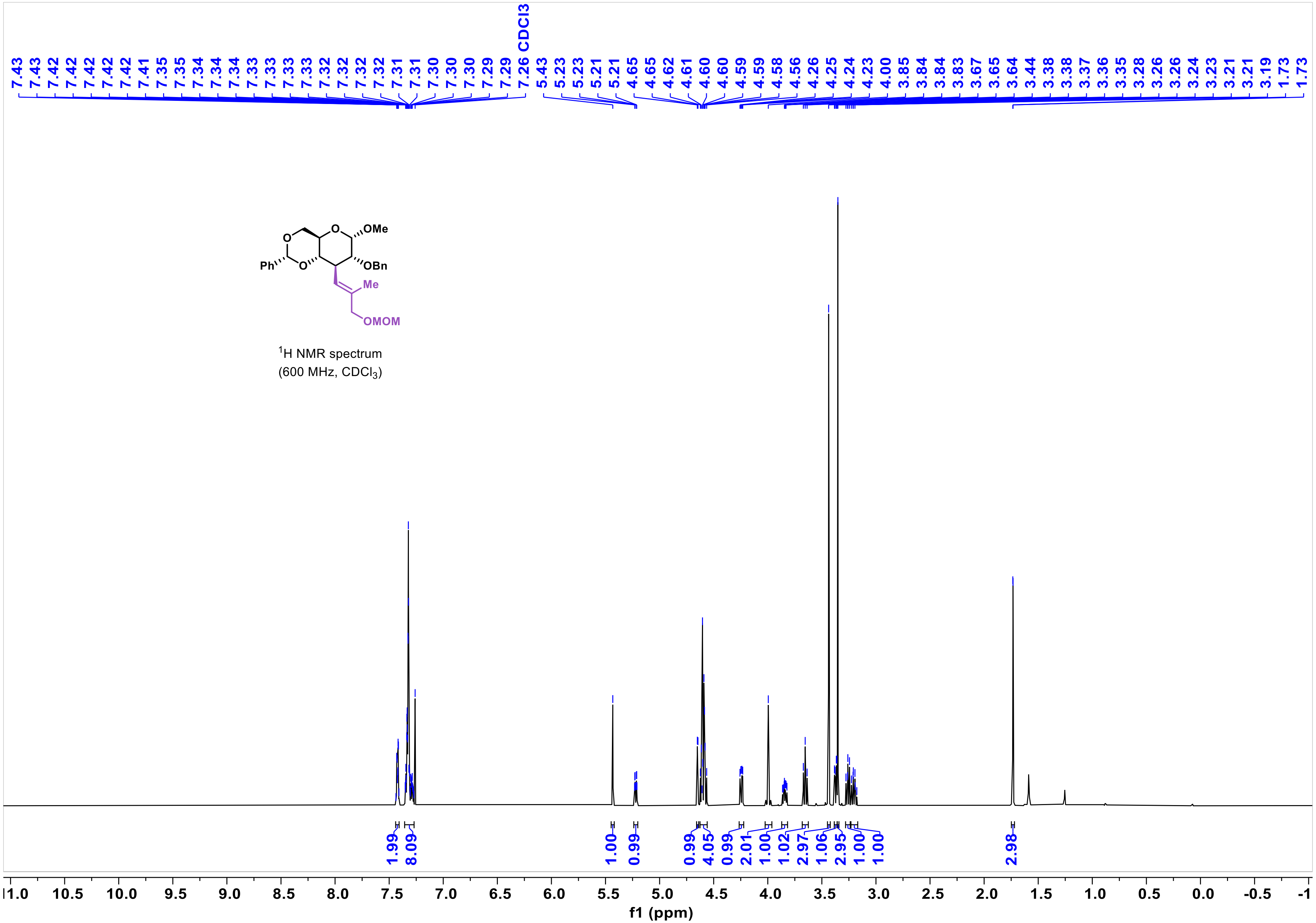

^{1}H NMR of compound **g-110**

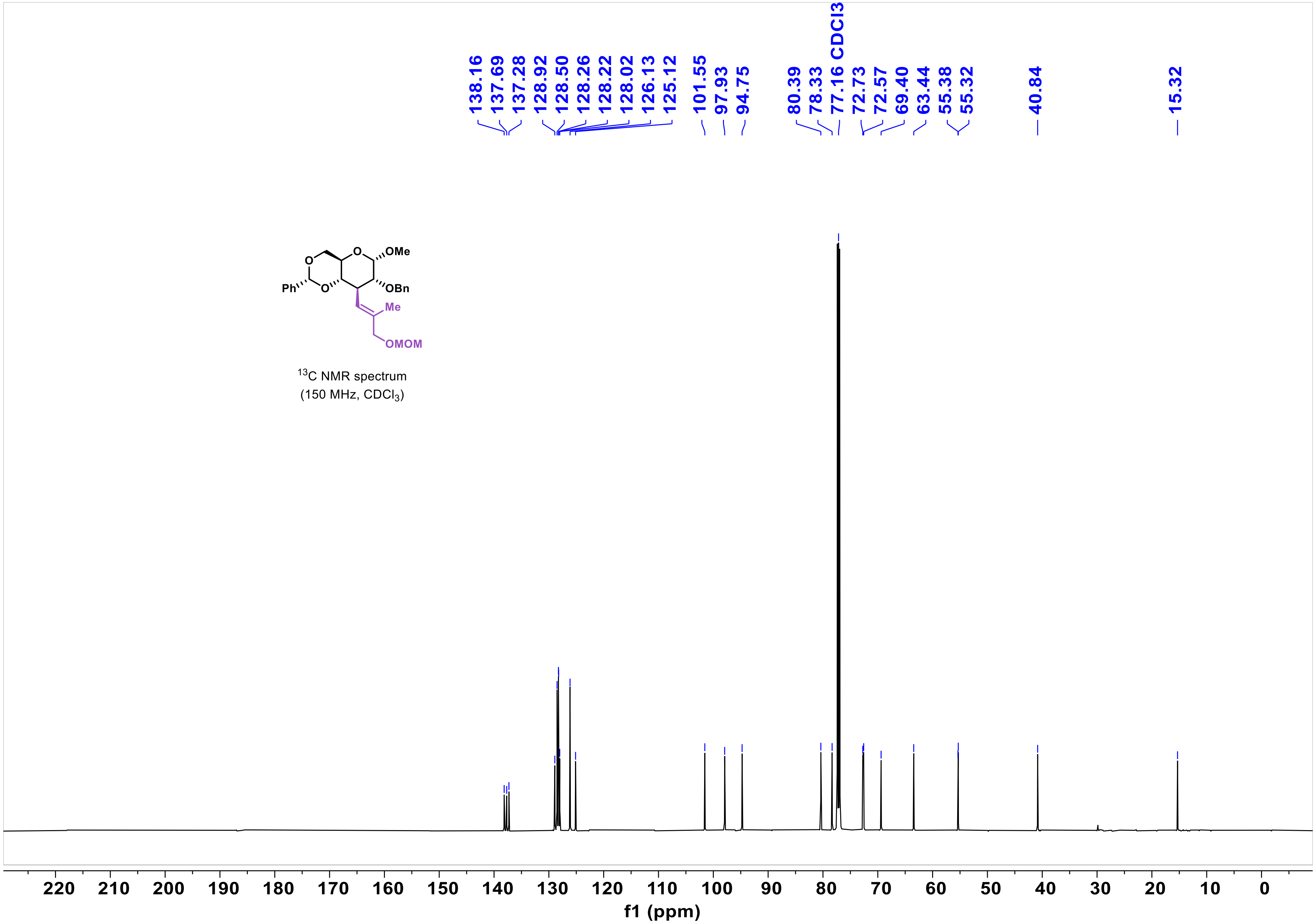

^{13}C NMR of compound **g-110**

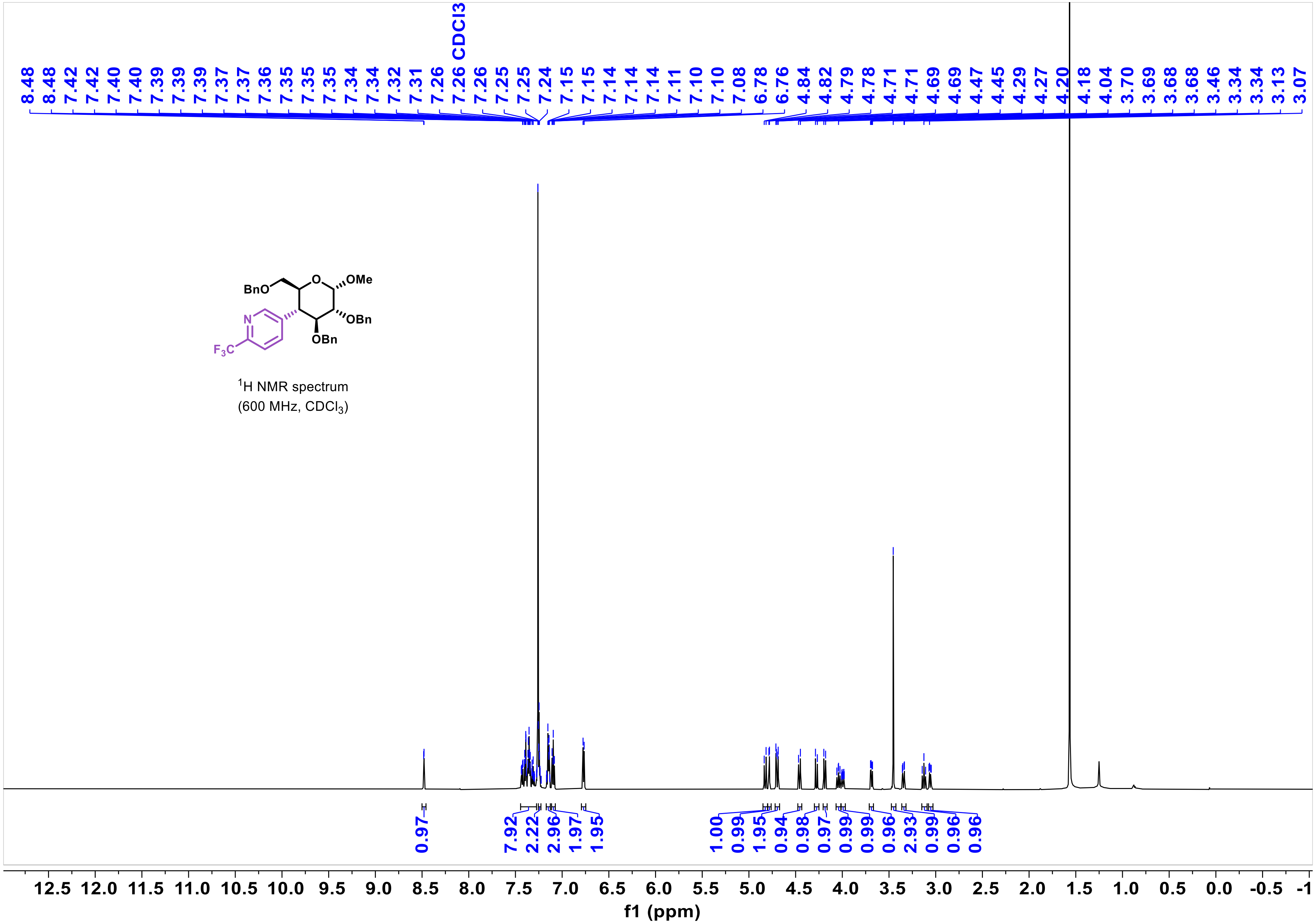

^{1}H NMR of compound **g-113**

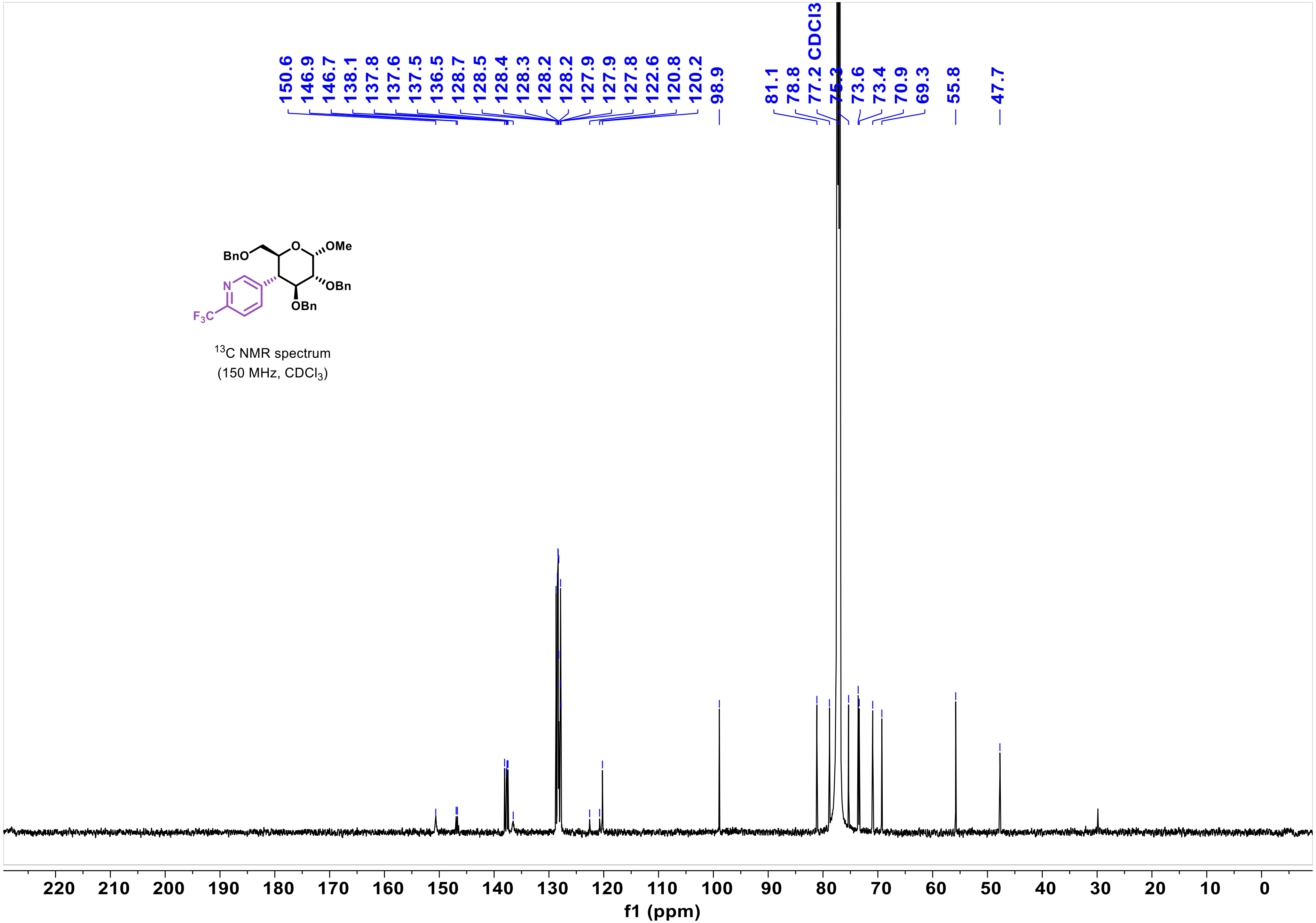

^{13}C NMR of compound **g-113**

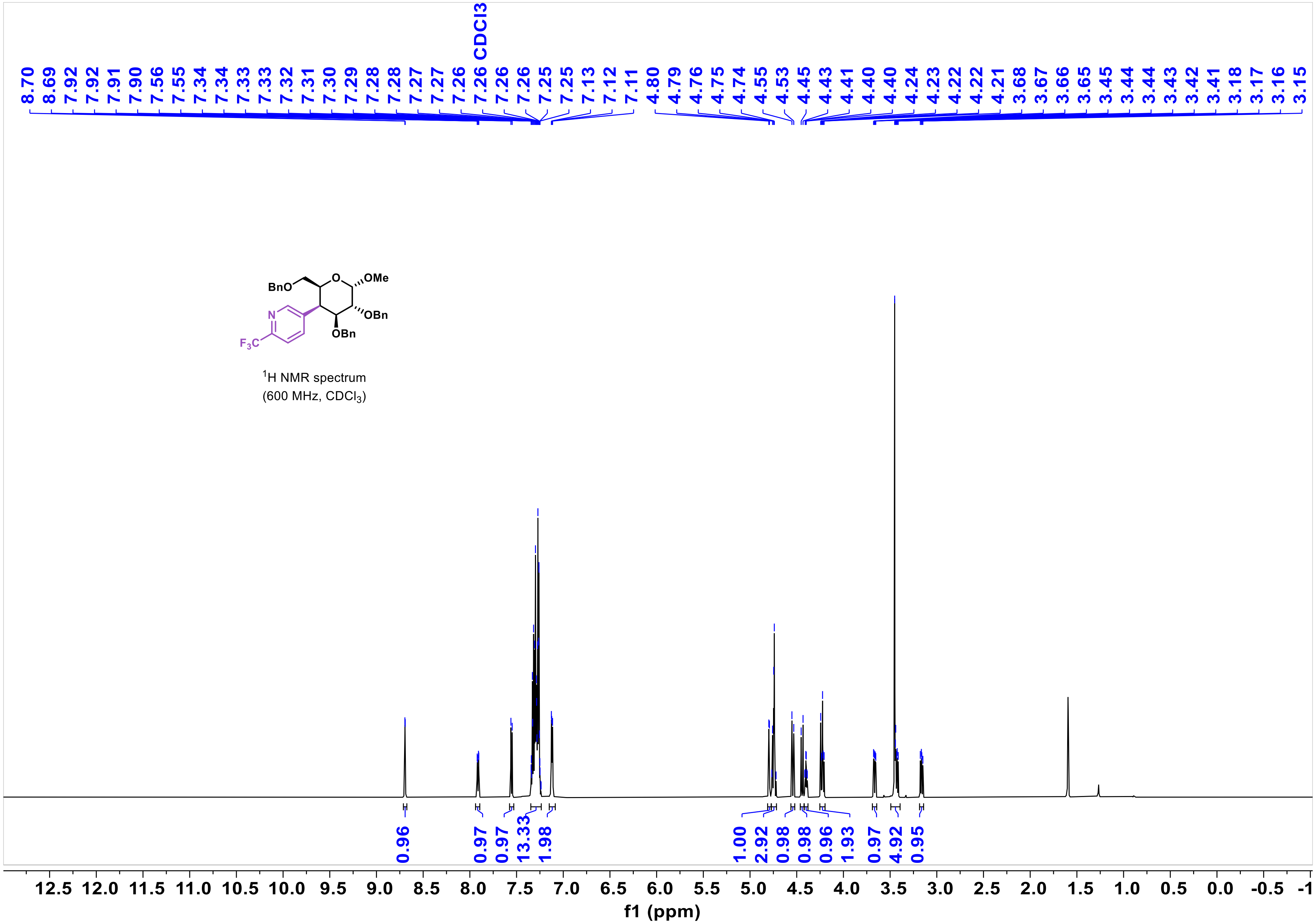

^{1}H NMR of compound **ga-113**

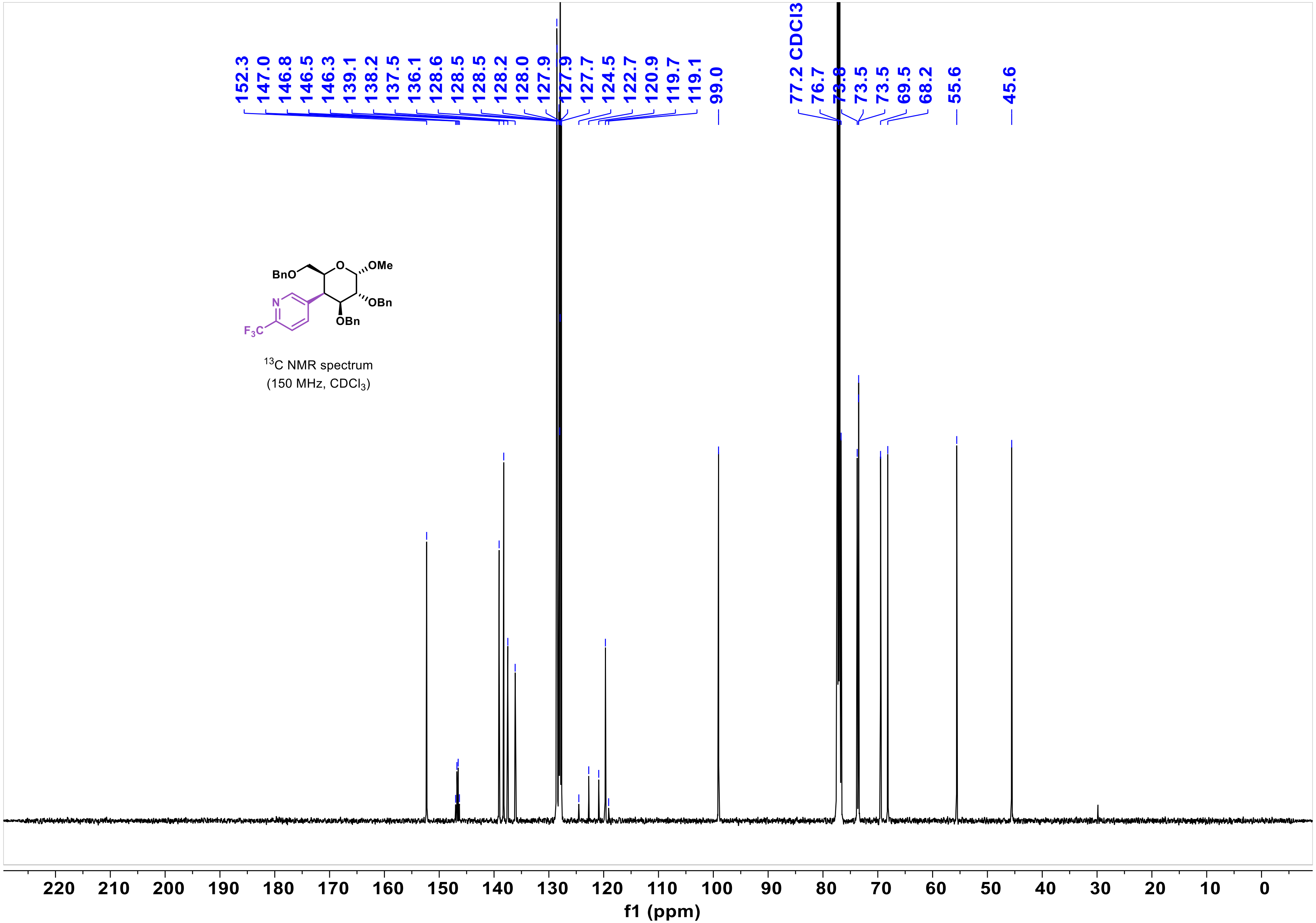

^{13}C NMR of compound **ga-113**

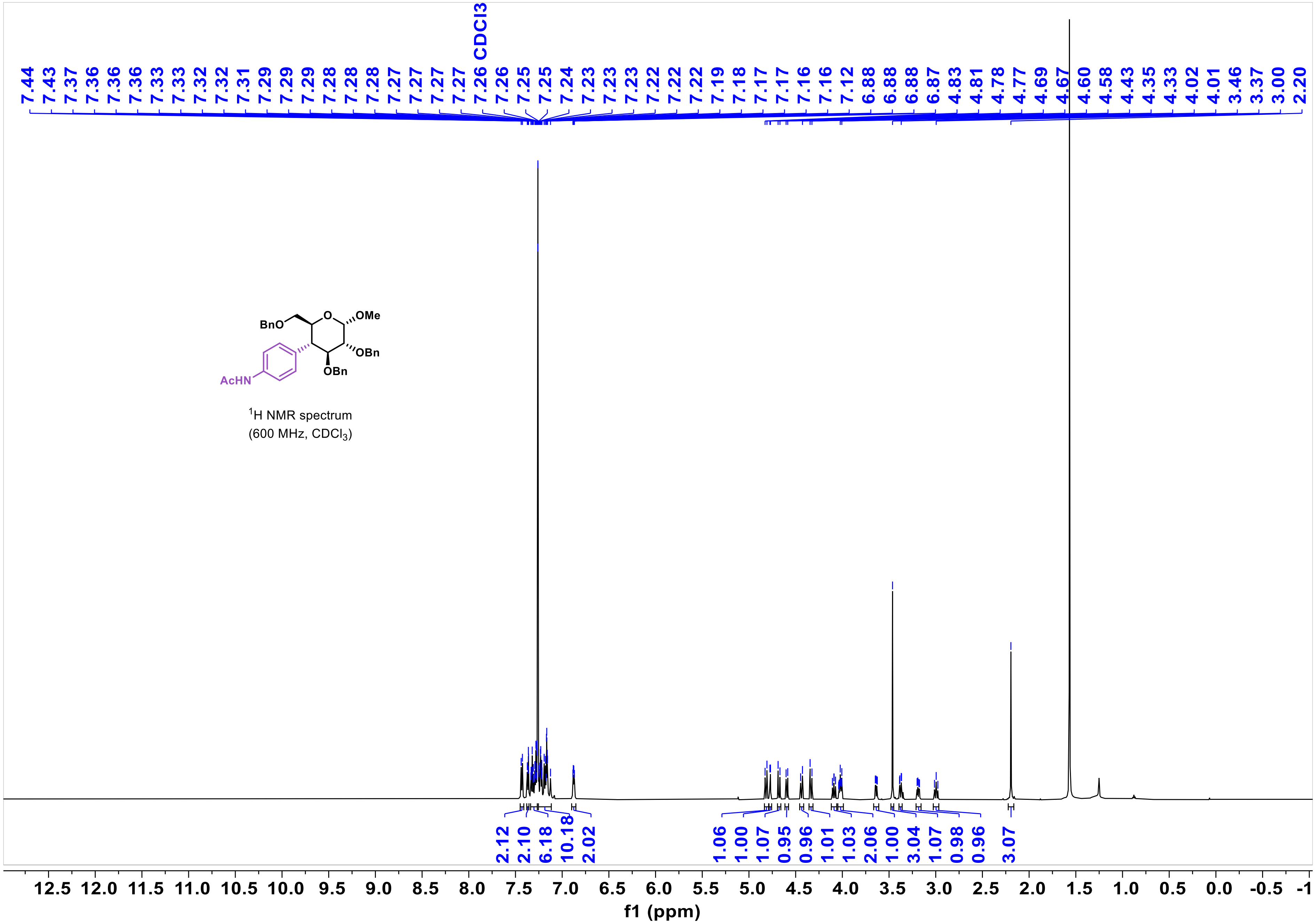

^{1}H NMR of compound **g-114**

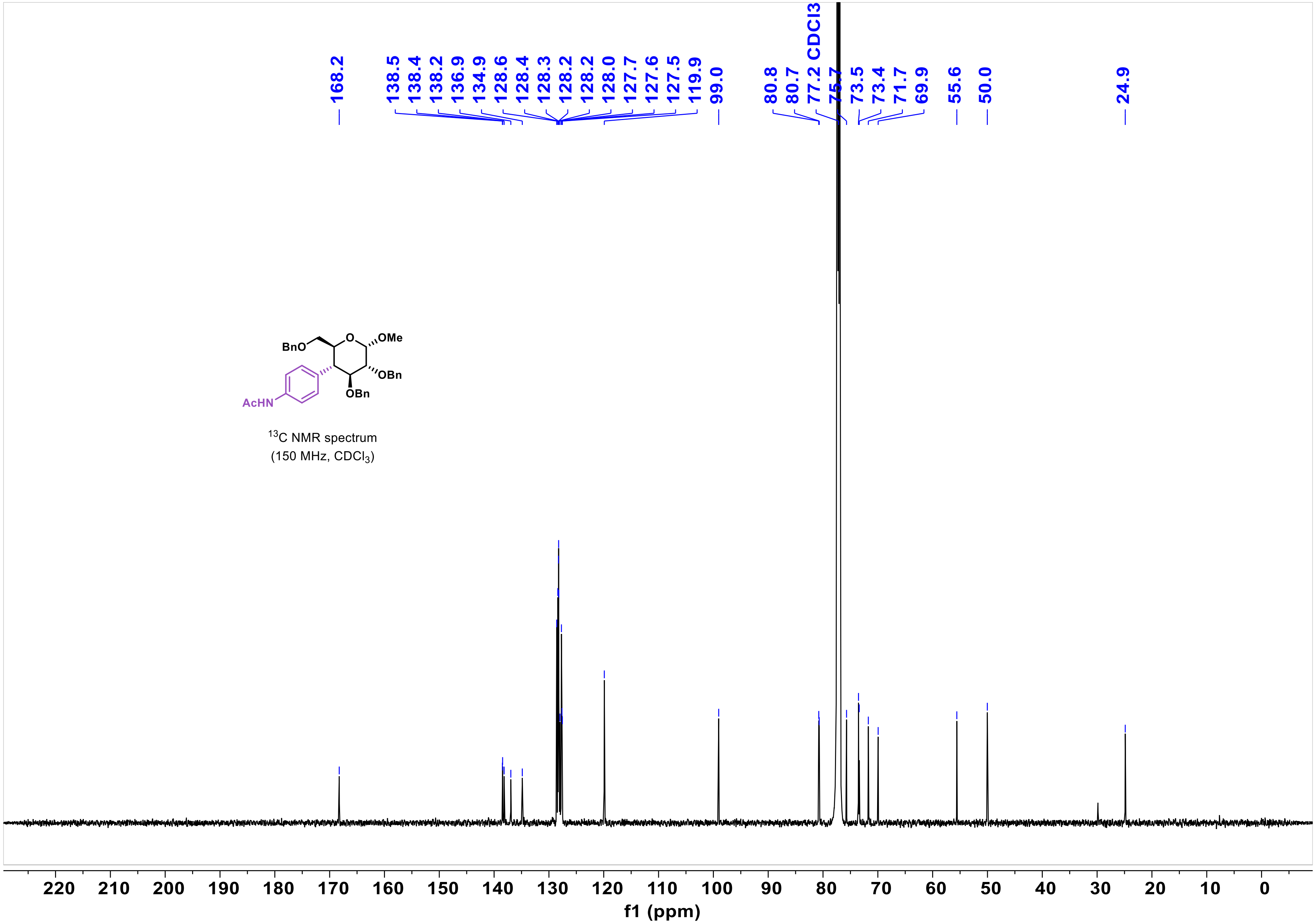

^{13}C NMR of compound **g-114**

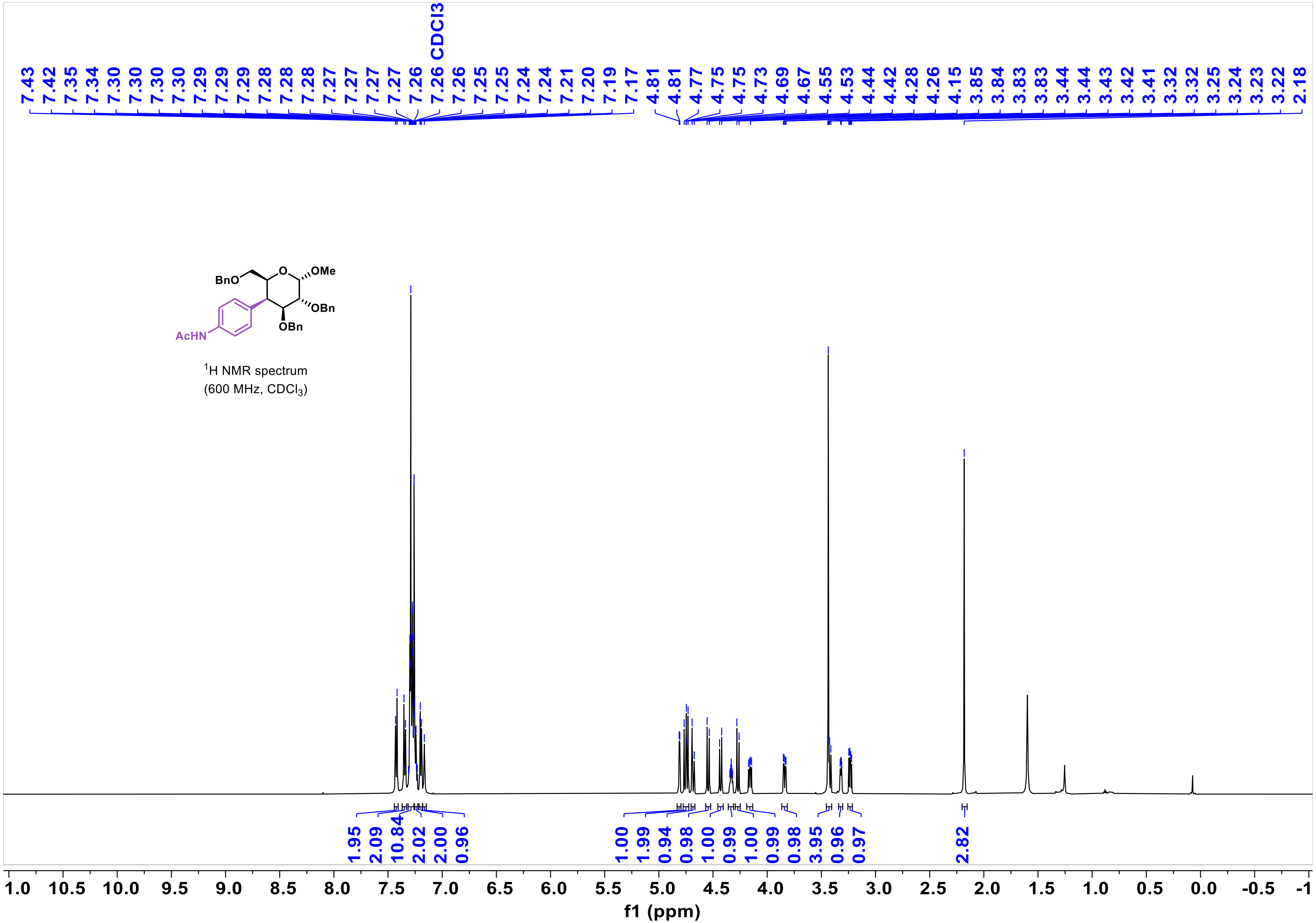

^{1}H NMR of compound **ga-114**

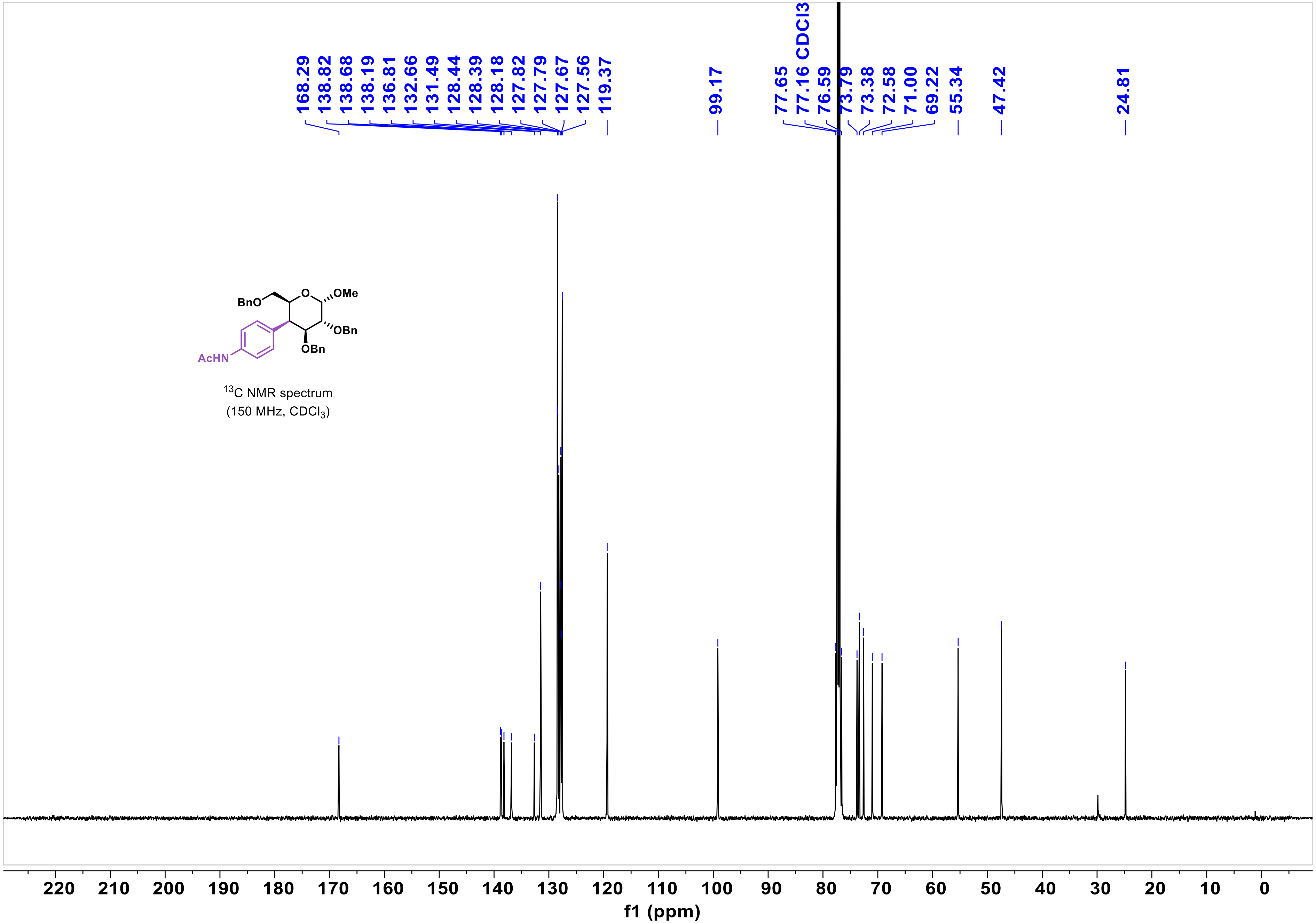

^{13}C NMR of compound **ga-114**

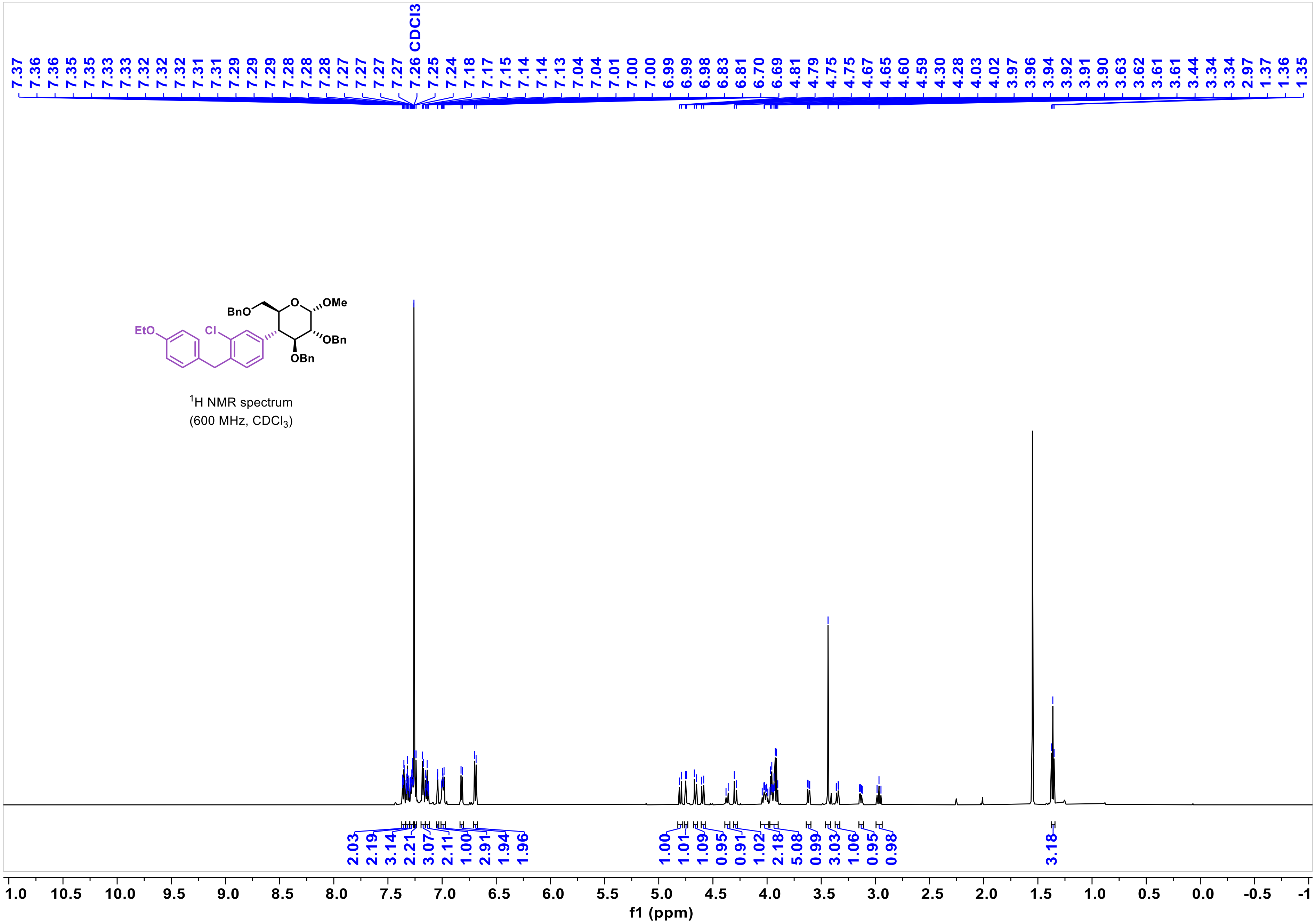

^{1}H NMR of compound **g-115**

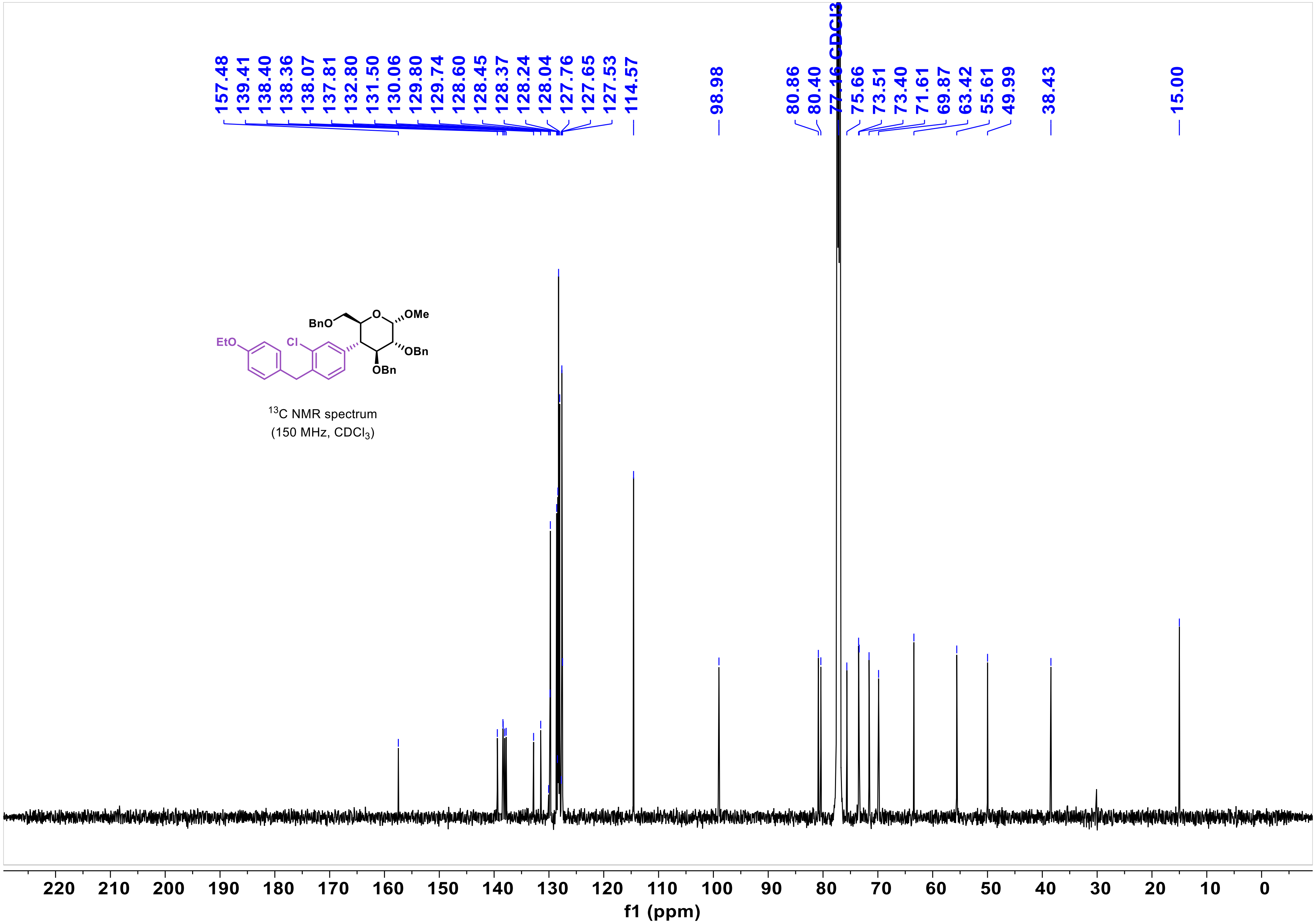

^{13}C NMR of compound **g-115**

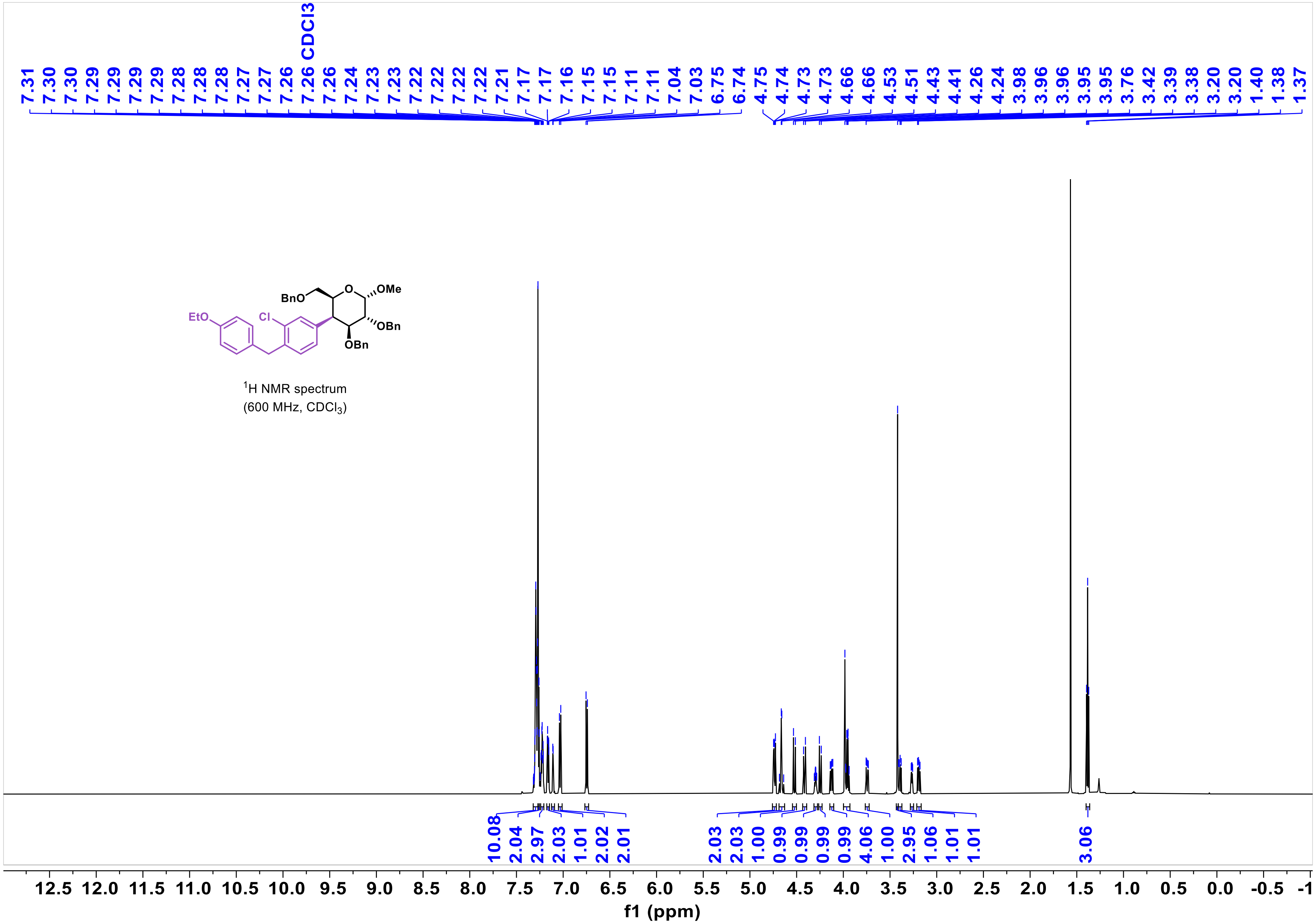

^{1}H NMR of compound **ga-115**

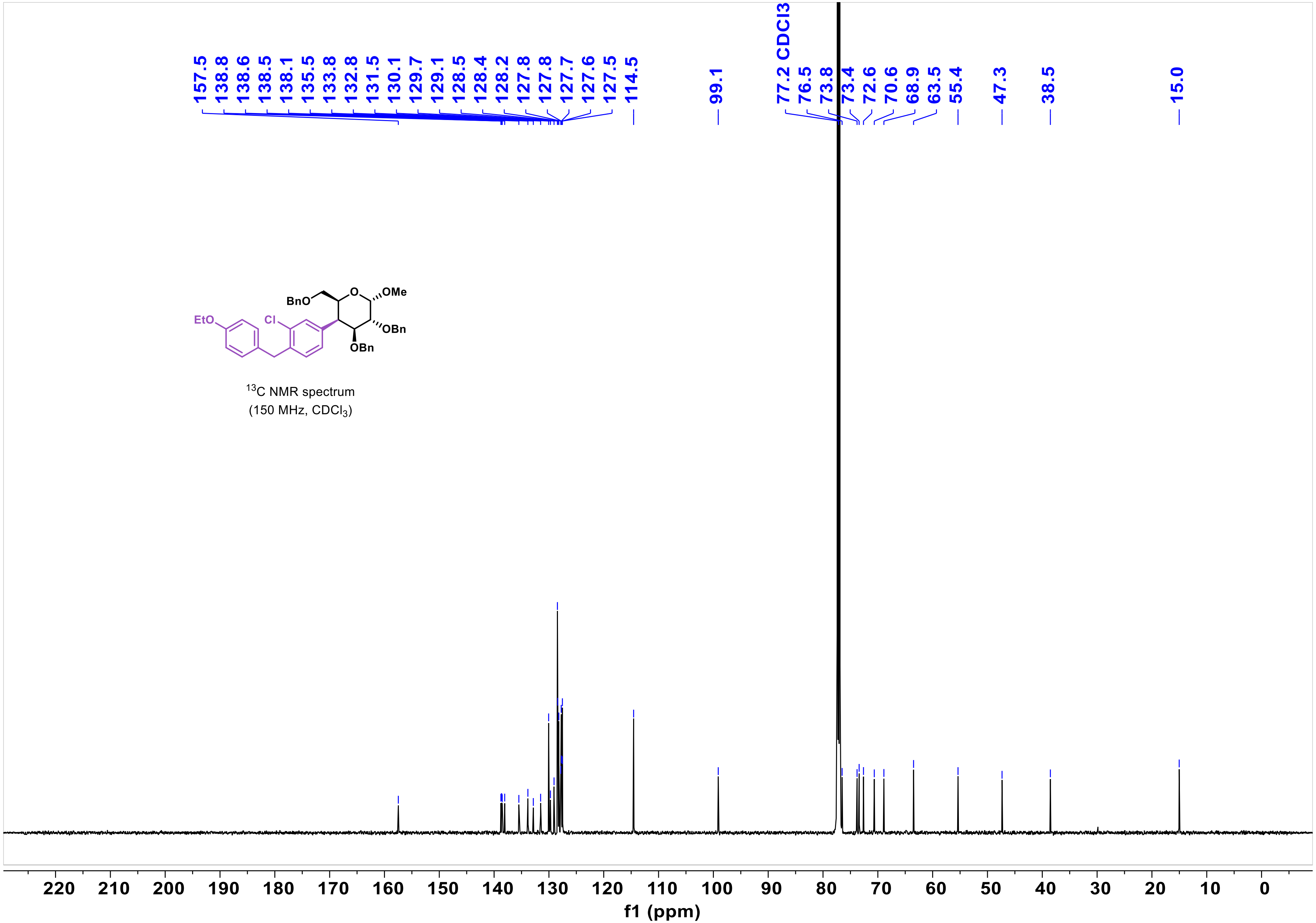

^{13}C NMR of compound **ga-115**

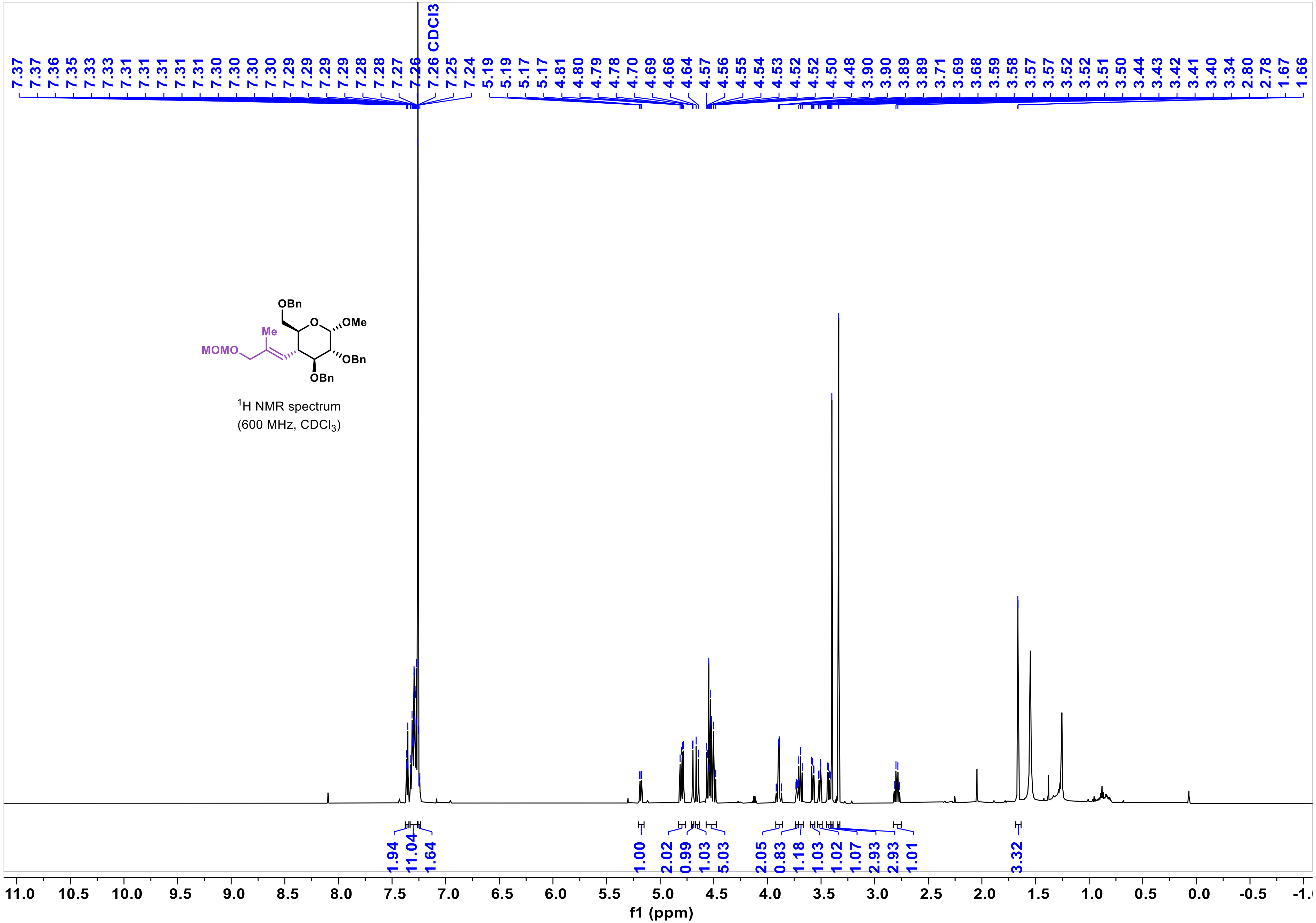

^{1}H NMR of compound **g-116**

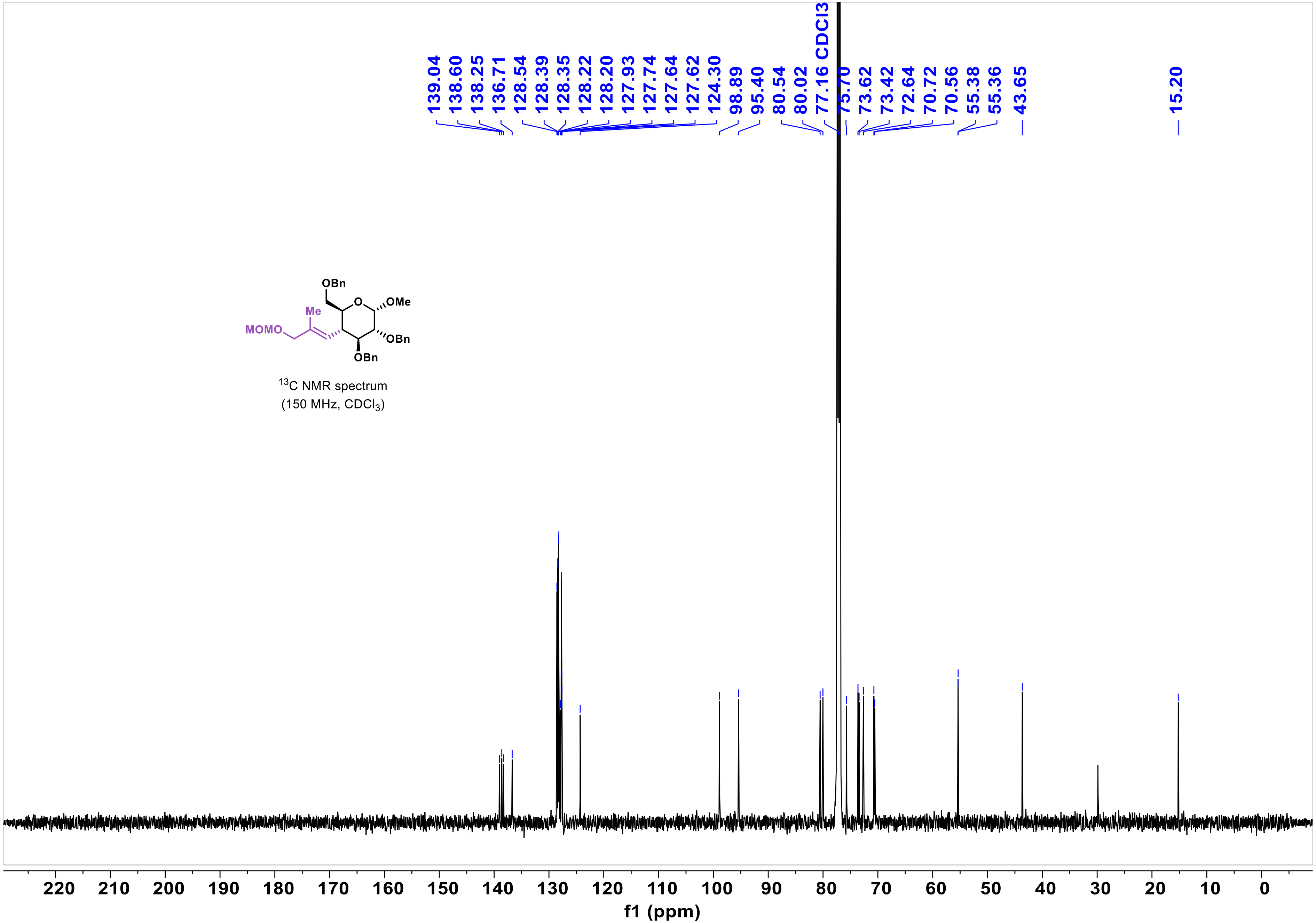

^{13}C NMR of compound **g-116**

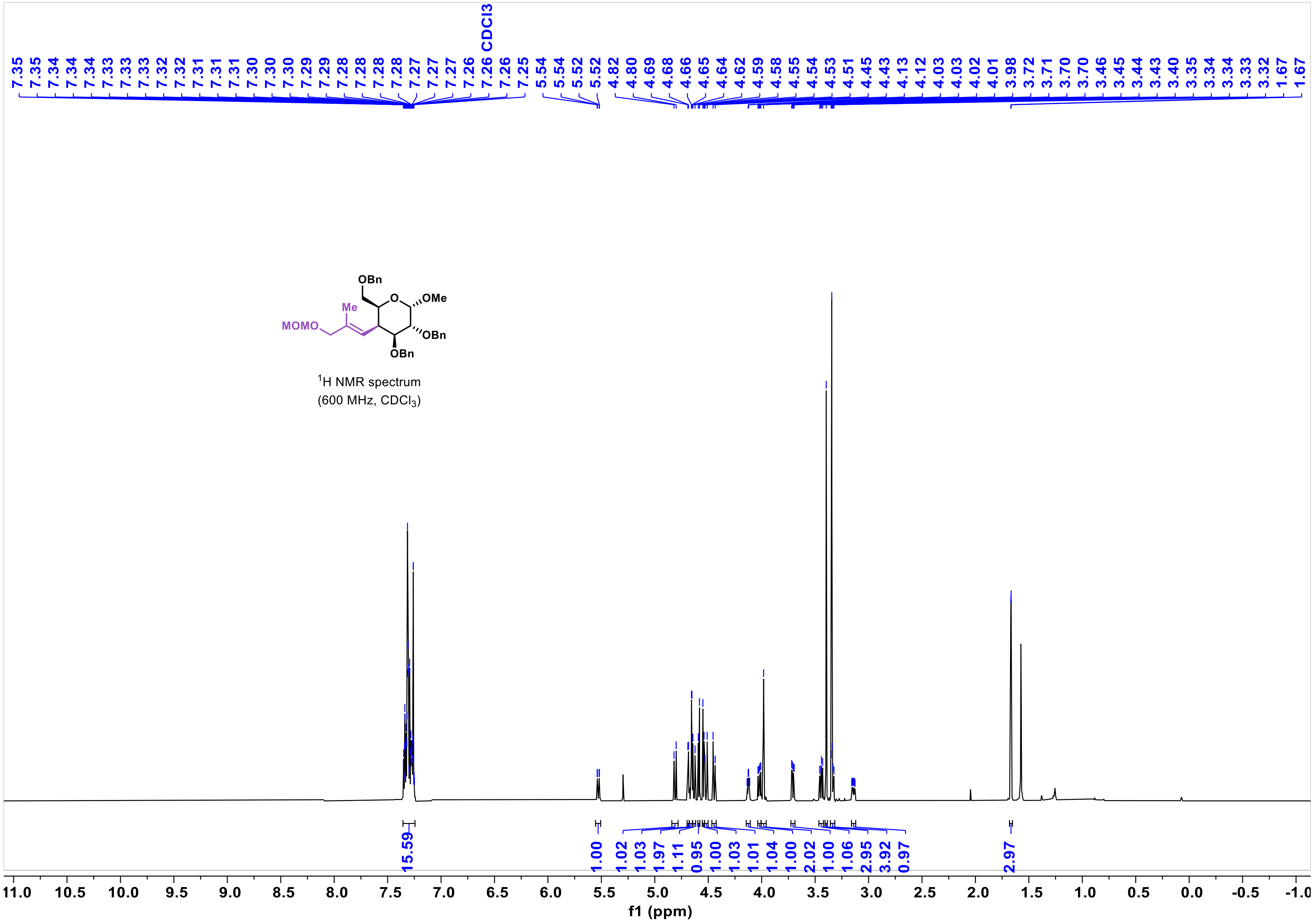

^{1}H NMR of compound **ga-116**

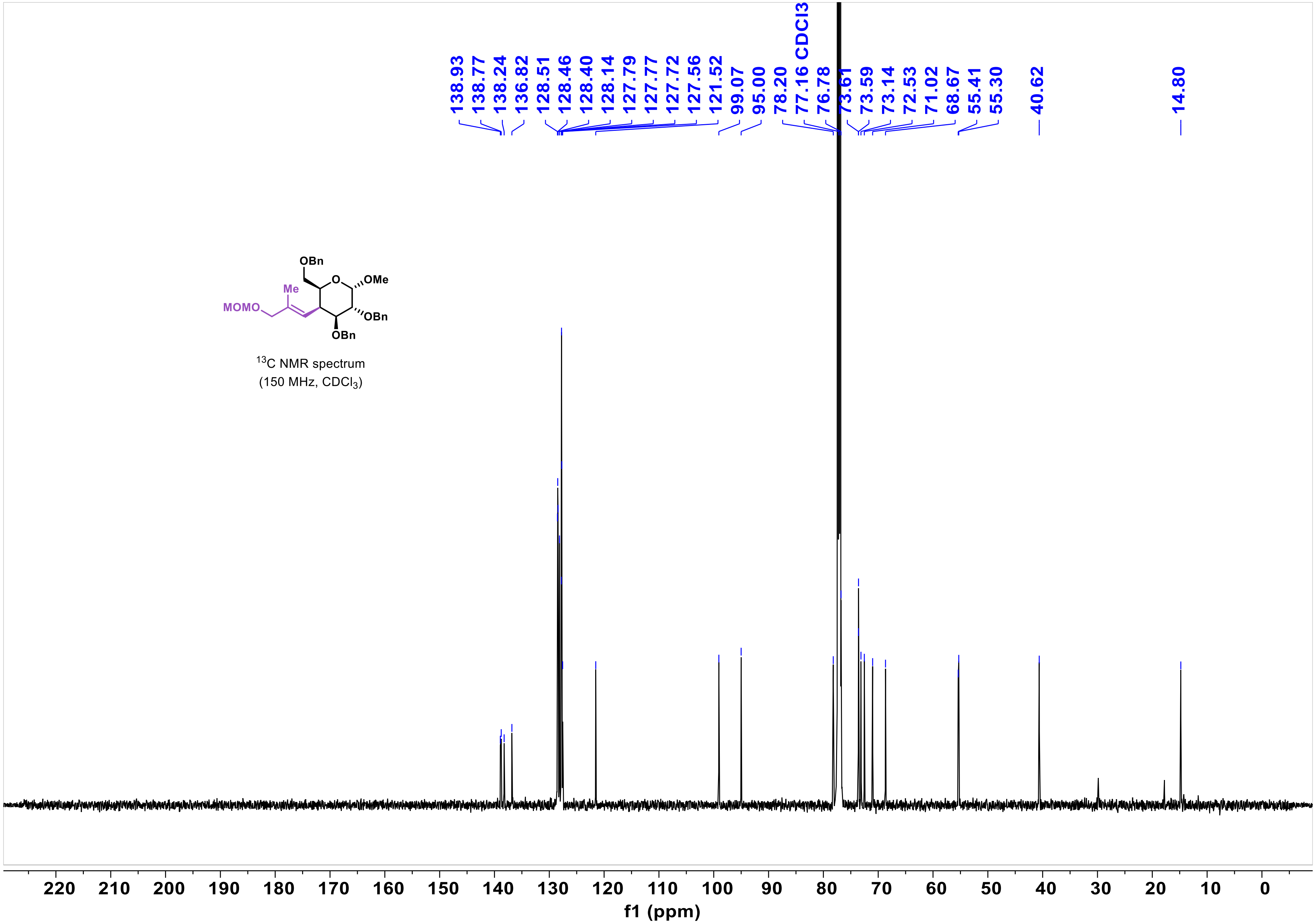

^{13}C NMR of compound **ga-116**

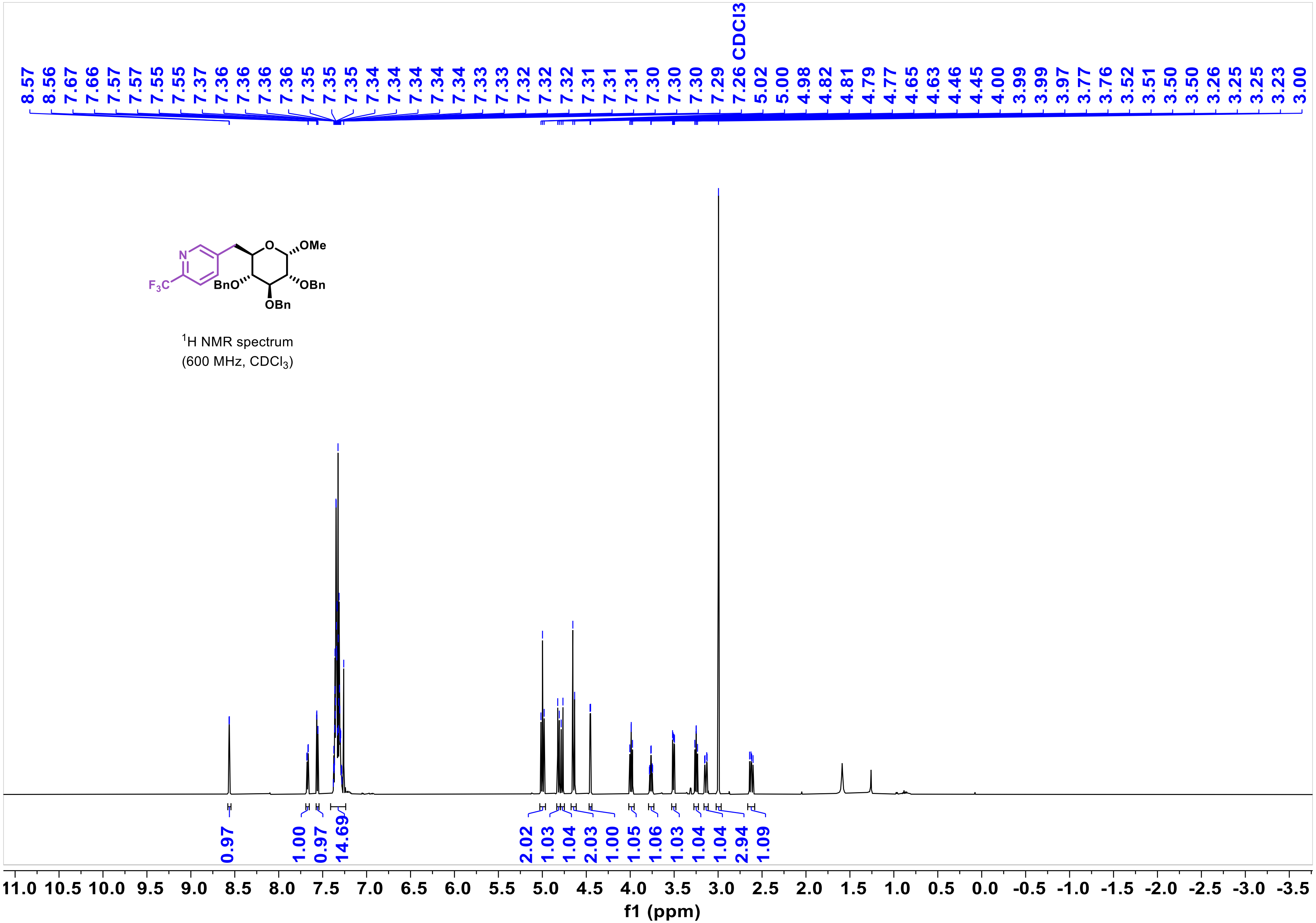

^{1}H NMR of compound **119**

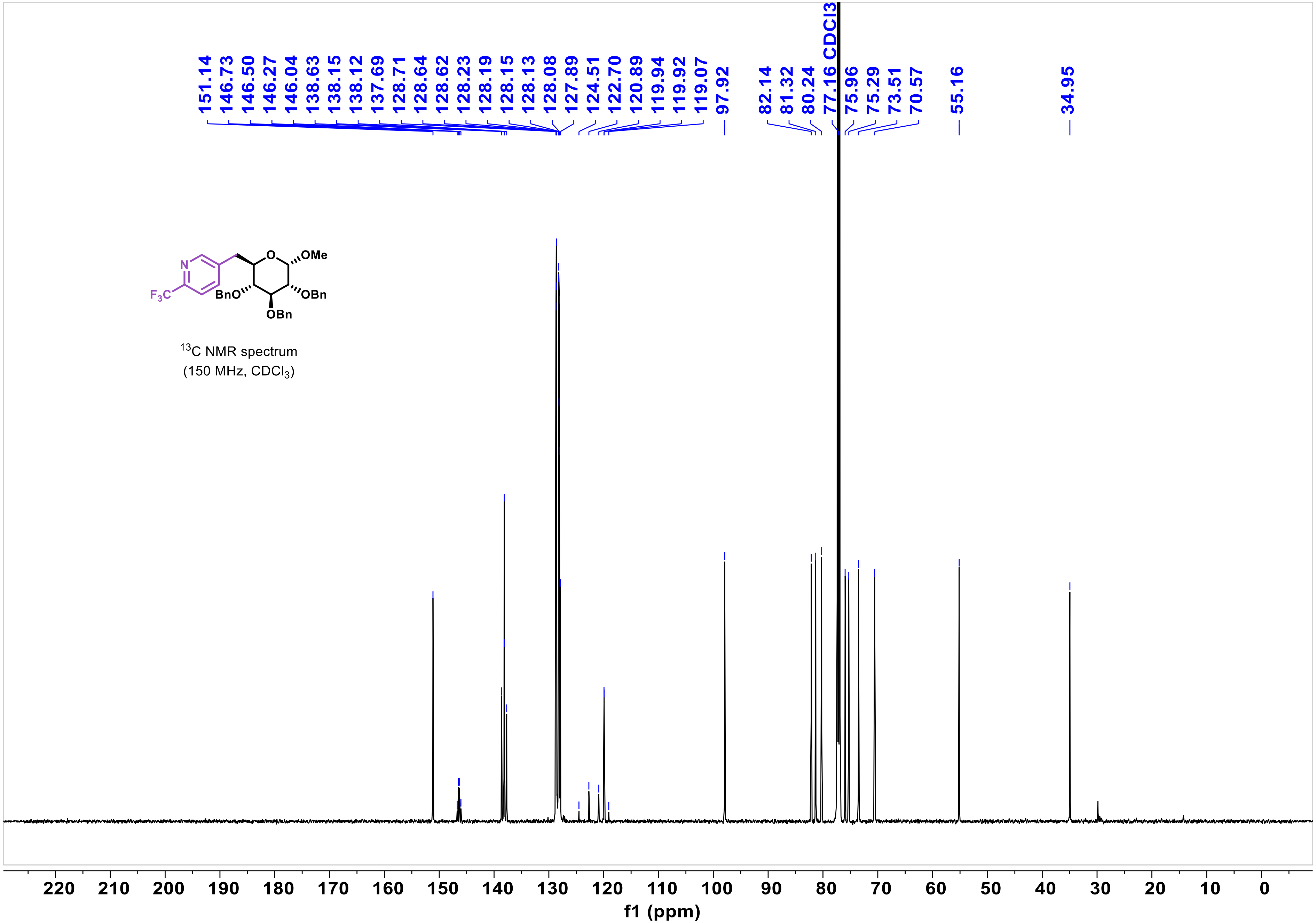

^{13}C NMR of compound **119**

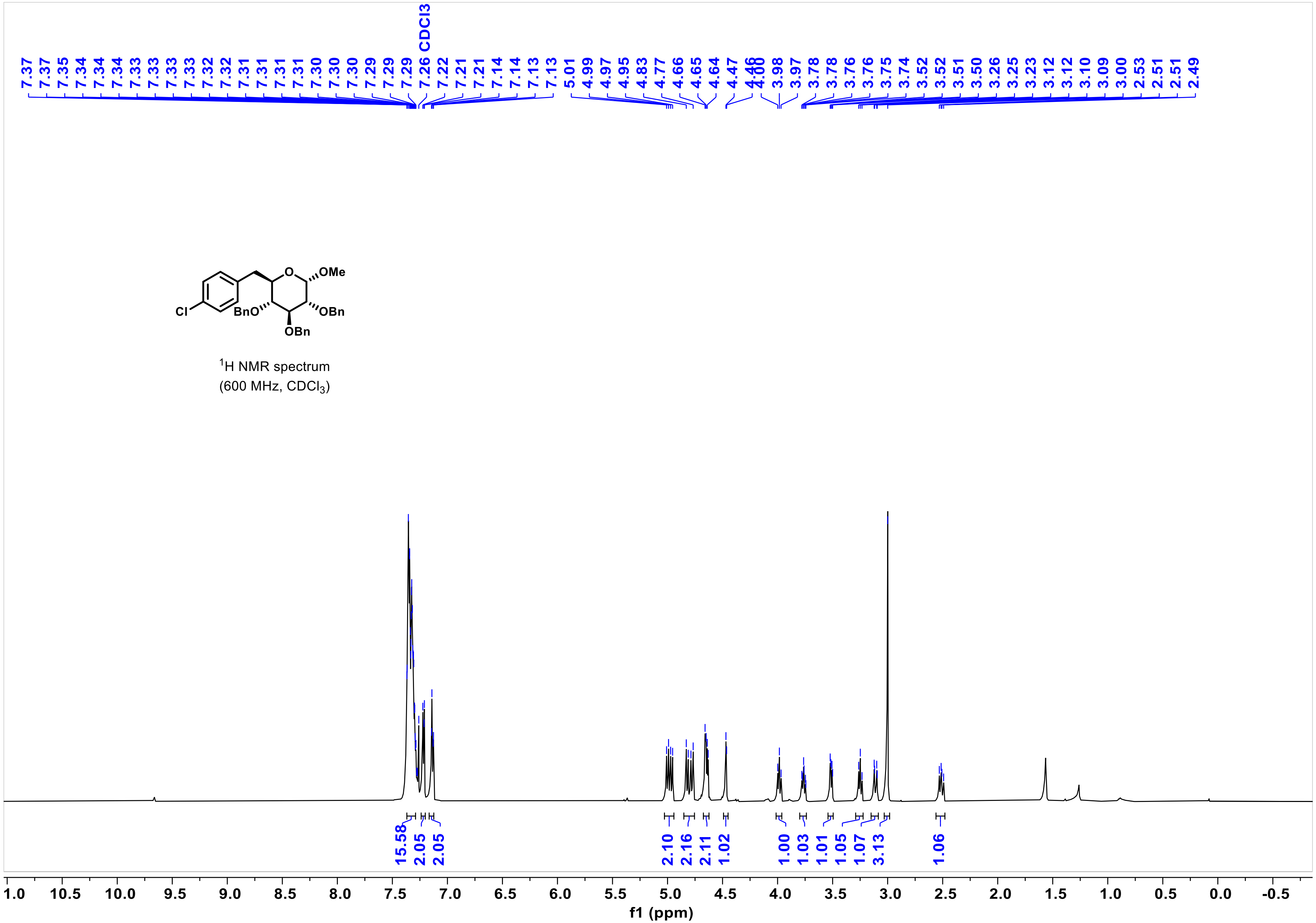

^{1}H NMR of compound **120**

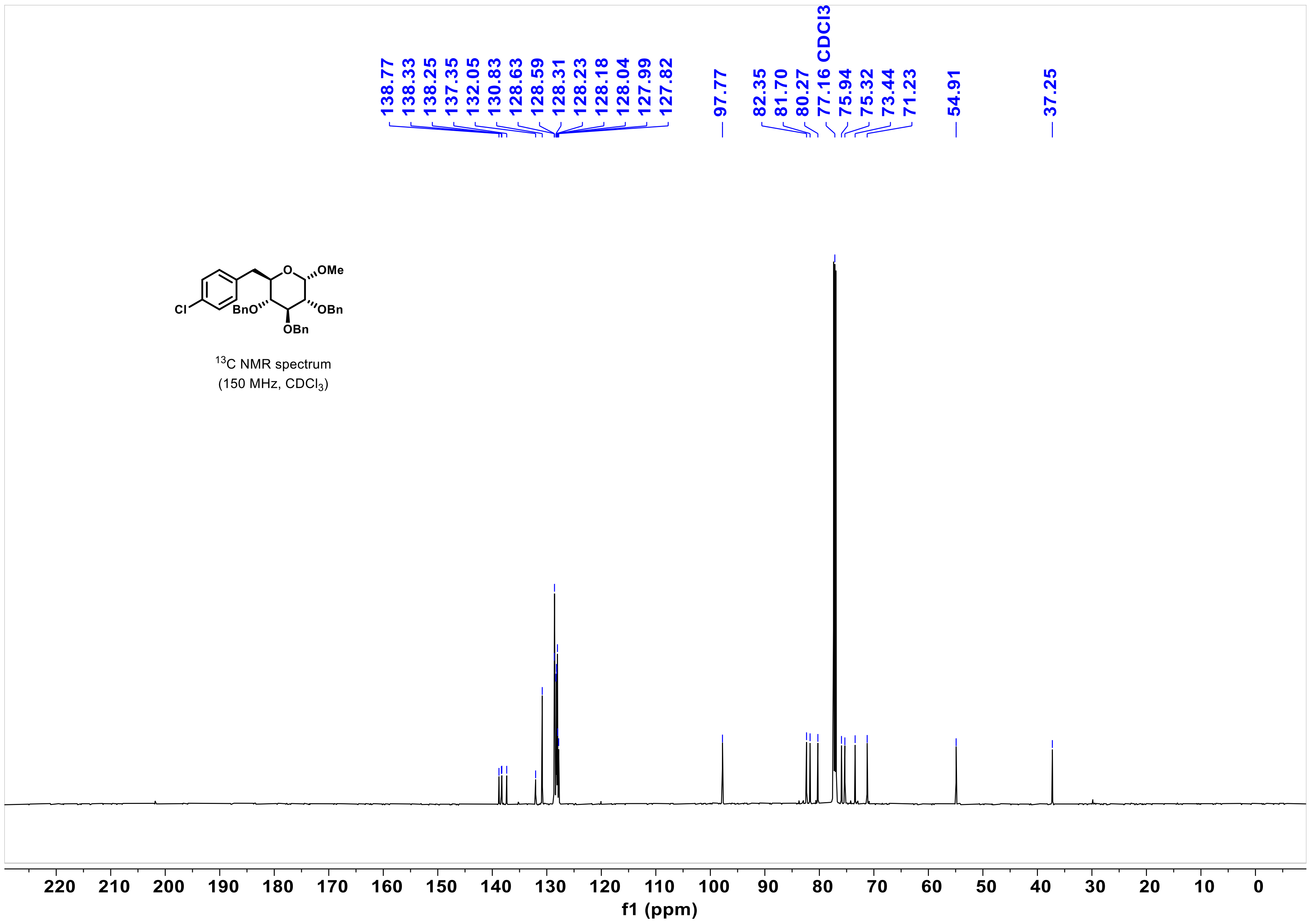

^{13}C NMR of compound **120**

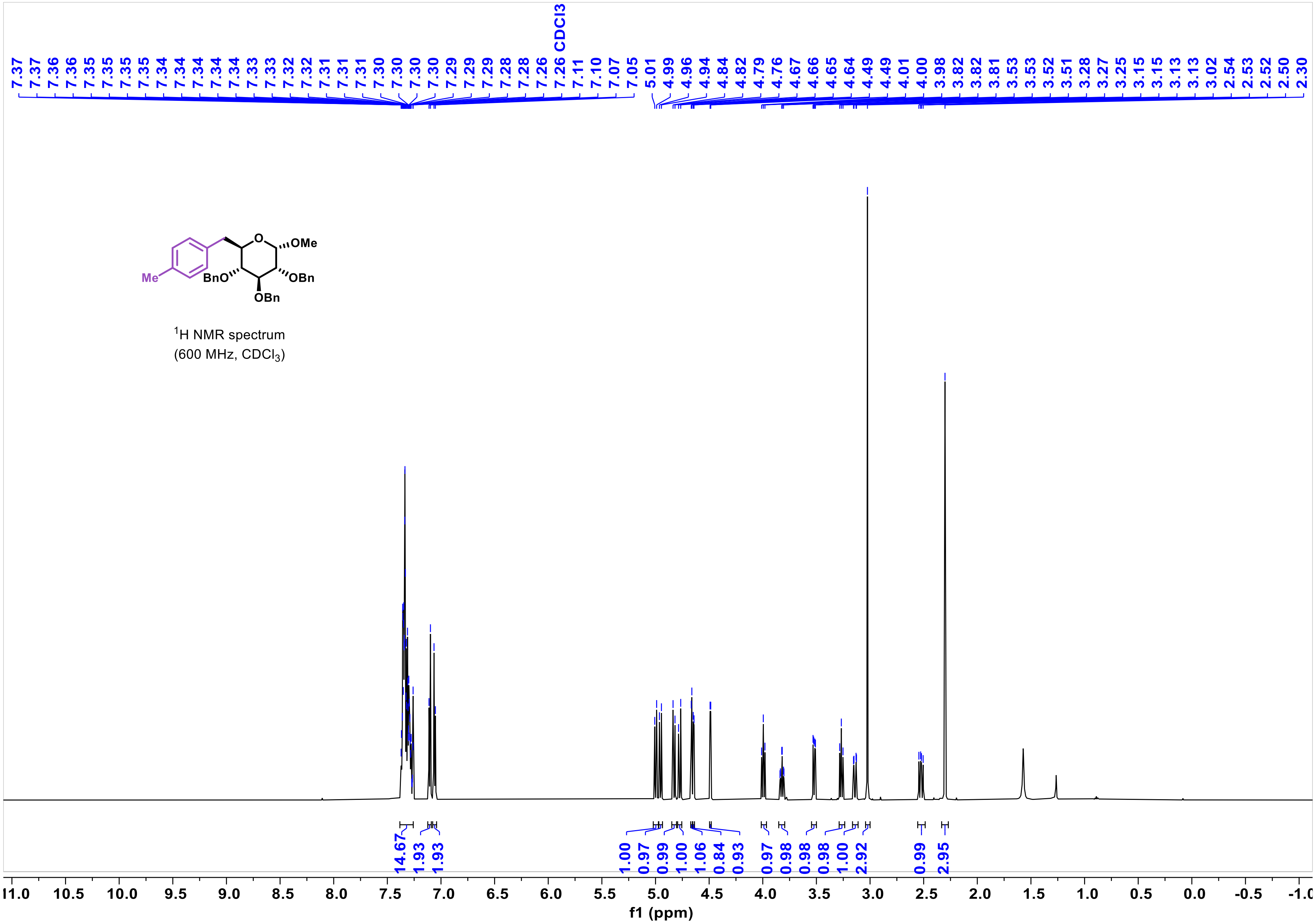

^{1}H NMR of compound **121**

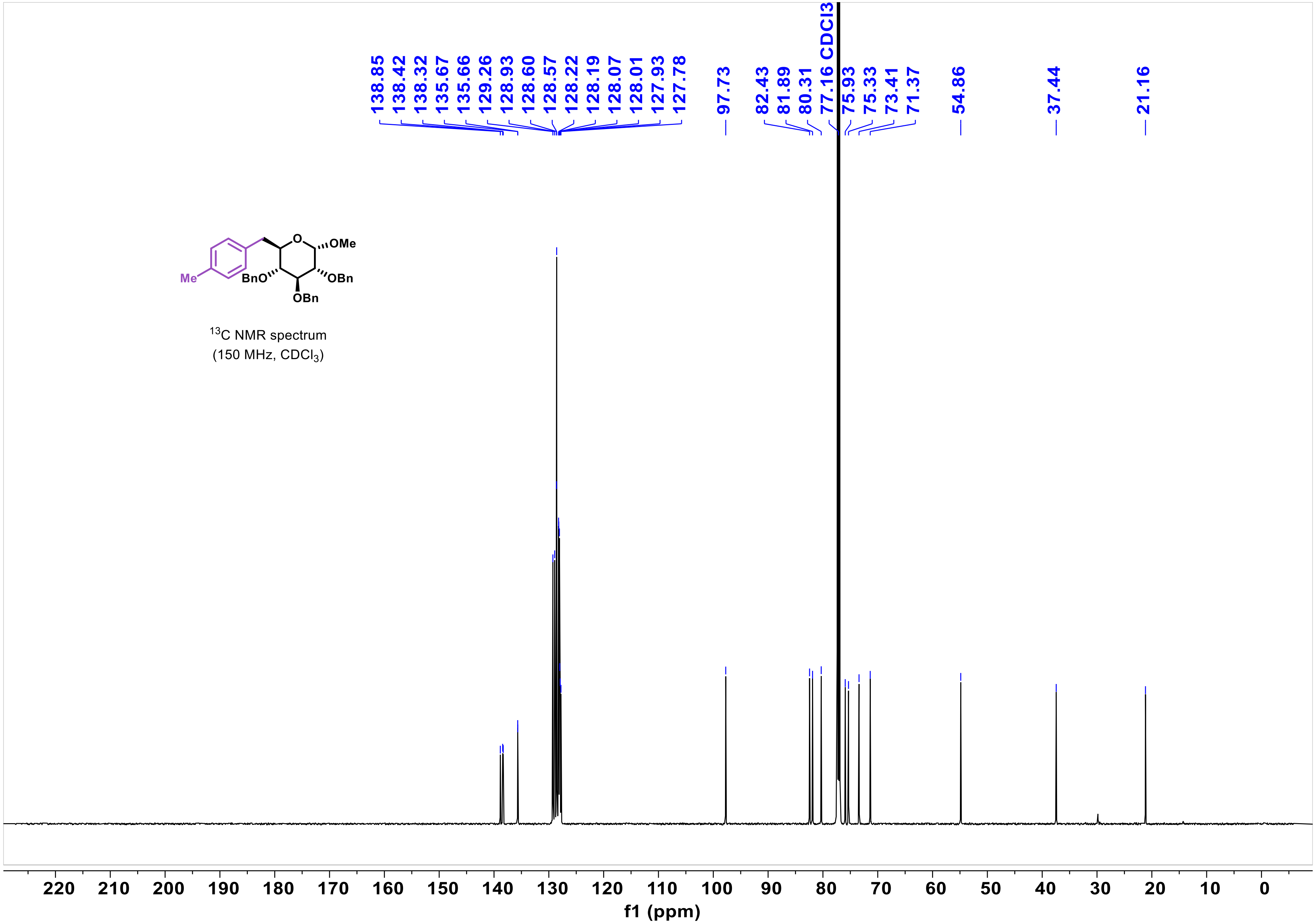

^{13}C NMR of compound **121**

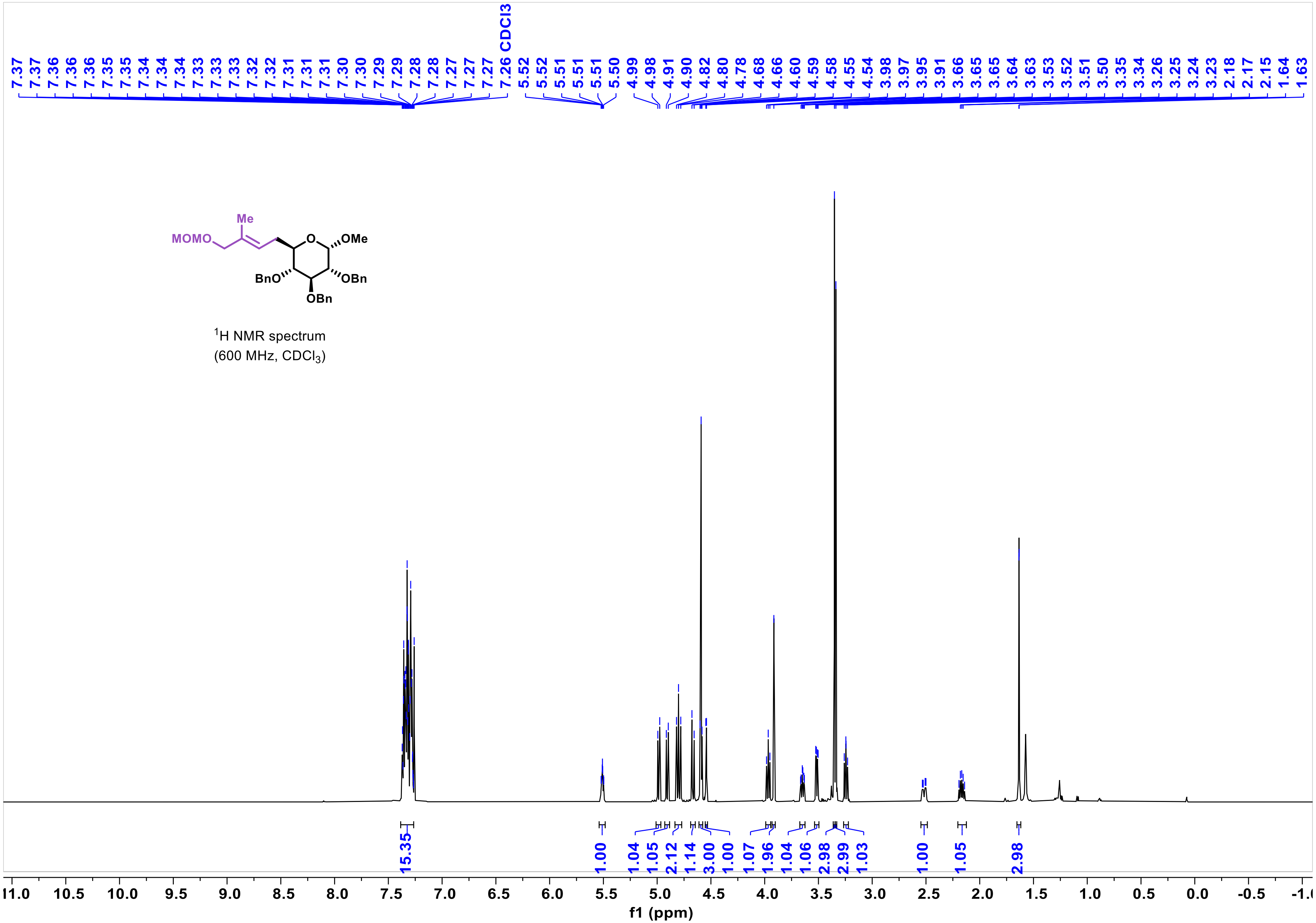

^{1}H NMR of compound **122**

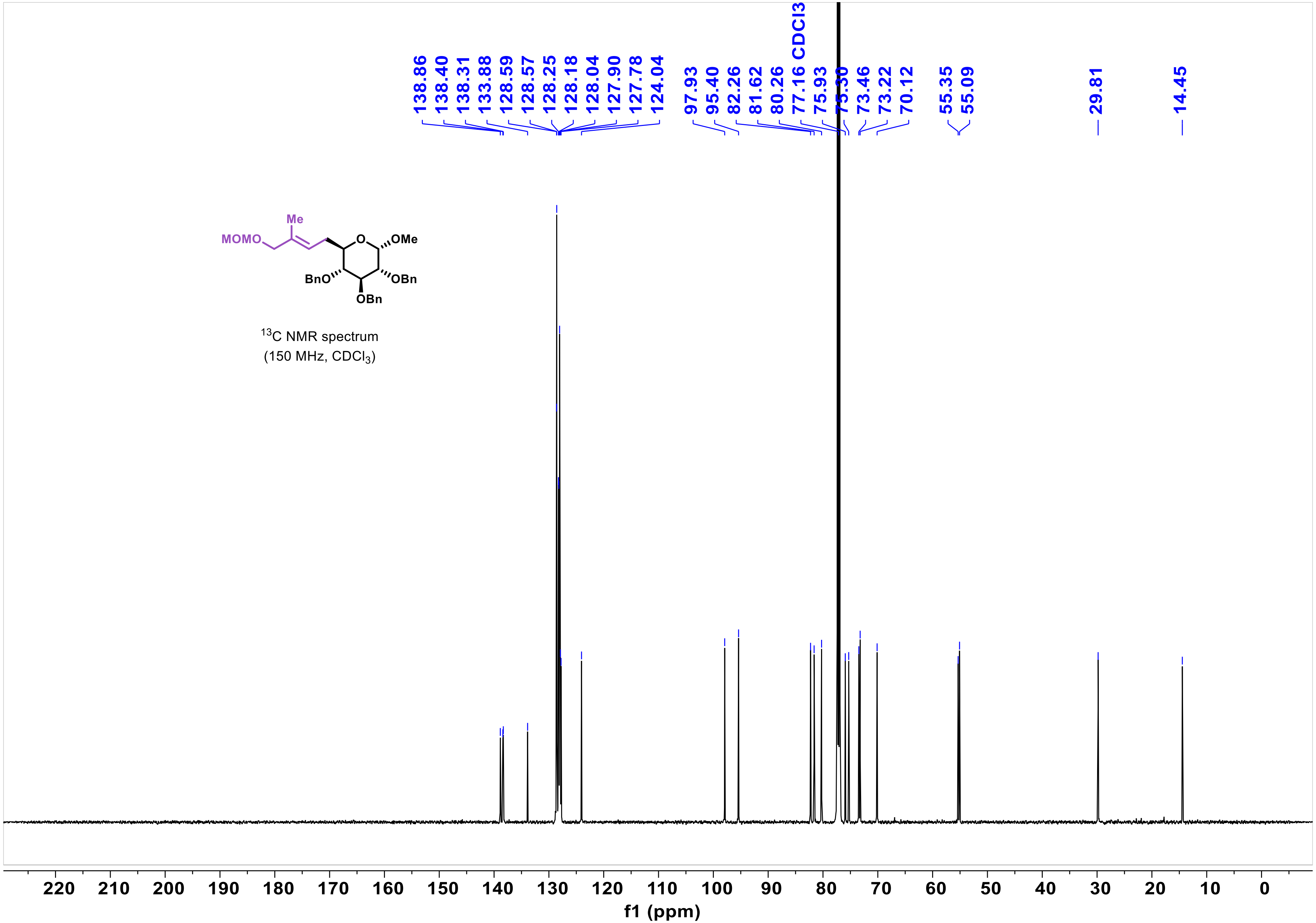

^{13}C NMR of compound **122**

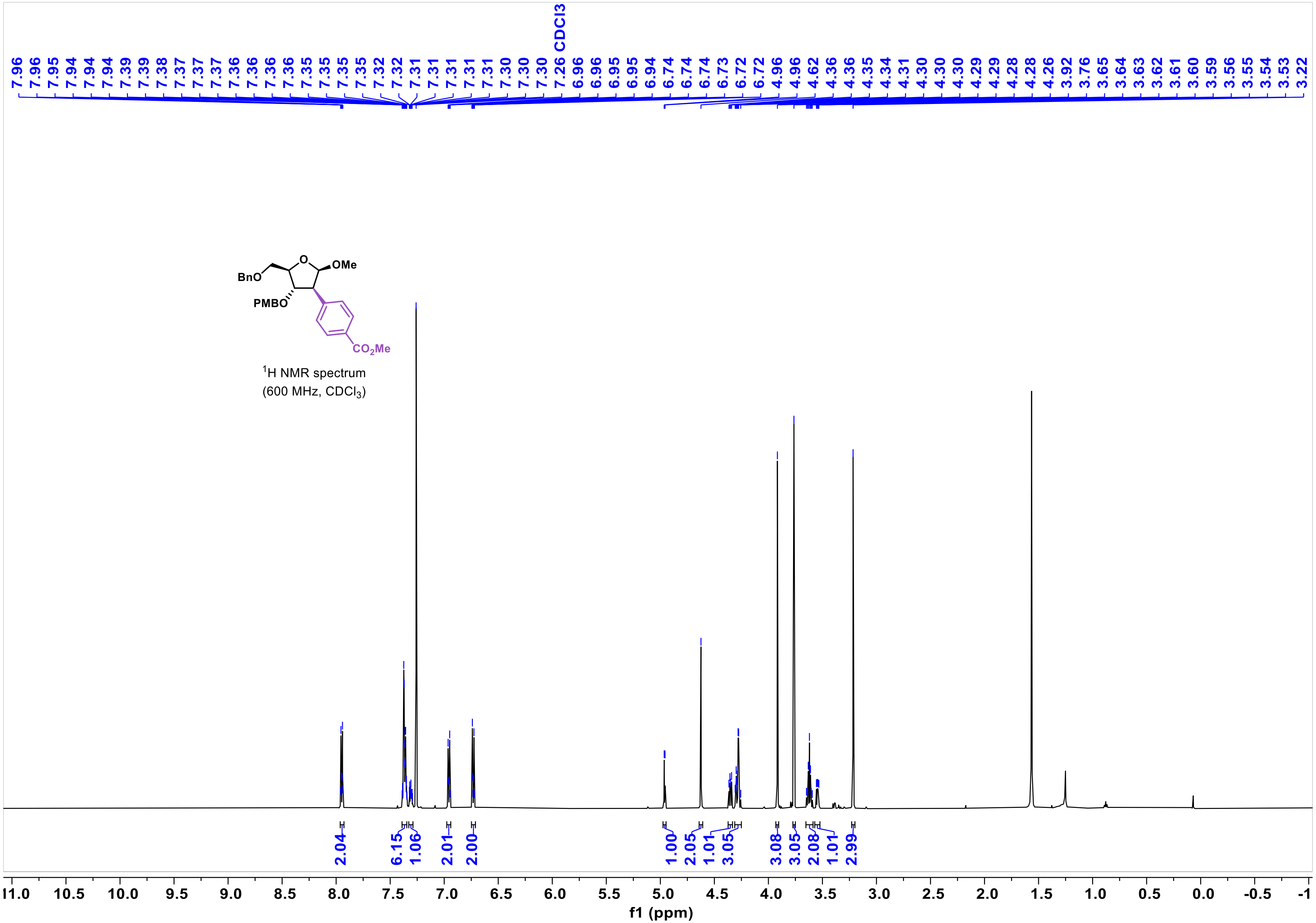

^{1}H NMR of compound **arabino-124**

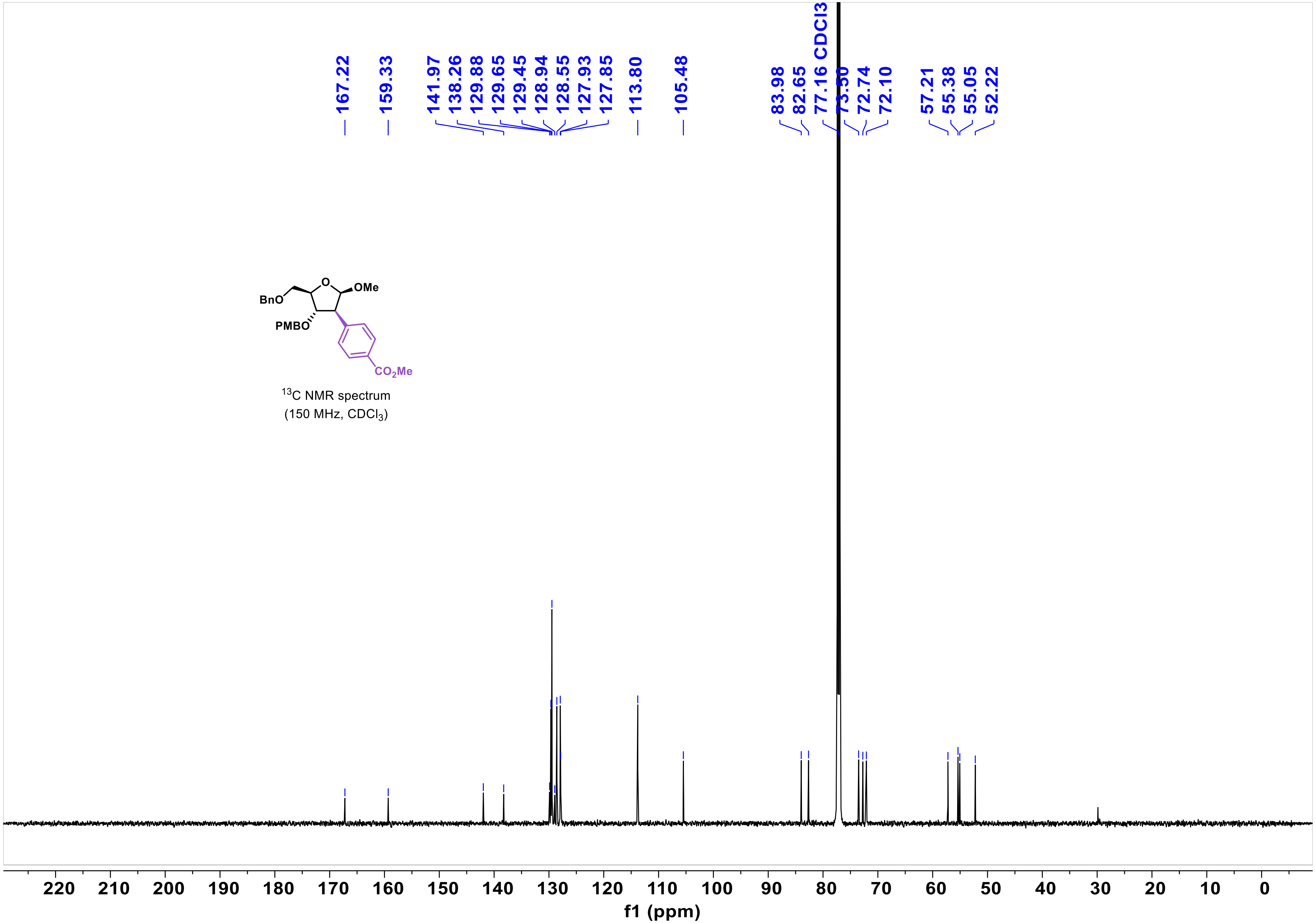

^{13}C NMR of compound **arabino-124**

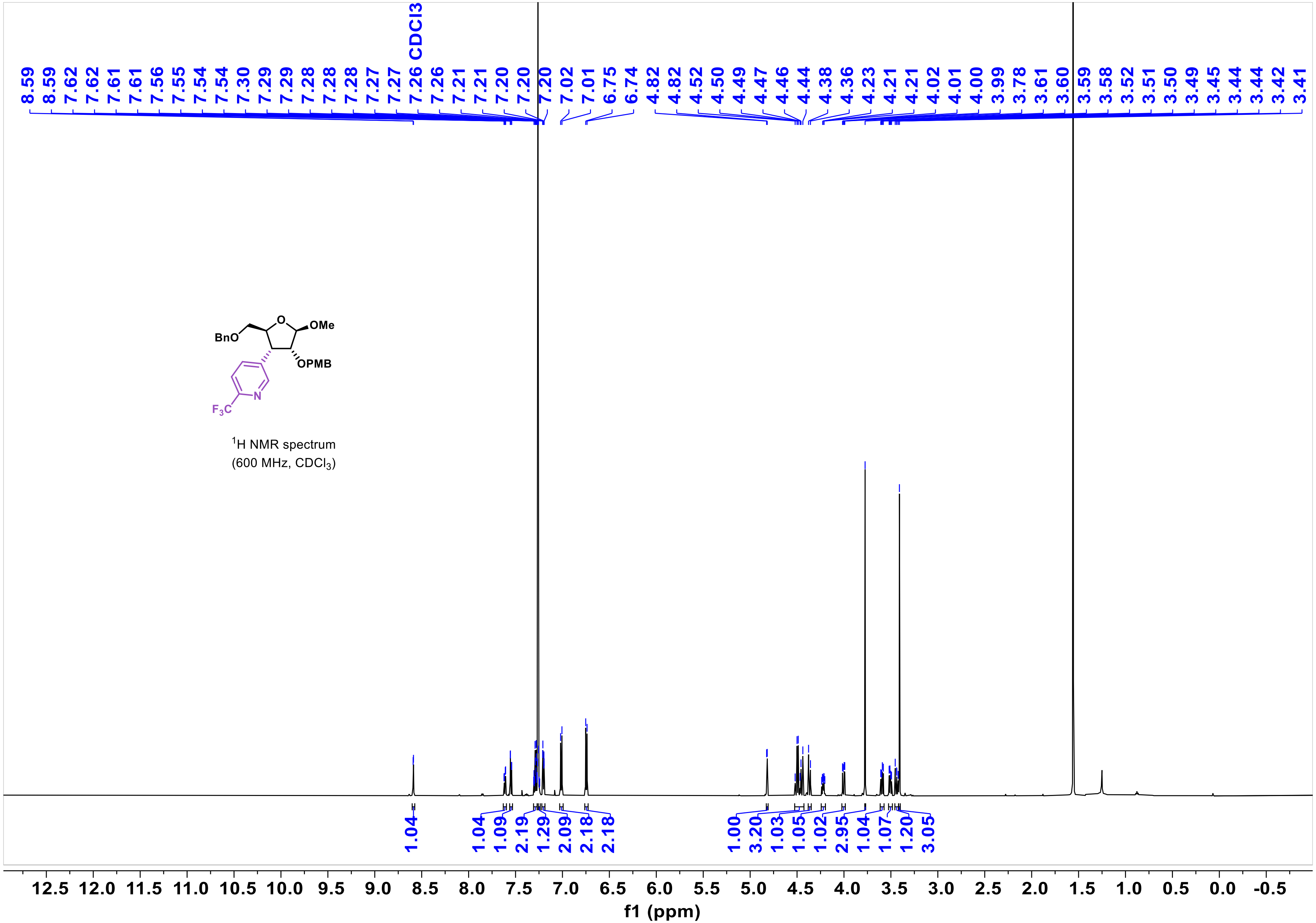

^{1}H NMR of compound **ribo-126**

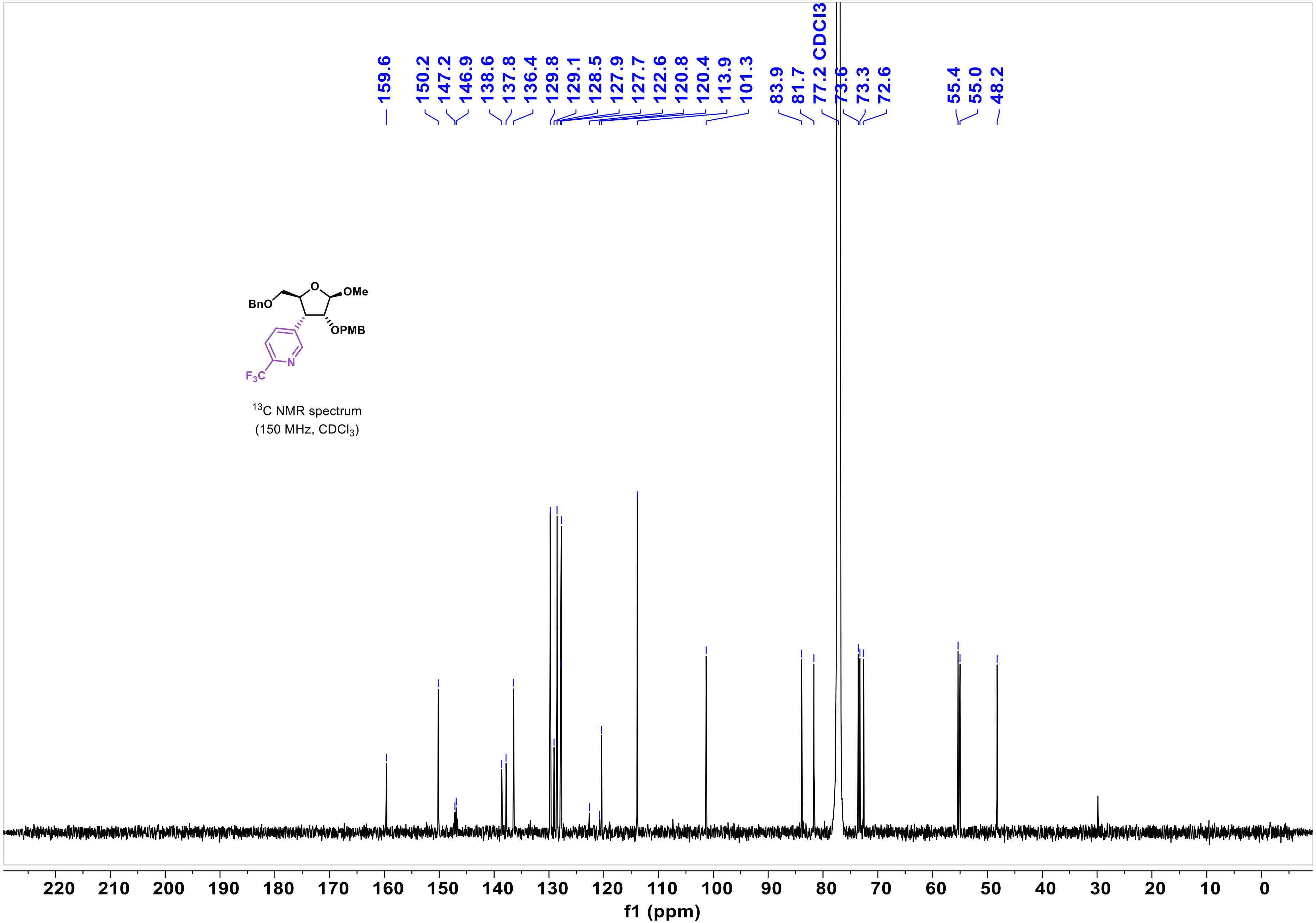

^{13}C NMR of compound **ribo-126**

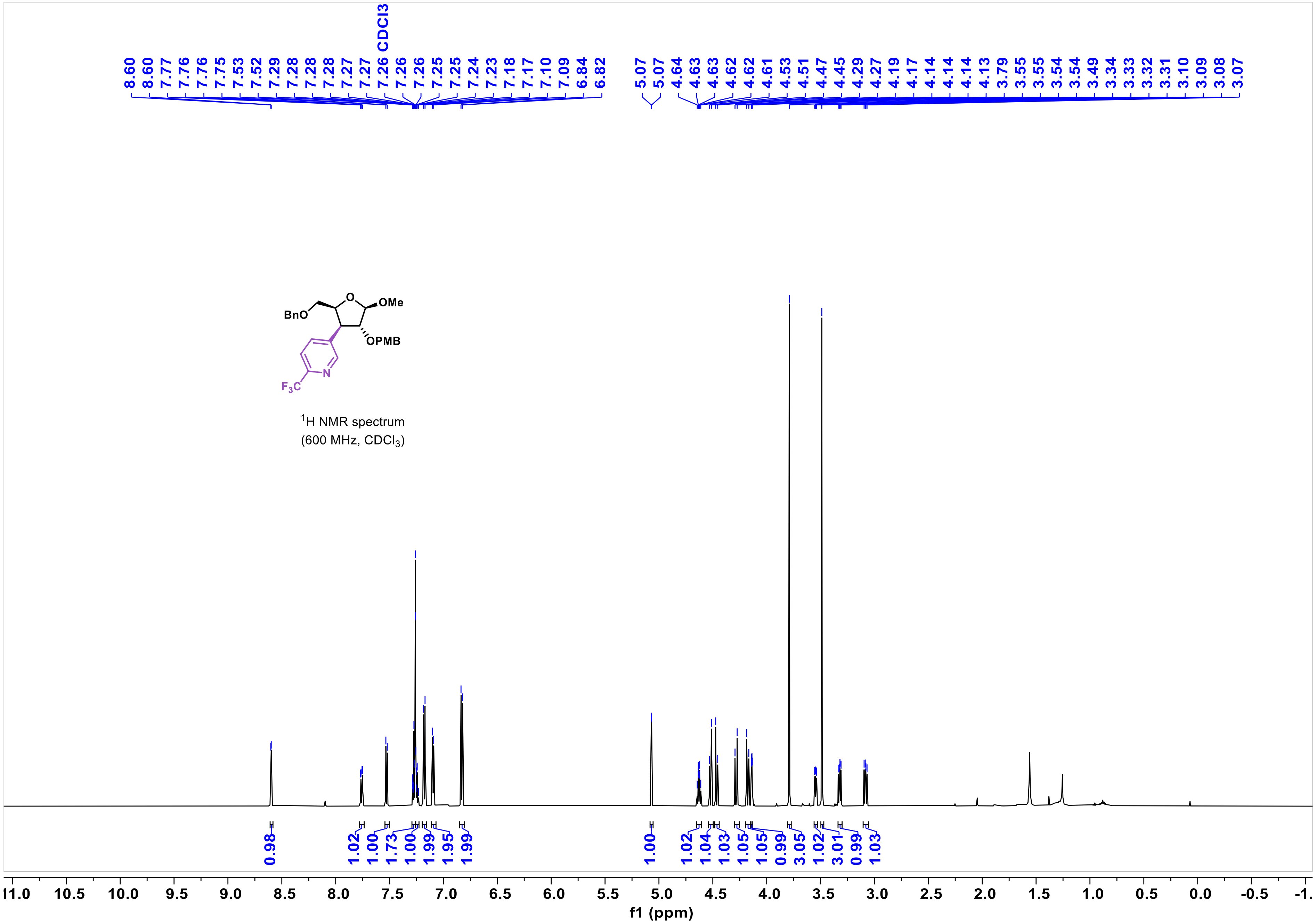

^{1}H NMR of compound **xylo-126**

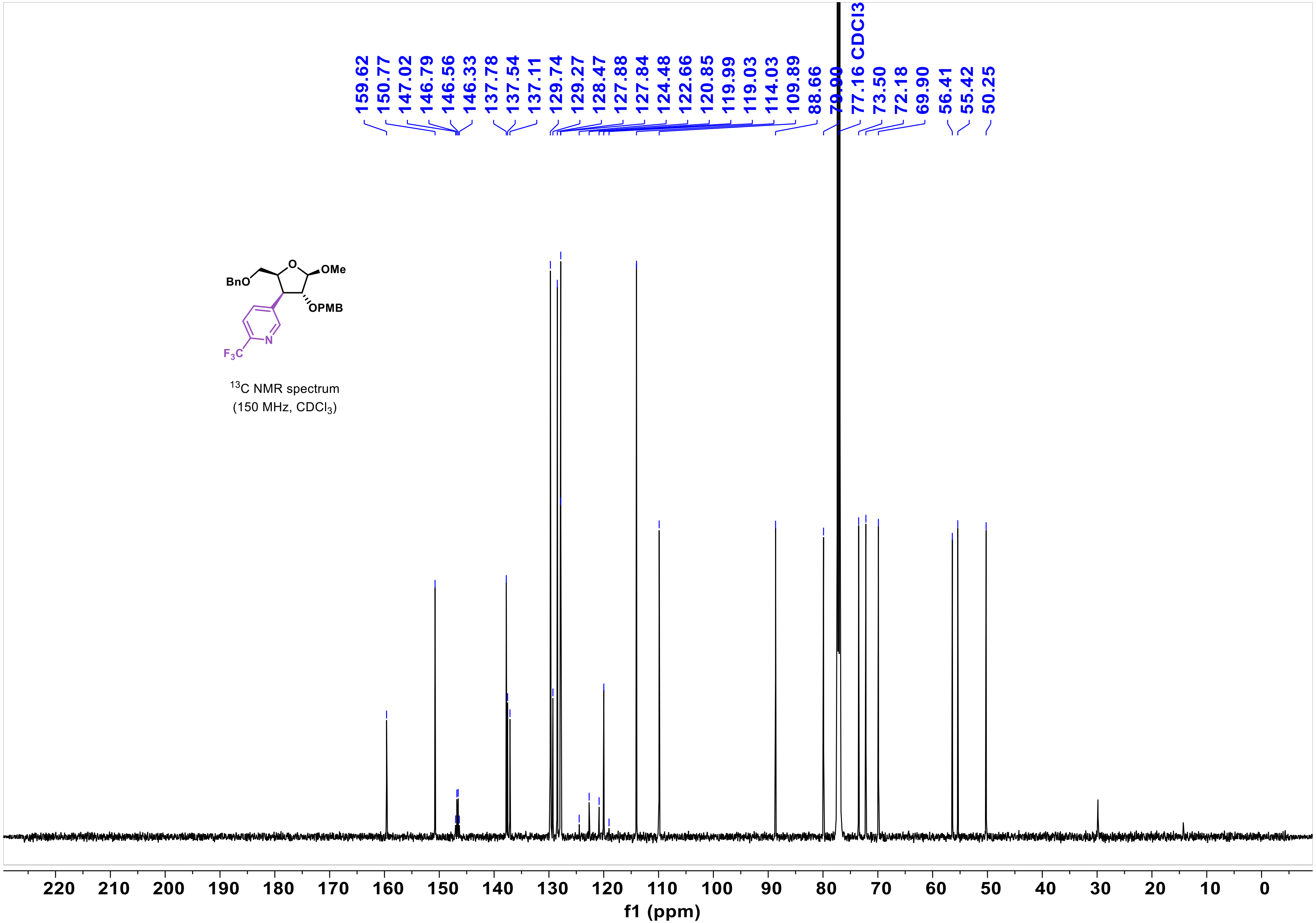

^{13}C NMR of compound **xylo-126**

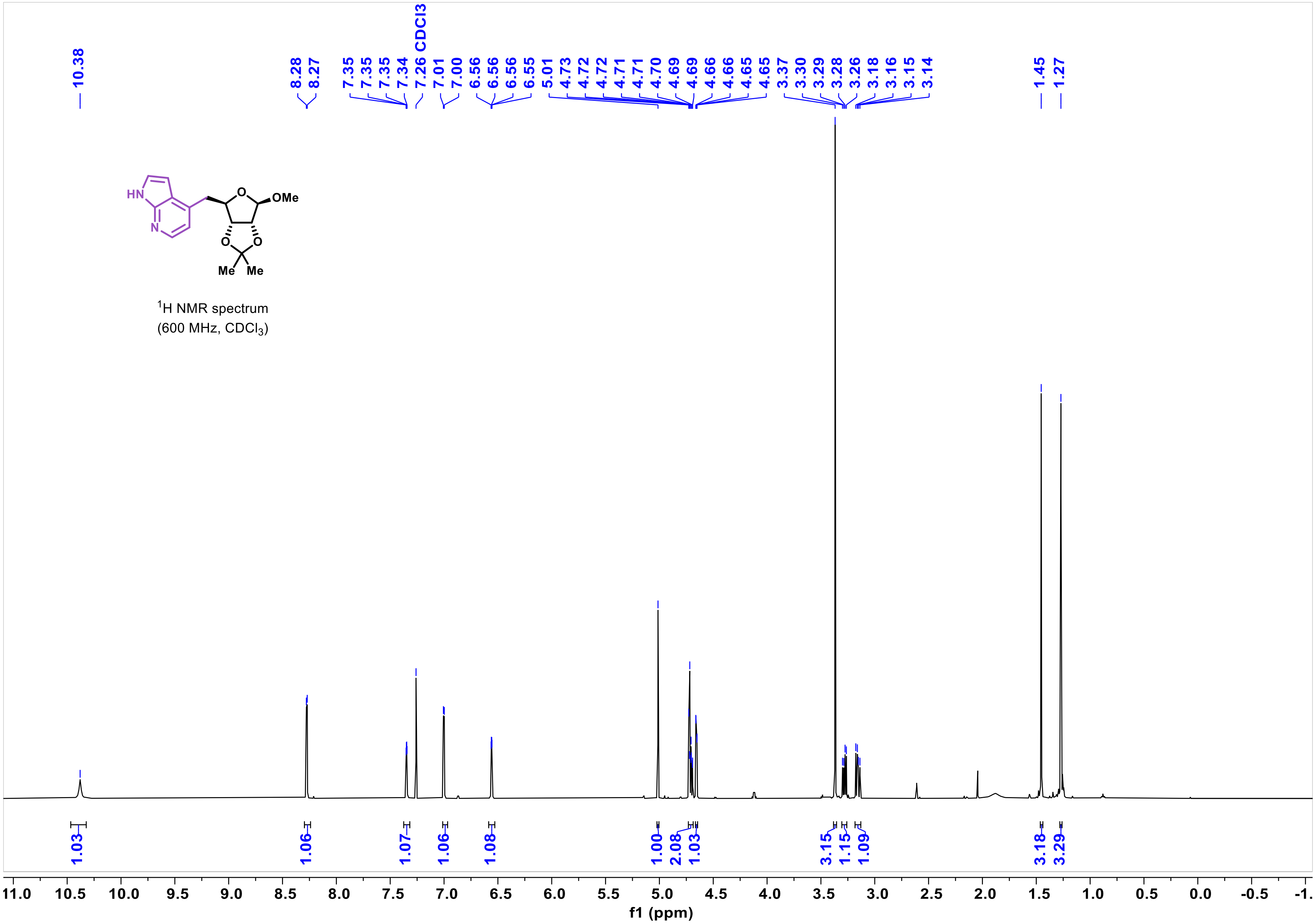

^{1}H NMR of compound **128**

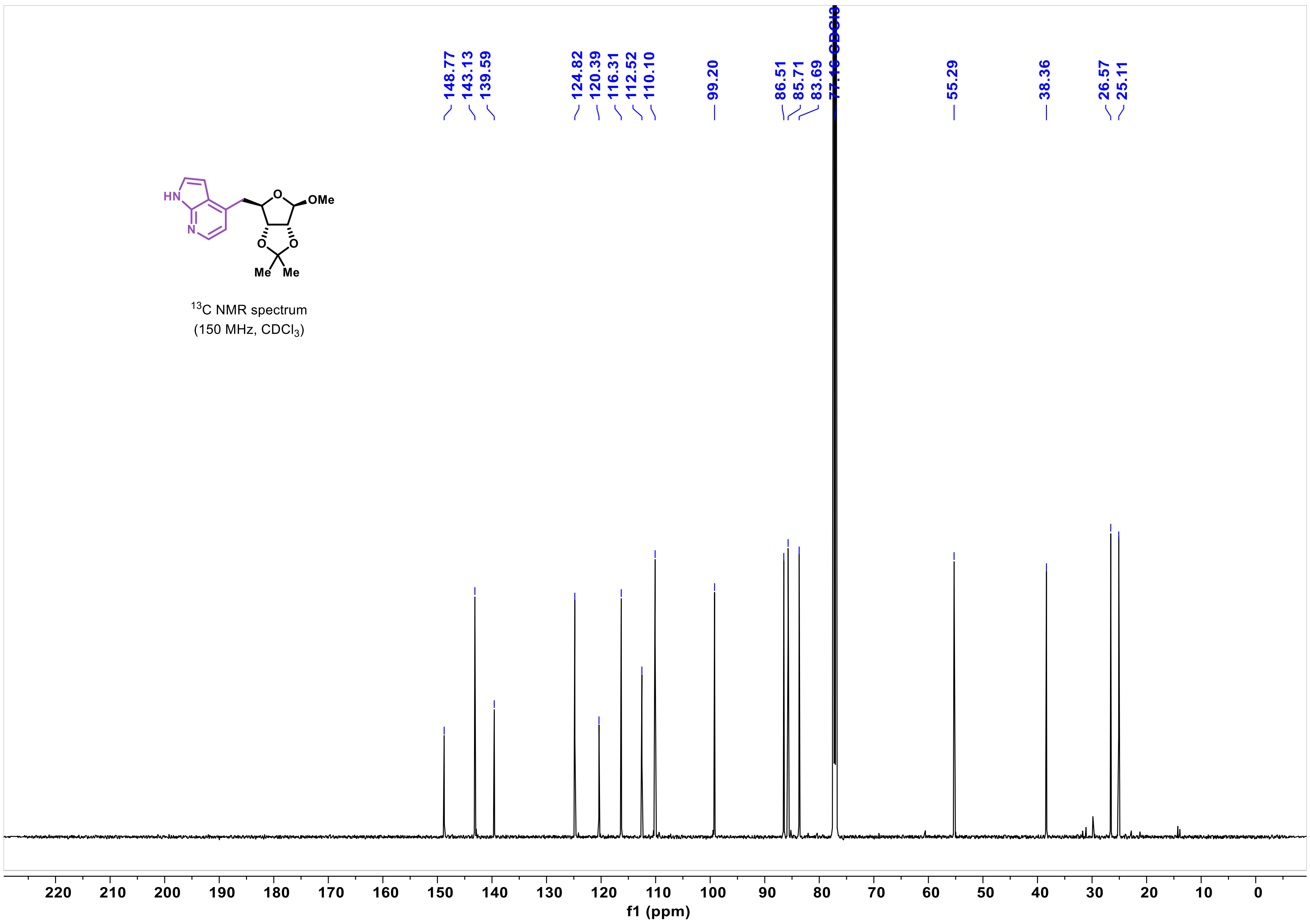

^{13}C NMR of compound **128**

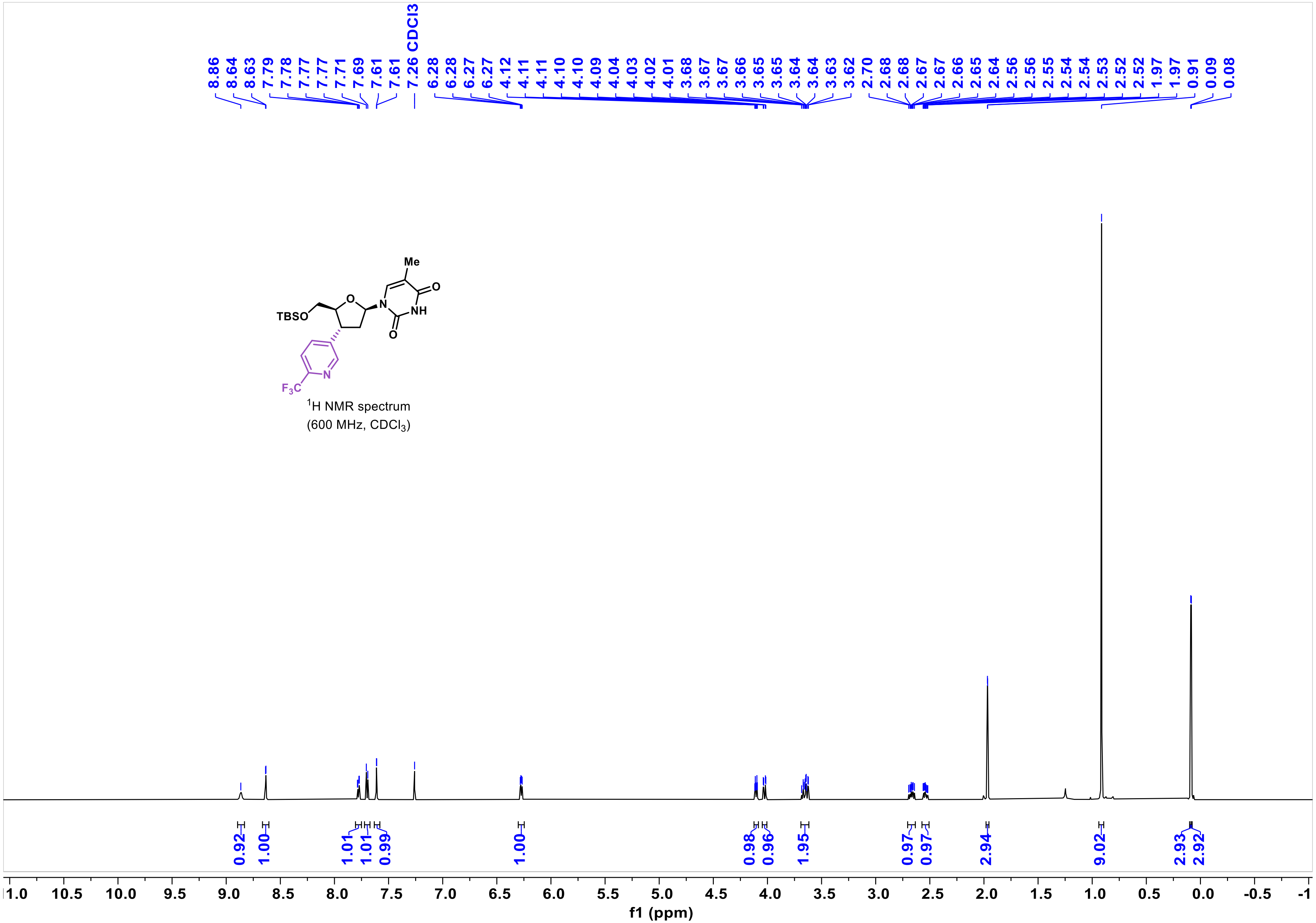

^{1}H NMR of compound **2-deoxy-ribo-130**

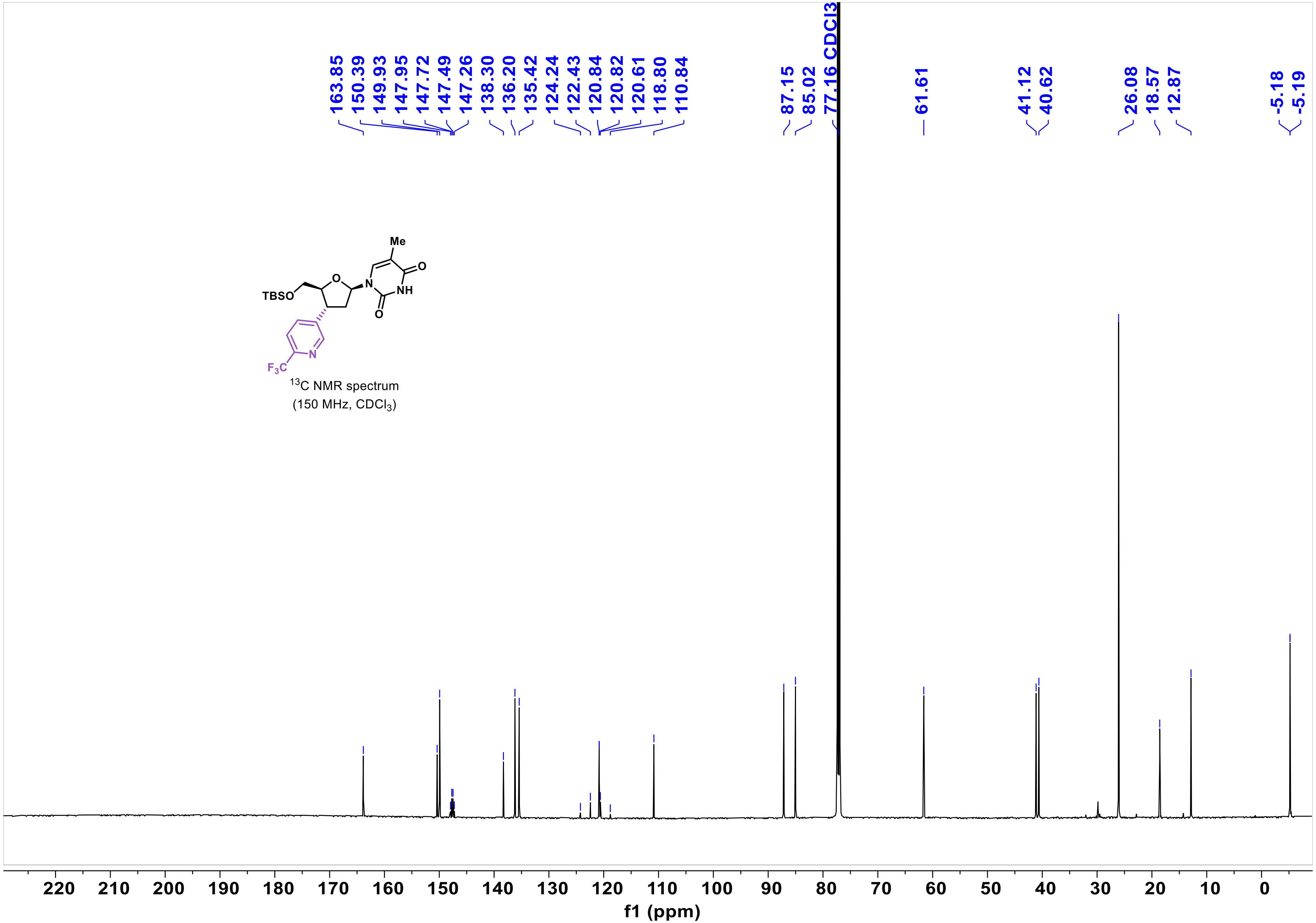

^{13}C NMR of compound **2-deoxy-ribo-130**

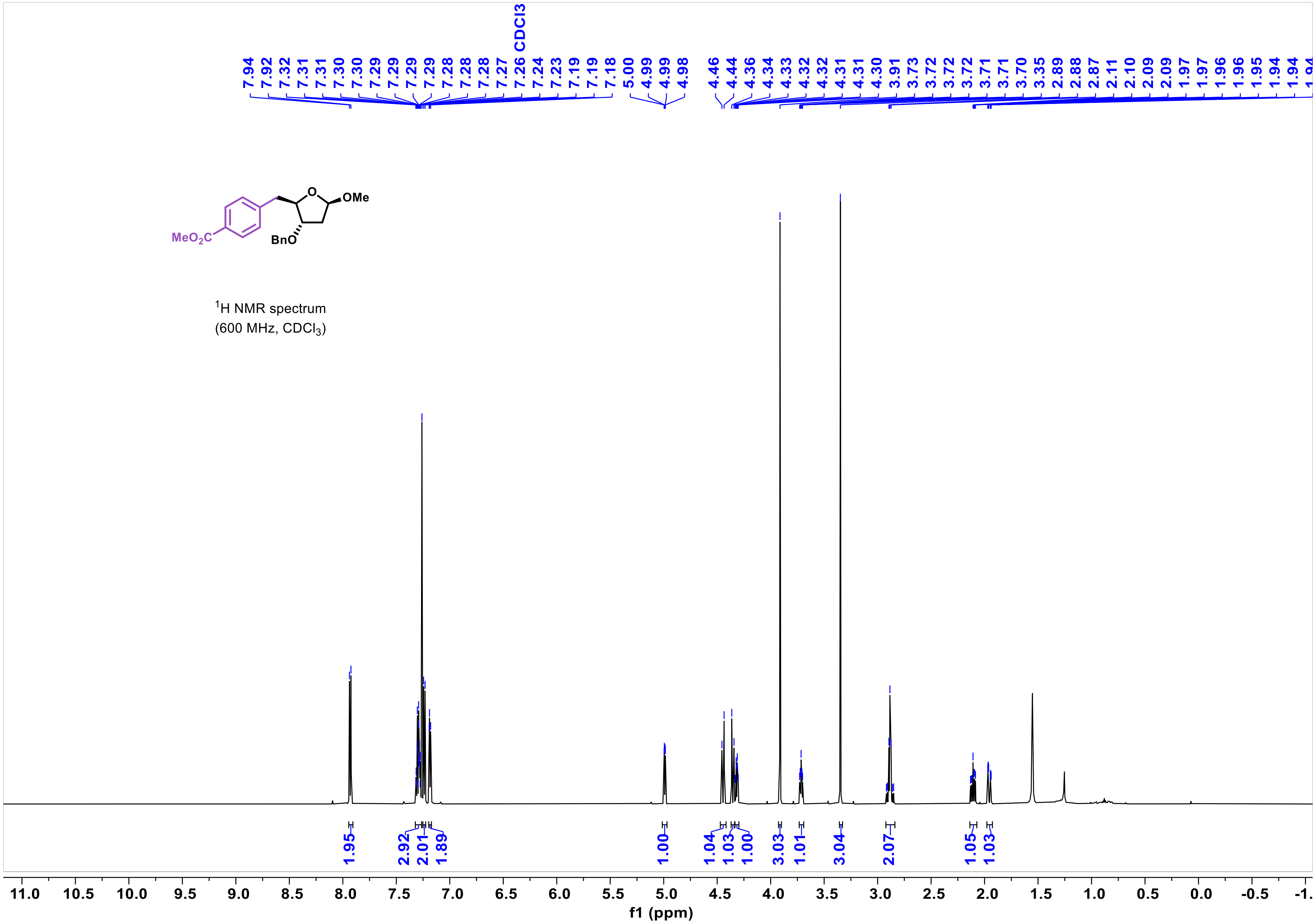

^{1}H NMR of compound **132**

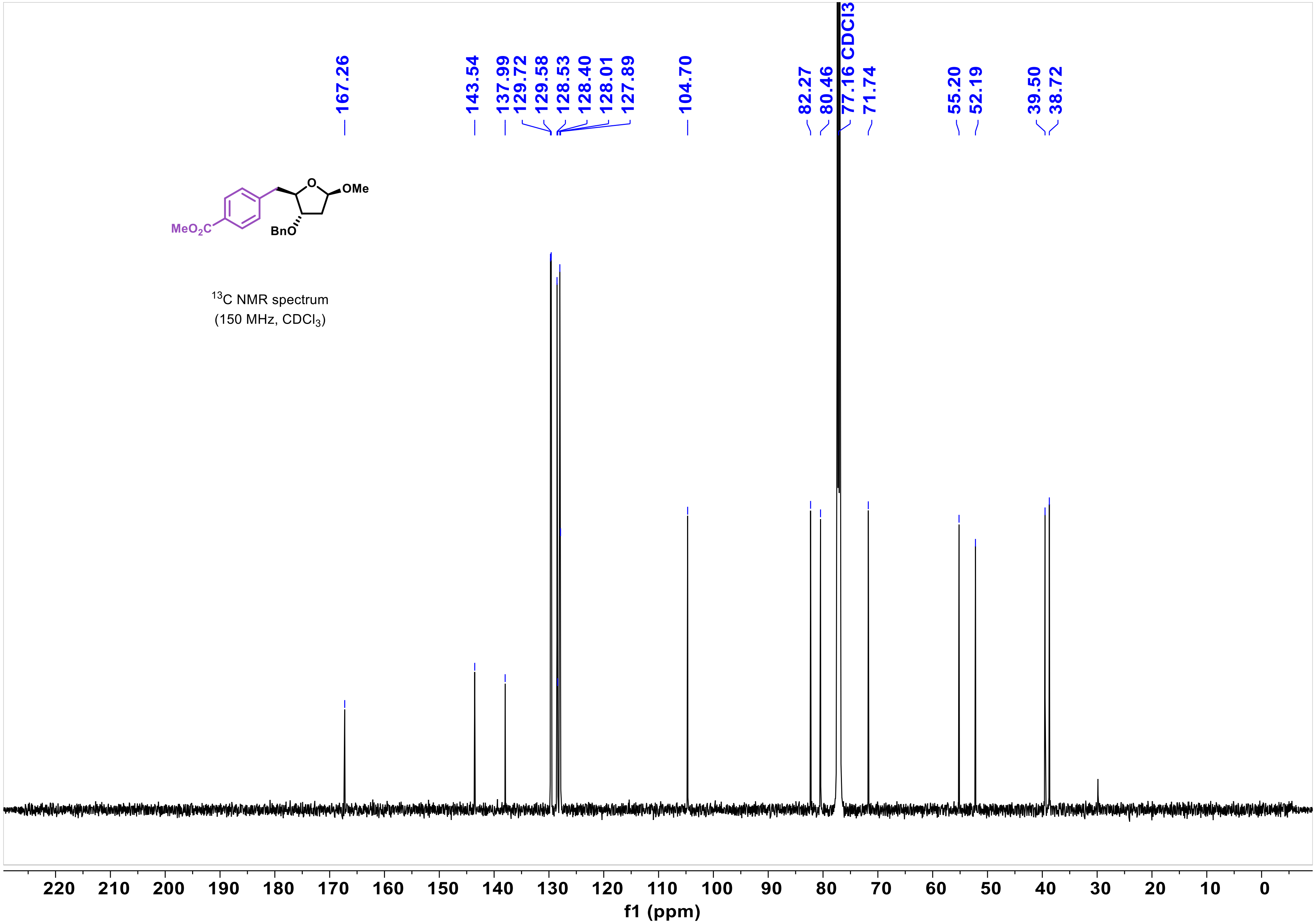

^{13}C NMR of compound **132**

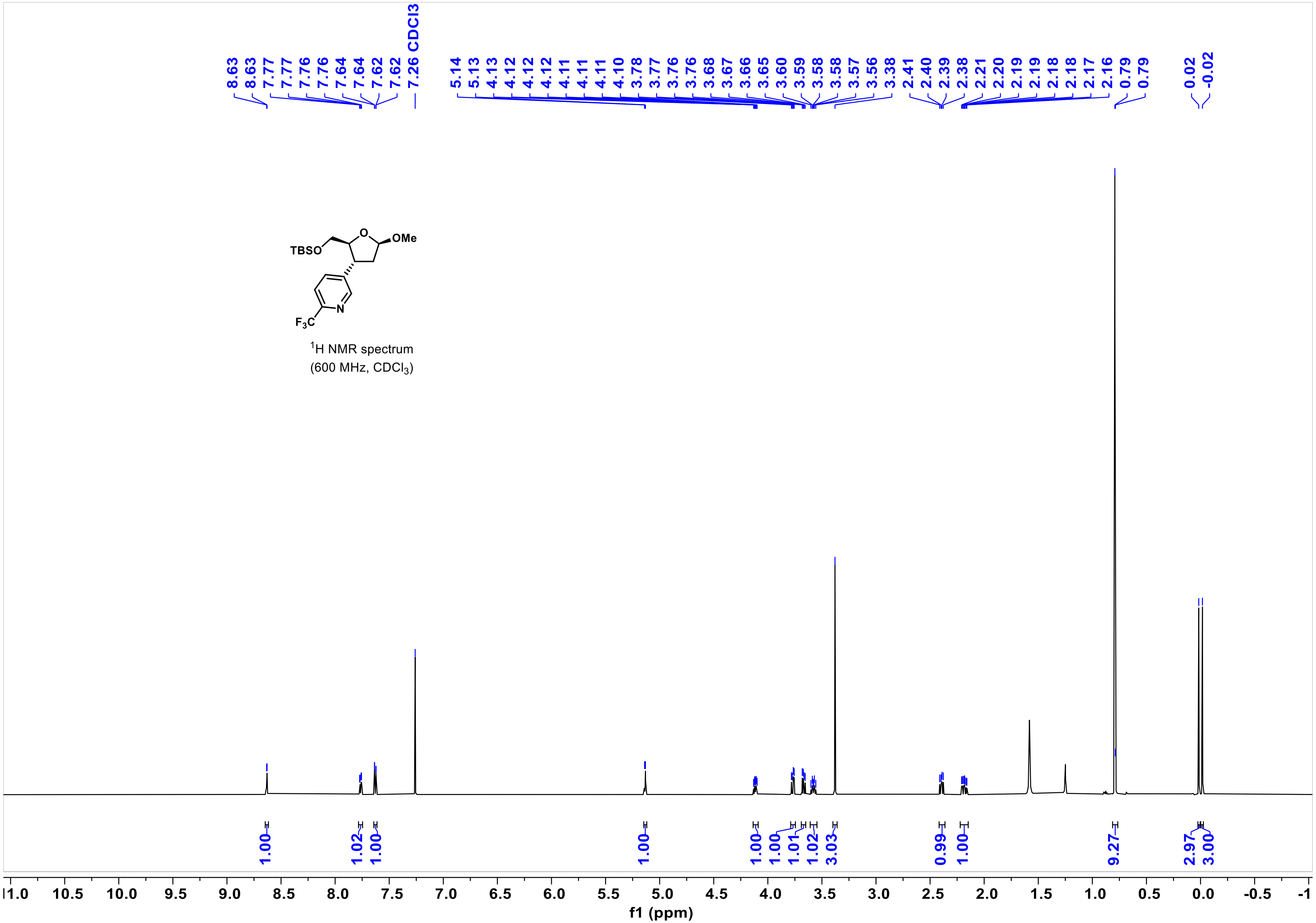

^{1}H NMR of compound **2-deoxy-ribo-SC8**

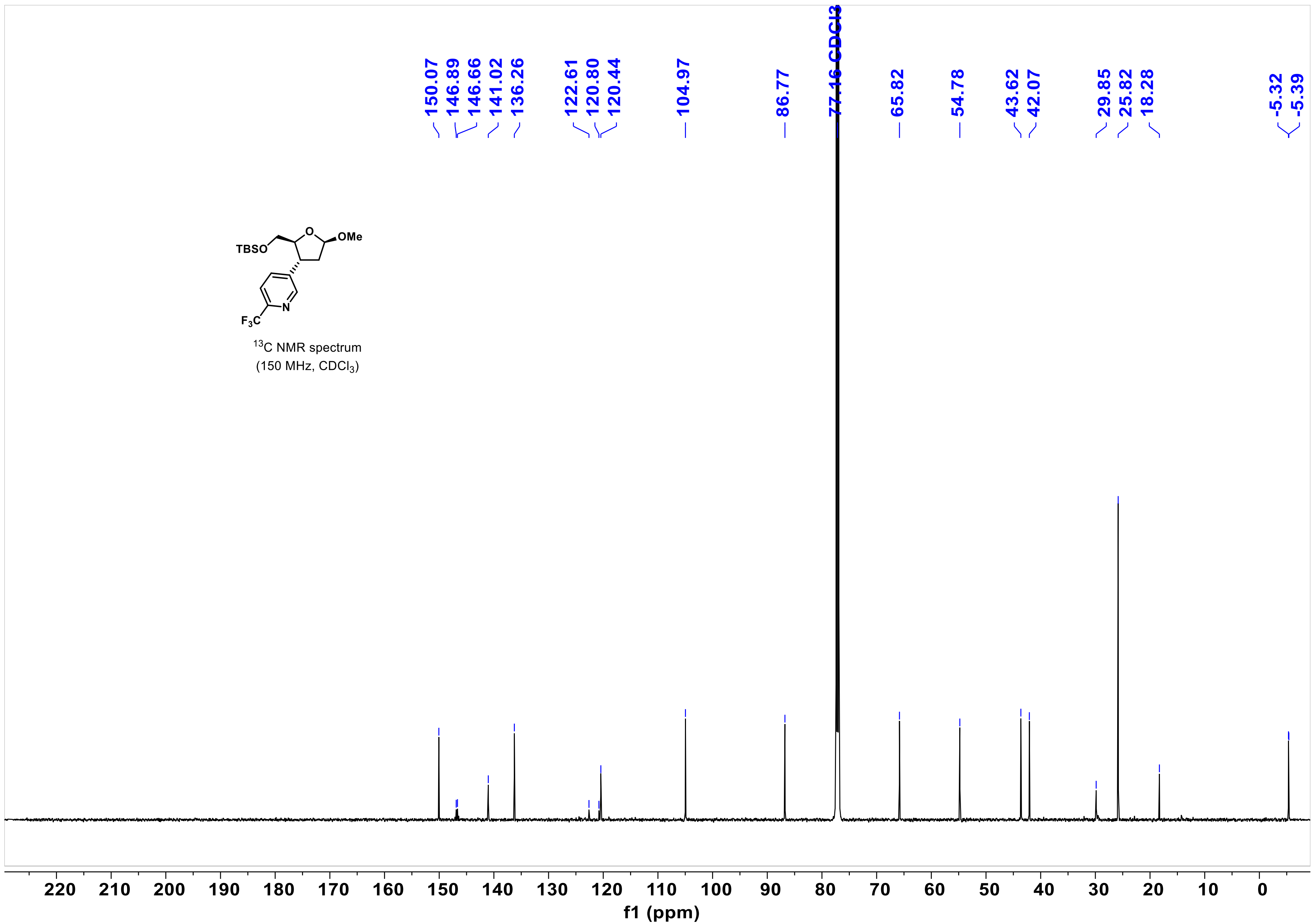

^{13}C NMR of compound **2-deoxy-ribo-SC8**

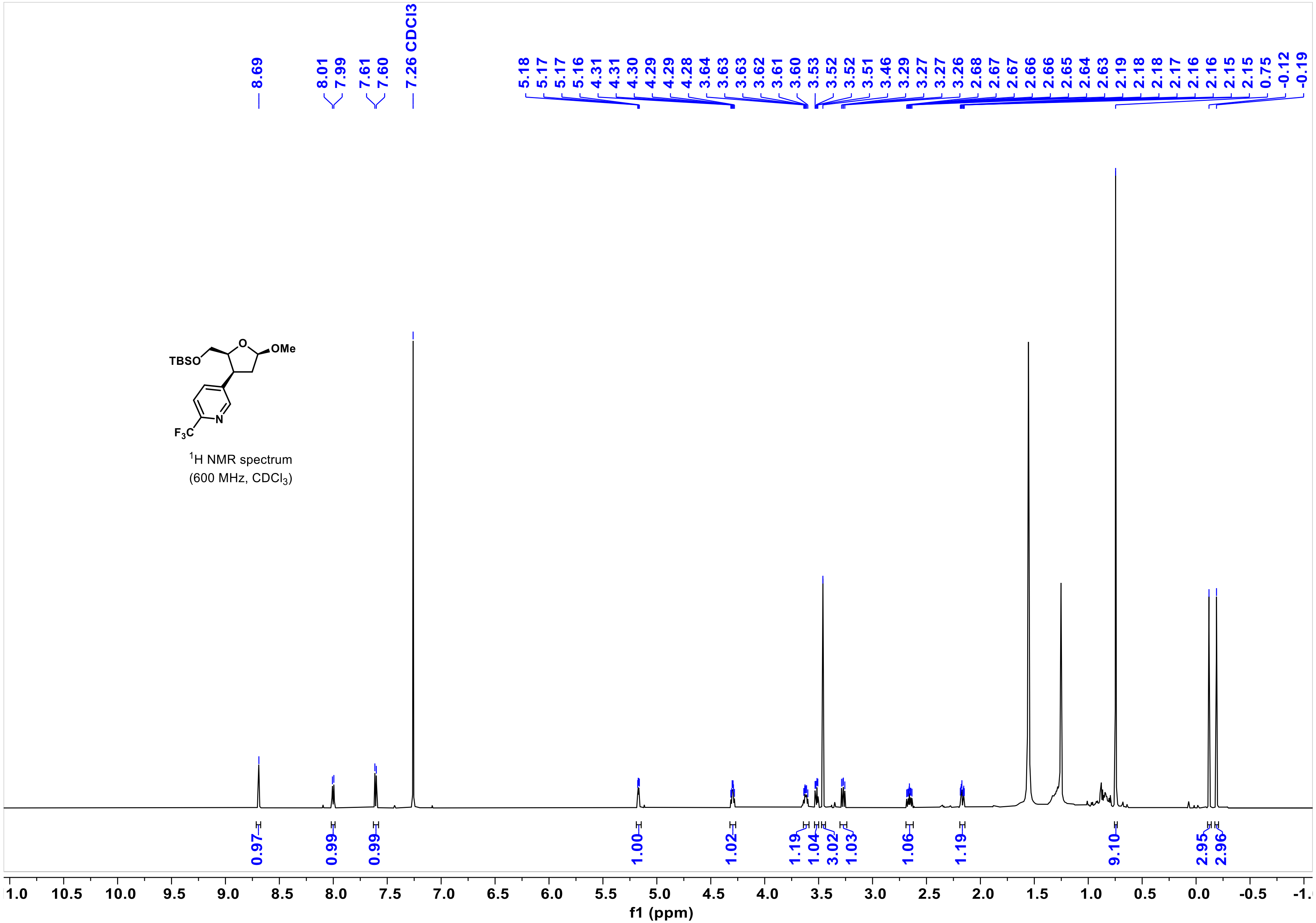

^{1}H NMR of compound **2-deoxy-xylo-SC8**

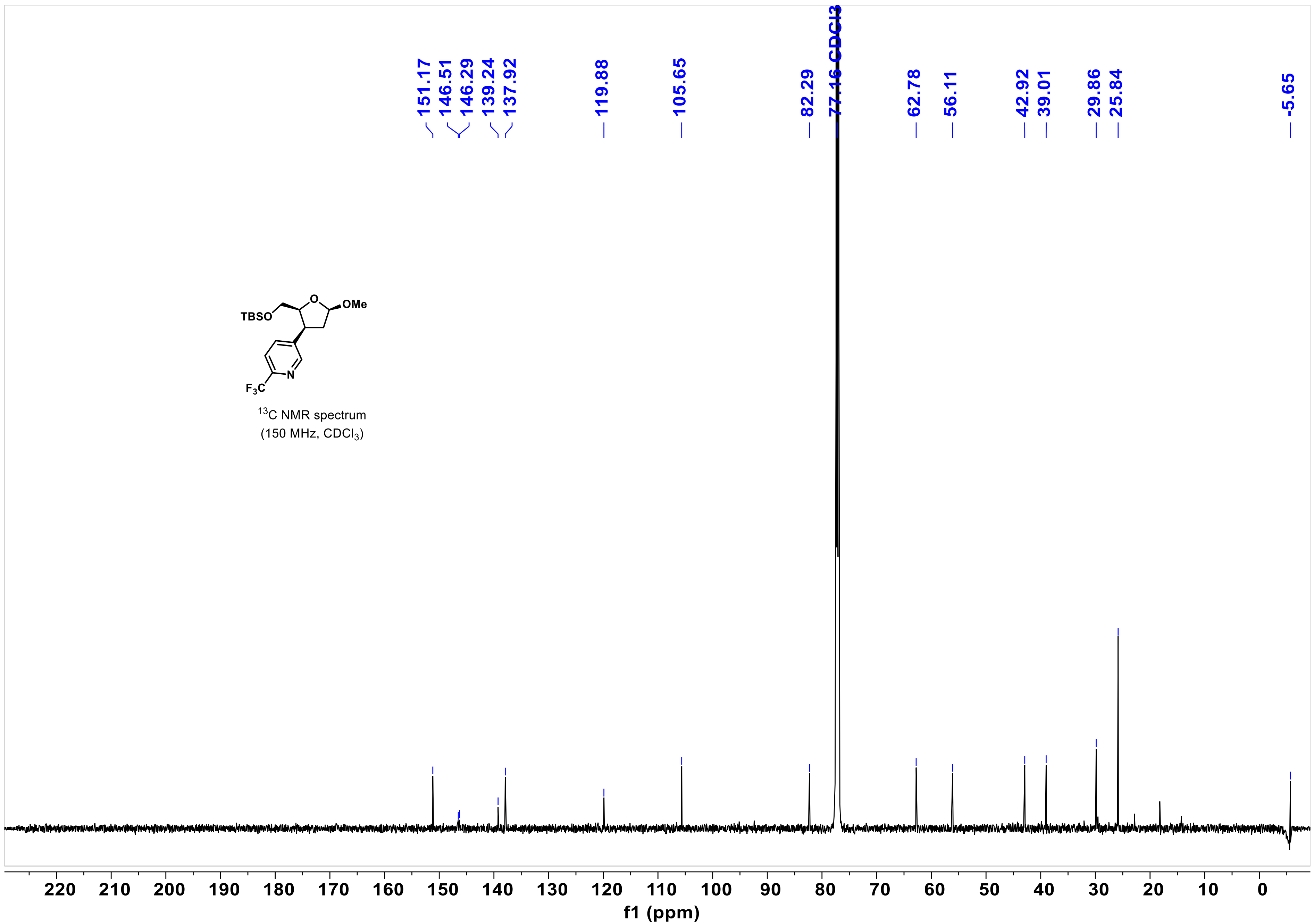

^{13}C NMR of compound **2-deoxy-xylo-SC8**

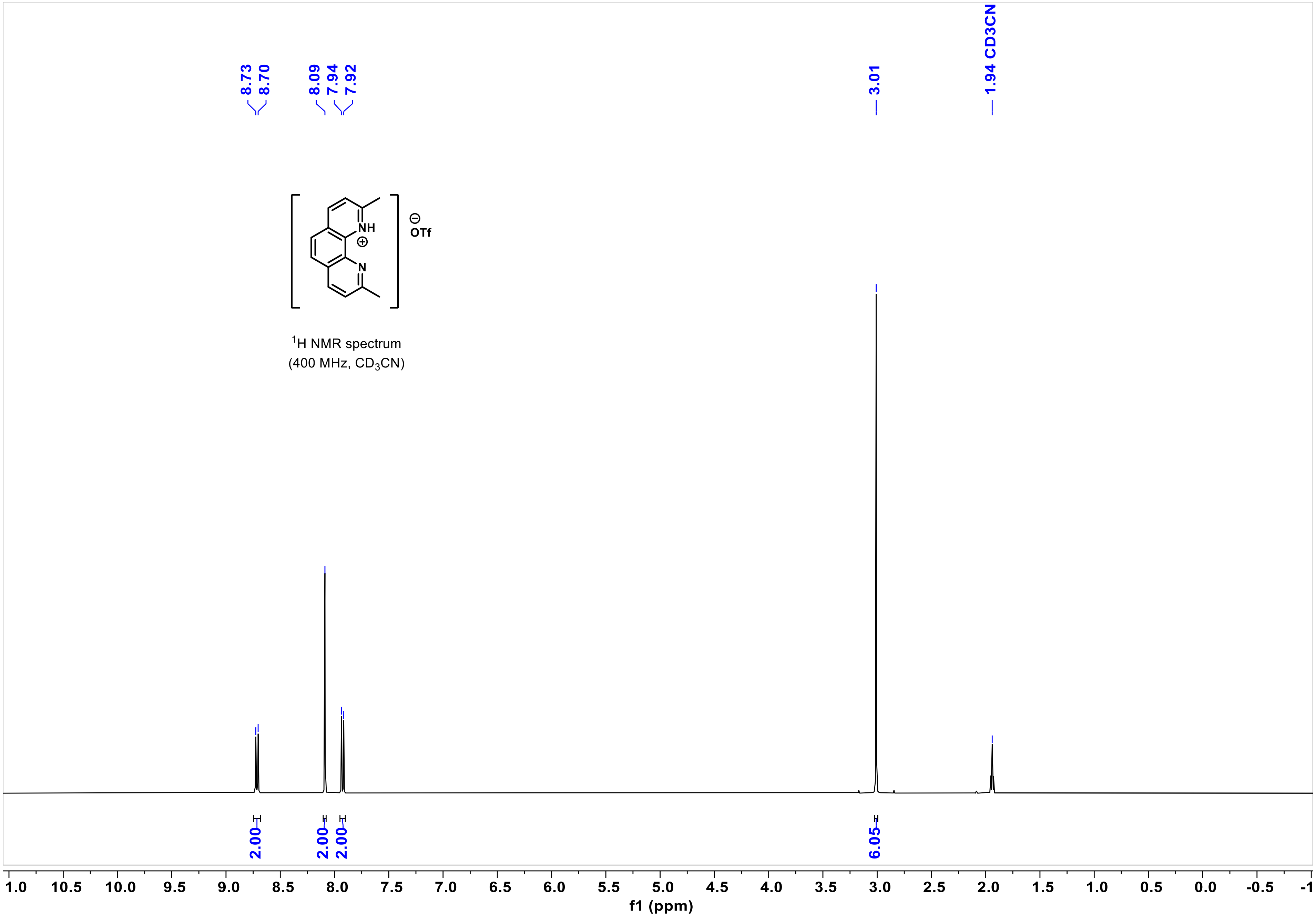

8.73
8.70
8.09
7.94
7.92
3.01
1.94 CD3CN
NH
N
OTf
^{1}H NMR spectrum
(400 MHz, CD_3CN)
2.00
2.00
2.00
6.05
1.0
10.5
10.0
9.5
9.0
8.5
8.0
7.5
7.0
6.5
6.0
5.5
5.0
4.5
4.0
3.5
3.0
2.5
2.0
1.5
1.0
0.5
0.0
-0.5
-1
f1 (ppm)

^{1}H NMR of **SL6**

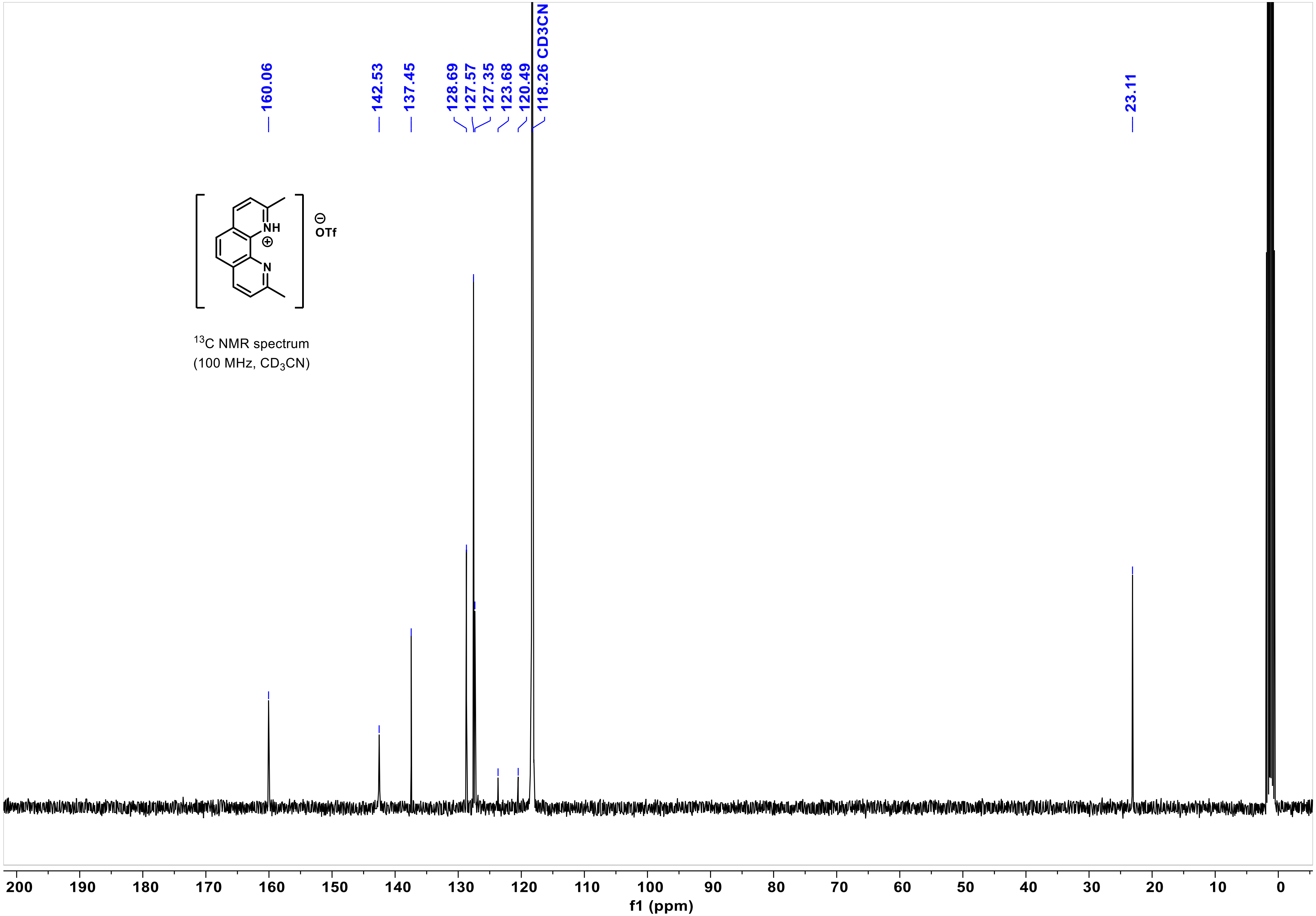

^{13}C NMR of **SL6**

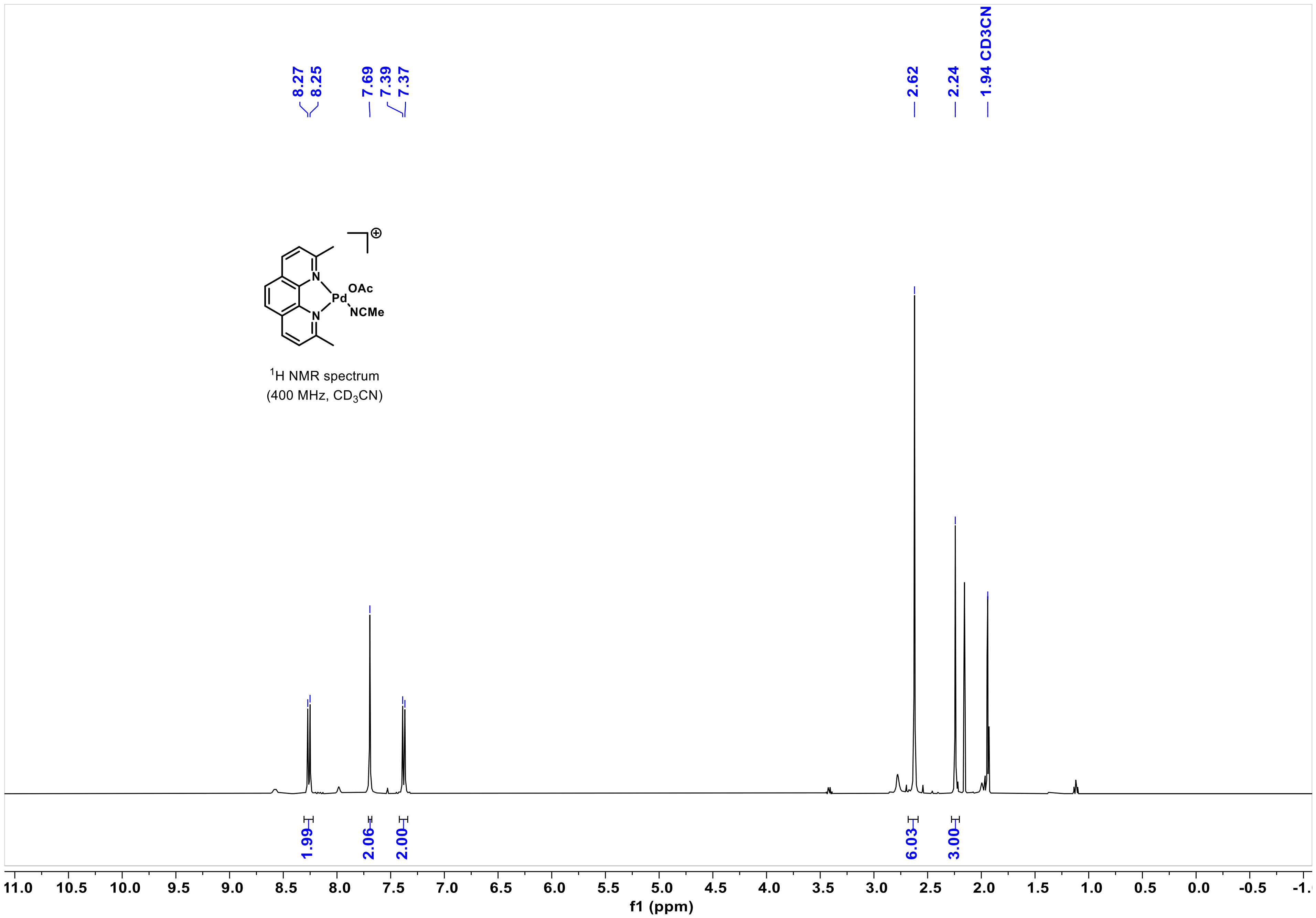

8.27
8.25
7.69
7.39
7.37
2.62
2.24
1.94 CD3CN
OAc
Pd
NCMe
N
N
1H NMR spectrum
(400 MHz, CD3CN)
1.99
2.06
2.00
6.03
3.00
f1 (ppm)

^{1}H NMR of **SL1**

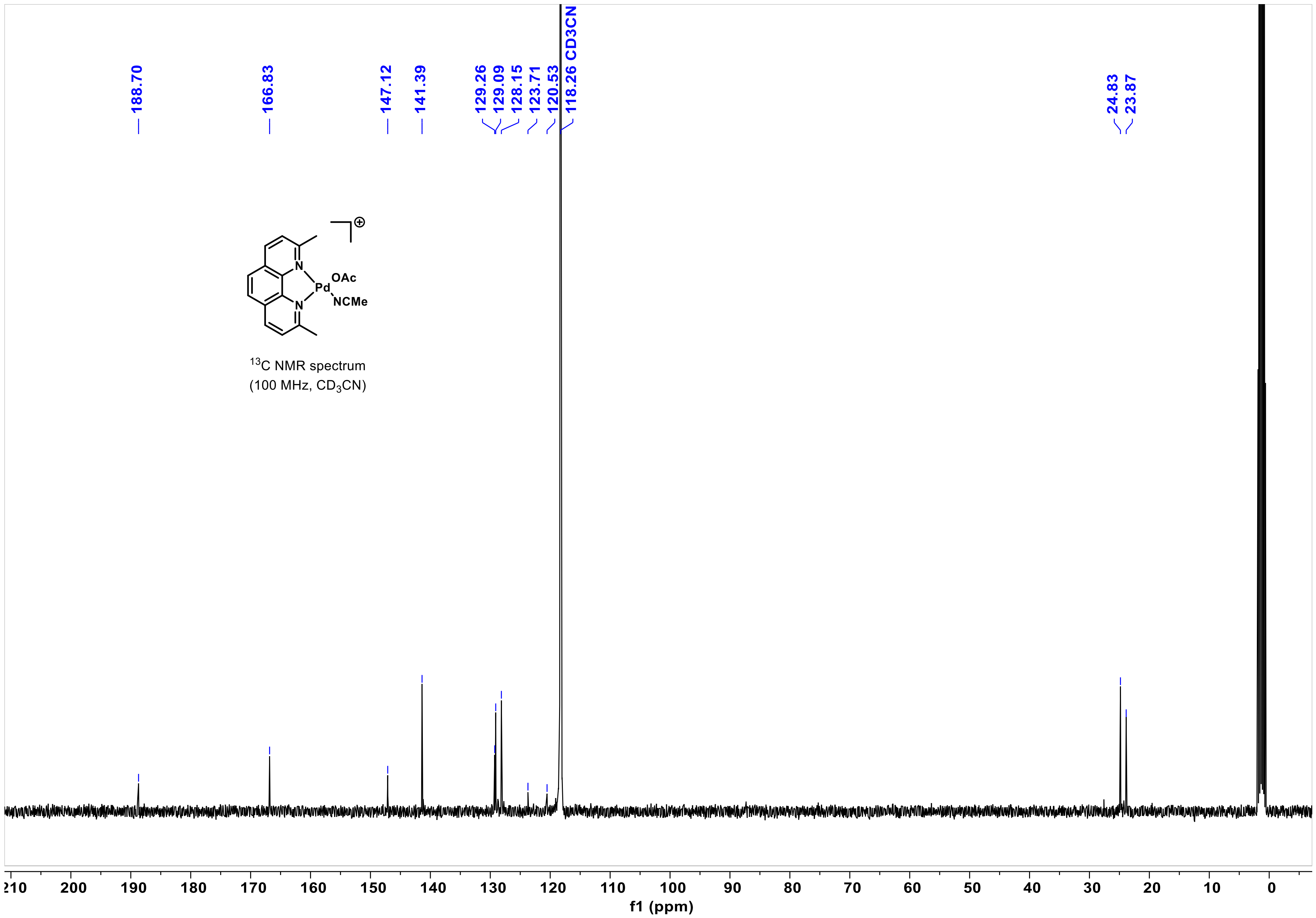

^{13}C NMR of **SL1**

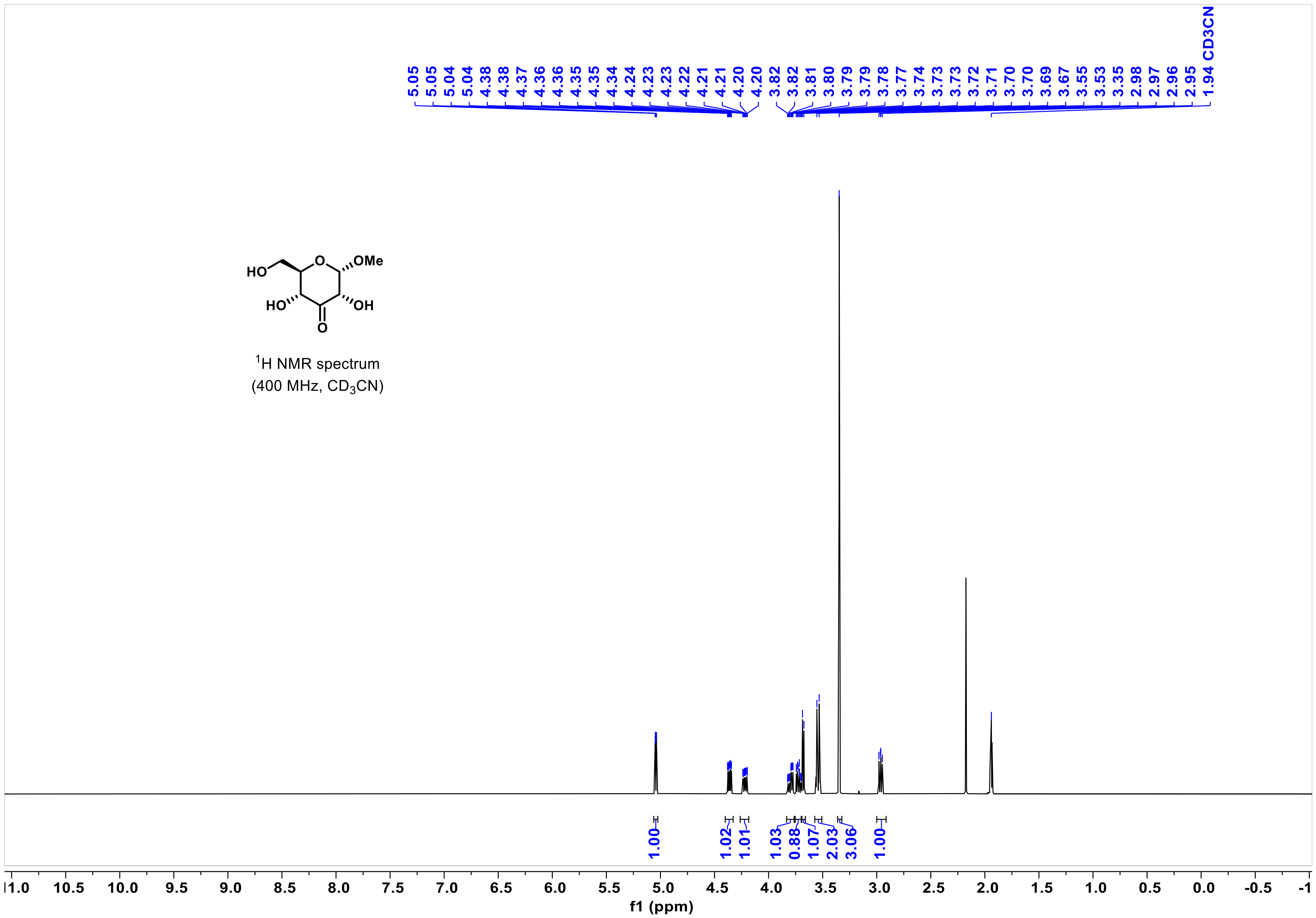
5.05 5.05 5.04 5.04 4.38 4.38 4.37 4.36 4.36 4.35 4.35 4.34 4.24 4.23 4.23 4.22 4.21 4.21 4.20 4.20 3.82 3.82 3.81 3.80 3.79 3.79 3.78 3.77 3.74 3.73 3.73 3.72 3.71 3.70 3.70 3.69 3.67 3.55 3.53 3.35 2.98 2.97 2.96 2.95 1.94 CD3CN
HO
O
OMe
HO
OH
O
^{1}H NMR spectrum
(400 MHz, CD_3CN)
1.00
1.02
1.01
1.03
0.88
1.07
2.03
3.06
1.00
11.0 10.5 10.0 9.5 9.0 8.5 8.0 7.5 7.0 6.5 6.0 5.5 5.0 4.5 4.0 3.5 3.0 2.5 2.0 1.5 1.0 0.5 0.0 -0.5 -1
f1 (ppm)

^{1}H NMR of **SL2**

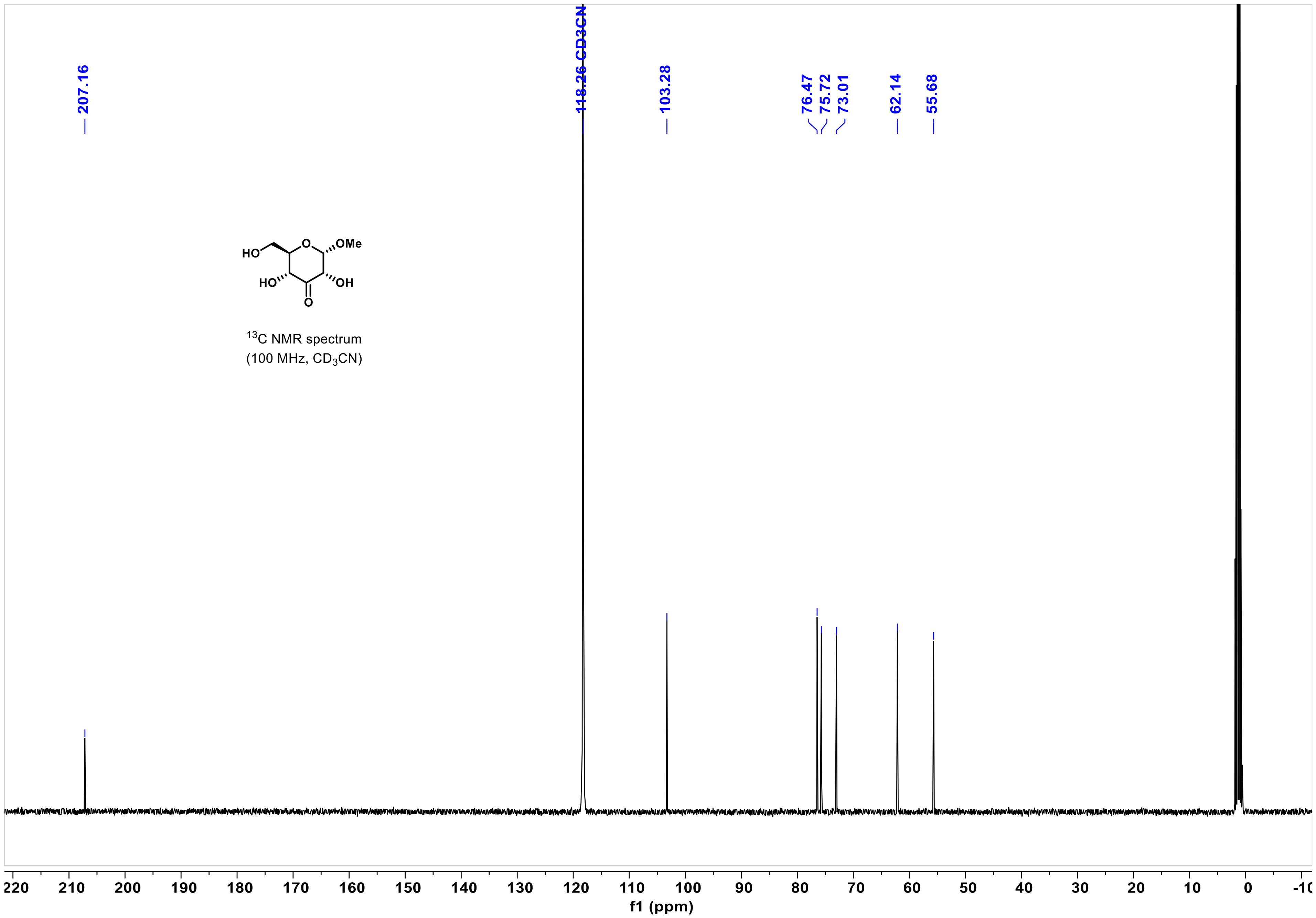

^{13}C NMR of **SL2**

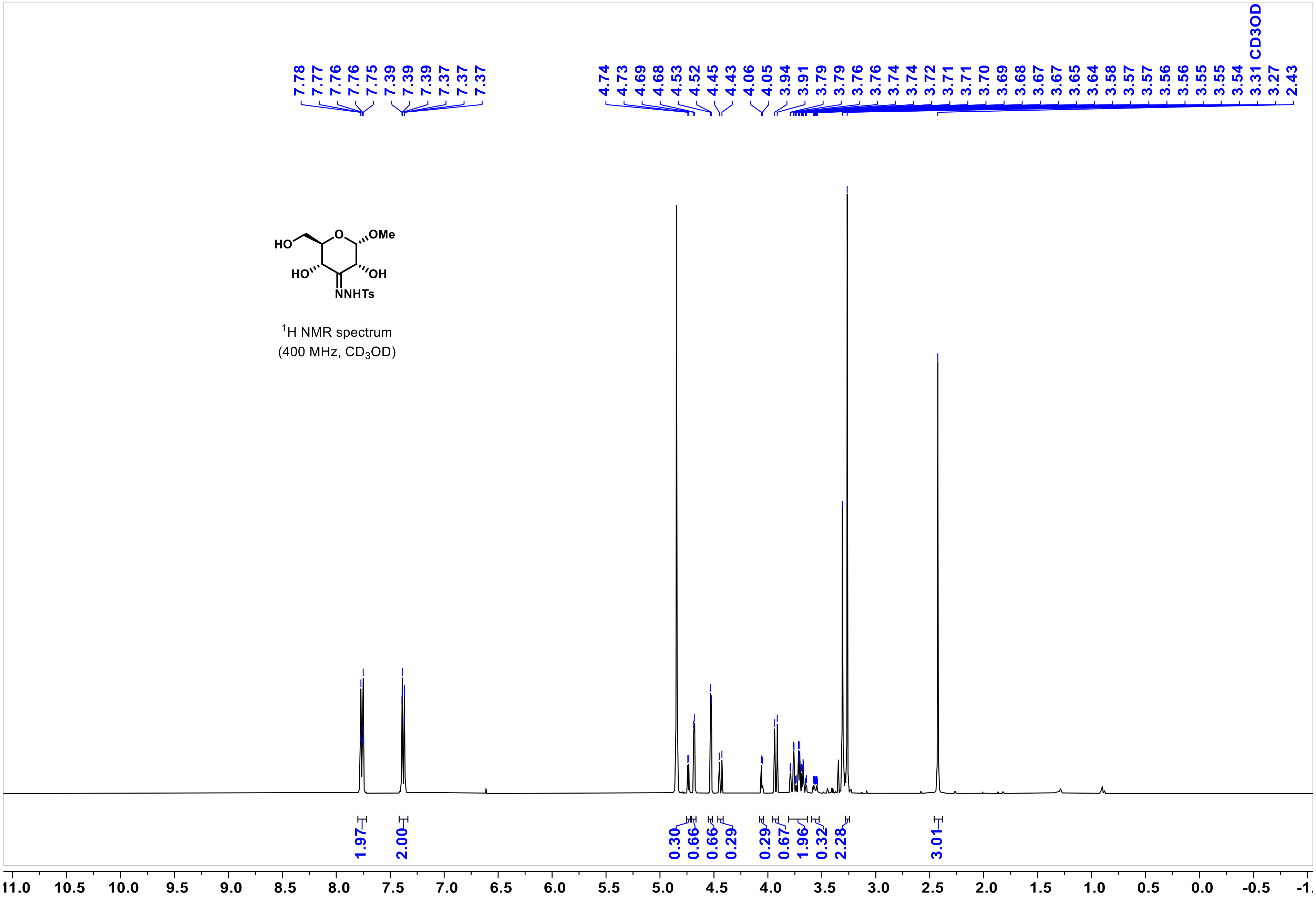

7.78
7.77
7.76
7.76
7.75
7.39
7.39
7.39
7.37
7.37
7.37
4.74
4.73
4.69
4.68
4.53
4.52
4.45
4.43
4.06
4.05
3.94
3.91
3.79
3.79
3.76
3.76
3.74
3.74
3.72
3.71
3.71
3.70
3.69
3.68
3.67
3.67
3.65
3.64
3.58
3.57
3.57
3.56
3.56
3.55
3.55
3.54
3.31 CD3OD
3.27
2.43
HO
O
OMe
HO
OH
NNHTs
1H NMR spectrum
(400 MHz, CD3OD)
1.97
2.00
0.30
0.66
0.66
0.29
0.29
0.67
1.96
0.32
2.28
3.01
11.0
10.5
10.0
9.5
9.0
8.5
8.0
7.5
7.0
6.5
6.0
5.5
5.0
4.5
4.0
3.5
3.0
2.5
2.0
1.5
1.0
0.5
0.0
-0.5
-1.
f1 (ppm)

^{1}H NMR of **SL3**

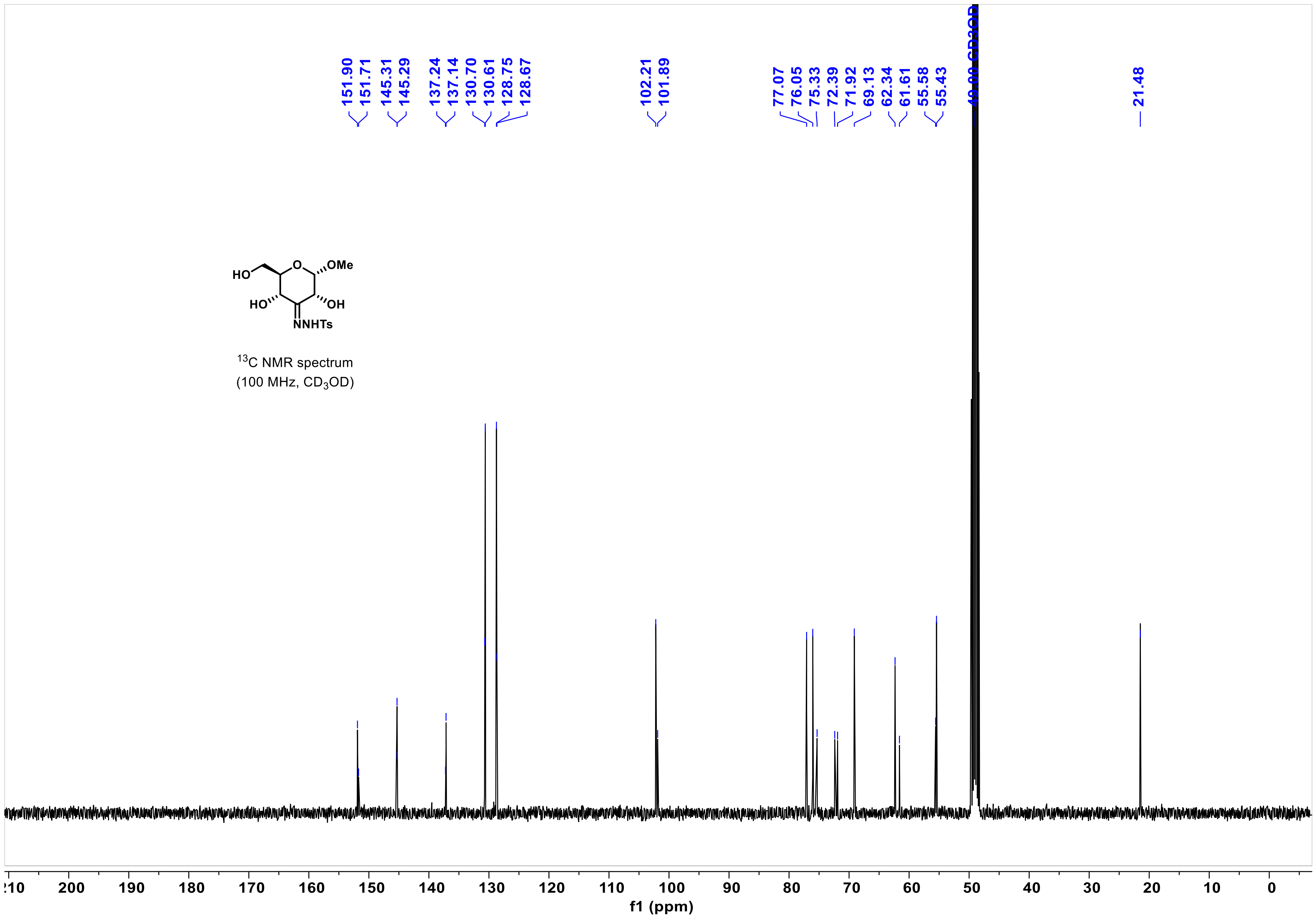

^{13}C NMR of **SL3**

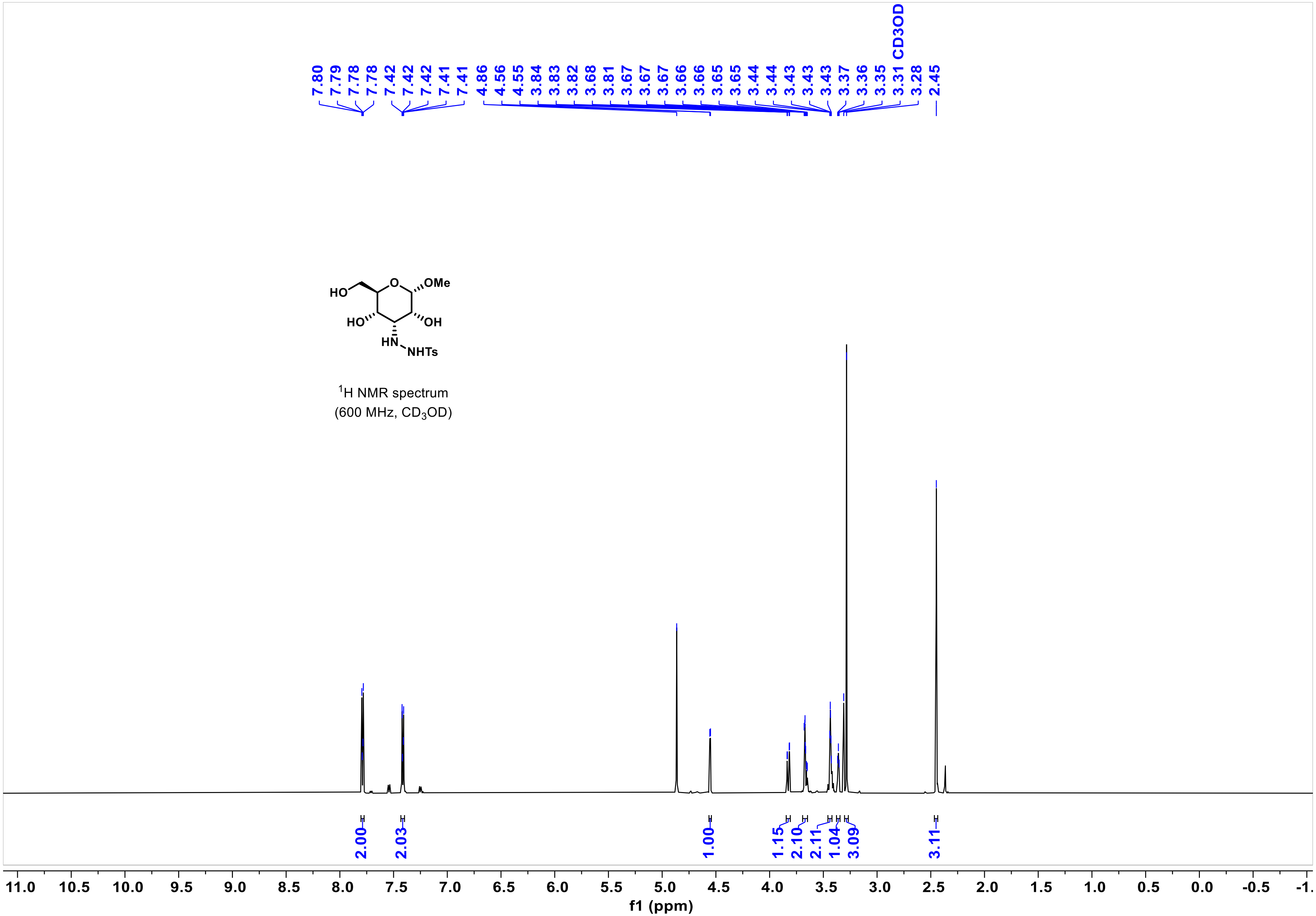

7.80 7.79 7.78 7.78 7.42 7.42 7.42 7.41 7.41 4.86 4.56 4.55 3.84 3.83 3.82 3.68 3.81 3.67 3.67 3.67 3.66 3.66 3.65 3.65 3.44 3.44 3.43 3.43 3.43 3.37 3.36 3.35 3.31 3.28 2.45
CD3OD
HO
O
OMe
HO
OH
HN
NHTs
^{1}H NMR spectrum
(600 MHz, CD_3OD)
2.00 2.03 1.00 1.15 2.10 2.11 1.04 3.09 3.11
f1 (ppm)

^{1}H NMR of **134**

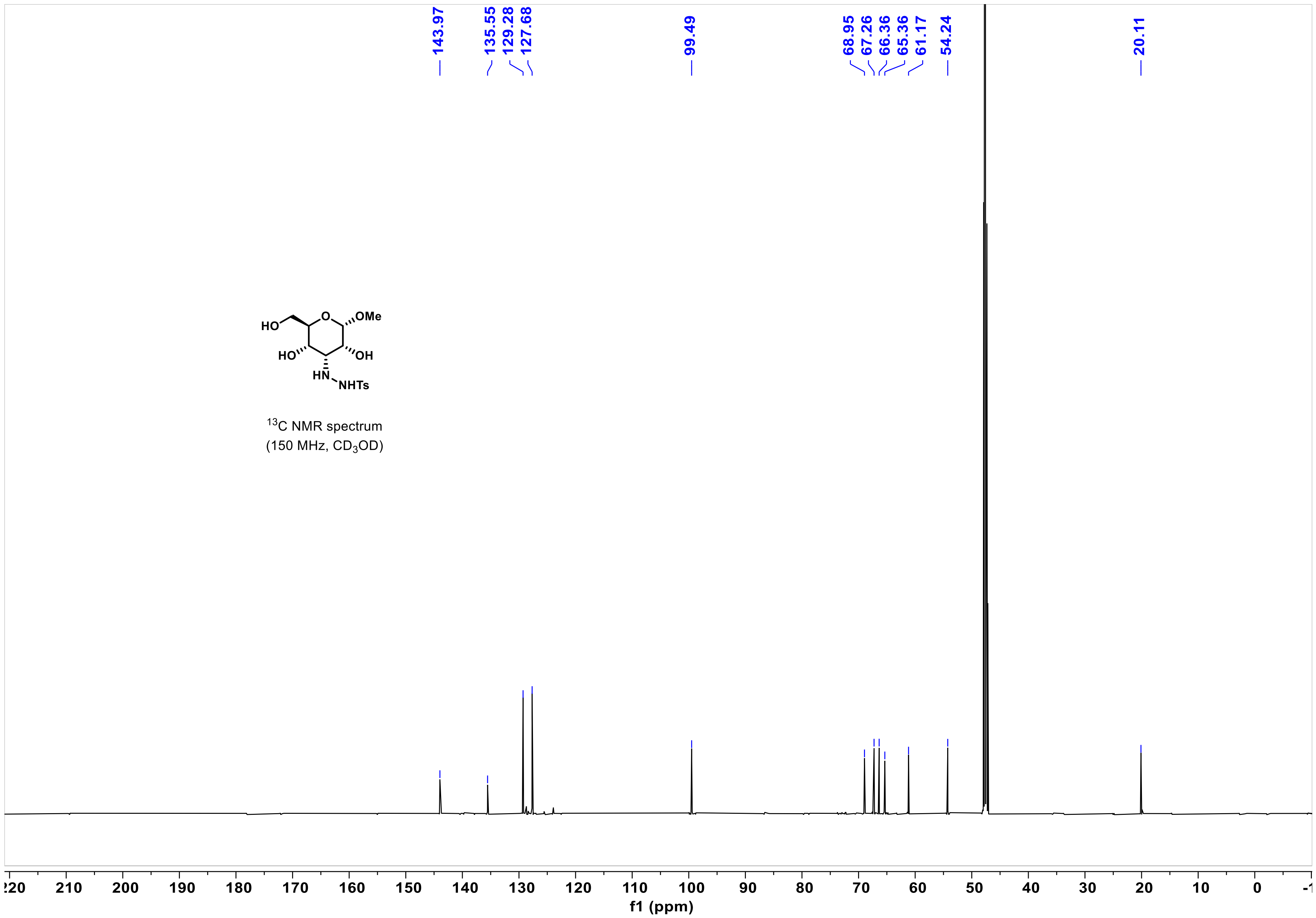

^{13}C NMR of **134**

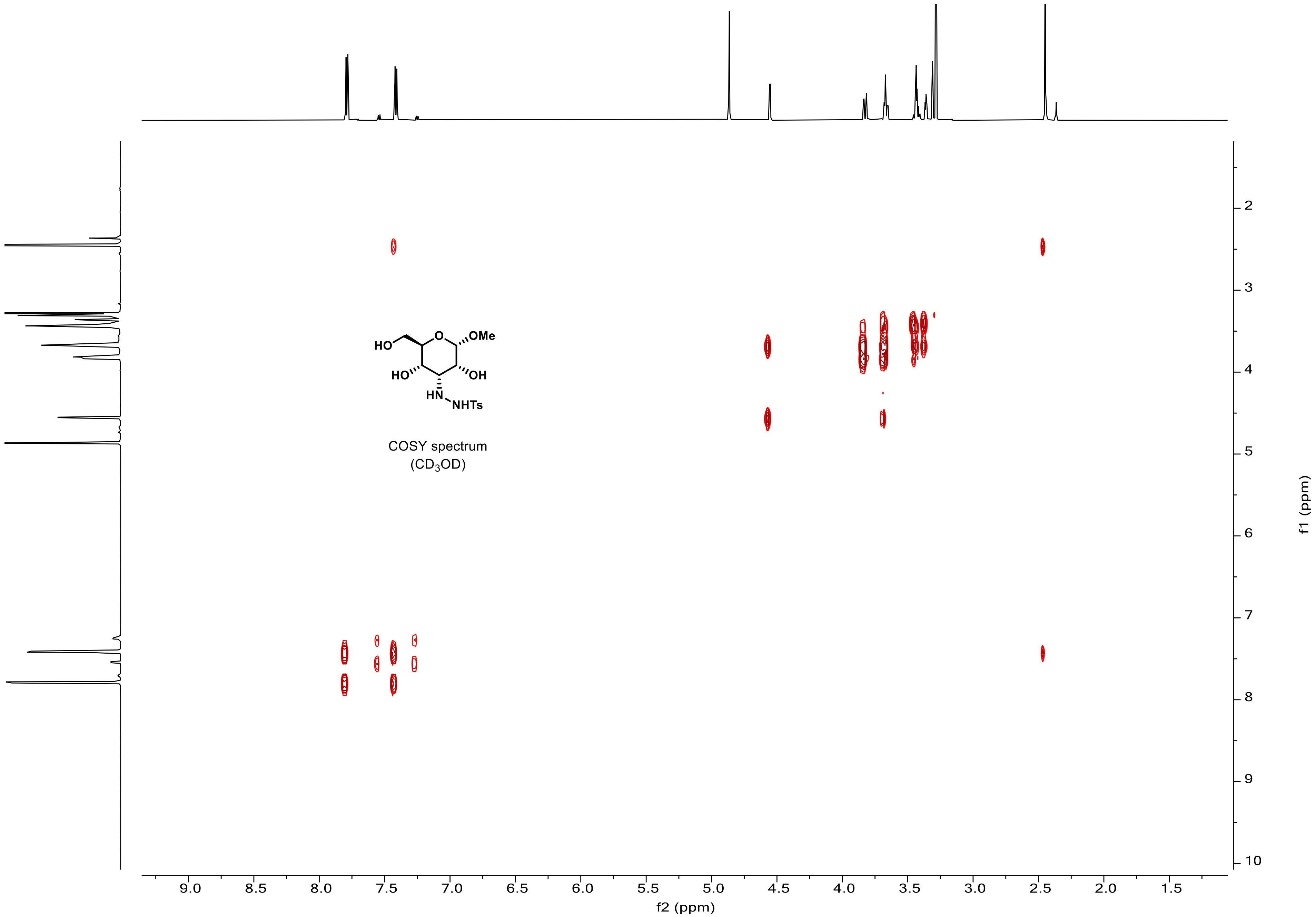

H-H COSY NMR Spectrum of compound **134**

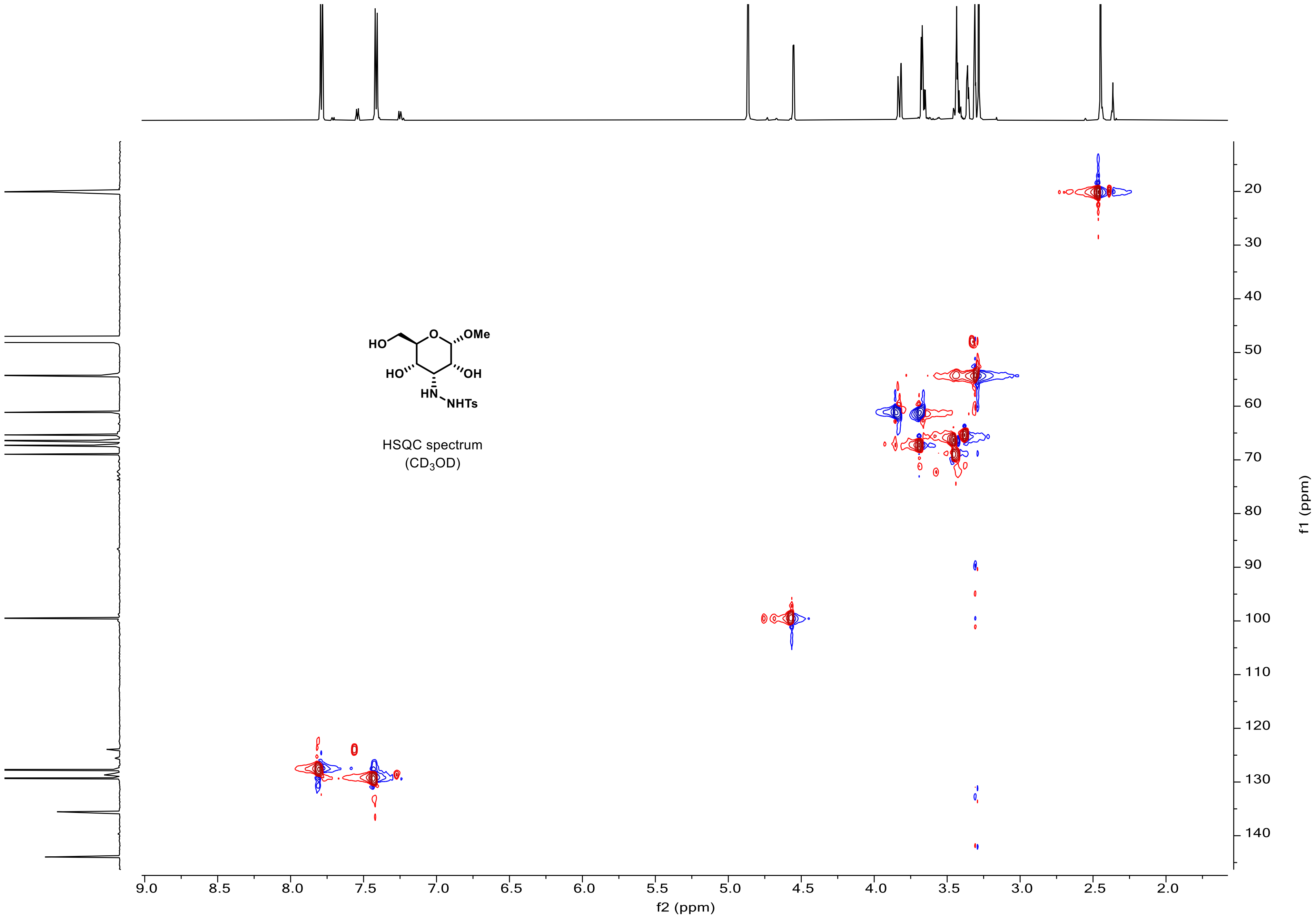

H-C HSQC NMR Spectrum of compound **134**

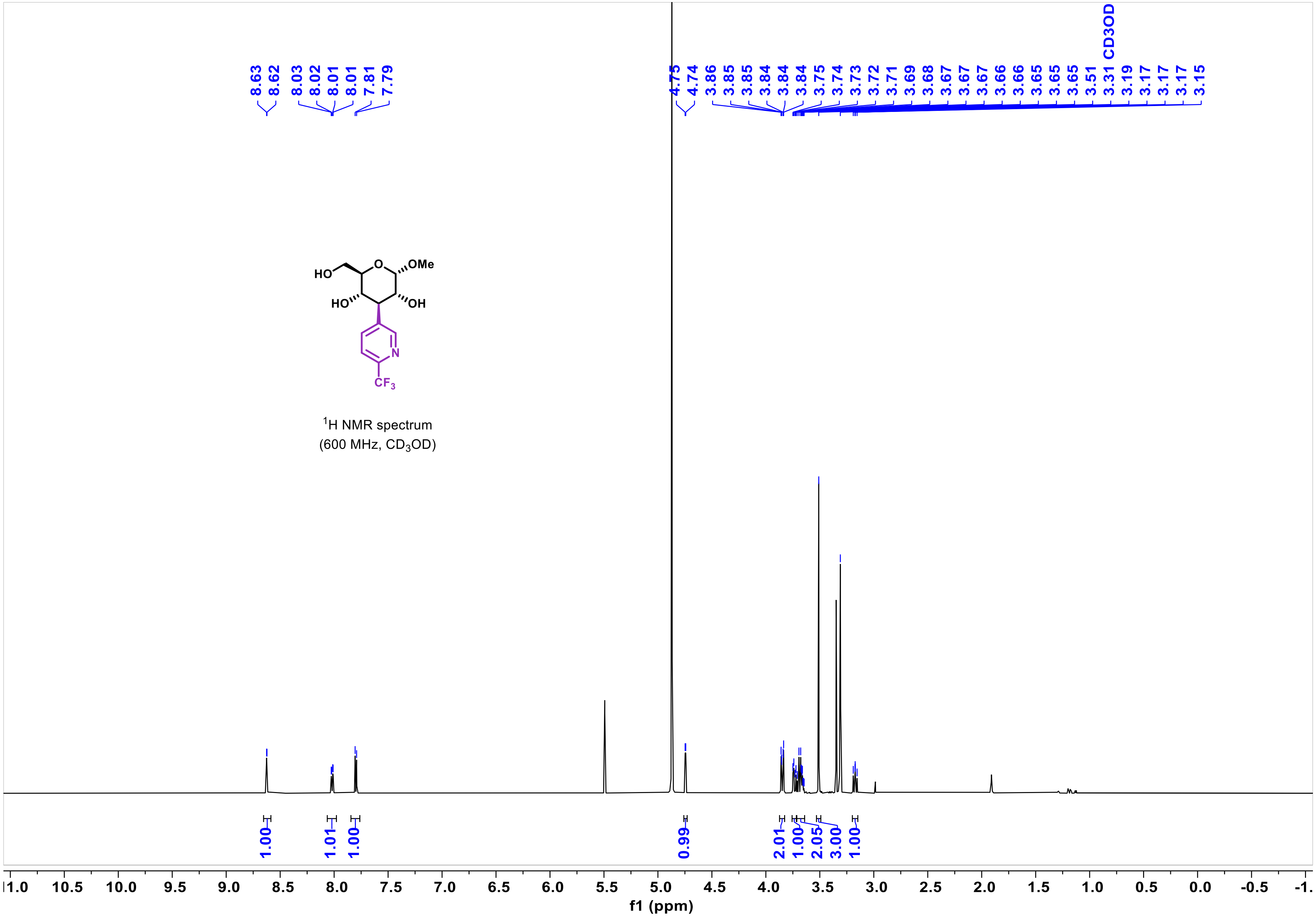
8.63
8.62
8.03
8.02
8.01
8.01
7.81
7.79
4.75
4.74
3.86
3.85
3.85
3.84
3.84
3.84
3.75
3.74
3.73
3.72
3.71
3.69
3.68
3.67
3.67
3.67
3.66
3.66
3.65
3.65
3.65
3.51
3.31
CD3OD
3.19
3.17
3.17
3.17
3.15
HO
O
OMe
HO
OH
N
CF3
1H NMR spectrum
(600 MHz, CD3OD)
1.00
1.01
1.00
0.99
2.01
1.00
2.05
3.00
1.00
11.0 10.5 10.0 9.5 9.0 8.5 8.0 7.5 7.0 6.5 6.0 5.5 5.0 4.5 4.0 3.5 3.0 2.5 2.0 1.5 1.0 0.5 0.0 -0.5 -1.
f1 (ppm)

^{1}H NMR of **136**

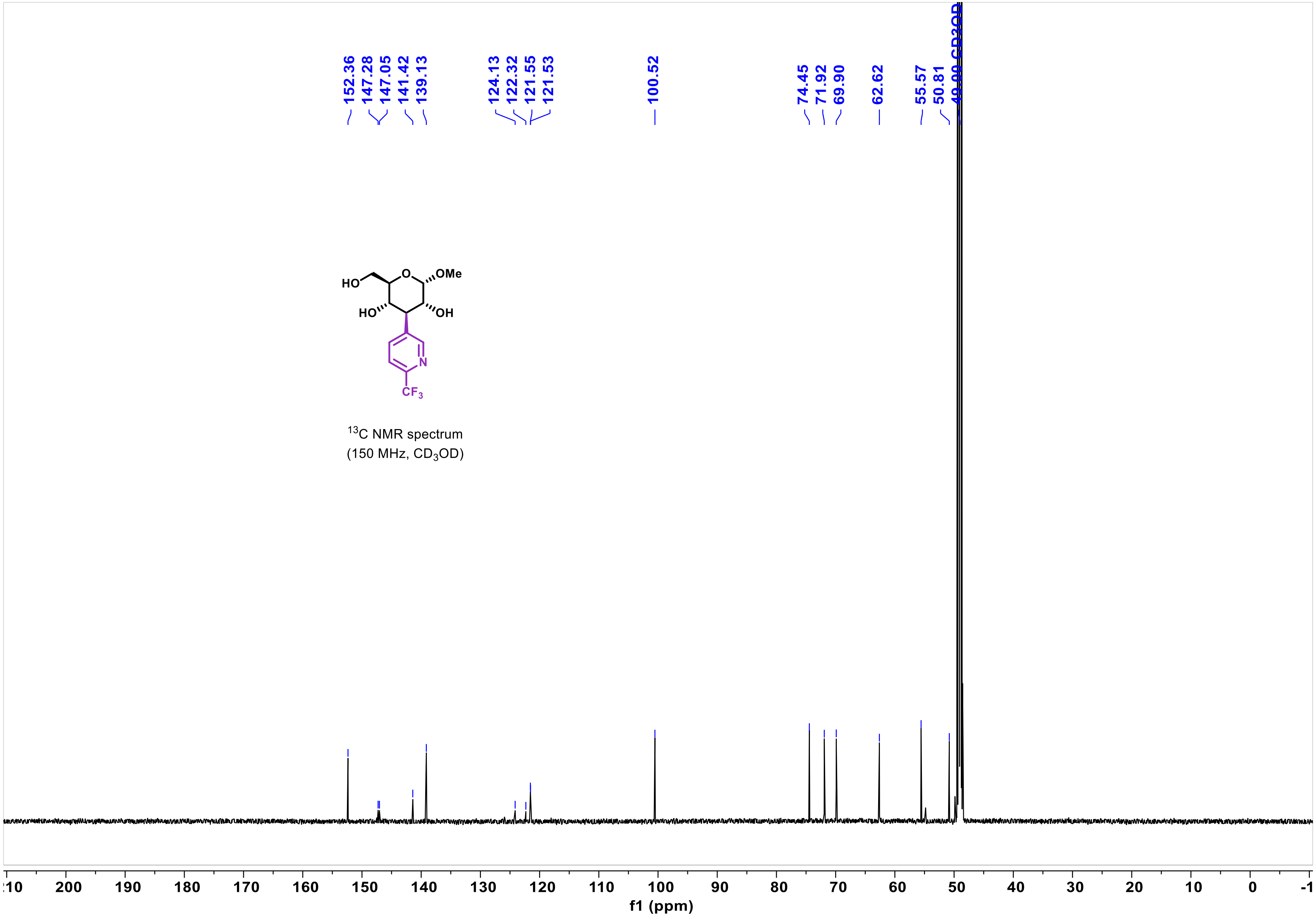

^{13}C NMR of **136**

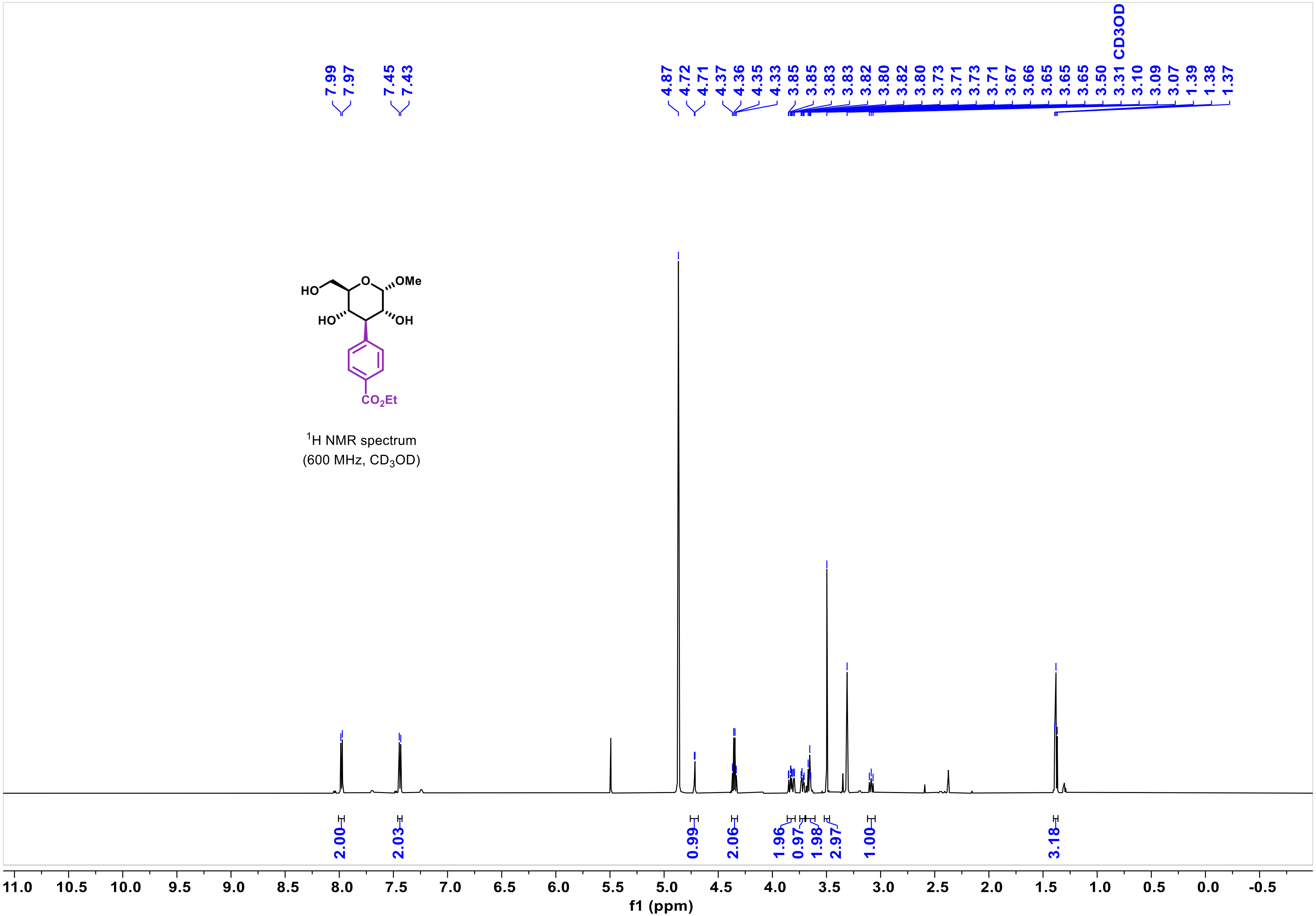
7.99
7.97
7.45
7.43
4.87
4.72
4.71
4.37
4.36
4.35
4.33
3.85
3.85
3.83
3.83
3.82
3.80
3.82
3.80
3.73
3.71
3.73
3.71
3.67
3.66
3.65
3.65
3.65
3.50
3.31 CD3OD
3.10
3.09
3.07
1.39
1.38
1.37
HO
O
OMe
HO
OH
CO_2Et
^{1}H NMR spectrum
(600 MHz, CD_3OD)
2.00
2.03
0.99
2.06
1.96
0.97
1.98
2.97
1.00
3.18
11.0
10.5
10.0
9.5
9.0
8.5
8.0
7.5
7.0
6.5
6.0
5.5
5.0
4.5
4.0
3.5
3.0
2.5
2.0
1.5
1.0
0.5
0.0
-0.5
f1 (ppm)

^{1}H NMR of **137**

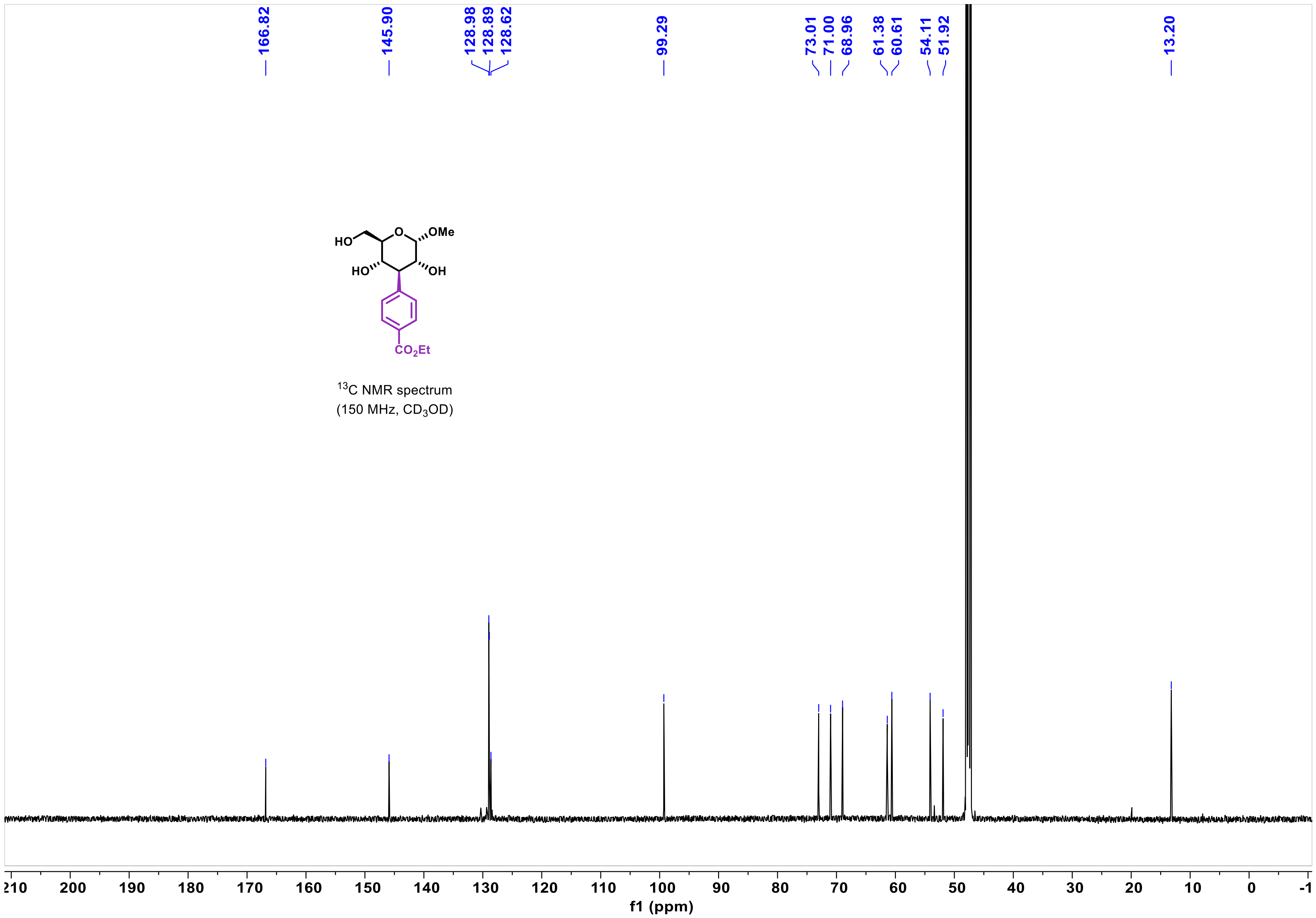

^{13}C NMR of **137**

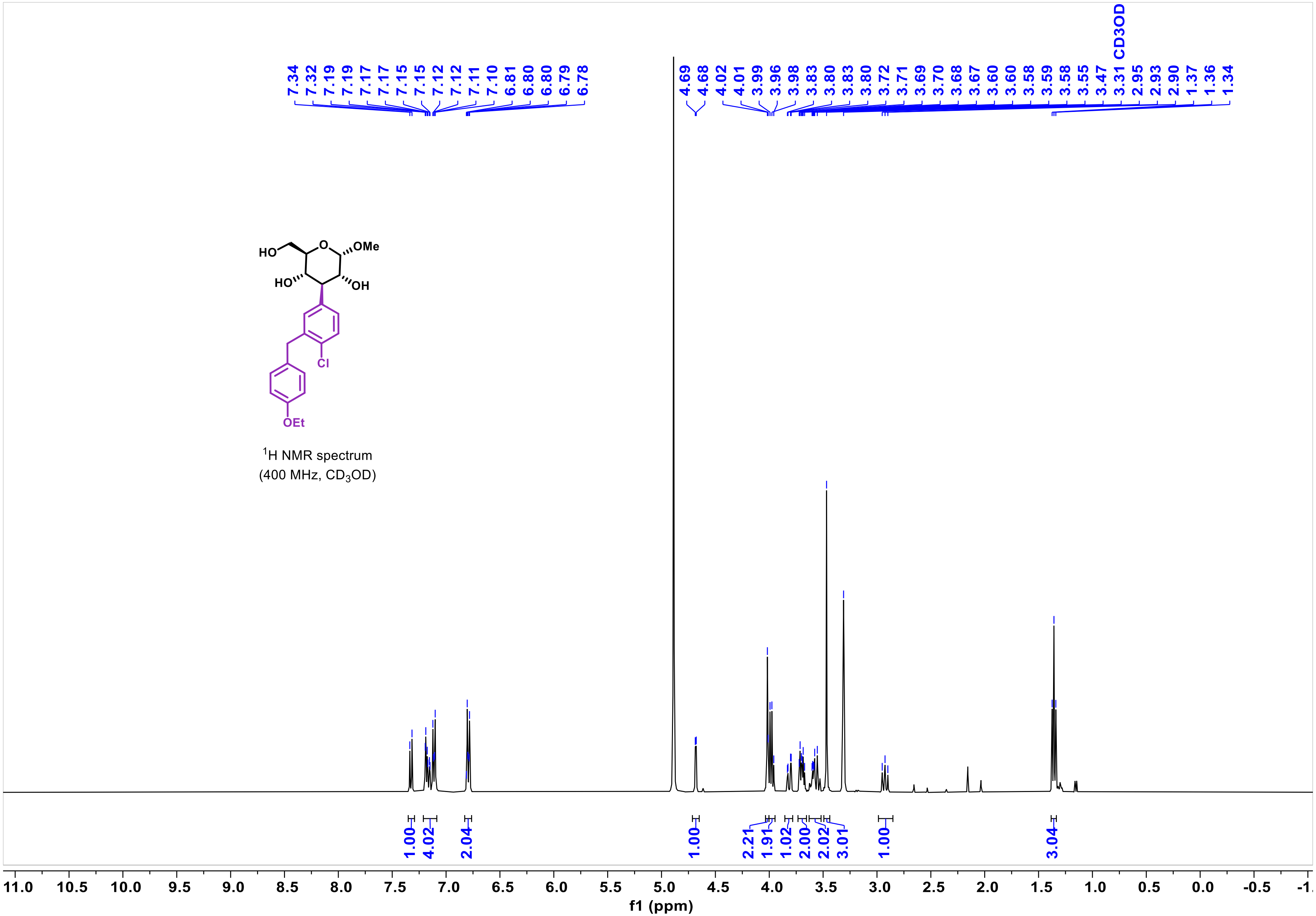
HO
O
OMe
HO
OH
Cl
OEt
^{1}H NMR spectrum
(400 MHz, CD3OD)
7.34 7.32 7.19 7.19 7.17 7.17 7.15 7.15 7.12 7.12 7.11 7.10 6.81 6.80 6.80 6.79 6.78
4.69 4.68 4.02 4.01 3.99 3.96 3.98 3.83 3.80 3.83 3.80 3.72 3.71 3.69 3.70 3.68 3.67 3.60 3.60 3.58 3.59 3.58 3.55 3.47 3.31 2.95 2.93 2.90 1.37 1.36 1.34
CD3OD
1.00 4.02 2.04 1.00 2.21 1.91 1.02 2.00 2.02 3.01 1.00 3.04
11.0 10.5 10.0 9.5 9.0 8.5 8.0 7.5 7.0 6.5 6.0 5.5 5.0 4.5 4.0 3.5 3.0 2.5 2.0 1.5 1.0 0.5 0.0 -0.5
f1 (ppm)

^{1}H NMR of **138**

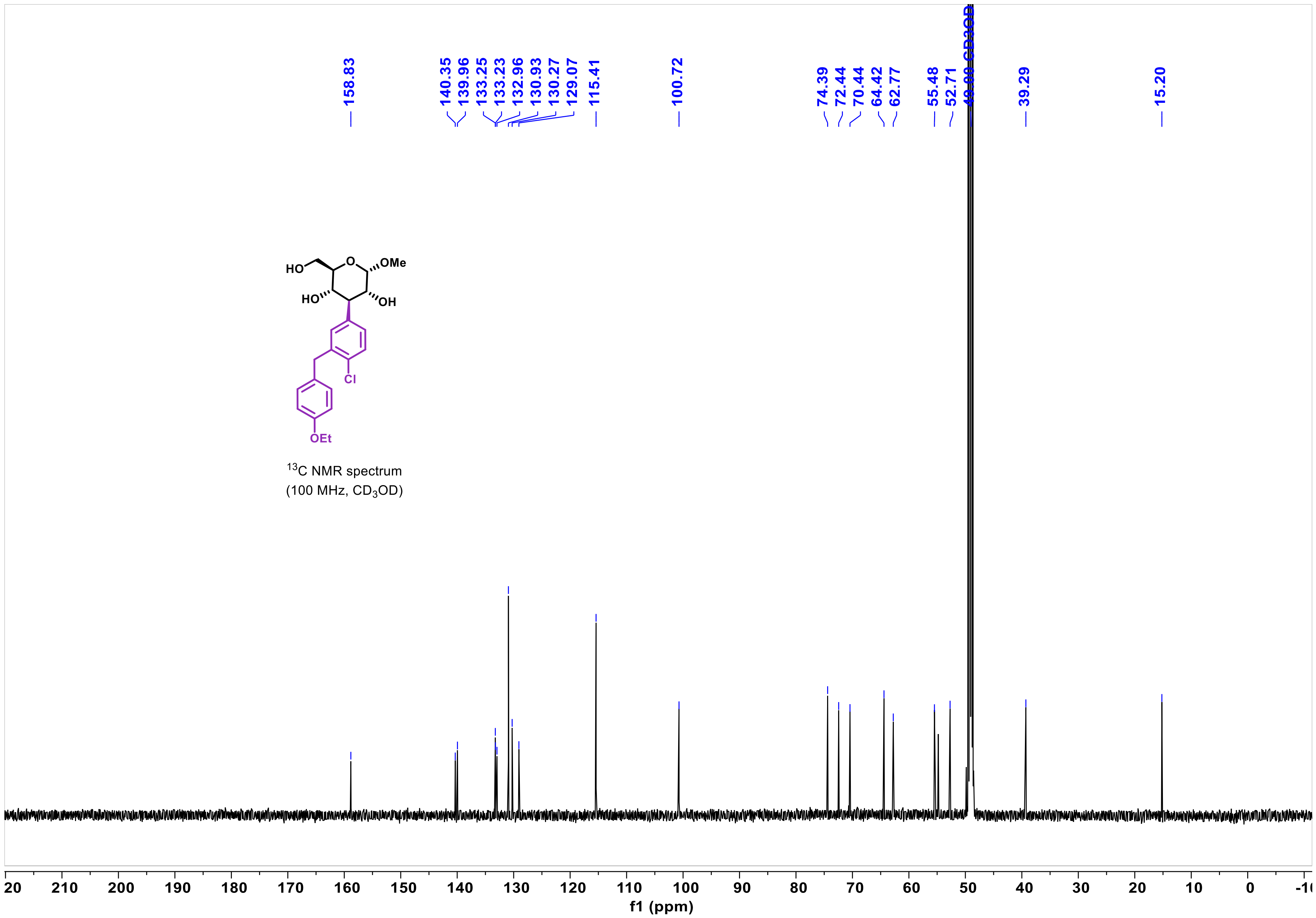

^{13}C NMR of **138**

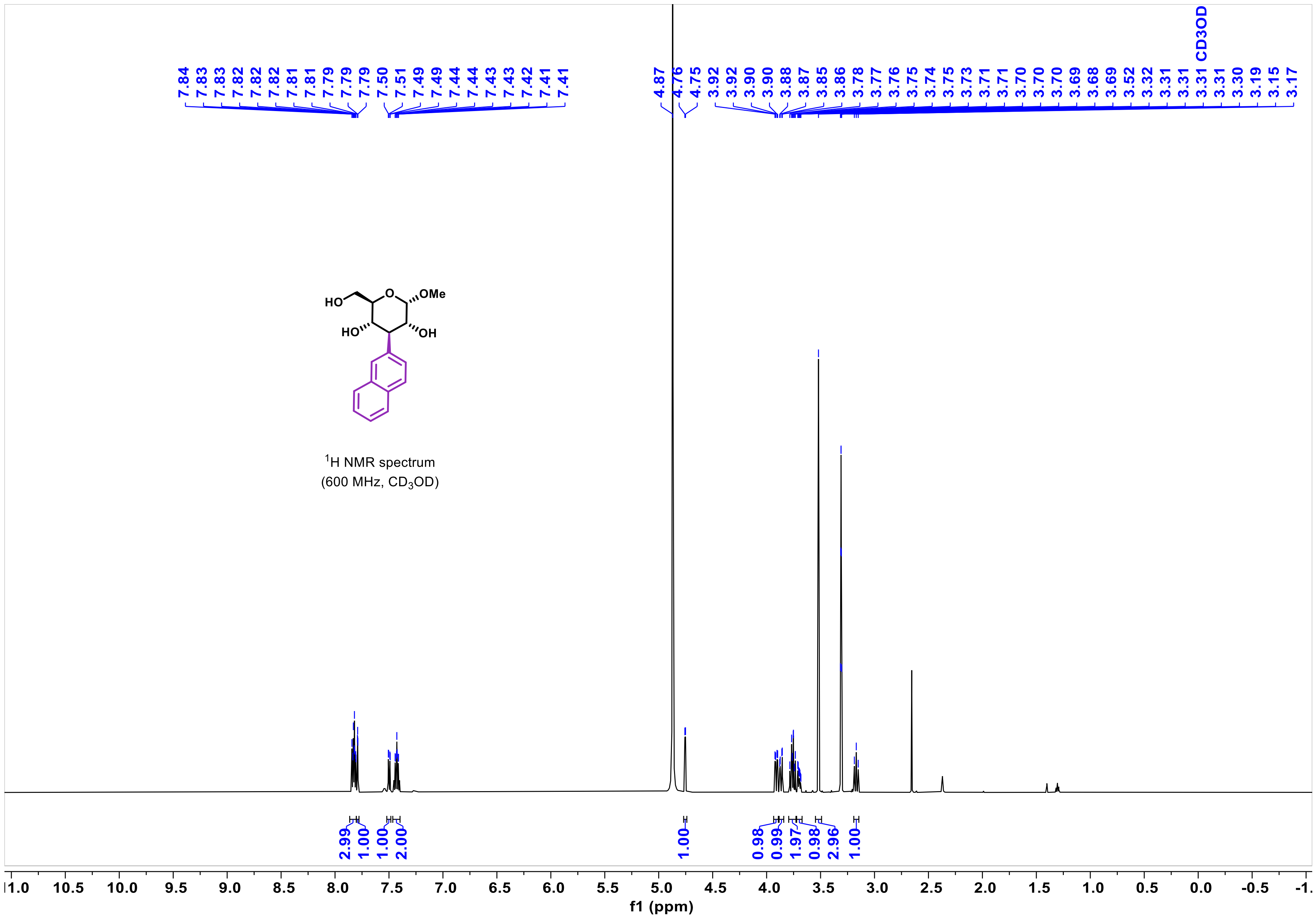

^{1}H NMR of **SL4**

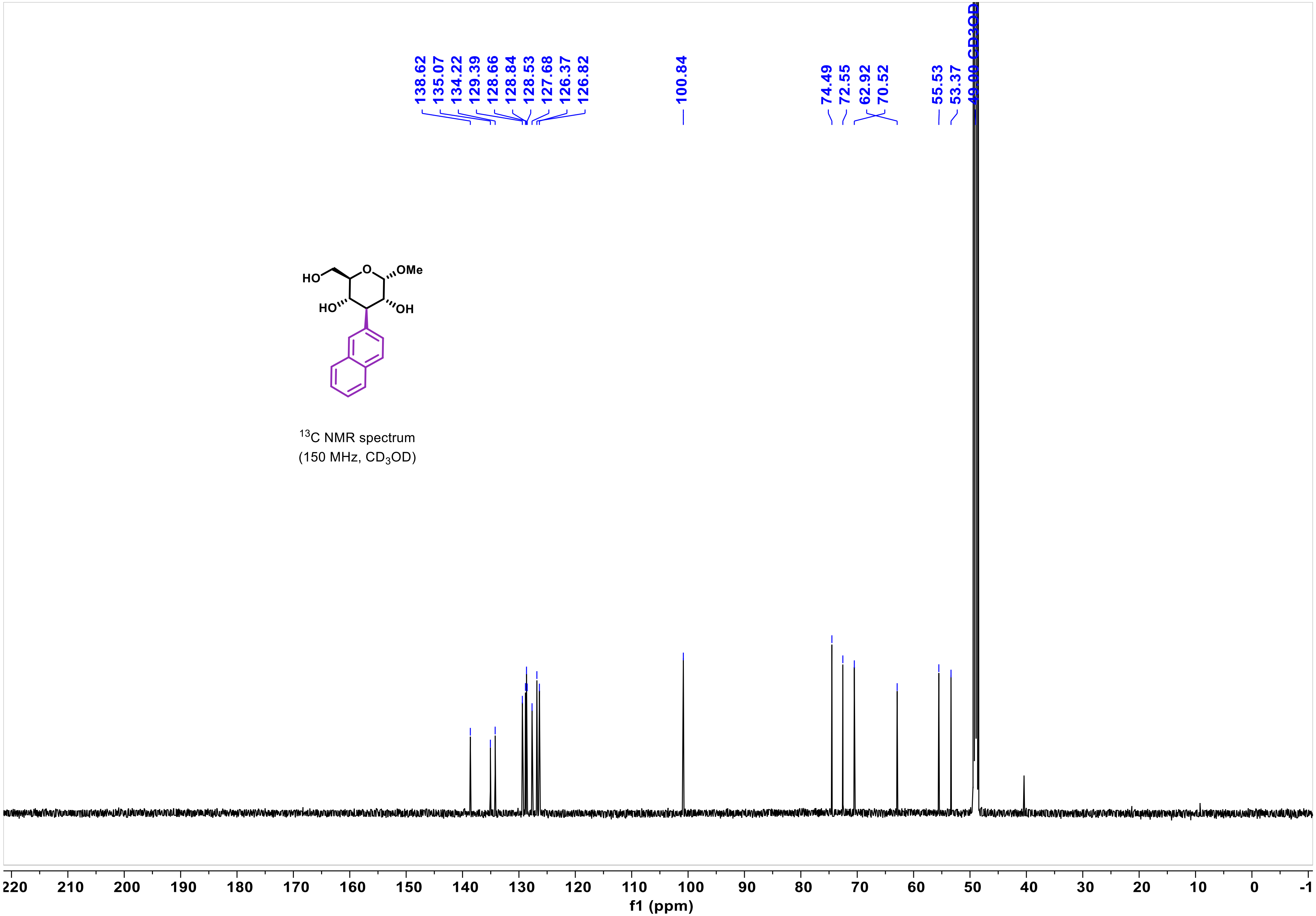

^{13}C NMR of **SL4**

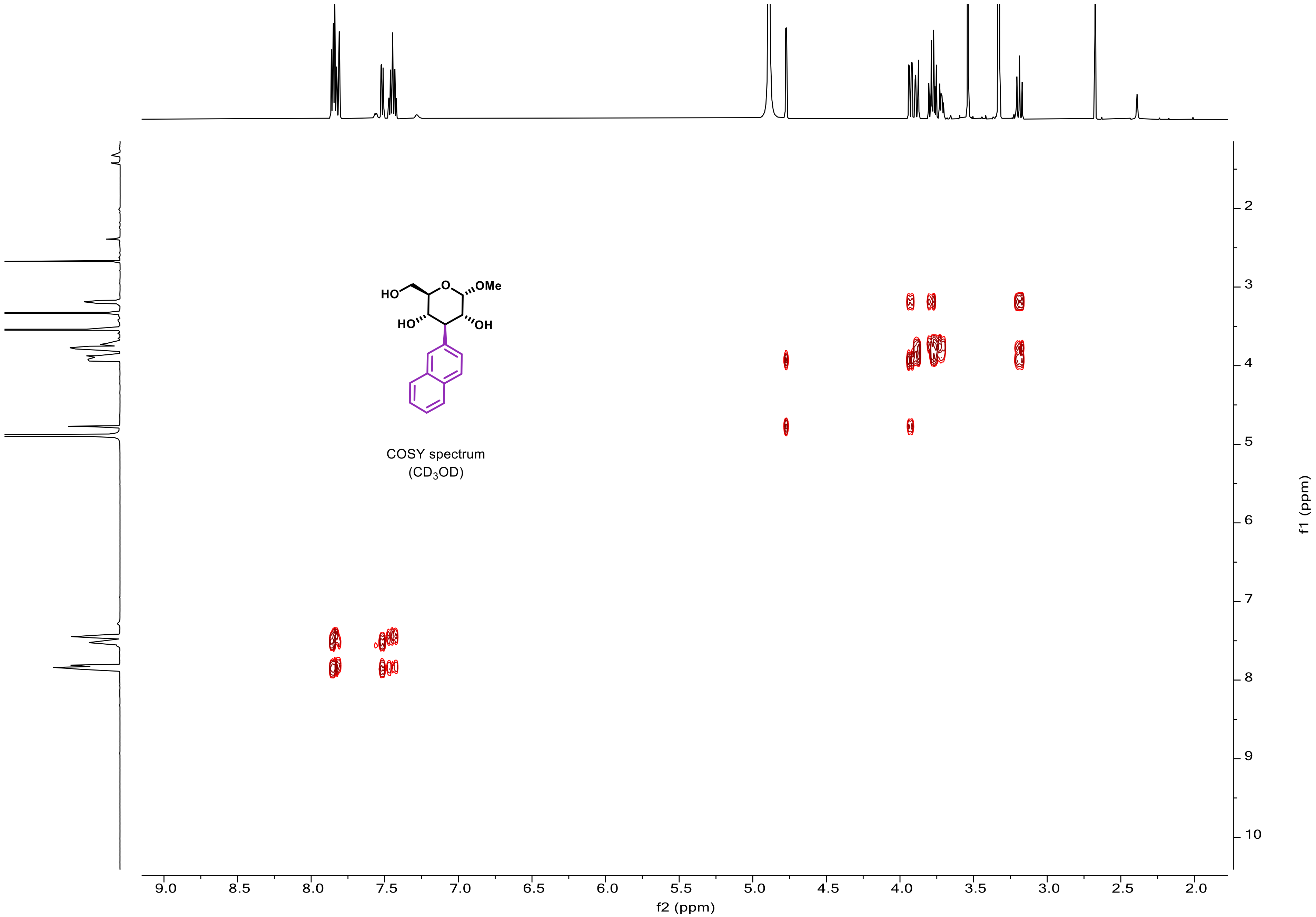

H-H COSY NMR Spectrum of compound **SL4**

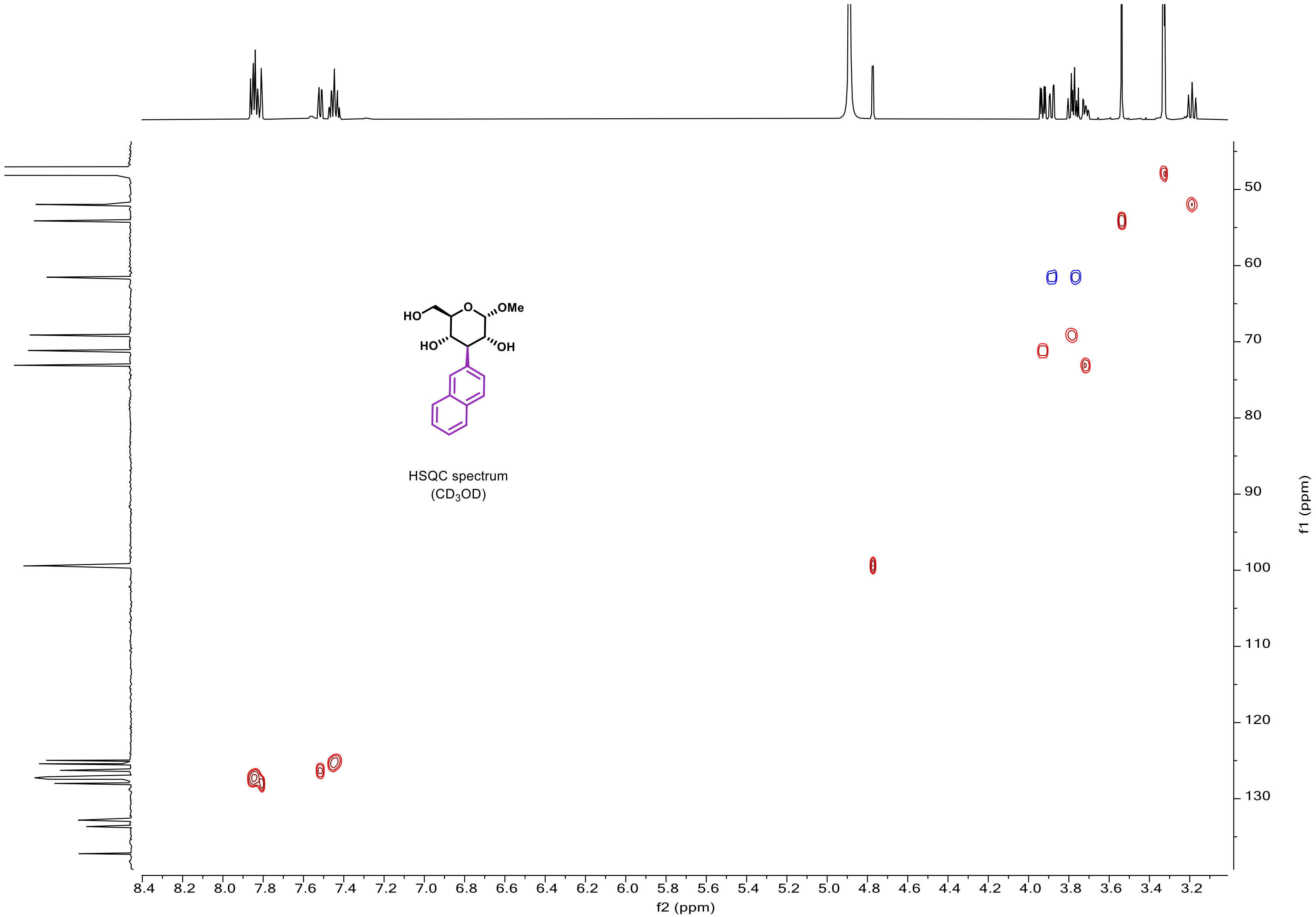

H-C HSQC NMR Spectrum of compound **SL4**

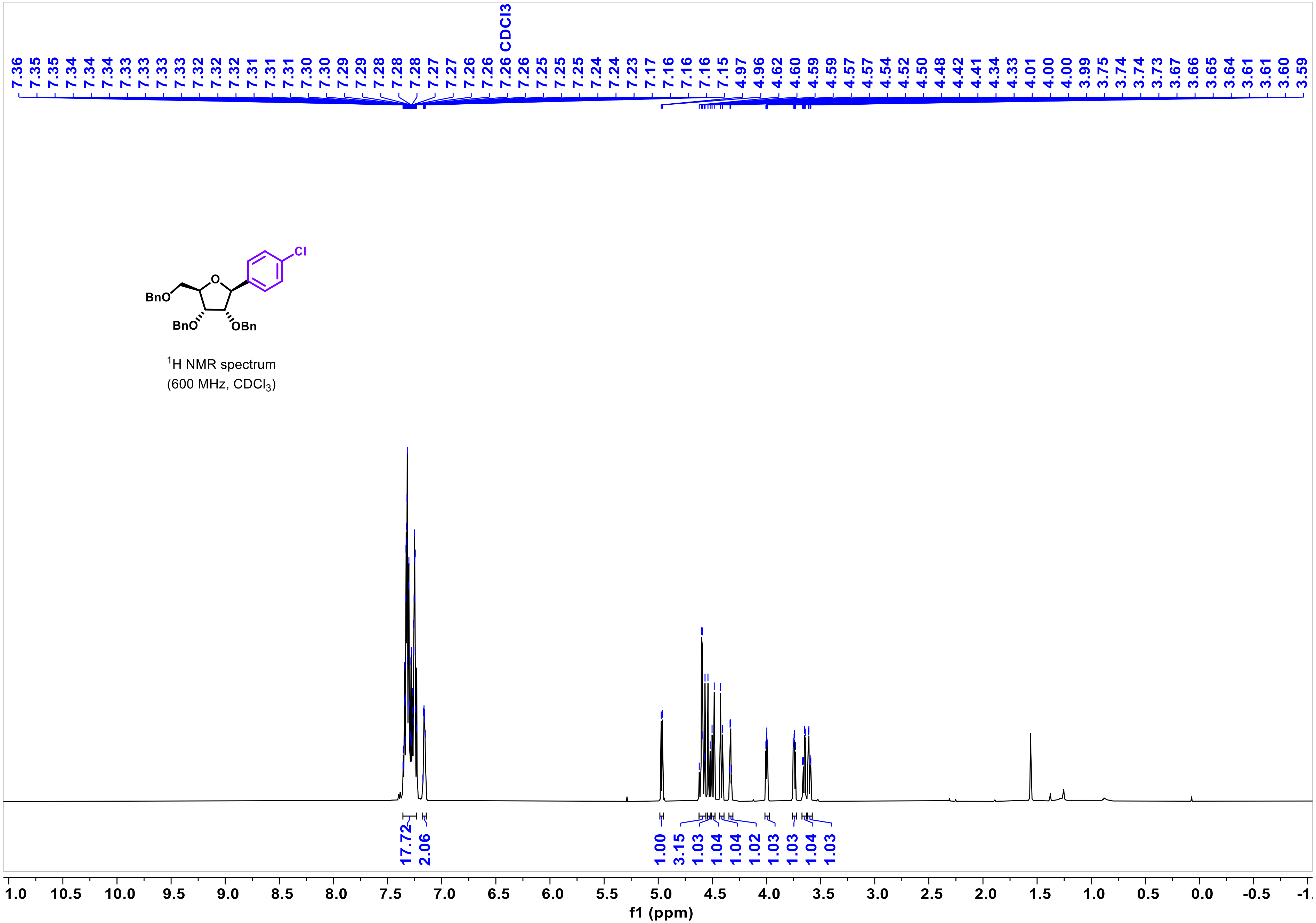

^{1}H NMR of compound **139**

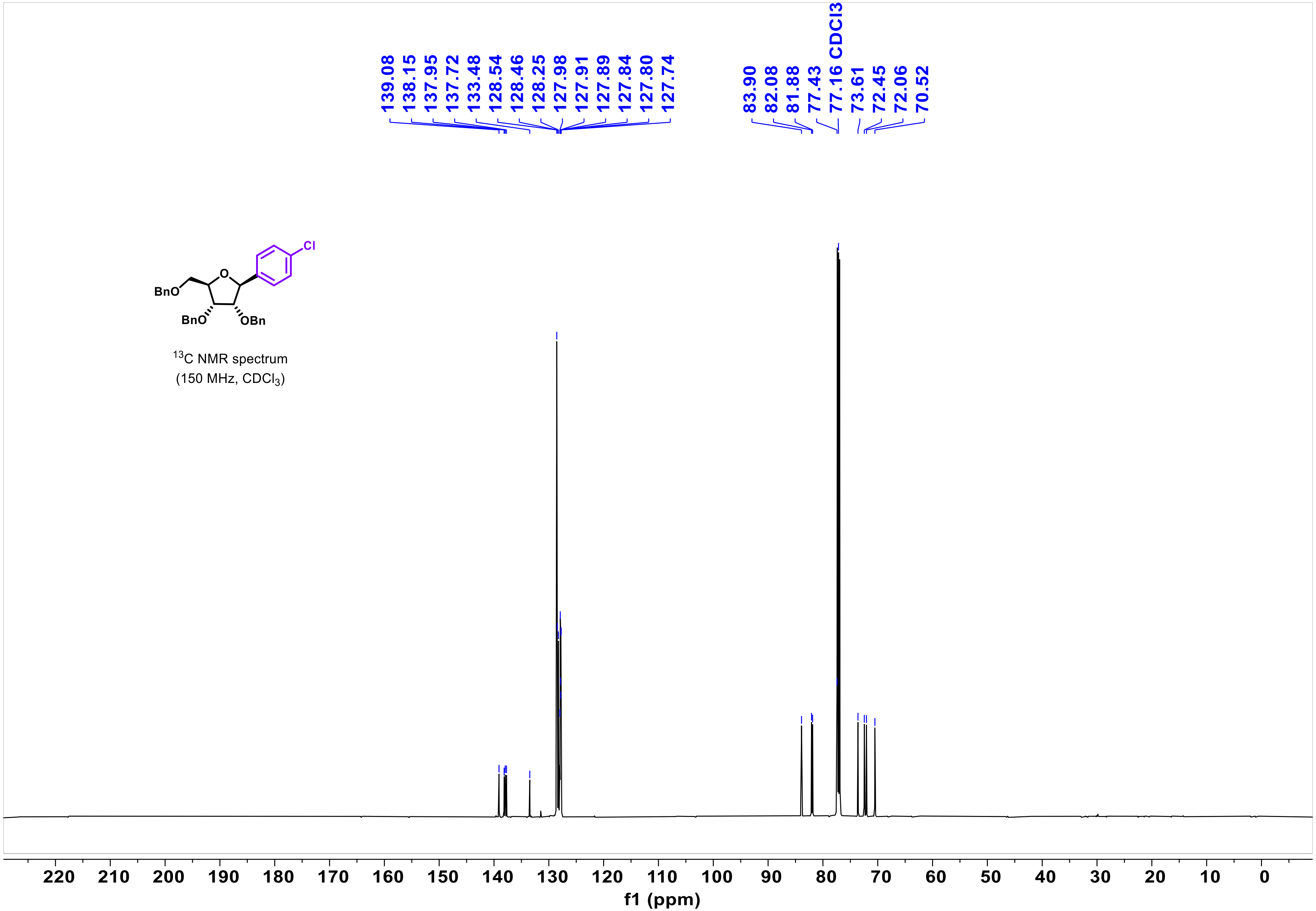

^{13}C NMR of compound **139**

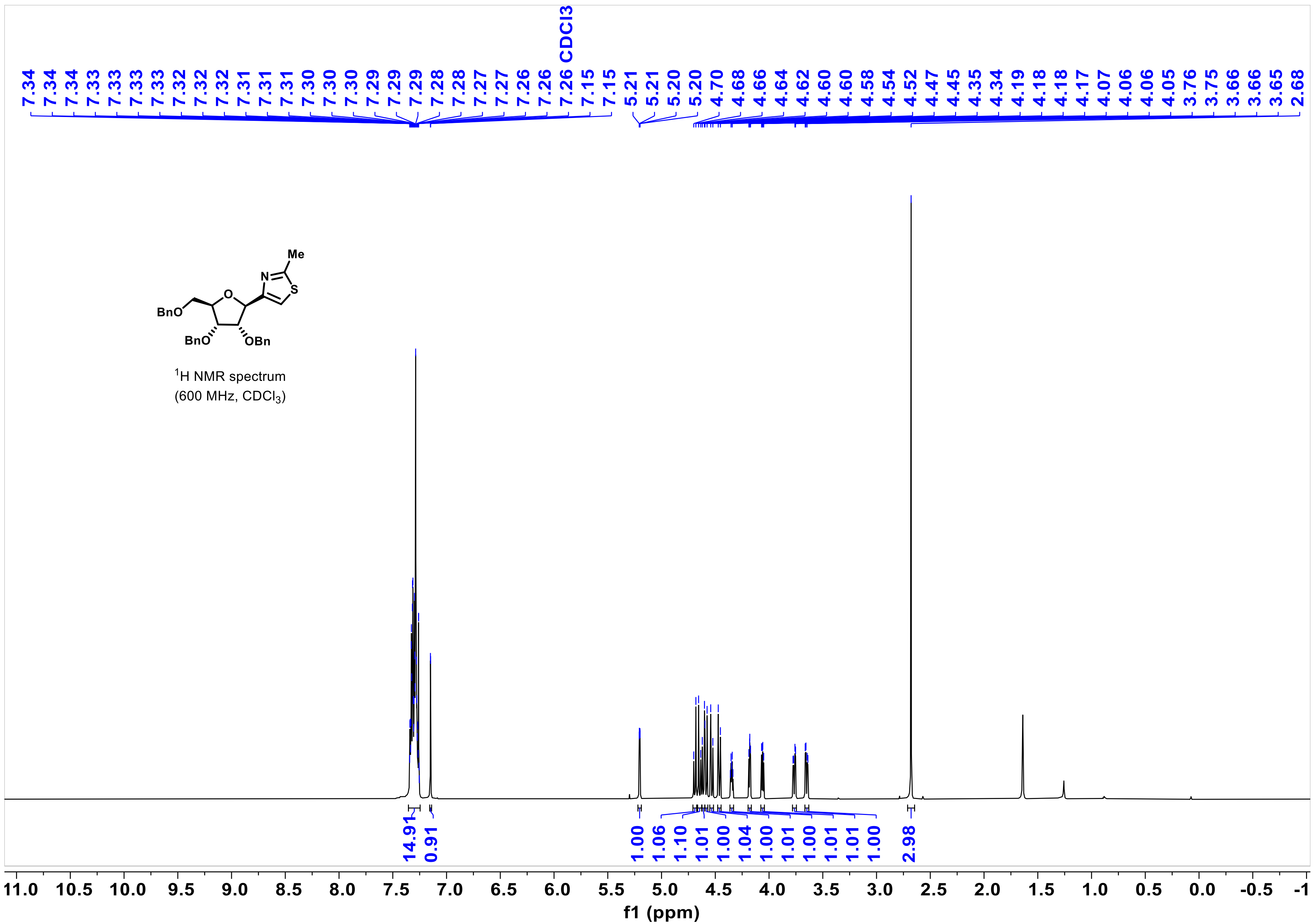

^{1}H NMR of compound **140**

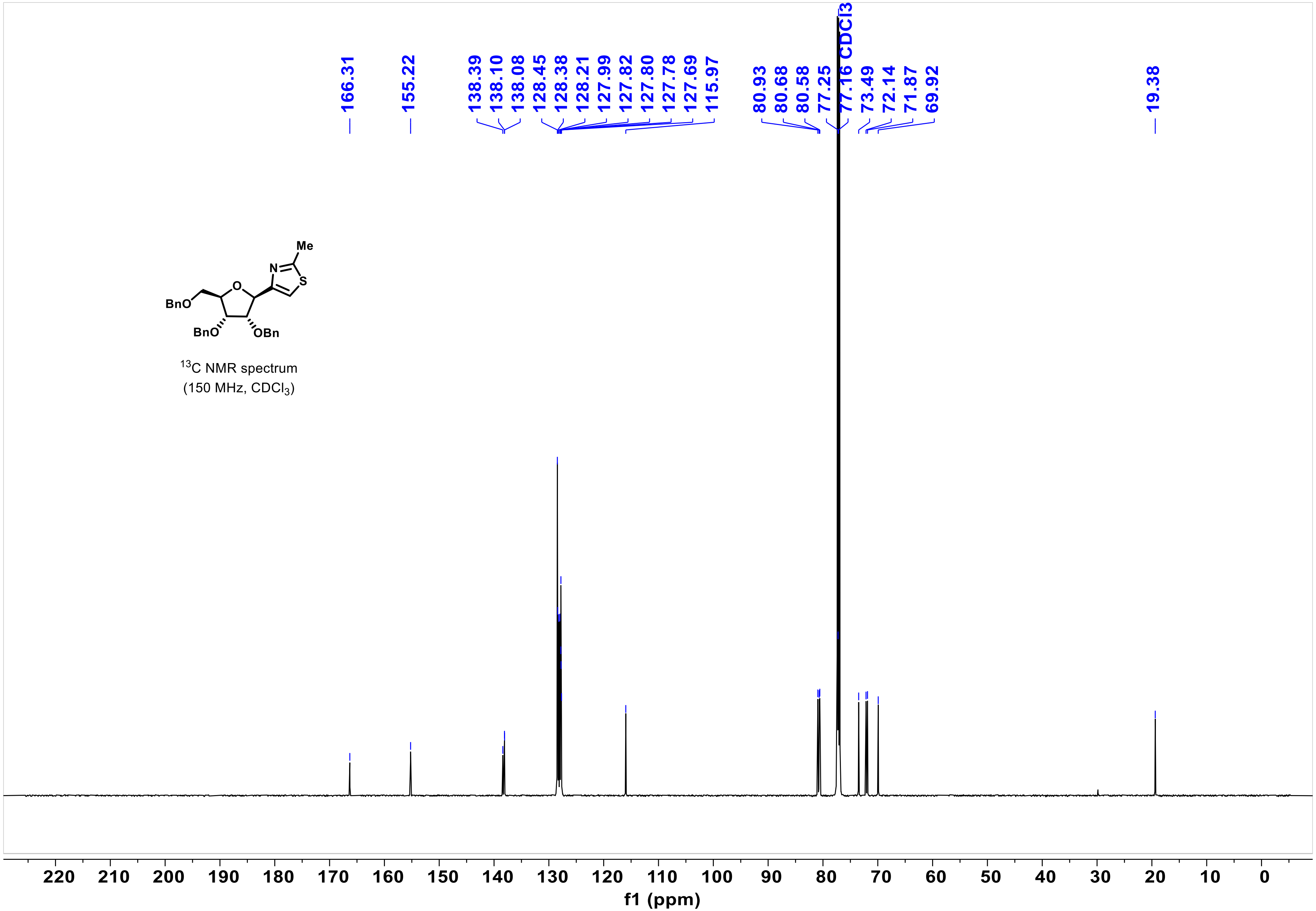

^{13}C NMR of compound **140**

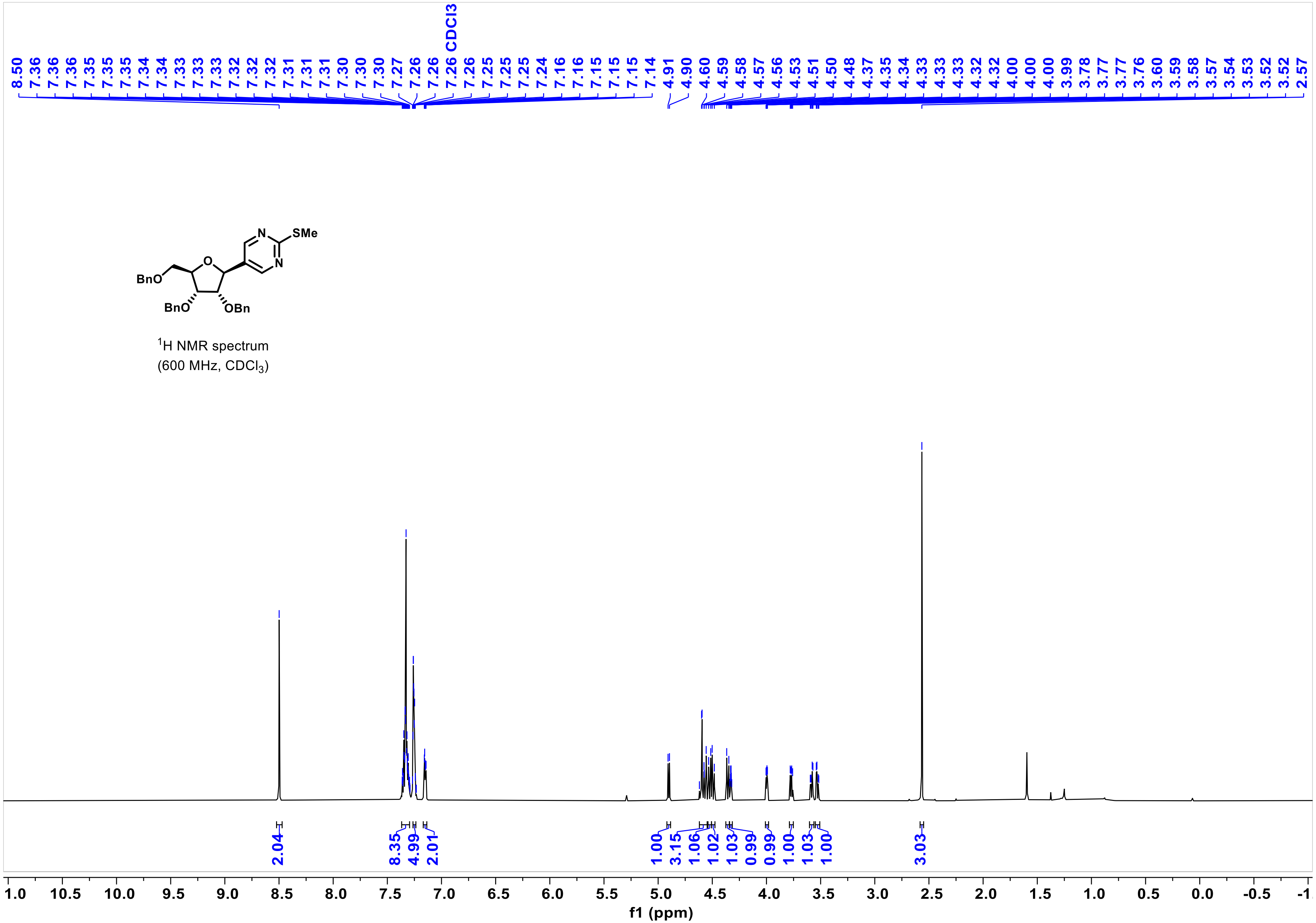

^{1}H NMR of compound **141**

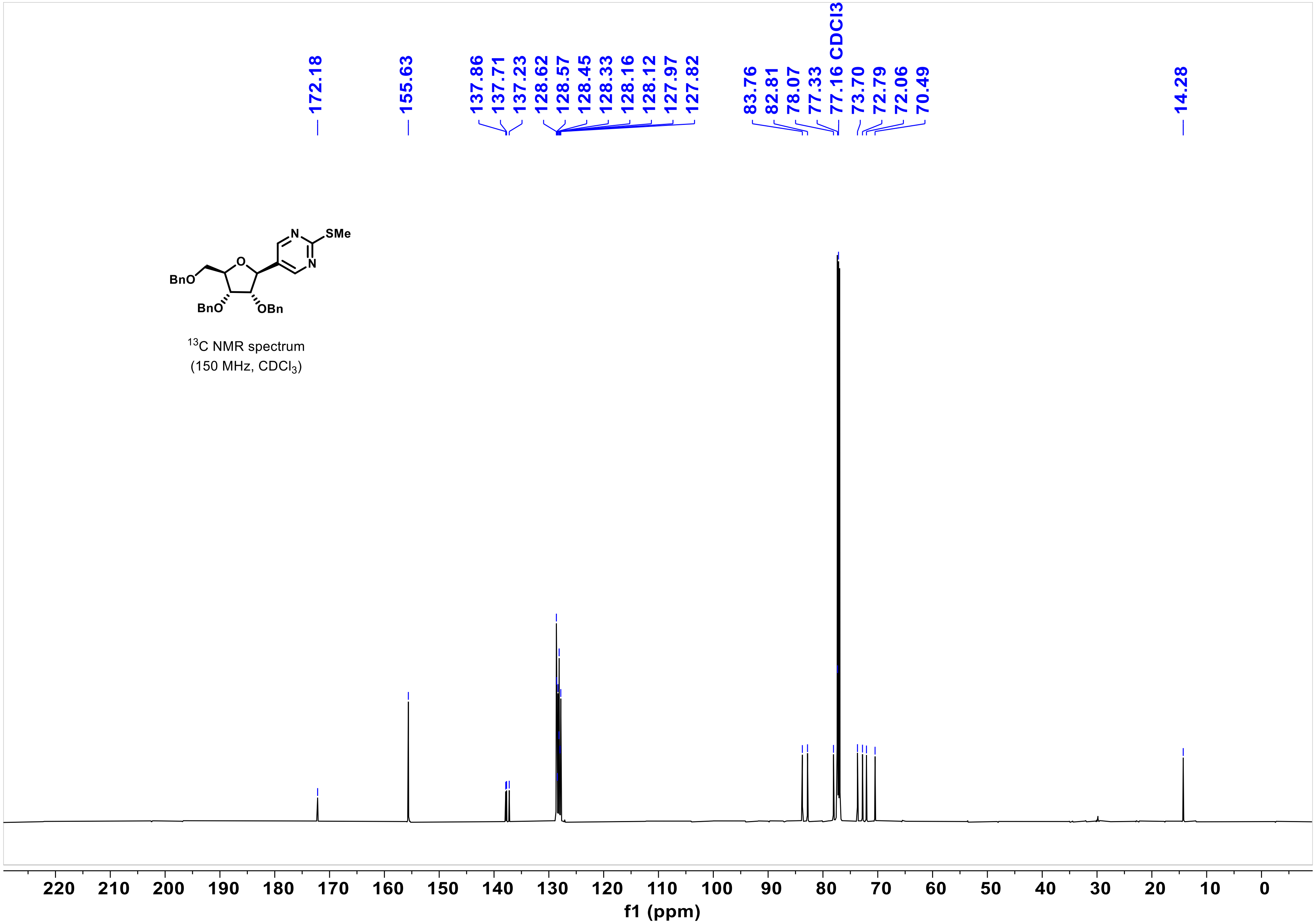

^{13}C NMR of compound **141**

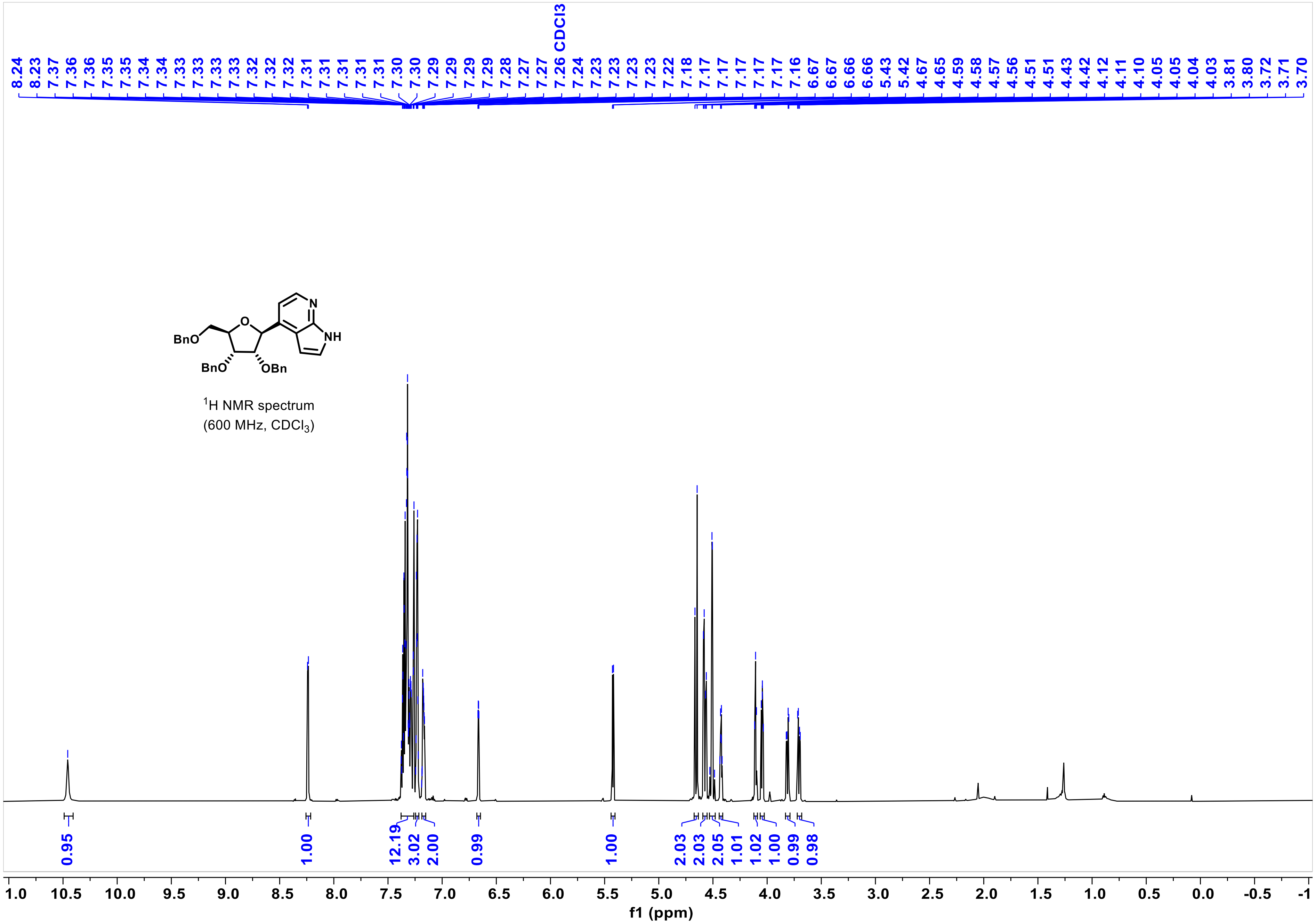

^{1}H NMR of compound **142**

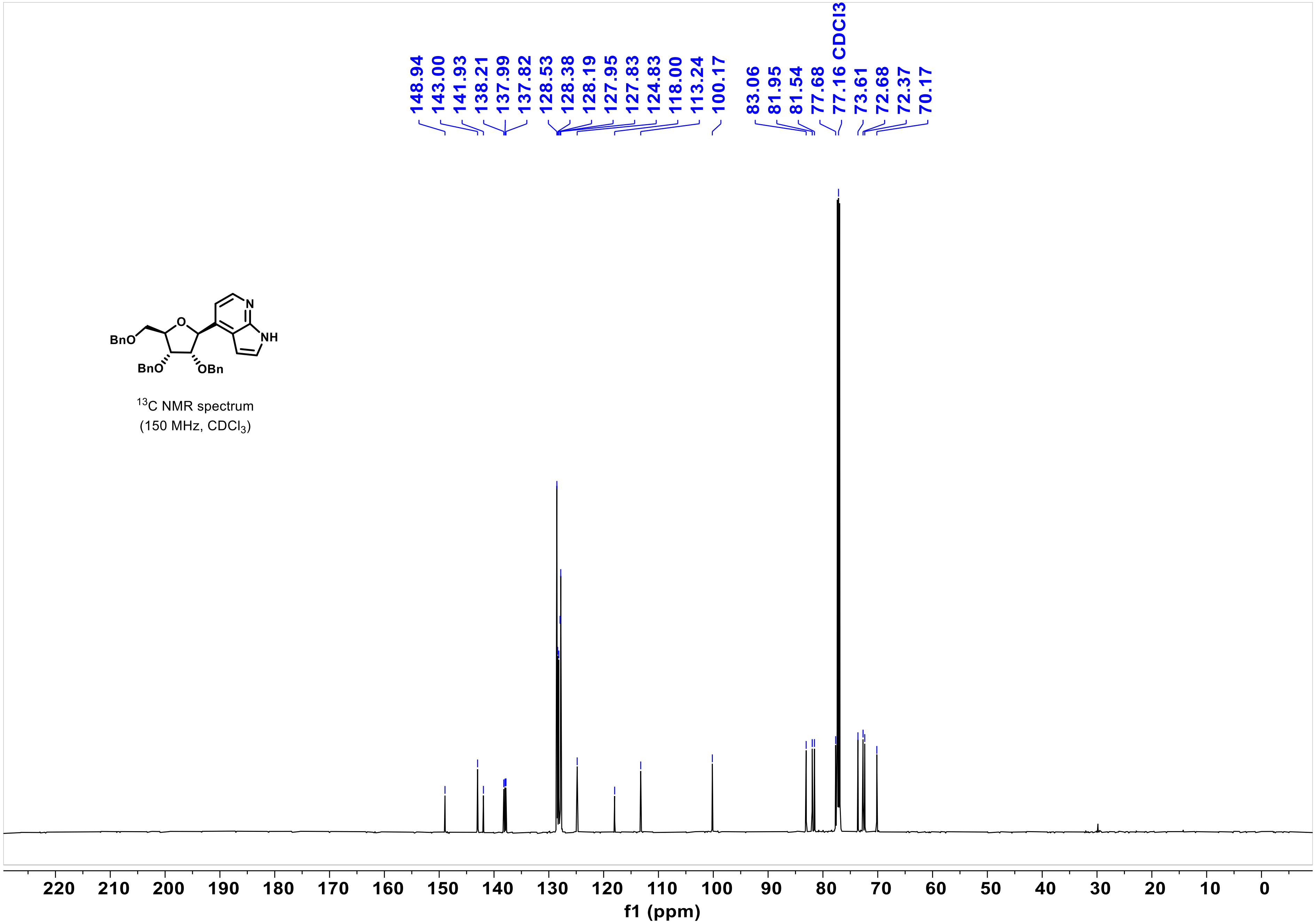

^{13}C NMR of compound **142**

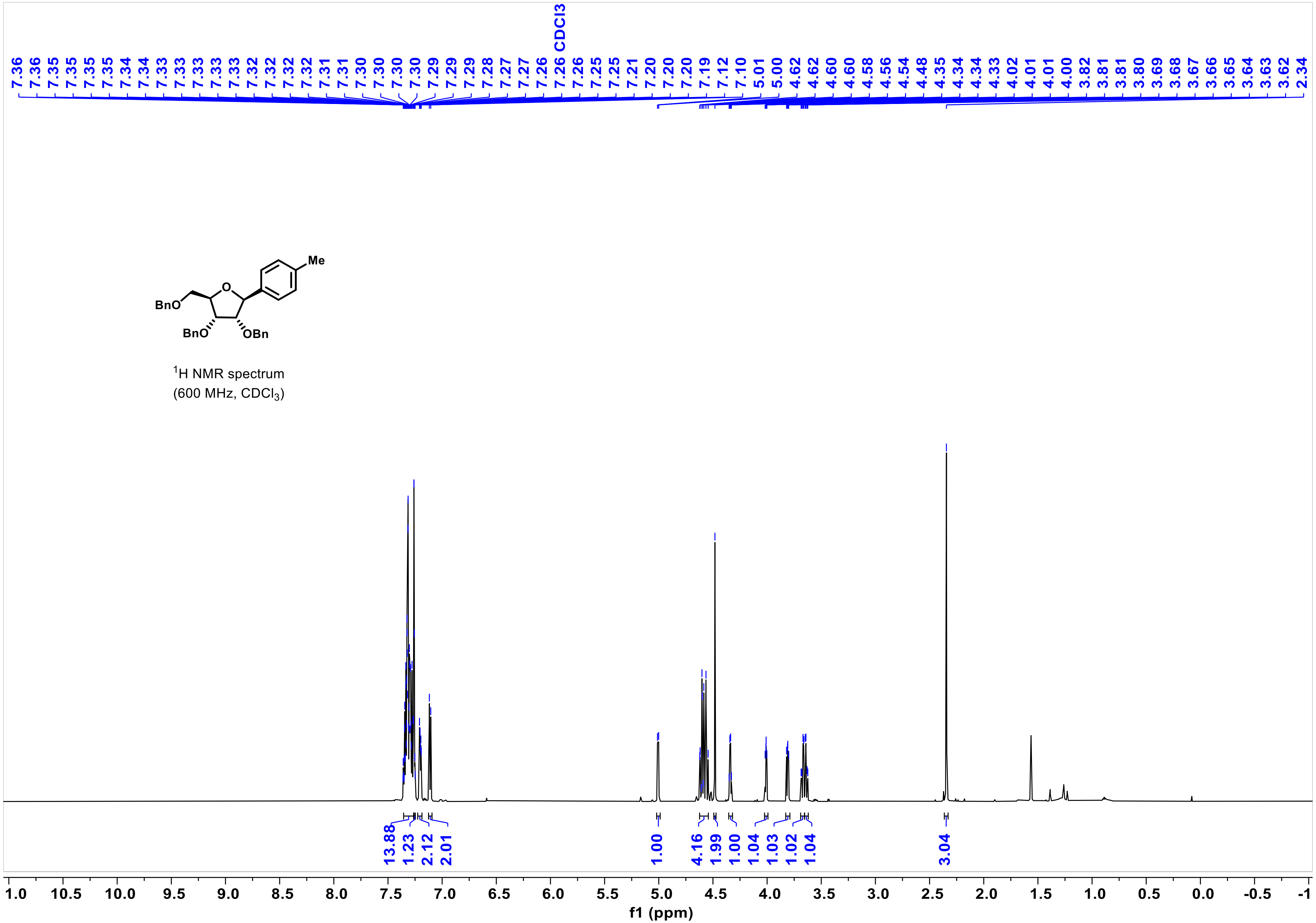

^{1}H NMR of compound **SD1**

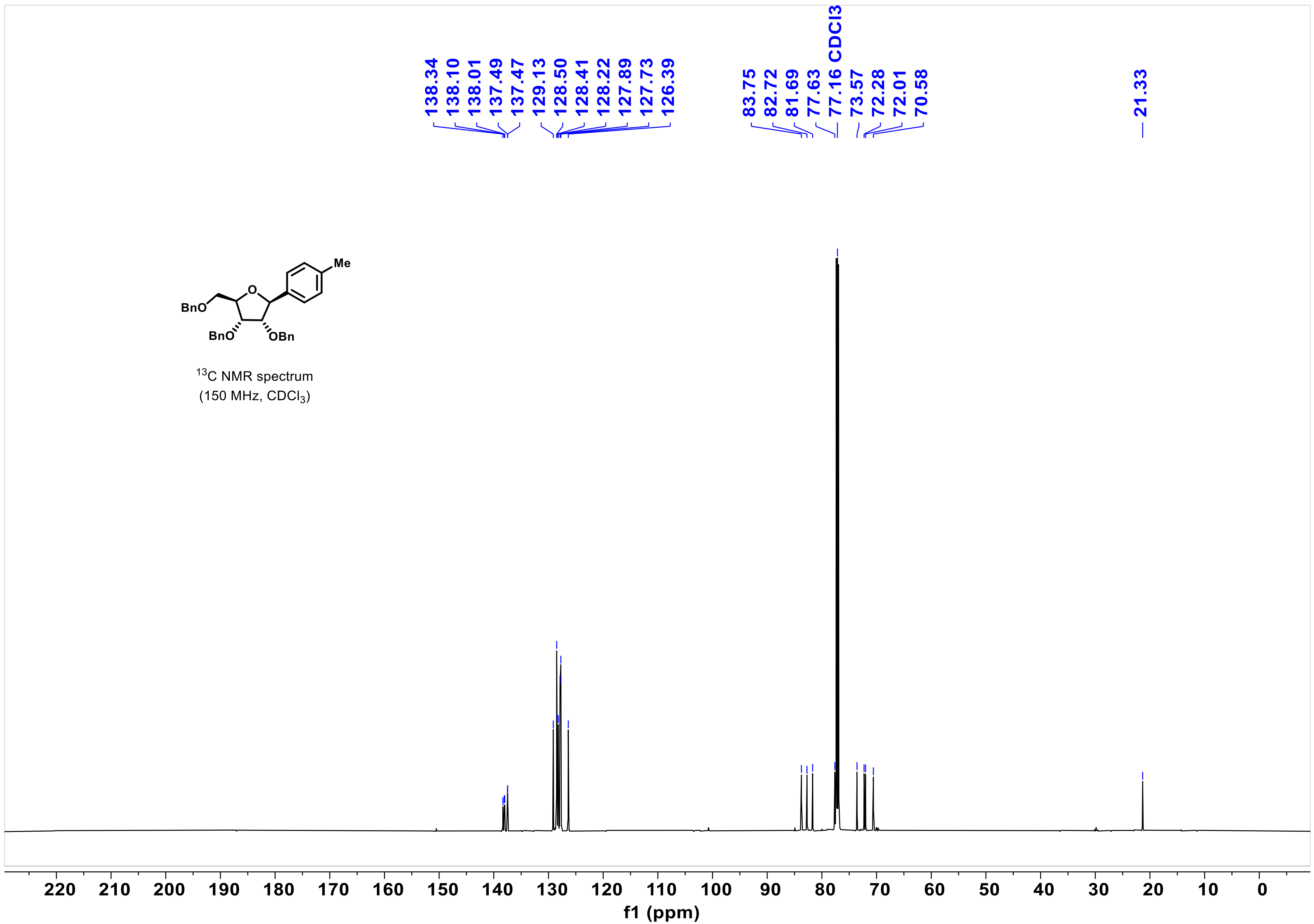

^{13}C NMR of compound **SD1**

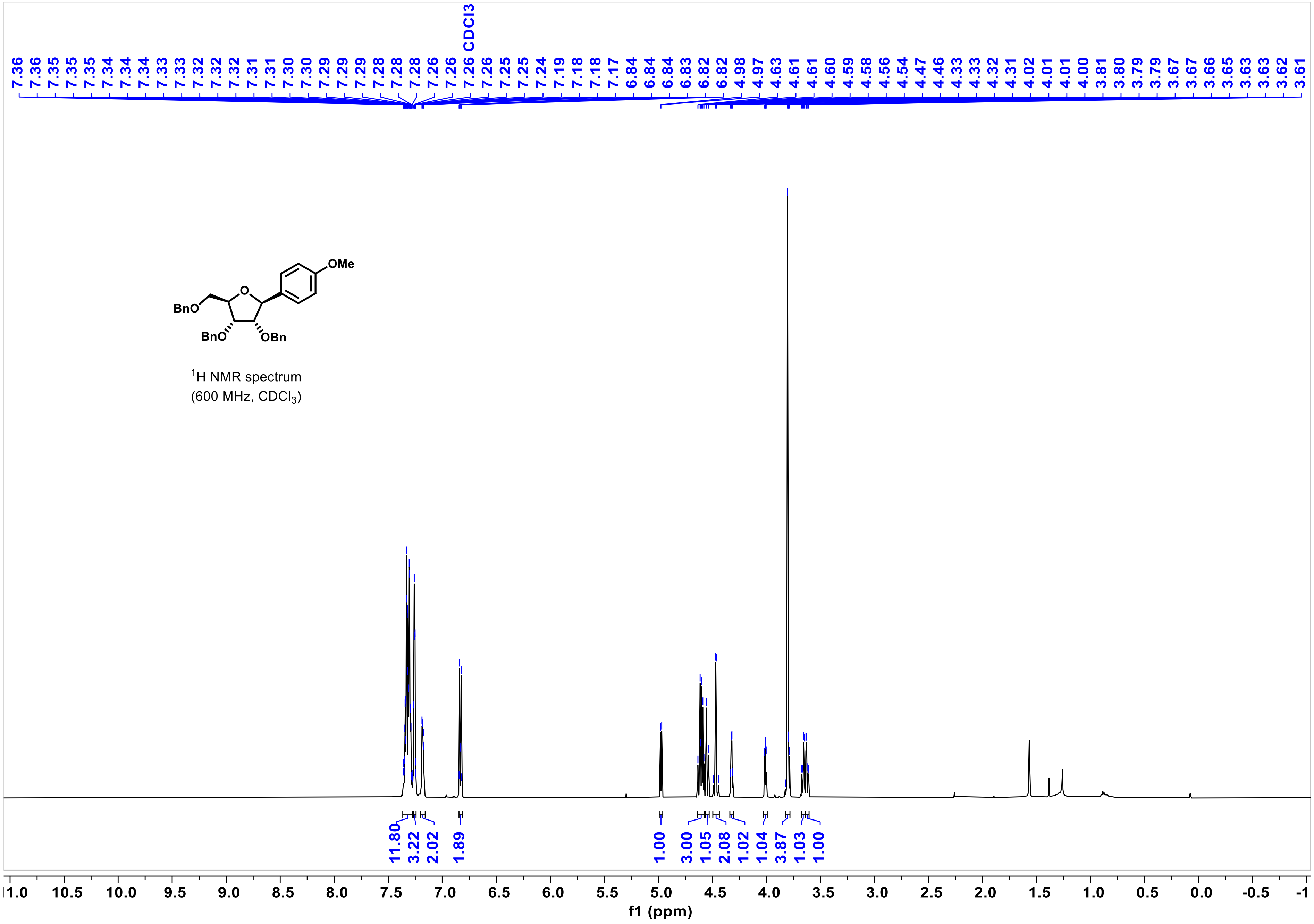

^{1}H NMR of compound **SD2**

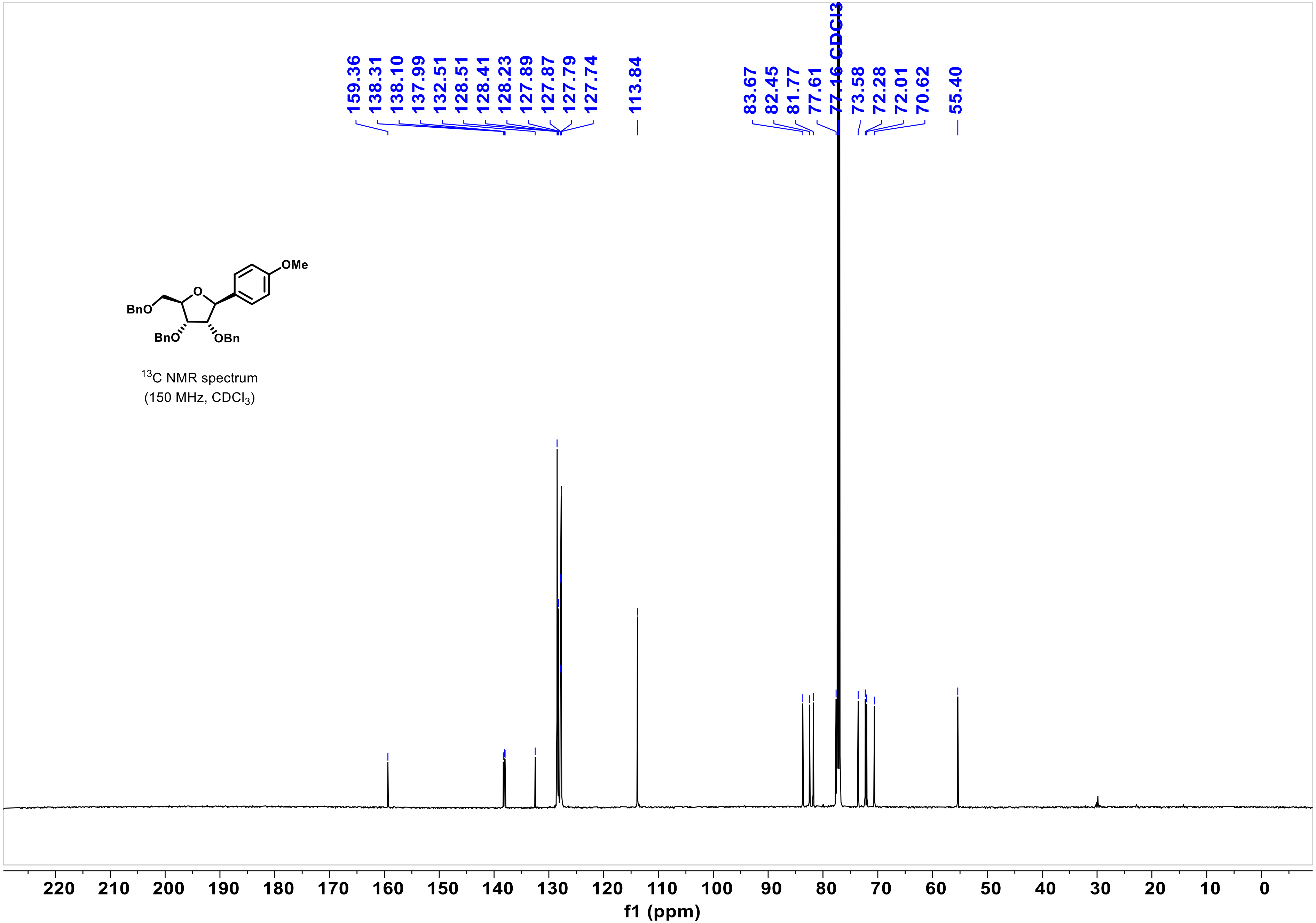

^{13}C NMR of compound **SD2**

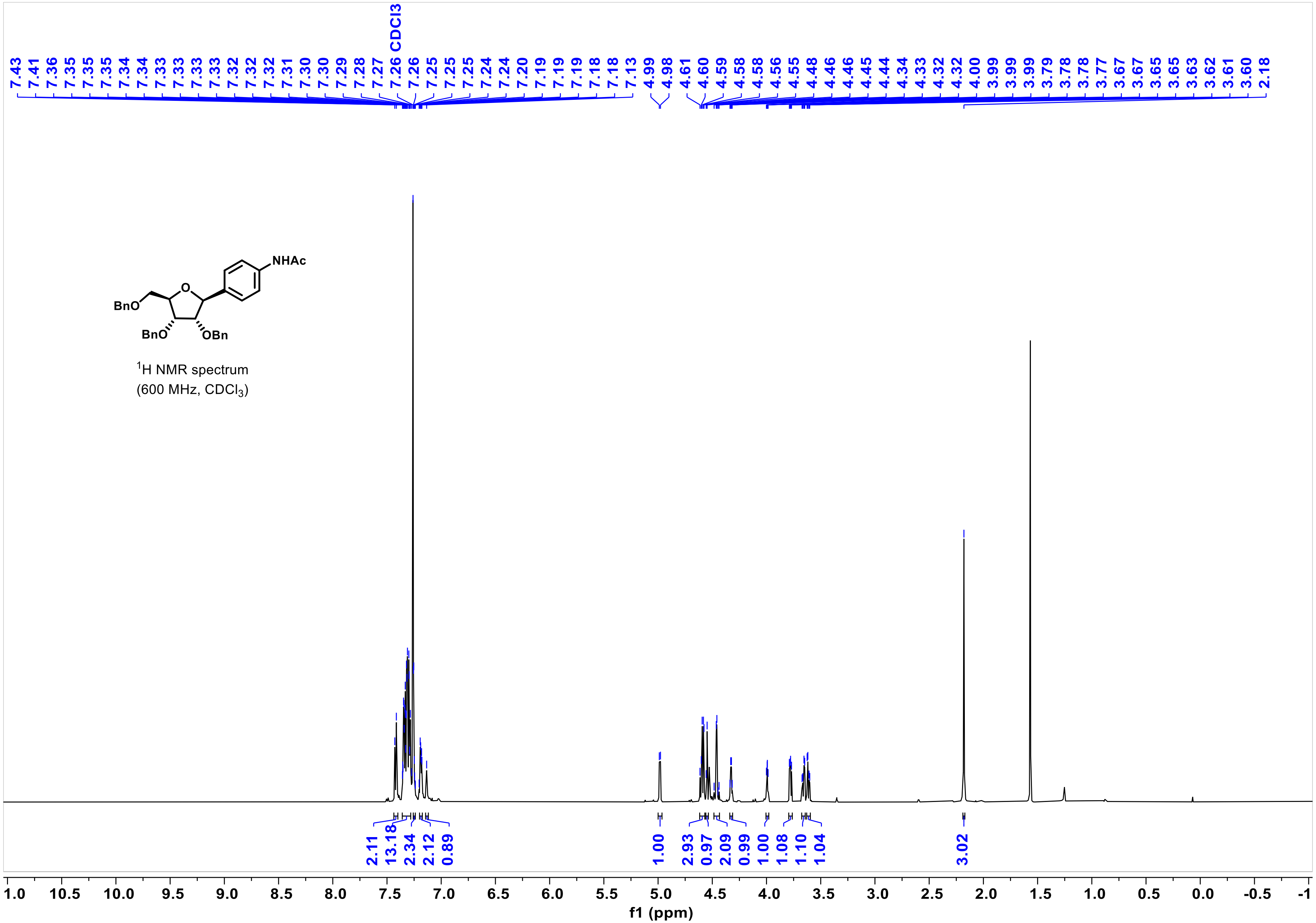

^{1}H NMR of compound **SD3**

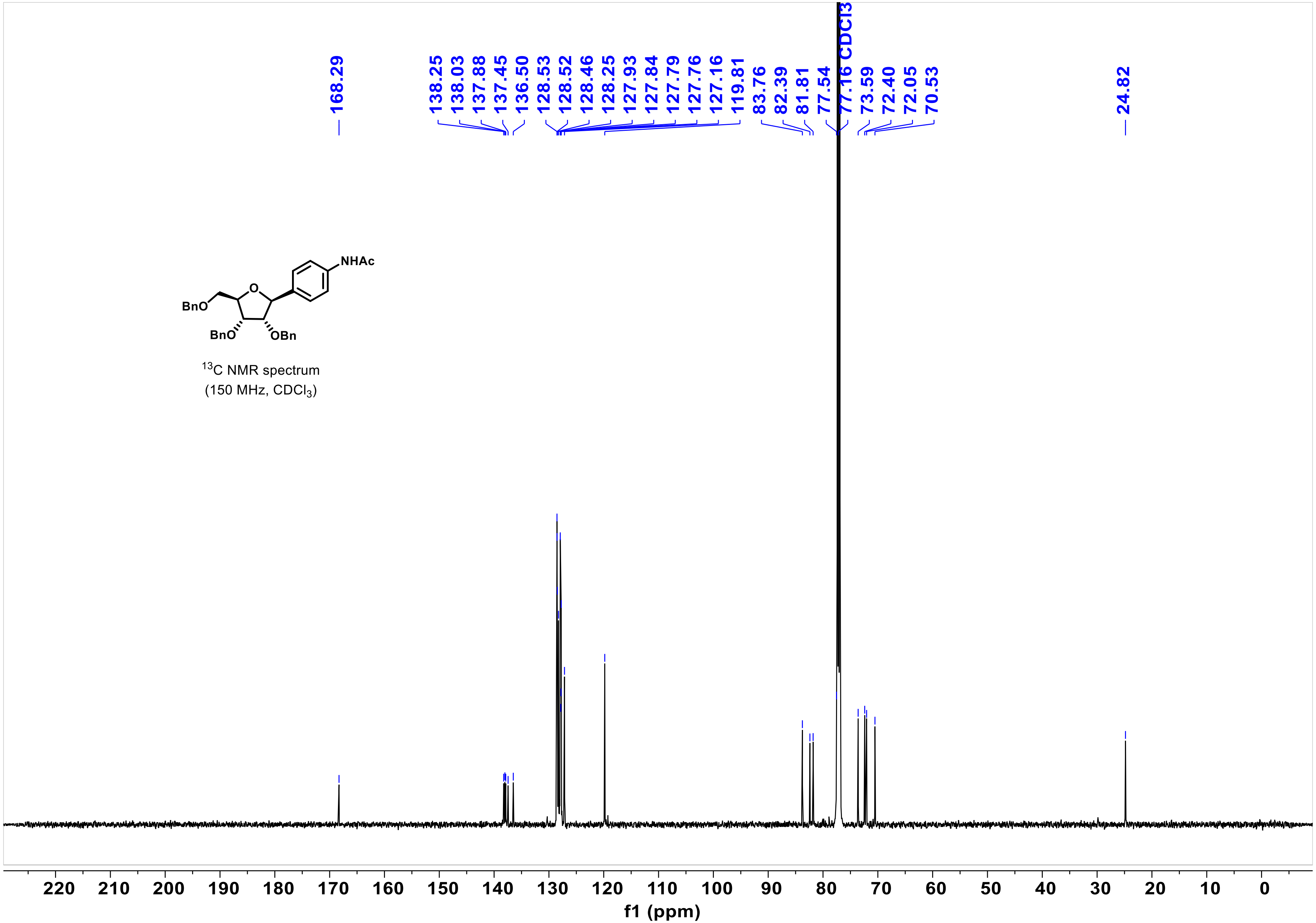

^{13}C NMR of compound **SD3**

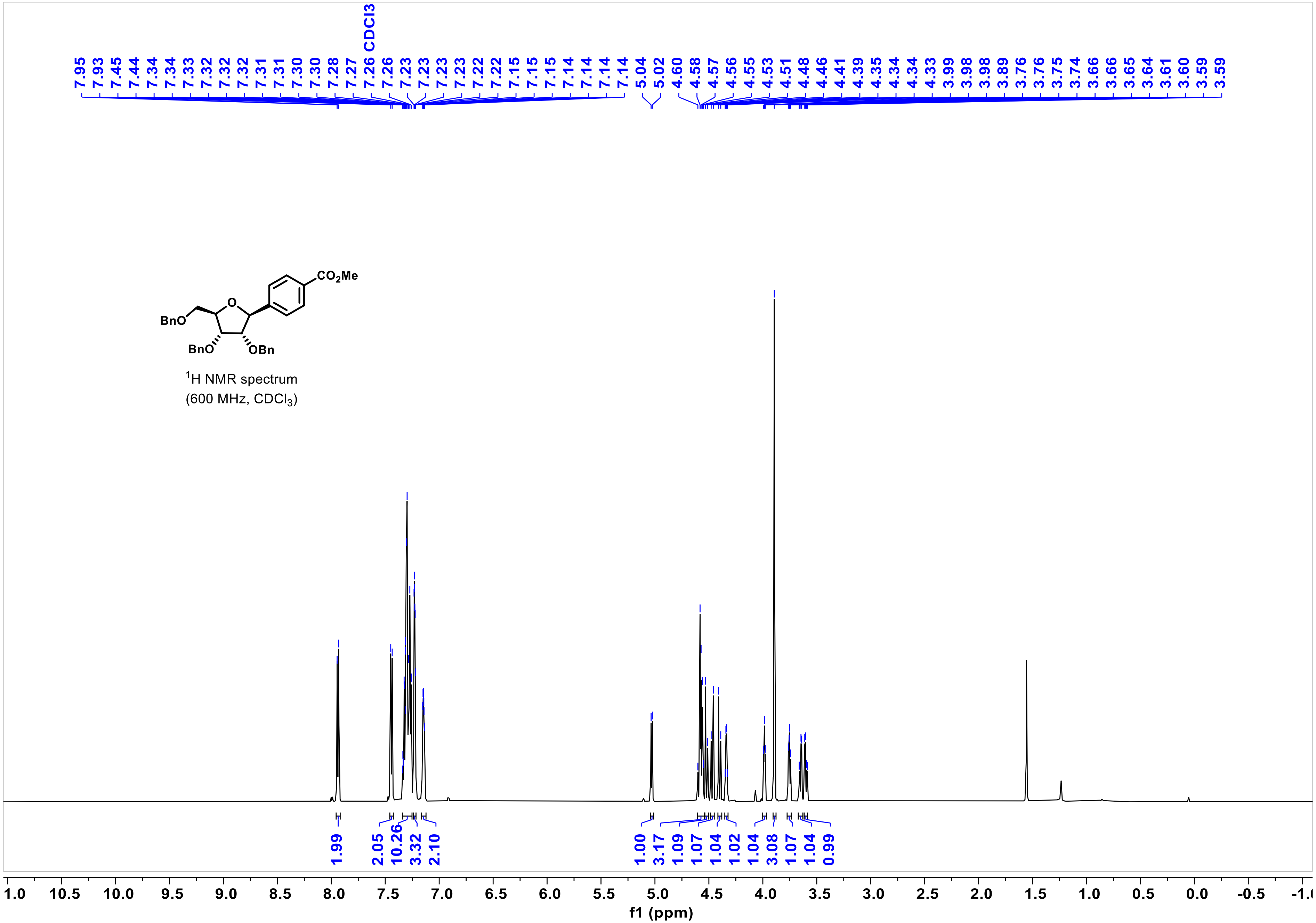

^{1}H NMR of compound **SD4**

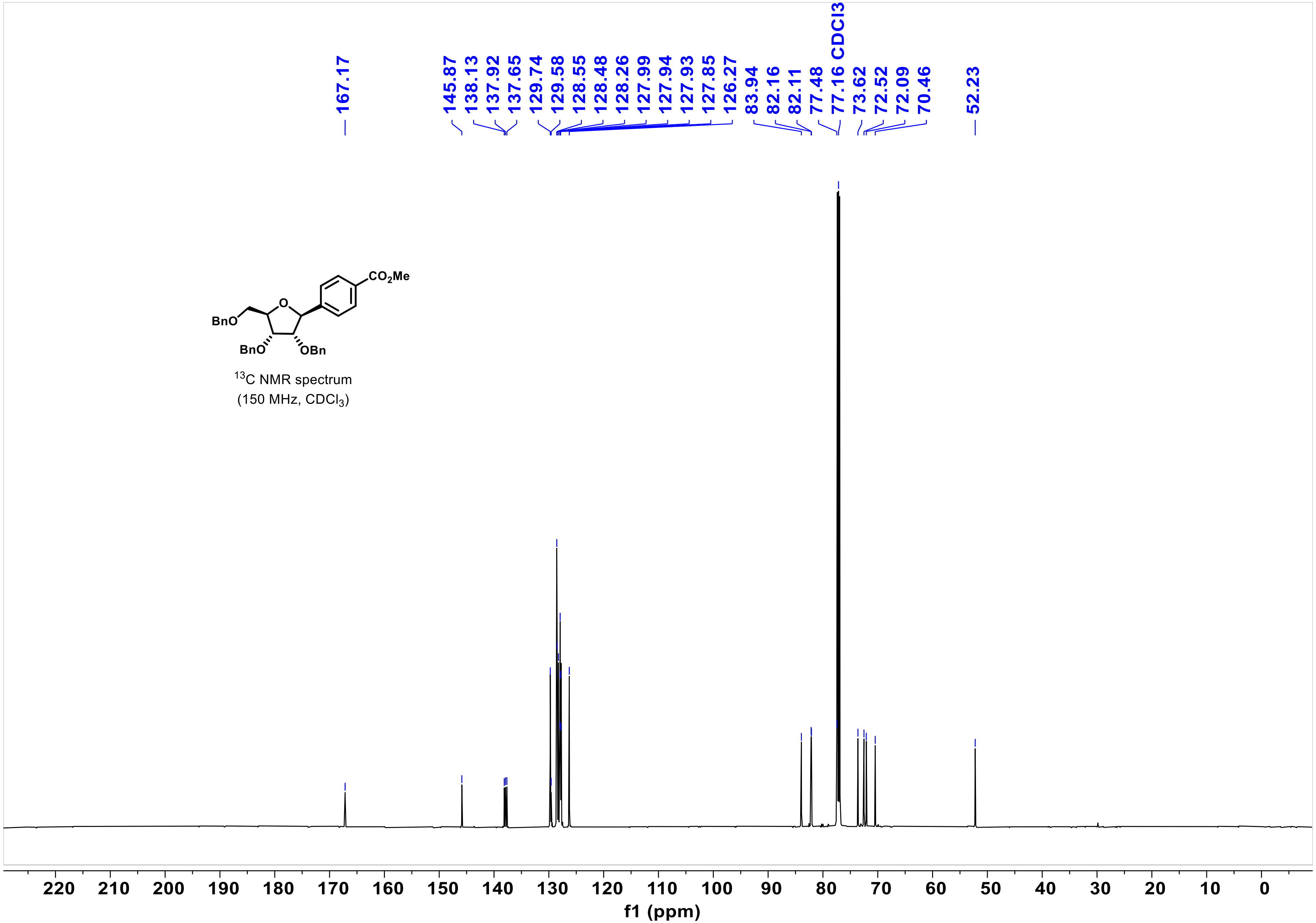

^{13}C NMR of compound **SD4**

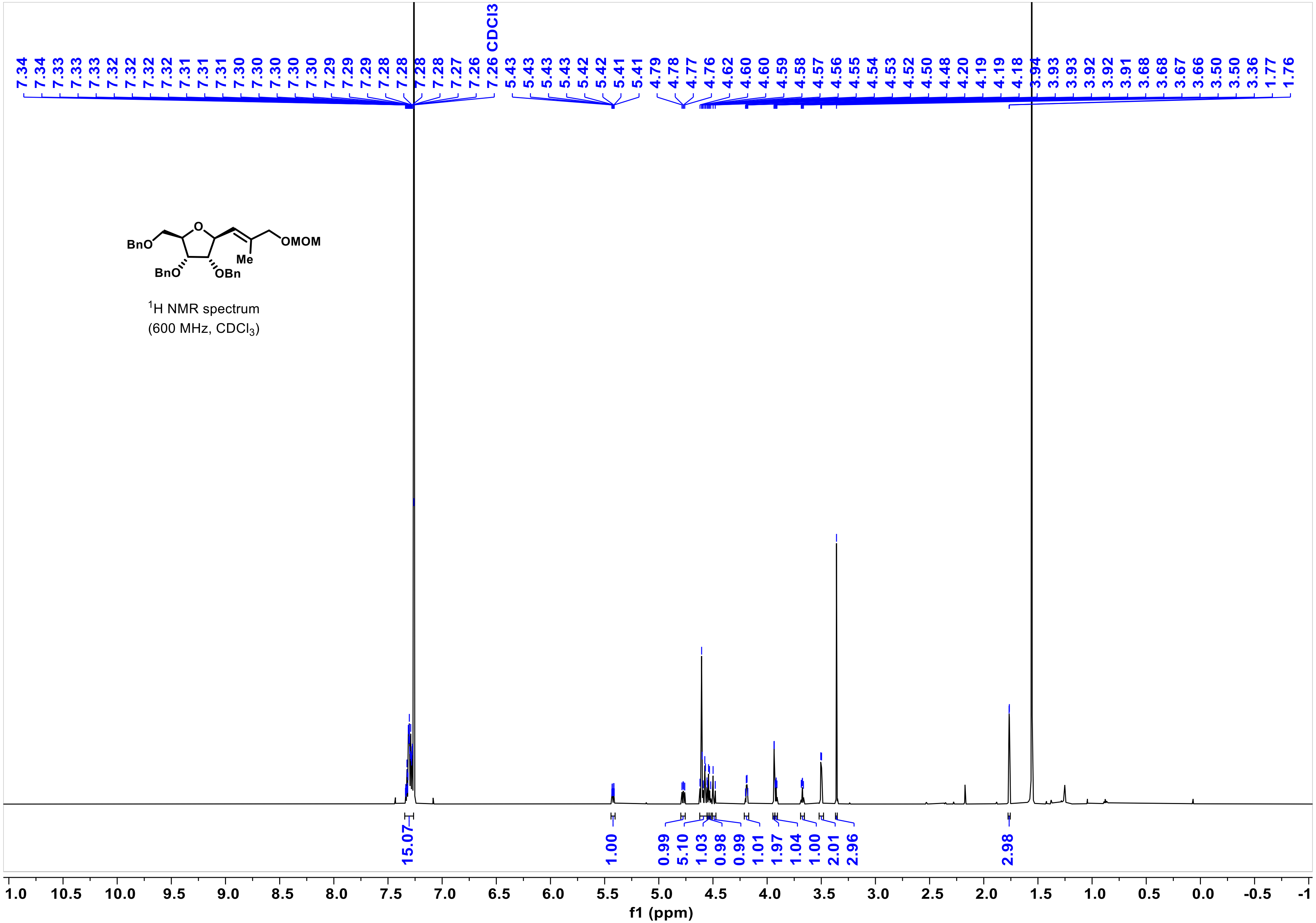

^{1}H NMR of compound **SD6**

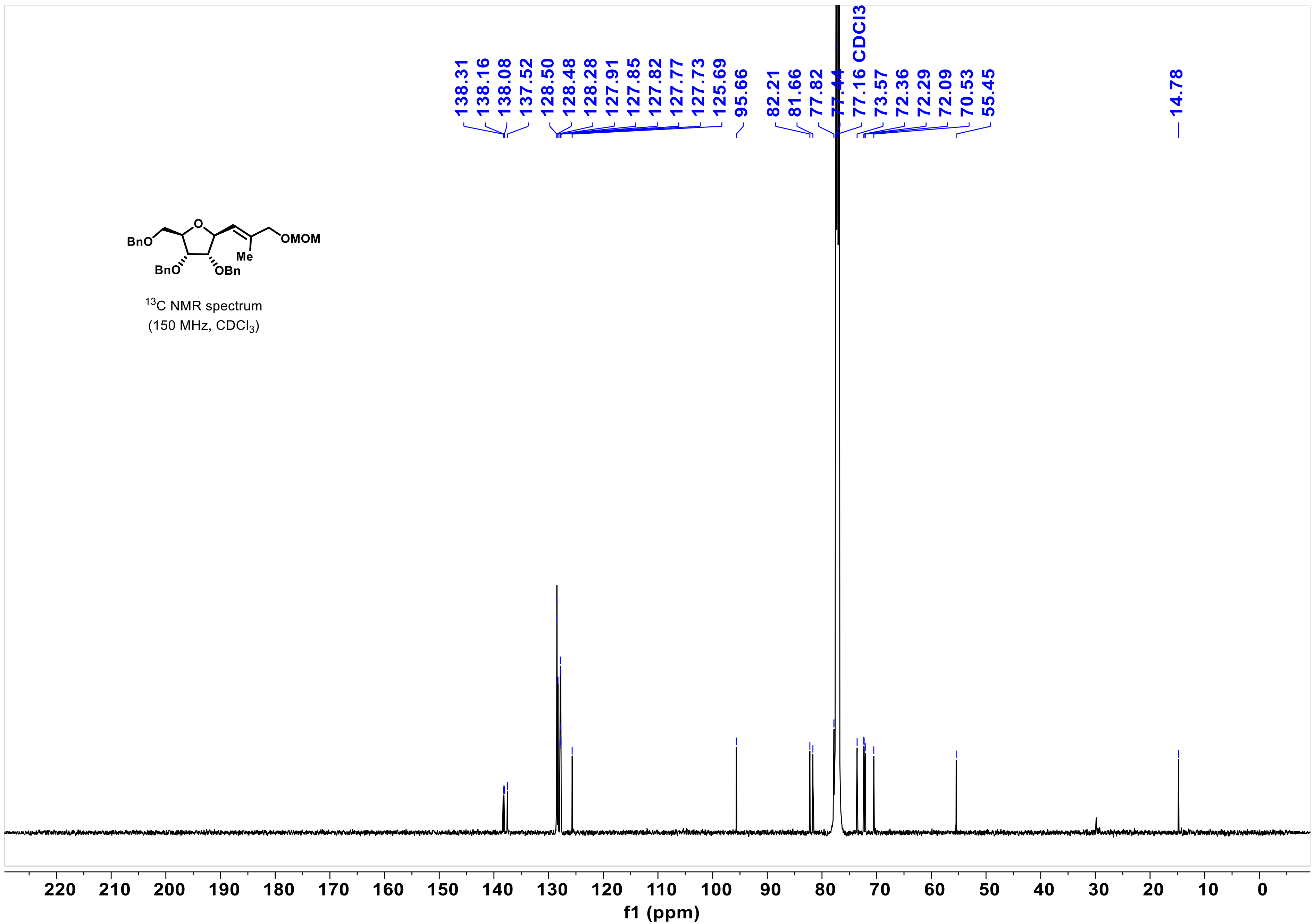

^{13}C NMR of compound **SD6**

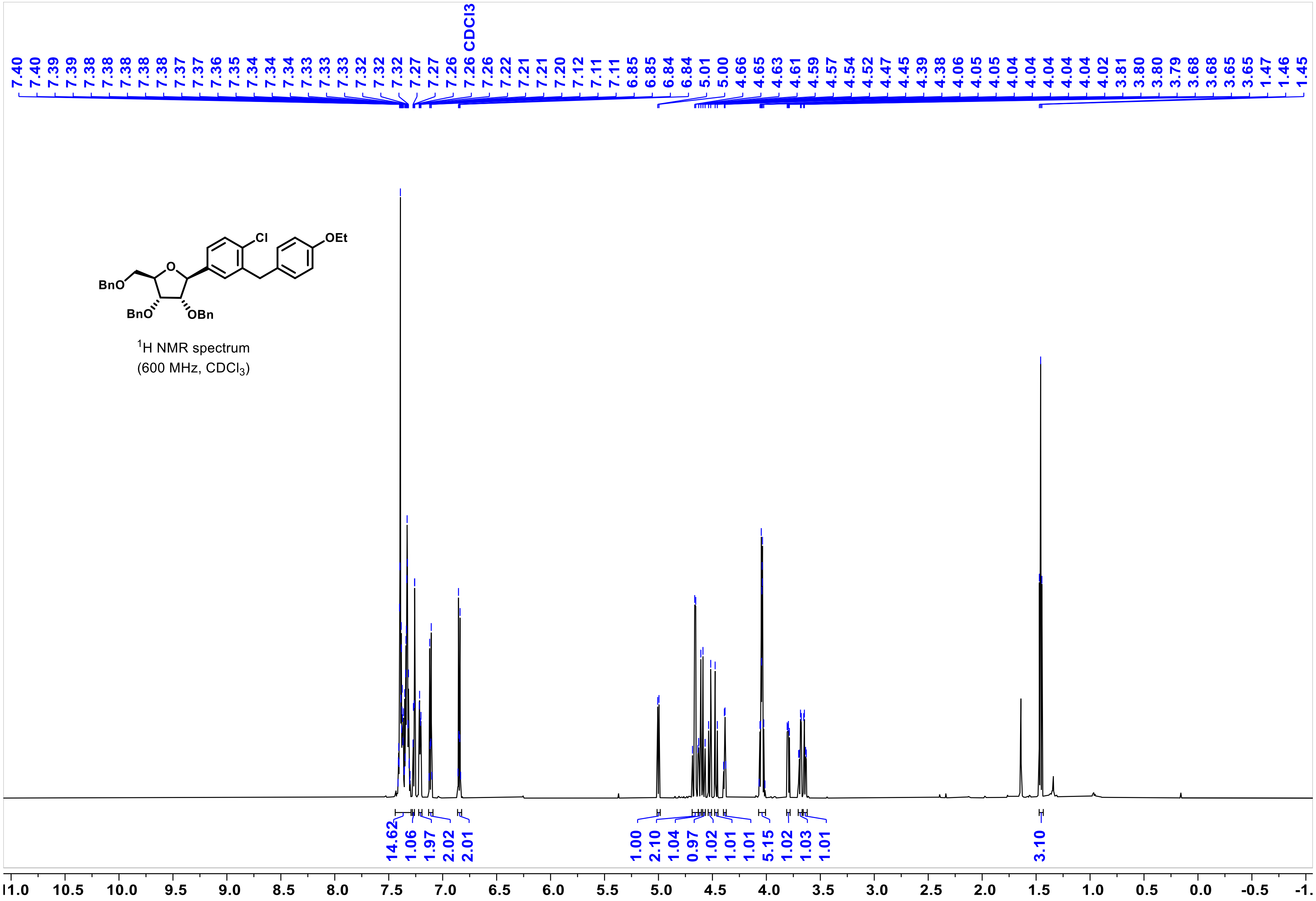

^{1}H NMR of compound **SD7**

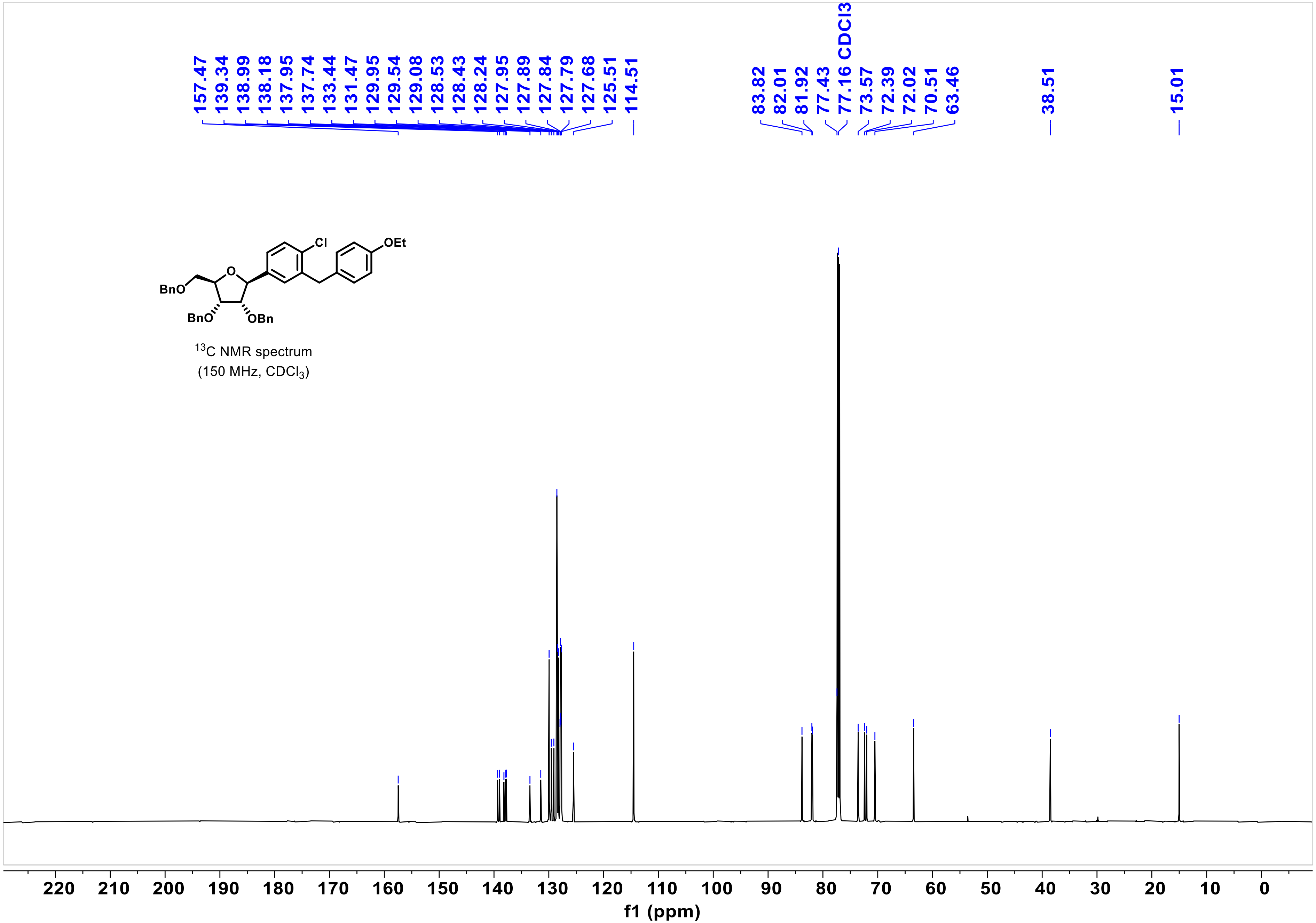

^{13}C NMR of compound **SD7**

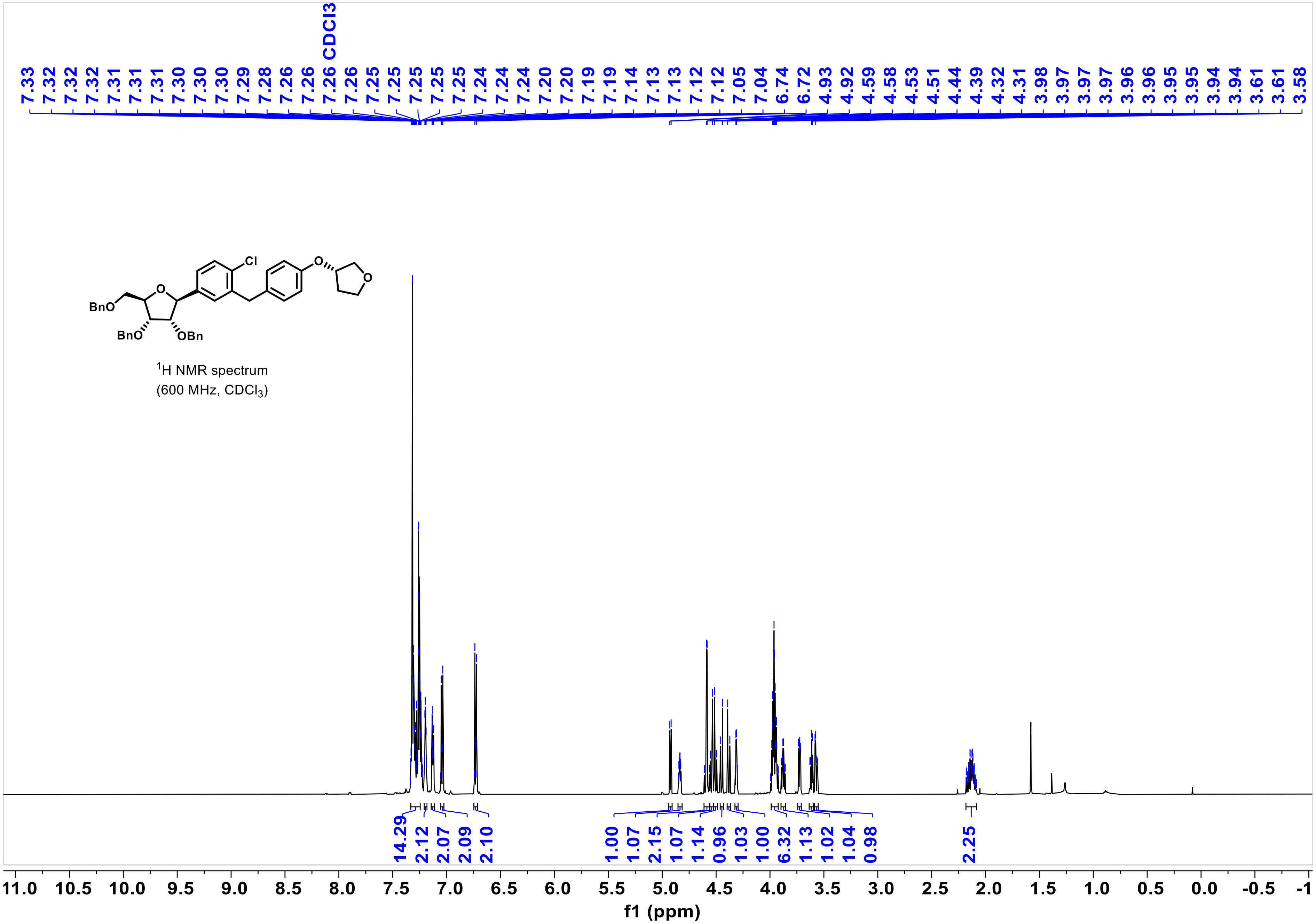

^{1}H NMR of compound **SD8**

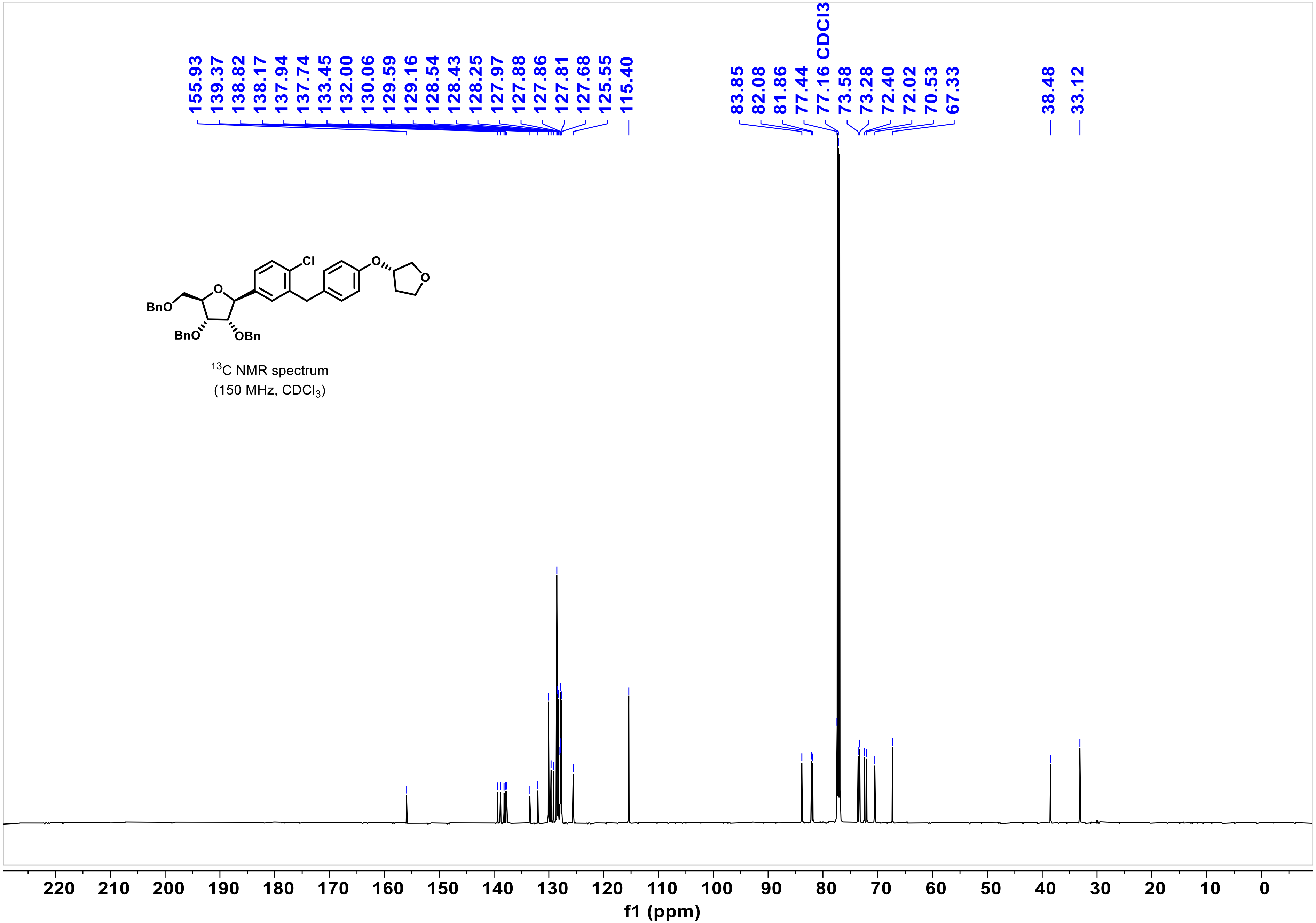

^{13}C NMR of compound **SD8**

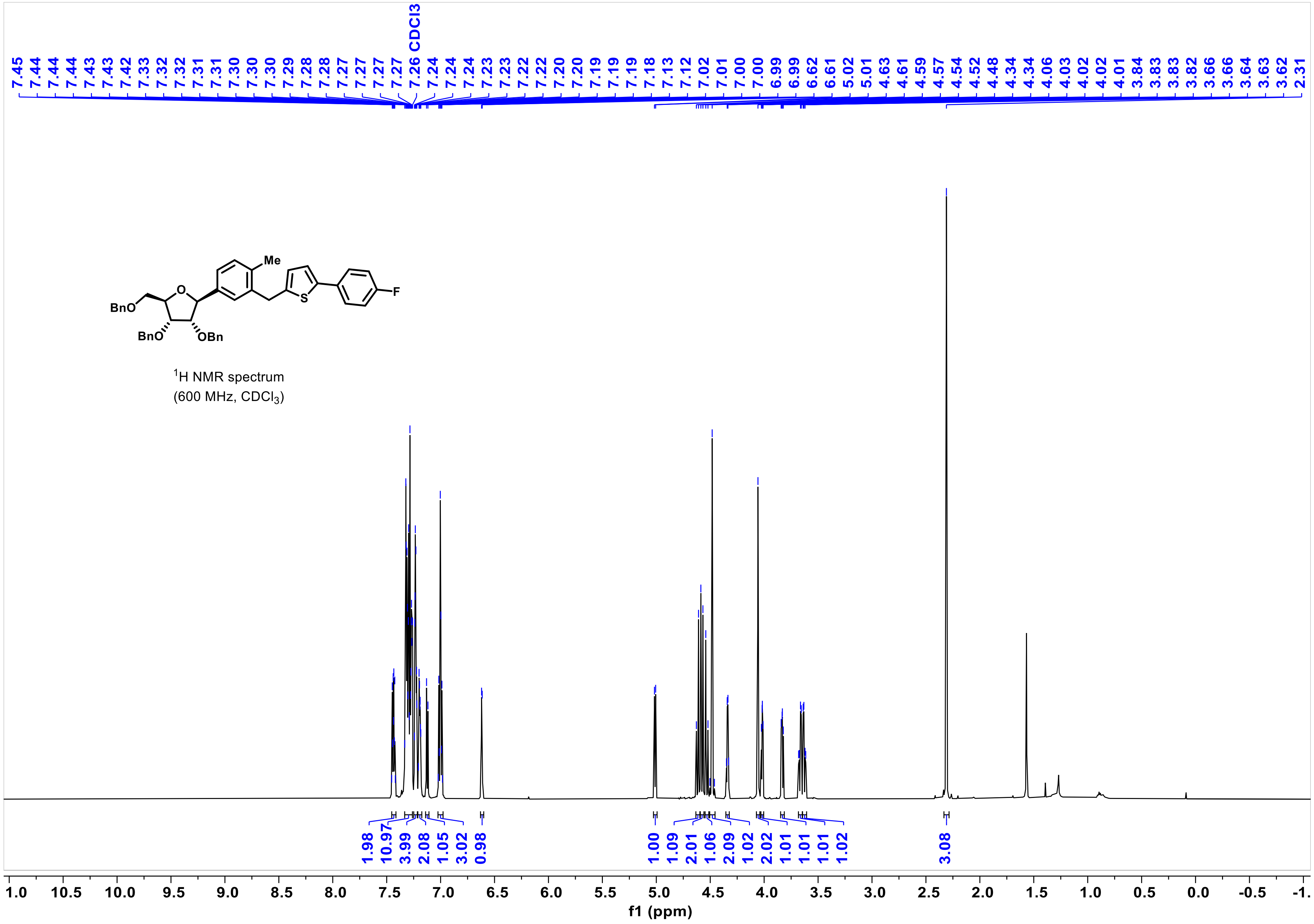

^{1}H NMR of compound **SD9**

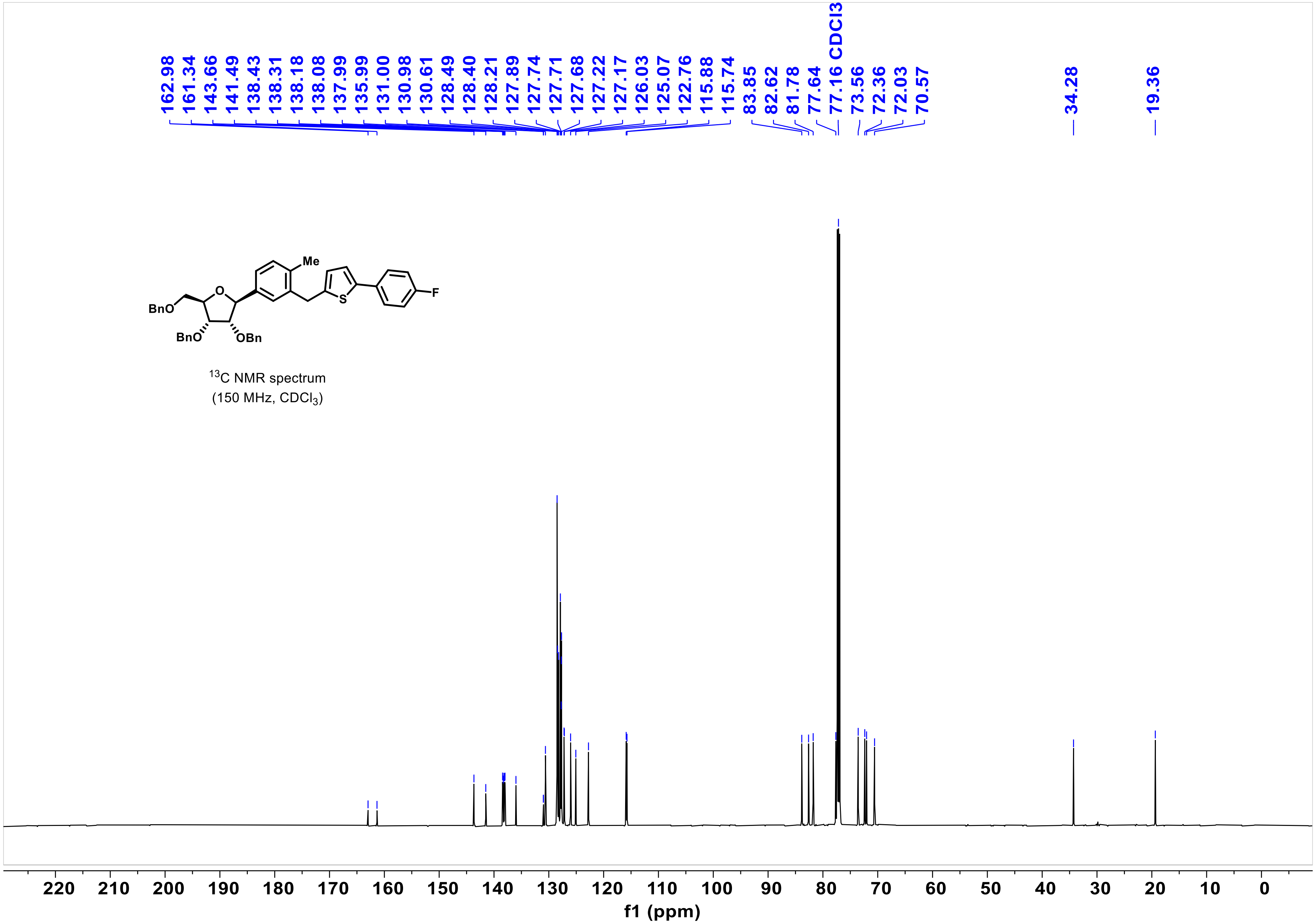

^{13}C NMR of compound **SD9**

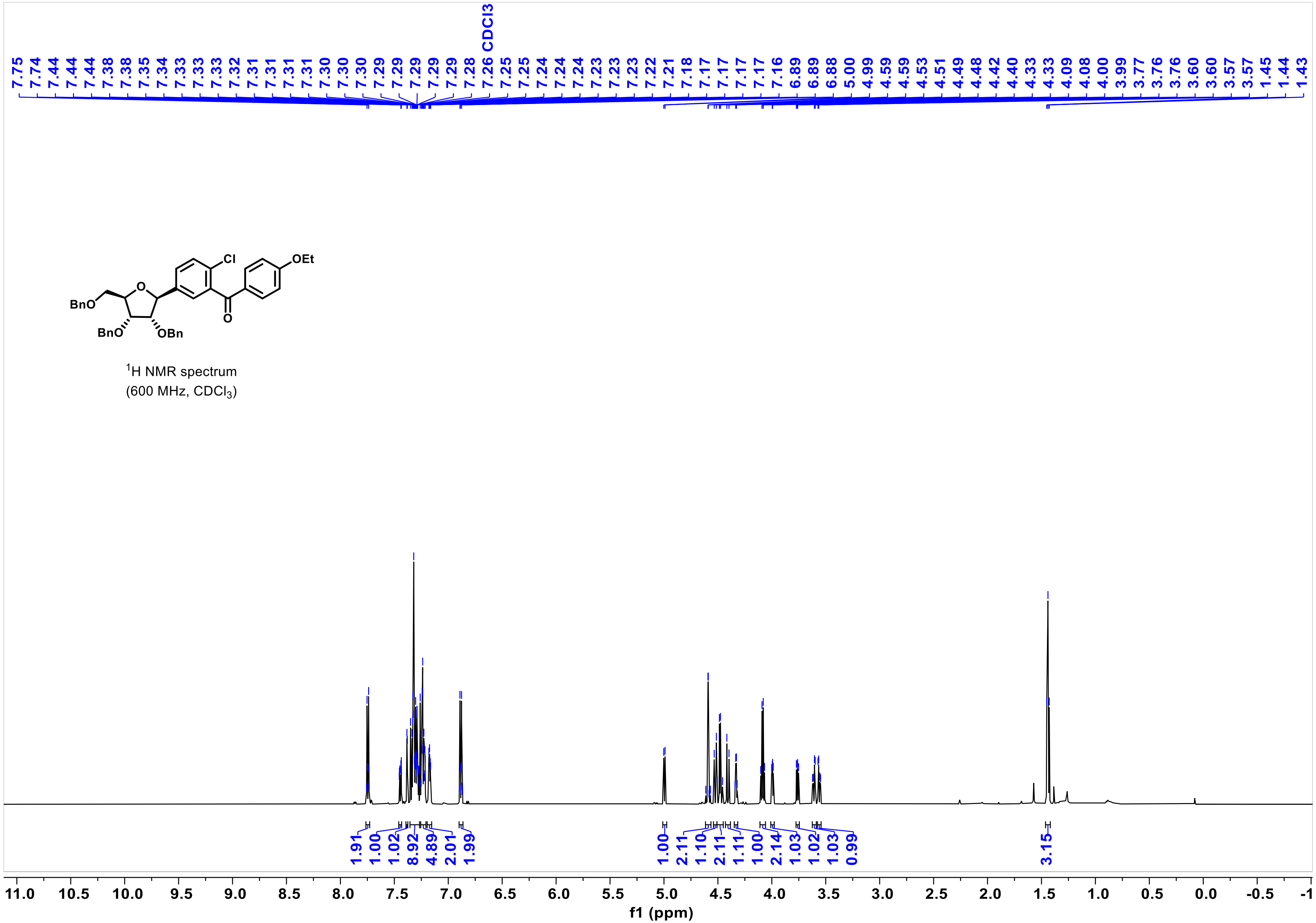

^{1}H NMR of compound **SD10**

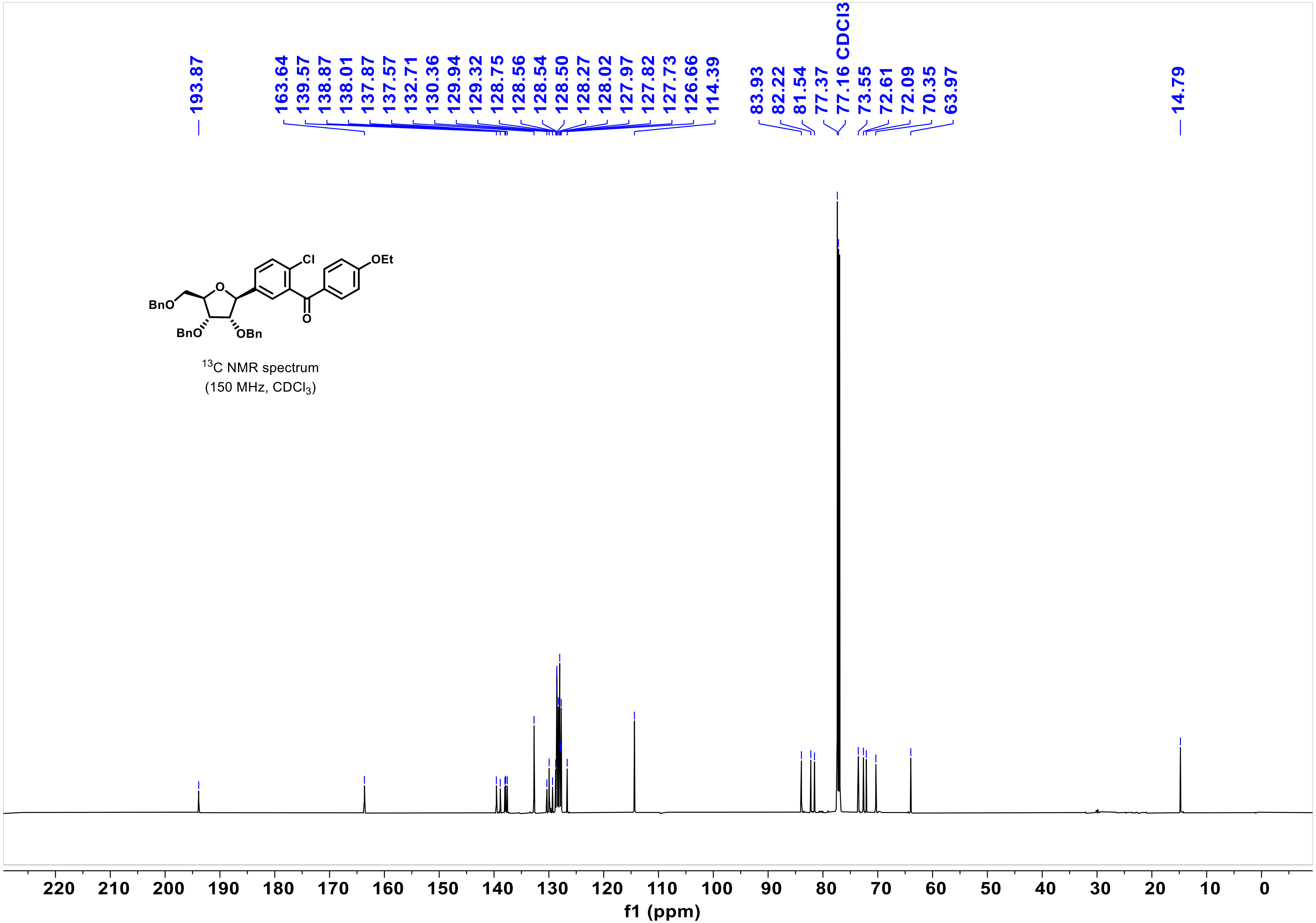

^{13}C NMR of compound **SD10**

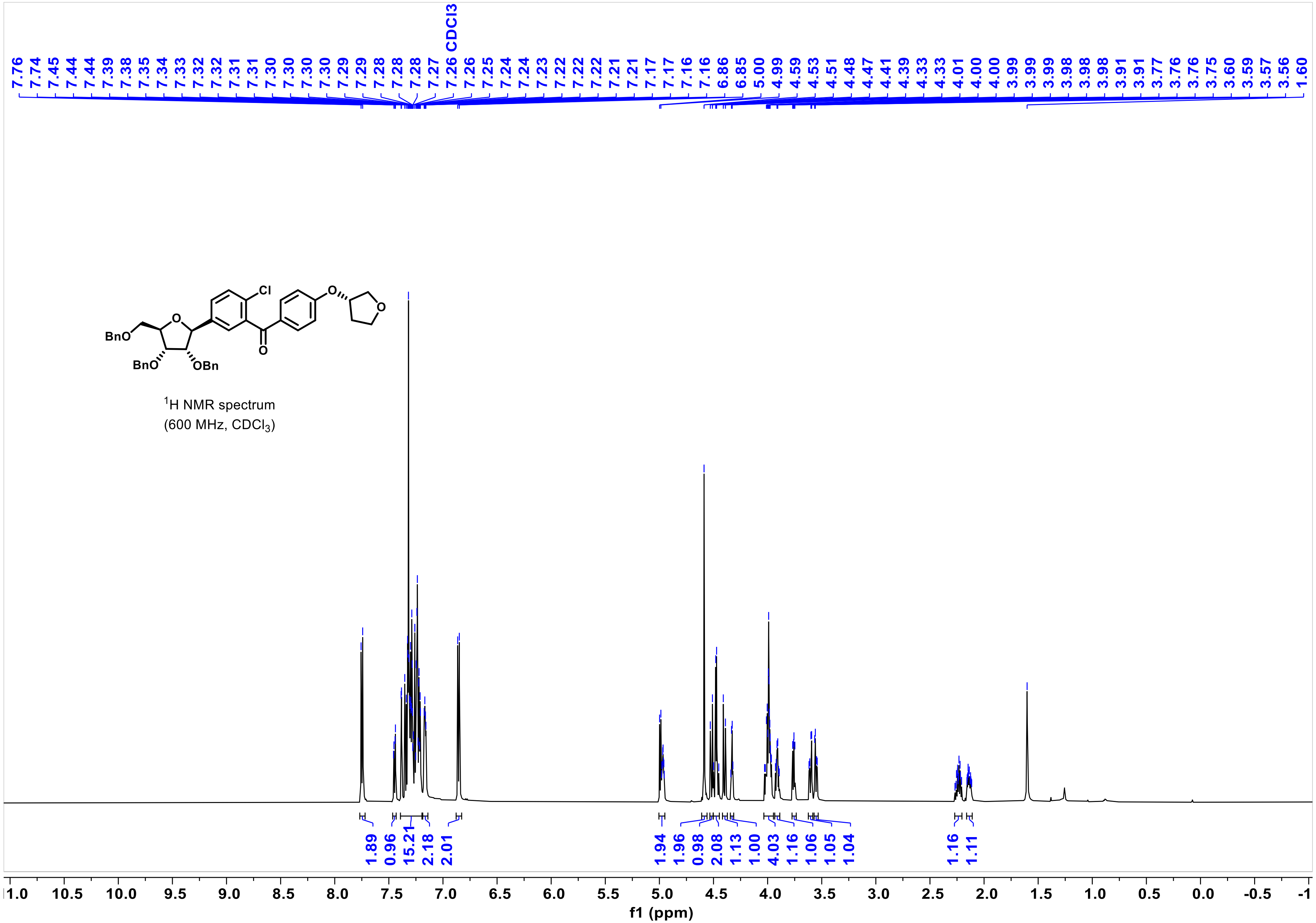

^{1}H NMR of compound **SD11**

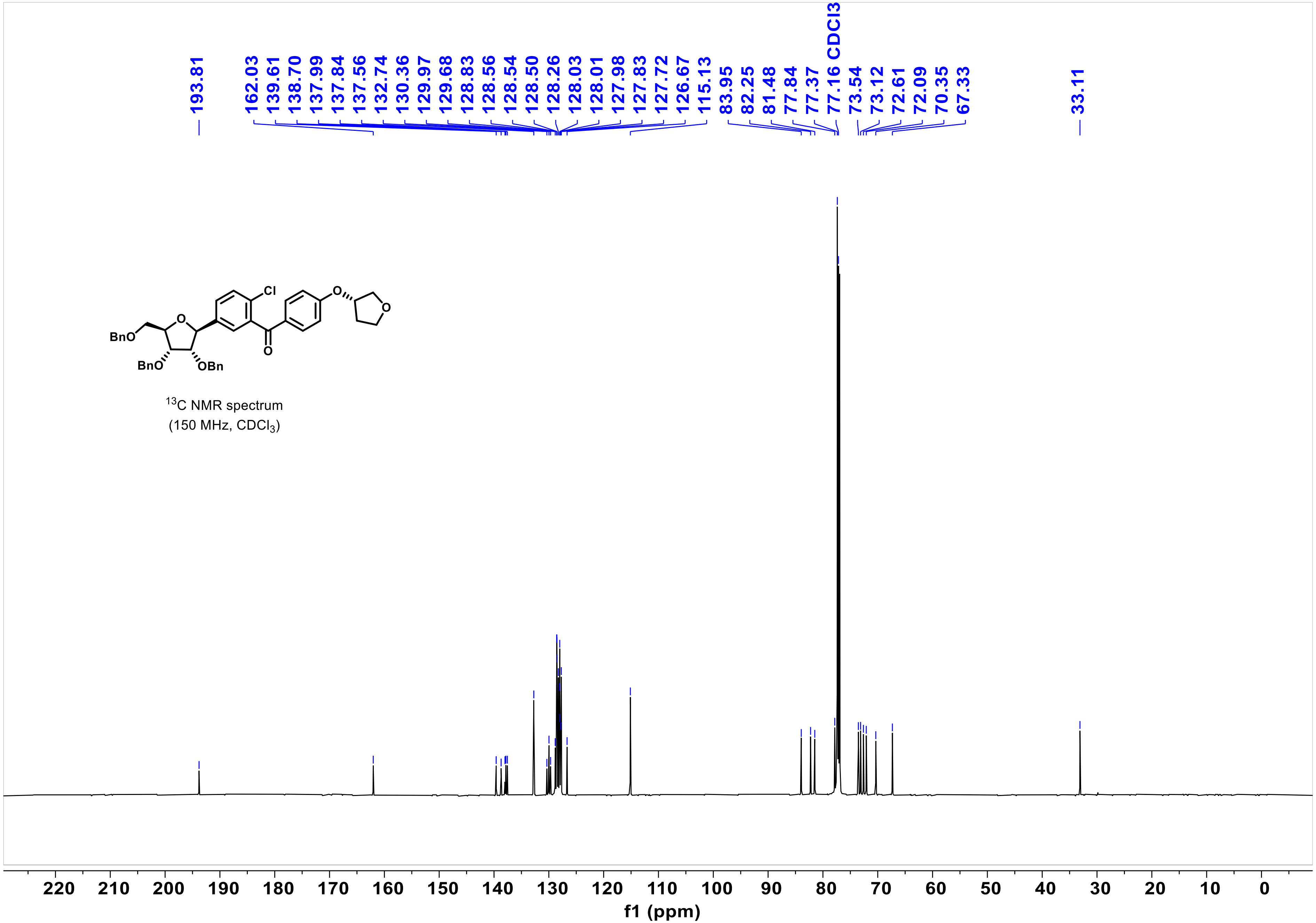

^{13}C NMR of compound **SD11**

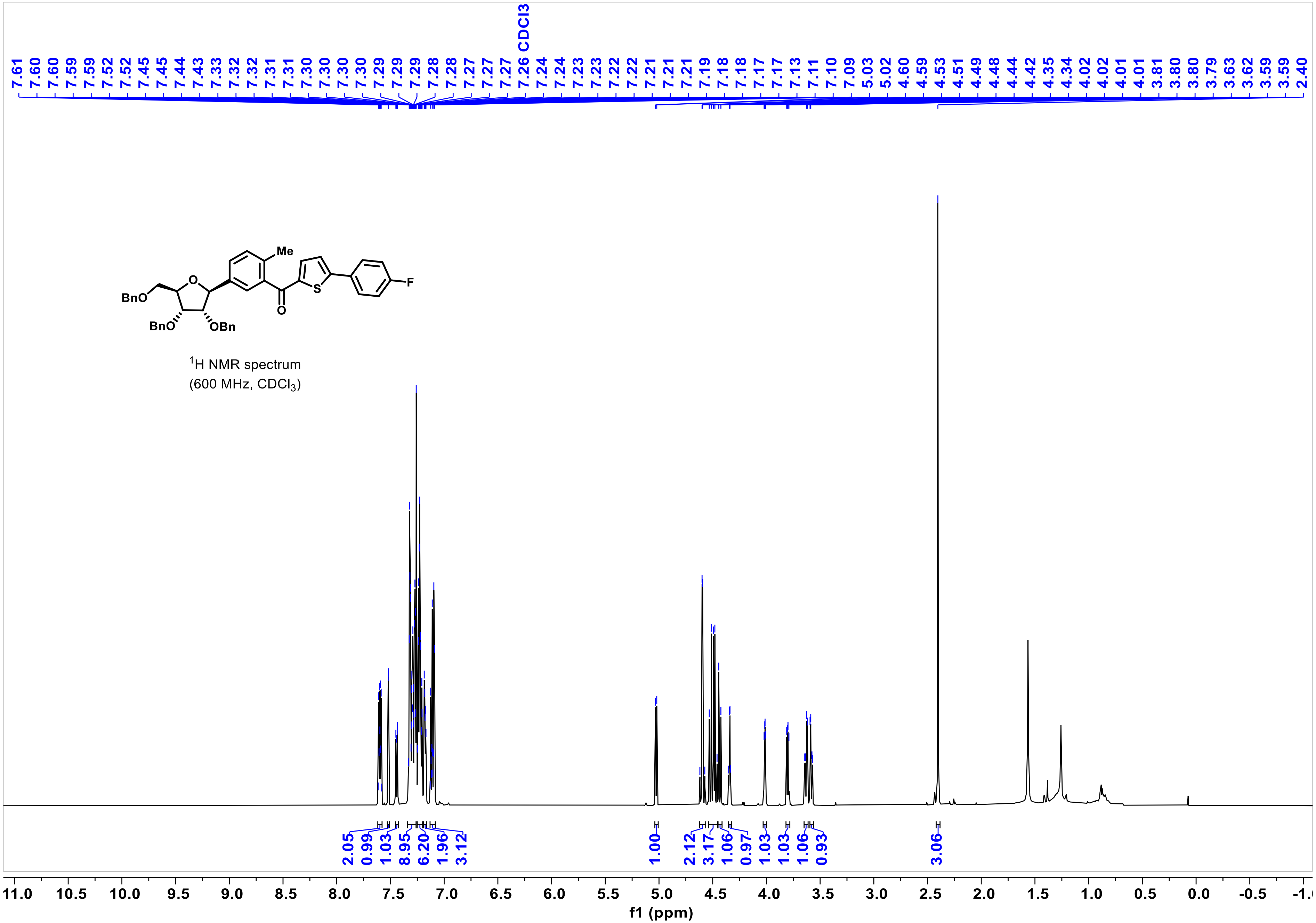

^{1}H NMR of compound **SD12**

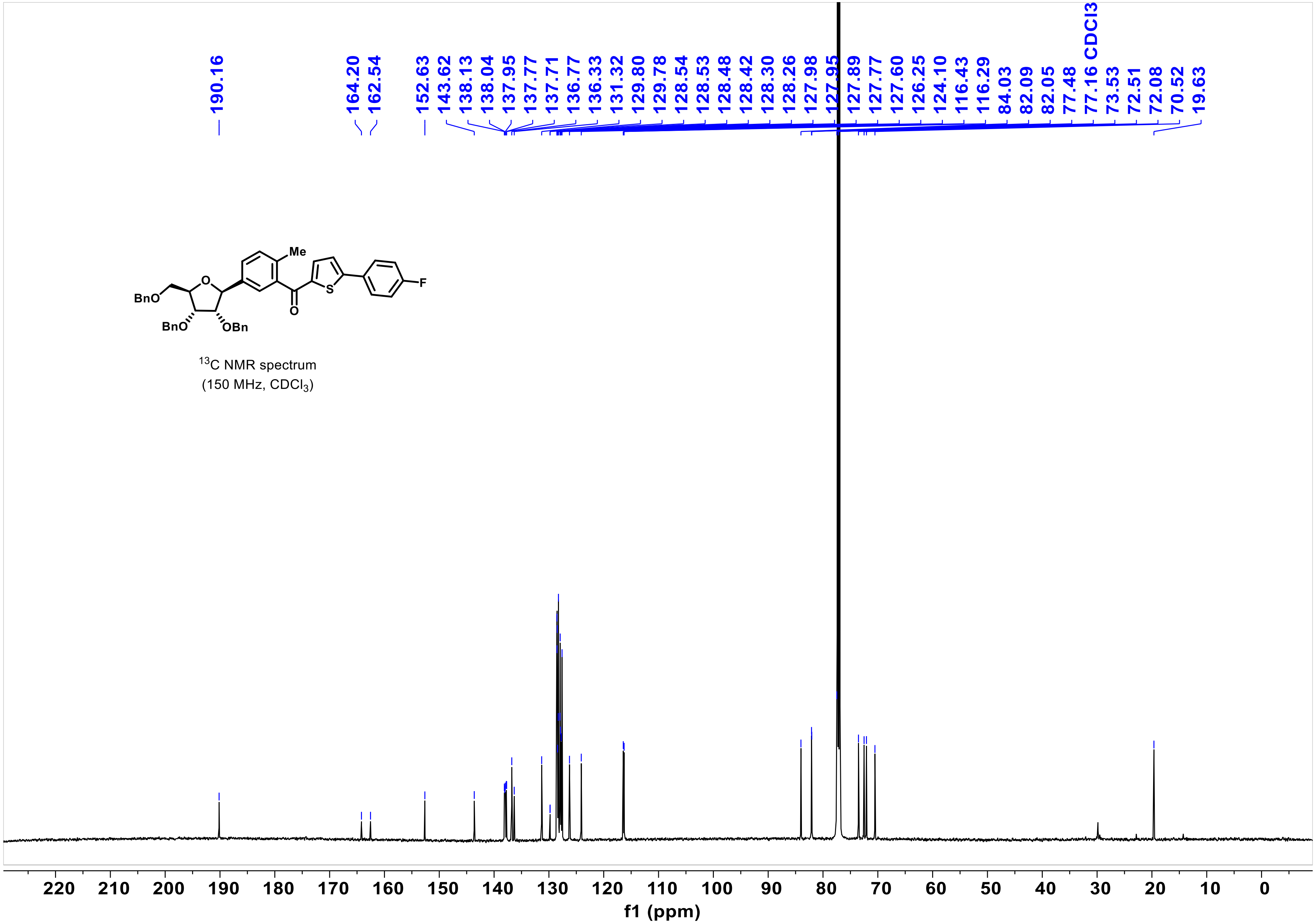

^{13}C NMR of compound **SD12**

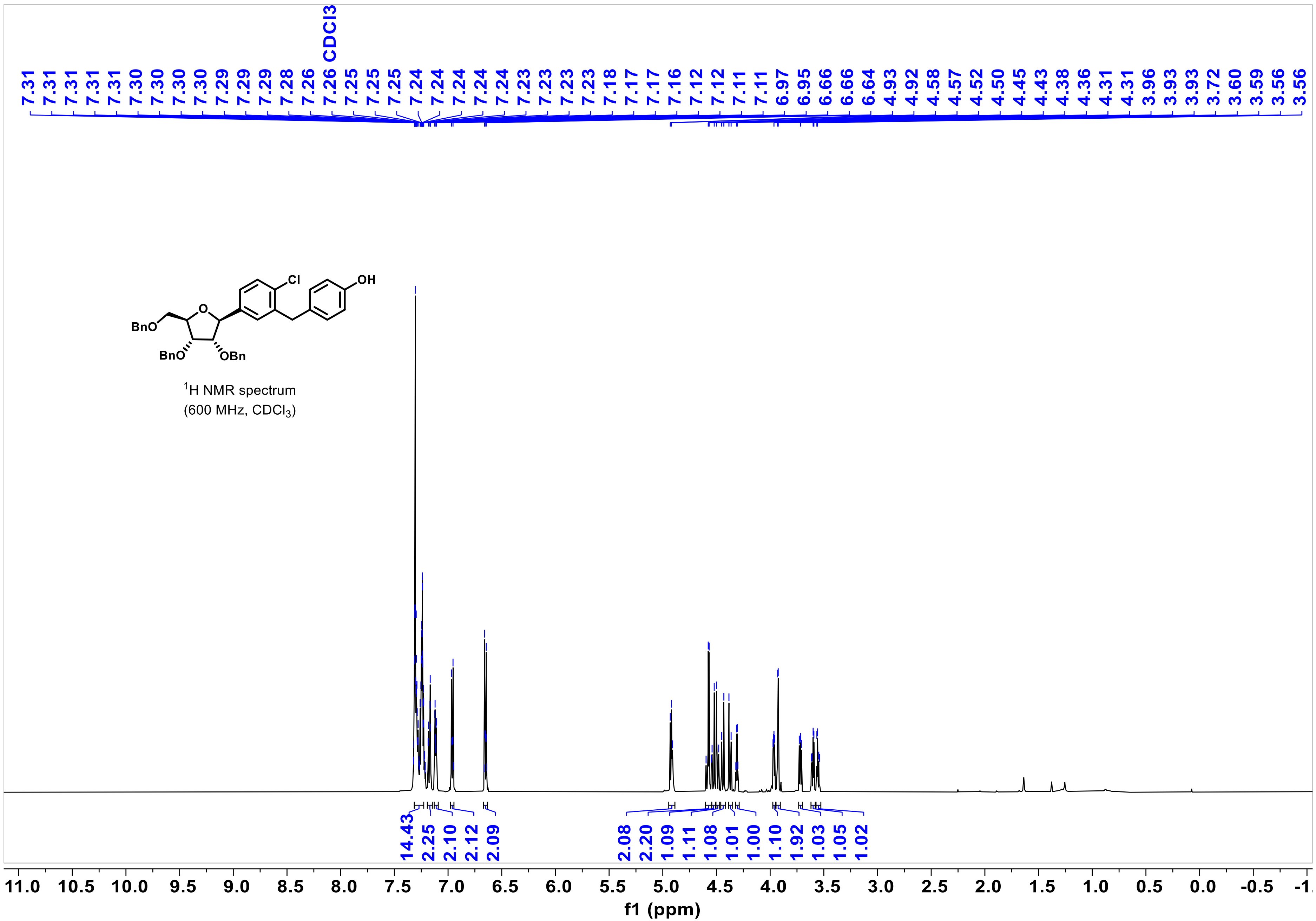

^{1}H NMR of compound **SD13**

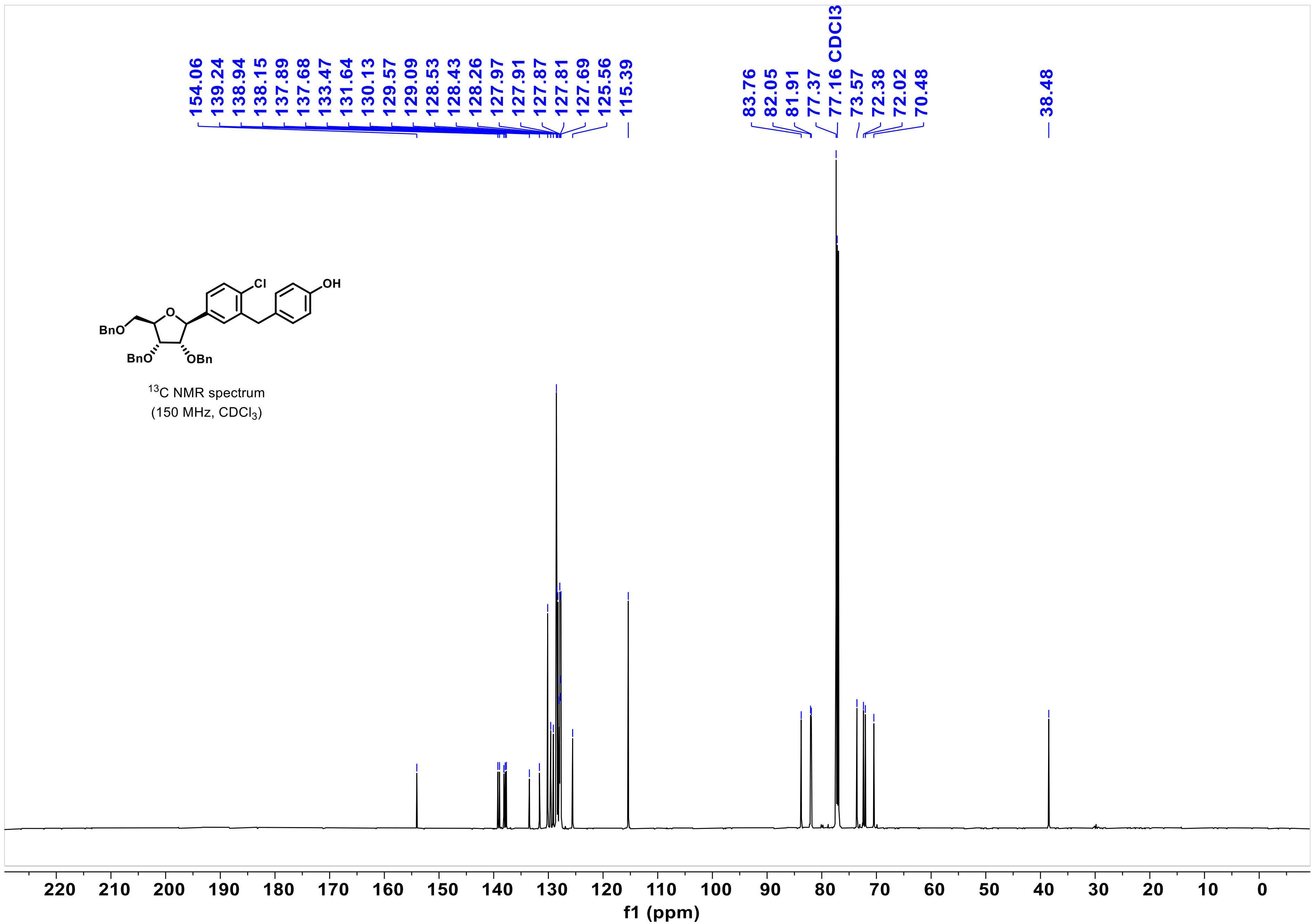

^{13}C NMR of compound **SD13**

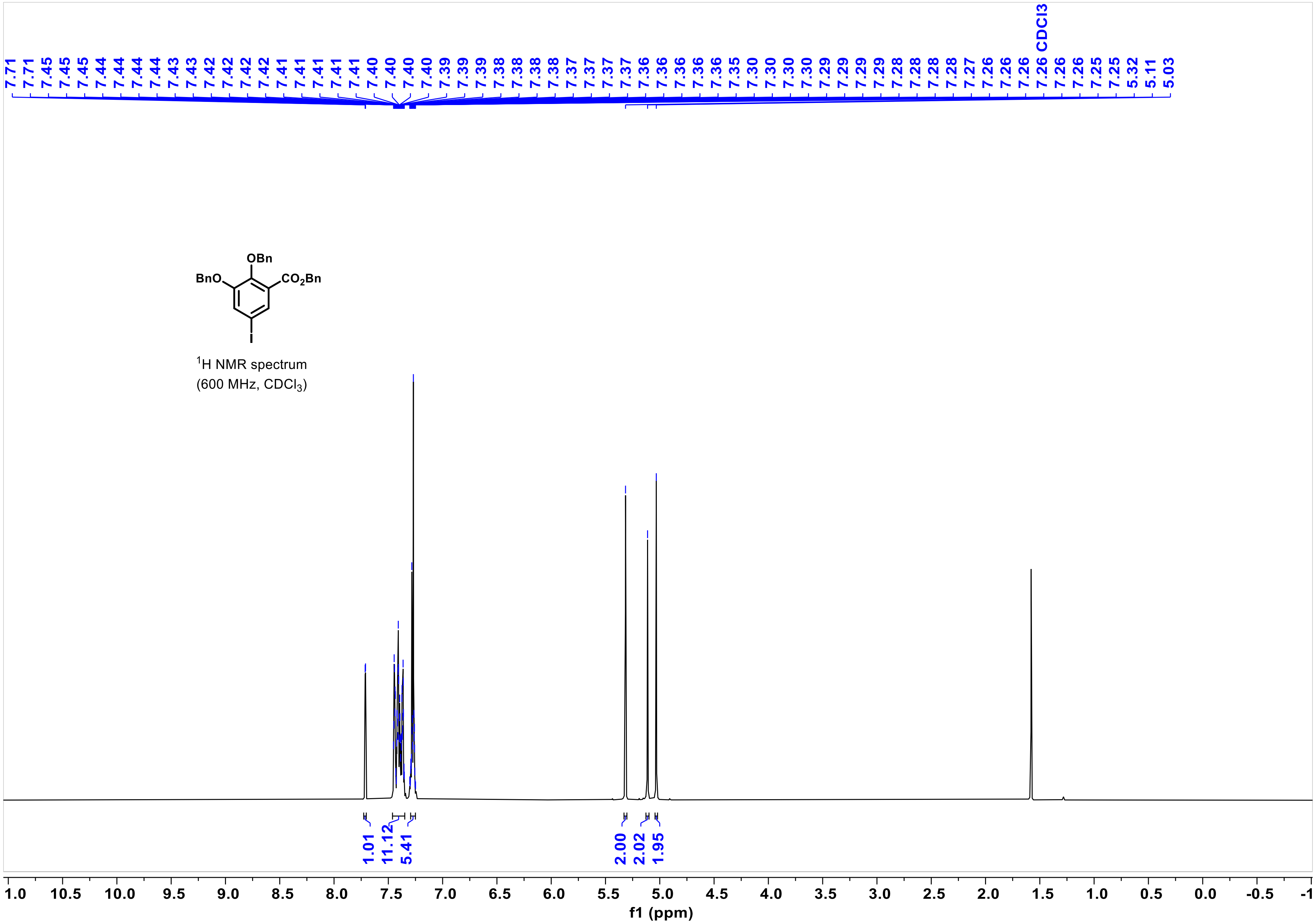

^{1}H NMR of compound **70**

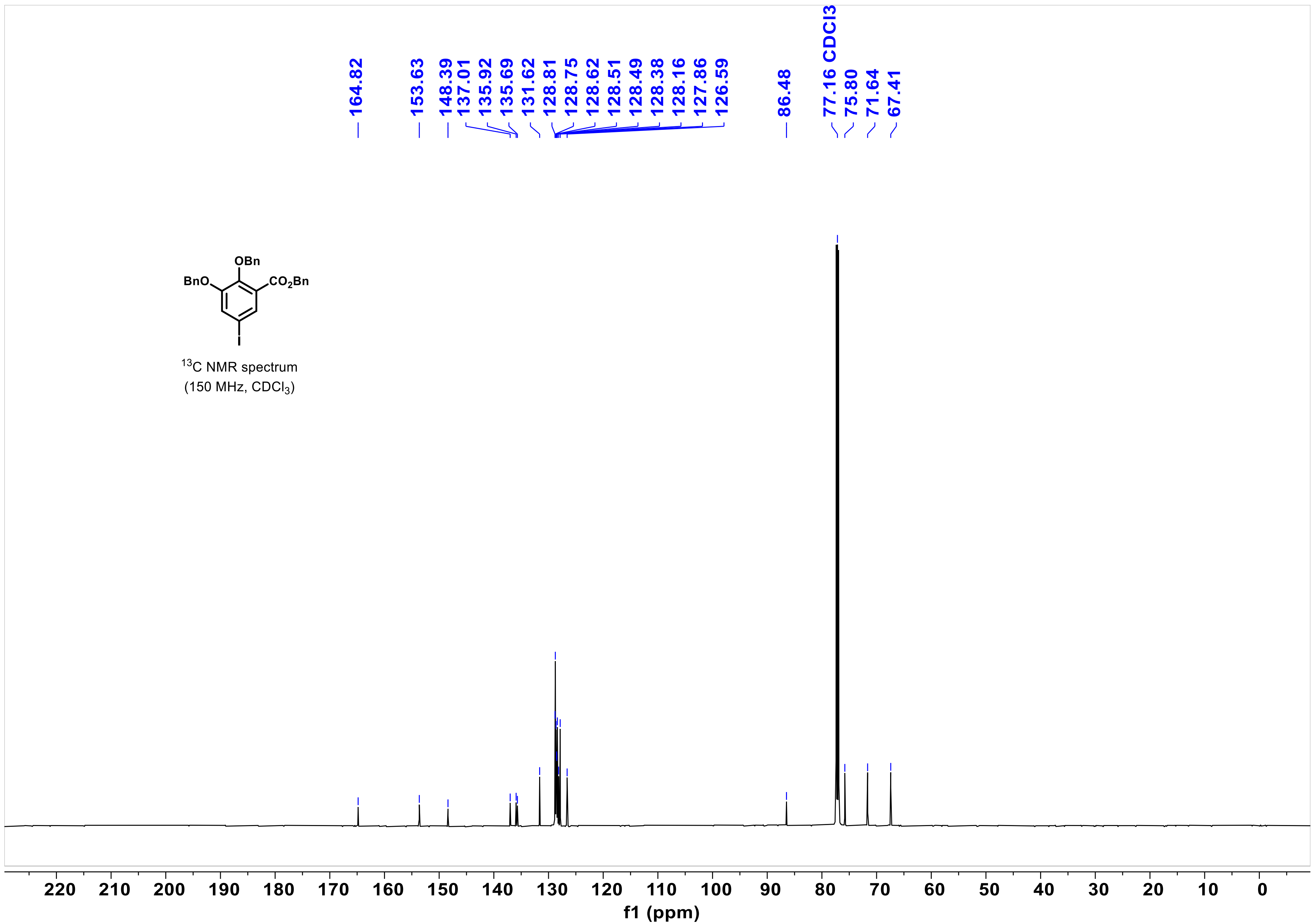

^{1}H NMR of compound **70**

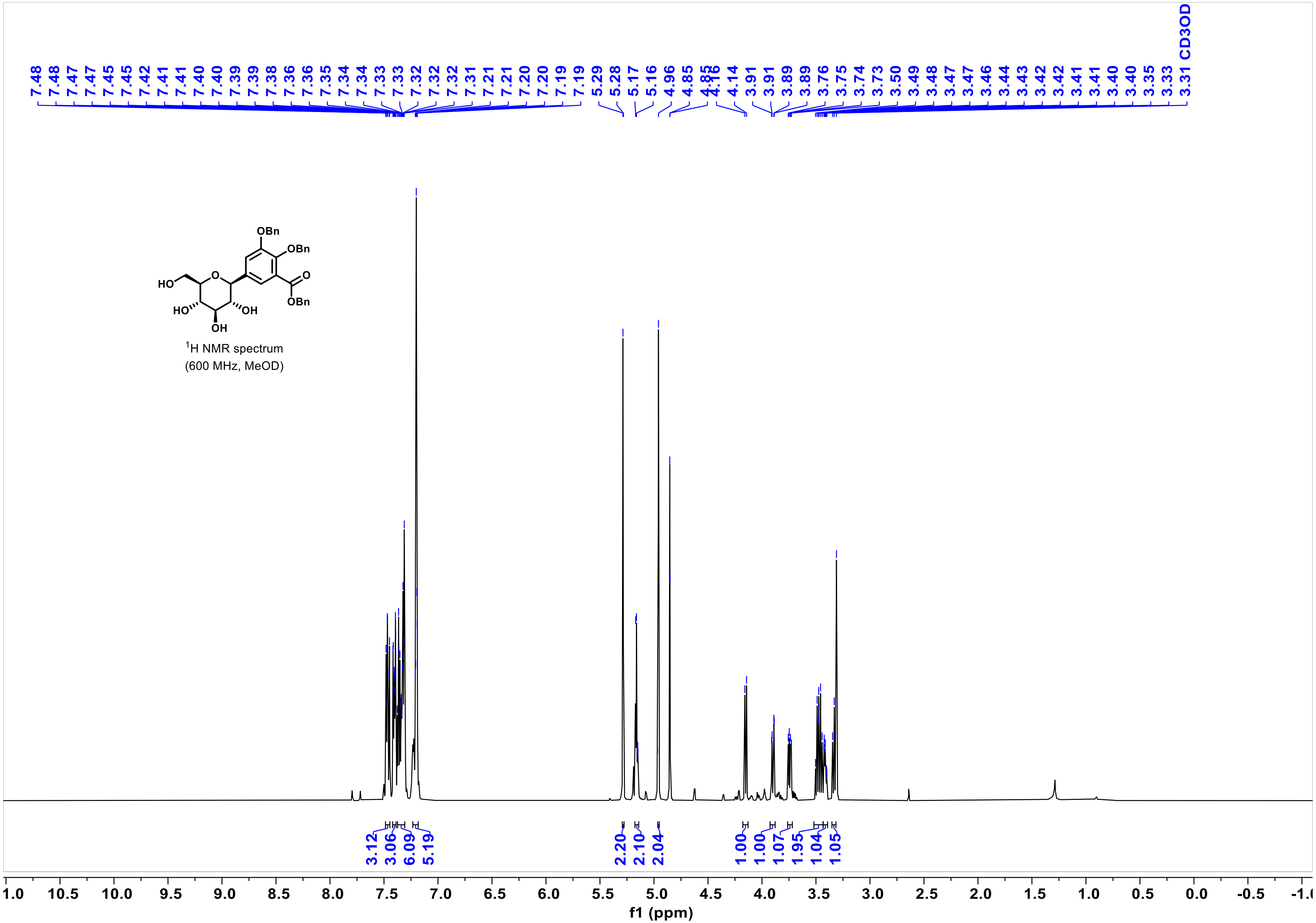

^{1}H NMR of compound **71**

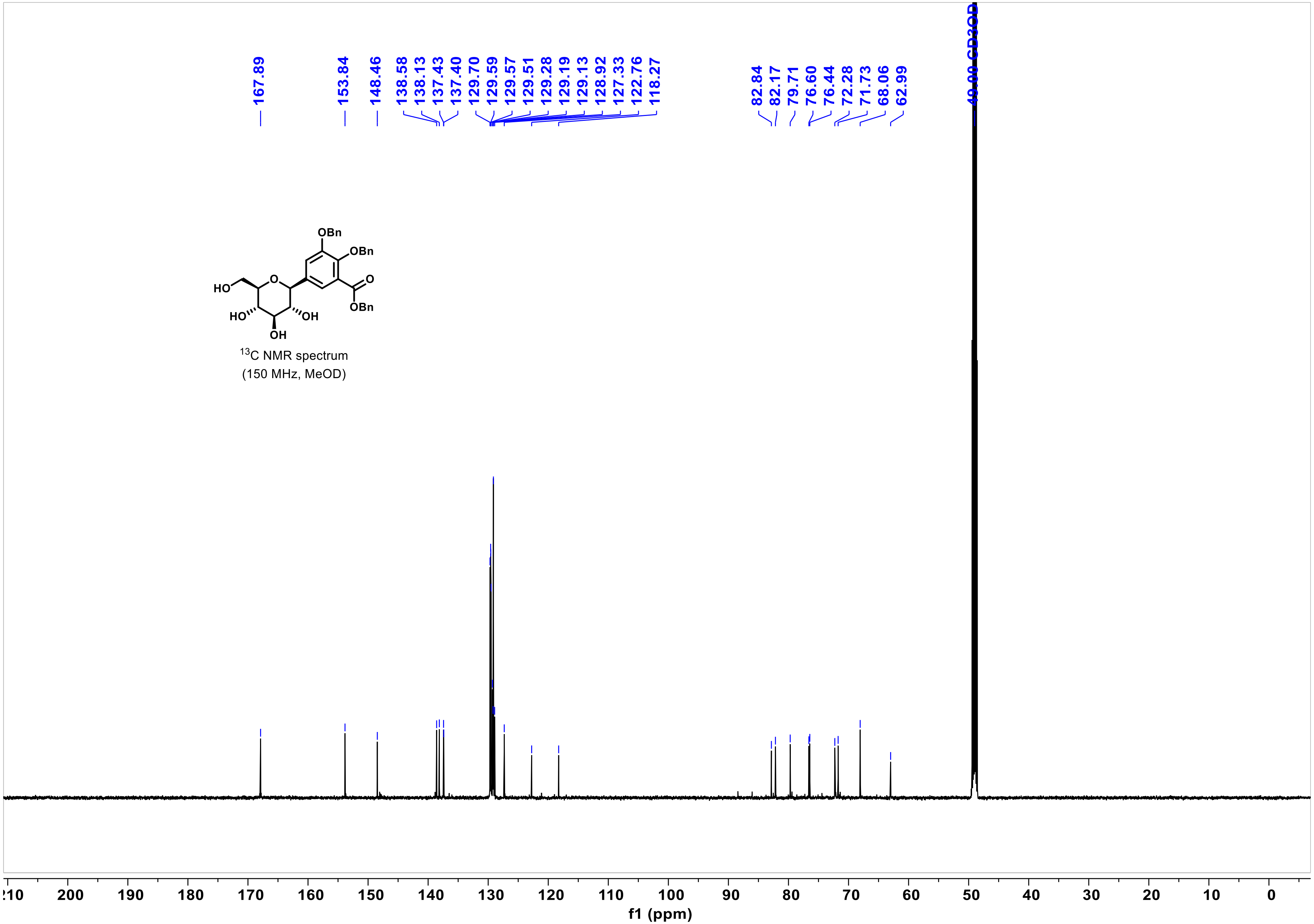

^{13}C NMR of compound **71**

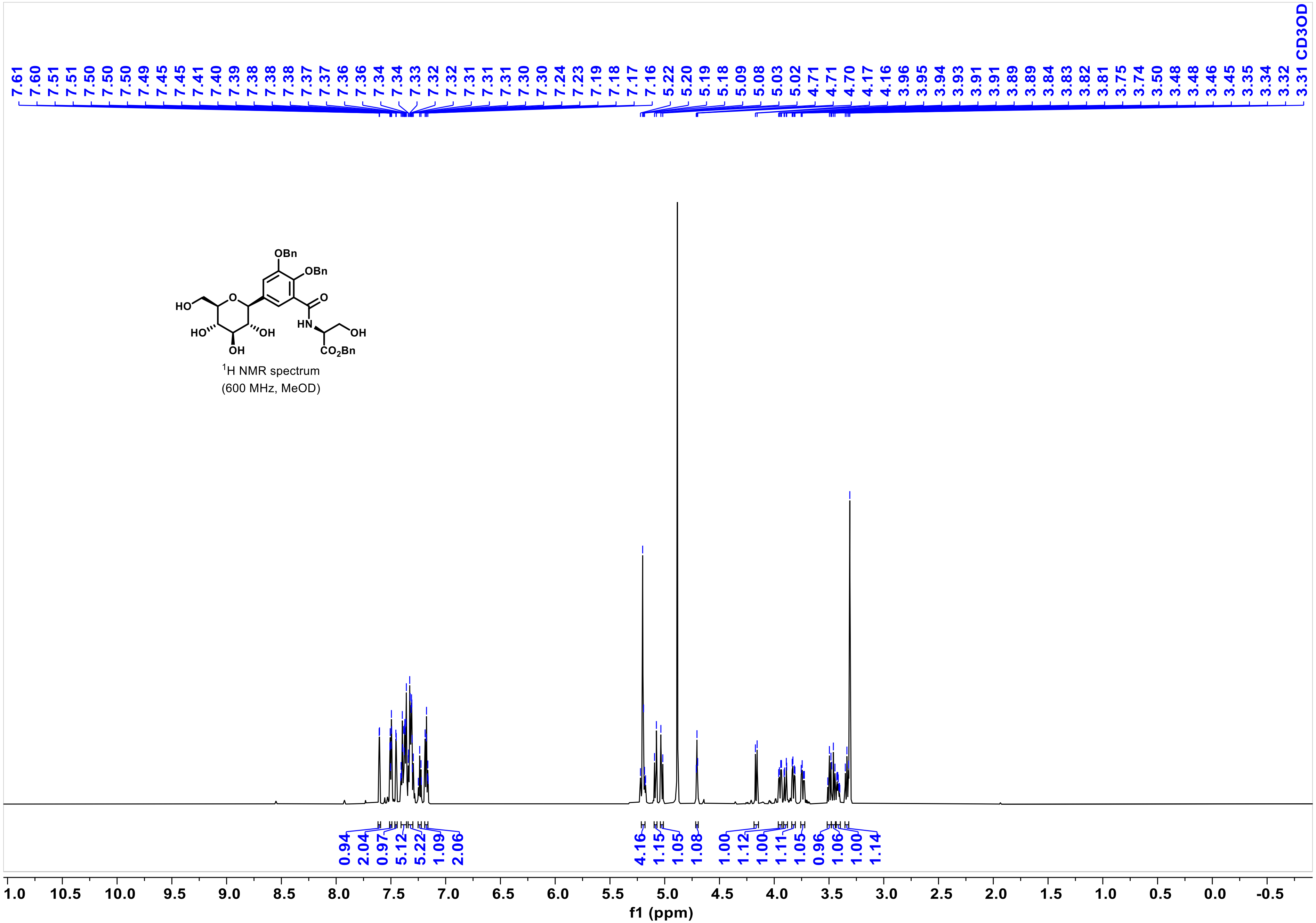

^{1}H NMR of compound **SI4**

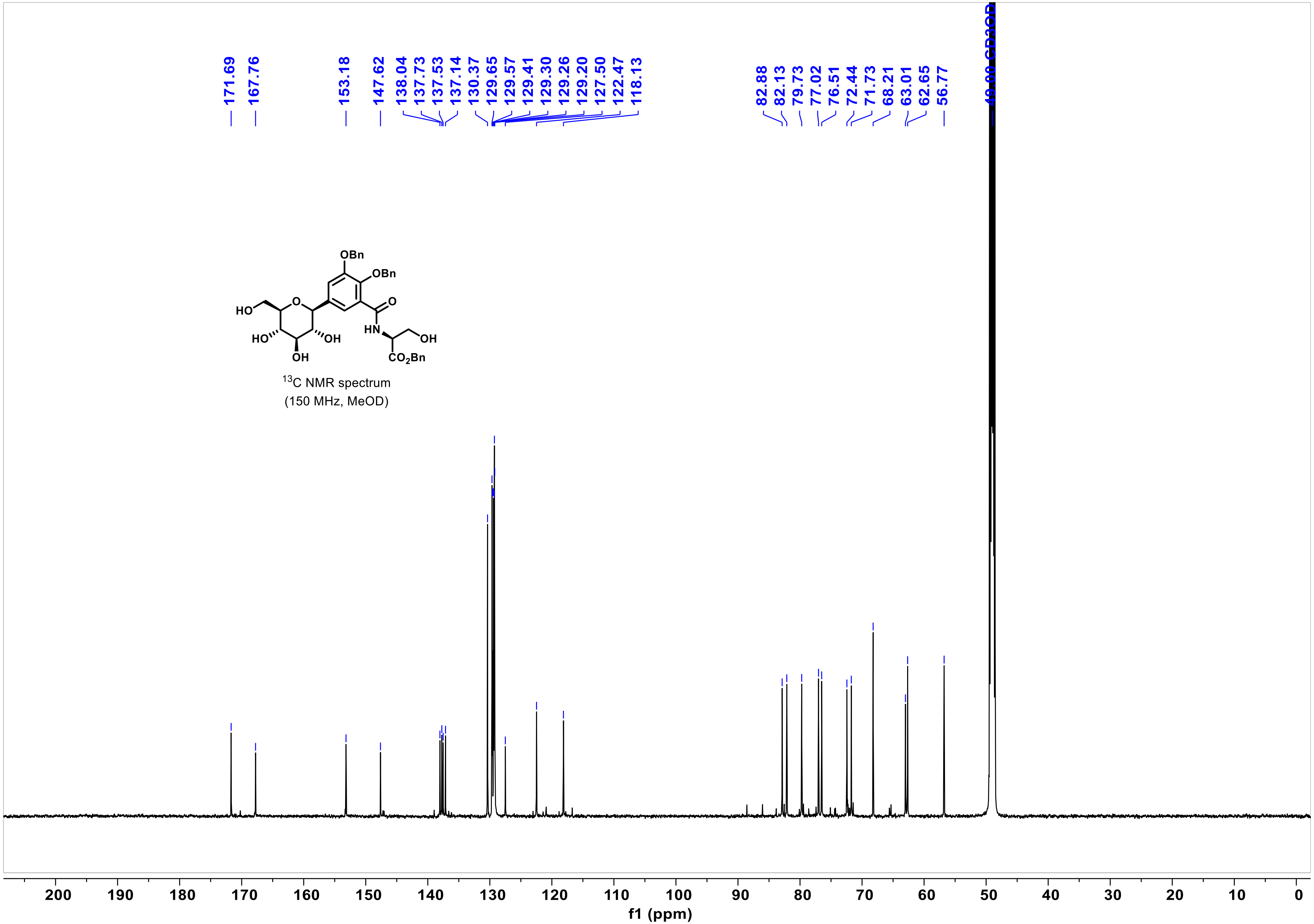

^{13}C NMR of compound **SI4**

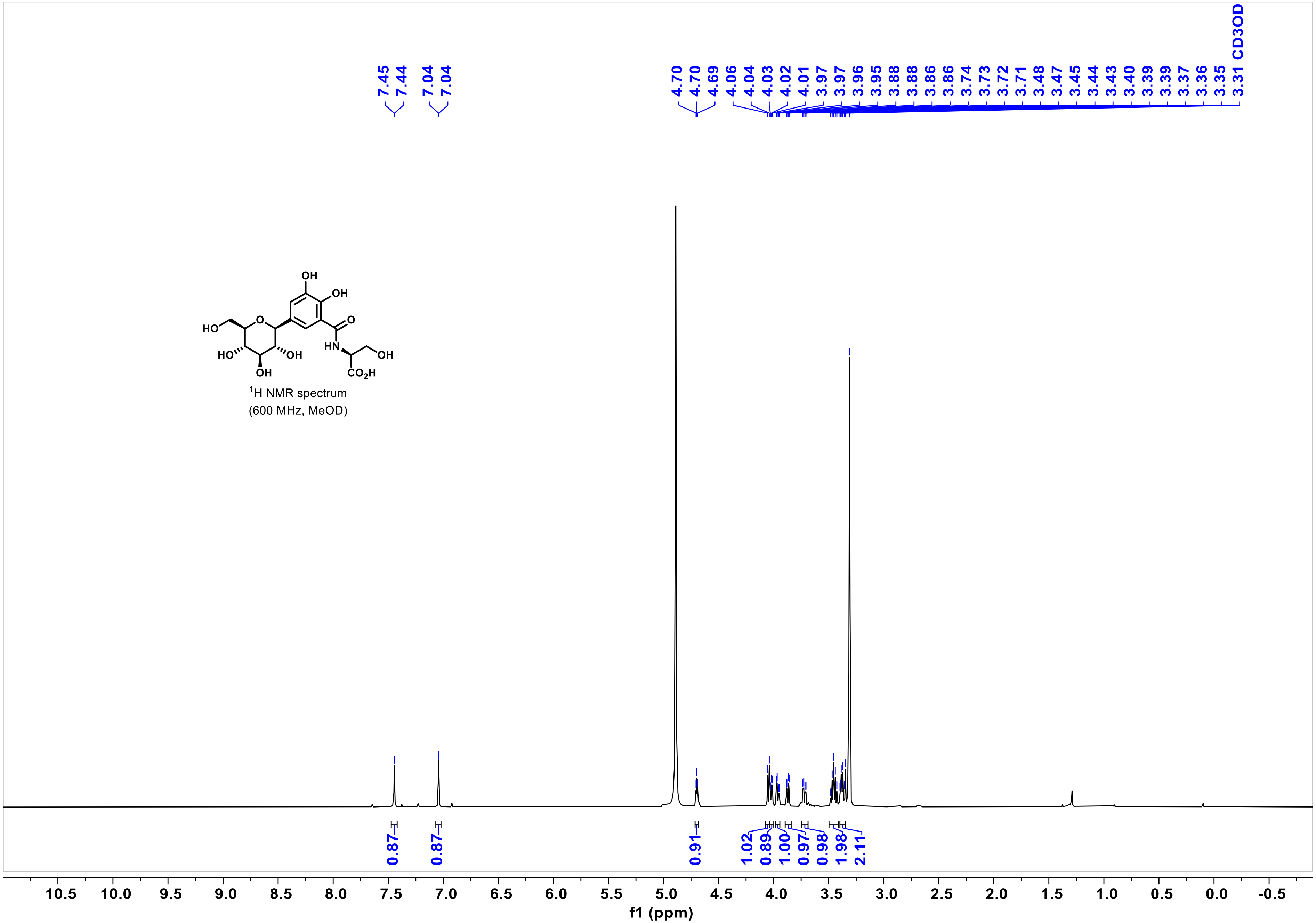

^{1}H NMR of compound **Salmochelin SX (72)**

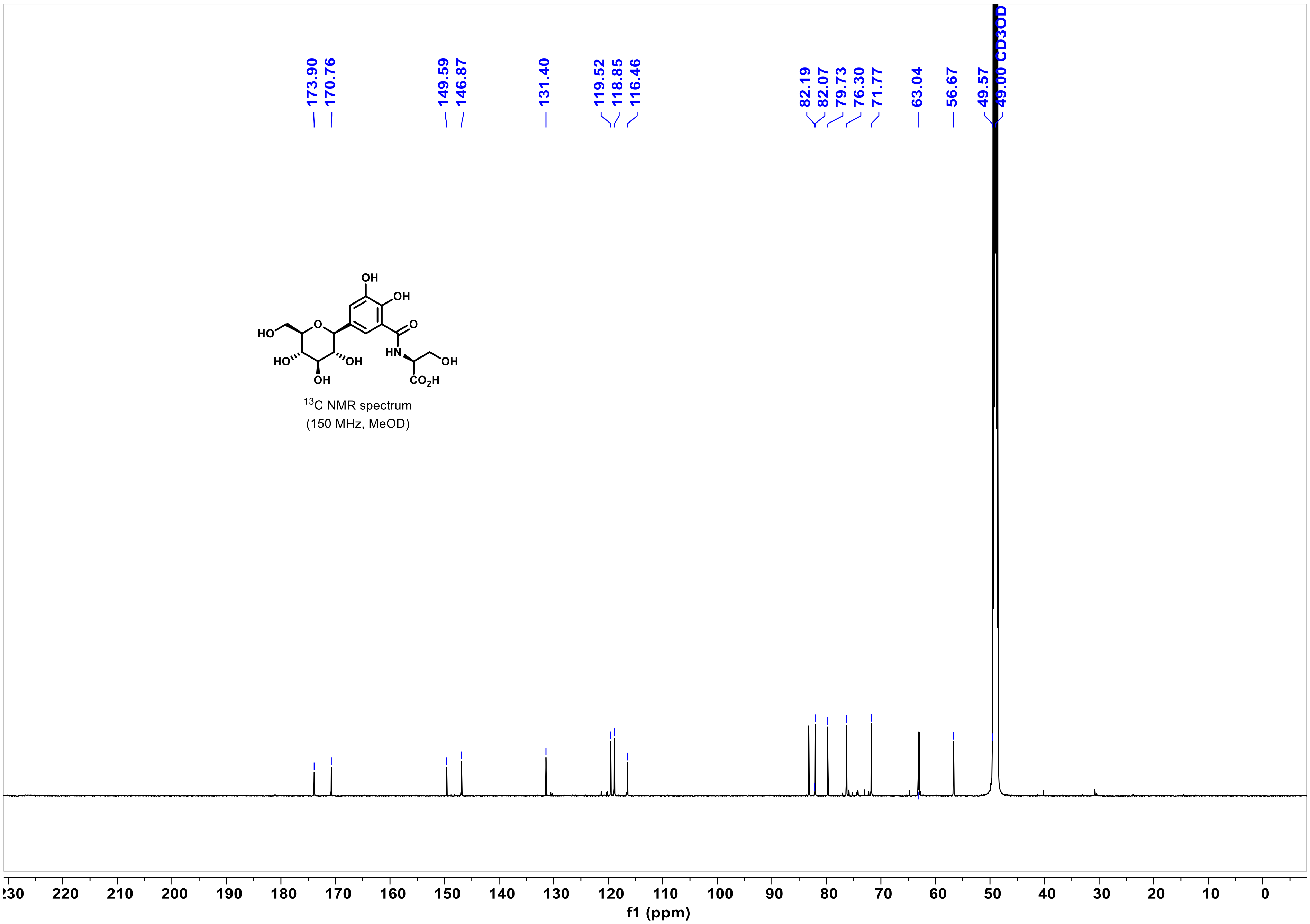

^{13}C NMR of compound **Salmochelin SX (72)**

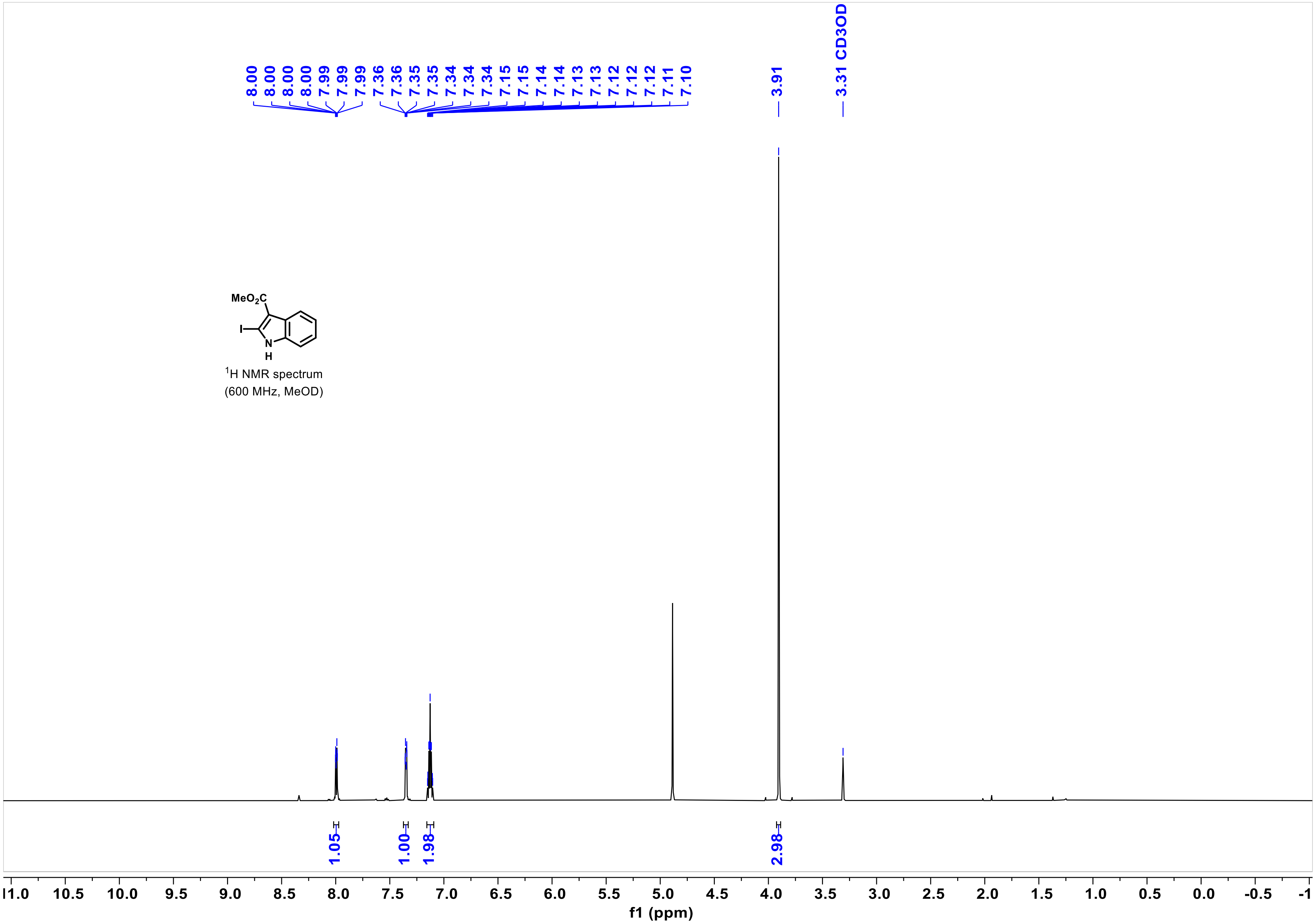

^{1}H NMR of compound **74**

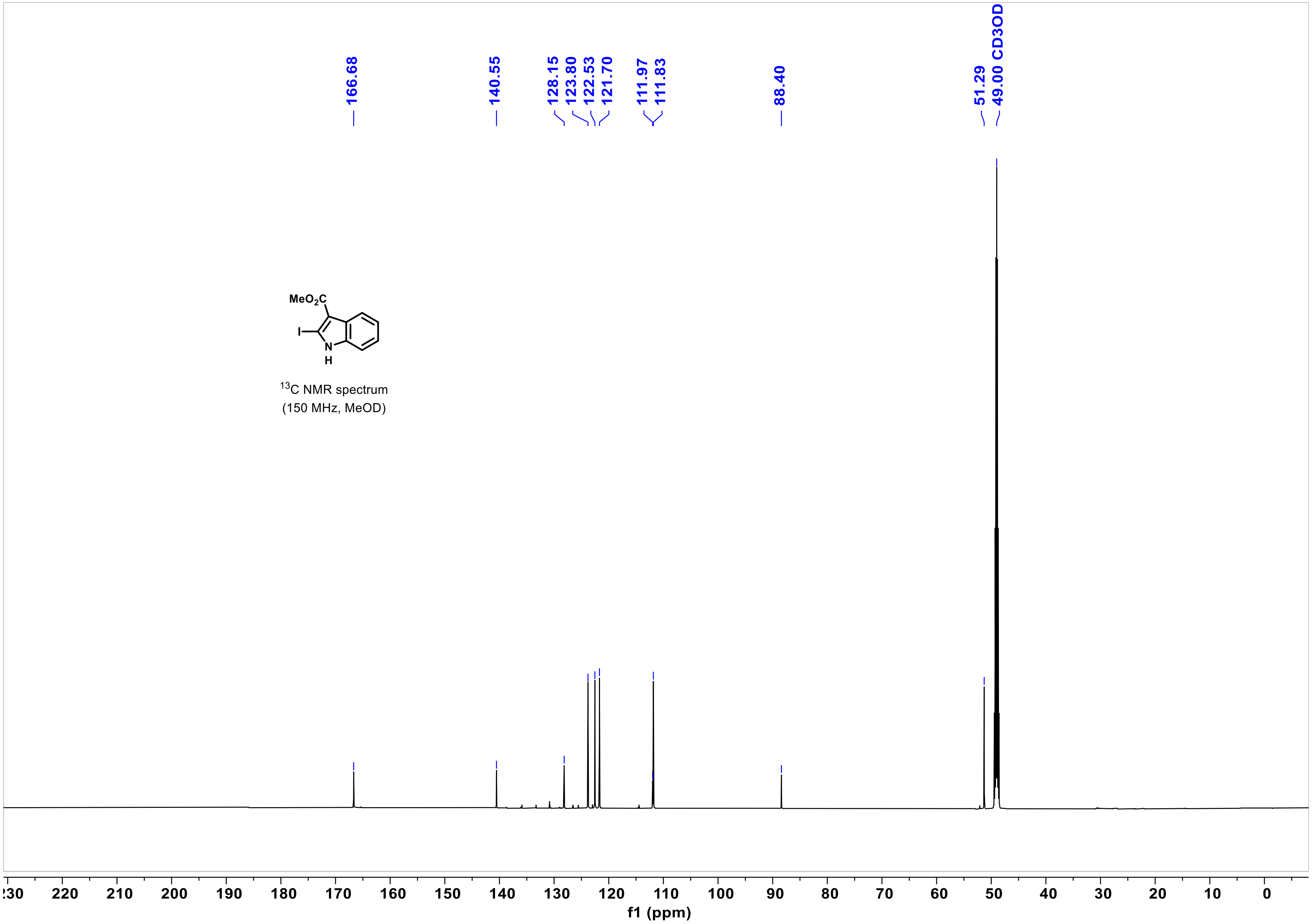

^{13}C NMR of compound **74**

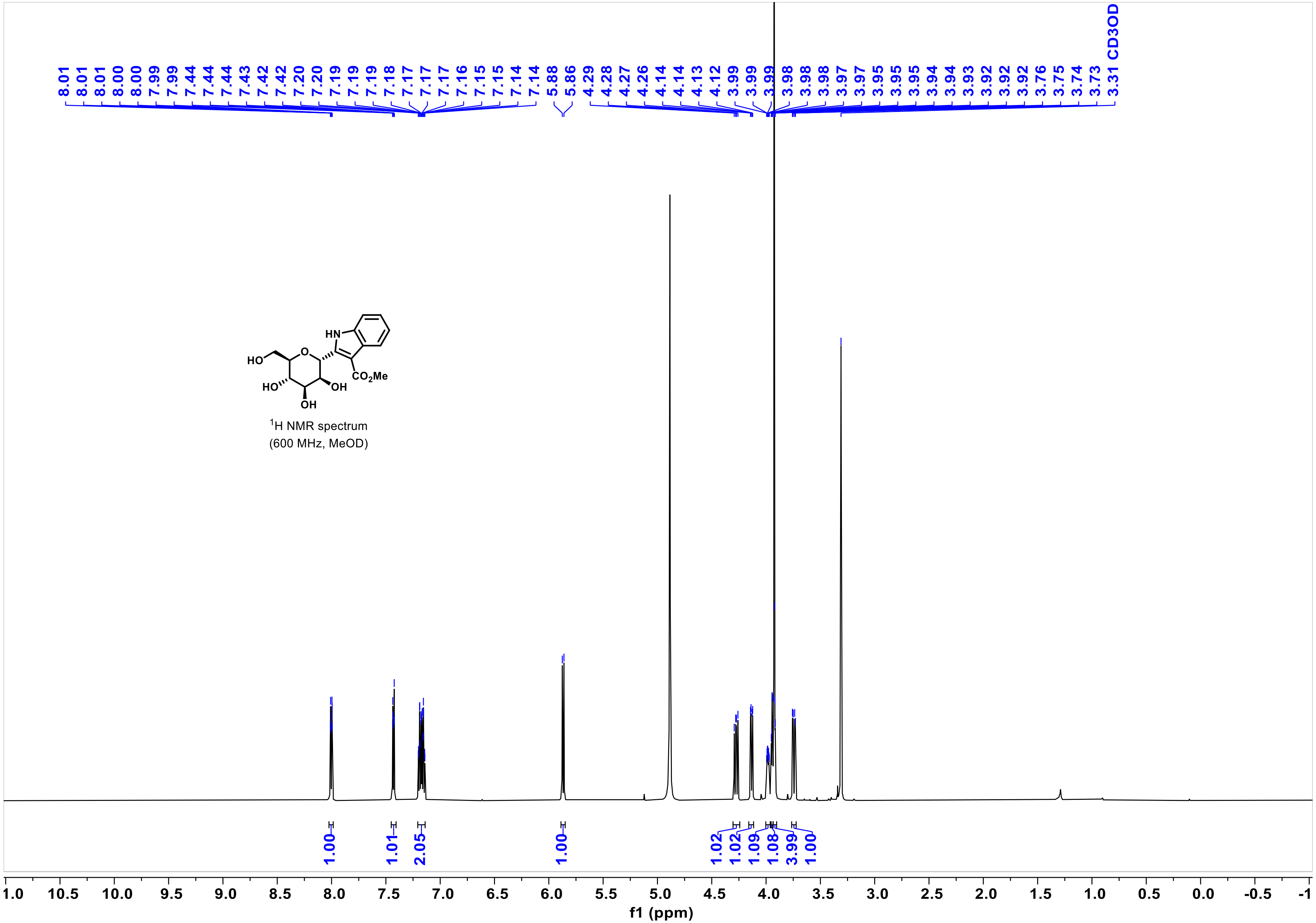

^{1}H NMR of compound **Neopetrosin C (75)**

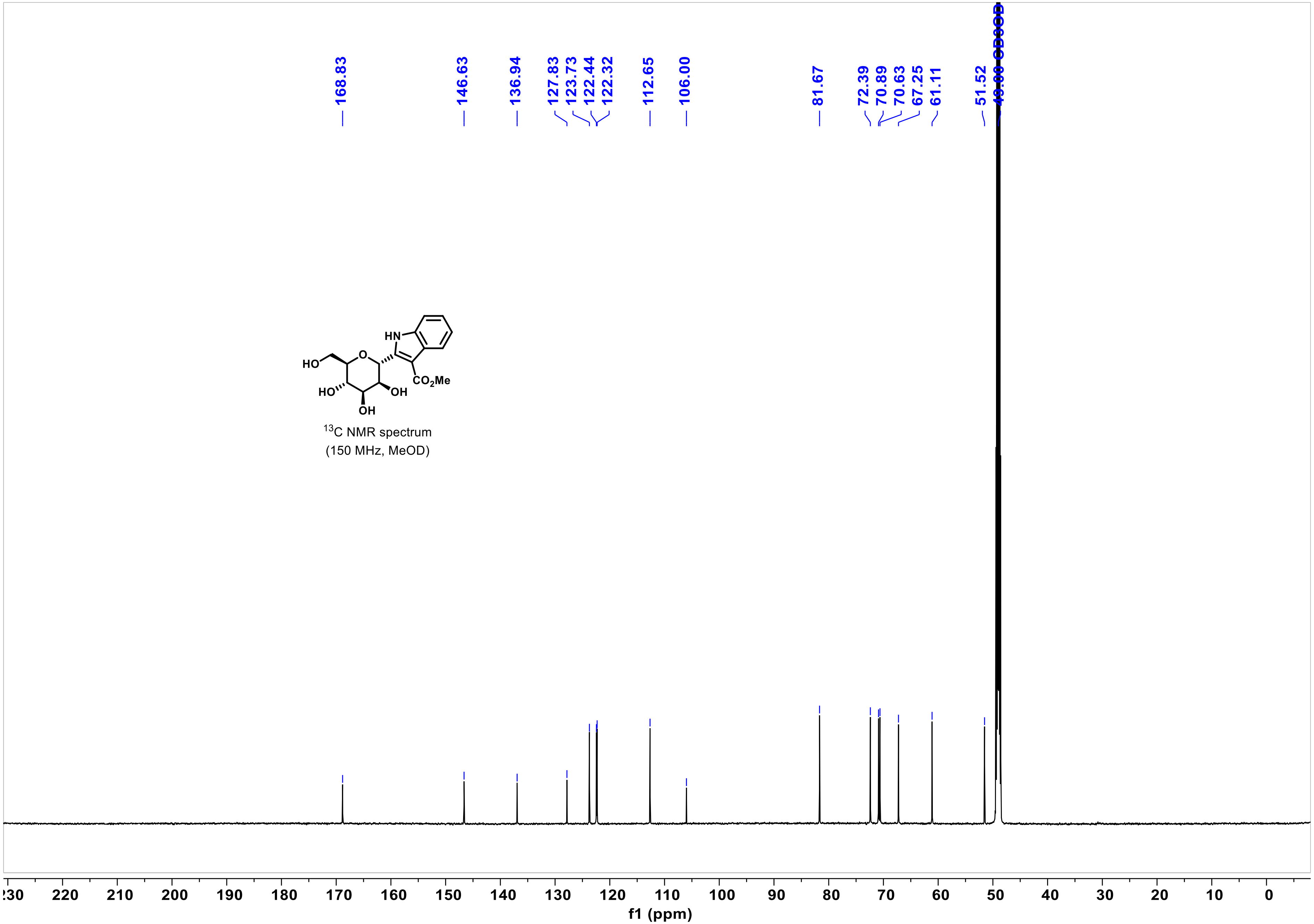

^{13}C NMR of compound **Neopetrosin C (75)**

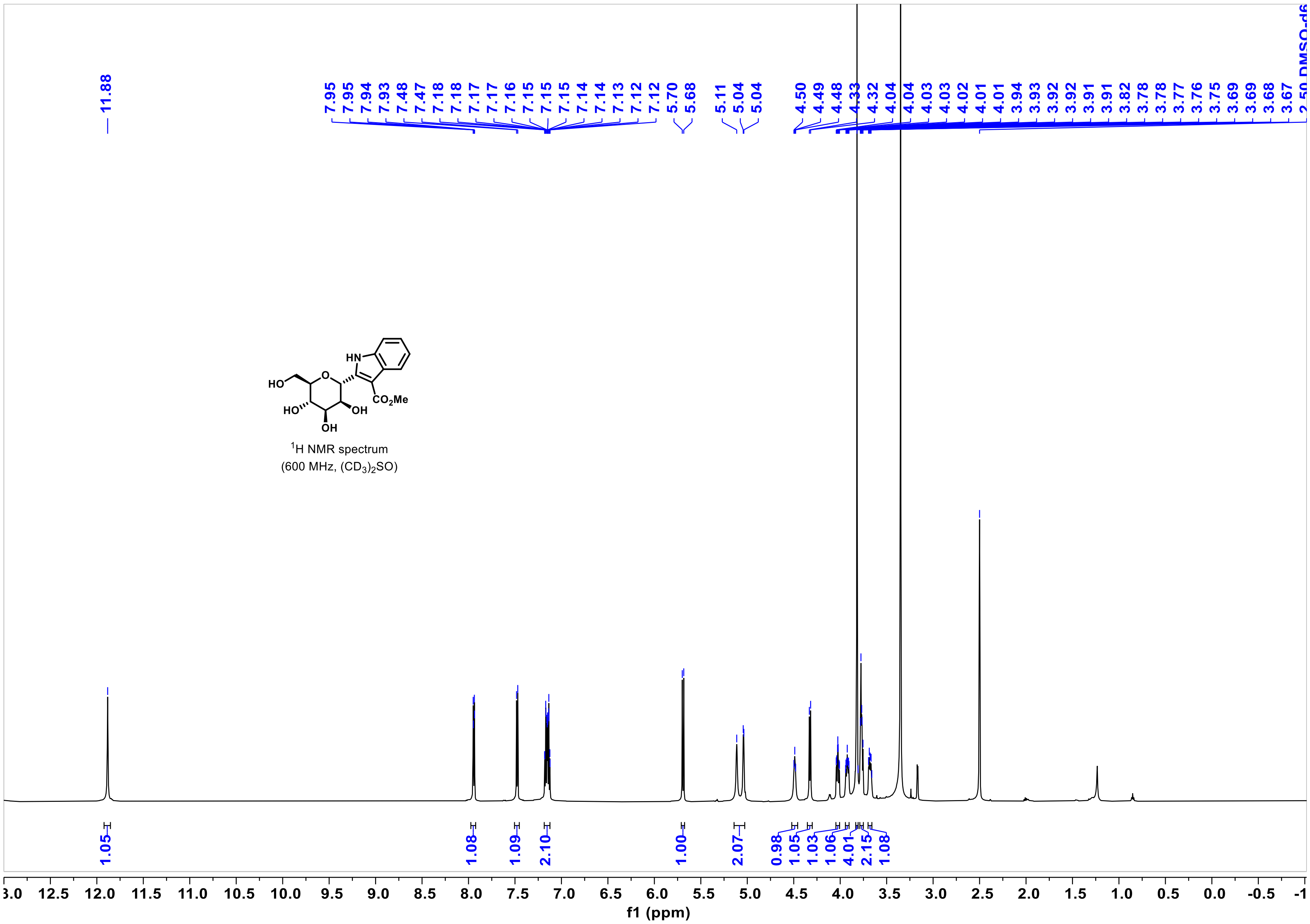

^{1}H NMR of compound **Neopetrosin C (75)**

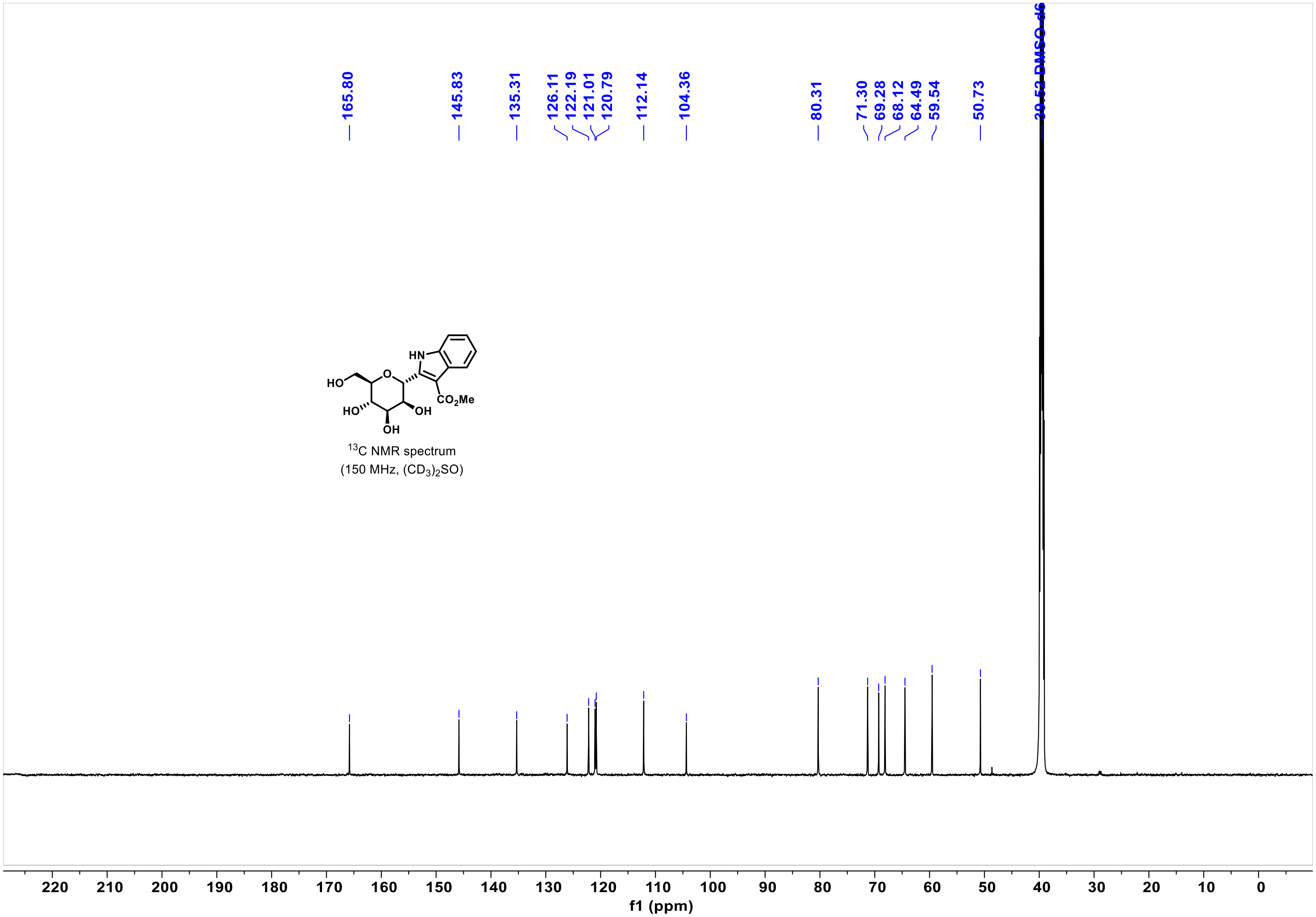

^{13}C NMR of compound **Neopetrosin C (75)**

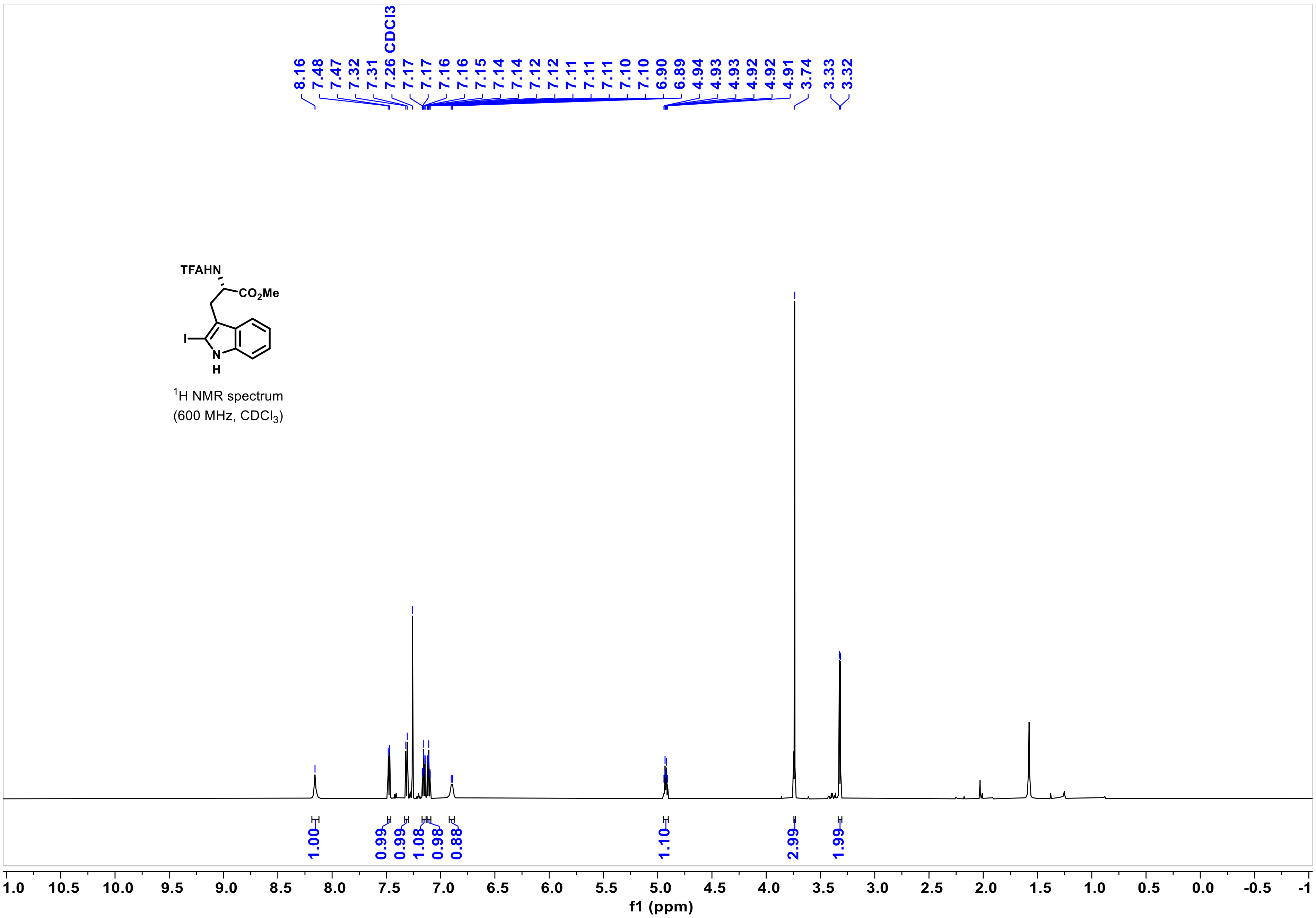

^{1}H NMR of compound **76**

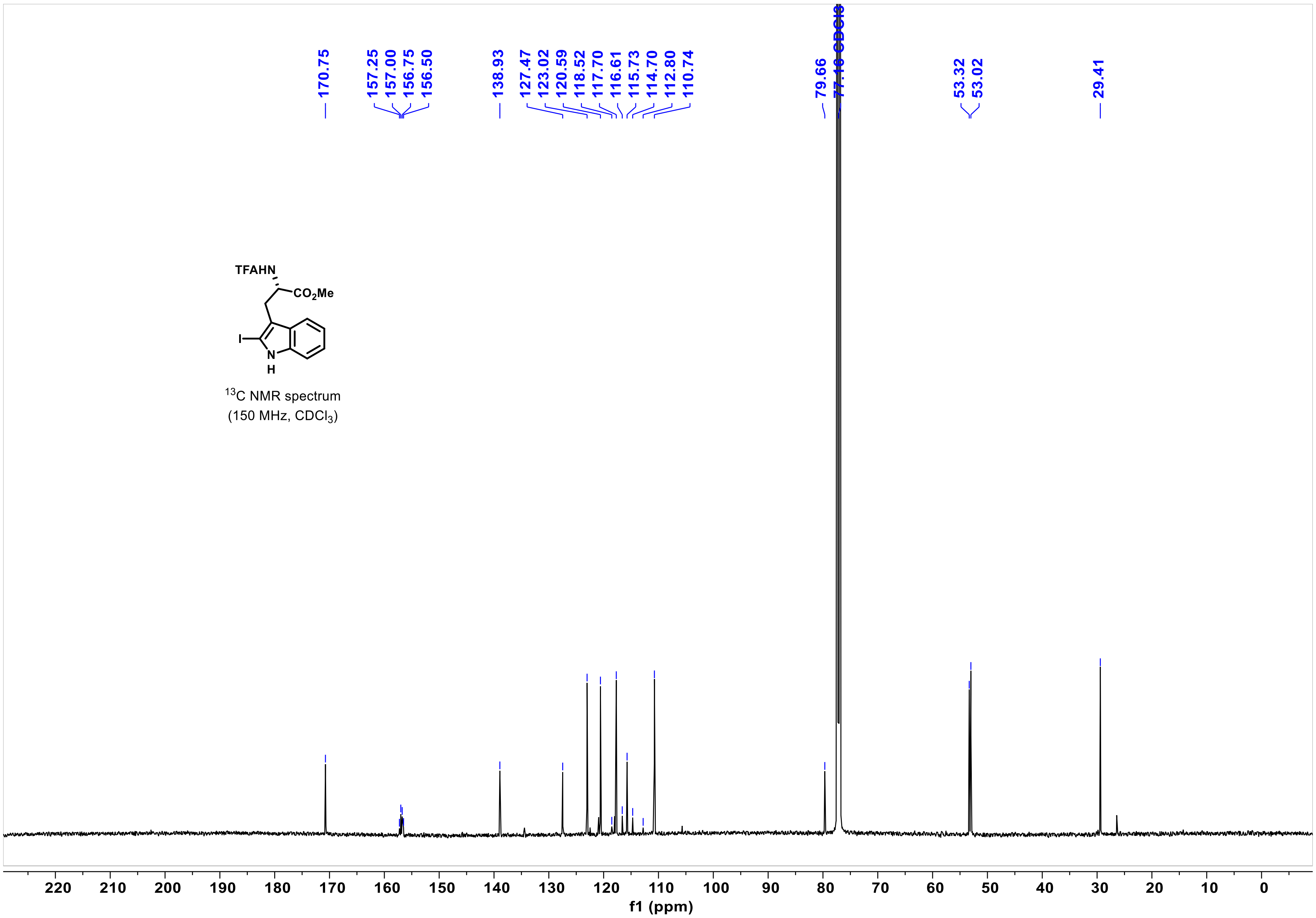

^{13}C NMR of compound **76**

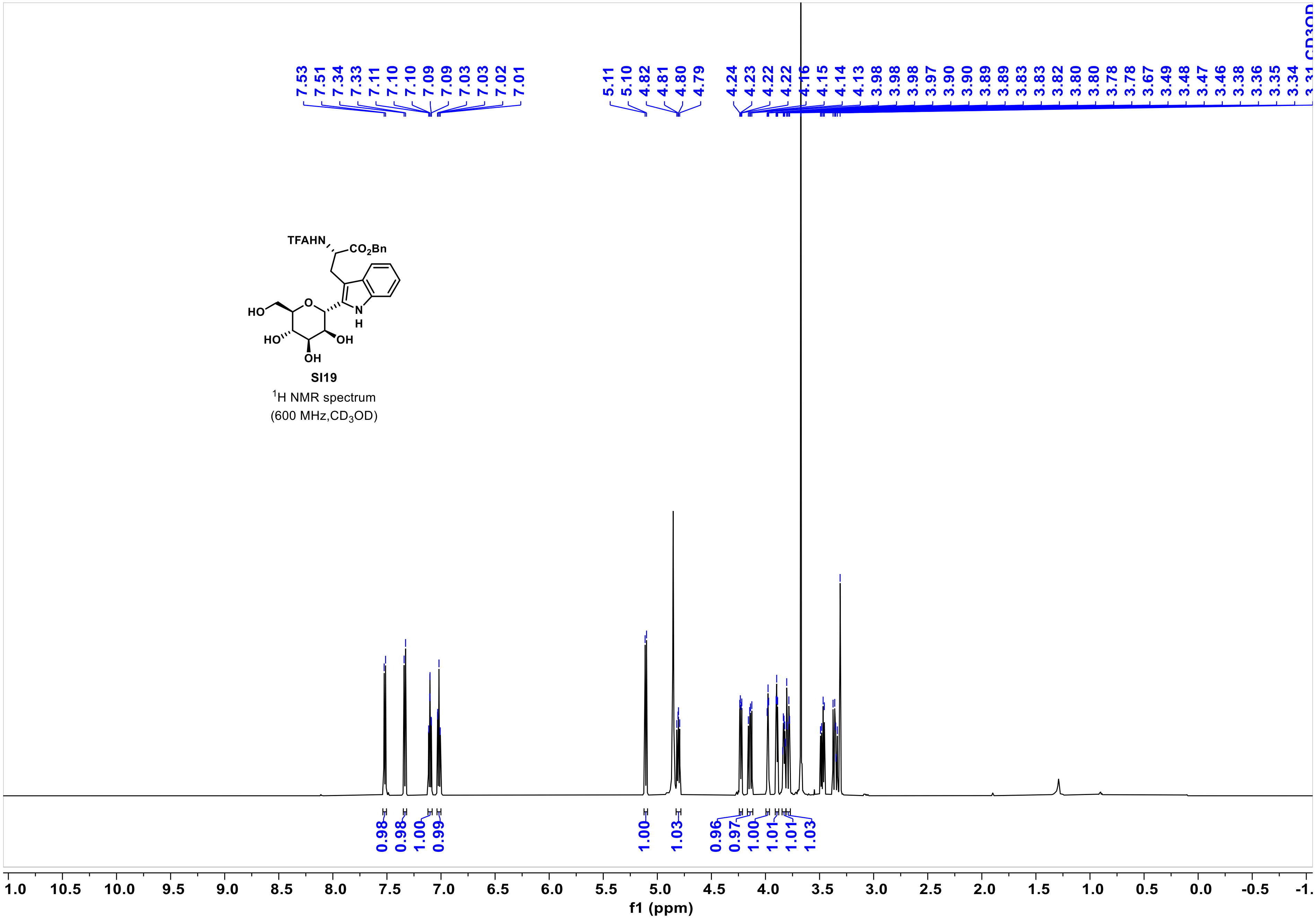

^{1}H NMR of compound **SI9**

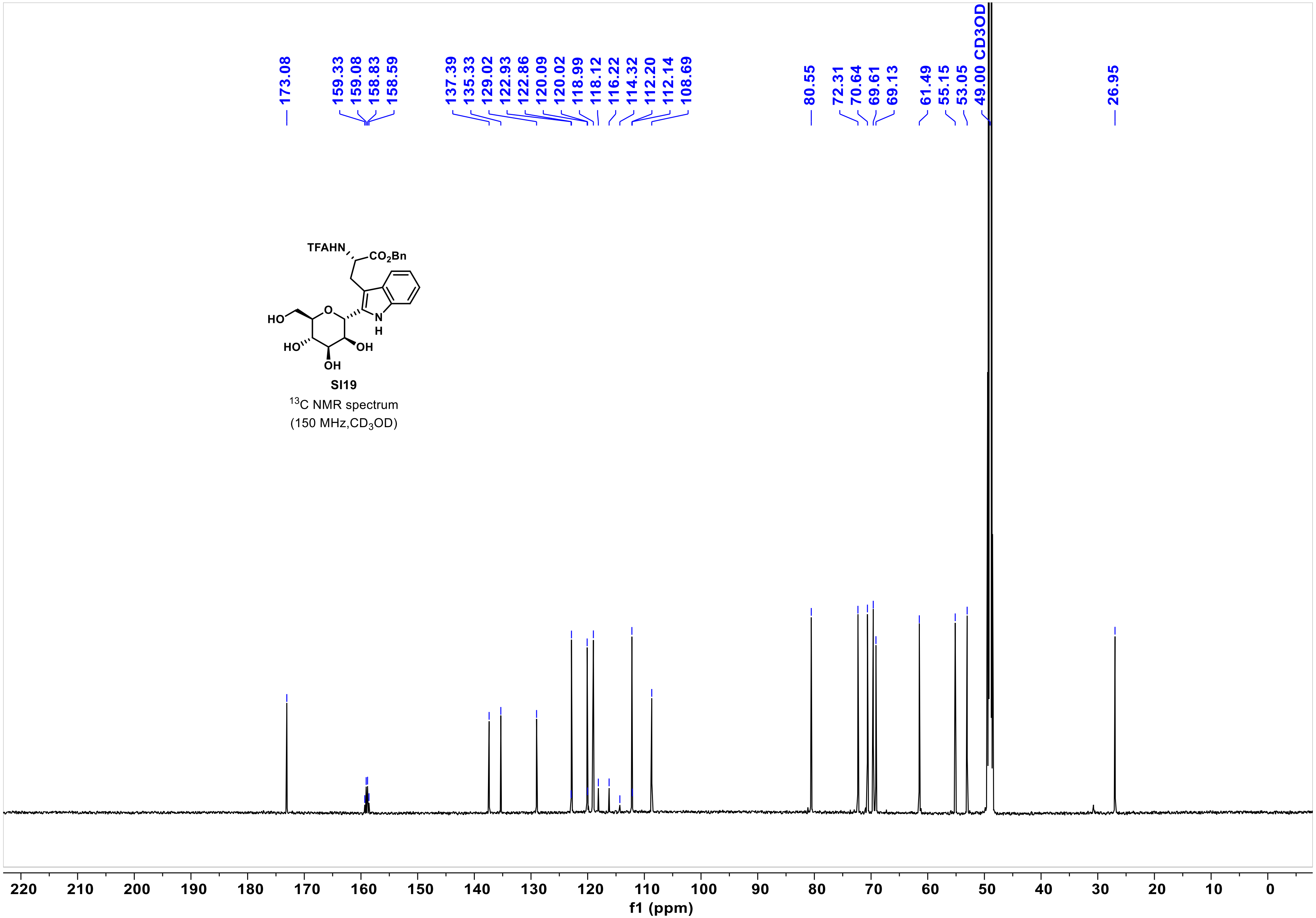

^{13}C NMR of compound **SI9**

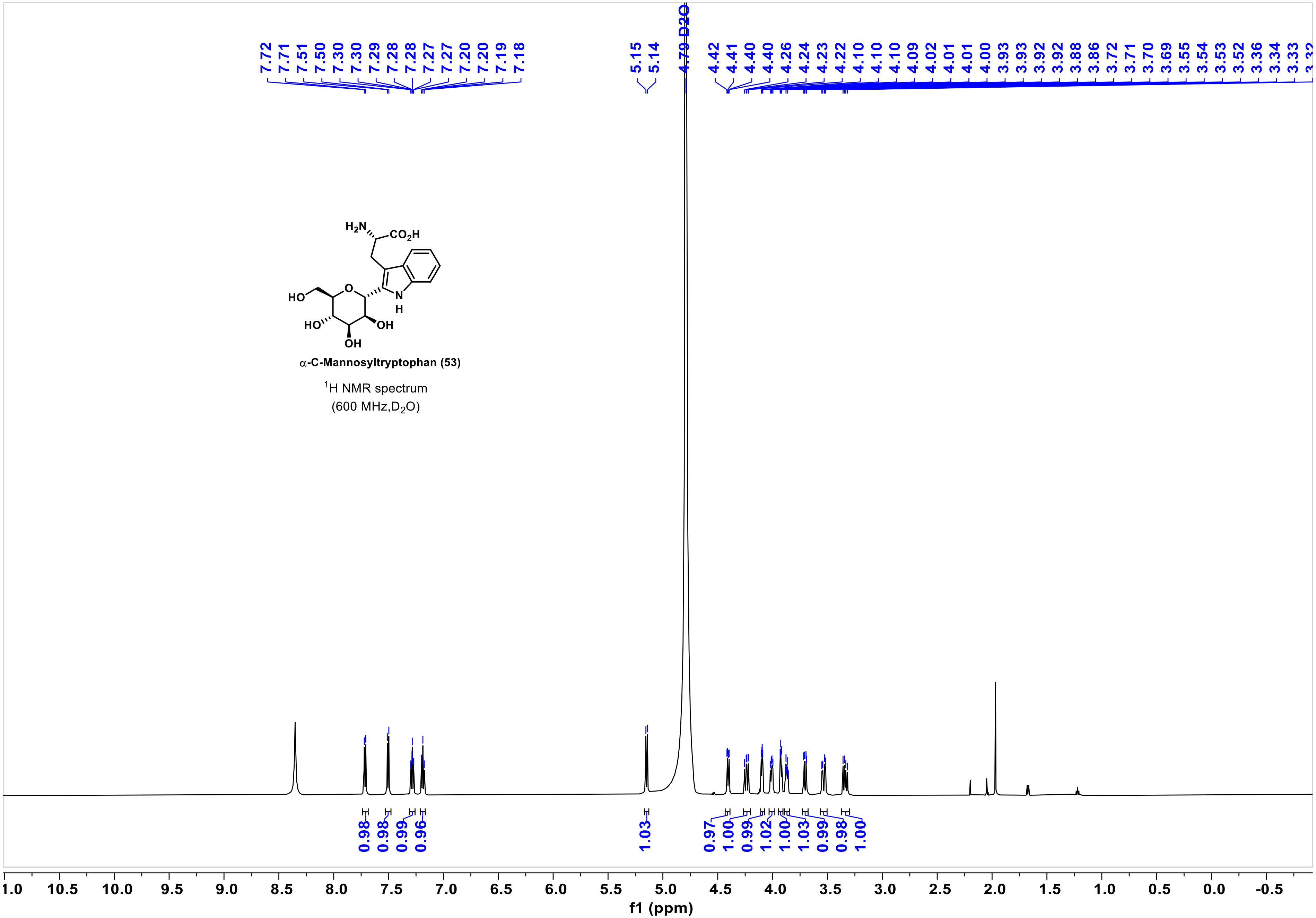

^{1}H NMR of **α-C-Mannosyltryptophan (77)**

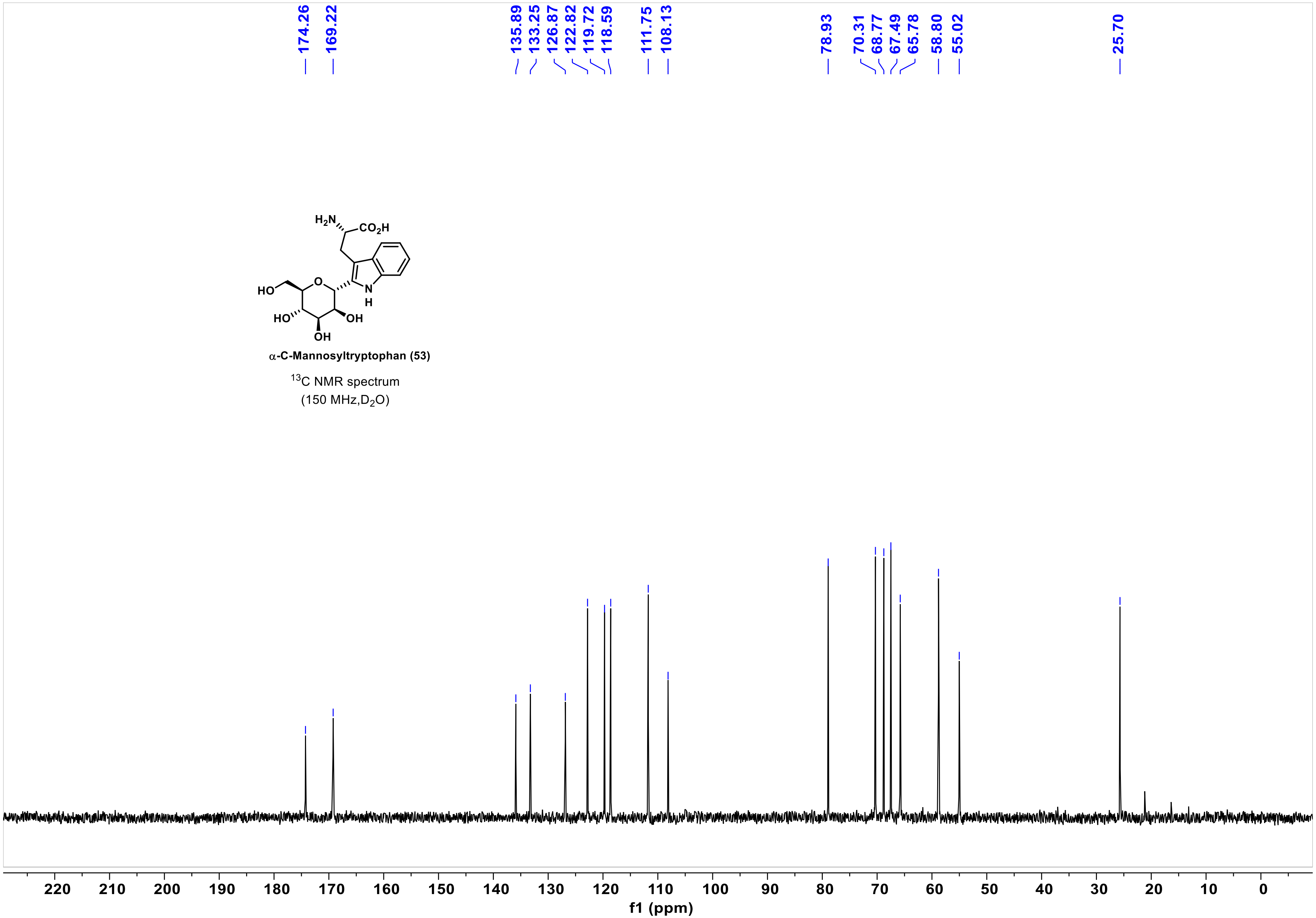

^{13}C NMR of **α-C-Mannosyltryptophan (77)**

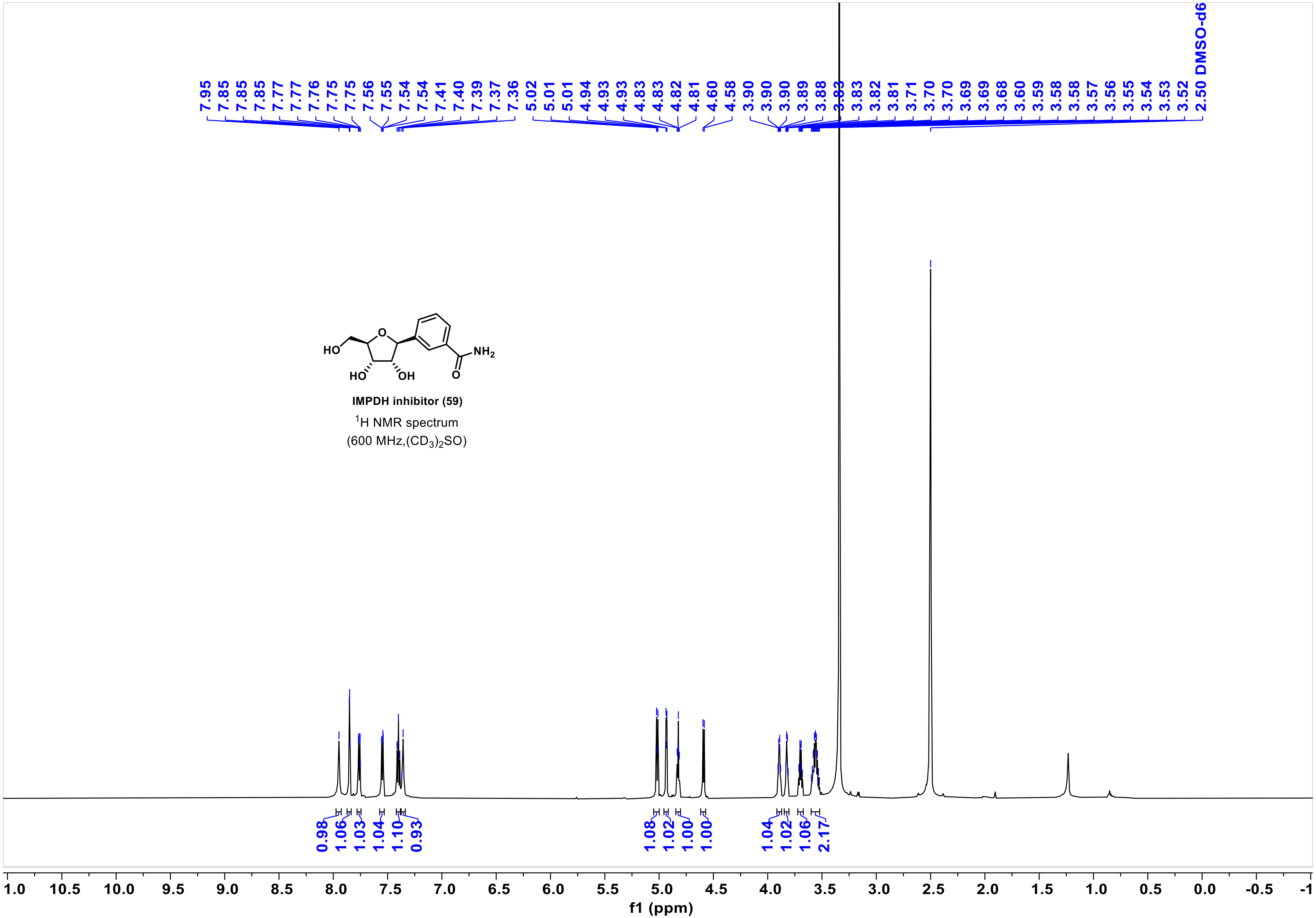

^{1}H NMR of **IMPDH inhibitor (80)**

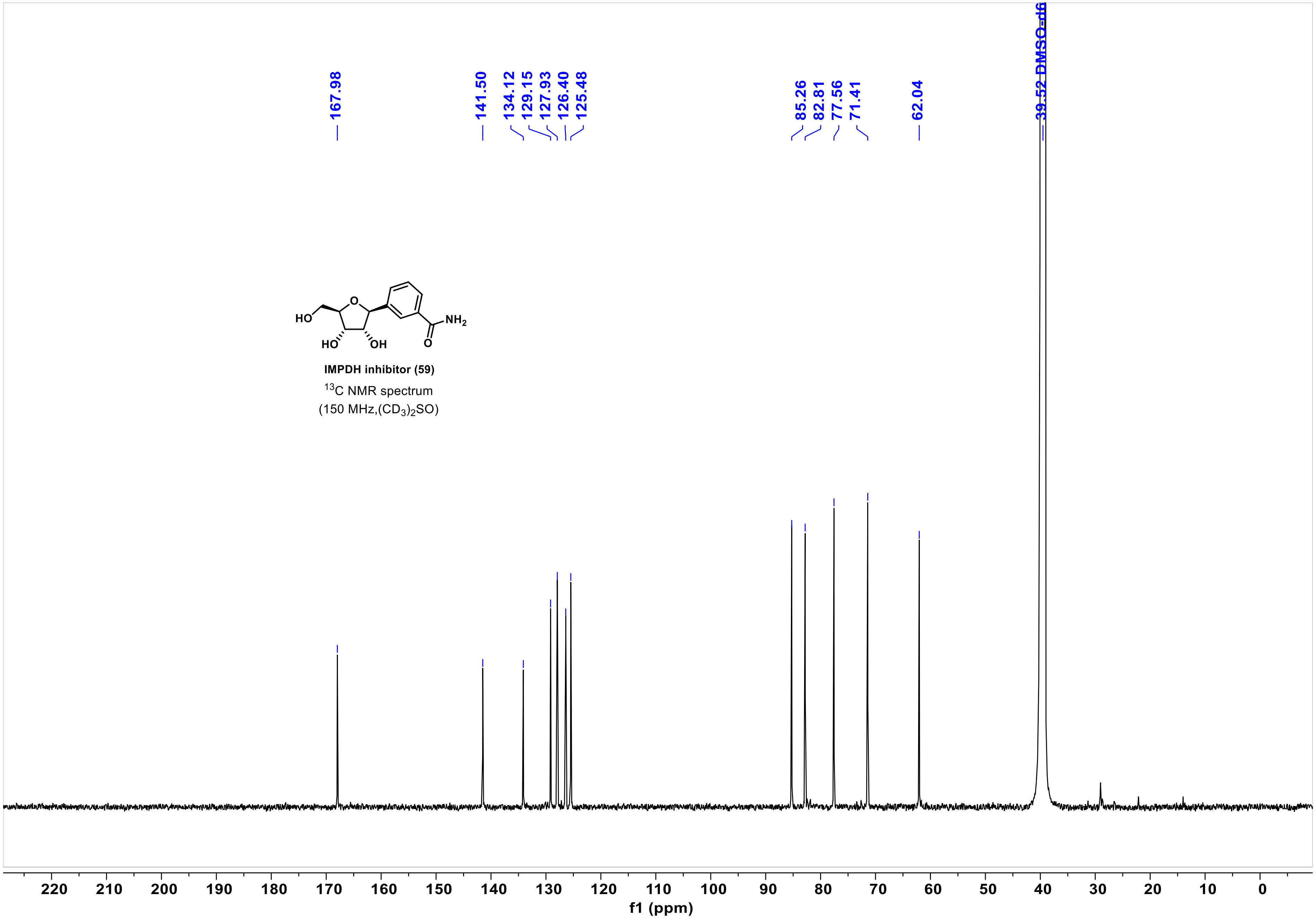

^{13}C NMR of **IMPDH inhibitor (80)**

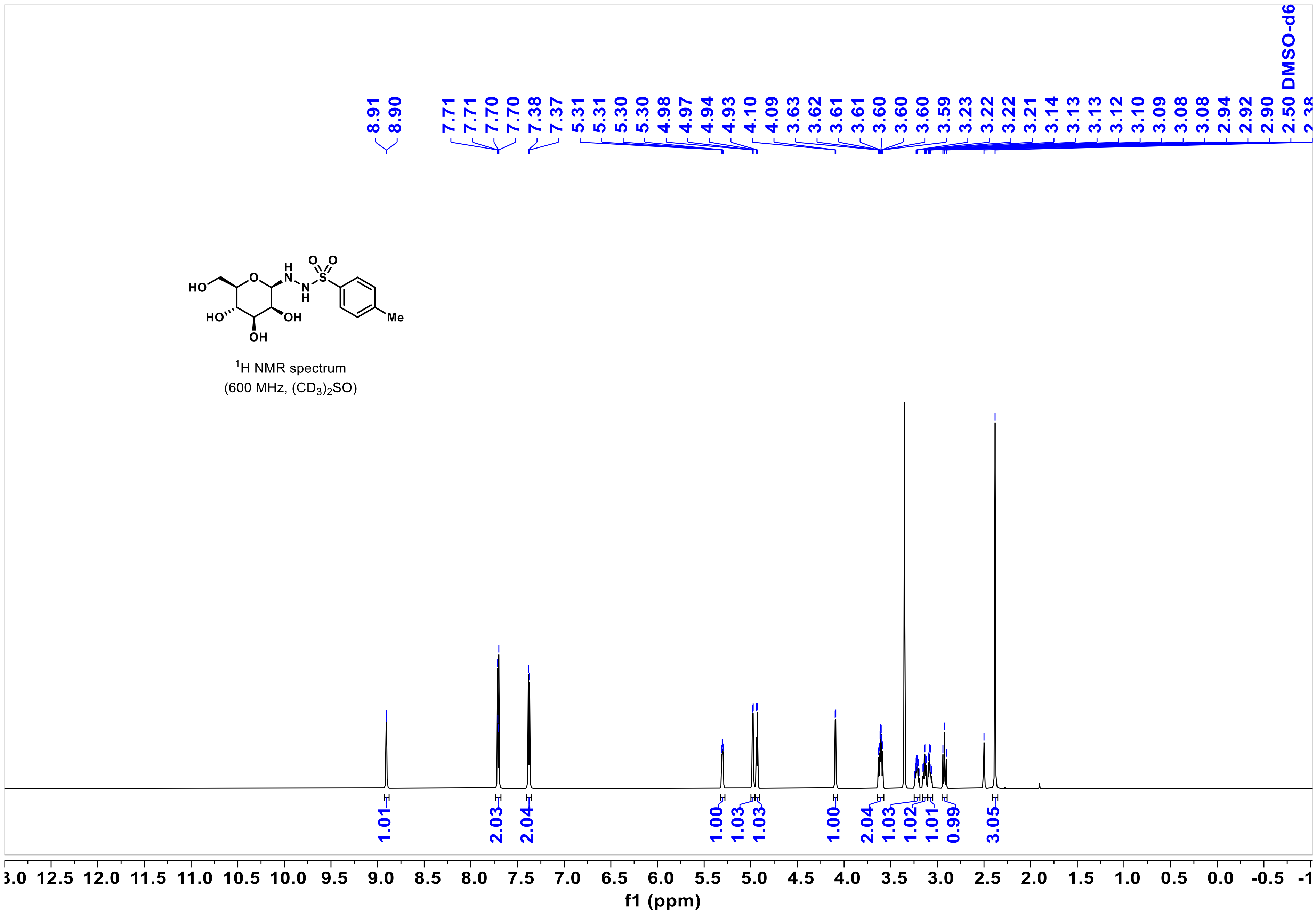

^{1}H NMR of compound **β-SH5**

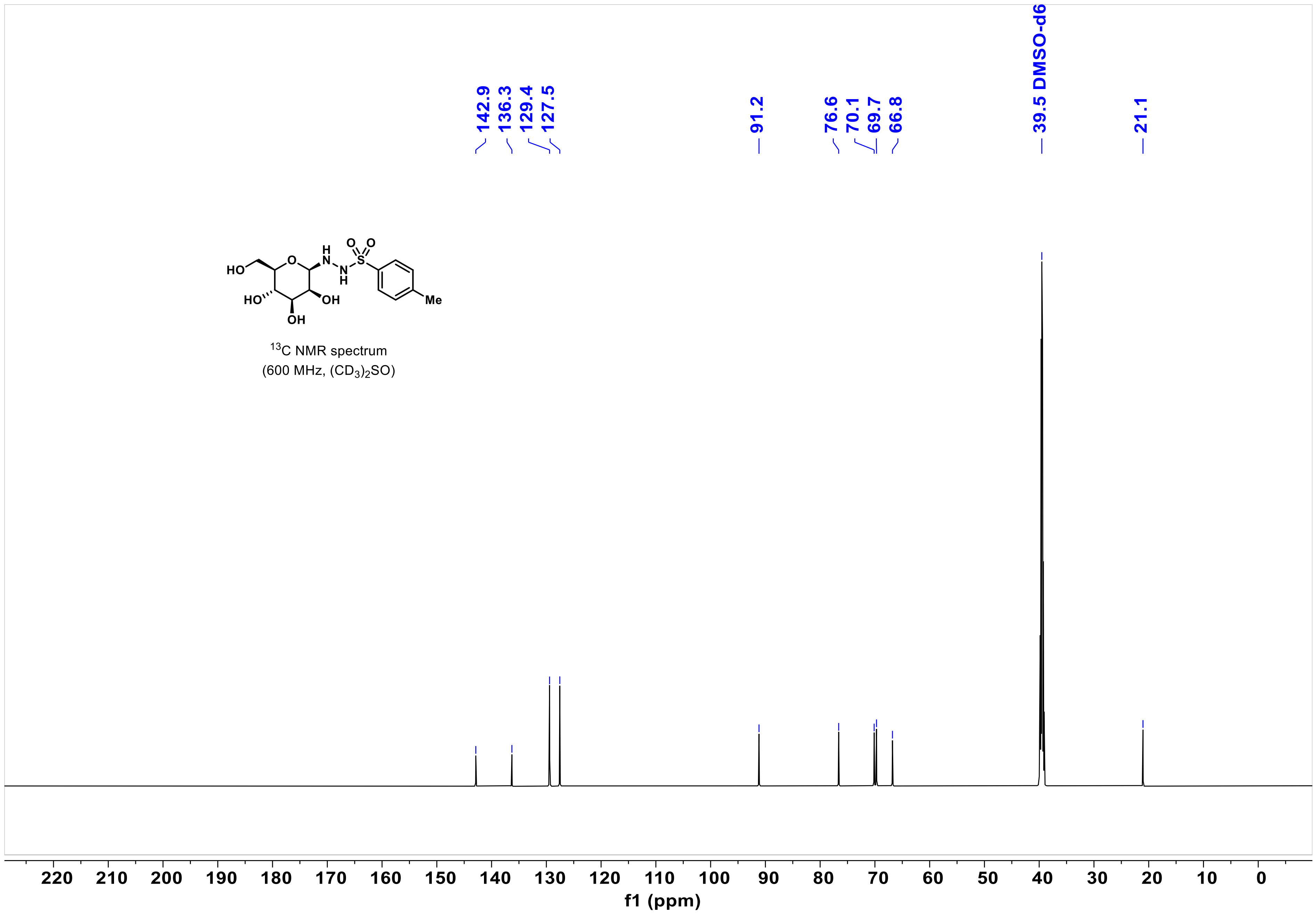

^{13}C NMR of compound **β-SH5**

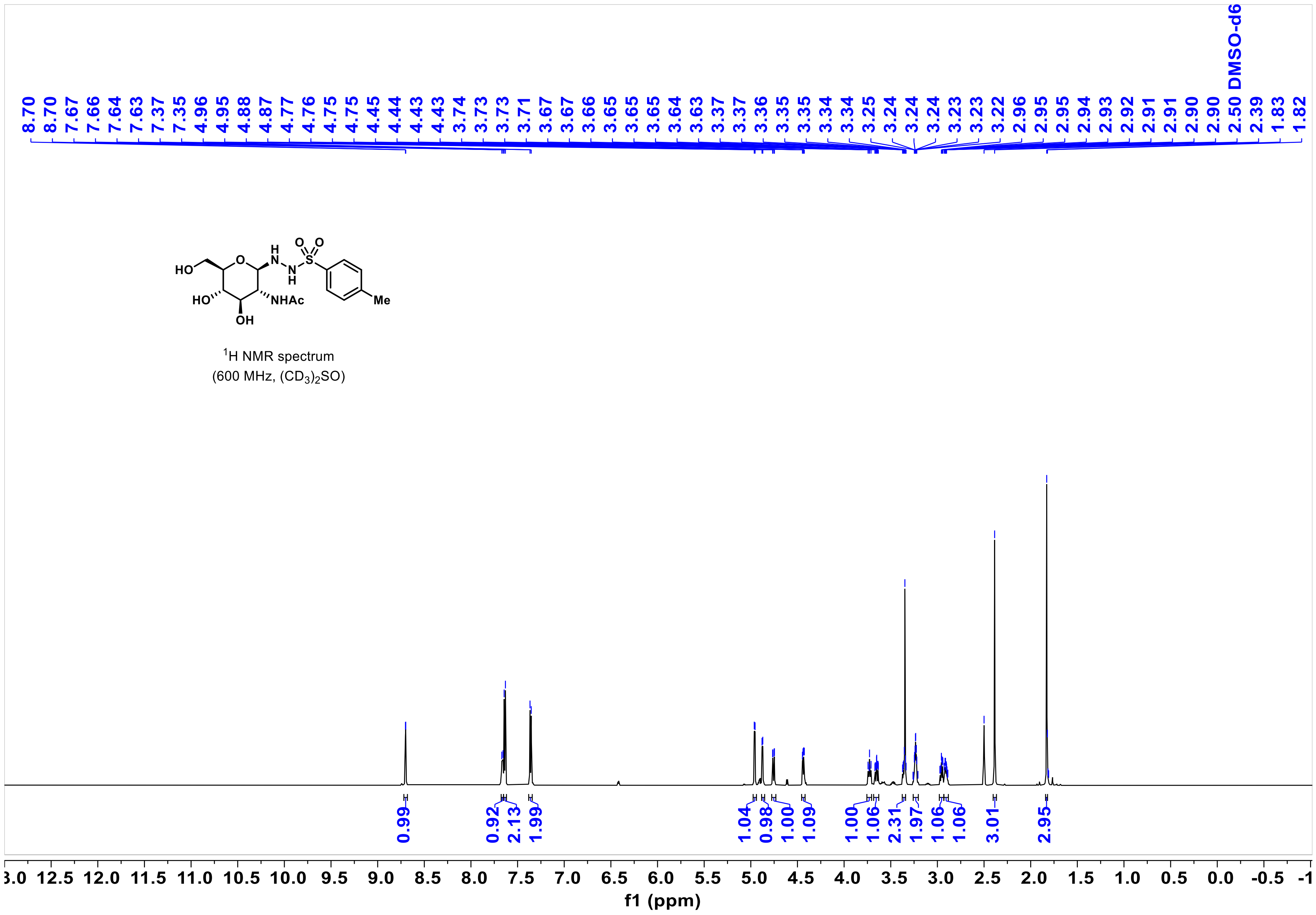

^{1}H NMR of compound **β-SH6**

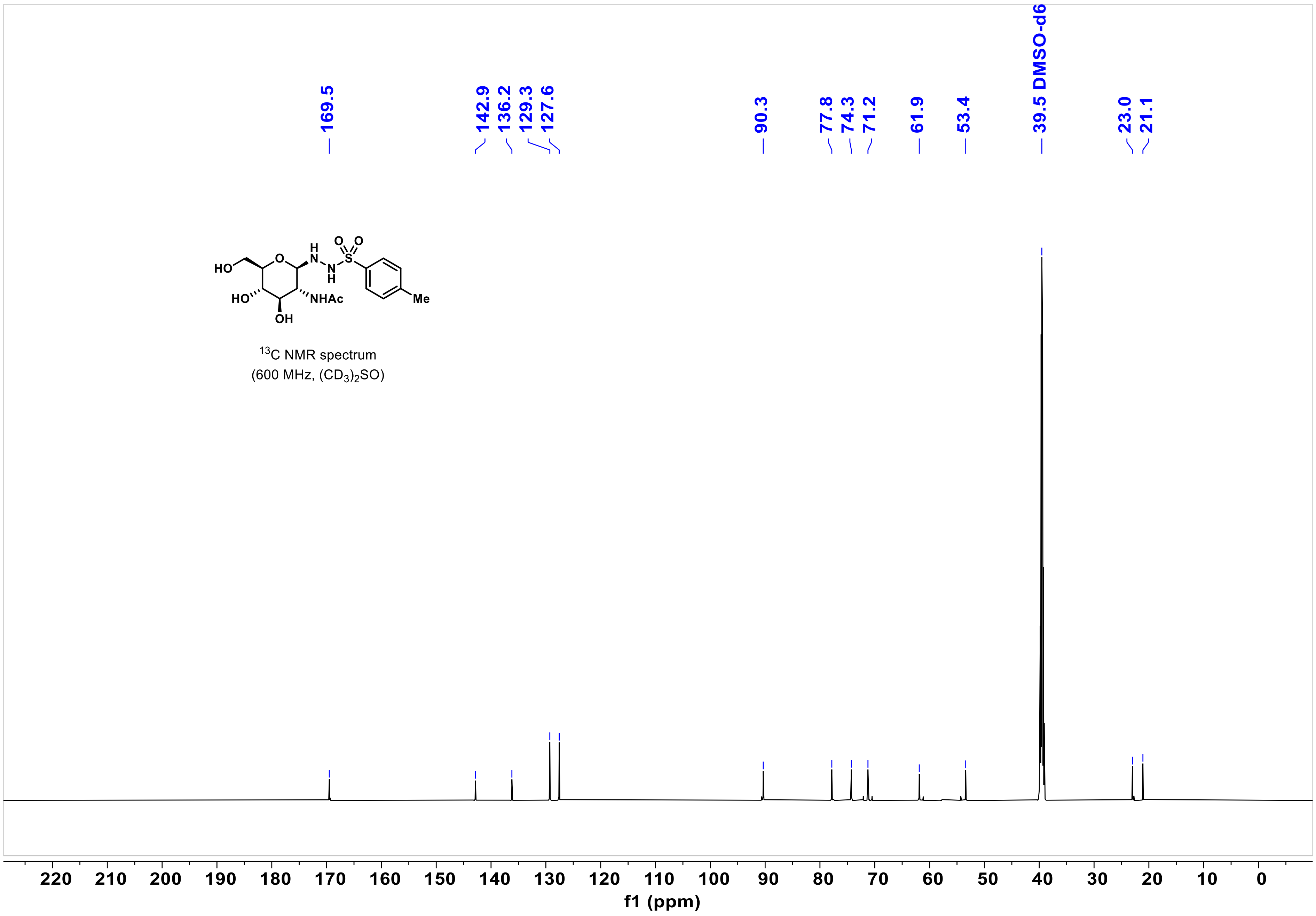

^{13}C NMR of compound **β-SH6**

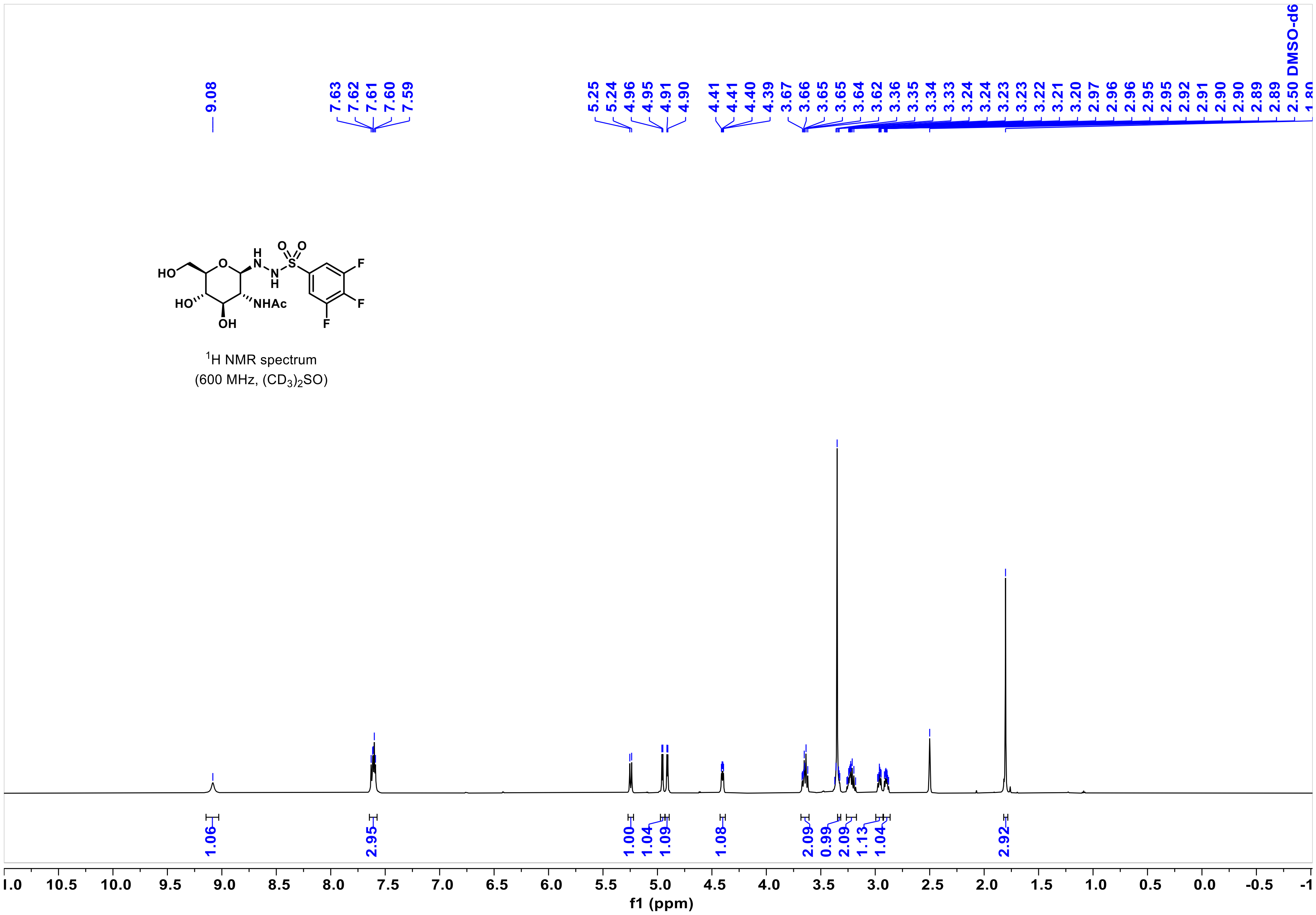

^{1}H NMR of compound **β-SH7**

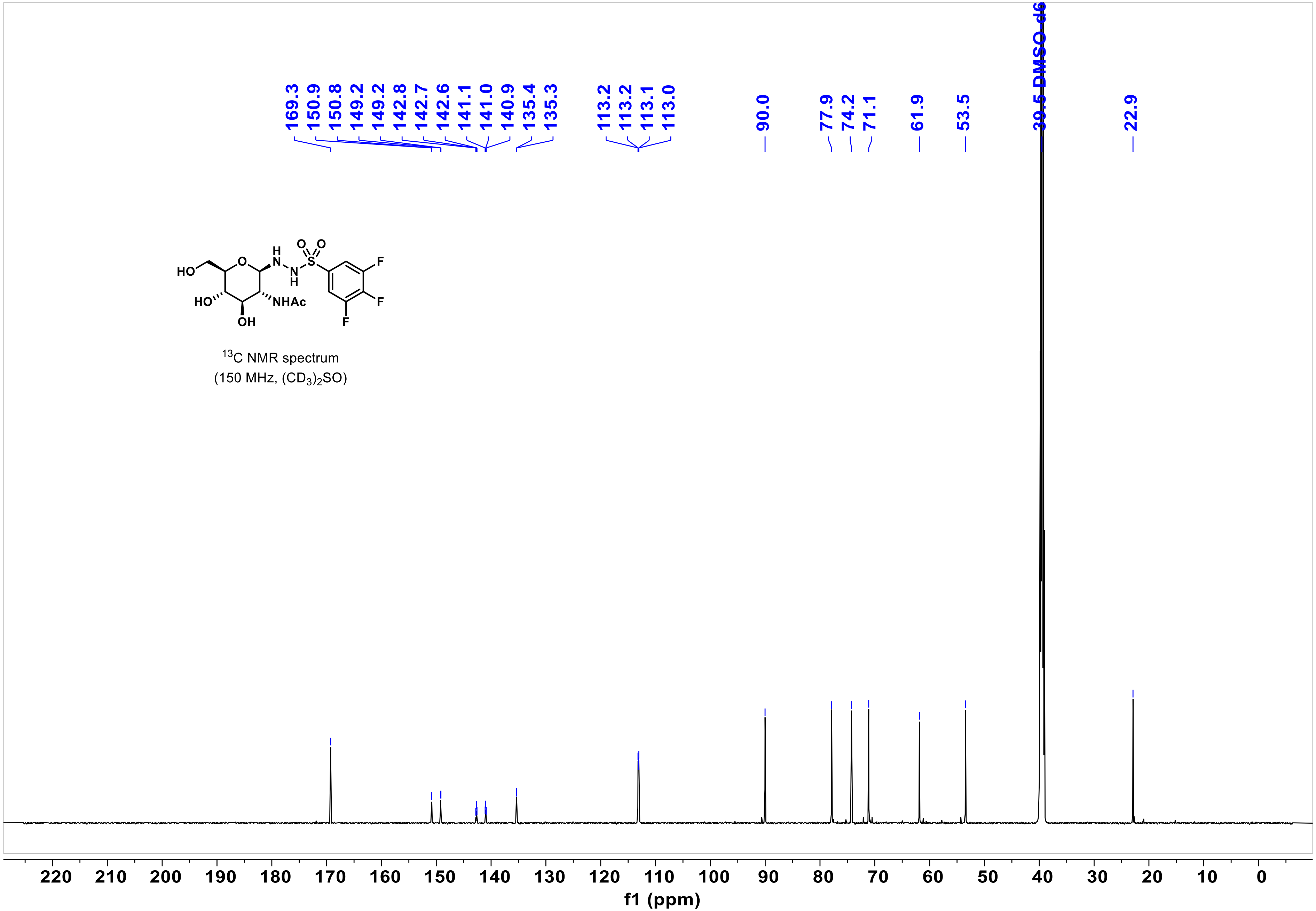

^{13}C NMR of compound **β-SH7**

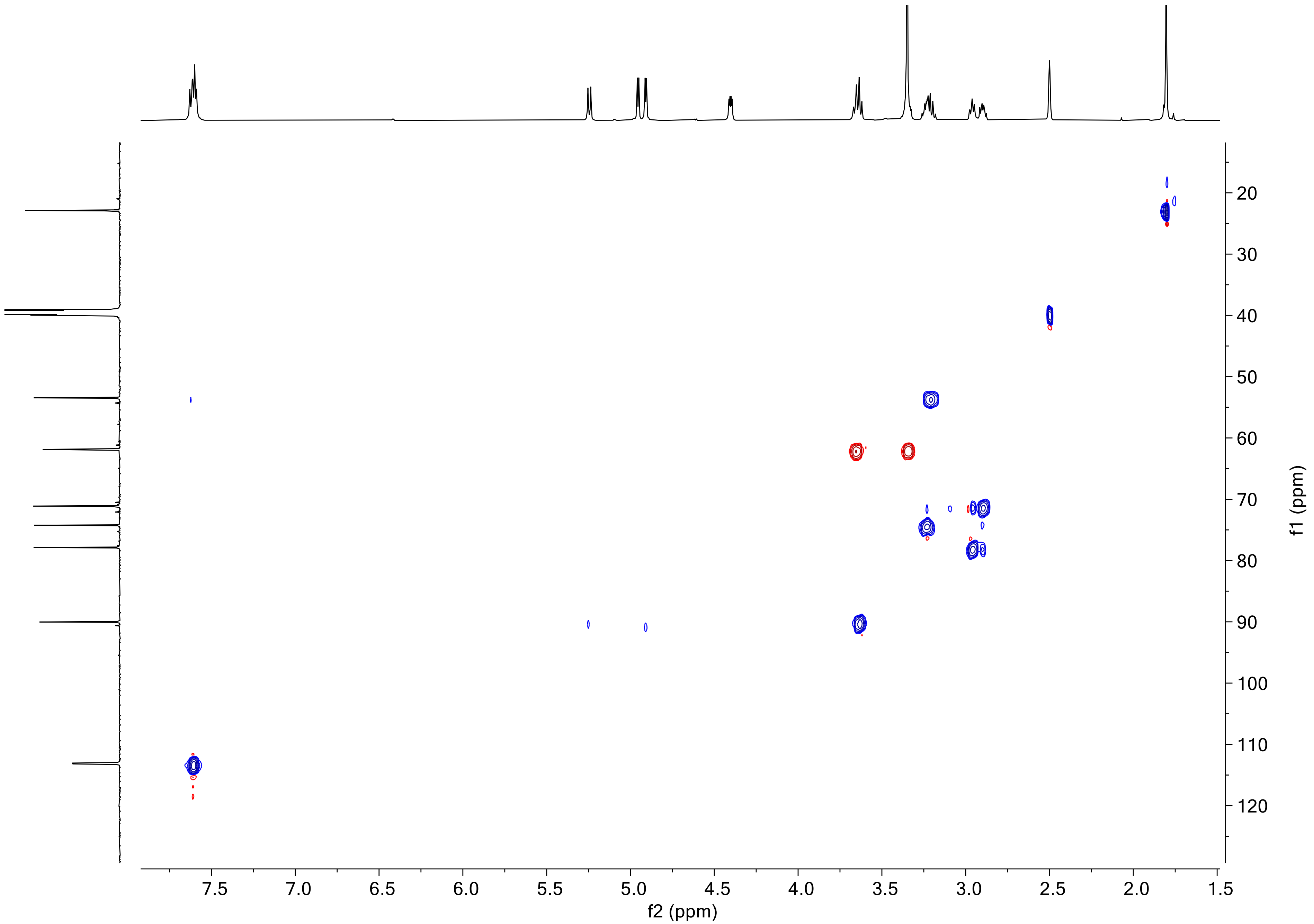

H-C HSQC NMR Spectrum of compound **β-SH7**

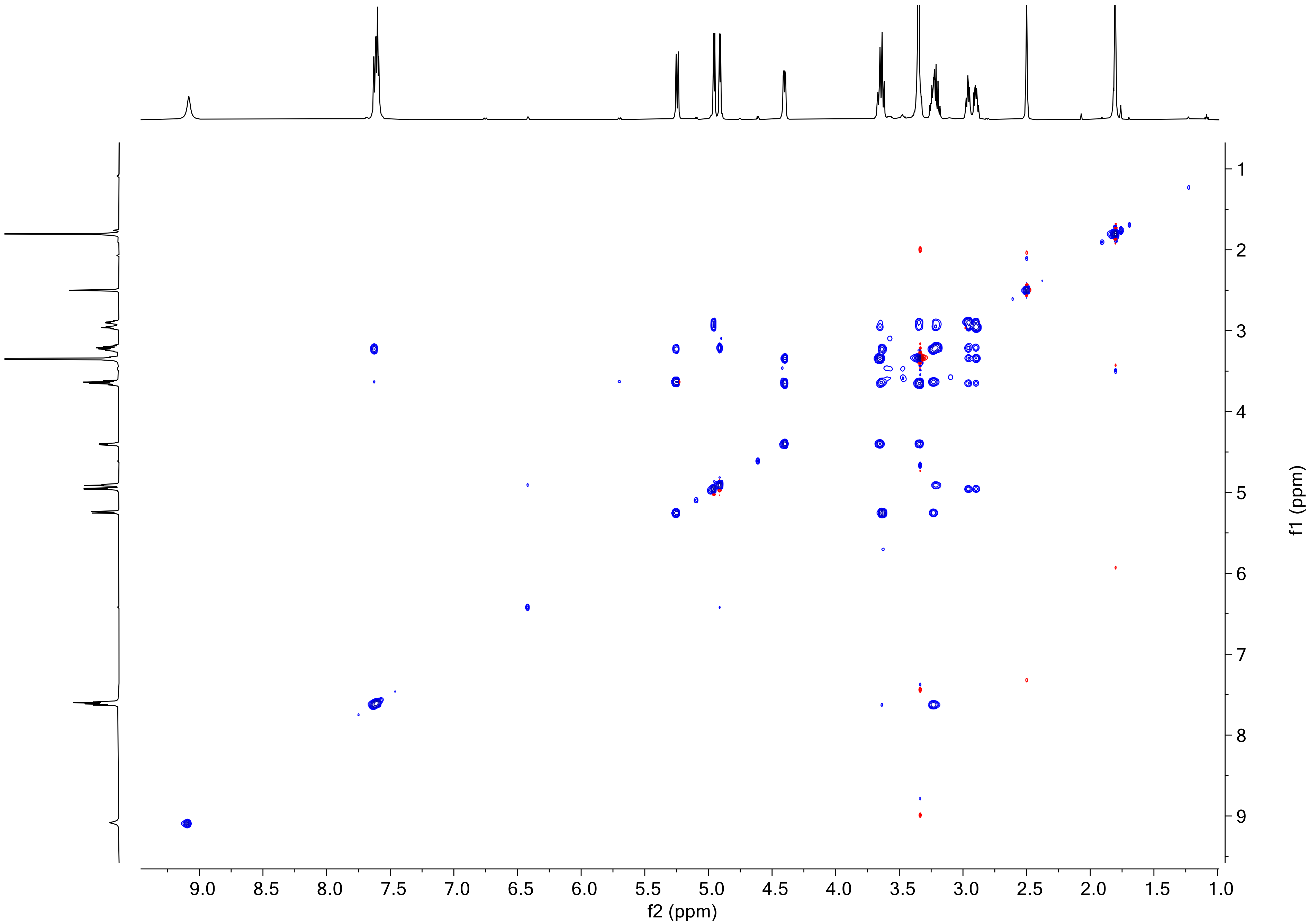

H-H COSY NMR Spectrum of compound **β-SH7**

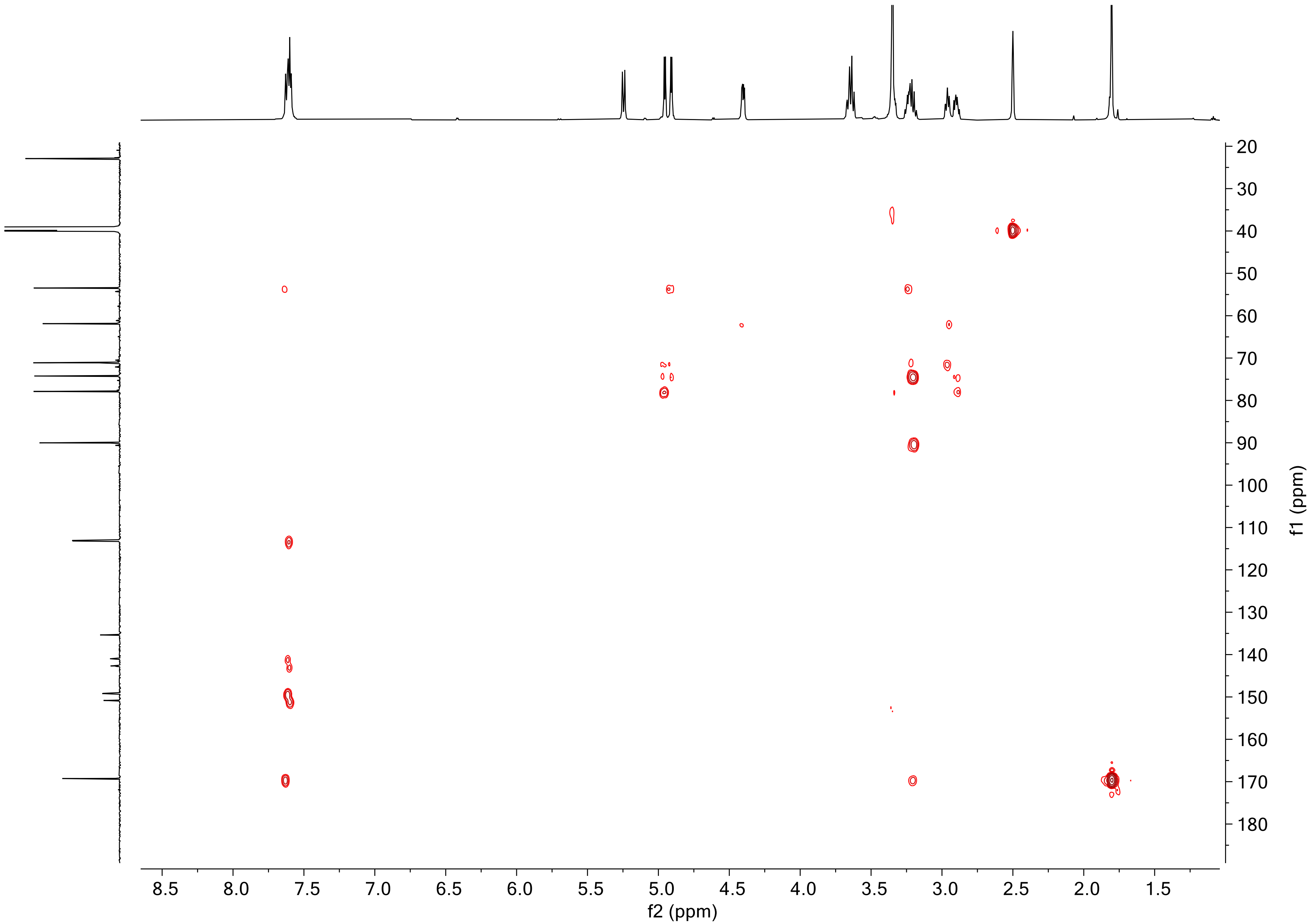

H-C HMBC NMR Spectrum of compound **β-SH7**

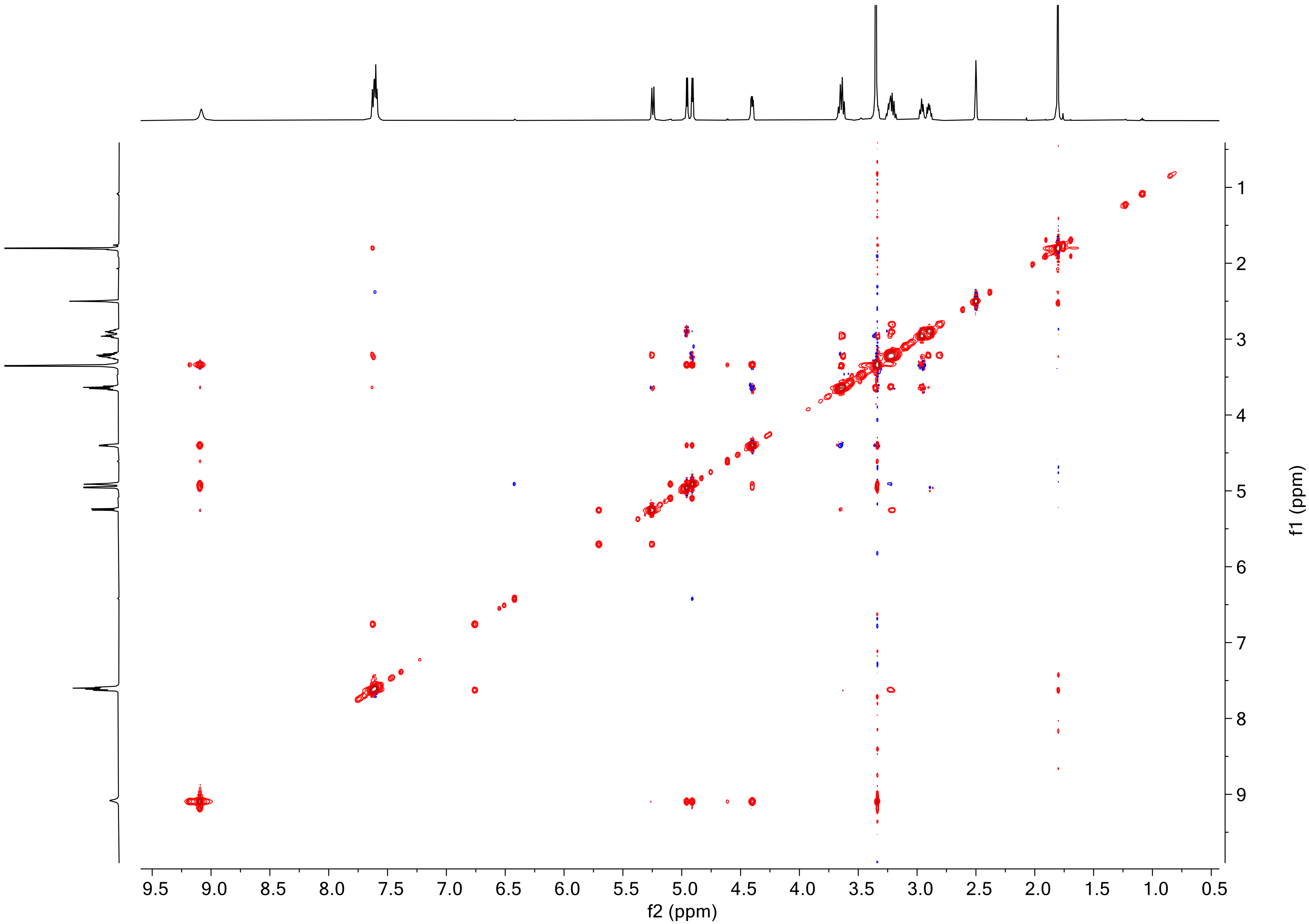

H-H NOESY NMR Spectrum of compound **β-SH7**

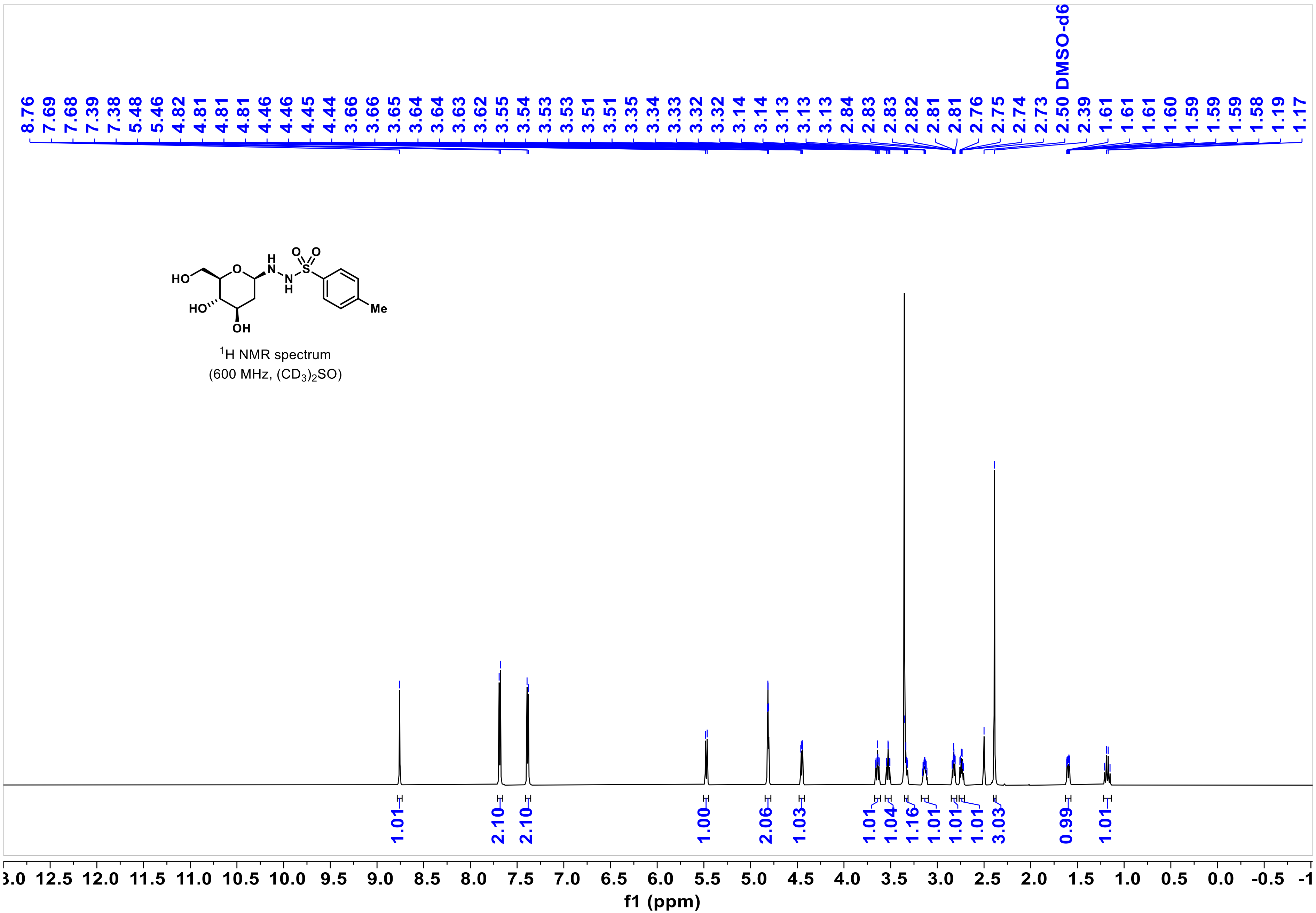

^{1}H NMR of compound **β-SH8**

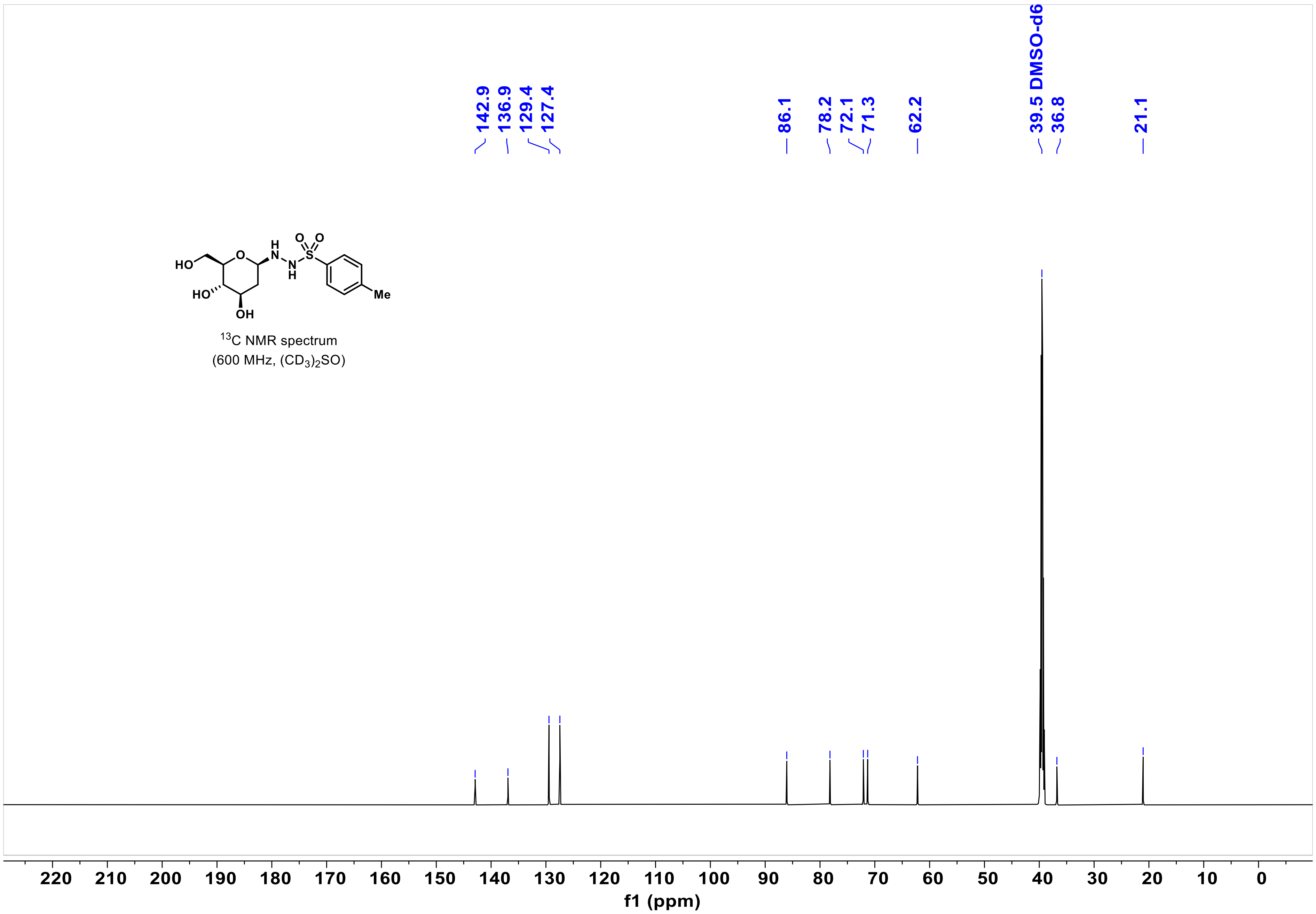

^{13}C NMR of compound **β-SH8**

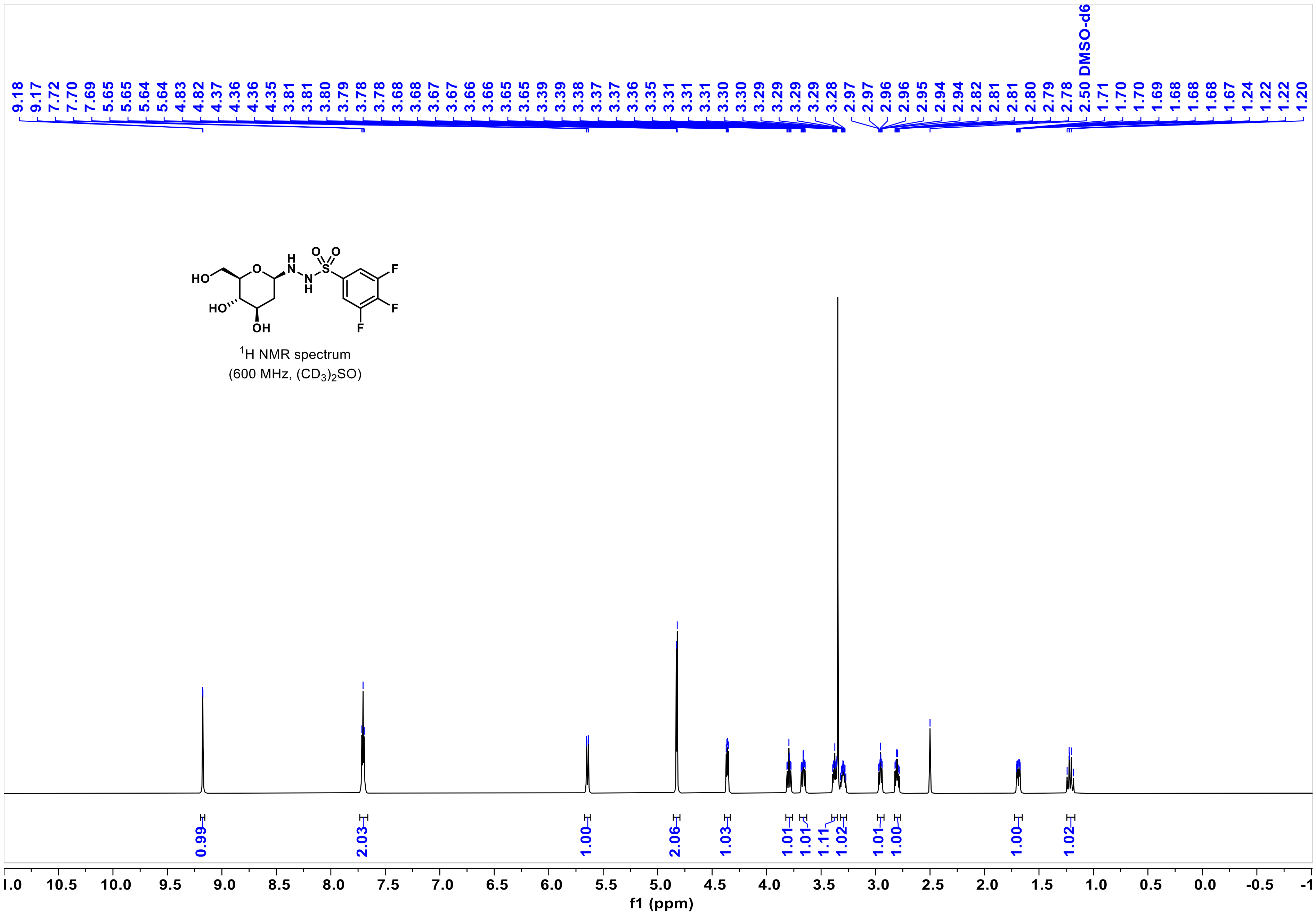

^{1}H NMR of compound **β-SH9**

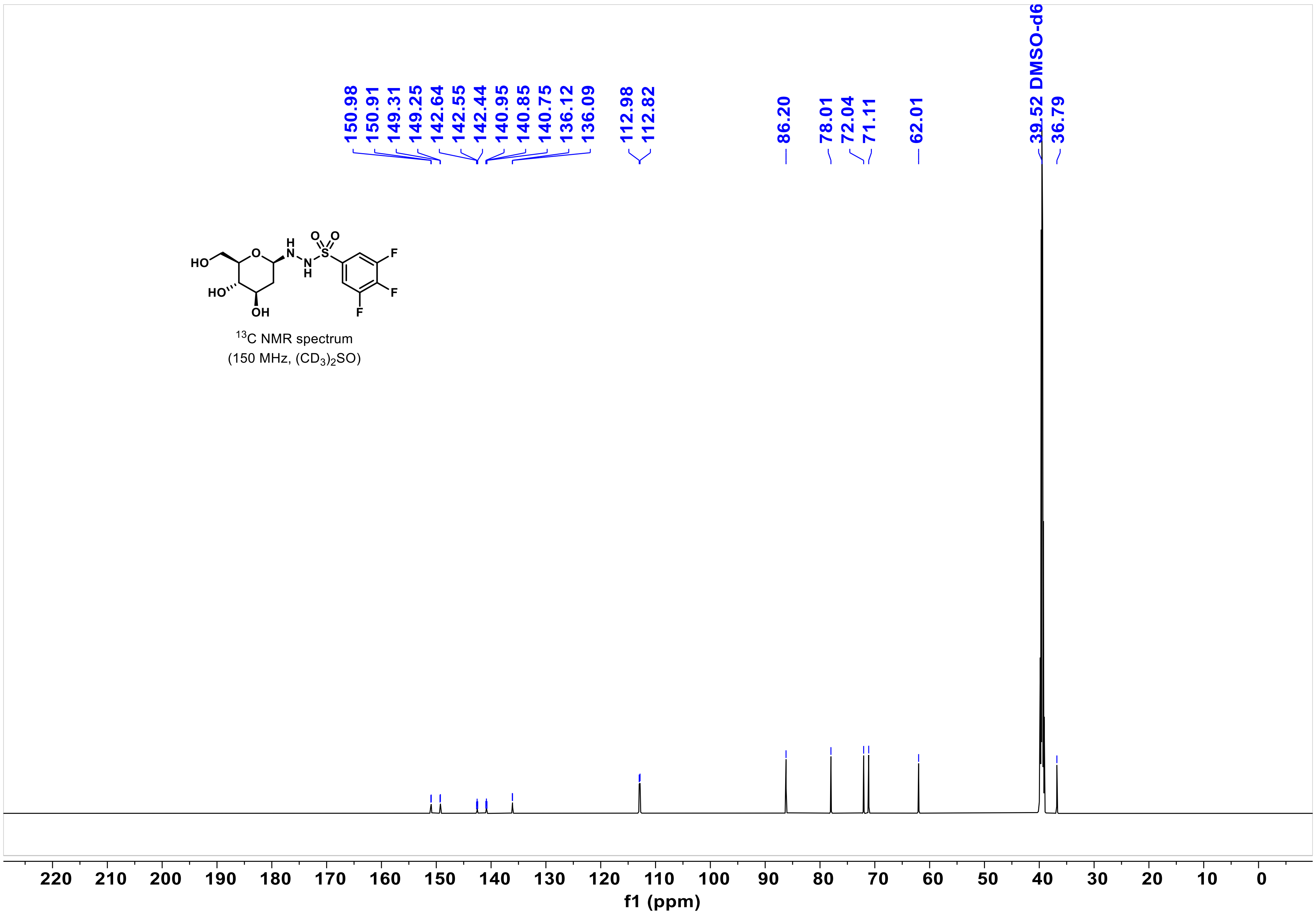

^{13}C NMR of compound **β-SH9**

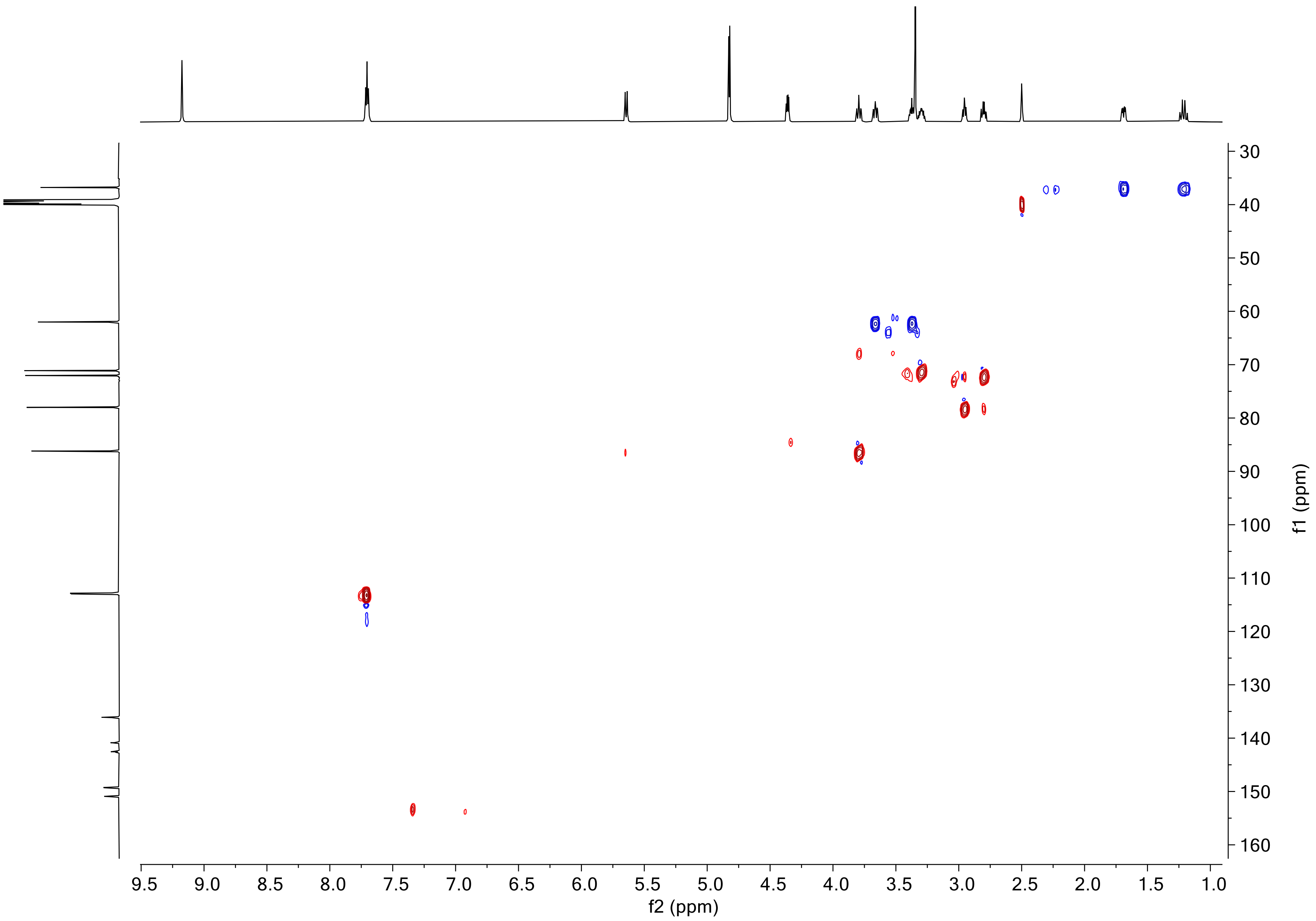

H-C HSQC NMR Spectrum of compound **β-SH9**

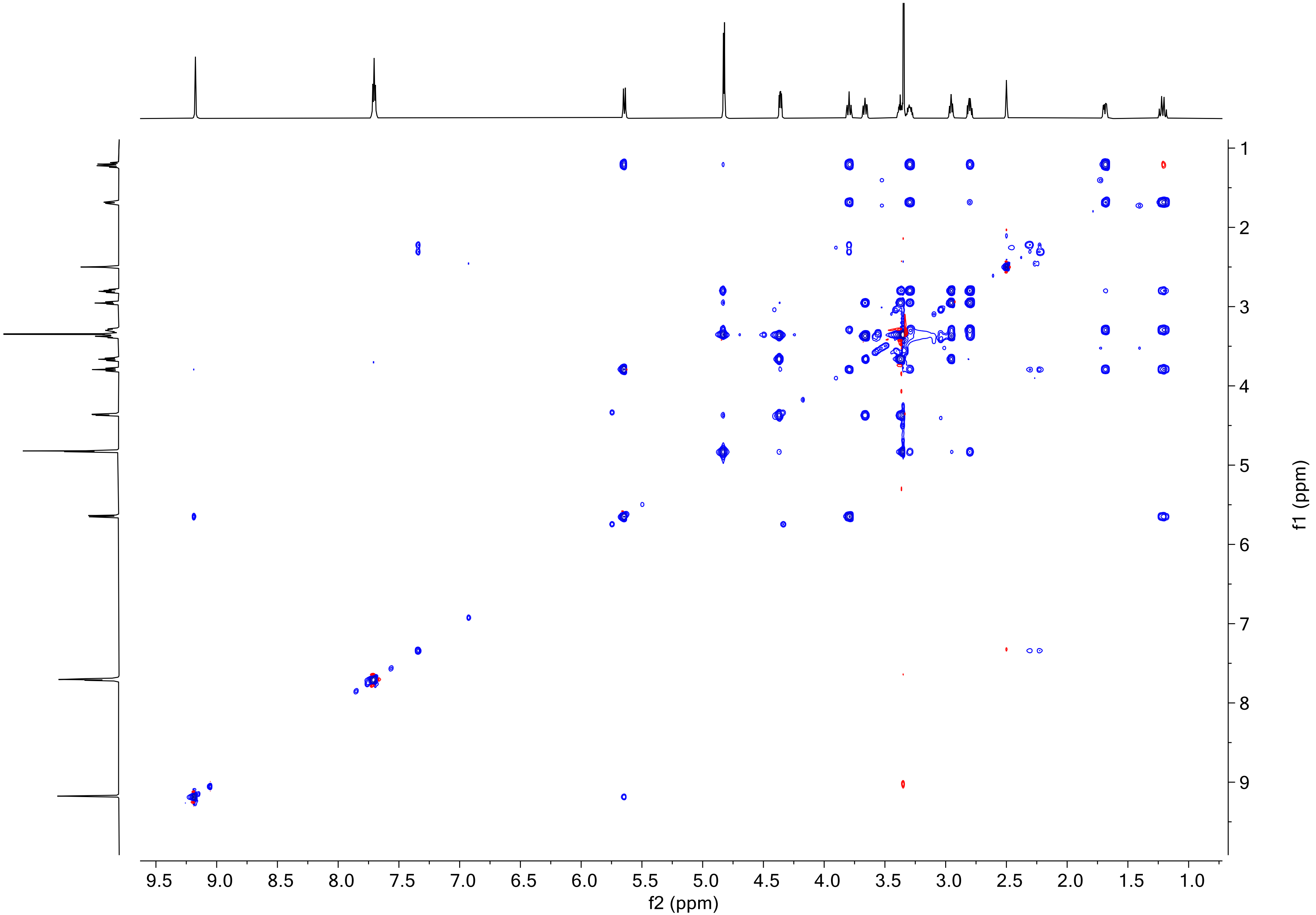

H-H COSY NMR Spectrum of compound **β-SH9**

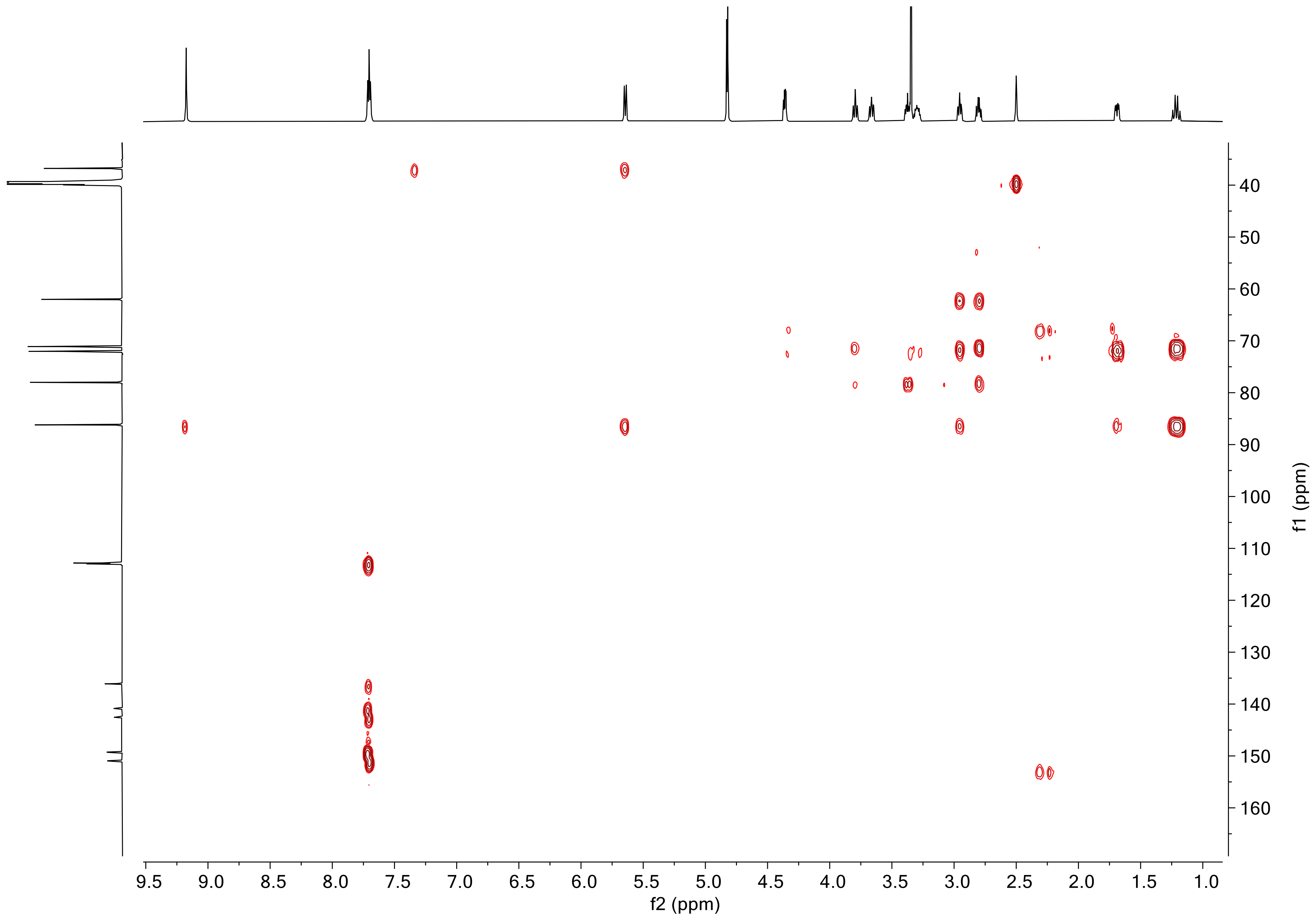

H-C HMBC NMR Spectrum of compound **β-SH9**

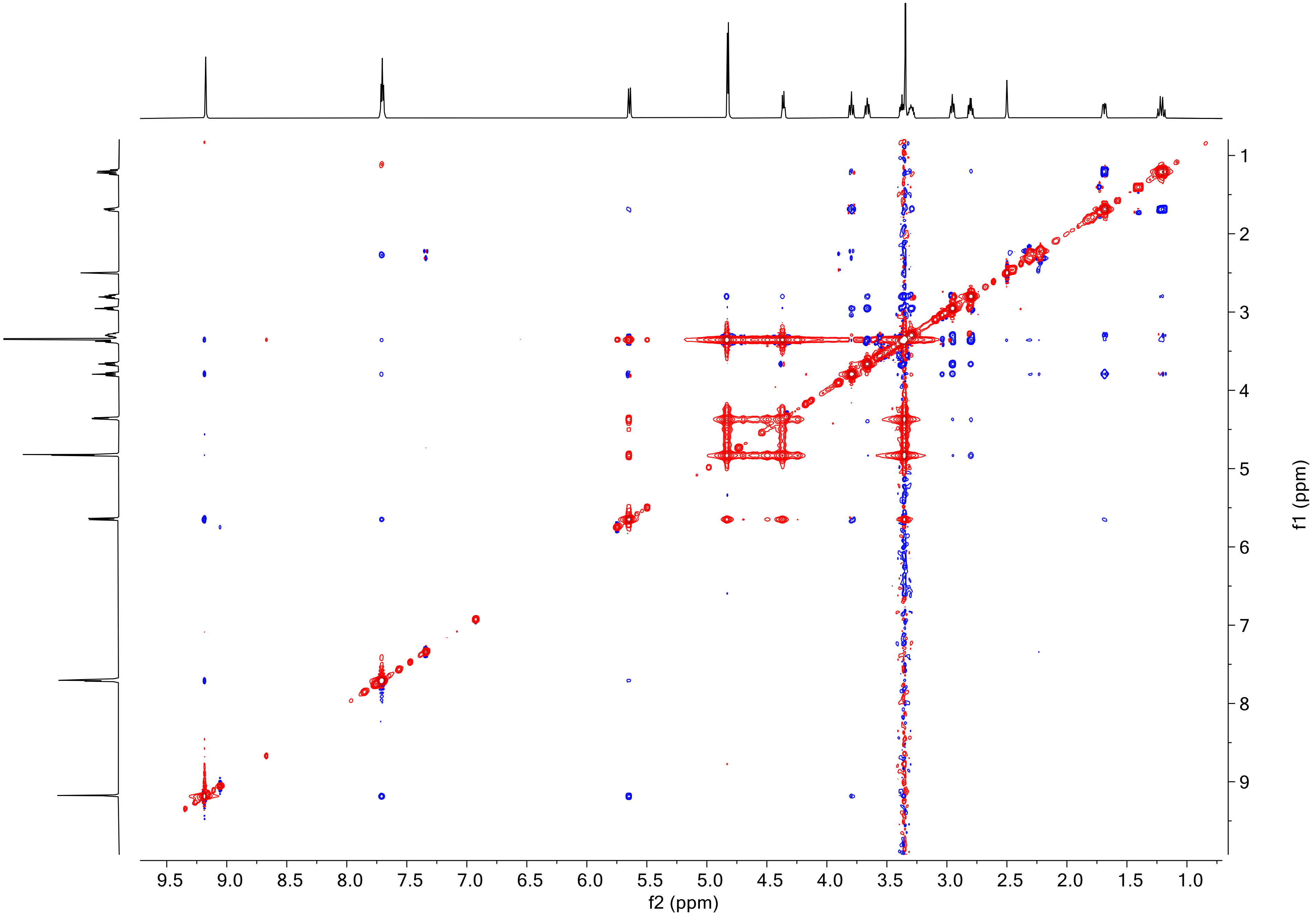

H-H NOESY NMR Spectrum of compound **β-SH9**

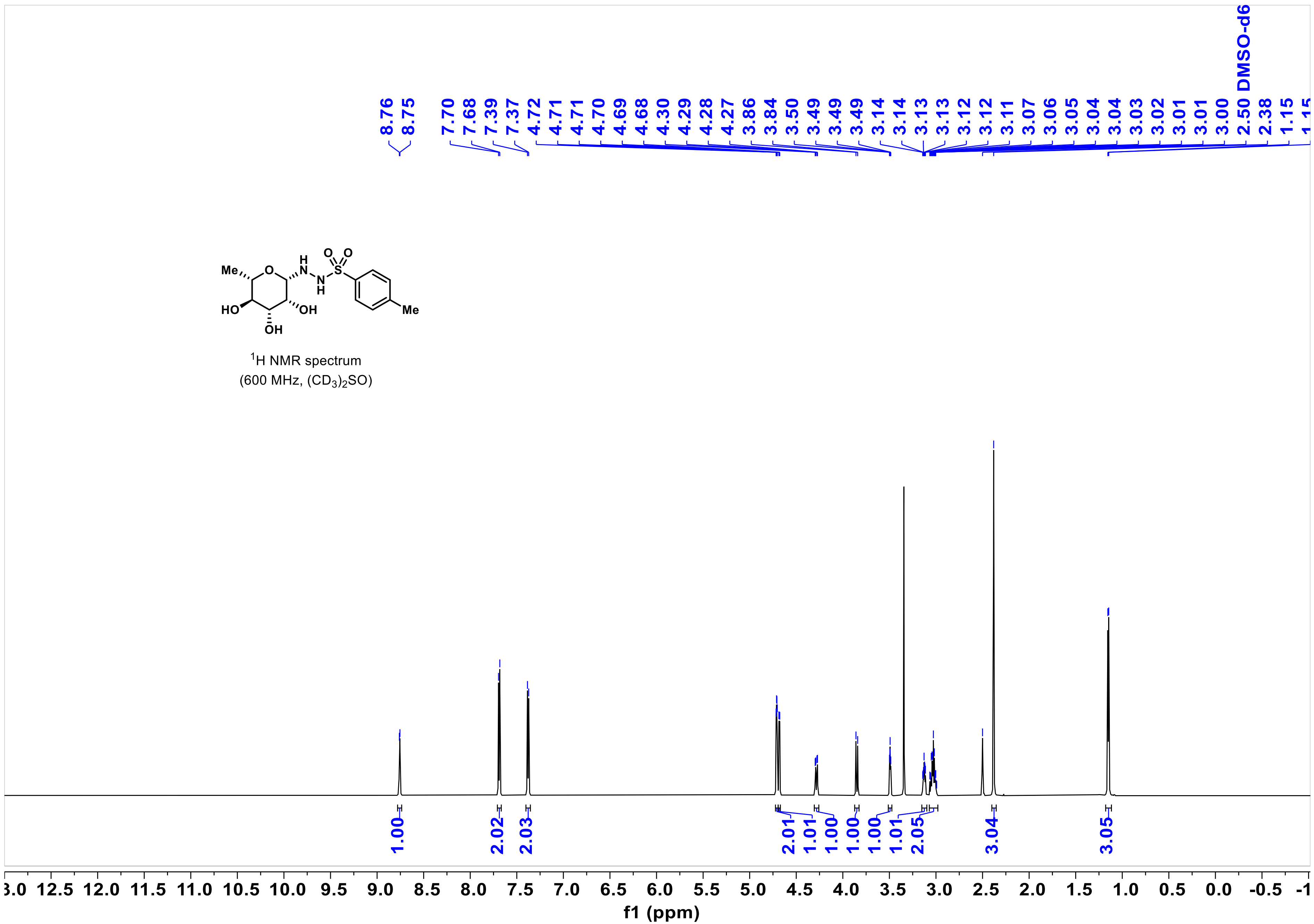

^{1}H NMR of compound **β-SH10**

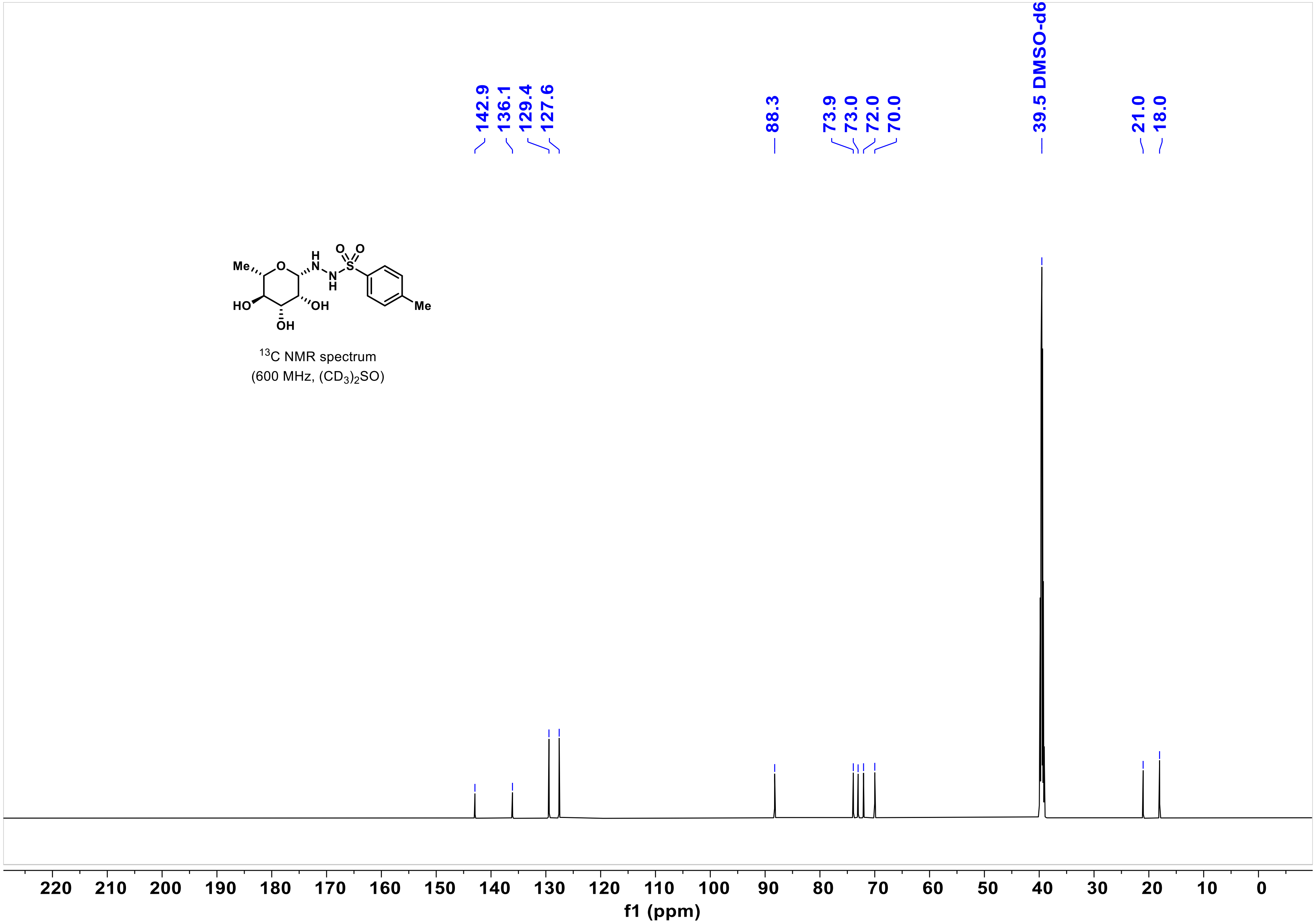

^{13}C NMR of compound **β-SH10**

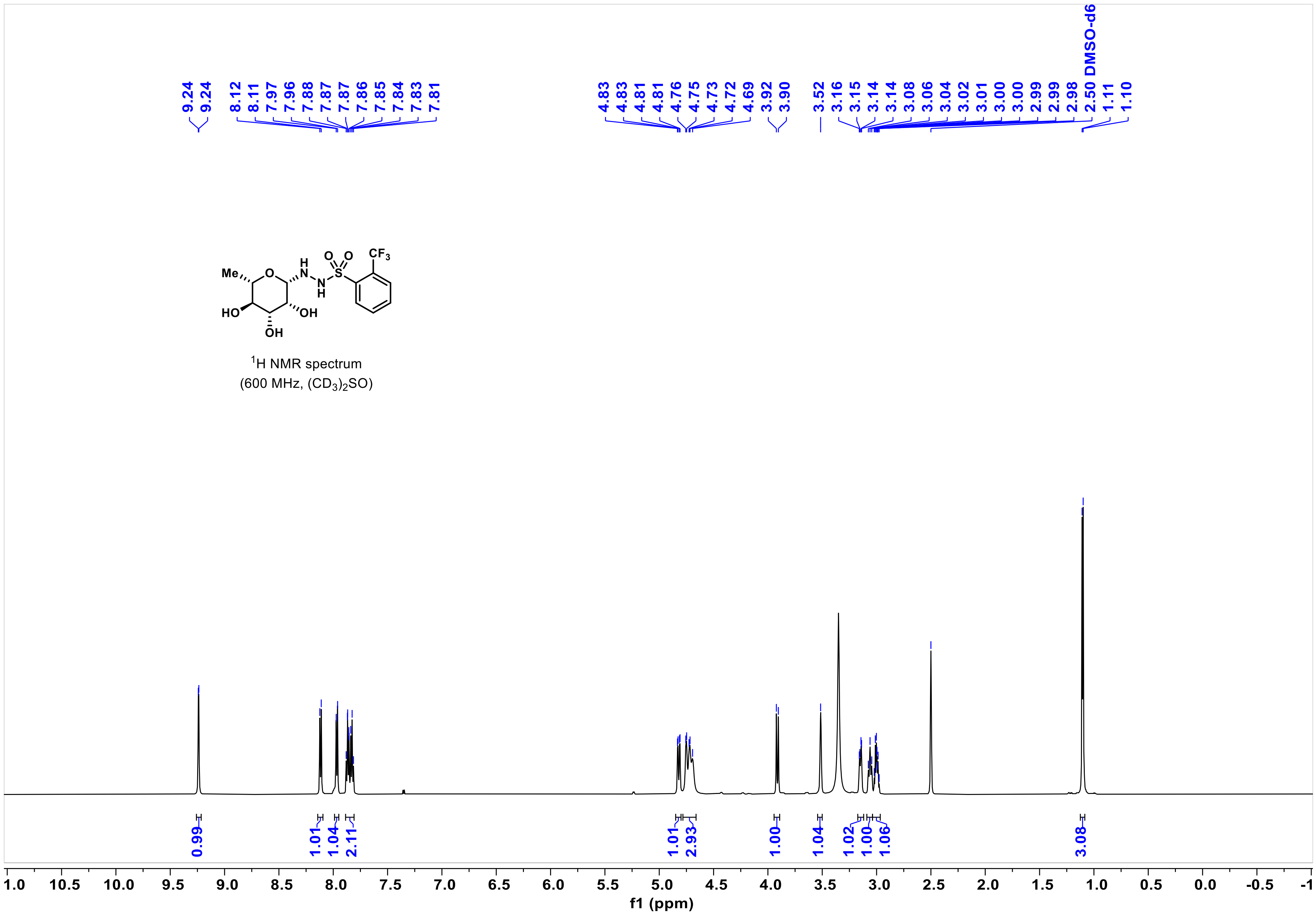

^{1}H NMR of compound **β-SH11**

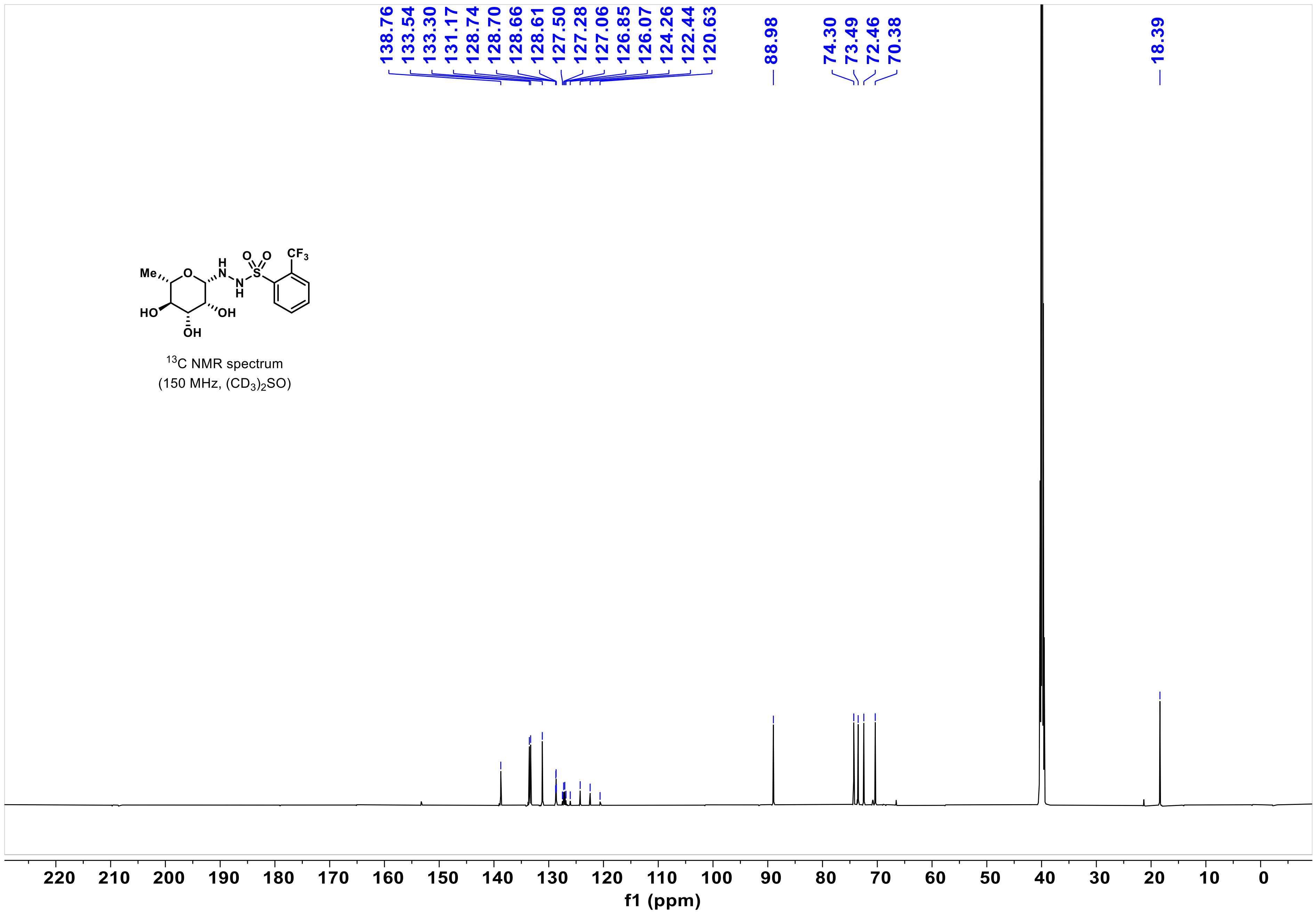

^{13}C NMR of compound **β-SH11**

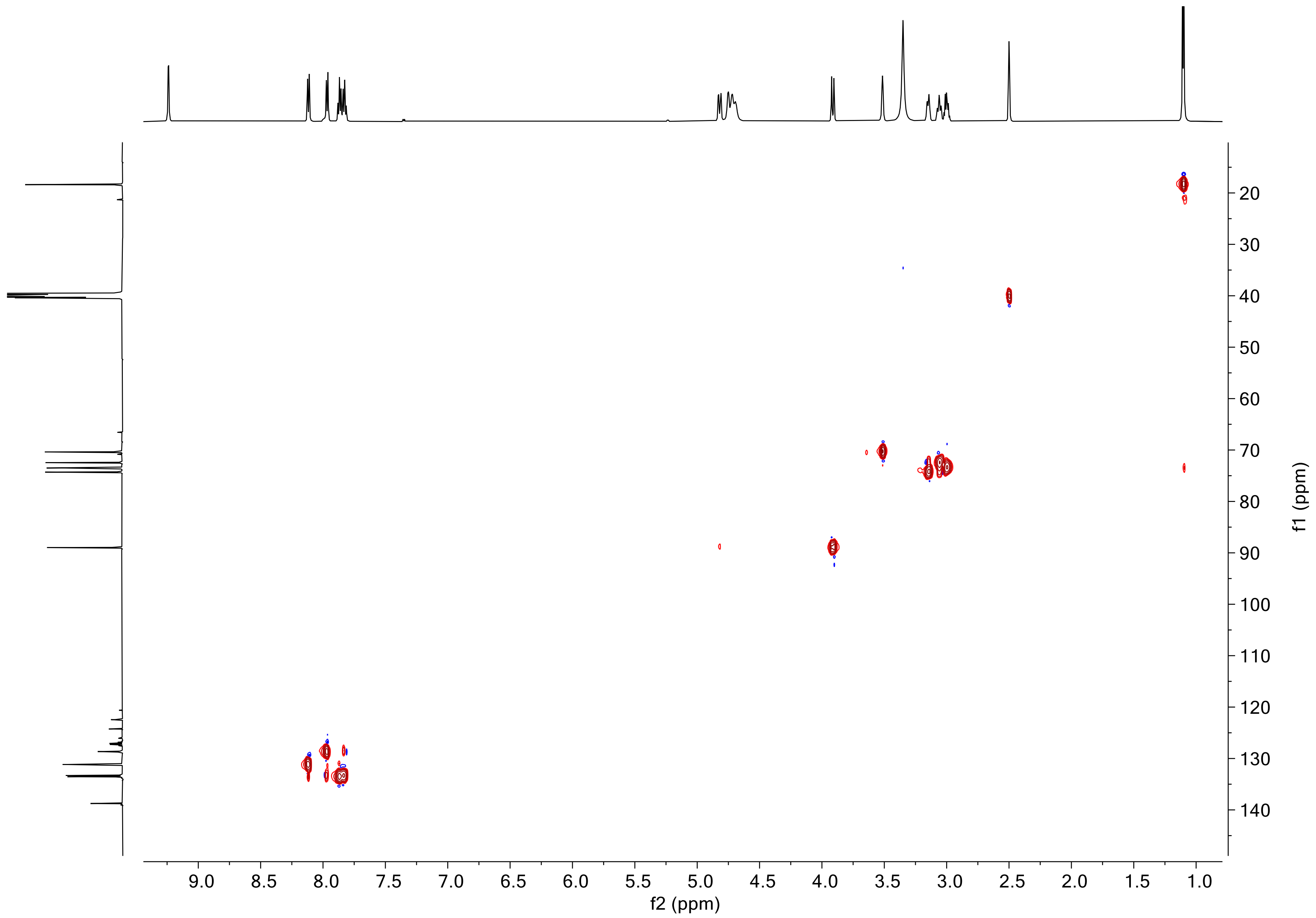

H-C HSQC NMR Spectrum of compound **β-SH11**

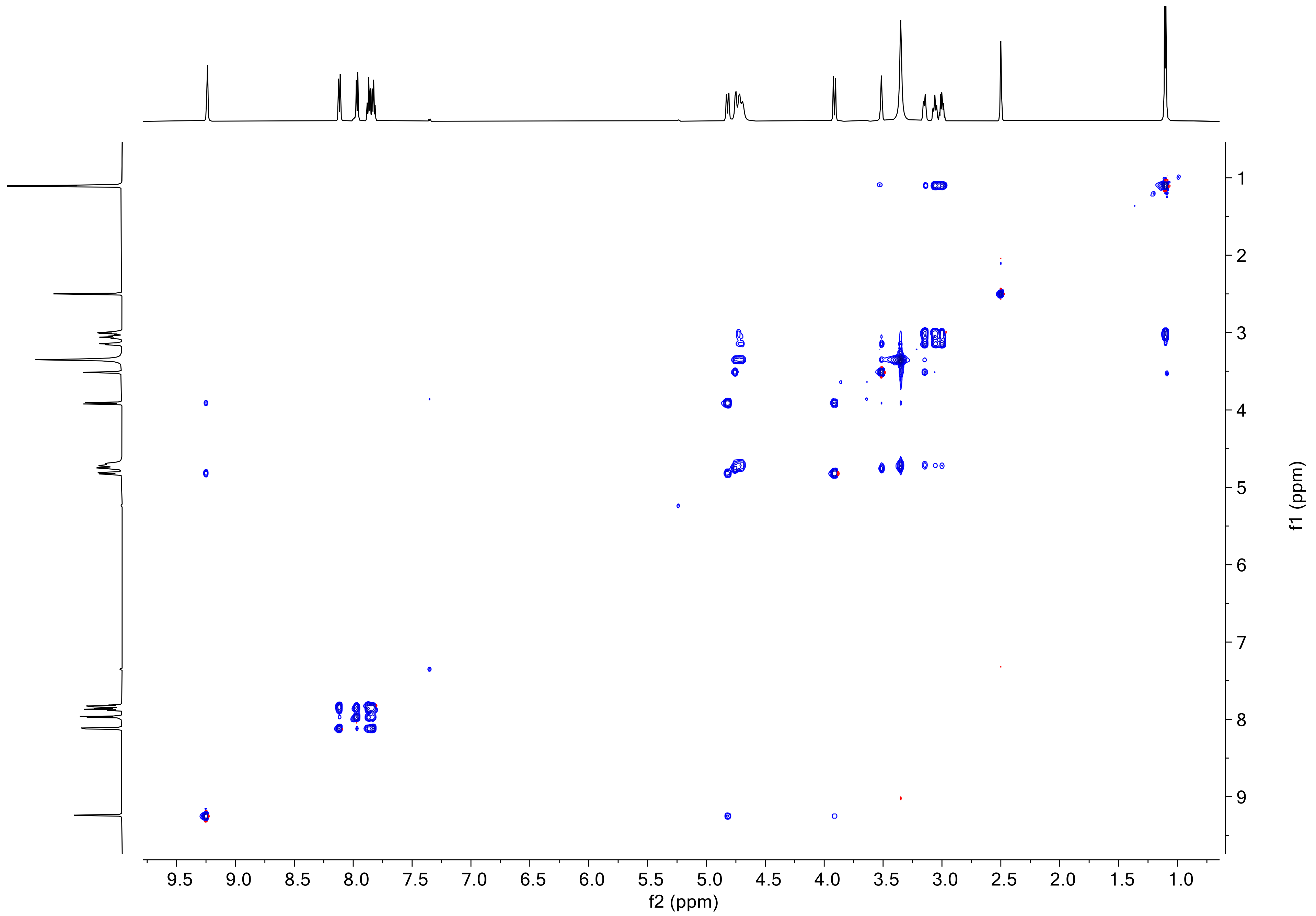

H-H COSY NMR Spectrum of compound **β-SH11**

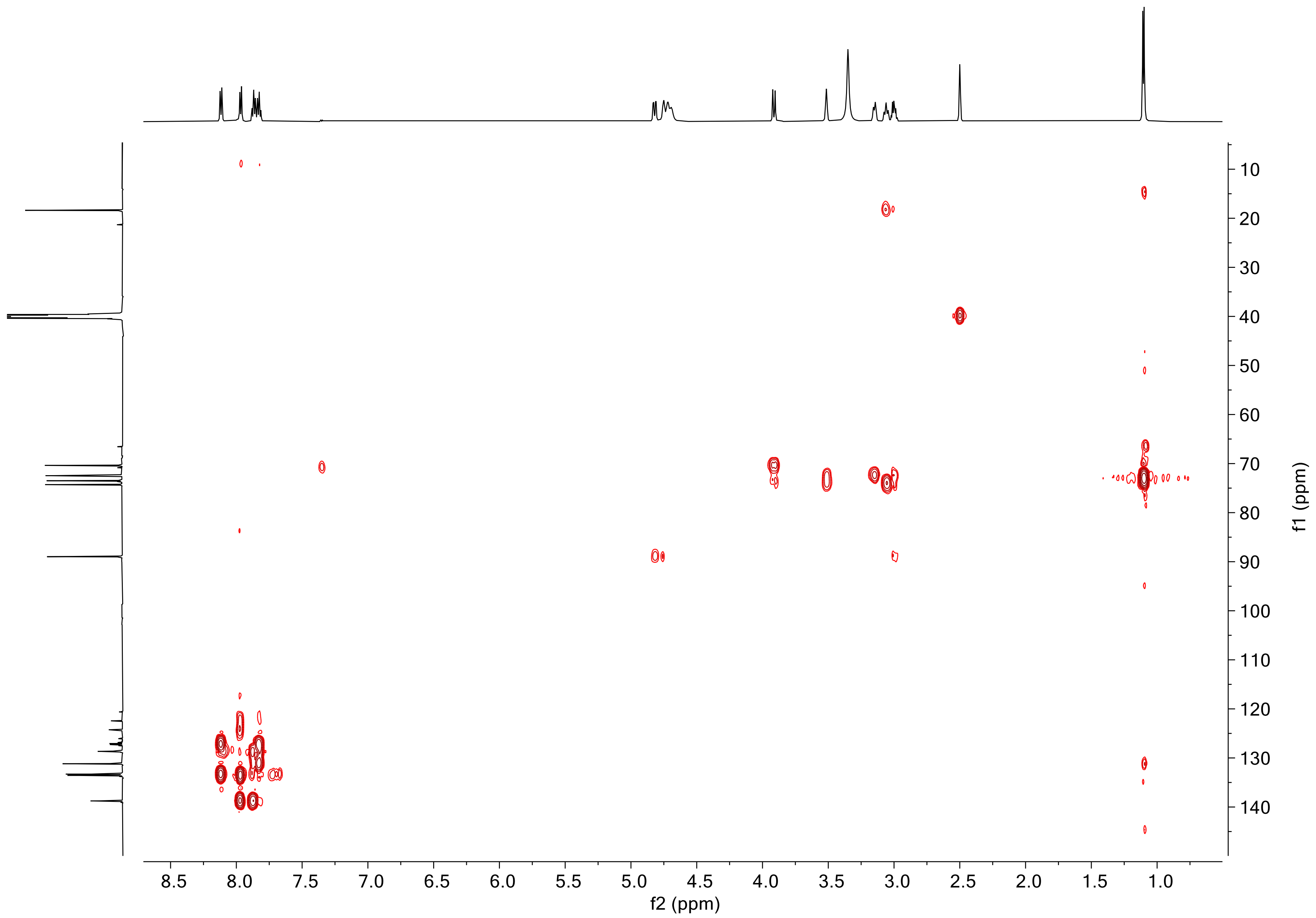

H-C HMBC NMR Spectrum of compound **β-SH11**

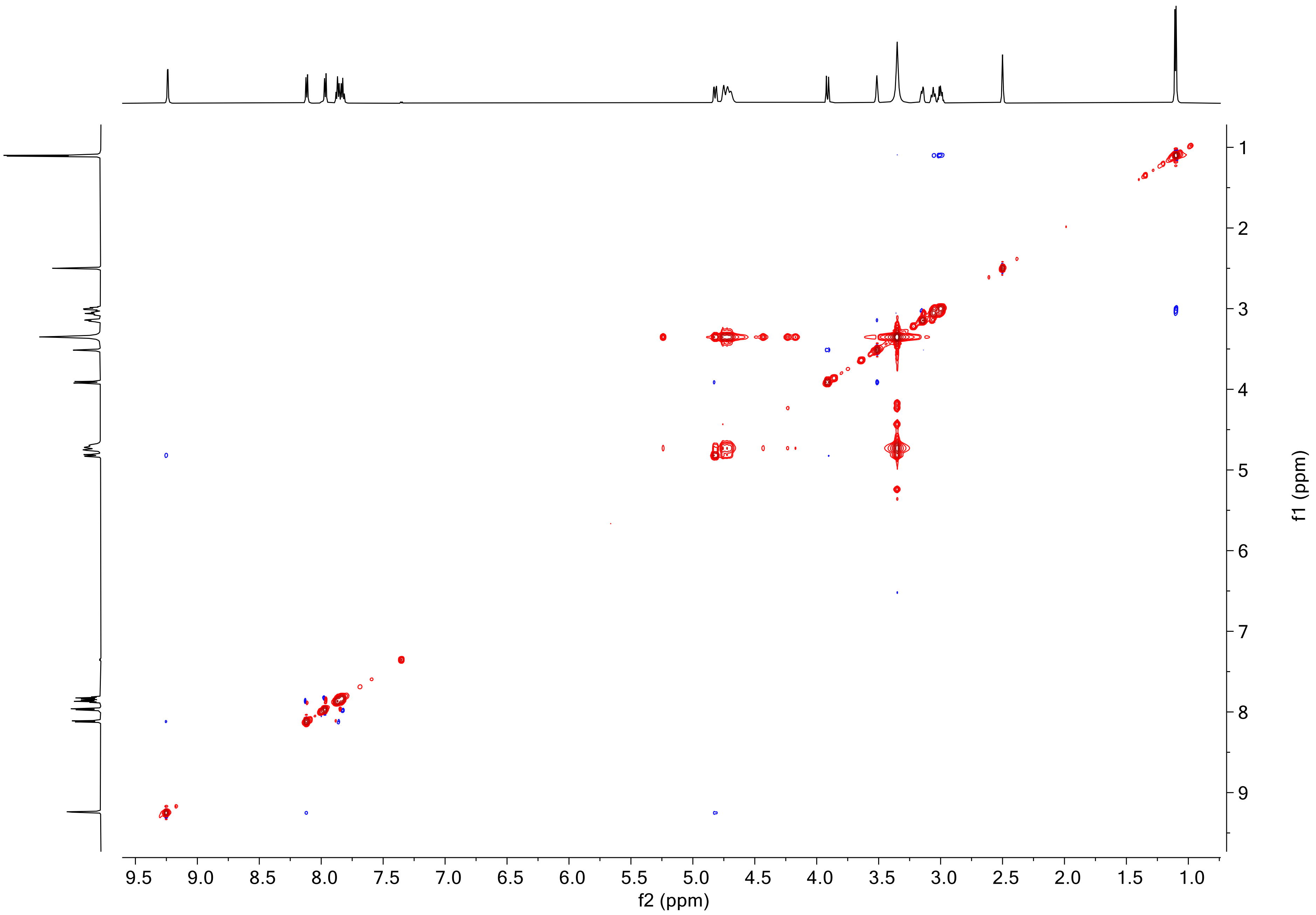

H-H NOESY NMR Spectrum of compound **β-SH11**

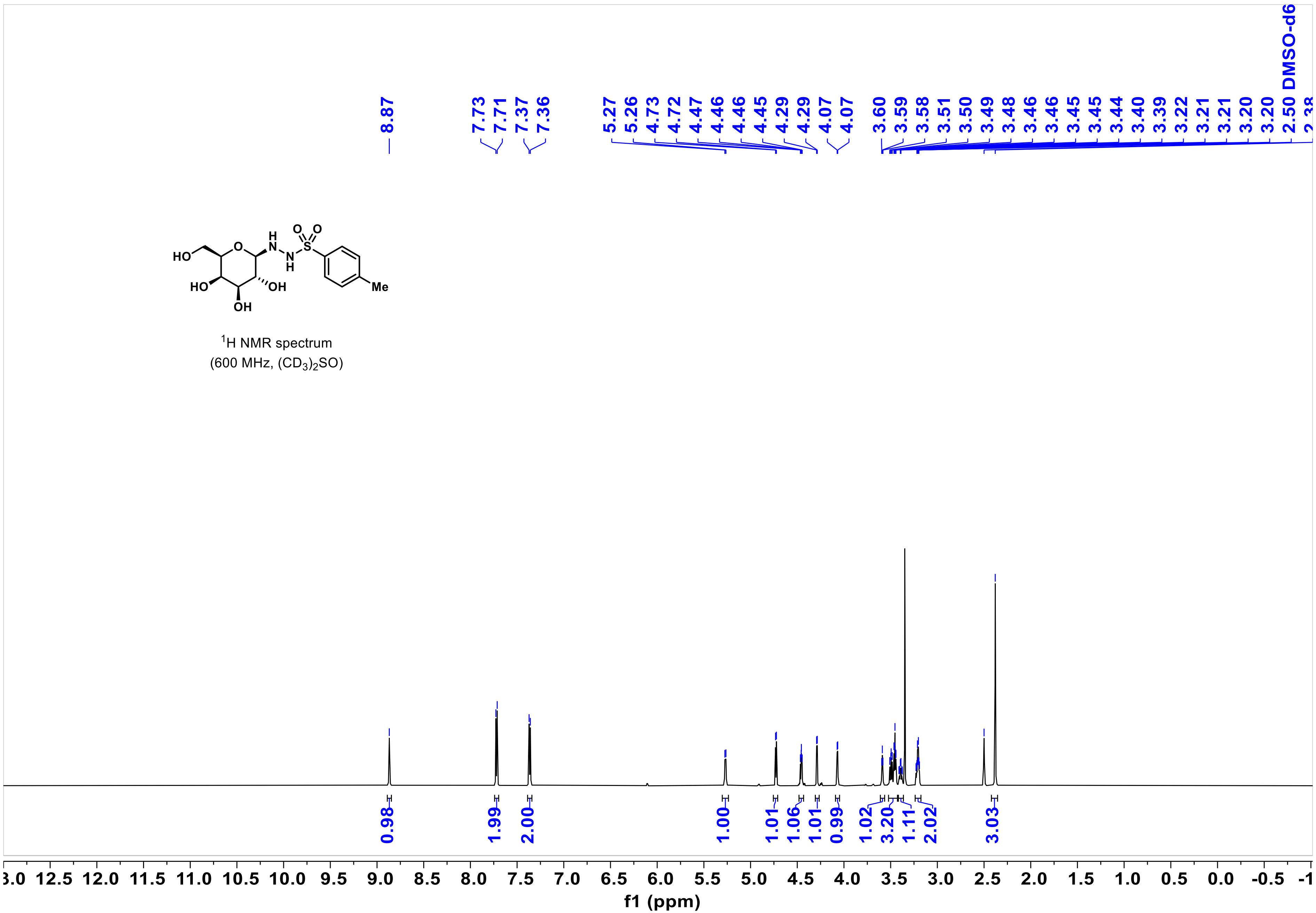

^{1}H NMR of compound **β-SH12**

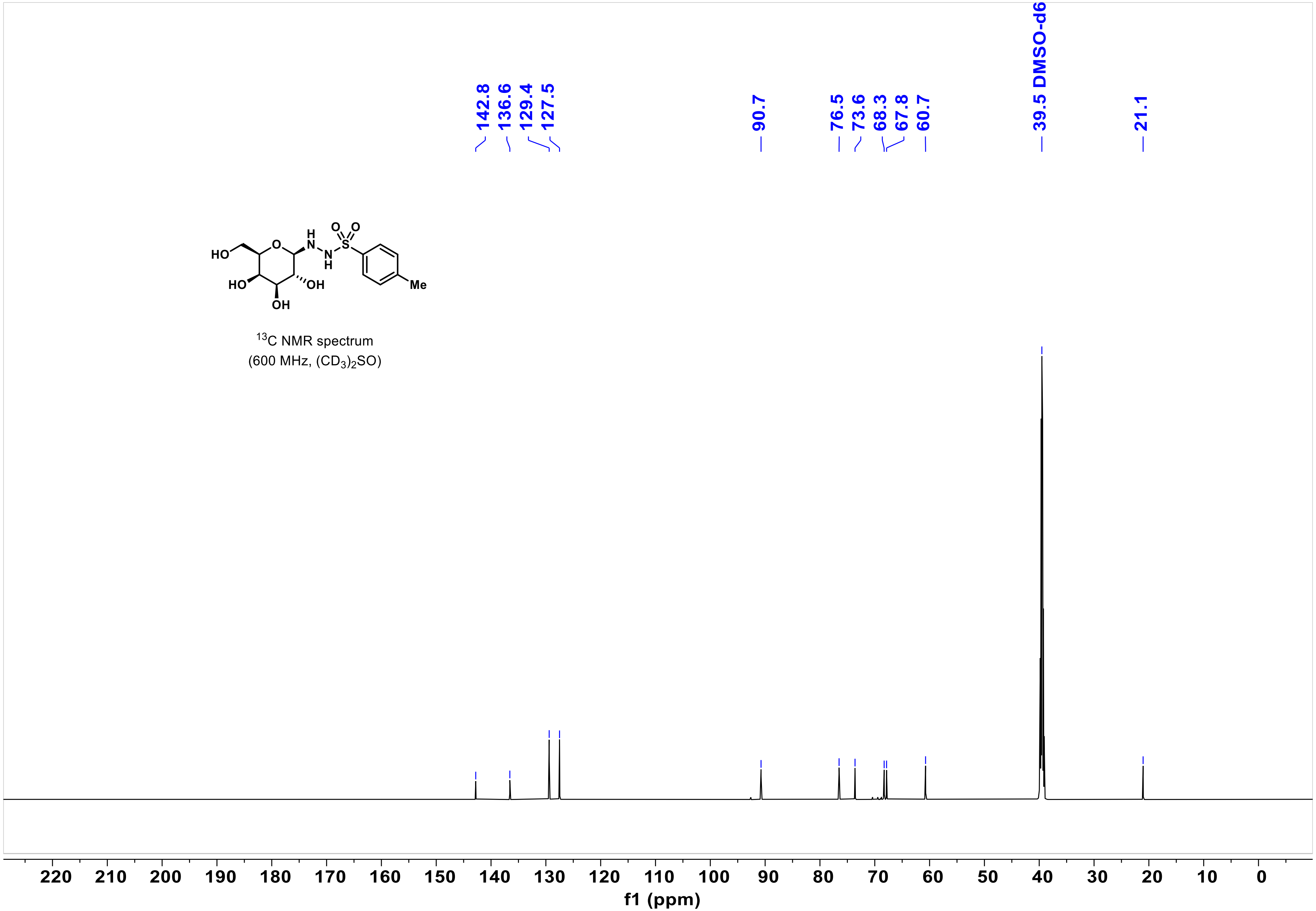

^{13}C NMR of compound **β-SH12**

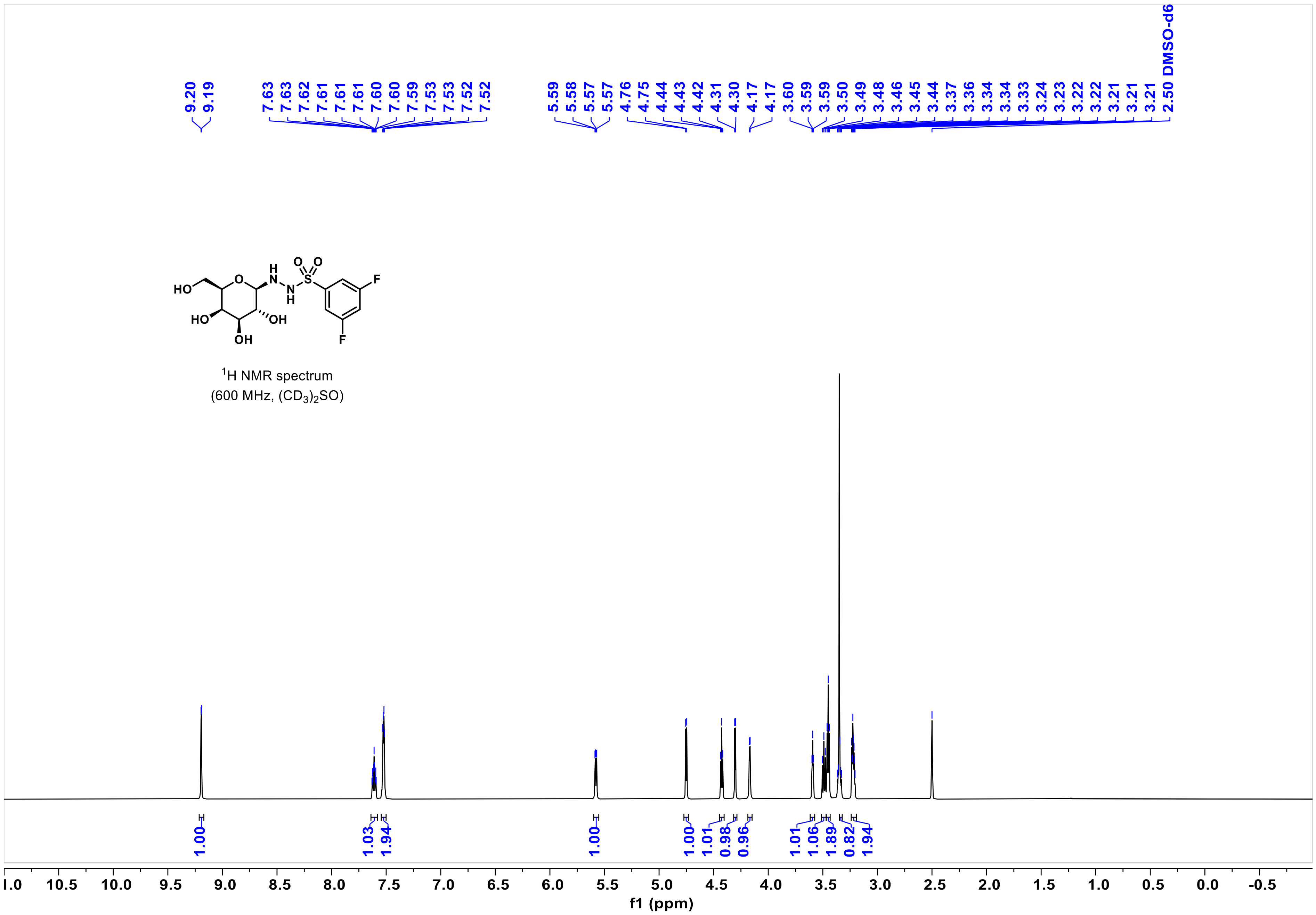

^{1}H NMR of compound **β-SH13**

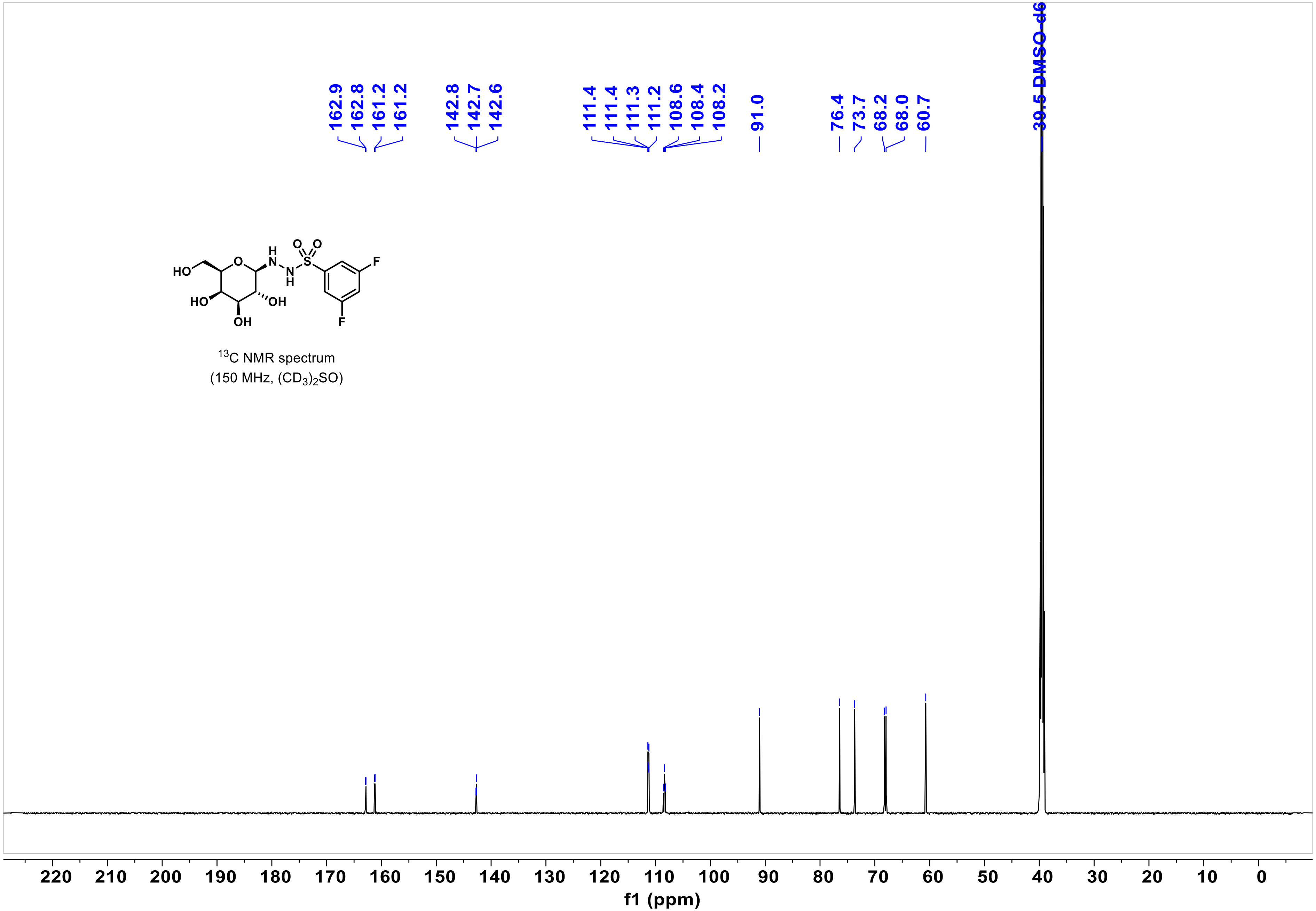

^{13}C NMR of compound **β-SH13**

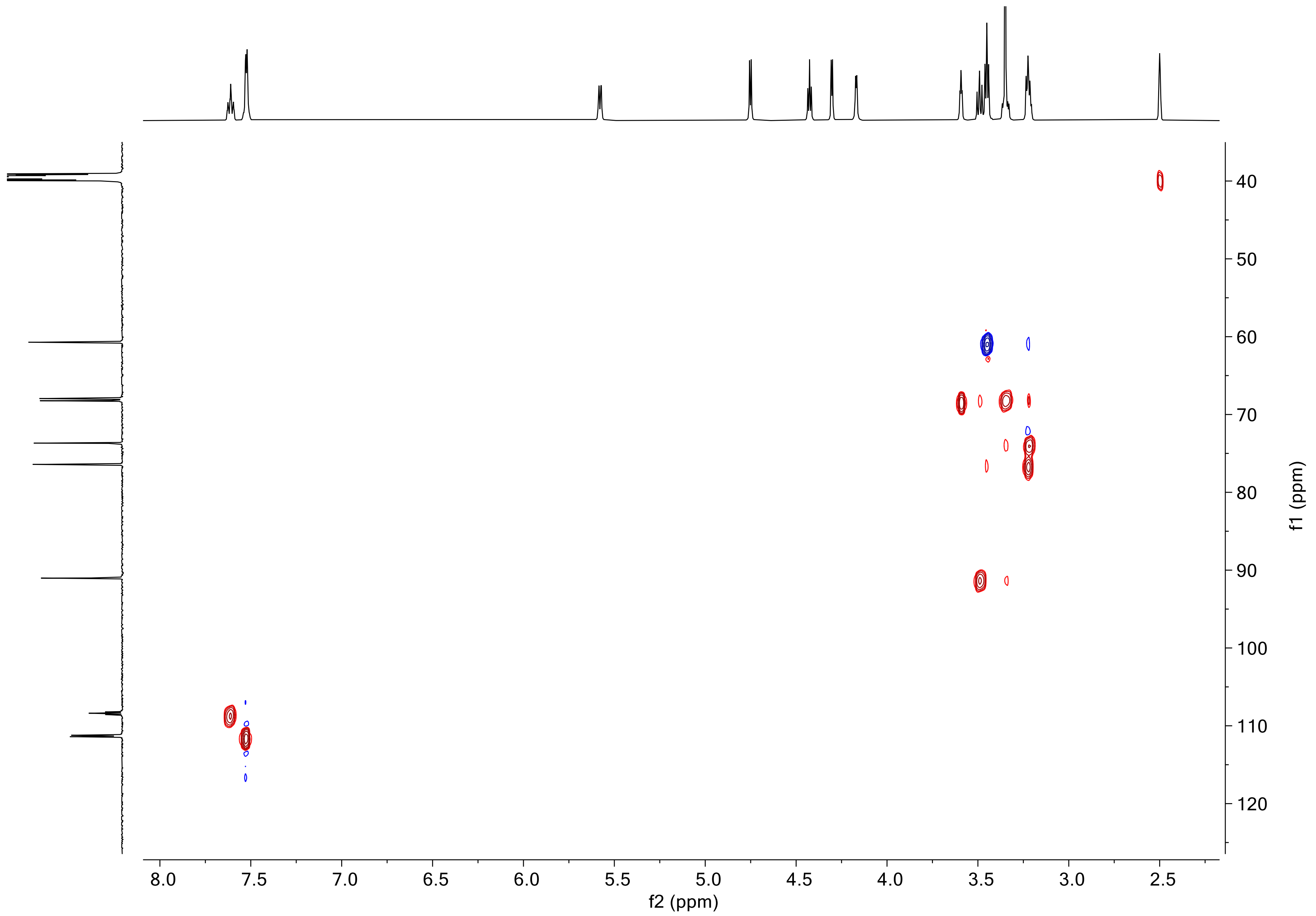

H-C HSQC NMR Spectrum of compound **β-SH13**

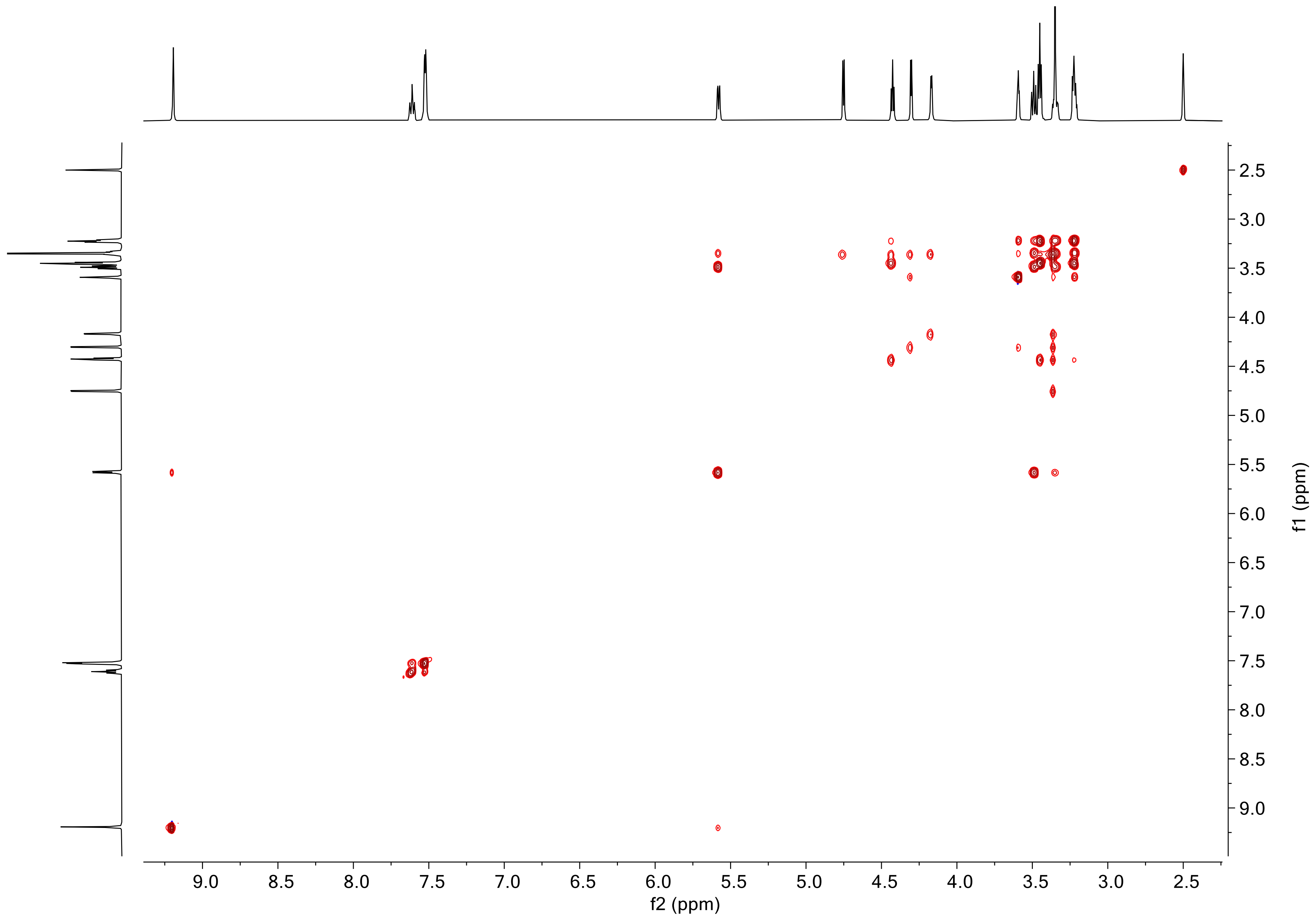

H-H COSY NMR Spectrum of compound **β-SH13**

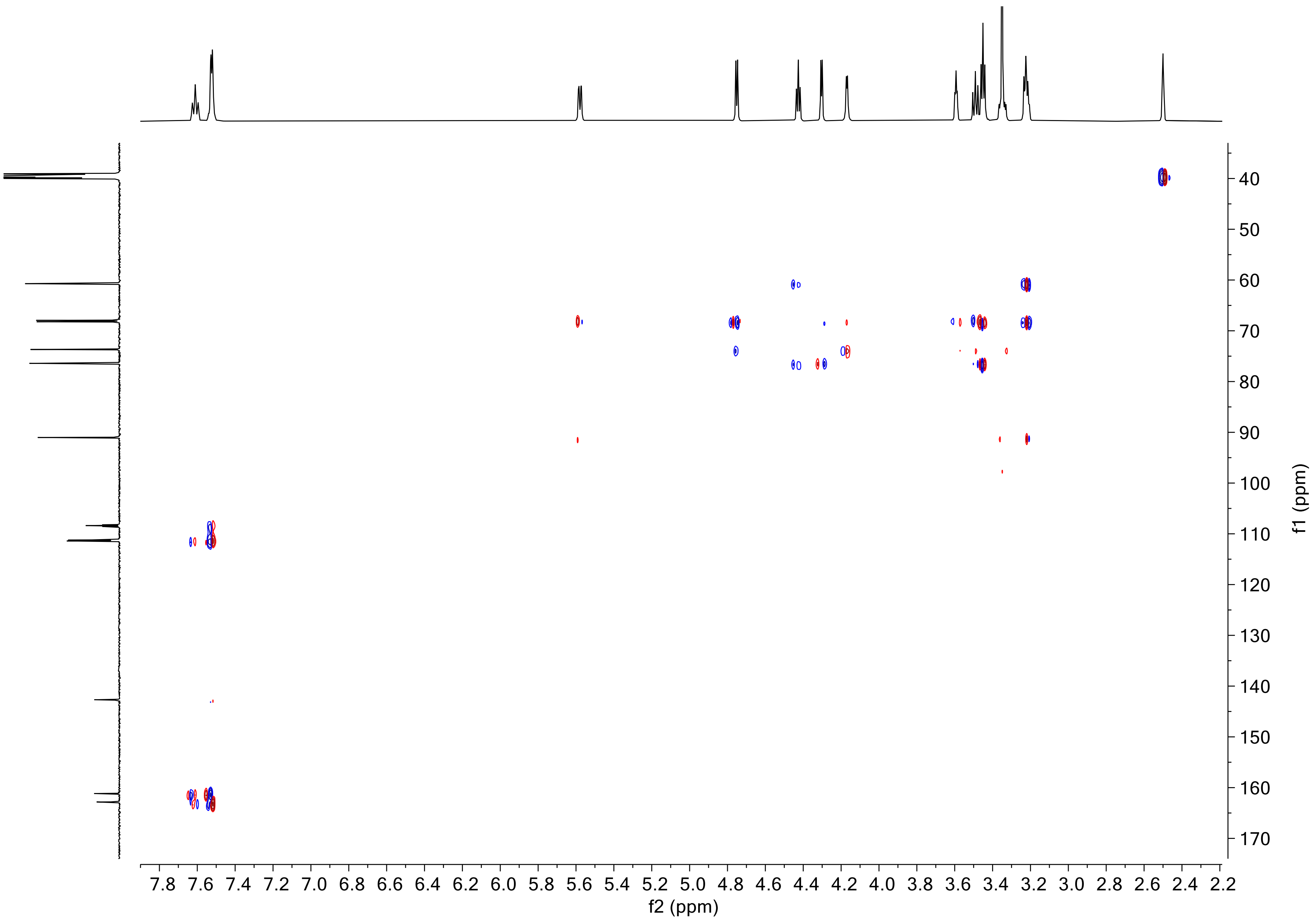

H-C HMBC NMR Spectrum of compound **β-SH13**

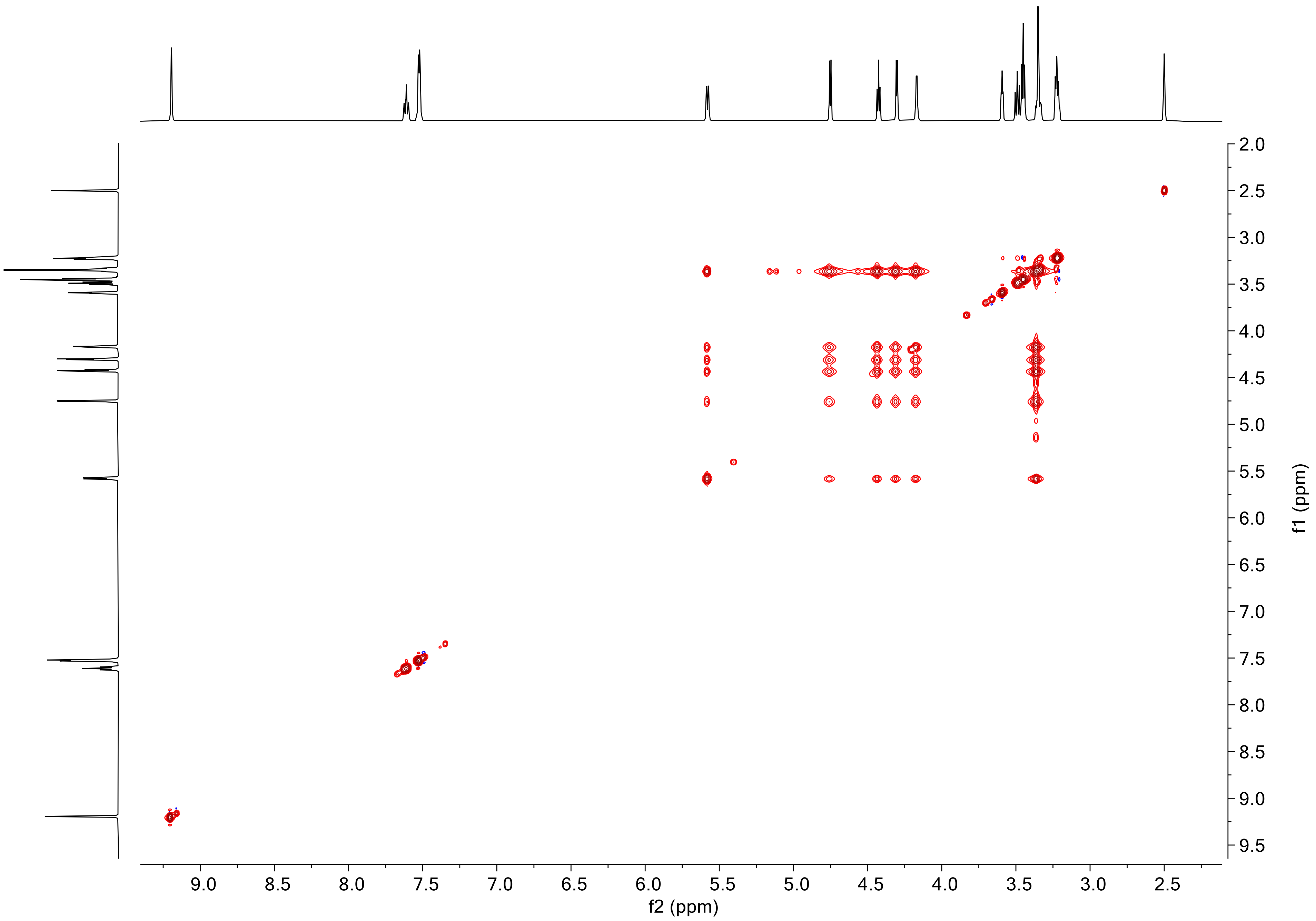

H-H NOESY NMR Spectrum of compound **β-SH13**

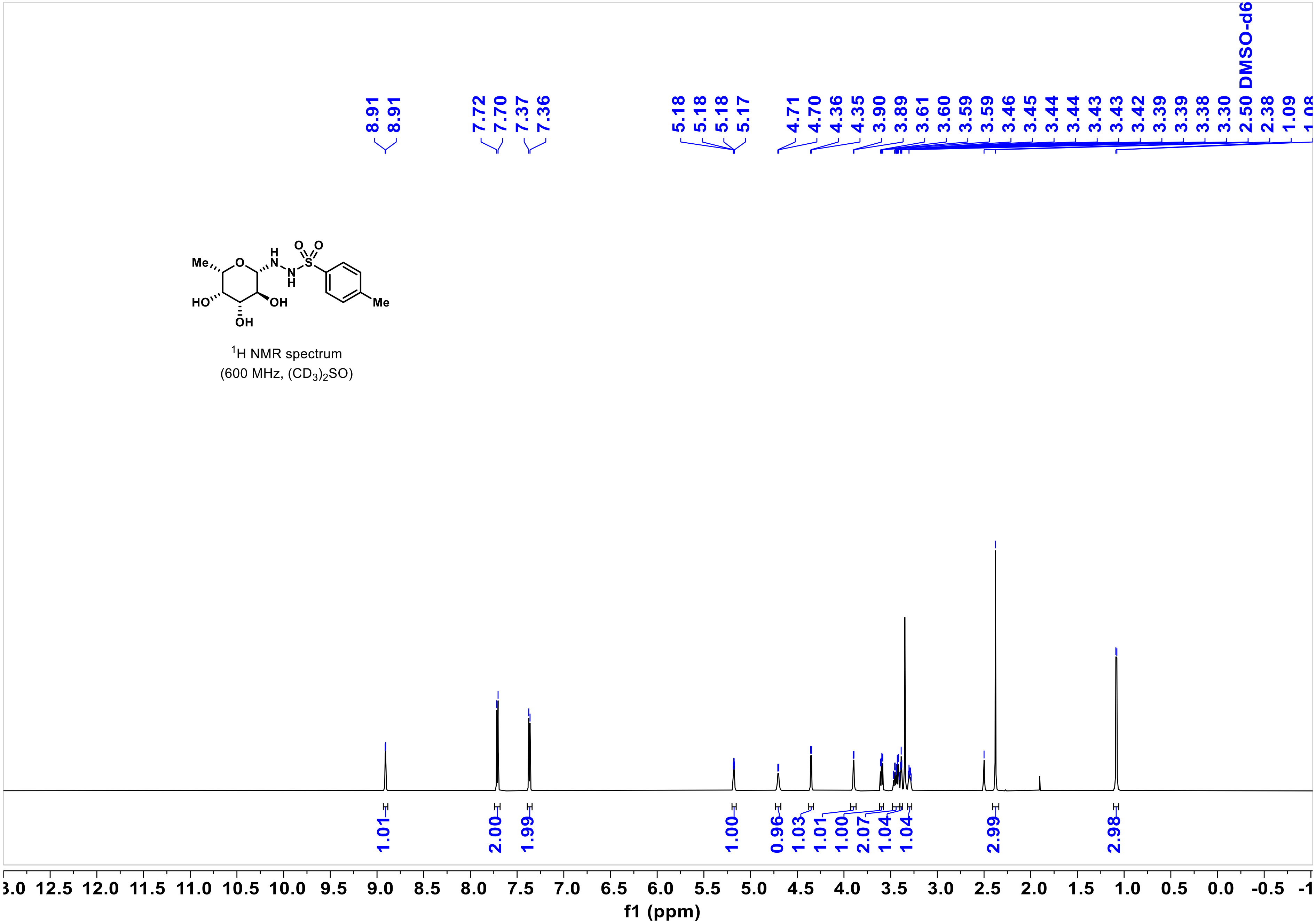

^{1}H NMR of compound **β-SH14**

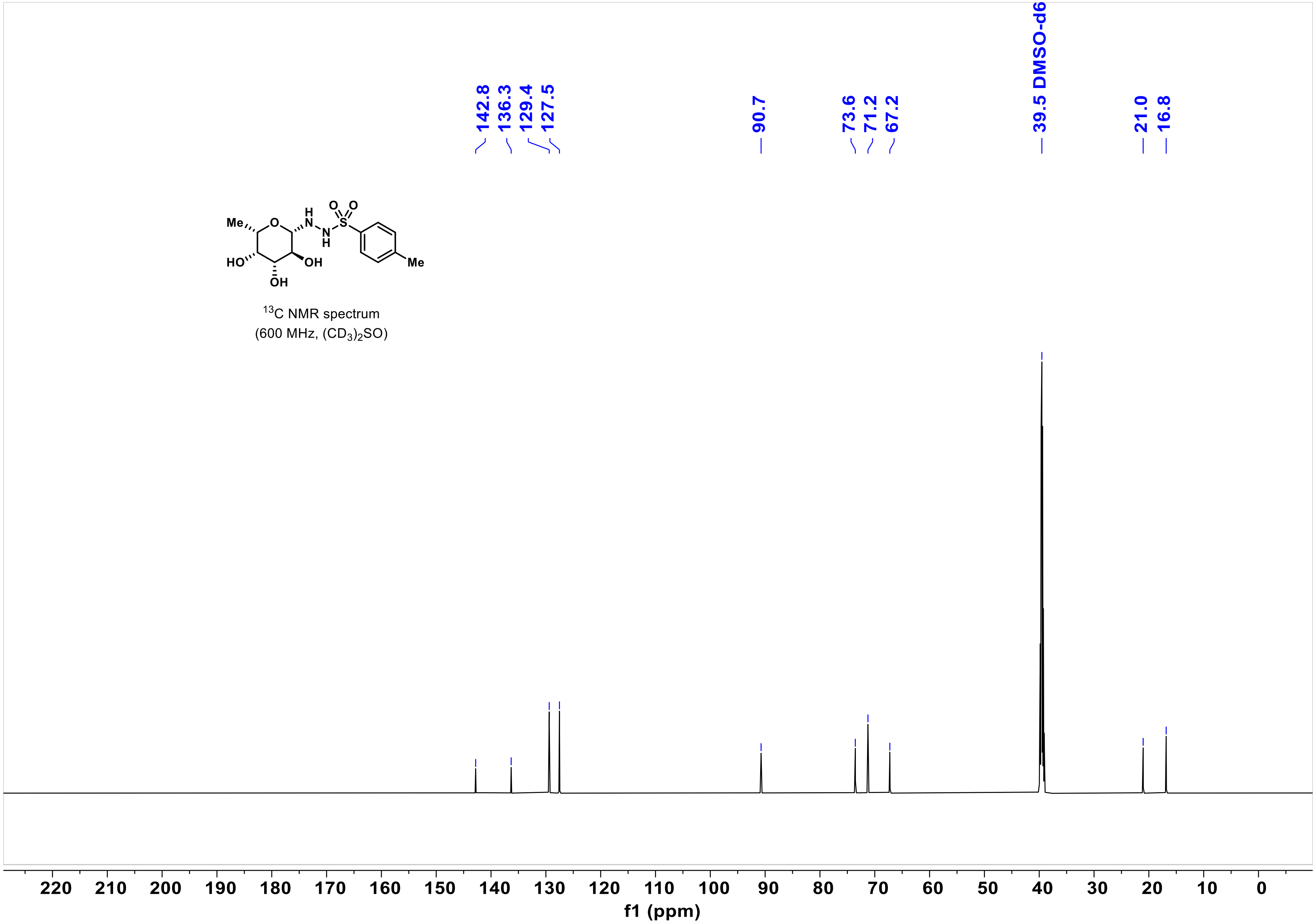

^{13}C NMR of compound **β-SH14**

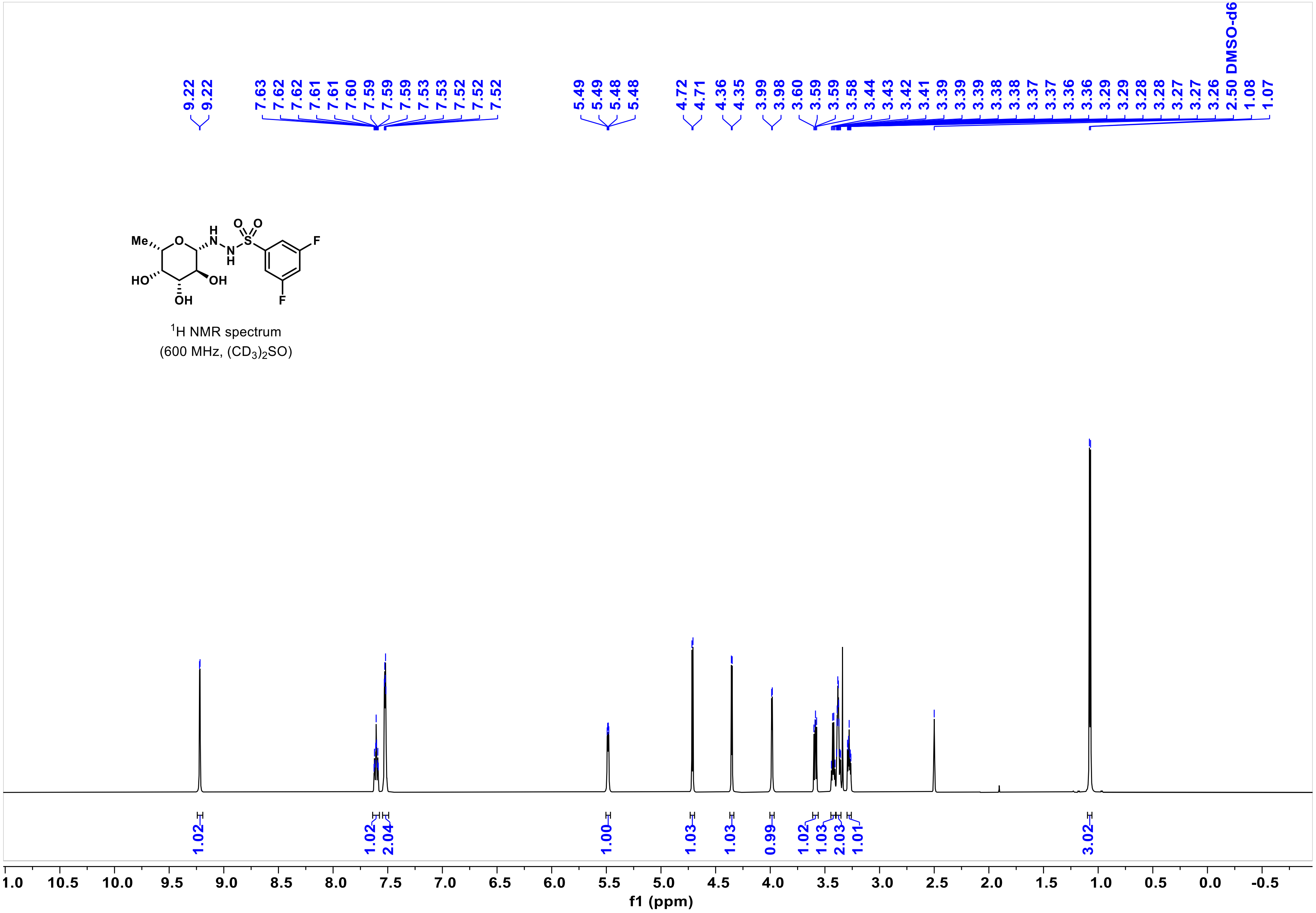

^{1}H NMR of compound **β-SH15**

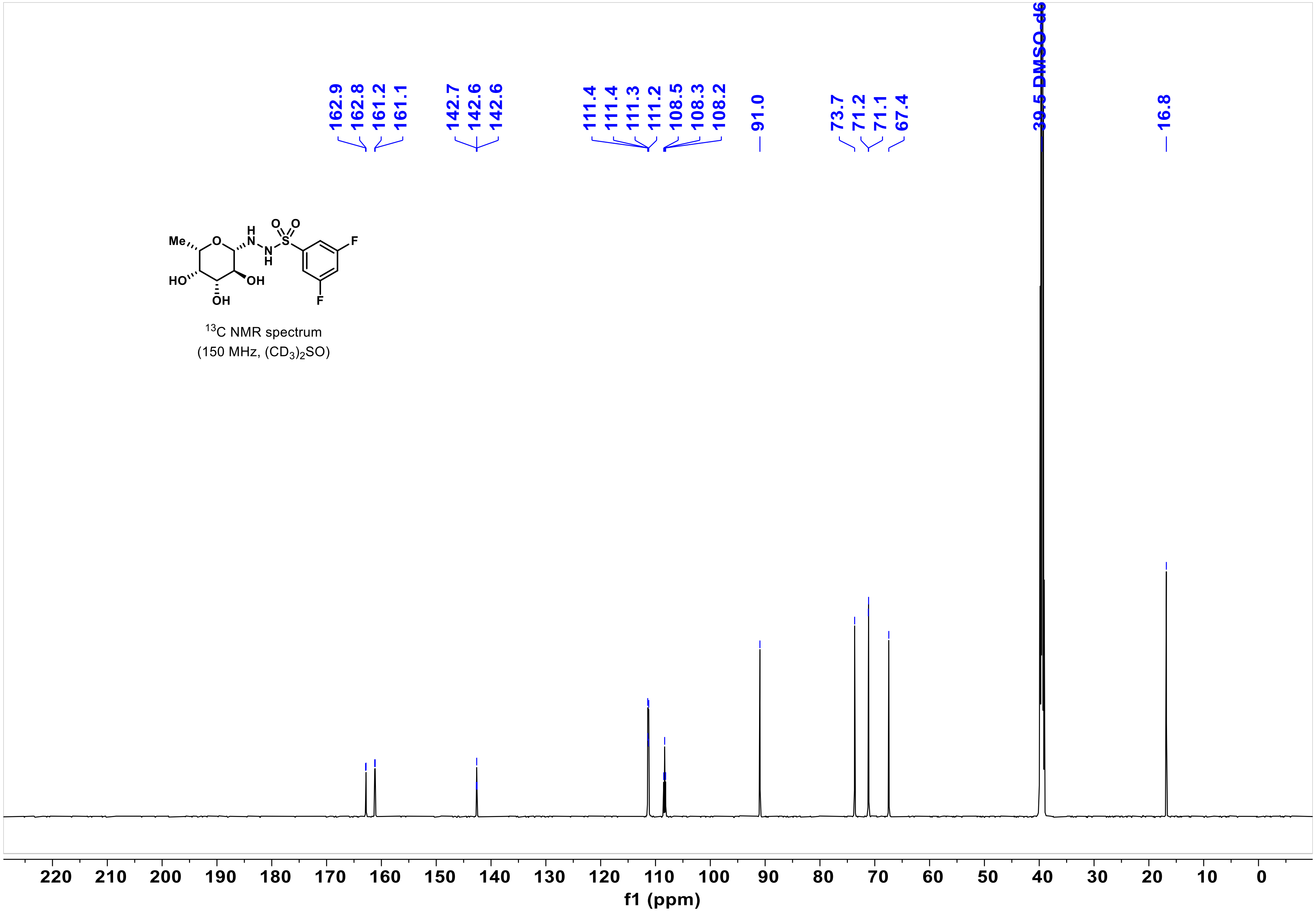

^{13}C NMR of compound **β-SH15**

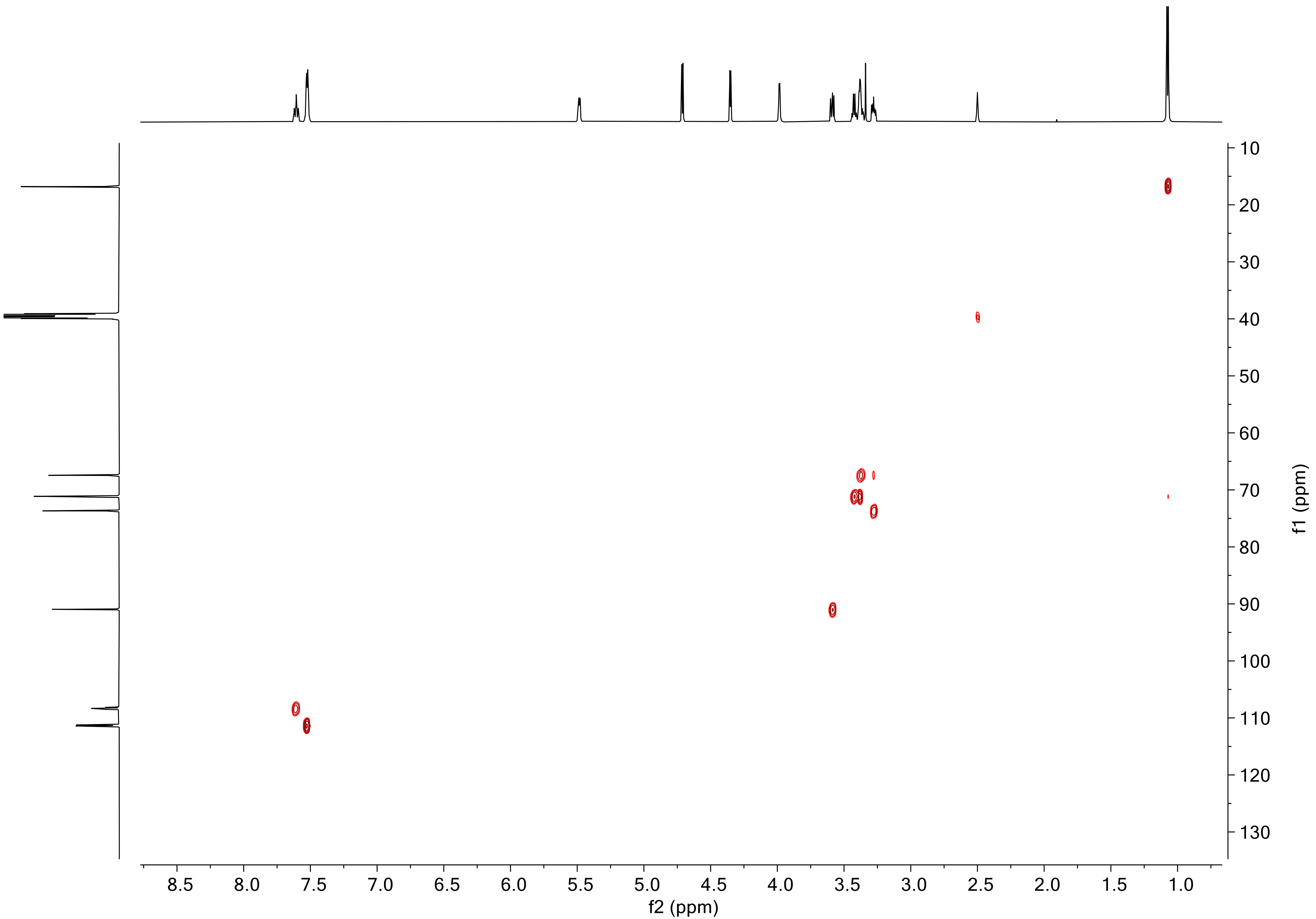

H-C HSQC NMR Spectrum of compound **β-SH15**

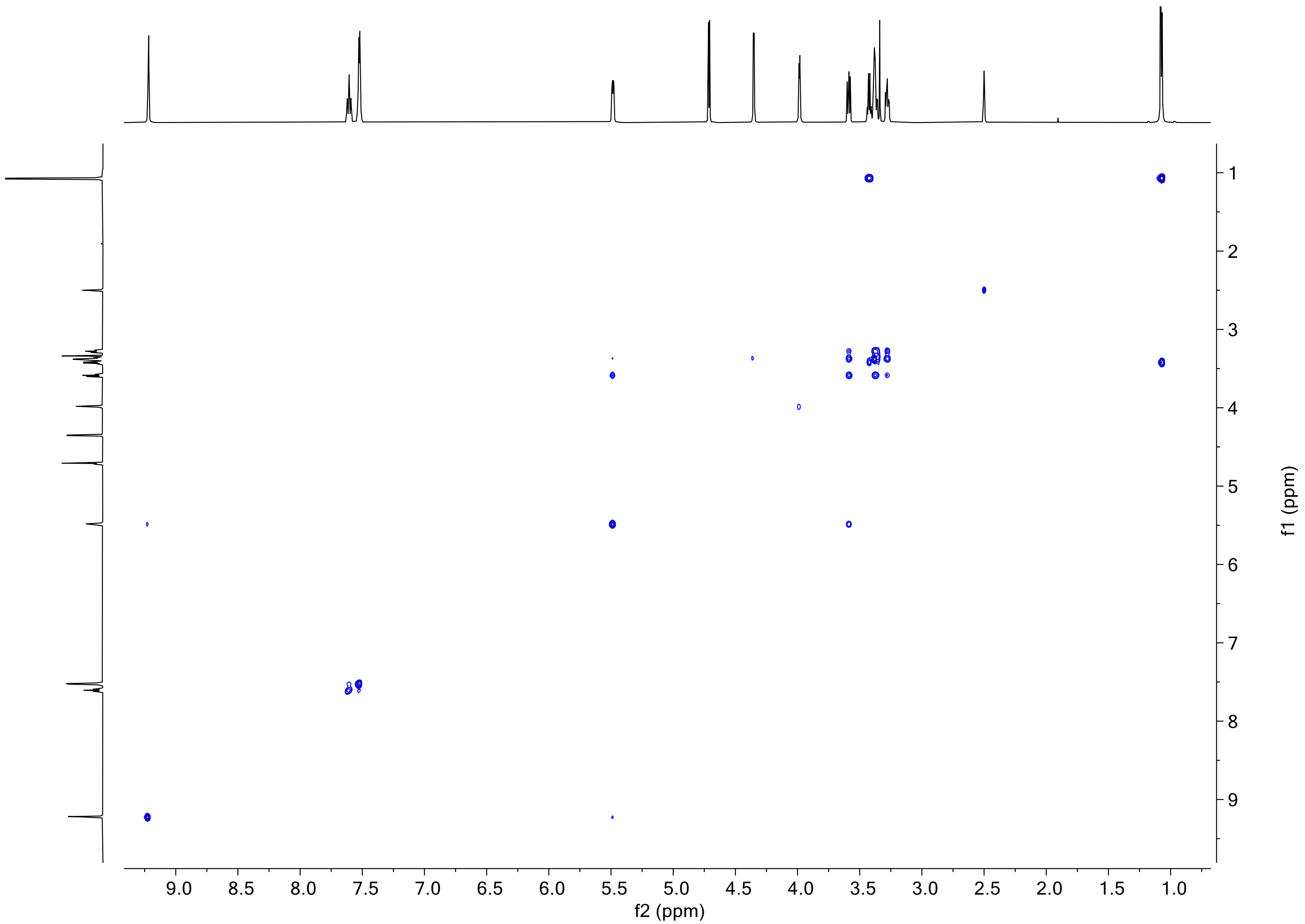

H-H COSY NMR Spectrum of compound **β-SH15**

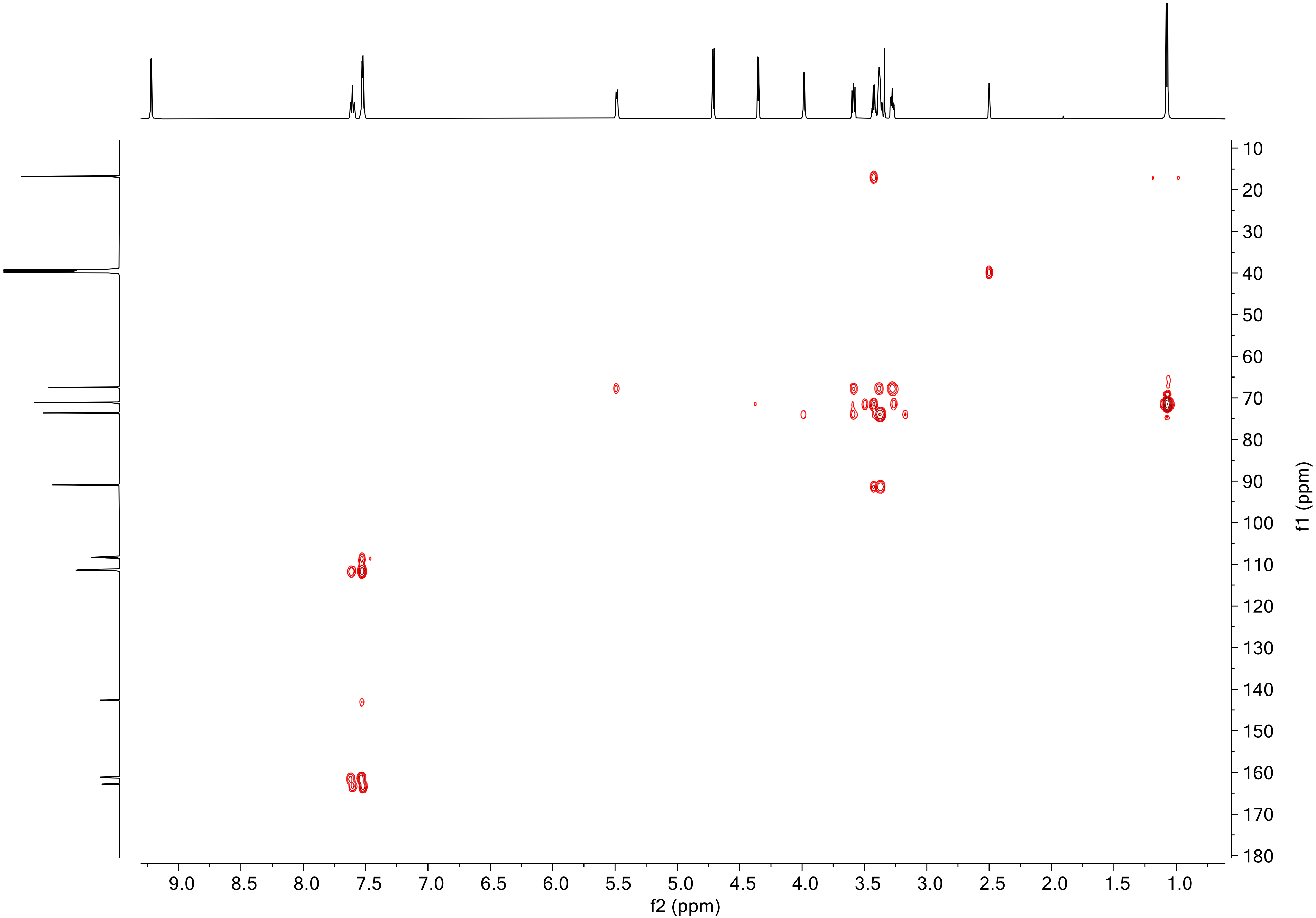

H-C HMBC NMR Spectrum of compound **β-SH15**

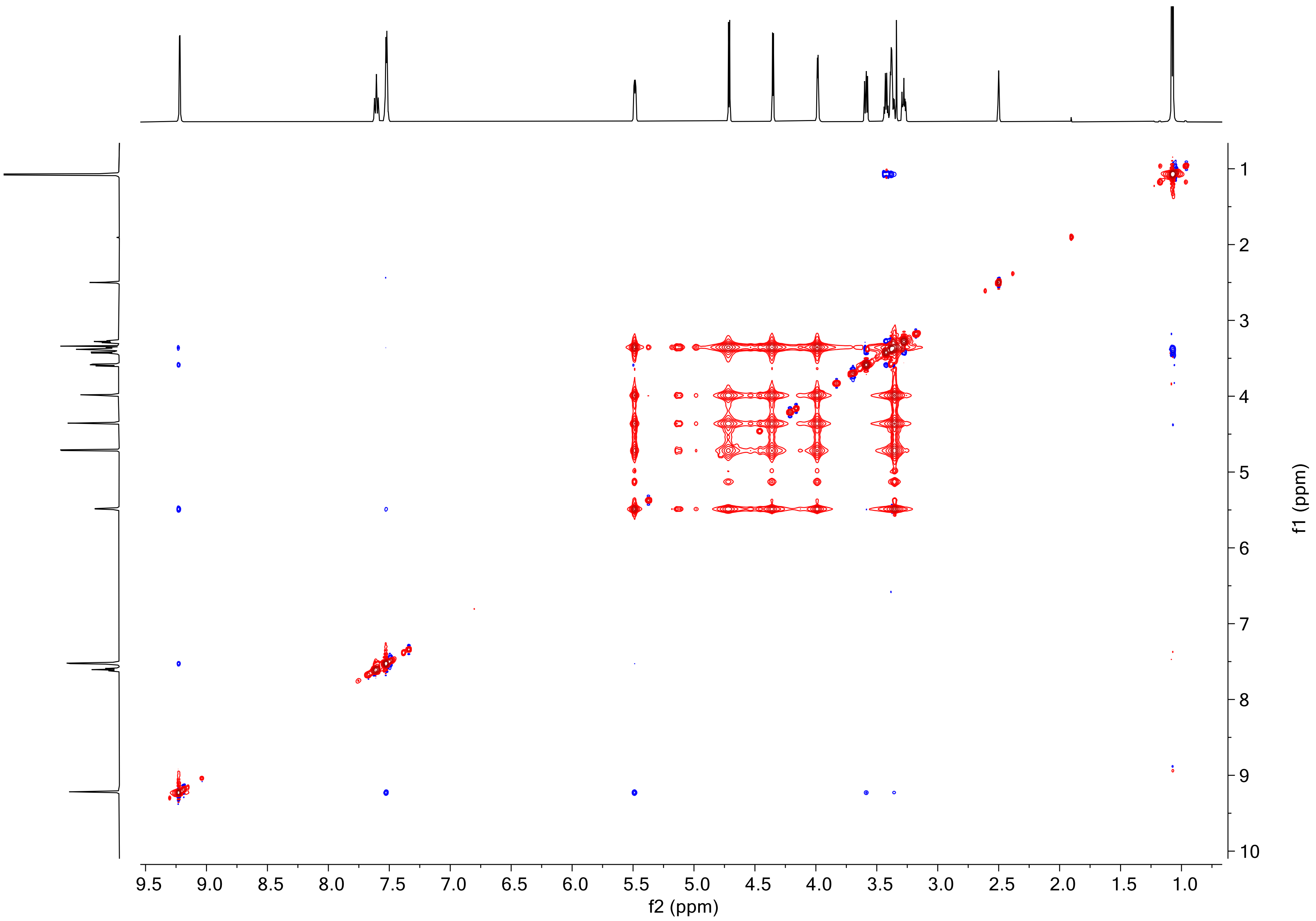

H-H NOESY NMR Spectrum of compound **β-SH15**

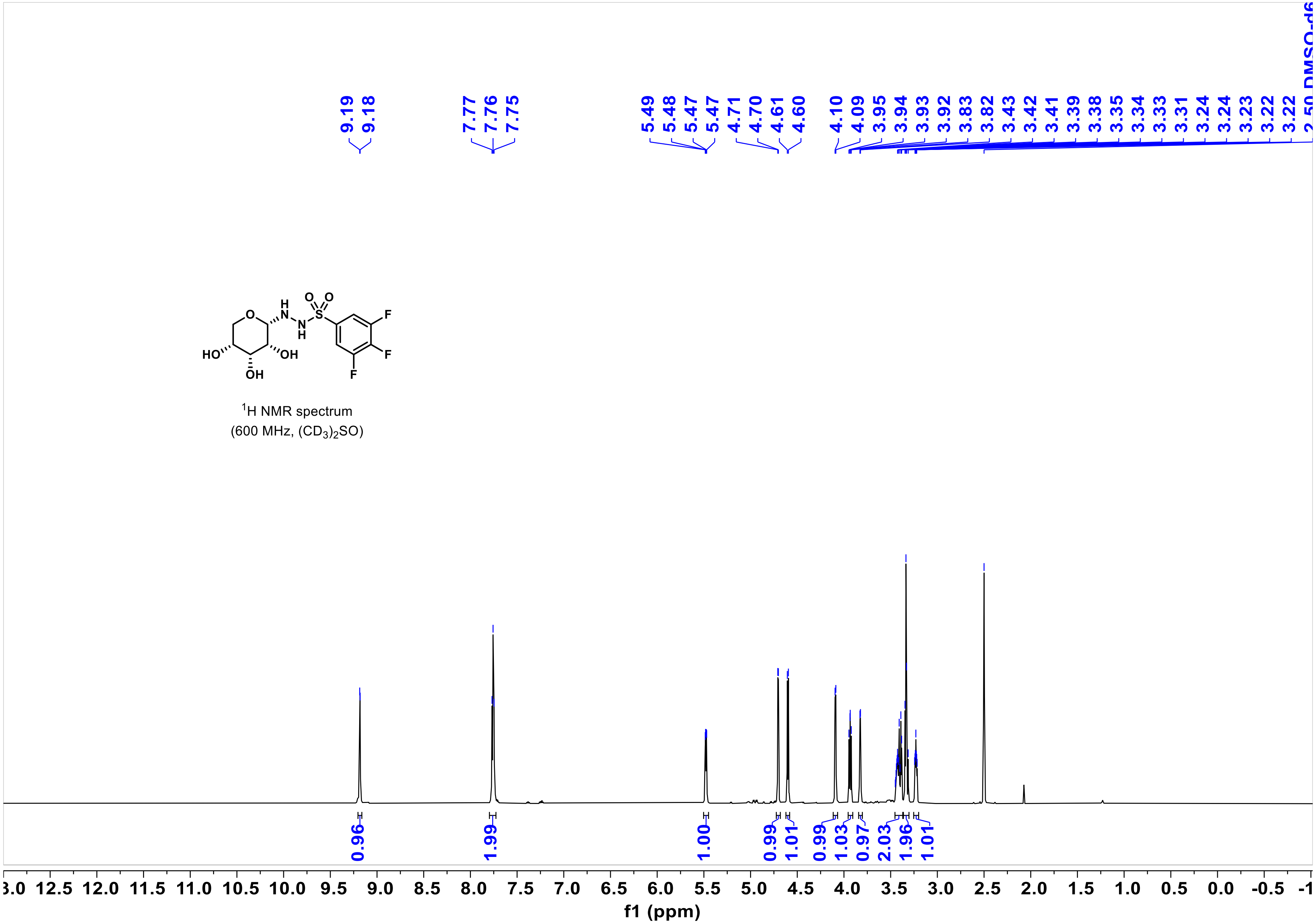

^{1}H NMR of compound **α-SH16**

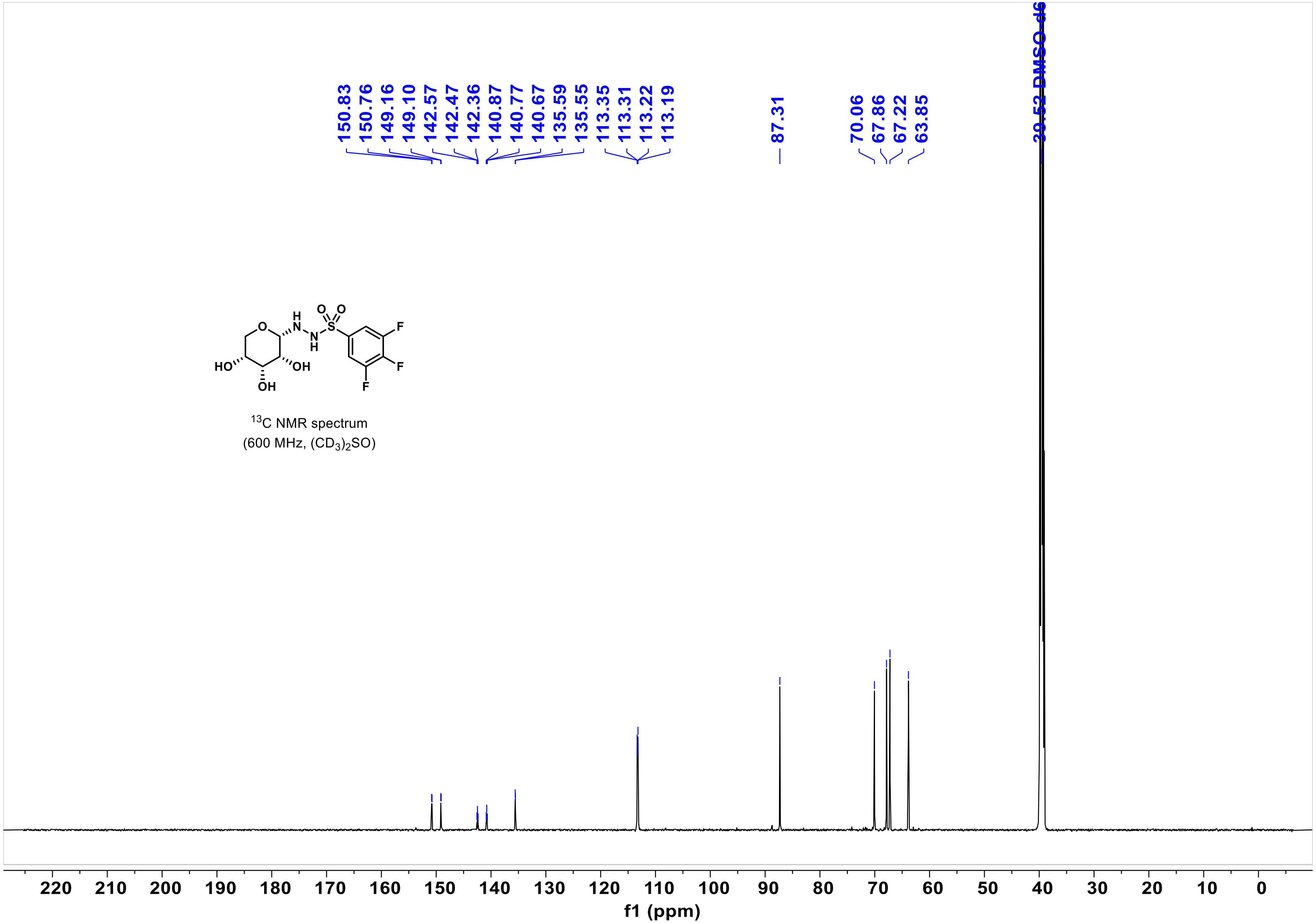

^{13}C NMR of compound **α-SH16**

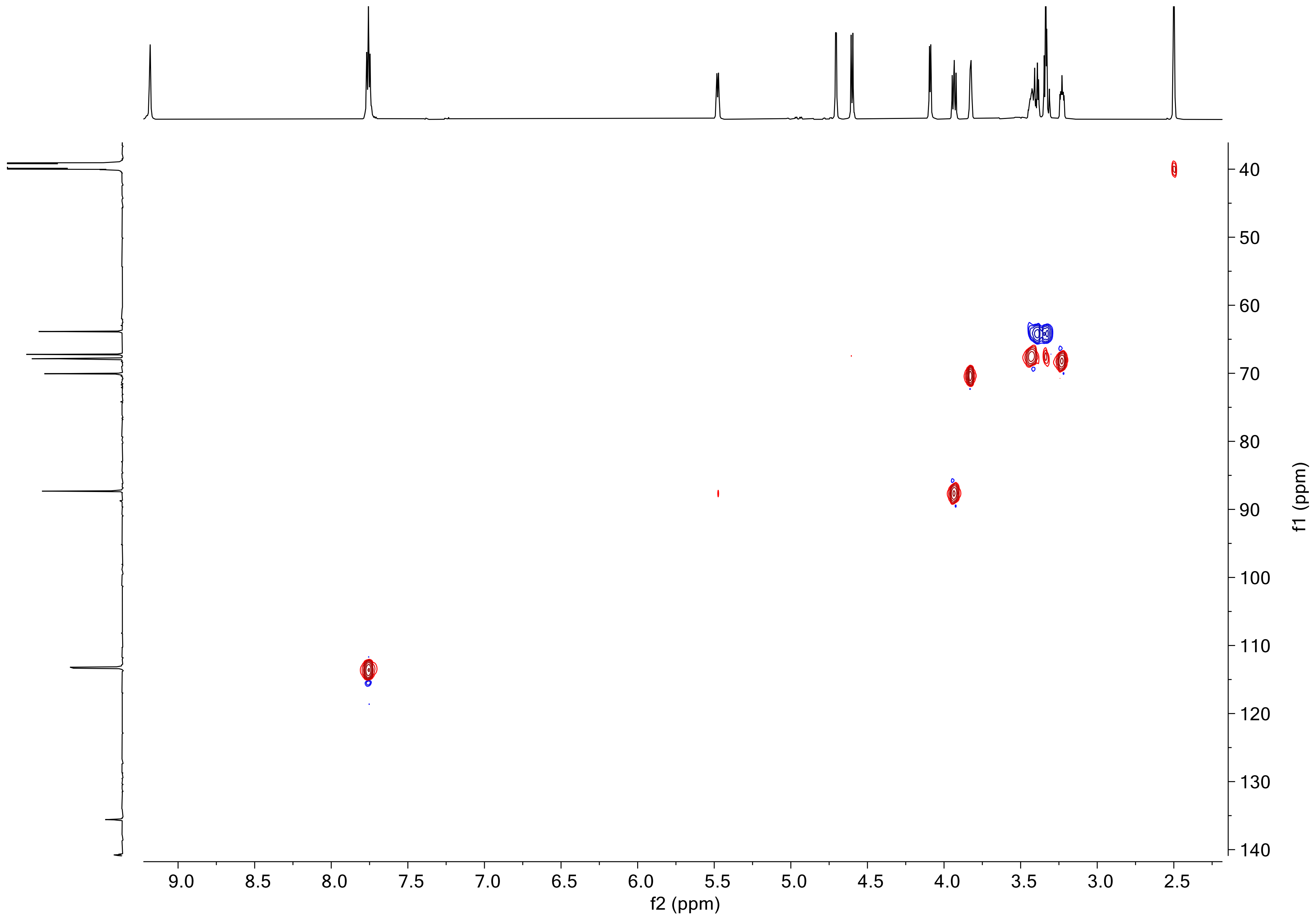

H-C HSQC NMR Spectrum of compound **α -SH16**

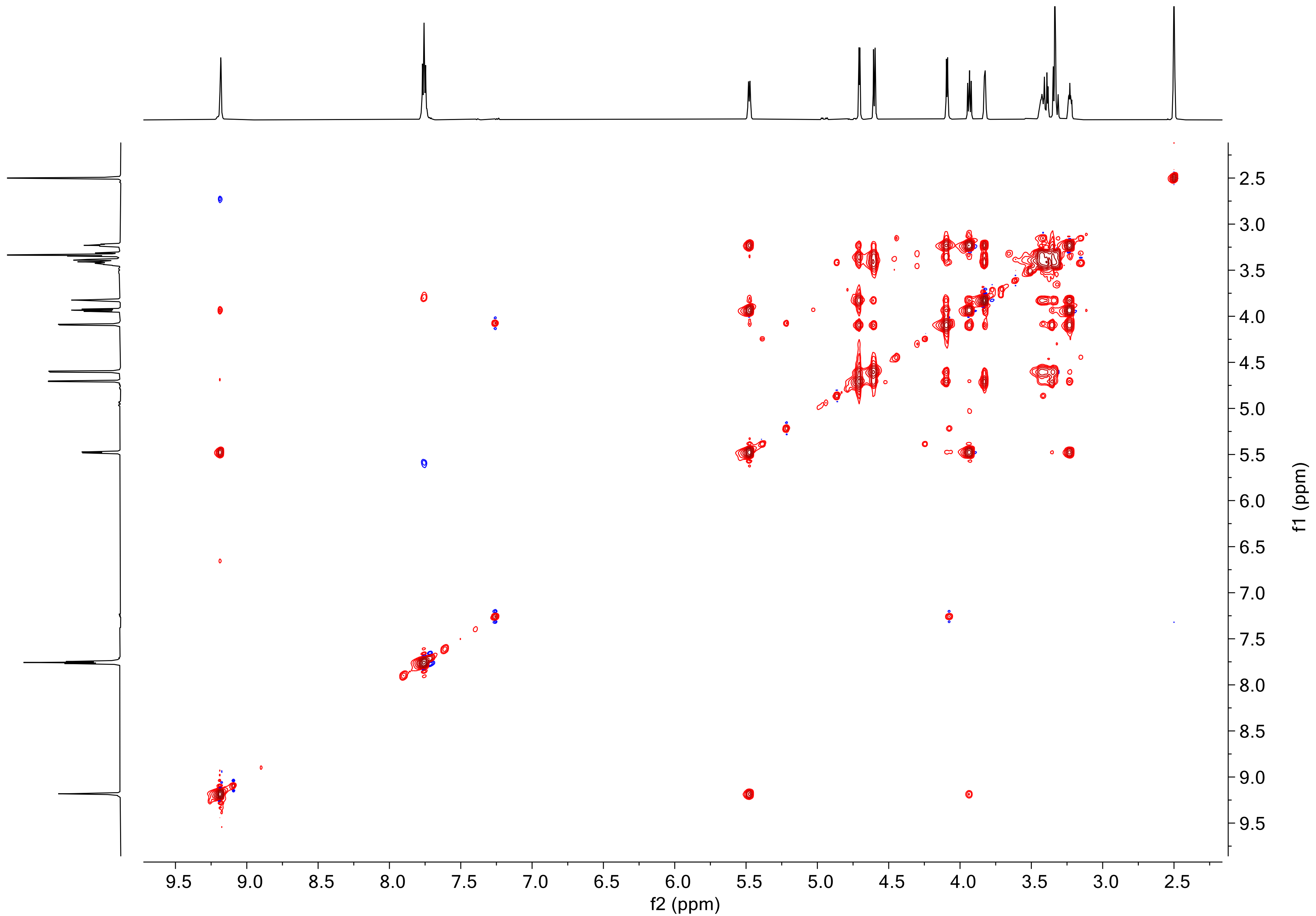

H-H COSY NMR Spectrum of compound **α-SH16**

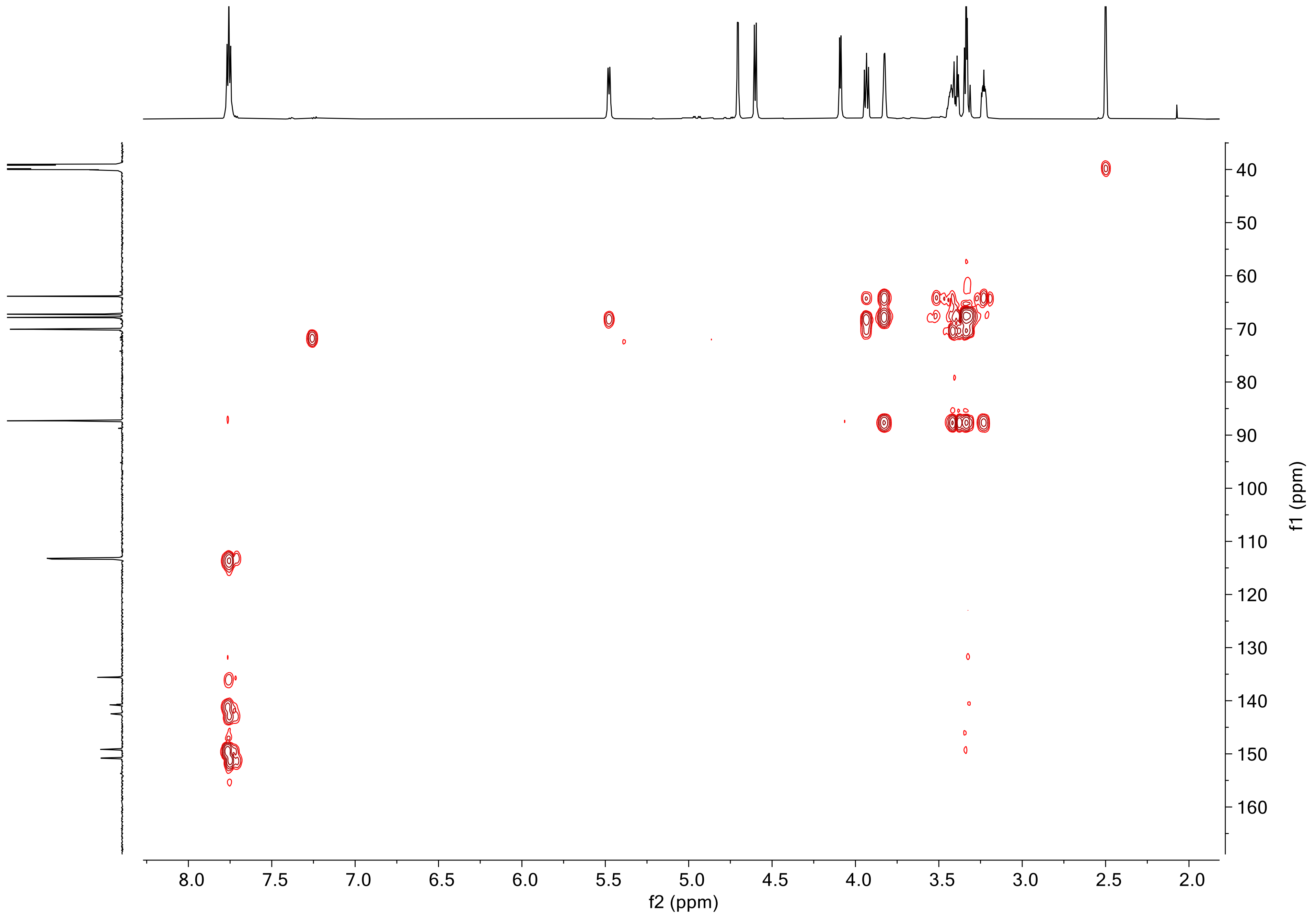

H-C HMBC NMR Spectrum of compound **α-SH16**

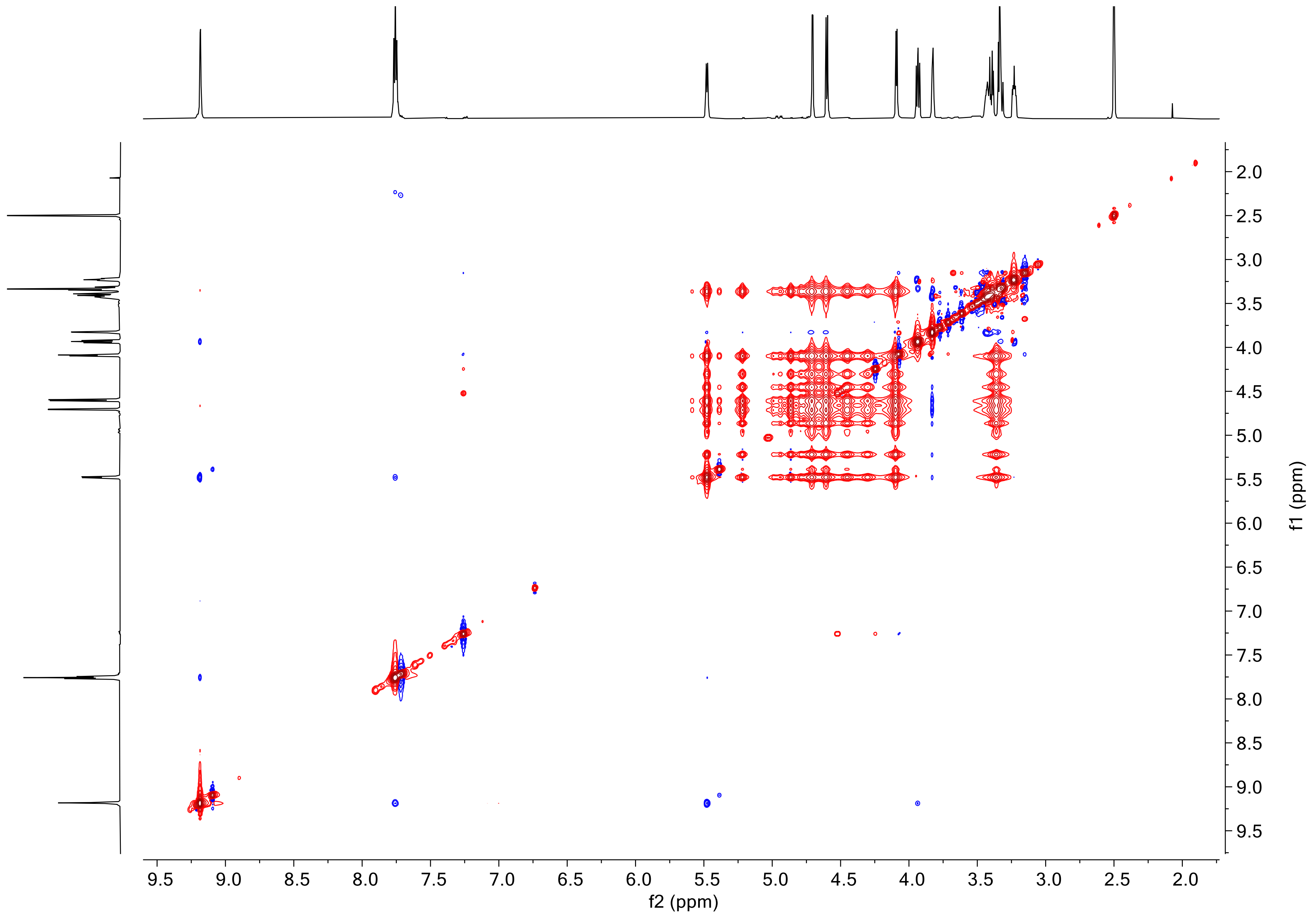

H-H NOESY NMR Spectrum of compound **α-SH16**

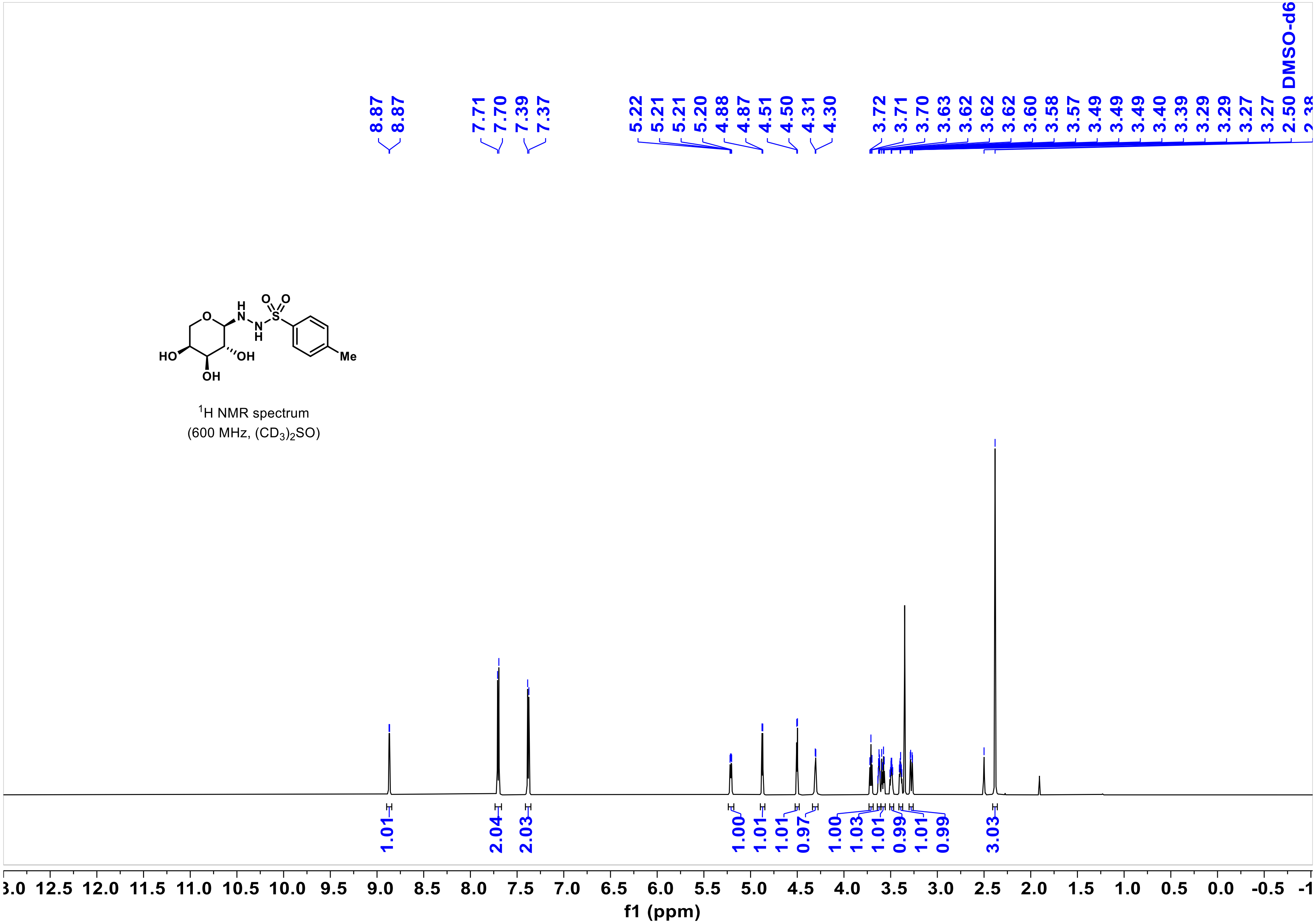

^{1}H NMR of compound **α-SH17**

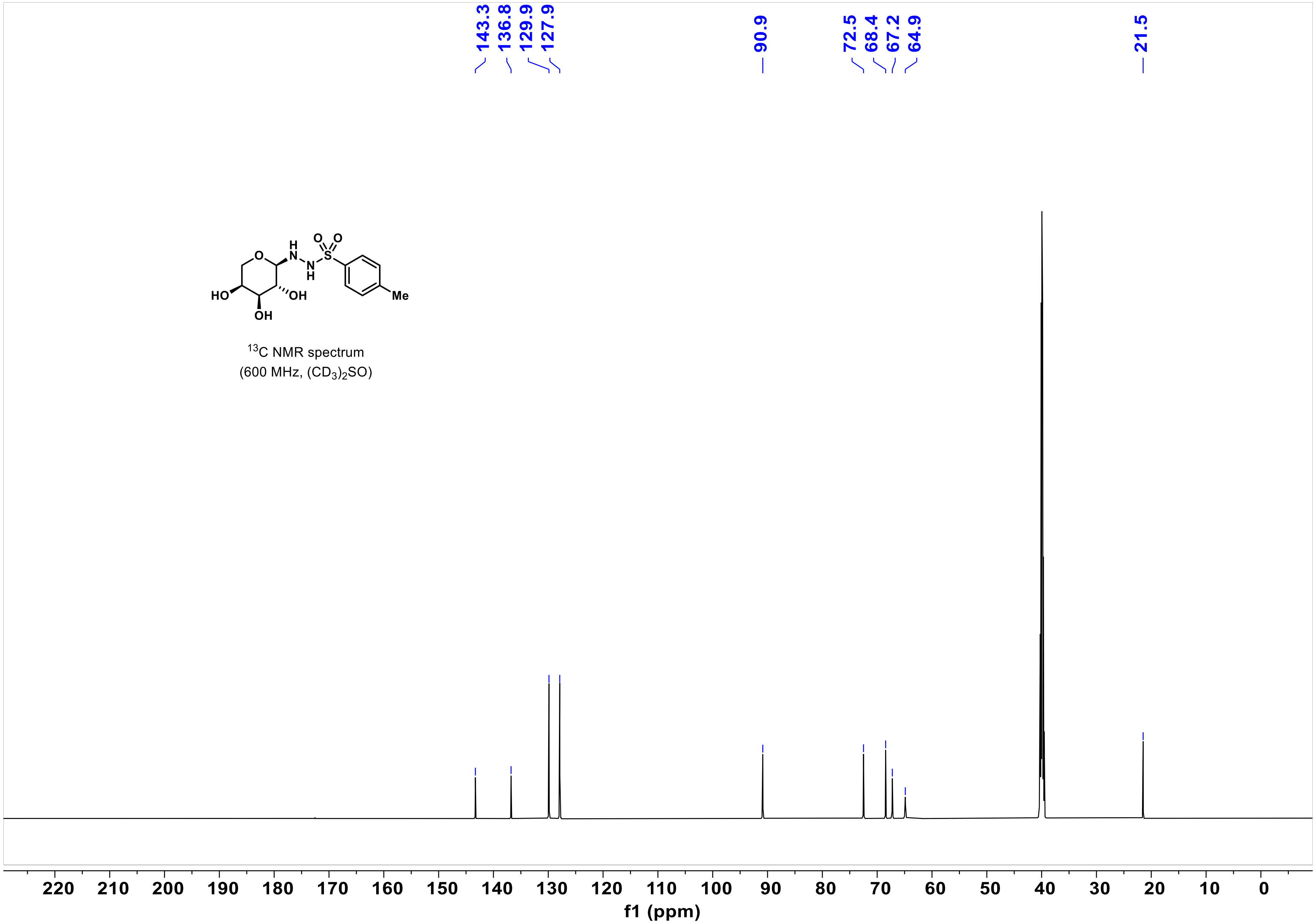

^{13}C NMR of compound **α-SH17**

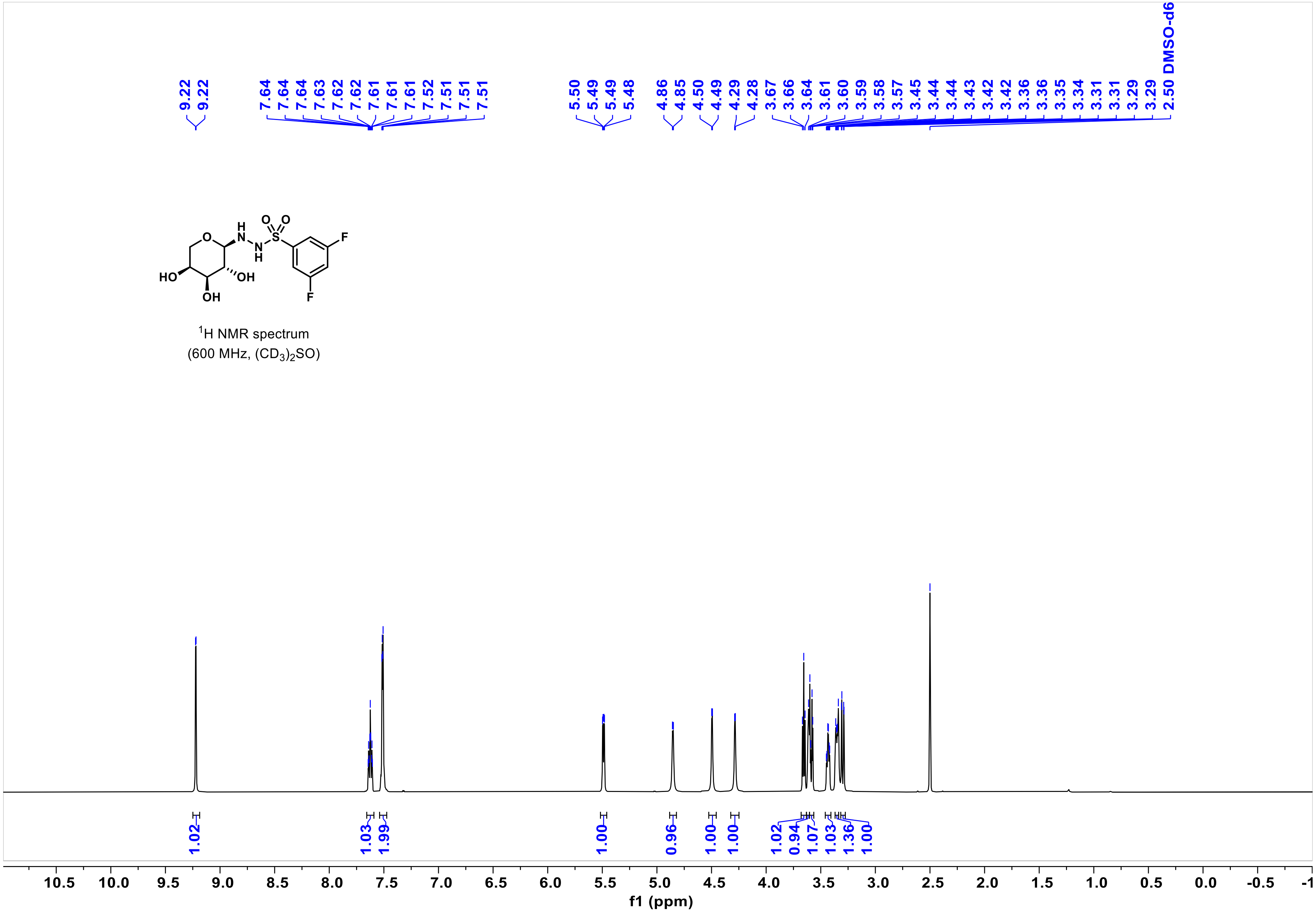

^{1}H NMR of compound **α-SH18**

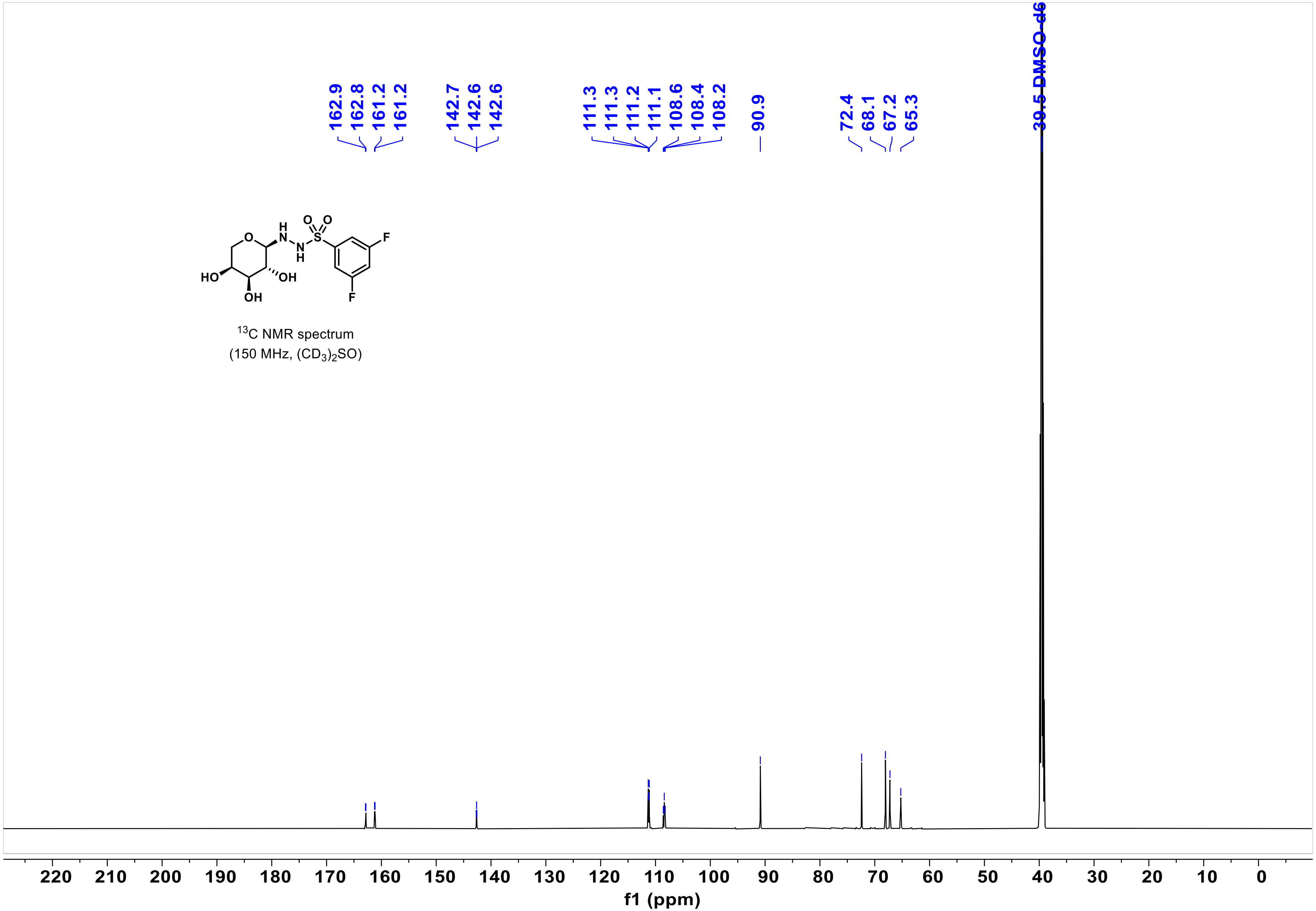

^{13}C NMR of compound **α-SH18**

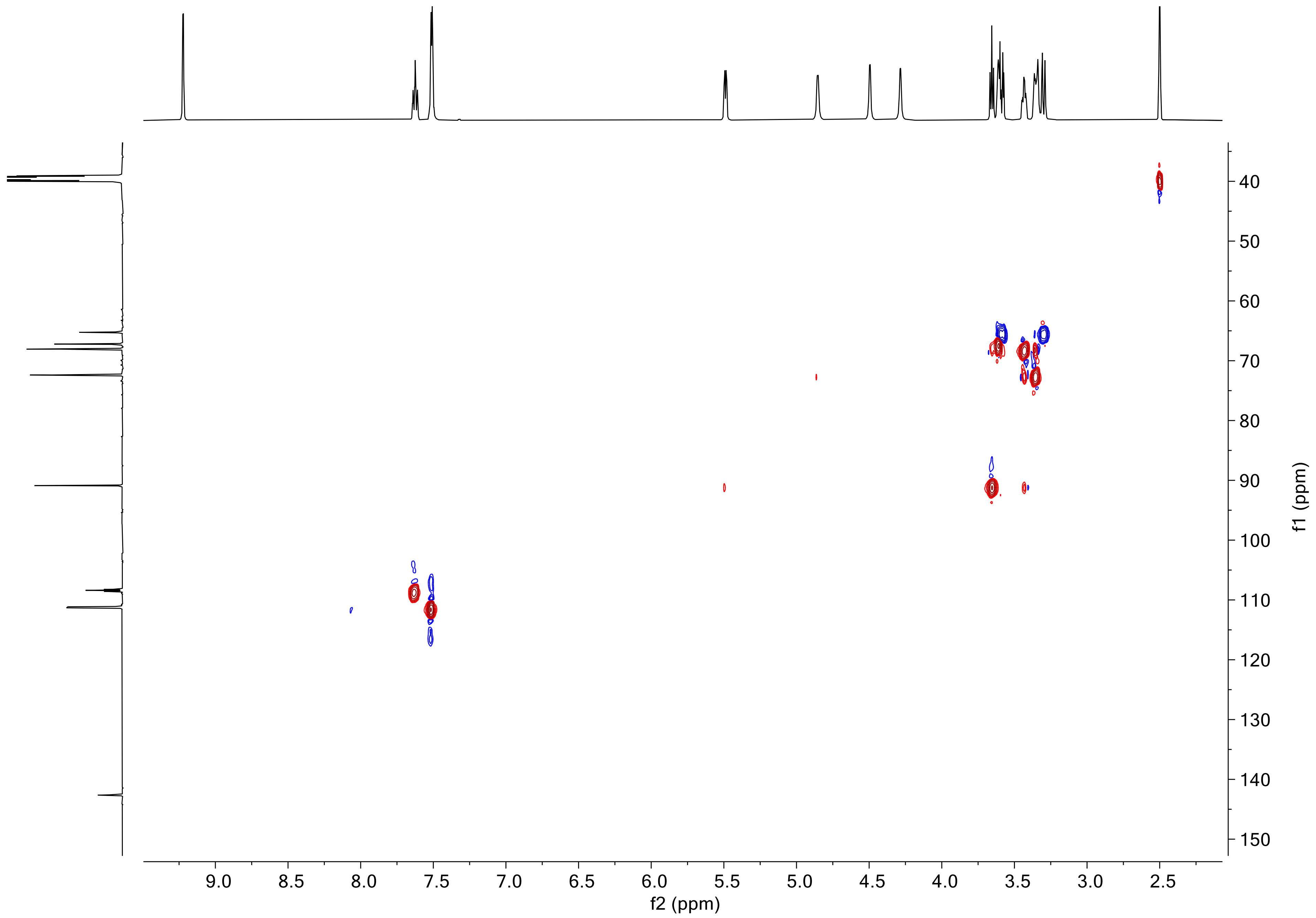

H-C HSQC NMR Spectrum of compound **α -SH18**

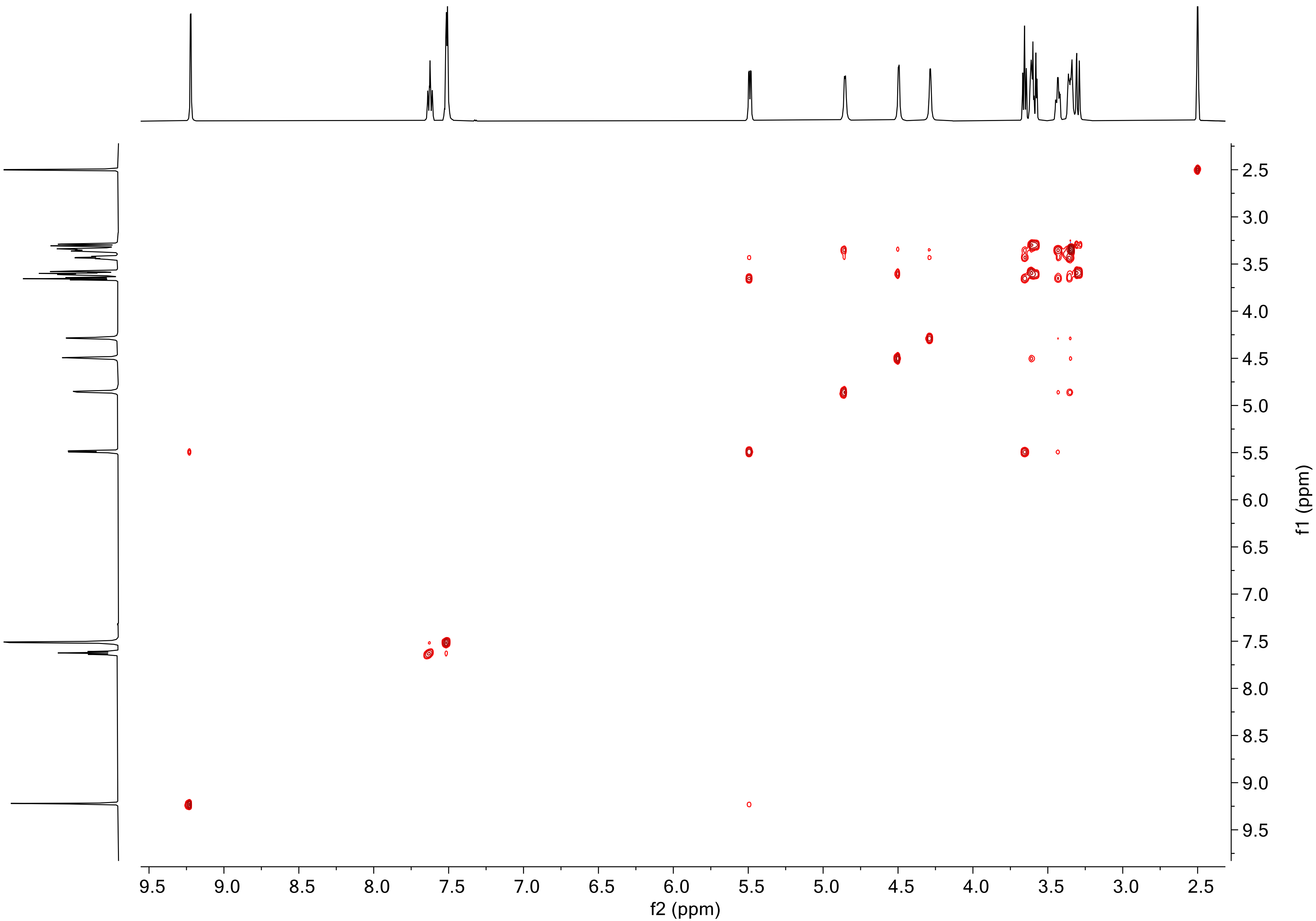

H-H COSY NMR Spectrum of compound **α-SH18**

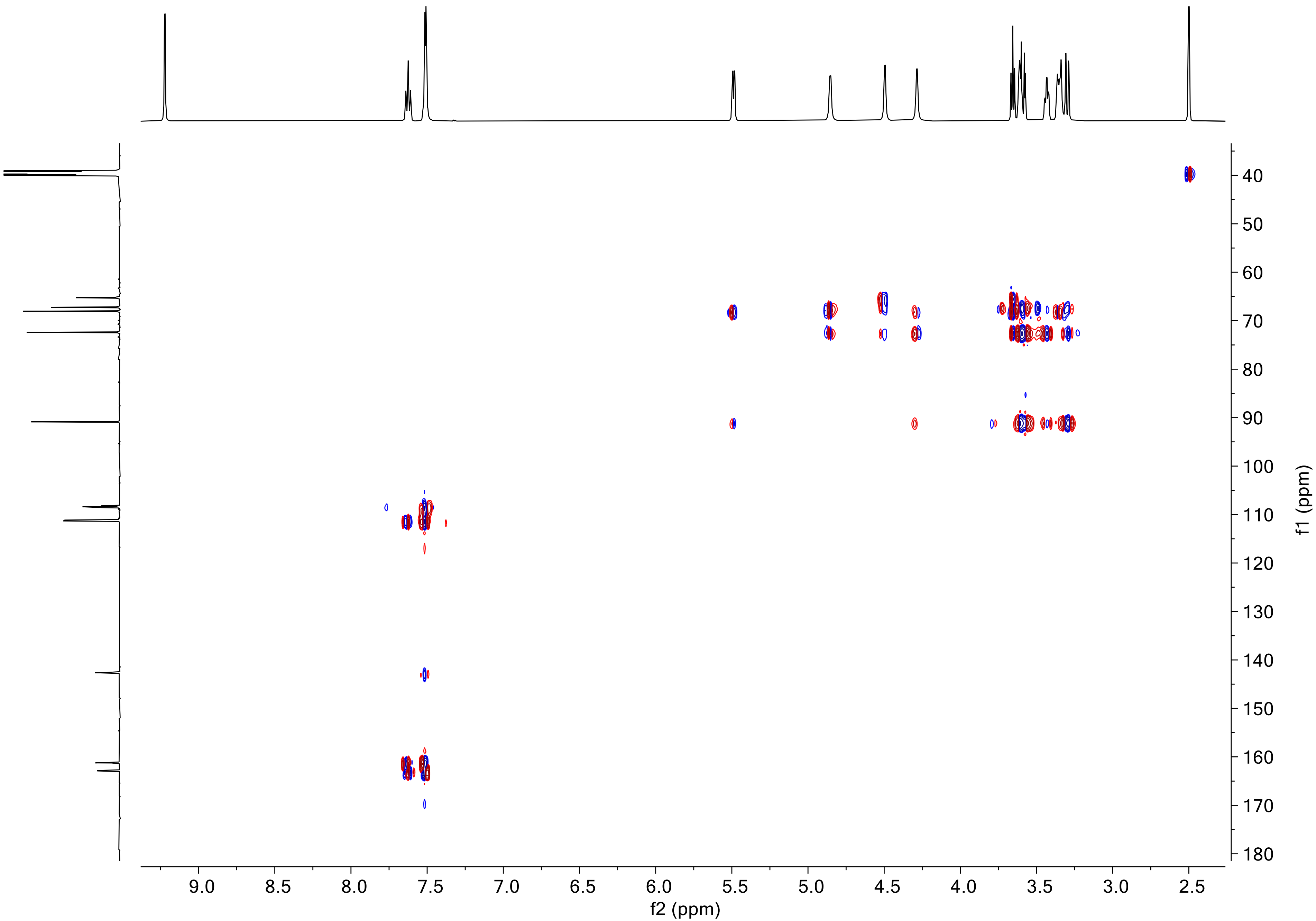

H-C HMBC NMR Spectrum of compound **α-SH18**

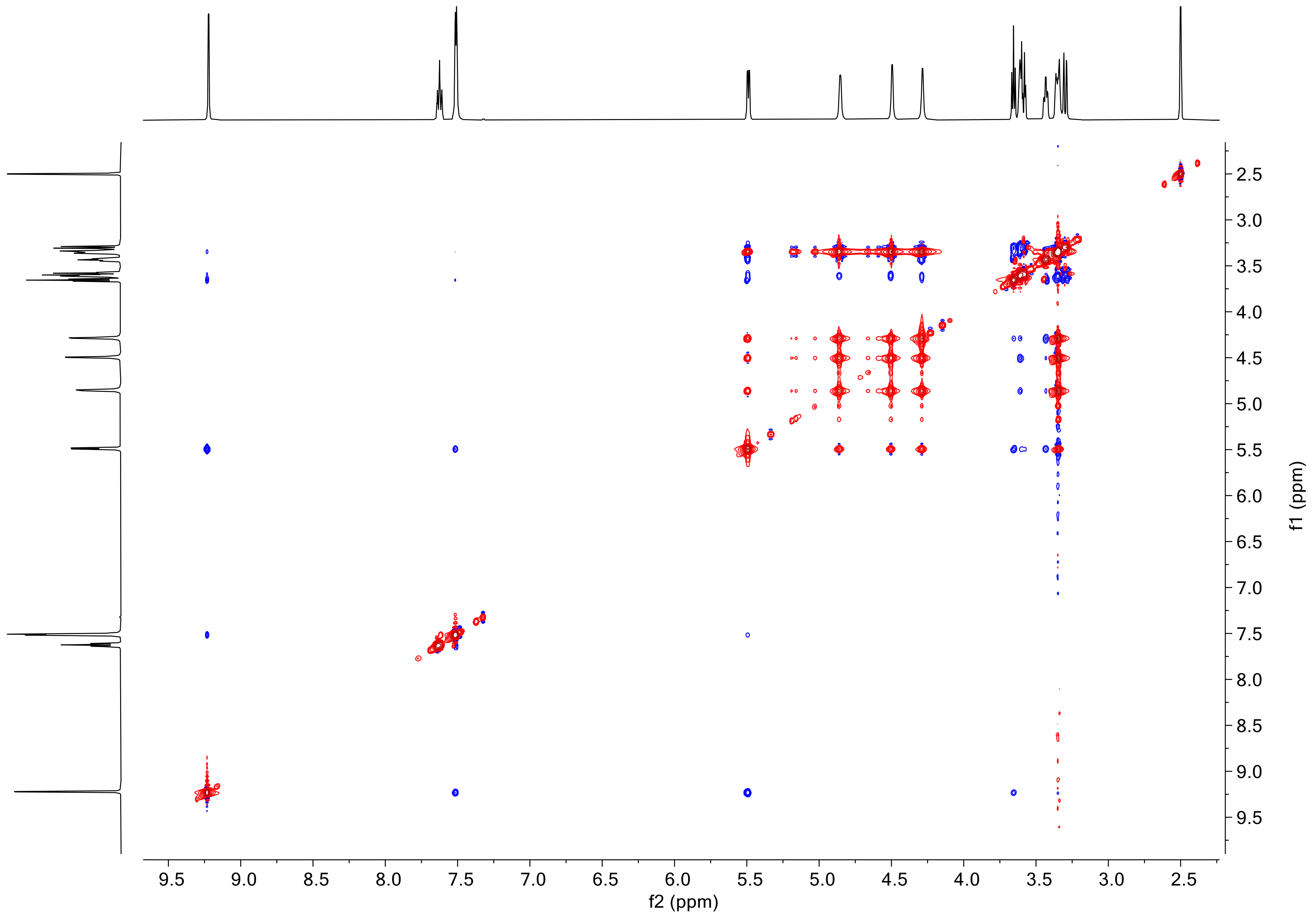

H-H NOESY NMR Spectrum of compound **α-SH18**

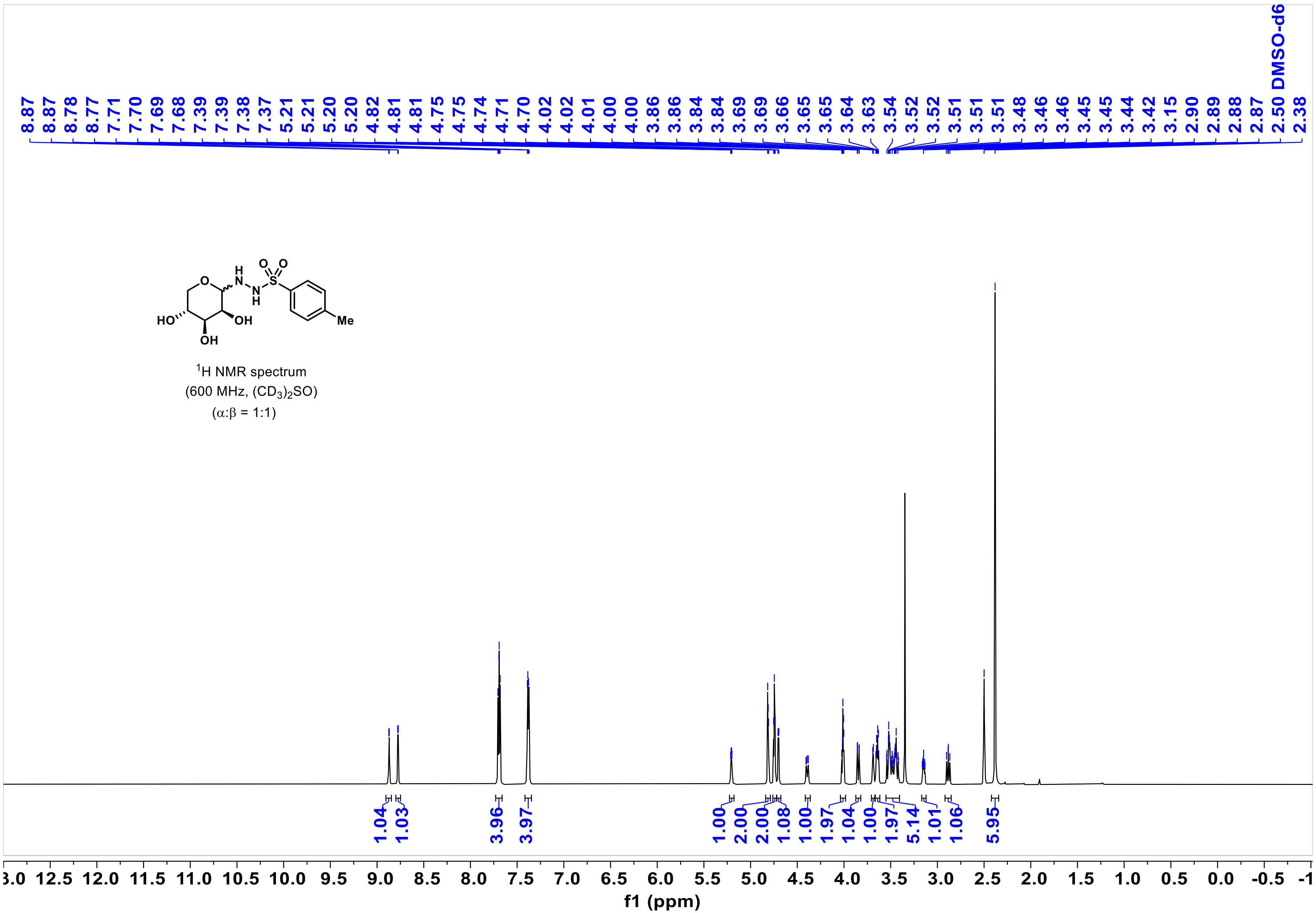

^{1}H NMR of compound **SH19** (dr = 1:1)

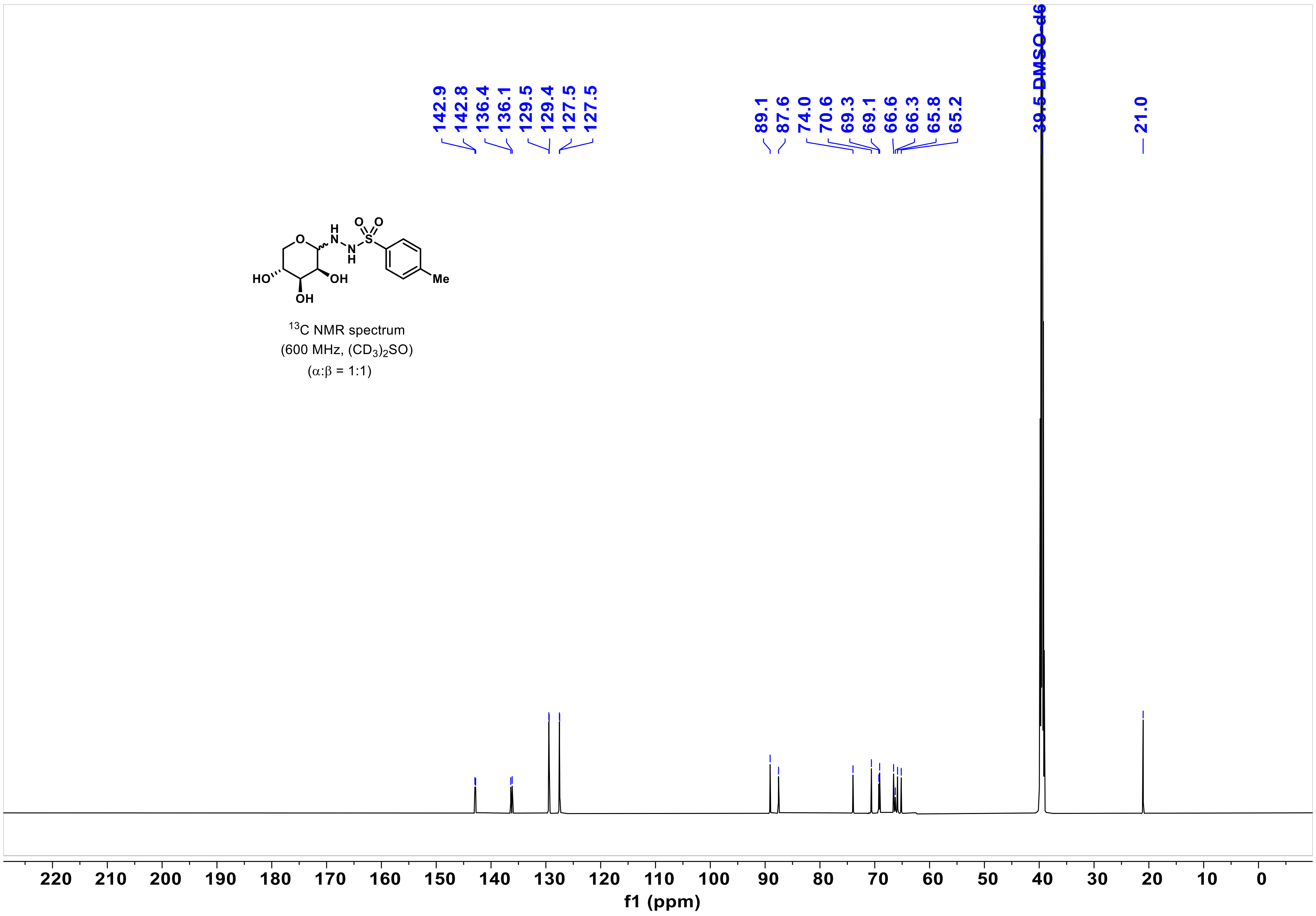

^{13}C NMR of compound **SH19** (dr = 1:1)

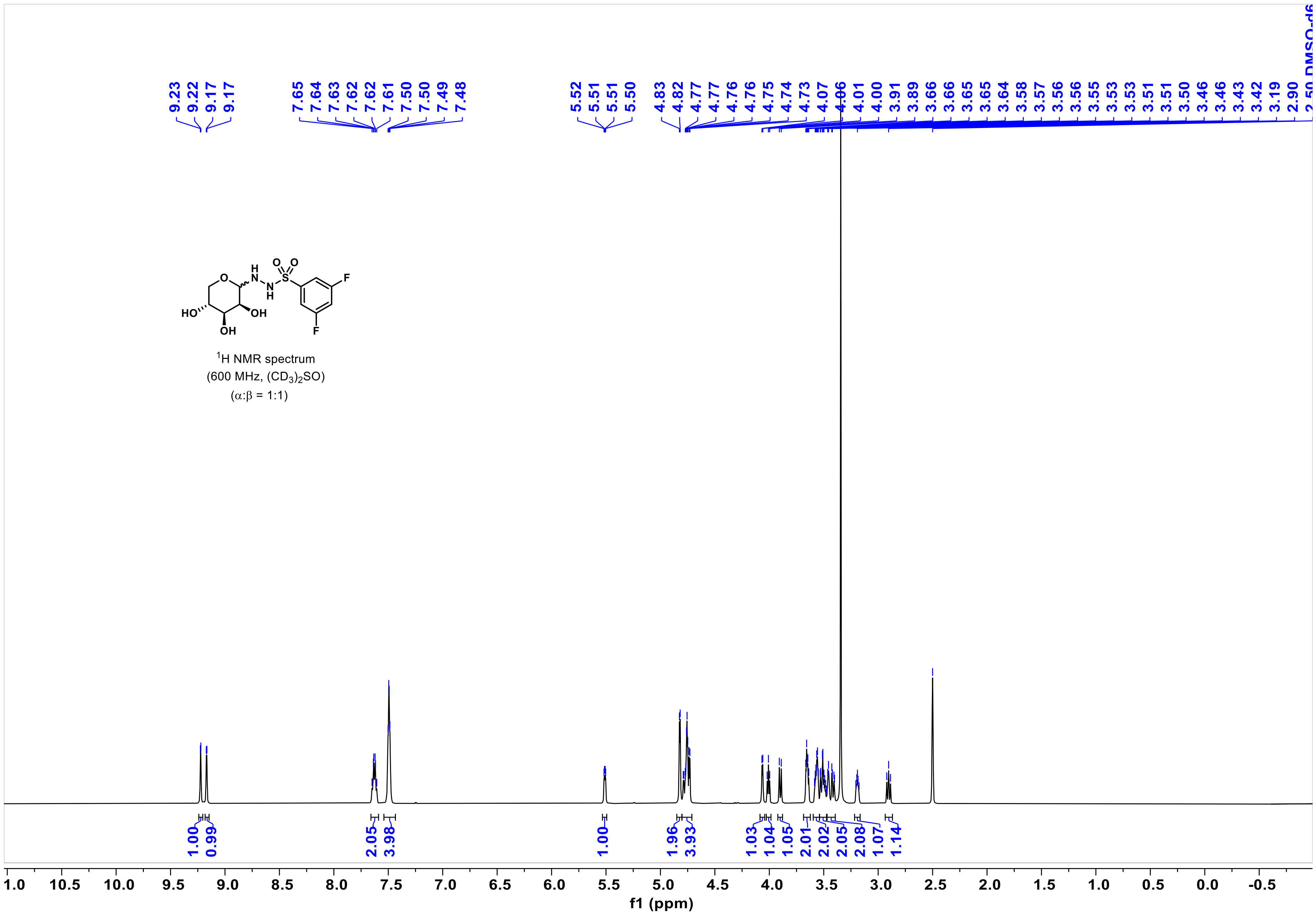

^{1}H NMR of compound **SH20** (dr = 1:1)

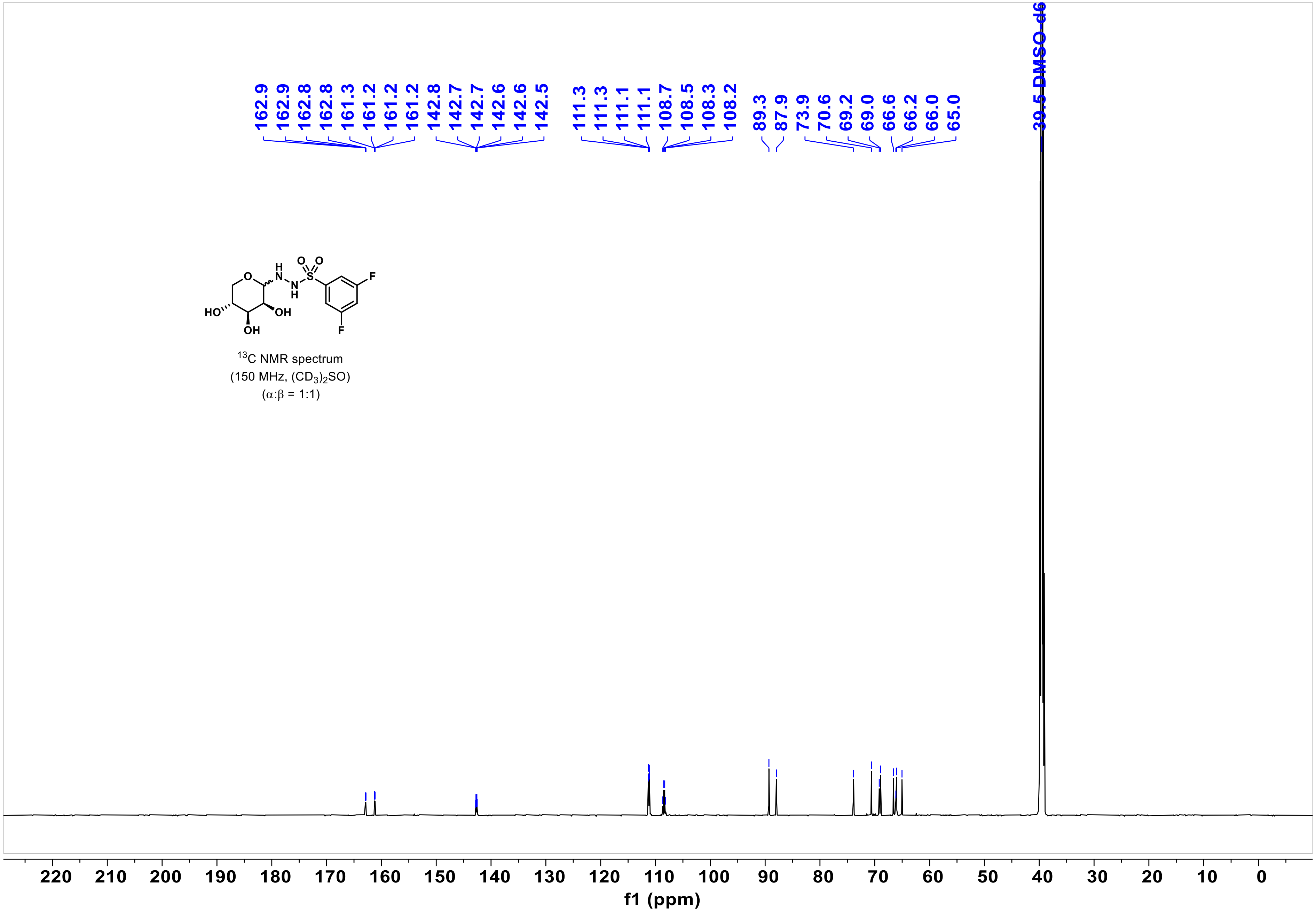

^{13}C NMR of compound **SH20** (dr = 1:1)

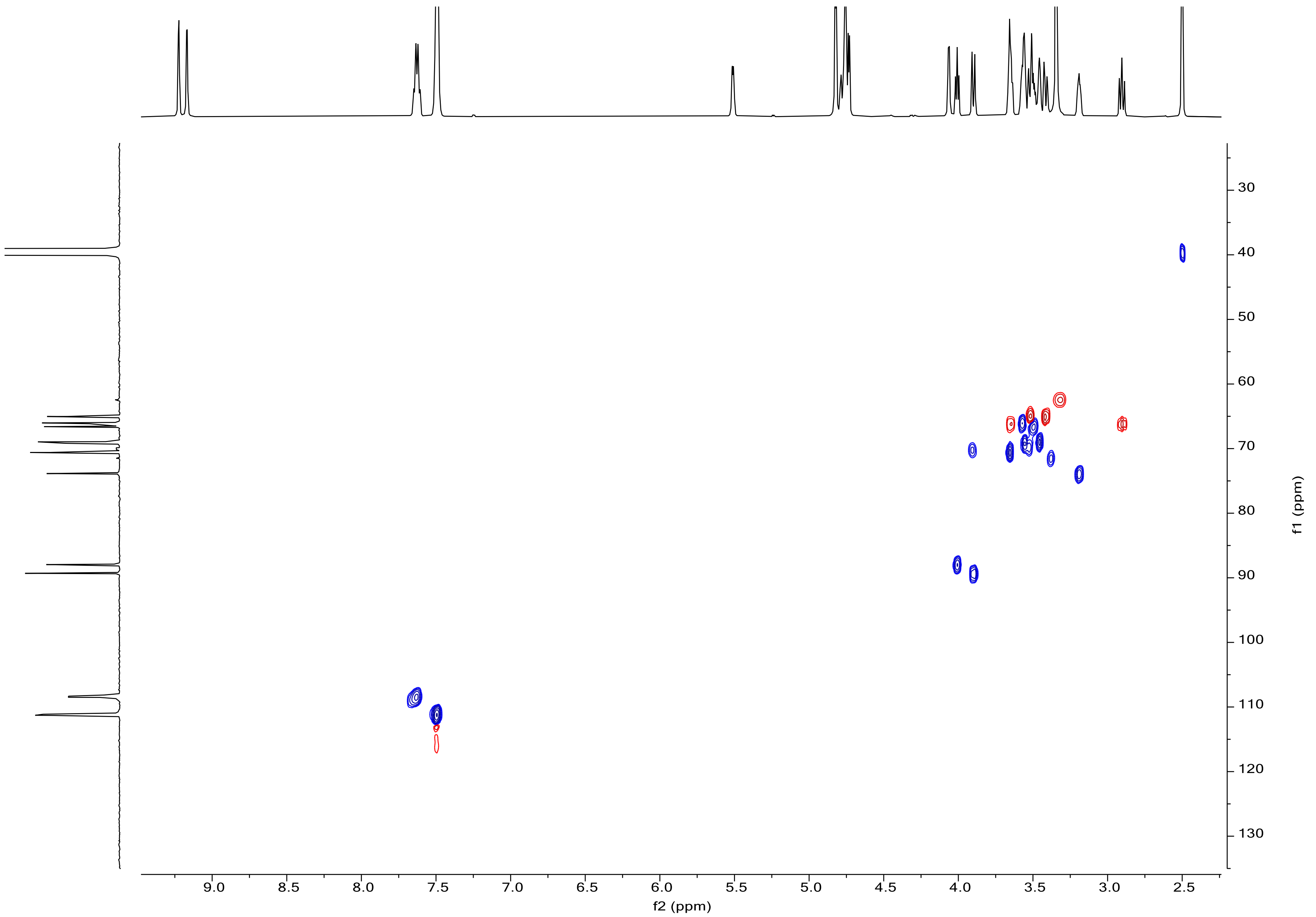

H-C HSQC NMR Spectrum of compound of **SH20** (dr = 1:1)

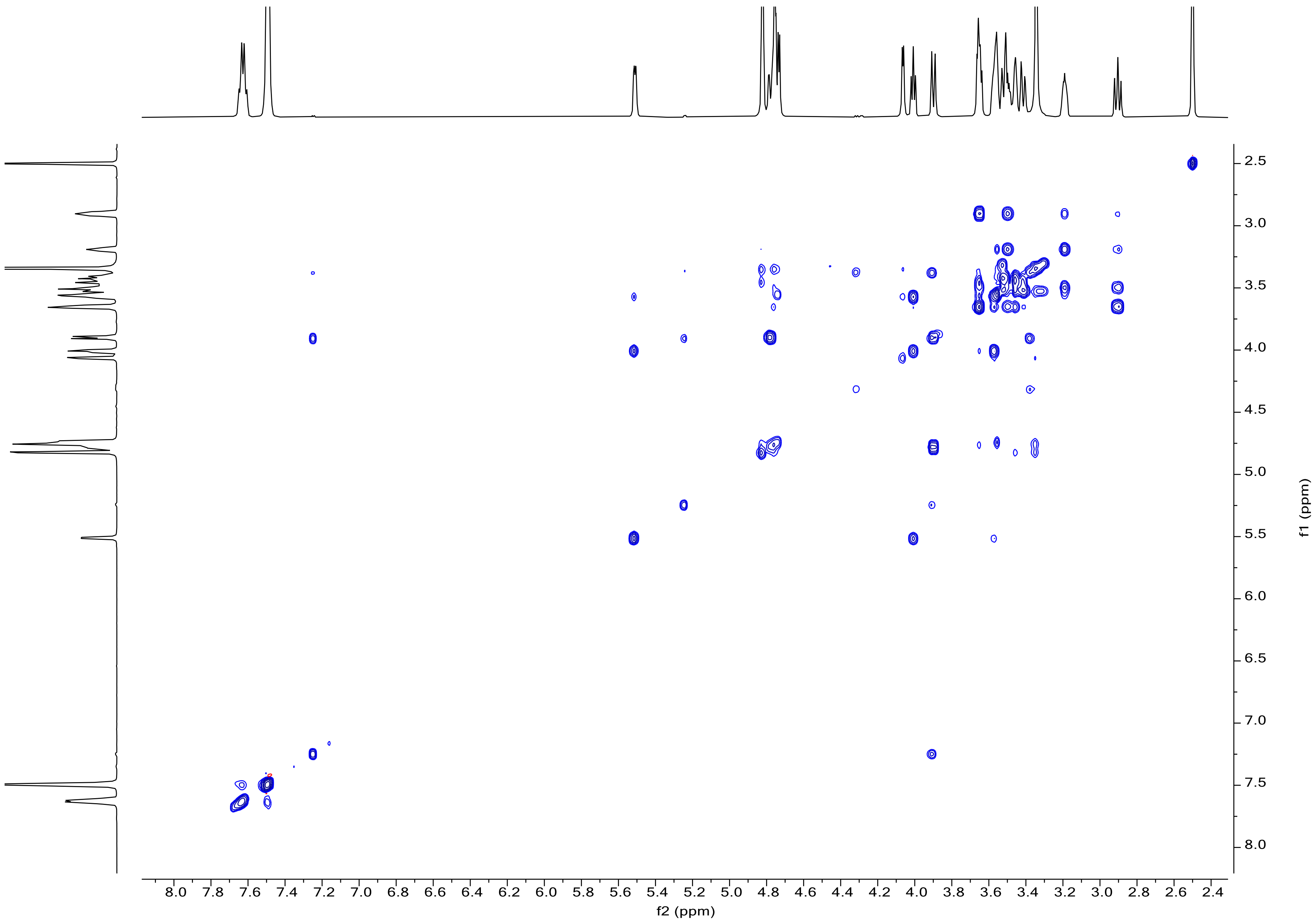

H-H COSY NMR Spectrum of compound of **SH20** (dr = 1:1)

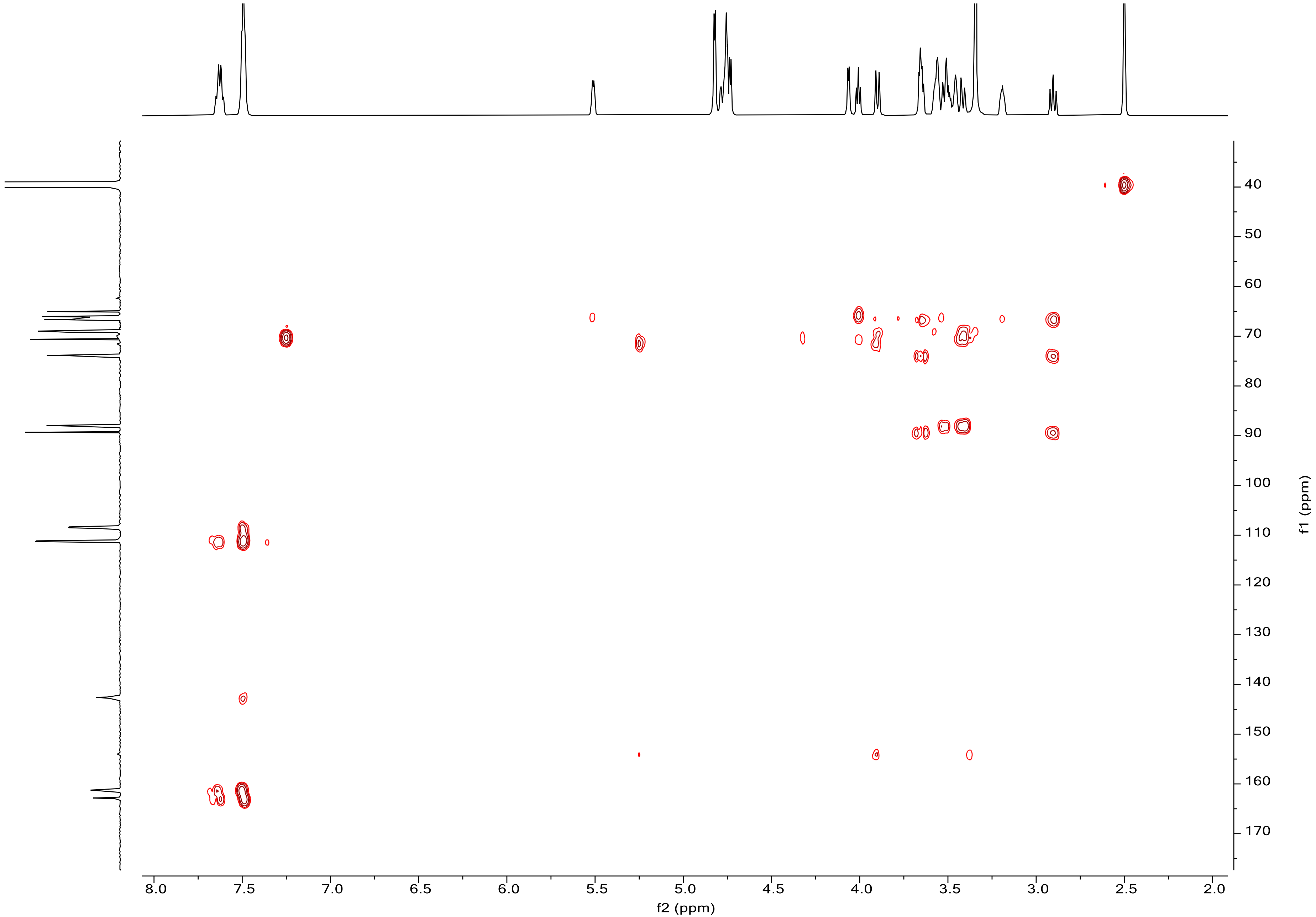

H-C HMBC NMR Spectrum of compound of **SH20** (dr = 1:1)

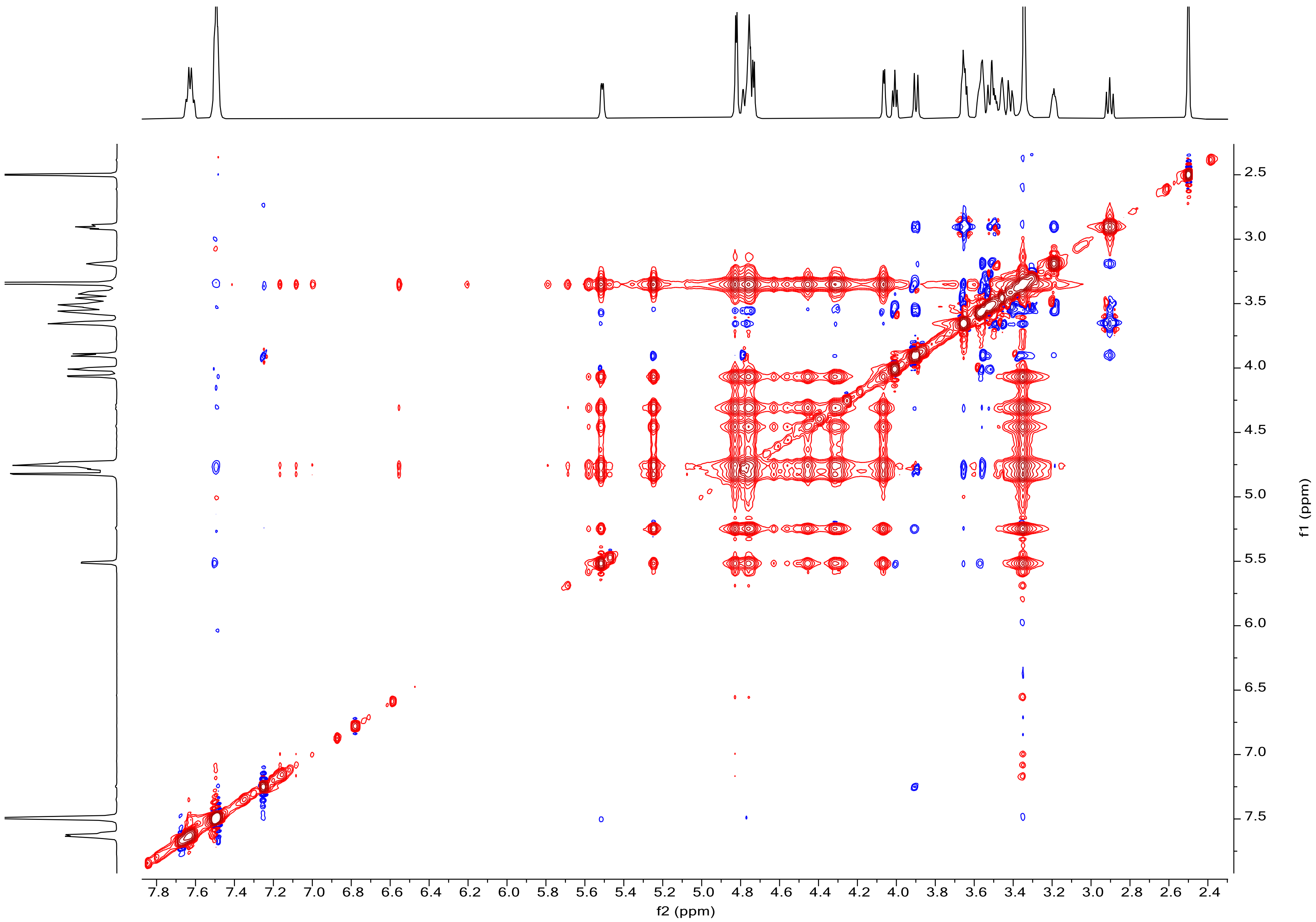

H-H NOESY NMR Spectrum of compound of **SH20** (dr = 1:1)

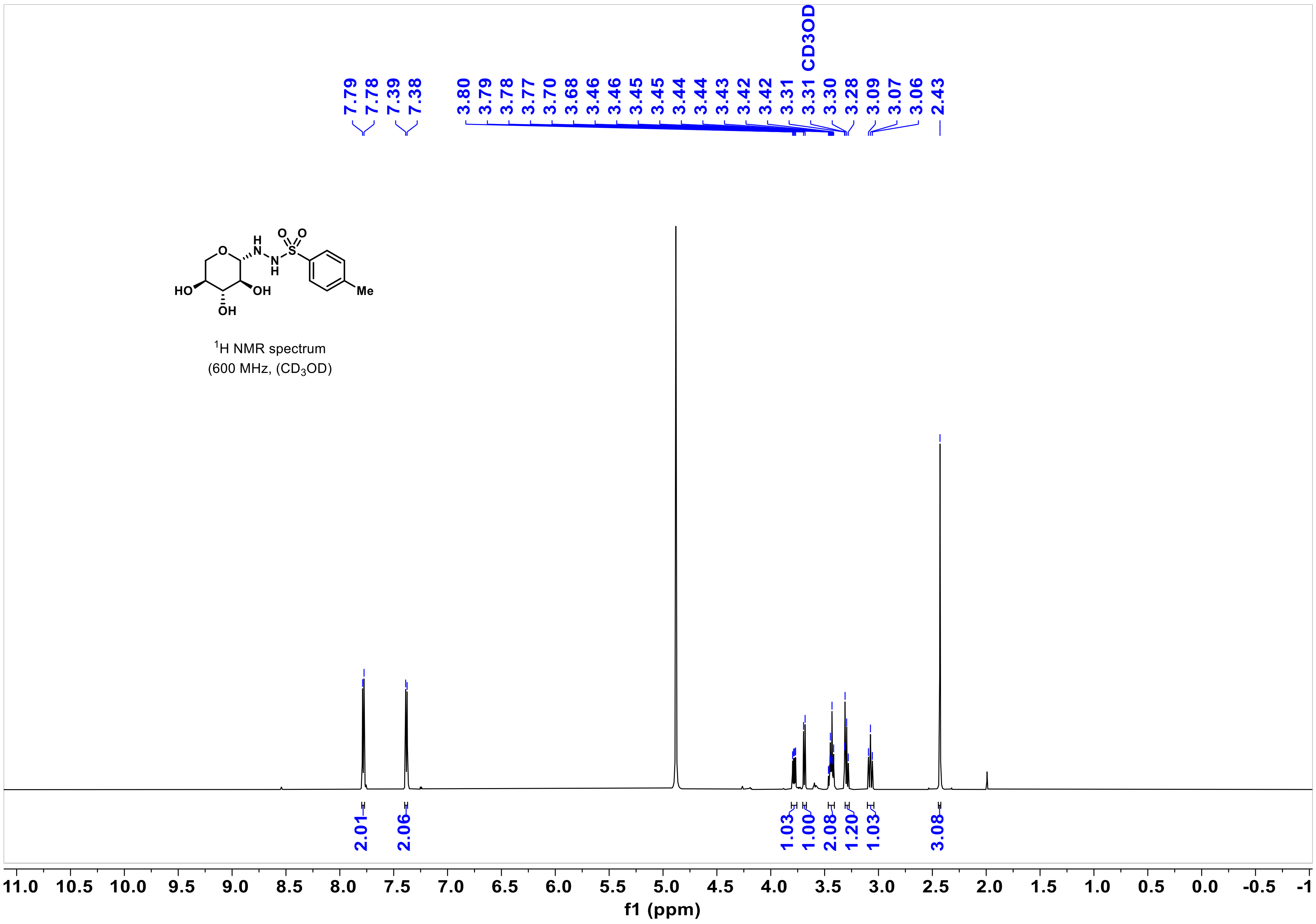

^{1}H NMR of compound **β-SH21**

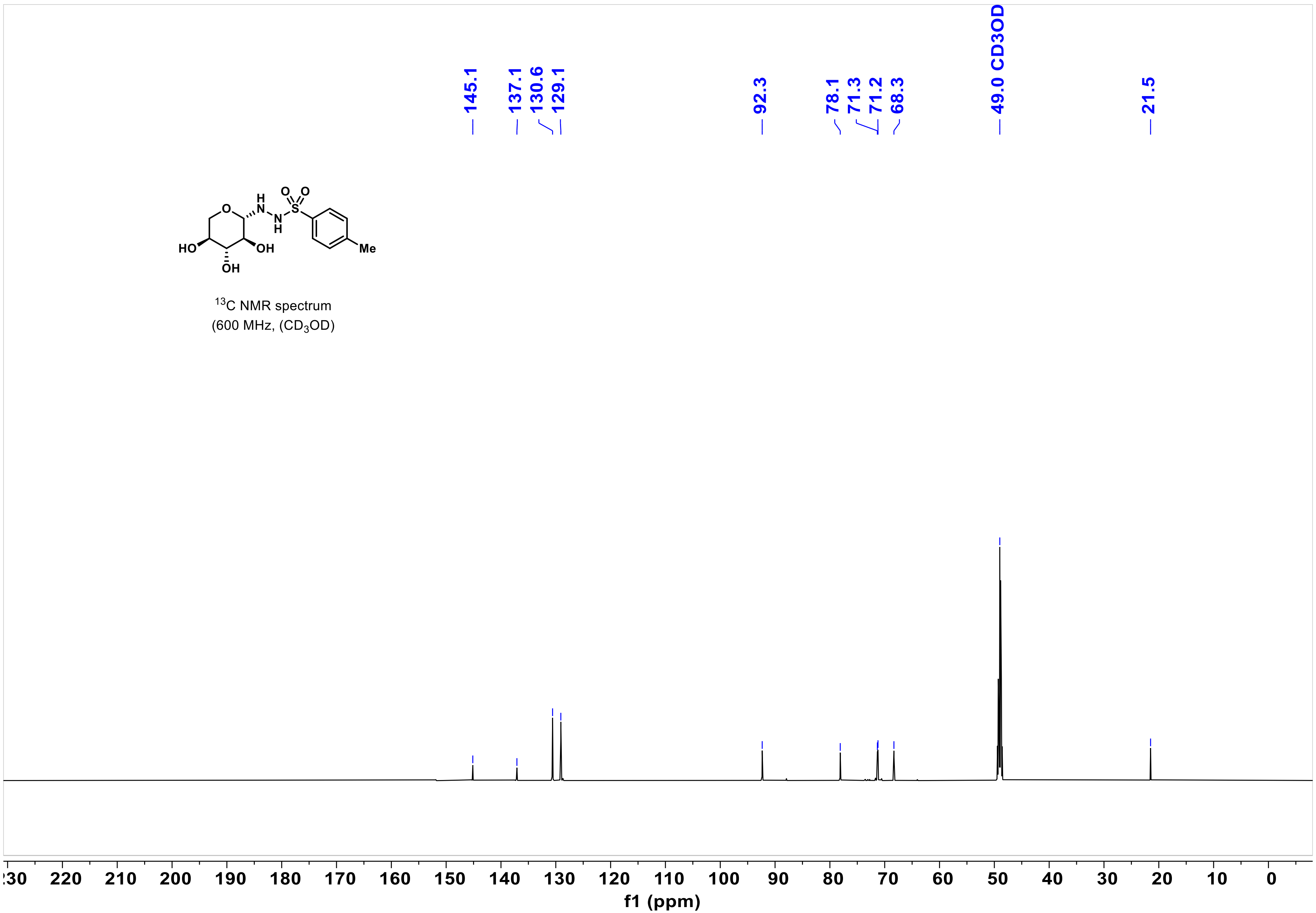

^{13}C NMR of compound **β-SH21**

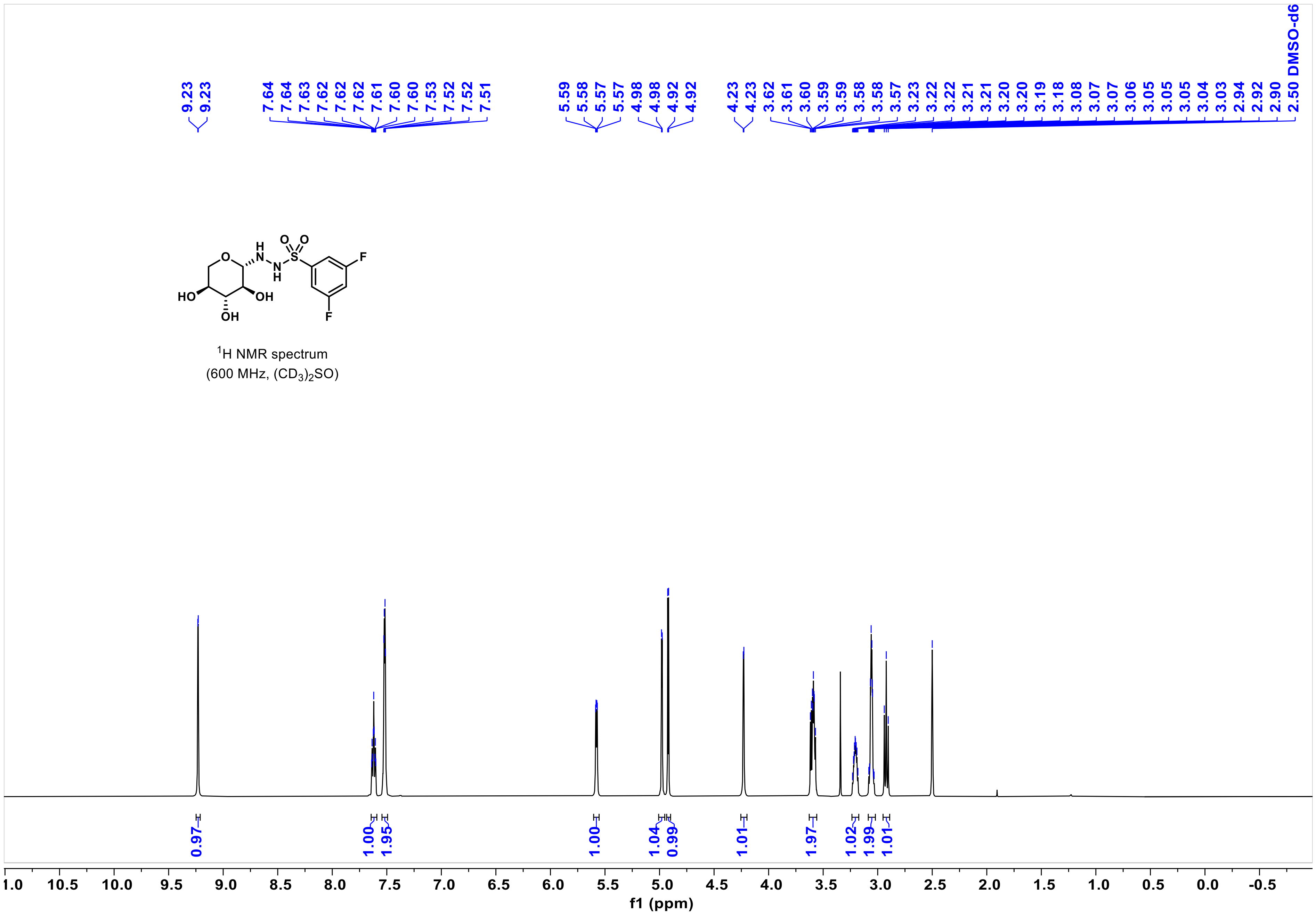

^{1}H NMR of compound **β-SH22**

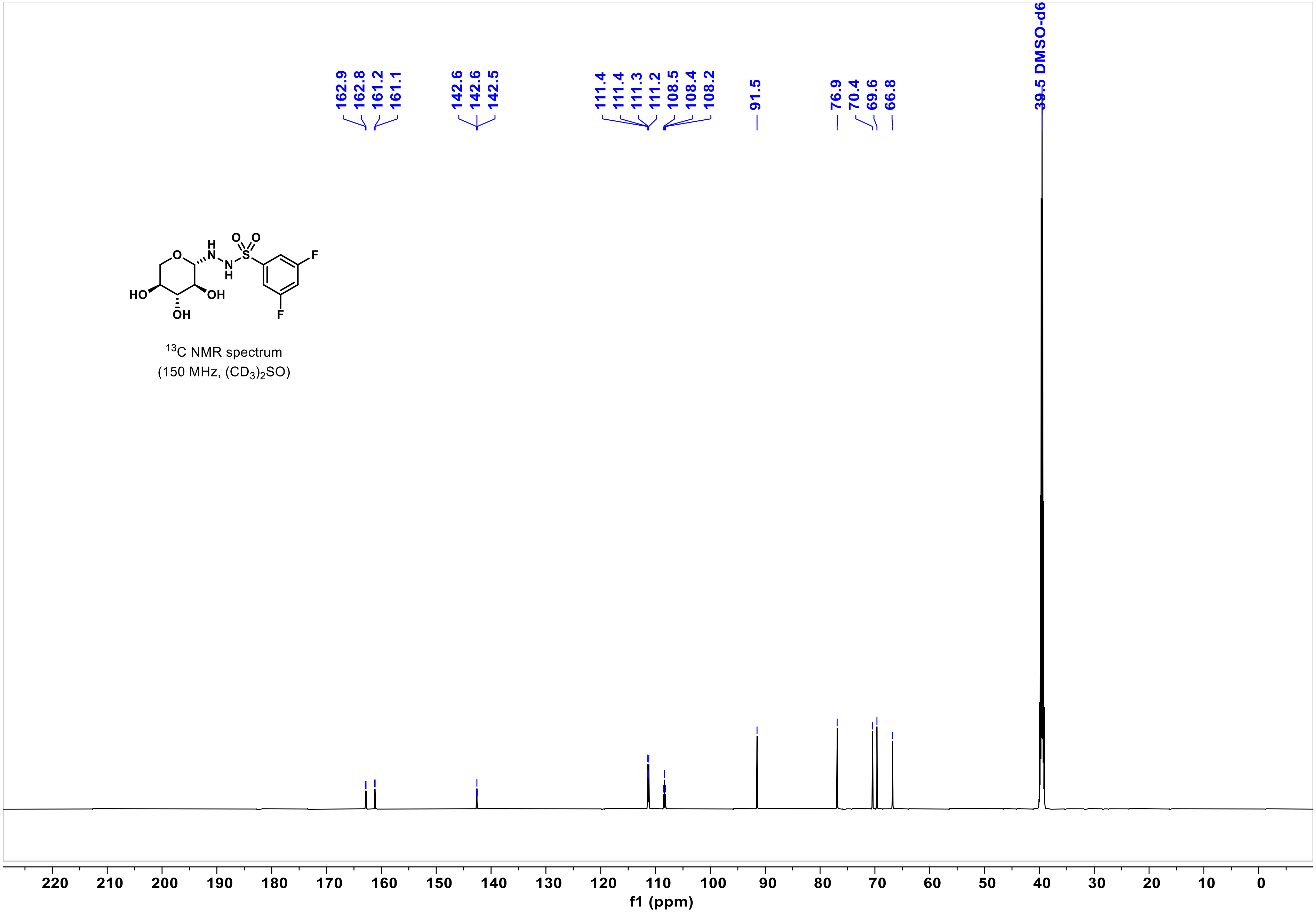

^{13}C NMR of compound **β-SH22**

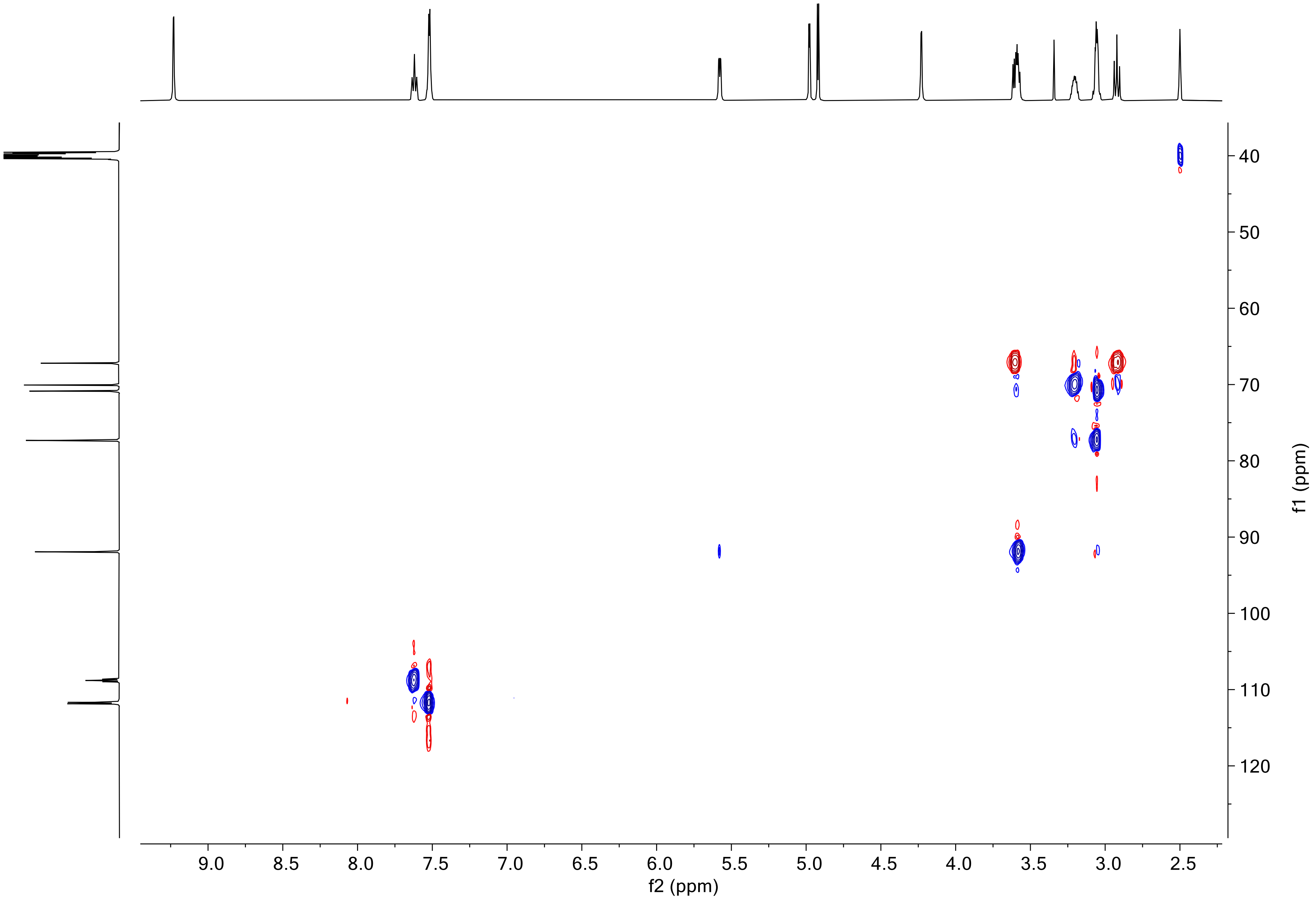

H-C HSQC NMR Spectrum of compound **β-SH22**

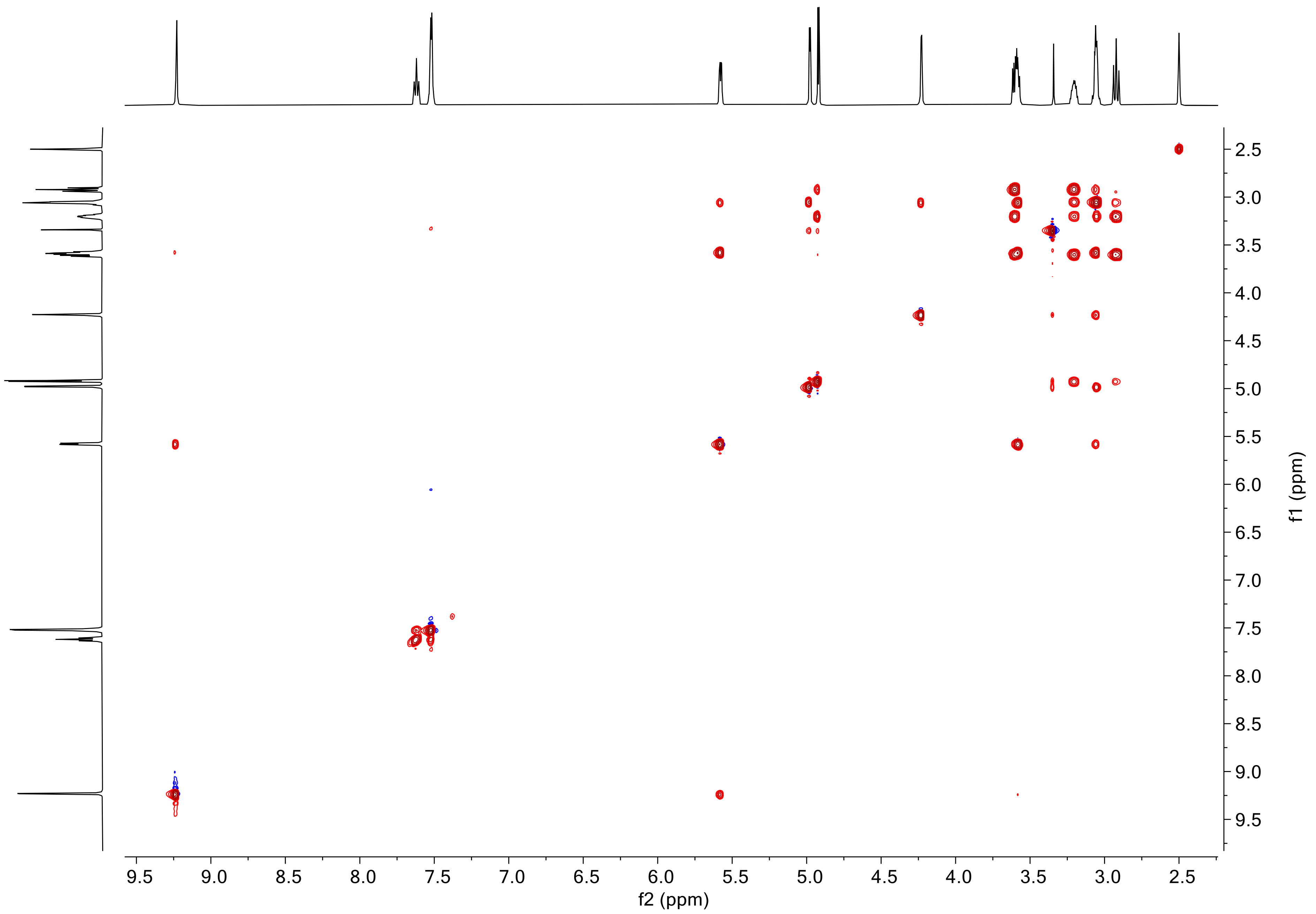

H-H COSY NMR Spectrum of compound **β-SH22**

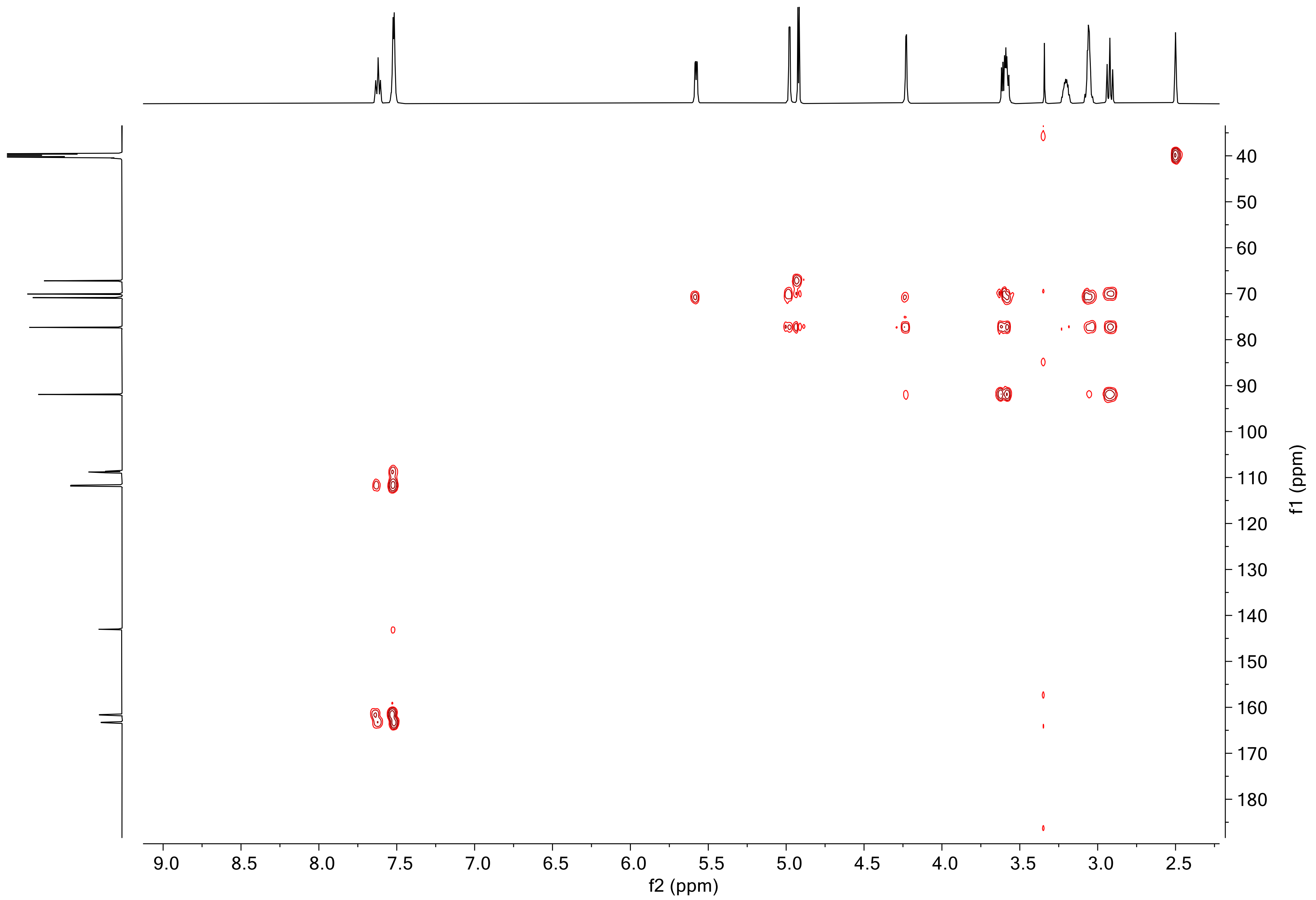

H-C HMBC NMR Spectrum of compound **β-SH22**

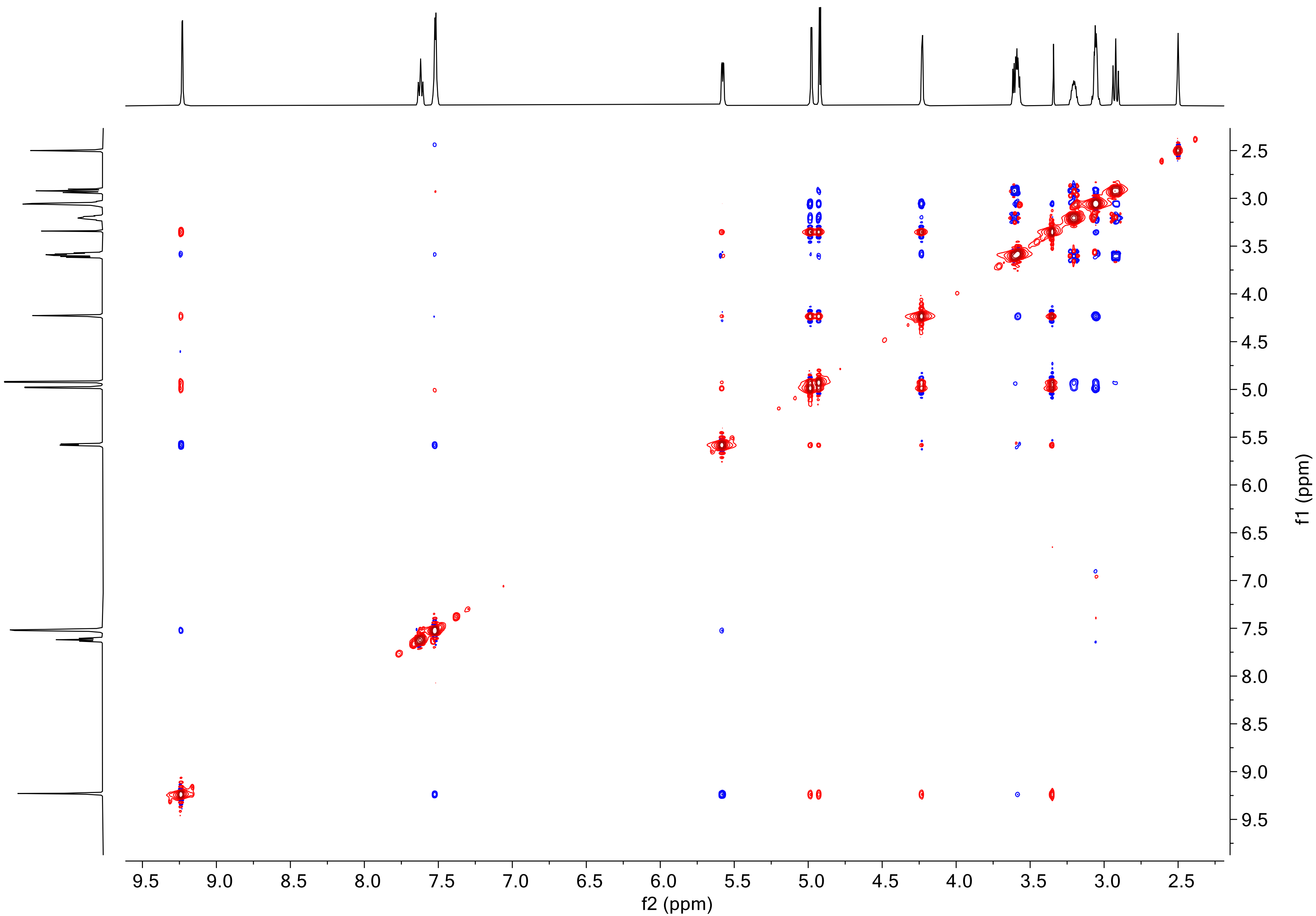

H-H NOESY NMR Spectrum of compound **β-SH22**

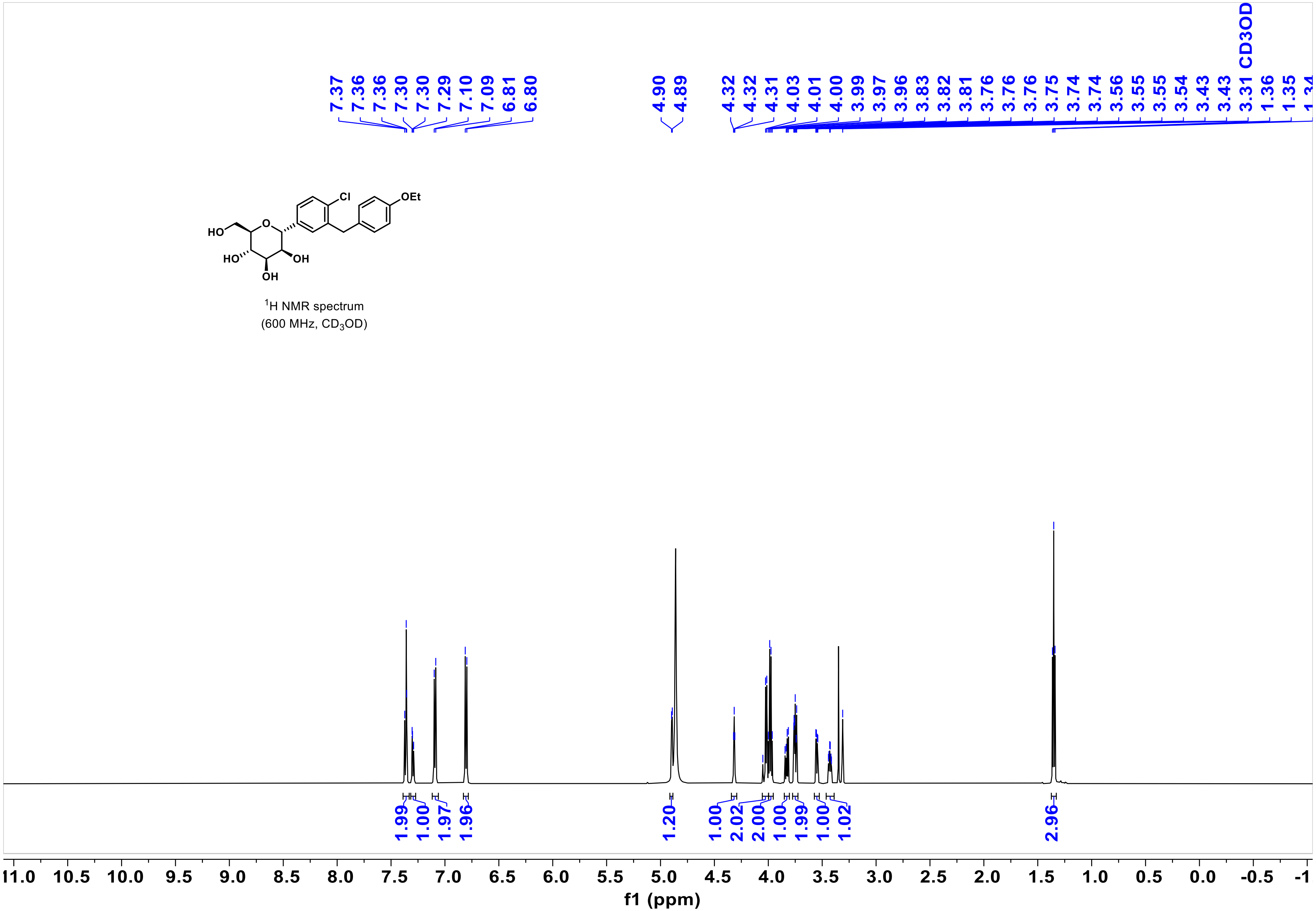

^{1}H NMR of compound **α-47**

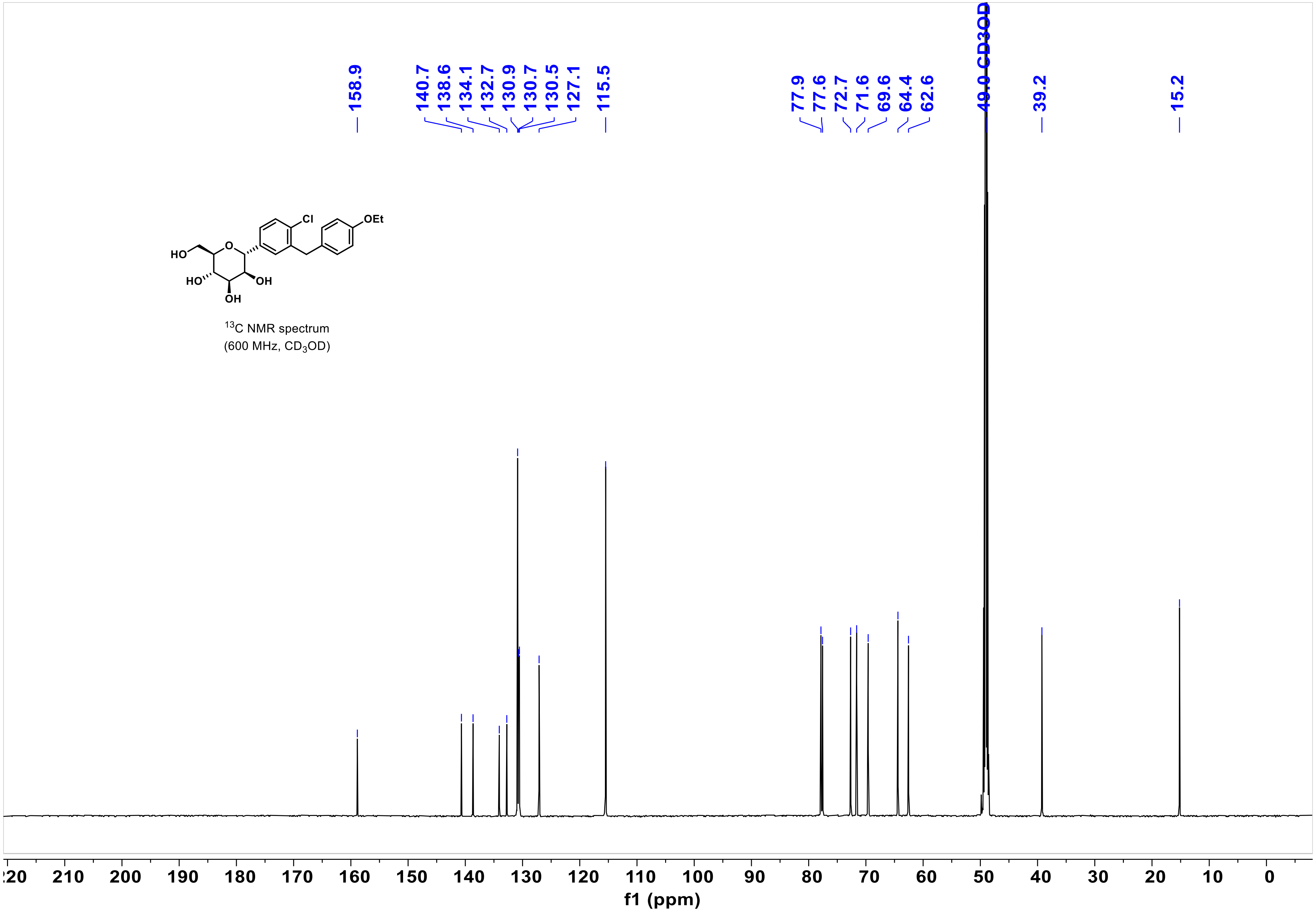

^{13}C NMR of compound **α-47**

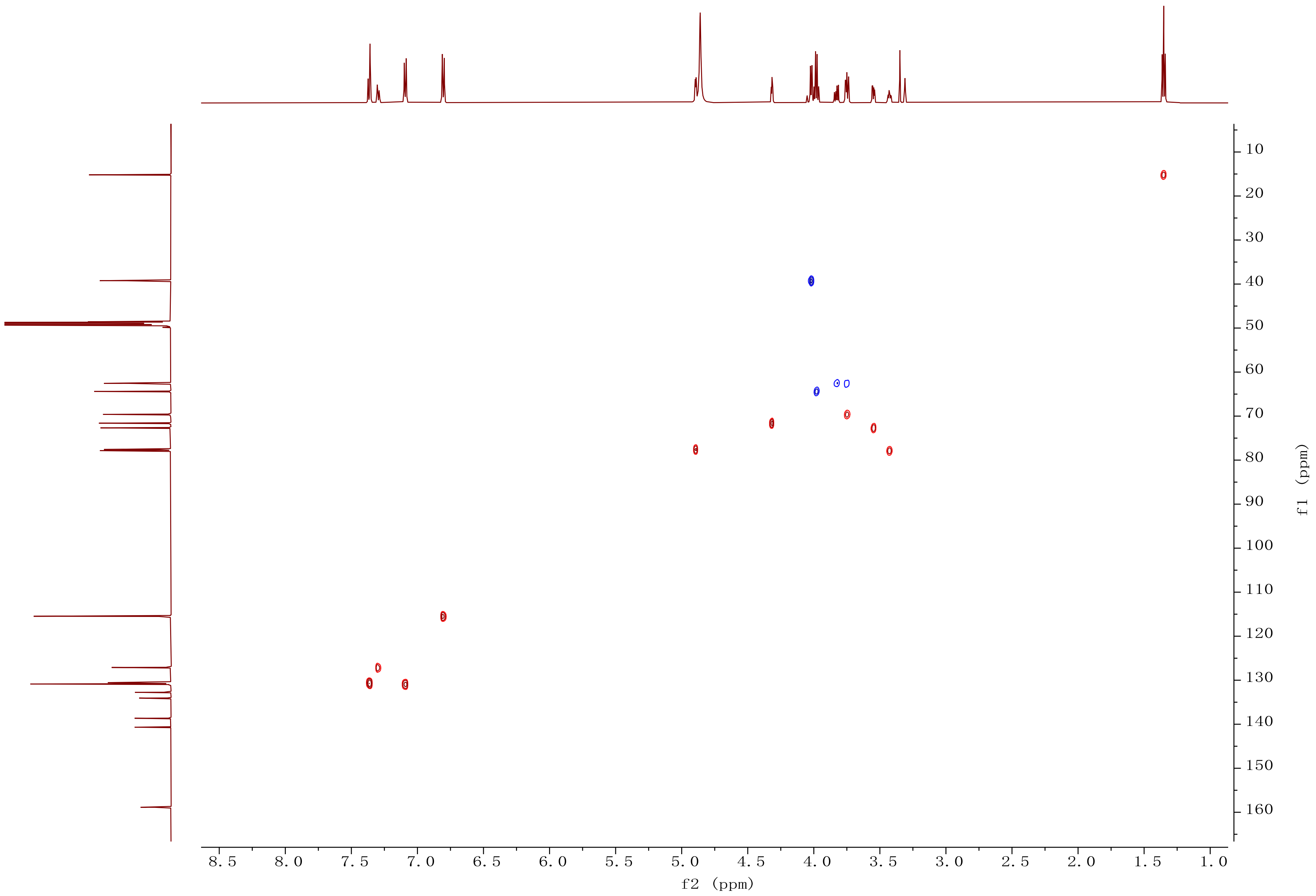

H-H HSQC NMR of compound **α-47**

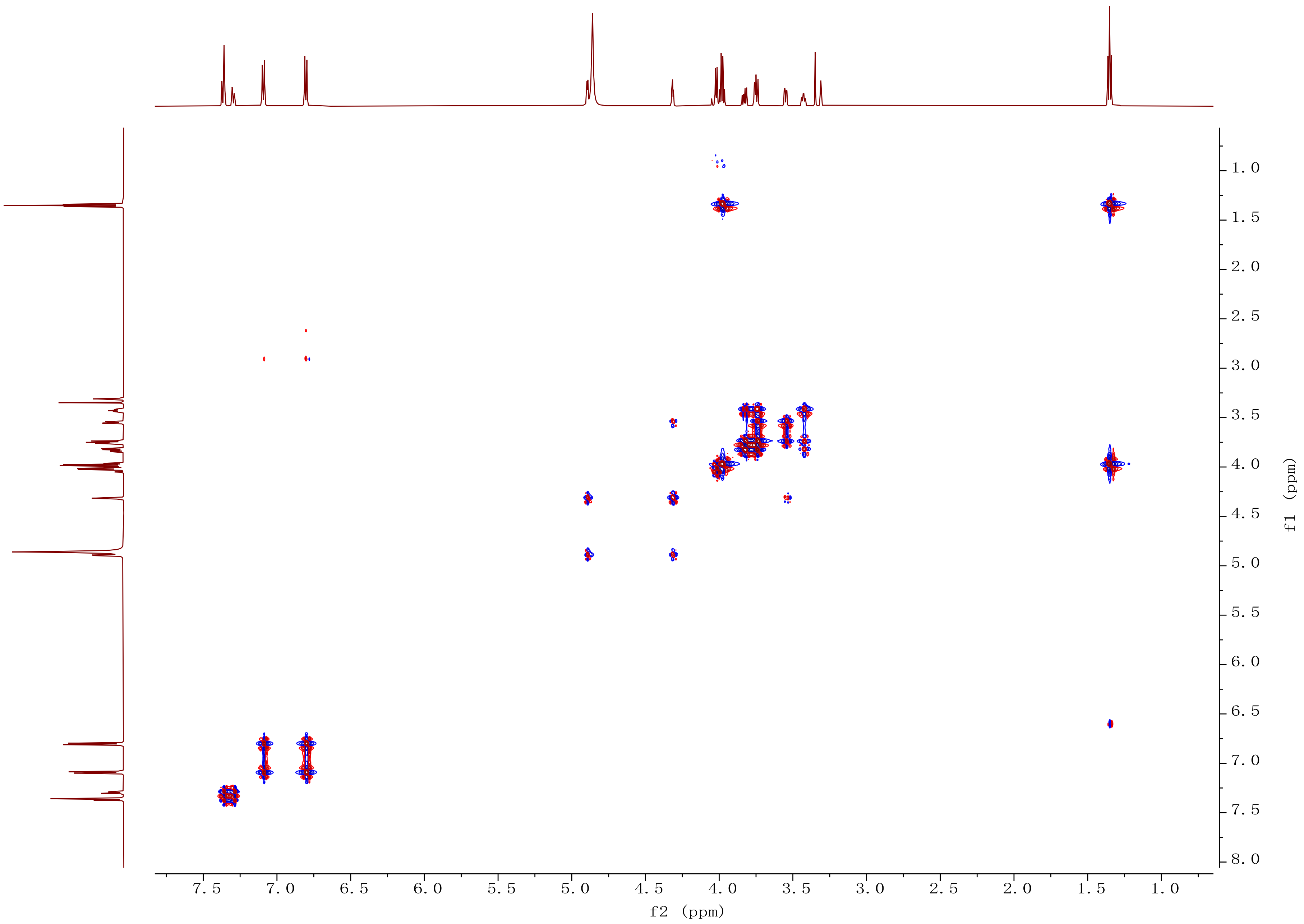

H-H COSY NMR of compound **α-47**

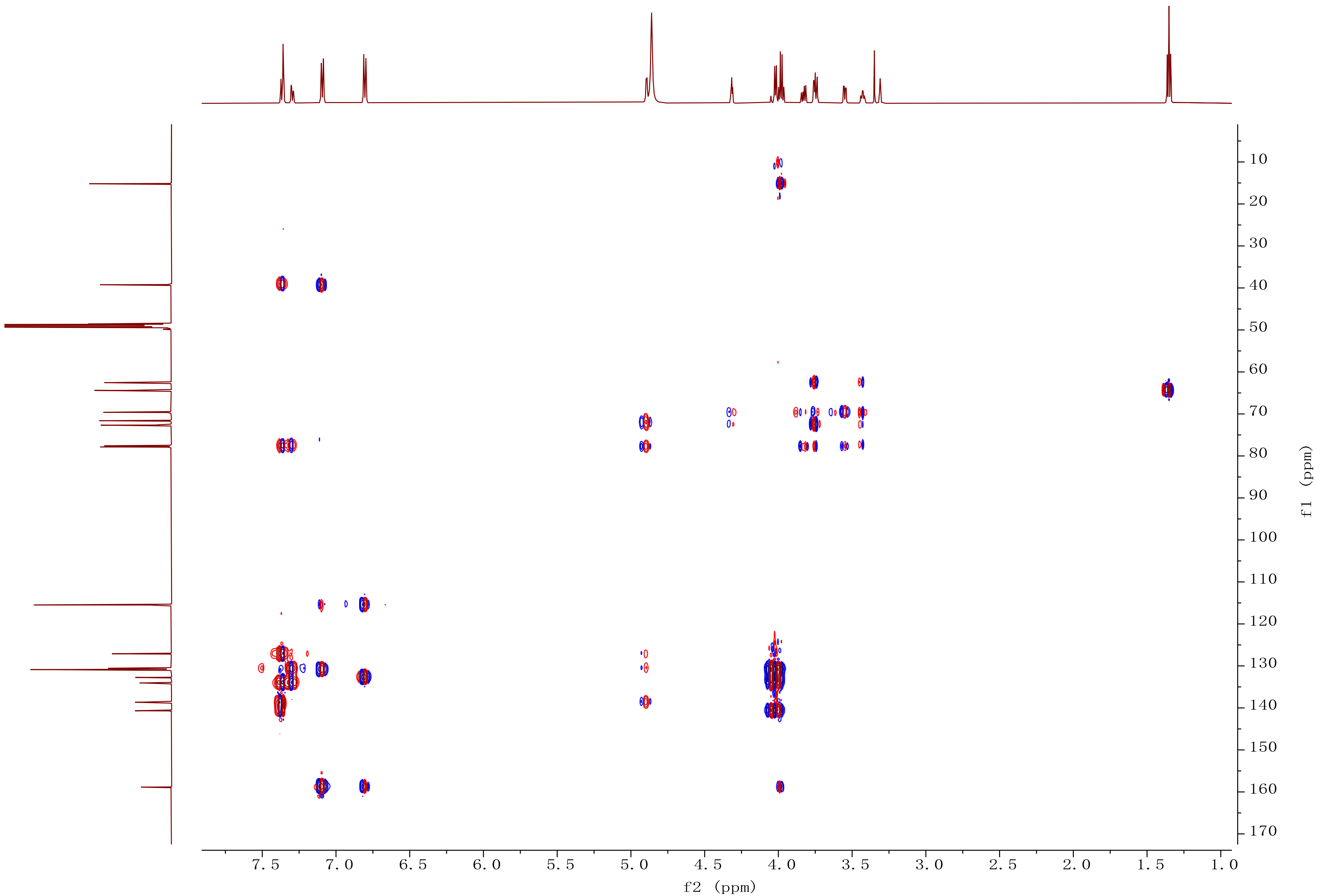

H-C HMBC NMR of compound **α-47**

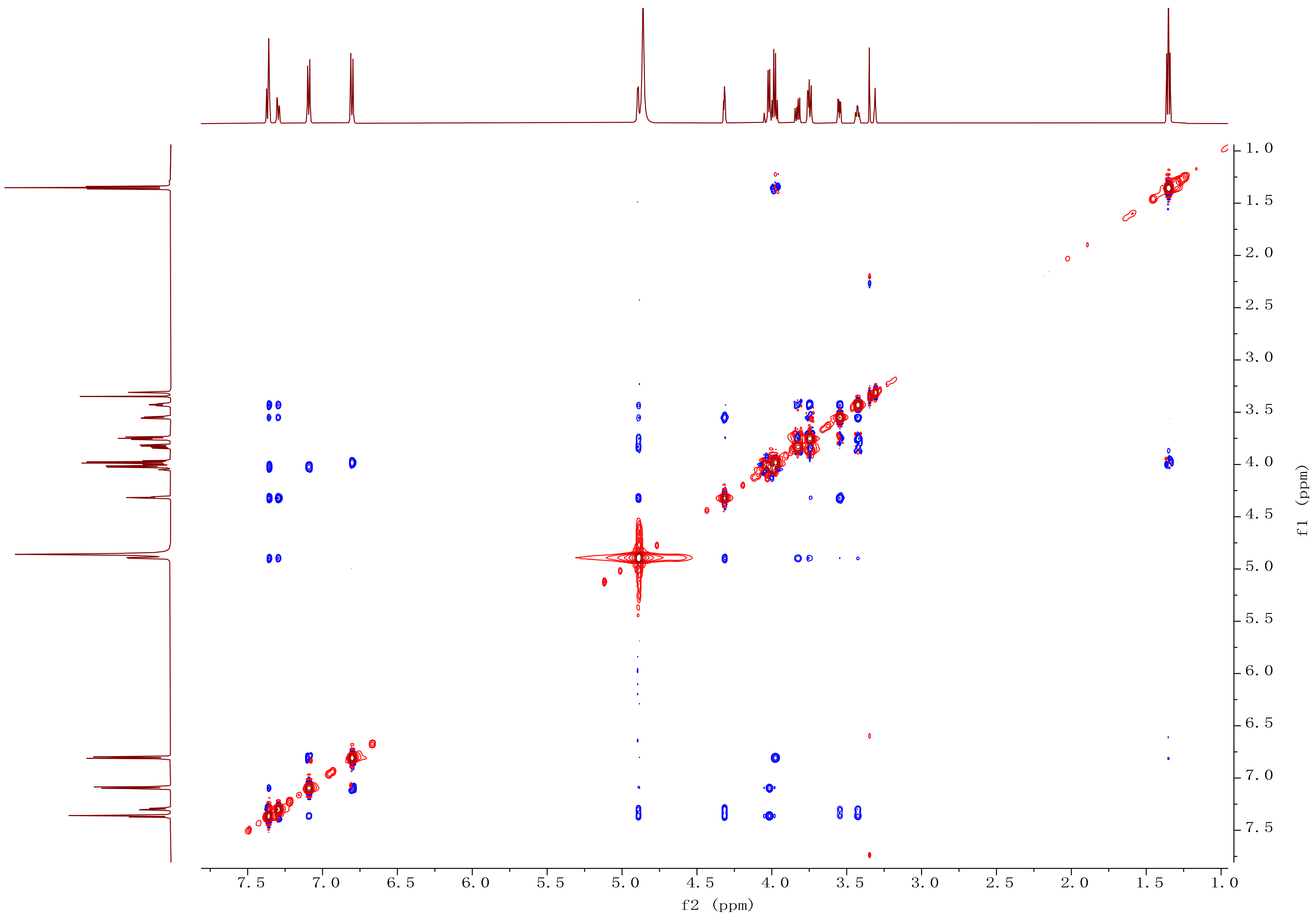

NOESY NMR of compound **α-47**

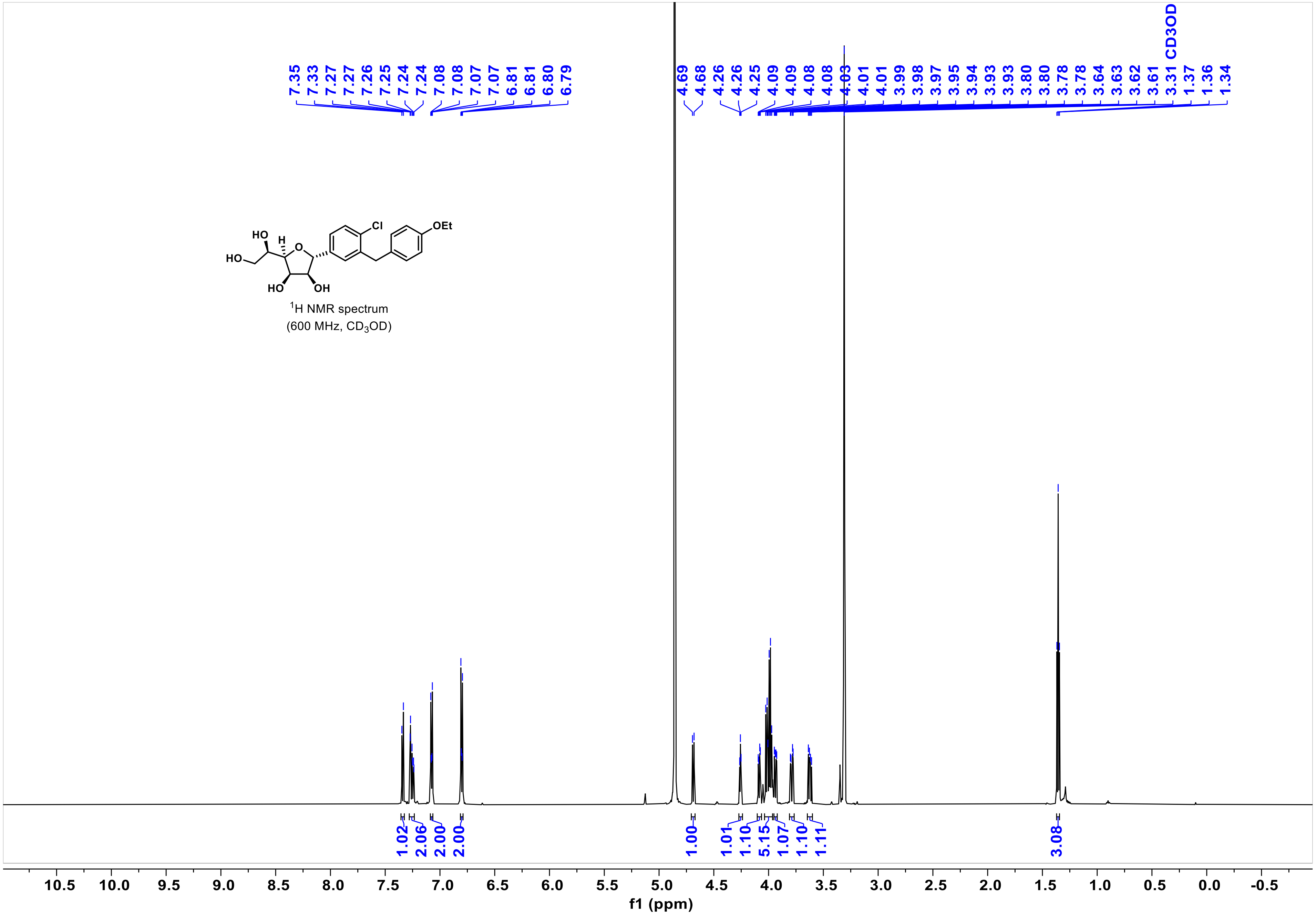

^{1}H NMR of compound **α-48**

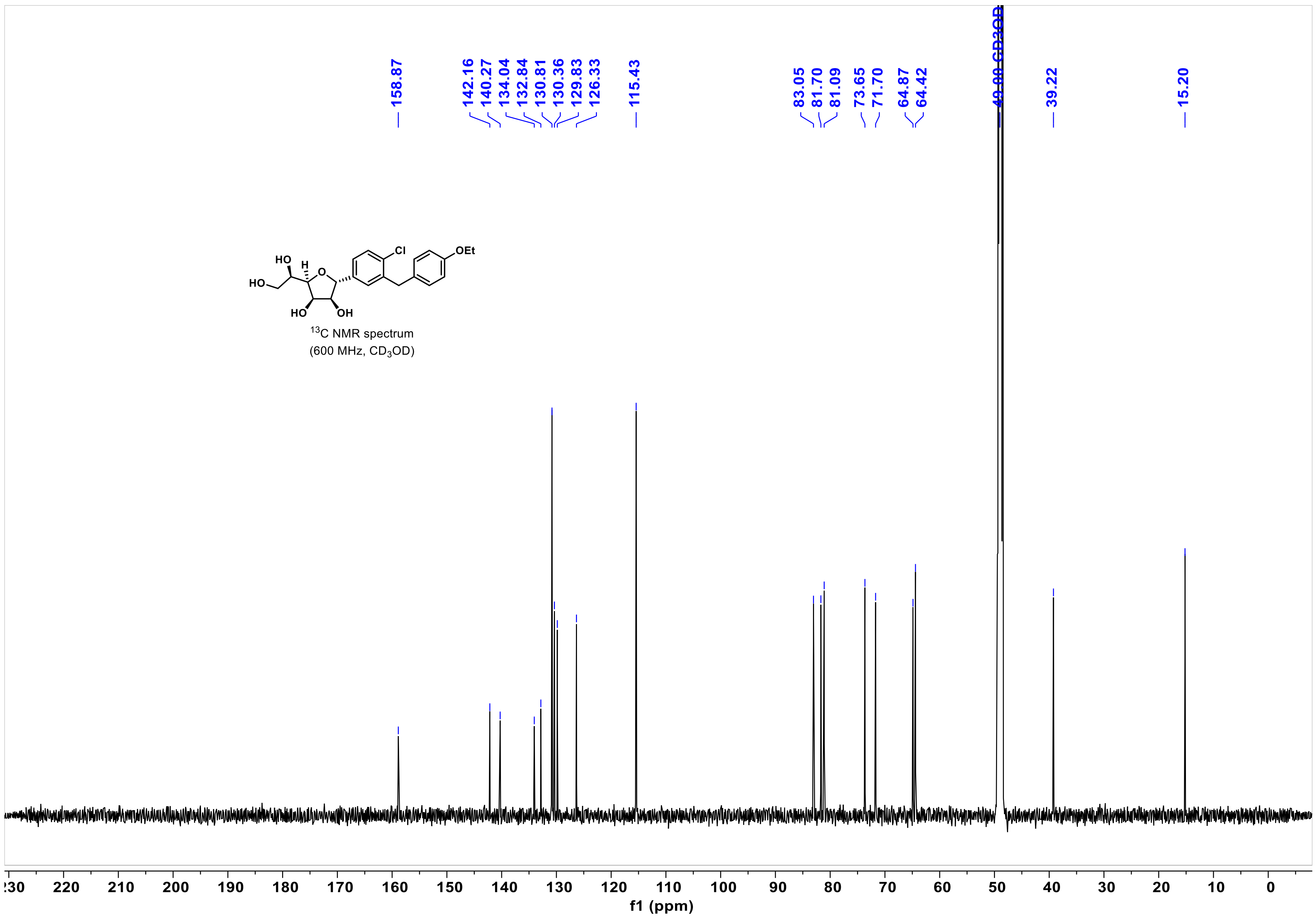

^{13}C NMR of compound **α-48**

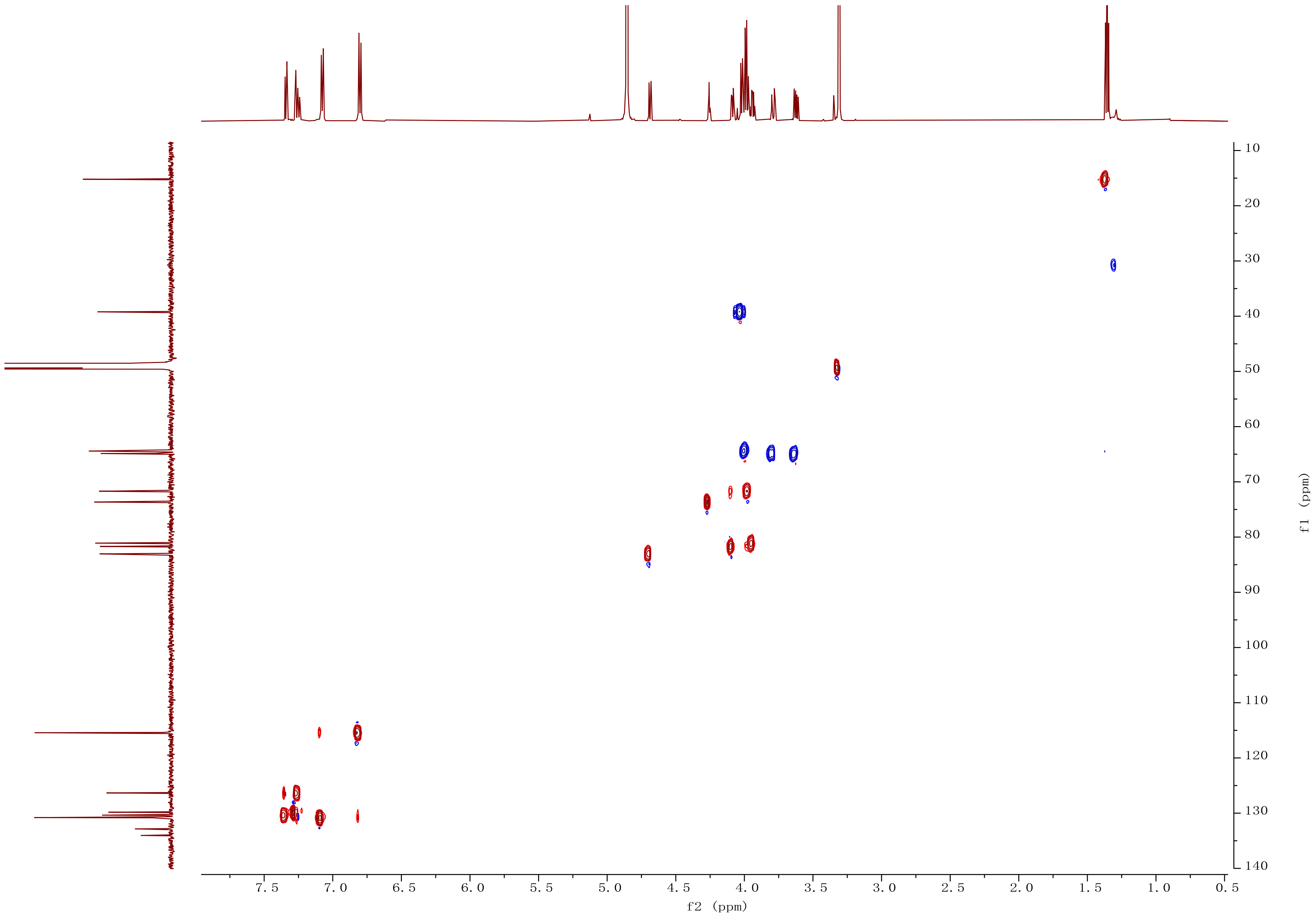

H-H HSQC NMR of compound **α-48**

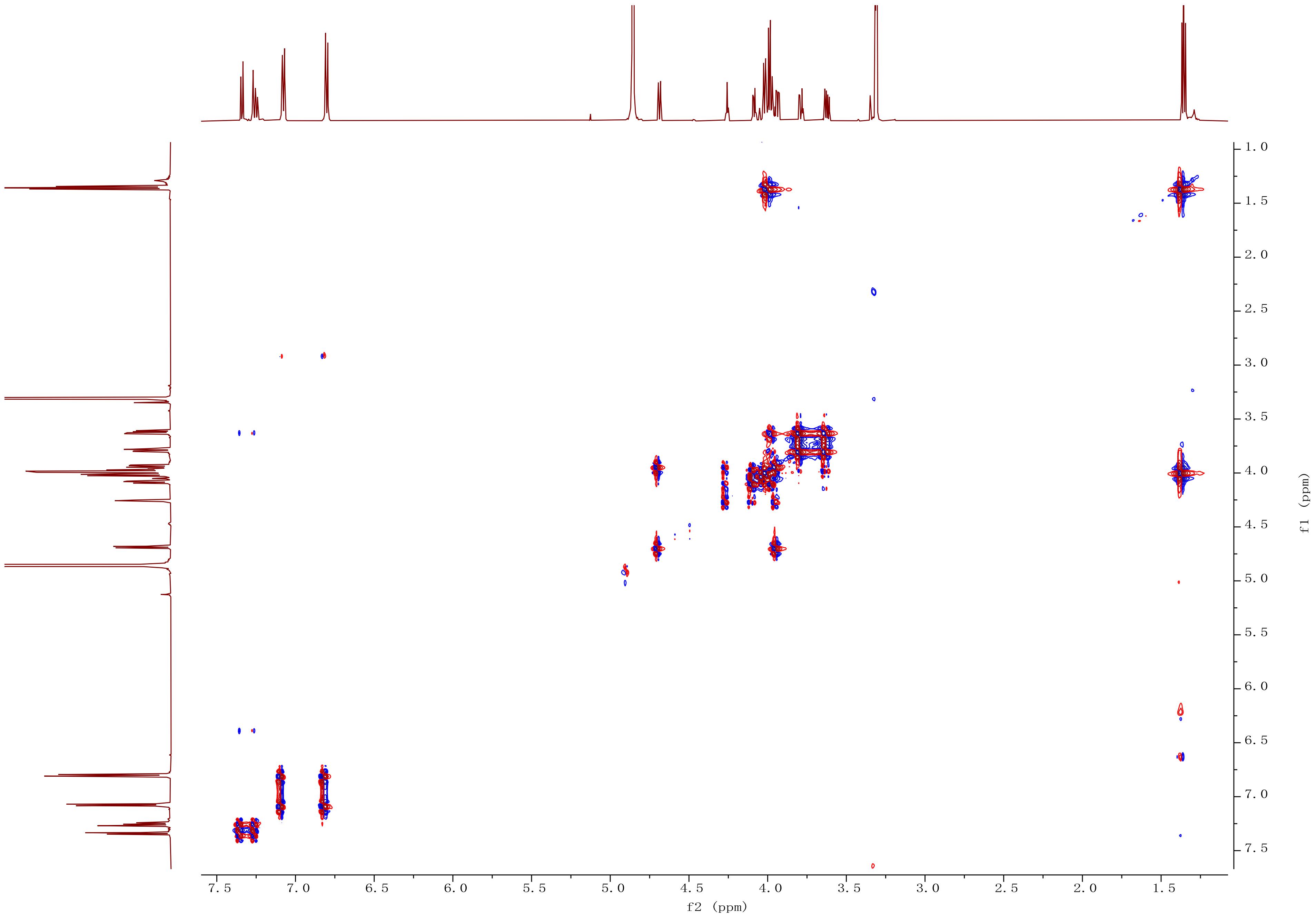

H-H COSY NMR of compound **α-48**

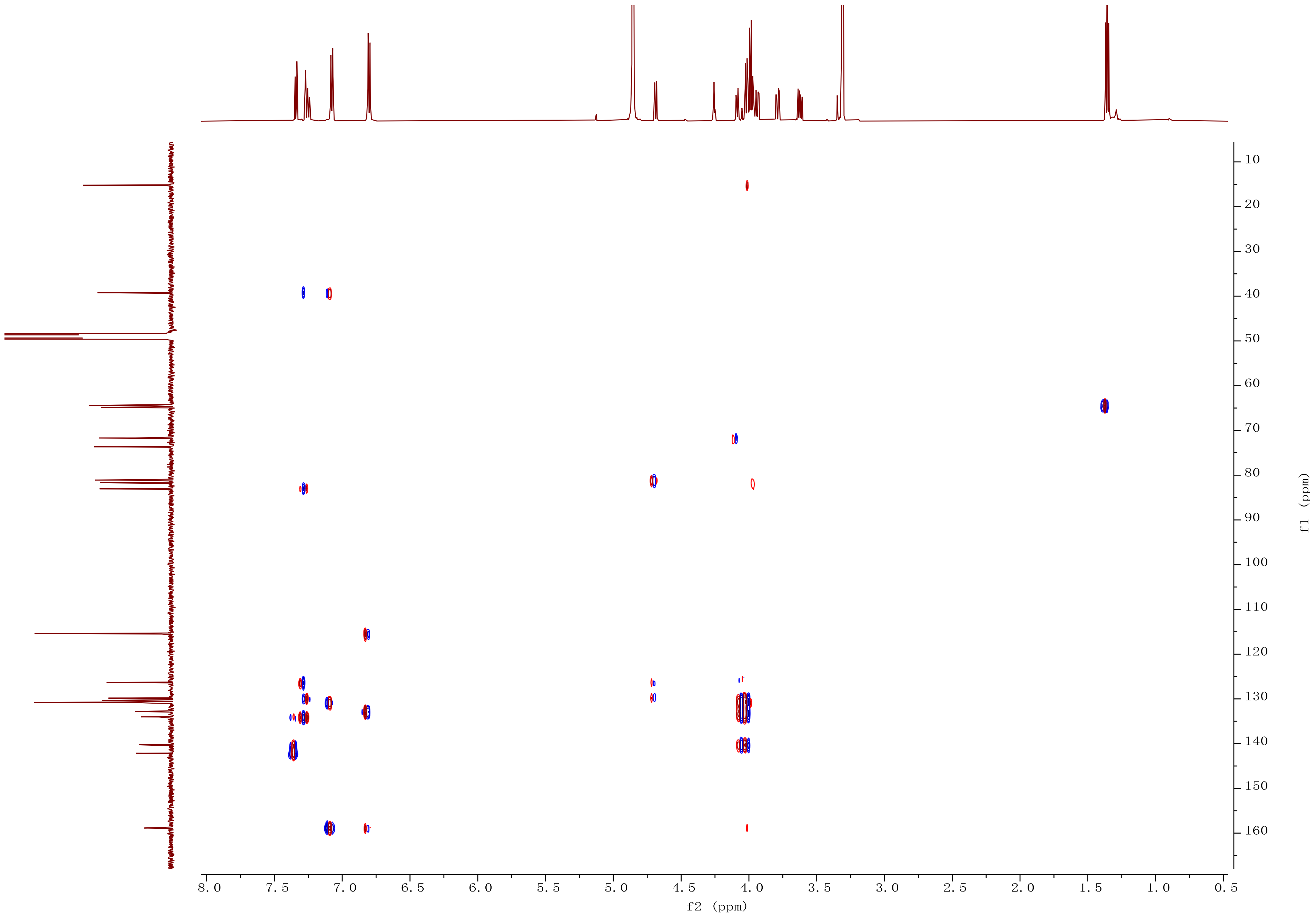

H-C HMBC NMR of compound **α-48**

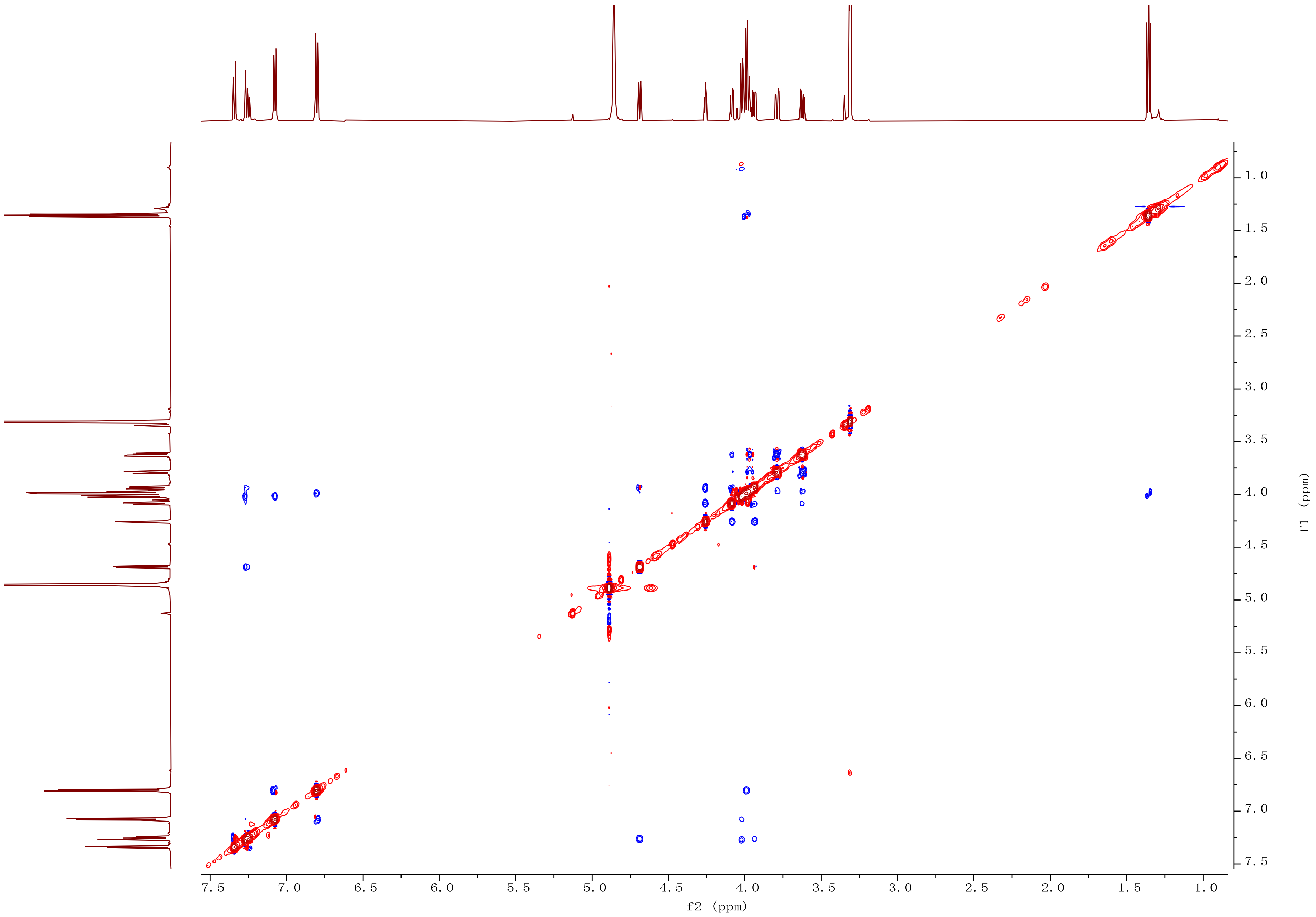

NOESY NMR of compound **α-48**

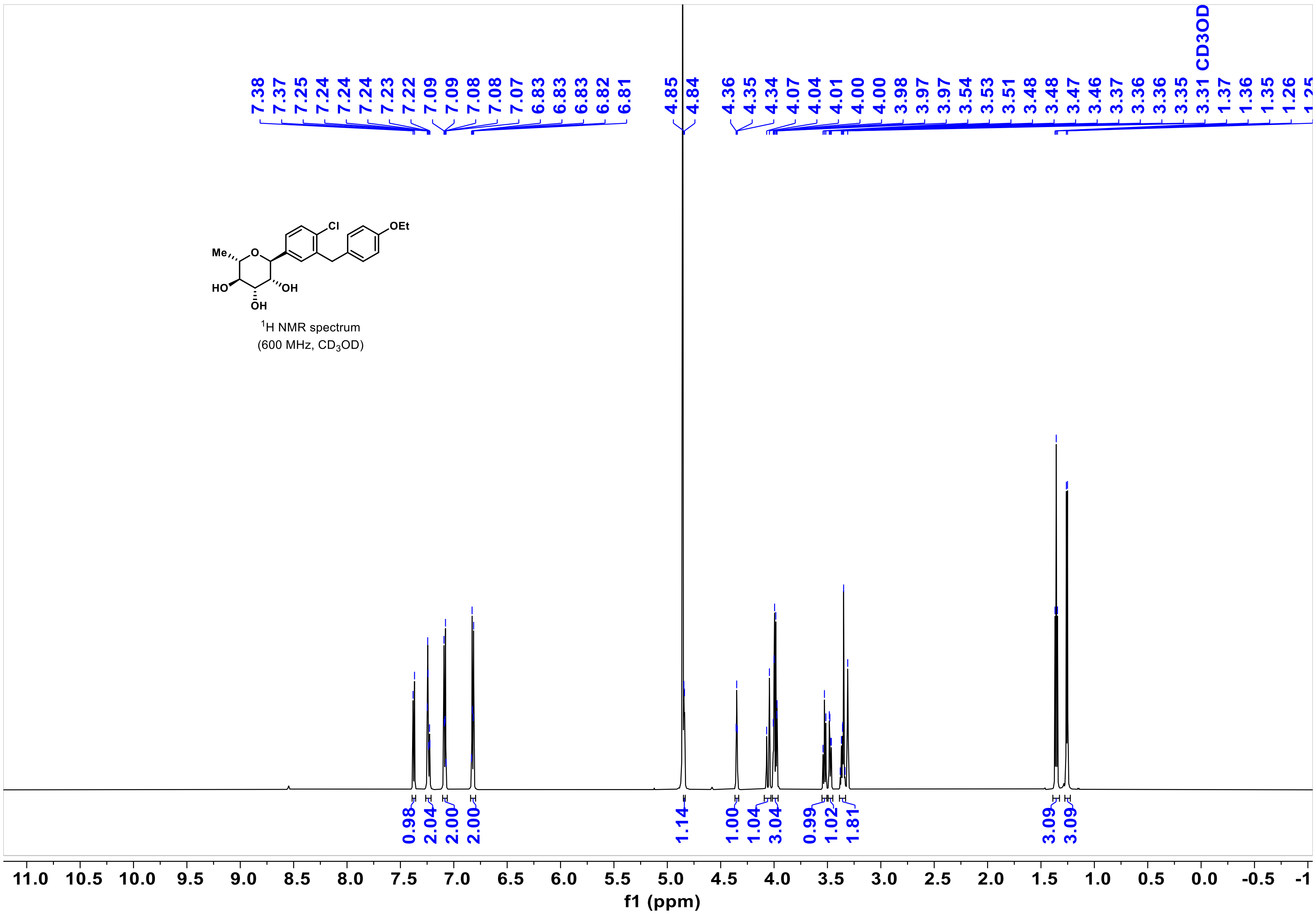

^{1}H NMR of compound **α-51**

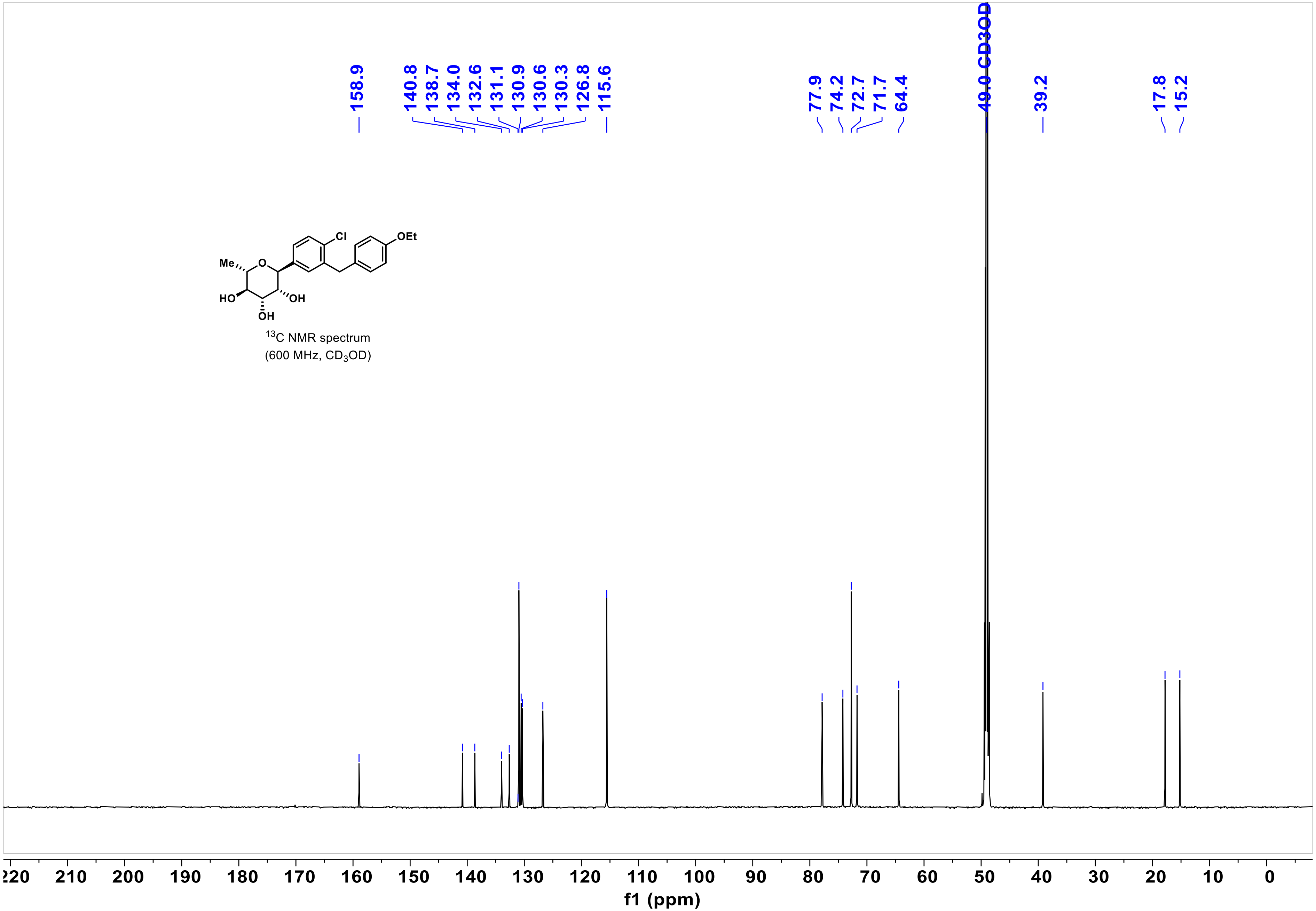

^{13}C NMR of compound **α-51**

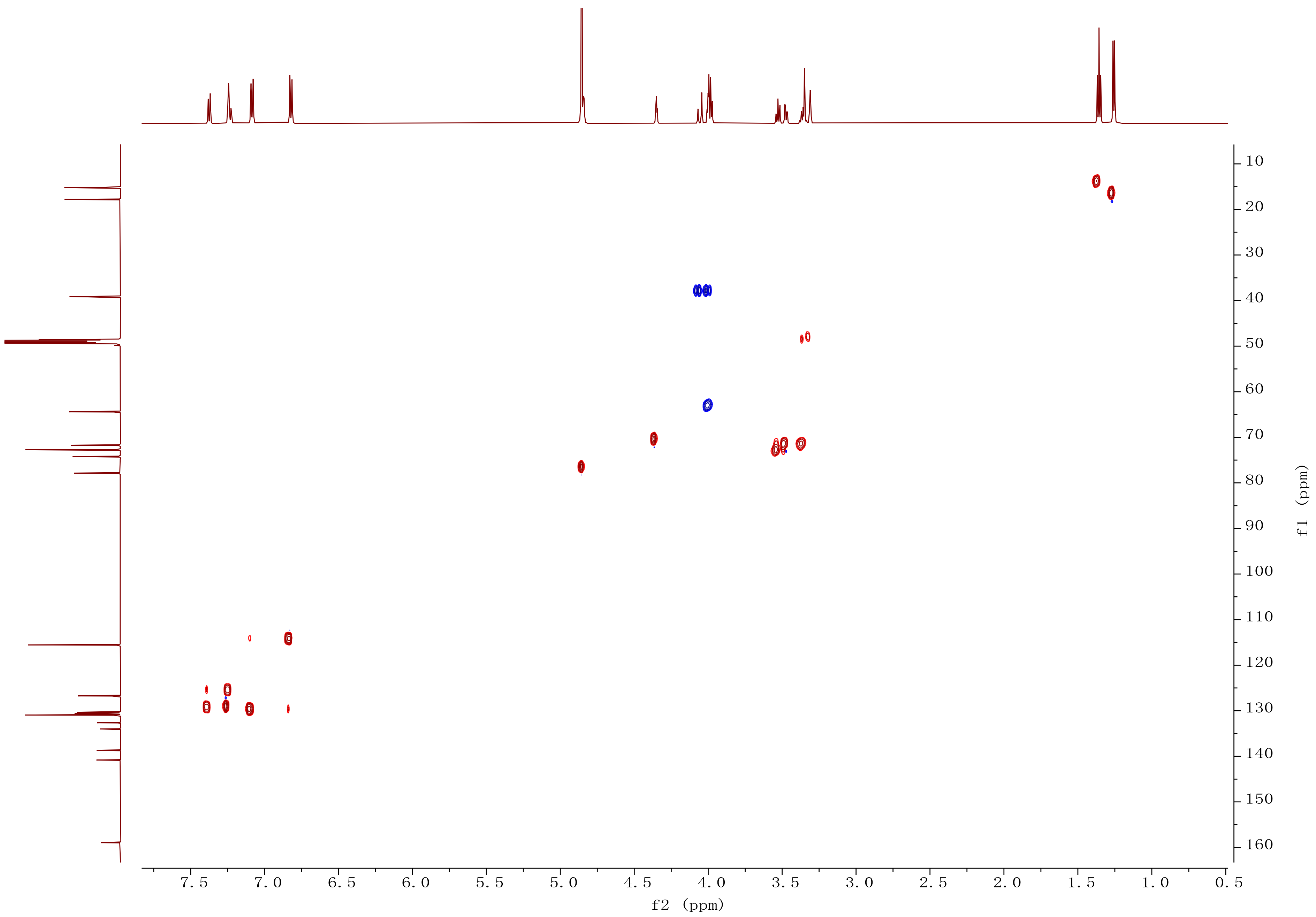

H-H HSQC NMR of compound **α-51**

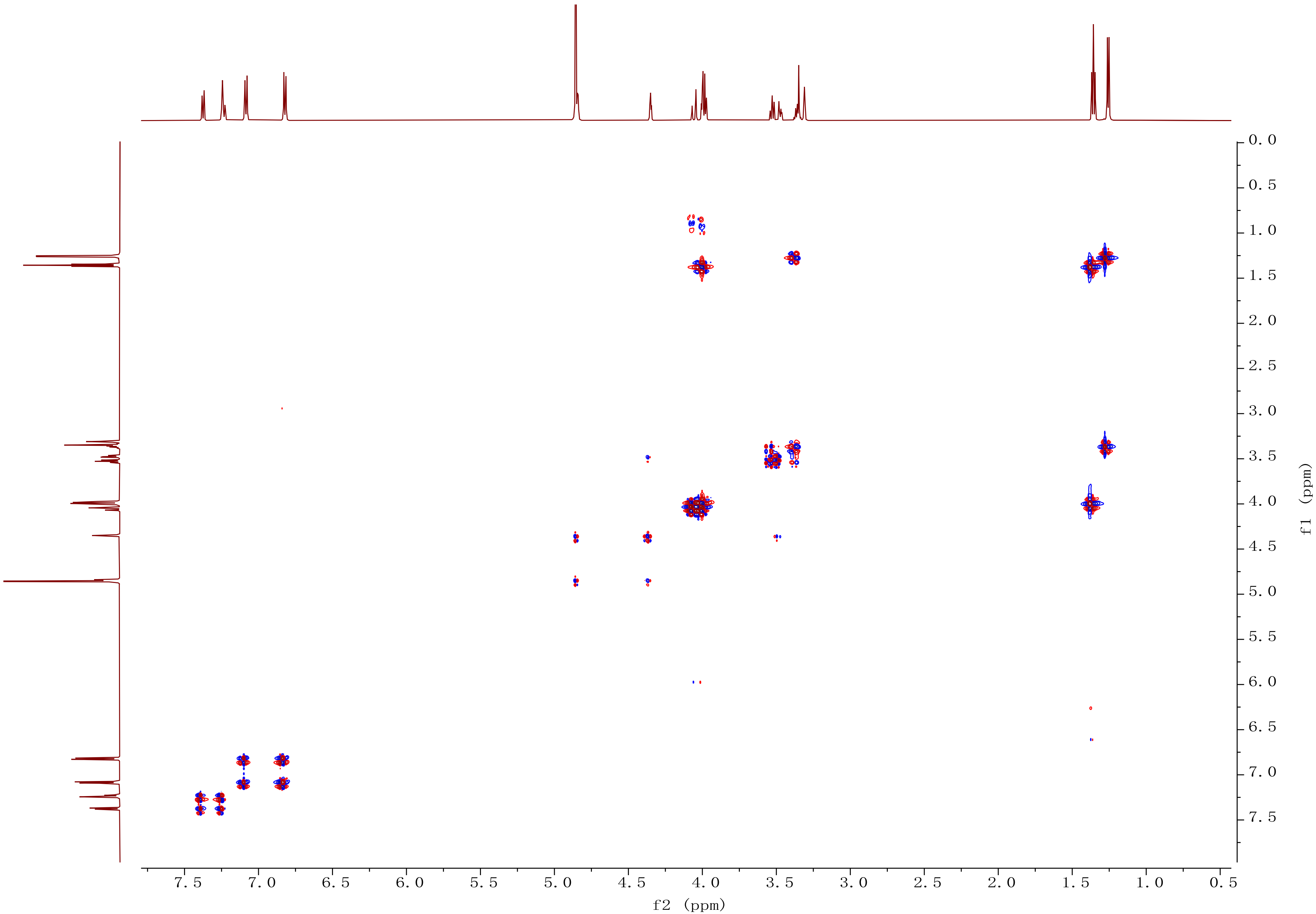

H-H COSY NMR of compound **α-51**

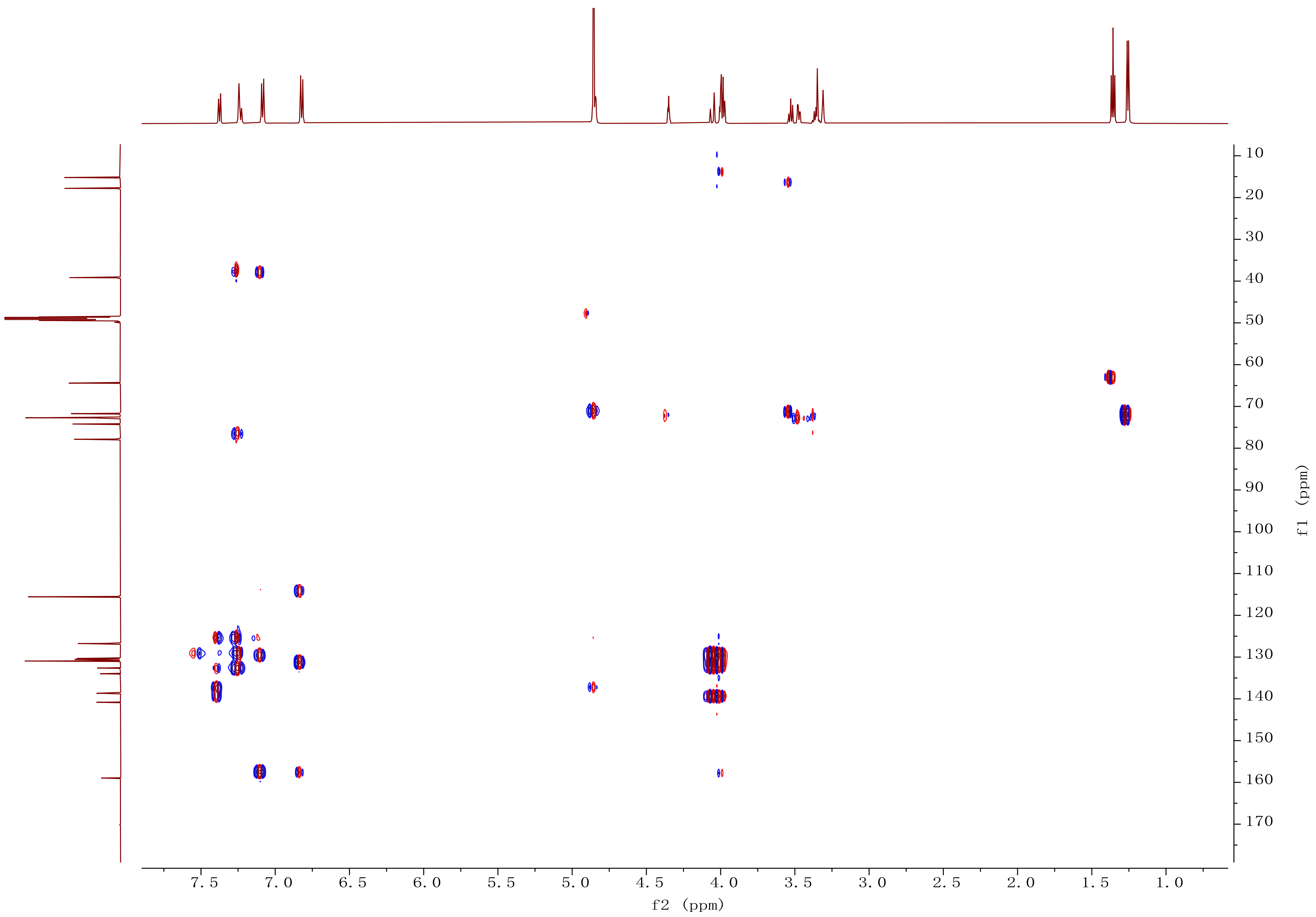

H-C HMBC NMR of compound **α-51**

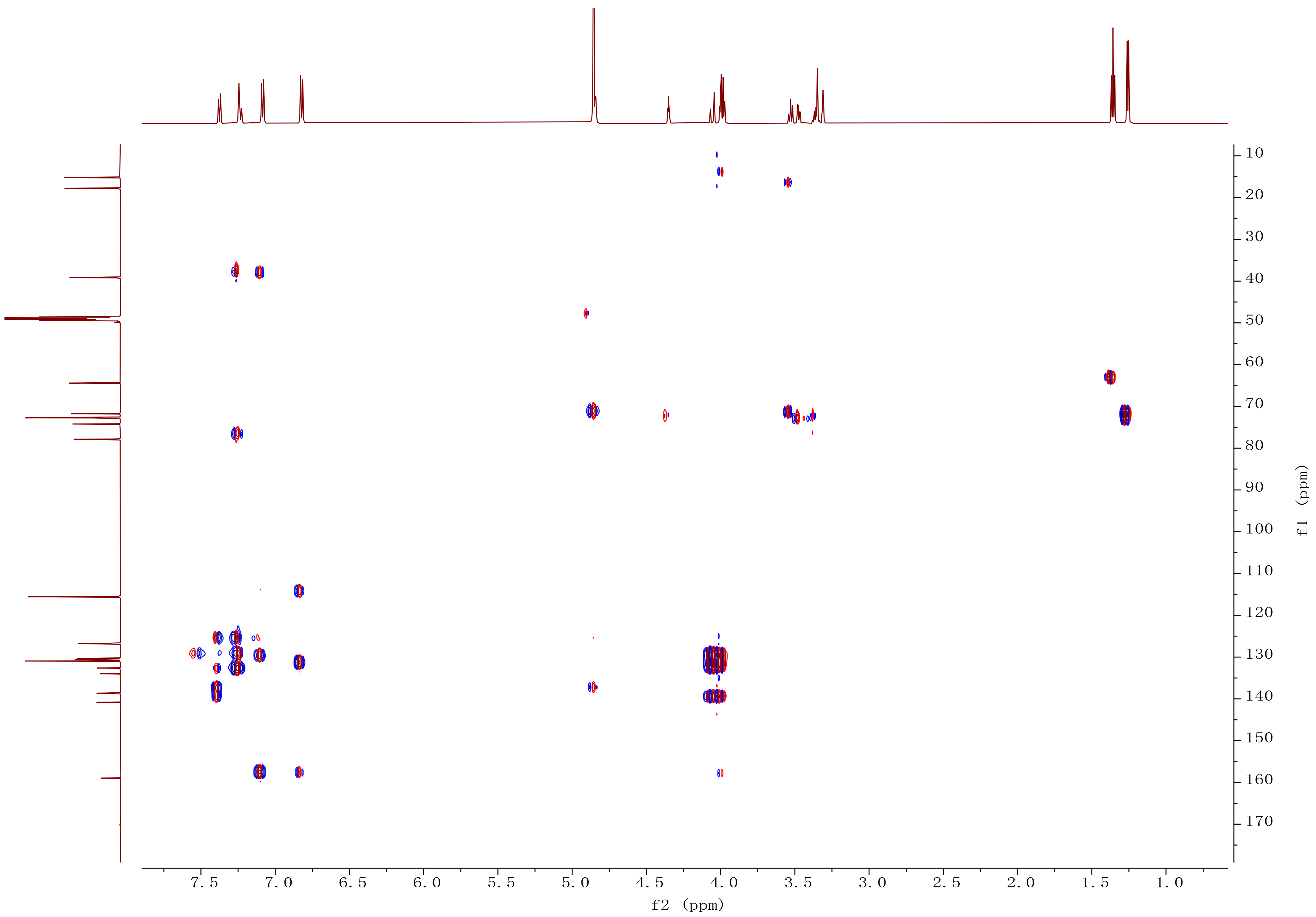

NOESY NMR of compound **α-51**

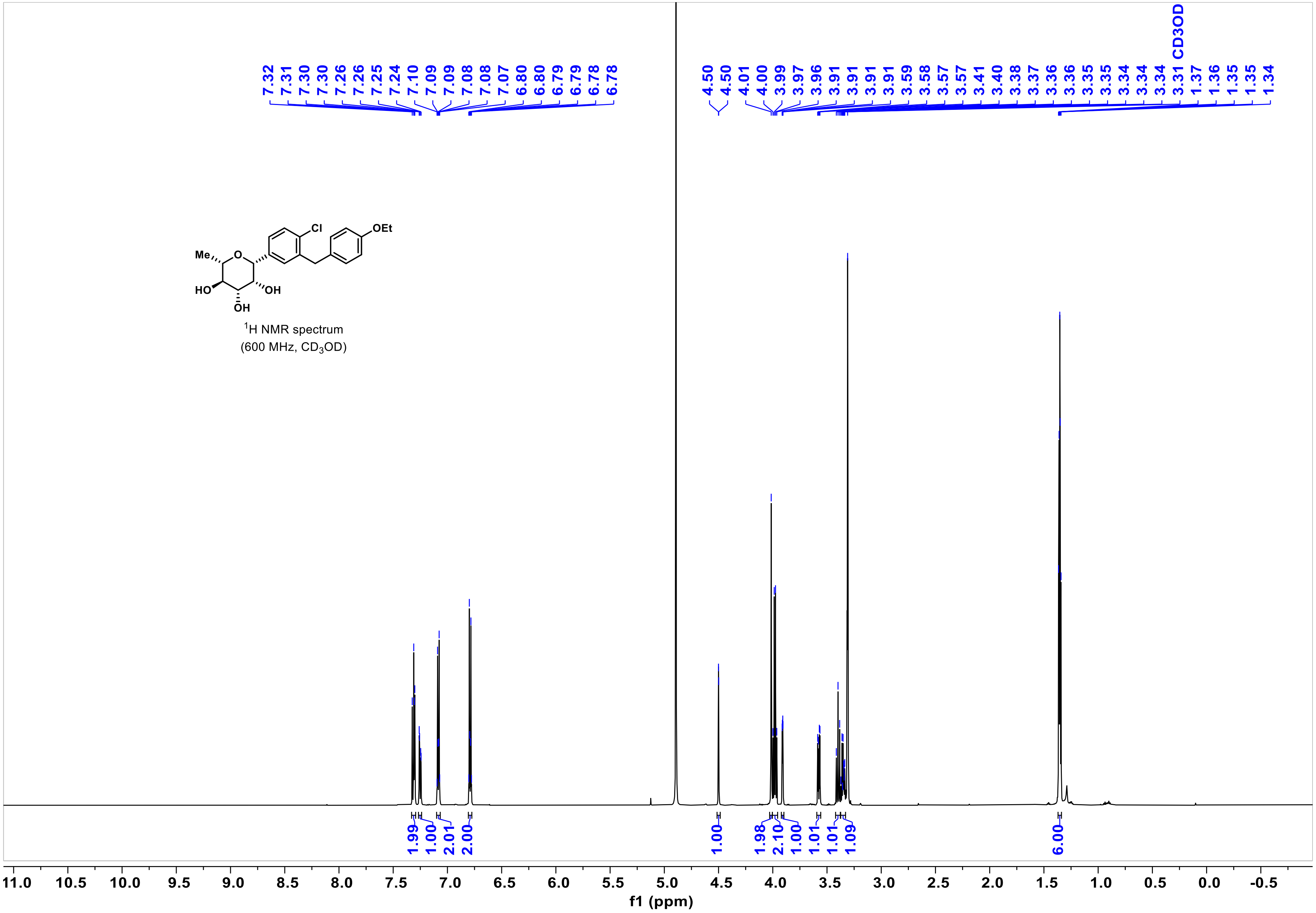

^{1}H NMR of compound **β-51**

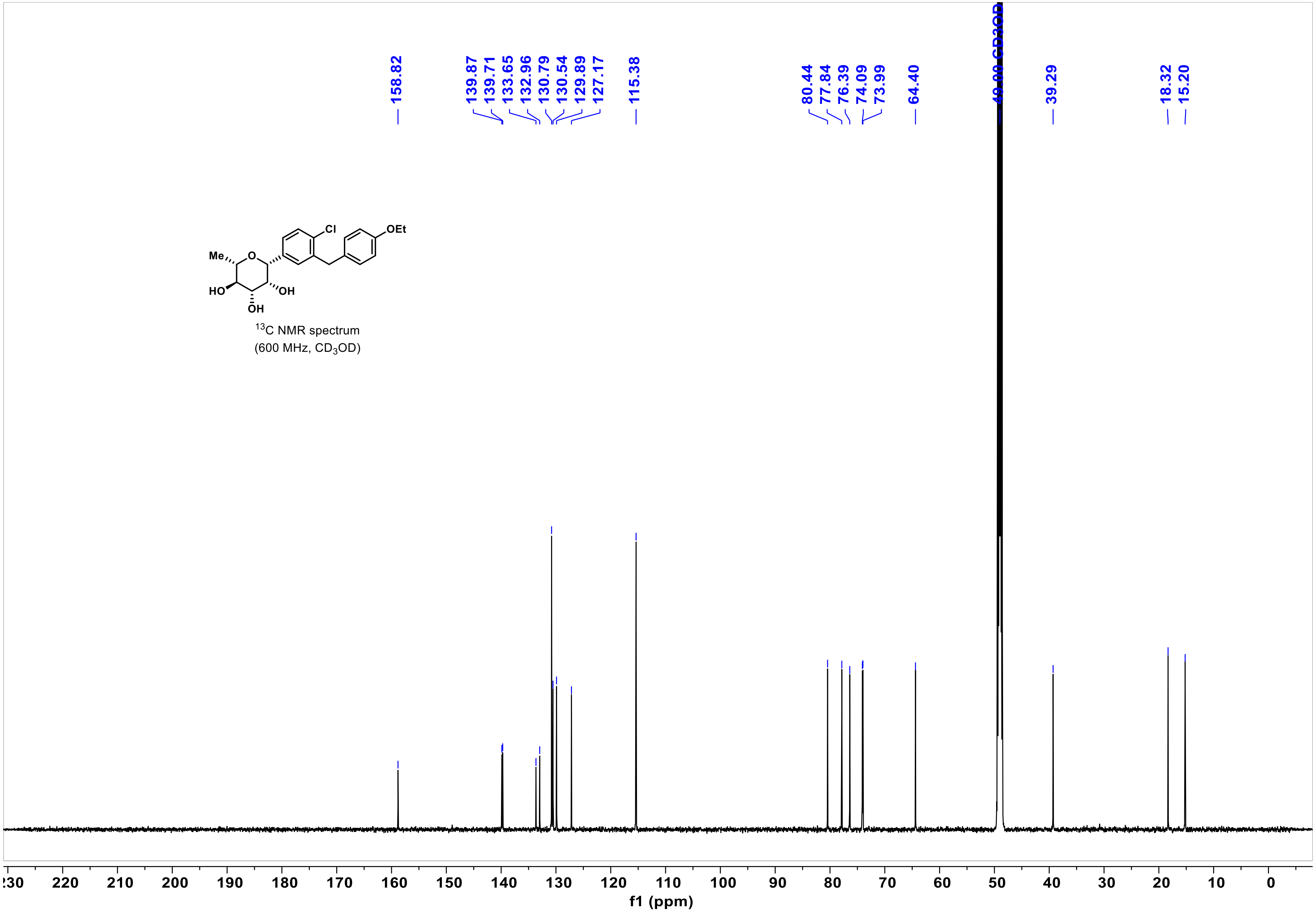

^{13}C NMR of compound **β-51**

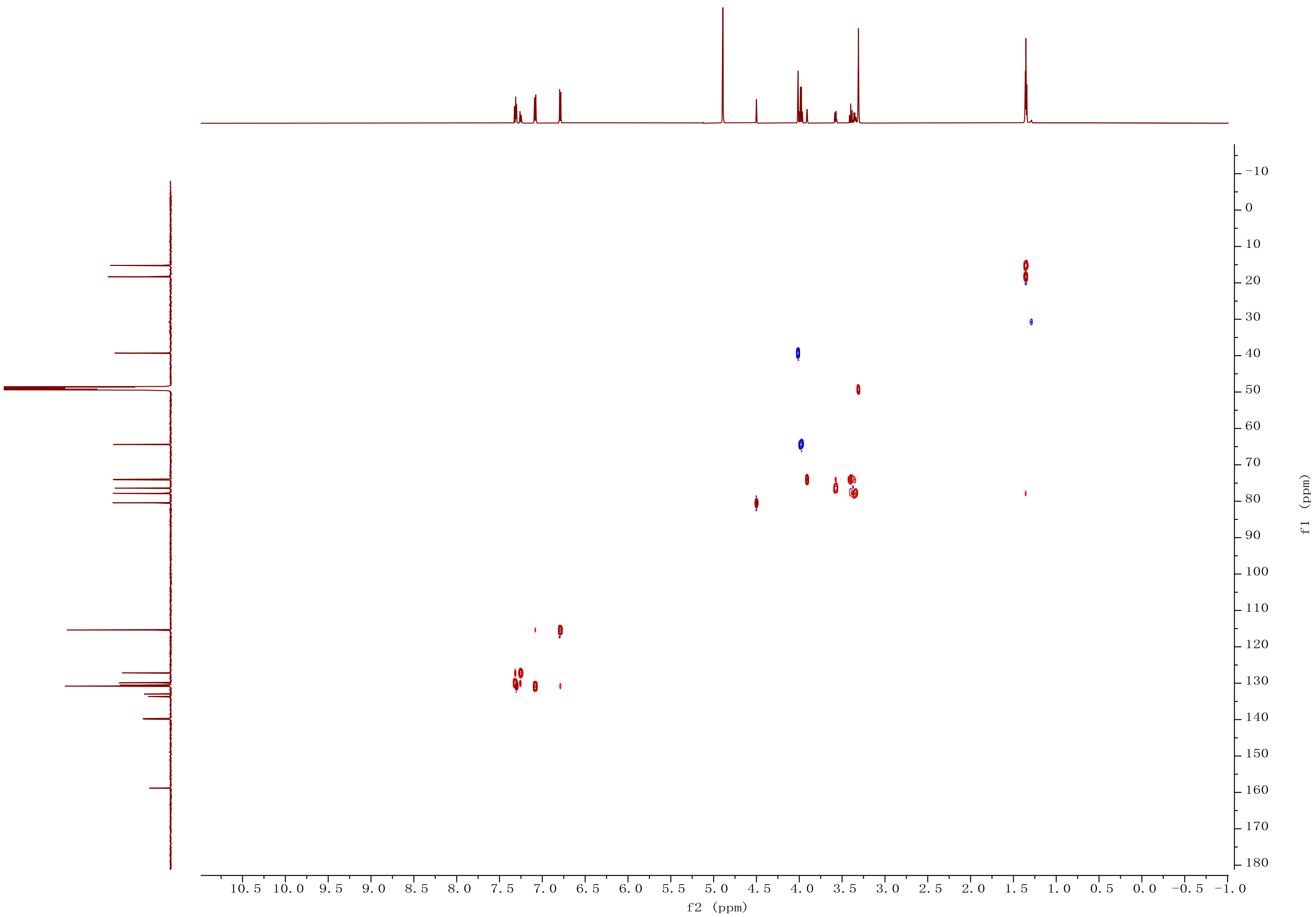

H-H HSQC NMR of compound **β-51**

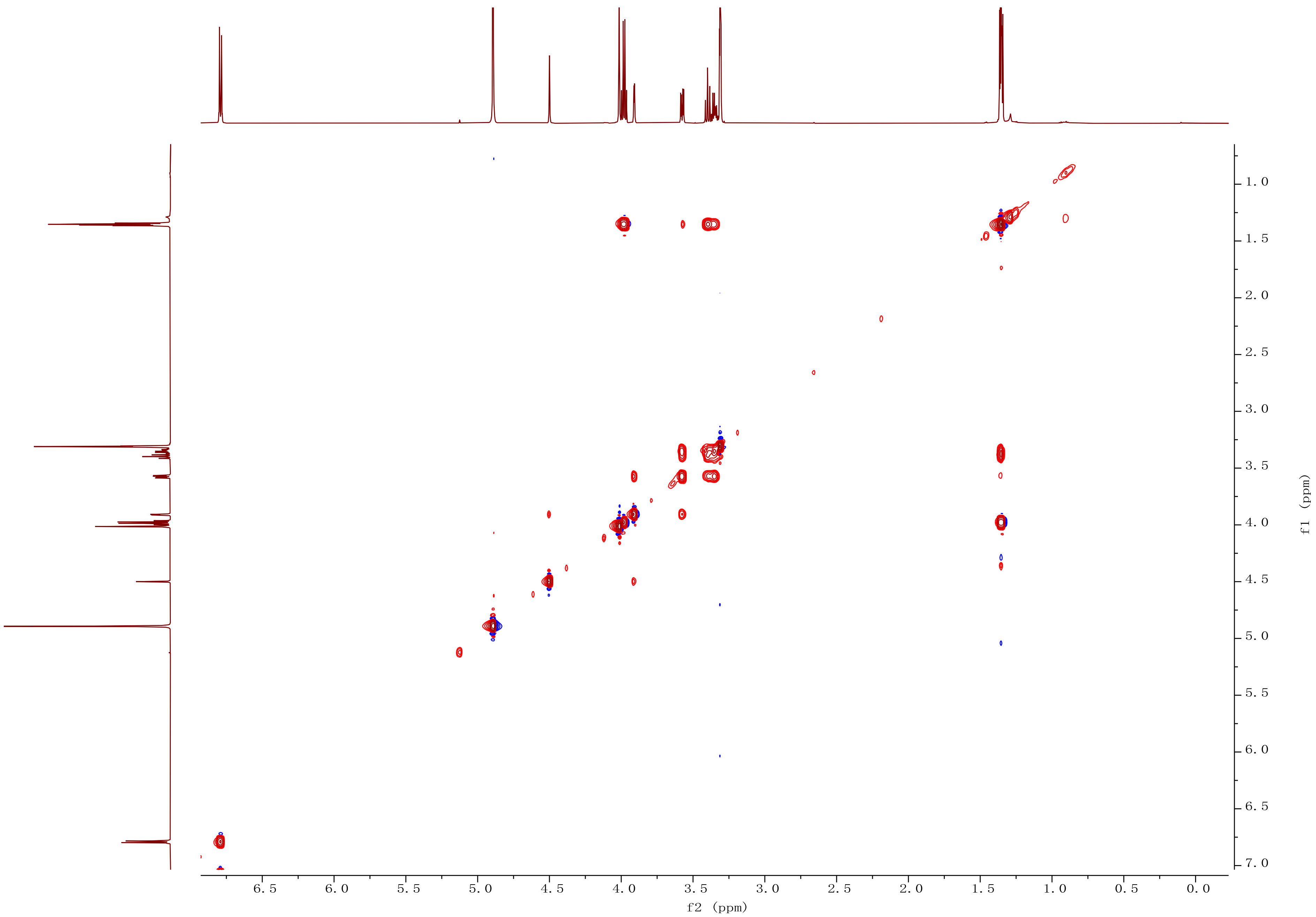

H-H COSY NMR of compound **β-51**

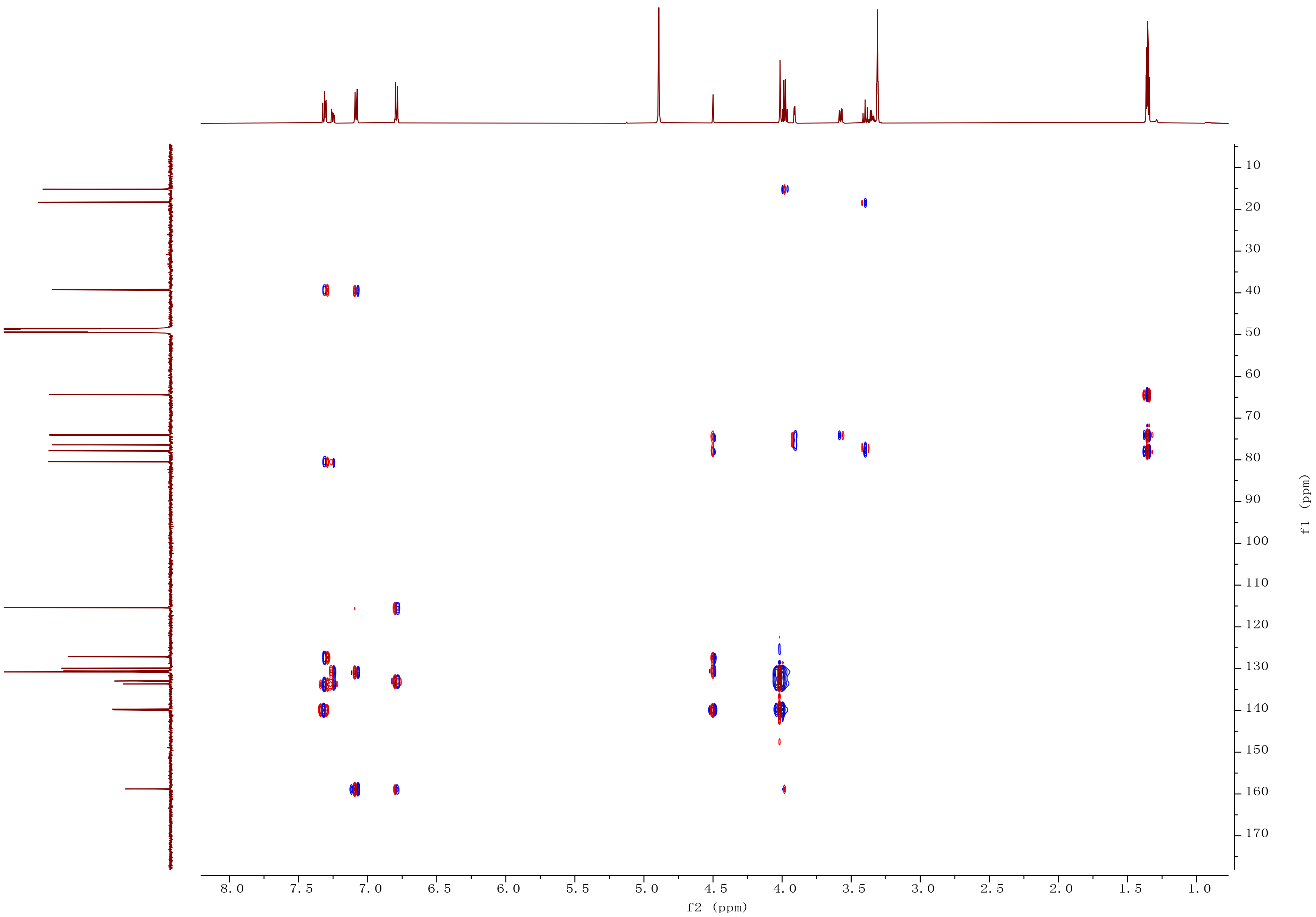

H-C HMBC NMR of compound **β-51**

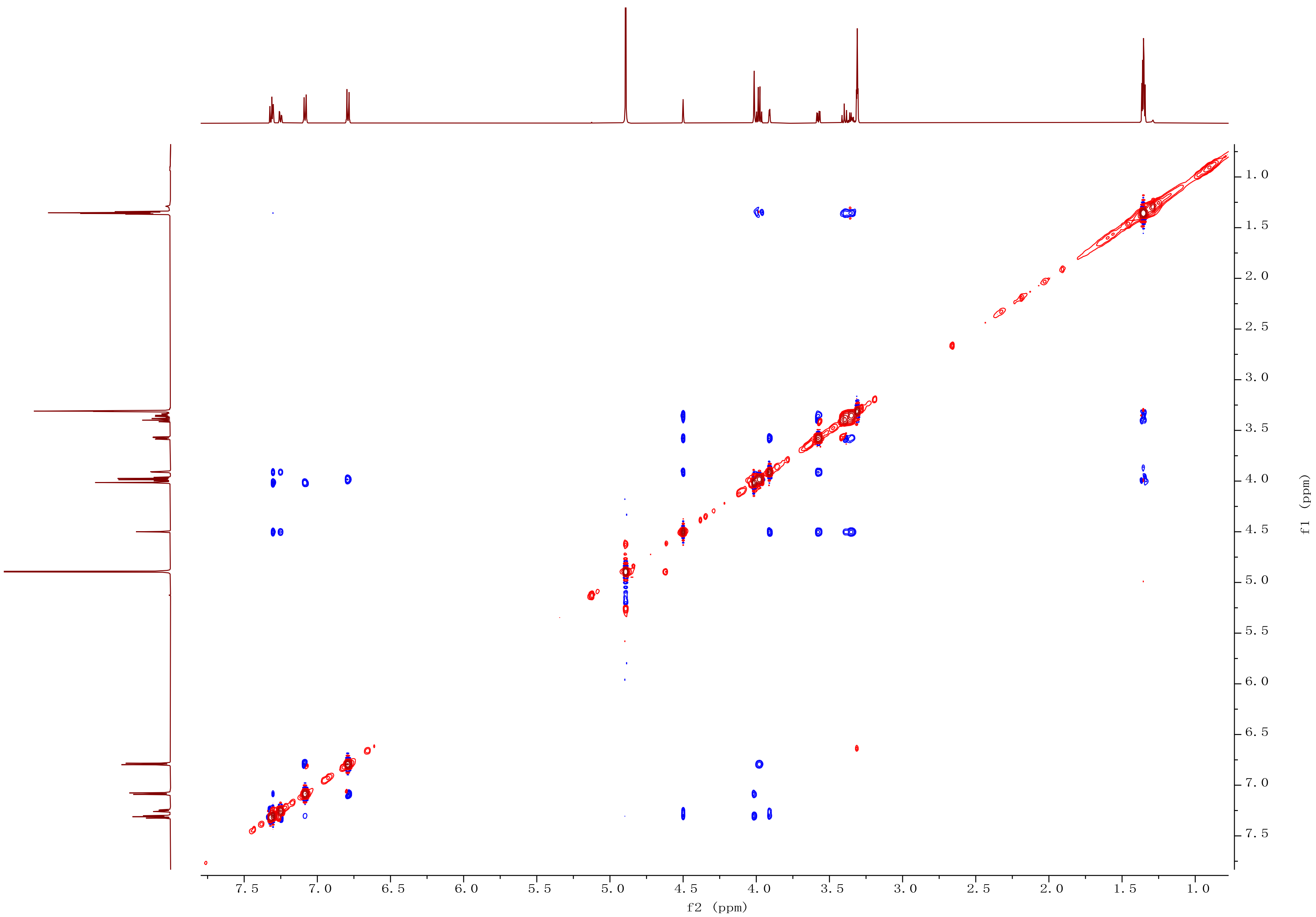

NOESY NMR of compound **β-51**

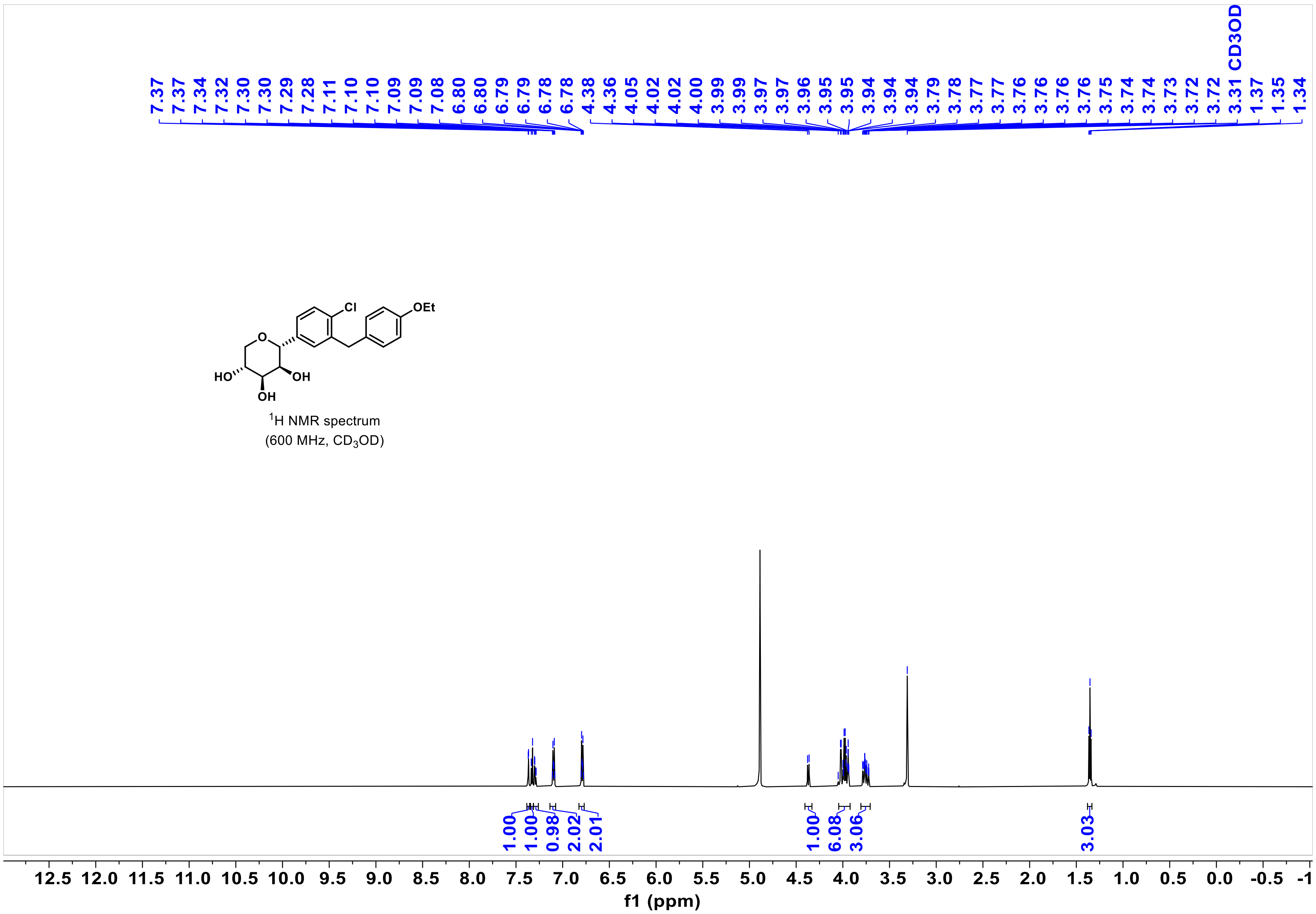

^{1}H NMR of compound **α–63**

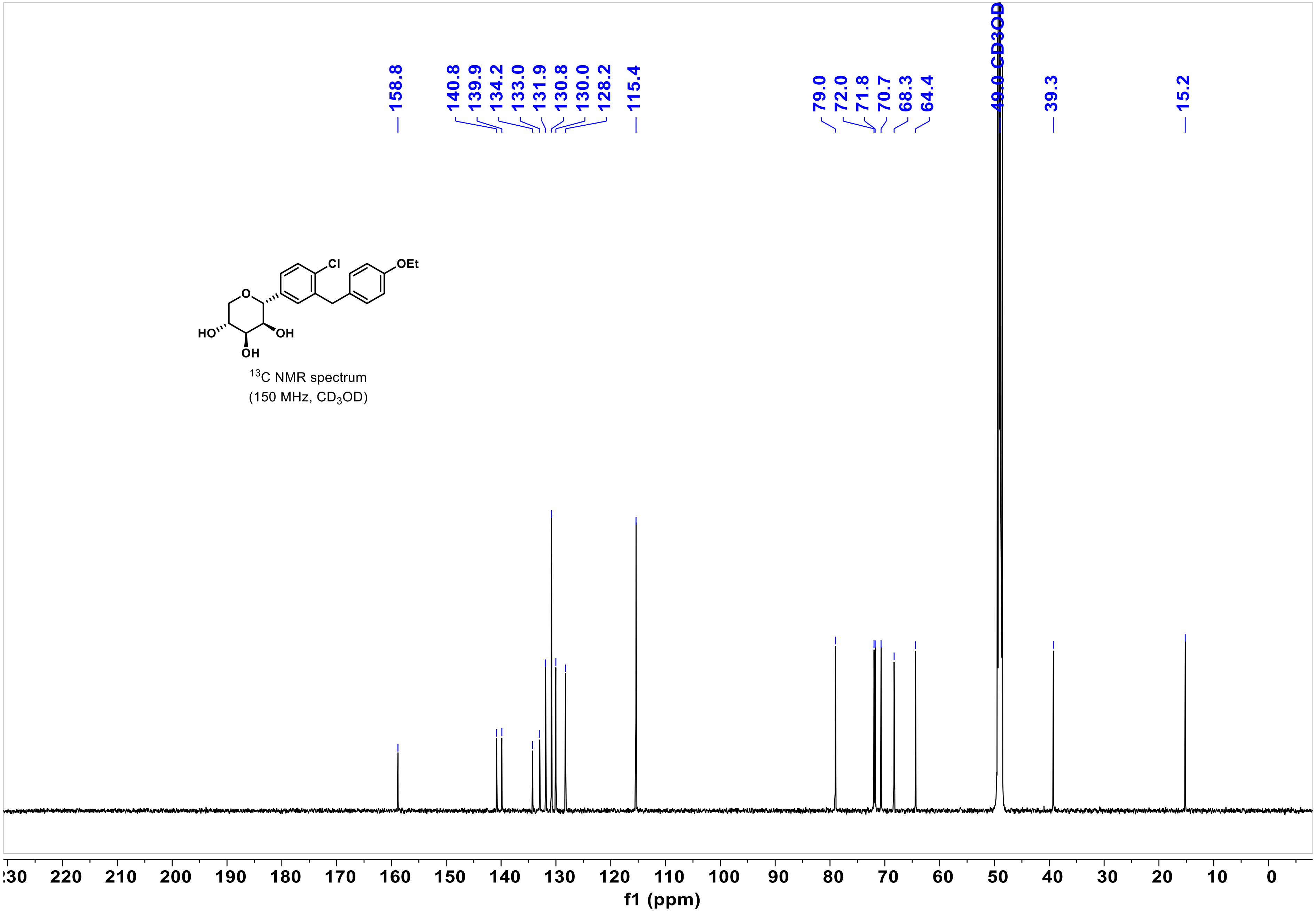

^{13}C NMR of compound **α–63**

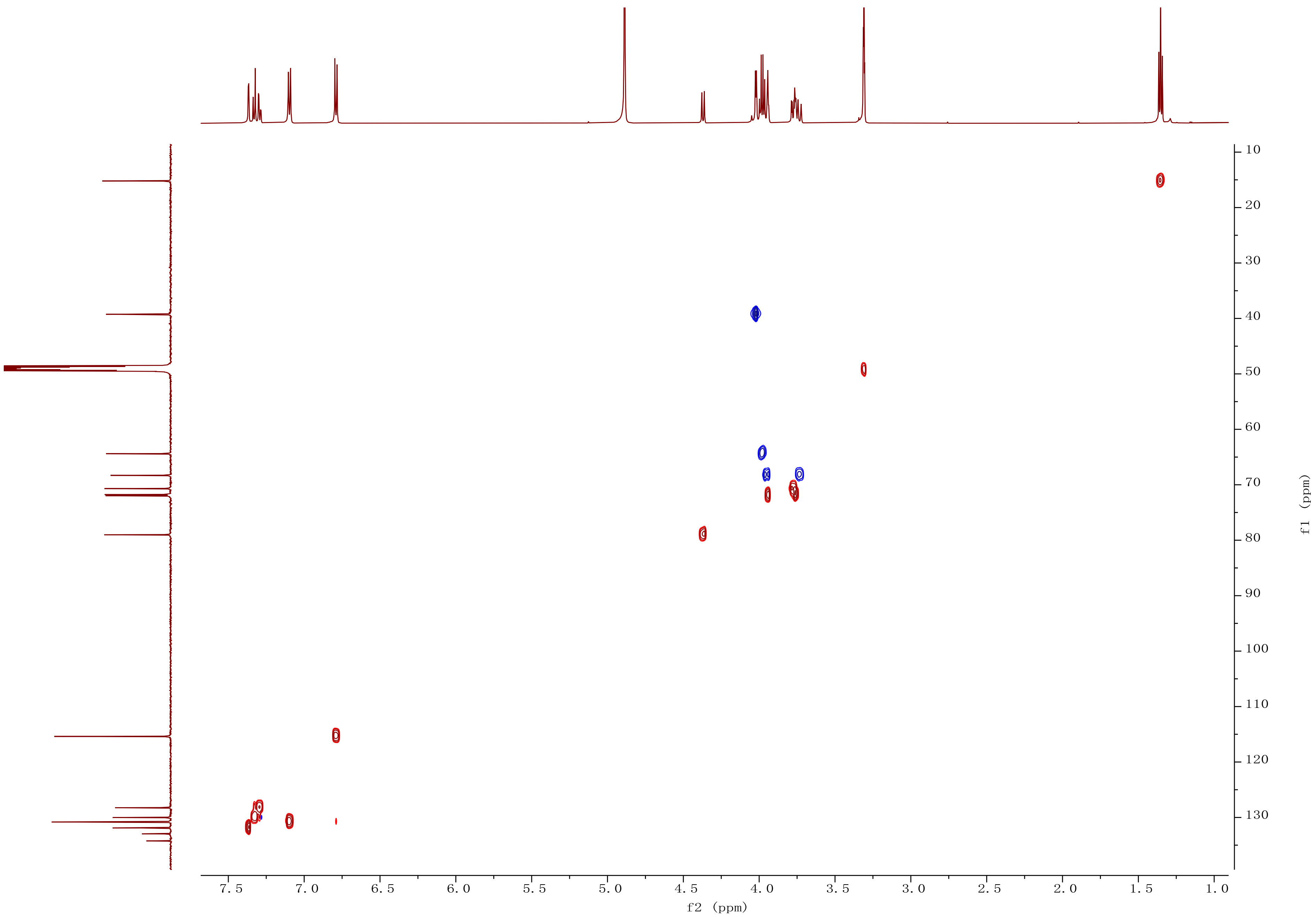

H-H HSQC NMR of compound **α–63**

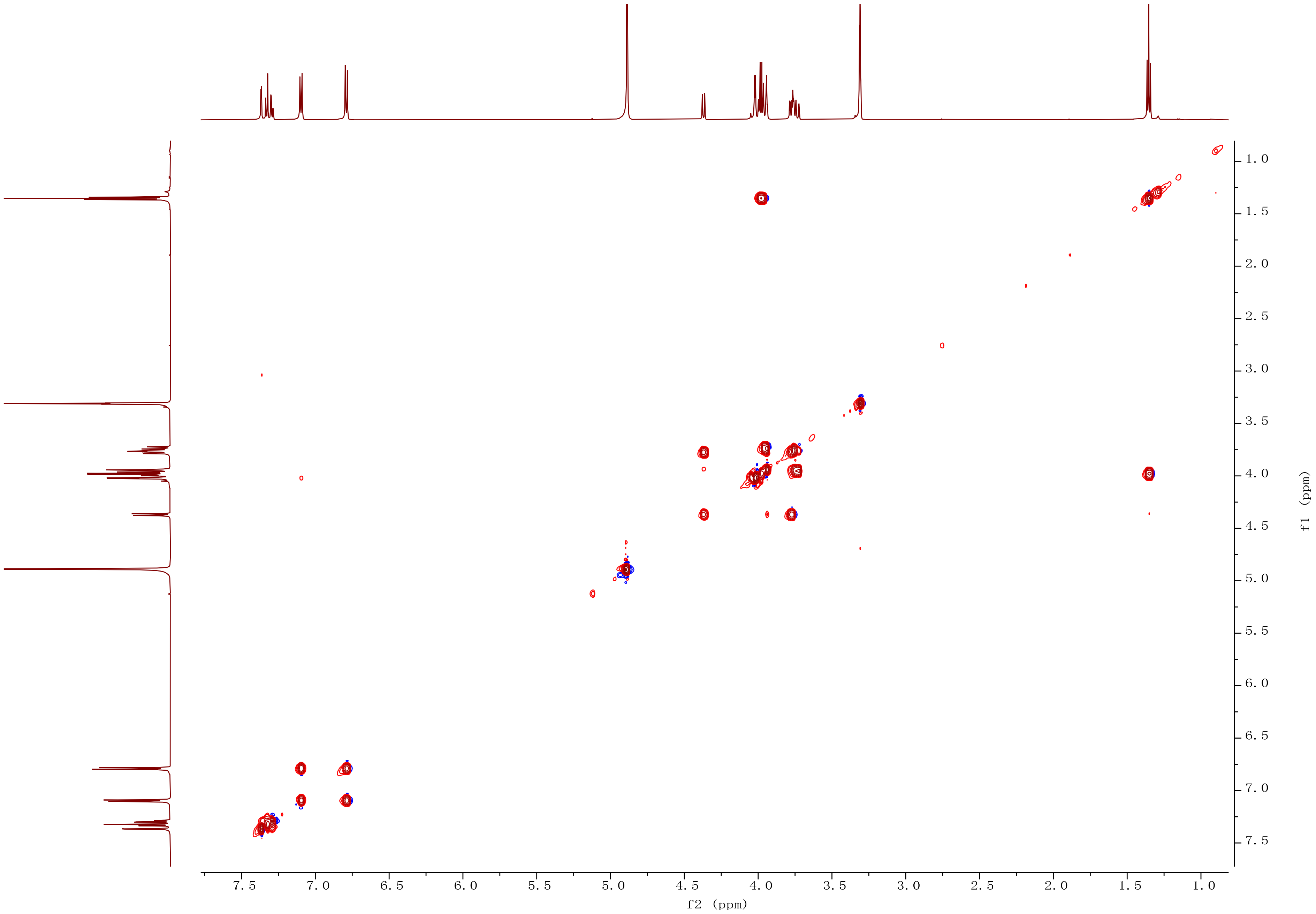

H-H COSY NMR of compound **α–63**

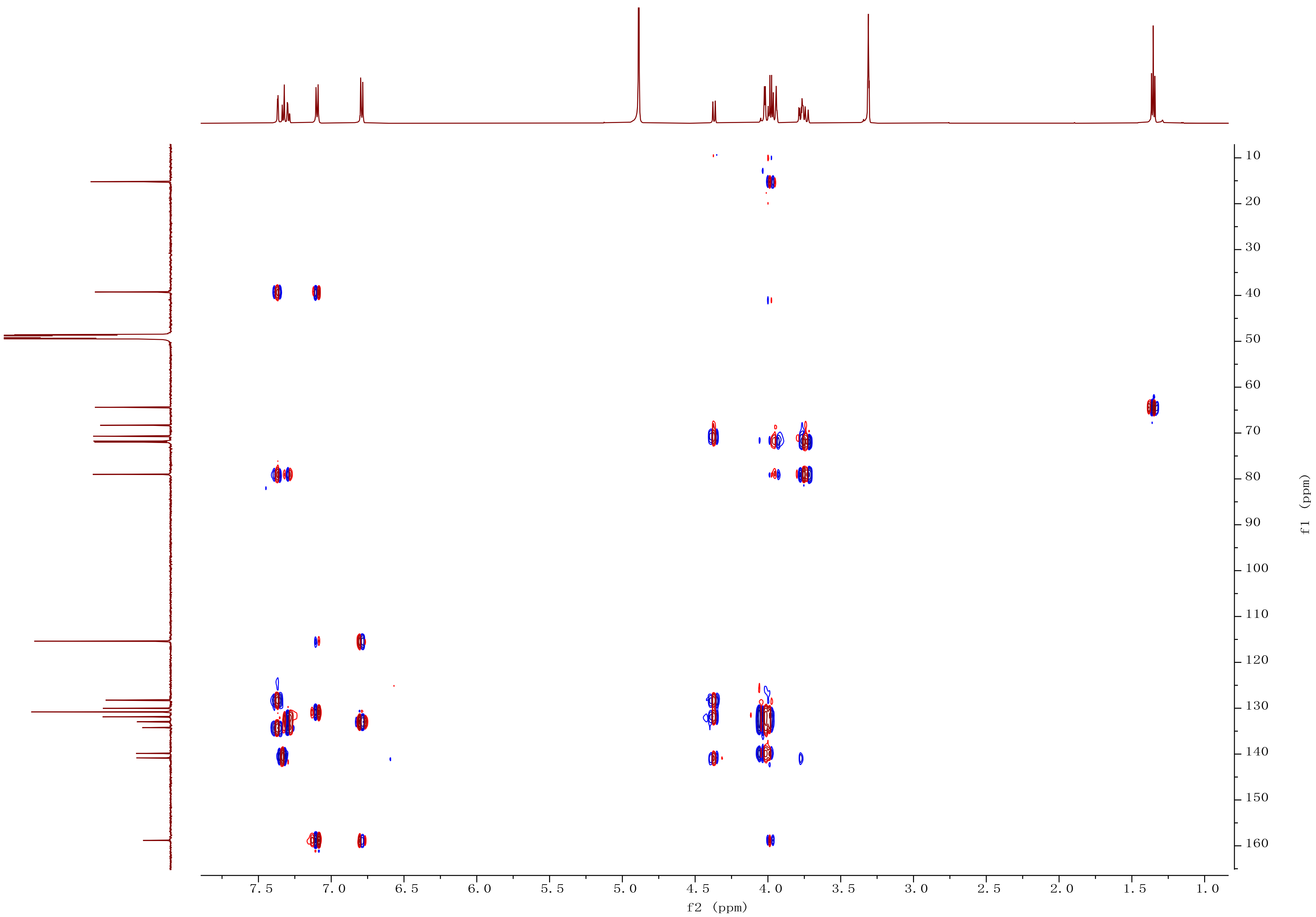

H-C HMBC NMR of compound **α–63**

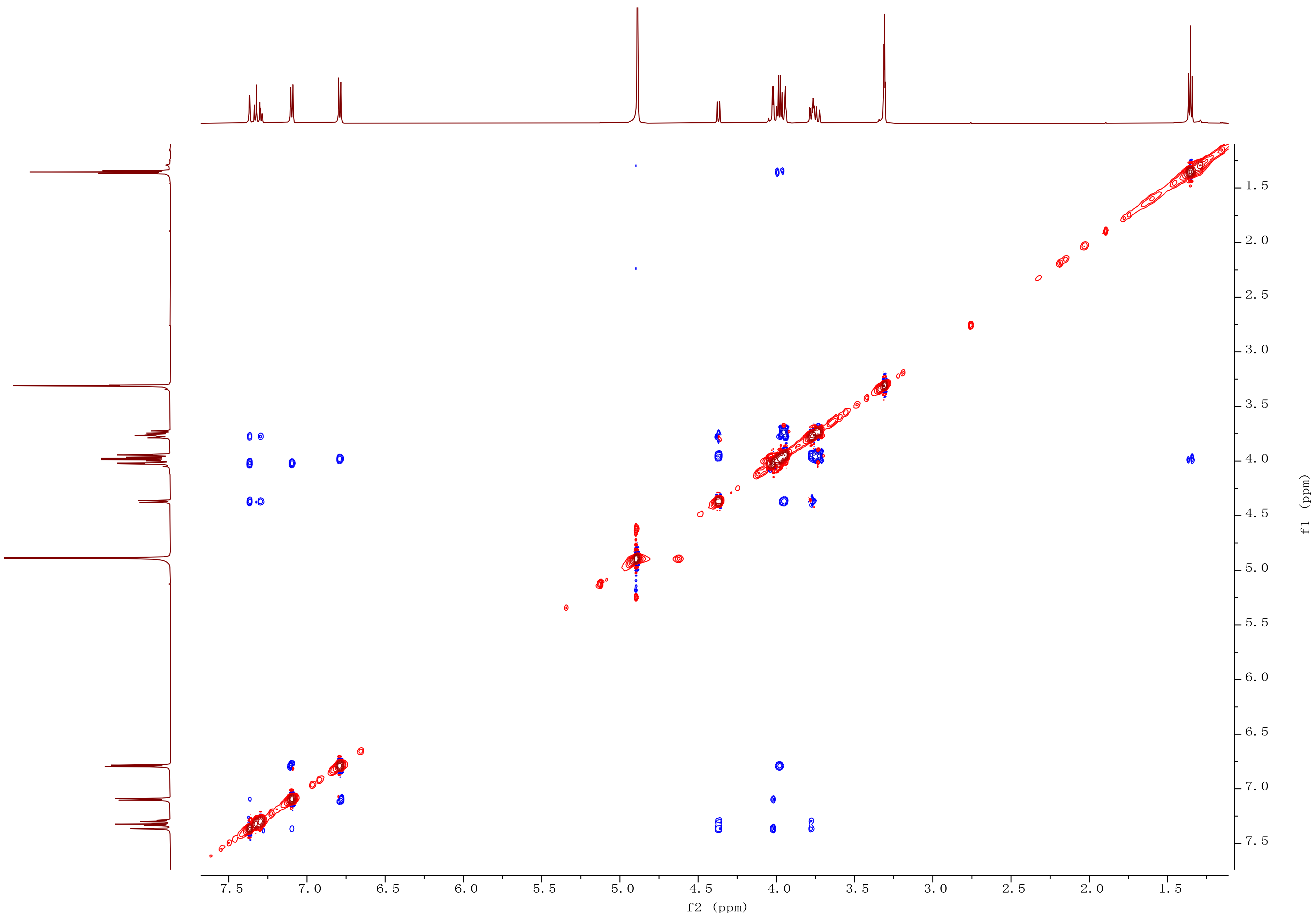

NOESY NMR of compound **α−63**

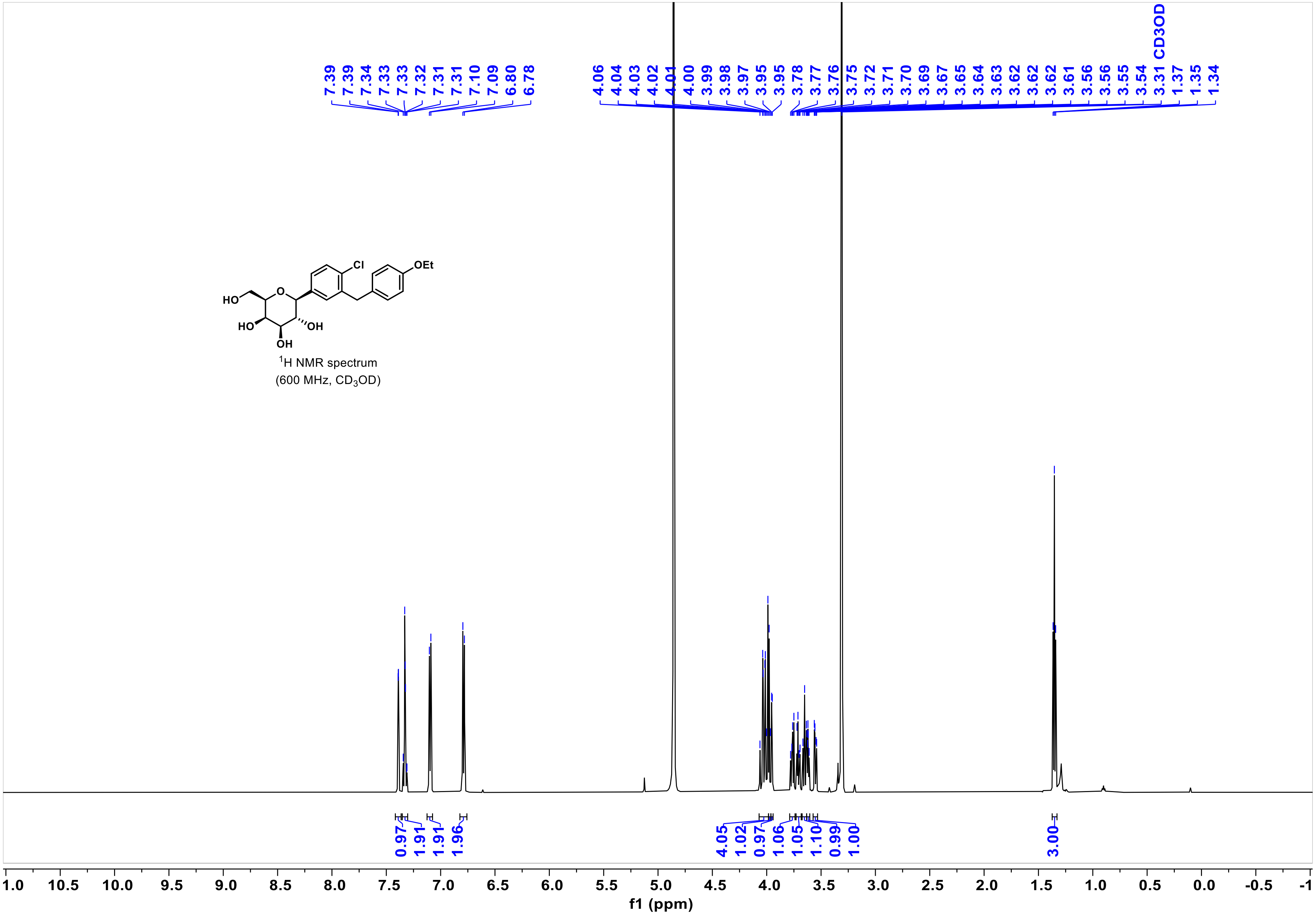

^{1}H NMR of compound **β–55**

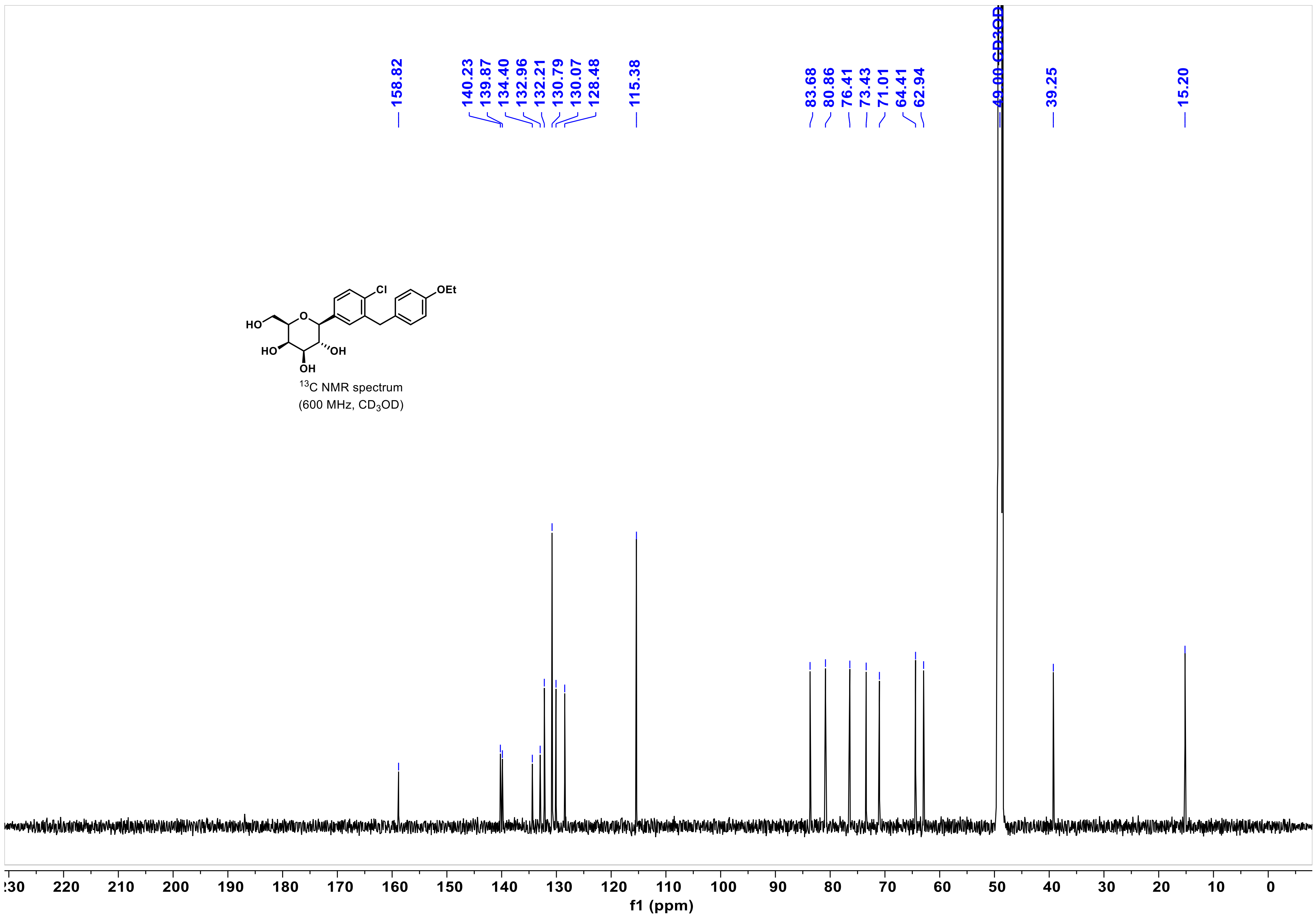

^{13}C NMR of compound **β–55**

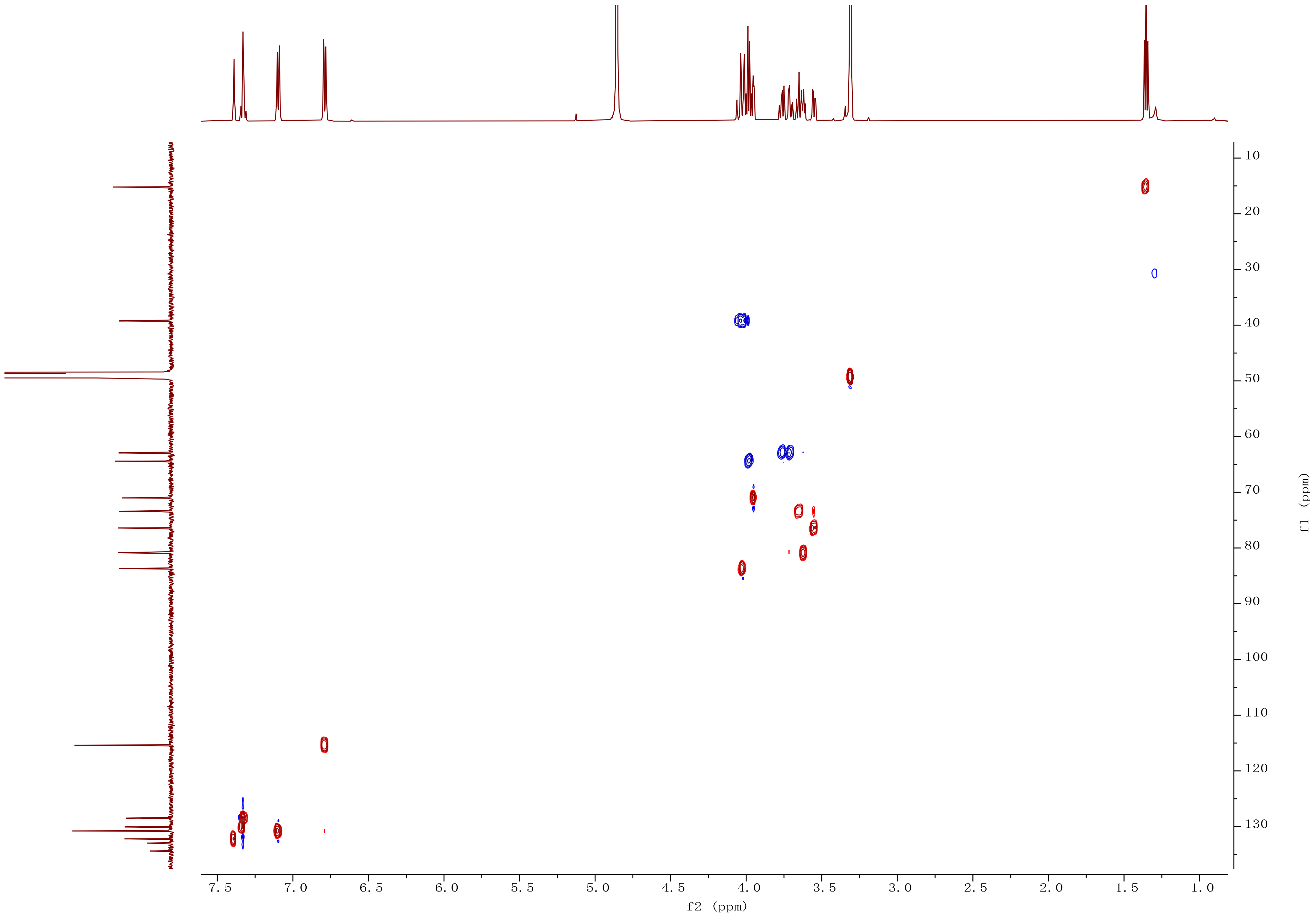

H-H HSQC NMR of compound **β–55**

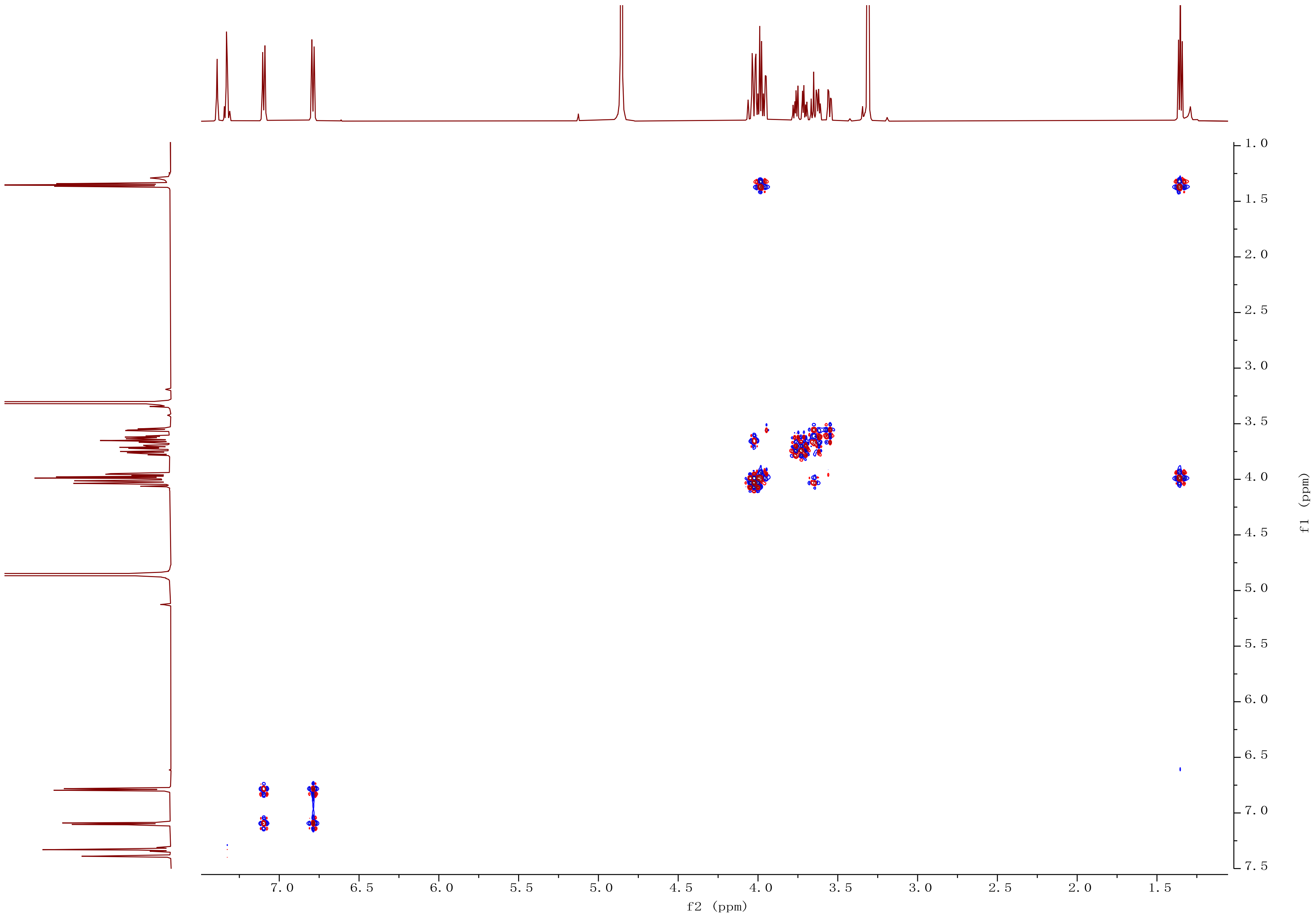

H-H COSY NMR of compound **β–55**

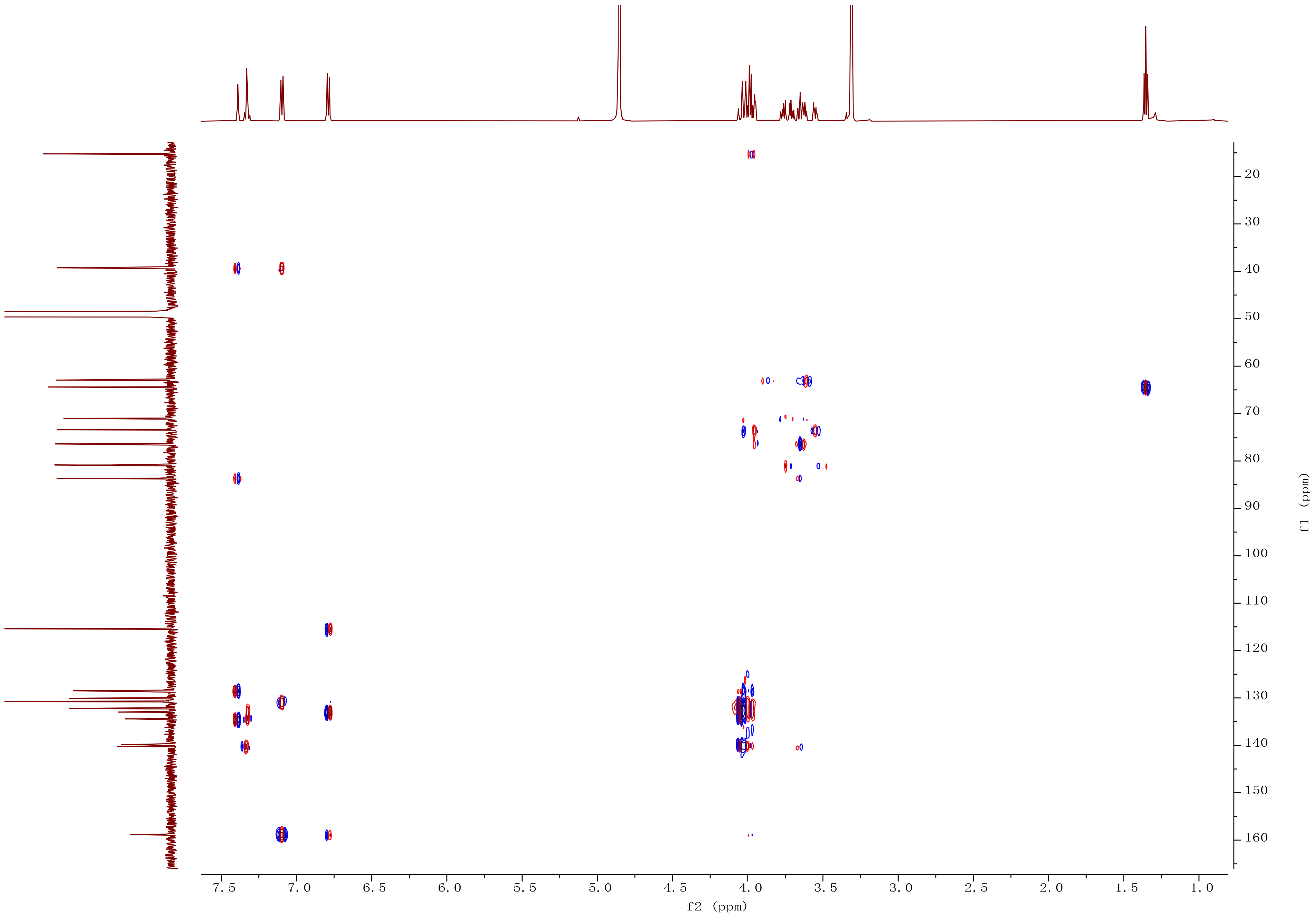

H-C HMBC NMR of compound **β–55**

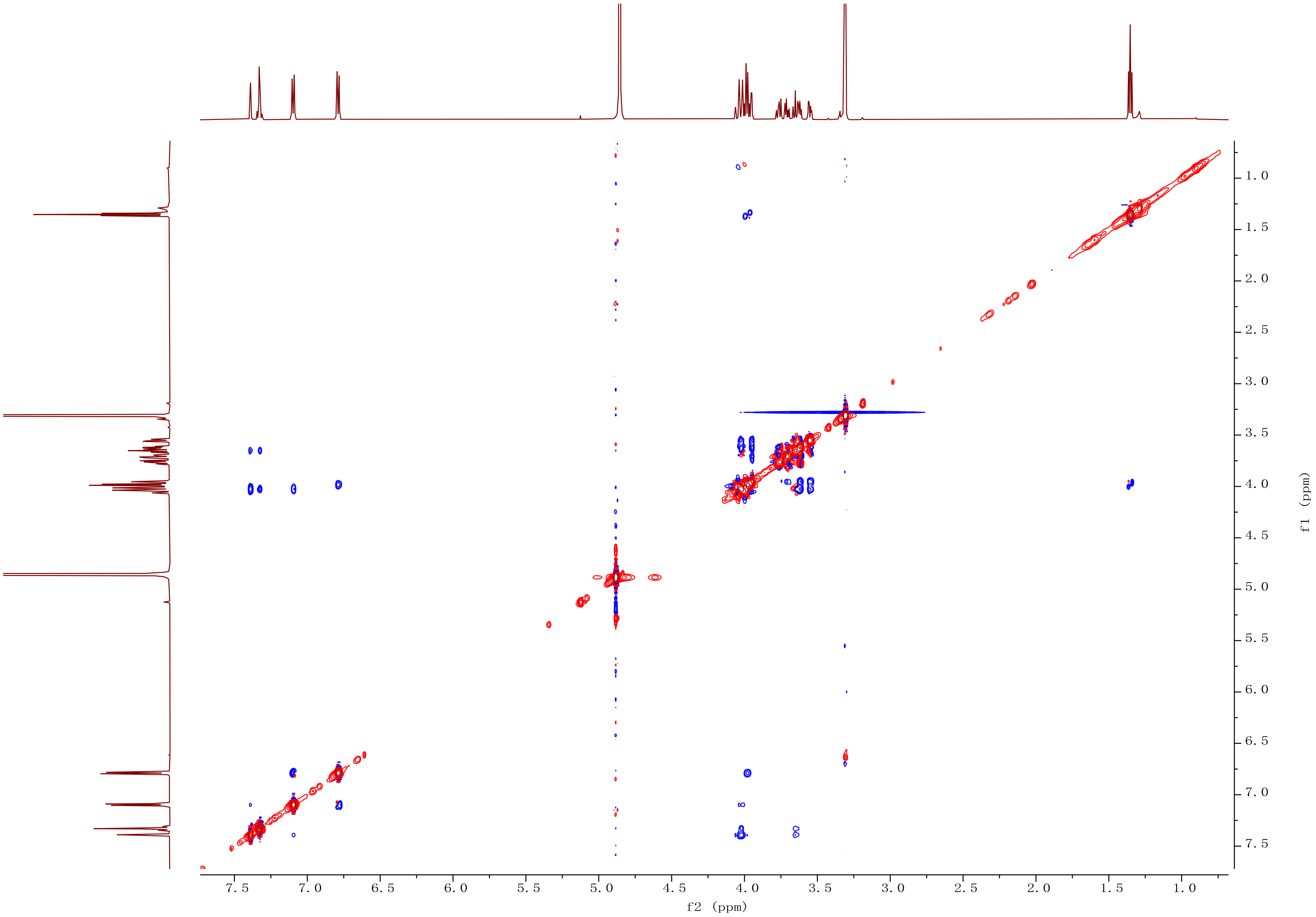

NOESY NMR of compound **β–55**

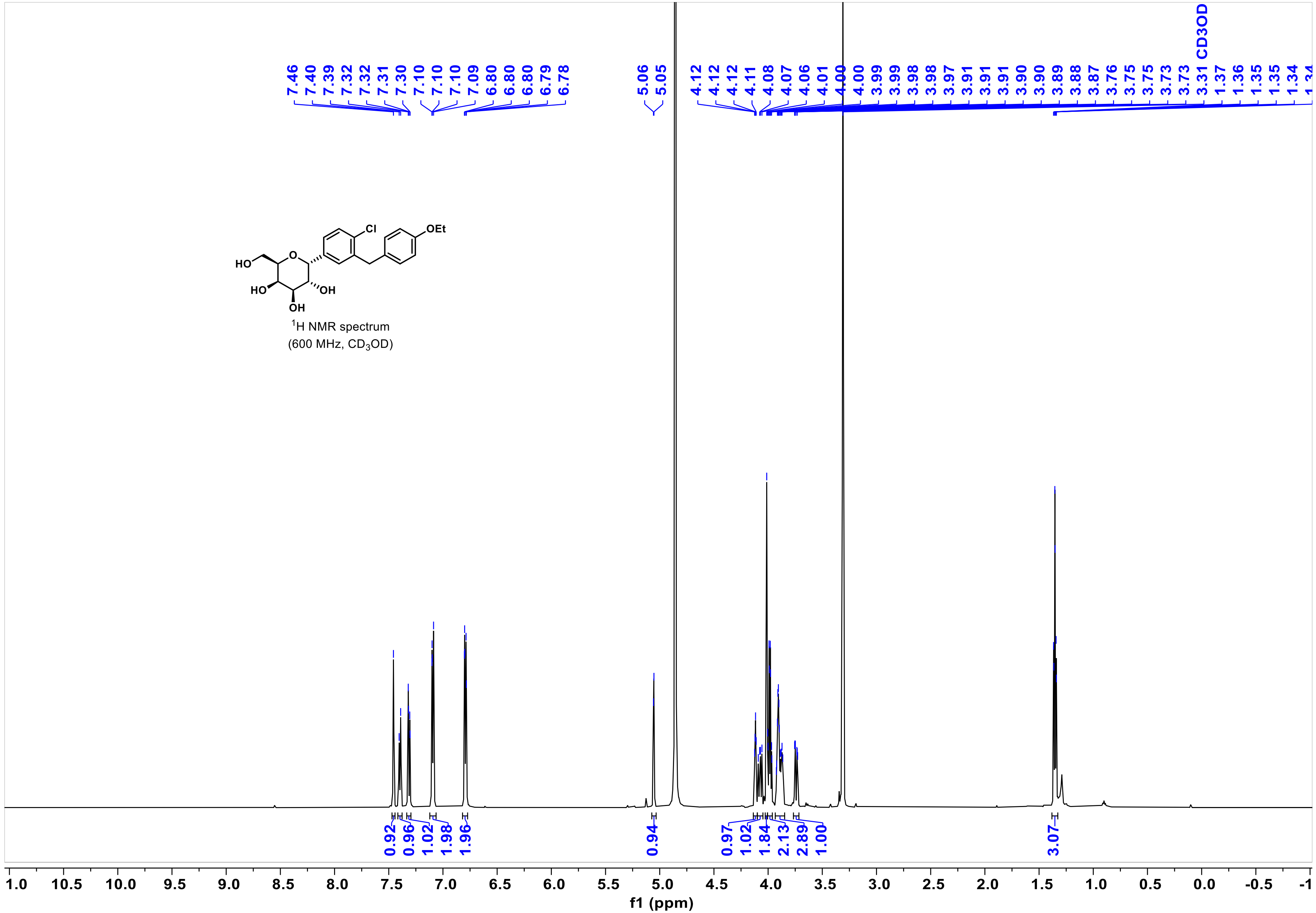

^{1}H NMR of compound **α–55**

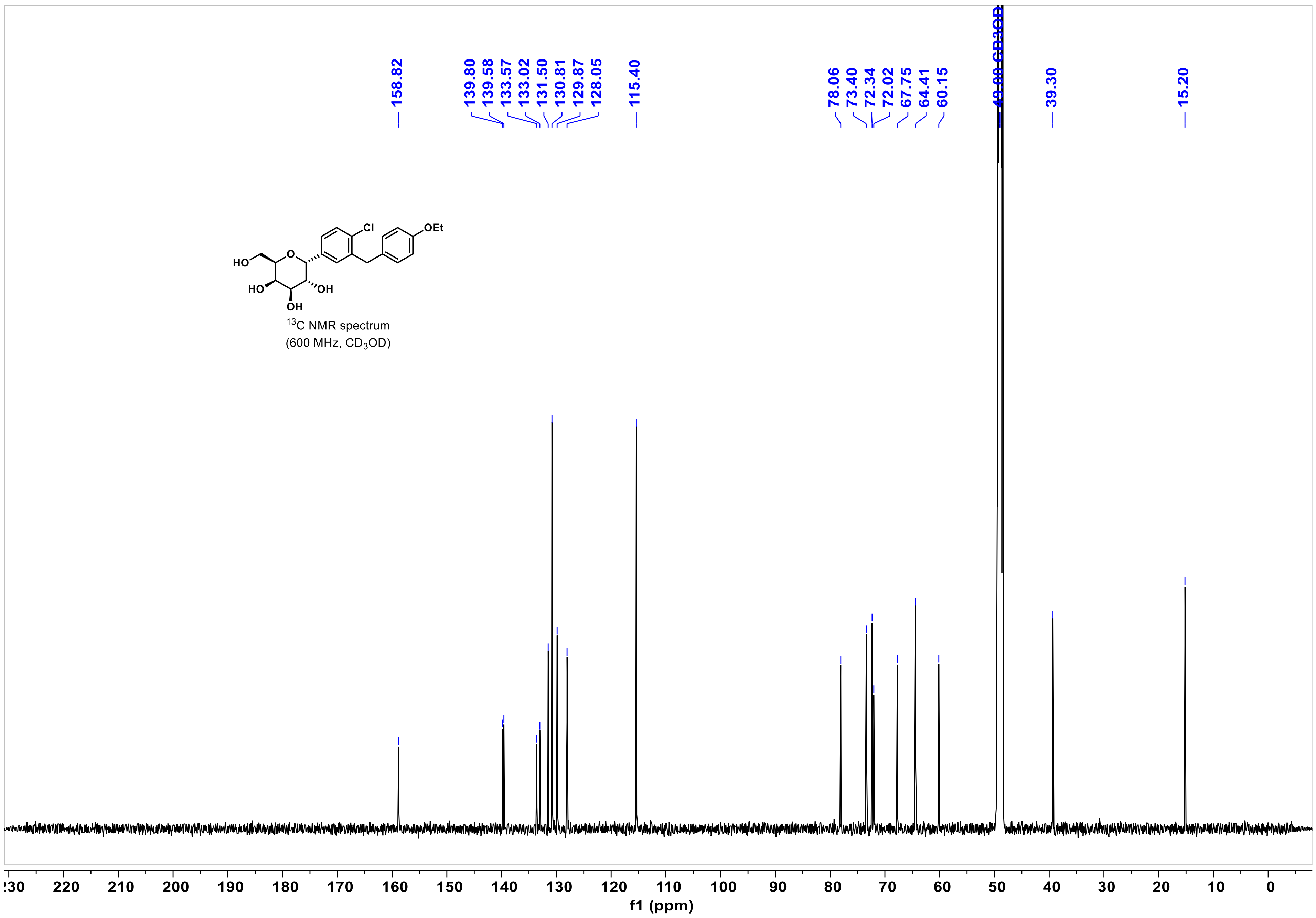

^{13}C NMR of compound **α–55**

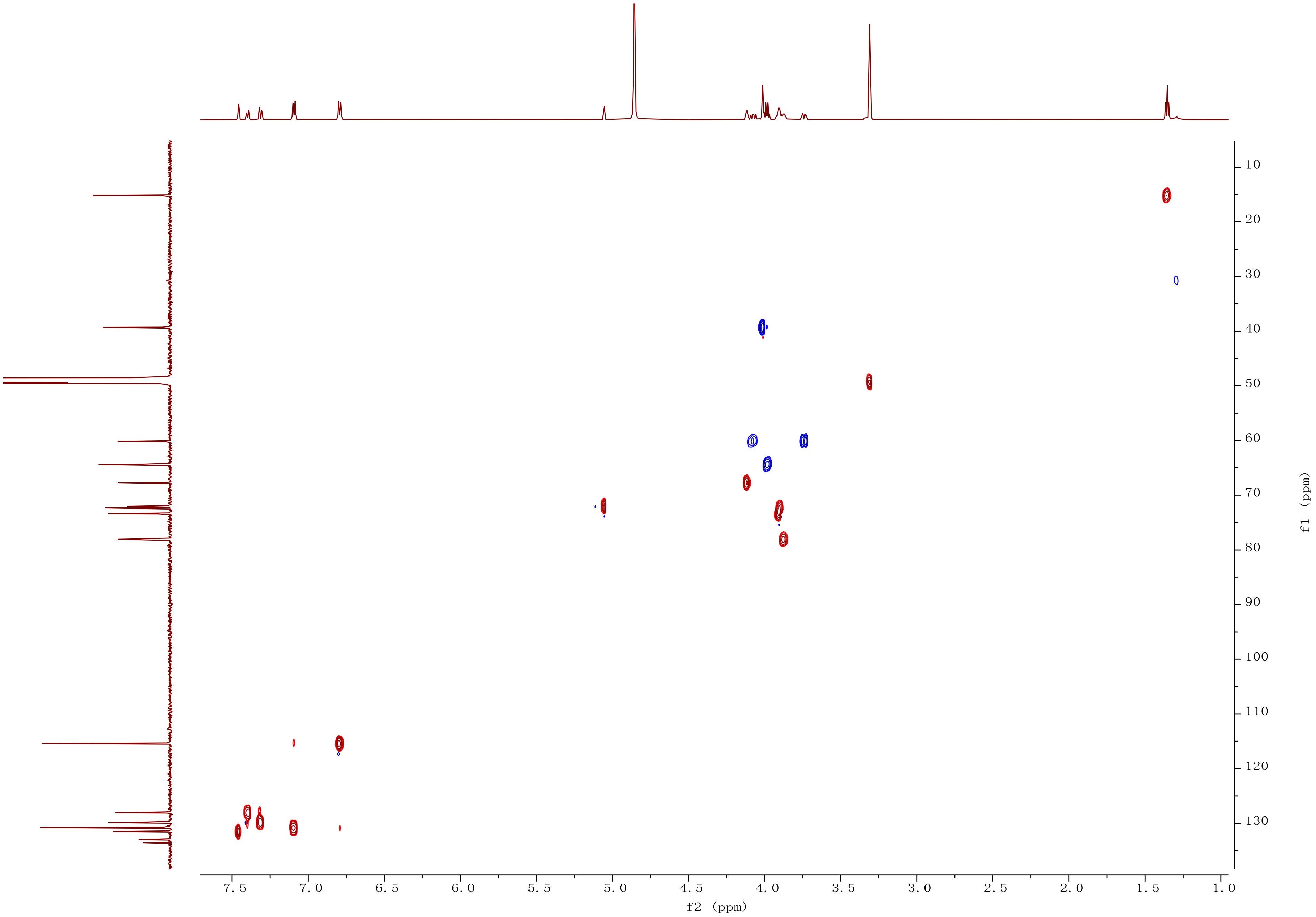

H-H HSQC NMR of compound **α–55**

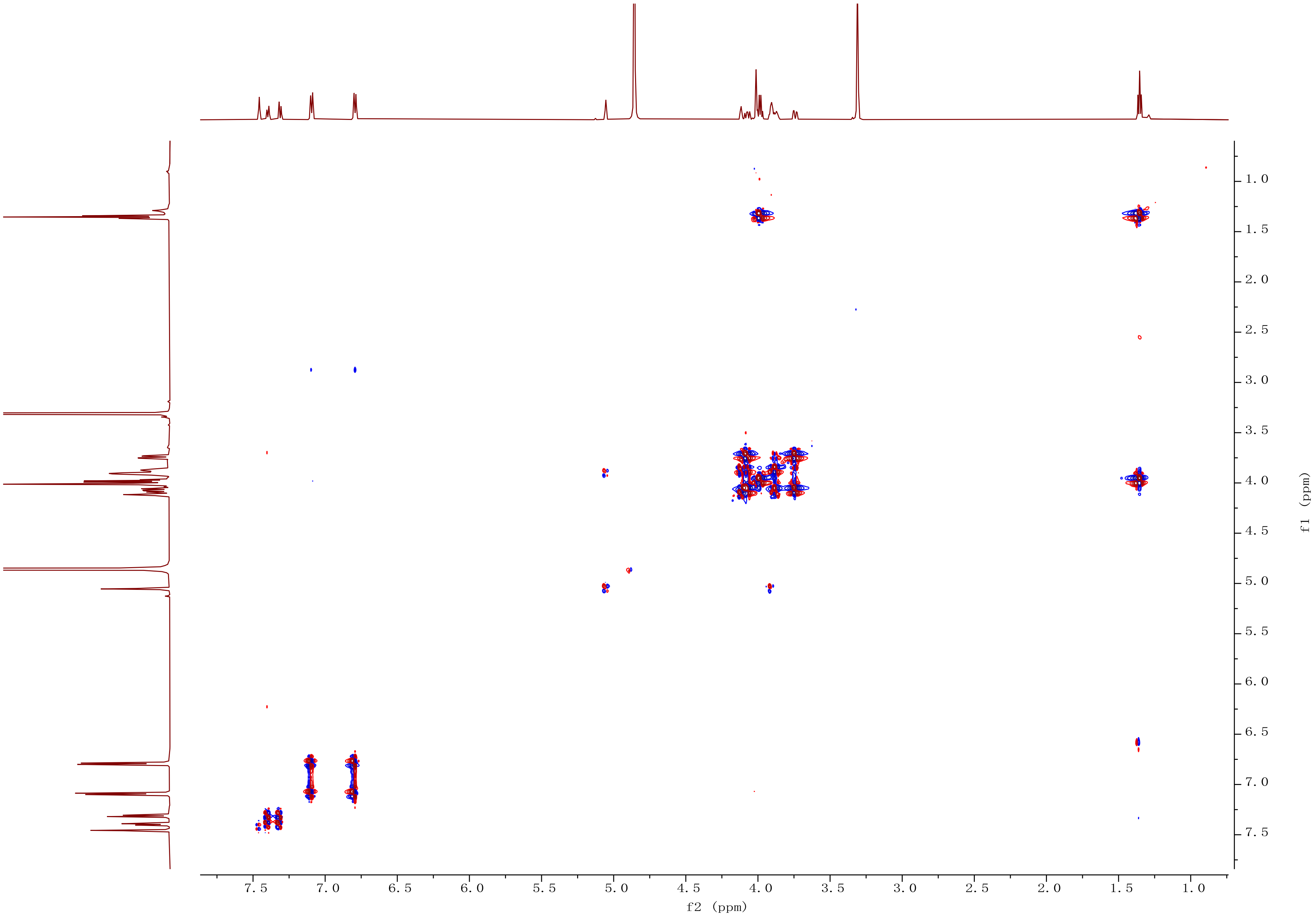

H-H COSY NMR of compound **α−55**

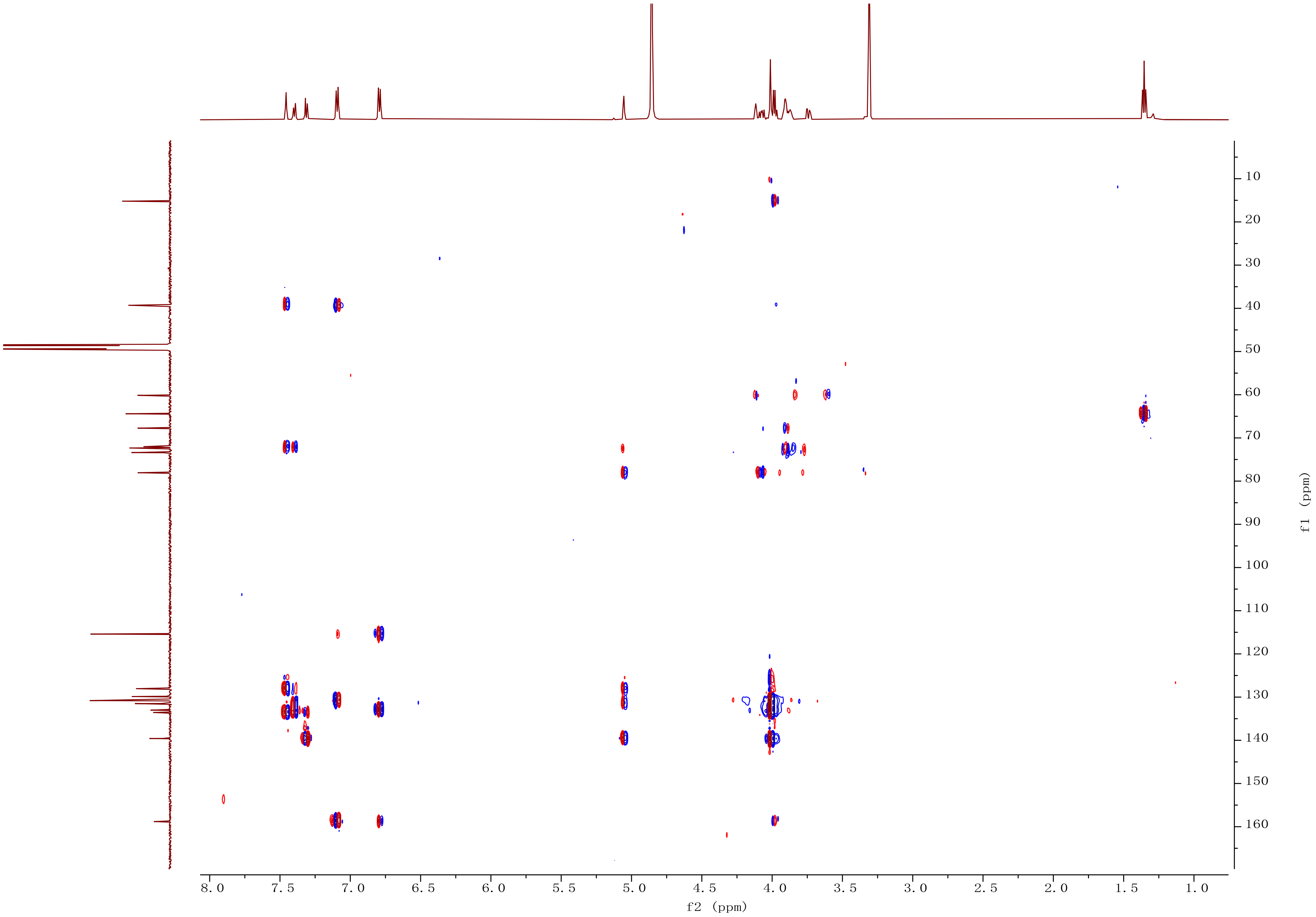

H-C HMBC NMR of compound **α–55**

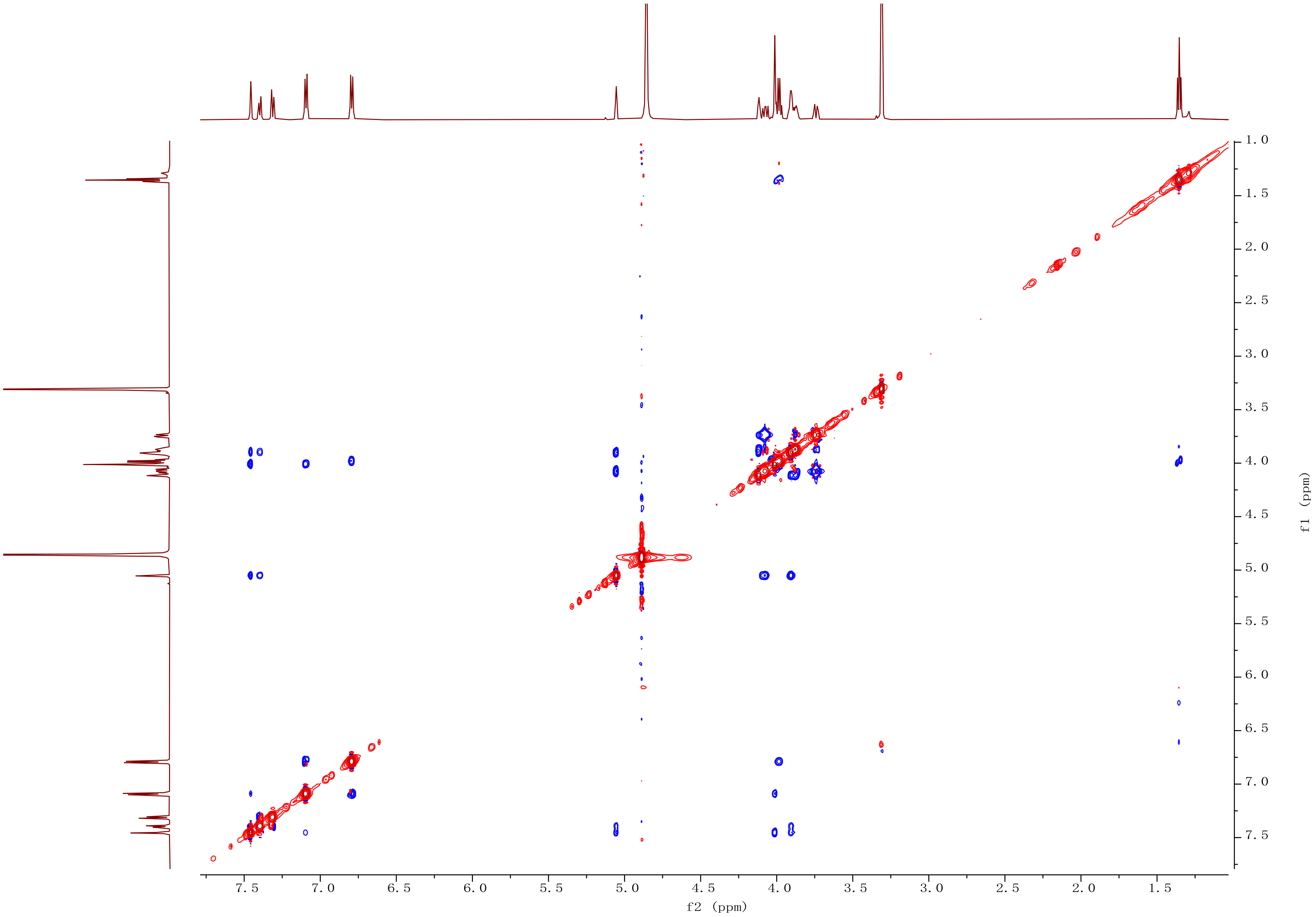

NOESY NMR of compound **α–55**

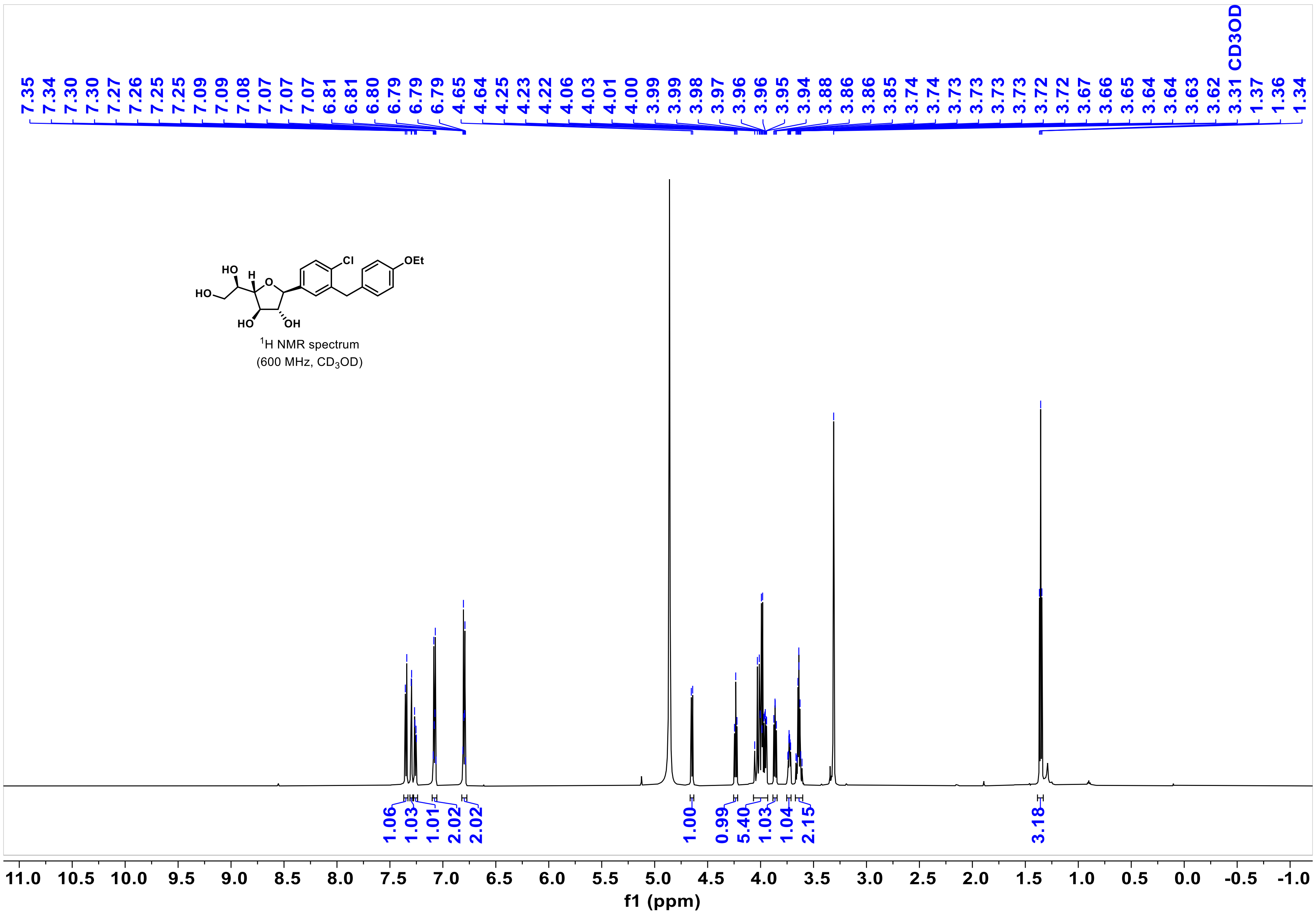

^{1}H NMR of compound **α–56**

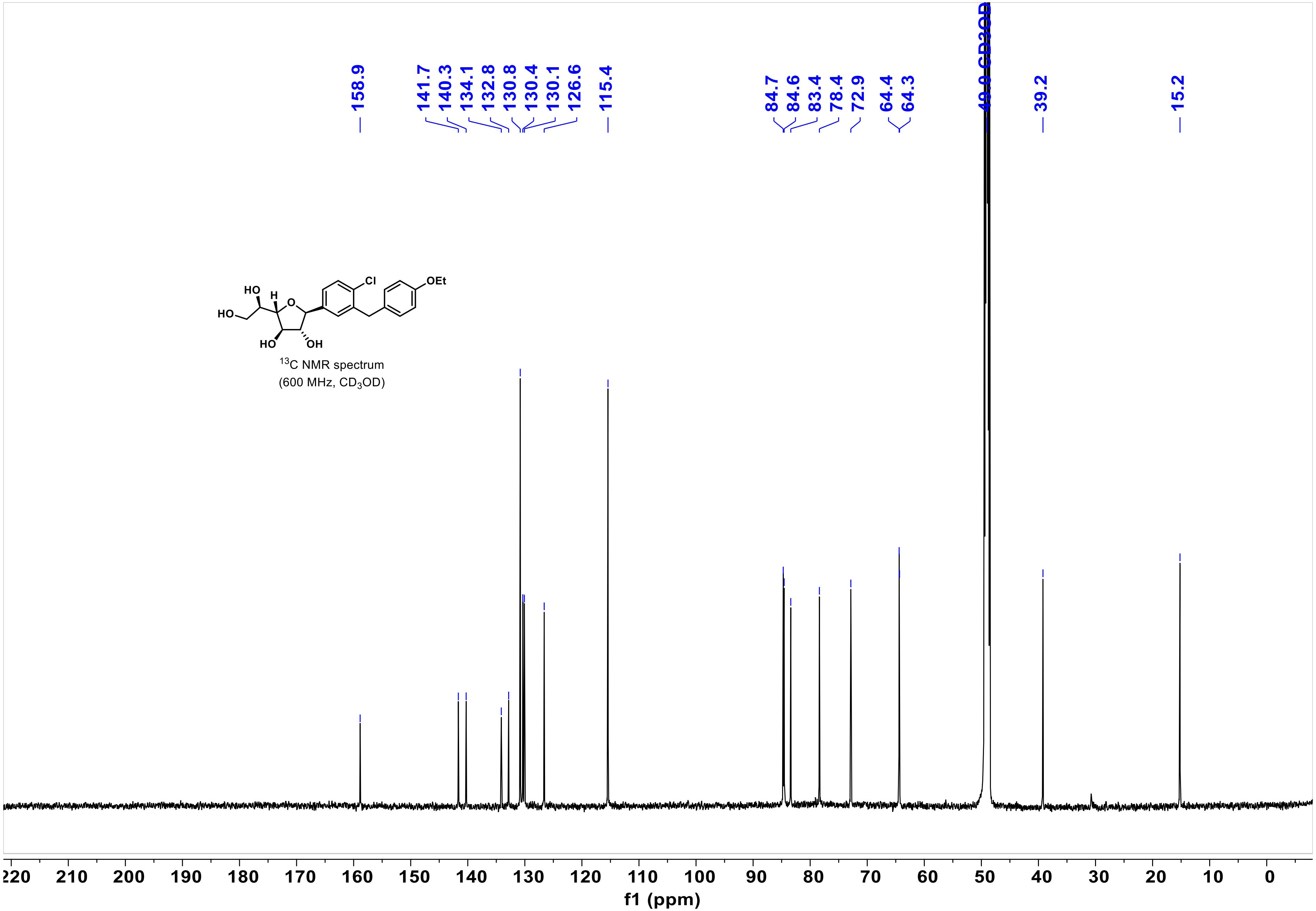

^{13}C NMR of compound **α–56**

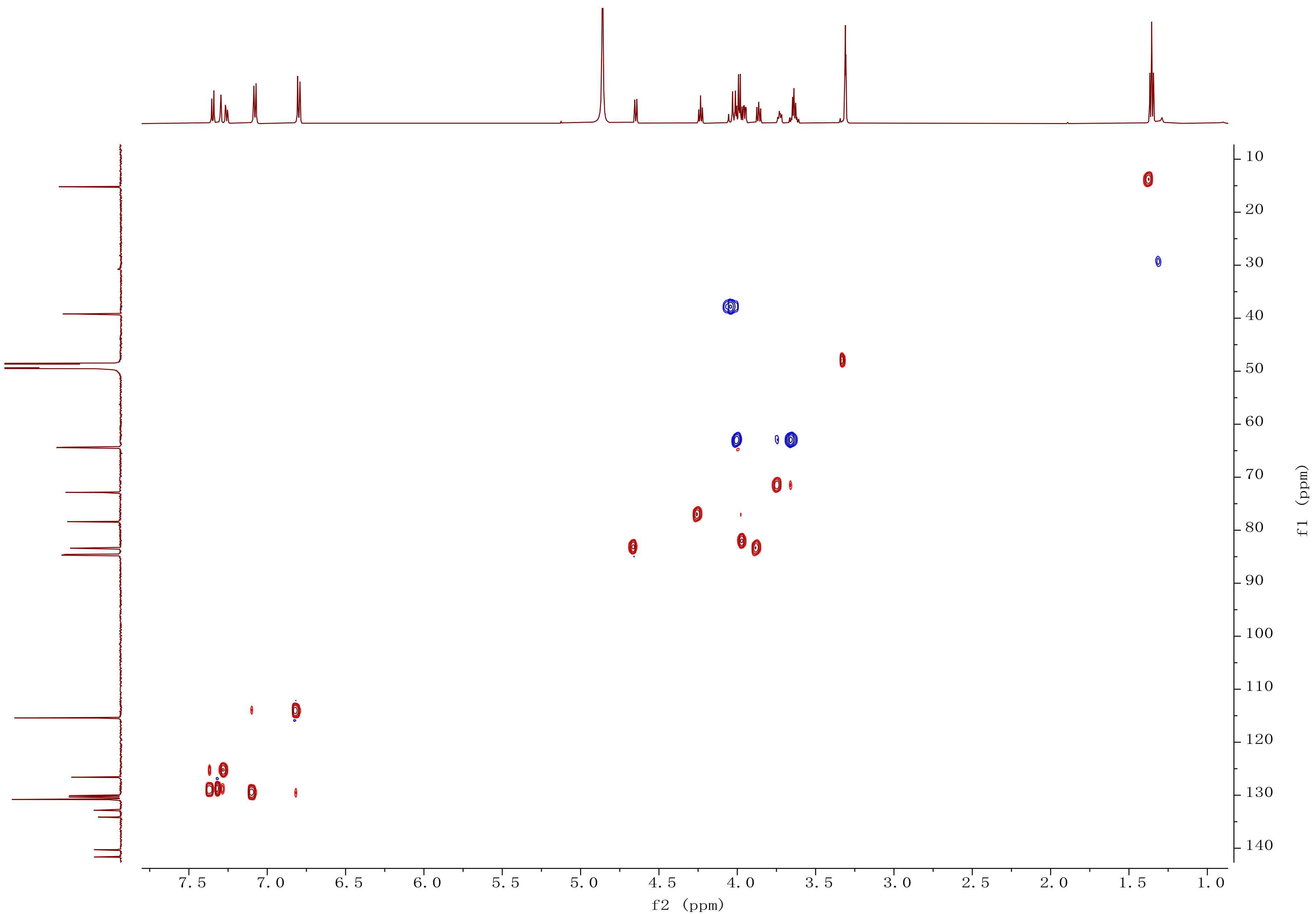

H-H HSQC NMR of compound **α–56**

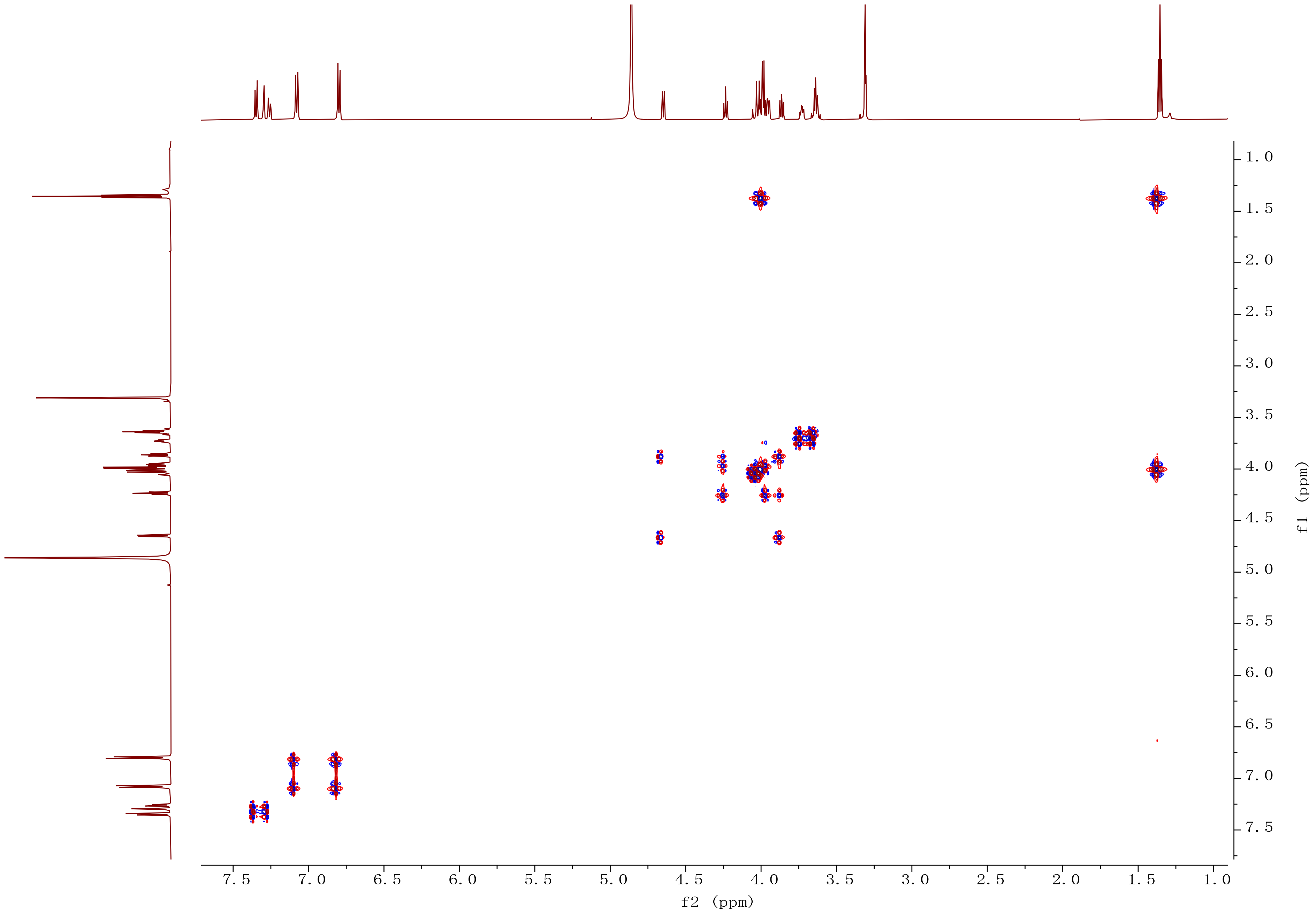

H-H COSY NMR of compound **α–56**

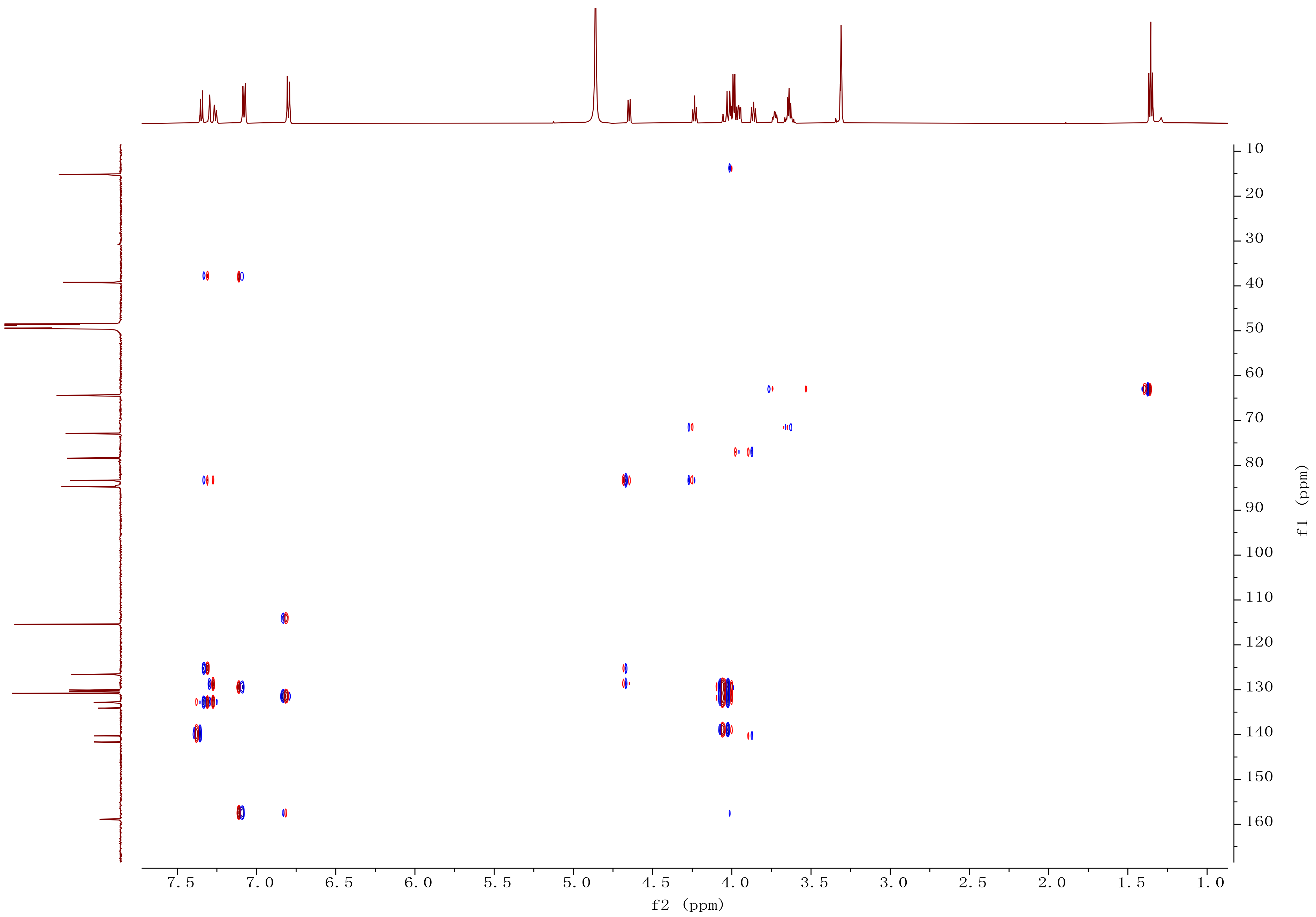

H-C HMBC NMR of compound **α–56**

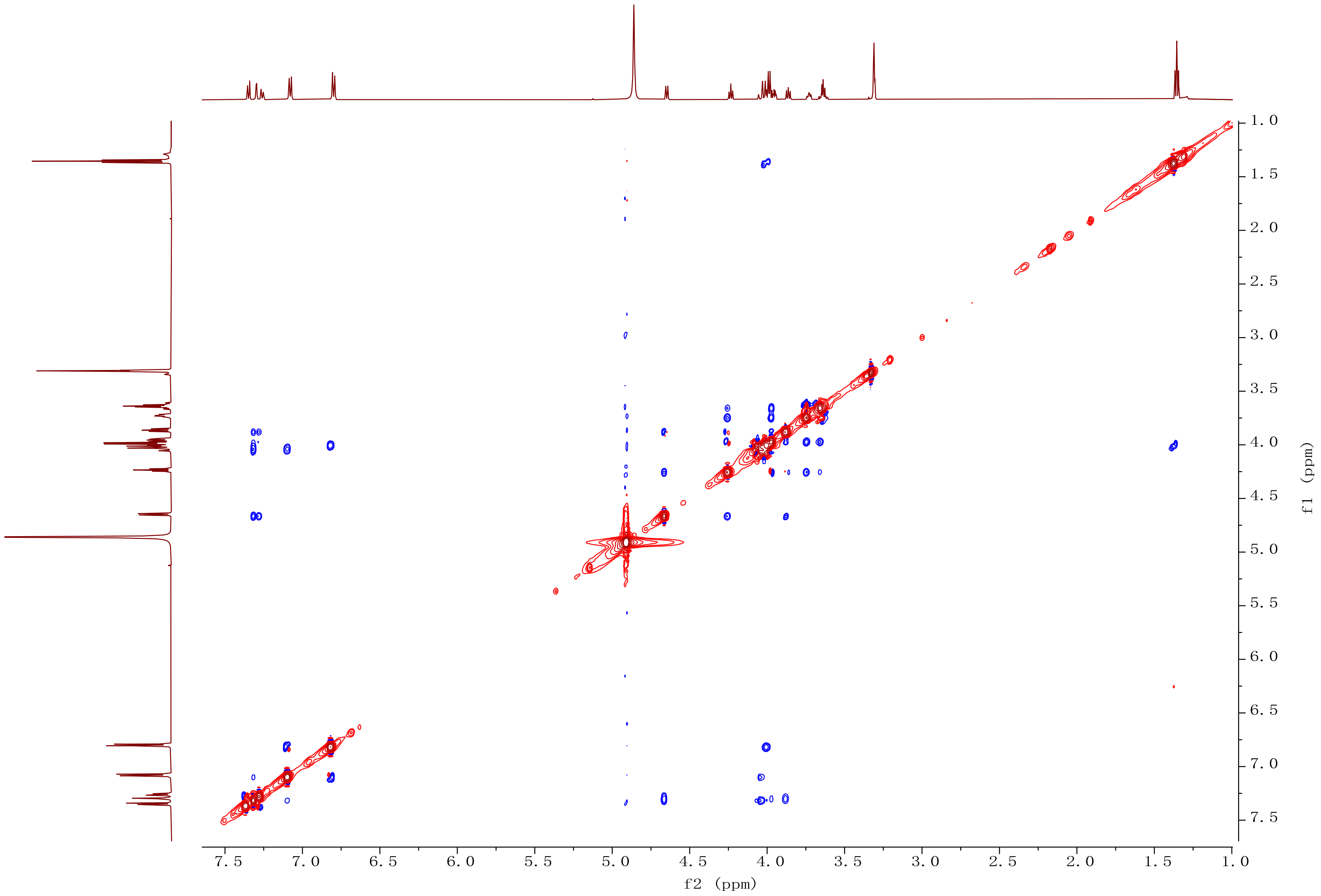

NOESY NMR of compound **α–56**

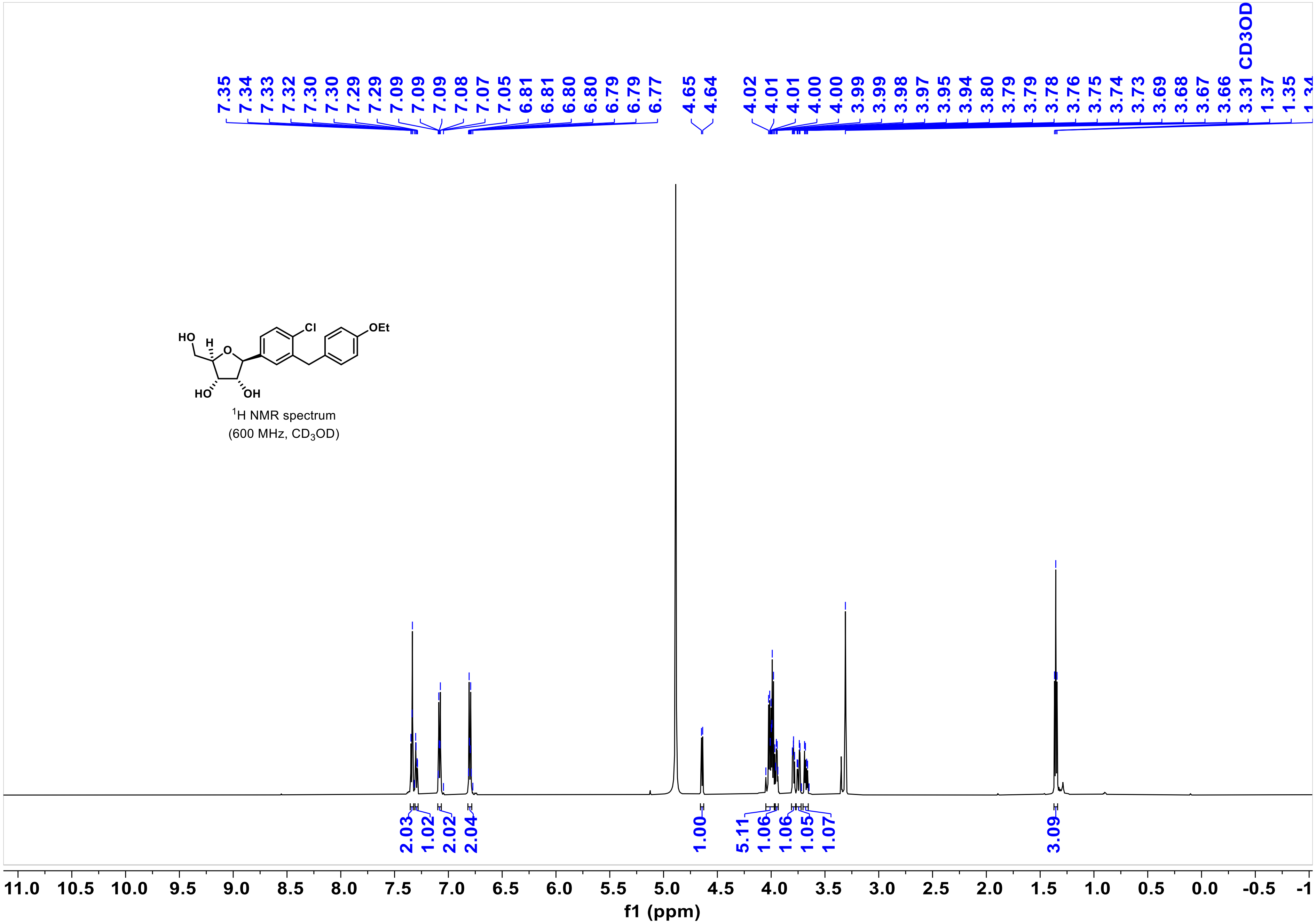

^{1}H NMR of compound **β–60**

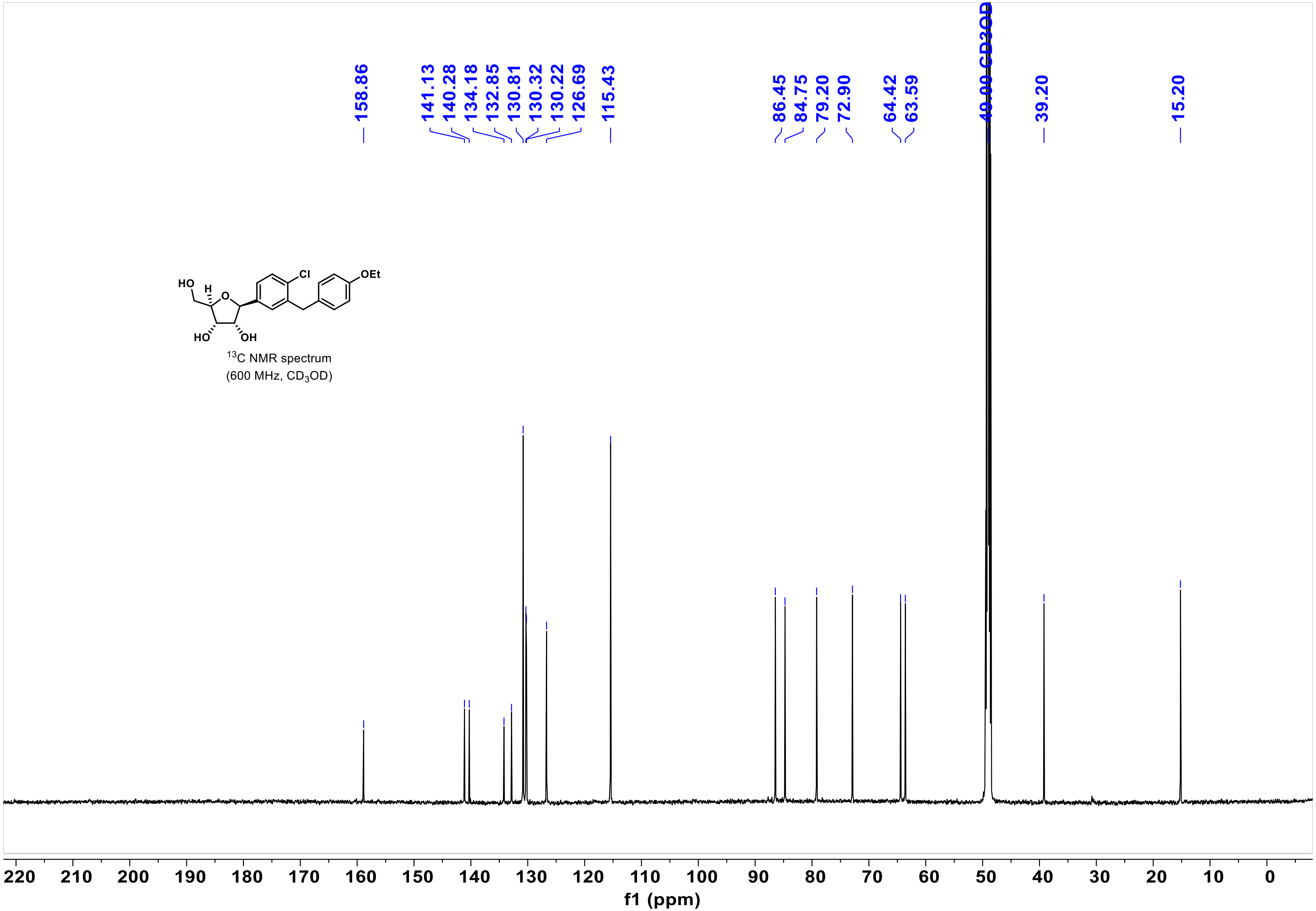

^{13}C NMR of compound **β–60**

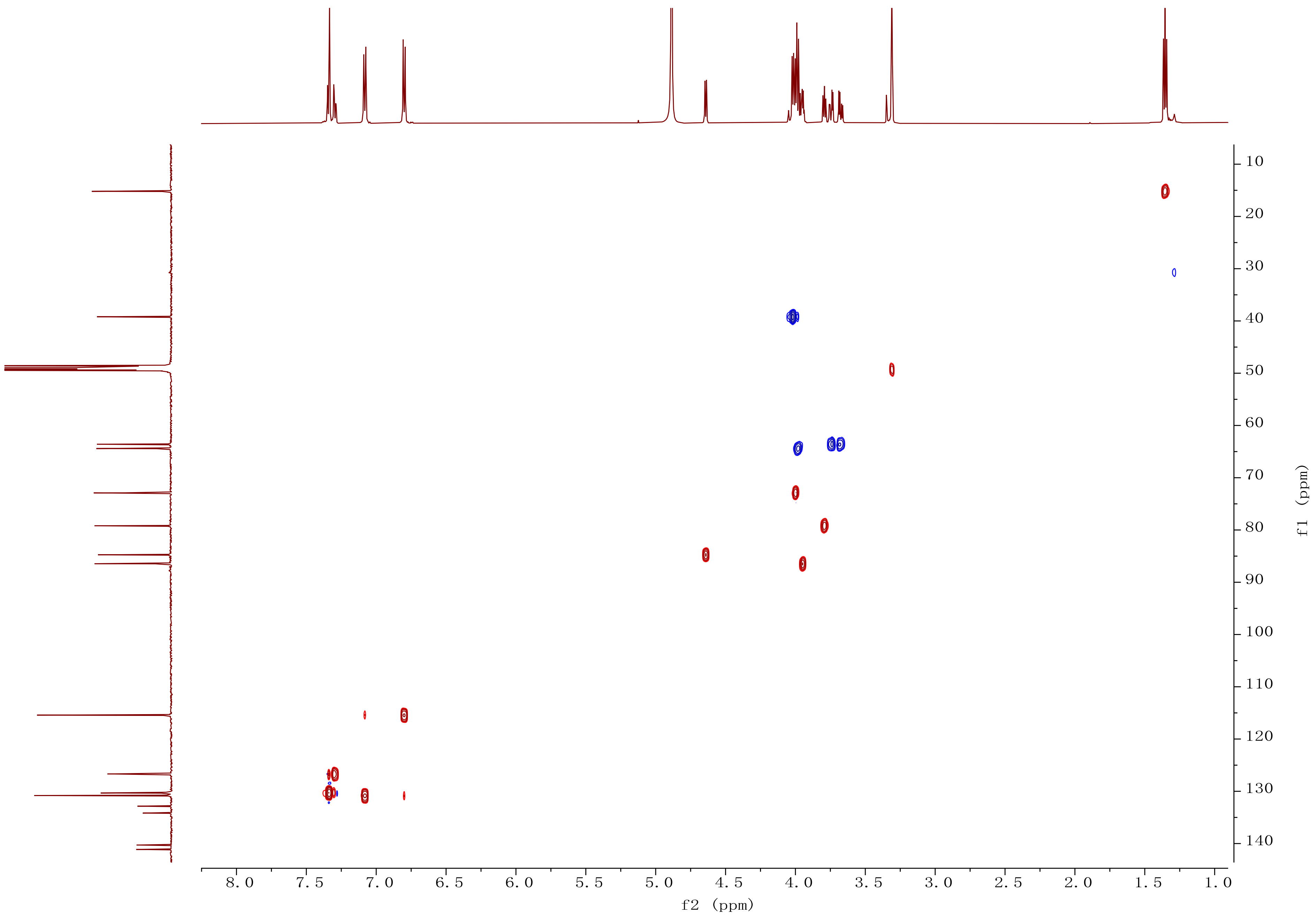

H-H HSQC NMR of compound **β–60**

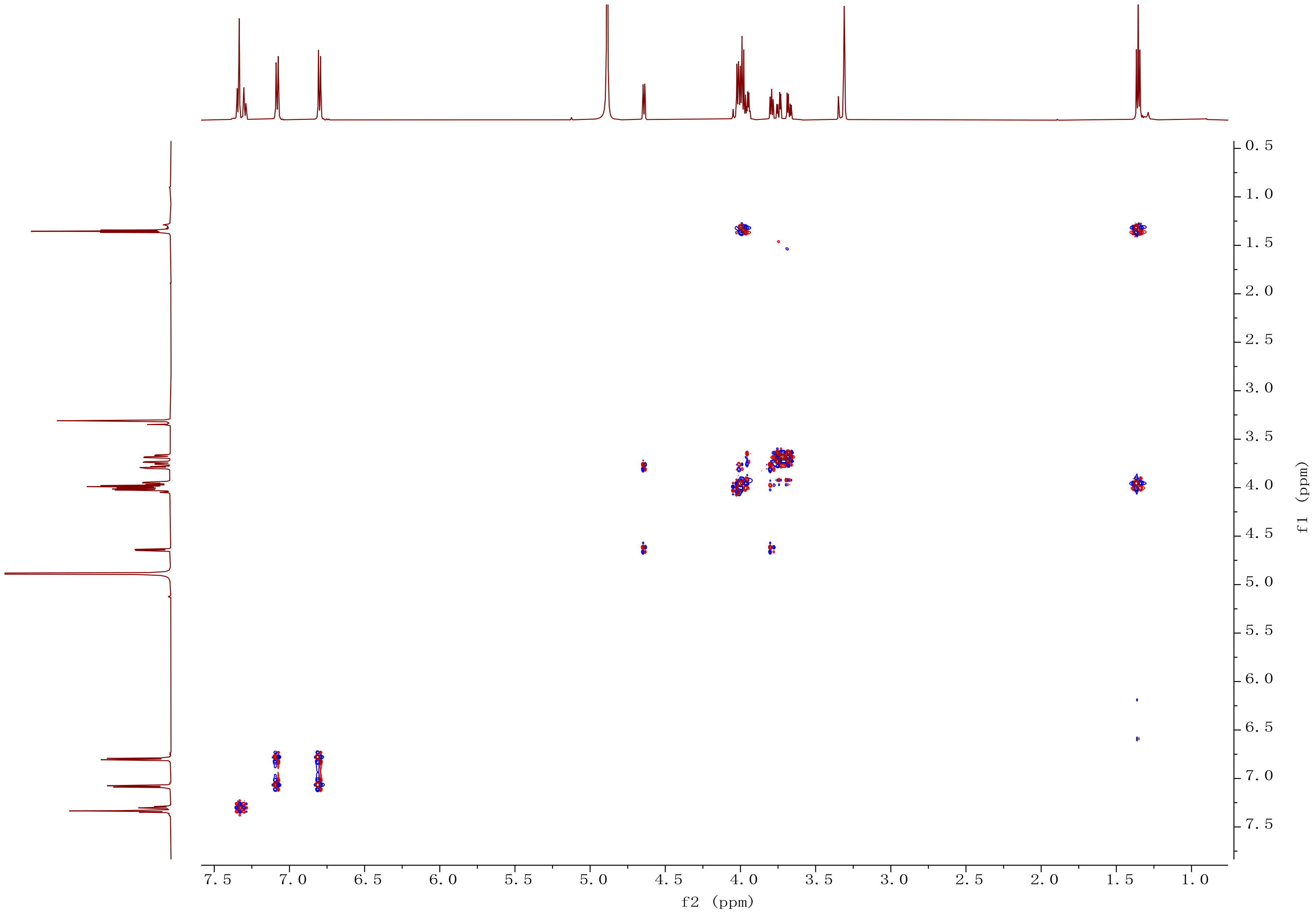

H-H COSY NMR of compound **β–60**

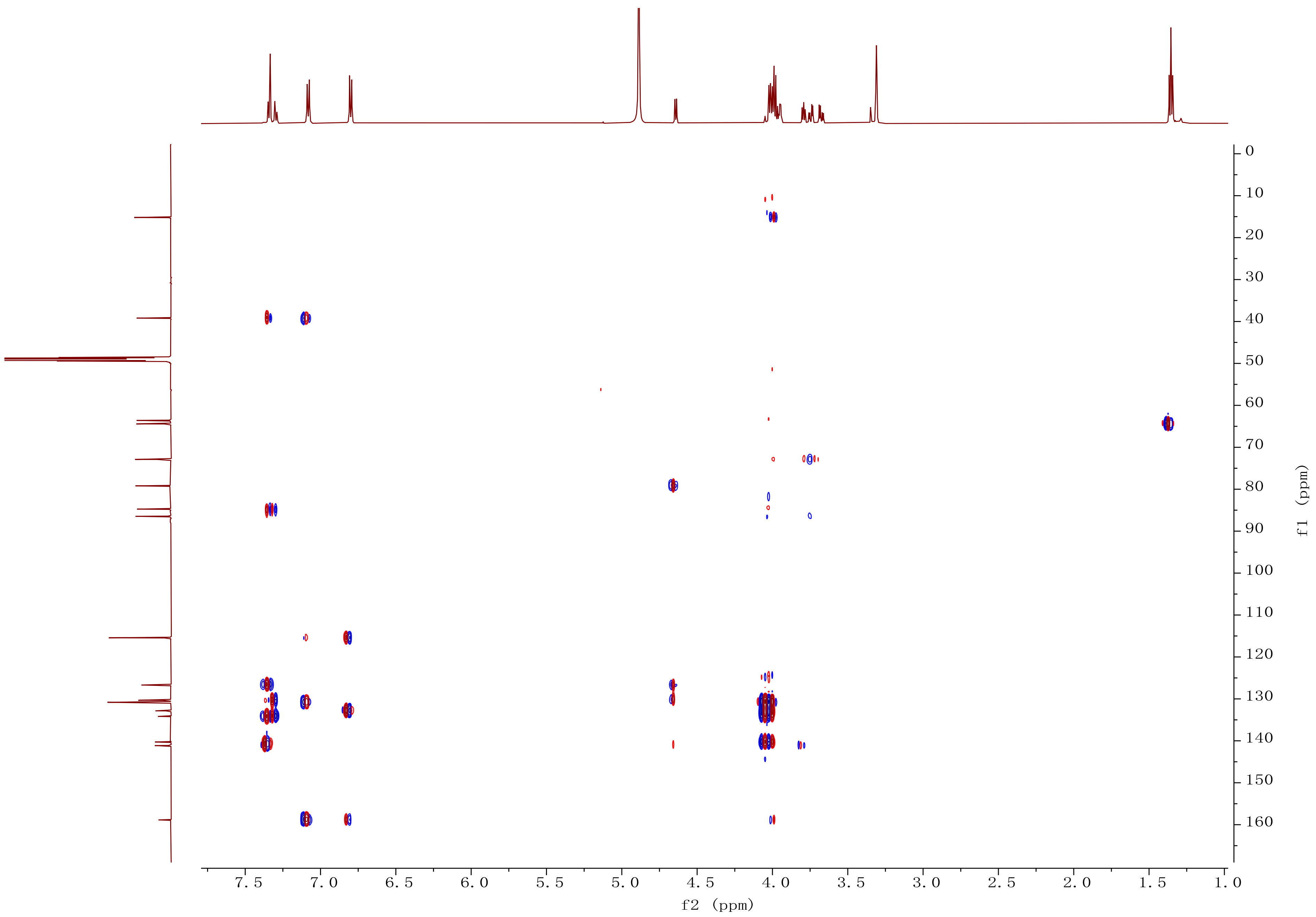

H-C HMBC NMR of compound **β–60**

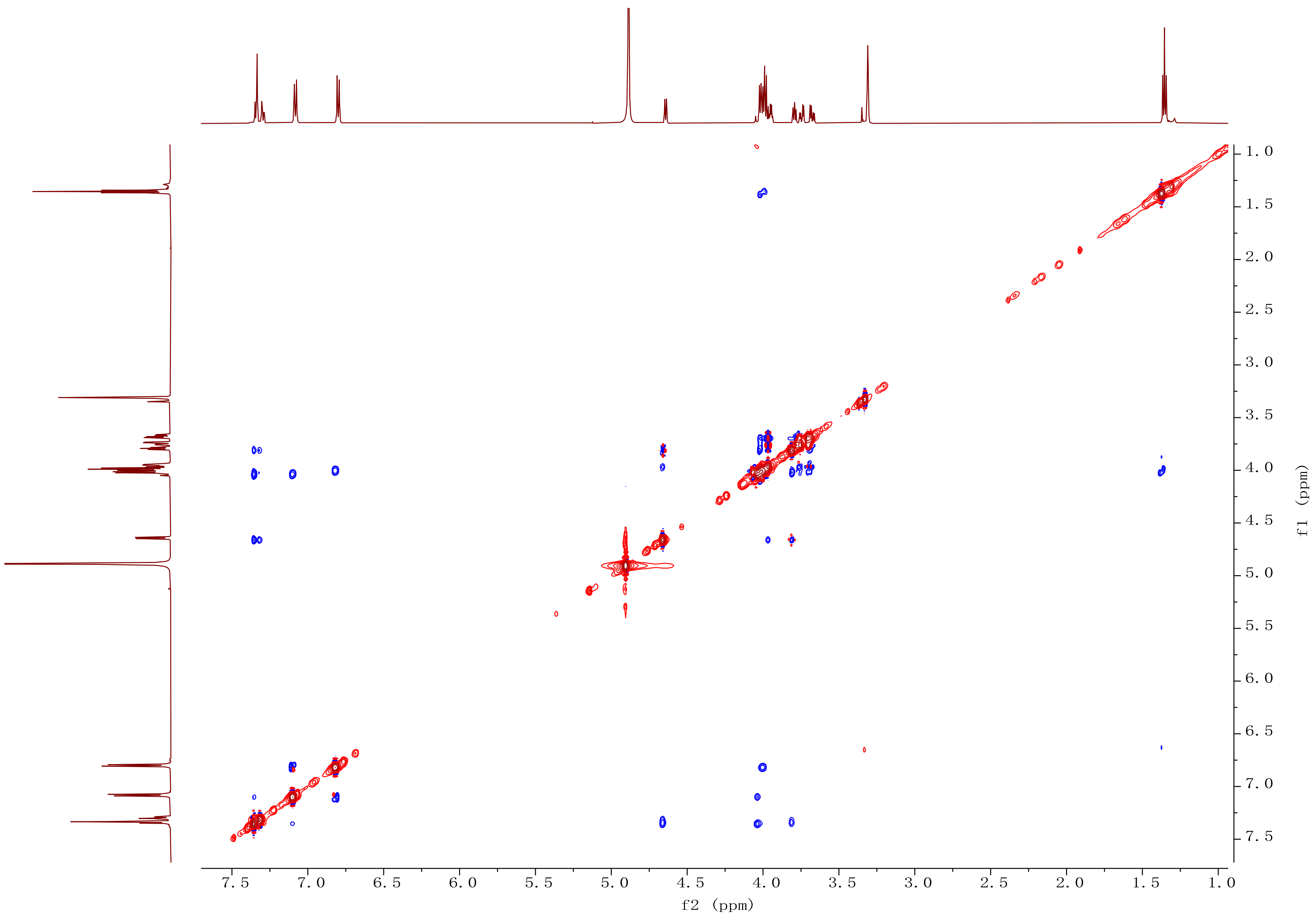

NOESY NMR of compound **β–60**

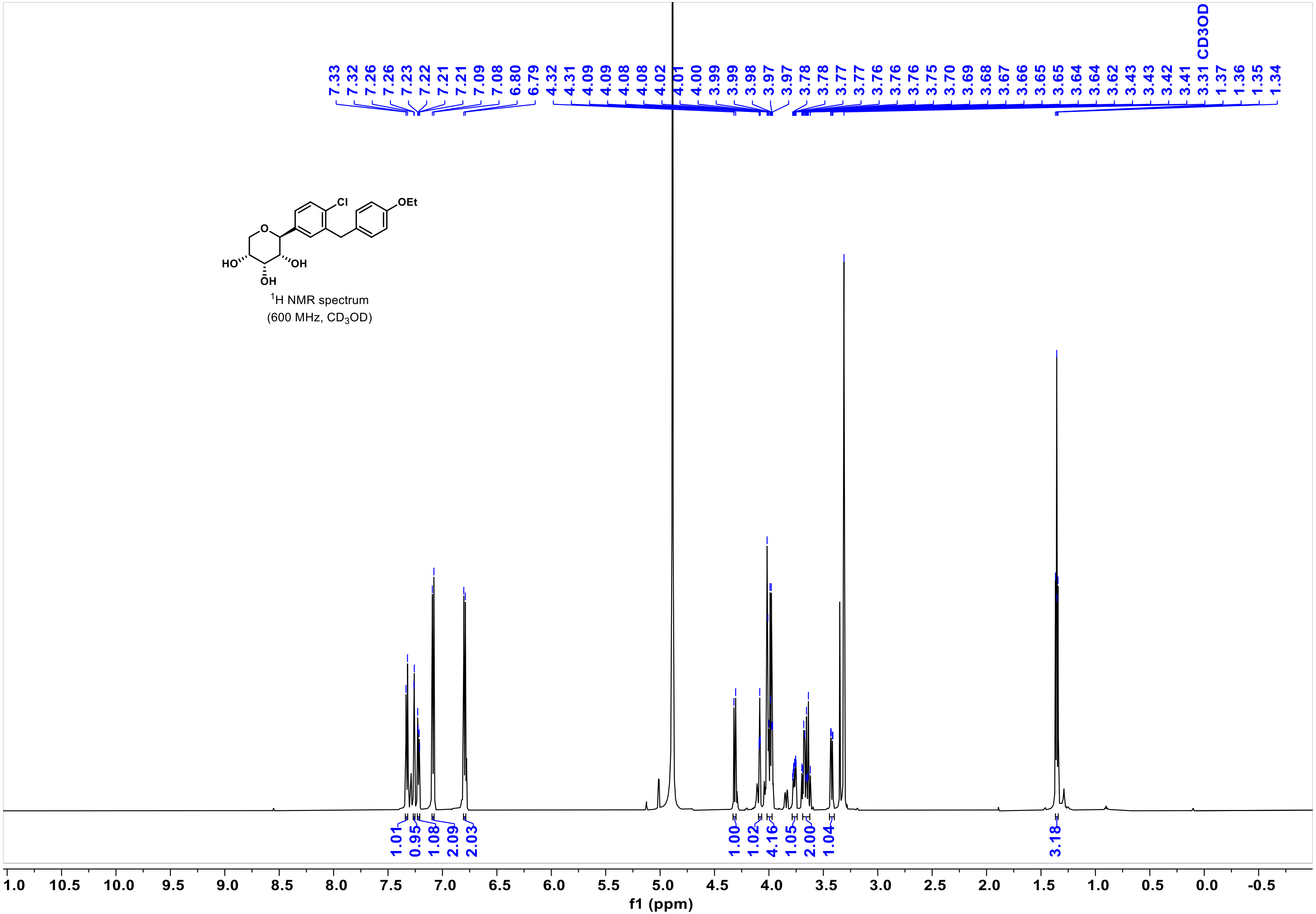

^{1}H NMR of compound β–59

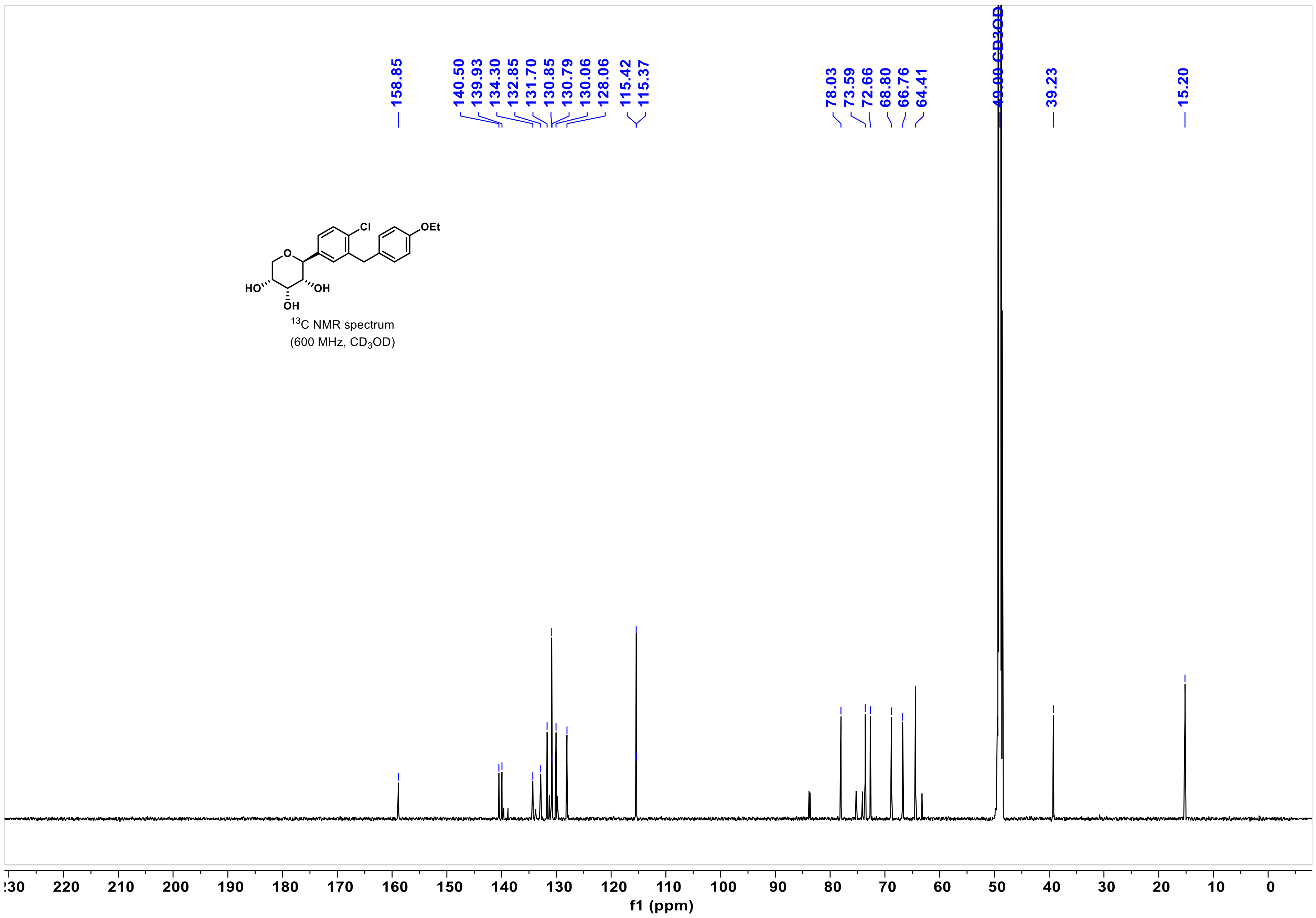

^{13}C NMR of compound **β–59**

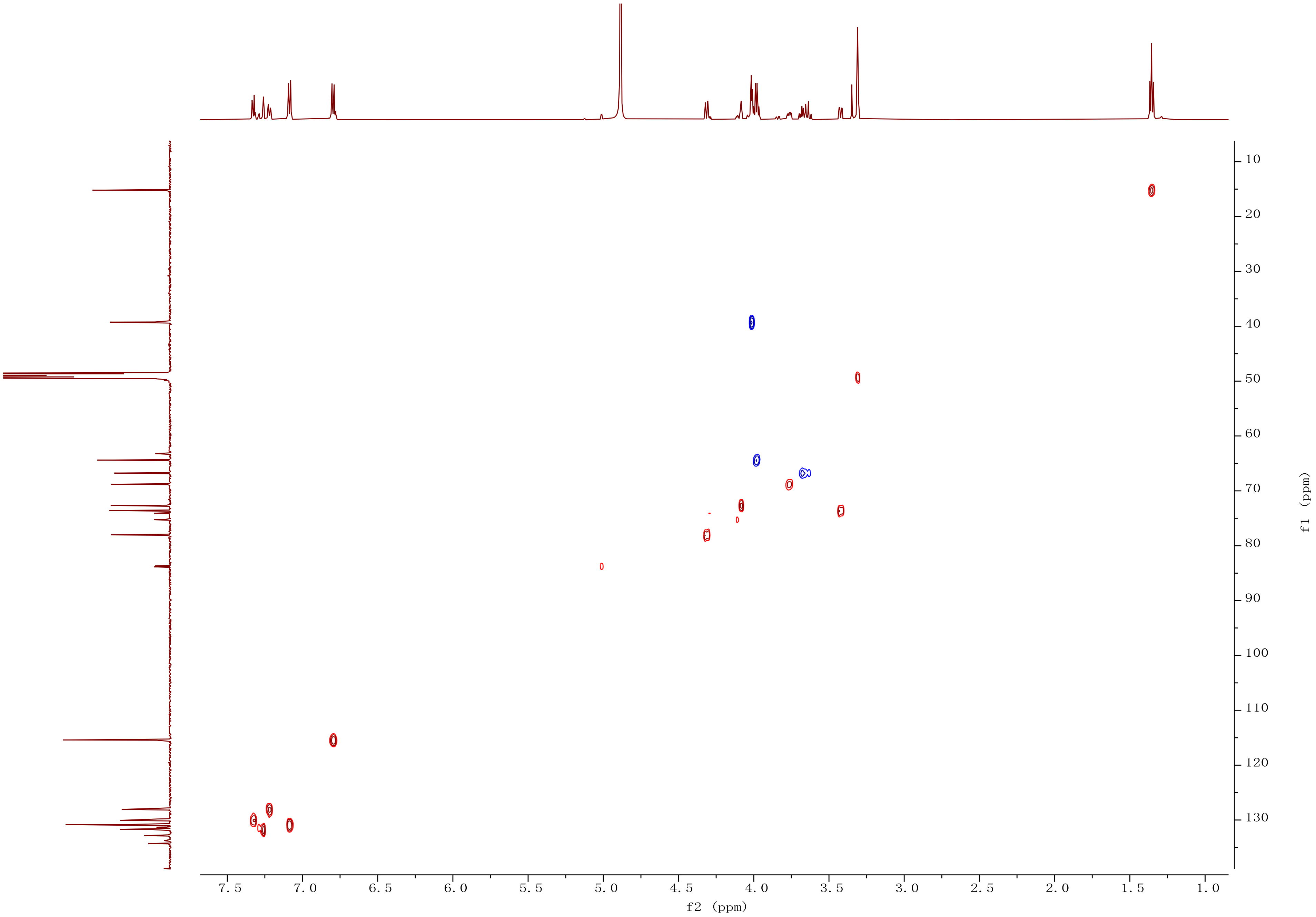

H-H HSQC NMR of compound **β–59**

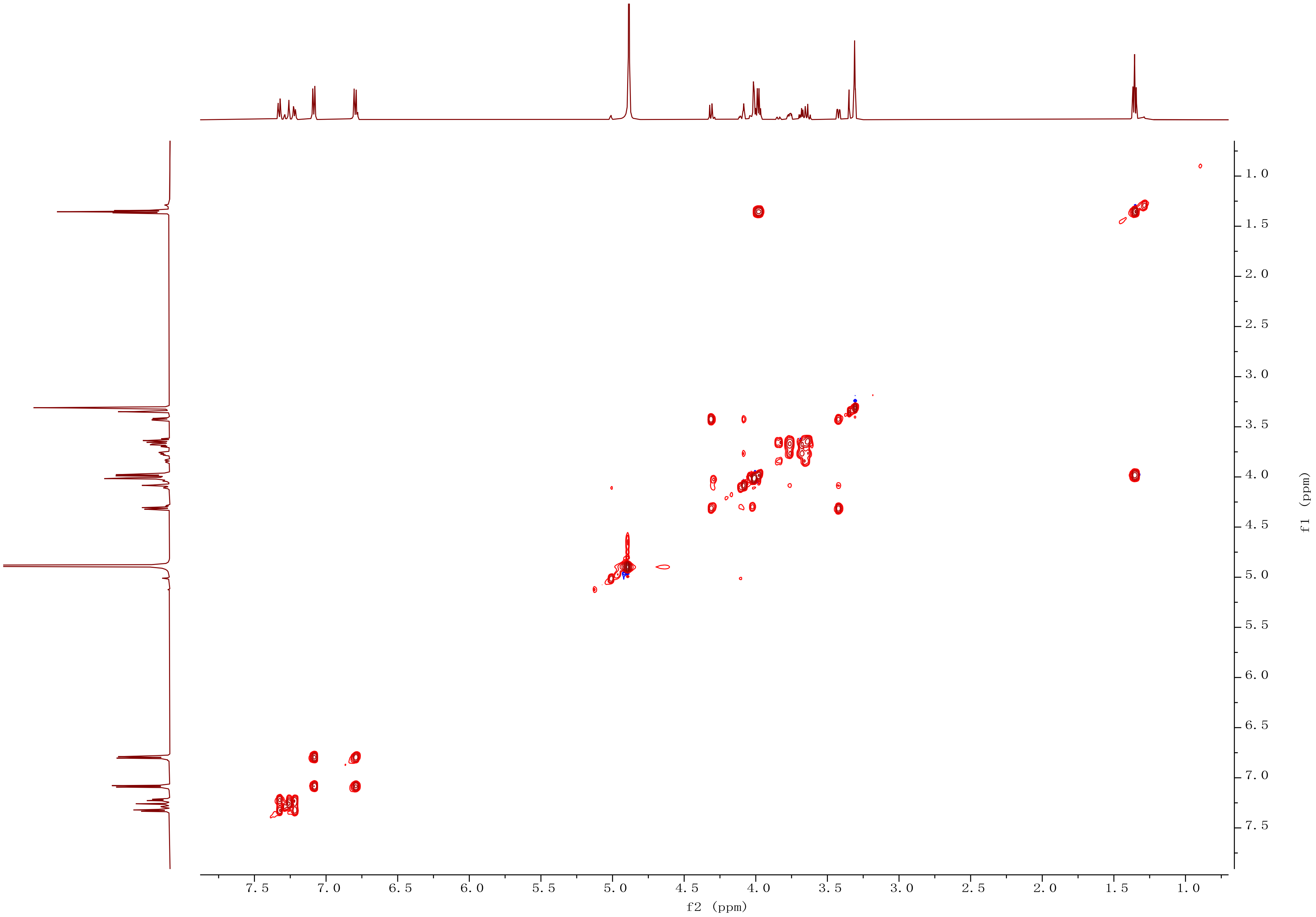

H-H COSY NMR of compound **β–59**

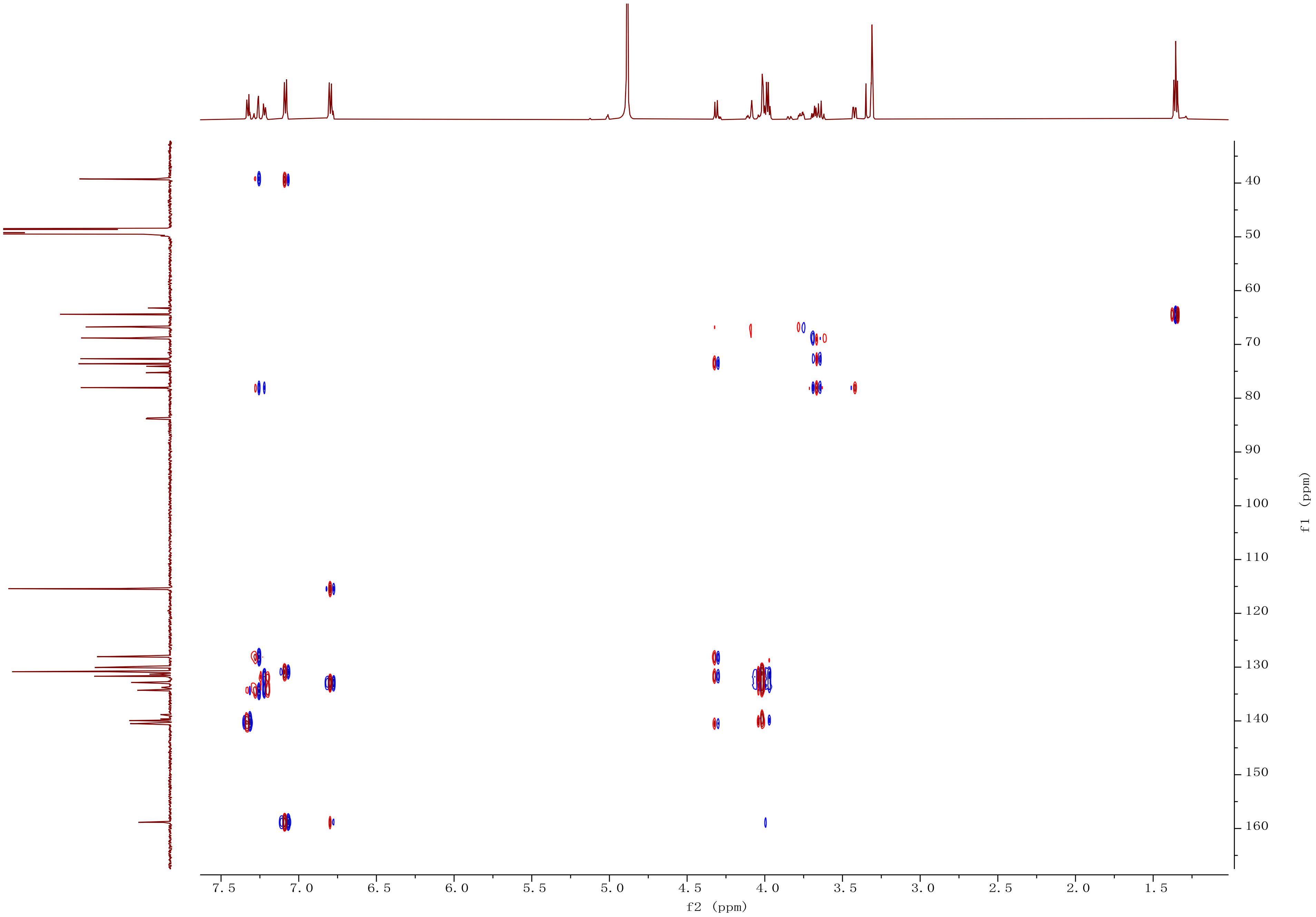

H-C HMBC NMR of compound **β–59**

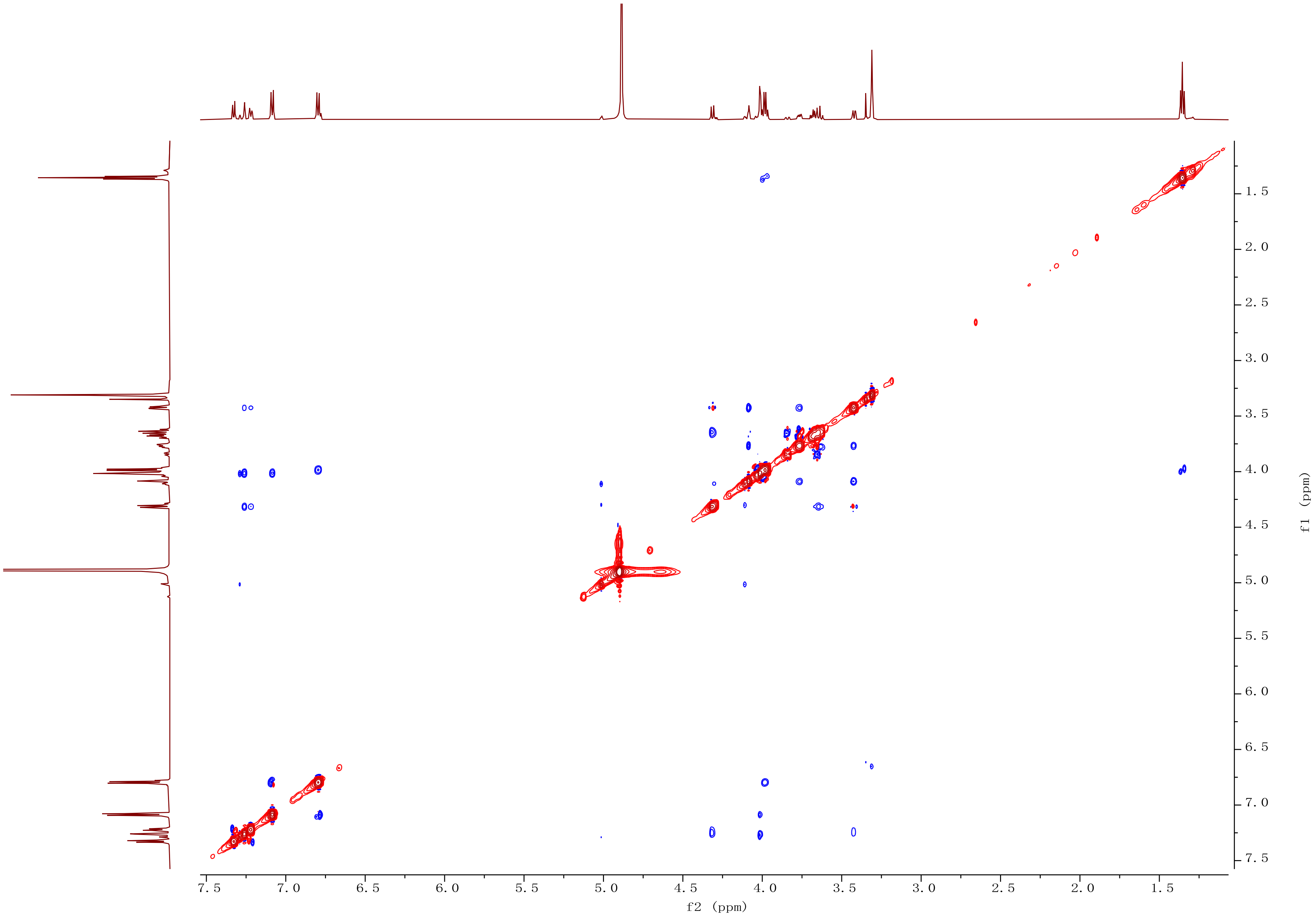

NOESY NMR of compound **β–59**

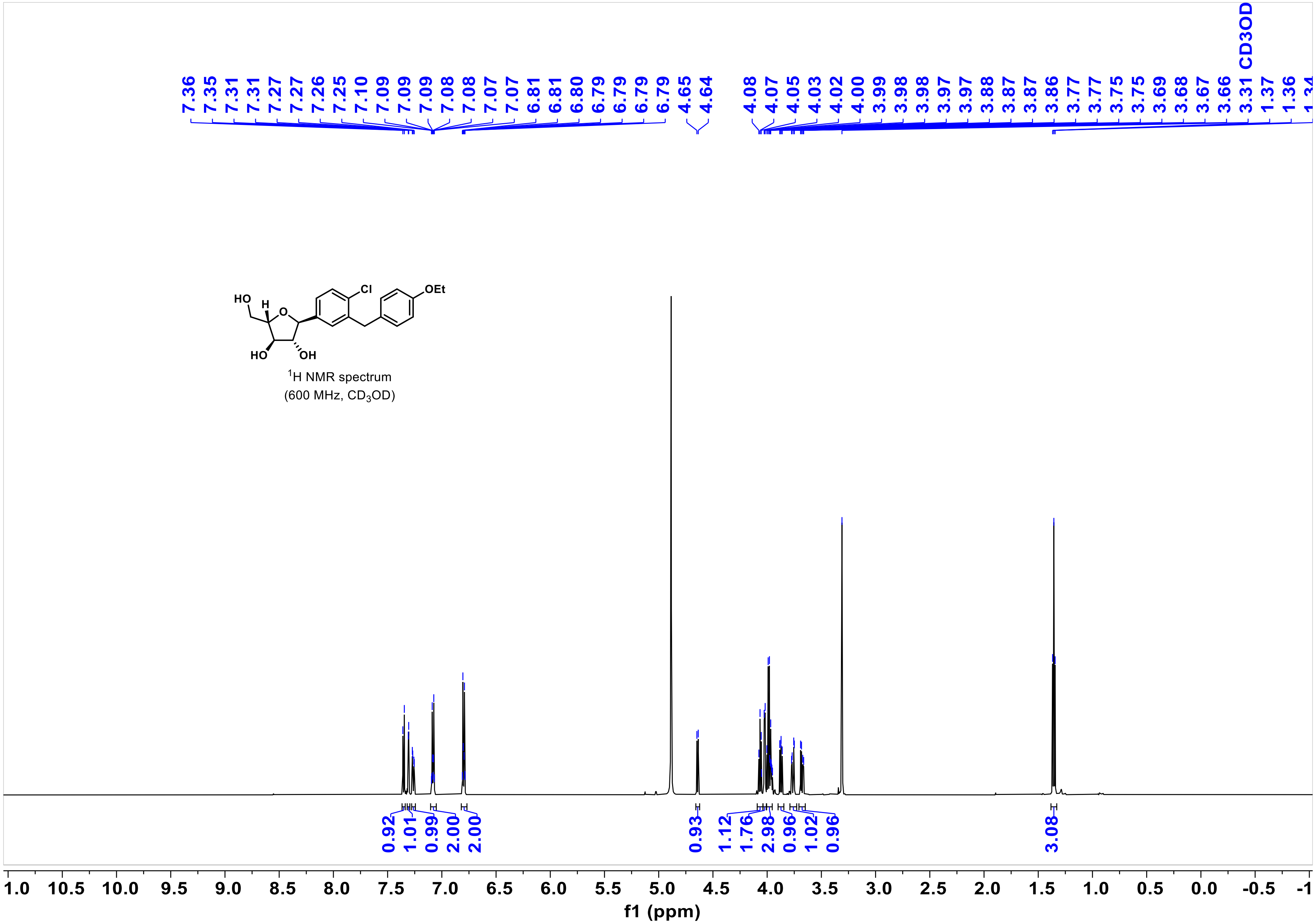

^{1}H NMR of compound **β–62**

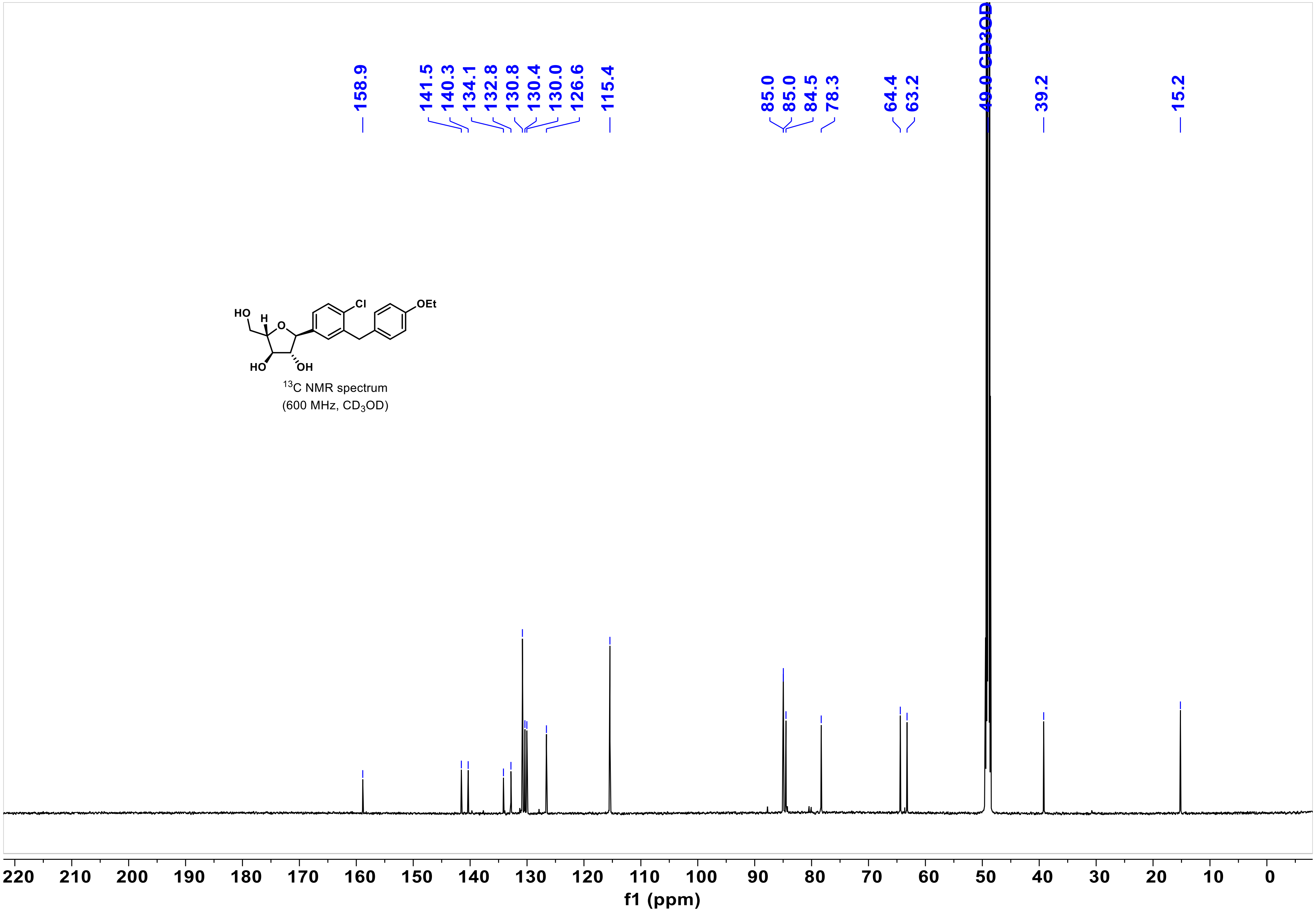

^{13}C NMR of compound **β–62**

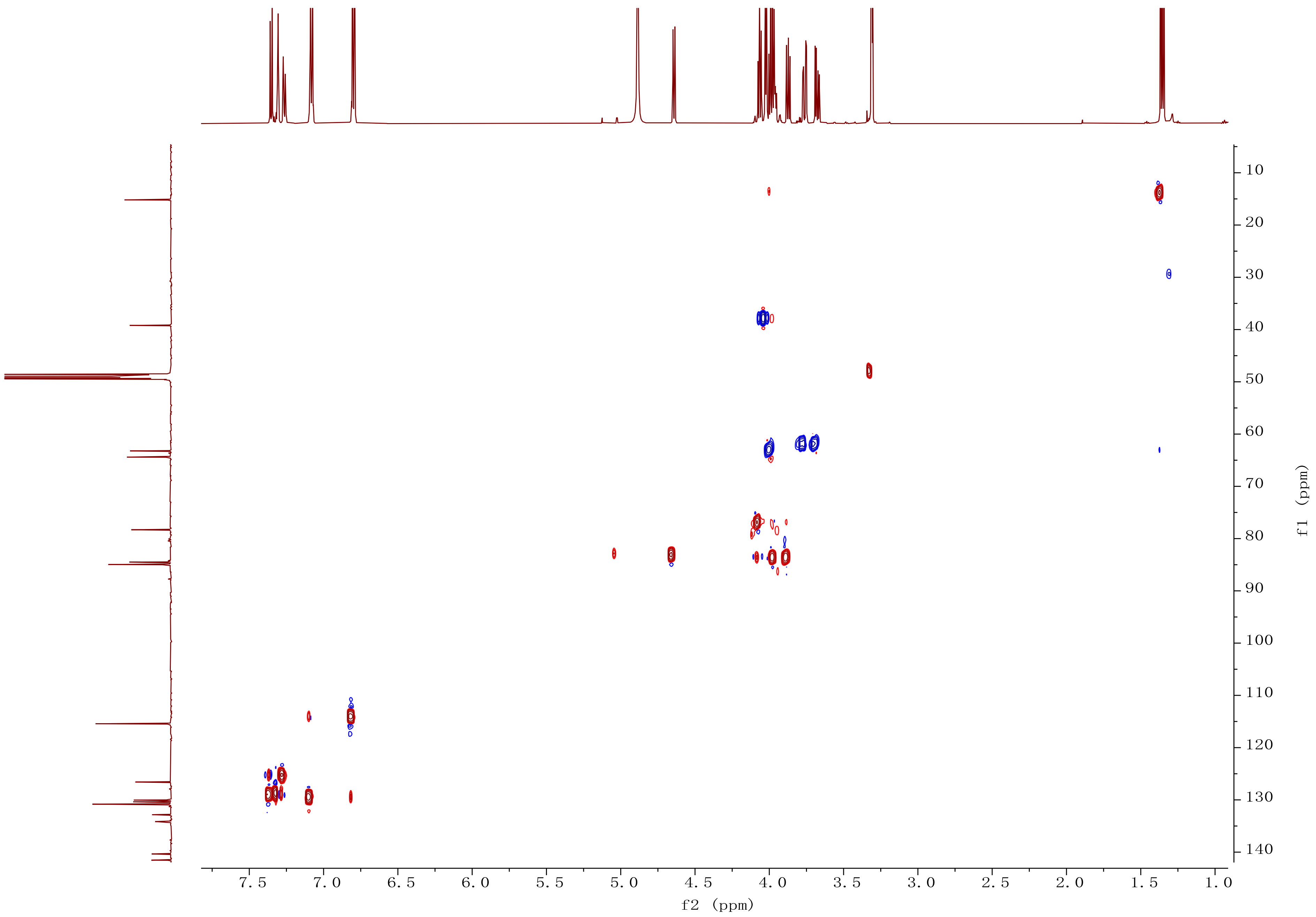

H-H HSQC NMR of compound **β–62**

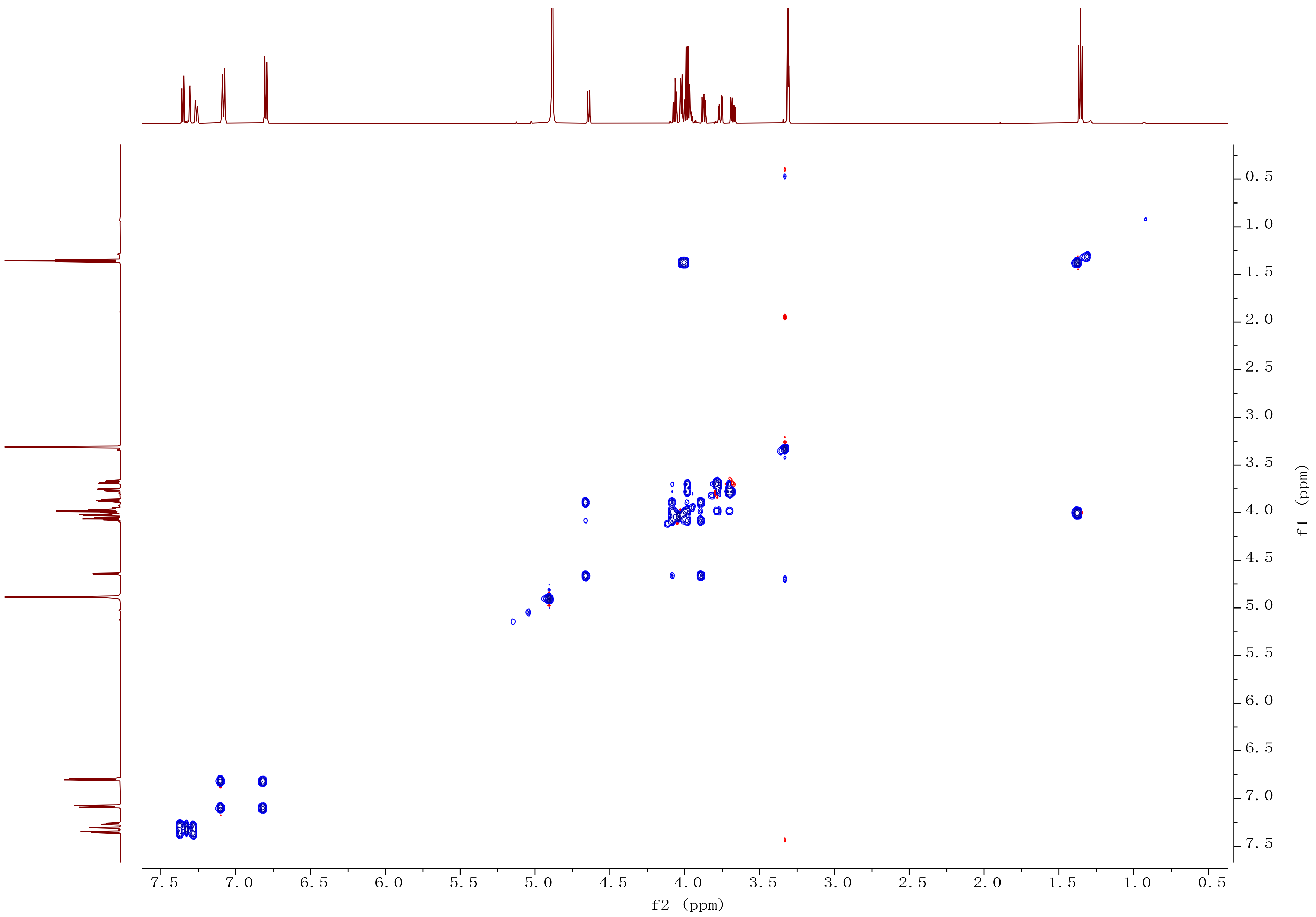

H-H COSY NMR of compound **β–62**

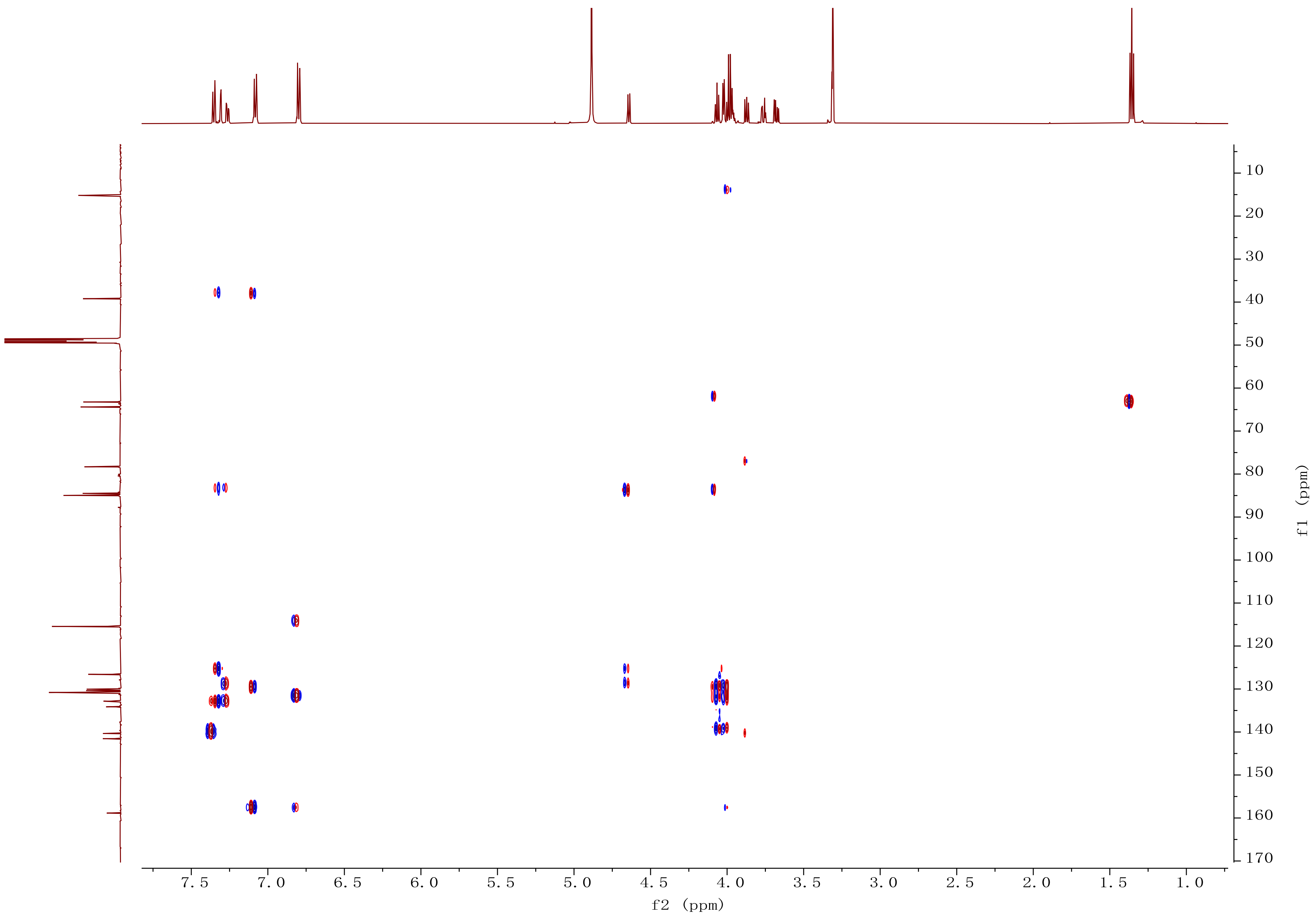

H-C HMBC NMR of compound **β–62**

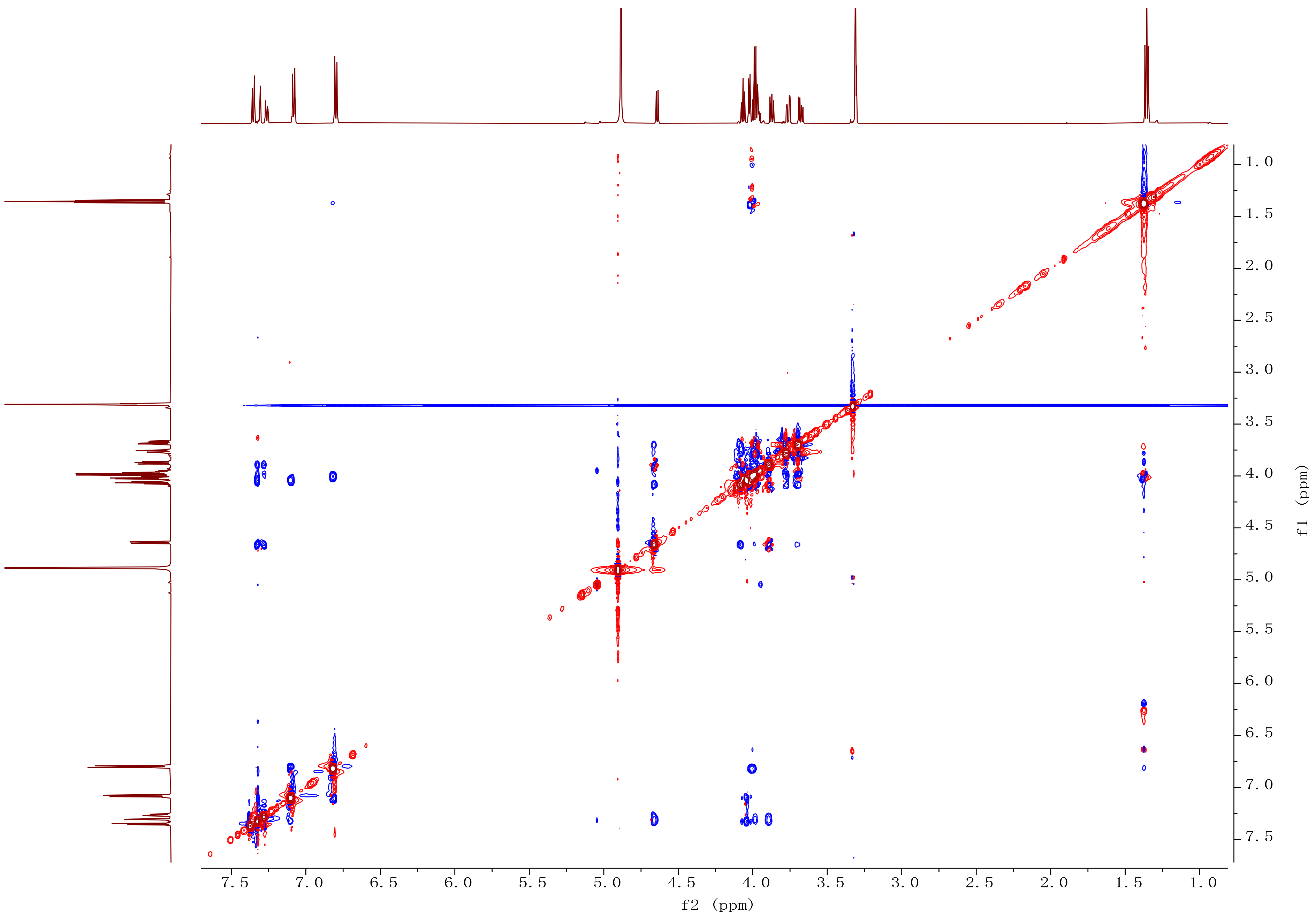

NOESY NMR of compound **β–62**

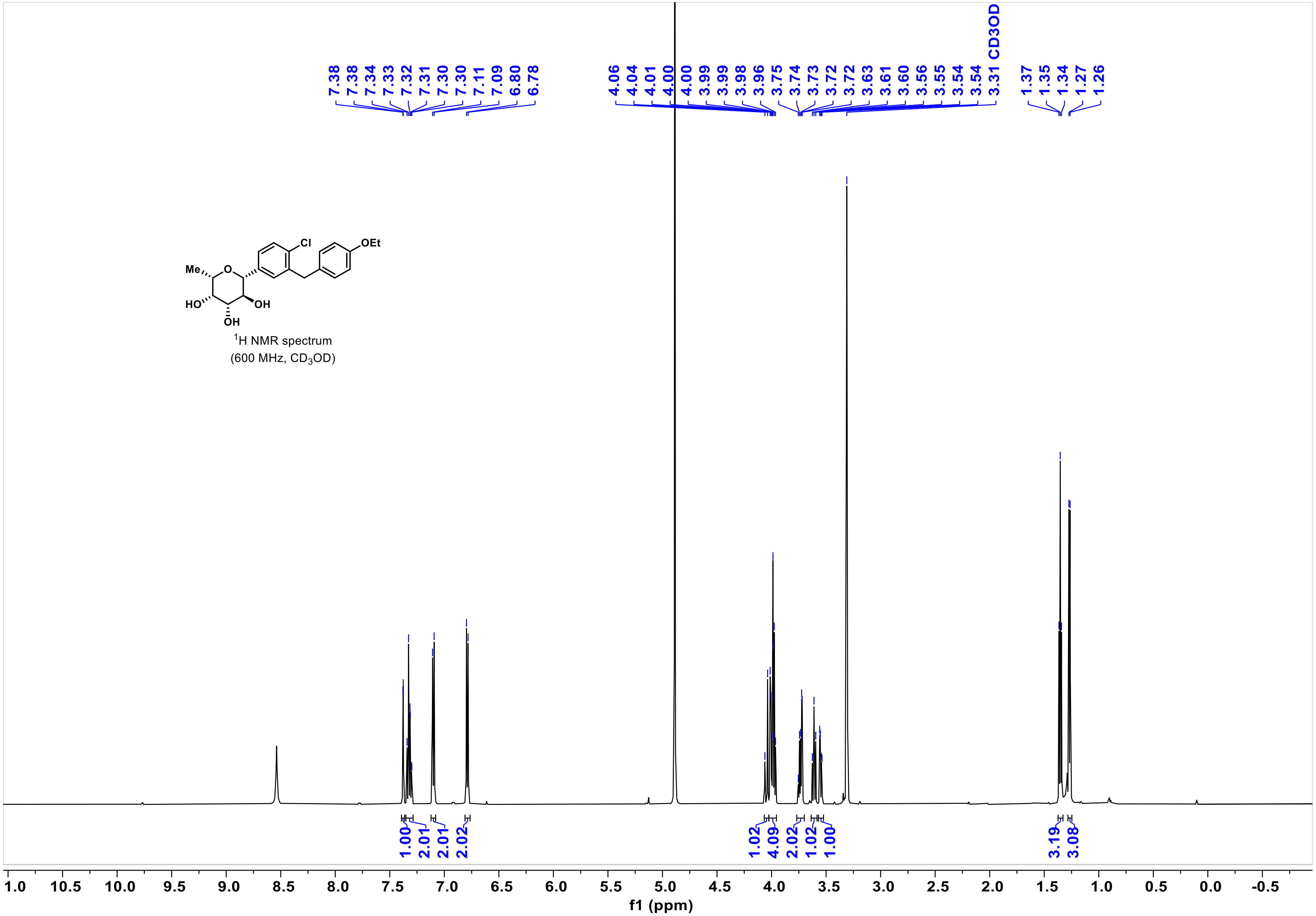

^{1}H NMR of compound β–57

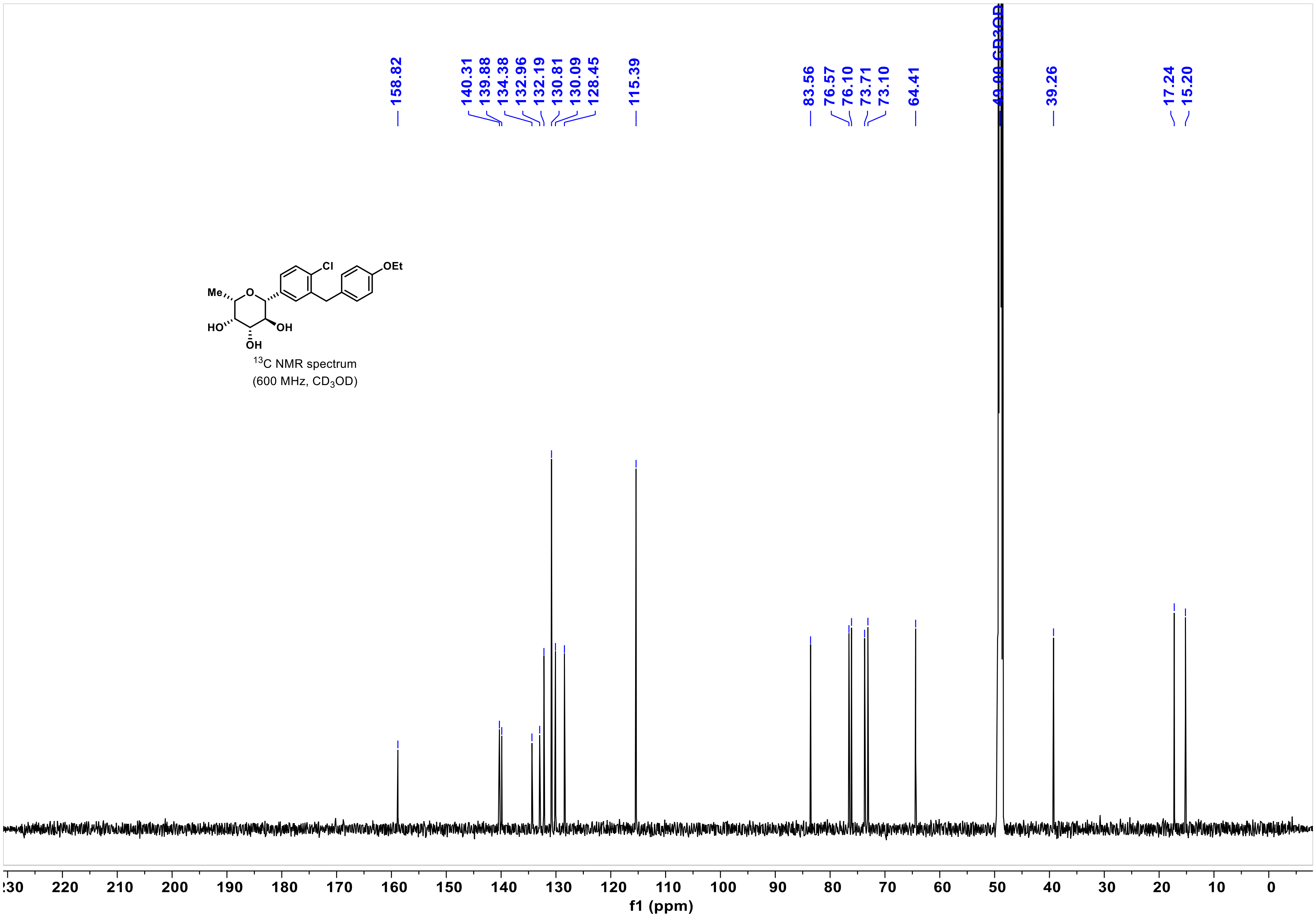

^{13}C NMR of compound **β–57**

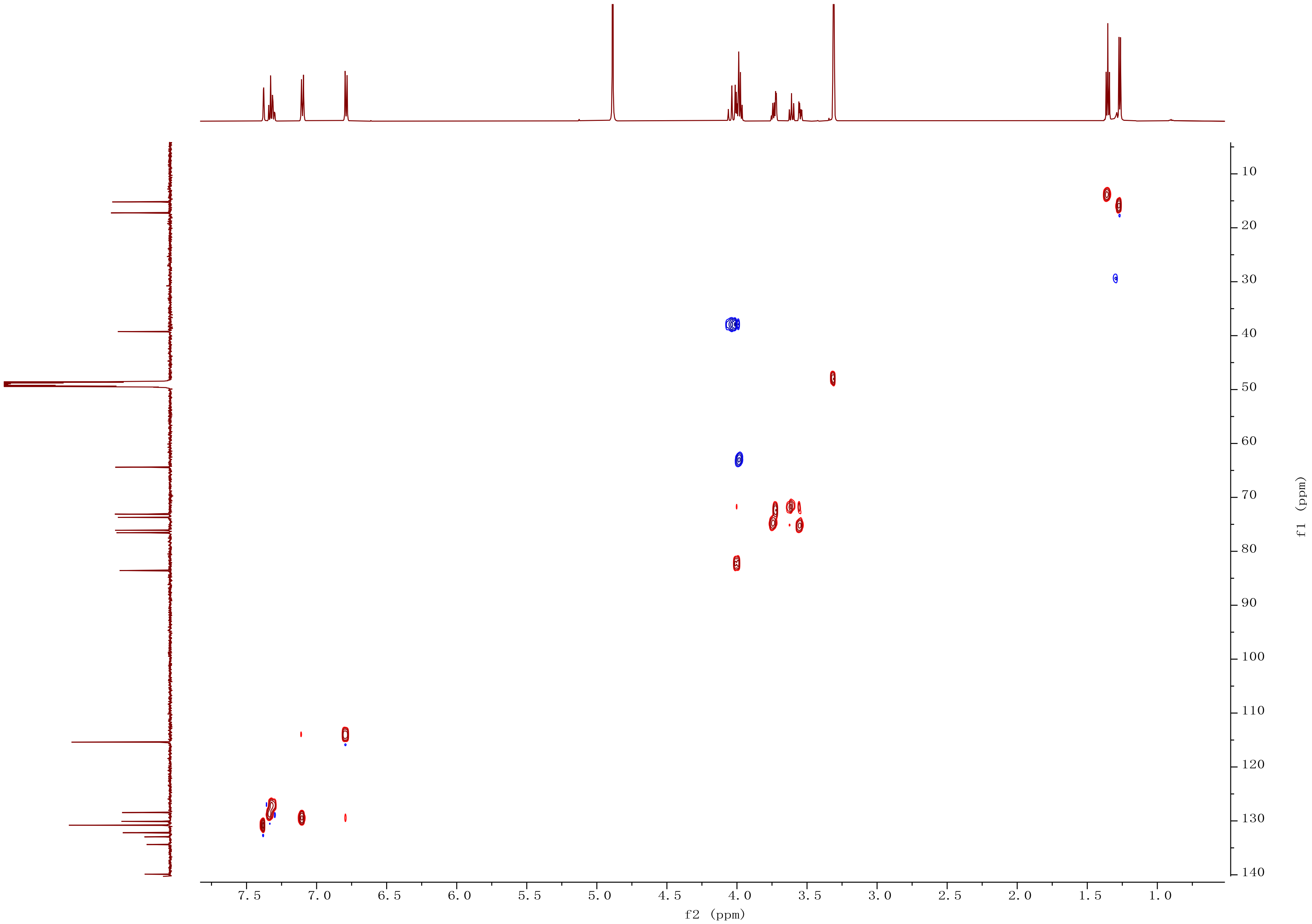

H-H HSQC NMR of compound **β–57**

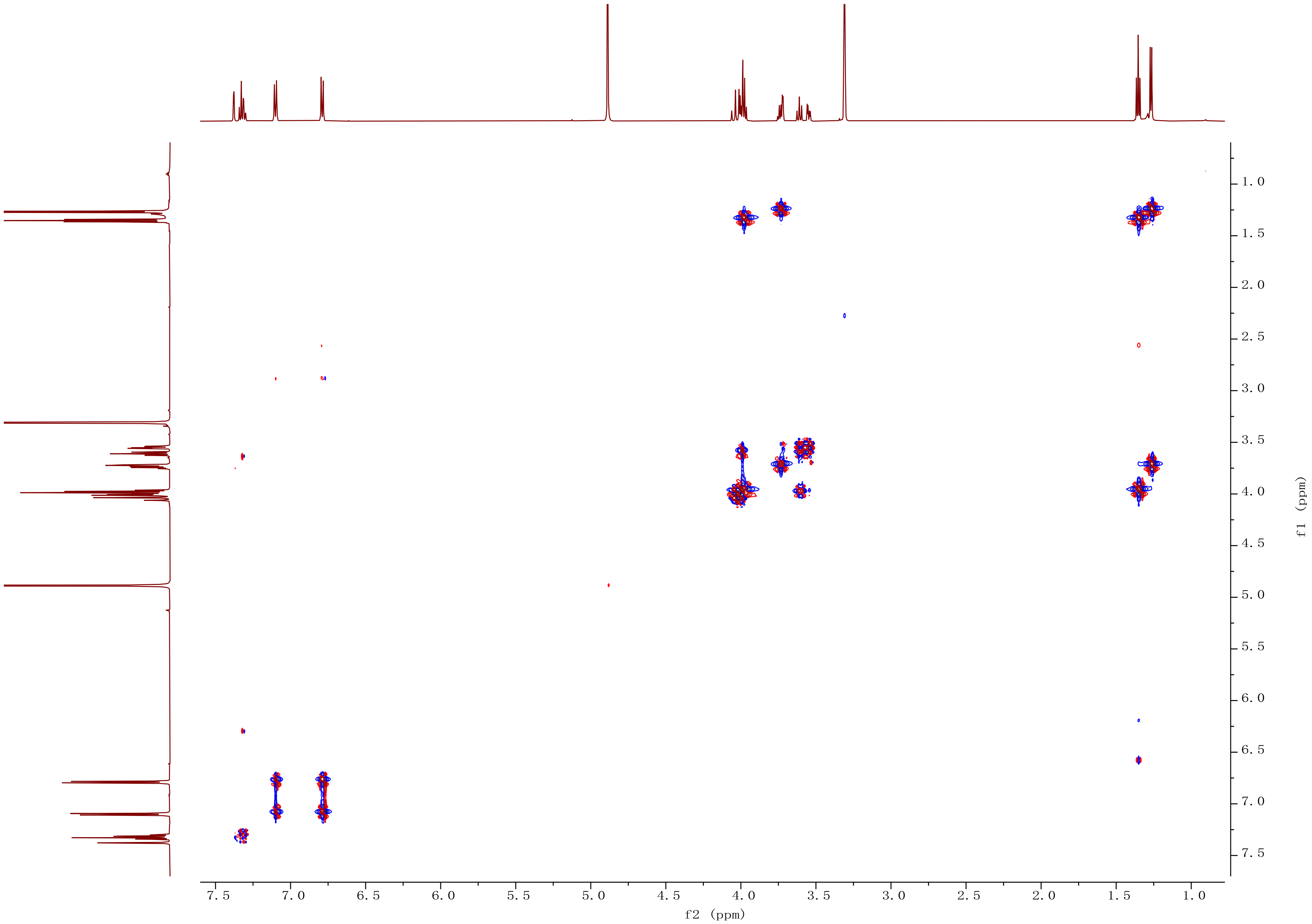

H-H COSY NMR of compound **β–57**

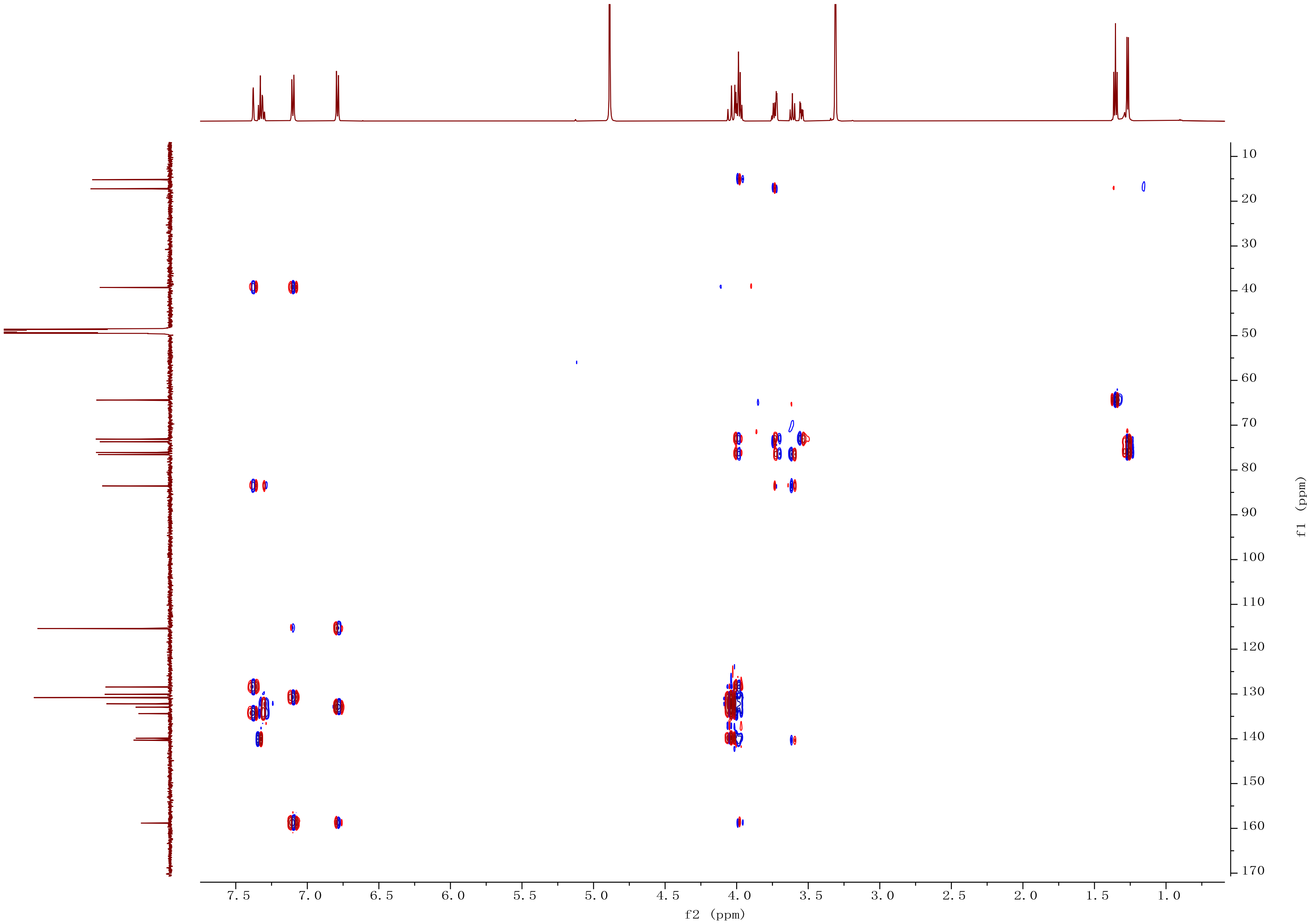

H-C HMBC NMR of compound **β–57**

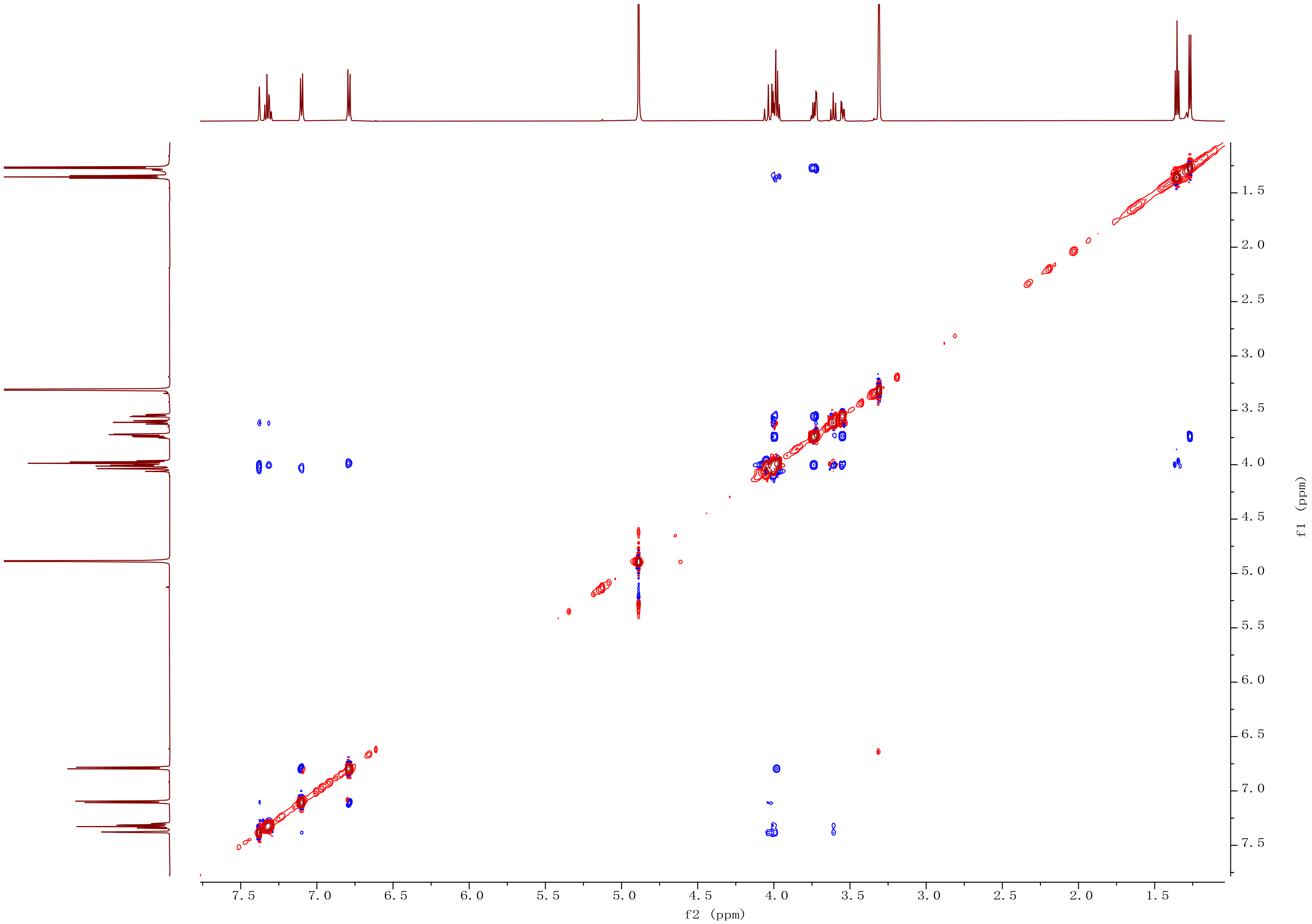

NOESY NMR of compound **β–57**

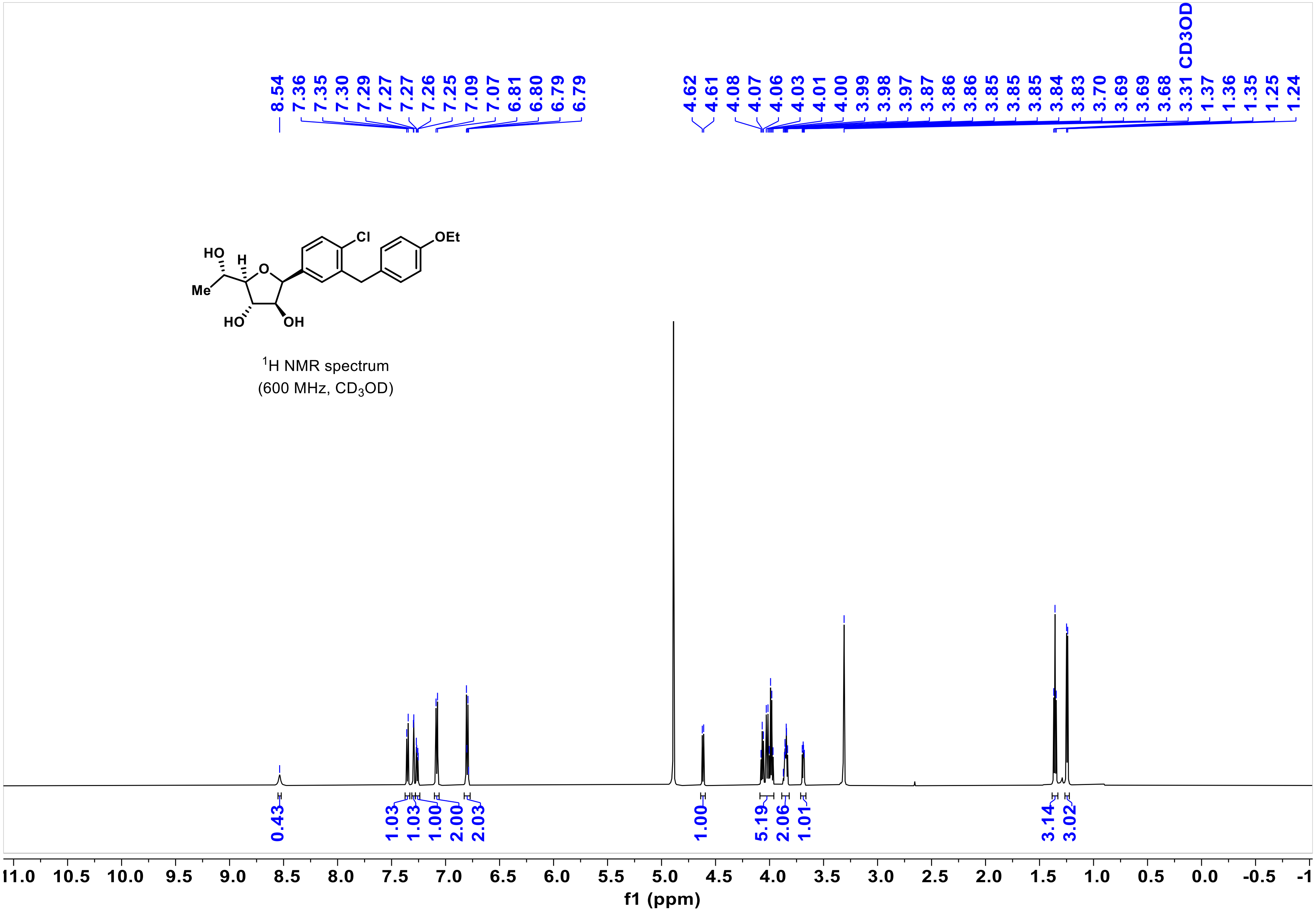

^{1}H NMR of compound α–58

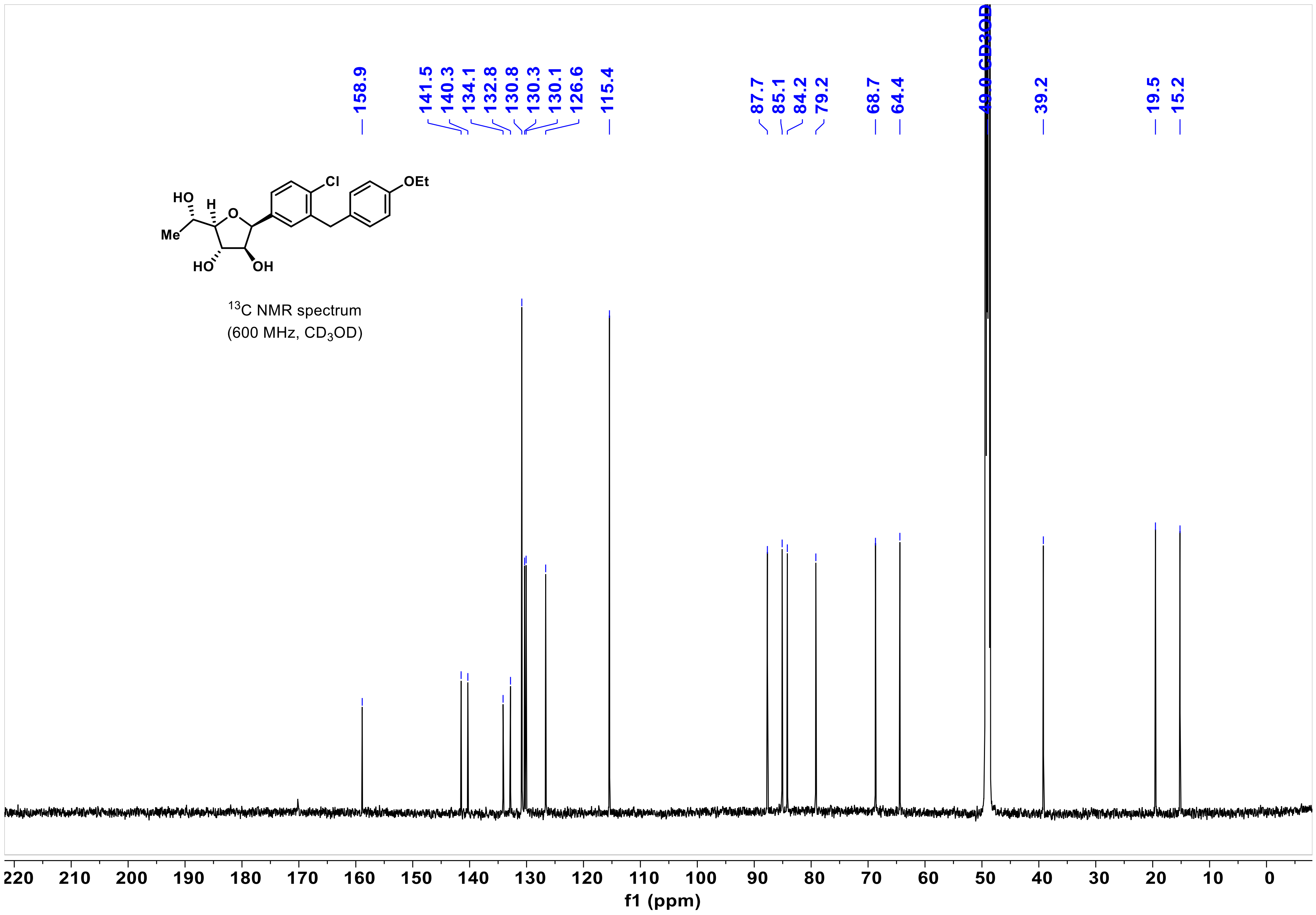

^{13}C NMR of compound α–58

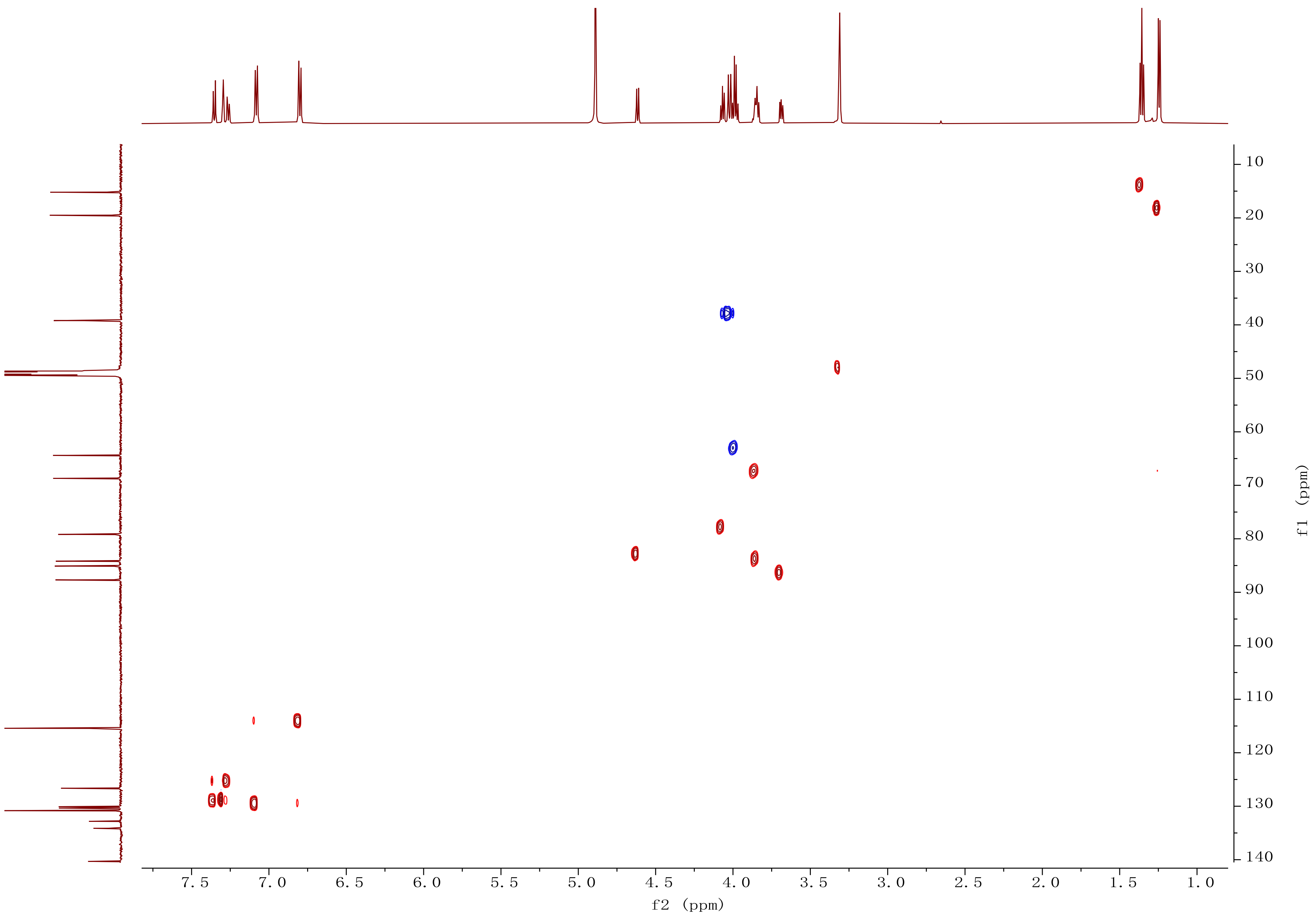

H-H HSQC NMR of compound α–58

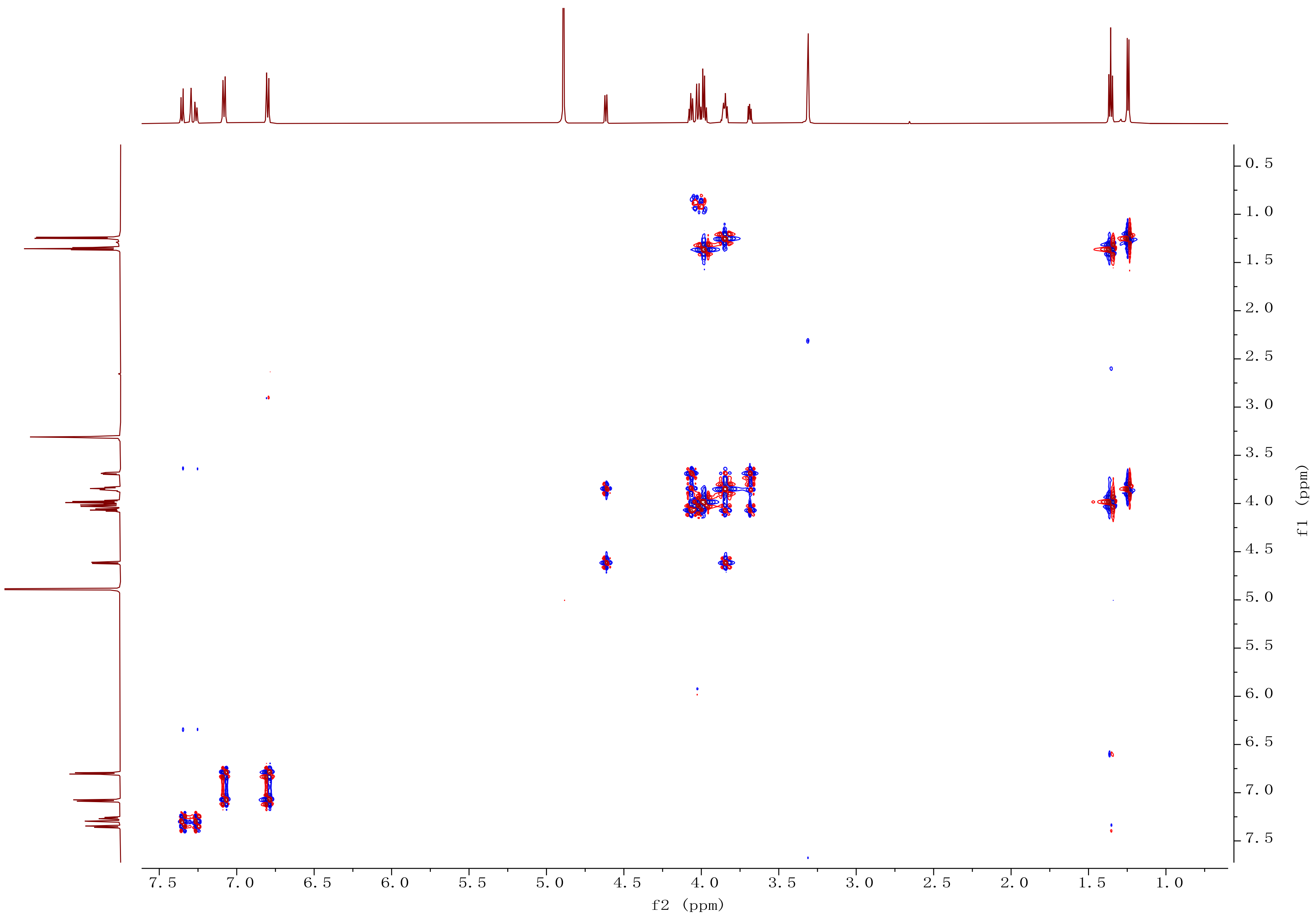

H-H COSY NMR of compound **α–58**

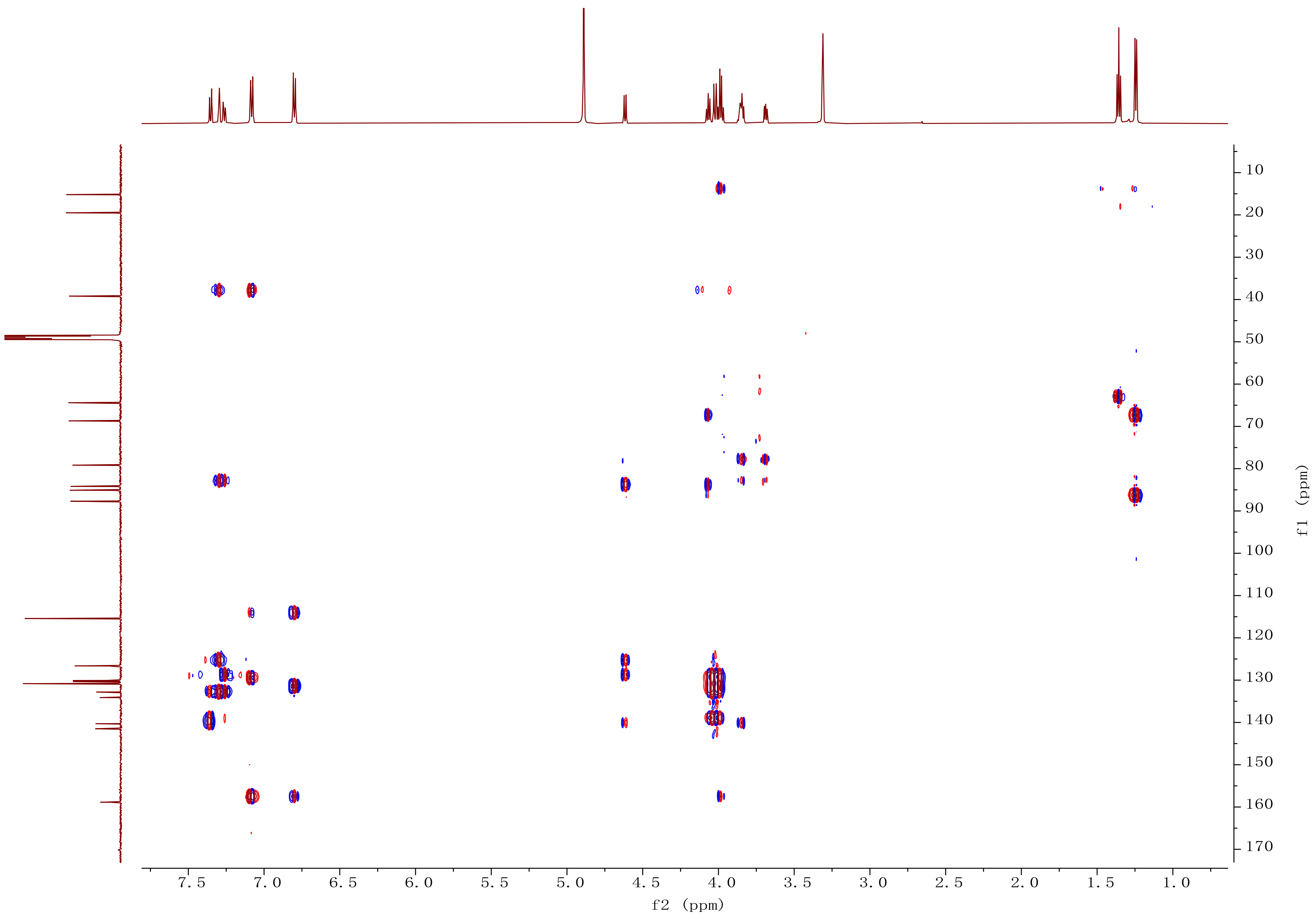

H-C HMBC NMR of compound α–58

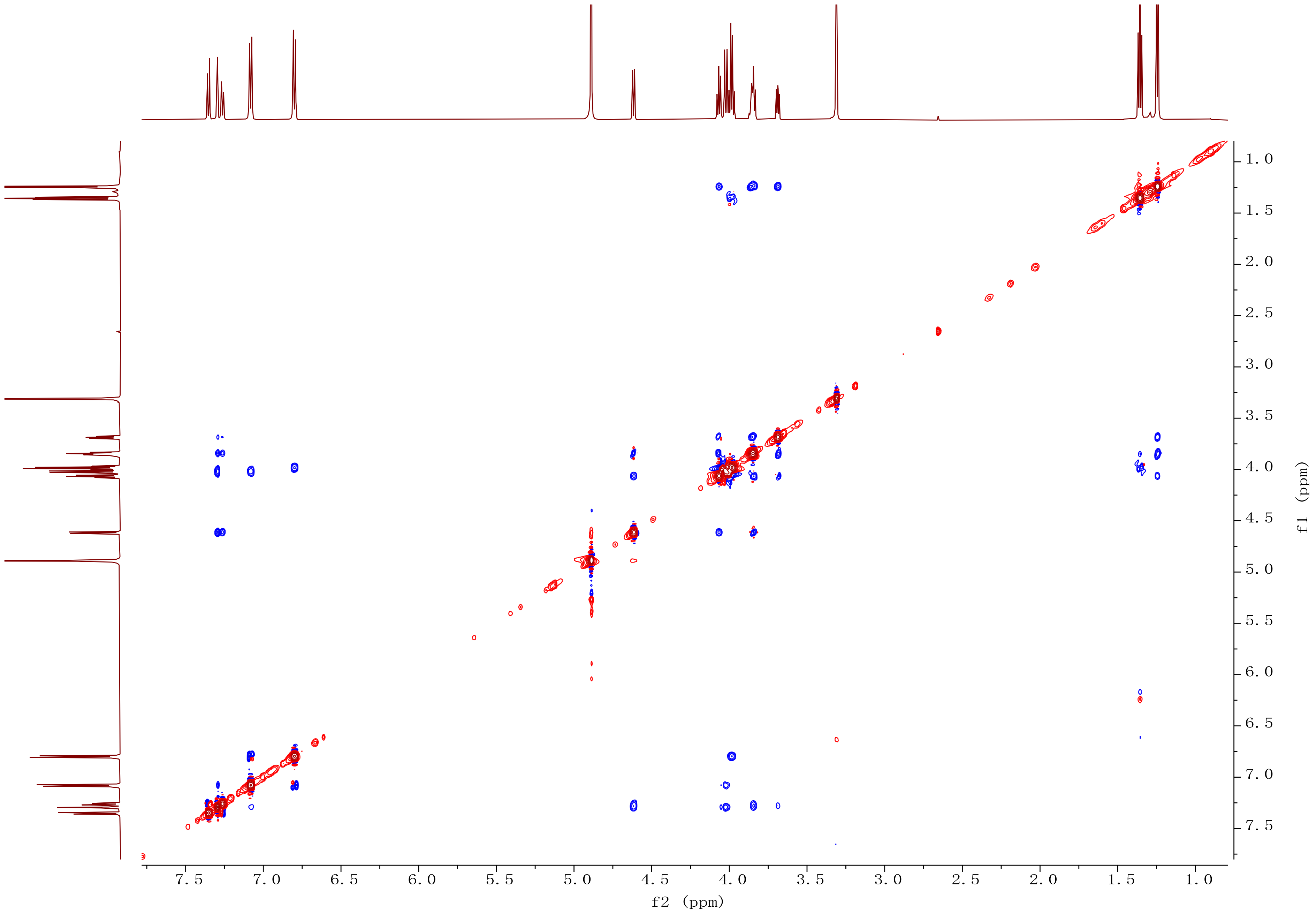

NOESY NMR of compound α–58

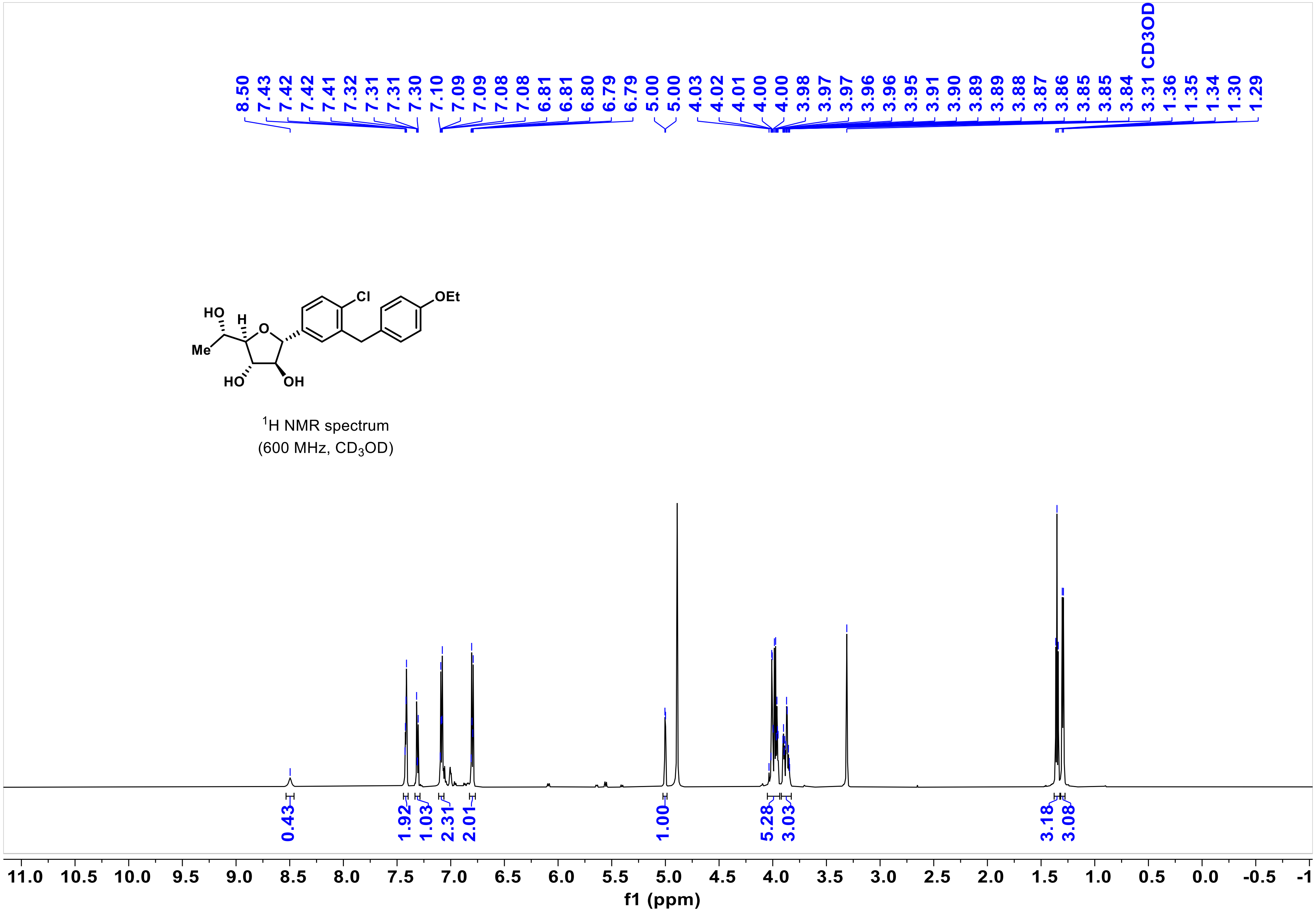

^{1}H NMR of compound β–58

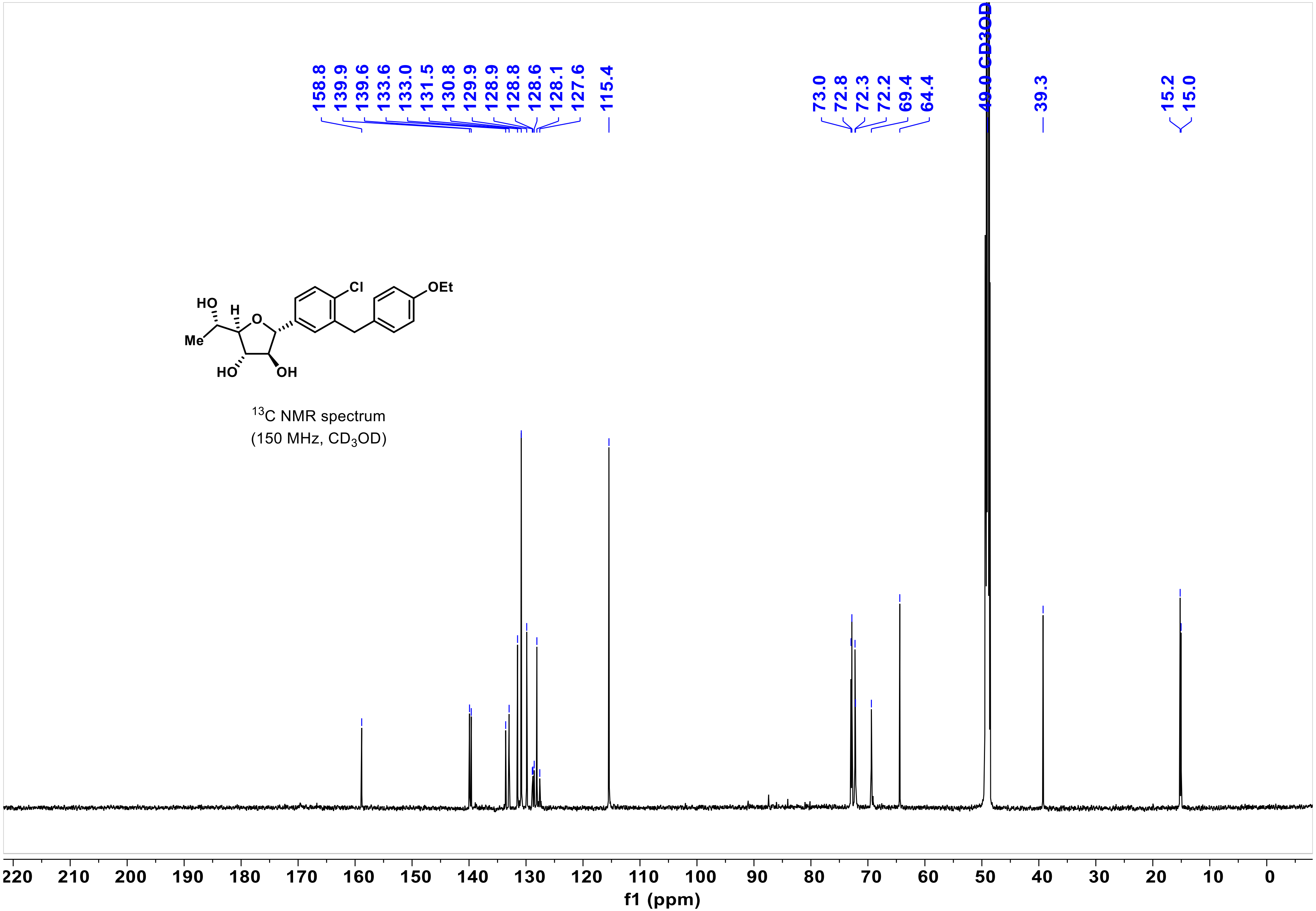

^{13}C NMR of compound β–58

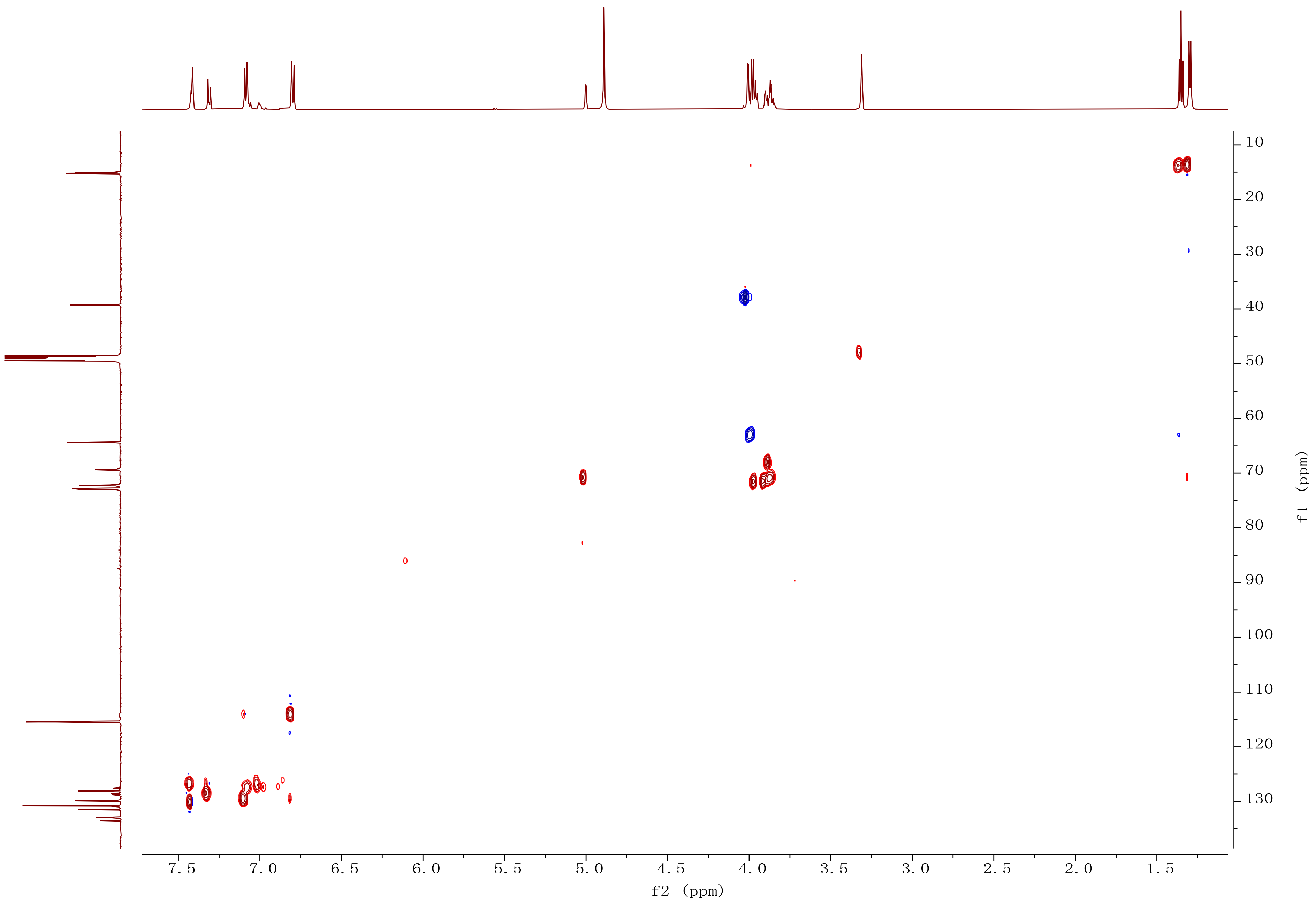

H-H HSQC NMR of compound **β–58**

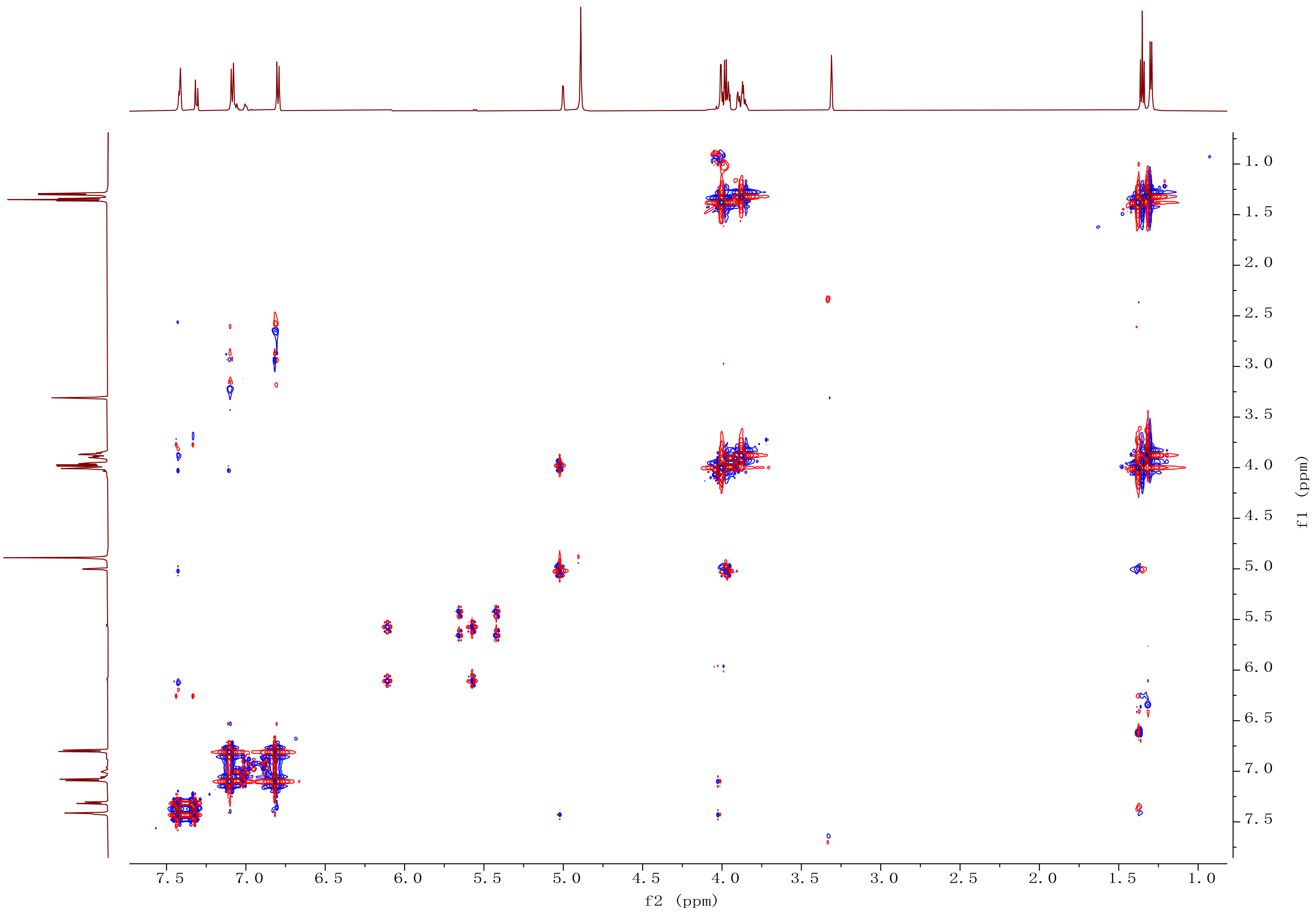

H-H COSY NMR of compound **β–58**

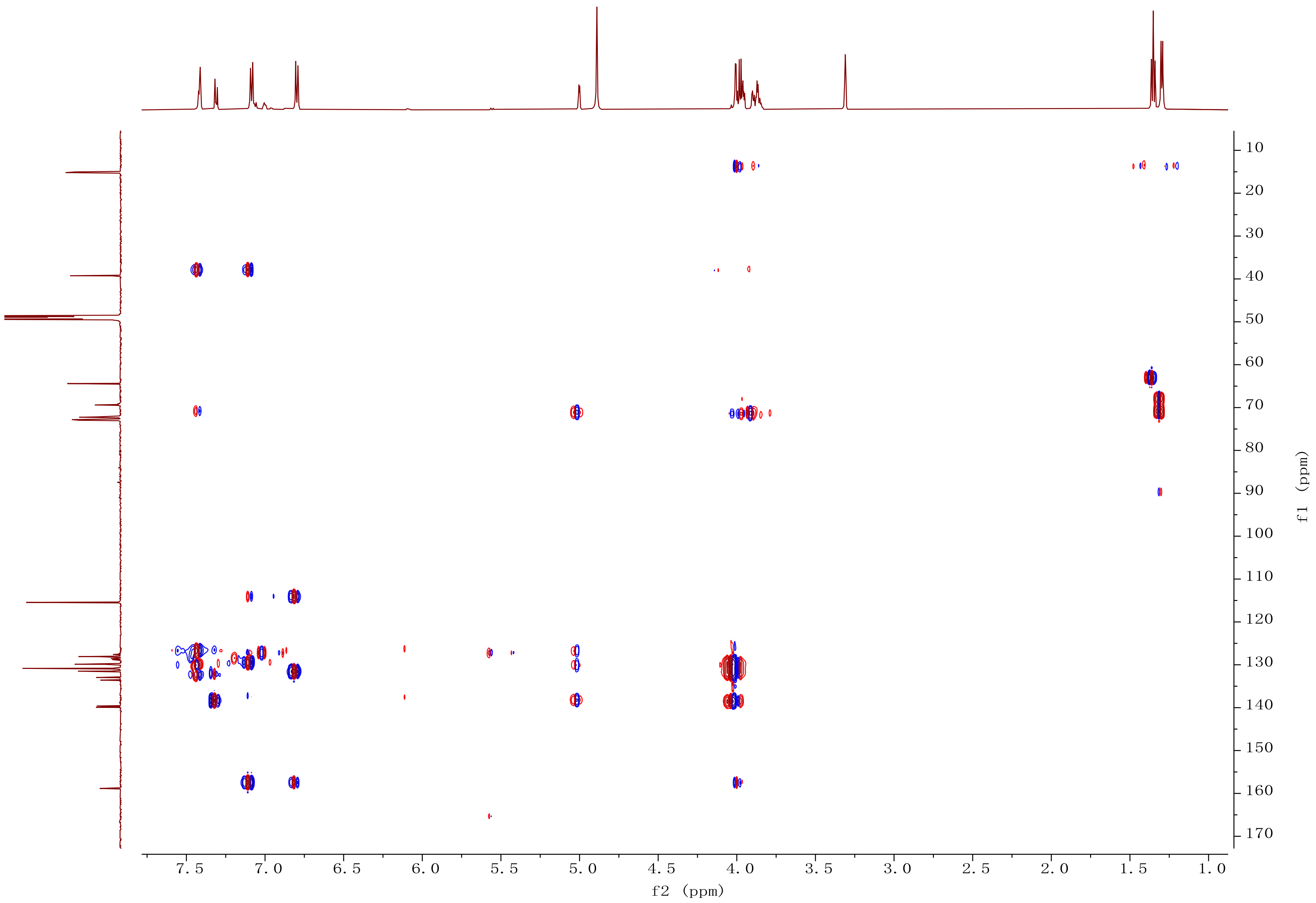

H-C HMBC NMR of compound **β–58**

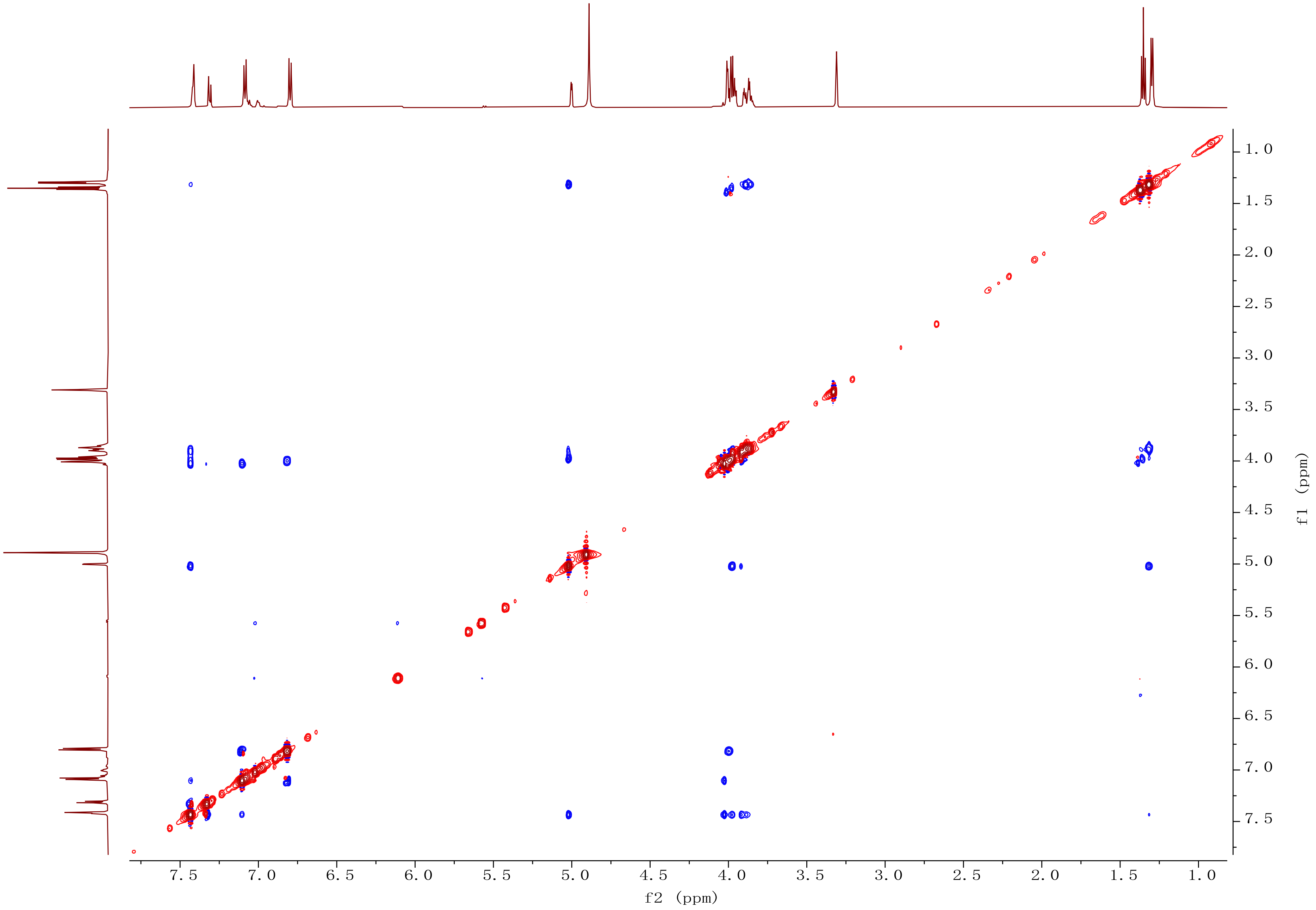

NOESY NMR of compound **β–58**

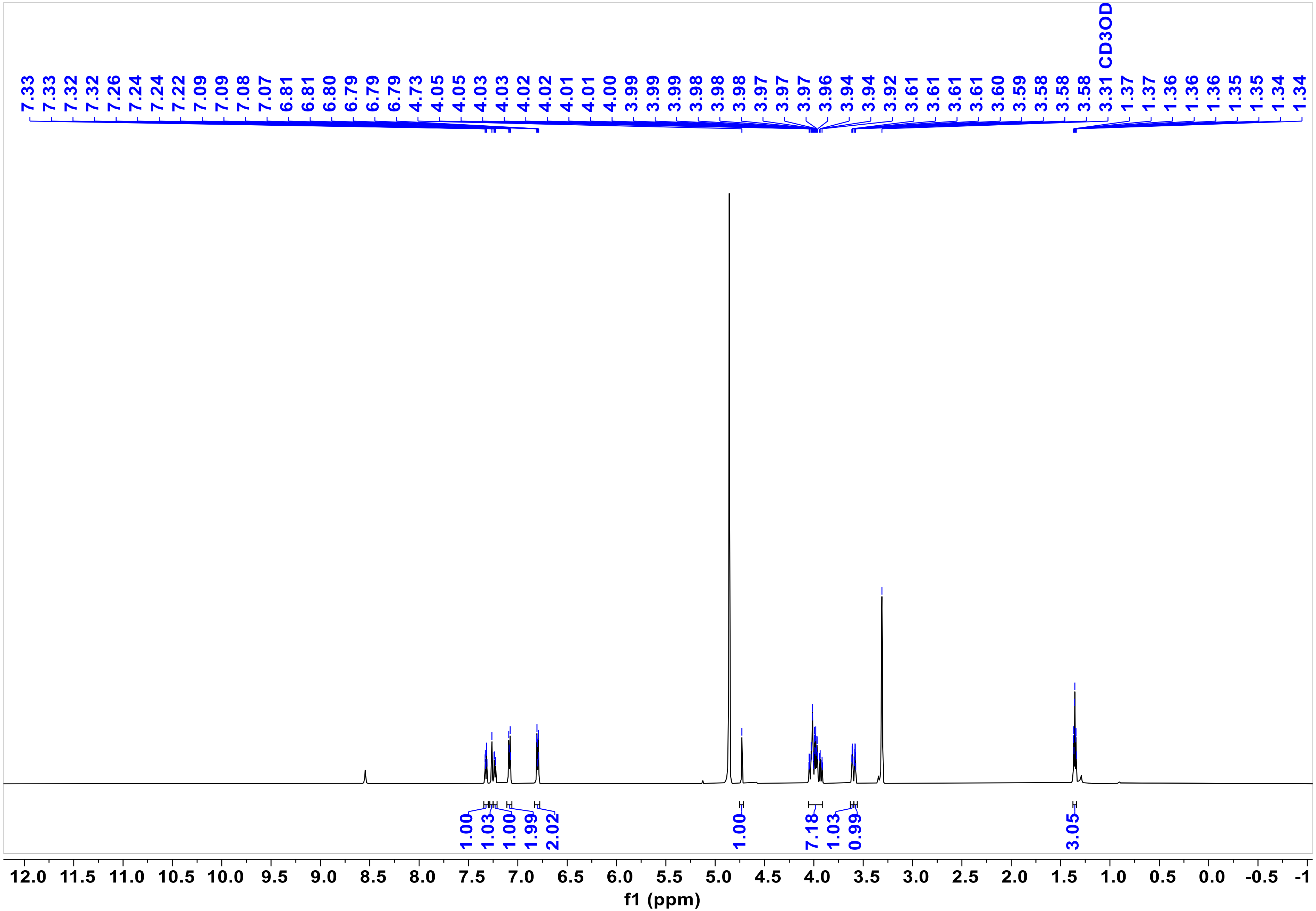

^{1}H NMR of compound α–65

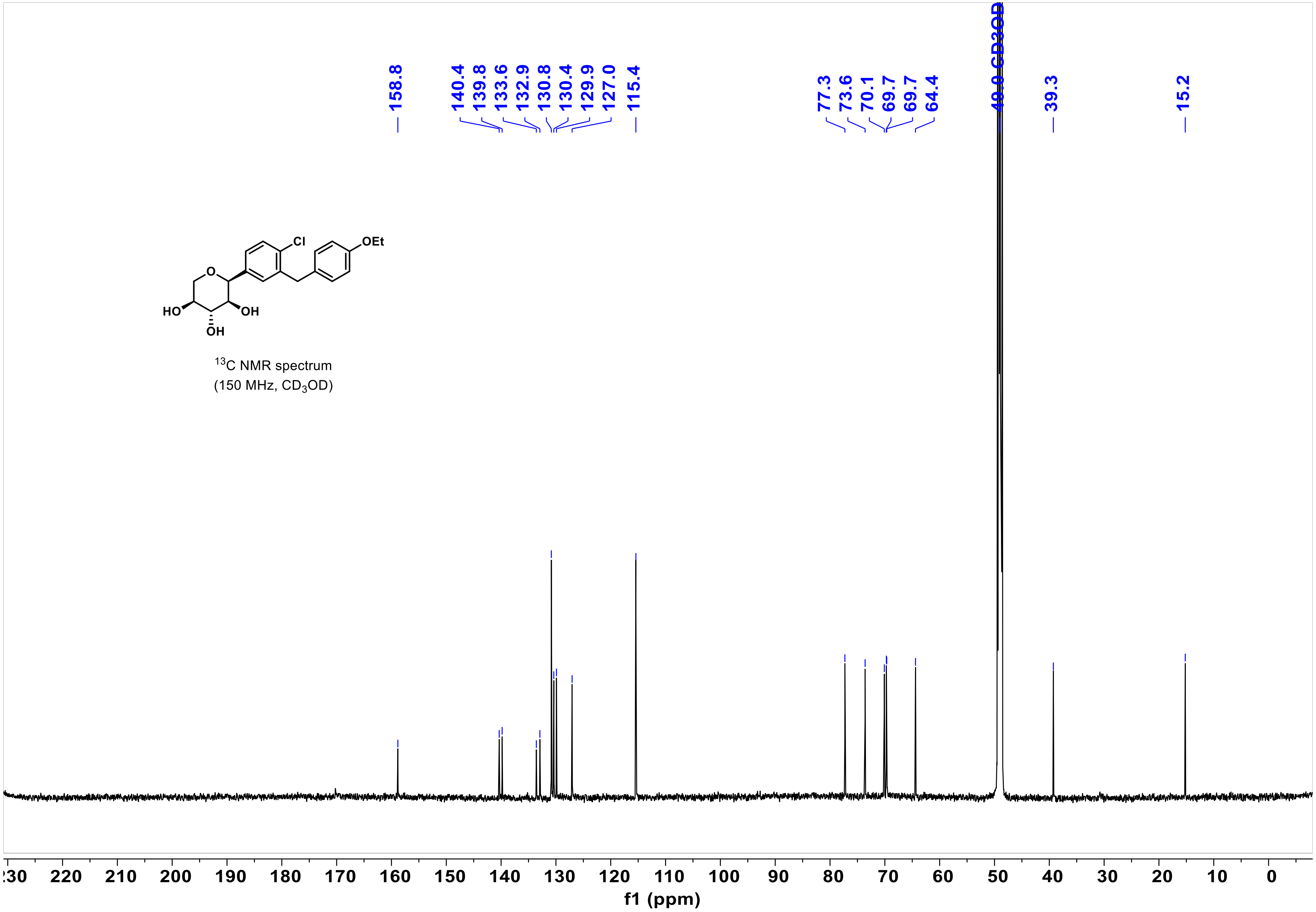

^{13}C NMR of compound α–65

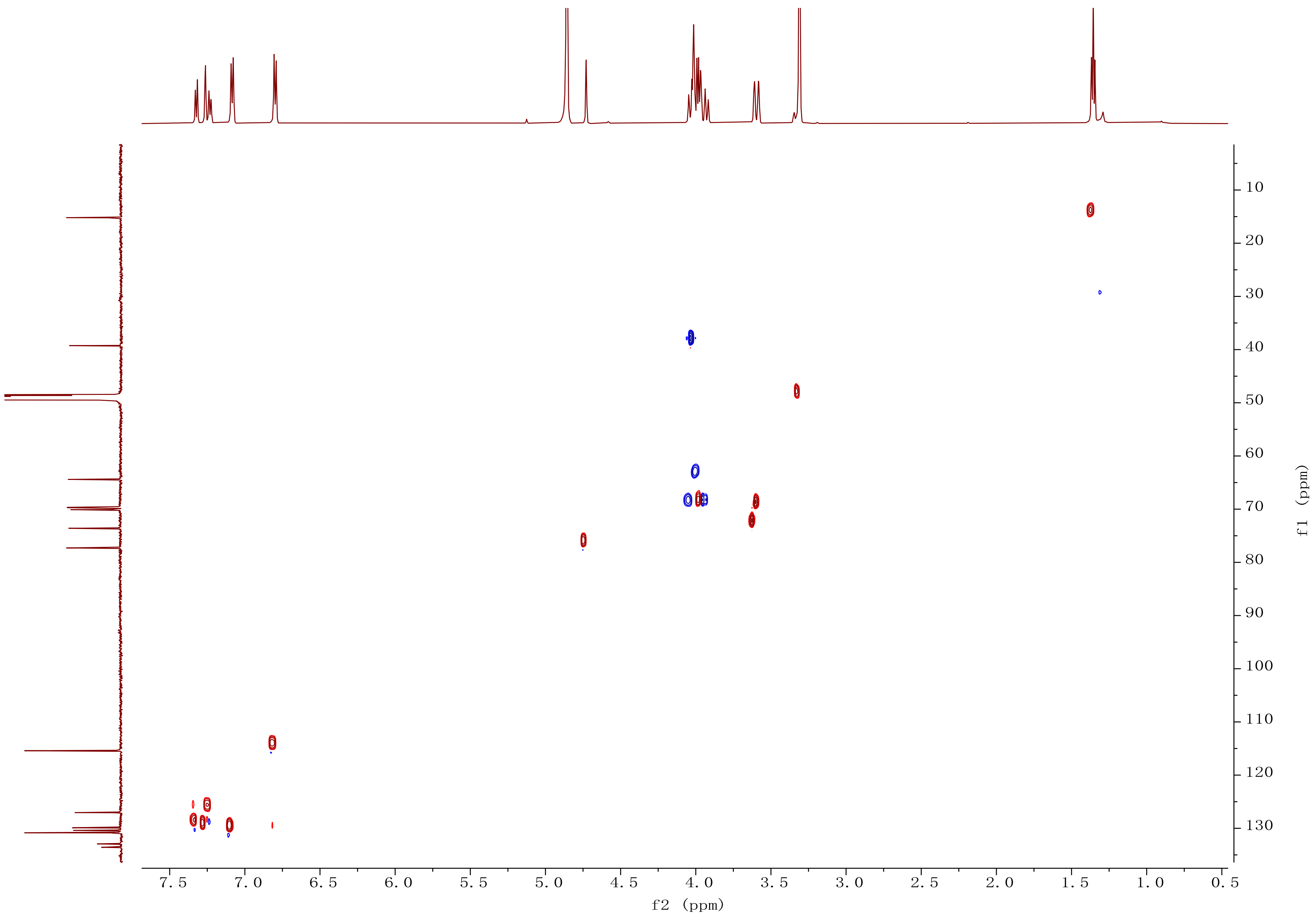

H-H HSQC NMR of compound α−65

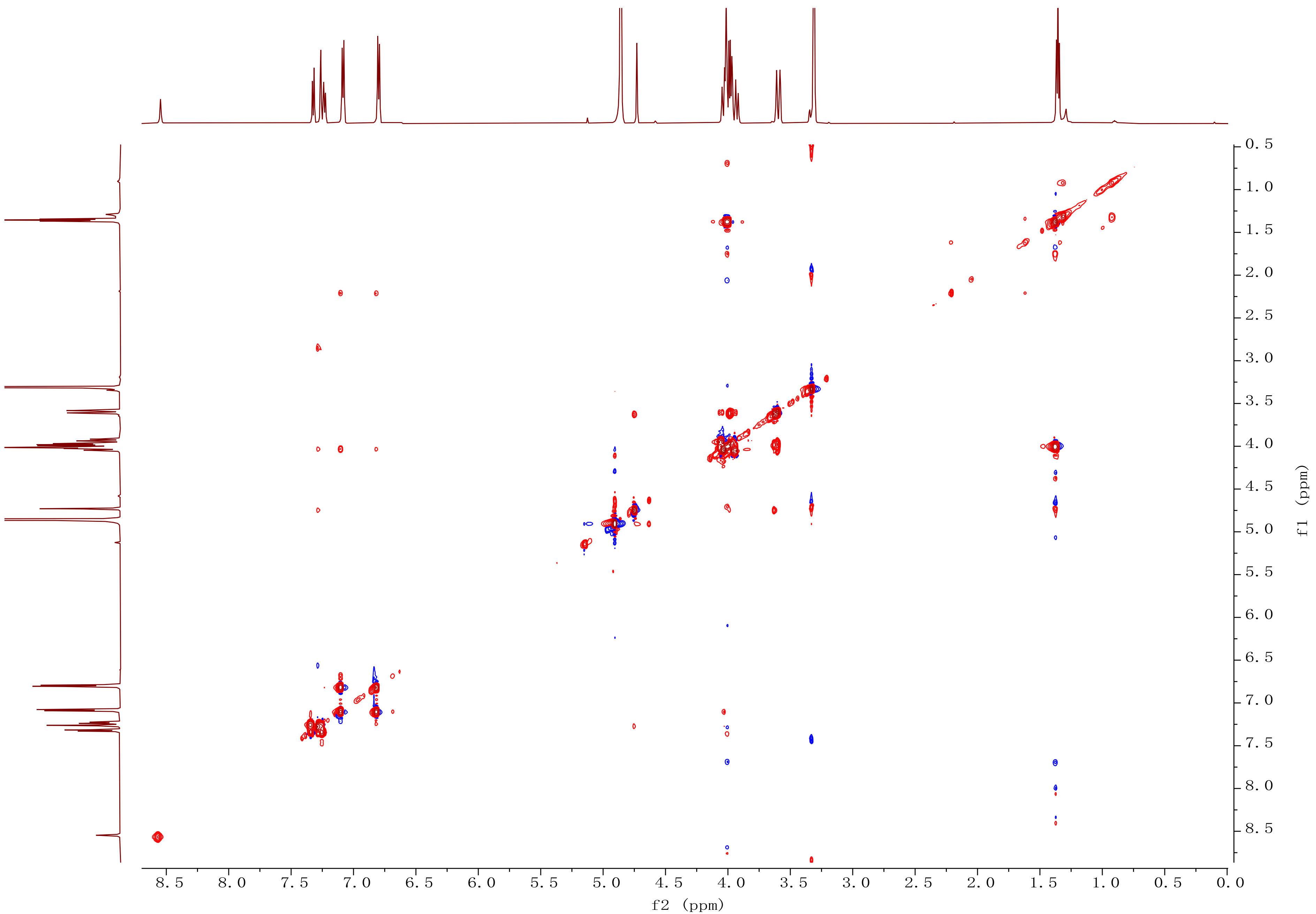

H-H COSY NMR of compound α–65

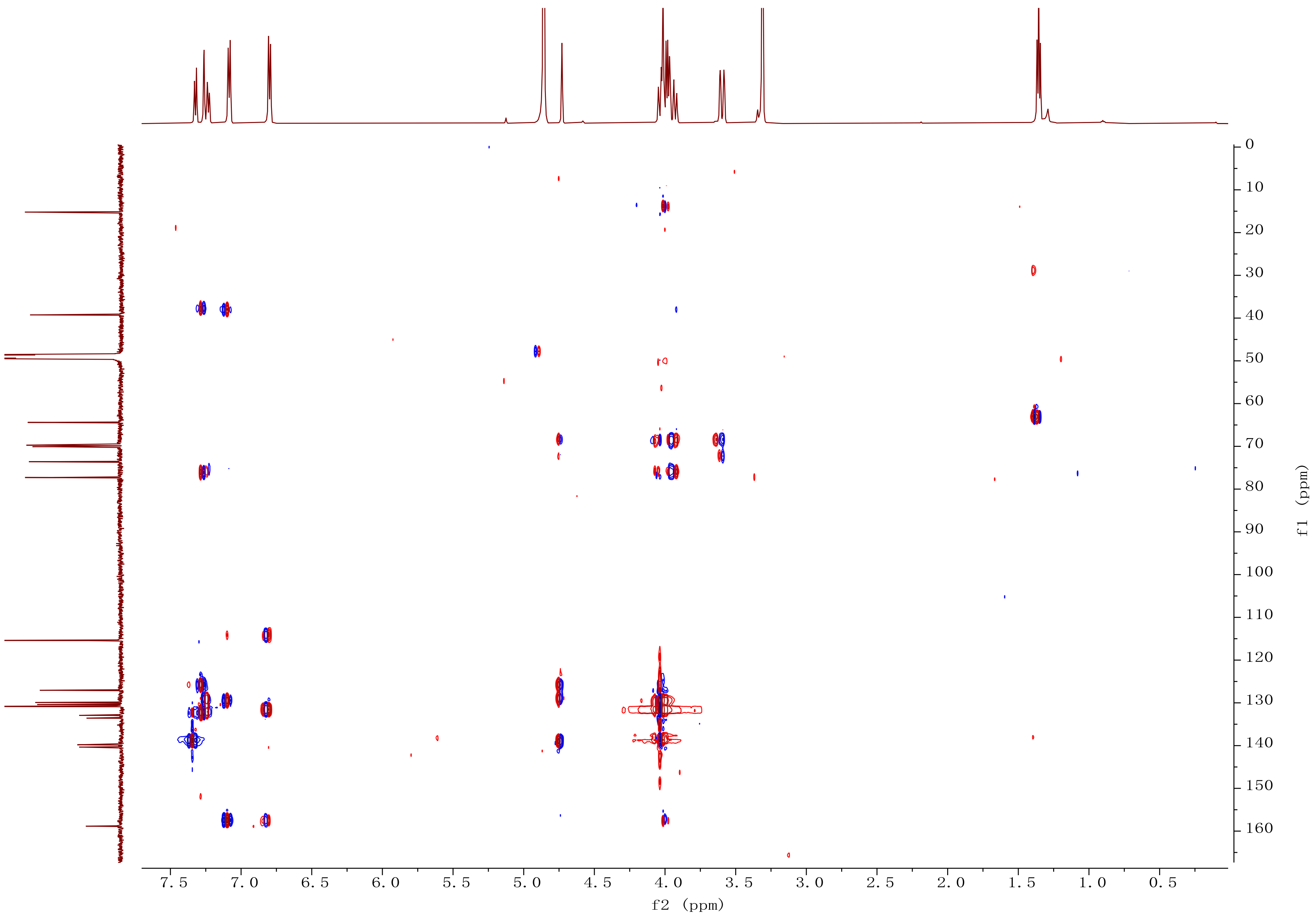

H-C HMBC NMR of compound **α–65**

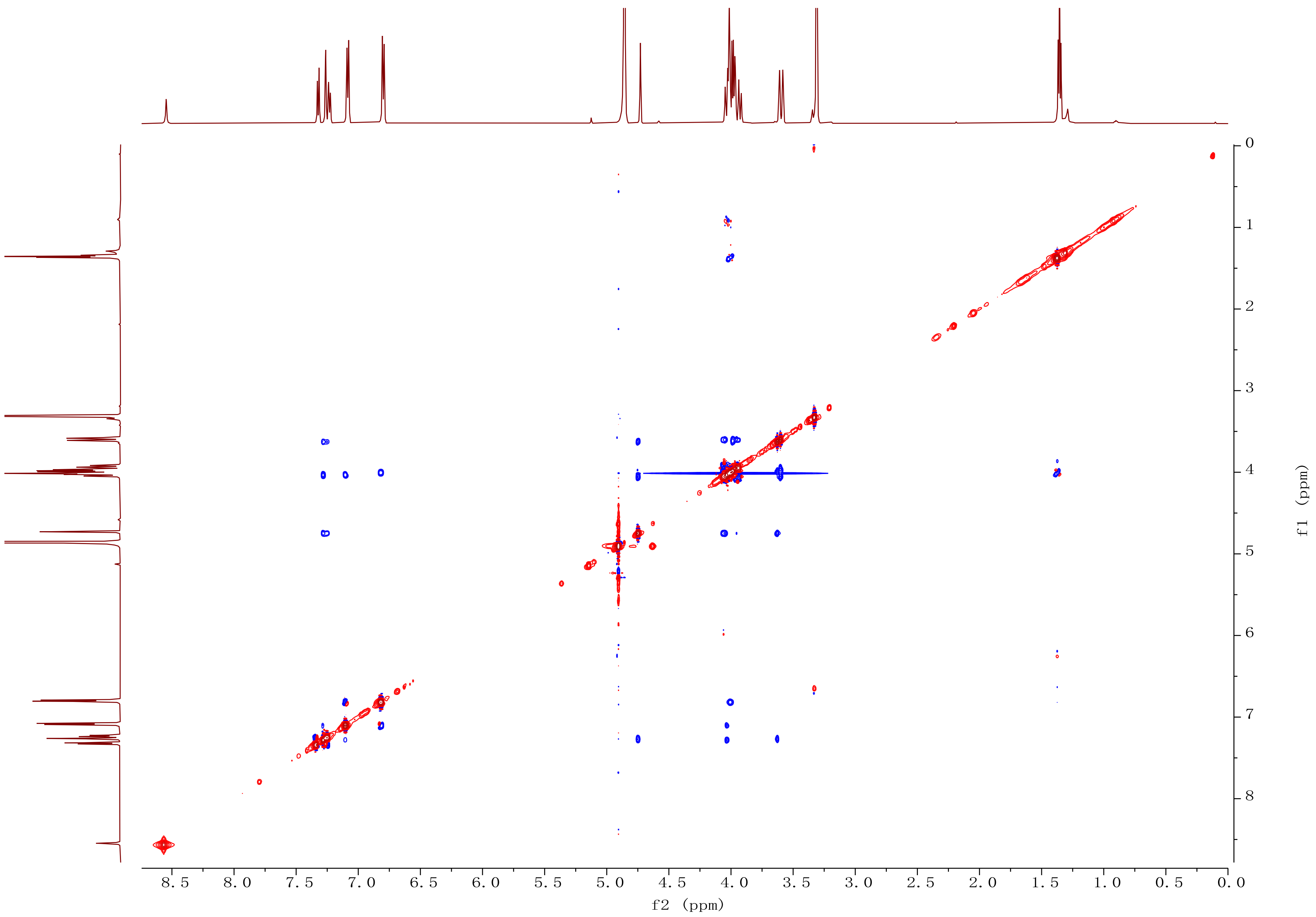

NOESY NMR of compound **α–65**

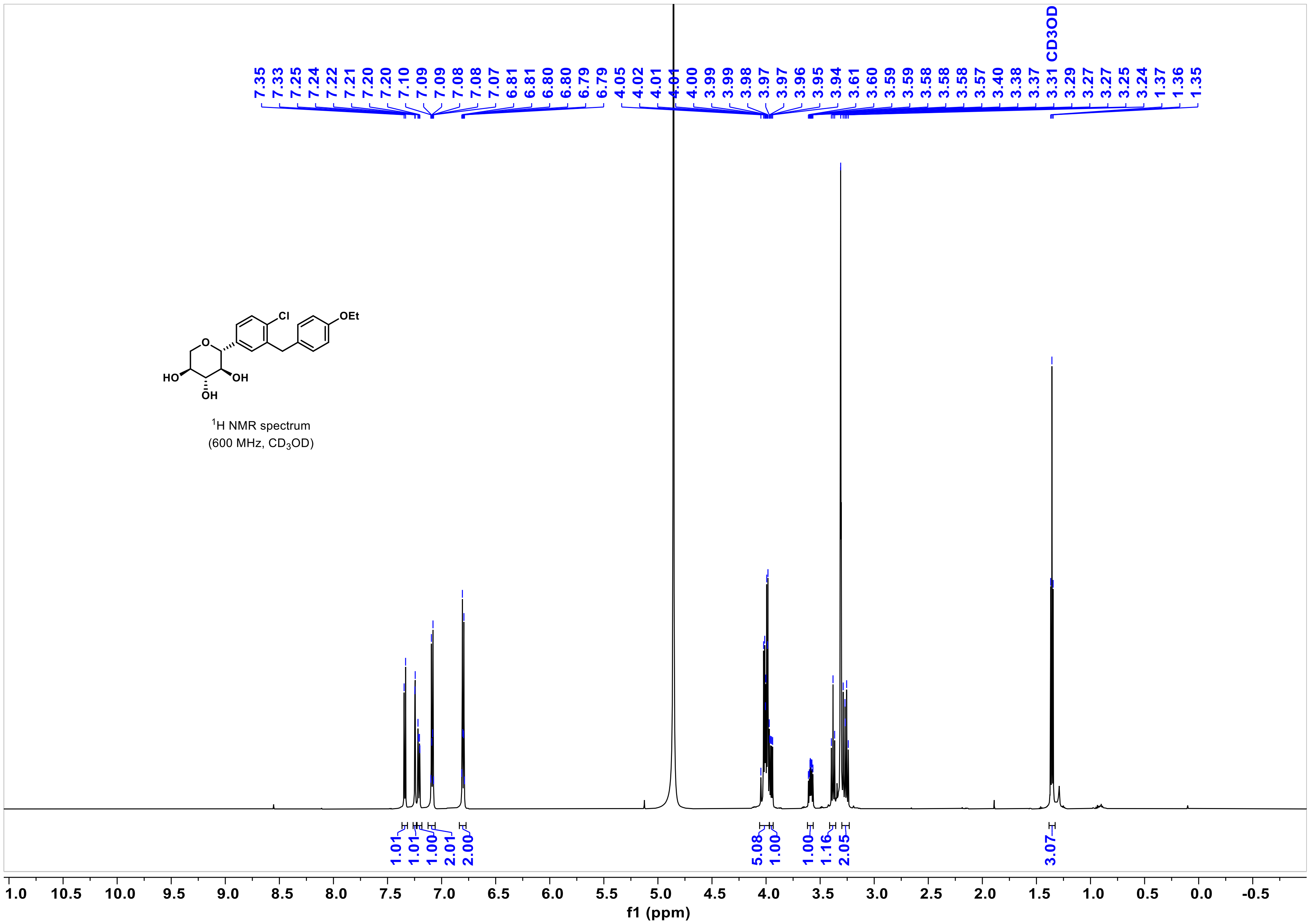

^{1}H NMR of compound β–65

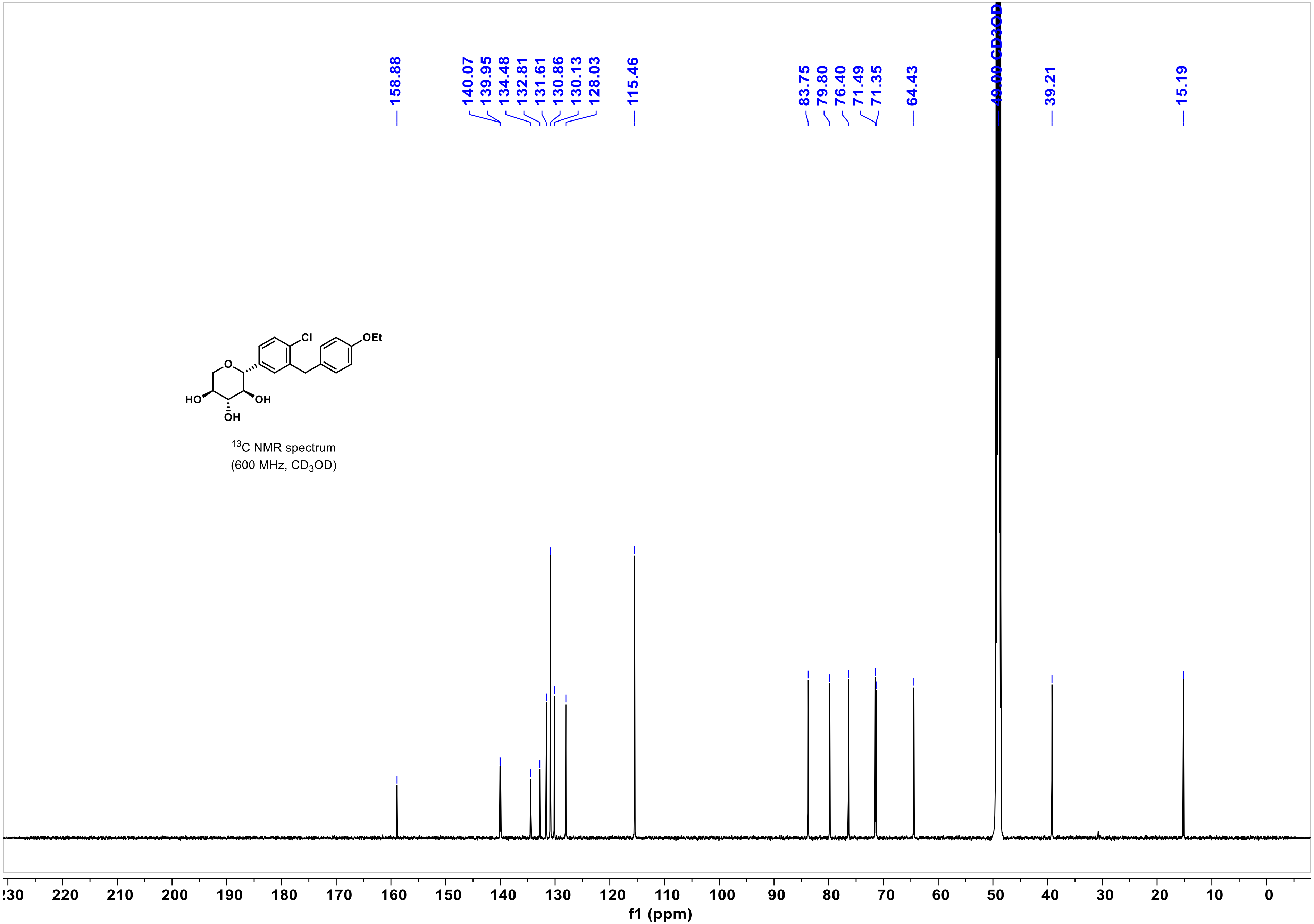

^{13}C NMR of compound **β–65**

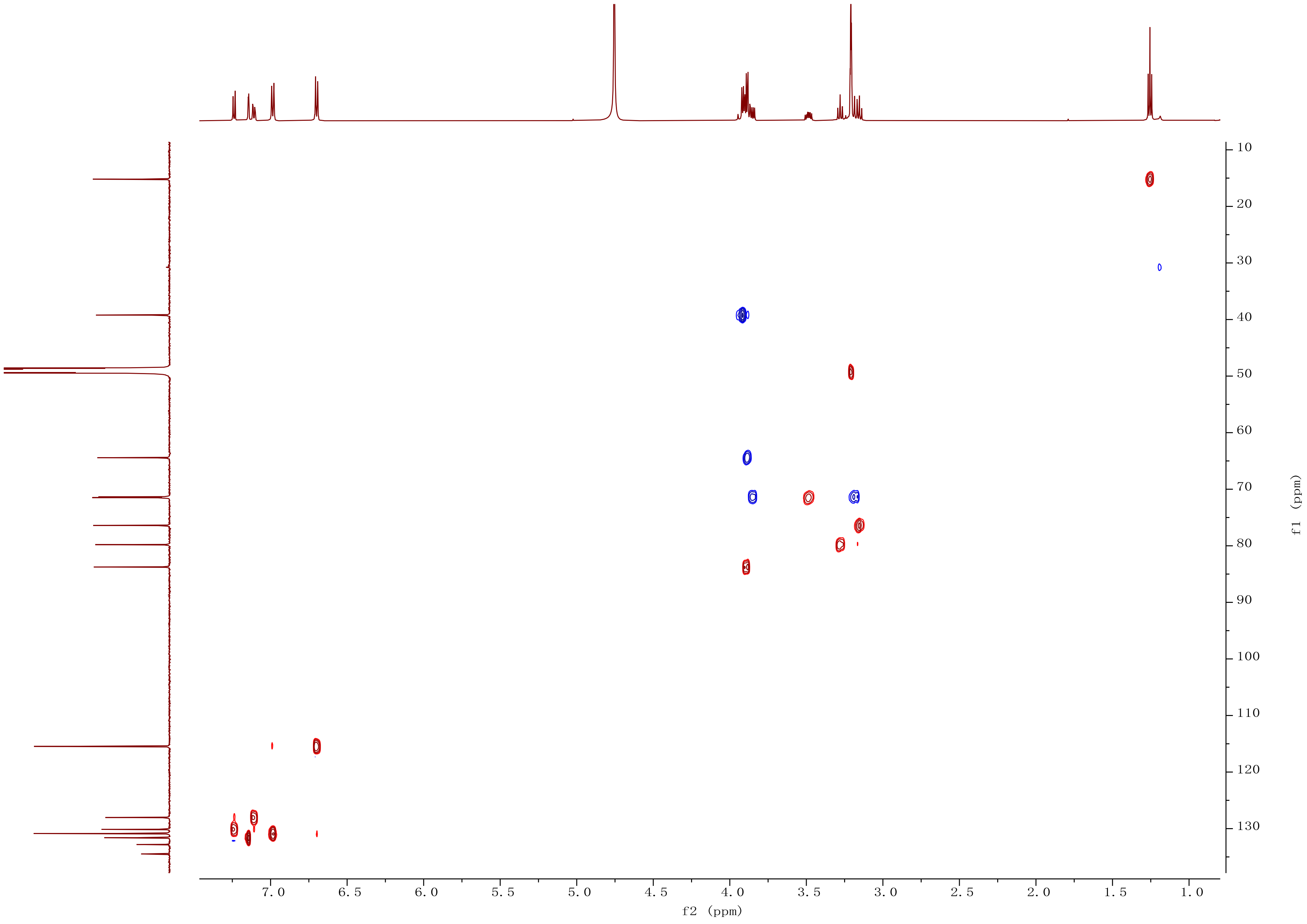

H-H HSQC NMR of compound **β–65**

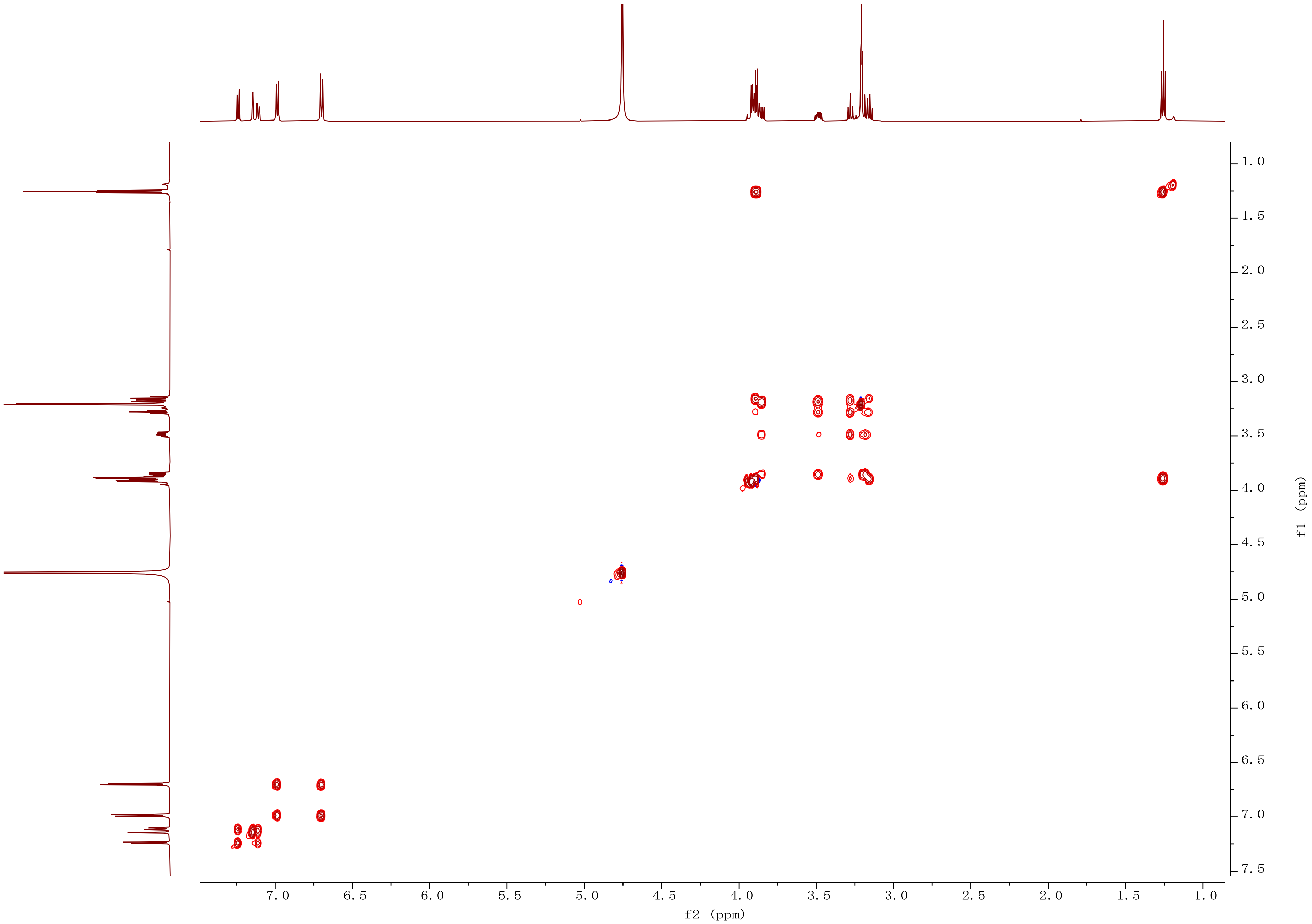

H-H COSY NMR of compound **β–65**

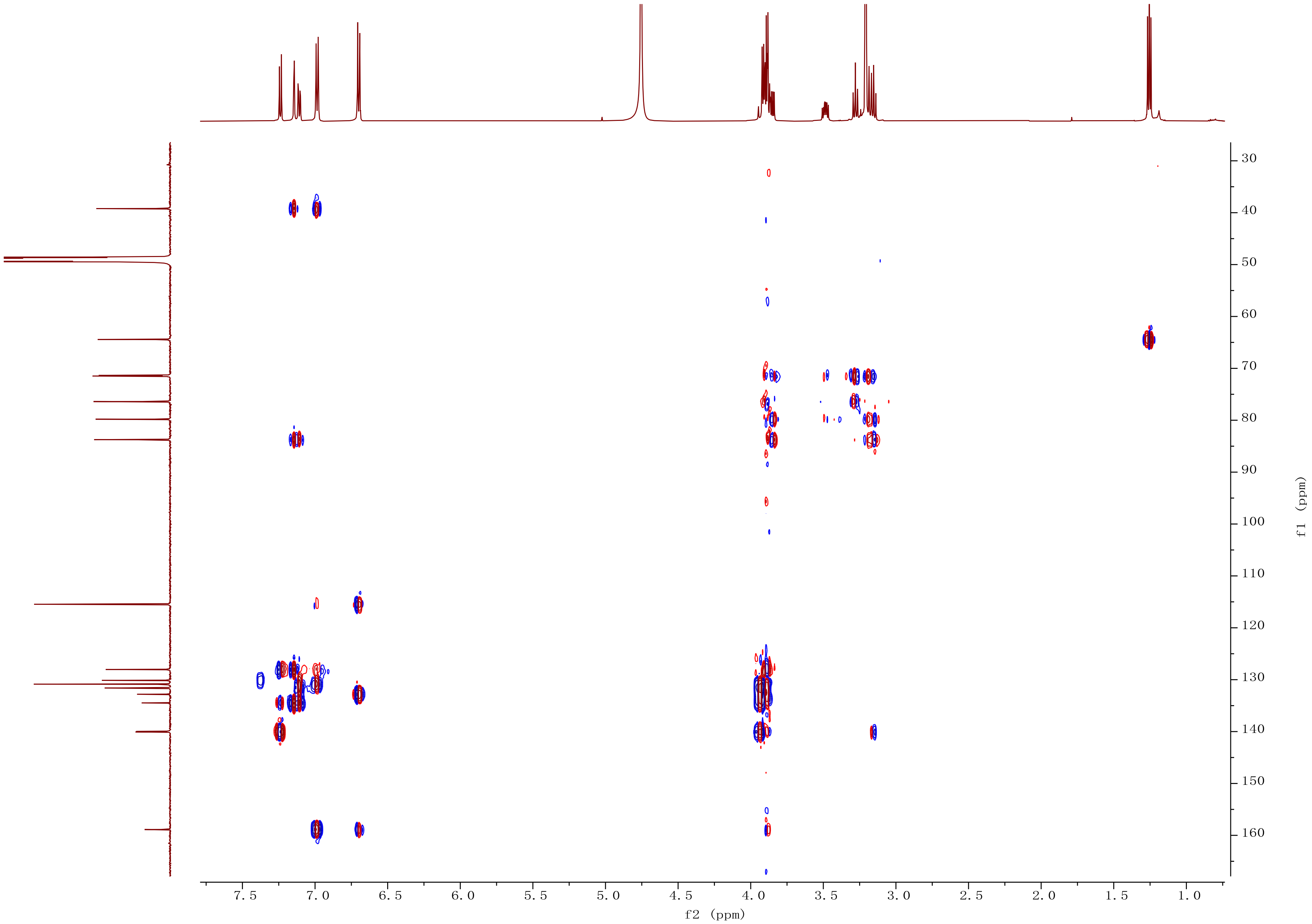

H-C HMBC NMR of compound **β–65**

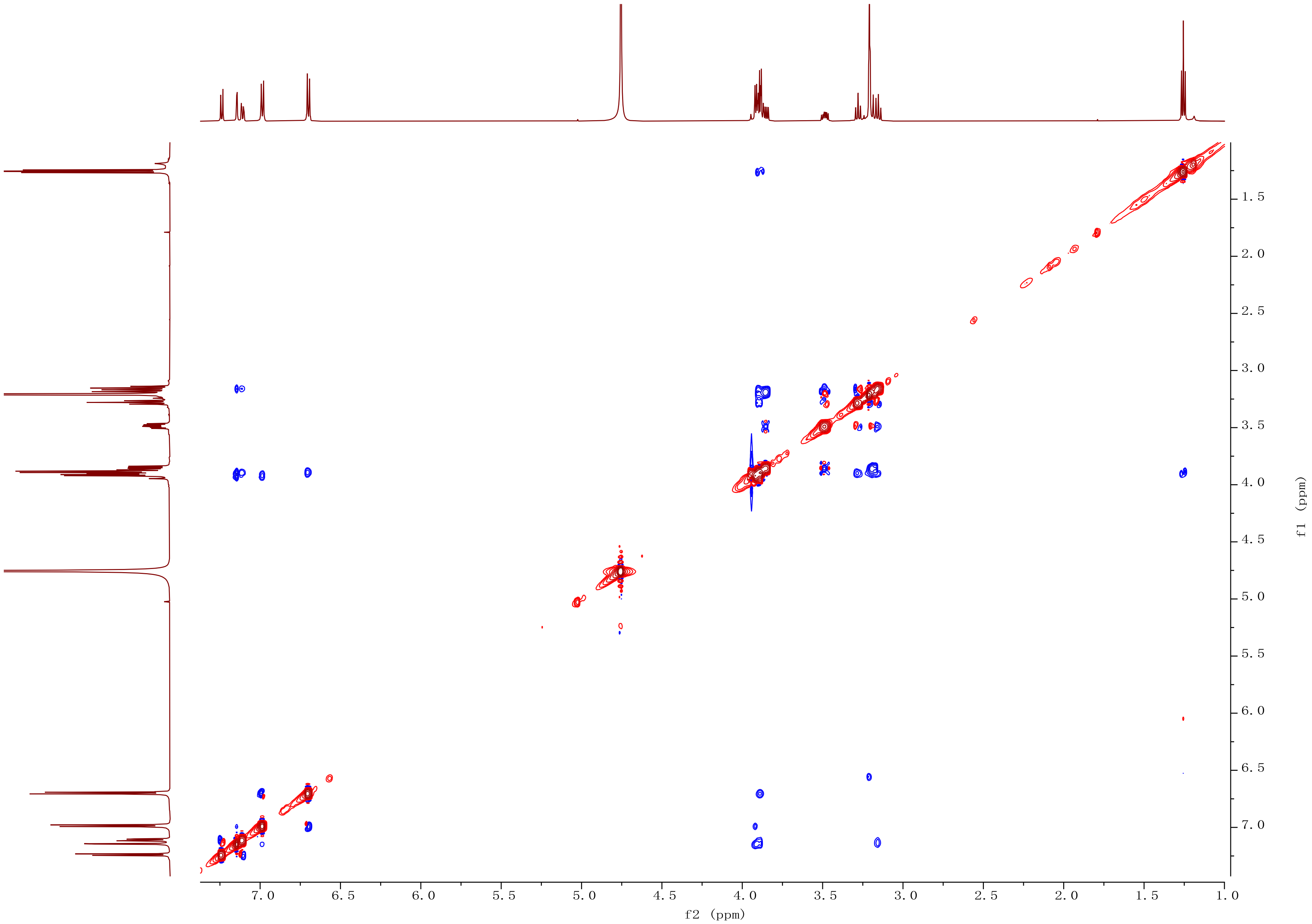

NOESY NMR of compound **β–65**

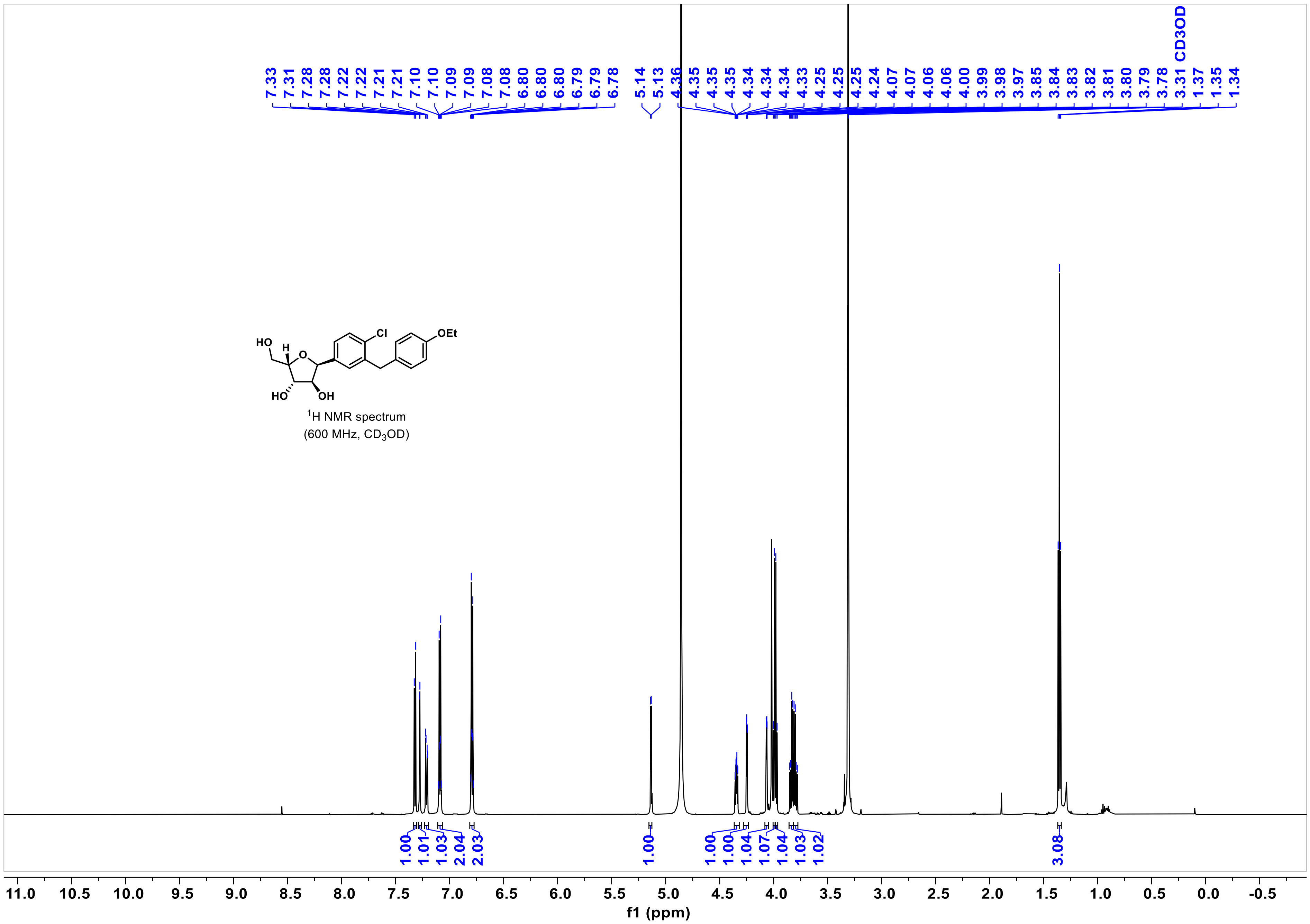

^{1}H NMR of compound α–66

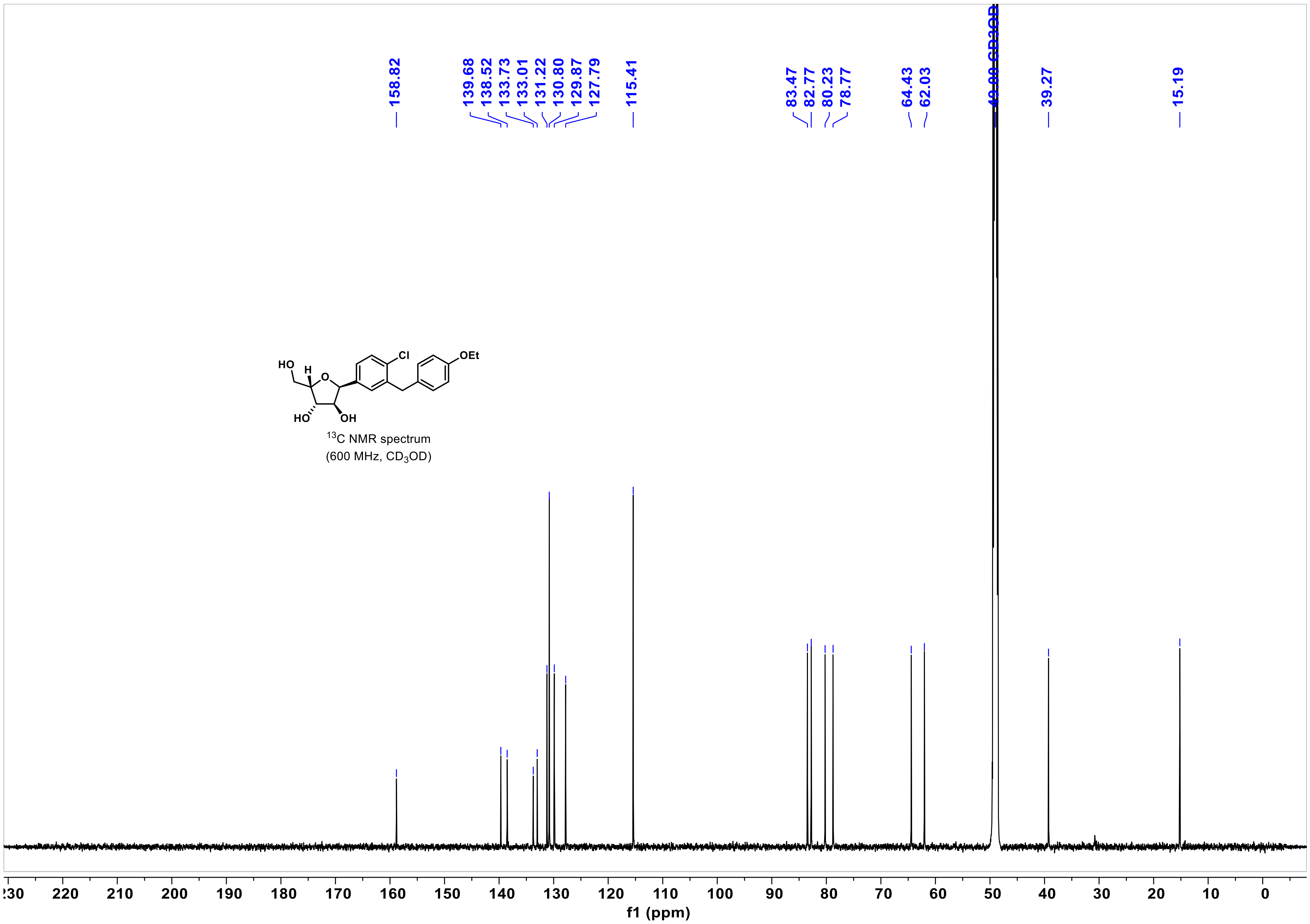

^{13}C NMR of compound α–66

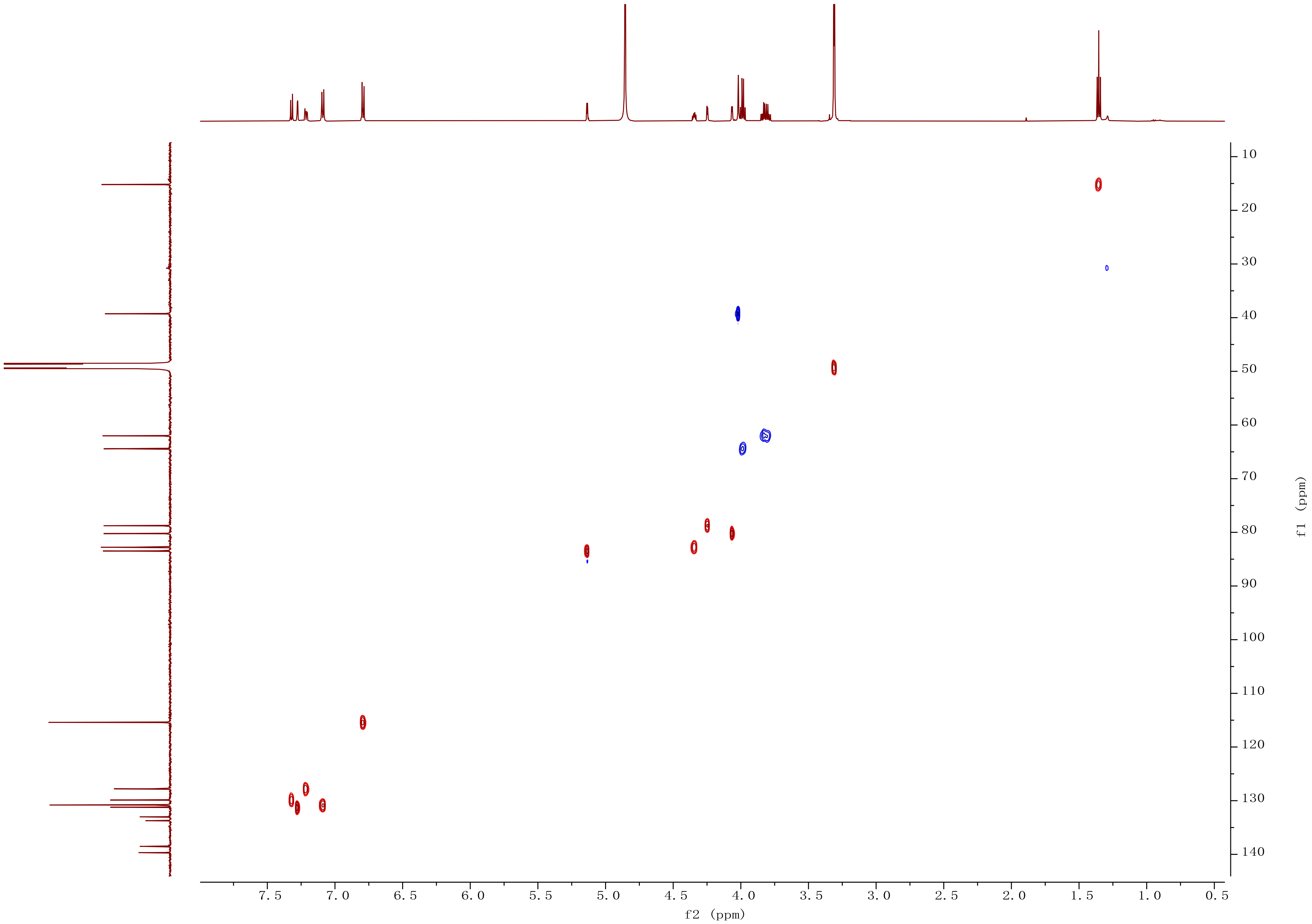

H-H HSQC NMR of compound α–66

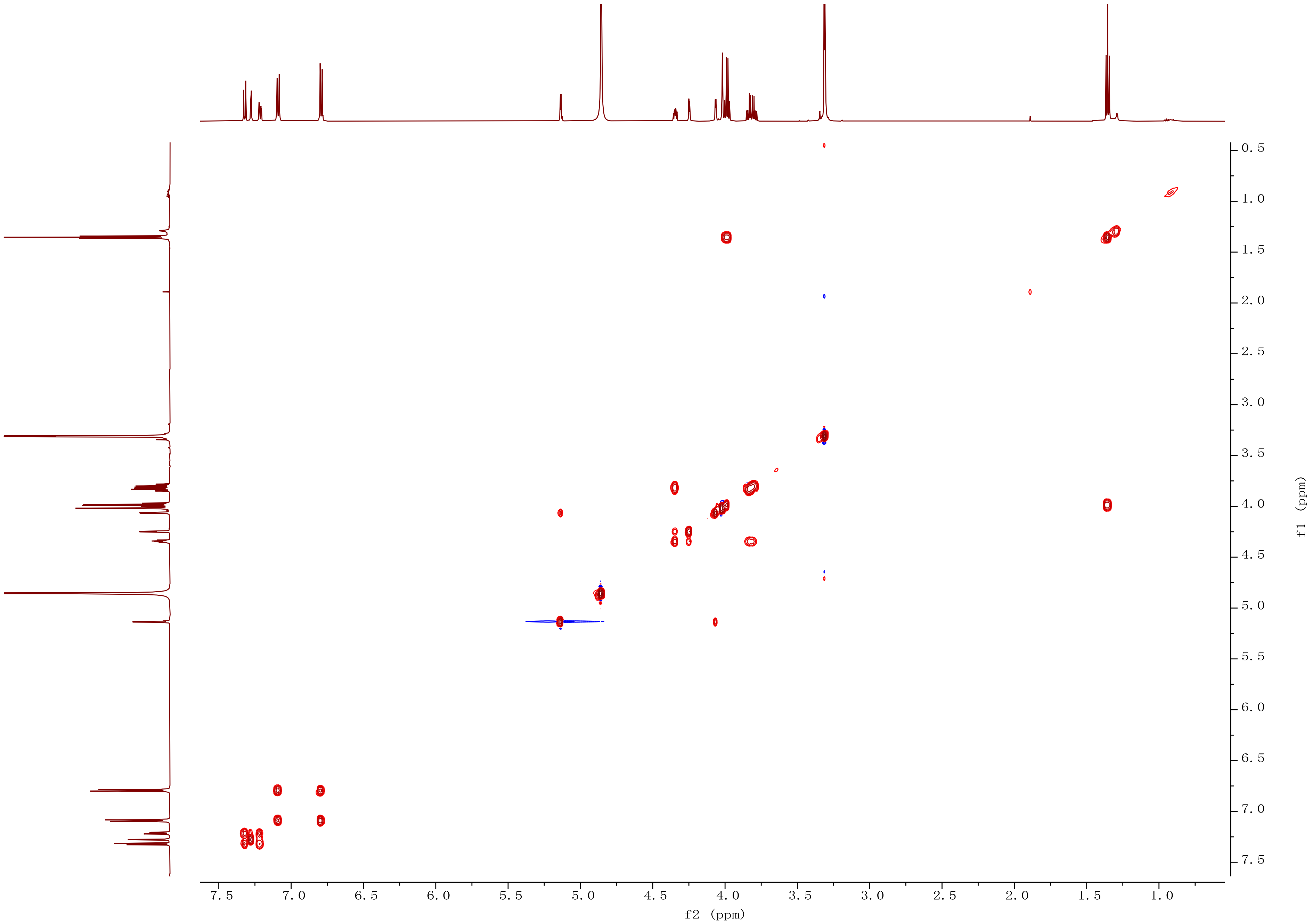

H-H COSY NMR of compound **α–66**

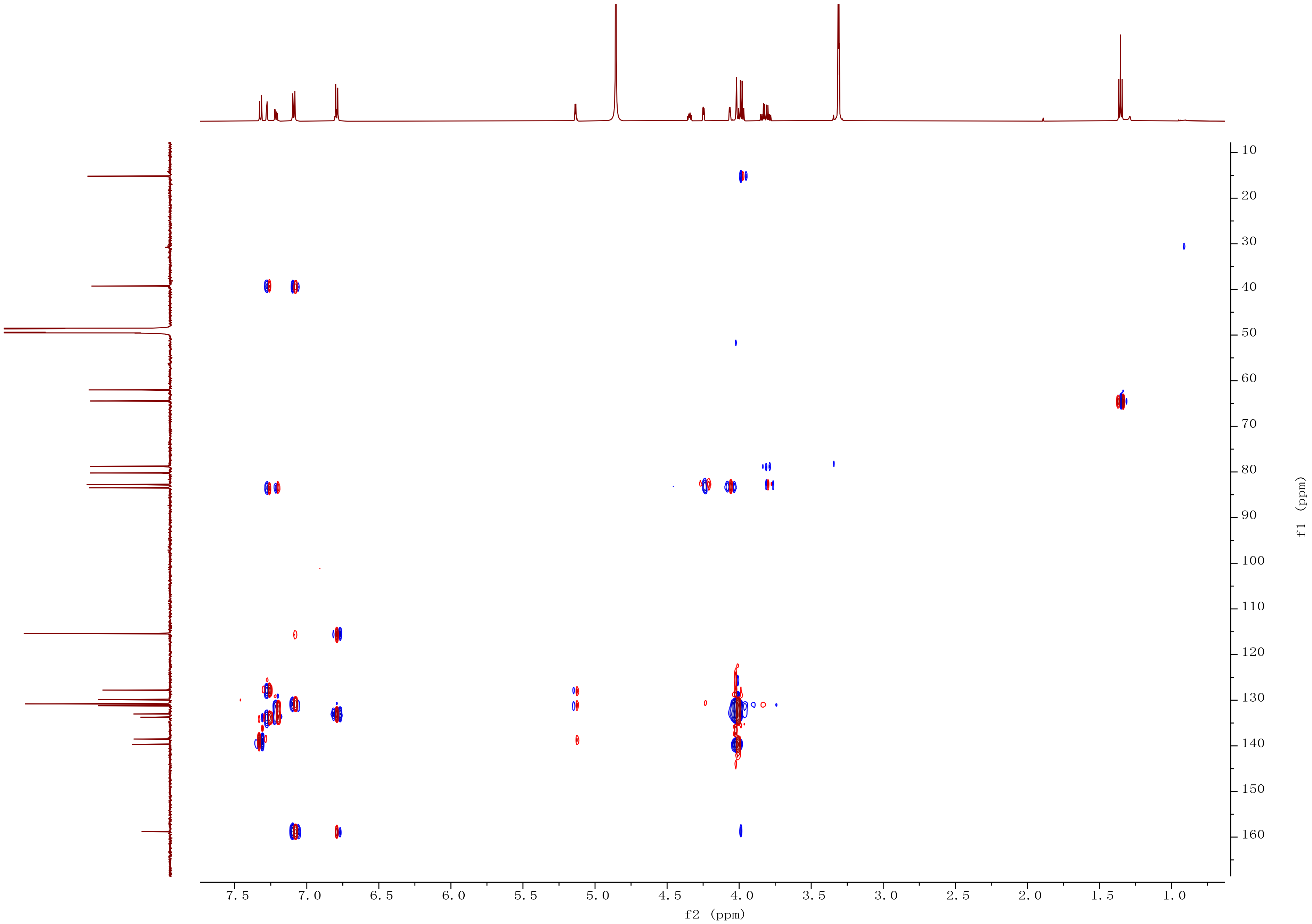

H-C HMBC NMR of compound **α–66**

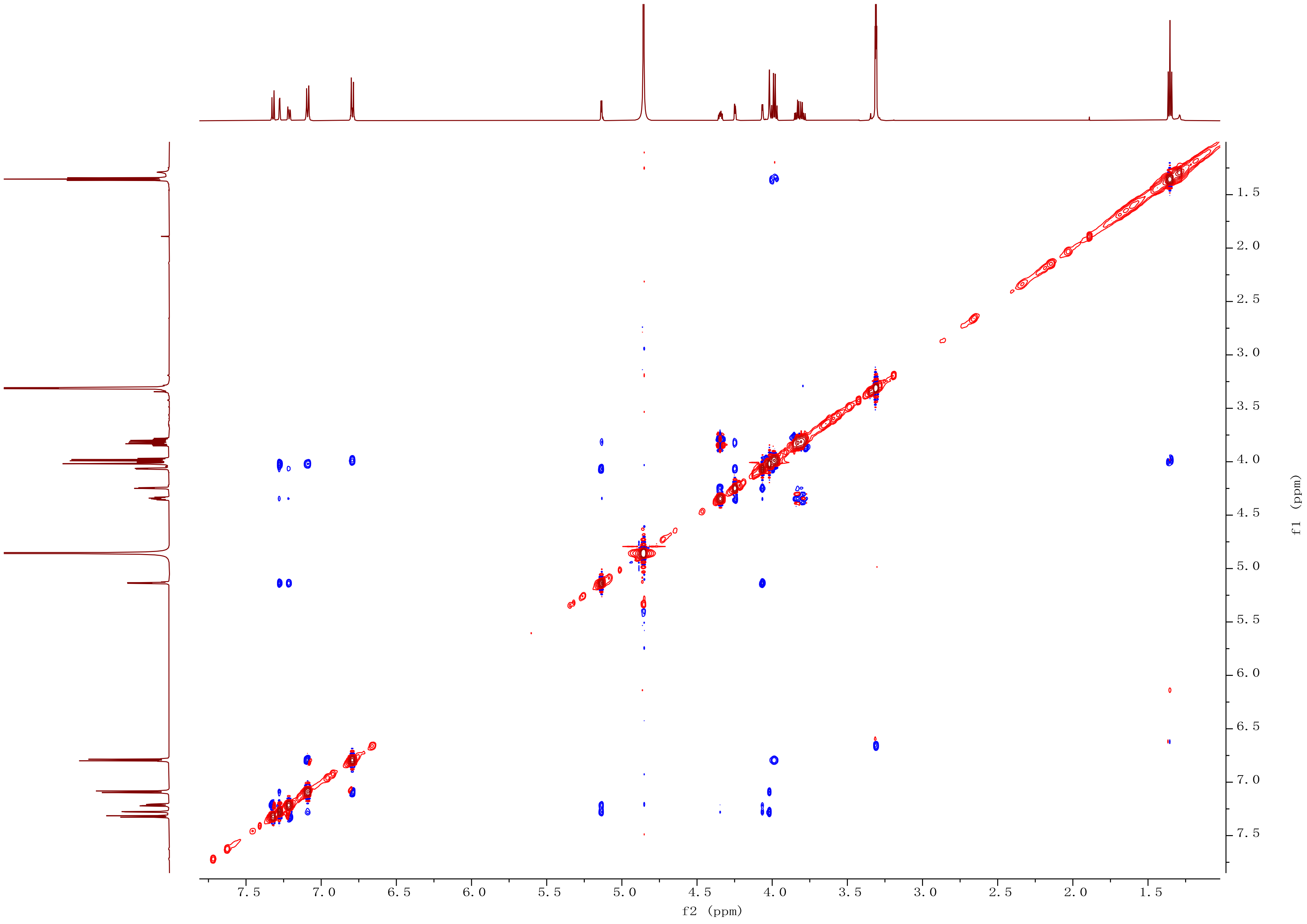

NOESY NMR of compound α–66

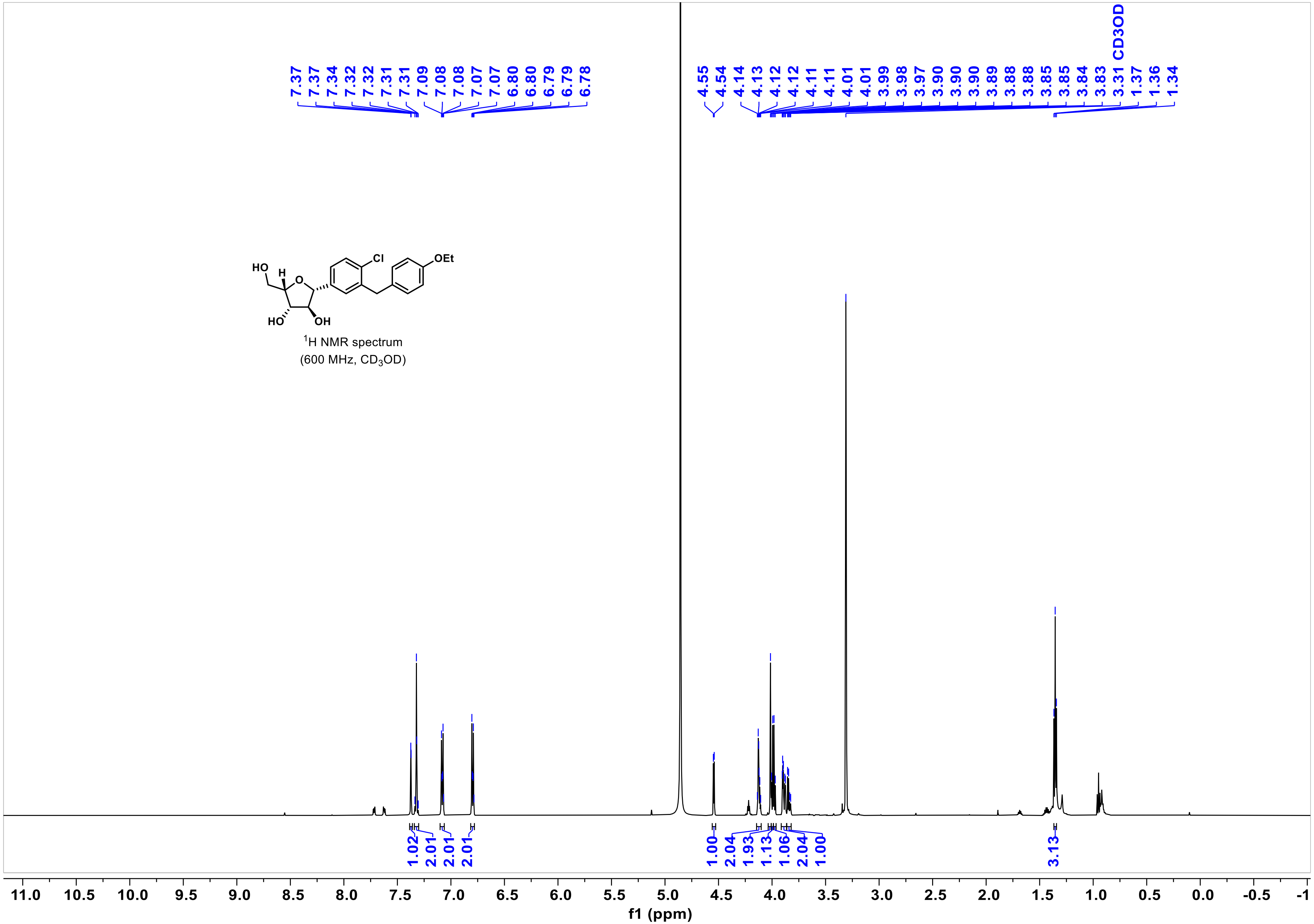

^{1}H NMR of compound **β–66**

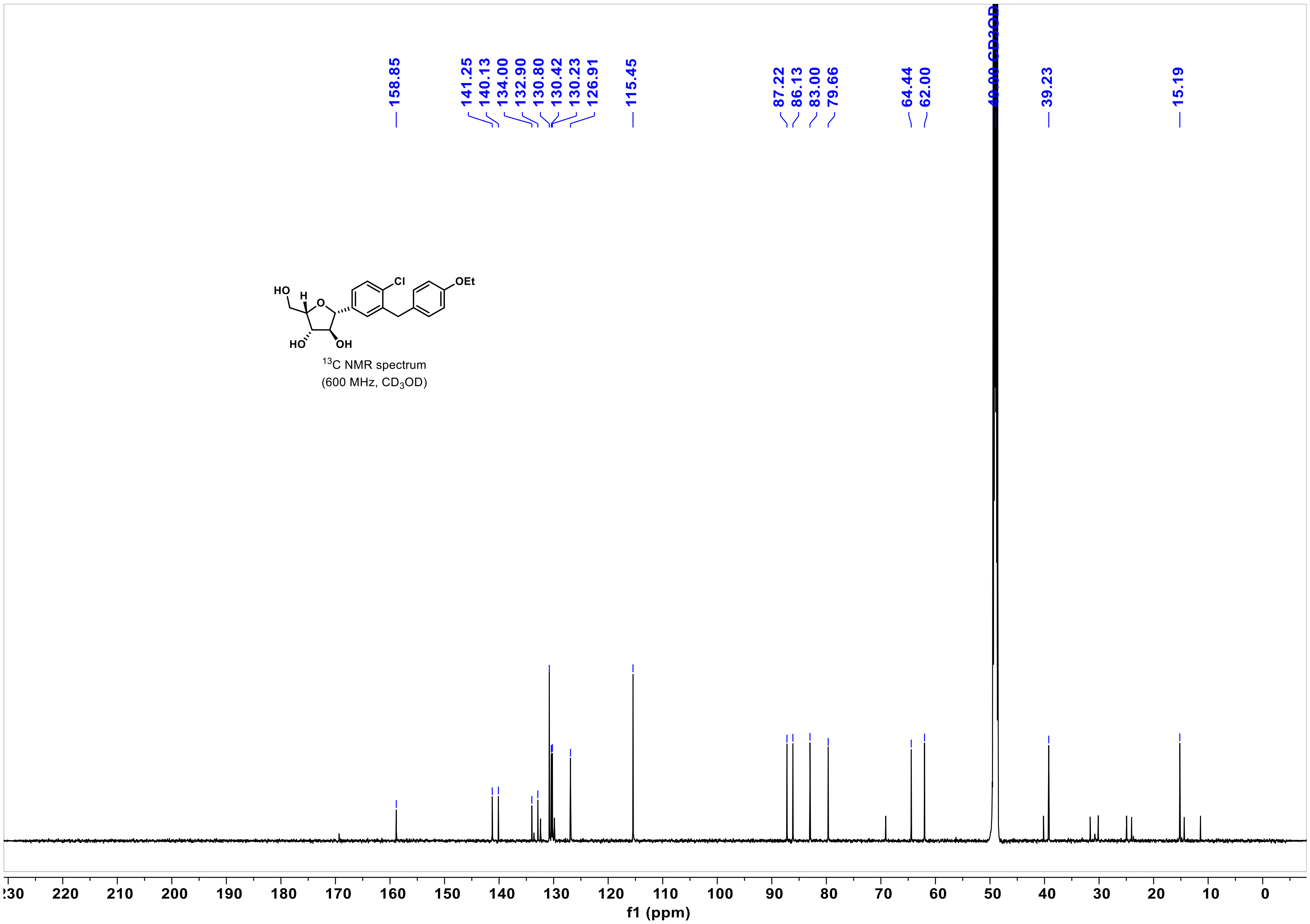

^{13}C NMR of compound **β–66**

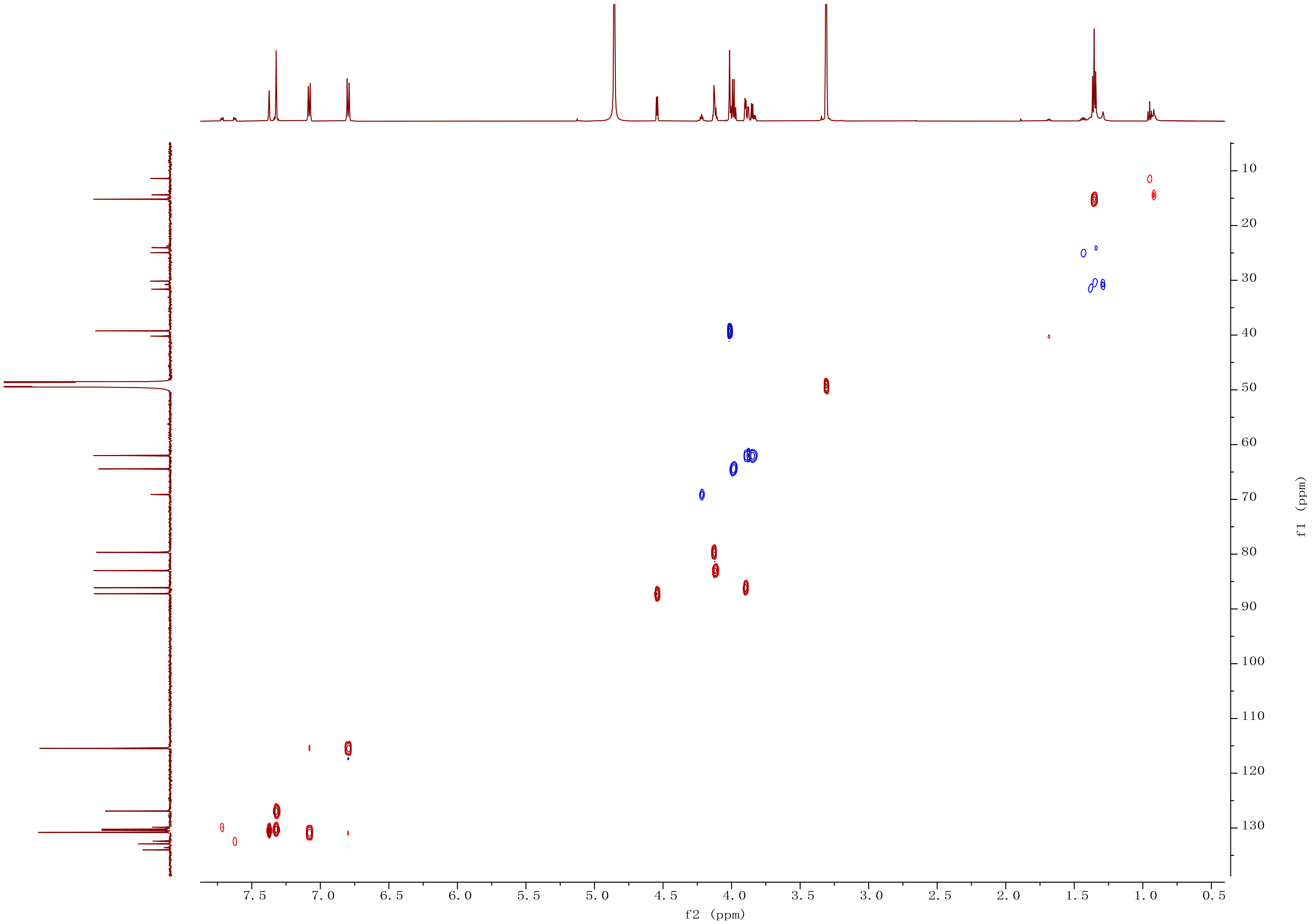

H-H HSQC NMR of compound **β–66**

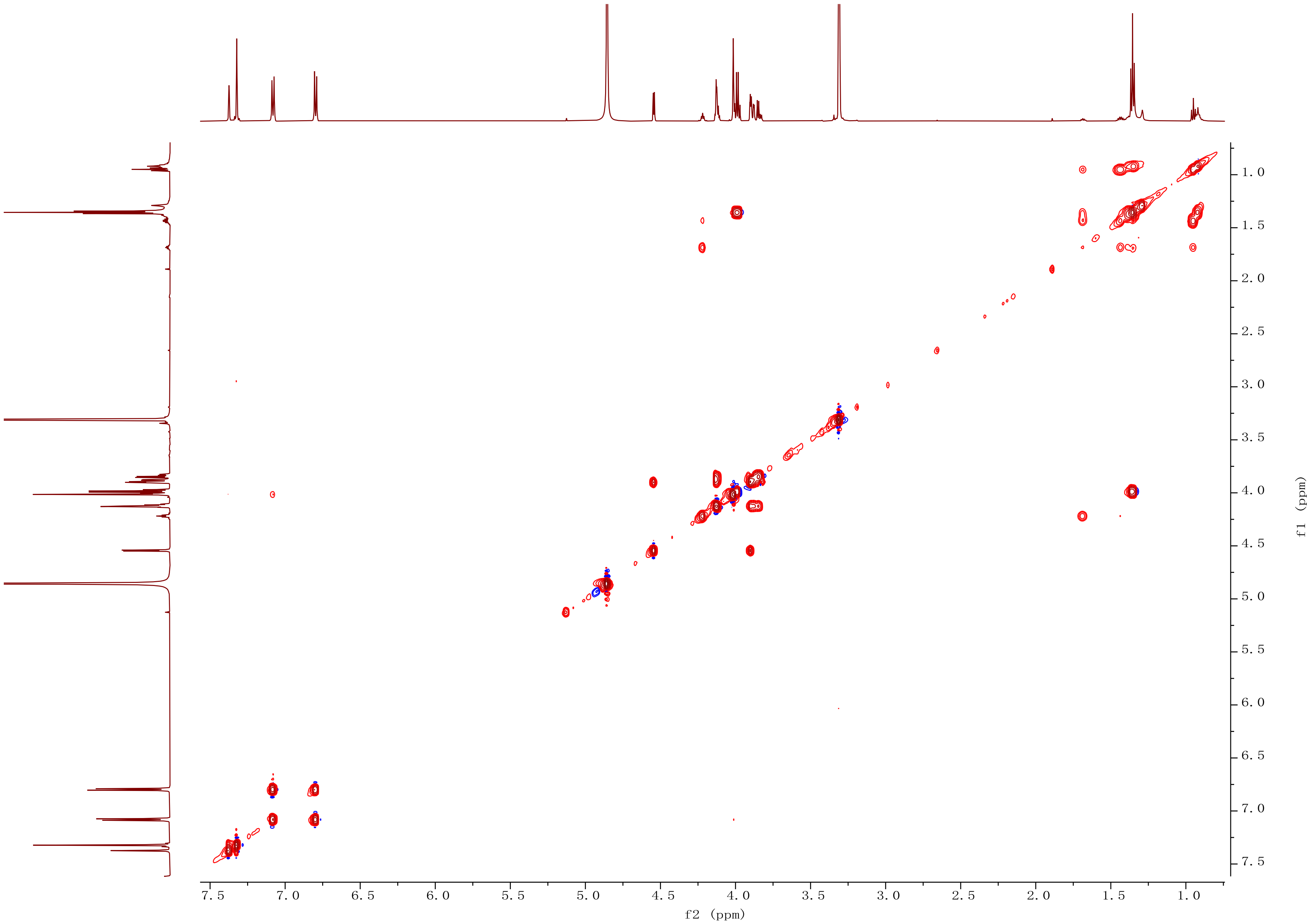

H-H COSY NMR of compound **β–66**

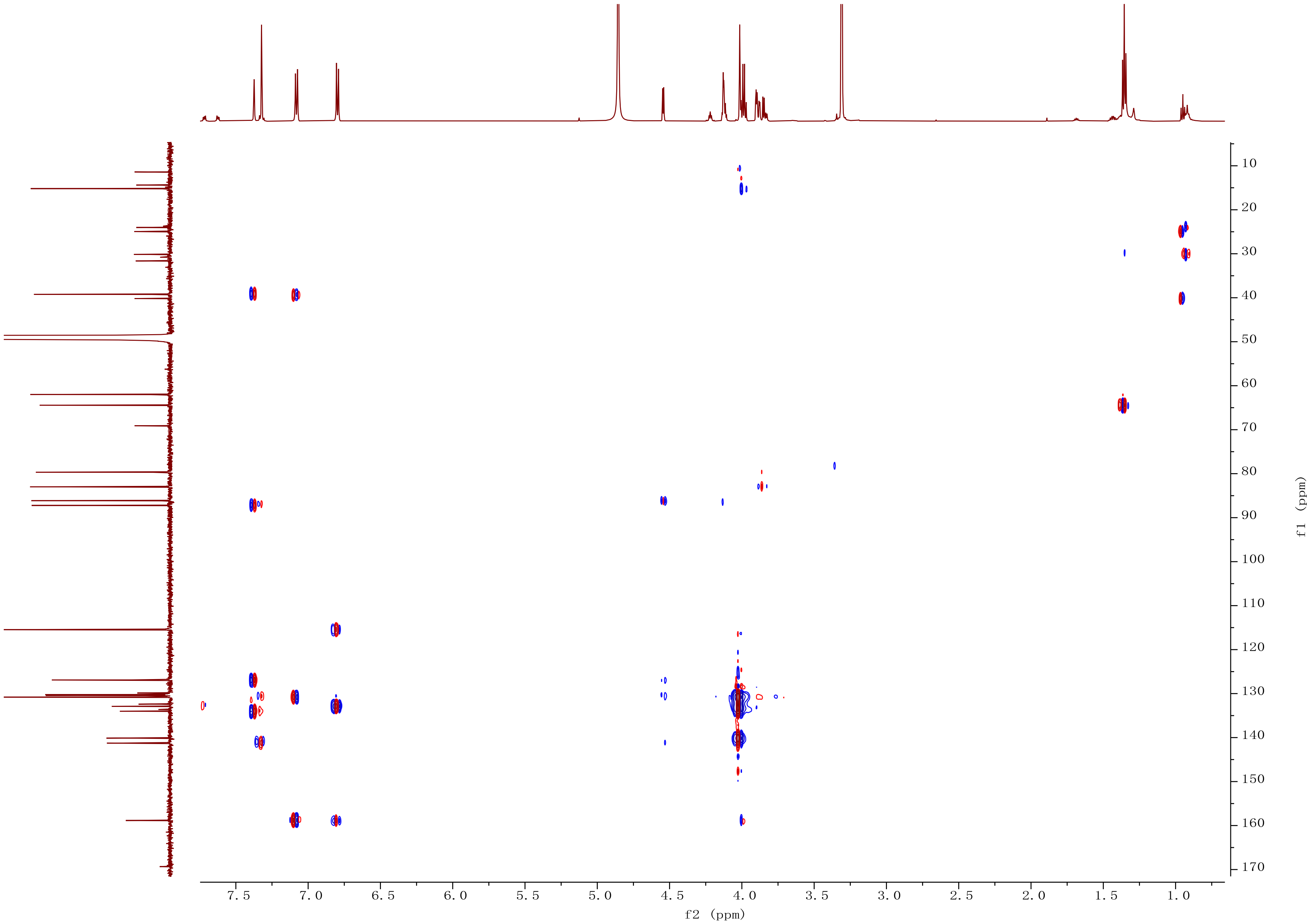

H-C HMBC NMR of compound **β–66**

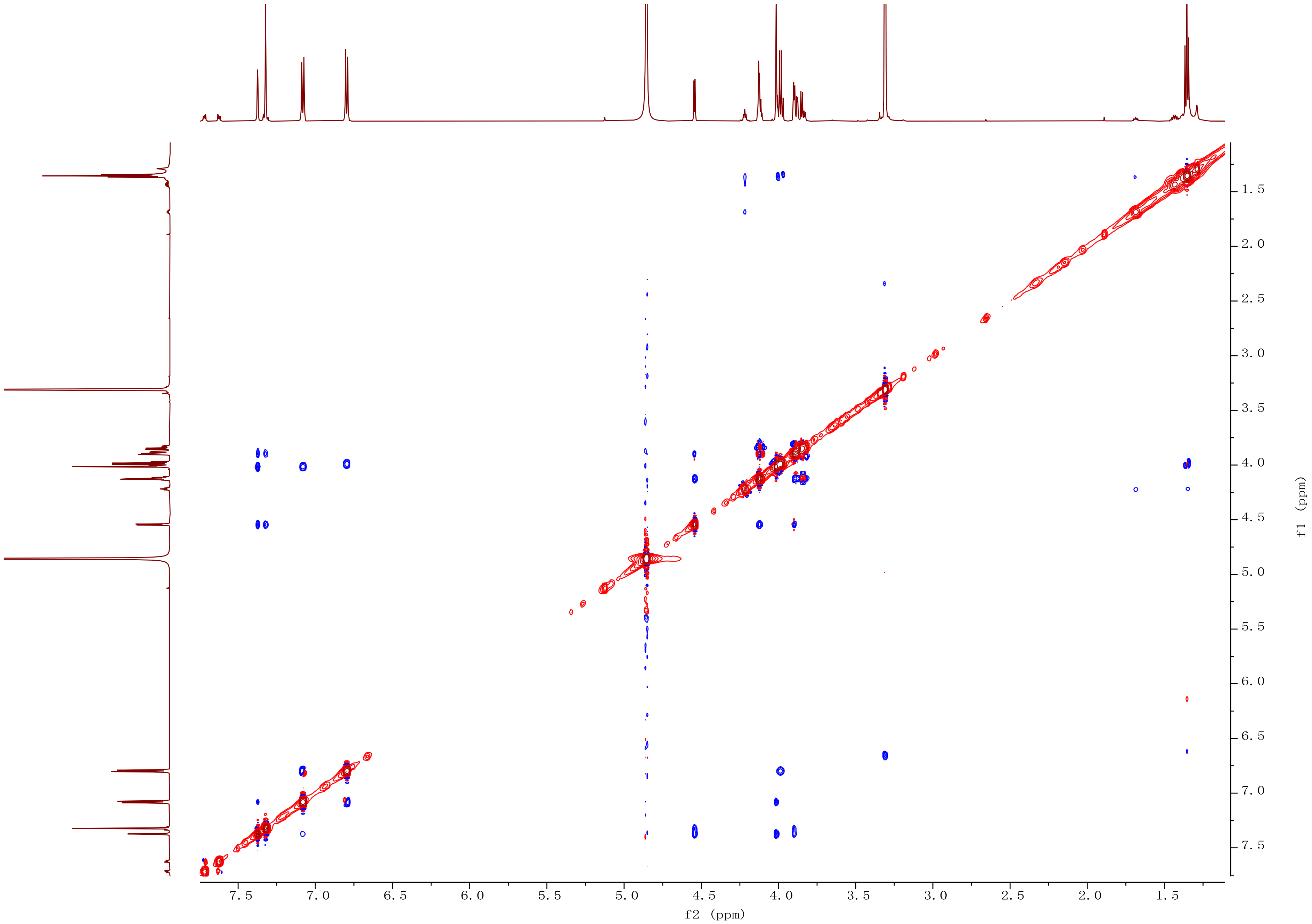

NOESY NMR of compound **β–66**

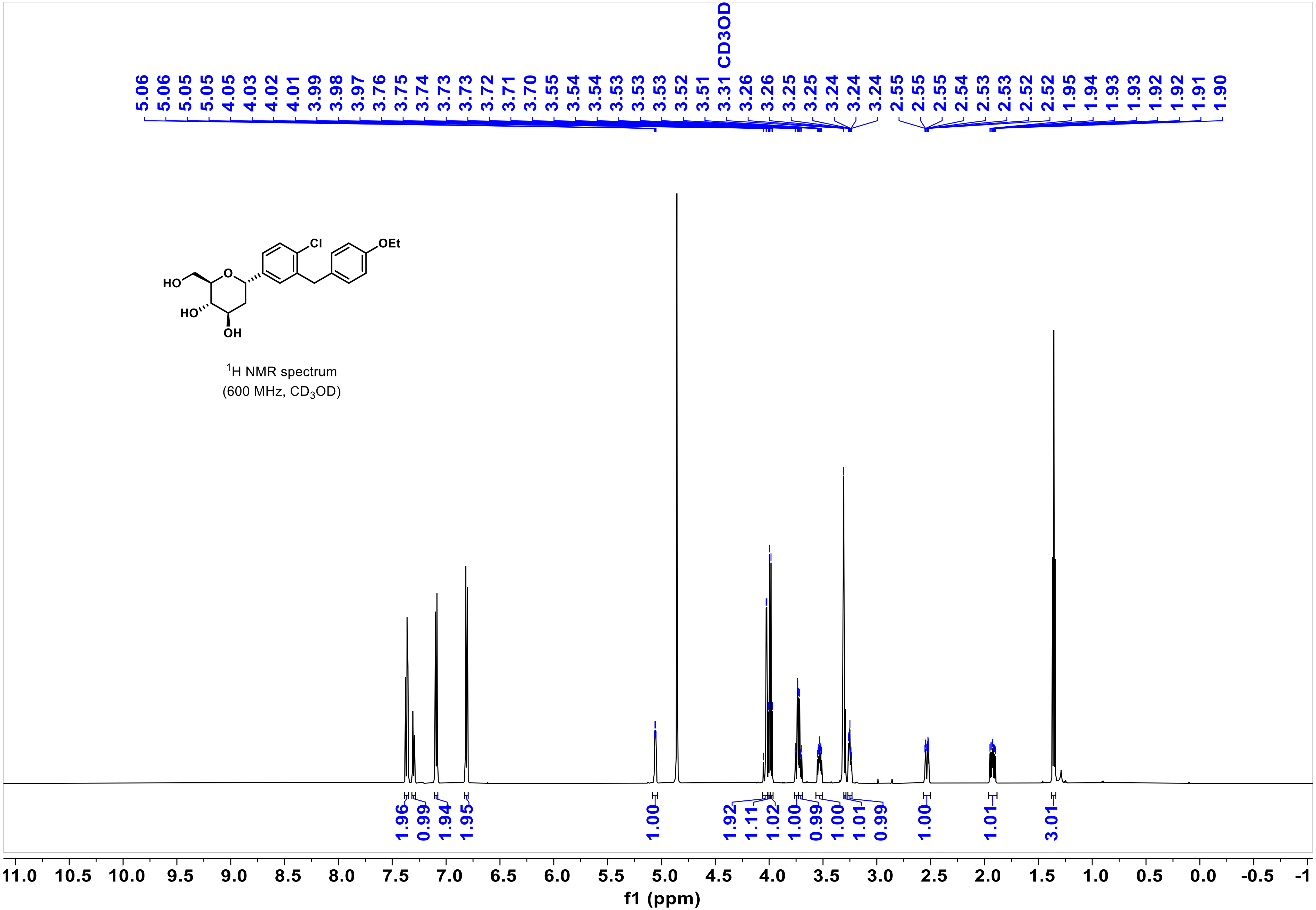

^{1}H NMR of compound **α–53**

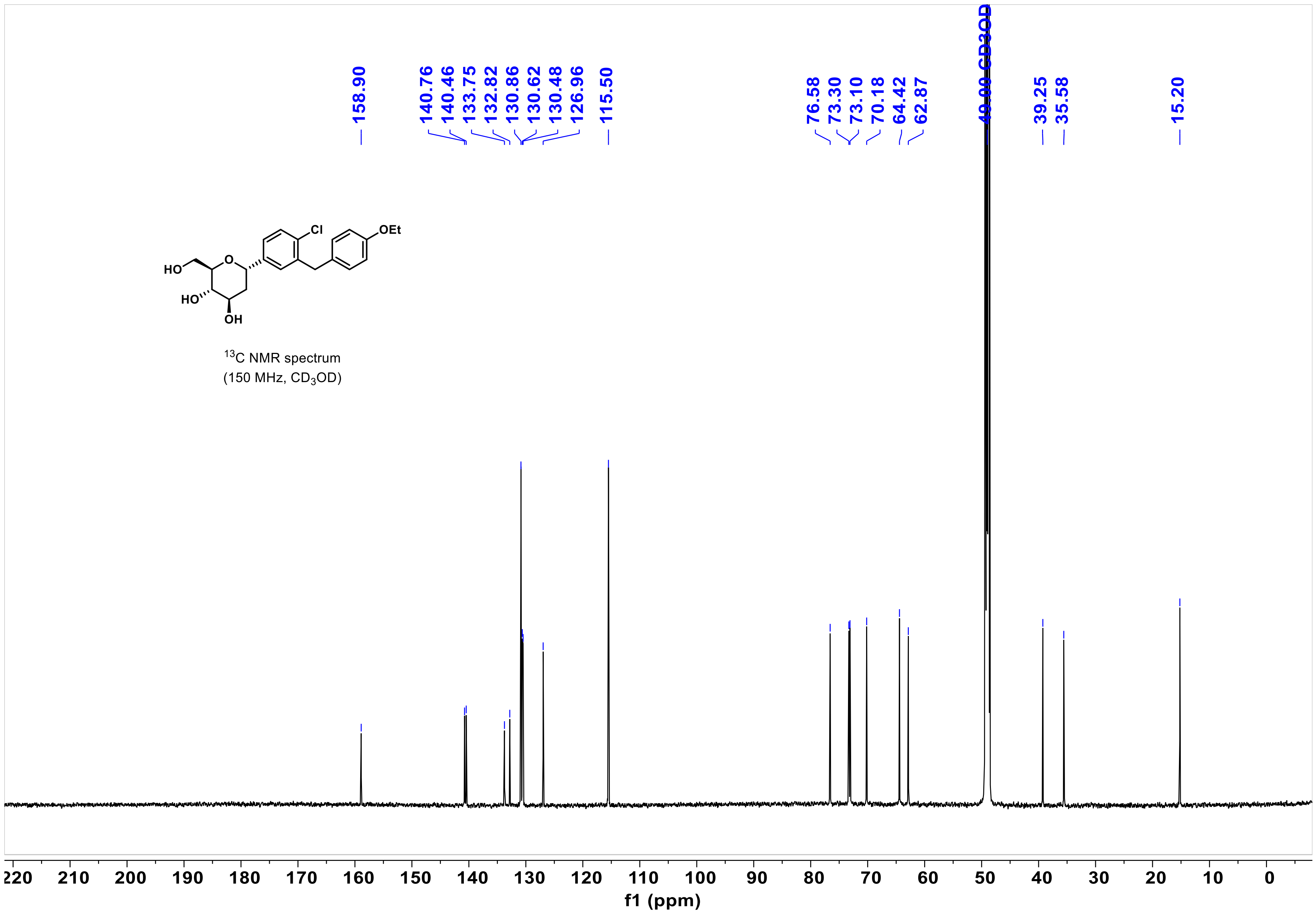

^{13}C NMR of compound **α–53**

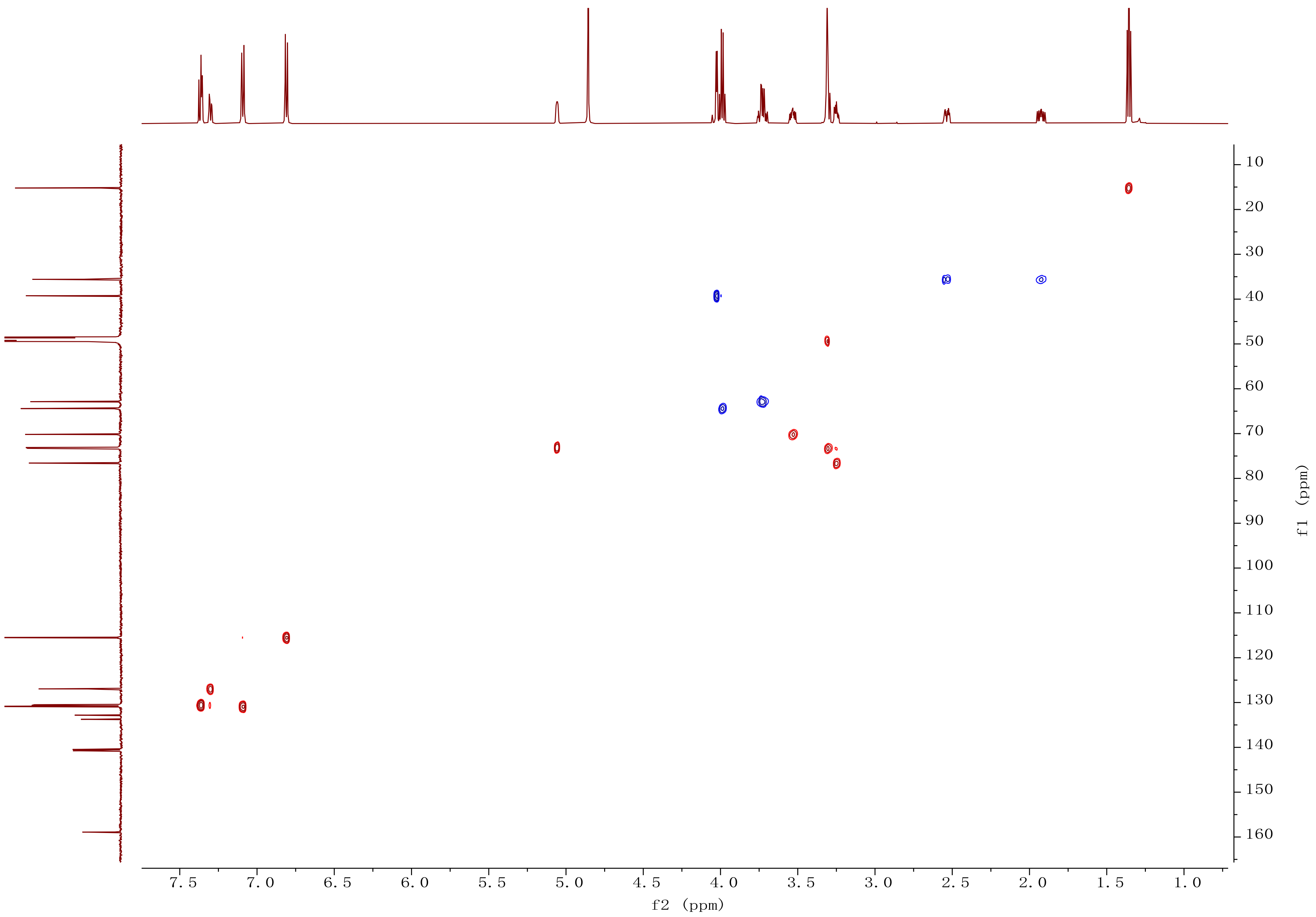

H-H HSQC NMR of compound **α–53**

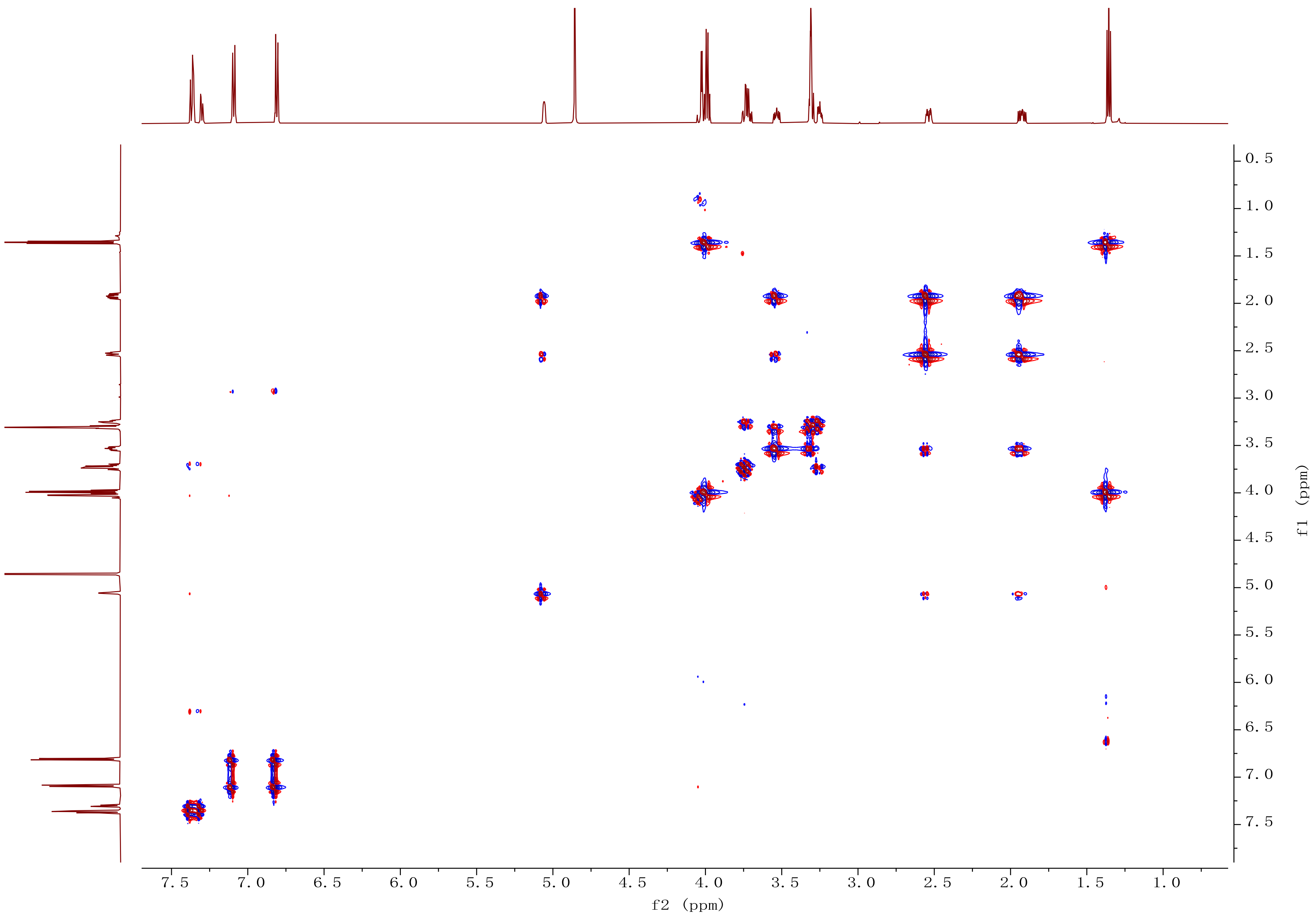

H-H COSY NMR of compound **α–53**

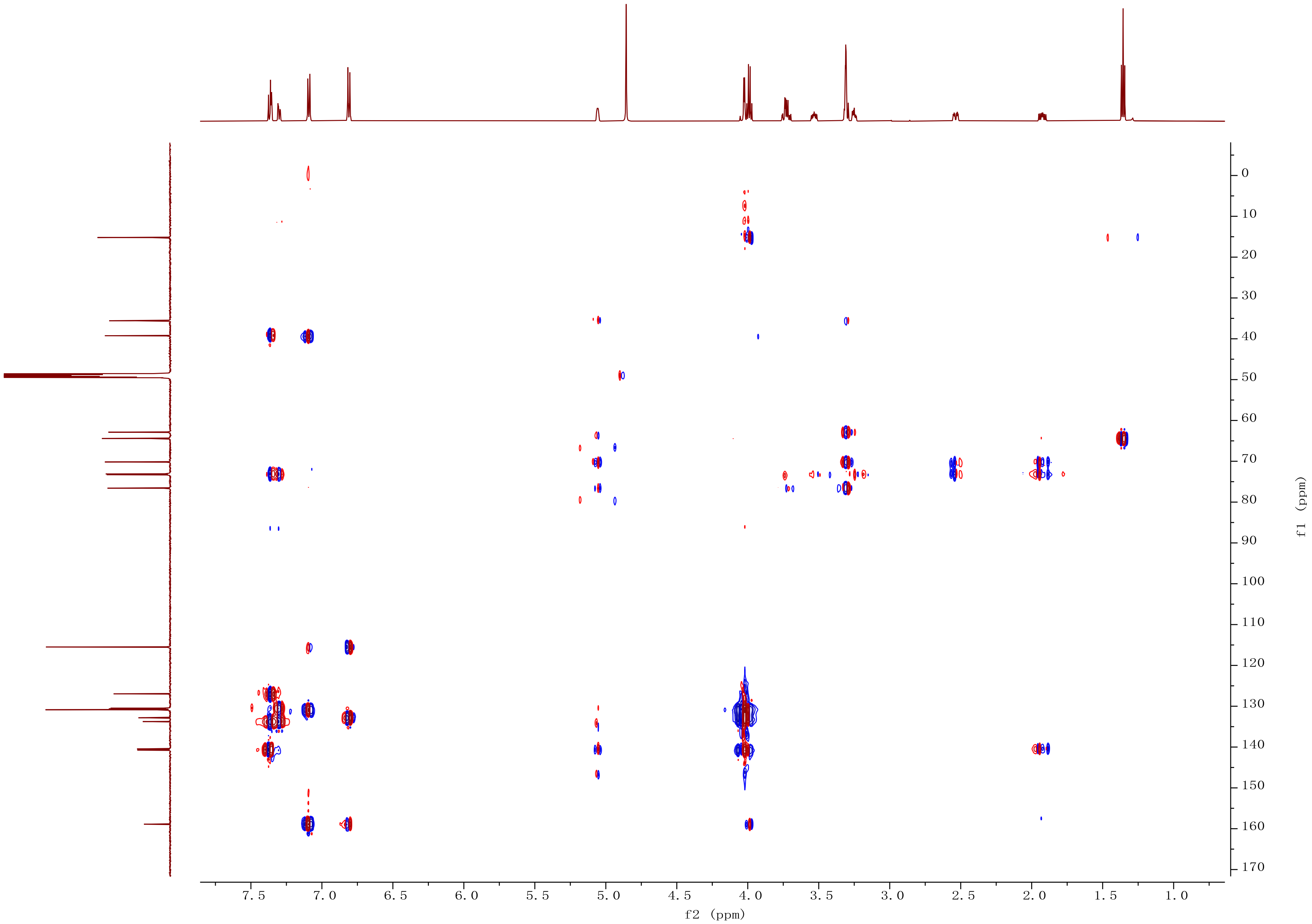

H-C HMBC NMR of compound **α–53**

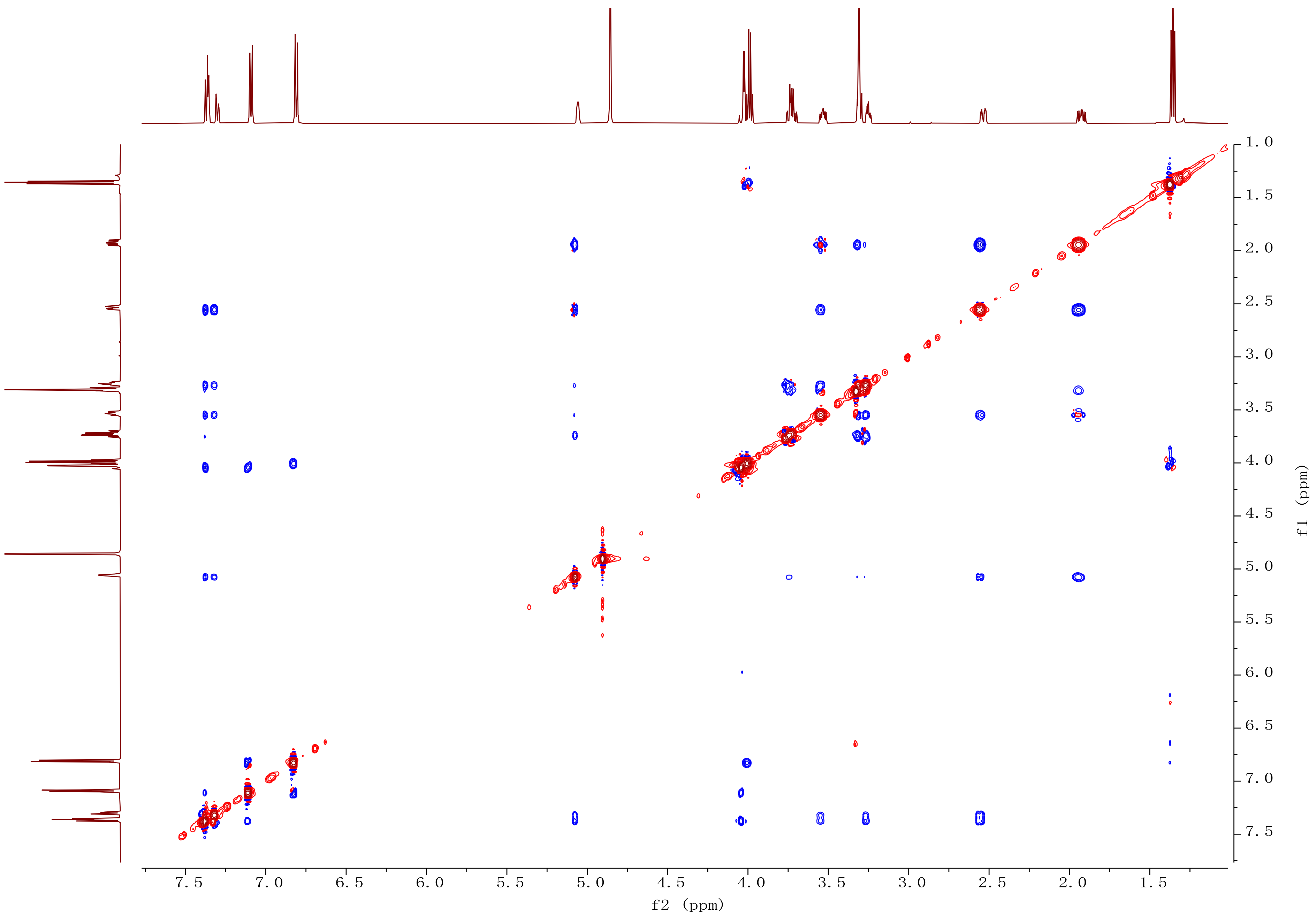

NOESY NMR of compound **α–53**

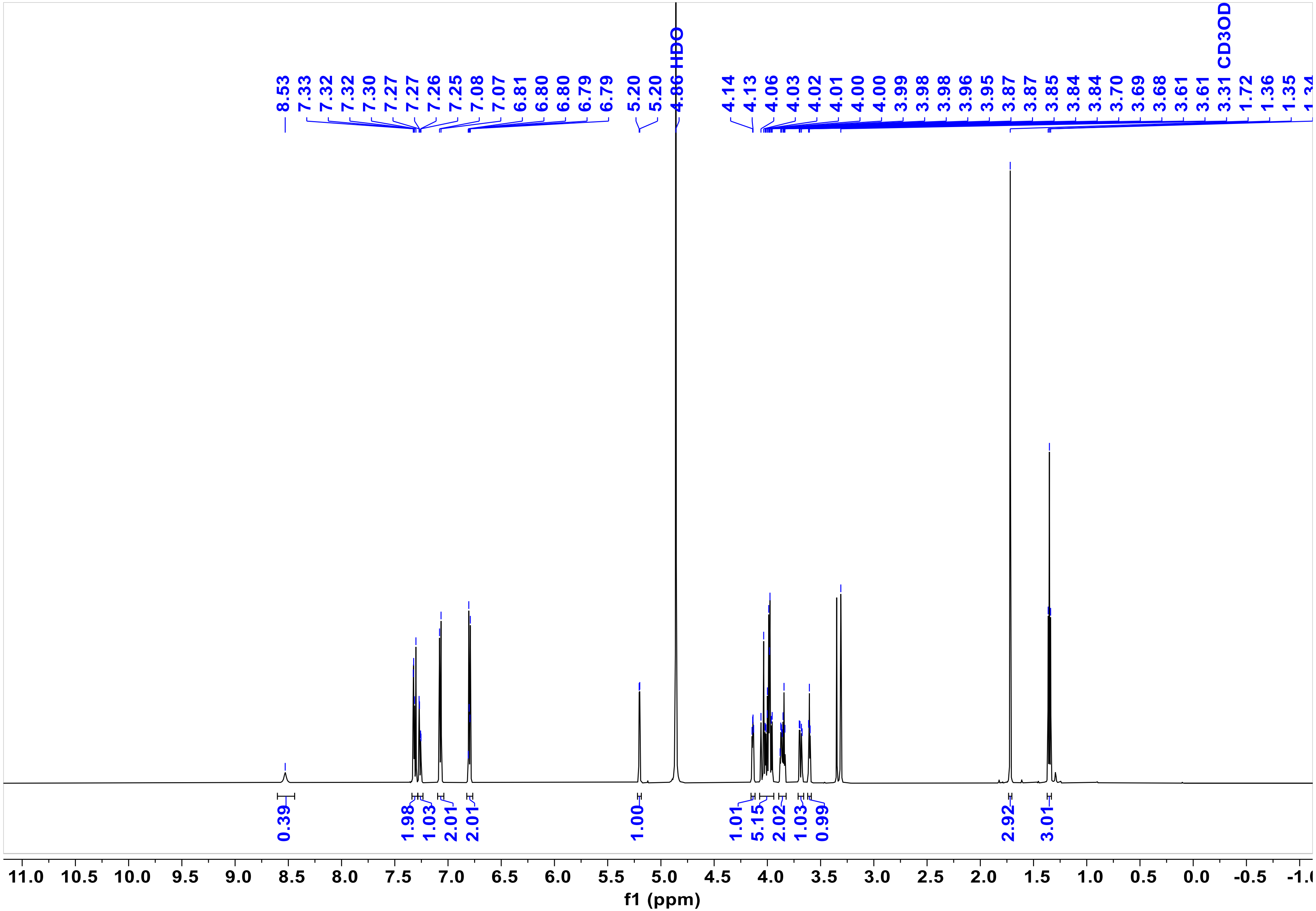

^{1}H NMR of compound **α–49**

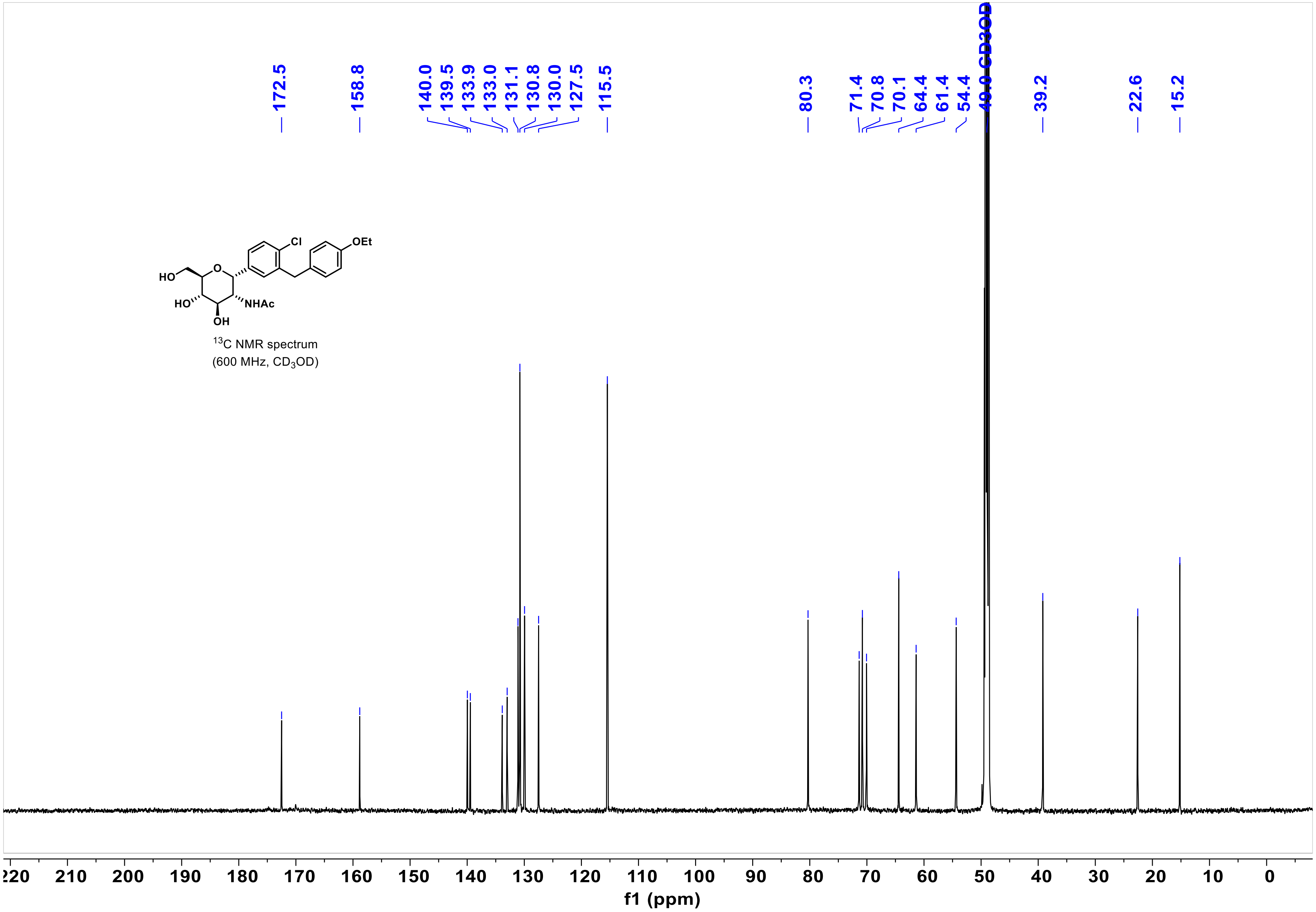

^{13}C NMR of compound **α–49**

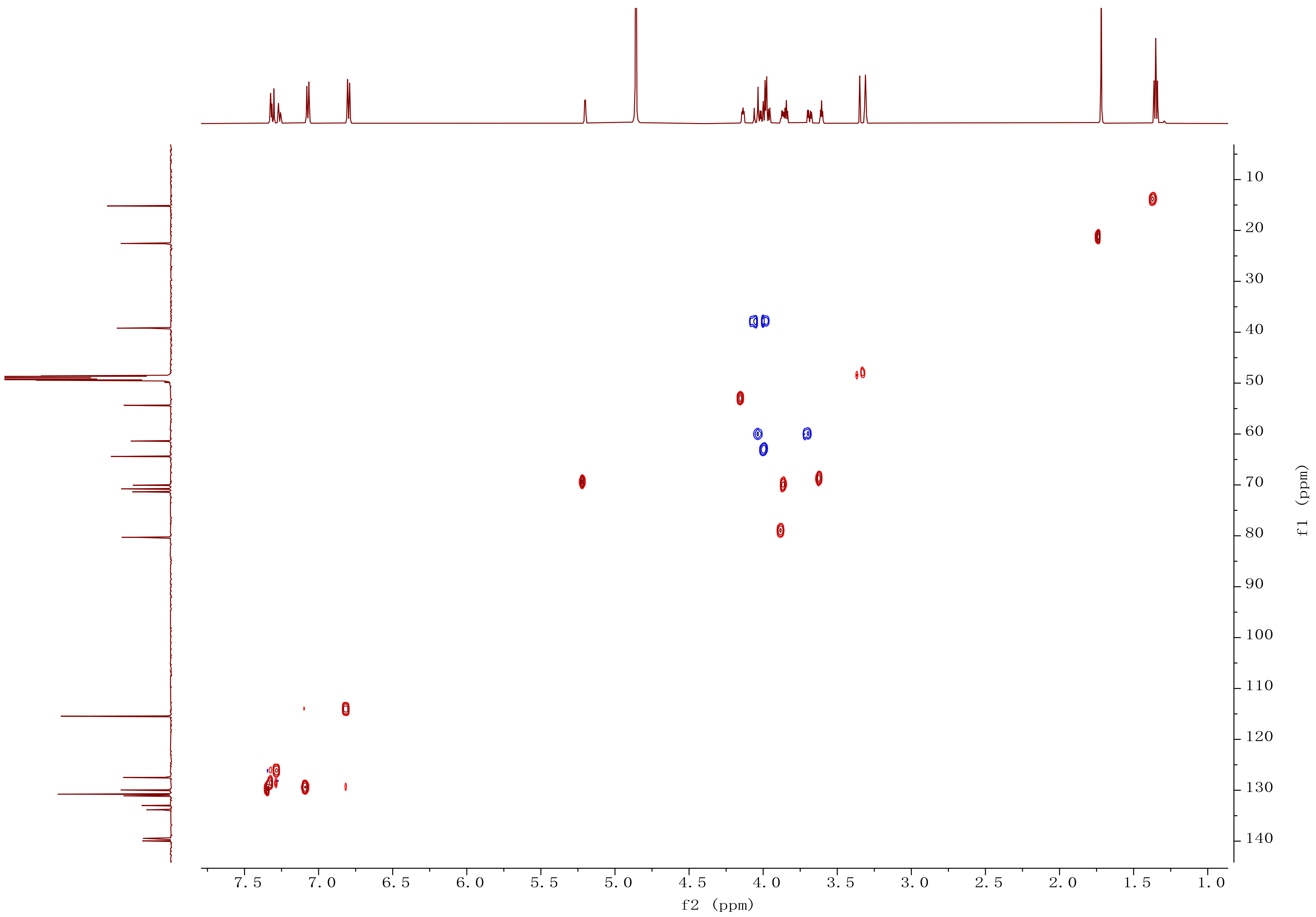

H-H HSQC NMR of compound **α–49**

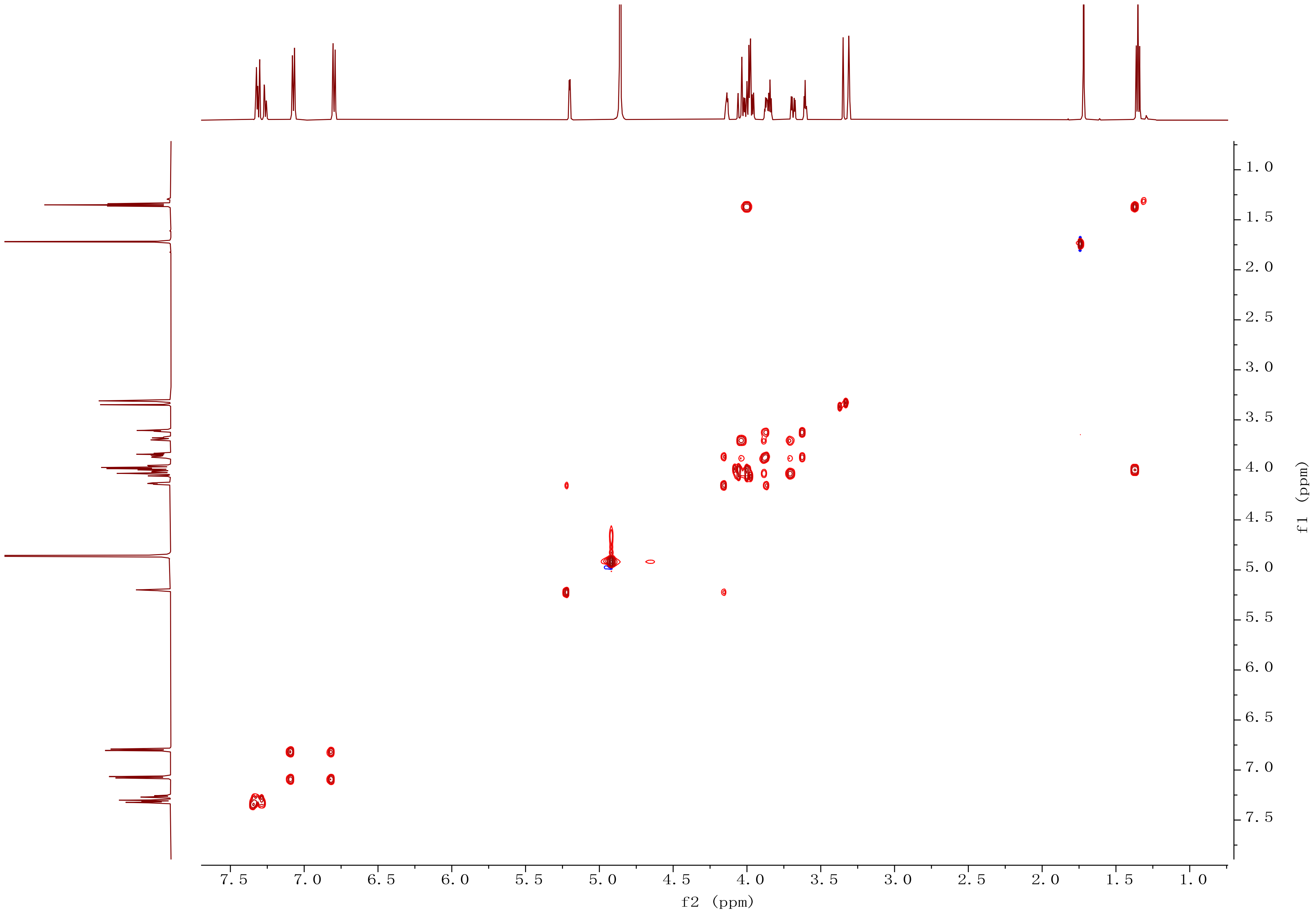

H-H COSY NMR of compound **α–49**

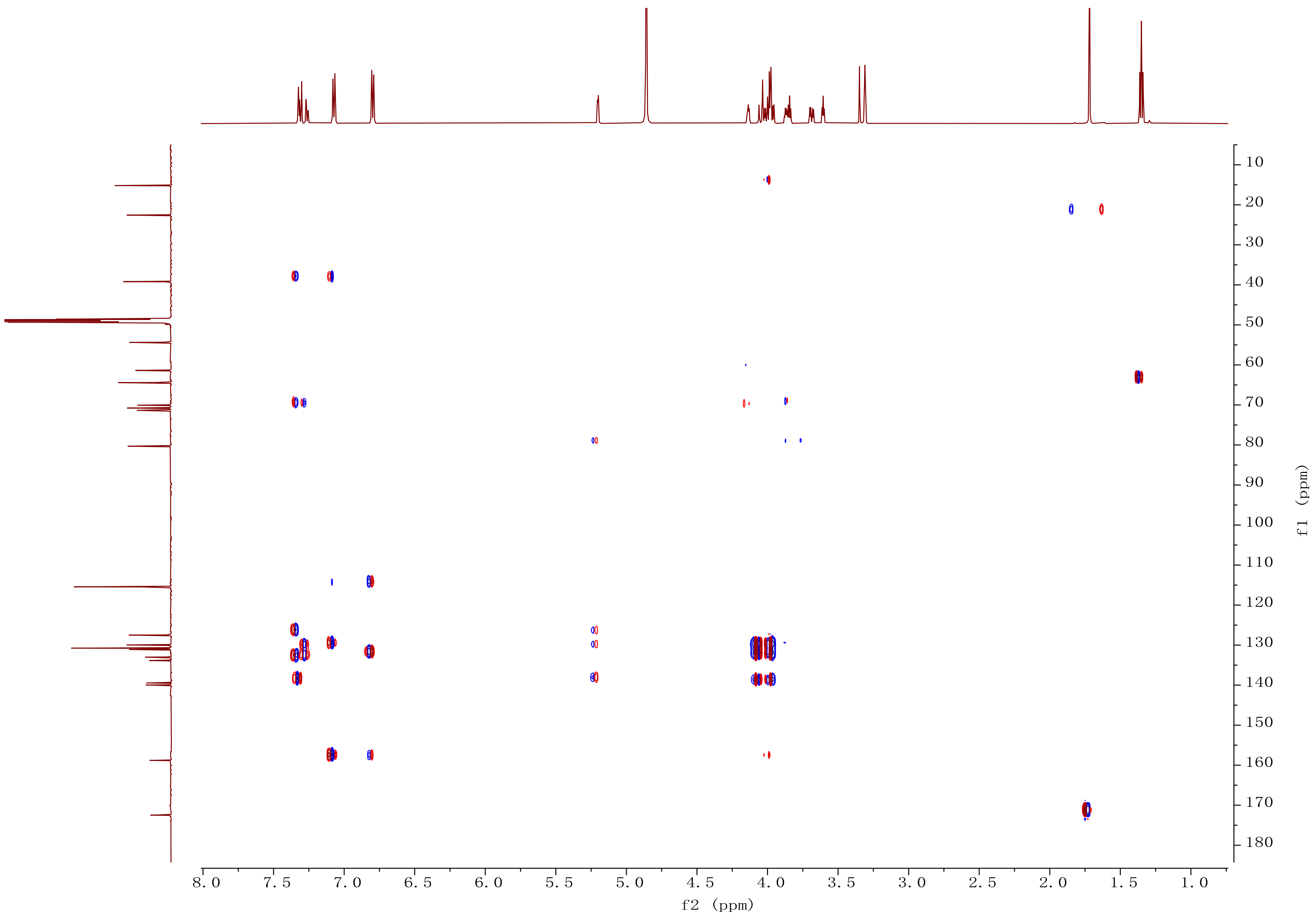

H-C HMBC NMR of compound **α–49**

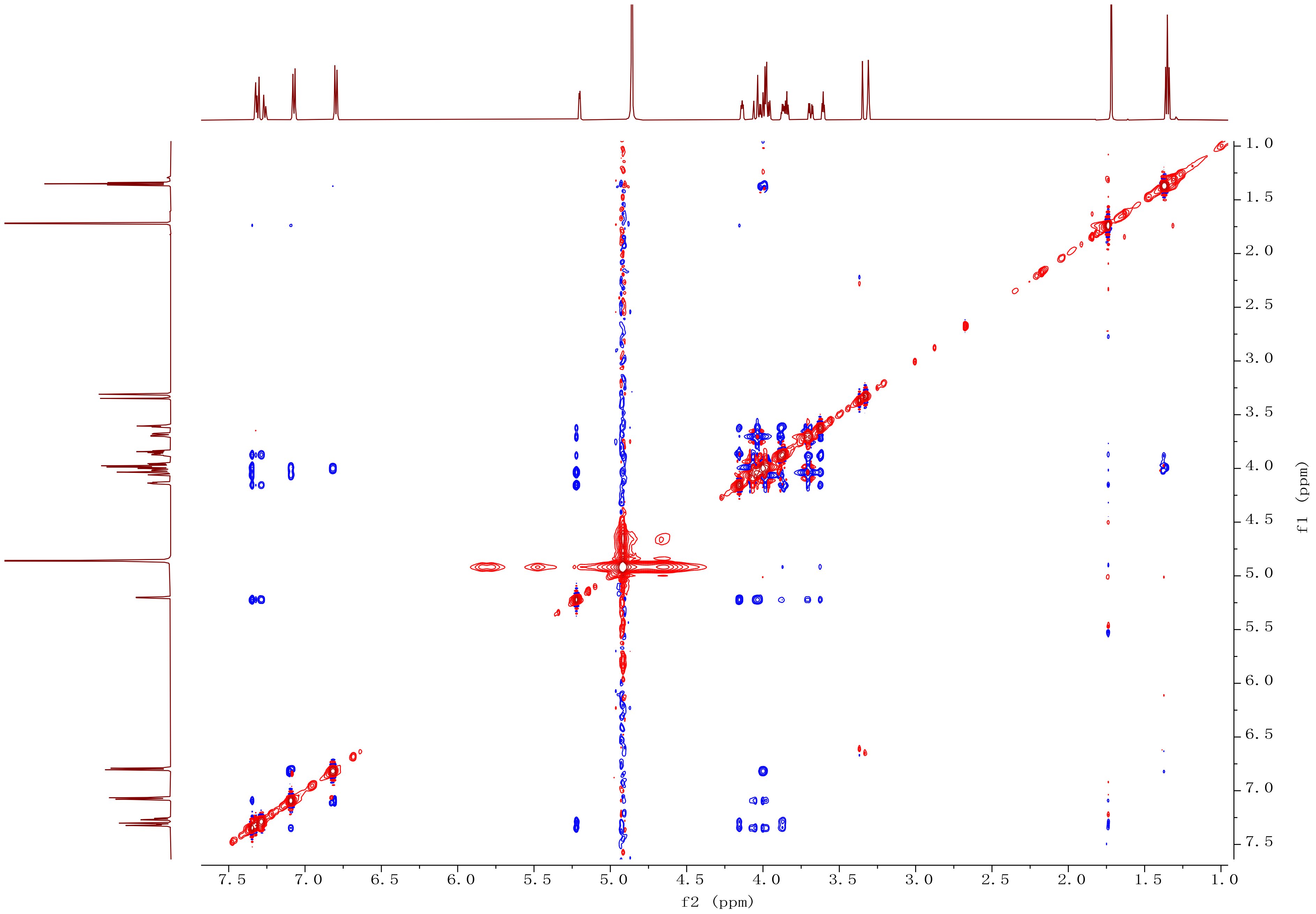

NOESY NMR of compound **α–49**

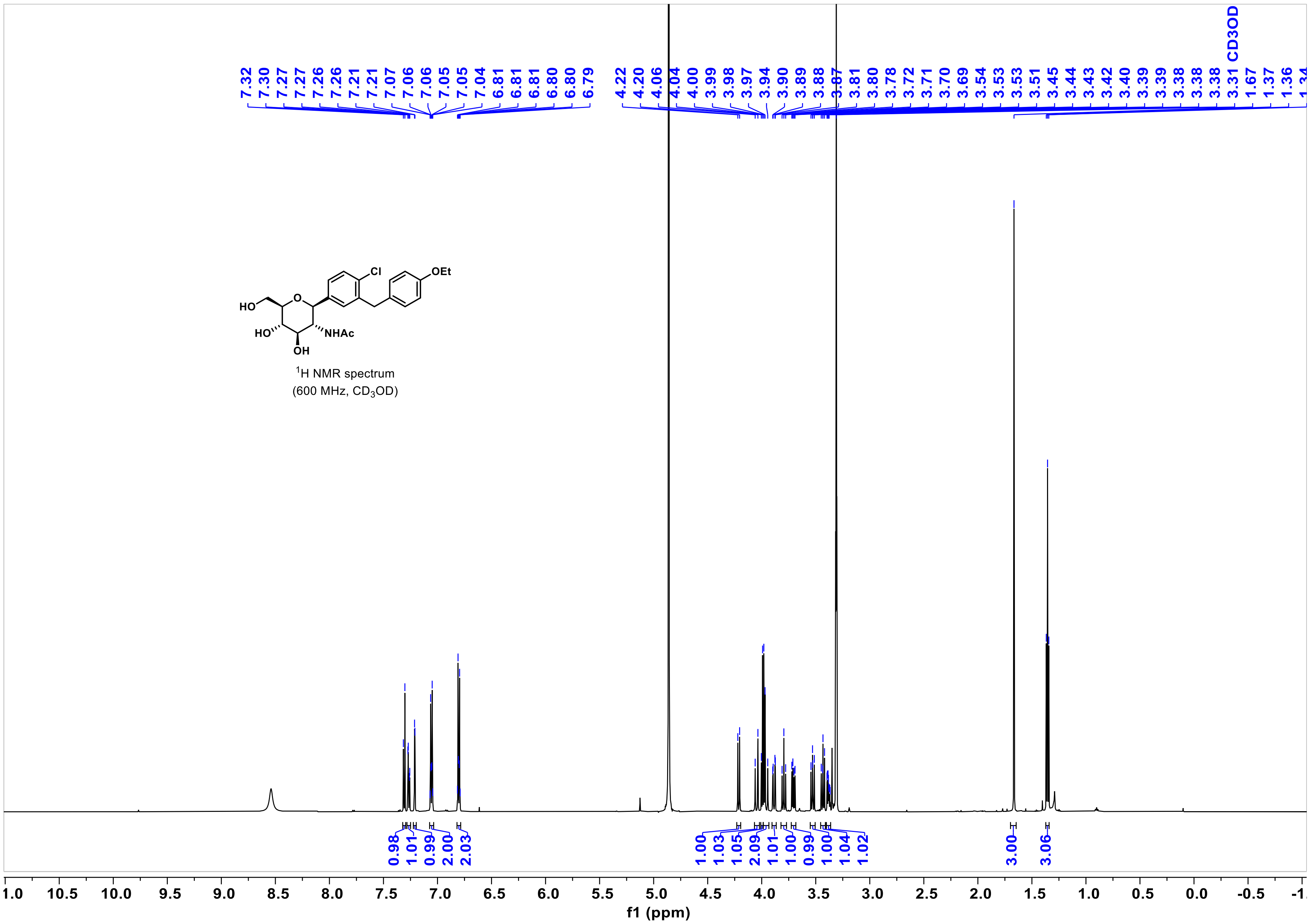

^{1}H NMR of compound **β–49**

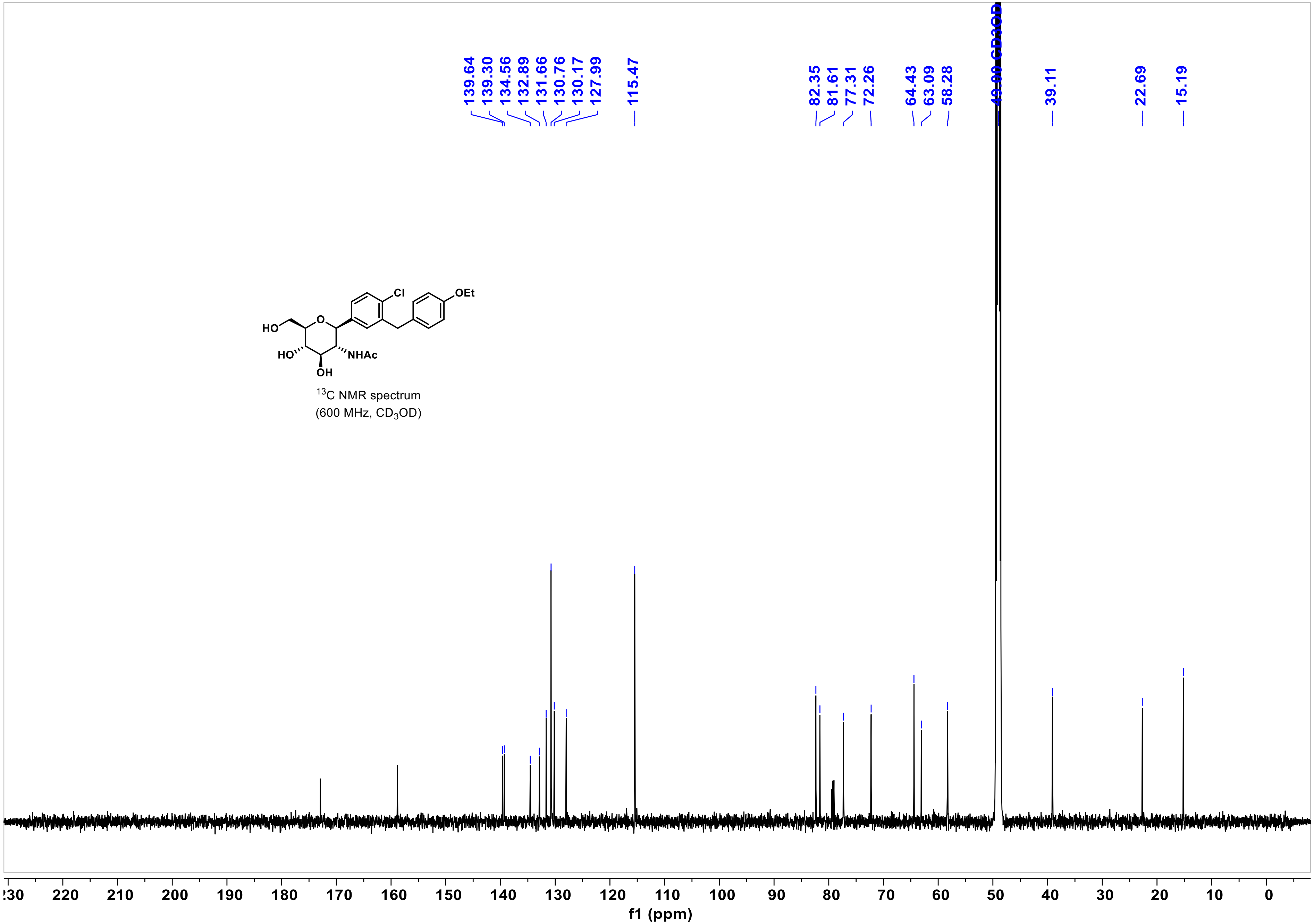

^{13}C NMR of compound **β–49**

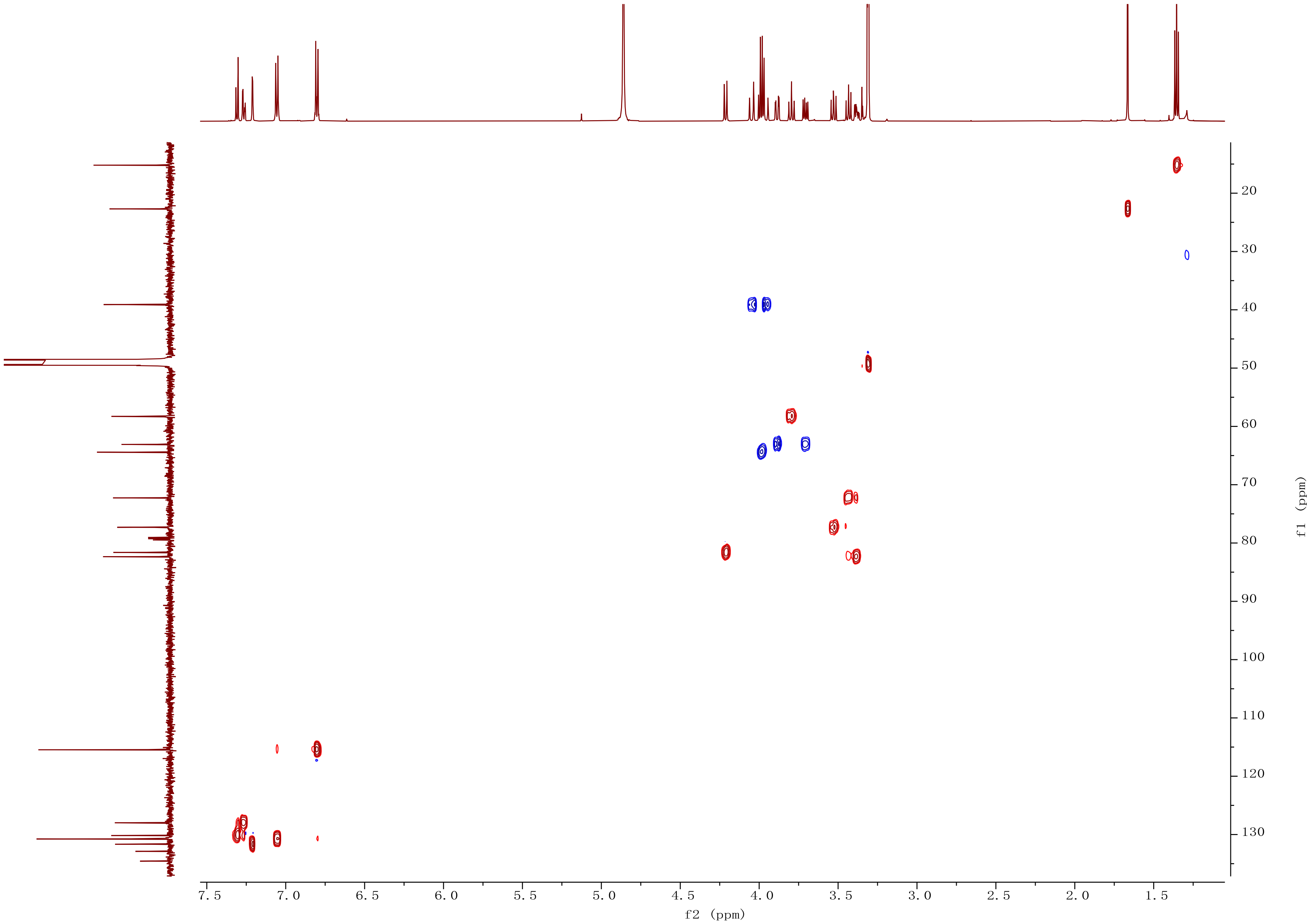

H-H HSQC NMR of compound **β–49**

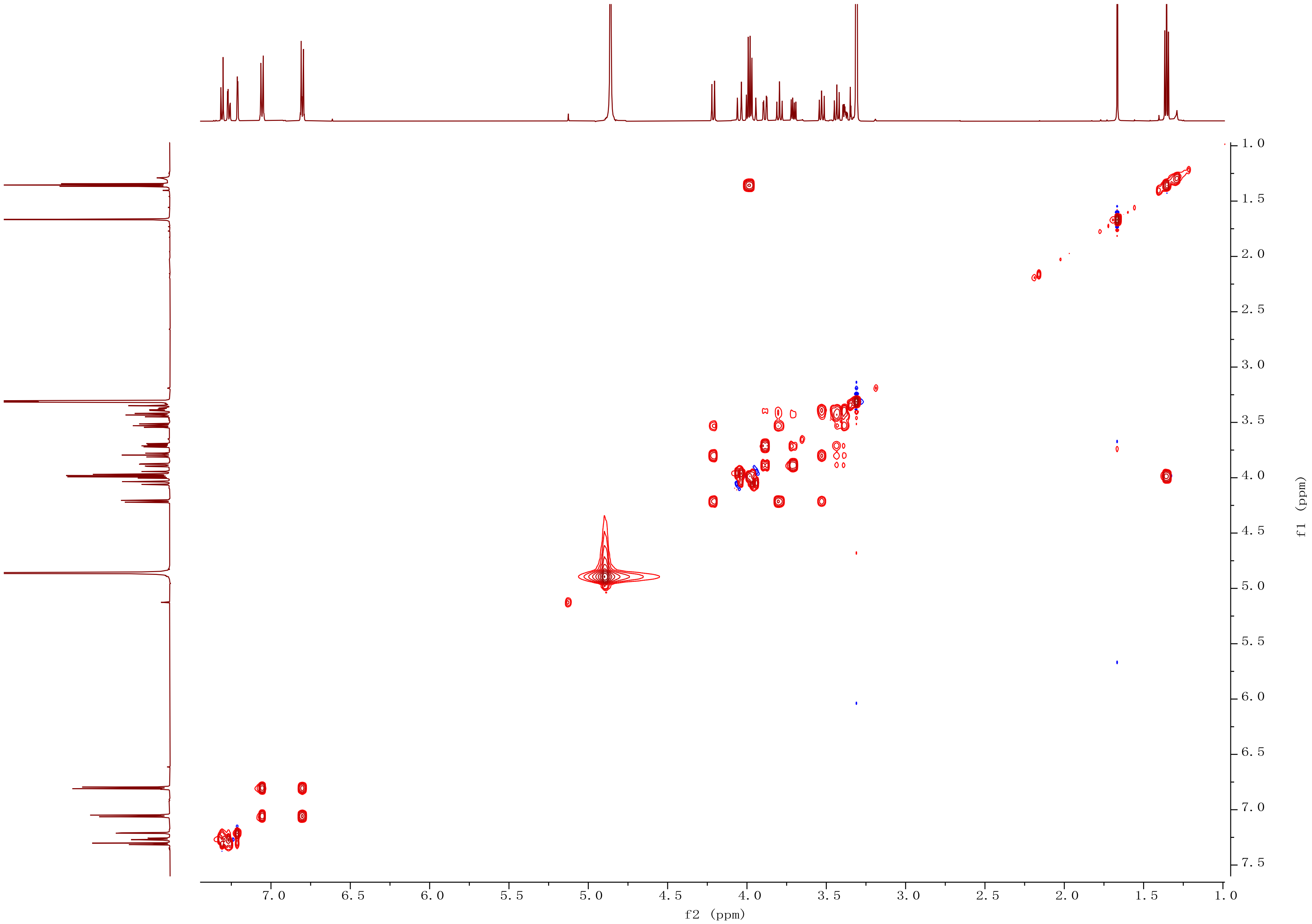

H-H COSY NMR of compound **β–49**

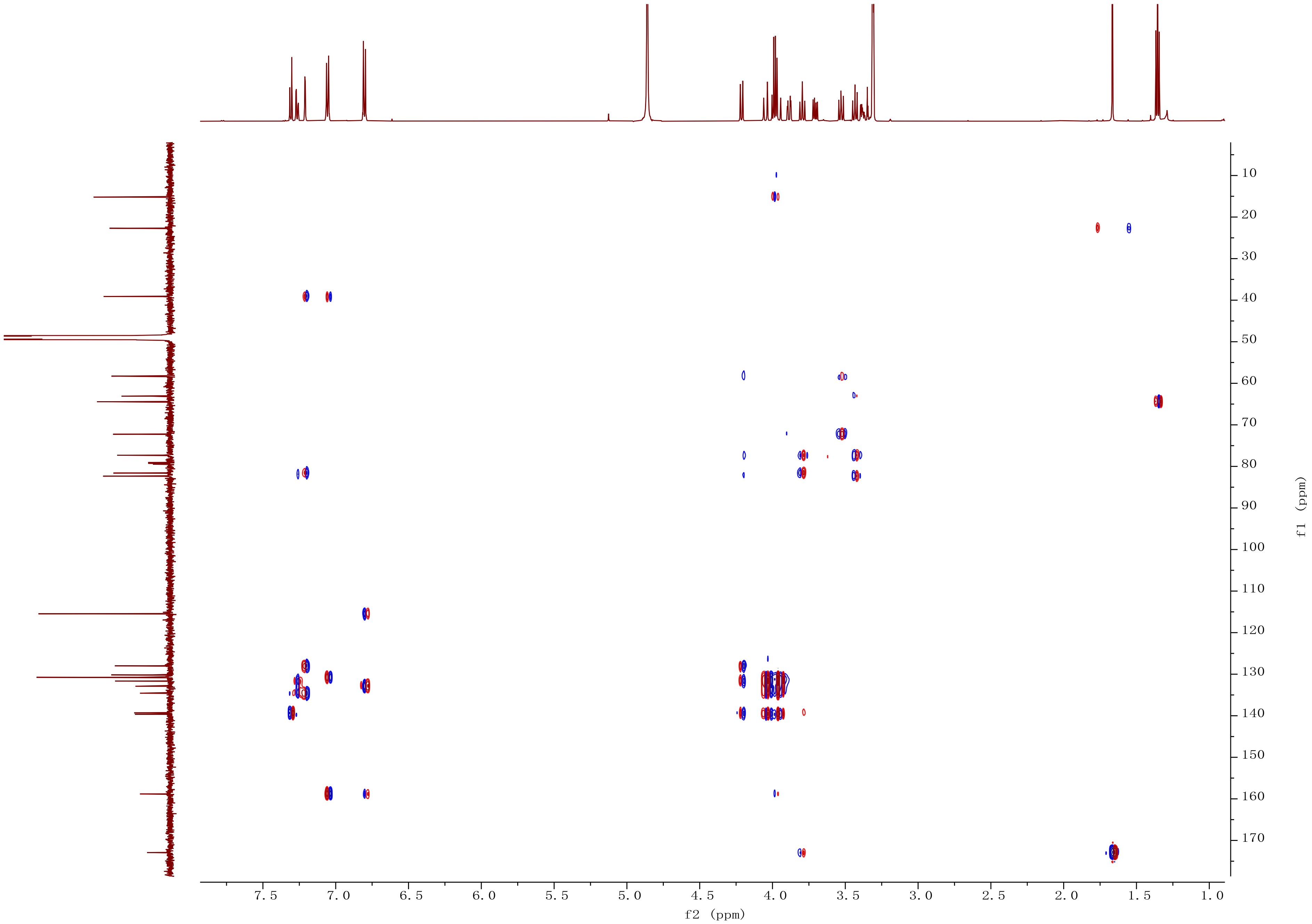

H-C HMBC NMR of compound **β–49**

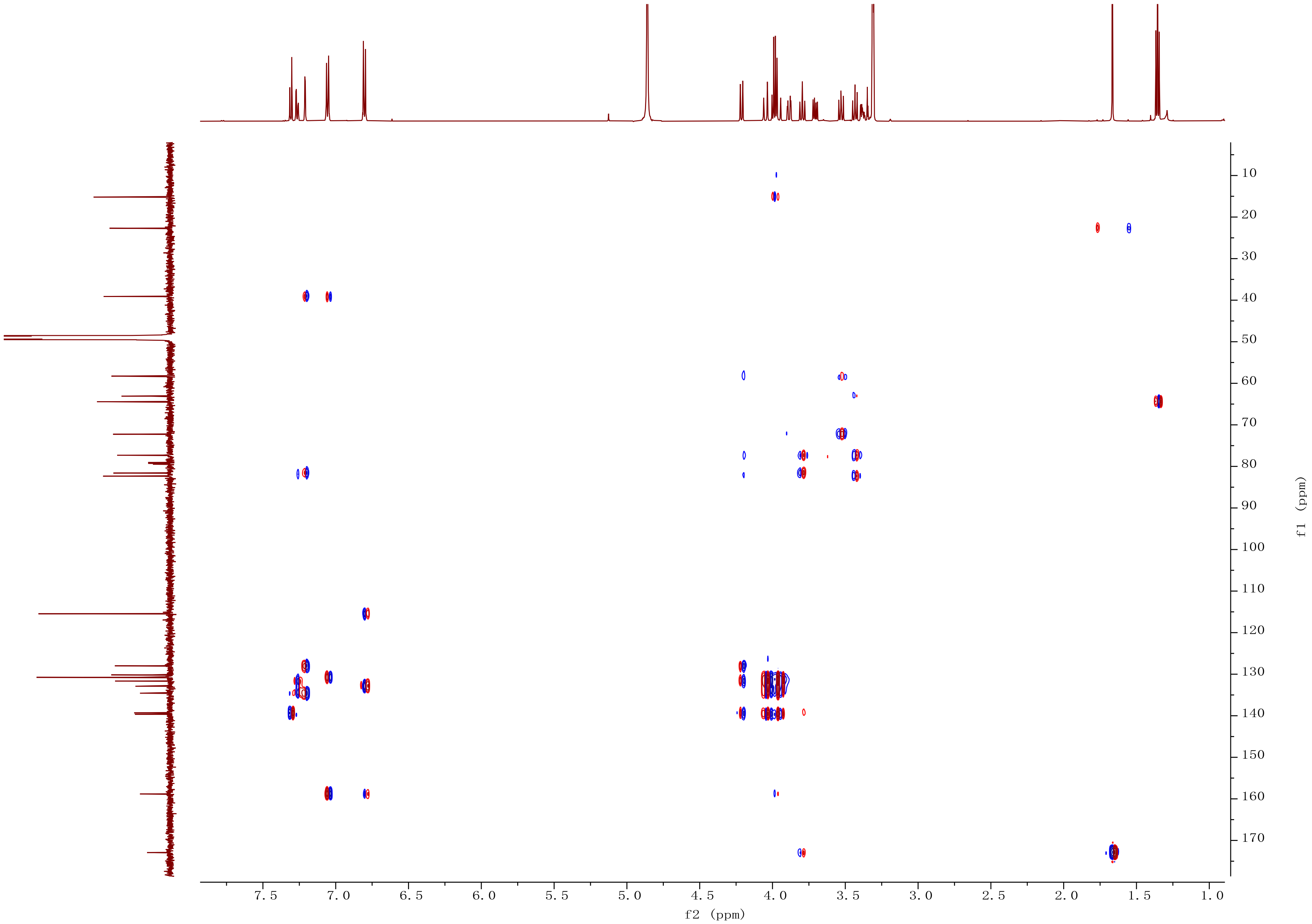

NOESY NMR of compound **β–49**

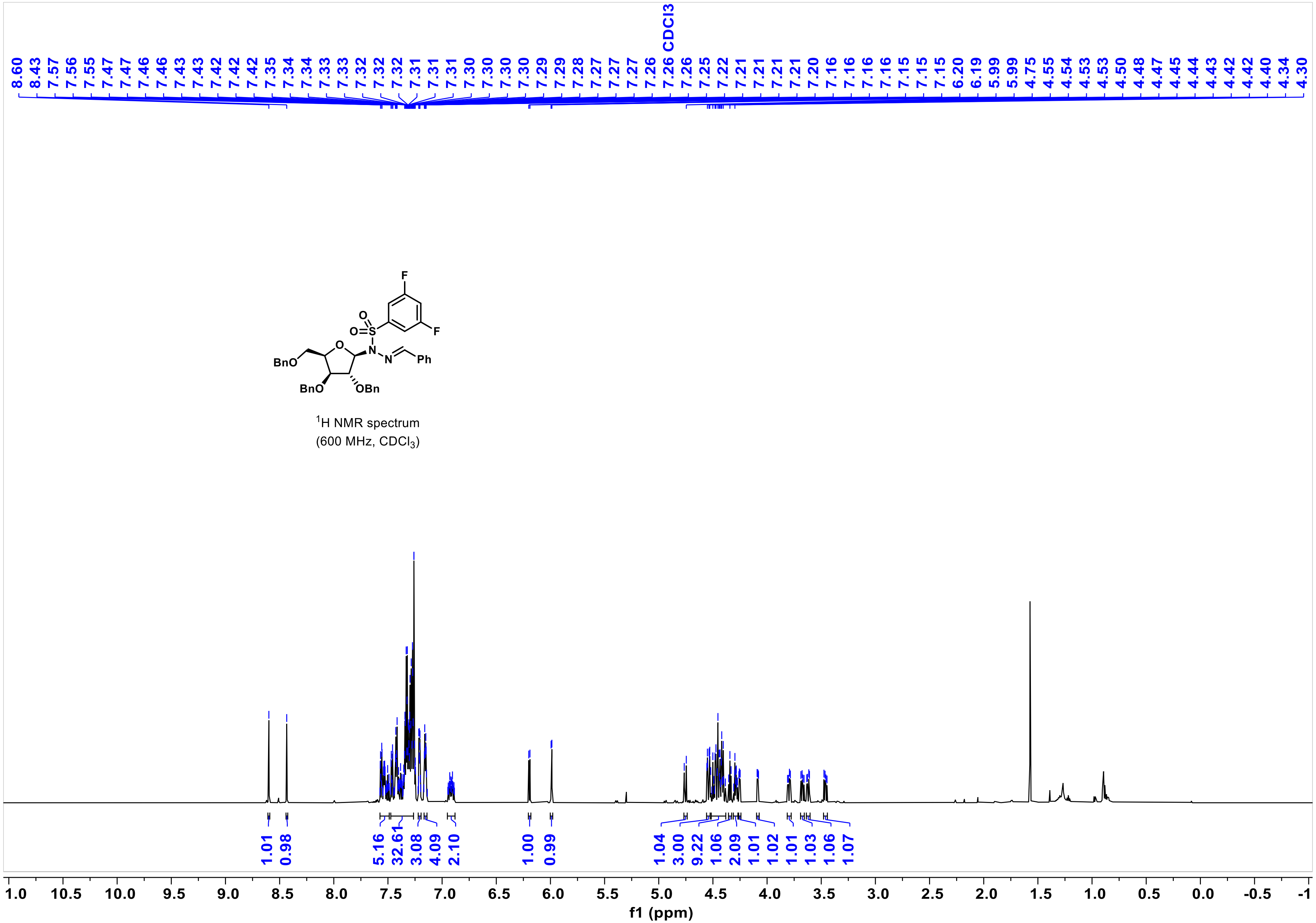

^{1}H NMR of compound **SJ4**

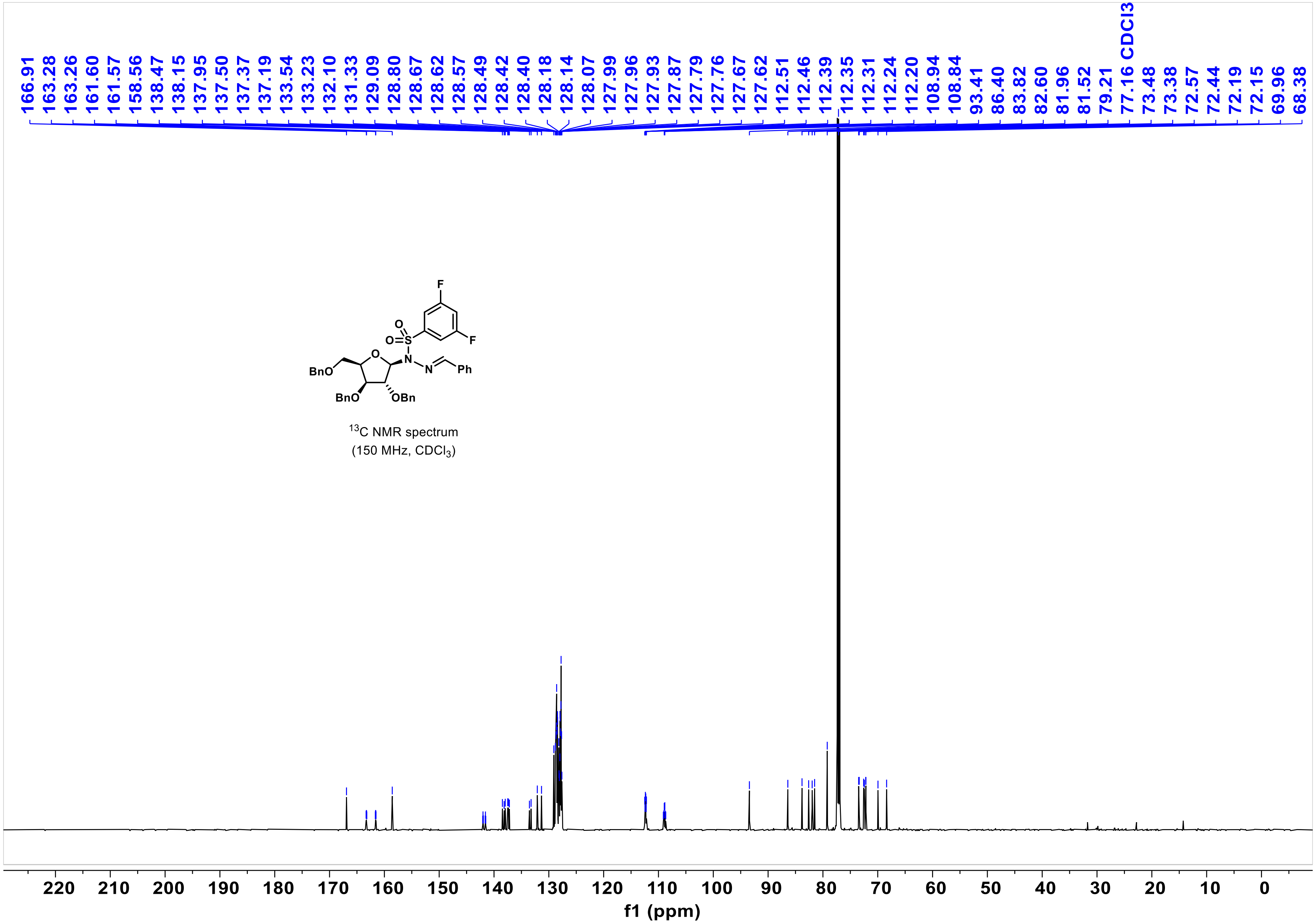

^{13}C NMR of compound **SJ4**

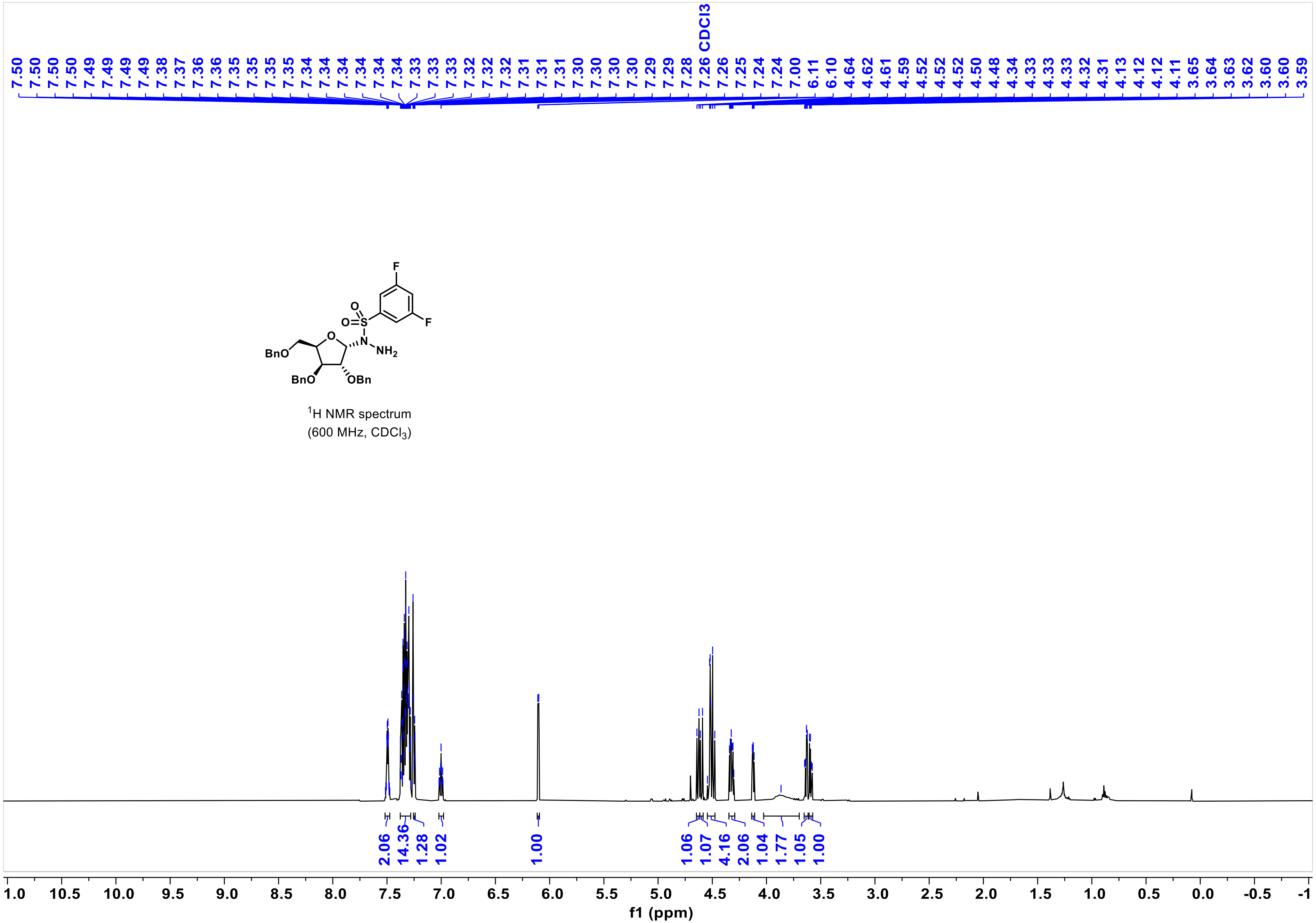

^{1}H NMR of compound **α-SJ5**

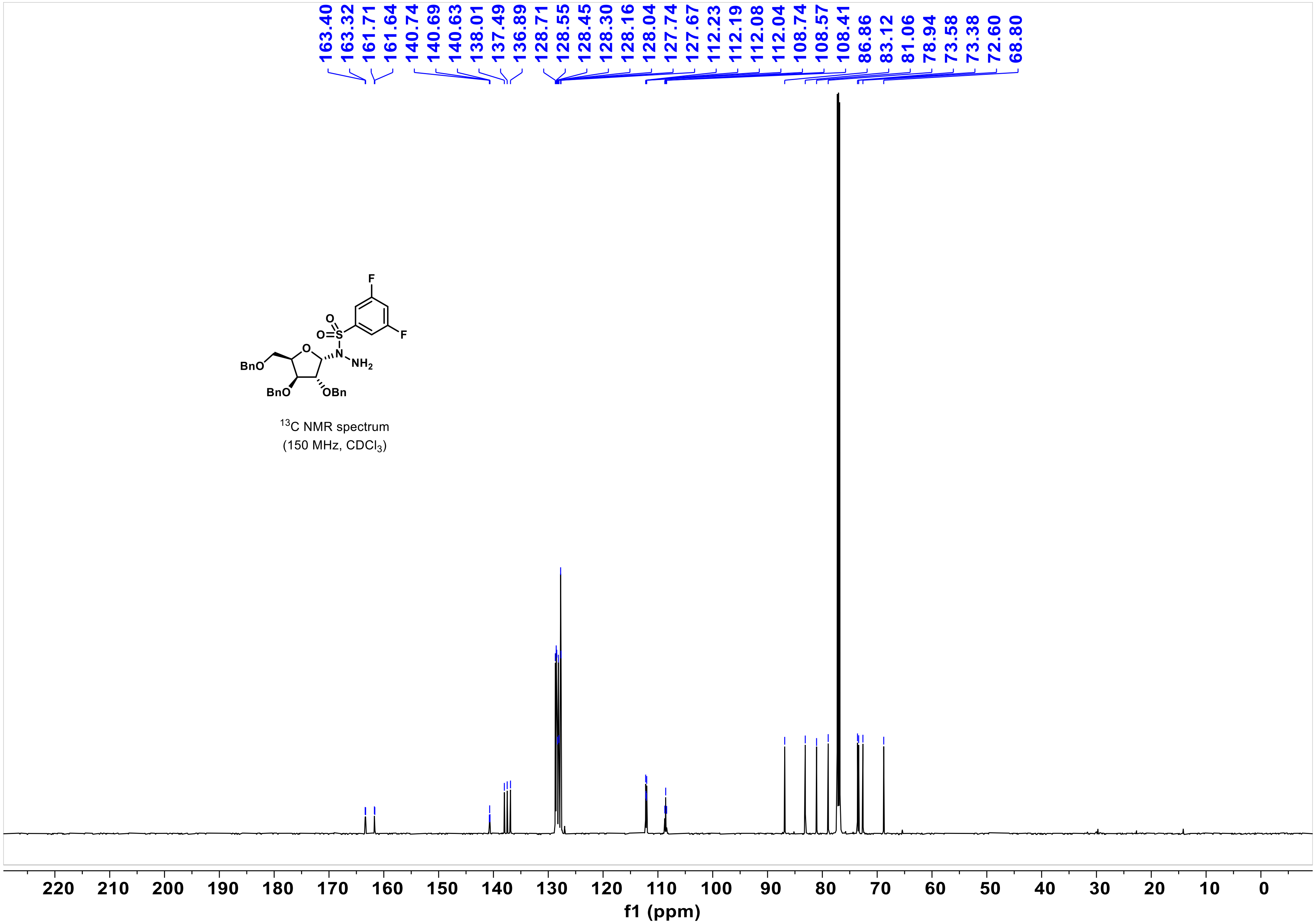

^{13}C NMR of compound **α-SJ5**

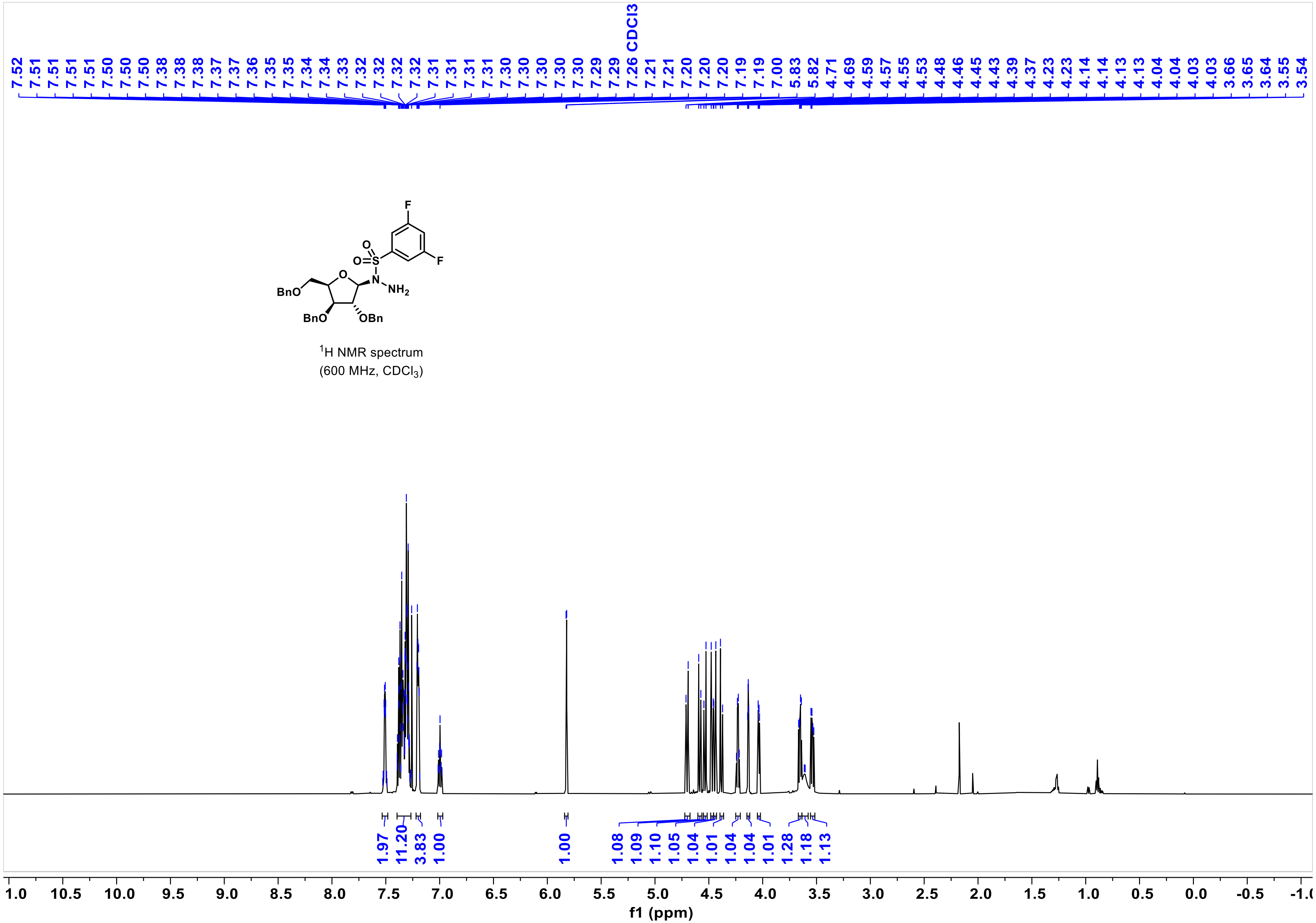

^{1}H NMR of compound **β-SJ5**

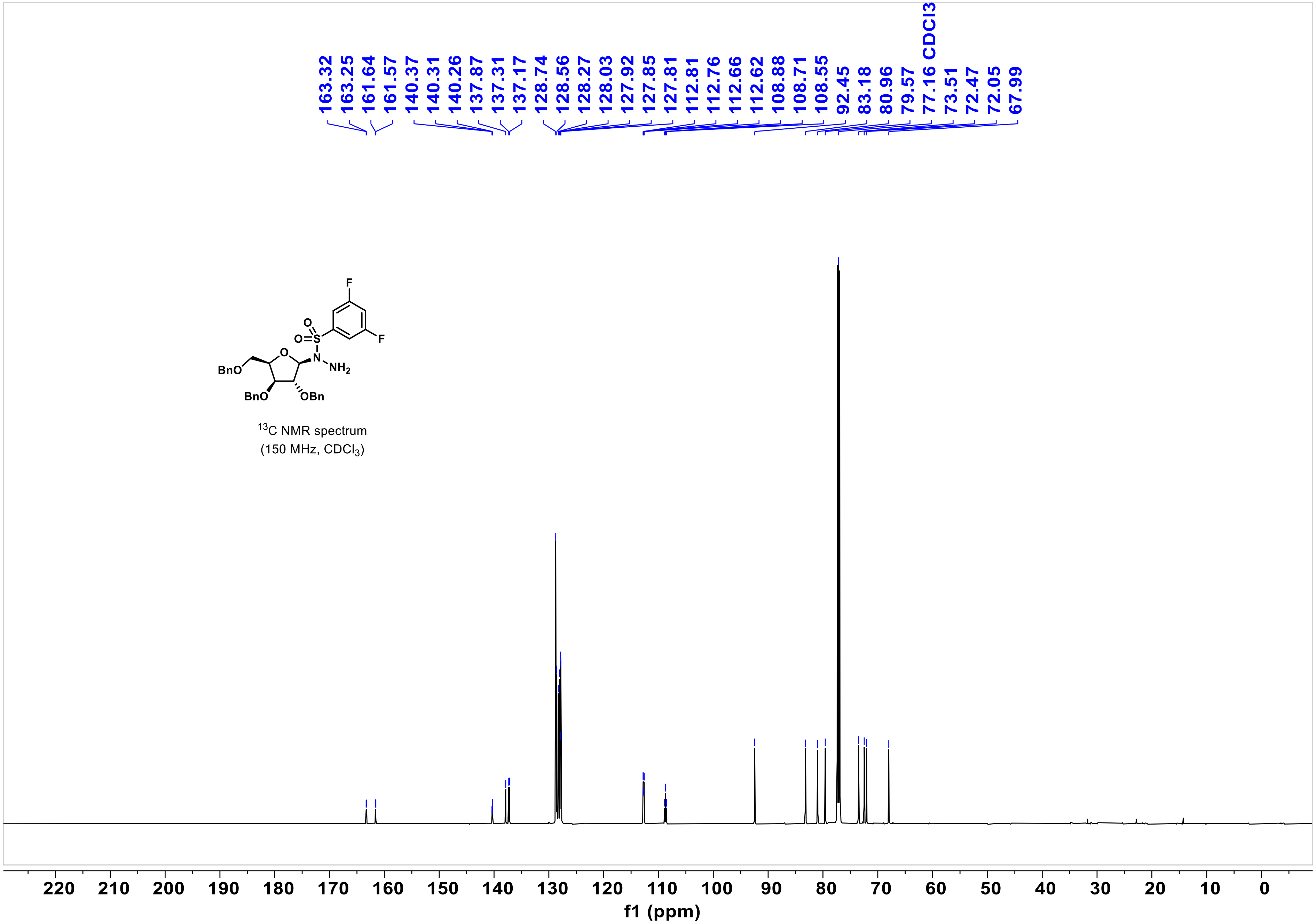

^{13}C NMR of compound **β-SJ5**

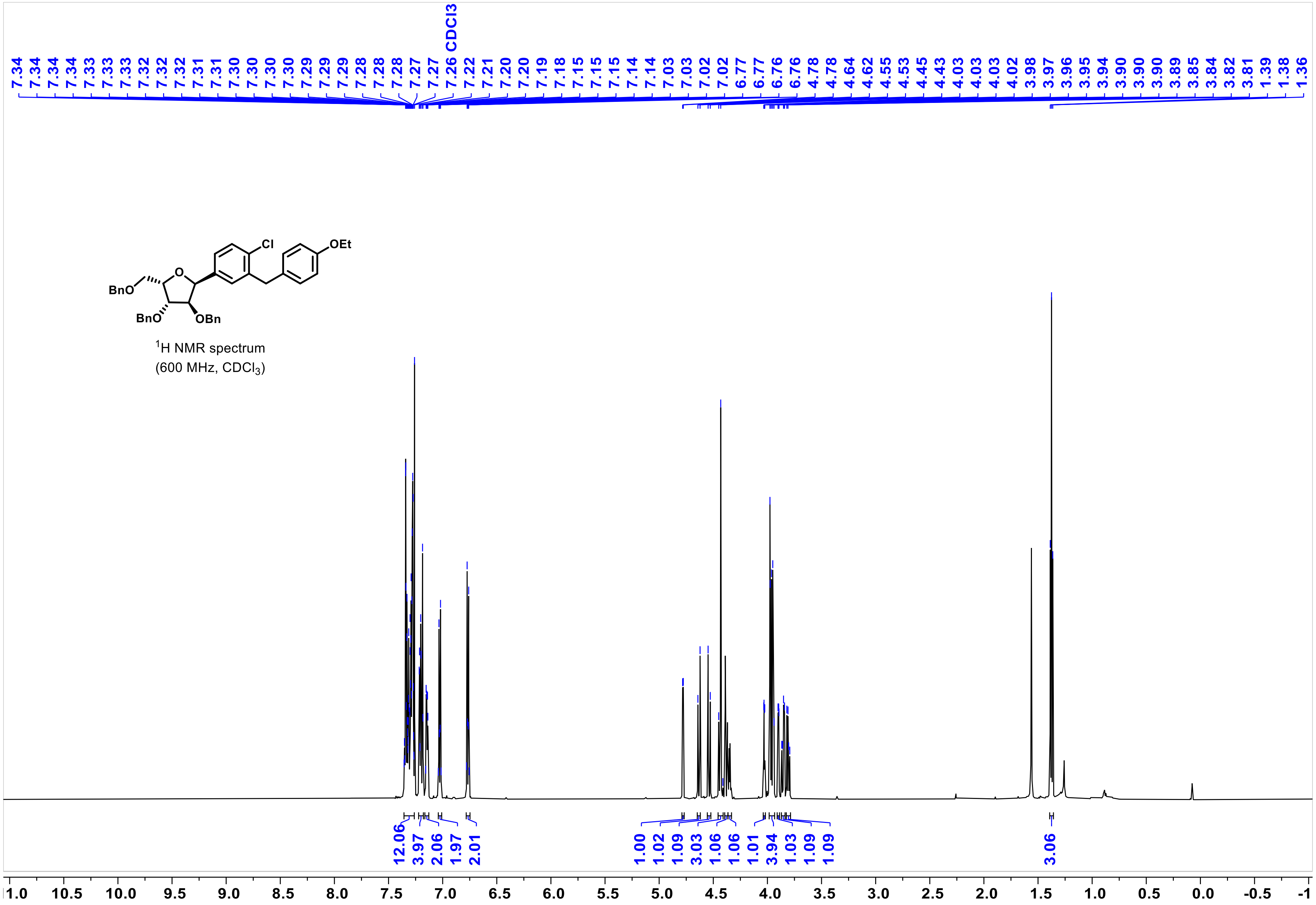

^{1}H NMR of compound **SJ11**

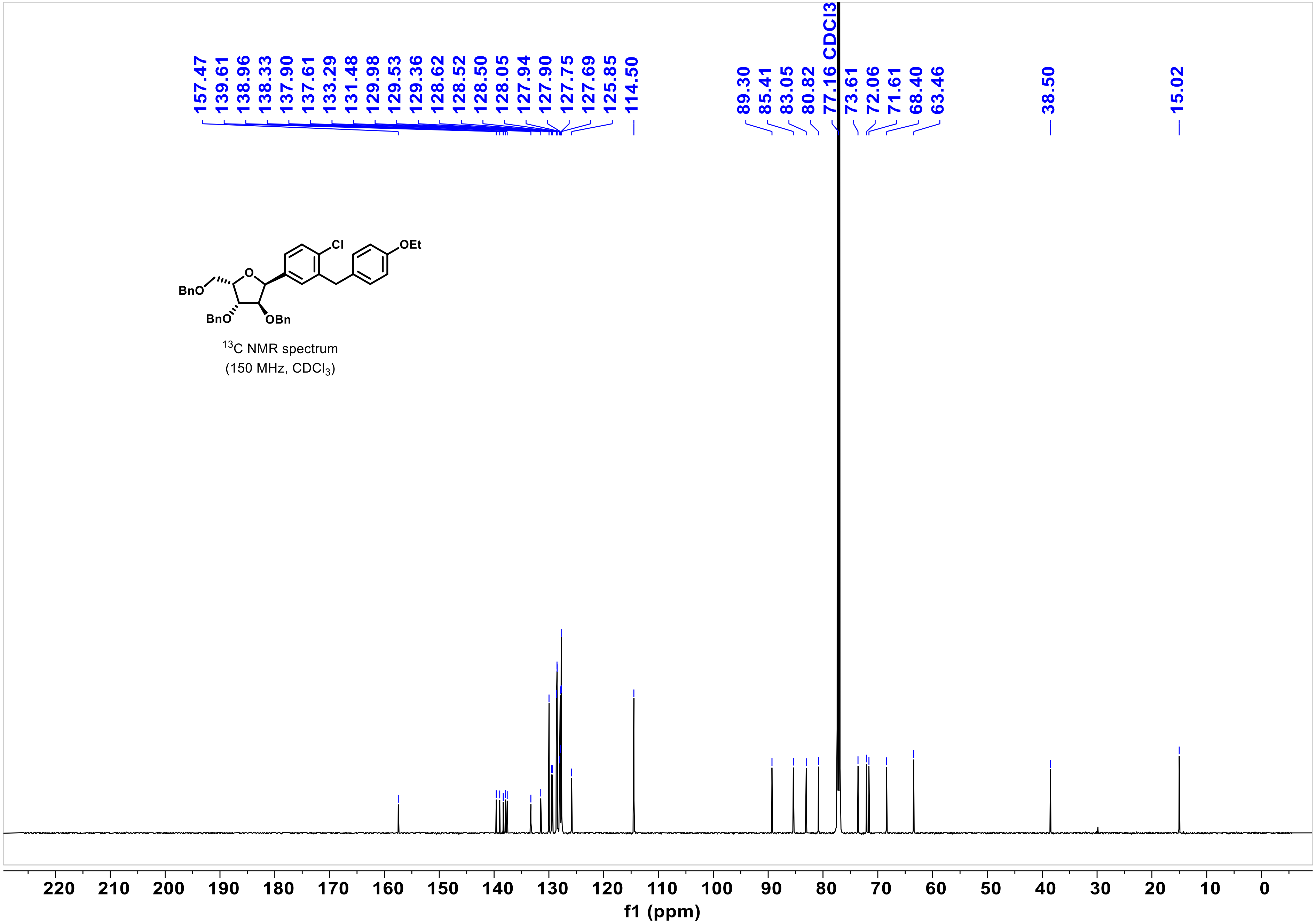

^{13}HC NMR of compound **SJ11**

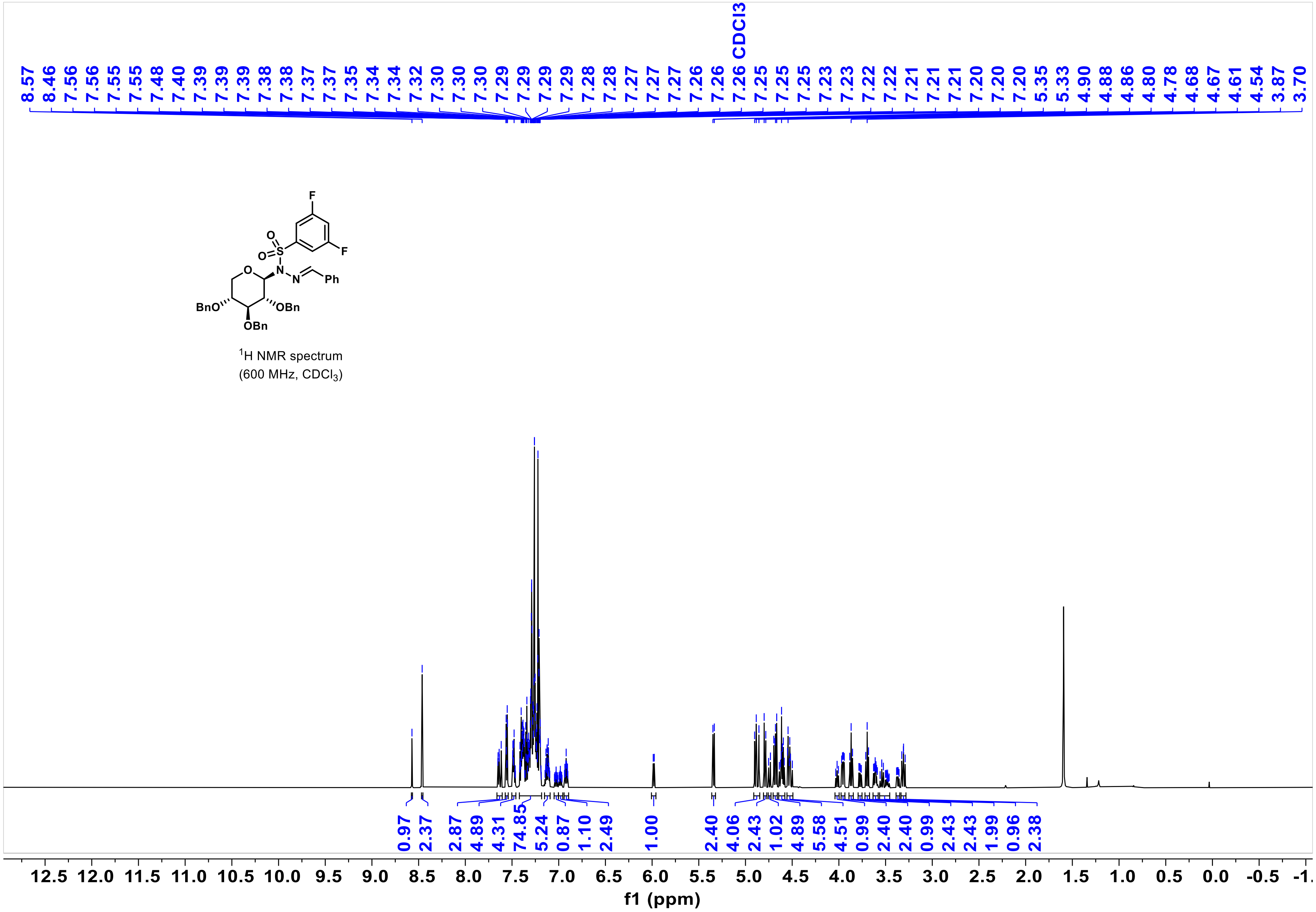

^{1}H NMR of compound **SJ9**

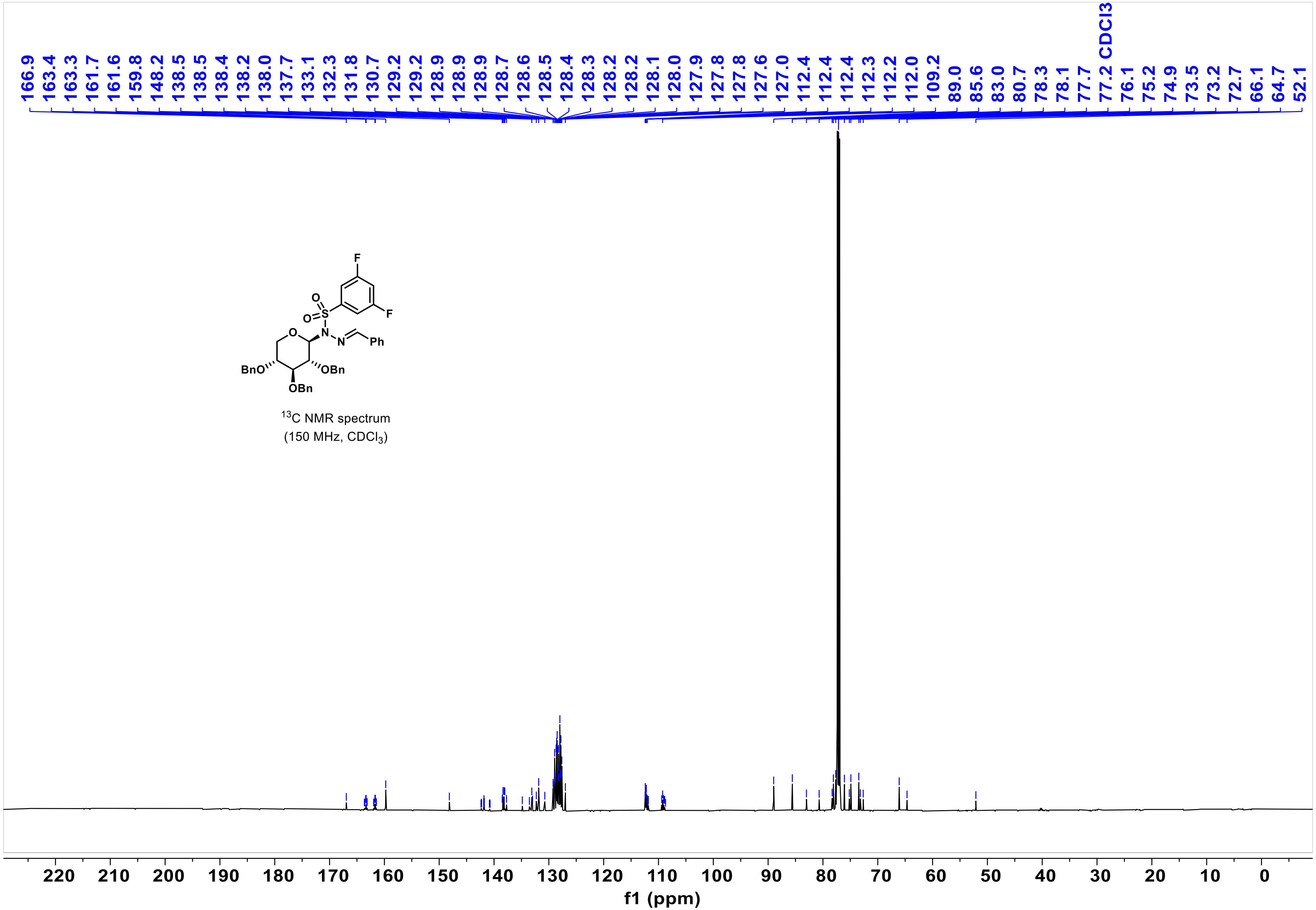

^{13}C NMR of compound **SJ9**

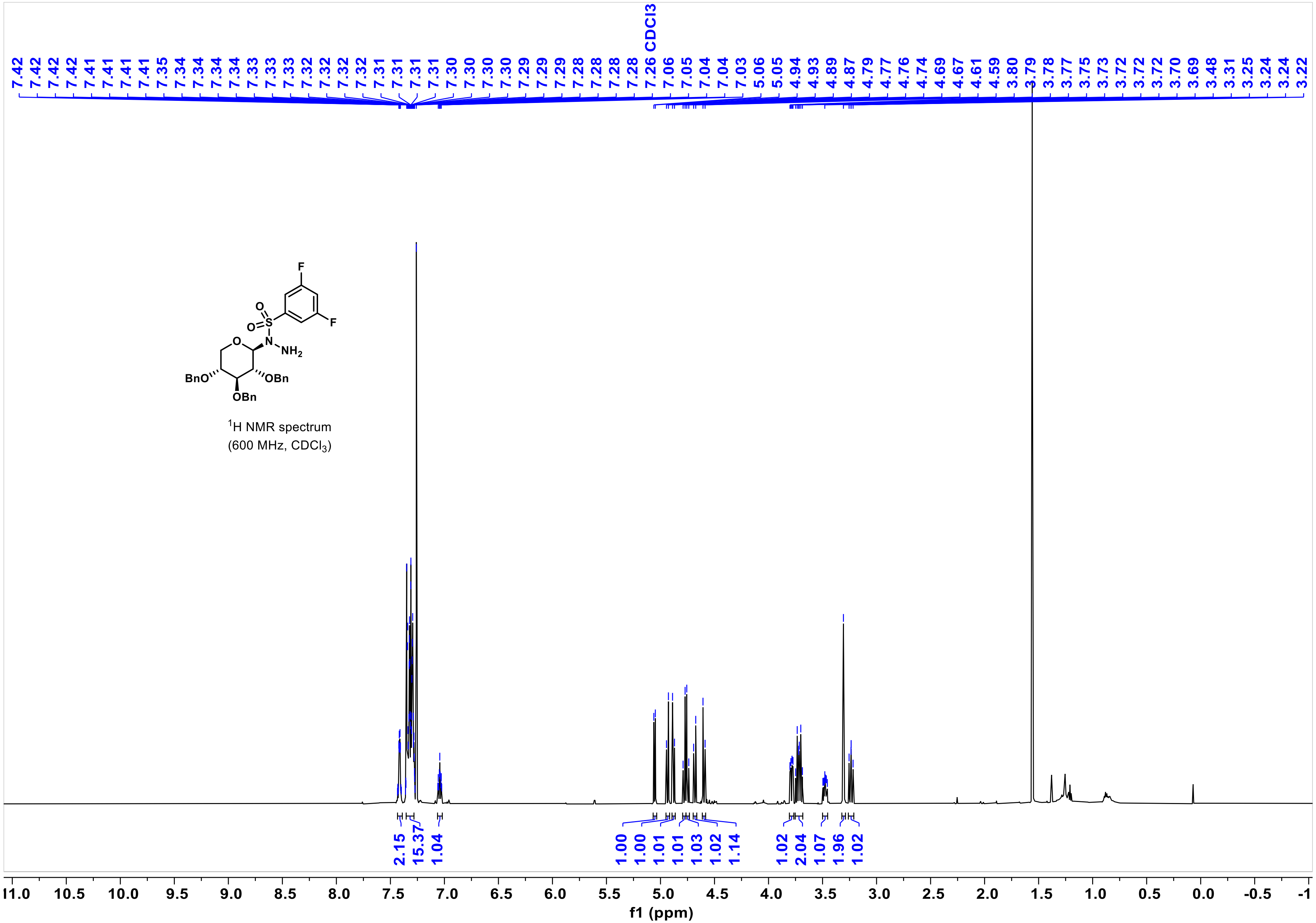

^{1}H NMR of compound **SJ10**

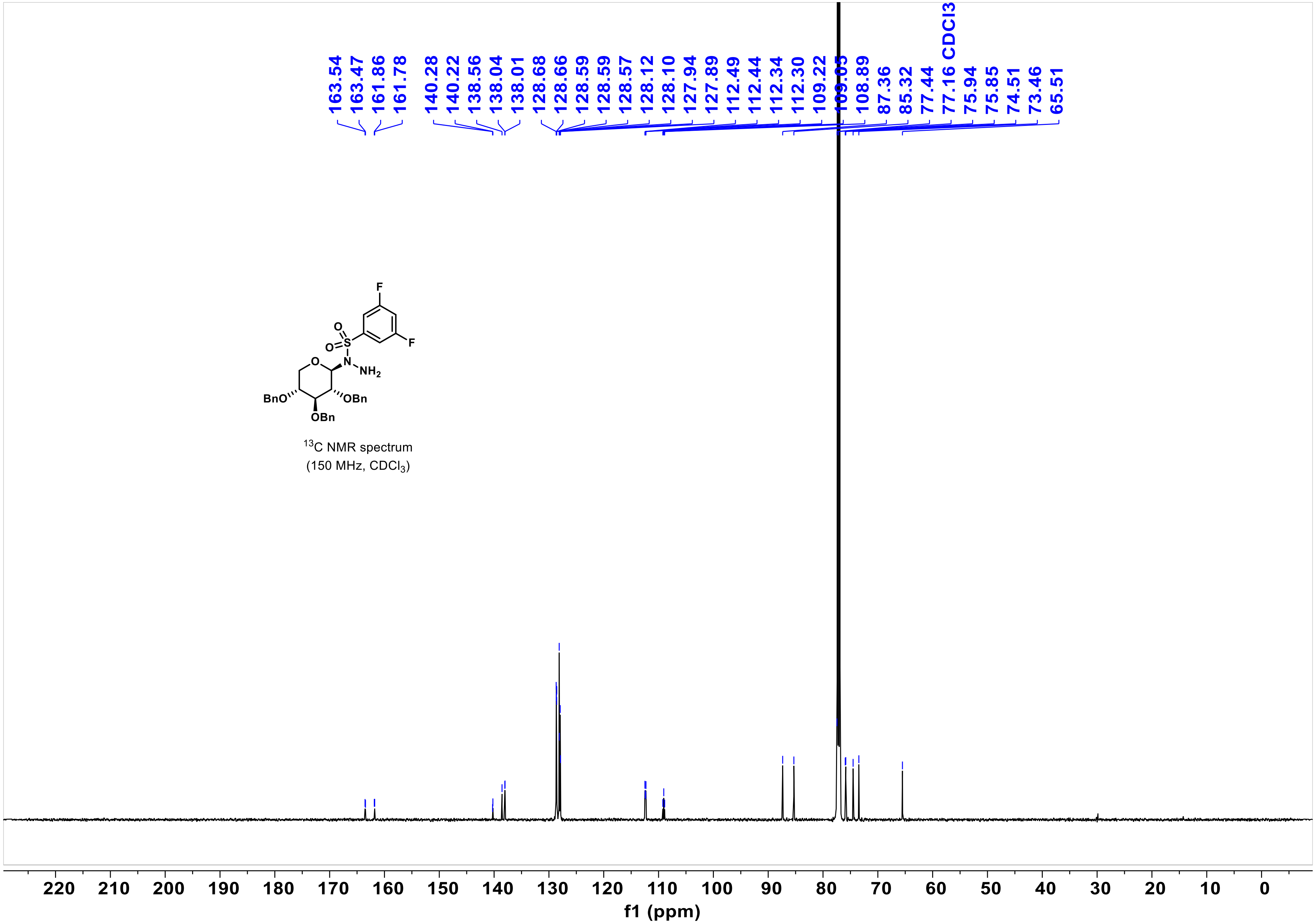

^{13}C NMR of compound **SJ10**

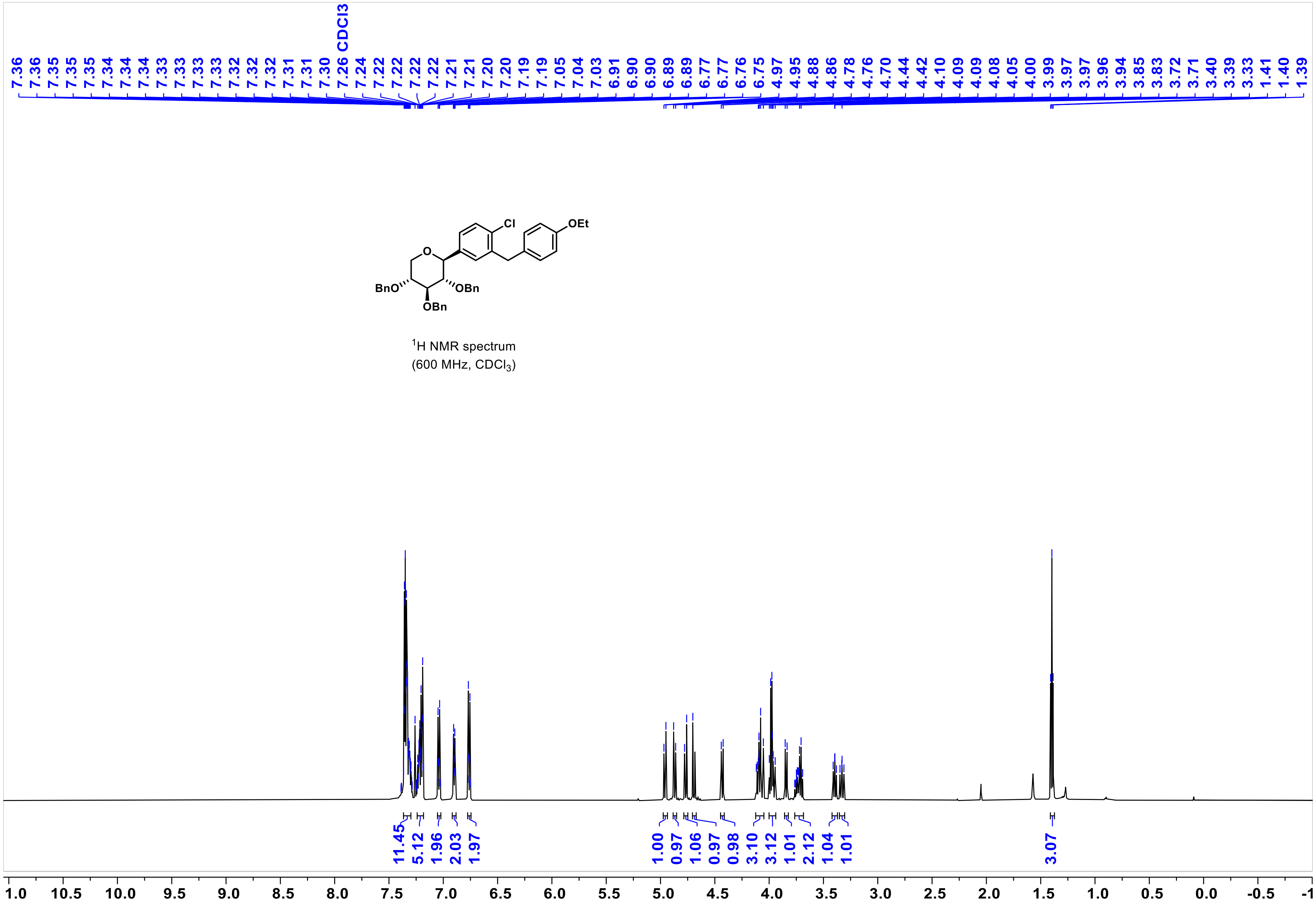

^{1}H NMR of compound **SJ12**

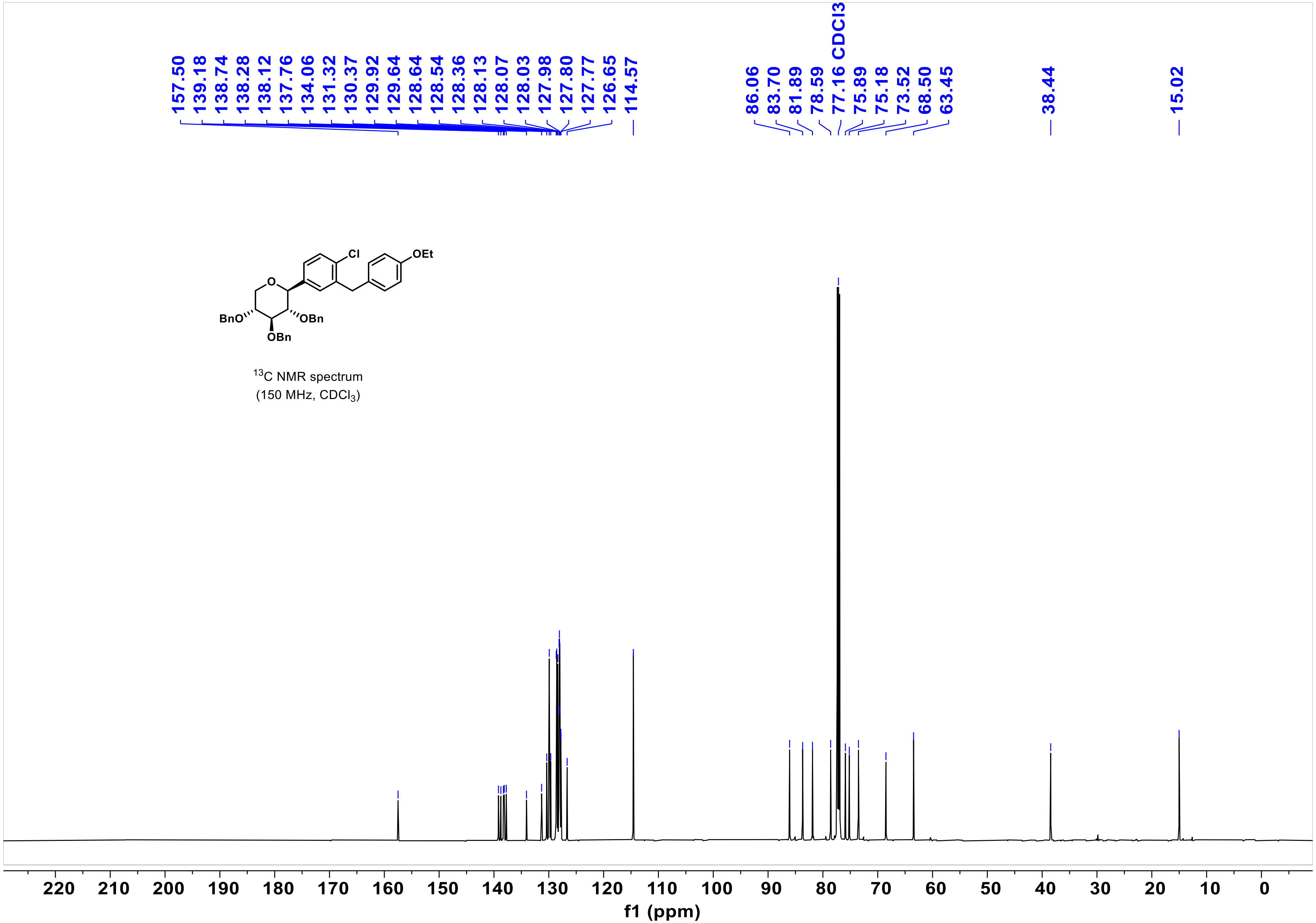

^{13}C NMR of compound **SJ12**
