# Supplementary material for: C-glycoside synthesis through radical cross-coupling of glycohydrazides
Source: Nature. 2026 Jun 22;655(8125):1195–203. doi: 10.1038/s41586-026-10807-x (PMC13421352; doi:10.1038/s41586-026-10807-x)
Supplement: Supplementary file 6 — Peer Review File [file 41586_2026_10807_MOESM6_ESM.pdf]

nature portfolio

Peer Review File

C-glycoside synthesis via radical cross-coupling of glycohydrazides

Corresponding Author: Professor Phil Baran

Any redactions in this file are there to maintain patient confidentiality, the confidentiality of unpublished data, or to remove third-party material.

This file contains all reviewer reports in order by version, followed by all author rebuttals in order by version.

Version 0:

Reviewer comments:

Referee #1

(Remarks to the Author)
The authors present a novel method for access to C-glycosides via a radical cross-coupling of glycohydrazides with aryl iodides and some aryl bromides. The work is very extensive showing a broad range of applications, including stereoretentive coupling (albeit with some erosion) for difficult to access stereoisomers, which will be of great interest to practioners. I find the work is well done, experimentals suitable for reproduction of the work and the referencing is complete. The work builds on strong precedent that the authors published in Science last year where they showed this type of reactivity was possible on non-carbohydrate substrates.
This strong precedent did give me some pause as to the novelty of this method relative to Nature's originality criteria. However, the complexity of what is accomplished here relative to the precedent to access these specific molecules is rather impressive. Carbohydrate chemistry typically relies on lengthy sequences involving many protection/deprotection steps in order to access specific regio/stereoisomers. The application of RCC to this C-glycoside problem, with the updated protocol which is simple and robust based on the data provided, is really a transformational advance for the field. I find the work compelling and well explained/layed out. The figures are busy but easy to follow. I believe this work will be appreciated by a broad synthetic chemistry audience, particularly carbohydrate chemists and medicinal chemists who routinely struggle to synthesize such molecules. That being said, I would recommend this manuscript for publication in Nature. I have noted a few observations that I hope the authors will consider to improve clarity/completeness of the work below

1. L6 is not draw in any figure in the paper only the dNH2-bpy nomenclature which I was unfamiliar with. Its structure was included in the SI but given it is explicitly discussed as part of optimization i would recommend its structure be included in the figure itself for more rapid identification by the reader.
2. The authors report B:a ratios of their reactions throughout the manuscript. As most products are isolated via chromatography it is not explicitly clear if these ratios reflect the ratios of the crude mixture or after purification. When discussing optimization, one would assume these are crude ratios. In substrate tables, it is less clear. I recommend the authors use more explicit language here. If it is the same throughout a simple footnote could be used.
3. I would also like to know if the authors attempted to isolate a single diastereoisomer when the ratios are closer to 1:1. I think this would be particularly useful for the substrates in Figure 4, where difficult to synthesize isomers are obtained in ratios that are 1:1 to 3:1.
4. Figure 5B shows reactivity on ribose and this is compared to the recently published Britton method in the text. Have the authors attempted this coupling on modified ribose cores as was done in the Britton work? Does the selectivity they observe translate? Medicinally relevant riboses often have 2' or 4' substitution or oxygen can be replaced with sulfur. Did the authors attempt any of these. Thioribose is commercially available so could be easily tried.

Referee #2

(Remarks to the Author)
In this article, Kawamata, Aggarwal, Baran and coworkers report the application of their "redox neutral cross-coupling" platform to C-aryl glycoside synthesis. The approach leverages unprotected sulfonyl hydrazine glycosyl donors – accessed

in one step from unprotected sugars – as substrates in Ni-catalyzed radical arylation. Some reaction condition optimization was necessary, however, the underlying reactivity builds trivially upon extensive recent work from the authors (science 2025, ACIE 2025, and related nature 2025, arxiv 2026), which has already outlined the concept, mechanistic framework and synthetic implementation of the “redox-neutral cross-coupling” platform. Since the platform is not new, the advance of the work then hinges on the importance of the products that can be obtained from the method and whether the authors’ approach fundamentally transforms synthetic access to this product class. In my view, the method has many strengths, but that scope, selectivity, and accessibility are on par with other methods and strategies.

The soaring strength of the method is synthetic practicality attendant to the compatibility of both the hydrazine donor synthesis and Ni-arylation conditions with unprotected substrates. Synthetic accessibility and scalability is maximized in cases where substrate bias enables good stereocontrol. For example, excellent levels of beta-selectivity are observed using glucose- and ribose-configured donors, which offers expedient and comprehensive entry into the c-aryl glycoside type SGLT2 inhibitor family and C-linked ribonucleoside analogs. Conversely, exclusive alpha-glycosylation is observed with a mannosyl donor, 46. Here, the scope of the reaction is under-explored given the authors’ claims of generality: for example, unprotected galactosides, rhamnosides, fucosides, and other furanosides inter alia are not systematically assessed.

For more challenging substrate classes (e.g. 2-deoxypyranosides and furanosides), the authors attempt to apply their “stereoretentive coupling conditions" to control glycosylation stereoselectivity. Success is partial, and the modest catalyst-controlled selectivity (e.g. from 1:2 to 3:1) does not rise to the level of synthetic need. Here, the requirement for O-protected sugars (“to simplify the preparation and purification of either anomeric series”) runs contrary to the overall synthetic premise of direct, selective, and general glycosylation.

Collectively then, the selectivity profile is unremarkable: some substrates are (as they are known to be) innately selective, others are not, and prospects for external stereocontrol are not yet realized. Put another way, the method leads to outcomes similar to those obtained using other radical arylation methods, but without the same level of tunable control (for example, 10.1021/acs.oprd.5c00302; 10.1021/acscatal.4c02322). I appreciate that these two arylation methods use protected rather than unprotected donors, but many compounds of type 12 (c.f. Fig 1) are commercial, easy to handle/purify, and step-competitive with the authors’ approach.

The arylation method also works at other positions (C2-C6) of the sugar molecule, although here the need for differential protection strategies to install the hydrazide at the requisite position cannot be avoided here, and substrate controlled diastereoselectivities are generally modest. The authors offer no insights into reaction mechanism, which presumably does not diverge substantially from the pathways outlined in the Science 2025 paper. This is fine, though it underscores that the present work is an application of an established platform. It might be interesting to follow up on the striking role of base in governing stereoselectivity, however.

Again, the work has many positive elements, and I expect will become a go-to method for access to selected substrate classes. However, the method does not fundamentally re-shape the landscape of stereoselective glycosylation: biased substrates remain generally accessible, and challenging substrates remain challenging. Here, the method is “not better, just different.” My suggestion is that the authors scale back what they are trying to claim (general access), and focus on the strengths of the method (beta glucosides and ribosides). My view is that other aspects of the story (e.g. catalyst control in challenging substrates, C2-C6 arylation) fall short, and deserve additional attention and development.

Minor:
In the introduction, the authors write that “their [SGLT2 inhibitors] clinical success illustrates how a robust C-C linkage can translate into favorable metabolic stability and durable biological activity…”, which seems to misattribute the myriad and complex determinants of clinical success to metabolic stability alone. A more nuanced rephrase here would improve scholarship without undermining the case for the importance of C-aryl glycosides.

Cross-coupling strategies are conventionally redox neutral. I appreciate that the authors have extensively developed radical cross-couplings initiated by redox steps, but the branding around a “redox neutral cross-coupling” platform makes less sense from a wider lens.

The authors could be a bit more comprehensive in discussing precedent for selective manipulations of unprotected sugars in the conclusion (e.g. “This work represents another step in dismantling the long-held belief that meaningful synthetic elaboration of carbohydrates demands extensive protecting groups and redox manipulations”). The potential advantages of working with unprotected sugar substrates is not a new insight, and an increasingly vibrant area of research (For example: 10.1038/s41586-022-04958-w, 10.1038/s41586-024-07695-4, 10.1038/s41586-020-1937-1, 10.1021/jacs.5b13384, along with nearly all chemoenzymatic approaches).

Referee #3

(Remarks to the Author)

The manuscript by Baran and co-workers describes the application of anomeric glycosyl hydrazides for the synthesis of aryl C-glycosides via Ni-mediated radical coupling. In the case of unprotected gluco configured sugars high levels of beta selectivity are obtained, provided TMG is present in the system. This is a very useful observation, and could lead to more

general methods for controlling selectivity. With protected systems, selectivity is attenuated, although the major anomer is formed through stereoretention. The authors also demonstrate that the hydrazide can be placed at various other positions of the ring to introduce aryl rings, albiet with modest selectivity. That being said this latter transformation is extremely challenging using conventional methods. All told, this is a solid piece of work, and I am supportive of publication in Nature once the following questions and comments have been addressed.
Questions/Comments
For compounds 79-96 alpha:beta is not the correct terminology. More appropriate would be gluco:manno (79-84), allo:gluco (85-90) and galacto:gluco (91-96). Similarly, compounds 103 and 104 should be arabino:ribo and 106/106 should be xylo:ribo please fix in manuscript and SI.
Can salmochelin be obtained without the need for benzyl ethers/esters?
Figure 4 – I would be extremely hesitant to call a reaction where a single anomer of substrate leads to a 2:1 or 3:1 mixture of products stereoretentive. Furthermore, in the SI compound 68 is reported as a 2:# mixture or isomers while in the manuscript it is reported as a 2:1 mixture. Based off of the NMR I would say the selectivity is likely 2.3:1. Please check for consistency throughout the manuscript and SI.
The sentence "This work represents another step17,20,48,49 in dismantling the long-held belief that meaningful synthetic elaboration of carbohydrates demands extensive protecting groups and redox manipulations." Is misleading and should be amended. Yes there exists a plethora of radical-based methods for anomeric C-C coupling, however, even in the case of the current manuscript, elaboration at other positions requires extensive protecting group manipulation. Unfortunately, this regiochemistry remains one of the biggest challenges in carbohydrate chemistry and is still one that has yet to be fully addressed.

SI:
Compound SF19 – what does it mean of a peak to integrate at 0H? The hardcopy of the NMR shows 13 protons, there should be 12. What is the impurity?
Compound SF20 – there are too many protons (13 should be 12) in the 1H NMR spectrum. Furthermore, the methyl singlet is not reported in the list of chemical shifts.
Compound SF27 why is a peak reported at 8.52 ppm? Is it because it is expected? There is nothing there. I appreciate that protons on nitrogen can exchange with residual water in the CD3CN, however if nothing is there do not report it.
Please check all NMR data (both spectra and data reporting) to make sure these types of errors do not appear elsewhere.

Version 1:

Reviewer comments:

Referee #1

(Remarks to the Author)
The reviewed mansucript addresses referee comments satisfactorily in my opinion and I recommend publication of the manuscript in Nature.

Referee #2

(Remarks to the Author)
The authors have made a sincere effort to address my concerns. And while it remains to be seen whether this approach can be further developed to overcome remaining significant limitations, I view the strengths to outweigh the weaknesses and I now support publication.

Referee #3

(Remarks to the Author)
Upon reviewing the revised manuscript and authors response they have addressed my previous concerns satisfactorily. I am therefore supportive of publication.

Open Access This Peer Review File is licensed under a Creative Commons Attribution 4.0 International License, which permits use, sharing, adaptation, distribution and reproduction in any medium or format, as long as you give appropriate credit to the original author(s) and the source, provide a link to the Creative Commons license, and indicate if changes were made.
In cases where reviewers are anonymous, credit should be given to 'Anonymous Referee' and the source.
The images or other third party material in this Peer Review File are included in the article's Creative Commons license, unless indicated otherwise in a credit line to the material. If material is not included in the article's Creative Commons license and your intended use is not permitted by statutory regulation or exceeds the permitted use, you will need to obtain permission directly from the copyright holder.
To view a copy of this license, visit https://creativecommons.org/licenses/by/4.0/

Point-by-Point Response to Reviewer's Questions

Comments from Reviewers

Referee 1

The authors present a novel method for access to C-glycosides via a radical cross-coupling of glycohydrazides with aryl iodides and some aryl bromides. The work is very extensive showing a broad range of applications, including stereoretentive coupling (albeit with some erosion) for difficult to access stereoisomers, which will be of great interest to practioners. I find the work is well done, experimentals suitable for reproduction of the work and the referencing is complete. The work builds on strong precedent that the authors published in Science last year where they showed this type of reactivity was possible on non-carbohydrate substrates.
This strong precedent did give me some pause as to the novelty of this method relative to Nature's originality criteria. However, the complexity of what is accomplished here relative to the precedent to access these specific molecules is rather impressive. Carbohydrate chemistry typically relies on lengthy sequences involving many protection/deprotection steps in order to access specific regio/stereoisomers. The application of RCC to this C-glycoside problem, with the updated protocol which is simple and robust based on the data provided, is really a transformational advance for the field. I find the work compelling and well explained/layed out. The figures are busy but easy to follow. I believe this work will be appreciated by a broad synthetic chemistry audience, particularly carbohydrate chemists and medicinal chemists who routinely struggle to synthesize such molecules. That being said, I would recommend this manuscript for publication in Nature. I have noted a few observations that I hope the authors will consider to improve clarity/completeness of the work below

1. L6 is not draw in any figure in the paper only the dNH2-bpy nomenclature which I was unfamiliar with. Its structure was included in the SI but given it is explicitly discussed as part of optimization i would recommend its structure be included in the figure itself for more rapid identification by the reader.
2. The authors report B:a ratios of their reactions throughout the manuscript. As most products are isolated via chromatography it is not explicitly clear if these ratios reflect the ratios of the

crude mixture or after purification. When discussing optimization, one would assume these are crude ratios. In substrate tables, it is less clear. I recommend the authors use more explicit language here. If it is the same throughout a simple footnote could be used.

3. I would also like to know if the authors attempted to isolate a single diastereoisomer when the ratios are closer to 1:1. I think this would be particularly useful for the substrates in Figure 4, where difficult to synthesize isomers are obtained in ratios that are 1:1 to 3:1.

4. Figure 5B shows reactivity on ribose and this is compared to the recently published Britton method in the text. Have the authors attempted this coupling on modified ribose cores as was done in the Britton work? Does the selectivity they observe translate? Medicinally relevant riboses often have 2' or 4' substitution or oxygen can be replaced with sulfur. Did the authors attempt any of these. Thioribose is commercially available so could be easily tried.

Referee 2

In this article, Kawamata, Aggarwal, Baran and coworkers report the application of their "redox neutral cross-coupling" platform to C-aryl glycoside synthesis. The approach leverages unprotected sulfonyl hydrazine glycosyl donors – accessed in one step from unprotected sugars – as substrates in Ni-catalyzed radical arylation. Some reaction condition optimization was necessary, however, the underlying reactivity builds trivially upon extensive recent work from the authors (science 2025, ACIE 2025, and related nature 2025, arxiv 2026), which has already outlined the concept, mechanistic framework and synthetic implementation of the "redox-neutral cross-coupling" platform. Since the platform is not new, the advance of the work then hinges on the importance of the products that can be obtained from the method and whether the authors' approach fundamentally transforms synthetic access to this product class. In my view, the method has many strengths, but that scope, selectivity, and accessibility are on par with other methods and strategies.

The soaring strength of the method is synthetic practicality attendant to the compatibility of both the hydrazine donor synthesis and Ni-arylation conditions with unprotected substrates. Synthetic accessibility and scalability is maximized in cases where substrate bias enables good stereocontrol. For example, excellent levels of beta-selectivity are observed using glucose- and ribose-configured donors, which offers expedient and comprehensive entry into the c-aryl

glycoside type SGLT2 inhibitor family and C-linked ribonucleoside analogs. Conversely, exclusive alpha-glycosylation is observed with a mannosyl donor, 46. Here, the scope of the reaction is under-explored given the authors' claims of generality: for example, unprotected galactosides, rhamnosides, fucosides, and other furanosides inter alia are not systematically assessed.

For more challenging substrate classes (e.g. 2-deoxypyranosides and furanosides), the authors attempt to apply their "stereoretentive coupling conditions" to control glycosylation stereoselectivity. Success is partial, and the modest catalyst-controlled selectivity (e.g. from 1:2 to 3:1) does not rise to the level of synthetic need. Here, the requirement for O-protected sugars ("to simplify the preparation and purification of either anomeric series") runs contrary to the overall synthetic premise of direct, selective, and general glycosylation.

Collectively then, the selectivity profile is unremarkable: some substrates are (as they are known to be) innately selective, others are not, and prospects for external stereocontrol are not yet realized. Put another way, the method leads to outcomes similar to those obtained using other radical arylation methods, but without the same level of tunable control (for example, 10.1021/acs.oprd.5c00302; 10.1021/acscatal.4c02322). I appreciate that these two arylation methods use protected rather than unprotected donors, but many compounds of type 12 (c.f. Fig 1) are commercial, easy to handle/purify, and step-competitive with the authors' approach.

The arylation method also works at other positions (C2-C6) of the sugar molecule, although here the need for differential protection strategies to install the hydrazide at the requisite position cannot be avoided here, and substrate controlled diastereoselectivities are generally modest. The authors offer no insights into reaction mechanism, which presumably does not diverge substantially from the pathways outlined in the Science 2025 paper. This is fine, though it underscores that the present work is an application of an established platform. It might be interesting to follow up on the striking role of base in governing stereoselectivity, however.

Again, the work has many positive elements, and I expect will become a go-to method for access to selected substrate classes. However, the method does not fundamentally re-shape the

landscape of stereoselective glycosylation: biased substrates remain generally accessible, and challenging substrates remain challenging. Here, the method is “not better, just different.” My suggestion is that the authors scale back what they are trying to claim (general access), and focus on the strengths of the method (beta glucosides and ribosides). My view is that other aspects of the story (e.g. catalyst control in challenging substrates, C2-C6 arylation) fall short, and deserve additional attention and development.

Minor:
In the introduction, the authors write that “their [SGLT2 inhibitors] clinical success illustrates how a robust C-C linkage can translate into favorable metabolic stability and durable biological activity…”, which seems to misattribute the myriad and complex determinants of clinical success to metabolic stability alone. A more nuanced rephrase here would improve scholarship without undermining the case for the importance of C-aryl glycosides.

Cross-coupling strategies are conventionally redox neutral. I appreciate that the authors have extensively developed radical cross-couplings initiated by redox steps, but the branding around a “redox neutral cross-coupling” platform makes less sense from a wider lens.

The authors could be a bit more comprehensive in discussing precedent for selective manipulations of unprotected sugars in the conclusion (e.g. “This work represents another step in dismantling the long-held belief that meaningful synthetic elaboration of carbohydrates demands extensive protecting groups and redox manipulations”). The potential advantages of working with unprotected sugar substrates is not a new insight, and an increasingly vibrant area of research (For example: 10.1038/s41586-022-04958-w, 10.1038/s41586-024-07695-4, 10.1038/s41586-020-1937-1, 10.1021/jacs.5b13384, along with nearly all chemoenzymatic approaches).

Referee 3

The manuscript by Baran and co-workers describes the application of anomeric glycosyl hydrazides for the synthesis of aryl C-glycosides via Ni-mediated radical coupling. In the case of unprotected gluco configured sugars high levels of beta selectivity are obtained, provided TMG

is present in the system. This is a very useful observation, and could lead to more general methods for controlling selectivity. With protected systems, selectivity is attenuated, although the major anomer is formed through stereoretention. The authors also demonstrate that the hydrazide can be placed at various other positions of the ring to introduce aryl rings, albiet with modest selectivity. That being said this latter transformation is extremely challenging using conventional methods. All told, this is a solid piece of work, and I am supportive of publication in Nature once the following questions and comments have been addressed.

Questions/Comments

For compounds 79-96 alpha:beta is not the correct terminology. More appropriate would be gluco:manno (79-84), allo:gluco (85-90) and galacto:gluco (91-96). Similarly, compounds 103 and 104 should be arabino:ribo and 106/106 should be xylo:ribo please fix in manuscript and SI.

Can salmochelin be obtained without the need for benzyl ethers/esters?

Figure 4 – I would be extremely hesitant to call a reaction where a single anomer of substrate leads to a 2:1 or 3:1 mixture of products stereoretentive. Furthermore, in the SI compound 68 is reported as a 2:# mixture or isomers while in the manuscript it is reported as a 2:1 mixture. Based off of the NMR I would say the selectivity is likely 2.3:1. Please check for consistency throughout the manuscript and SI.

The sentence “This work represents another step17,20,48,49 in dismantling the long-held belief that meaningful synthetic elaboration of carbohydrates demands extensive protecting groups and redox manipulations.” Is misleading and should be amended. Yes there exists a plethora of radical-based methods for anomeric C-C coupling, however, even in the case of the current manuscript, elaboration at other positions requires extensive protecting group manipulation. Unfortunately, this regiochemistry remains one of the biggest challenges in carbohydrate chemistry and is still one that has yet to be fully addressed.

SI:

Compound SF19 – what does it mean of a peak to integrate at 0H? The hardcopy of the NMR shows 13 protons, there should be 12. What is the impurity?

Compound SF20 – there are too many protons (13 should be 12) in the 1H NMR spectrum. Furthermore, the methyl singlet is not reported in the list of chemical shifts.

Compound SF27 why is a peak reported at 8.52 ppm? Is it because it is expected? There is nothing there. I appreciate that protons on nitrogen can exchange with residual water in the CD3CN, however if nothing is there do not report it.

Please check all NMR data (both spectra and data reporting) to make sure these types of errors do not appear elsewhere.

Response to Reviewer's Comments

Referee 1

[Critique 1]

1. L6 is not draw in any figure in the paper only the dNH2-bpy nomenclature which I was unfamiliar with. Its structure was included in the SI but given it is explicitly discussed as part of optimization i would recommend its structure be included in the figure itself for more rapid identification by the reader

[Response]

We sincerely appreciate reviewer's comments. We now have added the structure of dNH2-bpy to Figure 2A.

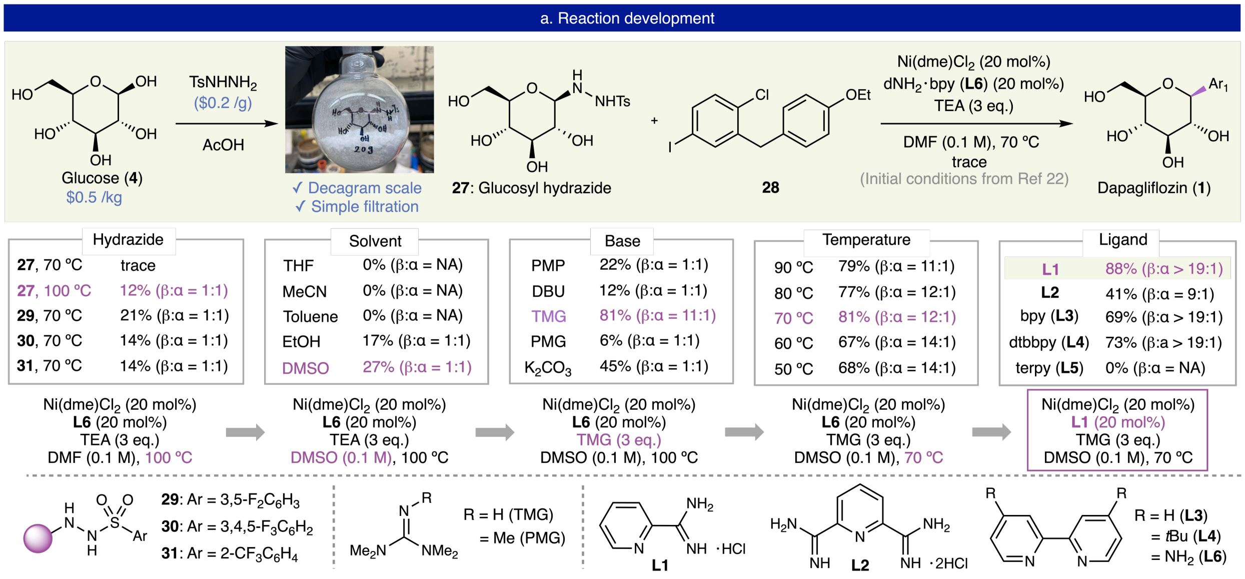

[Critique 2]

2. The authors report B:a ratios of their reactions throughout the manuscript. As most products are isolated via chromatography it is not explicitly clear if these ratios reflect the ratios of the

crude mixture or after purification. When discussing optimization, one would assume these are crude ratios. In substrate tables, it is less clear. I recommend the authors use more explicit language here. If it is the same throughout a simple footnote could be used.

[Response]

We sincerely appreciate the reviewer's comments and concerns. All the reported ratios reflect the ratios of the crude mixture. As suggested, we have added the footnote to Figure 2-6: "Diastereomeric ratio was determined from the crude reaction mixture by ^{1}H NMR or LCMS."

[Critique 3]

3. I would also like to know if the authors attempted to isolate a single diastereoisomer when the ratios are closer to 1:1. I think this would be particularly useful for the substrates in Figure 4, where difficult to synthesize isomers are obtained in ratios that are 1:1 to 3:1.

[Response]

We sincerely appreciate the reviewer's feedback. Yes, we are able to isolate single diastereomers for glucose, ribose, and 2-deoxyribose cross-coupled products (**94**, **98**, and **100**) presented in new Figure 5 (used to be Figure 4) using hexane/ether solvent system on PTLC. 2-deoxyglucose **96** is the only exception using current solvent system on PTLC. These were included in the Supporting Information [see SI—6.3.1 Characterization of C1-Hydrazides and RCC-products (for stereoretentive coupling)].

[Critique 4]

4. Figure 5B shows reactivity on ribose and this is compared to the recently published Britton method in the text. Have the authors attempted this coupling on modified ribose cores as was done in the Britton work? Does the selectivity they observe translate? Medicinally relevant riboses often have 2' or 4' substitution or oxygen can be replaced with sulfur. Did the authors attempt any of these. Thioribose is commercially available so could be easily tried.

[Response]

We sincerely appreciate the reviewer's feedback. We have not explored modified ribose cores such as 2-thioribose and 4-thioribose, as these compounds are not commercially available. Moreover, their derivatives are prohibitively expensive. For example, 4′-thiouridine (~$250 / 4 mg), 4′-thioguanosine (~$5700 / 250 mg), 5-methyl-4′-thiouridine (~$6,576 / 250 mg), and 4′-thioadenosine (~$5130 / 250 mg) are all associated with very high costs. In addition, the synthesis of protected thioribose derivatives (e.g., Bn-protected thioribose) typically requires more than eight steps (DOI: 10.1080/15257779408013205), further limiting their practical accessibility. More importantly, the primary focus of our work is the direct C1 glycosylation of unprotected sugars, emphasizing operational simplicity and broad applicability without the need for protecting groups. As such, we have not extended our study to these structurally modified and less accessible thioribose substrates.

Referee 2

[Critique 1]

Since the platform is not new, the advance of the work then hinges on the importance of the products that can be obtained from the method and whether the authors' approach fundamentally transforms synthetic access to this product class. In my view, the method has many strengths, but that scope, selectivity, and accessibility are on par with other methods and strategies.

[Response]

We have elaborated the critical differences between our approach and prior methods in this response letter. To summarize the key points:

(1) A practical glycosyl radical donor for radical cross-coupling has not been available.

At first glance, this statement may seem counterintuitive, because numerous glycosyl radical donors have been developed, as summarized in Figure 1. However, when these donors are evaluated simultaneously from the standpoints of practicality—how readily, reliably, and scalably the donor can be prepared—and reactivity—whether the donor enables productive

radical cross-coupling rather than only simple radical addition - the current landscape is remarkably limited.

For example, donor **17** developed by Koh et al. is, in our view, a practical glycosyl radical donor because it can be prepared in one step from the corresponding sugar. However, its demonstrated reactivity has thus far been largely confined to simple radical addition processes, such as Giese-type or Minisci reactions, rather than modular radical cross-coupling. Conversely, donors **15** and **16** have been shown to participate in radical cross-coupling, but their preparation is significantly more involved, limiting their broader utility as general synthetic building blocks.

Thus, we contend that glycohydrazides are currently unique in satisfying both requirements: they are readily accessible from native sugars and competent radical precursors for modular radical cross-coupling. This dual practicality/reactivity profile is the central reason we view this discovery as significant.

We have also carefully considered the reviewer's point that radical coupling platforms themselves are not new. We agree with this broader statement. However, the key advance here is not simply the use of radical chemistry, but the identification of a practical glycosyl radical donor class that enables cross-coupling at a level of generality not previously available. As a benchmark, the development of donor **17** was reported in Nature (DOI: 10.1038/s41586-024-07548-0), underscoring the recognized importance of practical glycosyl radical donors even when their demonstrated reactivity is limited primarily to radical addition. In the present work, the donor class is similarly practical, but it further enables modular radical cross-coupling with a scope that extends well beyond simple Giese-type addition. In that context, we believe the advance represents a substantive shift in what glycosyl radical chemistry can achieve.

(2) Stereoretentive radical cross-coupling at the glycosyl position is, to our knowledge, unprecedented.

We respectfully caution against conflating our recent work for stereoretentive radical coupling in non-glycosidic settings with the present work. Carbohydrate chemistry is a well-established

subdiscipline with its own stereochemical challenges, conventions, and strategic imperatives, and the glycosidic bond occupies a uniquely central role within it. To our knowledge, stereoretentive radical coupling at the glycosyl position has no precedent in the literature. Furthermore, and critically, we have demonstrated experimental access to stereoisomers that have remained inaccessible by any existing radical-based approach — this is not merely a conceptual reframing.

We therefore believe that this stereoretentive aspect alone could constitute a significant conceptual advance. Prior to this work, the idea that a glycosyl radical precursor could engage in a cross-coupling process while retaining its stereochemistry would have been viewed unreachable especially when the anomeric effect is overridden. Demonstrating that such a pathway is viable changes the way chemists can think about stereochemical control in radical C-glycoside synthesis. In our view, this represents a clear conceptual breakthrough, even as further improvements in scope and selectivity remain important future goals.

[Critique 2]

Conversely, exclusive alpha-glycosylation is observed with a mannosyl donor, 46. Here, the scope of the reaction is under-explored given the authors' claims of generality: for example, unprotected galactosides, rhamnosides, fucosides, and other furanosides inter alia are not systematically assessed.

[Response]

We thank the reviewer for this comment. In the previous version, additional sugar substrates were included in the Supporting Information (SI) as the reaction conditions were still being optimized. By leveraging the ring–chain tautomerization of sugar hydrazides, combined with systematic screening of ligands and bases, we have successfully expanded the substrate scope. The optimized results are now presented in the main text of the revised manuscript (see manuscript, Figure 3).

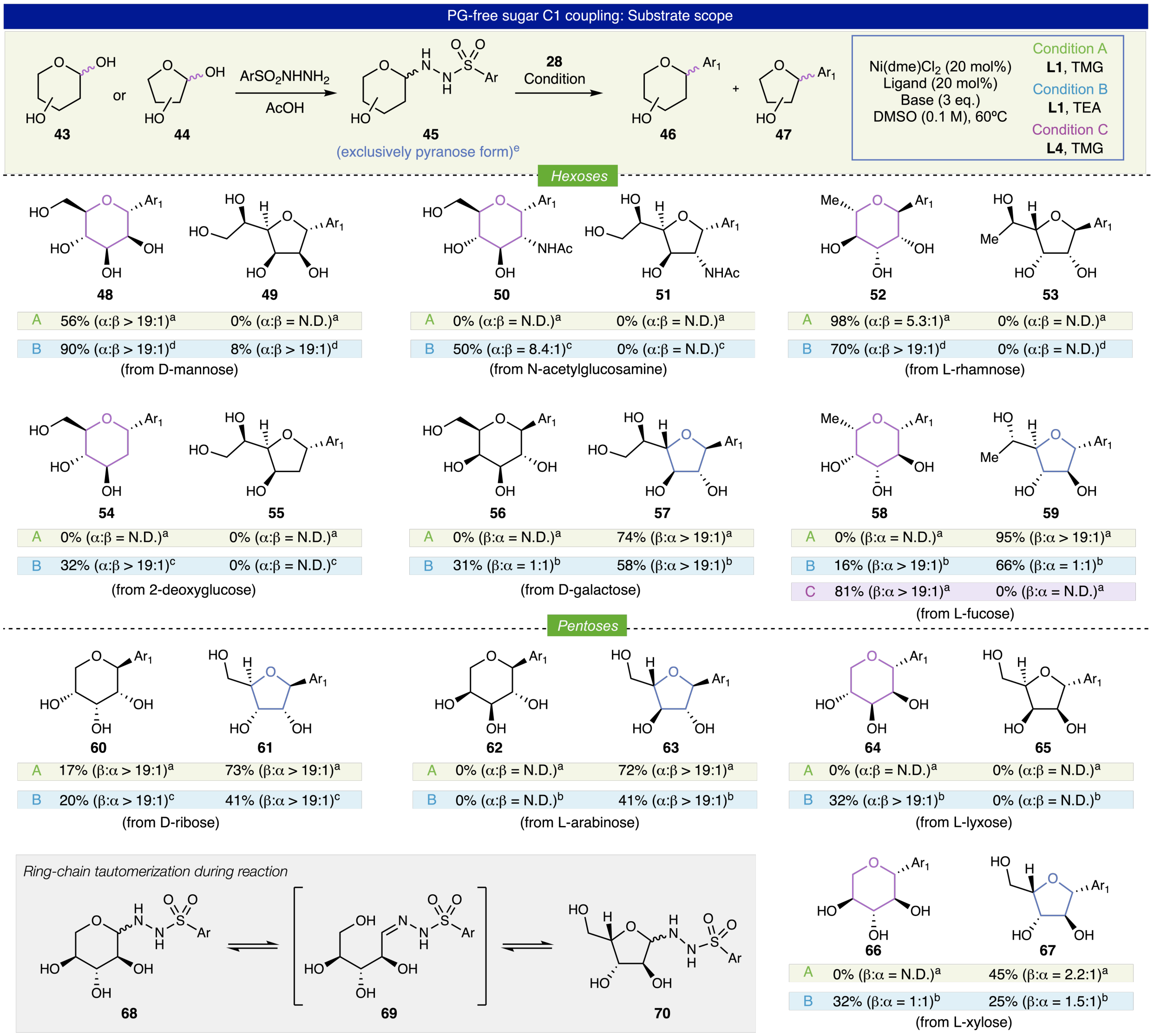

Figure 3. [a]Toluenesulfonyl hydrazide. [b]3,5-difluorobenzenesulfonyl hydrazide. [c]3,4,5-trifluorobenzenesulfonyl hydrazide. [d]2-(trifluoromethyl)benzenesulfonyl hydrazide. [e]Structures were assigned using 2D NMR (see SI for details). The structures of ribose, xylose, and galactose hydrazides were further confirmed by X-ray crystallography. Diastereomeric ratio was determined from the crude reaction mixture by ^{1}H NMR or LCMS. Ar_1 = 1-chloro-2-[(4-ethoxyphenyl)methyl]-benzene.

"To further investigate the substrate scope, a series of sugar hydrazides were prepared from native hexoses and pentoses using a common protocol ($TsNHNH_2$/AcOH), as shown in Figure 3. Notably, all of the resulting sugar hydrazides adopt the pyranose form, as confirmed by X-ray crystallography and 2D NMR analyses, despite the conventional assumption that pentoses preferentially exist as furanoses whereas hexoses predominantly adopt pyranose structures. More

intriguingly, these sugar hydrazides exhibit markedly divergent reactivity under different reaction conditions: hexose-derived hydrazides can furnish either pyranose or furanose products, and a similar variability is observed for pentose-derived substrates, presumably arising from the unique ring–chain tautomerization behavior of hydrazides (*cf.* **68** to **70**). Following systematic optimization, it was found that both the base and ligand play decisive roles in controlling the yield and selectivity of the major product. For hexose-derived substrates, most preferentially afford pyranose products. For example, mannose-, N-acetylglucosamine-, rhamnose-, and 2-deoxyglucose-derived hydrazides deliver products **48**, **50**, **52**, and **54**, respectively, with high diastereoselectivity. In contrast, the galactose-derived hydrazide predominantly yields the furanose product **57**. Notably, the reaction of fucose-derived hydrazide exhibits pronounced ligand dependence on the reactive tautomer: with **L1** (Condition A or B), the furanose product **59** is formed predominantly, whereas employing **L4** (Condition C) switches the selectivity to the pyranose isomer **58** as the major product. For pentose-derived substrates, ribose-, arabinose-, and xylose-derived hydrazides mainly furnish the corresponding furanose products **61**, **63**, and **67**, whereas the lyxose-derived hydrazide instead affords the pyranose product **64**. Overall, this transformation exhibits broad substrate generality and well-defined chemo- and stereoselectivity, enabling efficient access to either furanose or pyranose C1-glycosylation products in a substrate-dependent manner, with ligand- and base-dependent modulation of selectivity demonstrated in representative cases. These features provide a versatile platform for the rapid construction of structurally diverse C1-glycosides, offering potential utility in synthesis, chemical biology, and medicinal chemistry. To our best knowledge, the ligand-controlled divergency of the ring size in glycosyl RCC described above has been unprecedented." This paragraph has been updated to the main manuscript.

Different sugar hydrazides starting materials are summarized in the following table, which is now included in the SI.

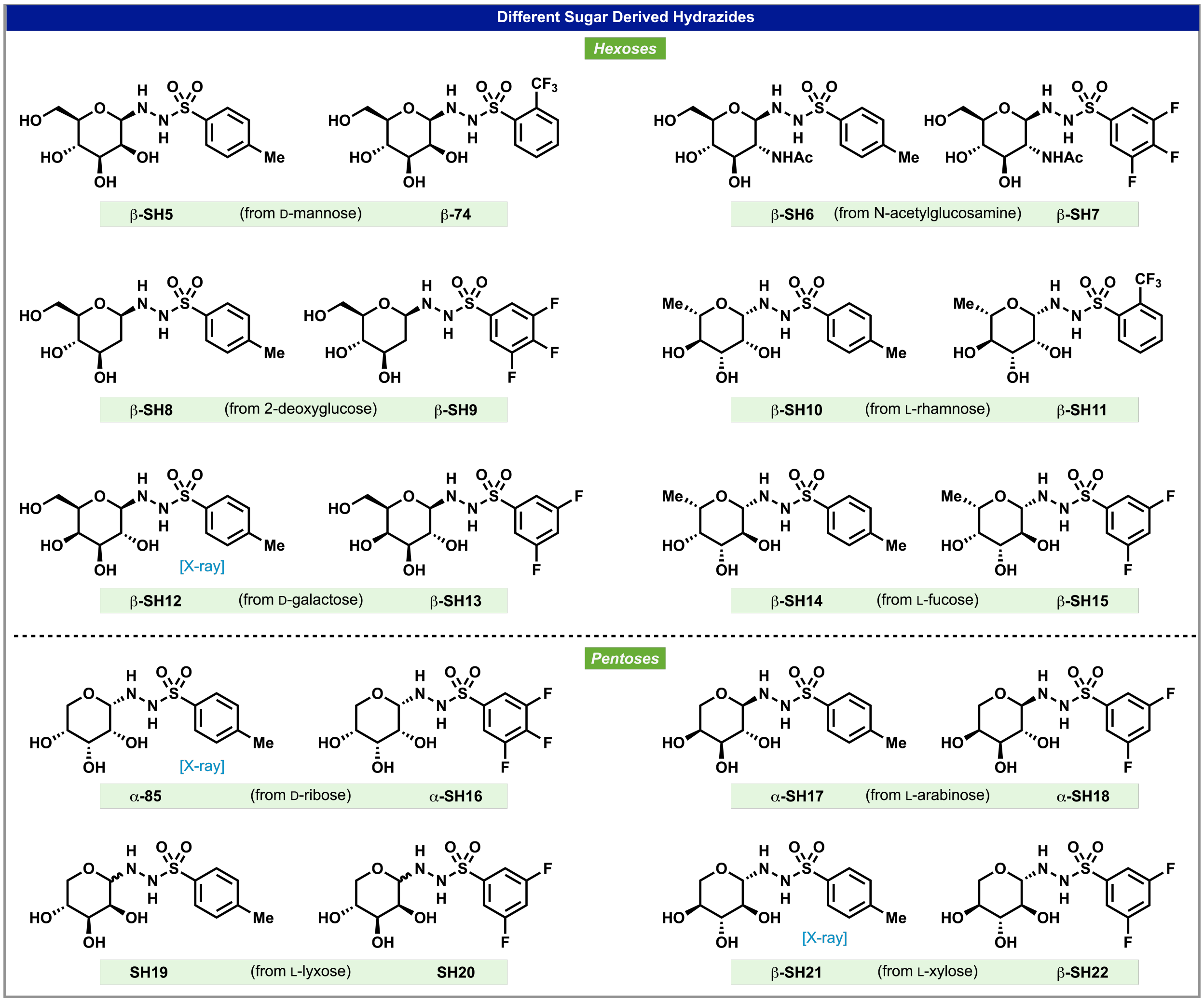

For all the sugar hydrazides, we characterized them with ^{1}H NMR, ^{13}C NMR, HSQC, COSY, HMBC and NOESY. For xylose, galactose, and ribose derived hydrazides (**β-SH21**, **β-SH12**, **β-85**), we further confirmed their structures with X-ray crystallography (see SI—11 X-ray Crystallography). For all the products, we characterized them with ^{1}H NMR, ^{13}C NMR, HSQC, COSY, HMBC and NOESY (see SI—9 RCC-Arylation of Different Sugars).

[Critique 3]

For more challenging substrate classes (e.g. 2-deoxypyranosides and furanosides), the authors attempt to apply their "stereoretentive coupling conditions" to control glycosylation stereoselectivity. Success is partial, and the modest catalyst-controlled selectivity (e.g. from 1:2 to 3:1) does not rise to the level of synthetic need. Here, the requirement for O-protected sugars

("to simplify the preparation and purification of either anomeric series") runs contrary to the overall synthetic premise of direct, selective, and general glycosylation.

[Response]

We thank the reviewer for this insightful comment and appreciate the opportunity to clarify this point. We agree that stereocontrol using the stereoretentive strategy under the present conditions is only partially successful. However, the inclusion of 2-deoxypyranosides and furanosides in this study is intended to demonstrate the feasibility of extending our stereoretentive radical cross-coupling strategy to these particularly challenging substrate classes. Although the observed stereoselectivities are modest, the results nevertheless highlight the potential of stereoretentive control in such systems. Notably, diastereomeric ratios of 2:1 to 3:1 in favor of the β-anomer are still synthetically useful, especially given the difficulty of accessing these compounds via other radical cross-coupling approaches. The observed selectivity is largely governed by the anomeric effect, which generally favors formation of the α-anomer. The use of O-protected sugars in these specific cases also reflects practical considerations for substrate preparation and is common across the C-glycosylation literature. We also have revised the text to better reflect the current scope and limitations of the method.

[Critique 4]

Collectively then, the selectivity profile is unremarkable: some substrates are (as they are known to be) innately selective, others are not, and prospects for external stereocontrol are not yet realized. Put another way, the method leads to outcomes similar to those obtained using other radical arylation methods, but without the same level of tunable control (for example, 10.1021/acs.oprd.5c00302; 10.1021/acscatal.4c02322). I appreciate that these two arylation methods use protected rather than unprotected donors, but many compounds of type 12 (c.f. Fig 1) are commercial, easy to handle/purify, and step-competitive with the authors' approach.

[Response]

To clarify, our study does include cases of external stereocontrol, conceptually analogous to the precedents cited by the reviewer. However, our platform also introduces an additional and distinct mode of stereochemical control that we describe as "pre-radical" control. This mode is

neither conventional external control nor ordinary substrate control. Rather, the stereochemical information is encoded before radical generation and can override the typical substrate bias commonly observed in glycosyl radical chemistry.

We believe this distinction is important. The presence of such a pre-radical stereochemical element demonstrates that our work is not simply another example within the existing framework of externally controlled glycosyl radical reactions. Instead, it points to an emerging class of stereocontrol in carbohydrate radical chemistry, one that is mechanistically and conceptually distinct from the precedents cited by the reviewer.

It is also worth noting that the glycosyl bromides employed in these references are not yet the best solution from a practical standpoint. Their preparation generally requires protecting-group manipulations, and their stability can vary substantially depending on the carbohydrate substrate. In our view, continued reliance on this class of donors limits the broader development of the field. A practical, readily accessible, and stereochemically programmable donor platform described in this work would move glycosyl radical chemistry beyond case-specific demonstrations and toward more general synthetic utility.

[Critique 5]

The arylation method also works at other positions (C2-C6) of the sugar molecule, although here the need for differential protection strategies to install the hydrazide at the requisite position cannot be avoided here, and substrate controlled diastereoselectivities are generally modest. The authors offer no insights into reaction mechanism, which presumably does not diverge substantially from the pathways outlined in the Science 2025 paper. This is fine, though it underscores that the present work is an application of an established platform. It might be interesting to follow up on the striking role of base in governing stereoselectivity, however.

[Response]

We thank the reviewer for these constructive comments. Regarding the protection strategies required for C2–C6 functionalization, we acknowledge this as a current limitation. However, we would also like to highlight newly added data: selective oxidation at the C3-position of

unprotected methyl glucoside **141** enables unprotected sugar coupling, which directly addresses the concern about protecting group requirements (see manuscript, Figure 6B; for experimental procedures, see SI—10 Selective Oxidation Followed by C3 Coupling). This further underscores the potential of this platform for direct sugar modification without the need for protecting group manipulation.

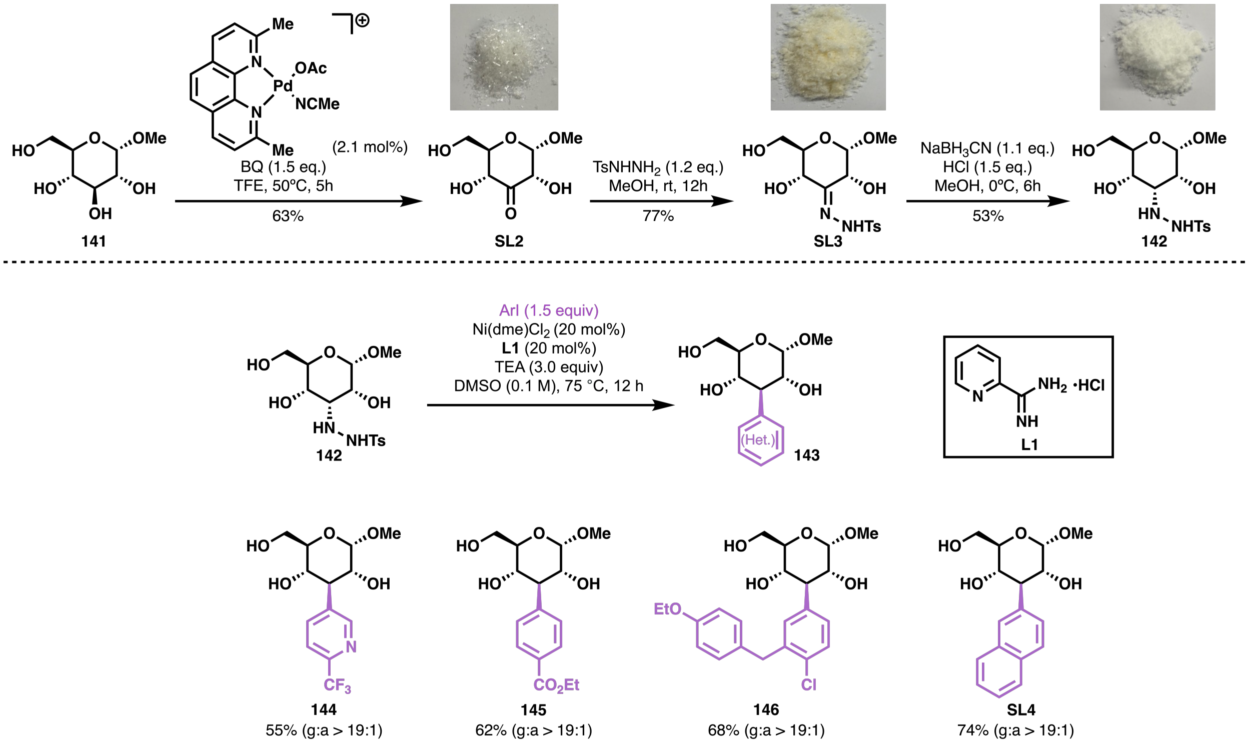

Accordingly, we have modified Figure 6 and added Figure 6B to include this new data as shown below.

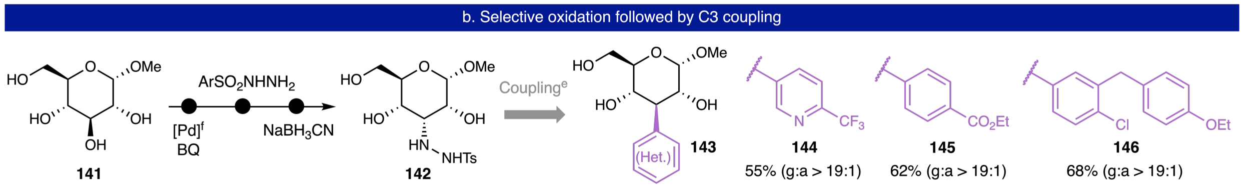

Figure 6B. [e]Condition: 20 mol% Ni(dme)Cl_2, 20 mol% **L1**, 3.0 equiv. TEA, 0.1 M DMSO, 75 ºC. [f][(neocuproine)Pd(μ-OAc)]$_2$[OTf]$_2$

"Of particular significance, glycohydrazide-based RCC proved amenable to unprotected glucoside **142**. Methyl 3-keto-α-D-glucopyranoside was accessed through selective oxidation of α-D-glucopyranoside **141**[47,48], followed by reductive amination to furnish the corresponding α-

hydrazide **142**. Subsequent coupling delivered adducts **144**, **145**, and **146** in good yield with high diastereoselectivity (β:α>19:1, Figure 6B). These results further indicate that, in addition to protecting-group strategies that facilitates handling and purification, unprotected glycohydrazides can also exhibit suitable reactivity and selectivity in RCC." This paragraph has been updated to the main manuscript.

References:

47. Jäger, M., Hartmann, M., de Vries, J. G. & Minnaard, A. J. Catalytic regioselective oxidation of glycosides. *Angew. Chem. Int. Ed.* **52**, 7809–7812 (2013).

48. Ho, W. C., Chung, K., Ingram, A. J. & Waymouth, R. M. Pd-catalyzed aerobic oxidation reactions: strategies to increase catalyst lifetimes. *J. Am. Chem. Soc.* **140**, 748–757 (2018).

[Critique 6]

Again, the work has many positive elements, and I expect will become a go-to method for access to selected substrate classes. However, the method does not fundamentally re-shape the landscape of stereoselective glycosylation: biased substrates remain generally accessible, and challenging substrates remain challenging. Here, the method is "not better, just different." My suggestion is that the authors scale back what they are trying to claim (general access), and focus on the strengths of the method (beta glucosides and ribosides). My view is that other aspects of the story (e.g. catalyst control in challenging substrates, C2-C6 arylation) fall short, and deserve additional attention and development.

[Response]

We thank the reviewer for the constructive feedback and agree that the work contains many positive elements. Our primary focus in this study is the development of a direct C1 glycosylation strategy from native sugars. The C2–C6 modifications are included as an extension of the current strategy to demonstrate its broader applicability, rather than as a central focus of the work. To the best of our knowledge, a systematic exploration of radical cross-coupling across all hydroxyl-bearing positions of riboses and glucoses has not been previously reported. Access to this chemical space could open new opportunities for the diversification of carbohydrate scaffolds. Stereospecific modification at the C2–C6 positions remains particularly challenging due to the complex steric environment surrounding the reaction centers. A comprehensive

investigation of these transformations would necessitate a dedicated and systematic study, which we believe is beyond the scope of the present work. The C2–C6 modifications are generally accessible under the present conditions, although diastereocontrol remains substrate-dependent and, in some cases, approaches nearly a 1:1 ratio. Importantly, both diastereomers can be readily separated, and each is valuable from the perspective of diversity-oriented synthesis as well as in medicinal chemistry. This point has also been recognized by Reviewer 1. Furthermore, we have included additional data on the C3 position of unprotected methyl glucoside, which provides very high diastereoselectivity (for details, see Referee 2, Critique 5). This feature may be particularly valuable for practical applications.

[Critique 7]

In the introduction, the authors write that "their [SGLT2 inhibitors] clinical success illustrates how a robust C-C linkage can translate into favorable metabolic stability and durable biological activity…", which seems to misattribute the myriad and complex determinants of clinical success to metabolic stability alone. A more nuanced rephrase here would improve scholarship without undermining the case for the importance of C-aryl glycosides.

[Response]

We appreciate this reviewer's comment and fully agree with it. Accordingly, we have revised the sentence to avoid overattributing the clinical success of SGLT2 inhibitors to metabolic stability alone, while preserving the intended point regarding the synthetic and medicinal significance of C-aryl glycosides. The revised sentence now reads:

"While the clinical success of SGLT2 inhibitors reflects many pharmacological, pharmacokinetic, and clinical factors, these compounds also exemplify how a robust $C(sp^3)$–$C(sp^2)$ linkage can contribute to favorable metabolic stability and durable biological activity, underscoring the need for efficient and scalable synthetic strategies to access diverse C-aryl glycoside architectures."

[Critique 8]

Cross-coupling strategies are conventionally redox neutral. I appreciate that the authors have

extensively developed radical cross-couplings initiated by redox steps, but the branding around a "redox neutral cross-coupling" platform makes less sense from a wider lens.

[Response]

We appreciate the reviewer's comment and understand the concern. To clarify, our use of the term "redox-neutral" is not intended to distinguish this work from conventional two-electron cross-coupling reactions, many of which are indeed redox-neutral overall. Rather, the intended contrast is with radical cross-coupling strategies, which commonly require external redox inputs, such as stoichiometric oxidants or reductants, photoredox catalysts, electrochemical setups, or other redox-initiating conditions. In this context, we use "redox-neutral radical cross-coupling" to emphasize that radical generation and cross-coupling occur without an external redox reagent or device. To reflect this point, we have used the word "redox-neutral" exclusively in the context of radical cross-coupling in the main text.

[Critique 9]

The authors could be a bit more comprehensive in discussing precedent for selective manipulations of unprotected sugars in the conclusion (e.g. "This work represents another step in dismantling the long-held belief that meaningful synthetic elaboration of carbohydrates demands extensive protecting groups and redox manipulations"). The potential advantages of working with unprotected sugar substrates is not a new insight, and an increasingly vibrant area of research (For example: 10.1038/s41586-022-04958-w, 10.1038/s41586-024-07695-4, 10.1038/s41586-020-1937-1, 10.1021/jacs.5b13384, along with nearly all chemoenzymatic approaches).

[Response]

We appreciate the reviewer's suggestion and revised the conclusion paragraph accordingly to reflect important advances in this area.

"The past decade has witnessed a vibrant and growing effort to enable synthetic elaboration of carbohydrates without the protecting group sequences[17,20,50-53] and redox manipulations that long dominated the field — advances spanning photocatalytic site-selective epimerization of

unprotected sugars[54], aqueous glycosylation of unprotected acceptors[55], catalyst-controlled glycosylation of minimally protected substrates[56,57] and the extensive progress achieved through chemoenzymatic strategies. The present work joins and extends this trajectory by addressing a distinct and hitherto unmet challenge: the absence of a practical, versatile radical glycosyl donor suitable for modular cross-coupling. By converting unprotected sugars in a single step into stable sulfonyl hydrazides that serve as such donors, a direct, efficient, highly chemoselective, and scalable route to C1-glycosides has been unlocked. This chemistry provides rapid access to current FDA-approved SGLT2 inhibitors, challenging natural products, and a host of new glyco-architectures — including modifications at non-anomeric positions and stereoisomers previously considered out of reach. In doing so, it shifts carbohydrates from being among the most cumbersome classes of molecules to synthesize into versatile and readily diversifiable building blocks. We anticipate that this platform, amenable to derivatization at multiple sites on sugars and to stereoretentive coupling, will catalyze a significant expansion in the exploration of carbohydrate chemical space and help unleash the full therapeutic potential of glycomimetics in the coming years."

References

17. Zhang, C. *et al.* Direct synthesis of unprotected aryl C-glycosides by photoredox Ni-catalysed cross-coupling. *Nat. Synth.* **2**, 251–260 (2023).

20. Jiang, Y. *et al.* Direct radical functionalization of native sugars. *Nature* **631**, 319–327 (2024).

50. Talukdar, R. & Fairbanks, A. J. Photoredox mediated one-step syntheses of aryl C-glycosides from unprotected glycosyl sulfinates. *Chem. Commun.* **61**, 15243–15245 (2025).

51. Xie, H., Wang, S. & Shu, X.-Z. C–OH bond activation for stereoselective radical C-glycosylation of native saccharides. *J. Am. Chem. Soc.* **146**, 32269–32275 (2024).

52. Bunt, D. V. *et al.* The synthesis of aryl-β-C-glycosides from native saccharides. *Chem. Eur. J.* **31**, e202501216 (2025).

53. Marinus, N. *et al.* Site-selective palladium-catalyzed oxidation of unprotected aminoglycosides and sugar phosphates. *Chem. Eur. J.* **30**, e202400017 (2024).

54. Wang, Y., Carder, H. M. & Wendlandt, A. E. Synthesis of rare sugar isomers through site-selective epimerization. *Nature* **578**, 403–408 (2020).

55. Pelletier, G., Zwicker, A., Allen, C. L., Schepartz, A. & Miller, S. J. Aqueous glycosylation of unprotected sucrose employing glycosyl fluorides in the presence of calcium ion and trimethylamine. *J. Am. Chem. Soc.* **138**, 3175–3182 (2016).

56. Li, Q., Levi, S. M., Wagen, C. C., Wendlandt, A. E. & Jacobsen, E. N. Site-selective, stereocontrolled glycosylation of minimally protected sugars. *Nature* **608**, 74–79 (2022).

57. Dang, Q.-D. *et al.* Catalytic glycosylation for minimally protected donors and acceptors. *Nature* **632**, 313–319 (2024).

Referee 3

[Critique 1]

For compounds 79-96 alpha:beta is not the correct terminology. More appropriate would be gluco:manno (79-84), allo:gluco (85-90) and galacto:gluco (91-96). Similarly, compounds 103 and 104 should be arabino:ribo and 106/106 should be xylo:ribo please fix in manuscript and SI.

[Response]

We sincerely appreciate the reviewer's comments and feedback. We have revised the manuscript (Figure 6) and SI accordingly.

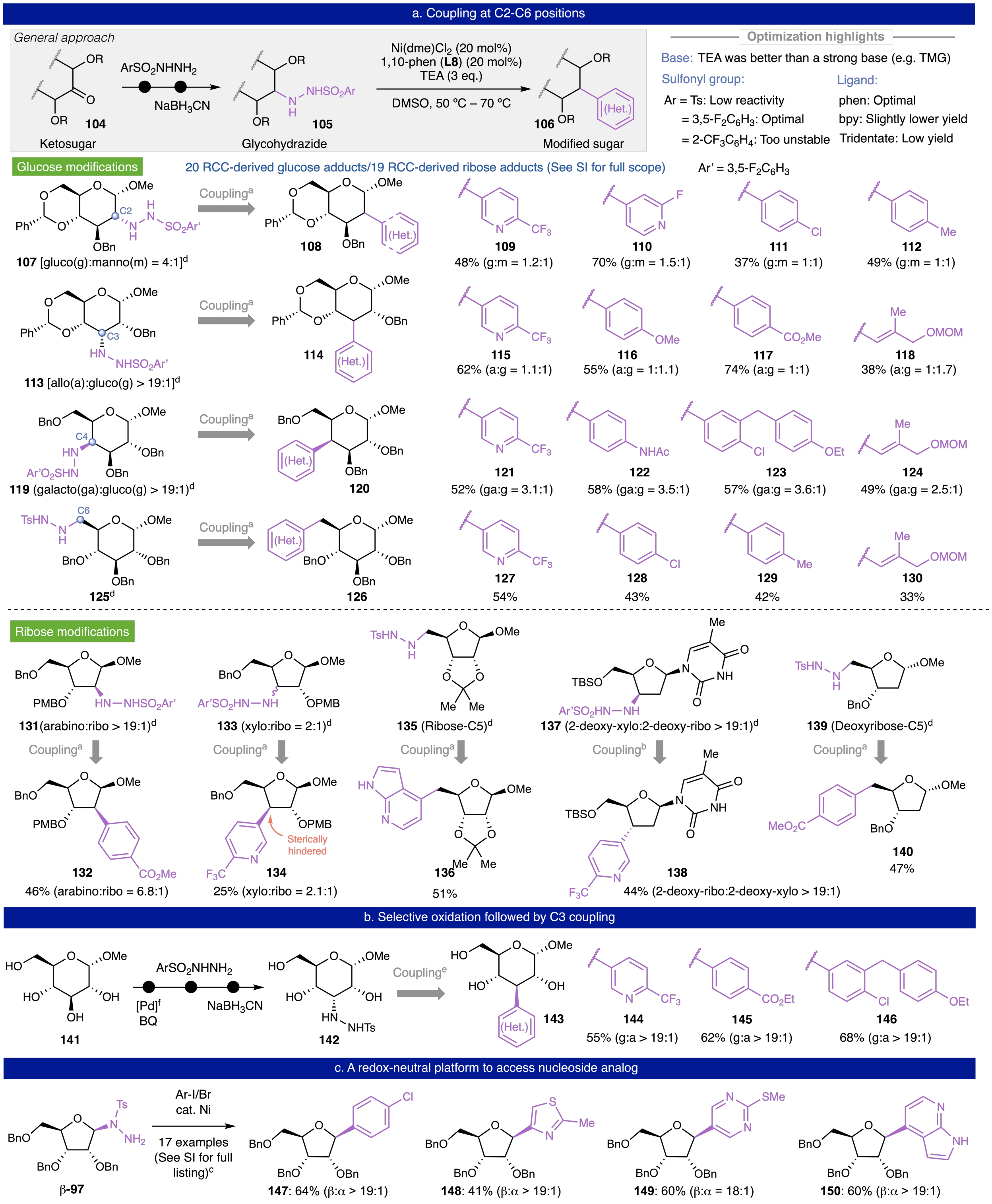

Figure 6. Coupling at C2-C6 positions. [a]Condition: 20 mol% Ni(dme)Cl_2, 20 mol% **L8**, 3.0 equiv. TEA, 0.1 M DMSO, 50 – 70 ºC. [b]Condition: 20 mol% Ni(dme)Cl_2, 20 mol% **L6**, 3.0 equiv. PMP, 0.1 M DMF, 75 ºC. [c]Condition: 20 mol% Ni(dme)Cl_2, 20 mol% **L4**, 3.0 equiv. TEA, 0.1 M DMF, 100 ºC. [d]Step counts for synthesizing the hydrazides: glucose C2 hydrazide

107 (3 steps from commercially available **SF5**); glucose C3 hydrazide **113** (3 steps from **SF5**); glucose C4 hydrazide **119** (4 steps from **SF5**); glucose C6 hydrazide **125** (4 steps from commercially available **SF9**); ribose C2 hydrazide **131** (5 steps from commercially available **SF12**); ribose C3 hydrazide **133** (5 steps from **SF12**); ribose C5 hydrazide **135** (2 steps from commercially available **SF15**); 2-deoxyribose C3 hydrazide **137** (5 steps from commercially available **SF16**); 2-deoxyribose C5 hydrazide **139** (6 steps from **SF16**). See SI for detailed procedures. [e]Condition: 20 mol% Ni(dme)Cl_2, 20 mol% **L1**, 3.0 equiv. TEA, 0.1 M DMSO, 75 ºC. [f][(neocuproine)Pd(μ-OAc)]$_2$[OTf]$_2$. Diastereomeric ratio was determined from the crude reaction mixture by ^{1}H NMR or LCMS.

[Critique 2]

Can salmochelin be obtained without the need for benzyl ethers/esters?

[Response]

We thank the reviewer for this excellent question. The synthesis of salmochelin without benzyl protecting groups would indeed be highly desirable and more aligned with the goal of direct, protecting-group-free glycosylation. This was also the first reaction we tried, where we attempted coupling with an unprotected phenol iodide; however, no product was observed under the current reaction conditions. We suspect that free phenolic hydroxyl groups may interfere with the reaction, possibly by diminishing the efficiency of oxidative addition or through unproductive coordination. At present, the use of benzyl ethers/esters remains necessary.

[Critique 3] *Figure 4 – I would be extremely hesitant to call a reaction where a single anomer of substrate leads to a 2:1 or 3:1 mixture of products stereoretentive. Furthermore, in the SI compound 68 is reported as a 2:# mixture or isomers while in the manuscript it is reported as a 2:1 mixture. Based off of the NMR I would say the selectivity is likely 2.3:1. Please check for consistency throughout the manuscript and SI.*

[Response]

We sincerely appreciate the reviewer's comments and feedback. We agree that the diastereoselectivity (dr) is not optimal for 2-deoxyglucose substrates. In this case, although the

stereoretentive pathway does not effectively compete with the inherent anomeric effect and steric factors, the reactions still afford the stereoretentive product as the major isomer in synthetically useful yields, thereby demonstrating the presence of a stereoretentive effect. Notably, the α-anomeric products are particularly difficult to access using other radical cross-coupling methods. The dr values reported in the manuscript are accurate and were determined from the crude ^{1}H NMR spectrum of each individual reaction. The NMR spectrum presented in the Supporting Information was obtained from a combined mixture of products from two reactions, following multiple rounds of separation and purification aimed at separating the two diastereomers.

[Critique 4]

The sentence "This work represents another step17,20,48,49 in dismantling the long-held belief that meaningful synthetic elaboration of carbohydrates demands extensive protecting groups and redox manipulations." Is misleading and should be amended. Yes there exists a plethora of radical-based methods for anomeric C-C coupling, however, even in the case of the current manuscript, elaboration at other positions requires extensive protecting group manipulation. Unfortunately, this regiochemistry remains one of the biggest challenges in carbohydrate chemistry and is still one that has yet to be fully addressed.

[Response]

The referenced sentence was adjusted in order to address the comment from Reviewer 2 (for details, please see Referee 2, Critique 9). In addition, to address the reviewer's concern on regioselectivity more directly, we have now included new experimental results demonstrating selective C3 oxidation of an unprotected sugar followed by successful protecting-group-free radical cross-coupling. While this result does not solve the broader regiochemical problem in full, it provides a concrete proof of concept that selective oxidation/cross-coupling sequences at non-anomeric positions are achievable. Given the rich literature on site-selective oxidation of unprotected carbohydrates, we believe this experiment supports the feasibility of combining established selective functionalization strategies with our radical cross-coupling platform (for details, please see Referee 2, Critique 5).

[Critique 5]

Compound SF19 – what does it mean of a peak to integrate at 0H? The hardcopy of the NMR shows 13 protons, there should be 12. What is the impurity?
Compound SF20 – there are too many protons (13 should be 12) in the 1H NMR spectrum. Furthermore, the methyl singlet is not reported in the list of chemical shifts.
Compound SF27 why is a peak reported at 8.52 ppm? Is it because it is expected? There is nothing there. I appreciate that protons on nitrogen can exchange with residual water in the CD3CN, however if nothing is there do not report it.
Please check all NMR data (both spectra and data reporting) to make sure these types of errors do not appear elsewhere.

[Response]

We sincerely appreciate the reviewer's comments and feedback. For compounds **SF19** and **SF20**, there are some identified impurities; therefore, we repurified these samples, and the updated spectra have been included in the Supporting Information.

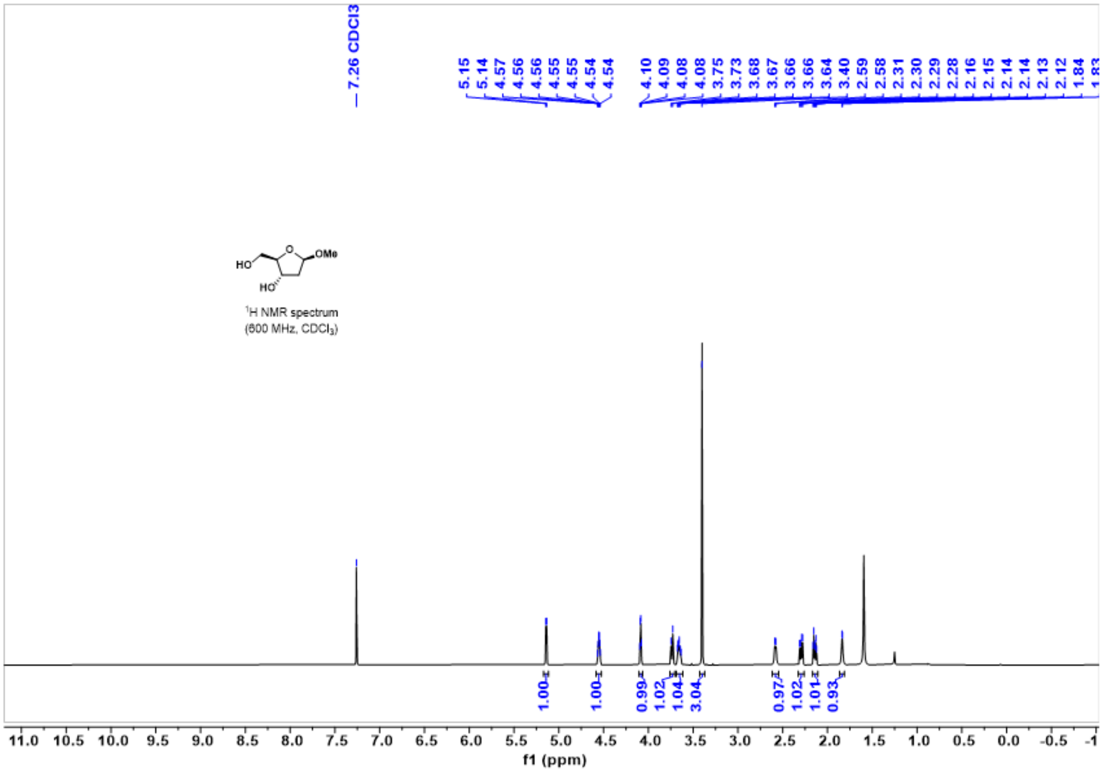

^{1}H NMR of compound **SF19**

138

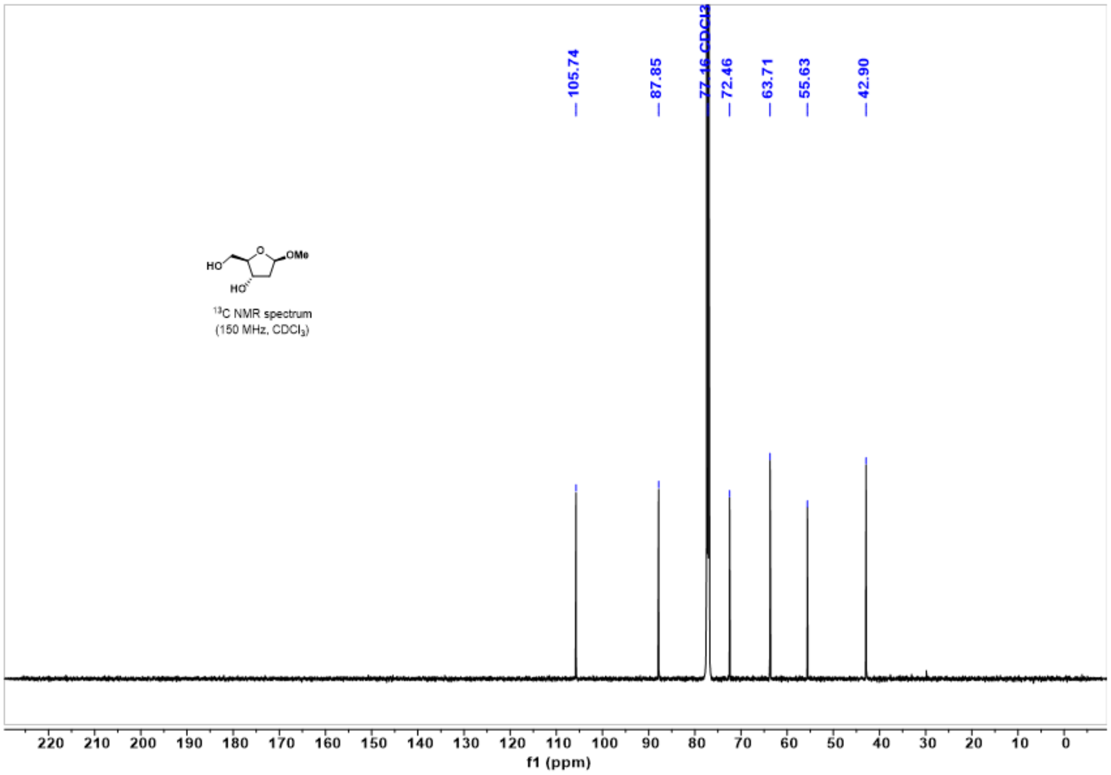

^{13}C NMR of compound **SF19**

139

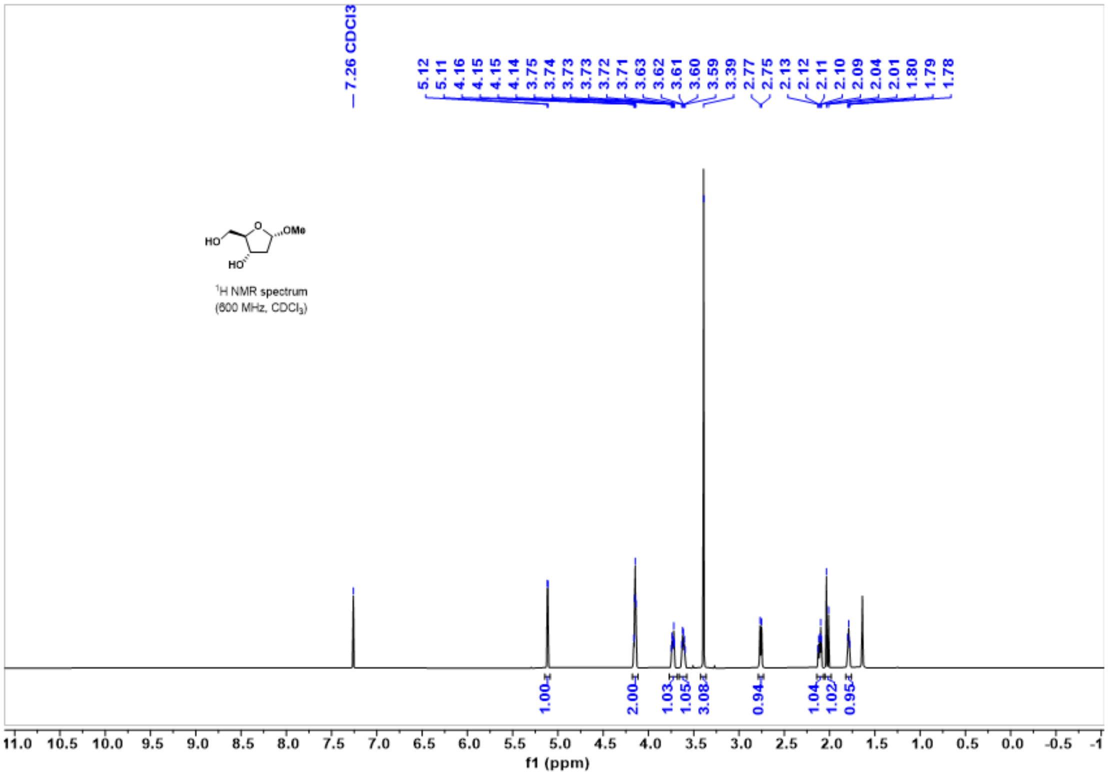

^{1}H NMR of compound **SF20**

140

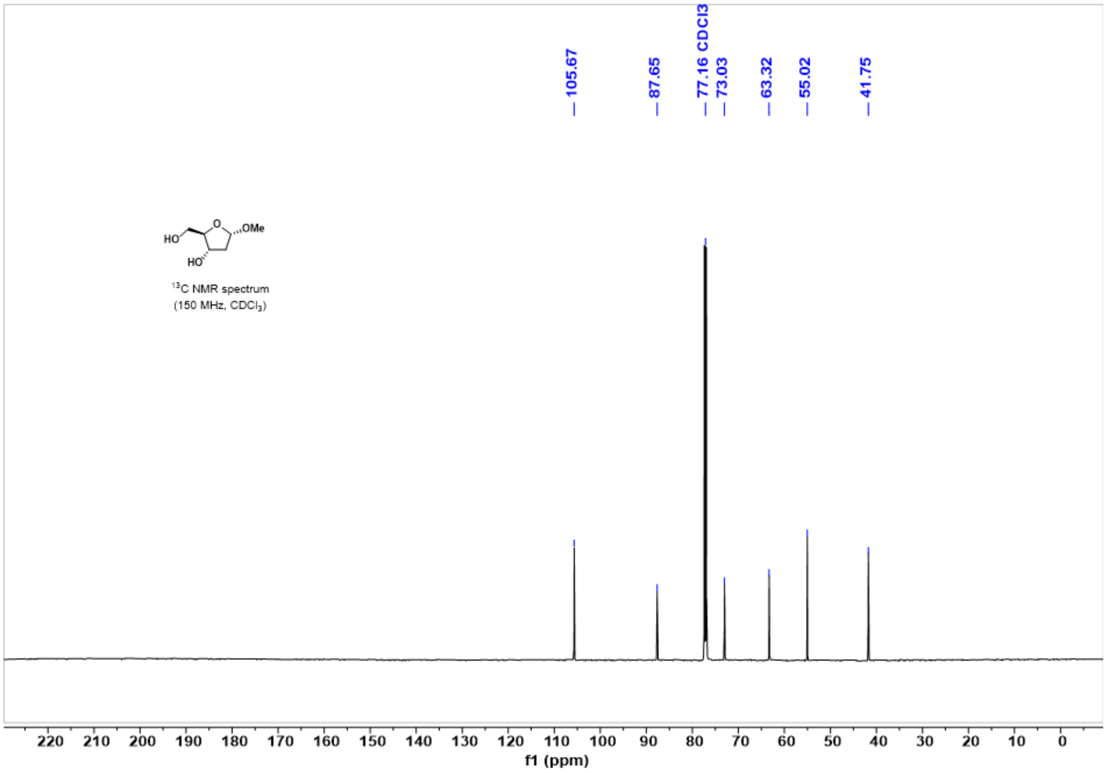

^{13}C NMR of compound **SF20**

141

For compound SF27, a broad peak is indeed observed, which is most likely attributable to the nitrogen-bound protons and can be clearly seen upon zooming in on the relevant region of the ^{1}H NMR spectrum. In addition, we have corrected an input error in which a peak was incorrectly integrated as 0H. We have also carefully re-examined all NMR data and corrected any related errors throughout the SI.

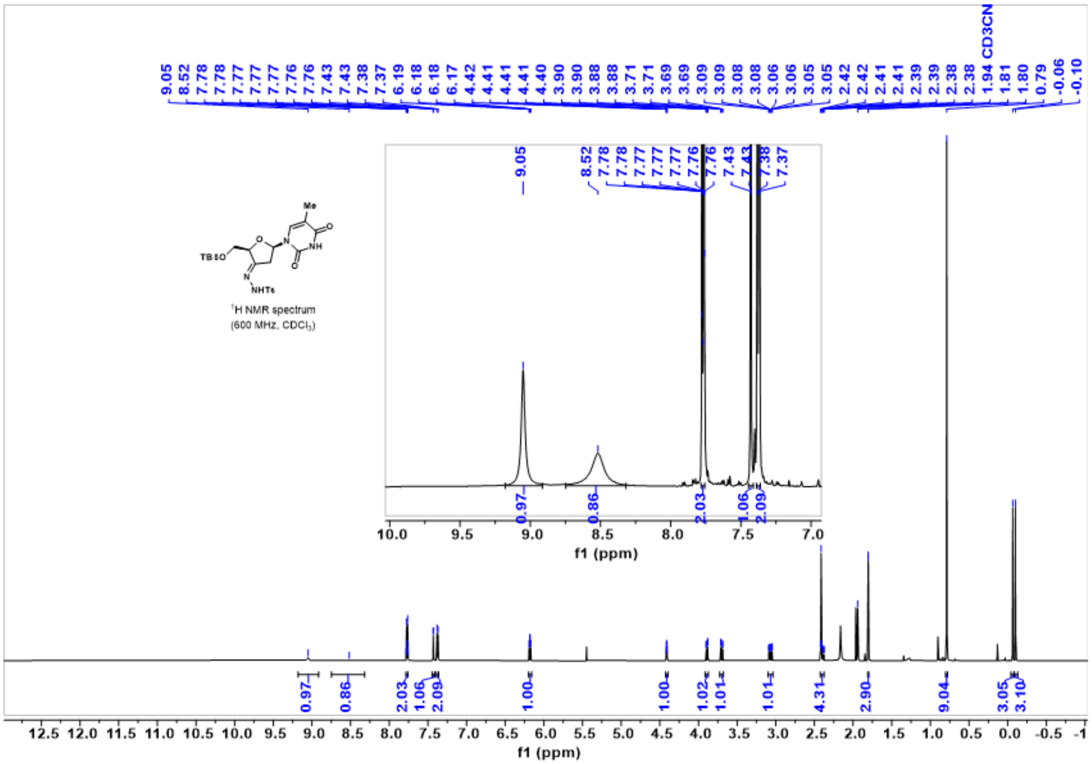

^{1}H NMR of compound **SF27**

146

We sincerely appreciate the reviewer's comments and feedback. We have also taken this opportunity to scrutinize the SI data and fix typos and small errors. During the processing and generation of the data, some copy-paste errors alongside some oversights occurred. These and several edits for the sake of consistency have been corrected by carefully checking all NMR data (both spectra and data reporting). The results are summarized here (Old compound numbers from the initial submission were used occasionally for better overview while surveying the data).

Unprotected sugar hydrazides

- Compound 27:
  - Corrected proton count in text: ^{1}H NMR: (multiplet at 3.37-3.32 should be one proton instead of 2)
- Compound SH1

- Correction ^{1}H NMR: Singlet at 2.30 ppm was not picked and reported in text

- Compound SH2
  - Corrected proton count in text: ^{1}H NMR: (multiplet at 3.24-3.06 should be 6 protons instead of 5)
- Compounds SH3
  - Corrected molecular formula (HRMS)
- Compounds SH4
  - Correction ^{1}H NMR: (multiplet at 3.37-3.32 should be 11 protons instead of 12)
  - Correction ^{13}C NMR (peak at 60.23 was removed; was identified as artifact)
- Compound 46
  - Text: ^{13}C Added second digits for peaks 133.14, 133.11
  - Added identification of J-coupling in text or ^{13}C
- Compound 57
  - Text and spectrum (number)

Coupling partners

- Compound SG7
  - Updated proton count in text (^{1}H) and molecular formula (to fit M+H)
- Compound SG8
  - Updated molecular formula (to fit M+H)
- Compound SG9
  - Updated proton count in text (^{1}H) and molecular formula (to fit M-H)
- Compound SG10
  - Updated molecular formula (to fit M+H)
- Compound SG11
  - Updated molecular formula (to fit M+H)
- Compound SG19 = SG25
  - Updated molecular formula (to fit M+H)

Gliflozins

- Ipragliflocin
  - Update ^{1}H spectrum (last peak is 1 instead of 0.64 and 0.52)
- Enavogliflocin
  - Update ^{1}H spectrum (third peak from last should be 3 instead of two small ones)
- Keto-Canagliflocin
  - Update ^{1}H text added peak 125.8 (also updated spectrum)
- Compound 39
  - Updated molecular formula (to fit M+H)
  - Corrected NMR solvent
- Compound 40
  - Updated molecular formula (to fit M+H)

  - Corrected NMR solvent
- Compound 41
  - Corrected NMR solvent
- Compound 42
  - Corrected NMR solvent
  - For ^{13}C added peak 129.4 (text), updated spectrum

Stereoretention hydrazides and synthesis

- Compound SE2
  - Updated molecular formula (to fit M+H)
  - Text: ^{13}C Added second digits for some peaks
- Compound SE6
  - Updated molecular formula (to fit M+H)
  - Alpha and beta for text in ^{13}C added second digits for some peaks
  - Alpha: changed proton count in bn region, also updated spectrum
- Compound SE7
  - Updated molecular formula (to fit M+H)
- Compound SE9
  - Updated molecular formula (to fit M+H)
  - Added alpha and beta anomer description in ^{1}H text
- Compound SE10
  - Updated molecular formula (to fit M+H)
- Compound SE12
  - Updated molecular formula (to fit M+H)
- Compound SE13
  - Updated molecular formula (to fit M+H)
  - Alpha and beta for text in ^{13}C added second digits for some peaks
- Compound 64
  - Alpha and beta for text in ^{13}C added second digits for some peaks
- Compound 67
  - Alpha and beta for text in ^{13}C added second digits for some peaks
- Compound 69
  - Alpha and beta for text in ^{13}C added second digits for some peaks
  - Updated spectrum of beta 69: removed third peak from left
- Compound 71
  - Alpha and beta for text in ^{13}C added second digits for some peaks

Stereoretentive coupling products

- Compound 66
  - Alpha and beta for text in ^{13}C added second digits for some peaks
  - Updated molecular formula (to fit M+H)

  - Added mulitplets in carbon spectra of beta 66
- Compound 70
  - Alpha and beta for text in ^{13}C added second digits for some peaks
  - Updated molecular formula (to fit M+H)
- Compound 72
  - Spectra are switched. Text alpha and text beta are correct. Plotted spectra are switched
  - Alpha and beta for text in ^{13}C added second digits for some peaks
  - Updated molecular formula (to fit M+H)

Characterization of C2-C6-Hydrazide
- Compound SF6
  - Updated molecular formula (to fit M+H)
- Compound SF7
  - Updated molecular formula (to fit M+H)
- Compound SF22
  - Updated molecular formula (to fit M+H)
  - For ^{13}C added second digits for some peaks
- Compound SF8
  - Updated 1H spectrum. Removed peak with integral 2 at ~2ppm.
  - For ^{13}C added second digits for some peaks
- Compound SF11
  - For ^{13}C added second digits for some peaks
- Compound SF23
  - Updated molecular formula (to fit M+H)
  - For ^{13}C added second digits for some peaks
- Compound SF24
  - Updated molecular formula (to fit M+H)
  - For ^{13}C added second digits for some peaks
- Compound SF13
  - Added proton peak (dublet) at 2.6 ppm (text and updated spectrum)
- Compound SF14
  - Added proton peak (sg) at 3.32 ppm (updated spectrum)
  -
- Compound SF19
- Compound SF20
- Compound SF21
  - For ^{13}C added second digits for some peaks
- Compound SF25
  - For ^{13}C added second digits for some peaks

- Compound SF27
    - Updated Spectrum with insert
- Compound 79
    - For ^{13}C added second digits for some peaks
- Compound 85
- Compound 91
    - For ^{13}C added second digits for some peaks
- Compound 97
    - For ^{13}C added second digits for some peaks
- Compound 103
    - For ^{13}C added second digits for some peaks
- Compound 111
    - For ^{13}C added second digits for some peaks
- Compound SF22
    - Updated molecular formula (to fit M+H)

Characterization Data of C2-C6 Arylated Products

- Compound 81
    - alpha
        - Updated molecular formula (to fit M+H)
        - Take care of j-couplings
        - Carbon spectrum double digit for consistency
    - Beta
        - Updated molecular formula (to fit M+H)
        - Added j-couplings in text
        - Carbon spectrum double digit for consistency
        -
- Compound 82
    - Alpha
        - Updated molecular formula (to fit M+H)
        - For ^{13}C added second digits for some peaks
        - Added j-couplings in text
    - Beta
        - Updated molecular formula (to fit M+H)
        - Added j-couplings in text
- Compound 83
    - Alpha
        - Updated molecular formula (to fit M+H)
    - Beta
        - Updated molecular formula (to fit M+H)
- Compound 84

- Alpha
    - Updated text for 1h spectrum
- Compound 87
  - Alpha
    - Updated molecular formula (to fit M+H)
    - Added j-couplings in text
    -
  - Beta
    - Updated molecular formula (to fit M+H)
    - For ^{13}C added second digits for some peaks
    -
  -
- Compound 88
  - Alpha
    - For ^{13}C added second digits for some peaks
  - Beta
    - For ^{13}C added second digits for some peaks
- Compound 89
  - Alpha
    - Updated molecular formula (to fit M+H)
    - Updated proton count in text (10H instead of 12H) to be consistent with spectrum
  - Beta
    - Updated molecular formula (to fit M+Na)
- Compound 90
  - Alpha
    - For ^{13}C added second digits for some peaks
  - Beta
    - Updated molecular formula (to fit M+H)
- Compound 93
  - Alpha
    - Updated molecular formula (to fit M+H)
    - Removed 1H peak in text at 4.69 ppm (copy error from software)
  - Beta
    - Updated molecular formula (to fit M+H)
    - Added j-couplings in text (carbon spectrum)
- Compound 94
  - Alpha
    - Updated molecular formula (to fit M+H)

    - Changed peak 7.25 – 7.11 (m) from 10H to 8H (overlap with chloroform peak)
  - Beta
    - Updated molecular formula (to fit M+H)
    - For ^{13}C added second digits for some peaks
    - Changed peak 7.31 – 7.26 (m) from 10H to 11H. Updated integration in spectrum
- Compound 95
  - Alpha
    - Updated molecular formula (to fit M+Na)
    - For ^{13}C added second digits for some peaks
  - Beta
    - Updated molecular formula (to fit M+Na)
- Compound 95
  - Alpha
    - For ^{13}C added second digits for some peaks
  - Beta
    - For ^{13}C added second digits for some peaks
- Compound 99
    - Updated molecular formula (to fit M+H)
    - For ^{13}C added second digits for some peaks
    - Changed peak 7.38 – 7.28 (m) from 14H to 15H. Updated integration in spectrum
- Compound 100
    - Updated molecular formula (to fit M+Na)
    - For ^{13}C added second digits for some peaks
- Compound 101
    - For ^{13}C added second digits for some peaks
    - Changed peak 7.39 – 7.26 (m) from 14H to 15H
- Compound 102
    - For ^{13}C added second digits for some peaks
- Compound 104
    - Updated molecular formula (to fit M+H)
- Compound 106
    - Alpha
      - Added j-couplings in text
      - Updated molecular formula (to fit M+H)
    - Beta
      - Added j-couplings in text
      - Updated molecular formula (to fit M+H)

- Compound 108
    - Updated molecular formula (to fit M+H)
- Compound 110
    - Updated molecular formula (to fit M+H)
    - For ^{13}C added second digits for some peaks
- Compound SC8
    - Alpha
        - Added j-couplings in text
        - Updated molecular formula (to fit M+H)
        - Added peak at 29.9 ppm in text
    - Beta
        - Added j-couplings in text
        - Updated molecular formula (to fit M+H)

Characterization data of C1-arylated Ribose

- Compound 113
    - For ^{13}C added second digits for some peaks
- Compound 114
    - Updated molecular formula (to fit M+H)
    - Changed peak 7.35 – 7.25 (m) from 14H to 15H. Updated integration in spectrum
- Compound 115
    - Updated molecular formula (to fit M+H)
- Compound SD1
    - For ^{13}C added second digits for some peaks
- Compound SD2
    - For ^{13}C added second digits for some peaks
- Compound SD3
    - For ^{13}C added second digits for some peaks
- Compound SD4
    - Updated molecular formula (to fit M+H)
- Compound SD6
    - For ^{13}C added second digits for some peaks
- Compound SD7
    - For ^{13}C added second digits for some peaks
- Compound SD8
    - For ^{13}C added second digits for some peaks
    - Changed peak 7.34 – 7.22 (m) from 16H to 14H. Updated integration in spectrum
- Compound SD9

- Updated molecular formula (to fit M+H)
- For ^{13}C added second digits for some peaks

- Compound SD10
    - Updated molecular formula (to fit M+H)
    - For ^{13}C added second digits for some peaks
- Compound SD11
    - Updated molecular formula (to fit M+H)
    - For ^{13}C added second digits for some peaks
- Compound SD12
    - Updated molecular formula (to fit M+H)
    - For ^{13}C added second digits for some peaks
- Compound SD13
    - For ^{13}C added second digits for some peaks
    - Changed peak 7.33 – 7.21 (m) from 15H to 14H. Updated integration in spectrum

Application

- Compound 43
    - Updated molecular formula (to fit M+H)
    - For ^{13}C added second digits for some peaks
- Compound SI3
    - Updated molecular formula (to fit M+H)
    - For ^{13}C added second digits for some peaks
- Compound SI4
    - For ^{13}C added second digits for some peaks
- Compound 52
    - Updated molecular formula (to fit M+H)
    - Added J-couplings in 13C

RCC-Arylation of Different Sugars

- Compound SH5
    - Mannose-tos (NMR p306)
        - Updated molecular formula (to fit M+H)
- Compound SH6
    - Rhamose-tos (NMR p308)
        - Updated molecular formula (to fit M+H)
        - Added peak 8.76 (s, 1H)
- Compound SH7
    - Rhamose-CF3 (NMR p290, SH5)
        - Updated molecular formula (to fit M+H)
        - Added J-couplings in 13C

- Compound SH8
    - lyxose-tos (NMR p310)
        - Updated molecular formula (to fit M+H)
- Compound SH9
    - lyxose-2F (NMR p294, SH7)
        - Updated molecular formula (to fit M-H)
- Compound SH10
    - galactose-tos (NMR p312)
        - Updated molecular formula (to fit M+H)
- Compound SH11
    - galactose-2F (NMR p96, SH8)
        - Updated molecular formula (to fit M+H)
- Compound SH12
    - ribose-3F
        - Updated molecular formula (to fit M+H)
- Compound SH13
    - arabinose-tos (NMR p316)
        - Updated molecular formula (to fit M+Na)
- Compound SH14
    - arabinose-2F (NMR p300, SH10)
        - Updated molecular formula
- Compound SH15
    - fucose-tos (NMR p318)
        - Updated molecular formula
- Compound SH16
    - fucose-2F (NMR p302, SH11)
        - Updated molecular formula (to fit M-H)
- Compound SH17
    - xylose-tos (NMR p320)
        - Updated molecular formula (to fit M-H)
        - Updated peaks (Insert missing H between 4 and 4 ppm)
- Compound SH18
    - xylose-2F (NMR p304, SH12)
- Compound SH19
    - deoxyglucose-tos (NMR p322)
- Compound SH20
    - deoxyglucose-3F (NMR p322)
        - added HRMS
- Compound SH21
    - N-Ac-Glu-tos (NMR p324)

        - Updated molecular formula (to fit M+H)
- Compound SH22
    - N-Ac-Glu-3F (NMR p298, SH9)
        - Updated molecular formula (to fit M+H)

Xylose

- Compound SJ4
        - Updated molecular formula (to fit M+H)
        - For ^{13}C added second digits for some peaks
- Compound SJ5
    - beta
        - Updated molecular formula (to fit M+H)
        - For ^{13}C added second digits for some peaks
    - Alpha
        - Updated molecular formula (to fit M+H)
        - For ^{13}C added second digits for some peaks
        -
- Compound SJ9
        - Updated molecular formula (to fit M+H)
        - For ^{13}C added second digits for some peaks
- Compound SJ10
        - Updated molecular formula (to fit M+H)
        - For ^{13}C added second digits for some peaks
- Compound SJ12
        - For ^{13}C added second digits for some peaks

Response to Editorial Comments

Below are our general guidelines for preparing files for the formal acceptance process. I also attach a checklist that you can refer to prepare ‘production-ready’ final files (there is no need to fill this in and send it back with your manuscript, it simply highlights things that the production team need — and that can result in delays later on if not attended to). Specific comments from us are:

[Critiques 1]

- I suggest a shortened title to “C-Glycoside synthesis via radical cross-coupling of glycohydrazides”

[Response]: We have changed the title from “General Access to C-Glycosides via Redox-Neutral Radical Cross-Coupling of Glycohydrazides” to “C-Glycoside synthesis via Radical Cross-Coupling of Glycohydrazides”.

[Critiques 2]

- The manuscript is a fair bit more substantial than a normal 2500-3000 words and 4 Figures, but as it’s succinctly and largely well written as is, this is fine to stay at pretty much its current length. Please remove/minimise the concluding remarks, and use short subtitles to break up the text – I suggest headings such as “synthetic method”, “general scope” “native “application to natural product synthesis” “pre-radical stereocontrol” “peripheral C-C bond formation” could guide the reader through the text, though these are just suggestions.

[Response]: We have minimized the concluding remarks, and short subtitles have been added to improve navigation throughout the manuscript.

[Critiques 3]

- Please change “a transformative approach” in line 69 to “an approach”

[Response] This has been changed.

[Critiques 4]

- The structures in Fig 1a are later recapitulated when they are synthesised in Fig 2, so please trim 1a to remove.

[Response]: Several structures have been removed from Fig. 1 to improve clarity and make the figure more concise.

[Critiques 5]
- Please remove blue bars in all the Figures, likewise background shading and highlighting boxes.

[Response]: All blue bars, background shading, and highlighting boxes have been removed from the figures.

[Critiques 6]
- Please ensure the titles for each figure panel are in the legend, not in the Figure itself

[Response]: The titles for all figure panels have been moved from the figures to the corresponding legends.

[Critiques 7]
- In Figure 3, conditions A/B/C colour coding can be removed with no loss of information for the reader (since the A/B/C labels remain); particularly as purple and blue shading is also used for the pyranose/furanose rings

[Response]: For Figure 3, the colour coding for Conditions A/B/C and the background shading have been removed.

[Critiques 8]
- Please remove the previous routes from Figure 4 and mention previous step count only. If you wish to retain the explicit previous routes I would suggest using an Extended Data Figure for this instead.

[Response]: The previous routes have been removed from Figure 4.

[Critiques 9]
SUMMARY PARAGRAPH: Nature papers begin with a fully referenced paragraph of <200 words. This paragraph starts with a 2- to 3-sentence, basic introduction to the field; continues with a 1-sentence statement of the main findings starting 'Here we show' or an equivalent phrase; and concludes with 2 to 3 sentences putting the main findings into context so it is clear how the

results have moved the field forward. To explain complex material for readers in other fields, summary paragraphs can be up to 230 words; the extra length is for introduction and context, and not for additional technical information.

[Response]: The summary paragraph is fully referenced with 203 words.

[Critiques 10]

MAIN TEXT: Further introductory material should be minimised, and — if used — kept to under 500 words. The main text should be a concise, focused account of the new research and findings, and can end with 1-2 short paragraphs of discussion, provided this does not recapitulate earlier text. Sections are separated with subheadings (< 40 characters including spaces) to aid navigation.

[Response]: The main text begins with 402 words of introduction, and the subheadings are now added to aid navigation.

[Critiques 11]

METHODS: The Methods section provides methodological information that allows other researchers to replicate the results; it should be written as concisely as possible but contain all elements necessary to allow interpretation and reproduction of the results. We define "Methods" quite broadly, so this is not limited to details of experimental protocols – supplementary discussion and analysis can also be included. The Methods section will not appear in the print version, but will be fully copy-edited and appear online in the full-text HTML and PDF versions. If there are additional references in the Methods section, their numbering should continue from the last reference in the main paper, and the list should follow the Methods section. If the methods require chemical structures, figures or tables, these should be supplied as Extended Data (see below), unless the number of figures or tables is extensive — in which case the entire Methods section should instead be included in Supplementary Information.

[Response]: N/A

[Critiques 12]

REFERENCES: As a guideline, Articles usually have 30-50 references in the main text; additional references can be cited in (and listed after) the Methods section, as detailed above.

[Response]: We have reviewed the reference list and believe that the 58 references included are necessary to provide appropriate background and context for the work.

[Critiques 13]

DATA AVAILABILITY: This journal requires authors to make data and custom code publicly available, preferably through public repositories or, if not possible, as Supplementary Information. If data and code can only be shared on request, please explain why in your Data Availability Statement. All Nature papers must include a Data Availability Statement (DAS); furthermore, all studies using custom code central to the conclusions must also include a Code Availability Statement, indicating how the code or algorithm can be accessed. In the data and code availability statements we discourage the use of the term "available upon reasonable request" as it implies a potential restriction on availability. We suggest a simple "available upon request" where relevant, or detailed clarification on any restrictions that might apply. If a dataset generated or analysed during the study is publicly available and has a unique Digital Object Identifier (DOI), we strongly encourage authors to include this in the reference list and to cite the dataset.

If you need help with our data sharing policies or finding a suitable data repository, you should consider finding a suitable data repository for your data from our recommended repository list. Where they are available, community specific repositories are preferred. Unstructured repositories are suitable alternatives if no structured public repositories exist.

[Response]: Data Availability session is added as well.

[Critiques 14]

MAIN TEXT STATEMENTS: Several statements — which will not appear in print but will appear online in the full-text HTML and PDF — are required after the Methods (and additional references, if present):

1) Acknowledgements, which should be non-financial.

2) Funding Statement, for which guidelines can be found here.

3) Author Contributions: the contributions of each author, particularly in terms of which authors performed specific experiments, must be listed using author initials.

4) Competing Interests: list financial and non-financial interests, as well as any patents. Authors employed by commercial entities should be noted. Patent information should include at a minimum the patent number, what is covered by the patent, and who submitted the patent application.

5) Additional Information statement: name the author(s) to whom correspondence and requests

for materials should be addressed, and include the sentence “Reprints and permissions information is available at http://www.nature.com/reprints”. Formatting details and an example are available here.

[Response]: All of these have been added.

[Critiques 15]

DISPLAY ITEMS: We ask that you take stock of all the data that have been generated throughout the review process and ensure that only the data most central to the conclusions are presented in the main figures. Figures should be as small and simple as is compatible with clarity, with all panels logically connected, presented on a single page and assembled into a rectangular shape. Nature's standard figure sizes are 89 mm (one column), 120 mm (one and a half columns), or 183 mm (two columns) wide; with a maximum figure height of 170 mm. Guidance from Nature’s art department can be found here.

Units follow SI nomenclature or the nomenclature common to a particular field, and have a single space between the number and the unit. Thousands are separated by commas (1,000). Error bars, unusual units or abbreviations are defined in the legend. Scale bars rather than magnification factors should be used. Authors should be aware that any image provided for publication may be subject to checks for image integrity and manipulation, in accordance with Nature portfolio policies on image standards.

Figure legends should be listed sequentially after the references in the main text and not in the figures files. Each legend begins with a brief title for the whole figure and continues with a short description of each panel.

[Response]: Questions regarding to the figures have been addressed point-by-point.

[Critiques 16]

CHEMICAL STRUCTURE PRESENTATION: Any chemical structures in the main text or Extended Data figures must conform to our chemical structure style guide encoded in the associated ChemDraw stylesheet. If it is absolutely necessary to shrink from the template, 80% size and 5pt font is the smallest permissible size.

[Response]: All chemical structures have been revised to conform to Nature’s chemical structure style guide and ChemDraw formatting requirements.

[Critiques 17]

EXTENDED DATA: Nature integrates the supplemental figures and tables into most papers as Extended Data, included online within the full-text HTML and at the end of the online PDF. All Extended Data must be referred to in the main text, figure legends and/or Methods section, and their legends listed at the end of the main text, not in the Extended Data files. Authors should assemble the Extended Data Figures and/or Tables into a maximum of ten, A4 size, multi-panelled display items, submitted as individual JPEG, TIFF or EPS files. They must be provided at the same quality as figures for print; more specific instructions are provided in the Extended Data Formatting Guide.

[Response]: N/A

[Critiques 18]

SUPPLEMENTARY INFORMATION: Supplementary Information is online-only material, with a maximum file size of 30 MB per file. For most papers, there should be no need for Supplementary Information beyond that already provided as Methods and Extended Data, the aim being to avoid fragmentation of the paper. Exceptions to this rule include large datasets, audio/video material, and more complex “Supplemental Methods” that do not readily fit within the constraints of the Methods/Extended Data formats. Please note that after the paper has been accepted you can only provide amended Supplementary Information files for changes to the scientific content, not for style. For optimal Supplementary Videos please use a H.264 encoding and the standard aspect ratio of 16:9 (4:3 is second best), and do not compress the video. Videos will be rendered using the Brightcove platform; for additional recommendations and specification please refer to this page.

[Response]: We have reviewed the Supplementary Information and ensured that it complies with Nature's guidelines.

[Critiques 19]

SOURCE DATA: Authors are encouraged to submit the data underlying graphical representations in the figures as spreadsheets (.xls, .xlsx, or .csv). The source data will be accessible online via the figure legends. One file per figure is allowed; if the figure has several panels the data should be included in multiple, clearly labelled sheets within a single Excel file (< 30MB). Please use the title field in the file description tab to indicate the figure to which the source data pertain.

[Response]: The source data files have been prepared and organized according to Nature's requirements.
